# Corticortophin releasing factor 2 receptor agonist treatment significantly slows disease progression in mdx mice

**DOI:** 10.1186/1741-7015-5-18

**Published:** 2007-07-12

**Authors:** Richard T Hinkle, Frank R Lefever, Elizabeth T Dolan, Deborah L Reichart, Jefferey A Dietrich, Kathryn E Gropp, Robert I Thacker, Jeffrey P Demuth, Paula J Stevens, Xiaoyan A Qu, Alex R Varbanov, Feng Wang, Robert J Isfort

**Affiliations:** 1 Research Division, Procter & Gamble Pharmaceuticals, Mason, OH, USA; 2 Department of Pathobiology and Molecular Medicine, University of Cincinnati, Cincinnati, OH, USA

## Abstract

**Background:**

Duchenne muscular dystrophy results from mutation of the dystrophin gene, causing skeletal and cardiac muscle loss of function. The mdx mouse model of Duchenne muscular dystrophy is widely utilized to evaluate the potential of therapeutic regimens to modulate the loss of skeletal muscle function associated with dystrophin mutation. Importantly, progressive loss of diaphragm function is the most consistent striated muscle effect observed in the mdx mouse model, which is the same as in patients suffering from Duchenne muscular dystrophy.

**Methods:**

Using the mdx mouse model, we have evaluated the effect that corticotrophin releasing factor 2 receptor (CRF2R) agonist treatment has on diaphragm function, morphology and gene expression.

**Results:**

We have observed that treatment with the potent CRF2R-selective agonist PG-873637 prevents the progressive loss of diaphragm specific force observed during aging of mdx mice. In addition, the combination of PG-873637 with glucocorticoids not only prevents the loss of diaphragm specific force over time, but also results in recovery of specific force. Pathological analysis of CRF2R agonist-treated diaphragm muscle demonstrates that treatment reduces fibrosis, immune cell infiltration, and muscle architectural disruption. Gene expression analysis of CRF2R-treated diaphragm muscle showed multiple gene expression changes including globally decreased immune cell-related gene expression, decreased extracellular matrix gene expression, increased metabolism-related gene expression, and, surprisingly, modulation of circadian rhythm gene expression.

**Conclusion:**

Together, these data demonstrate that CRF2R activation can prevent the progressive degeneration of diaphragm muscle associated with dystrophin gene mutation.

## Background

Duchenne muscular dystrophy (DMD) is a lethal progressive muscle-wasting disease with an incidence of 1 in 3500 live male births [1-3]. Duchenne muscular dystrophy is usually diagnosed by age 4 or 5 and results in the progressive loss of striated muscle function (including diaphragm function), cardiac malfunction, loss of mobility and muscle strength, such that DMD patients are typically wheelchair-bound by age 12, with death from respiratory and heart failure usually occuring by the late teens or early twenties [1-3]. DMD and the less severe, yet related, Becker muscular dystrophy (BMD) both result from mutation of the dystrophin gene [1-3]. The dystrophin gene is an X chromosome-linked gene that is one of the largest known, coding for a 427 kDa protein [1-3]. Dystrophin is a member of a multicomponent complex with multiple functions, including connecting the cytoskeleton to the extracellular matrix, reinforcing the sarcolemma to prevent membrane tearing during myocyte contraction, modulating calcium influx in the myocyte, and serving as a nucleation site for many enzymatic activities including nitric oxide synthetase [1-3].

The current treatment for DMD is corticosteroid therapy [2,4-7]. It has been observed that high-dose corticosteroid treatment, specifically with prednisone and deflazacort, slows disease progression through an as yet unknown mechanism [2,4-7]. Other treatment modalities currently being evaluated include gene replacement therapy, stem cell transfer, protease inhibitors, exon skipping therapeutics and translation modulating agents, such as aminoglycosides [2,4,5,7].

There are several animal models of DMD, including the mouse mdx model [8,9]. The mdx mouse resulted from a spontaneous mutation of the dystrophin gene that caused the formation of a premature stop codon and truncation of the dystrophin protein [8,9]. Mdx mouse striated muscle is normal at birth but undergoes a spontaneous degeneration/regeneration event at approximately 3 weeks of age [8,9]. After the regeneration event, mdx mouse striated muscle undergoes continual deterioration until premature death occurs [8,9]. Interestingly, in the mdx mouse the diaphragm undergoes rapid and continual deterioration while the limb muscles and the heart are less affected; this is in contrast to DMD patients where limb muscle and cardiac deterioration occurs at a similar rate to diaphragm degeneration [8-12]. Thus, the diaphragm is often used for evaluating the therapeutic potential of compounds in the mdx mouse model of DMD [8-12]. The mdx mouse model has been used to evaluate a number of compounds for efficacy, and correlation between the mdx mouse model and DMD patients appears to be quite good [2,9].

Recently, we have shown that corticotrophin releasing factor receptor 2 (CRF2R) agonists can modulate skeletal muscle mass by increasing muscle mass (hypertrophy) and decreasing loss from atrophying/wasting of muscle mass [13-15]. These effects occur by decreasing proteolysis during atrophying conditions and activation of anabolic signalling pathways [13-15]. Therefore, we have utilized potent CRF2R agonists in the mdx model in order to evaluate the therapeutic potential for these compounds in DMD.

## Methods

### Materials

The CRF2R selective agonist PG-873637 was synthesized at Procter & Gamble Pharmaceuticals (Cincinnati, OH, USA) as described previously [16-18]. Prednisone, Tween 80, and benzyl alcohol were purchased from SIGMA (St Louis, MO, USA). Methyl cellulose was purchased from Aldrich. Male 2 and 3 month old C57BL/10-DMD^mdx ^and C57BL/10 mice were purchased from the Jackson Laboratories (Bar Harbor, ME, USA). Mice were single-housed and acclimatized to the conditions of the facility for approximately 1 week before use. Mice had access to lab chow and water *ad libitum *and were subjected to standard conditions of humidity, temperature and a 12-hour light cycle. All studies described in this report were conducted in compliance with the US Animal Welfare Act, the rules and regulations of the State of Ohio Departments of Health, and in accordance with the Procter & Gamble Company policy on research involving animals with strict oversight for care and welfare. For details of the policy please contact the Procter & Gamble Company.

### Dosing and diaphragm functional analysis

PG-873637 was administered at 30 ug/kg by daily subcutaneous injection. Prednisone was administered at 1 mg/kg by daily subcutaneous injection. The vehicle for PG-873637 was 0.9% sodium chloride/0.2% Tween 80/water, while the vehicle for prednisone was 0.9% sodium chloride/1% methyl cellulose/1% benzyl alcohol/0.2% Tween 80/water. At the end of the study, mice were anesthetized with isoflurane, the mid-section of the mouse shaved, a lateral incision was made just below the ribcage, and the spinal cord cut to exsanguinate the animal. The diaphragm was then removed still attached to the ribs, placed in a Petri dish containing 25°C oxygenated (95% oxygen/5% carbon dioxide) Krebs-Ringer solution (137 mM sodium chloride, 24 mM sodium bicarbonate, 11 mM glucose, 5 mM potassium chloride, 1 mM magnesium sulfate, 1 mM sodium phosphate, pH 7.4). The diaphragm was cut into hemispheres, and the larger hemisphere containing the vertebrate was discarded. The smaller hemisphere was spread, pinned, a needle containing suture material was inserted through one of the ribs and tied, a section of diaphragm was cut from the rib to the central tendon parallel to the muscle fibers, and the central tendon was tied to suture. The diaphragm strip was then placed in a 25°C oxygenated bath containing Krebs-Ringer solution containing 0.025 mM d-turbocurarine chloride with one end attached to a force transducer and the other end attached to a fixed post for contractile properties testing. Diaphragm strips were aligned horizontally between a servomotor lever arm and the stainless steel hook of a force transducer (Aurora Scientific Inc., model 6650 LR) and field-stimulated by pulses transmitted between two platinum electrodes placed longitudinally on either side of the muscle. Square wave pulses (0.2 ms duration) generated by a personal computer with a Labview board (Model PCI-MIO-16E-4, Labview Inc., Austin, TX, USA) were amplified (Acurus Power Amplifier Model A250, Dobbs Ferry, NY, USA) to increase and sustain current intensity to a sufficient level to produce a maximum isometric titanic contraction. Testing included maximizing stimulation voltage and optimizing muscle length for maximum force development during twitch (1 Hz). Following twitch measurements, the diaphragm strip was stimulated at increasing frequencies (10–300 Hz) until a maximum tetanic force (Po) was obtained. At the end of the measurement of force, the diaphragm strip was measured from myotendinous junction to the point of insertion on the rib and optimal length (Lo) was obtained. Stimulation voltage and muscle length (Lo) were adjusted to obtain maximum isometric twitch force. Maximum tetanic force production (Po) was determined from the plateau of the frequency-force relationship. Immediately after testing, the muscle was trimmed of tendon and extraneous tissue and weighed. Additional strips of diaphragm muscle were removed, with one strip at resting length placed in formalin for histomorphological and myofiber cross-sectional area analysis, and a second strip snap frozen in liquid nitrogen for expression profiling analysis.

### Myofiber cross-sectional area analysis

Myofiber cross-sectional area analysis was performed on 10% neutral buffered formalin fixed resting length diaphragm muscle preparations from every treated animal. Following paraffin embedding, cross-sections were cut from the diaphragm muscle strip in triplicate. Sections were stained with Picro-sirius Red (Sirius red F3B, C.I. 35782), which stains endomysium collagen red resulting in clearly delineated and easily digitalized muscle fibers (myofibers stain light yellow). Digital images of the stained samples were acquired using a SPOT RT camera and the SPOT Advanced Imaging Software (Universal Imaging Corp., Downingtown, PA, USA) from the center third of each section. Automated segmentation and myofiber cross-sectional area measurements, based on differential staining of the endomysium and myofibers, was performed using custom software developed at Procter & Gamble Pharmaceuticals. Aphelion 3.2 software (Amerinex Applied Imaging, Amherst, MA, USA) was used to manually edit the processed images in order to ensure accurate measurement of only myofiber cross-sectional area. An average of approximately 165 myofibers per diaphragm per animal were evaluated.

### Histopathological analysis

Formalin-fixed diaphragm tissue was paraffin embedded, cross-sectioned and stained with either hematoxylin and eosin (H&E) or Masson's trichrome stain. The stained sections were evaluated and scored blind to treatment group by a Board Certified Pathologist (KEG) for inflammation and muscle fiber pathology (H&E stain) and fibrosis (Masson's trichrome stain). The severity of inflammation and fibrosis was graded on a six-point scale (none = 0, minimal = 1, slight = 2, mild = 3, moderate = 4, marked = 5) for each sample, with a composite score recorded and average severity score determined.

### Statistical analysis of animal data

Statistical analysis of the data was performed using an ANCOVA model with treatment effect and starting weight as the covariates. Pairwise comparisons for all end-points were generated using least-square means (SAS, Cary, NC, USA), adjusted for unequal sample sizes and starting weight.

### Expression profiling analysis

Snap frozen diaphragm muscle strips were homogenized in Trizol (Life Technologies, Rockville, MD, USA) using tungsten carbide beads (Qiagen, Chatsworth, CA, USA) with shaking in a mixer mill (Qiagen) as recommended by the manufacturer. RNA samples were prepared according to the recommendations of the manufacturer (Affymetrix; Santa Clara, CA, USA). Briefly, total RNA was prepared with the use of Trizol reagent (Life Technologies). After the Trizol extraction, the RNA was purified with a RNeasy Mini Kit (Qiagen). Reverse transcription was performed on 10 ug of total RNA with the use of SuperScript II reverse transcriptase and a T7-(dT)_24 _primer followed by second strand DNA synthesis utilizing T4 DNA polymerase (all from Life Technologies) as recommended by the manufacturer. Contaminants were removed from the double-stranded cDNA by phenol-chloroform-isoamyl alcohol extraction and then cDNA was recovered by ethanol precipitation. A RNA Transcript Labeling Kit (Enzo Diagnostics, Farmingdale, NY, USA) was used for production of biotin-labeled cRNA (complementary RNA) targets by *in vitro *transcription from T7 RNA polymerase promoters, all as recommended by the kit manufacturer. The cDNA prepared from total RNA was used as a template in the presence of a mixture of unlabeled ATP, GTP, CTP and UTP and biotinylated CTP and UTP. *In vitro *transcription products were purified with an RNeasy Mini Kit (Quiagen) to remove unincorporated NTPs and fragmented to approximately 35 to 200 bases by incubation at 94°C for 35 min in fragmentation buffer containing tris-acetate, potassium acetate, and magnesium acetate. Fragmented cRNA was stored at -20°C until the hybridization was performed. Biotinylated and fragmented cRNA was hybridized for 16 h at 45°C to mouse MOE430Plus arrays (Affymetrix) in a GeneChip Hybridization Oven 640 (Affymetrix). A series of stringency washes and staining with streptavidin-conjugated phycoerythrin was then performed in a GeneChip Fluidic Station 400 (Affymetric) according to the protocol recommended by Affymetrix. Probe arrays were then scanned with an Agilent GeneArray Scanner. The images were analyzed with the GeneChip Analysis software (Affymetrix).

### Statistical analysis of expression profiling data

The initial statistical analysis was focused on determining "outlier" chips. This includes exploratory data analysis using chip-descriptive statistics, pair plots, and principal component analysis. After "outlier" chips are eliminated from further statistical analysis, the gene expression signal (based on Affymetrix MAS 5.0 algorithm) is preprocessed using scaling and quantile normalization, and transformed to log (base 2) scale. Following this, an analysis of variance (ANOVA) statistical model was utilized to estimate Log fold change (LFC) and corresponding uncertainty measure, standard error (SE), for the paired conditions of interest. The ratio of LFC to SE was investigated to determine the statistical significance of the differential gene expression between two compared experimental conditions. Statistical significance was summarized by a quantity called NLOGP (= -log_10 _[P-value]). An NLOGP threshold equal to -log_10_(average false positive rate) is used to detect genes with statistically significant differential expression (corresponding NLOGP measure is greater than the NLOGP threshold). All data was tabulated as fold change in expression relative to the 0 timepoint. A gene is considered to be differentially expressed if all of the following three rules hold:

1. The NLOGP measure is greater than the threshold (NLOGP = 4.0).

2. There are at least 50% Affymetrix present calls for the overexpressed condition in the comparison.

3. The fold change is at least 1.2.

### Results of bioinformatics analysis of expression profiling

To assign annotation to the differentially-expressed genes to gain the best understanding of the functional roles they play in their respective biological processes, a variety of public resources as well as proprietary tools were used. These included: Affymetrix Netaffx analysis database (Affymetrix: Santa Clara, CA, USA), GeneCard (Weizmann Institute of Science, Israel), UniGene, RefSeq and LocusLink (NCBI, Bethesda, MD, USA), SwissProt/TrEMBL (Swiss Institute of Bioinformatics, Switzerland), FANTOM2 (RIKEN Yokohama Institute, Japan), and The Institute of Genomics Research (TIGR) Gene Index (Boston, MA, USA) databases. For those uncharacterized genes or ESTs, a semi-automatic annotation strategy was used that combined the following steps: (1) Homology searching against the major nucleotide and protein databases, including NCBI-nr (Bethesda, MD, USA), Ensembl (European Bioinformatics Institute, Germany) and SwissProt/SPTreMBL (Swiss Institute of Bioinformatics, Switzerland) using BLASTX/BLASTP (NCBI, Bethesda, MD, USA); (2) derivation of function from homolog/orthlog databases, including HomoloGene (NCBI, Bethesda, MD, USA) and TIGR Resourcerer/TOGA (Boston, MA, USA) databases; (3) assignment or prediction of functional roles by protein motif search using PFAM (Protein Family Database) (Welcome Trust Sanger Institute, Cambridge, UK), Prosite (Swiss Institute of Bioinformatics, Switzerland), and/or InterPro (European Bioinformatics Institute, Germany). In addition, curation from bioinformatics efforts was applied, based upon the available biomedical literature sources.

## Results

### Experimental design and goals

**Figure 3:**
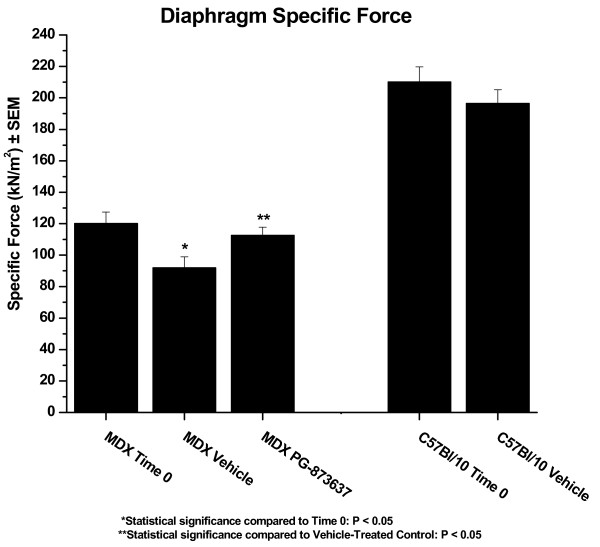
**Effect of 3 months of treatment on either mdx or C57BL10 diaphragm specific force**. Two-month-old mdx or C57BL10 mice were treated by daily subcutaneous injection with the indicated compound for 3 months and at the end of treatment, the diaphragms were removed and evaluated for force production. MDX Time 0, 2 month old mdx mice before treatment. MDX Vehicle, mdx mice treated for 3 months with vehicle. MDX PG-873637, mdx mice treated for 3 months with 30 ug/kg of PG-873637. C57BL10 Time 0, 2 month old C57BL10 mice before treatment. C57BL10 Vehicle, C57BL10 mice treated for 3 months with vehicle.

For all experiments, mdx mice at 2–3 months of age (at study start) were utilized. We chose this age for the mice because the major myofiber death/regeneration event is over, and the mice showed a steady progressive loss of diaphragm specific force with time (in mdx mice at 2–3 months of age there is about 50% loss ofdiaphragm specific force compared to C57BL10 wild-type mice – Figure 3). We investigated diaphragm muscle as it has been shown previously that mdx diaphragm muscle, unlike limb muscles, undergoes a progressive loss of function similar to that observed in DMD patients. The goal of the first experiment was to evaluate the effect of daily subcutaneous injection treatment with 1 mg/kg of prednisone, a glucocorticoid that has been shown to have efficacy at this dose in the mdx model and DMD patients [6,19,20], and a CRF2R agonist (PG-873637), either alone or in combination. In the first experiment, we assessed the following variables: 3-month-old mdx mice untreated (diaphragm muscle was analyzed at the start of the study; control time 0), mdx mice treated for 3 months with vehicle by once-daily subcutaneous injection (vehicle), mdx mice treated for 3 months with 1 mg/kg of prednisone by once-daily subcutaneous injection (prednisone), mdx mice treated for 3 months with 30 ug/kg PG-873637 by once-daily subcutaneous injection (PG-873637), and mdx mice treated for 3 months with 1 mg/kg prednisone and 30 ug/kg PG-873637 by once-daily subcutaneous injection (prednisone + PG-873637). At the end of the study, diaphragm function, histomorphology and myofiber cross-sectional area were analyzed. The goal of the second experiment was to evaluate the effect of daily subcutaneous injection treatment with a CRF2R agonist (PG-873637) on diaphragm function and gene expression. In the second experiment, we assessed the following: 2-month-old mdx mice untreated with diaphragm analysis at 2 months of age (mdx control time 0), mdx mice treated for 3 months with vehicle by once-daily subcutaneous injection (mdx vehicle), mdx mice treated for 3 months with 30 ug/kg PG-873637 administered by once-daily subcutaneous injection (mdx PG-873637), 2-month-old C57BL10 mice untreated with diaphragm analysis at 2 months of age (C57BL10 control time 0), and C57BL10 mice treated for 3 months with vehicle by once-daily subcutaneous injection (C57BL10 vehicle). At the end of the study, diaphragm function and gene expression were analyzed.

### Effect of CRF2R agonist and corticosteroid treatment on mdx diaphragm muscle function and structure

**Figure 1:**
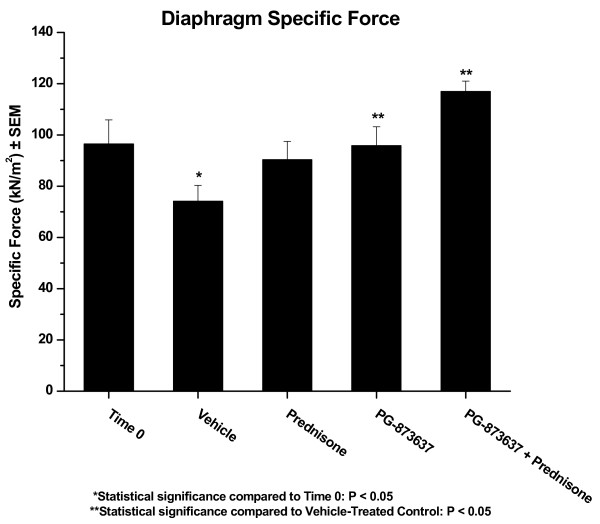
**Effect of 3 months of treatment on mdx diaphragm specific force**. Three-month-old mdx mice were treated by daily subcutaneous injection with the indicated compounds for 3 months and, at the end of treatment, the diaphragms were removed and evaluated for force production. Time 0, 3 month old mdx mice before treatment. Vehicle, mdx mice treated for 3 months with vehicle. Prednisone, mdx mice treated for 3 months with 1 mg/kg of prednisone. PG-873637, mdx mice treated for 3 months with 30 ug/kg of PG-873637. PG-873637 + prednisone, mdx mice treated for 3 months with the combination of 30 ug/kg PG-873637 plus 1 mg/kg prednisone.

**Figure 2:**
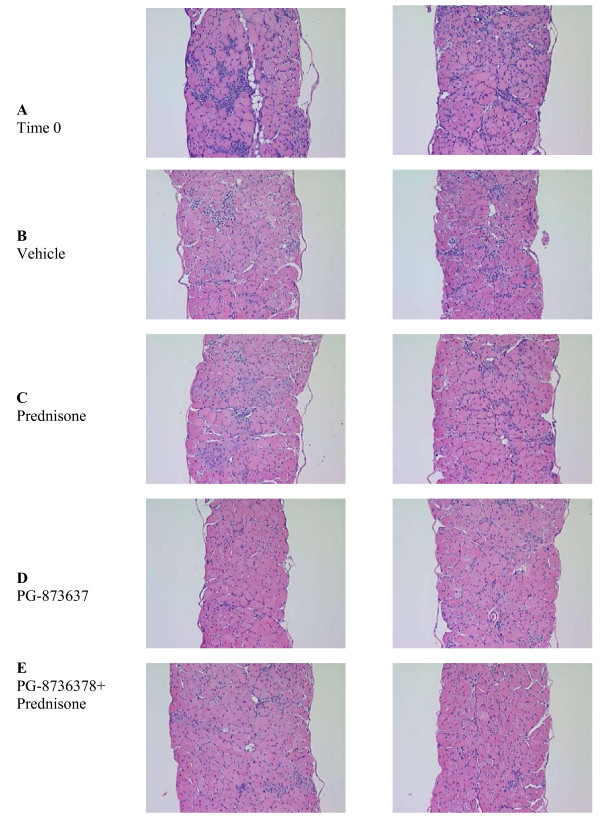
**Histomorphological analysis of diaphragm muscle from mdx mice treated with vehicle, prednisone, PG-87367 and PG-873637+prednisone for 3 months**. Three-month-old mdx mice were treated by daily subcutaneous injection with the indicated compounds for 3 months and at the end of treatment, the diaphragms were removed and a histomorphological analysis performed. Time 0, 3 month old mdx mice before treatment. Vehicle, mdx mice treated for 3 months with vehicle. Prednisone, mdx mice treated for 3 months with 1 mg/kg of prednisone. PG-873637, mdx mice treated for 3 months with 30 ug/kg of PG-873637. PG-873637 + prednisone, mdx mice treated for 3 months with the combination of 30 ug/kg PG-873637 plus 1 mg/kg prednisone. The two photographs in each treatment group are from representative diaphragm sections from two different animals in each treatment group. All samples were hematoxylin and eosin stained.

In experiment 1 we observed the following: 3 months of daily subcutaneous vehicle injection of mdx mice resulted in an approximate 20% loss of diaphragm specific force; treatment with 1 mg/kg of prednisone abrogated most of the loss of diaphragm specific force (p = 0.09 versus vehicle); treatment with 30 ug/kg of PG-873637 completely blocked the loss of diaphragm specific force (p < 0.05 versus vehicle); and treatment with 1 mg/kg prednisone plus 30 ug/kg PG-873637 not only abrogated the loss of diaphragm specific force (p < 0.05 versus vehicle) but increased diaphragm specific force over that observed at time 0 (p = 0.09 versus time 0) (Figure 1 and Table 1). Diaphragm muscle myofiber cross-sectional area changes were in parallel with the time- and treatment-related changes observed in diaphragm specific force; diaphragm muscle mass was increased at all time and treatment data points relative to time 0, diaphragm absolute force increased with PG-873637 and PG-873637 + prednisone treatment, and there were relatively few time- and treatment-related changes in diaphragm muscle peak twitch force, half relaxation time, and time to peak tension (Table 1). Histopathological analysis of the diaphragm muscle from the various treatment groups of experiment 1 showed increased fibrosis following 3 months of vehicle treatment relative to time 0; relative to vehicle treatment, there was less fibrosis with prednisone, PG-873637, and prednisone+PG-873637 treatment, and the fibrotic index actually fell below time 0 with the combination treatment of prednisone + PG-873637 treatment (Figure 2 and Table 2). There was no increase in inflammation in the diaphragm following 3 months of vehicle treatment; treatment with prednisone, PG-873637, and prednisone + PG-873637 decreased inflammation below the time 0 level (Figure 2 and Table 2). The general histopathology for diaphragms from mdx mice had typical findings for this genotype: interstitial inflammation (lymphocytic and mononuclear), interstitial fibrosis, internal myofiber nuclei, increase in myofiber area variability, evidence of myofiber regeneration, rare degenerative myofibers, rare myofiber mineralization and infiltration of adipocytes.

**Table 1:** Summary of diaphragm muscle parameters from experiment 1. Effect of 3 months of treatment with either vehicle, 1 mg/kg prednisone, 30 ug/kg PG873637 or the combination of prednisone plus PG873637 on male 3-month-old (at time 0) mdx mice.

	**Time 0**	**Vehicle**	**Prednisone**	**PG-873637**	**PG-873637 + prednisone**
Number of animals	10	10	10	9	8
Initial body mass (g)	28.49 (0.47)	28.53 (0.74)	28.36 (0.77)	28.39 (0.60)	27.37 (0.75)
Final body mass (g)	28.49 (0.47)	31.36† (0.90)	29.83† (0.62)	34.56† (0.38)	32.09† (0.48)
Diaphragm mass (g)	0.0031 (0.0002)	0.0035 (0.0003)	0.0038† (0.0002)	0.0041† (0.0003)	0.0038† (0.0003)
Diaphragm sPo (kN/m2)	96.478 (9.441)	74.100† (6.171)	90.340 (7.126)	95.811* (7.389)	116.91* (4.067)
Diaphragm Po (mN)	42.133 (3.239)	37.467 (4.043)	46.270 (2.720)	49.844* (3.557)	56.838*† (4.075)
Diaphragm Pt (mN)	11.511 (1.148)	9.778 (1.474)	12.470 (0.776)	10.278 (1.031)	12.063 (1.058)
Diaphragm HRT (ms)	56.289 (2.195)	50.222 (6.179)	51.070 (4.049)	50.089 (3.987)	45.875† (2.344)
Diaphragm TPT (ms)	33.511 (0.870)	33.989 (1.814)	33.650 (0.949)	31.267 (1.650)	29.588*† (0.685)
Diaphragm myofiber CSA	1634.2 (56.89)	1158.4† (55.55)	1429.5* (91.73)	1375.7*† (59.17)	1366.0*† (36.45)

sPo, muscle specific force; Po, muscle absolute force; Pt, muscle peak twitch force; HRT, muscle half relaxation time; TPT, muscle time to peak tension; CSA, myofiber cross-sectional area. All data are given as the mean with standard error of the mean in parenthesis. *Statistically significant difference (p < 0.05) versus the appropriate vehicle control; †statistically significant difference (p < 0.05) versus time 0.

**Table 2:** Summary of histopathology analysis for fibrosis and inflammation in H&E and Masson's trichrome stained diaphragm muscle from mdx mice in experiment 1. Effect of 3 months of treatment with either vehicle, 1 mg/kg prednisone, 30 ug/kg PG-873637 or the combination of prednisone plus PG-873637 on male 3-month-old (at time 0) mdx mice.

	**Time 0**	**Vehicle**	**Prednisone**	**PG-873637**	**PG-873637 + prednisone**
Number of animals	10	10	10	9	8
Fibrosis	2.0 (0)	3.6† (0.16)	3.2† (0.25)	2.6*† (0.29)	1.9*‡ (0.13)
Inflammation	1.7 (0.26)	1.7 (0.15)	1.4 (0.16)	1.3* (0.15)	1.2* (0.15)

Diaphragm muscle samples were evaluated as described in Methods. All data are given as the mean with standard error of the mean in parenthesis. *Statistically significant difference (p < 0.05) versus the appropriate vehicle control; † statistically significant difference (p < 0.05) versus time 0; ‡ statistically significant difference (p < 0.05) versus PG-873637.

### Transcriptional profiling of CRF2R agonist-treated and untreated mdx diaphragm muscle and wild-type diaphragm muscle

In experiment 2, 3 months of daily subcutaneous vehicle injection of mdx mice resulted in an approximate 20% loss of diaphragm specific force ; treatment with 30 ug/kg of PG-873637 blocked the loss of diaphragm specific force (p < 0.05 versus vehicle; Table 3 and Figure 3). Comparison of the specific force of the time 0 diaphragm from mdx mice to that of age-matched C57BL10 showed an approximate 50% loss in specific force (Table 3 and Figure 3). Analysis of additional diaphragm muscle parameters from experiment 2 did not demonstrate major differences between the mdx-treated groups and the age-matched C57BL10 controls (Table 3). Histopathological analysis of the diaphragm muscle from experiment 2 showed similar findings to those observed in experiment 1 (data not shown). The diaphragm muscle from experiment 2 was utilized for expression profiling analysis. For this analysis, gene expression profiles were obtained from mdx time 0, mdx 3 month vehicle-treated, mdx 3 month PG-873637-treated, C57BL10 time 0 and C57BL10 3 month vehicle-treated diaphragms. The number of statisticallysignificant gene expression changes for the various comparisons are as follows: mdx vehicle versus mdx time 0, 437 genes; mdx PG-873637 versus mdx vehicle, 683 genes; C57BL10 vehicle versus mdx vehicle, 4636 genes. Multiple comparisons were made between these groups with the following observations: the gene expression changes in common between mdx PG-873637-treated versus mdx vehicle-treated and mdx vehicle-treated and mdx time 0, 38 common genes; mdx PG-873637-treated versus C57BL10 vehicle-treated versus mdx vehicle-treated, 410 common genes. For this report, only the comparison of statistically significant changes in differential gene expression between mdx PG-873637-treated and mdx vehicle-treated is shown in Table 2 (also given in Table 2 are the differences in expression between mdx vehicle-treated versus mdx time 0, C57BL10 vehicle-treated versus mdx vehicle-treated, and C57BL10 vehicle-treated versus C57BL10 time 0 for comparison purposes). Comparisons between mdx vehicle-treated versus mdx time 0 and C57BL10 vehicle-treated versus mdx vehicle-treated are given in Additional files 1, 2, 3, 4. As can be seen in Additional file 1, differential gene expression between mdx PG-873637-treated and mdx vehicle-treated diaphragm was observed, with the genes demonstrating changes in expression in many different functional classes including signal transduction genes, proteolytic, extracellular matrix, protein synthesis, metabolism, cytoskeleton/contractile apparatus, transport/channels and genes of unknown function. Interestingly, most differential gene expression observed between mdx PG-873637 treatment versus mdx vehicle treatment were also observed between C57BL10 vehicle treatment versus mdx vehicle treatments, indicating a normalization of mdx muscle to a more wild-type phenotype. In addition, most differential gene expression changes between mdx PG-873637 treatment versus mdx vehicle treatment in metabolism-related genes were increases in expression, while those in the extracellular matrix-related genes were decreases in expression. Subgroup analysis (Additional file 2) showed that most changes in differential gene expression between mdx PG-873637 treatment versus mdx vehicle treatment groups were also observed in the C57BL10 vehicle treatment versus mdx vehicle treatment groups, including immune cell expressed genes, myofiber expressed genes and neuronal cell expressed genes. Interestingly, dystrophin complex-related genes showed differential expression for some but not all genes when mdx PG-873637 treatment versus mdx vehicle treatment was compared. In addition, an effect unique to PG-873637 treatment was observed in the circadian rhythm subgroup of genes; no changes in these genes were observed in mdx vehicle-treated versus C57BL10 vehicle-treated groups.

**Table 3:** Summary of diaphragm muscle parameters from experiment 2. Effect of 3 months of treatment with either vehicle or 30 ug/kg PG873637 on male 2-month-old (at time 0) mdx and C57BL10 mice.

	**Mdx time 0**	**Mdx Vehicle**	**Mdx PG-873637**	**C57BL10 time 0**	**C57BL10 vehicle**
Number of animals	10	9	11	10	10
Initial body mass (g)	21.90 (0.64)	22.43 (0.67)	22.32 (0.97)	24.21 (0.71)	24.69 (0.45)
Final body mass (g)	21.90 (0.64)	29.79† (0.50)	31.19† (0.63)	24.21 (0.71)	30.35† (0.66)
Diaphragm mass (g)	0.0027 (0.0002)	0.0040† (0.0003)	0.0043† (0.0002)	0.0023 (0.0001)	0.0027 (0.0002)
Diaphragm sPo (kN/m2)	120.12 (7.35)	91.90† (7.06)	112.58* (5.21)	210.22 (9.51)	196.49 (8.69)
Diaphragm Po (mN)	45.19 (5.92)	49.73 (5.46)	60.36† (3.09)	59.10 (3.75)	57.86 (3.98)
Diaphragm Pt (mN)	13.43 (1.72)	13.99 (1.40)	16.72 (1.05)	15.40 (1.04)	20.03* (1.51)
Diaphragm HRT (ms)	65.65 (2.75)	67.31 (4.42)	62.52 (2.49)	85.56 (3.44)	90.69 (6.27)
Diaphragm TPT (ms)	39.21 (1.31)	38.15 (1.26)	37.80 (0.56)	40.00 (0.86)	39.99 (1.14)

sPo, muscle specific force; Po, muscle absolute force; Pt, muscle peak twitch force; HRT, muscle half relaxation time; TPT, muscle time to peak tension. All data are given as the mean with standard error of the mean in parenthesis. *Statistically significant difference (p < 0.05) versus the appropriate vehicle control; † statistically significant difference (p < 0.05) versus time 0.

## Discussion

In this report, we demonstrate that treatment of mdx mice with a CRF2R agonist slows the loss of diaphragm specific force that occurs during disease progression. The effect of the CRF2R agonist is comparable to that observed with glucocorticoid (prednisone) treatment. In addition, treatment with the combination of a CRF2R agonist and glucocorticoid not only slowed the loss of diaphragm specific force associated with disease progression better than either agent by itself, but this combination treatment actually increased diaphragm specific force to a level observed before the start of the experiment. Histopathological analysis of CRF2R agonist-treated diaphragm showed a reduction in fibrosis, a reduction in inflammation, and increased myofiber cross-sectional area. Finally, gene expression changes showed that PG-873637 agonist treatment decreased extracellular matrix gene expression and reduced immune cell gene expression, findings that support the histopathological findings. Together, these observations indicate that treatment of mdx mice with a CRF2R agonist slows the disease progression in mdx mice.

The mechanism by which CRF2R agonist treatment slows disease progression in mdx mice is complex and involves changes in most cell types that comprise diaphragm muscle tissue. This includes: a direct effect on myofiber function, as evident from the increase in myofiber cross-sectional area and changes in myofiber gene expression; an effect on immune cell function that is evident from reduced inflammation and reduced immune cell specific gene expression in the treated mdx diaphragm; an effect on fibroblasts and other connective tissue cells resulting in reduced fibrosis and reduced extracellular matrix gene expression; and effects on neuronal cells that are evident from changes in neuronal cell specific gene expression. Comparison of the relative levels of differential gene expression in mdx diaphragm muscle following CRF2R agonist treatment with that of age-matched C57BL10 diaphragm muscle demonstrates that CRF2R agonist treatment reverts the mdx diaphragm to a more normal phenotype. In summary, the histopathological and gene expression changes resulting from CRF2R agonist treatment leads to an apparent normalization of mdx diaphragm muscle.

How does CRF2R agonist treatment affect mdx diaphragm function? In general, the data suggest that CRF2R agonist treatment has multiple effects on the mdx diaphragm muscle including increased myofiber cross-sectional area, decreased fibrosis and decreased inflammation, all of which probably contribute to the benefit observed. With regard to specific mechanisms involved in the above-described changes, several observations can be made from the gene expression analysis. Firstly, changes in expression of dystrophin complex-related genes; we did not observe changes in the expression of dystrophin or utrophin, nitric oxide synthase 1, dystroglycan 1 or dystrophin related protein 2 [21]. We did, however, observe increased expression of sarcoglycan alpha, sarcoglycan beta and dystrobrevin alpha. While these changes could result in increased functionality of the dystrophin complex, without the presence of dystrophin, it is difficult to understand how these changes lead to improved functionality. More work will be required in order to better understand whether the CRF2R agonist induced changes in dystrophin-related complex genes translate into increased protein and better functionality of the dystrophin complex. Secondly, analysis of changes in immune cell specific genes demonstrates that the majority of these genes show a decrease in expression following CRF2R agonist treatment. This observation, along with histopathological observation of decreased inflammation following CRF2R agonist treatment, supports the concept that CRF2R agonist treatment decreases immune cell activity in the diaphragm of mdx mice. Interestingly, three immune cell-related genes showed a relative increase in expression following CRF2R agonist treatment: small chemokine (C-C motif) ligand 11, interleukin 15 and nuclear factor of activated T-cells. Of these, interleukin-15 is particularly interesting as it has been shown by Harcourt et al [22] that interleukin 15 administration to mdx mice increases diaphragm function, increases diaphragm myofiber cross-sectional area and decreases fibrosis. Thus, increased expression of interleukin 15 following CRF2R agonist treatment could contribute to the efficacy of the CRF2R agonist treatment. Thirdly, in general extracellular matrix gene expression decreased following CRF2R agonist treatment. This observation, along with the histopathology analysis, supports the observation of decreased fibrosis associated with CRF2R agonist treatment. Two exceptions to the general trend of decreased extracellular matrix gene expression were noted – matrix metalloproteinase 24 and tissue inhibitor of metalloproteinase 3, both of which showed increased expression following CRF2R agonist treatment. The significance of these two changes is at present unknown, as two other matrix metalloproteinases (14 and 3) showed decreased expression, as did tissue inhibitor of metalloproteinase 1. These findings indicate that extracellular matrix proteins are downregulated. Fourthly, most metabolism-related genes showed increases in expression to levels similar to those observed in the C57BL10 muscle. Thus, CRF2R agonist treatment normalized metabolic function of the muscle to a more wild-type status, a change that probably contributes to improvement in overall muscle functionality. Finally, the most interesting group of gene expression changes associated with CRF2R activation were genes involved in the control of circadian rhythm. We observed that treatment of mdx mice with a CRF2R agonist increased the expression of the major circadian rhythm control genes including period homolog 2 (PER2), period homolog 3 (PER3), cryptochrome 2 (CRY2), thyrotroph embryonic factor (TEF), nuclear receptor subfamily 1, group D member 2 (NR1D2), RAR-related orphan receptor alpha (RORA), D site albumin promoter binding protein (DBP) genes and decreased the expression of circadian locomoter output cycles kaput (CLOCK) and aryl hydrocarbon receptor nuclear translocator-like (ARNTL/BMAL1/MOP3) genes. The CLOCK and ARNTL proteins function to activate expression of a variety of clock genes via binding to an E-box element, including PER, CRY, RORA and NR1D; PER and CRY proteins then feedback to decrease the expression of ARNTL and CLOCK [23-25]. This regulatory control loop agrees well with the observation of CRF2R agonist-mediated increased expression of PER 2, PER3, CRY2, DBP, RORA and NR1D2 and decreased expression of CLOCK and ARNTL. In addition to the changes in gene expression of these major circadian rhythm control genes, we also observed changes in the expression of other circadian rhythm associated genes including the RevErbA/ROR responsive element genes H3 histone, tubulin alpha, splicing factor arginine/serine-rich, protein tyrosine phosphatase type IVa, LPS-induced TNF-alpha factor, solute carrier family 25 and ADP-ribosylation factor; the DBP responsive element genes fibroblast growth factor and LPS-induced TNF-alpha factor; the E-box responsive element genes tubulin alpha, growth arrest and DNA damage inducible 45, adenylate kinase, solute carrier family, mitogen-activated protein kinase kinase; the cAMP responsive element genes dual specificity phosphatase, RNA binding motif protein, B-cell translocation gene, splicing factor arginine/serine-rich, protein tyrosine phosphatase type IVA, ADP-ribosylation-like factor 6 interacting protein and ADP-ribosylation factor; and additional genes such as glutathione S-transferase and UDP-glucose pyrophosphorylase [24-26]. The function of CRF2R activation of circadian rhythm genes in muscle is as present unknown. The known role of circadian rhythm in regulating metabolism [27-29] suggest that the changes in metabolic gene expression observed following CRF2R activation could be related to the change in circadian rhythm gene expression. Also, it is possible that the same changes that resulted in altered metabolic gene expression could change circadian rhythm gene expression, as it has been observed previously that changes in NAD and NADPH modify circadian rhythm gene expression [28,29]. Alternatively, CRF2R-mediated skeletal muscle circadian rhythm modulation could be secondary to the effects of CRF2R activation on food intake, as it is known that modulation of food intake can change circadian rhythm and CRF2R activation can decrease food intake [28,30]. The changes in circadian rhythm resulting from CRF2R activation could possibly be a result of cAMP modulation in skeletal muscle, as it has been shown previously that CRF2R activation in skeletal muscle increases cAMP levels [15] and modulation of intracellular cAMP (as well as calcium and PKC) can affect circadian rhythm gene expression [28]. Interestingly, other hormones associated with the hypothalamus-pituitary-adrenal (HPA) axis can potentially change circadian rhythm by acting as soluble factors to synchronize the circadian rhythm in peripheral tissues [28,31,32]. As the CRF2R is an integral part of the HPA axis, the observation that activation of the CRF2R modulates circadian rhythm gene expression in skeletal muscle indicates a further function of the HPA axis in the regulation of circadian rhythm. Obviously, more work will be required to clearly understand the mechanism by which activation of CRF2R can affect circadian rhythm gene expression.

## Conclusion

In summary, treatment of mdx mice with a CRF2R agonist results in a slowing of disease progression. The magnitude of this effect was equivalent to that observed with high-dose glucocorticoid administration. In addition, the combination of CRF2R agonist plus glucocorticoid not only slowed disease progression but also actually reversed the loss of specific force. Therefore, CRF2R agonists, such as glucocorticoids, IL-15 and myostatin, have been shown to have beneficial effects on diaphragm function in the mdx model of DMD.

## Competing interests

All authors of this manuscript performed the work described in this report as Procter & Gamble employees.

## Authors' contributions

RTH, FRL, ETD, DLR and JAD performed the animal work, the diaphragm muscle force analysis, and participated in data interpretation. KEG performed the histopathological analysis of the diaphragm. RIT performed the myofiber cross-sectional area analysis. JPD, PJS, XAQ, ARV and FW performed the transcriptional profiling, statistical, and bioinformatics analysis of the diaphragm. RJI conceived the study, participated in data interpretation, coordinated the overall project and drafted the manuscript. All authors read and approved the final manuscript.

## Pre-publication history

The pre-publication history for this paper can be accessed here:



## Supplementary Material

Additional file 1 **Differential gene expression changes, grouped by gene function, in mdx mice treated for 3 months with either vehicle or PG873637**. All differential genes showed statistically significant differences in expression (NLogP = 4.0). Click here for file

Additional file 2 **Tissue-specific subgroup analysis of differential gene expression changes in mdx mice treated for 3 months with either vehicle or PG873637**. All differential genes showed statistically significant differences in expression (NLogP = 4.0). Click here for file

Additional file 3 **Differential gene expression profile of mdx vehicle versus mdx time 0**. All differential genes showed statistically significant differences in expression (NLogP = 4.0). Click here for file

Additional file 4 **C57BL10 vehicle versus mdx vehicle**. All differential genes showed statistically significant differences in expression (NLogP = 4.0). Click here for file
