# Supplementary material for: Corticortophin releasing factor 2 receptor agonist treatment significantly slows disease progression in mdx mice
Source: BMC Med. 2007 Jul 12;5:18. doi: 10.1186/1741-7015-5-18 (PMC1936998; doi:10.1186/1741-7015-5-18)
Supplement: Additional file 1 — Differential gene expression changes, grouped by gene function, in mdx mice treated for 3 months with either vehicle or PG873637. All differential genes showed statistically significant differences in expression (NLogP = 4.0). [file 1741-7015-5-18-S1.doc]

# Additional files

### Additional File 1 – Differential gene expression changes in mdx and C57BL10 mice treated for 3 months with either vehicle or PG873637.

All differential genes demonstrated statistically significant differences in expression (NLogP=4.0).

| **Affy ID** | **Gene** | **mdx vehicle** versus **mdx time 0** | | **mdx PG873637** versus  **mdx vehicle** | | **C57BL10 vehicle** versus  **mdx vehicle** | **C57BL10 vehicle** versus. **C57BL10 time 0** |
| --- | --- | --- | --- | --- | --- | --- | --- |
| **Signal Transduction Genes** | | | | | | | |
| *Receptors and associated proteins* | | | | | | | |
| 1416958_AT | Nuclear receptor subfamily 1, group D, member 2 | 1.2 | | 2.3* | | 1.6 | 1.1 |
| 1421866_AT | Nuclear receptor subfamily 3, group C, member 1 | -1.1 | | 1.6* | | 1.5* | 1.1 |
| 1458129_AT | RAR-related orphan receptor alpha | 1.1 | | 1.2* | | 1.0 | -1.1 |
| 1418092_S_AT | Thyroid hormone receptor interactor 10 | 1.0 | | 1.4* | | 1.6* | 1.1 |
| 1417481_AT | Receptor activity modifying protein 1 | 1.1 | | 2.0* | | 1.8* | -1.1 |
| 1449433_AT | Purinergic receptor P2X | -1.1 | | 1.3* | | 1.2 | 1.0 |
| 1452353_AT | G protein coupled receptor 155 | -1.1 | | 1.5* | | 1.2 | 1.0 |
| 1455462_AT | Adenylate cyclase 2 | 1.0 | | 1.2* | | 1.5* | 1.1 |
| 1418663_AT | Multiple PDZ domain protein | 1.0 | | 1.3* | | 1.4* | 1.0 |
| 1420896_AT | Synaptosomal-associated protein 23 | -1.3* | | 1.2* | | 1.1 | -1.1 |
| 1422635_AT | Acetylcholinesterase | 1.0 | | 1.6* | | 1.4* | 1.1 |
| 1426462_AT | Gephyrin | 1.1 | | 1.2* | | 1.3* | 1.0 |
| 1426951_AT | Cysteine-rich motor neuron 1 | 1.1 | | 1.5* | | 2.1* | 1.2 |
| 1427191_AT | Natriuretic peptide receptor 2 | -1.1 | | 1.3* | | 1.6* | 1.0 |
| 1429607_AT | Amyotrophic lateral sclerosis 2 chromosome region, candidate 3 homolog | -1.3 | | 1.6* | | 1.8* | 1.1 |
| 1432281_A_AT | Integrin beta 6 | 1.0 | | 1.4* | | 1.1 | 1.0 |
| 1435016_AT | Amyotrophic lateral sclerosis 2, candidate 3 homolog | 1.0 | | 1.3* | | 1.3* | 1.0 |
| 1435472_AT | Kringle containing transmembrane protein 1 | -1.1 | | 1.4* | | 1.4* | 1.0 |
| 1438967_X_AT | Anti-Mullerian hormone type 2 receptor | 1.0 | | 1.3* | | 1.6* | 1.1 |
| 1451871_A_AT | Growth hormone receptor | 1.1 | | 1.3* | | 1.3* | 1.1 |
| 1454137_S_AT | RGM domain family, member C | 1.1 | | 1.2* | | 1.3* | 1.0 |
| 1454824_S_AT | Mitochondrial tumor suppressor 1 | 1.1 | | 1.2* | | 1.5* | 1.1 |
| 1416010_A_AT | EH-domain containing 1 | 1.0 | | -1.3* | | 1.0 | 1.1 |
| 1417185_AT | Lymphocyte antigen 6 complex, locus A | 1.3* | | -1.3* | | -1.1 | -1.1 |
| 1417378_AT | Immunoglobulin superfamily, member 4A | 1.4* | | -1.3* | | -1.5* | -1.1 |
| 1418571_AT | Tumor necrosis receptor superfamily, member 12a | -1.3 | | -3.9* | | -3.0* | -1.7 |
| 1418674_AT | Oncostatin M receptor | 1.2 | | -1.4* | | -1.4* | 1.0 |
| 1450020_AT | Chemokine (C-X3-C) receptor 1 | -1.4 | | -1.9* | | -3.4* | 1.0 |
| 1454783_AT | Interleukin 13 receptor, alpha 1 | 1.1 | | -1.5* | | -2.3* | -1.1 |
| 1455899_X_AT | Suppressor of cytokine signaling 3 | 1.5 | | -2.1* | | -3.4* | -1.4 |
| 1448891_AT | Macrophage scavenger receptor 2 | -1.1 | | -1.5* | | -1.7* | 1.1 |
| 1419272_AT | Myeloid differentiation primary response gene 88 | 1.0 | | -1.4* | | -1.8* | -1.1 |
| 1420895_AT | Transforming growth factor, beta receptor I | 1.0 | | -1.2* | | -1.8* | 1.0 |
| 1422818_AT | Neural precursor cell expressed, developmentally down regulated gene 9 | -1.1 | | -1.5* | | 1.2 | 1.1 |
| 1423955_A_AT | Longevity assurance homolog 2 | 1.0 | | -1.3* | | -1.3* | -1.2 |
| 1434153_AT | Src homology 2 domain containing transforming protein B | -1.1 | | -1.4* | | -1.4* | 1.1 |
| 1437279_X_AT | Syndecan 1 | 1.3 | | -1.7* | | -2.2* | -1.1 |
| 1448793_A_AT | Syndecan 4 | 1.1 | | -1.3* | | -1.2 | 1.0 |
| 1437308_S_AT | Coagulation factor II (thrombin) receptor | 1.1 | | -1.3* | | -1.3* | 1.1 |
| 1450295_S_AT | Poliovirus receptor | -1.1 | | -1.5* | | -2.1* | -1.1 |
| 1450757_AT | Cadherin 11 | -1.2 | | -1.5* | | -1.1 | -1.3 |
| 1450792_AT | TYRO protein tyrosine kinase binding protein | 1.1 | | -1.5* | | -6.1* | -1.1 |
| 1451350_A_AT | Leptin receptor gene related protein | 1.1 | | -1.3* | | -1.4* | -1.1 |
| 1449556_AT | Histocompatibility 2, T region locus 23 | 1.2 | | -1.4* | | -1.5* | 1.1 |
| 1451721_A_AT | Histocompatibility 2, class II antigen A, beta 1 | 1.4 | | -1.7* | | -3.1* | 1.1 |
| 1451784_X_AT | Histocompatibility 2, D region | 1.3 | | -1.4* | | -1.3 | 1.2 |
| 1452431_S_AT | Histocompatibility 2, class II antigen A, alpha | 1.3 | | -1.6* | | -3.0* | 1.3 |
|  | | | | | | | |
| *Growth and differentiation factors* | | | | | | | |
| 1416658_AT | Frizzled-related protein | -1.5* | | 1.8* | | 1.7* | -1.3 |
| 1417190_AT | Pre-B-cell colony-enhancing factor 1 | 1.1 | | 1.4* | | 1.4* | 1.1 |
| 1417789_AT | Small chemokine (C-C motif) ligand 11 | 1.1 | | 2.0* | | 3.0* | 1.4 |
| 1418093_A_AT | Epidermal growth factor | 1.0 | | 1.5* | | 1.4* | -1.1 |
| 1418219_AT | Interleukin 15 | 1.2 | | 2.1* | | 3.0* | 1.5 |
| 1418498_AT | Fibroblast growth factor 13 | 1.0 | | 1.4* | | 1.1 | 1.0 |
| 1422912_AT | Bone morphogenetic protein 4 | -1.2 | | 1.5* | | 1.2 | -1.3 |
| 1423454_A_AT | Sema domain, transmembrane domain and cytoplasmic domain (semaphoring) 6C | 1.0 | | 1.4* | | 1.1 | -1.1 |
| 1427747_A_AT | Lipocalin 2 | 1.1 | | 2.8* | | -1.3 | -1.3 |
| 1434735_AT | Hepatic leukemia factor | 1.1 | | 1.7* | | 1.5* | 1.0 |
| 1449528_AT | c-fos induced growth factor (VEGF-D) | 1.0 | | 1.5* | | 1.4* | 1.1 |
| 1448607_AT | Pre B-cell colony enhancing factor 1 | 1.1 | | 1.5* | | 1.8* | 1.3 |
| 1460242_AT | Decay accelerating factor 1 | 1.0 | | 1.8* | | -1.1 | -1.1 |
| 1416953_AT | Connective tissue growth factor | 2.7* | | -1.6* | | -1.5* | 1.0 |
| 1418350_AT | Diptheria toxin receptor (HB-EGF) | 1.0 | | -1.9* | | -1.4* | -1.2 |
| 1423783_AT | Torsin family 2,member A | -1.1 | | -1.2* | | -1.4* | -1.1 |
| 1448316_AT | Chemokine like factor super family 3 | -1.1 | | -1.2* | | -1.6* | 1.0 |
| 1449195_S_AT | Chemokine (C-X-C motif) ligand 16 | 1.3 | | -1.8* | | -3.9* | -1.1 |
| 1460187_AT | Secreted frizzled related sequence protein 1 | 1.1 | | -1.5* | | -1.9* | -1.3 |
|  |  |  | |  | |  |  |
| *Kinases and associated proteins* | | | | | | | |
| 1419169_AT | Mitogen activated protein kinase 6 | -1.1 | | -1.2* | | -1.6* | -1.1 |
| 1422615_AT | Mitogen activated protein kinase kinase kinase kinase 4 | -1.2 | | -1.3* | | -1.5* | -1.1 |
| 1419766_AT | SNF1-like kinase | 1.3 | | -1.6* | | -1.1 | 1.1 |
| 1459601_AT | SNF1-like kinase | 2.7* | | -2.1* | | 1.5 | 1.4 |
| 1434082_AT | PCTAIRE-motif protein kinase 2 | 1.0 | | -1.3* | | -1.1 | 1.0 |
| 1437132_X_AT | Neural precursor cell expressed, developmentally down regulated gene 9 | 1.1 | | -1.8* | | 1.0 | 1.0 |
| 1448370_AT | Unc-51 like kinase 1 | 1.1 | | -1.2* | | 1.2* | 1.0 |
| 1452478_AT | Heart alpha-kinase | -1.2 | | -1.6* | | 1.8* | 1.1 |
| 1420858_AT | Protein kinase inhibitor, alpha | 1.0 | | 1.2* | | 1.6* | 1.1 |
| 1422254_A_AT | Dual specificity tyrosine phosphorylation regulated kinase 1b | 1.0 | | 1.4* | | 1.2 | -1.1 |
| 1422743_AT | Phosphorylase kinase alpha 1 | 1.0 | | 1.6* | | 1.6* | -1.1 |
| 1424511_AT | Phosphorylase kinase beta | 1.2 | | 1.3* | | 1.3* | 1.1 |
| 1426044_A_AT | Protein kinase C theta | 1.1 | | 1.3* | | 1.2* | 1.1 |
| 1426850_A_AT | Mitogen activated protein kinase kinase 6 | -1.1 | | 2.0* | | 1.5* | 1.0 |
| 1449283_A_AT | Mitogen activated protein kinase 12 | 1.0 | | 1.3* | | 1.2* | -1.1 |
| 1427414_AT | Protein kinase, cAMP dependent regulatory, type II alpha | -1.2 | | 1.3* | | 1.7* | -1.1 |
| 1444232_AT | Protein kinase, cGMP dependent type I | 1.0 | | 2.2* | | 2.2* | 1.1 |
| 1434766_AT | Protein kinase, AMP-activated, alpha 2 catalytic subunit | 1.1 | | 1.4* | | 1.4* | 1.1 |
| 1435874_AT | Protein kinase, AMP-activated, beta 2 non-catalytic subunit | 1.2 | | 1.3* | | 1.0 | 1.1 |
| 1435769_AT | A kinase anchor protein 9 | 1.0 | | 1.2* | | 1.0 | 1.0 |
| 1440859_AT | A kinase anchor protein 6 | 1.1 | | 1.2* | | 1.2* | 1.1 |
| 1434348_AT | Fasciculation and elongation protein zeta 2 | -1.1 | | 1.3* | | 1.5* | 1.0 |
| 1441111_AT | Similar to myosin light chain kinase | -1.2 | | 3.1* | | 3.5* | -1.2 |
| 1448864_AT | SNF related kinase | 1.0 | | 1.3* | | 1.7* | 1.1 |
| 1450113_AT | Membrane protein, palmitoylated 5 (MAGUK 5) | 1.1 | | 1.5* | | 1.3 | 1.1 |
|  |  |  | |  | |  |  |
| *Phosphatases/ phosphodiesterases/associated proteins* | | | | | | | |
| 1417068_A_AT | Protein tyrosine phosphatase, non-receptor type 1 | 1.1 | | -1.4* | | -1.7* | 1.0 |
| 1417801_A_AT | Protein tyrosine phosphatase, receptor type, F interacting protein binding protein 2 | 1.2 | | -1.5* | | 1.1 | 1.0 |
| 1422124_A_AT | Protein tyrosine phosphatase, receptor type, C | 1.1 | | -1.4* | | -3.6* | 1.2 |
| 1422473_AT | Phosphodiesterase 4B, cAMP specific | 1.4 | | -1.9* | | 1.0 | 1.1 |
| 1455105_AT | Protein tyrosine phosphatase, non-receptor type 12 | 1.1 | | -1.3* | | -1.5* | 1.0 |
| 1438657_X_AT | Protein tyrosine phosphatase 4a1 | 1.2 | | -1.3* | | 1.1 | 1.1 |
| 1420612_S_AT | Protein tyrosine phosphatase 4a2 | 1.0 | | 1.2* | | -1.1 | -1.1 |
| 1438012_AT | Protein phosphatase 1-like | 1.1 | | 1.5* | | 1.6* | 1.1 |
| 1448908_AT | Phosphatidic acid phosphatase type 2B | -1.1 | | 1.3* | | 1.3* | 1.0 |
| 1452485_AT | Phosphatase, orphan 1 | -1.3 | | 1.4* | | 1.9* | 1.0 |
| 1453074_AT | Dual specificity phosphatase 23 | 1.1 | | 1.2* | | 1.3* | 1.0 |
| 1453127_AT | Protein phosphatase 2a, catalytic subunit, zeta isoform | -1.3 | | 1.6* | | 1.3 | 1.1 |
|  |  |  | |  | |  |  |
| *Transcription factors, transcription related* | | | | | | | |
| 1416065_A_AT | Ankyrin repeat domain 10 | 1.1 | | -1.7* | | -2.5* | -1.1 |
| 1428444_AT | Ankyrin repeat and SOCS box-containing protein 2 | -1.1 | | -1.5* | | 1.7* | 1.0 |
| 1417244_A_AT | Interferon regulatory factor 7 | -1.1 | | -1.6* | | -1.9* | -1.1 |
| 1416067_AT | Interferon-related developmental regulator 1 | 1.0 | | -1.5* | | 1.1 | 1.1 |
| 1423796_AT | Splicing factor proline/glutamine rich | -1.1 | | -1.4* | | -1.6* | -1.1 |
| 1428099_A_AT | Splicing factor, arginine/serine rich 1 | 1.0 | | -1.3* | | -1.4* | 1.0 |
| 1448454_AT | Splicing factor, arginine/serine rich 6 | -1.1 | | -1.2* | | -1.3* | 1.0 |
| 1454993_A_AT | Splicing factor, arginine/serine rich 3 | -1.1 | | -1.6* | | -1.3* | -1.1 |
| 1450986_AT | Nucleolar protein 5 | -1.1 | | -1.3* | | -1.8* | 1.1 |
| 1451184_AT | Heterogeneous nuclear ribonucleoprotein A3 | -1.1 | | -1.2* | | -1.4* | 1.0 |
| 1456664_X_AT | Heterogenous nuclear ribonucleoprotein F | 1.1 | | -1.2* | | -1.4* | 1.0 |
| 1417400_AT | Retinoic acid induced 14 | 1.1 | | -1.4* | | -1.7* | -1.1 |
| 1417483_AT | Nuclear factor of kappa light polypeptide gene enhancer inB-cells inhibitor, zeta | 1.3 | | -1.4* | | -1.2 | 1.0 |
| 1418133_AT | B-cell leukemia/lymphoma 3 | -1.2 | | -1.6* | | -2.1* | -1.3 |
| 1418420_AT | Myogenic differentiation 1 | -1.2 | | -1.6* | | -2.9* | -1.1 |
| 1419391_AT | Myogenin | 1.0 | | -1.6* | | -12.6* | -1.4 |
| 1420473_AT | Myotrophin | -1.1 | | -1.2* | | -1.3* | -1.1 |
| 1419642_AT | Purine rich element binding protein B | 1.0 | | -1.2* | | -1.4* | -1.1 |
| 1423484_AT | Bicaudal c homolog 1 | 1.2 | | -1.3* | | -1.6* | -1.2 |
| 1426587_A_AT | Signal transducer and activator of transcription 3 (STAT3) | 1.1 | | -1.3* | | -1.3* | -1.1 |
| 1429088_AT | Limb bud and heart | 1.0 | | -1.3* | | -2.0* | -1.2 |
| 1433575_AT | SRY-box containing gene 4 | 1.0 | | -1.6* | | -1.9* | -1.2 |
| 1435176_A_AT | Inhibitor of DNA binding 2 | 1.1 | | -1.7* | | -3.3* | 1.0 |
| 1436050_AT | Hairy and enhancer of split 6 | 1.0 | | -1.4* | | -2.2* | 1.0 |
| 1437239_X_AT | Polyhomeotic-like 2 | -1.1 | | -1.3* | | -1.3* | -1.1 |
| 1437247_AT | Fos-like antigen 2 | 1.2 | | -1.8* | | -1.4* | 1.0 |
| 1437503_A_AT | Scotin gene | 1.0 | | -1.3* | | -1.6* | -1.1 |
| 1438368_A_AT | Matrin 3 | 1.1 | | -1.2* | | -1.3* | 1.0 |
| 1448208_AT | MAD homolog 1 | 1.0 | | -1.4* | | -1.4* | 1.0 |
| 1454960_AT | MAD homolog 3 | 1.1 | | -1.6* | | -1.1 | -1.2 |
| 1425099_A_AT | Aryl hydrocarbon receptor nuclear translocator-like | -1.1 | | -3.4* | | -1.1 | 1.0 |
| 1418660_AT | Circadian locomoter output cycles kaput | 1.0 | | -1.7* | | -1.2 | -1.1 |
| 1417602_AT | Period homolog 2 | 1.2 | | 5.1* | | 1.8 | 1.9 |
| 1418174_AT | D site albumin promoter binding protein | 1.4 | | 9.2* | | 2.1 | 1.3 |
| 1426383_AT | Cryptochrome 2 | 1.1 | | 1.4* | | 1.1 | 1.1 |
| 1424175_AT | Thyrotroph embryonic factor | 1.2 | | 2.2* | | 1.2 | 1.1 |
| 1417889_AT | Apolipoprotein B editing complex 2 | 1.1 | | 1.3* | | 1.2 | -1.2 |
| 1418467_AT | SWI/SNF related matrix associated actin dependent regulator of chromatin, subfamily d,member 3 | -1.1 | | 1.3* | | 1.3* | -1.1 |
| 1419743_S_AT | Coactivator-associated arginine methyltransferase 1 | -1.1 | | 1.2* | | 1.2* | 1.0 |
| 1421028_A_AT | Myocyte enhancer factor 2C | 1.2 | | 1.4* | | -1.2 | 1.1 |
| 1421087_AT | Period homolog 3 | 1.4 | | 3.9* | | 1.4 | 1.0 |
| 1421265_A_AT | RNA-binding region (RNP1, RRM) containing | 1.0 | | 1.5* | | 1.6* | 1.0 |
| 1421466_AT | Ankyrin repeat and SOCS box containing protein 10 | 1.1 | | 1.3* | | 1.6* | 1.0 |
| 1424225_AT | Ankyrin repeat and SOCS box containing protein 8 | -1.1 | | 1.3* | | 1.3* | 1.1 |
| 1422195_S_AT | T box 15 | 1.1 | | 1.4* | | 1.1 | -1.1 |
| 1423540_AT | RNA binding motif, single stranded interacting protein 2 | -1.2* | | 1.2* | | 1.4* | 1.1 |
| 1424531_A_AT | Transcription elongation factor A, 3 | 1.0 | | 1.3* | | 1.8* | 1.1 |
| 1424667_A_AT | Cut-like 1 | -1.1 | | 1.3* | | 1.3* | -1.1 |
| 1424797_A_AT | Paired-like homeodomain transcription factor 2 | 1.1 | | 1.7* | | 1.1 | 1.4 |
| 1425527_AT | Paired related homeobox 1 | -1.1 | | 1.3* | | 1.1 | 1.1 |
| 1427361_AT | Homeobox C6 | -1.1 | | 1.4* | | 1.4* | 1.0 |
| 1437637_AT | Putative homeodomain transcription factor 2 | 1.1 | | 1.4* | | 1.4* | 1.0 |
| 1438042_AT | Short stature homeobox 2 | 1.1 | | 1.5* | | 1.6* | 1.1 |
| 1425533_A_AT | Staufen homolog 2 | 1.0 | | 1.4* | | 1.4* | 1.1 |
| 1425599_A_AT | Ocular development associated gene | -1.1 | | 1.2* | | 1.2* | 1.1 |
| 1425990_A_AT | Nuclear factor of activated T-cells, cytoplasmic, calcineurin dependent 2 (induces IL-4 expression in myocytes for differentiation) | 1.0 | | 1.7* | | -1.3 | 1.0 |
| 1430519_A_AT | CCR4-NOT transcription complex, subunit 7 | -1.2 | | 1.4* | | 1.4* | 1.0 |
| 1431890__A_AT | Myeloid/lymphoic or mixed lineage-leukemia translocation to 3 homolog | 1.1 | | 1.4* | | 1.2 | 1.1 |
| 1434735_AT | Hepatic leukemia factor | 1.1 | | 1.7* | | 1.5* | 1.0 |
| 1434938_AT | RNA binding motif protein 9 | 1.0 | | 1.2* | | 1.0 | 1.1 |
| 1438294_AT | Spinocerebellar ataxia 1 homolog | 1.0 | | 1.4* | | 1.3* | -1.1 |
| 1454670_AT | Arginine glutamic acid dipeptide repeats | 1.1 | | 1.3* | | 1.2* | 1.0 |
| 1456785_AT | Cofactor required for Sp1 transcriptional activation subunit 2 | 1.0 | | 1.3* | | 1.4* | 1.1 |
| 1456796_AT | Snail homolog 3 | 1.1 | | 5.8* | | 1.3 | 1.3 |
| 1460246_AT | Methyl CpG binding protein 2 | 1.1 | | 1.3* | | 1.1 | 1.0 |
|  |  |  | |  | |  |  |
| *G-proteins and associated proteins* | | | | | | | |
| 1416794_AT | ADP-ribosylation factor-like 6 interacting protein 2 | 1.0 | | 1.5* | | 1.5* | 1.1 |
| 1419553_A_AT | RAB geranylgeranyl transferase, b subunit | 1.0 | | 1.2* | | 1.3* | 1.0 |
| 1421146_AT | Rap guanine nucleotide exchange factor (GEF) 1 | -1.2 | | 1.4* | | 1.7* | 1.2 |
| 1422919_AT | HRAS-like suppressor | 1.0 | | 1.3* | | 2.3* | 1.1 |
| 1431164_AT | Ras-related GTP binding D | -1.1 | | 1.5* | | 1.5* | 1.3 |
| 1435292_AT | TBC1 domain family, member 4 | 1.1 | | 1.2* | | 1.6* | 1.1 |
| 1450899_AT | Neural precursor cell expressed developmentally down-regulated gene 1 | -1.1 | | 1.5* | | 1.5* | 1.0 |
| 1451553_AT | ADP-ribosyltransferase 5 | 1.3* | | 1.3* | | 1.2 | 1.1 |
| 1429206_AT | Rho related BTB domain containing 1 | 1.2 | | 1.3* | | 1.6* | 1.2 |
| 1435674_AT | Rho-related BTB domain containing 2 | 1.0 | | 1.2* | | 1.1 | 1.0 |
| 1433682_AT | Rho guanine nucleotide exchange factor 17 | -1.1 | | 1.4* | | 1.1 | 1.0 |
| 1424842_A_AT | Rho GTPase activating protein 24 | 1.1 | | -1.3* | | -1.2 | 1.0 |
| 1451309_AT | Rho GTPase activating protein | 1.3 | | -2.0* | | -5.1* | -1.1 |
| 1416897_AT | Poly (ADP-ribose) polymerase family, member 9 | 1.0 | | -1.3* | | -1.3* | 1.0 |
| 1418822_AT | ADP-ribosylation factor 6 | 1.1 | | -1.4* | | -1.8* | 1.1 |
| 1422562_AT | Ras-related associated with diabetes | 1.2 | | -2.4* | | -6.7* | 1.8 |
| 1454060_A_AT | Neuroblastoma ras oncogene | -1.1 | | -1.2* | | -1.6* | -1.1 |
| 1448885_AT | RAP2B, member of RAS oncogene family | -1.1 | | -1.3* | | -1.7* | 1.0 |
| 1450656_AT | Guanine nucleotide binding protein, alpha 13 | 1.0 | | -1.3* | | -1.3* | 1.0 |
|  |  |  | |  | |  |  |
| *Calcium signaling* | | | | | | | |
| 1417421_AT | S100 calcium binding protein A1 | 1.1 | | 1.3* | | 1.4* | 1.1 |
| 1419394_S_AT | S100 calcium binding protein A8 (calgranulin A) | -1.1 | | 68.4* | | -1.2 | 1.2 |
| 1419109_AT | Histidine rich calcium binding protein | 1.1 | | 1.2* | | 1.3* | 1.1 |
| 1423359_AT | Phospholamban | -1.2 | | 2.0* | | 1.7* | 1.2 |
| 1454611_A_AT | Calmodulin 1 | 1.0 | | -1.2* | | -1.1 | 1.0 |
|  |  |  | |  | |  |  |
| *Cell cycle* | | | | | | | |
| 1421679_A_AT | Cyclin-dependent kinase inhibitor 1A (P21) | 1.1 | | -2.0* | | -3.1* | 1.0 |
| 1448272_AT | B-cell translocation gene 2, anti-proliferative | 2.1* | | -1.5* | | 1.5* | 1.0 |
| 1449007_AT | B-cell translocation gene 3 | -1.1 | | -1.3* | | -1.2 | -1.1 |
| 1449519_AT | Growth arrest and DNA damage inducible 45 alpha | 2.0* | | -1.6* | | -1.8* | 1.1 |
| 1421887_A_AT | Amyloid beta (A4) precursor-like protein 2 | 1.0 | | 1.2* | | 1.3* | 1.0 |
| 1422624_AT | REV1-like | 1.0 | | 1.3* | | 1.2 | 1.0 |
| 1422795_AT | Cullin 3 | -1.1 | | 1.2* | | 1.6* | 1.1 |
| 1425743_AT | CDK2 associated protein 1 | -1.2 | | 1.4* | | 1.3* | -1.2 |
| 1448204_AT | Salvador homolog 1 | -1.1 | | 1.3* | | 1.1 | -1.1 |
| 1450016_AT | Cyclin G1 | 1.0 | | 1.3* | | 1.6* | 1.0 |
|  |  |  | |  | |  |  |
| *Apoptosis* | | | | | | | |
| 1417956_AT | Cell death inducing DNA fragmentation factor, alpha subunit like effector A | -1.2 | | 1.8* | | 1.7* | 1.2 |
| 1451503_AT | Nucleolar protein 3 | 1.1 | | 1.2* | | 1.0 | 1.0 |
| 1460718_S_AT | Mitochondrial carrier homolog 1 | -1.2 | | 1.6* | | -1.1 | -1.1 |
| 1450997_AT | Serine/threonine kinase 17b (apoptosis-inducing) | 1.2 | | -1.5* | | -2.4* | 1.1 |
| 1449839_AT | Caspase 3 | 1.0 | | -1.4* | | -2.5* | 1.0 |
|  |  |  | |  | |  |  |
| *Others* | | | | | | | |
| 1415877_AT | Dihydropyrimidinase-like 3 | -1.1 | | -1.5* | | -2.0* | -1.2 |
| 1416178_A_AT | Pleckstrin homology domain containing, family B member 1 | 1.0 | | 1.8* | | 2.1* | 1.2 |
| 1416529_AT | Epithelial membrane protein 1 | 1.0 | | -1.4* | | -2.0* | -1.2 |
| 1416600_A_AT | Down syndrome critical region homolog 1 | 1.1 | | -1.7* | | -1.3* | -1.1 |
| 1417381_AT | Complement component 1, q subcomponent, alpha polypeptide | 1.3 | | -1.5* | | -3.7* | -1.1 |
| 1417848_AT | Glucocorticoid induced gene 1 | 1.2 | | -1.3* | | -1.4* | -1.1 |
| 1417928_AT | PDZ and LIM domain 4 | -1.1 | | -1.4* | | -2.2* | -1.3 |
| 1418501_A_AT | Oxidation resistance 1 | 1.1 | | 1.2* | | 1.2* | 1.1 |
| 1419309_AT | Glycoprotein 38 | 1.1 | | -1.4* | | -1.9* | -1.2 |
| 1419365_AT | Peroxisomal biogenesis factor 11a | 1.0 | | 1.3* | | 1.7* | 1.1 |
| 1419963_AT | DEP domain containing 6 | -1.1 | | 1.7* | | 1.3* | -1.1 |
| 1425241_A_AT | WD repeat and SOCS box containing 1 | 1.0 | | -1.7* | | -1.3 | 1.1 |
| 1425331_AT | Zinc finger protein 106 | -1.1 | | 1.3* | | 1.5* | 1.1 |
| 1426719_AT | Amyloid beta (A$) precursor protein binding family B, member 2 | 1.0 | | 1.5* | | 1.4* | 1.0 |
| 1426906_AT | Interferon activated gene 203 | 1.1 | | -1.4* | | -1.6* | 1.0 |
| 1427064_A_AT | Scribbled homolog | -1.1 | | -1.4* | | -2.0* | 1.0 |
| 1428622_AT | DEP domain containing 6 | 1.1 | | 1.3* | | 1.0 | 1.0 |
| 1434219_AT | Stromal interaction molecule 2 | 1.0 | | -1.3* | | -1.5* | 1.0 |
| 1434928_AT | Growth arrest specific 2 like 1 | 1.1 | | -1.2* | | -1.3* | -1.1 |
| 1435066_AT | Phosphatidylinositol transfer protein, cytoplasmic 1 | -1.1 | | 1.4* | | 1.7* | -1.1 |
| 1435697_A_AT | Pleckstrin homology, sec& and coiled-coil domain, binding protein | 1.7* | | -1.6* | | -2.5* | 1.0 |
| 1437226_X_AT | MARCKS-like protein | -1.3 | | -1.6* | | -2.3* | -1.4 |
| 1437502_X_AT | CD24a antigen | 1.2 | | 1.6* | | -1.1 | 1.3 |
| 1456700_X_AT | MARCKS | 1.0 | | -1.4* | | -1.9* | -1.2 |
| 1438168_X_AT | DEAD box peptide 39 | -1.1 | | -1.3* | | -1.9* | 1.0 |
| 1440435_AT | Kyphoscoliosis | 1.3 | | 2.7* | | 1.2 | 1.0 |
| 1448005_AT | SAM and SH3 domain containing 1 | 1.2 | | -1.3* | | -1.1 | 1.0 |
| 1449303_AT | Sestrin 3 | 1.1 | | -1.4* | | -1.4* | 1.0 |
| 1449315_AT | Odd Oz/ten-m homolog 3 | 1.0 | | -1.4* | | -1.2* | 1.0 |
| 1449442_AT | Peroxisomal biogenesis factor11a | 1.0 | | 1.3* | | 1.7* | 1.0 |
| 1453622_S_AT | Myeloid/lymphoid or mixed lineage leukemia translocation to 3 homolog | 1.1 | | 1.5* | | 1.3* | 1.1 |
|  | | | | | | | |
| **Proteolysis** | | | | | | | |
| 1417168_A_AT | Ubiquitin specific protease 2 | | 1.2 | | 1.7* | 1.2 | -1.1 |
| 1419278_AT | Ubiquitin specific protease 48 | | 1.0 | | 1.2* | 1.1 | 1.1 |
| 1435325_AT | Ubiquitin specific protease 46 | | -1.2 | | 1.3* | 1.6* | 1.0 |
| 1454036_A_AT | Ubiquitin specific protease 15 | | 1.0 | | 1.2* | 1.5* | 1.0 |
| 1418318_AT | Ring finger protein 128 | | 1.1 | | 1.7* | 1.2 | -1.2 |
| 1419440_AT | Ring finger protein 30 | | 1.0 | | 1.4* | 1.8* | 1.2 |
| 1449036_AT | Ring finger protein 128 | | 1.1 | | 1.7* | 1.3* | -1.1 |
| 1434565_AT | Cell growth regulator with ring finger domain 1 | | -1.1 | | 1.3* | 1.3* | -1.1 |
| 1426824_AT | Proteasome activator subunit 4 | | 1.0 | | 1.2* | 1.1 | 1.0 |
| 1419495_AT | Inner mitochondrial membrane peptidase 2-like | | 1.1 | | 1.2* | 1.3* | 1.1 |
| 1423098_AT | Calpain 7 | | -1.1 | | 1.3* | 1.3* | 1.0 |
| 1428442_AT | Matrix metalloproteinase 24 | | 1.0 | | 1.3* | 1.1 | 1.1 |
| 1449334_AT | Tissue inhibitor of metalloproteinase 3 | | 1.1 | | 1.5* | 2.5* | 1.1 |
| 1432385_A_AT | ATP/GTP binding protein | | -1.3 | | 2.4* | 2.7* | 1.2 |
| 1453988_A_AT | Insulin degrading enzyme | | -1.1 | | 1.3* | 1.4* | 1.1 |
| 1450138_A_AT | Serine (or cysteine) proteinase inhibitor, clade B, member 6a | | 1.3* | | 1.4* | -3.5* | 1.1 |
| 1416625_AT | Serine (or cysteine) proteinase inhibitor, clade G, member 1 | | 1.2 | | -1.3* | -1.3* | -1.1 |
| 1416048_AT | Polyhomeotic-like 2 | | -1.2 | | -1.3* | -1.3* | -1.1 |
| 1416303_AT | LPS-induced TN factor | | 1.0 | | -1.3* | -1.5* | 1.0 |
| 1416572_AT | Matrix metalloproteinase 14 | | -1.2 | | -1.5* | -2.1* | -1.1 |
| 1418945_AT | Matrix metalloproteinase 3 | | 3.3* | | -2.2* | -5.0* | 1.3 |
| 1460227_AT | Tissue inhibitor of metalloproteinase 1 | | 1.1 | | -1.9* | -8.1* | -1.3 |
| 1425974_A_AT | Tripartitie motif protein 25 | | -1.1 | | -1.4* | -1.6* | -1.1 |
| 1451860_A_AT | Tripartite motif protein 30 | | 1.0 | | -1.3* | -1.5* | -1.1 |
| 1459860_X_AT | Tripartite motif protein 2 | | 1.1 | | -1.3* | -1.3* | -1.2 |
| 1429399_AT | Ring finger protein 125 | | 1.4 | | -1.7* | 1.1 | 1.2 |
| 1448632_AT | Proteasome subunit beta type 10 | | 1.0 | | -1.3* | -1.4* | 1.0 |
| 1436905_X_AT | Lysosomal associated protein transmembrane 5 | | 1.0 | | -1.4* | -4.3* | -1.1 |
| 1451791_AT | Tissue factor pathway inhibitor | | -1.1 | | -1.3* | -1.8* | -1.2 |
| 1460248_AT | Carboxypeptidase X2 | | 1.1 | | -1.4* | -1.4* | 1.1 |
|  | | | | | | | |
| **Metabolism** | | | | | | | |
| 1416230_AT | Riboflavin kinase | | 1.0 | | 1.2* | 1.3* | 1.0 |
| 1416409_AT | Acyl-coenzyme A oxidase 1, palmitoyl | | 1.1 | | 1.3* | 1.5* | 1.2 |
| 1416416_AT | Glutathione S-transferase, mu 1 | | 1.0 | | 1.3* | 2.1* | 1.1 |
| 1416665_AT | Demethyl Q7 | | 1.0 | | 1.2* | 1.4* | 1.0 |
| 1416737_AT | Glycogen synthase 3, brain | | 1.1 | | 1.3* | 1.2* | 1.0 |
| 1416842_AT | Glutathione S-transferase, mu 5 | | -1.1 | | 1.2* | 1.3* | 1.0 |
| 1417303_AT | Mevalonate decarboxylase | | 1.2 | | 1.9* | -1.2 | 1.2 |
| 1417716_AT | Glutamate oxaloacetate transaminase 2, mitochondrial | | -1.1 | | 1.3* | 1.5* | 1.0 |
| 1418472_AT | Aspartoacylase 2 | | 1.3* | | 1.4* | 1.0 | 1.1 |
| 1418888_A_AT | Selenoprotein X1 | | 1.2 | | 1.4* | -1.2 | -1.2 |
| 1419367_AT | 2,4-dienoyl CoA reductase 1, mitochondrial | | 1.0 | | 1.3* | 1.9* | 1.1 |
| 1419382_A_AT | Dehydrogenase/reductase member 4 | | -1.2 | | 1.4* | 2.3* | 1.1 |
| 1419428_A_AT | Glucosidase, alpha, acid | | 1.0 | | 1.2* | 1.3* | 1.1 |
| 1420654_A_AT | Glucan (1,4-alpha), branching enzyme 1 | | -1.1 | | 1.4* | 1.9* | 1.0 |
| 1422465_A_AT | Nucleoredoxin | | 1.1 | | 1.2* | -1.1 | 1.0 |
| 1422538_AT | Exotoses like 2 | | -1.1 | | 1.3* | 1.2 | 1.0 |
| 1422612_AT | Hexokinase 2 | | 1.1 | | 1.3* | 1.7* | 1.2 |
| 1422678_AT | Diacylglycerol O-acyltransferase 2 | | -1.2 | | 1.3* | 2.6* | 1.1 |
| 1422780_AT | Peroxisomal membrane protein 4 | | 1.0 | | 1.2* | 1.4* | 1.0 |
| 1422904_AT | Flavin containing monooxygenase 2 | | 1.1 | | 1.5* | 1.8* | -1.2 |
| 1423394_AT | Prenylcysteine oxidase 1 | | -1.1 | | 1.2* | 1.1 | 1.0 |
| 1424048_A_AT | NAD(P)H:quinine oxidoreductase type 3, polypeptide A2 | | 1.0 | | 1.2* | -1.3* | -1.1 |
| 1424453_AT | Phosphate cytidylyltransferase 1, choline, alpha isoform | | -1.1 | | 1.4* | 1.4* | 1.0 |
| 1425834_A_AT | Glycerol-3-phosphate acyltransferase, mitochondrial | | -1.3 | | 2.0* | 2.0* | 1.1 |
| 1429019_S_AT | Paraoxonase 2 | | -1.1 | | 1.2* | 1.0 | 1.0 |
| 1430896_S_AT | Nudix-type motif 7 | | -1.2 | | 1.4* | 1.8* | 1.0 |
| 1434485_A_AT | UDP-glucose pyrophosphorylase 2 | | 1.1 | | 1.2* | 1.4* | 1.1 |
| 1434511_AT | Phosphorylase kinase beta | | 1.2 | | 1.3* | 1.3* | 1.1 |
| 1434934_AT | ATP synthase mitochondrial F1 complex assembly factor 1 | | -1.1 | | 1.3* | 1.9* | 1.1 |
| 1435459_AT | Flavin containing monoxygenase 2 | | 1.1 | | 1.9* | 2.5* | -1.1 |
| 1438011_AT | Phosphate cytidylyltransferase 1, choline, alpha isoform | | 1.0 | | 1.3* | 1.3* | 1.1 |
| 1439029_AT | Glutamic pyruvate transaminase 2 | | -1.2 | | 1.5* | 1.8* | 1.0 |
| 1447277_S_AT | Prenylcysteine oxidase 1 | | 1.0 | | 1.2* | 1.1 | 1.0 |
| 1448244_AT | Lysophospholipase 1 | | 1.1 | | 1.2* | 1.3* | 1.0 |
| 1448499_A_AT | Epoxide hydrolase 2, cytoplasmic | | -1.1 | | 1.6* | 3.2* | 1.1 |
| 1448530_AT | Guanosine monophosphate reductase | | 1.0 | | 1.4* | 1.5* | -1.1 |
| 1448602_AT | Muscle glycogen phosphorylase | | 1.0 | | 1.2* | 1.4* | 1.0 |
| 1448663_S_AT | Mevalonate decarboxylase | | -1.3 | | 3.7* | -1.2 | 1.2 |
| 1448825_AT | Pyruvate dehydrogenase kinase, isoenzyme 2 | | 1.0 | | 1.2* | 1.8* | 1.1 |
| 1449078_AT | Sialyltransferase 10 | | 1.1 | | 1.2* | 1.4* | 1.1 |
| 1449137_AT | Pyruvate dehydrogenase E1 alpha 1 | | -1.1 | | 1.3* | 1.5* | 1.0 |
| 1449442_AT | Peroxisomal biogenesis factor11a | | 1.0 | | 1.3* | 1.7* | 1.0 |
| 1450196_S_AT | Glycogen synthase 1, muscle | | 1.0 | | 1.4* | 1.4* | 1.0 |
| 1450970_AT | Glutmate oxaloacetate transaminase 1, soluble | | 1.0 | | 1.2* | 1.5* | 1.1 |
| 1451050_AT | 5”-nucleotidase, cytosolic III | | 1.0 | | 1.3* | 1.2* | -1.1 |
| 1451149_AT | Phosphoglucomutase 2 | | 1.0 | | 1.3* | 1.4* | -1.2 |
| 1451274_AT | Oxoglutarate dehydrogenase | | 1.0 | | 1.2* | 1.5* | 1.1 |
| 1451559_A_AT | Dehydrogenase/reductase member 4 | | -1.2 | | 1.4* | 2.2* | 1.1 |
| 1451742_A_AT | UDP-glucose pyrophosphorylase 2 | | 1.1 | | 1.3* | 1.4* | 1.0 |
| 1452346_AT | UDP-GlcNAc:betaGal beta-1,3-N-acetylglucaosaminyltransferase 6 | | 1.0 | | 1.2* | 1.0 | 1.0 |
| 1460360_AT | Asparaginase like 1 | | 1.0 | | 1.3* | 1.5* | 1.0 |
| 1460256_AT | Carbonic anhydrase 3 | | 1.2 | | 2.2* | 1.5 | -1.3 |
| 1460319_AT | Fucosyltransferase 8 | | 1.0 | | 1.4* | -1.1 | -1.1 |
| 1418252_AT | Peptidyl arginine deiminase, type II | | -1.1 | | -1.9* | 1.8* | 1.4 |
| 1426959_AT | 3-hydroxybutyrate dehydrogenase (mitochondrial) | | 1.0 | | -1.5* | 2.8* | 1.3 |
| 1435133_AT | UDP-glucose ceramide glucosyltransferase | | 1.1 | | -1.3* | -1.7* | 1.0 |
| 1436905_X_AT | Lysosomal-associated protein transmembrane 5 | | 1.0 | | -1.4* | -2.3* | -1.1 |
| 1438386_X_AT | Methionine adenosyltransferase II, alpha | | -1.1 | | -1.2* | -1.3* | -1.1 |
| 1439012_A_AT | Deoxycytidine kinase | | -1.1 | | -1.6* | -2.8* | -1.1 |
| 1448558_A_AT | Phospholipase A2, group IVA | | 1.1 | | -1.4* | -1.7* | -1.2 |
|  | | | | | | | |
| **Transport/Channels** | | | | | | | |
| 1416671_A_AT | Mucolipin 1 | | 1.0 | | 1.2* | 1.2* | 1.0 |
| 1426219_AT | Sterol carrier protein 2, liver | | 1.0 | | 1.3* | 1.2* | 1.0 |
| 1450964_A_AT | Oxysterol binding protein-like 9 | | 1.0 | | 1.3* | 1.2* | 1.0 |
| 1433464_AT | Importin 13 | | 1.0 | | 1.2* | 1.4* | 1.0 |
| 1460197__A_AT | Tumor necrosis factor, alpha-induced protein 9 | | 1.1 | | -1.3* | 1.1 | -1.1 |
| 1415958_AT | Solute carrier family 2 member 4 (insulin-regulated facilitative glucose transporter | | 1.0 | | 1.6* | 1.9* | 1.1 |
| 1420148_AT | Solute carrier family 6, member 6 | | -1.4 | | 1.7* | 1.7* | 1.5 |
| 1450311_AT | Solute carrier family 8, member 3 | | 1.7* | | 1.8* | -1.3 | 1.2 |
| 1452976_A_AT | Solute carrier family 9, isoform 3 regulator 2 | | -1.4* | | 1.3* | 2.1* | 1.0 |
| 1448780_AT | Solute carrier family 12, member 2 | | 1.1 | | 1.5* | 1.4* | 1.2 |
| 1429727_AT | Solute carrier family 16, member 9 | | 1.0 | | -1.8* | 1.1 | 1.0 |
| 1448568_A_AT | Solute carrier family 20, member 1 | | 1.0 | | -1.5* | -1.3* | 1.2 |
| 1451768_A_AT | Solute carrier family 20, member 2 | | -1.1 | | 1.4* | 1.1 | 1.0 |
| 1426586_AT | Solute carrier family 25 (mitochondrial), member 11 | | -1.1 | | 1.3* | 1.5* | 1.0 |
| 1428440_AT | Solute carrier family 25 (mitochondrial), member 12 | | 1.0 | | 1.2* | 1.4* | -1.1 |
| 1452059_AT | Solute carrier family 35, member F5 | | 1.1 | | -1.2* | -1.8* | -1.1 |
| 1426722_AT | Solute carrier family 38, member 2 | | -1.2 | | -1.4* | -1.2 | -1.2 |
| 1420442_AT | Calcium channel, voltage dependent, L type, alpha 1S subunit | | 1.0 | | 1.2* | 1.6* | 1.0 |
| 1450952_AT | Phospholamban | | 1.3 | | 1.8* | 1.6 | 1.5 |
| 1416610_A_AT | Chloride channel 3 | | 1.1 | | 1.5* | 1.8* | 1.1 |
| 1438606_A_AT | Chloride intracellular channel 4 (mitochondrial) | | 1.0 | | -1.3* | 1.0 | 1.1 |
| 1423936_AT | Potassium channel tetramerisation domain containing 5 | | 1.1 | | -1.3* | -1.3* | 1.0 |
| 1435342_AT | Potassium inwardly-rectifying channel, subfamily K, member 6 | | -1.1 | | -1.3* | -1.4* | 1.0 |
| 1427426_AT | Potassium voltage-gated channel, subfamily Q, member 5 | | 1.4 | | 1.5* | -1.2 | 1.0 |
| 1450490_AT | Potassium voltage-gated channel, shaker related subfamily, member 7 | | 1.2 | | 1.4* | 1.9* | 1.0 |
| 1450557_AT | Sodium channel, voltage gated, type IV, alpha polypeptide | | 1.0 | | 1.3* | 1.7* | 1.1 |
| 1418738_AT | Sodium channel, voltage gated, type I, beta polypeptide | | 1.1 | | 1.3* | 1.1 | 1.0 |
| 1420408_A_AT | ATP-binding cassette, subfamily C (CFTR/MRP), member 9 | | -1.1 | | 1.4* | 1.7* | 1.0 |
| 1427490_AT | ATP-binding cassette subfamily B, member 7 | | 1.0 | | 1.4* | 1.3* | 1.0 |
| 1419748_AT | ATP-binding cassette, subfamily D, member 2 | | -1.2 | | 1.5* | 1.5* | -1.1 |
| 1422906_AT | ATP-binding cassette, subfamily G, member 2 | | 1.2 | | 1.3* | 1.0 | 1.1 |
| 1434893_AT | ATPase, Na+/K+ transporting, alpha 2 | | 1.1 | | 1.2* | 1.4* | 1.0 |
| 1451152_A_AT | ATPase, Na+/K+ transporting beta 1 | | 1.0 | | 1.3* | 1.4* | 1.1 |
| 1422009_AT | ATPase, Na+/K+ transporting beta 2 | | 1.0 | | 1.6* | -1.1 | -1.2 |
|  | | | | | | | |
| **Cytoskeleton/Contractile Apparatus** | | | | | | | |
| 1416226_AT | Actin related protein 2/3 complex, subunit 1B | | -1.1 | | -1.3* | -2.2* | -1.2 |
| 1452051_AT | ARP3 actin related protein 3 homolog | | 1.0 | | -1.3* | -1.8* | 1.0 |
| 1416514_A_AT | Fascin homolog 1, actin bundling protein | | -1.2 | | -1.4* | -2.0* | -1.3 |
| 1424770_AT | Caldesmon 1 | | -1.1 | | -1.3* | -1.1 | -1.1 |
| 1427567_A_AT | Tropomyosin 3 gamma | | -1.1 | | -1.3* | -1.6* | -1.1 |
| 1418884_X_AT | Tubulin, alpha 1 | | -1.1 | | -1.4* | -2.9* | -1.3 |
| 1423846_X_AT | Tubulin, alpha 2 | | -1.1 | | -1.2* | -2.7* | -1.1 |
| 1439455_X_AT | Capping protein muscle Z line, alpha 1 | | -1.1 | | -1.3* | -1.8* | -1.2 |
| 1424269_A_AT | Myosin light polypeptide 6, alkali, smooth muscle and nonmuscle | | -1.1 | | -1.2* | -1.5* | -1.1 |
| 1428266_AT | Myosin light polypeptide 3 | | 1.0 | | -1.4* | 1.8* | 1.1 |
| 1456380_X_AT | Calponin 3, acidic | | 1.0 | | -1.6* | -3.0* | -1.2 |
| 1436902_X_AT | Thymosin beta 10 | | -1.1 | | -1.4* | -5.7* | -1.3 |
| 1419220_AT | Cardiomyopathy associated 1 | | 1.0 | | -1.7* | -1.1 | 1.0 |
| 1423725_AT | Plastin 3 | | 1.0 | | -1.3* | 1.0 | 1.0 |
| 1448169_AT | Keratin complex 1, acidic, gene 18 | | 1.3 | | -1.9* | -1.9* | -1.1 |
| 1450732_A_AT | Bicaudal D homolog 2 | | 1.1 | | -1.2* | -1.2* | 1.0 |
| 1418289_AT | Nestin | | 1.1 | | -1.5* | -1.7* | 1.0 |
| 1452929_AT | Restin | | 1.2 | | 1.3* | 1.3* | 1.1 |
| 1454749_AT | Pericentrin 2 | | 1.1 | | 1.3* | 1.5* | 1.2 |
| 1422654_AT | Sarcoglycan alpha (dystrophin associated glycoprotein) | | 1.1 | | 1.5* | 1.3* | 1.0 |
| 1419667_AT | Sarcoglycan, beta | | -1.2 | | 1.3* | 1.1 | 1.0 |
| 1426066_A_AT | Dystrobrevin alpha | | 1.1 | | 1.4* | -1.2 | 1.0 |
| 1423145_A_AT | Titin cap | | 1.2 | | 1.4* | 1.4* | 1.1 |
| 1431928_AT | Titin | | 1.1 | | 1.3* | 1.4* | 1.1 |
| 1450917_AT | Myomesin 2 | | 1.2 | | 1.3* | -1.7* | -1.2 |
| 1419056_AT | Reticulon 2 (Z-band associated protein) | | 1.1 | | 1.2* | 1.3* | 1.0 |
| 1416840_AT | Mid1 interacting protein 1 | | 1.1 | | 1.8* | 1.0 | 1.1 |
| 1419739_AT | Tropomyosin 2, beta | | 1.0 | | 1.2* | 1.2* | -1.1 |
| 1425270_AT | Kinesin family member 1B | | 1.0 | | 1.3* | 1.1 | -1.1 |
| 1426245_S_AT | Microtubule-associated protein RP/EB family, member 2 | | 1.0 | | 1.2* | 1.2* | 1.0 |
| 1451290_AT | Microtubule-associated protein 1 light chain 3 alpha | | 1.1 | | 1.5* | -1.3 | -1.1 |
| 1452345_AT | Leiomodin 2 | | 1.3 | | 1.4* | 1.1 | 1.0 |
|  | | | | | | | |
| **Protein Synthesis/Protein Folding** | | | | | | | |
| 1426645_AT | Heat shock protein 1, alpha | | -1.1 | | -1.3* | -1.6* | 1.0 |
| 1434986_A_AT | Sec61 alpha 1 subunit | | -1.2 | | -1.4* | -1.9* | -1.2 |
| 1441682_S_AT | Exportin tRNA | | 1.0 | | -1.2* | -1.3* | -1.1 |
| 1416603_AT | Ribosomal protein L22 | | 1.3 | | -1.5* | -1.8* | 1.0 |
| 1449398_AT | Ribosomal protein L3-like | | 1.1 | | 1.3* | 1.6* | -1.1 |
| 1423220_AT | Eukaryotic translation initiation factor 4E | | -1.1 | | 1.4* | 1.7* | 1.0 |
| 1455690_AT | Eukaryotic translation elongation factor 1 alpha 2 | | -1.1 | | 1.3* | 1.2 | -1.1 |
|  | | | | | | | |
| **Extracellular Matrix/Extracellular** | | | | | | | |
| 1415935_AT | SPARC related modular calcium binding 2 | | 1.2* | | -1.2* | -1.7* | -1.1 |
| 1418424_AT | Tumor necrosis factor alpha induced protein 6 | | 1.4 | | -1.5* | -1.2 | 1.1 |
| 1418511_AT | Dermatopontin | | 1.2 | | -1.3* | -2.0* | -1.2 |
| 1421694_A_AT | Chondroitin sulfate proteoglycan 2 | | -1.3 | | -1.8* | -3.9* | -1.3 |
| 1421811_AT | Thrombospondin 1 | | 1.5 | | -1.9* | 1.0 | 1.2 |
| 1421855_AT | Fibrinogen-like protein 2 | | 1.6* | | -1.5* | -1.6* | -1.1 |
| 1424041_S_AT | Complement component 1, s subcomponent | | 1.3* | | -1.4* | -1.3* | -1.1 |
| 1437726_X_AT | Complement component 1, q subcomponent, beta polypeptide | | 1.2 | | -1.5* | -4.1* | -1.1 |
| 1449401_AT | Complement component 1, q subcomponent, gamma polypeptide | | 1.2 | | -1.5* | -3.5* | -1.1 |
| 1448380_AT | Lectin, galactoside-binding, soluble 3, binding protein | | -1.1 | | -1.4* | -1.4* | -1.1 |
| 1448416_AT | Matrix gamma-carboxyglutamate (Gla) protein | | 1.3* | | -1.3* | -2.5* | -1.2 |
| 1449289_A_AT | Beta 2 microglobulin | | 1.2 | | -1.4* | -1.3* | 1.1 |
| 1451119_A_AT | Fibulin 1 | | 1.0 | | -1.3* | 1.1 | -1.1 |
| 1452209_AT | Plakophilin 4 | | 1.1 | | -1.3* | 1.2 | 1.0 |
| 1455978_A_AT | Matrilin 2 | | 1.2 | | -1.8* | -2.3* | -1.4 |
| 1416572_AT | Matrix metalloproteinase 14 | | -1.2 | | -1.5* | -2.1* | -1.1 |
| 1418945_AT | Matrix metalloproteinase 3 | | 3.3* | | -2.2* | -5.0* | 1.3 |
| 1428442_AT | Matrix metalloproteinase 24 | | 1.0 | | 1.3* | 1.1 | 1.1 |
| 1460227_AT | Tissue inhibitor of metalloproteinase 1 | | 1.1 | | -1.9* | -8.1* | -1.3 |
| 1449334_AT | Tissue inhibitor of metalloproteinase 3 | | 1.1 | | 1.5* | 2.5* | 1.1 |
|  | | | | | | | |
| **Unknown Function** | | | | | | | |
| 1415721_A_AT | RIKEN cDNA 1200013P24 gene | | -1.1 | | 1.3* | 1.2 | 1.0 |
| 1415806_AT | Gb:NM_008872.1/DB_XREF=gi:6679374?GEN=PLAT/FEA= | | 1.0 | | -1.5* | -1.5* | -1.1 |
| 1415882_AT | Growth hormone inducible transmembrane protein | | 1.0 | | 1.2* | 1.3* | 1.1 |
| 1415961_AT | Integral membrane protein 2C | | 1.0 | | -1.2* | -1.7* | -1.1 |
| 1416034_AT | CD24a antigen | | 1.0 | | 1.8* | 1.1 | 1.3 |
| 1416165_AT | Riken cDNA 1700093E07 | | 1.0 | | -1.4* | -2.7* | -1.2 |
| 1416431_AT | RIKEN cDNA 2310057H16 | | -1.2 | | -1.6* | -8.1* | 1.0 |
| 1416766_AT | RIKEN cDNA 2810484M10 | | 1.0 | | 1.3* | 1.2* | 1.0 |
| 1417383_AT | Gb:BB810113/DB_XREF=gi:16982742/DB_XREF=BB810113 | | -1.2 | | 1.6* | 1.8* | 1.1 |
| 1417400_AT | Retinoic acid induced 14 | | 1.1 | | -1.4* | -1.7* | -1.1 |
| 1417460_AT | Interferon induced transmembrane protein 2 | | 1.0 | | -1.2* | -1.3* | -1.1 |
| 1417768_AT | Riken cDNA 1200006O19 | | 1.0 | | 1.2* | 1.3* | 1.1 |
| 1417807_AT | RIKEN cDNA 2700038N03 | | 1.0 | | 1.4* | 1.3* | 1.0 |
| 1418580_AT | Gb:NM_023386.1/DB_XREF=gi:12963656/GEN=5830458K1 | | -1.3 | | -1.5* | -1.3 | -1.2 |
| 1418952_AT | RIKEN cDNA 2310001N14 | | 1.0 | | 1.3* | 1.8* | 1.2 |
| 1419352_AT | RIKEN cDNA 0610007P06 | | -1.1 | | 1.2* | 1.3* | 1.0 |
| 1419499_AT | Gb:NM_008149.1/DB_XREF=gi:6680056?GEN=Gpam/FEA+ | | 1.0 | | 1.5* | 1.5* | 1.0 |
| 1419657_A_AT | RIKEN cDNA C330005L02 gene | | -1.1 | | -1.4* | -1.1 | 1.0 |
| 1420136_A_AT | Gb:AI427540/DB_XREF=gi:4273466/DB_XREF=mm09d03.x | | -1.2 | | 1.7* | 1.4* | 1.0 |
| 1420329_AT | RIKEN cDNA 4930455C21 gene | | -1.1 | | -1.3* | 1.0 | -1.1 |
| 1420542_AT | Open reading frame 28 | | -1.3 | | 1.4* | 2.1* | 1.1 |
| 1421815_AT | Expressed sequence AU040950 | | -1.2 | | 1.3* | 1.5* | 1.0 |
| 1421922_AT | Gb:BC018237.1/DB_XREF=gi:17390546/FEA=FLmTNA/CN | | -1.2 | | -1.5* | 1.5* | 1.0 |
| 1421923_AT | Gb:BQ179335/DB_XREF=gi:20354814/DB_XREF=UI-M-EWO | | 1.0 | | -1.8* | 1.3 | 1.0 |
| 1422466_AT | Gb:BB366804/DB_XREF=gi:16406472/DB_XREF=BB366804 | | 1.0 | | 1.4* | -1.1 | -1.1 |
| 1422565_S_AT | Gb:BF785921/DB_XREF=gi:12090957/DB_XREF=60211255 | | 1.1 | | 1.4* | 1.1 | -1.1 |
| 1422595_S_AT | RIKEN cDNA 5730470L24 | | 1.0 | | -1.2* | -1.3* | -1.1 |
| 1422644_AT | RIKEN cDNA 5430437A18 gene | | 1.1 | | 1.2* | 1.1 | 1.0 |
| 1422705_AT | Transmembrane, prostate androgen induced RNA | | -1.1 | | -1.5* | -1.8* | -1.1 |
| 1423042_AT | Fibroblast growth factor inducible 14 | | 1.1 | | -1.2* | 1.0 | 1.0 |
| 1423306_AT | RIKEN cDNA 201002N04 gene | | 1.1 | | -1.3* | -1.6* | 1.0 |
| 1423754_AT | Interferon induced transmembrane protein 3 | | 1.0 | | -1.4* | -1.4* | -1.1 |
| 1423851_A_AT | RIKEN cDNA 9430059P22 | | 1.0 | | 1.7* | 1.1 | 1.0 |
| 1423905_AT | DNA segment, Chr 7, ERATO Doi 458, expressed | | -1.1 | | -1.4* | -1.8* | 1.0 |
| 1423965_AT | MIC2-like 1 | | 1.1 | | 1.2* | 1.0 | 1.0 |
| 1423997_AT | DNA segment, Chr 3, MJeffers 1 | | 1.1 | | 1.2* | 1.3* | 1.1 |
| 1424039_AT | RIKEN cDNA 1810045K07 | | 1.1 | | 1.2* | 1.2* | 1.0 |
| 1424052_AT | THAP domain containing 4 | | -1.1 | | 1.3* | 1.4* | 1.0 |
| 1424177_AT | RIKEN cDNA 1110001E17 | | 1.1 | | 1.3* | 1.3* | 1.0 |
| 1424194_AT | cDNA sequence BC025872 | | 1.1 | | 1.4* | 1.3* | 1.0 |
| 1424318_AT | RIKEN cDNA 1110067D22 | | -1.1 | | 1.5* | 2.1* | -1.1 |
| 1424505_AT | RIKEN cDNA 0610042C05 gene | | -1.1 | | 1.2* | 1.6* | 1.0 |
| 1424520_AT | RIKEN cDNA 2010305A19 gene | | -1.3* | | 1.4* | 1.5* | -1.1 |
| 1424700_AT | DNA segment, Chr 4, ERATO Doi 89, expressed | | 1.0 | | 1.3* | -1.1 | -1.1 |
| 1424760_A_AT | SET and MYND domain containing 2 | | 1.0 | | -1.3* | 1.0 | 1.0 |
| 1424790_AT | RIKEN cDNA 2900084M01 gene | | 1.0 | | 1.3* | 1.7* | 1.0 |
| 1424904_AT | Gb:BC019143.1/DB_XREF=gi:17512347/FEA=FLmRNA/CN | | 1.0 | | 1.2* | 1.5* | 1.1 |
| 1425020_AT | UBX domain containing 4 | | 1.1 | | 1.2* | 1.5* | 1.1 |
| 1425274_AT | Gb:AF221854.1/DB_XREF=gi:12655824/FEA=FLmRNA/CN | | 1.0 | | -1.5* | -1.2 | -1.3 |
| 1425275_AT | Gb:AF289490.1/DB_XREF=gi:11878117/FEA=FLmRNA/CN | | -1.1 | | -1.5* | 1.0 | -1.3 |
| 1425327_AT | cDNA sequence BC008163 | | 1.0 | | 1.3* | 1.2* | 1.0 |
| 1425344_AT | RIKEN cDNA 4430402O11 gene | | -1.3 | | 1.6* | 1.5* | -1.1 |
| 1425529_S_AT | DNA segment, Chr19, Wayne State University 162, expressed | | 1.1 | | -1.4* | 1.0 | 1.1 |
| 1425616_A_AT | RIKEN cDNA 2410005K17 | | 1.0 | | -1.3* | -2.0* | -1.1 |
| 1425784_A_AT | Olfactomedin 1 | | -1.4 | | -1.8* | -1.8* | -1.3 |
| 1426112_A_AT | Gb:BC003824.1/DB_XREF=gi:3277905/FEA=FLmRNA/CN | | 1.2 | | -1.7* | -2.3* | -1.1 |
| 1426371_AT | Male sterility domain containing 2 | | -1.1 | | -1.3* | -1.5* | 1.0 |
| 1427074_AT | RIKEN cDNA 5330414D10 | | 1.1 | | 1.2* | 1.2* | 1.1 |
| 1427312_AT | Cardiomyopathy associated 5 | | 1.1 | | 1.2* | 1.4* | 1.1 |
| 1427347_S_AT | Gb:BC003475.1/DB_XREF=gi:13097482/FEA=mRNA/CNT= | | -1.3 | | -1.6* | -4.4* | -1.3 |
| 1427769_X_AT | Gb:X67685.1/DB_XREF=gi:53299/GEN=Mylc/FEA=mRNA | | 1.0 | | -1.5* | 1.8* | 1.1 |
| 1428128_AT | RIKEN cDNA 4921506J03 | | -1.1 | | -1.2* | -1.2* | -1.1 |
| 1428136_AT | RIKEN cDNA 2210415K03 gene | | 1.2 | | -1.5* | -2.1* | -1.3 |
| 1428183_AT | Gb:BG793483?DB_XREF=gi:14129054/DB_XREF=UTSW_SM1 | | 1.1 | | -1.5* | 1.6* | 1.2 |
| 1428342_AT | REST corepressor 3 | | 1.0 | | 1.3* | 1.3* | 1.1 |
| 1428347_AT | Gb:AK005148.1/DB_XREF=gi:12836871/FEA=mRNA/CNT= | | 1.2 | | 1.9* | -6.8* | 1.4 |
| 1428513_AT | RIKEN cDNA 1810009B06 | | 1.0 | | 1.2* | 1.3* | 1.1 |
| 1428696_AT | RIKEN cDNA 2310015N21 gene | | -1.2 | | -1.3* | 1.1 | -1.1 |
| 1429081_AT | RIKEN cDNA 2600014C01 | | 1.1 | | 1.2* | 1.3* | 1.1 |
| 1429107_AT | RIKEN cDNA 1110059H15 | | 1.1 | | 1.2* | 1.2* | 1.0 |
| 1429122_A_AT | RIKEN cDNA 1700040I03 gene | | -1.1 | | 1.3* | 1.2 | 1.0 |
| 1429148_AT | RIKEN cDNA 1110019L22 gene | | 1.1 | | 1.3* | 1.0 | -1.1 |
| 1429159_AT | RIKEN cDNA 4631408O11 gene | | 1.2 | | -1.3* | -1.5* | -1.1 |
| 1429205_AT | RIKEN cDNA 2610012I03 | | 1.0 | | 1.5* | 1.5* | 1.3 |
| 1429458_AT | Gb.AV083806/DB_XREF=gi.15404348/DB_XREF=AV083806 | | 1.0 | | 1.3* | 1.9* | 1.0 |
| 1429622_AT | RIKEN cDNA 2210404G23 | | 1.0 | | 1.3* | 1.3* | 1.0 |
| 1429652_AT | RIKEN cDNA 1190002C06 gene | | 1.0 | | -1.2* | -1.1 | 1.0 |
| 1429678_AT | RIKEN cDNA 5730508B09 gene | | -1.1 | | 1.4* | 1.2 | -1.1 |
| 1430125_S_AT | PQ loop repeat containing 1 | | 1.1 | | -1.6* | -1.3 | -1.1 |
| 1430176_AT | RIKEN cDNA 5430433E21 gene | | -1.2 | | 1.4* | 1.3 | -1.2 |
| 1430596_S_AT | RIKEN cDNA 1700110N18 | | -1.1 | | -1.5* | -1.5* | -1.1 |
| 1431018_AT | RIKEN cDNA 3010002C02 gene | | -1.1 | | 1.4* | 1.2 | 1.1 |
| 1431213_A_AT | Gb:BG297038/DB_XREF=gi:13060290/DB_XREF=60239489 | | -1.3 | | 2.0* | 1.7 | -1.4 |
| 1431382_A_AT | RIKEN cDNA 1700024K14 gene | | -1.1 | | 1.3* | 1.6* | 1.1 |
| 1432107_AT | RIKEN cDNA 2310010M20 | | 1.3 | | 1.9* | 2.6* | 1.0 |
| 1432198_AT | Gb:AK018172.1/DB_XREF=gi:12857769/FEA=mRNA/CNT= | | -1.1 | | 1.6* | -6.3* | 1.3 |
| 1432332_A_AT | DNA segment, Chr 7, Roswell Park 2 complex, expressed | | -1.1 | | 1.2* | 1.3* | 1.0 |
| 1432420_A_AT | RIKEN cDNA 2310002L09 gene | | -1.2 | | 1.3* | 2.0* | -1.1 |
| 1433599_AT | cDNA sequence BC065123 | | 1.2 | | -1.4* | -2.2* | 1.0 |
| 1433735_A_AT | RIKEN cDNA 9630015D15 gene | | -1.1 | | 1.3* | 1.2 | 1.0 |
| 1433819_S_AT | Gb:AV296997/DB_XREF=gi:15405860 | | 1.0 | | -1.5* | 1.0 | 1.0 |
| 1433834_AT | RIKEN cDNA F830029L24 | | 1.1 | | 1.2* | 1.4* | 1.0 |
| 1433842_AT | Gb:BG069059/DB_XREF=gi:12551628/DB_XREF=H3072C08 | | 1.1 | | -1.6* | -2.2* | -1.1 |
| 1433928_A_AT | Gb:BF146301/DB_SREF=gi.11027696/DB_XREF=uy27b03 | | 1.0 | | -1.3* | -2.7* | -1.1 |
| 1434072_AT | Gb:AV299469/DB_XREF=gi:16393162/DB_XREF=AV299469 | | 1.0 | | 1.2* | 1.3* | 1.0 |
| 1434133_S_AT | WD repeat domain 42A | | 1.1 | | 1.2* | 1.3* | 1.1 |
| 1434184_S_AT | RIKEN cDNA 9430080K19 | | 1.1 | | -1.5* | -2.1* | -1.2 |
| 1434219_AT | Stromal interacting molecule 2 | | 1.0 | | -1.3* | -1.5* | 1.0 |
| 1434228_AT | Gene model 1024 (NCBI) | | 1.2 | | 1.3* | 1.0 | 1.1 |
| 1434339_AT | RIKEN cDNA 2610318I01 gene | | -1.1 | | -1.3* | 1.4* | 1.0 |
| 1434647_AT | Expressed sequence AU040377 | | -1.3 | | 1.6* | 2.3* | 1.0 |
| 1434671_AT | RIKEN cDNA B230337E12 gene | | 1.0 | | -1.3* | 1.4* | -1.1 |
| 1434738_AT | RIKEN cDNA A530046H20 | | -1.1 | | 1.4* | 1.6* | 1.0 |
| 1434870_AT | RIKEN cDNA 0610039J04 | | 1.1 | | 1.2* | 1.3* | 1.1 |
| 1434909_AT | RIKEN cDNA C030003H22 | | 1.1 | | 1.4* | 1.2* | 1.0 |
| 1434960_AT | cDNA sequence BC066223 | | -1.1 | | 1.4* | 1.3 | 1.2 |
| 1435013_AT | Similar to hypothetical proteinFLJ20397 | | -1.1 | | 1.3* | 1.2 | 1.1 |
| 1435207_AT | DIX domain containing 1 | | -1.1 | | 1.5* | 1.3 | 1.0 |
| 1435504_AT | Gb:BM217861/DB_XREF=gi:17777216/DB_XREF=C0903B02 | | 1.1 | | 1.2* | 1.5* | 1.1 |
| 1435527_AT | RIKEN cDNA 1500041O16 gene | | 1.1 | | 1.3* | 1.0 | -1.1 |
| 1435542_S_AT | Gb:BB231897/DB_XREF=gi:16354491/DB_XREF=BB231897 | | 1.1 | | -1.3* | -1.4* | 1.0 |
| 1435567_AT | Gb:A1504378/DB_XREF=gi:4402229/DB_XREF=vl09b03.x | | 1.1 | | 1.4* | 1.6* | -1.1 |
| 1435589_AT | Expressed sequence A1842788 | | 1.0 | | 1.5* | 1.1 | 1.1 |
| 1435653_AT | Gb:BG070904/DB_XREF=gi:2553396/DB_XREF=H3091E12 | | 1.0 | | -1.3* | -1.6* | -1.1 |
| 1435763_AT | TBC1 domain family, member 16 | | -1.1 | | 1.3* | 1.5* | 1.0 |
| 1435775_AT | Gb:BQ173970/DB_XREF=gi:20349461/DB_XREF=UI-M-DJ2 | | 1.1 | | -1.6* | -1.2 | -1.1 |
| 1435784_AT | Expessed sequence AU019532 | | 1.0 | | 1.2* | 1.3* | 1.0 |
| 1435808_AT | RIKEN cDNA A230051G13 gene | | -1.1 | | 1.2* | 1.2* | 1.1 |
| 1435813_AT | RIKEN cDNA 1110056A04 gene | | 1.1 | | 1.3* | 1.1 | 1.1 |
| 1435872_AT | Gb:BE631223/DB_XREF=gi:9913911/DB_XREF=uu05g03.x | | 1.3 | | -1.4* | -1.3 | 1.2 |
| 1435929_AT | RIKEN cDNA 9630033F20 | | 1.0 | | 1.5* | 1.9* | 1.1 |
| 1436029_AT | RIKEN cDNA 4930533K18 gene | | 1.1 | | -1.3* | -1.6* | -1.2 |
| 1436431_AT | RIKEN cDNA 2900042O04 gene | | 1.1 | | 1.2* | 1.1 | -1.1 |
| 1436622_AT | IQ motif and sec 7 domain 2 | | -1.1 | | 1.4* | 1.6* | 1.0 |
| 1436678_AT | Gb:A1844814/DB_XREF=gi:5488760/DB_XREF=UI-M-AH1- | | 1.1 | | 1.3* | 1.1 | -1.1 |
| 1436795_AT | RIKEN cDNA 9630058J28 | | 1.0 | | 1.3* | 1.4* | 1.1 |
| 1436981_A_AT | Gb:BB706206/DB_XREF=gi:16055041/DB_XREF=BB706206 | | 1.2 | | -1.6* | -1.6* | -1.2 |
| 1437180_AT | RIKEN cDNA 6530403A03 | | -1.1 | | -1.2* | -1.3* | 1.0 |
| 1437449_AT | cDNA sequence BC056485 | | -1.1 | | 1.5* | 1.1 | 1.0 |
| 1437765_AT | Gb:BB249892/DB_XREF=gi:8942638/DB_XREF=BB249892 | | 1.0 | | 1.3* | 1.7* | 1.1 |
| 1437797_AT | DNA segment, Chr 5, Wayne State Univ. 150, expressed | | 1.2 | | -1.6* | -1.4 | 1.0 |
| 1437863_AT | Gb:BB667762/DB_XREF=gi:16399211/DB_XREF=BB667762 | | -1.2 | | 1.6* | 1.7* | 1.0 |
| 1437869_AT | RIKEN cDNA 3222402P14 gene | | 1.1 | | 1.2* | 1.1 | 1.0 |
| 1438006_AT | RIKEN cDNA 4933439F18 gene | | 1.0 | | 1.2* | 1.5* | 1.0 |
| 1438059_AT | Transcribed locus, weakly similar to NP_899138.1 cortexin | | 2.2* | | 2.1* | -4.0* | 1.2 |
| 1438185_AT | Gb:BM243573?DB_XREF=gi:17878843?DB_XREF=K0648G09 | | -1.2 | | 1.6* | -1.4 | 1.1 |
| 1438283_AT | RIKEN cDNA 3110057O12 gene | | -1.3 | | 1.7* | 1.6* | -1.2 |
| 1438321_X_AT | RIKEN cDNA 4930504E06 gene | | 1.2* | | -1.3* | -1.2* | 1.0 |
| 1438422_AT | Leucine rich repeat containing 20 | | 1.0 | | 1.3* | 1.6* | 1.1 |
| 1438429_AT | RIKEN cDNA 2610319H10 | | 1.1 | | -1.5* | -2.5* | 1.0 |
| 1438431_AT | Gb:BB197269?DB_XREF=gi:16271050/DB_XREF=BB197269 | | -1.3 | | 1.9* | 1.9* | -1.2 |
| 1438559_X_AT | RIKEN cDNA 1110028E10 gene | | 1.1 | | 1.2* | -1.4* | -1.1 |
| 1438680_AT | RIKEN cDNA 2700063G02 gene | | 1.0 | | -1.5* | -1.1 | -1.1 |
| 1438684_AT | RIKEN cDNA B230104P22 gene | | 1.0 | | -1.6* | 1.0 | 1.0 |
| 1438758_AT | Gb:AU048270/DB_XREF=gi:3982453/DB_XREF-AU046270 | | 1.1 | | 1.3* | 1.8* | 1.2 |
| 1438855_X_AT | Gb:BB233088/DB_XREF=gi:8912943/DB_XREF=BB233088 | | 1.5* | | -1.5* | -1.5* | 1.0 |
| 1438858_X_AT | BG:AV018723?DB_XREF=gi:4795715?DB_XREF=AV018723 | | 1.4 | | -1.6* | -3.3* | 1.2 |
| 1439087_A_AT | RIKEN cDNA 1500004A08 gene | | 1.2 | | 1.5* | 1.2 | 1.2 |
| 1439658_AT | Leiomodin 3 | | 1.0 | | 1.3* | 1.3* | -1.1 |
| 1439740_S_AT | Expressed sequence AI481316 | | 1.1 | | -1.6* | -1.8* | -1.1 |
| 1439859_AT | RIKEN cDNA 9630033F20 | | -1.1 | | 1.4* | 1.6* | 1.0 |
| 1440084_AT | Expressed sequence AI663975 | | 1.1 | | 1.9* | 1.3 | -1.1 |
| 1440461_AT | RIKEN cDNA 0910001A06 | | -1.1 | | -1.6* | -1.9* | -1.3 |
| 1440603_AT | RIKEN cDNA C030004I15 | | 1.1 | | 1.2* | 1.6* | 1.0 |
| 1440874_AT | Gb:BE686667/DB_XREF=gi:10074291/DB_XREF=uw03f01 | | 1.1 | | 2.0* | 1.3 | 1.1 |
| 1440884_S_AT | RIKEN cDNA A530047J11 | | 1.0 | | 1.4* | 1.2* | 1.0 |
| 1441338_AT | RIKEN cDNA 5930412G12 gene | | -1.4* | | 1.4* | 1.1 | -1.1 |
| 1441396_AT | Gb:AV328619/DB_XREF=gi.16395920/DB_XREF=AV328619 | | 1.2 | | 1.8* | 1.9* | 1.3 |
| 1441551_AT | RIKEN cDNA 1110056A04 gene | | 1.1 | | 1.5* | 1.1 | 1.1 |
| 1441915_S_AT | RIKEN cDNA 2310076L09 gene | | -1.2 | | -1.8* | 1.9* | -1.1 |
| 1442197_AT | RIKEN cDNA 2810450G17 gene | | 1.1 | | -1.3* | -1.2 | -1.1 |
| 1442489_AT | Gb:BG068396?DB_XREF=gi:12550965?DB_XREF=H3065A06 | | 1.3 | | -1.8* | -1.3 | -1.1 |
| 1442710_AT | Gb:AV352204?DB_XREF=gi:16397221/DB_XREF=AV352204 | | 2.1* | | -1.7* | 1.3 | 1.1 |
| 1442725_AT | Gb:AI661167/DB_XREF=gi:4764750/DB_XREF=va01h03.x | | 1.0 | | 1.6* | 1.2 | -1.1 |
| 1442977_AT | Gb:BG075843/DB_XREF=gi:12558412/DB_XREF=H3152D12 | | -1.3 | | -1.7* | -2.0* | -1.2 |
| 1443162_AT | Gb:BB043897/DB_XREF=gi:16259271/DB_XREF=BB043897 | | 1.2 | | 1.4* | 1.6* | 1.2 |
| 1443299_AT | Gb:BB480432/DB_XREF=gi:16440686/DB_XREF=BB480432 | | 1.0 | | -1.4* | -2.1* | -1.2 |
| 1443579_S_AT | DEP domain containing 6 | | 1.0 | | 1.5* | 1.1 | 1.0 |
| 1443783_X_AT | Gb:AV086906/DB_XREF=gi:5218354/DB_XREF=AV086906 | | 1.4 | | -1.6* | -3.1* | 1.1 |
| 1444341_AT | RIKEN cDNA8030451F13 | | 1.0 | | 1.3* | 1.2* | 1.0 |
| 1444504_AT | RIKEN cDNA 1110001P11 gene | | -1.2 | | 1.6* | 1.6* | -1.3 |
| 1444612_AT | RIKEN cDNA 3222402P14 | | 1.0 | | 1.4* | 1.3* | 1.0 |
| 1444643_AT | Gb:BB133024/DB_XREF=gi:16266511/DB_XREF=BB133024 | | 1.1 | | 1.2* | 1.4* | -1.1 |
| 1445449_AT | Gb:BG095184/DB_XREF=gi:12577747/DB_XREF=uu84h11 | | 1.5 | | -1.8* | -1.1 | -1.1 |
| 1446972_AT | gb:BG063165/DB_XREF=gi:12545728/DB_XREF=H3003G11 | | -1.1 | | -1.4* | -1.3* | -1.1 |
| 1447483_S_AT | Gb:AV011566/DB_XREF=gi:4788553/DB_XREF=AV011566 | | 1.0 | | -1.3* | -1.3* | 1.2 |
| 1447822_X_AT | RIKEN cDNA 2700038N03 gene | | -1.1 | | 1.4* | 1.6* | 1.1 |
| 1448383_AT | Gb:NM_008608.1/DB_XREF=gi:6678897/GEN=Mmp14/FEA | | -1.1 | | -1.4* | -2.7* | -1.2 |
| 1448396_AT | DNA seqment, Chr 1, Brigham & Women’s Genetics 0491 expressed | | -1.2 | | 1.3* | 1.2 | 1.0 |
| 1448480_AT | RIKEN cDNA 1110017C15 | | -1.1 | | -1.2* | -1.6* | 1.1 |
| 1448550_AT | Gb:NM_008489.1?DB_XREF=gi:6678669?GEN=Lbp/FEA=F | | 1.1 | | -1.4* | -1.1 | -1.1 |
| 1448584_AT | RIKEN cDNA 1200013F24 gene | | -1.1 | | 1.3* | 1.1 | 1.0 |
| 1448672_A_AT | Zinc finger protein 289 | | -1.1 | | 1.2* | 1.4* | 1.1 |
| 1449620_S_AT | Gb:AW125421/DB_XREF=gi:6100951/DB_XREF-UI-M-BH2 | | 1.1 | | 1.3* | 1.3* | 1.0 |
| 1449668_S_AT | RIKEN cDNA A730024A03 gene | | 1.1 | | 1.5* | 1.2 | 1.1 |
| 1450017_AT | Gb:BG065754/DB_XREF=gi:12548317/DB_XREF=H3034H06 | | 1.0 | | 1.2* | 1.6* | 1.0 |
| 1450738_AT | Gb:NM_016705.1/DB_XREF=gi:7710045?GEN=Kif21a/FE | | 1.1 | | 1.2* | 1.6* | 1.1 |
| 1450958_AT | Transmembrane 4 superfamily member 1 | | -1.1 | | -1.3* | 1.3* | 1.1 |
| 1451313_A_AT | RIKEN cDNA 1110067D22 gene | | -1.2 | | 1.3* | 2.1* | -1.1 |
| 1451335_AT | Placenta-specific 8 | | 1.3 | | -2.0* | -5.1* | -1.1 |
| 1451348_AT | DEP domain containing 6 | | 1.0 | | 1.5* | 1.2 | 1.0 |
| 1451486_AT | RIKEN cDNA 1200006F02 gene | | 1.0 | | 1.4* | 1.1 | -1.1 |
| 1451508_AT | RIKEN cDNA 1700108L22 gene | | 1.0 | | 1.2* | 1.0 | -1.1 |
| 1451622_AT | RIKEN cDNA 0910001K20 | | 1.0 | | 1.2* | 1.2* | 1.1 |
| 1451678_AT | RIKEN cDNA 4430402O11 gene | | 1.0 | | 1.2* | 1.1 | 1.0 |
| 1451932_A_AT | Thrombospondin repeat containing 1 | | 1.1 | | 1.5* | 1.3* | 1.1 |
| 1452151_AT | cDNA sequence BC021523 | | 1.3 | | -1.5* | 1.0 | -1.1 |
| 1452175_AT | RIKEN cDNA 1810026J23 gene | | -1.1 | | 1.3* | 1.2 | 1.1 |
| 1452214_AT | RIKEN cDNA 9130011J04 | | 1.0 | | -1.4* | -1.3* | -1.1 |
| 1452362_AT | Tripartite motif protein 16 | | 1.0 | | 1.3* | 1.3* | -1.1 |
| 1452382_AT | Neoplastic progression 1 | | 1.1 | | -1.8* | -4.8* | -1.1 |
| 1452679_AT | RIKEN cDNA 2410129E14 | | -1.2 | | -1.7* | -7.7* | -1.3 |
| 1452876_X_AT | RIKEN cDNA 2610044O15 gene | | -1.2 | | 1.5* | 1.6* | 1.1 |
| 1452895_AT | RIKEN cDNA 2610017J04 gene | | -1.1 | | 1.3* | 1.4* | 1.0 |
| 1453035_AT | Limb and neural patterns | | 1.1 | | 1.7* | 1.2 | 1.2 |
| 1453059_AT | RIKEN cDNA 2810047F03 gene | | 1.1 | | 1.6* | 1.0 | -1.1 |
| 1453119_AT | RIKEN cDNA 4933428L19 gene | | -1.2 | | -1.8* | -1.1 | 1.0 |
| 1453141_AT | RIKEN cDNA 0610009L18 | | 1.0 | | 1.3* | 1.3* | -1.1 |
| 1453304_S_AT | Lymphocyte antigen 6 complex, locus E | | 1.0 | | -1.4* | -1.2 | -1.1 |
| 1453355_AT | RIKEN cDNA 1810073P09 | | -1.1 | | 1.5* | 2.1* | 1.1 |
| 1453377_AT | SH2 domain containing 4A | | 1.3 | | -1.9* | 1.2 | 1.0 |
| 1453592_AT | RIKEN cDNA 9430028I06 gene | | -1.1 | | 1.3* | 1.7* | 1.1 |
| 1453593_AT | RIKEN cDNA 1700110N18 | | -1.1 | | -1.5* | -1.4* | -1.2 |
| 1453732_AT | RIKEN cDNA 6230416A05 gene | | -1.2 | | 1.3* | 1.5* | -1.1 |
| 1453744_A_AT | RIKEN cDNA 5530600A18 gene | | -1.3* | | 1.3* | 1.6* | -1.1 |
| 1453855_AT | RIKEN cDNA 1810057P16 | | -1.1 | | -1.5* | -1.7* | 1.0 |
| 1454197_A_AT | DNA segment, Chr 19, ERATO Doi 678 | | -1.2 | | -1.3* | -1.6* | 1.0 |
| 1454613_AT | Gb:AV162270/DB_XREF=gi:16383390/DB_XREF=AV162270 | | -1.1 | | -1.4* | -2.1* | -1.3 |
| 1454709_AT | RIKEN cDNA 9630015D15 gene | | 1.0 | | 1.3* | 1.1 | 1.0 |
| 1454728_S_AT | Gb:AW610650/DB_XREF=gi:7315466/DB_XREF=up35d10.x | | 1.4* | | -1.4* | -1.5* | 1.2 |
| 1454808_AT | EF hand domain family A1 | | 1.1 | | 1.3* | -1.2 | 1.1 |
| 1454877_AT | SERTA domain containing 4 | | -1.2 | | -1.3* | -2.0* | -1.1 |
| 1454984_AT | Gb:AV246615/DB_XREF=gi:16387119/DB_XREF=AV246615 | | 1.1 | | 1.4* | 1.5* | -1.1 |
| 1455091_AT | Gb:AI642021/DB_XREF=gi:4720496/DB_XREF=ub74g08.x | | 1.1 | | 1.2* | 1.1 | 1.0 |
| 1455271_AT | RIKEN cDNA 1810020C19 | | 1.2 | | -3.2* | -3.3* | 1.0 |
| 1455320_AT | Gb:BQ176847/DB_XREF=gi:20352339/DB_XREF=UI-M-DJ2 | | 1.3 | | 1.4* | 1.7* | 1.2 |
| 1455372_AT | Gb:BB770826/DB_XREF=gi:16197205/DB_XREF=BB770826 | | 1.0 | | 1.3* | 1.8* | 1.1 |
| 1455450_AT | Gb:AV231357/DB_XREF=gi:16385846/DB_XREF=AV231357 | | 1.1 | | 1.6* | 2.1* | 1.2 |
| 1455475_AT | RIKEN cDNA 3110057O12 gene | | -1.1 | | 1.4* | 1.6* | -1.3 |
| 1455493_AT | 0 day neonate cerebellum cDNA, RIKEN full-length enriched library, clone:C230022A12 product:unknown EST, full insert sequence | | 1.1 | | -1.4* | 1.1 | 1.2 |
| 1455551_AT | RIKEN cDNA 8430408E05 gene | | -1.3 | | 1.5* | 1.3 | 1.0 |
| 1455665_AT | Gb:BB705689/DB_XREF=gi:16054524/DB_XREF=BB705689 | | 1.0 | | 1.8* | 1.4 | 1.2 |
| 1455732_AT | RIKEN cDNA 1700025G04 gene | | 1.0 | | 1.2* | -1.2* | -1.1 |
| 1456048_AT | Gb:BB281000/DB_XREF=gi:15411106/DB_XREF=BB281000 | | 1.1 | | 1.3* | 1.6* | 1.1 |
| 1456377_X_AT | RIKEN cDNA 0610025L06 | | -1.2 | | -1.3* | -2.8* | -1.1 |
| 1456480_AT | RIKEN cDNA 9330186A19 | | 1.0 | | 1.2* | 1.5* | 1.0 |
| 1456859_AT | Gb:Bl133590/DB_XREF=gi:14583838/DB_XREF=UI-M-BH3 | | -1.1 | | 2.2* | 1.7* | 1.2 |
| 1457139_AT | RIKEN cDNA 2700063G02 gene | | 1.2 | | -1.7* | -1.3 | -1.1 |
| 1458296_AT | Gb:BI465650/DB_XREF=gi:15278528/DB_XREF=ie17e03 | | -1.2 | | -1.3* | -1.6* | -1.2 |
| 1458308_AT | cDNA sequence BC019206 | | 1.1 | | -1.7* | -1.7* | 1.0 |
| 1460621_X_AT | Gb:AV124281/DB_XREF=gi:5310515/DB_XREF=AV124281 | | 1.1 | | -1.2* | -1.3* | -1.1 |
| 1460629_AT | Gb:BB033733/DB_XREF=gi:15403628/DB_XREF=BB033733 | | -1.1 | | 1.3* | 1.3* | 1.0 |
| 1460695_A_AT | RIKEN cDNA 2300006M17 gene | | 1.1 | | -1.2* | -1.2* | 1.1 |
|  |  | |  | |  |  |  |
