# Supplementary material for: Corticortophin releasing factor 2 receptor agonist treatment significantly slows disease progression in mdx mice
Source: BMC Med. 2007 Jul 12;5:18. doi: 10.1186/1741-7015-5-18 (PMC1936998; doi:10.1186/1741-7015-5-18)
Supplement: Additional file 2 — Tissue-specific subgroup analysis of differential gene expression changes in mdx mice treated for 3 months with either vehicle or PG873637. All differential genes showed statistically significant differences in expression (NLogP = 4.0). [file 1741-7015-5-18-S2.doc]

# Additional files

### Additional File 2 – Subgroup analysis of differential gene expression changes in mdx mice treated for 3 months with either vehicle or PG873637.

All differential genes demonstrated statistically significant differences in expression (NLogP=4.0).

| **Affy ID** | | **Gene** | **mdx vehicle** versus **mdx time 0** | | **mdx PG873637** versus  **mdx vehicle** | | **C57BL10 vehicle** versus  **mdx vehicle** | **C57BL10 vehicle** versus **C57BL10 time 0** |
| --- | --- | --- | --- | --- | --- | --- | --- | --- |
| **Dystrophin Complex Genes** | | | | | | | | |
| 1417307_AT | Dystrophin | | | -1.1 | | 1.1 | 3.3* | 1.0 |
| 1422654_AT | Sarcoglycan, alpha | | | 1.1 | | 1.5* | 1.3* | 1.0 |
| 1419667_AT | Sarcoglycan, beta | | | -1.2 | | 1.3* | 1.1 | 1.0 |
| 1426066_A_AT | Dystrobrevin alpha | | | 1.1 | | 1.4* | -1.2 | 1.0 |
| 1422949_AT | Nitric oxide synthase 1 | | | -1.2 | | 1.1 | 2.9* | 1.2 |
| 1426778_AT | Dystroglycan 1 | | | -1.1 | | 1.1 | -1.3* | -1.2 |
| 1440452_AT | Dystrophin related proteins 2 | | | 1.0 | | -1.1 | -1.4* | 1.1 |
|  | | | | | | | | |
| **Immune Cell Expressed Genes** | | | | | | | | |
| 1449289_A_AT | Beta 2 microglobulin | | | 1.2 | | -1.4* | -1.3* | 1.1 |
| 1451784_X_AT | Histocompatibility 2, D region | | | 1.3 | | -1.4* | -1.3 | 1.2 |
| 1449556_AT | Histocompatibility 2, T region locus 23 | | | 1.2 | | -1.4* | -1.5* | 1.1 |
| 1452431_S_AT | Histocompatibility 2, class II antigen A, alpha | | | 1.3 | | -1.6* | -3.0* | 1.3 |
| 1435290_X_AT | Histocompatibility 2, class II antigen A, alpha | | | 1.3 | | -1.4* | -2.5* | 1.2 |
| 1451721_A_AT | Histocompatibility 2, class II antigen A, beta 1 | | | 1.4 | | -1.7* | -3.1* | 1.1 |
| 1449195_S_AT | Chemokine (C-X-C motif) ligand 16 | | | 1.3 | | -1.8* | -3.9* | -1.1 |
| 1448316_AT | Chemokine like factor super family 3 | | | -1.1 | | -1.2* | -1.6* | 1.0 |
| 1450020_AT | Chemokine (C-X3-C) receptor 1 | | | -1.4 | | -1.9* | -3.4* | 1.0 |
| 1453304_S_AT | Lymphocyte antigen 6 complex, locus E | | | 1.0 | | -1.4* | -1.2 | -1.1 |
| 1426906_AT | Interferon activated gene 203 | | | 1.1 | | -1.4* | -1.6* | 1.0 |
| 1423754_AT | Interferon induced transmembrane protein 3 | | | 1.0 | | -1.4* | -1.4* | -1.1 |
| 1417244_A_AT | Interferon regulatory factor 7 | | | -1.1 | | -1.6* | -1.9* | -1.1 |
| 1419272_AT | Myeloid differentiation primary response gene 88 | | | 1.0 | | -1.4 | -1.8 | -1.1 |
| 1433575_AT | SRY-box containing gene 4 | | | 1.0 | | -1.6* | -1.9* | -1.2 |
| 1435697_A_AT | Pleckstrin homology, sec& and coiled-coil domain, binding protein | | | 1.7* | | -1.6* | -2.5* | 1.0 |
| 1436905_X_AT | Lysosomal-associated protein transmembrane 5 | | | 1.0 | | -1.4* | -2.3* | -1.1 |
| 1437503_A_AT | Scotin gene | | | 1.0 | | -1.3* | -1.6* | -1.1 |
| 1448380_AT | Lectin, galactoside-binding, soluble 3, binding protein | | | -1.1 | | -1.4* | -1.4* | -1.1 |
| 1448891_AT | Macrophage scavenger receptor 2 | | | -1.1 | | -1.5* | -1.7* | 1.1 |
| 1450792_AT | TYRO protein tyrosine kinase binding protein | | | 1.1 | | -1.5* | -6.1* | -1.1 |
| 1450997_AT | Serine/threonine kinase 17b (apoptosis-inducing) | | | 1.2 | | -1.5* | -2.4* | 1.1 |
| 1451335_AT | Placenta-specific 8 | | | 1.3 | | -2.0* | -5.1* | -1.1 |
| 1451860_A_AT | Tripartite motif protein 30 | | | 1.0 | | -1.3* | -1.5* | -1.1 |
| 1453304_S_AT | Lymphocyte antigen 6 complex, locus E | | | 1.0 | | -1.4* | -1.2 | -1.1 |
| 1454783_AT | Interleukin 13 receptor, alpha 1 | | | 1.1 | | -1.5* | -2.3* | -1.1 |
| 1455899_X_AT | Suppressor of cytokine signaling 3 | | | 1.5 | | -2.1* | -3.4* | -1.4 |
| 1436905_X_AT | Lysosomal associated protein transmembrane 5 | | | 1.0 | | -1.4* | -4.3* | -1.1 |
| 1416303_AT | LPS-induced TN factor | | | 1.0 | | -1.3* | -1.5* | 1.0 |
| 1417381_AT | Complement component 1, q subcomponent, alpha polypeptide | | | 1.3 | | -1.5* | -3.7* | -1.1 |
| 1434366_X_AT | Complement component 1, q subcomponent, beta polypeptide | | | 1.2 | | -1.3* | -2.2* | -1.1 |
| 1417483_AT | Nuclear factor of kappa light polypeptide gene enhancer in B-cells, zeta | | | 1.3 | | -1.4* | -1.2 | 1.0 |
| 1417185_AT | Lymphocyte antigen 6 complex, locus A | | | 1.3* | | -1.3* | -1.1 | -1.1 |
| 1417378_AT | Immunoglobulin superfamily, member 4A | | | 1.4* | | -1.3* | -1.5* | -1.1 |
| 1424041_S_AT | Complement component 1, s subcomponent | | | 1.3* | | -1.4* | -1.3* | -1.1 |
| 1426587_A_AT | Signal transducer and activator of transcription 3 (STAT3) | | | 1.1 | | -1.3* | -1.3* | -1.1 |
| 1437279_X_AT | Syndecan 1 | | | 1.3 | | -1.7* | -2.2* | -1.1 |
| 1417789_AT | Small chemokine (C-C motif) ligand 11 | | | 1.1 | | 2.0* | 3.0* | 1.4 |
| 1418219_AT | Interleukin 15 (demonstrated beneficial effects on mdx diaphragm) | | | 1.2 | | 2.1* | 3.0* | 1.5 |
| 1425990_A_AT | Nuclear factor of activated T-cells, cytoplasmic, calcineurin dependent 2 (induces IL-4 expression in myocytes for differentiation) | | | 1.0 | | 1.7* | -1.3 | 1.0 |
|  | | | | | | | | |
| **Neuronal Cell Expressed Genes** | | | | | | | | |
| 1415877_AT | Dihydropyrimidinase-like 3 | | | -1.1 | | -1.5* | -2.0* | -1.2 |
| 1418289_AT | Nestin | | | 1.1 | | -1.5* | -1.7* | 1.0 |
| 1437457_A_AT | Myotrophin | | | -1.1 | | -1.3* | -1.5* | -1.1 |
| 1425784_A_AT | Olfactomedin 1 | | | -1.4 | | -1.8* | -1.8* | -1.3 |
| 1427064_A_AT | Scribbled homolog | | | -1.1 | | -1.4* | -2.0* | 1.0 |
| 1450295_S_AT | Poliovirus receptor | | | -1.1 | | -1.5* | -2.1* | -1.1 |
| 1448370_AT | Unc-51 like kinase 1 | | | 1.1 | | -1.2* | 1.2* | 1.0 |
| 1449315_AT | Odd Oz/tenm homolog 3 | | | 1.0 | | -1.4* | -1.2* | 1.0 |
| 1416610_A_AT | Chloride channel 3 | | | 1.1 | | 1.5* | 1.8* | 1.1 |
| 1422635_AT | Acetylcholinesterase | | | 1.0 | | 1.6* | 1.4* | 1.1 |
| 1423454_A_AT | Sema domain (semaphorin) 6C | | | 1.0 | | 1.4* | 1.1 | -1.1 |
| 1426462_AT | Gephyrin | | | 1.1 | | 1.2* | 1.3* | 1.0 |
| 1440435_AT | Kyphoscoliosis | | | 1.2 | | 2.7* | 1.2 | 1.0 |
| 1426951_AT | Cysteine-rich motor neuron 1 | | | 1.1 | | 1.5* | 2.1* | 1.2 |
| 1416178_A_AT | Pleckstrin homology domain containing, family B member 1 | | | 1.0 | | 1.8* | 2.1* | 1.2 |
| 1425533_A_AT | Staufen homolog 2 | | | 1.0 | | 1.4* | 1.4* | 1.1 |
| 1432385_A_AT | ATP/GTP binding protein | | | -1.3 | | 2.4* | 2.7* | 1.2 |
| 1434735_AT | Hepatic leukemia factor | | | 1.1 | | 1.7* | 1.5* | 1.0 |
| 1435292_AT | TBC1 domain family, member 4 | | | 1.1 | | 1.2* | 1.6* | 1.1 |
| 1438294_AT | Spinocerebellar ataxia 1 homolog | | | 1.0 | | 1.4* | 1.3* | -1.1 |
| 1450899_AT | Neural precursor cell expressed developmentally down-regulated gene 1 | | | -1.1 | | 1.5* | 1.5* | 1.0 |
|  | | | | | | | | |
| **Myocyte Expressed Genes** | | | | | | | | |
| 1416067_AT | Interferon-related developmental regulator 1 | | | 1.0 | | -1.5* | 1.1 | 1.1 |
| 1418420_AT | Myogenic differentiation 1 | | | -1.2 | | -1.6* | -2.9* | -1.1 |
| 1419391_AT | Myogenin | | | 1.0 | | -1.6* | -12.6* | -1.4 |
| 1424770_AT | Caldesmon 1 | | | -1.1 | | -1.3* | -1.1 | -1.1 |
| 1428266_AT | Myosin, light polypeptide 3 | | | 1.0 | | -1.4* | 1.8* | 1.1 |
| 1439455_X_AT | Capping protein muscle Z-line, alpha 1 | | | -1.1 | | -1.3* | -1.8* | -1.2 |
| 1419667_AT | Sarcoglycan, beta | | | -1.2 | | 1.3* | 1.1 | 1.0 |
| 1421028_A_AT | Myocyte enhancer factor 2C | | | 1.2 | | 1.4* | -1.2 | 1.1 |
| 1422744_AT | Phosphorylase kinase alpha 1 | | | 1.0 | | 1.6* | 1.6* | -1.1 |
| 1424852_AT | Myocyte enhancer factor 2C | | | 1.2 | | 1.3* | -1.1 | 1.1 |
| 1425990_A_AT | Nuclear factor of activated T-cells, cytoplasmic, calcineurin dependent 2 (induces IL-4 expression in myocytes for differentiation) | | | 1.0 | | 1.7* | -1.3 | 1.0 |
| 1440435_AT | Kyphoscoliosis | | | 1.3 | | 2.7* | 1.2 | 1.0 |
| 1450196_S_AT | Glycogen synthase 1, muscle | | | 1.0 | | 1.4* | 1.4* | 1.0 |
| 1454808_AT | EF hand domain family 1 | | | 1.1 | | 1.3* | -1.2 | 1.1 |
| 1450952_AT | Phospholamban | | | 1.3 | | 1.8* | 1.6 | 1.5 |
| 1451503_AT | Nucleolar protein 3 | | | 1.1 | | 1.2* | 1.0 | 1.0 |
| 1457435_X_AT | Myomesin 2 | | | 1.1 | | 1.3* | -1.5* | -1.2 |
|  | | | | | | | | |
| **Circadian Rhythm Related Genes** | | | | | | | | |
| 1417602_AT | Period homolog 2 | | | 1.2 | | 5.1* | 1.8 | 1.9 |
| 1421087_AT | Period homolog 3 | | | 1.4 | | 3.9* | 1.5 | 1.0 |
| 1418174_AT | D site albumin promoter binding protein | | | 1.4 | | 9.2* | 2.1 | 1.3 |
| 1426383_AT | Cryptochrome 2 | | | 1.1 | | 1.4* | 1.1 | 1.1 |
| 1450184_S_AT | Thyrotroph embryonic factor | | | 1.0 | | 2.2* | 1.5 | 1.3 |
| 1416958_AT | Nuclear receptor subfamily 1, group D, member 2 | | | 1.2 | | 2.3* | 1.6 | 1.1 |
| 1458129_AT | RAR-related orphan receptor alpha | | | 1.1 | | 1.2* | 1.0 | -1.1 |
| 1418660_AT | Circadian locomoter output cycles kaput | | | 1.0 | | -1.7* | -1.2 | -1.1 |
| 1425099_A_AT | Aryl hydrocarbon receptor nuclear translocator-like | | | -1.1 | | -3.4* | -1.1 | 1.0 |
