# Supplementary material for: Corticortophin releasing factor 2 receptor agonist treatment significantly slows disease progression in mdx mice
Source: BMC Med. 2007 Jul 12;5:18. doi: 10.1186/1741-7015-5-18 (PMC1936998; doi:10.1186/1741-7015-5-18)
Supplement: Additional file 3 — Differential gene expression profile of mdx vehicle versus mdx time 0. All differential genes showed statistically significant differences in expression (NLogP = 4.0). [file 1741-7015-5-18-S3.doc]

…

# Additional files

### Additional File 3 – Differential gene expression profile of mdx vehicle verus mdx time 0.

All differential genes demonstrated statistically significant differences in expression (NLogP=4.0).

| **AffyID** | **NetAffx Title** | **Acronym** | **Molecular Function** | **OMIM** | **MDX vehicle versus MDX Time 0 Fold Change** |
| --- | --- | --- | --- | --- | --- |
| 1415712_AT | zinc finger, RAN-binding domain containing 1 | [ZRANB1](http://www.gene.ucl.ac.uk/cgi-bin/nomenclature/searchgenes.pl?field=symbol&anchor=equals&symbol_search=Search&number=100&format=html&sortby=symbol&match=ZRANB1) | actin binding |  | 1.23 |
| 1415920_AT | cleavage stimulation factor, 3' pre-RNA subunit 2, tau | [CSTF2T](http://www.gene.ucl.ac.uk/cgi-bin/nomenclature/searchgenes.pl?field=symbol&anchor=equals&symbol_search=Search&number=100&format=html&sortby=symbol&match=CSTF2T) | aminoacylase activity;hydrolase activity, acting on ester bonds;aspartoacylase activity;hydrolase activity |  | -1.2 |
| 1415935_AT | SPARC related modular calcium binding 2 | [SMOC2](http://www.gene.ucl.ac.uk/cgi-bin/nomenclature/searchgenes.pl?field=symbol&anchor=equals&symbol_search=Search&number=100&format=html&sortby=symbol&match=SMOC2) | bile acid:sodium symporter activity | [607223](http://www.ncbi.nlm.nih.gov/entrez/dispomim.cgi?id=607223) | 1.23 |
| 1415939_AT | fibromodulin | [FMOD](http://www.gene.ucl.ac.uk/cgi-bin/nomenclature/searchgenes.pl?field=symbol&anchor=equals&symbol_search=Search&number=100&format=html&sortby=symbol&match=FMOD) | bisphosphoglycerate mutase activity;isomerase activity;intramolecular transferase activity, phosphotransferases;bisphosphoglycerate phosphatase activity;catalytic activity;hydrolase activity;phosphoglycerate mutase activity | [600245](http://www.ncbi.nlm.nih.gov/entrez/dispomim.cgi?id=600245) | -1.82 |
| 1415949_AT | carboxypeptidase E | [CPE](http://www.gene.ucl.ac.uk/cgi-bin/nomenclature/searchgenes.pl?field=symbol&anchor=equals&symbol_search=Search&number=100&format=html&sortby=symbol&match=CPE) | calcium ion binding | [114855](http://www.ncbi.nlm.nih.gov/entrez/dispomim.cgi?id=114855) | 1.21 |
| 1415996_AT | thioredoxin interacting protein | [TXNIP](http://www.gene.ucl.ac.uk/cgi-bin/nomenclature/searchgenes.pl?field=symbol&anchor=equals&symbol_search=Search&number=100&format=html&sortby=symbol&match=TXNIP) | calcium ion binding | [606599](http://www.ncbi.nlm.nih.gov/entrez/dispomim.cgi?id=606599) | 2.29 |
| 1415997_AT | thioredoxin interacting protein | [TXNIP](http://www.gene.ucl.ac.uk/cgi-bin/nomenclature/searchgenes.pl?field=symbol&anchor=equals&symbol_search=Search&number=100&format=html&sortby=symbol&match=TXNIP) | calcium ion binding | [606599](http://www.ncbi.nlm.nih.gov/entrez/dispomim.cgi?id=606599) | 2.2 |
| 1416039_X_AT | cysteine rich protein 61 | [CYR61](http://www.gene.ucl.ac.uk/cgi-bin/nomenclature/searchgenes.pl?field=symbol&anchor=equals&symbol_search=Search&number=100&format=html&sortby=symbol&match=CYR61) | calcium ion binding | [602369](http://www.ncbi.nlm.nih.gov/entrez/dispomim.cgi?id=602369) | 2.55 |
| 1416041_AT | serum/glucocorticoid regulated kinase | [SGK](http://www.gene.ucl.ac.uk/cgi-bin/nomenclature/searchgenes.pl?field=symbol&anchor=equals&symbol_search=Search&number=100&format=html&sortby=symbol&match=SGK) | calcium ion binding | [602958](http://www.ncbi.nlm.nih.gov/entrez/dispomim.cgi?id=602958) | 1.7 |
| 1416101_A_AT | histone 1, H1c | [HIST1H1C](http://www.gene.ucl.ac.uk/cgi-bin/nomenclature/searchgenes.pl?field=symbol&anchor=equals&symbol_search=Search&number=100&format=html&sortby=symbol&match=HIST1H1C) | calcium ion binding | [142710](http://www.ncbi.nlm.nih.gov/entrez/dispomim.cgi?id=142710) | 1.37 |
| 1416110_AT | solute carrier family 35, member A4 | [SLC35A4](http://www.gene.ucl.ac.uk/cgi-bin/nomenclature/searchgenes.pl?field=symbol&anchor=equals&symbol_search=Search&number=100&format=html&sortby=symbol&match=SLC35A4) | calcium ion binding |  | -1.24 |
| 1416129_AT | RIKEN cDNA 1300002F13 gene | [1300002F13RIK](http://www.gene.ucl.ac.uk/cgi-bin/nomenclature/searchgenes.pl?field=symbol&anchor=equals&symbol_search=Search&number=100&format=html&sortby=symbol&match=1300002F13RIK) | calcium ion binding |  | 3.58 |
| 1416138_AT | annexin A7 | [ANXA7](http://www.gene.ucl.ac.uk/cgi-bin/nomenclature/searchgenes.pl?field=symbol&anchor=equals&symbol_search=Search&number=100&format=html&sortby=symbol&match=ANXA7) | calcium ion binding | [186360](http://www.ncbi.nlm.nih.gov/entrez/dispomim.cgi?id=186360) | 1.23 |
| 1416152_A_AT | splicing factor, arginine/serine-rich 3 (SRp20) | [SFRS3](http://www.gene.ucl.ac.uk/cgi-bin/nomenclature/searchgenes.pl?field=symbol&anchor=equals&symbol_search=Search&number=100&format=html&sortby=symbol&match=SFRS3) | calcium ion binding | [603364](http://www.ncbi.nlm.nih.gov/entrez/dispomim.cgi?id=603364) | -1.36 |
| 1416191_AT | Sec61 alpha 1 subunit (S. cerevisiae) | [SEC61A1](http://www.gene.ucl.ac.uk/cgi-bin/nomenclature/searchgenes.pl?field=symbol&anchor=equals&symbol_search=Search&number=100&format=html&sortby=symbol&match=SEC61A1) | calcium ion binding;electron transporter activity;nitric-oxide synthase activity;oxidoreductase activity;calmodulin binding;FMN binding |  | -1.34 |
| 1416250_AT | B-cell translocation gene 2, anti-proliferative | [BTG2](http://www.gene.ucl.ac.uk/cgi-bin/nomenclature/searchgenes.pl?field=symbol&anchor=equals&symbol_search=Search&number=100&format=html&sortby=symbol&match=BTG2) | calcium ion binding;G-protein coupled receptor activity | [601597](http://www.ncbi.nlm.nih.gov/entrez/dispomim.cgi?id=601597) | 2.24 |
| 1416256_A_AT | tubulin, beta 5 | [TUBB5](http://www.gene.ucl.ac.uk/cgi-bin/nomenclature/searchgenes.pl?field=symbol&anchor=equals&symbol_search=Search&number=100&format=html&sortby=symbol&match=TUBB5) | calcium ion binding;heparin binding | [602662](http://www.ncbi.nlm.nih.gov/entrez/dispomim.cgi?id=602662) | -1.21 |
| 1416286_AT | regulator of G-protein signaling 4 | [RGS4](http://www.gene.ucl.ac.uk/cgi-bin/nomenclature/searchgenes.pl?field=symbol&anchor=equals&symbol_search=Search&number=100&format=html&sortby=symbol&match=RGS4) | calcium ion binding;mannosyl-oligosaccharide 1,2-alpha-mannosidase activity;hydrolase activity, acting on glycosyl bonds;hydrolase activity |  | -1.7 |
| 1416331_A_AT | nuclear factor, erythroid derived 2,-like 1 | [NFE2L1](http://www.gene.ucl.ac.uk/cgi-bin/nomenclature/searchgenes.pl?field=symbol&anchor=equals&symbol_search=Search&number=100&format=html&sortby=symbol&match=NFE2L1) | calcium ion binding;structural constituent of muscle | [163260](http://www.ncbi.nlm.nih.gov/entrez/dispomim.cgi?id=163260) | 1.2 |
| 1416332_AT | cold inducible RNA binding protein | [CIRBP](http://www.gene.ucl.ac.uk/cgi-bin/nomenclature/searchgenes.pl?field=symbol&anchor=equals&symbol_search=Search&number=100&format=html&sortby=symbol&match=CIRBP) | calcium:sodium antiporter activity;calmodulin binding;carrier activity | [602649](http://www.ncbi.nlm.nih.gov/entrez/dispomim.cgi?id=602649) | 1.63 |
| 1416407_AT | phosphoprotein enriched in astrocytes 15 | [PEA15](http://www.gene.ucl.ac.uk/cgi-bin/nomenclature/searchgenes.pl?field=symbol&anchor=equals&symbol_search=Search&number=100&format=html&sortby=symbol&match=PEA15) | calcium:sodium antiporter activity;carrier activity;calmodulin binding | [603434](http://www.ncbi.nlm.nih.gov/entrez/dispomim.cgi?id=603434) | -1.26 |
| 1416505_AT | nuclear receptor subfamily 4, group A, member 1 | [NR4A1](http://www.gene.ucl.ac.uk/cgi-bin/nomenclature/searchgenes.pl?field=symbol&anchor=equals&symbol_search=Search&number=100&format=html&sortby=symbol&match=NR4A1) | calcium-dependent phospholipid binding;phospholipase A2 inhibitor activity;phospholipase inhibitor activity;calcium ion binding | [139139](http://www.ncbi.nlm.nih.gov/entrez/dispomim.cgi?id=139139) | 2.71 |
| 1416564_AT | SRY-box containing gene 7 | [SOX7](http://www.gene.ucl.ac.uk/cgi-bin/nomenclature/searchgenes.pl?field=symbol&anchor=equals&symbol_search=Search&number=100&format=html&sortby=symbol&match=SOX7) | calcium-dependent phospholipid binding;protein binding;calcium ion binding |  | -1.72 |
| 1416573_AT | protein O-fucosyltransferase 2 | [POFUT2](http://www.gene.ucl.ac.uk/cgi-bin/nomenclature/searchgenes.pl?field=symbol&anchor=equals&symbol_search=Search&number=100&format=html&sortby=symbol&match=POFUT2) | cAMP-specific phosphodiesterase activity;3',5'-cyclic-nucleotide phosphodiesterase activity;catalytic activity;hydrolase activity |  | -1.47 |
| 1416625_AT | serine (or cysteine) proteinase inhibitor, clade G, member 1 | [SERPING1](http://www.gene.ucl.ac.uk/cgi-bin/nomenclature/searchgenes.pl?field=symbol&anchor=equals&symbol_search=Search&number=100&format=html&sortby=symbol&match=SERPING1) | carboxy-lyase activity;phosphatidylserine decarboxylase activity;lyase activity | [606860](http://www.ncbi.nlm.nih.gov/entrez/dispomim.cgi?id=606860) | 1.25 |
| 1416658_AT | frizzled-related protein | [FRZB](http://www.gene.ucl.ac.uk/cgi-bin/nomenclature/searchgenes.pl?field=symbol&anchor=equals&symbol_search=Search&number=100&format=html&sortby=symbol&match=FRZB) | cation channel activity;potassium channel activity;ion channel activity;voltage-gated ion channel activity;voltage-gated potassium channel activity | [605083](http://www.ncbi.nlm.nih.gov/entrez/dispomim.cgi?id=605083) | -1.51 |
| 1416926_AT | transformation related protein 53 inducible nuclear protein 1 | [TRP53INP1](http://www.gene.ucl.ac.uk/cgi-bin/nomenclature/searchgenes.pl?field=symbol&anchor=equals&symbol_search=Search&number=100&format=html&sortby=symbol&match=TRP53INP1) | cation channel activity;potassium channel activity;voltage-gated ion channel activity;ion channel activity;voltage-gated potassium channel activity |  | 1.46 |
| 1416940_AT | peptidylprolyl isomerase F (cyclophilin F) | [PPIF](http://www.gene.ucl.ac.uk/cgi-bin/nomenclature/searchgenes.pl?field=symbol&anchor=equals&symbol_search=Search&number=100&format=html&sortby=symbol&match=PPIF) | chaperone activity;isomerase activity;peptidyl-prolyl cis-trans isomerase activity | [604486](http://www.ncbi.nlm.nih.gov/entrez/dispomim.cgi?id=604486) | -1.29 |
| 1416953_AT | connective tissue growth factor | [CTGF](http://www.gene.ucl.ac.uk/cgi-bin/nomenclature/searchgenes.pl?field=symbol&anchor=equals&symbol_search=Search&number=100&format=html&sortby=symbol&match=CTGF) | chemokine activity;cytokine activity | [121009](http://www.ncbi.nlm.nih.gov/entrez/dispomim.cgi?id=121009) | 2.73 |
| 1417065_AT | early growth response 1 | [EGR1](http://www.gene.ucl.ac.uk/cgi-bin/nomenclature/searchgenes.pl?field=symbol&anchor=equals&symbol_search=Search&number=100&format=html&sortby=symbol&match=EGR1) | chemokine activity;cytokine activity | [128990](http://www.ncbi.nlm.nih.gov/entrez/dispomim.cgi?id=128990) | 2.79 |
| 1417110_AT | mannosidase 1, alpha | [MAN1A](http://www.gene.ucl.ac.uk/cgi-bin/nomenclature/searchgenes.pl?field=symbol&anchor=equals&symbol_search=Search&number=100&format=html&sortby=symbol&match=MAN1A) | chemokine activity;cytokine activity |  | 1.27 |
| 1417185_AT | lymphocyte antigen 6 complex, locus A | [LY6A](http://www.gene.ucl.ac.uk/cgi-bin/nomenclature/searchgenes.pl?field=symbol&anchor=equals&symbol_search=Search&number=100&format=html&sortby=symbol&match=LY6A) | chemokine activity;cytokine activity;heparin binding |  | 1.26 |
| 1417204_AT | KDEL (Lys-Asp-Glu-Leu) endoplasmic reticulum protein retention receptor 2 | [KDELR2](http://www.gene.ucl.ac.uk/cgi-bin/nomenclature/searchgenes.pl?field=symbol&anchor=equals&symbol_search=Search&number=100&format=html&sortby=symbol&match=KDELR2) | DNA binding |  | -1.22 |
| 1417265_S_AT | DNA segment, Chr 5, ERATO Doi 33, expressed | [D5ERTD33E](http://www.gene.ucl.ac.uk/cgi-bin/nomenclature/searchgenes.pl?field=symbol&anchor=equals&symbol_search=Search&number=100&format=html&sortby=symbol&match=D5ERTD33E) | DNA binding |  | -1.26 |
| 1417271_A_AT | endoglin | [ENG](http://www.gene.ucl.ac.uk/cgi-bin/nomenclature/searchgenes.pl?field=symbol&anchor=equals&symbol_search=Search&number=100&format=html&sortby=symbol&match=ENG) | DNA binding | [131195](http://www.ncbi.nlm.nih.gov/entrez/dispomim.cgi?id=131195) | -1.53 |
| 1417311_AT | cysteine rich protein 2 | [CRIP2](http://www.gene.ucl.ac.uk/cgi-bin/nomenclature/searchgenes.pl?field=symbol&anchor=equals&symbol_search=Search&number=100&format=html&sortby=symbol&match=CRIP2) | DNA binding | [601183](http://www.ncbi.nlm.nih.gov/entrez/dispomim.cgi?id=601183) | -1.24 |
| 1417378_AT | immunoglobulin superfamily, member 4A | [IGSF4A](http://www.gene.ucl.ac.uk/cgi-bin/nomenclature/searchgenes.pl?field=symbol&anchor=equals&symbol_search=Search&number=100&format=html&sortby=symbol&match=IGSF4A) | DNA binding;GTPase activator activity |  | 1.38 |
| 1417522_AT | F-box only protein 32 | [FBXO32](http://www.gene.ucl.ac.uk/cgi-bin/nomenclature/searchgenes.pl?field=symbol&anchor=equals&symbol_search=Search&number=100&format=html&sortby=symbol&match=FBXO32) | DNA binding;nucleic acid binding | [606604](http://www.ncbi.nlm.nih.gov/entrez/dispomim.cgi?id=606604) | 1.81 |
| 1417574_AT | chemokine (C-X-C motif) ligand 12 | [CXCL12](http://www.gene.ucl.ac.uk/cgi-bin/nomenclature/searchgenes.pl?field=symbol&anchor=equals&symbol_search=Search&number=100&format=html&sortby=symbol&match=CXCL12) | DNA binding;nucleic acid binding;mRNA binding | [600835](http://www.ncbi.nlm.nih.gov/entrez/dispomim.cgi?id=600835) | -1.55 |
| 1417601_AT | regulator of G-protein signaling 1 | [RGS1](http://www.gene.ucl.ac.uk/cgi-bin/nomenclature/searchgenes.pl?field=symbol&anchor=equals&symbol_search=Search&number=100&format=html&sortby=symbol&match=RGS1) | DNA binding;protein homodimerization activity | [600323](http://www.ncbi.nlm.nih.gov/entrez/dispomim.cgi?id=600323) | 3.8 |
| 1417633_AT | superoxide dismutase 3, extracellular | [SOD3](http://www.gene.ucl.ac.uk/cgi-bin/nomenclature/searchgenes.pl?field=symbol&anchor=equals&symbol_search=Search&number=100&format=html&sortby=symbol&match=SOD3) | DNA binding;RNA polymerase II transcription factor activity | [185490](http://www.ncbi.nlm.nih.gov/entrez/dispomim.cgi?id=185490) | -1.24 |
| 1417985_AT | Notch-regulated ankyrin repeat protein | [NRARP](http://www.gene.ucl.ac.uk/cgi-bin/nomenclature/searchgenes.pl?field=symbol&anchor=equals&symbol_search=Search&number=100&format=html&sortby=symbol&match=NRARP) | DNA binding;subtilase activity |  | -1.58 |
| 1418059_AT | EGF, latrophilin seven transmembrane domain containing 1 | [ELTD1](http://www.gene.ucl.ac.uk/cgi-bin/nomenclature/searchgenes.pl?field=symbol&anchor=equals&symbol_search=Search&number=100&format=html&sortby=symbol&match=ELTD1) | DNA binding;transcription factor activity |  | -1.39 |
| 1418142_AT | potassium inwardly-rectifying channel, subfamily J, member 8 | [KCNJ8](http://www.gene.ucl.ac.uk/cgi-bin/nomenclature/searchgenes.pl?field=symbol&anchor=equals&symbol_search=Search&number=100&format=html&sortby=symbol&match=KCNJ8) | DNA binding;transcription factor activity | [600935](http://www.ncbi.nlm.nih.gov/entrez/dispomim.cgi?id=600935) | -1.39 |
| 1418187_AT | receptor (calcitonin) activity modifying protein 2 | [RAMP2](http://www.gene.ucl.ac.uk/cgi-bin/nomenclature/searchgenes.pl?field=symbol&anchor=equals&symbol_search=Search&number=100&format=html&sortby=symbol&match=RAMP2) | DNA binding;transcription factor activity | [605154](http://www.ncbi.nlm.nih.gov/entrez/dispomim.cgi?id=605154) | -1.28 |
| 1418280_AT | core promoter element binding protein | [COPEB](http://www.gene.ucl.ac.uk/cgi-bin/nomenclature/searchgenes.pl?field=symbol&anchor=equals&symbol_search=Search&number=100&format=html&sortby=symbol&match=COPEB) | DNA binding;transcription factor activity | [602053](http://www.ncbi.nlm.nih.gov/entrez/dispomim.cgi?id=602053) | 1.43 |
| 1418313_AT | zinc finger protein (C2H2 type) 276 | [ZFP276](http://www.gene.ucl.ac.uk/cgi-bin/nomenclature/searchgenes.pl?field=symbol&anchor=equals&symbol_search=Search&number=100&format=html&sortby=symbol&match=ZFP276) | DNA binding;transcription factor activity | [608460](http://www.ncbi.nlm.nih.gov/entrez/dispomim.cgi?id=608460) | -1.51 |
| 1418446_AT | solute carrier family 16 (monocarboxylic acid transporters), member 2 | [SLC16A2](http://www.gene.ucl.ac.uk/cgi-bin/nomenclature/searchgenes.pl?field=symbol&anchor=equals&symbol_search=Search&number=100&format=html&sortby=symbol&match=SLC16A2) | DNA binding;transcription factor activity;signal transducer activity;histone acetyltransferase binding | [300095](http://www.ncbi.nlm.nih.gov/entrez/dispomim.cgi?id=300095) | 1.25 |
| 1418456_A_AT | chemokine (C-X-C motif) ligand 14 | [CXCL14](http://www.gene.ucl.ac.uk/cgi-bin/nomenclature/searchgenes.pl?field=symbol&anchor=equals&symbol_search=Search&number=100&format=html&sortby=symbol&match=CXCL14) | DNA binding;transcription factor activity;transcriptional activator activity | [604186](http://www.ncbi.nlm.nih.gov/entrez/dispomim.cgi?id=604186) | 1.73 |
| 1418457_AT | chemokine (C-X-C motif) ligand 14 | [CXCL14](http://www.gene.ucl.ac.uk/cgi-bin/nomenclature/searchgenes.pl?field=symbol&anchor=equals&symbol_search=Search&number=100&format=html&sortby=symbol&match=CXCL14) | epoxide hydrolase activity;catalytic activity;aminopeptidase activity;hydrolase activity | [604186](http://www.ncbi.nlm.nih.gov/entrez/dispomim.cgi?id=604186) | 2.48 |
| 1418469_AT | gb:AV272221 /DB_XREF=gi:16390145 /DB_XREF=AV272221 |  | growth factor activity |  | 1.39 |
| 1418472_AT | aspartoacylase (aminoacylase) 2 | [ASPA](http://www.gene.ucl.ac.uk/cgi-bin/nomenclature/searchgenes.pl?field=symbol&anchor=equals&symbol_search=Search&number=100&format=html&sortby=symbol&match=ASPA) | growth factor activity;chemokine activity;cytokine activity | [608034](http://www.ncbi.nlm.nih.gov/entrez/dispomim.cgi?id=608034) | 1.29 |
| 1418473_AT | cutC copper transporter homolog (E.coli) | [CUTC](http://www.gene.ucl.ac.uk/cgi-bin/nomenclature/searchgenes.pl?field=symbol&anchor=equals&symbol_search=Search&number=100&format=html&sortby=symbol&match=CUTC) | growth factor binding;insulin-like growth factor binding;protein binding;heparin binding |  | 1.3 |
| 1418599_AT | procollagen, type XI, alpha 1 | [COL11A1](http://www.gene.ucl.ac.uk/cgi-bin/nomenclature/searchgenes.pl?field=symbol&anchor=equals&symbol_search=Search&number=100&format=html&sortby=symbol&match=COL11A1) | growth factor binding;protein binding;insulin-like growth factor binding;heparin binding | [120280](http://www.ncbi.nlm.nih.gov/entrez/dispomim.cgi?id=120280) | -1.74 |
| 1418945_AT | matrix metalloproteinase 3 | [MMP3](http://www.gene.ucl.ac.uk/cgi-bin/nomenclature/searchgenes.pl?field=symbol&anchor=equals&symbol_search=Search&number=100&format=html&sortby=symbol&match=MMP3) | GTP binding | [185250](http://www.ncbi.nlm.nih.gov/entrez/dispomim.cgi?id=185250) | 3.28 |
| 1419100_AT | serine (or cysteine) proteinase inhibitor, clade A, member 3N | [SERPINA3N](http://www.gene.ucl.ac.uk/cgi-bin/nomenclature/searchgenes.pl?field=symbol&anchor=equals&symbol_search=Search&number=100&format=html&sortby=symbol&match=SERPINA3N) | GTPase activator activity;signal transducer activity |  | 2.67 |
| 1419300_AT | FMS-like tyrosine kinase 1 | [FLT1](http://www.gene.ucl.ac.uk/cgi-bin/nomenclature/searchgenes.pl?field=symbol&anchor=equals&symbol_search=Search&number=100&format=html&sortby=symbol&match=FLT1) | guanyl-nucleotide exchange factor activity | [165070](http://www.ncbi.nlm.nih.gov/entrez/dispomim.cgi?id=165070) | -1.22 |
| 1419589_AT | complement component 1, q subcomponent, receptor 1 | [C1QR1](http://www.gene.ucl.ac.uk/cgi-bin/nomenclature/searchgenes.pl?field=symbol&anchor=equals&symbol_search=Search&number=100&format=html&sortby=symbol&match=C1QR1) | helicase activity;ATP-dependent helicase activity;nucleic acid binding;RNA binding;ATP binding;hydrolase activity | [120577](http://www.ncbi.nlm.nih.gov/entrez/dispomim.cgi?id=120577) | -1.52 |
| 1419684_AT | chemokine (C-C motif) ligand 8 | [CCL8](http://www.gene.ucl.ac.uk/cgi-bin/nomenclature/searchgenes.pl?field=symbol&anchor=equals&symbol_search=Search&number=100&format=html&sortby=symbol&match=CCL8) | histone acetyltransferase activity;protein binding;N-acetyltransferase activity;transferase activity | [602283](http://www.ncbi.nlm.nih.gov/entrez/dispomim.cgi?id=602283) | 1.96 |
| 1419816_S_AT | RIKEN cDNA 1300002F13 gene | [1300002F13RIK](http://www.gene.ucl.ac.uk/cgi-bin/nomenclature/searchgenes.pl?field=symbol&anchor=equals&symbol_search=Search&number=100&format=html&sortby=symbol&match=1300002F13RIK) | holocytochrome-c synthase activity;lyase activity |  | 3.03 |
| 1419833_S_AT | RIKEN cDNA E030006K04 gene | [E030006K04RIK](http://www.gene.ucl.ac.uk/cgi-bin/nomenclature/searchgenes.pl?field=symbol&anchor=equals&symbol_search=Search&number=100&format=html&sortby=symbol&match=E030006K04RIK) | hormone activity |  | -1.3 |
| 1419927_S_AT | RAB interacting factor | [RABIF](http://www.gene.ucl.ac.uk/cgi-bin/nomenclature/searchgenes.pl?field=symbol&anchor=equals&symbol_search=Search&number=100&format=html&sortby=symbol&match=RABIF) | insulin-like growth factor binding;protein binding;integrin binding;heparin binding | [603417](http://www.ncbi.nlm.nih.gov/entrez/dispomim.cgi?id=603417) | -1.28 |
| 1420088_AT | gb:AI462015 /DB_XREF=gi:4316045 /DB_XREF=ub69d10.x |  | isomerase activity;peptidyl-prolyl cis-trans isomerase activity |  | 1.72 |
| 1420089_AT | gb:AI462015 /DB_XREF=gi:4316045 /DB_XREF=ub69d10.x |  | KDEL sequence binding;receptor activity;protein transporter activity |  | 1.6 |
| 1420127_S_AT | cell cycle progression 1 | [CCPG1](http://www.gene.ucl.ac.uk/cgi-bin/nomenclature/searchgenes.pl?field=symbol&anchor=equals&symbol_search=Search&number=100&format=html&sortby=symbol&match=CCPG1) | kinase activity;adenylate kinase activity;shikimate kinase activity;GTP binding;phosphotransferase activity, phosphate group as acceptor;ATP binding;transferase activity |  | 1.27 |
| 1420376_A_AT | H3 histone, family 3B | [H3F3B](http://www.gene.ucl.ac.uk/cgi-bin/nomenclature/searchgenes.pl?field=symbol&anchor=equals&symbol_search=Search&number=100&format=html&sortby=symbol&match=H3F3B) | kinase activity;inositol-trisphosphate 3-kinase activity | [601058](http://www.ncbi.nlm.nih.gov/entrez/dispomim.cgi?id=601058) | 1.33 |
| 1420609_AT | axotrophin | [AXOT](http://www.gene.ucl.ac.uk/cgi-bin/nomenclature/searchgenes.pl?field=symbol&anchor=equals&symbol_search=Search&number=100&format=html&sortby=symbol&match=AXOT) | kinase activity;magnesium ion binding;catalytic activity;ATP binding;6-phosphofructokinase activity;transferase activity |  | 1.21 |
| 1420686_AT | crystallin, beta A4 | [CRYBA4](http://www.gene.ucl.ac.uk/cgi-bin/nomenclature/searchgenes.pl?field=symbol&anchor=equals&symbol_search=Search&number=100&format=html&sortby=symbol&match=CRYBA4) | kinase activity;nucleotidyltransferase activity;transcription factor activity;UTP-glucose-1-phosphate uridylyltransferase activity;transferase activity | [123631](http://www.ncbi.nlm.nih.gov/entrez/dispomim.cgi?id=123631) | -2.3 |
| 1420772_A_AT | delta sleep inducing peptide, immunoreactor | [DSIP1](http://www.gene.ucl.ac.uk/cgi-bin/nomenclature/searchgenes.pl?field=symbol&anchor=equals&symbol_search=Search&number=100&format=html&sortby=symbol&match=DSIP1) | kinase activity;protein kinase activity;ATP binding;transferase activity |  | 1.77 |
| 1420829_A_AT | tyrosine 3-monooxygenase/tryptophan 5-monooxygenase activation protein, theta polypeptide | [YWHAQ](http://www.gene.ucl.ac.uk/cgi-bin/nomenclature/searchgenes.pl?field=symbol&anchor=equals&symbol_search=Search&number=100&format=html&sortby=symbol&match=YWHAQ) | kinase activity;protein kinase activity;protein serine/threonine kinase activity;ATP binding;transferase activity | [609009](http://www.ncbi.nlm.nih.gov/entrez/dispomim.cgi?id=609009) | -1.28 |
| 1420845_AT | mitochondrial ribosomal protein S2 | [MRPS2](http://www.gene.ucl.ac.uk/cgi-bin/nomenclature/searchgenes.pl?field=symbol&anchor=equals&symbol_search=Search&number=100&format=html&sortby=symbol&match=MRPS2) | kinase activity;protein kinase activity;protein serine/threonine kinase activity;ATP binding;transferase activity |  | -1.3 |
| 1420879_A_AT | tyrosine 3-monooxygenase/tryptophan 5-monooxygenase activation protein, beta polypeptide | [YWHAB](http://www.gene.ucl.ac.uk/cgi-bin/nomenclature/searchgenes.pl?field=symbol&anchor=equals&symbol_search=Search&number=100&format=html&sortby=symbol&match=YWHAB) | kinase activity;protein kinase activity;protein serine/threonine kinase activity;ATP binding;transferase activity | [601289](http://www.ncbi.nlm.nih.gov/entrez/dispomim.cgi?id=601289) | -1.26 |
| 1420889_AT | holocytochrome c synthetase | [HCCS](http://www.gene.ucl.ac.uk/cgi-bin/nomenclature/searchgenes.pl?field=symbol&anchor=equals&symbol_search=Search&number=100&format=html&sortby=symbol&match=HCCS) | kinase activity;protein kinase activity;protein serine/threonine kinase activity;ATP binding;transferase activity | [300056](http://www.ncbi.nlm.nih.gov/entrez/dispomim.cgi?id=300056) | -1.21 |
| 1420896_AT | synaptosomal-associated protein 23 | [SNAP23](http://www.gene.ucl.ac.uk/cgi-bin/nomenclature/searchgenes.pl?field=symbol&anchor=equals&symbol_search=Search&number=100&format=html&sortby=symbol&match=SNAP23) | kinase activity;receptor activity;protein serine/threonine kinase activity;ATP binding;transferase activity;vascular endothelial growth factor receptor activity;protein kinase activity;protein-tyrosine kinase activity;transmembrane receptor protein tyrosine kinase activity | [602534](http://www.ncbi.nlm.nih.gov/entrez/dispomim.cgi?id=602534) | -1.26 |
| 1420940_X_AT | regulator of G-protein signaling 5 | [RGS5](http://www.gene.ucl.ac.uk/cgi-bin/nomenclature/searchgenes.pl?field=symbol&anchor=equals&symbol_search=Search&number=100&format=html&sortby=symbol&match=RGS5) | kinase activity;SNF1A/AMP-activated protein kinase activity | [603276](http://www.ncbi.nlm.nih.gov/entrez/dispomim.cgi?id=603276) | -1.28 |
| 1420941_AT | regulator of G-protein signaling 5 | [RGS5](http://www.gene.ucl.ac.uk/cgi-bin/nomenclature/searchgenes.pl?field=symbol&anchor=equals&symbol_search=Search&number=100&format=html&sortby=symbol&match=RGS5) | latrotoxin receptor activity | [603276](http://www.ncbi.nlm.nih.gov/entrez/dispomim.cgi?id=603276) | -1.31 |
| 1420942_S_AT | gb:BF585144 /DB_XREF=gi:11658862 /DB_XREF=60210188 |  | ligand-dependent nuclear receptor activity;steroid hormone receptor activity;DNA binding;receptor activity;transcription factor activity;transcription regulator activity |  | -1.59 |
| 1421027_A_AT | myocyte enhancer factor 2C | [MEF2C](http://www.gene.ucl.ac.uk/cgi-bin/nomenclature/searchgenes.pl?field=symbol&anchor=equals&symbol_search=Search&number=100&format=html&sortby=symbol&match=MEF2C) | ligase activity;glutamate-ammonia ligase activity | [600662](http://www.ncbi.nlm.nih.gov/entrez/dispomim.cgi?id=600662) | 1.27 |
| 1421335_A_AT | EGF-like domain 7 | [EGFL7](http://www.gene.ucl.ac.uk/cgi-bin/nomenclature/searchgenes.pl?field=symbol&anchor=equals&symbol_search=Search&number=100&format=html&sortby=symbol&match=EGFL7) | ligase activity;ubiquitin-protein ligase activity | [608582](http://www.ncbi.nlm.nih.gov/entrez/dispomim.cgi?id=608582) | -1.64 |
| 1421855_AT | fibrinogen-like protein 2 | [FGL2](http://www.gene.ucl.ac.uk/cgi-bin/nomenclature/searchgenes.pl?field=symbol&anchor=equals&symbol_search=Search&number=100&format=html&sortby=symbol&match=FGL2) | L-lactate dehydrogenase activity;oxidoreductase activity | [605351](http://www.ncbi.nlm.nih.gov/entrez/dispomim.cgi?id=605351) | 1.58 |
| 1422047_AT | cadherin 5 | [CDH5](http://www.gene.ucl.ac.uk/cgi-bin/nomenclature/searchgenes.pl?field=symbol&anchor=equals&symbol_search=Search&number=100&format=html&sortby=symbol&match=CDH5) | magnesium ion binding;nucleoside-diphosphatase activity;hydrolase activity | [601120](http://www.ncbi.nlm.nih.gov/entrez/dispomim.cgi?id=601120) | -1.61 |
| 1422134_AT | FBJ osteosarcoma oncogene B | [FOSB](http://www.gene.ucl.ac.uk/cgi-bin/nomenclature/searchgenes.pl?field=symbol&anchor=equals&symbol_search=Search&number=100&format=html&sortby=symbol&match=FOSB) | metal ion binding;antioxidant activity;oxidoreductase activity;superoxide dismutase activity;copper, zinc superoxide dismutase activity | [164772](http://www.ncbi.nlm.nih.gov/entrez/dispomim.cgi?id=164772) | 2.08 |
| 1422264_S_AT | basic transcription element binding protein 1 | [BTEB1](http://www.gene.ucl.ac.uk/cgi-bin/nomenclature/searchgenes.pl?field=symbol&anchor=equals&symbol_search=Search&number=100&format=html&sortby=symbol&match=BTEB1) | methyltransferase activity;S-adenosylmethionine-dependent methyltransferase activity;transferase activity | [602902](http://www.ncbi.nlm.nih.gov/entrez/dispomim.cgi?id=602902) | 1.53 |
| 1422557_S_AT | metallothionein 1 | [MT1](http://www.gene.ucl.ac.uk/cgi-bin/nomenclature/searchgenes.pl?field=symbol&anchor=equals&symbol_search=Search&number=100&format=html&sortby=symbol&match=MT1) | molecular_function unknown |  | 1.73 |
| 1422571_AT | thrombospondin 2 | [THBS2](http://www.gene.ucl.ac.uk/cgi-bin/nomenclature/searchgenes.pl?field=symbol&anchor=equals&symbol_search=Search&number=100&format=html&sortby=symbol&match=THBS2) | molecular_function unknown | [188061](http://www.ncbi.nlm.nih.gov/entrez/dispomim.cgi?id=188061) | -1.64 |
| 1422605_AT | protein phosphatase 1, regulatory (inhibitor) subunit 1A | [PPP1R1A](http://www.gene.ucl.ac.uk/cgi-bin/nomenclature/searchgenes.pl?field=symbol&anchor=equals&symbol_search=Search&number=100&format=html&sortby=symbol&match=PPP1R1A) | molecular_function unknown |  | -1.67 |
| 1422622_AT | nitric oxide synthase 3, endothelial cell | [NOS3](http://www.gene.ucl.ac.uk/cgi-bin/nomenclature/searchgenes.pl?field=symbol&anchor=equals&symbol_search=Search&number=100&format=html&sortby=symbol&match=NOS3) | molecular_function unknown | [163729](http://www.ncbi.nlm.nih.gov/entrez/dispomim.cgi?id=163729) | -1.25 |
| 1422813_AT | gb:NM_007582.1 /DB_XREF=gi:6671657 /GEN=Cacng1 /FE |  | molecular_function unknown |  | 1.41 |
| 1422831_AT | fibrillin 2 | [FBN2](http://www.gene.ucl.ac.uk/cgi-bin/nomenclature/searchgenes.pl?field=symbol&anchor=equals&symbol_search=Search&number=100&format=html&sortby=symbol&match=FBN2) | molecular_function unknown | [121050](http://www.ncbi.nlm.nih.gov/entrez/dispomim.cgi?id=121050) | -2.87 |
| 1422850_AT | gb:AV028400 /DB_XREF=gi:4783308 /DB_XREF=AV028400 |  | molecular_function unknown |  | -1.33 |
| 1422972_S_AT | GCN5 general control of amino acid synthesis-like 2 (yeast) | [GCN5L2](http://www.gene.ucl.ac.uk/cgi-bin/nomenclature/searchgenes.pl?field=symbol&anchor=equals&symbol_search=Search&number=100&format=html&sortby=symbol&match=GCN5L2) | molecular_function unknown | [602301](http://www.ncbi.nlm.nih.gov/entrez/dispomim.cgi?id=602301) | -1.27 |
| 1423037_AT | angiotensin receptor-like 1 | [AGTRL1](http://www.gene.ucl.ac.uk/cgi-bin/nomenclature/searchgenes.pl?field=symbol&anchor=equals&symbol_search=Search&number=100&format=html&sortby=symbol&match=AGTRL1) | molecular_function unknown | [600052](http://www.ncbi.nlm.nih.gov/entrez/dispomim.cgi?id=600052) | -1.62 |
| 1423100_AT | FBJ osteosarcoma oncogene | [FOS](http://www.gene.ucl.ac.uk/cgi-bin/nomenclature/searchgenes.pl?field=symbol&anchor=equals&symbol_search=Search&number=100&format=html&sortby=symbol&match=FOS) | molecular_function unknown | [164810](http://www.ncbi.nlm.nih.gov/entrez/dispomim.cgi?id=164810) | 2.04 |
| 1423153_X_AT | complement component factor h | [CFH](http://www.gene.ucl.ac.uk/cgi-bin/nomenclature/searchgenes.pl?field=symbol&anchor=equals&symbol_search=Search&number=100&format=html&sortby=symbol&match=CFH) | molecular_function unknown | [134370](http://www.ncbi.nlm.nih.gov/entrez/dispomim.cgi?id=134370) | 1.5 |
| 1423233_AT | CCAAT/enhancer binding protein (C/EBP), delta | [CEBPD](http://www.gene.ucl.ac.uk/cgi-bin/nomenclature/searchgenes.pl?field=symbol&anchor=equals&symbol_search=Search&number=100&format=html&sortby=symbol&match=CEBPD) | molecular_function unknown | [116898](http://www.ncbi.nlm.nih.gov/entrez/dispomim.cgi?id=116898) | 2.8 |
| 1423294_AT | mesoderm specific transcript | [MEST](http://www.gene.ucl.ac.uk/cgi-bin/nomenclature/searchgenes.pl?field=symbol&anchor=equals&symbol_search=Search&number=100&format=html&sortby=symbol&match=MEST) | molecular_function unknown | [601029](http://www.ncbi.nlm.nih.gov/entrez/dispomim.cgi?id=601029) | -1.93 |
| 1423452_AT | serine/threonine kinase 17b (apoptosis-inducing) | [STK17B](http://www.gene.ucl.ac.uk/cgi-bin/nomenclature/searchgenes.pl?field=symbol&anchor=equals&symbol_search=Search&number=100&format=html&sortby=symbol&match=STK17B) | molecular_function unknown | [604727](http://www.ncbi.nlm.nih.gov/entrez/dispomim.cgi?id=604727) | 1.44 |
| 1423718_AT | adenylate kinase 3 alpha-like 1 | [AK3L1](http://www.gene.ucl.ac.uk/cgi-bin/nomenclature/searchgenes.pl?field=symbol&anchor=equals&symbol_search=Search&number=100&format=html&sortby=symbol&match=AK3L1) | molecular_function unknown;nucleic acid binding |  | -1.31 |
| 1423865_AT | CDW92 antigen | [CDW92](http://www.gene.ucl.ac.uk/cgi-bin/nomenclature/searchgenes.pl?field=symbol&anchor=equals&symbol_search=Search&number=100&format=html&sortby=symbol&match=CDW92) | molecular_function unknown;protein binding | [606105](http://www.ncbi.nlm.nih.gov/entrez/dispomim.cgi?id=606105) | 1.27 |
| 1424041_S_AT | complement component 1, s subcomponent | [C1S](http://www.gene.ucl.ac.uk/cgi-bin/nomenclature/searchgenes.pl?field=symbol&anchor=equals&symbol_search=Search&number=100&format=html&sortby=symbol&match=C1S) | molecular_function unknown;protein binding | [120580](http://www.ncbi.nlm.nih.gov/entrez/dispomim.cgi?id=120580) | 1.33 |
| 1424158_AT | EH-domain containing 2 | [EHD2](http://www.gene.ucl.ac.uk/cgi-bin/nomenclature/searchgenes.pl?field=symbol&anchor=equals&symbol_search=Search&number=100&format=html&sortby=symbol&match=EHD2) | molecular_function unknown;zinc ion binding;ubiquitin-protein ligase activity | [605890](http://www.ncbi.nlm.nih.gov/entrez/dispomim.cgi?id=605890) | -1.53 |
| 1424213_AT | RIKEN cDNA 1200002M06 gene | [1200002M06RIK](http://www.gene.ucl.ac.uk/cgi-bin/nomenclature/searchgenes.pl?field=symbol&anchor=equals&symbol_search=Search&number=100&format=html&sortby=symbol&match=1200002M06RIK) | NAD(P)+-protein-arginine ADP-ribosyltransferase activity;NAD+ nucleosidase activity;transferase activity, transferring glycosyl groups;transferase activity |  | -1.26 |
| 1424376_AT | CDC42 effector protein (Rho GTPase binding) 1 | [CDC42EP1](http://www.gene.ucl.ac.uk/cgi-bin/nomenclature/searchgenes.pl?field=symbol&anchor=equals&symbol_search=Search&number=100&format=html&sortby=symbol&match=CDC42EP1) | nucleic acid binding | [606084](http://www.ncbi.nlm.nih.gov/entrez/dispomim.cgi?id=606084) | -1.29 |
| 1424408_AT | LIM and senescent cell antigen like domains 2 | [LIMS2](http://www.gene.ucl.ac.uk/cgi-bin/nomenclature/searchgenes.pl?field=symbol&anchor=equals&symbol_search=Search&number=100&format=html&sortby=symbol&match=LIMS2) | nucleic acid binding;ATPase activity;ATP-binding cassette (ABC) transporter activity;ATPase activity, coupled to transmembrane movement of substances;ATP binding | [607908](http://www.ncbi.nlm.nih.gov/entrez/dispomim.cgi?id=607908) | -1.39 |
| 1424520_AT | RIKEN cDNA 2010305A19 gene | [2010305A19RIK](http://www.gene.ucl.ac.uk/cgi-bin/nomenclature/searchgenes.pl?field=symbol&anchor=equals&symbol_search=Search&number=100&format=html&sortby=symbol&match=2010305A19RIK) | nucleic acid binding;pre-mRNA splicing factor activity;RNA binding |  | -1.3 |
| 1424595_AT | F11 receptor | [F11R](http://www.gene.ucl.ac.uk/cgi-bin/nomenclature/searchgenes.pl?field=symbol&anchor=equals&symbol_search=Search&number=100&format=html&sortby=symbol&match=F11R) | nucleic acid binding;pre-mRNA splicing factor activity;RNA binding | [605721](http://www.ncbi.nlm.nih.gov/entrez/dispomim.cgi?id=605721) | -1.33 |
| 1424820_A_AT | Nedd4 family interacting protein 1 | [NDFIP1](http://www.gene.ucl.ac.uk/cgi-bin/nomenclature/searchgenes.pl?field=symbol&anchor=equals&symbol_search=Search&number=100&format=html&sortby=symbol&match=NDFIP1) | nucleic acid binding;RNA binding |  | 1.23 |
| 1424843_A_AT | gb:BC004622.1 /DB_XREF=gi:13435499 /FEA=FLmRNA /CN |  | nucleotidyltransferase activity;transferase activity;phosphatidate cytidylyltransferase activity |  | -1.41 |
| 1424932_AT | epidermal growth factor receptor | [EGFR](http://www.gene.ucl.ac.uk/cgi-bin/nomenclature/searchgenes.pl?field=symbol&anchor=equals&symbol_search=Search&number=100&format=html&sortby=symbol&match=EGFR) | PDZ domain binding | [131550](http://www.ncbi.nlm.nih.gov/entrez/dispomim.cgi?id=131550) | 1.48 |
| 1425210_S_AT | zinc finger protein 84 | [ZFP84](http://www.gene.ucl.ac.uk/cgi-bin/nomenclature/searchgenes.pl?field=symbol&anchor=equals&symbol_search=Search&number=100&format=html&sortby=symbol&match=ZFP84) | phosphatidylinositol binding;clathrin binding |  | -1.44 |
| 1425281_A_AT | delta sleep inducing peptide, immunoreactor | [DSIP1](http://www.gene.ucl.ac.uk/cgi-bin/nomenclature/searchgenes.pl?field=symbol&anchor=equals&symbol_search=Search&number=100&format=html&sortby=symbol&match=DSIP1) | phosphoprotein phosphatase activity;MAP kinase phosphatase activity;protein tyrosine/serine/threonine phosphatase activity;protein tyrosine phosphatase activity;hydrolase activity |  | 1.57 |
| 1425292_AT | dystrobrevin alpha | [DTNA](http://www.gene.ucl.ac.uk/cgi-bin/nomenclature/searchgenes.pl?field=symbol&anchor=equals&symbol_search=Search&number=100&format=html&sortby=symbol&match=DTNA) | phosphoprotein phosphatase activity;sugar binding;receptor activity;protein tyrosine phosphatase activity;hydrolase activity | [601239](http://www.ncbi.nlm.nih.gov/entrez/dispomim.cgi?id=601239) | 1.26 |
| 1425356_AT | Zinc finger protein 142 | [ZFP142](http://www.gene.ucl.ac.uk/cgi-bin/nomenclature/searchgenes.pl?field=symbol&anchor=equals&symbol_search=Search&number=100&format=html&sortby=symbol&match=ZFP142) | potassium channel activity;ion channel activity;inward rectifier potassium channel activity;voltage-gated ion channel activity;ATP-activated inward rectifier potassium channel activity |  | -1.58 |
| 1425550_A_AT | protein kinase, cAMP dependent regulatory, type I, alpha | [PRKAR1A](http://www.gene.ucl.ac.uk/cgi-bin/nomenclature/searchgenes.pl?field=symbol&anchor=equals&symbol_search=Search&number=100&format=html&sortby=symbol&match=PRKAR1A) | protein binding | [188830](http://www.ncbi.nlm.nih.gov/entrez/dispomim.cgi?id=188830) | -1.21 |
| 1425809_AT | Fatty acid binding protein 4, adipocyte | [FABP4](http://www.gene.ucl.ac.uk/cgi-bin/nomenclature/searchgenes.pl?field=symbol&anchor=equals&symbol_search=Search&number=100&format=html&sortby=symbol&match=FABP4) | protein binding | [600434](http://www.ncbi.nlm.nih.gov/entrez/dispomim.cgi?id=600434) | 1.41 |
| 1425810_A_AT | cysteine and glycine-rich protein 1 | [CSRP1](http://www.gene.ucl.ac.uk/cgi-bin/nomenclature/searchgenes.pl?field=symbol&anchor=equals&symbol_search=Search&number=100&format=html&sortby=symbol&match=CSRP1) | protein binding | [123876](http://www.ncbi.nlm.nih.gov/entrez/dispomim.cgi?id=123876) | -1.29 |
| 1426063_A_AT | GTP binding protein (gene overexpressed in skeletal muscle) | [GEM](http://www.gene.ucl.ac.uk/cgi-bin/nomenclature/searchgenes.pl?field=symbol&anchor=equals&symbol_search=Search&number=100&format=html&sortby=symbol&match=GEM) | protein binding | [600164](http://www.ncbi.nlm.nih.gov/entrez/dispomim.cgi?id=600164) | 2.67 |
| 1426144_X_AT | Triadin | [TRDN](http://www.gene.ucl.ac.uk/cgi-bin/nomenclature/searchgenes.pl?field=symbol&anchor=equals&symbol_search=Search&number=100&format=html&sortby=symbol&match=TRDN) | protein binding | [603283](http://www.ncbi.nlm.nih.gov/entrez/dispomim.cgi?id=603283) | 1.23 |
| 1426236_A_AT | glutamate-ammonia ligase (glutamine synthase) | [GLUL](http://www.gene.ucl.ac.uk/cgi-bin/nomenclature/searchgenes.pl?field=symbol&anchor=equals&symbol_search=Search&number=100&format=html&sortby=symbol&match=GLUL) | protein binding | [138290](http://www.ncbi.nlm.nih.gov/entrez/dispomim.cgi?id=138290) | 1.41 |
| 1426461_AT | UDP-glucose pyrophosphorylase 2 | [UGP2](http://www.gene.ucl.ac.uk/cgi-bin/nomenclature/searchgenes.pl?field=symbol&anchor=equals&symbol_search=Search&number=100&format=html&sortby=symbol&match=UGP2) | protein binding | [191760](http://www.ncbi.nlm.nih.gov/entrez/dispomim.cgi?id=191760) | 1.22 |
| 1426554_A_AT | phosphoglycerate mutase 1 | [PGAM1](http://www.gene.ucl.ac.uk/cgi-bin/nomenclature/searchgenes.pl?field=symbol&anchor=equals&symbol_search=Search&number=100&format=html&sortby=symbol&match=PGAM1) | protein binding | [172250](http://www.ncbi.nlm.nih.gov/entrez/dispomim.cgi?id=172250) | -1.53 |
| 1426562_A_AT | olfactomedin 1 | [OLFM1](http://www.gene.ucl.ac.uk/cgi-bin/nomenclature/searchgenes.pl?field=symbol&anchor=equals&symbol_search=Search&number=100&format=html&sortby=symbol&match=OLFM1) | protein binding | [605366](http://www.ncbi.nlm.nih.gov/entrez/dispomim.cgi?id=605366) | -1.41 |
| 1426640_S_AT | tribbles homolog 2 (Drosophila) | [TRIB2](http://www.gene.ucl.ac.uk/cgi-bin/nomenclature/searchgenes.pl?field=symbol&anchor=equals&symbol_search=Search&number=100&format=html&sortby=symbol&match=TRIB2) | protein binding |  | -1.45 |
| 1426678_AT | RIKEN cDNA 3110006P09 gene | [3110006P09RIK](http://www.gene.ucl.ac.uk/cgi-bin/nomenclature/searchgenes.pl?field=symbol&anchor=equals&symbol_search=Search&number=100&format=html&sortby=symbol&match=3110006P09RIK) | protein binding |  | -1.27 |
| 1426680_AT | selenoprotein N, 1 | [SEPN1](http://www.gene.ucl.ac.uk/cgi-bin/nomenclature/searchgenes.pl?field=symbol&anchor=equals&symbol_search=Search&number=100&format=html&sortby=symbol&match=SEPN1) | protein binding |  | -1.24 |
| 1426754_X_AT | Cytoskeleton-associated protein 4 | [CKAP4](http://www.gene.ucl.ac.uk/cgi-bin/nomenclature/searchgenes.pl?field=symbol&anchor=equals&symbol_search=Search&number=100&format=html&sortby=symbol&match=CKAP4) | protein binding |  | -1.39 |
| 1426758_S_AT | GTL2, imprinted maternally expressed untranslated mRNA | [GTL2](http://www.gene.ucl.ac.uk/cgi-bin/nomenclature/searchgenes.pl?field=symbol&anchor=equals&symbol_search=Search&number=100&format=html&sortby=symbol&match=GTL2) | protein binding |  | -3.08 |
| 1426870_AT | F-box only protein 33 | [FBXO33](http://www.gene.ucl.ac.uk/cgi-bin/nomenclature/searchgenes.pl?field=symbol&anchor=equals&symbol_search=Search&number=100&format=html&sortby=symbol&match=FBXO33) | protein binding | [609103](http://www.ncbi.nlm.nih.gov/entrez/dispomim.cgi?id=609103) | 1.78 |
| 1426871_AT | F-box only protein 33 | [FBXO33](http://www.gene.ucl.ac.uk/cgi-bin/nomenclature/searchgenes.pl?field=symbol&anchor=equals&symbol_search=Search&number=100&format=html&sortby=symbol&match=FBXO33) | protein binding | [609103](http://www.ncbi.nlm.nih.gov/entrez/dispomim.cgi?id=609103) | 2.07 |
| 1427059_AT | RIKEN cDNA 4732495E13 gene | [4732495E13RIK](http://www.gene.ucl.ac.uk/cgi-bin/nomenclature/searchgenes.pl?field=symbol&anchor=equals&symbol_search=Search&number=100&format=html&sortby=symbol&match=4732495E13RIK) | protein binding |  | -1.24 |
| 1427200_AT | zinc finger, RAN-binding domain containing 1 | [ZRANB1](http://www.gene.ucl.ac.uk/cgi-bin/nomenclature/searchgenes.pl?field=symbol&anchor=equals&symbol_search=Search&number=100&format=html&sortby=symbol&match=ZRANB1) | protein binding;binding |  | 1.28 |
| 1427202_AT | RIKEN cDNA 4833442J19 gene | [AI256744](http://www.gene.ucl.ac.uk/cgi-bin/nomenclature/searchgenes.pl?field=symbol&anchor=equals&symbol_search=Search&number=100&format=html&sortby=symbol&match=AI256744) | protein binding;calcium ion binding |  | 1.31 |
| 1427486_AT | protein tyrosine phosphatase, receptor type, B | [PTPRB](http://www.gene.ucl.ac.uk/cgi-bin/nomenclature/searchgenes.pl?field=symbol&anchor=equals&symbol_search=Search&number=100&format=html&sortby=symbol&match=PTPRB) | protein binding;calcium ion binding | [176882](http://www.ncbi.nlm.nih.gov/entrez/dispomim.cgi?id=176882) | -1.28 |
| 1427556_AT | myosin, light polypeptide kinase 2, skeletal muscle | [MYLK2](http://www.gene.ucl.ac.uk/cgi-bin/nomenclature/searchgenes.pl?field=symbol&anchor=equals&symbol_search=Search&number=100&format=html&sortby=symbol&match=MYLK2) | protein binding;DNA binding | [606566](http://www.ncbi.nlm.nih.gov/entrez/dispomim.cgi?id=606566) | 1.52 |
| 1427580_A_AT | RNA imprinted and accumulated in nucleus | [RIAN](http://www.gene.ucl.ac.uk/cgi-bin/nomenclature/searchgenes.pl?field=symbol&anchor=equals&symbol_search=Search&number=100&format=html&sortby=symbol&match=RIAN) | protein binding;DNA binding |  | -2.36 |
| 1427683_AT | early growth response 2 | [EGR2](http://www.gene.ucl.ac.uk/cgi-bin/nomenclature/searchgenes.pl?field=symbol&anchor=equals&symbol_search=Search&number=100&format=html&sortby=symbol&match=EGR2) | protein binding;DNA binding | [129010](http://www.ncbi.nlm.nih.gov/entrez/dispomim.cgi?id=129010) | 1.92 |
| 1427932_S_AT | RIKEN cDNA 1200016E24 gene | [1200016E24RIK](http://www.gene.ucl.ac.uk/cgi-bin/nomenclature/searchgenes.pl?field=symbol&anchor=equals&symbol_search=Search&number=100&format=html&sortby=symbol&match=1200016E24RIK) | protein binding;DNA binding |  | 1.49 |
| 1428011_A_AT | Erbb2 interacting protein | [ERBB2IP](http://www.gene.ucl.ac.uk/cgi-bin/nomenclature/searchgenes.pl?field=symbol&anchor=equals&symbol_search=Search&number=100&format=html&sortby=symbol&match=ERBB2IP) | protein binding;DNA binding;calcium ion binding | [606944](http://www.ncbi.nlm.nih.gov/entrez/dispomim.cgi?id=606944) | 1.25 |
| 1428055_AT | RNA imprinted and accumulated in nucleus | [RIAN](http://www.gene.ucl.ac.uk/cgi-bin/nomenclature/searchgenes.pl?field=symbol&anchor=equals&symbol_search=Search&number=100&format=html&sortby=symbol&match=RIAN) | protein binding;enzyme inhibitor activity |  | -2.81 |
| 1428192_AT | RIKEN cDNA 1110008P08 gene | [1110008P08RIK](http://www.gene.ucl.ac.uk/cgi-bin/nomenclature/searchgenes.pl?field=symbol&anchor=equals&symbol_search=Search&number=100&format=html&sortby=symbol&match=1110008P08RIK) | protein binding;enzyme inhibitor activity |  | -1.54 |
| 1428288_AT | RIKEN cDNA 2310051E17 gene | [2310051E17RIK](http://www.gene.ucl.ac.uk/cgi-bin/nomenclature/searchgenes.pl?field=symbol&anchor=equals&symbol_search=Search&number=100&format=html&sortby=symbol&match=2310051E17RIK) | protein binding;isomerase activity;electron transporter activity |  | 1.53 |
| 1428289_AT | basic transcription element binding protein 1 | [BTEB1](http://www.gene.ucl.ac.uk/cgi-bin/nomenclature/searchgenes.pl?field=symbol&anchor=equals&symbol_search=Search&number=100&format=html&sortby=symbol&match=BTEB1) | protein binding;kinase activity;nucleotide binding;3',5'-cAMP binding;cAMP-dependent protein kinase regulator activity | [602902](http://www.ncbi.nlm.nih.gov/entrez/dispomim.cgi?id=602902) | 1.6 |
| 1428306_AT | DNA-damage-inducible transcript 4 | [DDIT4](http://www.gene.ucl.ac.uk/cgi-bin/nomenclature/searchgenes.pl?field=symbol&anchor=equals&symbol_search=Search&number=100&format=html&sortby=symbol&match=DDIT4) | protein binding;molecular_function unknown | [607729](http://www.ncbi.nlm.nih.gov/entrez/dispomim.cgi?id=607729) | 3.05 |
| 1428352_AT | arrestin domain containing 2 | [ARRDC2](http://www.gene.ucl.ac.uk/cgi-bin/nomenclature/searchgenes.pl?field=symbol&anchor=equals&symbol_search=Search&number=100&format=html&sortby=symbol&match=ARRDC2) | protein binding;molecular_function unknown |  | 3.53 |
| 1428416_AT | RIKEN cDNA 3110050N22 gene | [3110050N22RIK](http://www.gene.ucl.ac.uk/cgi-bin/nomenclature/searchgenes.pl?field=symbol&anchor=equals&symbol_search=Search&number=100&format=html&sortby=symbol&match=3110050N22RIK) | protein binding;nucleic acid binding;RNA binding |  | 1.39 |
| 1428418_S_AT | RIKEN cDNA 3110050N22 gene | [3110050N22RIK](http://www.gene.ucl.ac.uk/cgi-bin/nomenclature/searchgenes.pl?field=symbol&anchor=equals&symbol_search=Search&number=100&format=html&sortby=symbol&match=3110050N22RIK) | protein binding;odorant binding |  | 1.48 |
| 1428765_AT | GTL2, imprinted maternally expressed untranslated mRNA | [GTL2](http://www.gene.ucl.ac.uk/cgi-bin/nomenclature/searchgenes.pl?field=symbol&anchor=equals&symbol_search=Search&number=100&format=html&sortby=symbol&match=GTL2) | protein binding;odorant binding |  | -2.01 |
| 1428766_AT | RIKEN cDNA 4833420N02 gene | [RNMTL1](http://www.gene.ucl.ac.uk/cgi-bin/nomenclature/searchgenes.pl?field=symbol&anchor=equals&symbol_search=Search&number=100&format=html&sortby=symbol&match=RNMTL1) | protein binding;peptidase activity |  | -1.26 |
| 1428776_AT | RIKEN cDNA 8430417G17 gene | [8430417G17RIK](http://www.gene.ucl.ac.uk/cgi-bin/nomenclature/searchgenes.pl?field=symbol&anchor=equals&symbol_search=Search&number=100&format=html&sortby=symbol&match=8430417G17RIK) | protein binding;protein domain specific binding |  | 1.82 |
| 1428942_AT | metallothionein 2 | [MT2](http://www.gene.ucl.ac.uk/cgi-bin/nomenclature/searchgenes.pl?field=symbol&anchor=equals&symbol_search=Search&number=100&format=html&sortby=symbol&match=MT2) | protein binding;protein domain specific binding;monooxygenase activity |  | 2.08 |
| 1429004_AT | pleckstrin homology domain interacting protein | [PHIP](http://www.gene.ucl.ac.uk/cgi-bin/nomenclature/searchgenes.pl?field=symbol&anchor=equals&symbol_search=Search&number=100&format=html&sortby=symbol&match=PHIP) | protein binding;protein kinase binding;insulin receptor binding |  | 1.29 |
| 1429129_AT | RIKEN cDNA 1200008A14 gene | [1200008A14RIK](http://www.gene.ucl.ac.uk/cgi-bin/nomenclature/searchgenes.pl?field=symbol&anchor=equals&symbol_search=Search&number=100&format=html&sortby=symbol&match=1200008A14RIK) | protein binding;protein kinase binding;insulin receptor binding |  | 1.52 |
| 1429169_AT | RNA binding motif protein 3 | [RBM3](http://www.gene.ucl.ac.uk/cgi-bin/nomenclature/searchgenes.pl?field=symbol&anchor=equals&symbol_search=Search&number=100&format=html&sortby=symbol&match=RBM3) | protein binding;protein kinase C binding;sugar porter activity | [300027](http://www.ncbi.nlm.nih.gov/entrez/dispomim.cgi?id=300027) | -5.14 |
| 1429201_AT | cylindromatosis (turban tumor syndrome) | [CYLD](http://www.gene.ucl.ac.uk/cgi-bin/nomenclature/searchgenes.pl?field=symbol&anchor=equals&symbol_search=Search&number=100&format=html&sortby=symbol&match=CYLD) | protein binding;protein transporter activity;t-SNARE activity | [605018](http://www.ncbi.nlm.nih.gov/entrez/dispomim.cgi?id=605018) | 1.25 |
| 1429257_AT | GTL2, imprinted maternally expressed untranslated mRNA | [GTL2](http://www.gene.ucl.ac.uk/cgi-bin/nomenclature/searchgenes.pl?field=symbol&anchor=equals&symbol_search=Search&number=100&format=html&sortby=symbol&match=GTL2) | protein binding;receptor activity;SH3/SH2 adaptor protein activity |  | -1.82 |
| 1429273_AT | BMP-binding endothelial regulator | [BMPER](http://www.gene.ucl.ac.uk/cgi-bin/nomenclature/searchgenes.pl?field=symbol&anchor=equals&symbol_search=Search&number=100&format=html&sortby=symbol&match=BMPER) | protein binding;structural constituent of ribosome | [608699](http://www.ncbi.nlm.nih.gov/entrez/dispomim.cgi?id=608699) | 1.65 |
| 1429555_AT | RIKEN cDNA 1110019C08 gene | [1110019C08RIK](http://www.gene.ucl.ac.uk/cgi-bin/nomenclature/searchgenes.pl?field=symbol&anchor=equals&symbol_search=Search&number=100&format=html&sortby=symbol&match=1110019C08RIK) | protein binding;structural constituent of ribosome |  | -1.21 |
| 1429618_AT | cylindromatosis (turban tumor syndrome) | [CYLD](http://www.gene.ucl.ac.uk/cgi-bin/nomenclature/searchgenes.pl?field=symbol&anchor=equals&symbol_search=Search&number=100&format=html&sortby=symbol&match=CYLD) | protein binding;sugar binding;calcium ion binding;receptor activity | [605018](http://www.ncbi.nlm.nih.gov/entrez/dispomim.cgi?id=605018) | 1.36 |
| 1429749_AT | RIKEN cDNA 9330180L21 gene | [9330180L21RIK](http://www.gene.ucl.ac.uk/cgi-bin/nomenclature/searchgenes.pl?field=symbol&anchor=equals&symbol_search=Search&number=100&format=html&sortby=symbol&match=9330180L21RIK) | protein binding;sugar binding;calcium ion binding;receptor activity |  | 1.34 |
| 1429810_AT | RIKEN cDNA 4921505C17 gene | [4921505C17RIK](http://www.gene.ucl.ac.uk/cgi-bin/nomenclature/searchgenes.pl?field=symbol&anchor=equals&symbol_search=Search&number=100&format=html&sortby=symbol&match=4921505C17RIK) | protein binding;zinc ion binding |  | 1.4 |
| 1429900_AT | RIKEN cDNA 5330406M23 gene | [5330406M23RIK](http://www.gene.ucl.ac.uk/cgi-bin/nomenclature/searchgenes.pl?field=symbol&anchor=equals&symbol_search=Search&number=100&format=html&sortby=symbol&match=5330406M23RIK) | protein binding;zinc ion binding |  | 2.33 |
| 1430089_AT | RIKEN cDNA 5830469G19 gene | [5830469G19RIK](http://www.gene.ucl.ac.uk/cgi-bin/nomenclature/searchgenes.pl?field=symbol&anchor=equals&symbol_search=Search&number=100&format=html&sortby=symbol&match=5830469G19RIK) | protein binding;zinc ion binding;nucleic acid binding;DNA binding |  | -1.6 |
| 1430164_A_AT | growth factor receptor bound protein 10 | [GRB10](http://www.gene.ucl.ac.uk/cgi-bin/nomenclature/searchgenes.pl?field=symbol&anchor=equals&symbol_search=Search&number=100&format=html&sortby=symbol&match=GRB10) | protein domain specific binding | [601523](http://www.ncbi.nlm.nih.gov/entrez/dispomim.cgi?id=601523) | -1.45 |
| 1430357_AT | RIKEN cDNA 9430068D06 gene | [9430068D06RIK](http://www.gene.ucl.ac.uk/cgi-bin/nomenclature/searchgenes.pl?field=symbol&anchor=equals&symbol_search=Search&number=100&format=html&sortby=symbol&match=9430068D06RIK) | protein domain specific binding;calcium ion binding;GTPase activity;GTP binding;ATP binding |  | 4.13 |
| 1430404_AT | RIKEN cDNA 4833416J08 gene | [4833416J08RIK](http://www.gene.ucl.ac.uk/cgi-bin/nomenclature/searchgenes.pl?field=symbol&anchor=equals&symbol_search=Search&number=100&format=html&sortby=symbol&match=4833416J08RIK) | protein domain specific binding;GTPase activity;calcium ion binding;GTP binding;ATP binding |  | 1.76 |
| 1430542_A_AT | solute carrier family 25 (mitochondrial carrier, adenine nucleotide translocator), member 5 | [SLC25A5](http://www.gene.ucl.ac.uk/cgi-bin/nomenclature/searchgenes.pl?field=symbol&anchor=equals&symbol_search=Search&number=100&format=html&sortby=symbol&match=SLC25A5) | protein kinase activity;kinase activity;protein serine/threonine kinase activity;ATP binding;transferase activity | [300150](http://www.ncbi.nlm.nih.gov/entrez/dispomim.cgi?id=300150) | -1.29 |
| 1430556_AT | gb:AK014638.1 /DB_XREF=gi:12852619 /FEA=mRNA /CNT= |  | protein kinase activity;kinase activity;protein serine/threonine kinase activity;small GTPase regulatory/interacting protein activity;ATP binding;protein-tyrosine kinase activity;transferase activity |  | 1.68 |
| 1430560_AT | protein phosphatase 1, regulatory subunit 10 | [PPP1R10](http://www.gene.ucl.ac.uk/cgi-bin/nomenclature/searchgenes.pl?field=symbol&anchor=equals&symbol_search=Search&number=100&format=html&sortby=symbol&match=PPP1R10) | protein kinase activity;protein serine/threonine kinase activity;ATP binding;myosin-light-chain kinase activity;calmodulin binding;transferase activity | [603771](http://www.ncbi.nlm.nih.gov/entrez/dispomim.cgi?id=603771) | -1.64 |
| 1430787_AT | RIKEN cDNA 2310050B05 gene | [2310050B05RIK](http://www.gene.ucl.ac.uk/cgi-bin/nomenclature/searchgenes.pl?field=symbol&anchor=equals&symbol_search=Search&number=100&format=html&sortby=symbol&match=2310050B05RIK) | protein kinase activity;protein serine/threonine kinase activity;ATP binding;protein-tyrosine kinase activity;transferase activity |  | 1.63 |
| 1430982_AT | splicing factor, arginine/serine-rich 1 (ASF/SF2) | [SFRS1](http://www.gene.ucl.ac.uk/cgi-bin/nomenclature/searchgenes.pl?field=symbol&anchor=equals&symbol_search=Search&number=100&format=html&sortby=symbol&match=SFRS1) | protein phosphatase inhibitor activity | [600812](http://www.ncbi.nlm.nih.gov/entrez/dispomim.cgi?id=600812) | -1.3 |
| 1431037_A_AT | ELAV (embryonic lethal, abnormal vision, Drosophila)-like 1 (Hu antigen R) | [ELAVL1](http://www.gene.ucl.ac.uk/cgi-bin/nomenclature/searchgenes.pl?field=symbol&anchor=equals&symbol_search=Search&number=100&format=html&sortby=symbol&match=ELAVL1) | protein translocase activity | [603466](http://www.ncbi.nlm.nih.gov/entrez/dispomim.cgi?id=603466) | -1.39 |
| 1431094_AT | RIKEN cDNA 1110006E14 gene | [1110006E14RIK](http://www.gene.ucl.ac.uk/cgi-bin/nomenclature/searchgenes.pl?field=symbol&anchor=equals&symbol_search=Search&number=100&format=html&sortby=symbol&match=1110006E14RIK) | purinergic nucleotide receptor activity, G-protein coupled;receptor activity;G-protein coupled receptor activity;rhodopsin-like receptor activity |  | -2.57 |
| 1431429_A_AT | ADP-ribosylation factor-like 4 | [ARL4](http://www.gene.ucl.ac.uk/cgi-bin/nomenclature/searchgenes.pl?field=symbol&anchor=equals&symbol_search=Search&number=100&format=html&sortby=symbol&match=ARL4) | purinergic nucleotide receptor activity, G-protein coupled;receptor activity;G-protein coupled receptor activity;rhodopsin-like receptor activity | [604786](http://www.ncbi.nlm.nih.gov/entrez/dispomim.cgi?id=604786) | 1.29 |
| 1433148_AT | gb:AK015786.1 /DB_XREF=gi:12854257 /FEA=mRNA /CNT= |  | receptor activity;GTP binding |  | 2.01 |
| 1433293_AT | RIKEN cDNA 1500032O14 gene | [1500032O14RIK](http://www.gene.ucl.ac.uk/cgi-bin/nomenclature/searchgenes.pl?field=symbol&anchor=equals&symbol_search=Search&number=100&format=html&sortby=symbol&match=1500032O14RIK) | receptor activity;kinase activity;protein serine/threonine kinase activity;ATP binding;transferase activity;protein binding;protein kinase activity;protein-tyrosine kinase activity;signal transducer activity;epidermal growth factor receptor activity;transmembrane receptor protein tyrosine kinase activity |  | -1.66 |
| 1433508_AT | gb:AV025472 /DB_XREF=gi:16356441 /DB_XREF=AV025472 |  | receptor activity;kinase activity;protein serine/threonine kinase activity;ATP binding;transferase activity;vascular endothelial growth factor receptor activity;protein kinase activity;protein-tyrosine kinase activity;transmembrane receptor protein tyrosine kinase activity |  | 1.55 |
| 1433532_A_AT | myelin basic protein | [MBP](http://www.gene.ucl.ac.uk/cgi-bin/nomenclature/searchgenes.pl?field=symbol&anchor=equals&symbol_search=Search&number=100&format=html&sortby=symbol&match=MBP) | receptor activity;kinase activity;transmembrane receptor protein serine/threonine kinase activity;protein serine/threonine kinase activity;ATP binding;transforming growth factor beta receptor activity;transferase activity;receptor signaling protein serine/threonine kinase activity;protein kinase activity;protein-tyrosine kinase activity | [159430](http://www.ncbi.nlm.nih.gov/entrez/dispomim.cgi?id=159430) | -1.69 |
| 1433836_A_AT | RIKEN cDNA 8430408G22 gene | [8430408G22RIK](http://www.gene.ucl.ac.uk/cgi-bin/nomenclature/searchgenes.pl?field=symbol&anchor=equals&symbol_search=Search&number=100&format=html&sortby=symbol&match=8430408G22RIK) | receptor activity;protein transporter activity;coreceptor, soluble ligand activity |  | 4.9 |
| 1433837_AT | RIKEN cDNA 8430408G22 gene | [8430408G22RIK](http://www.gene.ucl.ac.uk/cgi-bin/nomenclature/searchgenes.pl?field=symbol&anchor=equals&symbol_search=Search&number=100&format=html&sortby=symbol&match=8430408G22RIK) | receptor binding;hormone activity |  | 3 |
| 1433956_AT | cadherin 5 | [CDH5](http://www.gene.ucl.ac.uk/cgi-bin/nomenclature/searchgenes.pl?field=symbol&anchor=equals&symbol_search=Search&number=100&format=html&sortby=symbol&match=CDH5) | RNA binding | [601120](http://www.ncbi.nlm.nih.gov/entrez/dispomim.cgi?id=601120) | -1.5 |
| 1434025_AT | gb:BG069607 /DB_XREF=gi:12552176 /DB_XREF=H3078B09 |  | RNA binding |  | 1.39 |
| 1434157_AT | taxilin | [TXLN](http://www.gene.ucl.ac.uk/cgi-bin/nomenclature/searchgenes.pl?field=symbol&anchor=equals&symbol_search=Search&number=100&format=html&sortby=symbol&match=TXLN) | RNA binding |  | -1.33 |
| 1434170_AT | WD repeat domain 40B | [WDR40B](http://www.gene.ucl.ac.uk/cgi-bin/nomenclature/searchgenes.pl?field=symbol&anchor=equals&symbol_search=Search&number=100&format=html&sortby=symbol&match=WDR40B) | RNA binding |  | -2.4 |
| 1434411_AT | gb:BB114398 /DB_XREF=gi:16261775 /DB_XREF=BB114398 |  | RNA binding |  | -1.76 |
| 1434424_AT | RIKEN cDNA 9630055N22 gene | [9630055N22RIK](http://www.gene.ucl.ac.uk/cgi-bin/nomenclature/searchgenes.pl?field=symbol&anchor=equals&symbol_search=Search&number=100&format=html&sortby=symbol&match=9630055N22RIK) | RNA binding;structural constituent of ribosome |  | 1.84 |
| 1434805_AT | myeloid/lymphoid or mixed lineage-leukemia translocation to 1 homolog (Drosophila) | [MLLT1](http://www.gene.ucl.ac.uk/cgi-bin/nomenclature/searchgenes.pl?field=symbol&anchor=equals&symbol_search=Search&number=100&format=html&sortby=symbol&match=MLLT1) | RNA methyltransferase activity;methyltransferase activity;RNA binding;transferase activity | [159556](http://www.ncbi.nlm.nih.gov/entrez/dispomim.cgi?id=159556) | -1.34 |
| 1434975_X_AT | similar to phosphatidylserine decarboxylase | [MGC65558](http://www.gene.ucl.ac.uk/cgi-bin/nomenclature/searchgenes.pl?field=symbol&anchor=equals&symbol_search=Search&number=100&format=html&sortby=symbol&match=MGC65558) | serine-type endopeptidase inhibitor activity |  | 1.28 |
| 1435137_S_AT | RIKEN cDNA 1200015M12 gene | [1200015M12RIK](http://www.gene.ucl.ac.uk/cgi-bin/nomenclature/searchgenes.pl?field=symbol&anchor=equals&symbol_search=Search&number=100&format=html&sortby=symbol&match=1200015M12RIK) | serine-type endopeptidase inhibitor activity |  | 1.61 |
| 1435265_AT | gb:BF466929 /DB_XREF=gi:11536112 /DB_XREF=UI-M-CG0 |  | serine-type endopeptidase inhibitor activity;endopeptidase inhibitor activity;peptidase activity |  | 1.81 |
| 1435284_AT | reticulon 4 | [RTN4](http://www.gene.ucl.ac.uk/cgi-bin/nomenclature/searchgenes.pl?field=symbol&anchor=equals&symbol_search=Search&number=100&format=html&sortby=symbol&match=RTN4) | signal transducer activity | [604475](http://www.ncbi.nlm.nih.gov/entrez/dispomim.cgi?id=604475) | -2.51 |
| 1435327_AT | expressed sequence AW112037 | [AW112037](http://www.gene.ucl.ac.uk/cgi-bin/nomenclature/searchgenes.pl?field=symbol&anchor=equals&symbol_search=Search&number=100&format=html&sortby=symbol&match=AW112037) | signal transducer activity |  | -1.28 |
| 1435333_AT | gb:AV156568 /DB_XREF=gi:16383280 /DB_XREF=AV156568 |  | signal transducer activity;GTPase activator activity |  | -1.26 |
| 1435547_AT | gb:BQ175722 /DB_XREF=gi:20351214 /DB_XREF=UI-M-DJ2 |  | signal transducer activity;GTPase activator activity |  | 1.23 |
| 1435640_X_AT | gb:BE634869 /DB_XREF=gi:9917484 /DB_XREF=uv85h11.x |  | signal transducer activity;GTPase activator activity |  | 1.58 |
| 1435693_AT | cDNA sequence BC012256 | [BC012256](http://www.gene.ucl.ac.uk/cgi-bin/nomenclature/searchgenes.pl?field=symbol&anchor=equals&symbol_search=Search&number=100&format=html&sortby=symbol&match=BC012256) | structural constituent of cell wall;RNA binding;pre-mRNA splicing factor activity |  | -1.36 |
| 1435697_A_AT | pleckstrin homology, Sec7 and coiled-coil domains, binding protein | [PSCDBP](http://www.gene.ucl.ac.uk/cgi-bin/nomenclature/searchgenes.pl?field=symbol&anchor=equals&symbol_search=Search&number=100&format=html&sortby=symbol&match=PSCDBP) | structural constituent of eye lens | [604448](http://www.ncbi.nlm.nih.gov/entrez/dispomim.cgi?id=604448) | 1.7 |
| 1435785_AT | EH-domain containing 2 | [EHD2](http://www.gene.ucl.ac.uk/cgi-bin/nomenclature/searchgenes.pl?field=symbol&anchor=equals&symbol_search=Search&number=100&format=html&sortby=symbol&match=EHD2) | structural constituent of ribosome | [605890](http://www.ncbi.nlm.nih.gov/entrez/dispomim.cgi?id=605890) | -1.48 |
| 1435823_X_AT | EGF-like domain 7 | [EGFL7](http://www.gene.ucl.ac.uk/cgi-bin/nomenclature/searchgenes.pl?field=symbol&anchor=equals&symbol_search=Search&number=100&format=html&sortby=symbol&match=EGFL7) | structural constituent of ribosome | [608582](http://www.ncbi.nlm.nih.gov/entrez/dispomim.cgi?id=608582) | -1.39 |
| 1435875_AT | protein kinase, AMP-activated, beta 2 non-catalytic subunit | [PRKAB2](http://www.gene.ucl.ac.uk/cgi-bin/nomenclature/searchgenes.pl?field=symbol&anchor=equals&symbol_search=Search&number=100&format=html&sortby=symbol&match=PRKAB2) | structural molecule activity;extracellular matrix structural constituent;extracellular matrix structural constituent conferring tensile strength | [602741](http://www.ncbi.nlm.nih.gov/entrez/dispomim.cgi?id=602741) | 1.24 |
| 1436033_AT | cDNA sequence BC031353 | [BC031353](http://www.gene.ucl.ac.uk/cgi-bin/nomenclature/searchgenes.pl?field=symbol&anchor=equals&symbol_search=Search&number=100&format=html&sortby=symbol&match=BC031353) | structural molecule activity;extracellular matrix structural constituent;extracellular matrix structural constituent conferring tensile strength |  | 1.57 |
| 1436202_AT | RIKEN cDNA 9430072K23 gene | [9430072K23RIK](http://www.gene.ucl.ac.uk/cgi-bin/nomenclature/searchgenes.pl?field=symbol&anchor=equals&symbol_search=Search&number=100&format=html&sortby=symbol&match=9430072K23RIK) | structural molecule activity;GTPase activity;GTP binding;structural constituent of cytoskeleton |  | 1.94 |
| 1436214_AT | RIKEN cDNA C430010P07 gene | [C430010P07RIK](http://www.gene.ucl.ac.uk/cgi-bin/nomenclature/searchgenes.pl?field=symbol&anchor=equals&symbol_search=Search&number=100&format=html&sortby=symbol&match=C430010P07RIK) | structural molecule activity;motor activity;structural constituent of cytoskeleton |  | 1.29 |
| 1436240_AT | RIKEN cDNA B230214O09 gene | [B230214O09RIK](http://www.gene.ucl.ac.uk/cgi-bin/nomenclature/searchgenes.pl?field=symbol&anchor=equals&symbol_search=Search&number=100&format=html&sortby=symbol&match=B230214O09RIK) | structural molecule activity;protein binding;calcium ion binding;heparin binding |  | -1.77 |
| 1436367_AT | gb:BB119527 /DB_XREF=gi:16261957 /DB_XREF=BB119527 |  | structural molecule activity;protein binding;extracellular matrix structural constituent |  | -1.27 |
| 1436509_AT | RIKEN cDNA 2410014A08 gene | [2410014A08RIK](http://www.gene.ucl.ac.uk/cgi-bin/nomenclature/searchgenes.pl?field=symbol&anchor=equals&symbol_search=Search&number=100&format=html&sortby=symbol&match=2410014A08RIK) | structural molecule activity;structural constituent of myelin sheath |  | -1.33 |
| 1436627_AT | Hypothetical gene supported by AK048221 | [LOC433065](http://www.gene.ucl.ac.uk/cgi-bin/nomenclature/searchgenes.pl?field=symbol&anchor=equals&symbol_search=Search&number=100&format=html&sortby=symbol&match=LOC433065) | sugar porter activity |  | 1.32 |
| 1436763_A_AT | basic transcription element binding protein 1 | [BTEB1](http://www.gene.ucl.ac.uk/cgi-bin/nomenclature/searchgenes.pl?field=symbol&anchor=equals&symbol_search=Search&number=100&format=html&sortby=symbol&match=BTEB1) | symporter activity;transporter activity;carrier activity | [602902](http://www.ncbi.nlm.nih.gov/entrez/dispomim.cgi?id=602902) | 1.74 |
| 1436874_X_AT | solute carrier family 25 (mitochondrial carrier, adenine nucleotide translocator), member 5 | [SLC25A5](http://www.gene.ucl.ac.uk/cgi-bin/nomenclature/searchgenes.pl?field=symbol&anchor=equals&symbol_search=Search&number=100&format=html&sortby=symbol&match=SLC25A5) | transcription coactivator activity;protein binding;DNA binding;transcription factor activity;RNA polymerase II transcription factor activity | [300150](http://www.ncbi.nlm.nih.gov/entrez/dispomim.cgi?id=300150) | -1.33 |
| 1436919_AT | tumor protein p53 inducible protein 11 | [TP53I11](http://www.gene.ucl.ac.uk/cgi-bin/nomenclature/searchgenes.pl?field=symbol&anchor=equals&symbol_search=Search&number=100&format=html&sortby=symbol&match=TP53I11) | transcription coactivator activity;protein binding;DNA binding;transcription factor activity;RNA polymerase II transcription factor activity |  | -1.34 |
| 1436952_AT | basic transcription element binding protein 1 | [BTEB1](http://www.gene.ucl.ac.uk/cgi-bin/nomenclature/searchgenes.pl?field=symbol&anchor=equals&symbol_search=Search&number=100&format=html&sortby=symbol&match=BTEB1) | transcription factor activity | [602902](http://www.ncbi.nlm.nih.gov/entrez/dispomim.cgi?id=602902) | 1.46 |
| 1436994_A_AT | histone 1, H1c | [HIST1H1C](http://www.gene.ucl.ac.uk/cgi-bin/nomenclature/searchgenes.pl?field=symbol&anchor=equals&symbol_search=Search&number=100&format=html&sortby=symbol&match=HIST1H1C) | transcription factor activity | [142710](http://www.ncbi.nlm.nih.gov/entrez/dispomim.cgi?id=142710) | 1.51 |
| 1437064_AT | expressed sequence AW320017 | [AW320017](http://www.gene.ucl.ac.uk/cgi-bin/nomenclature/searchgenes.pl?field=symbol&anchor=equals&symbol_search=Search&number=100&format=html&sortby=symbol&match=AW320017) | transcription regulator activity;structural constituent of ribosome |  | 1.68 |
| 1437100_X_AT | proviral integration site 3 | [PIM3](http://www.gene.ucl.ac.uk/cgi-bin/nomenclature/searchgenes.pl?field=symbol&anchor=equals&symbol_search=Search&number=100&format=html&sortby=symbol&match=PIM3) | transferase activity, transferring glycosyl groups;transferase activity |  | 1.8 |
| 1437584_AT | gb:BE685667 /DB_XREF=gi:10073343 /DB_XREF=uu57b06. |  | transmembrane receptor activity;Wnt-protein binding |  | 2.48 |
| 1437591_A_AT | WD repeat domain 1 | [WDR1](http://www.gene.ucl.ac.uk/cgi-bin/nomenclature/searchgenes.pl?field=symbol&anchor=equals&symbol_search=Search&number=100&format=html&sortby=symbol&match=WDR1) | transporter activity;binding | [604734](http://www.ncbi.nlm.nih.gov/entrez/dispomim.cgi?id=604734) | -1.34 |
| 1437685_X_AT | fibromodulin | [FMOD](http://www.gene.ucl.ac.uk/cgi-bin/nomenclature/searchgenes.pl?field=symbol&anchor=equals&symbol_search=Search&number=100&format=html&sortby=symbol&match=FMOD) | transporter activity;binding | [600245](http://www.ncbi.nlm.nih.gov/entrez/dispomim.cgi?id=600245) | -1.65 |
| 1437718_X_AT | fibromodulin | [FMOD](http://www.gene.ucl.ac.uk/cgi-bin/nomenclature/searchgenes.pl?field=symbol&anchor=equals&symbol_search=Search&number=100&format=html&sortby=symbol&match=FMOD) | transporter activity;GTPase activity;GTP binding | [600245](http://www.ncbi.nlm.nih.gov/entrez/dispomim.cgi?id=600245) | -1.8 |
| 1437845_X_AT | gb:BB027731 /DB_XREF=gi:8205516 /DB_XREF=BB027731 |  | transporter activity;lipid binding;binding |  | -1.2 |
| 1437892_AT | gb:BQ084812 /DB_XREF=gi:20044016 /DB_XREF=ii48e11. |  | trypsin activity;calcium ion binding;serine-type endopeptidase activity;hydrolase activity;peptidase activity;chymotrypsin activity |  | -1.75 |
| 1437917_AT | RIKEN cDNA D530037H12 gene | [D530037H12RIK](http://www.gene.ucl.ac.uk/cgi-bin/nomenclature/searchgenes.pl?field=symbol&anchor=equals&symbol_search=Search&number=100&format=html&sortby=symbol&match=D530037H12RIK) | tubulin-tyrosine ligase activity;ligase activity |  | -1.45 |
| 1438041_AT | gb:BG963325 /DB_XREF=gi:14350962 /DB_XREF=60282774 |  | ubiquitin thiolesterase activity;cysteine-type endopeptidase activity;hydrolase activity;cysteine-type peptidase activity |  | 1.48 |
| 1438059_AT | Transcribed locus, weakly similar to NP_899138.1 cortexin [Mus musculus] |  | ubiquitin thiolesterase activity;cysteine-type endopeptidase activity;hydrolase activity;cysteine-type peptidase activity |  | 2.16 |
| 1438133_A_AT | cysteine rich protein 61 | [CYR61](http://www.gene.ucl.ac.uk/cgi-bin/nomenclature/searchgenes.pl?field=symbol&anchor=equals&symbol_search=Search&number=100&format=html&sortby=symbol&match=CYR61) | ubiquitin thiolesterase activity;cysteine-type endopeptidase activity;hydrolase activity;cysteine-type peptidase activity | [602369](http://www.ncbi.nlm.nih.gov/entrez/dispomim.cgi?id=602369) | 2.31 |
| 1438157_S_AT | nuclear factor of kappa light chain gene enhancer in B-cells inhibitor, alpha | [NFKBIA](http://www.gene.ucl.ac.uk/cgi-bin/nomenclature/searchgenes.pl?field=symbol&anchor=equals&symbol_search=Search&number=100&format=html&sortby=symbol&match=NFKBIA) | zinc ion binding | [164008](http://www.ncbi.nlm.nih.gov/entrez/dispomim.cgi?id=164008) | 2.01 |
| 1438215_AT | splicing factor, arginine/serine-rich 3 (SRp20) | [SFRS3](http://www.gene.ucl.ac.uk/cgi-bin/nomenclature/searchgenes.pl?field=symbol&anchor=equals&symbol_search=Search&number=100&format=html&sortby=symbol&match=SFRS3) | zinc ion binding;actin binding | [603364](http://www.ncbi.nlm.nih.gov/entrez/dispomim.cgi?id=603364) | -1.26 |
| 1438244_AT | gb:BB092799 /DB_XREF=gi:15407789 /DB_XREF=BB092799 |  | zinc ion binding;carboxypeptidase A activity;carboxypeptidase activity;metallopeptidase activity;carboxypeptidase E activity;metallocarboxypeptidase activity;hydrolase activity |  | -1.31 |
| 1438245_AT | gb:BI664122 /DB_XREF=gi:15578355 /DB_XREF=60328923 |  | zinc ion binding;DNA binding;nucleic acid binding |  | -1.43 |
| 1438321_X_AT | RIKEN cDNA 4930504E06 gene | [4930504E06RIK](http://www.gene.ucl.ac.uk/cgi-bin/nomenclature/searchgenes.pl?field=symbol&anchor=equals&symbol_search=Search&number=100&format=html&sortby=symbol&match=4930504E06RIK) | zinc ion binding;DNA binding;nucleic acid binding |  | 1.24 |
| 1438651_A_AT | angiotensin receptor-like 1 | [AGTRL1](http://www.gene.ucl.ac.uk/cgi-bin/nomenclature/searchgenes.pl?field=symbol&anchor=equals&symbol_search=Search&number=100&format=html&sortby=symbol&match=AGTRL1) | zinc ion binding;DNA binding;nucleic acid binding | [600052](http://www.ncbi.nlm.nih.gov/entrez/dispomim.cgi?id=600052) | -1.88 |
| 1438704_AT | Ring finger protein 28 | [RNF28](http://www.gene.ucl.ac.uk/cgi-bin/nomenclature/searchgenes.pl?field=symbol&anchor=equals&symbol_search=Search&number=100&format=html&sortby=symbol&match=RNF28) | zinc ion binding;DNA binding;nucleic acid binding;transcription factor activity |  | 1.6 |
| 1438855_X_AT | gb:BB233088 /DB_XREF=gi:8912943 /DB_XREF=BB233088 |  | zinc ion binding;DNA binding;nucleic acid binding;transcription factor activity |  | 1.51 |
| 1438957_X_AT | CDP-diacylglycerol synthase (phosphatidate cytidylyltransferase) 2 | [CDS2](http://www.gene.ucl.ac.uk/cgi-bin/nomenclature/searchgenes.pl?field=symbol&anchor=equals&symbol_search=Search&number=100&format=html&sortby=symbol&match=CDS2) | zinc ion binding;DNA binding;nucleic acid binding;transcription regulator activity | [603549](http://www.ncbi.nlm.nih.gov/entrez/dispomim.cgi?id=603549) | -1.25 |
| 1439148_A_AT | phosphofructokinase, liver, B-type | [PFKL](http://www.gene.ucl.ac.uk/cgi-bin/nomenclature/searchgenes.pl?field=symbol&anchor=equals&symbol_search=Search&number=100&format=html&sortby=symbol&match=PFKL) | zinc ion binding;DNA binding;nucleic acid binding;transcription regulator activity | [171860](http://www.ncbi.nlm.nih.gov/entrez/dispomim.cgi?id=171860) | -1.36 |
| 1439159_AT | gb:BB021107 /DB_XREF=gi:15403370 /DB_XREF=BB021107 |  | zinc ion binding;DNA binding;nucleic acid binding;transcription regulator activity |  | 1.41 |
| 1439191_AT | Follistatin-like 1 | [FSTL1](http://www.gene.ucl.ac.uk/cgi-bin/nomenclature/searchgenes.pl?field=symbol&anchor=equals&symbol_search=Search&number=100&format=html&sortby=symbol&match=FSTL1) | zinc ion binding;DNA binding;nucleic acid binding;transcription regulator activity | [605547](http://www.ncbi.nlm.nih.gov/entrez/dispomim.cgi?id=605547) | -1.59 |
| 1439293_AT | RIKEN cDNA C130047D21 gene | [C130047D21RIK](http://www.gene.ucl.ac.uk/cgi-bin/nomenclature/searchgenes.pl?field=symbol&anchor=equals&symbol_search=Search&number=100&format=html&sortby=symbol&match=C130047D21RIK) | zinc ion binding;DNA binding;RNA binding;ubiquitin-protein ligase activity |  | 1.82 |
| 1439298_AT | gb:BM200180 /DB_XREF=gi:17753366 /DB_XREF=C0206H04 |  | zinc ion binding;guanyl-nucleotide exchange factor activity;protein transporter activity |  | -1.55 |
| 1439311_AT | RIKEN cDNA B830012L14 gene | [B830012L14RIK](http://www.gene.ucl.ac.uk/cgi-bin/nomenclature/searchgenes.pl?field=symbol&anchor=equals&symbol_search=Search&number=100&format=html&sortby=symbol&match=B830012L14RIK) | zinc ion binding;metal ion binding |  | -2.41 |
| 1439368_A_AT | solute carrier family 9 (sodium/hydrogen exchanger), isoform 3 regulator 2 | [SLC9A3R2](http://www.gene.ucl.ac.uk/cgi-bin/nomenclature/searchgenes.pl?field=symbol&anchor=equals&symbol_search=Search&number=100&format=html&sortby=symbol&match=SLC9A3R2) | zinc ion binding;metal ion binding;copper ion binding | [606553](http://www.ncbi.nlm.nih.gov/entrez/dispomim.cgi?id=606553) | -1.4 |
| 1439643_AT | Activin receptor IIA | [ACVR2](http://www.gene.ucl.ac.uk/cgi-bin/nomenclature/searchgenes.pl?field=symbol&anchor=equals&symbol_search=Search&number=100&format=html&sortby=symbol&match=ACVR2) | zinc ion binding;molecular_function unknown;nucleic acid binding;RNA binding | [102581](http://www.ncbi.nlm.nih.gov/entrez/dispomim.cgi?id=102581) | -1.4 |
| 1439650_AT | reticulon 4 | [RTN4](http://www.gene.ucl.ac.uk/cgi-bin/nomenclature/searchgenes.pl?field=symbol&anchor=equals&symbol_search=Search&number=100&format=html&sortby=symbol&match=RTN4) | zinc ion binding;molecular_function unknown;ubiquitin-protein ligase activity | [604475](http://www.ncbi.nlm.nih.gov/entrez/dispomim.cgi?id=604475) | -1.69 |
| 1439691_AT | DNA segment, Chr 5, ERATO Doi 579, expressed | [D5ERTD579E](http://www.gene.ucl.ac.uk/cgi-bin/nomenclature/searchgenes.pl?field=symbol&anchor=equals&symbol_search=Search&number=100&format=html&sortby=symbol&match=D5ERTD579E) | zinc ion binding;nucleic acid binding |  | -1.62 |
| 1439840_AT | RIKEN cDNA A430088C08 gene | [A430088C08RIK](http://www.gene.ucl.ac.uk/cgi-bin/nomenclature/searchgenes.pl?field=symbol&anchor=equals&symbol_search=Search&number=100&format=html&sortby=symbol&match=A430088C08RIK) | zinc ion binding;nucleic acid binding |  | -1.57 |
| 1440001_AT | RIKEN cDNA C130089L09 gene | [C130089L09RIK](http://www.gene.ucl.ac.uk/cgi-bin/nomenclature/searchgenes.pl?field=symbol&anchor=equals&symbol_search=Search&number=100&format=html&sortby=symbol&match=C130089L09RIK) | zinc ion binding;nucleic acid binding |  | -1.98 |
| 1440311_AT | Sorbin and SH3 domain containing 1 | [SORBS1](http://www.gene.ucl.ac.uk/cgi-bin/nomenclature/searchgenes.pl?field=symbol&anchor=equals&symbol_search=Search&number=100&format=html&sortby=symbol&match=SORBS1) | zinc ion binding;nucleic acid binding;DNA binding | [605264](http://www.ncbi.nlm.nih.gov/entrez/dispomim.cgi?id=605264) | 2.35 |
| 1440358_AT | RIKEN cDNA D530030K12 gene | [ARHGEF15](http://www.gene.ucl.ac.uk/cgi-bin/nomenclature/searchgenes.pl?field=symbol&anchor=equals&symbol_search=Search&number=100&format=html&sortby=symbol&match=ARHGEF15) | zinc ion binding;nucleic acid binding;DNA binding |  | -1.52 |
| 1440392_AT | RIKEN cDNA 9630026M06 gene | [9630026M06RIK](http://www.gene.ucl.ac.uk/cgi-bin/nomenclature/searchgenes.pl?field=symbol&anchor=equals&symbol_search=Search&number=100&format=html&sortby=symbol&match=9630026M06RIK) | zinc ion binding;nucleic acid binding;DNA binding;transcription regulator activity |  | 1.49 |
| 1440417_AT | gb:BG918834 /DB_XREF=gi:14299310 /DB_XREF=60281933 |  | zinc ion binding;protein carrier activity |  | 1.34 |
| 1440637_AT | gb:BG074656 /DB_XREF=gi:12557225 /DB_XREF=H3137G01 |  | zinc ion binding;stromelysin 1 activity;metallopeptidase activity;calcium ion binding;metalloendopeptidase activity;hydrolase activity;peptidase activity |  | 1.49 |
| 1440841_AT | Tyrosine 3-monooxygenase/tryptophan 5-monooxygenase activation protein, epsilon polypeptide | [YWHAE](http://www.gene.ucl.ac.uk/cgi-bin/nomenclature/searchgenes.pl?field=symbol&anchor=equals&symbol_search=Search&number=100&format=html&sortby=symbol&match=YWHAE) |  | [605066](http://www.ncbi.nlm.nih.gov/entrez/dispomim.cgi?id=605066) | -1.45 |
| 1440962_AT | solute carrier family 8 (sodium/calcium exchanger), member 3 | [SLC8A3](http://www.gene.ucl.ac.uk/cgi-bin/nomenclature/searchgenes.pl?field=symbol&anchor=equals&symbol_search=Search&number=100&format=html&sortby=symbol&match=SLC8A3) |  | [607991](http://www.ncbi.nlm.nih.gov/entrez/dispomim.cgi?id=607991) | 2.33 |
| 1440966_AT | Axotrophin | [AXOT](http://www.gene.ucl.ac.uk/cgi-bin/nomenclature/searchgenes.pl?field=symbol&anchor=equals&symbol_search=Search&number=100&format=html&sortby=symbol&match=AXOT) |  |  | -1.63 |
| 1441058_AT | inositol 1,4,5-trisphosphate 3-kinase B | [ITPKB](http://www.gene.ucl.ac.uk/cgi-bin/nomenclature/searchgenes.pl?field=symbol&anchor=equals&symbol_search=Search&number=100&format=html&sortby=symbol&match=ITPKB) |  | [147522](http://www.ncbi.nlm.nih.gov/entrez/dispomim.cgi?id=147522) | -1.52 |
| 1441228_AT | Gene model 1075, (NCBI) | [LOC381823](http://www.gene.ucl.ac.uk/cgi-bin/nomenclature/searchgenes.pl?field=symbol&anchor=equals&symbol_search=Search&number=100&format=html&sortby=symbol&match=LOC381823) |  |  | 1.79 |
| 1441338_AT | RIKEN cDNA 5930412G12 gene | [5930412G12RIK](http://www.gene.ucl.ac.uk/cgi-bin/nomenclature/searchgenes.pl?field=symbol&anchor=equals&symbol_search=Search&number=100&format=html&sortby=symbol&match=5930412G12RIK) |  |  | -1.41 |
| 1441437_AT | gb:BM119933 /DB_XREF=gi:17087959 /DB_XREF=L0932G04 |  |  |  | -1.67 |
| 1441465_AT | RIKEN cDNA A930039N10 gene | [A930039N10RIK](http://www.gene.ucl.ac.uk/cgi-bin/nomenclature/searchgenes.pl?field=symbol&anchor=equals&symbol_search=Search&number=100&format=html&sortby=symbol&match=A930039N10RIK) |  |  | -1.48 |
| 1441584_AT | Friend leukemia integration 1 | [FLI1](http://www.gene.ucl.ac.uk/cgi-bin/nomenclature/searchgenes.pl?field=symbol&anchor=equals&symbol_search=Search&number=100&format=html&sortby=symbol&match=FLI1) |  | [193067](http://www.ncbi.nlm.nih.gov/entrez/dispomim.cgi?id=193067) | -1.57 |
| 1441632_AT | RIKEN cDNA C130079B09 gene | [C130079B09RIK](http://www.gene.ucl.ac.uk/cgi-bin/nomenclature/searchgenes.pl?field=symbol&anchor=equals&symbol_search=Search&number=100&format=html&sortby=symbol&match=C130079B09RIK) |  |  | -1.59 |
| 1441799_AT | gb:AI098139 /DB_XREF=gi:3447664 /DB_XREF=ue27a06.x |  |  |  | 4.34 |
| 1442018_AT | expressed sequence AI426953 | [AI426953](http://www.gene.ucl.ac.uk/cgi-bin/nomenclature/searchgenes.pl?field=symbol&anchor=equals&symbol_search=Search&number=100&format=html&sortby=symbol&match=AI426953) |  |  | 1.75 |
| 1442111_AT | RIKEN cDNA D430033H22 gene | [D430033H22RIK](http://www.gene.ucl.ac.uk/cgi-bin/nomenclature/searchgenes.pl?field=symbol&anchor=equals&symbol_search=Search&number=100&format=html&sortby=symbol&match=D430033H22RIK) |  |  | -1.43 |
| 1442381_AT | gb:BG068971 /DB_XREF=gi:12551540 /DB_XREF=H3071C09 |  |  |  | 1.56 |
| 1442393_AT | gb:BB488200 /DB_XREF=gi:16441615 /DB_XREF=BB488200 |  |  |  | -1.81 |
| 1442427_AT | RIKEN cDNA 9630026M06 gene | [9630026M06RIK](http://www.gene.ucl.ac.uk/cgi-bin/nomenclature/searchgenes.pl?field=symbol&anchor=equals&symbol_search=Search&number=100&format=html&sortby=symbol&match=9630026M06RIK) |  |  | 1.75 |
| 1442538_AT | gb:AW742720 /DB_XREF=gi:7654516 /DB_XREF=up58h09.x |  |  |  | -1.42 |
| 1442618_AT | Lactate dehydrogenase 2, B chain | [LDH2](http://www.gene.ucl.ac.uk/cgi-bin/nomenclature/searchgenes.pl?field=symbol&anchor=equals&symbol_search=Search&number=100&format=html&sortby=symbol&match=LDH2) |  |  | -2.05 |
| 1442700_AT | phosphodiesterase 4B, cAMP specific | [PDE4B](http://www.gene.ucl.ac.uk/cgi-bin/nomenclature/searchgenes.pl?field=symbol&anchor=equals&symbol_search=Search&number=100&format=html&sortby=symbol&match=PDE4B) |  | [600127](http://www.ncbi.nlm.nih.gov/entrez/dispomim.cgi?id=600127) | 1.62 |
| 1442710_AT | gb:AV352204 /DB_XREF=gi:16397221 /DB_XREF=AV352204 |  |  |  | 2.12 |
| 1443027_AT | gb:BB667435 /DB_XREF=gi:16398884 /DB_XREF=BB667435 |  |  |  | 1.5 |
| 1443471_AT | zinc finger and BTB domain containing 20 | [ZBTB20](http://www.gene.ucl.ac.uk/cgi-bin/nomenclature/searchgenes.pl?field=symbol&anchor=equals&symbol_search=Search&number=100&format=html&sortby=symbol&match=ZBTB20) |  | [606025](http://www.ncbi.nlm.nih.gov/entrez/dispomim.cgi?id=606025) | 1.51 |
| 1443534_AT | gb:BM201095 /DB_XREF=gi:17754698 /DB_XREF=C0220C02 |  |  |  | -1.38 |
| 1443866_AT | RIKEN cDNA A930016D02 gene | [A930016D02RIK](http://www.gene.ucl.ac.uk/cgi-bin/nomenclature/searchgenes.pl?field=symbol&anchor=equals&symbol_search=Search&number=100&format=html&sortby=symbol&match=A930016D02RIK) |  |  | -1.41 |
| 1443983_AT | gb:BB218653 /DB_XREF=gi:8883606 /DB_XREF=BB218653 |  |  |  | 3.03 |
| 1444073_AT | avian musculoaponeurotic fibrosarcoma (v-maf) AS42 oncogene homolog | [MAF](http://www.gene.ucl.ac.uk/cgi-bin/nomenclature/searchgenes.pl?field=symbol&anchor=equals&symbol_search=Search&number=100&format=html&sortby=symbol&match=MAF) |  | [177075](http://www.ncbi.nlm.nih.gov/entrez/dispomim.cgi?id=177075) | 1.5 |
| 1444565_AT | expressed sequence BB166591 | [BB166591](http://www.gene.ucl.ac.uk/cgi-bin/nomenclature/searchgenes.pl?field=symbol&anchor=equals&symbol_search=Search&number=100&format=html&sortby=symbol&match=BB166591) |  |  | 1.44 |
| 1444722_AT | proteasome (prosome, macropain) activator subunit 4 | [PSME4](http://www.gene.ucl.ac.uk/cgi-bin/nomenclature/searchgenes.pl?field=symbol&anchor=equals&symbol_search=Search&number=100&format=html&sortby=symbol&match=PSME4) |  | [607705](http://www.ncbi.nlm.nih.gov/entrez/dispomim.cgi?id=607705) | 1.57 |
| 1445518_AT | gb:BG061923 /DB_XREF=gi:12531906 /DB_XREF=L0953F01 |  |  |  | -1.54 |
| 1445562_AT | gb:BB283832 /DB_XREF=gi:8984281 /DB_XREF=BB283832 |  |  |  | 2.46 |
| 1445689_AT | gb:AA717264 /DB_XREF=gi:2729538 /DB_XREF=vt02g03.r |  |  |  | -1.8 |
| 1445837_AT | Potassium voltage-gated channel, subfamily Q, member 5 | [KCNQ5](http://www.gene.ucl.ac.uk/cgi-bin/nomenclature/searchgenes.pl?field=symbol&anchor=equals&symbol_search=Search&number=100&format=html&sortby=symbol&match=KCNQ5) |  | [607357](http://www.ncbi.nlm.nih.gov/entrez/dispomim.cgi?id=607357) | 1.55 |
| 1445850_AT | RIKEN cDNA 9530009M10 gene | [9530009M10RIK](http://www.gene.ucl.ac.uk/cgi-bin/nomenclature/searchgenes.pl?field=symbol&anchor=equals&symbol_search=Search&number=100&format=html&sortby=symbol&match=9530009M10RIK) |  |  | 1.52 |
| 1445862_AT | cDNA sequence BC031575 | [BC031575](http://www.gene.ucl.ac.uk/cgi-bin/nomenclature/searchgenes.pl?field=symbol&anchor=equals&symbol_search=Search&number=100&format=html&sortby=symbol&match=BC031575) |  |  | -1.28 |
| 1446085_AT | gb:BB022048 /DB_XREF=gi:16257729 /DB_XREF=BB022048 |  |  |  | 1.87 |
| 1446212_AT | Potassium voltage-gated channel, subfamily Q, member 5 | [KCNQ5](http://www.gene.ucl.ac.uk/cgi-bin/nomenclature/searchgenes.pl?field=symbol&anchor=equals&symbol_search=Search&number=100&format=html&sortby=symbol&match=KCNQ5) |  | [607357](http://www.ncbi.nlm.nih.gov/entrez/dispomim.cgi?id=607357) | 1.6 |
| 1446326_AT | procollagen, type I, alpha 2 | [COL1A2](http://www.gene.ucl.ac.uk/cgi-bin/nomenclature/searchgenes.pl?field=symbol&anchor=equals&symbol_search=Search&number=100&format=html&sortby=symbol&match=COL1A2) |  | [120160](http://www.ncbi.nlm.nih.gov/entrez/dispomim.cgi?id=120160) | -2.09 |
| 1446537_AT | gb:BB162318 /DB_XREF=gi:16268683 /DB_XREF=BB162318 |  |  |  | 1.42 |
| 1446569_AT | gb:BB535350 /DB_XREF=gi:16446535 /DB_XREF=BB535350 |  |  |  | -2.3 |
| 1446968_AT | Phosphatidylinositol binding clathrin assembly protein | [PICALM](http://www.gene.ucl.ac.uk/cgi-bin/nomenclature/searchgenes.pl?field=symbol&anchor=equals&symbol_search=Search&number=100&format=html&sortby=symbol&match=PICALM) |  | [603025](http://www.ncbi.nlm.nih.gov/entrez/dispomim.cgi?id=603025) | 1.57 |
| 1447147_AT | gb:AI747732 /DB_XREF=gi:5125996 /DB_XREF=ul21e12.x |  |  |  | -1.5 |
| 1447448_S_AT | gb:C86813 /DB_XREF=gi:2918770 /DB_XREF=C86813 /CLO |  |  |  | 1.52 |
| 1447584_S_AT | gb:AI642973 /DB_XREF=gi:4721448 /DB_XREF=vd98a02.x |  |  |  | -1.52 |
| 1447849_S_AT | gb:AV323441 /DB_XREF=gi:6293358 /DB_XREF=AV323441 |  |  |  | 1.42 |
| 1447891_AT | gb:BI466416 /DB_XREF=gi:15279294 /DB_XREF=ie26c04. |  |  |  | 2.75 |
| 1448151_AT | ELAV (embryonic lethal, abnormal vision, Drosophila)-like 1 (Hu antigen R) | [ELAVL1](http://www.gene.ucl.ac.uk/cgi-bin/nomenclature/searchgenes.pl?field=symbol&anchor=equals&symbol_search=Search&number=100&format=html&sortby=symbol&match=ELAVL1) |  | [603466](http://www.ncbi.nlm.nih.gov/entrez/dispomim.cgi?id=603466) | -1.2 |
| 1448181_AT | Kruppel-like factor 15 | [KLF15](http://www.gene.ucl.ac.uk/cgi-bin/nomenclature/searchgenes.pl?field=symbol&anchor=equals&symbol_search=Search&number=100&format=html&sortby=symbol&match=KLF15) |  | [606465](http://www.ncbi.nlm.nih.gov/entrez/dispomim.cgi?id=606465) | 2.26 |
| 1448272_AT | B-cell translocation gene 2, anti-proliferative | [BTG2](http://www.gene.ucl.ac.uk/cgi-bin/nomenclature/searchgenes.pl?field=symbol&anchor=equals&symbol_search=Search&number=100&format=html&sortby=symbol&match=BTG2) |  | [601597](http://www.ncbi.nlm.nih.gov/entrez/dispomim.cgi?id=601597) | 2.05 |
| 1448306_AT | nuclear factor of kappa light chain gene enhancer in B-cells inhibitor, alpha | [NFKBIA](http://www.gene.ucl.ac.uk/cgi-bin/nomenclature/searchgenes.pl?field=symbol&anchor=equals&symbol_search=Search&number=100&format=html&sortby=symbol&match=NFKBIA) |  | [164008](http://www.ncbi.nlm.nih.gov/entrez/dispomim.cgi?id=164008) | 1.79 |
| 1448416_AT | matrix gamma-carboxyglutamate (gla) protein | [MGLAP](http://www.gene.ucl.ac.uk/cgi-bin/nomenclature/searchgenes.pl?field=symbol&anchor=equals&symbol_search=Search&number=100&format=html&sortby=symbol&match=MGLAP) |  |  | 1.29 |
| 1448538_A_AT | DNA segment, Chr 4, Wayne State University 53, expressed | [D4WSU53E](http://www.gene.ucl.ac.uk/cgi-bin/nomenclature/searchgenes.pl?field=symbol&anchor=equals&symbol_search=Search&number=100&format=html&sortby=symbol&match=D4WSU53E) |  |  | -1.41 |
| 1448747_AT | F-box only protein 32 | [FBXO32](http://www.gene.ucl.ac.uk/cgi-bin/nomenclature/searchgenes.pl?field=symbol&anchor=equals&symbol_search=Search&number=100&format=html&sortby=symbol&match=FBXO32) |  | [606604](http://www.ncbi.nlm.nih.gov/entrez/dispomim.cgi?id=606604) | 2.01 |
| 1448830_AT | dual specificity phosphatase 1 | [DUSP1](http://www.gene.ucl.ac.uk/cgi-bin/nomenclature/searchgenes.pl?field=symbol&anchor=equals&symbol_search=Search&number=100&format=html&sortby=symbol&match=DUSP1) |  | [600714](http://www.ncbi.nlm.nih.gov/entrez/dispomim.cgi?id=600714) | 1.9 |
| 1448844_AT | RIKEN cDNA 1810044O22 gene | [1810044O22RIK](http://www.gene.ucl.ac.uk/cgi-bin/nomenclature/searchgenes.pl?field=symbol&anchor=equals&symbol_search=Search&number=100&format=html&sortby=symbol&match=1810044O22RIK) |  |  | -1.21 |
| 1448862_AT | intercellular adhesion molecule 2 | [ICAM2](http://www.gene.ucl.ac.uk/cgi-bin/nomenclature/searchgenes.pl?field=symbol&anchor=equals&symbol_search=Search&number=100&format=html&sortby=symbol&match=ICAM2) |  | [146630](http://www.ncbi.nlm.nih.gov/entrez/dispomim.cgi?id=146630) | -1.37 |
| 1448940_AT | tripartite motif protein 21 | [TRIM21](http://www.gene.ucl.ac.uk/cgi-bin/nomenclature/searchgenes.pl?field=symbol&anchor=equals&symbol_search=Search&number=100&format=html&sortby=symbol&match=TRIM21) |  | [109092](http://www.ncbi.nlm.nih.gov/entrez/dispomim.cgi?id=109092) | -1.33 |
| 1449015_AT | resistin like alpha | [RETNLA](http://www.gene.ucl.ac.uk/cgi-bin/nomenclature/searchgenes.pl?field=symbol&anchor=equals&symbol_search=Search&number=100&format=html&sortby=symbol&match=RETNLA) |  |  | 1.99 |
| 1449135_AT | SRY-box containing gene 18 | [SOX18](http://www.gene.ucl.ac.uk/cgi-bin/nomenclature/searchgenes.pl?field=symbol&anchor=equals&symbol_search=Search&number=100&format=html&sortby=symbol&match=SOX18) |  | [601618](http://www.ncbi.nlm.nih.gov/entrez/dispomim.cgi?id=601618) | -1.58 |
| 1449190_A_AT | ectonucleoside triphosphate diphosphohydrolase 4 | [ENTPD4](http://www.gene.ucl.ac.uk/cgi-bin/nomenclature/searchgenes.pl?field=symbol&anchor=equals&symbol_search=Search&number=100&format=html&sortby=symbol&match=ENTPD4) |  | [607577](http://www.ncbi.nlm.nih.gov/entrez/dispomim.cgi?id=607577) | -1.21 |
| 1449229_A_AT | cyclin-dependent kinase-like 2 (CDC2-related kinase) | [CDKL2](http://www.gene.ucl.ac.uk/cgi-bin/nomenclature/searchgenes.pl?field=symbol&anchor=equals&symbol_search=Search&number=100&format=html&sortby=symbol&match=CDKL2) |  | [603442](http://www.ncbi.nlm.nih.gov/entrez/dispomim.cgi?id=603442) | 1.55 |
| 1449333_AT | splicing factor 3a, subunit 1 | [SF3A1](http://www.gene.ucl.ac.uk/cgi-bin/nomenclature/searchgenes.pl?field=symbol&anchor=equals&symbol_search=Search&number=100&format=html&sortby=symbol&match=SF3A1) |  | [605595](http://www.ncbi.nlm.nih.gov/entrez/dispomim.cgi?id=605595) | -1.35 |
| 1449379_AT | kinase insert domain protein receptor | [KDR](http://www.gene.ucl.ac.uk/cgi-bin/nomenclature/searchgenes.pl?field=symbol&anchor=equals&symbol_search=Search&number=100&format=html&sortby=symbol&match=KDR) |  | [191306](http://www.ncbi.nlm.nih.gov/entrez/dispomim.cgi?id=191306) | -1.35 |
| 1449519_AT | growth arrest and DNA-damage-inducible 45 alpha | [GADD45A](http://www.gene.ucl.ac.uk/cgi-bin/nomenclature/searchgenes.pl?field=symbol&anchor=equals&symbol_search=Search&number=100&format=html&sortby=symbol&match=GADD45A) |  | [126335](http://www.ncbi.nlm.nih.gov/entrez/dispomim.cgi?id=126335) | 1.97 |
| 1449731_S_AT | gb:AI462015 /DB_XREF=gi:4316045 /DB_XREF=ub69d10.x |  |  |  | 1.81 |
| 1449818_AT | ATP-binding cassette, sub-family B (MDR/TAP), member 4 | [ABCB4](http://www.gene.ucl.ac.uk/cgi-bin/nomenclature/searchgenes.pl?field=symbol&anchor=equals&symbol_search=Search&number=100&format=html&sortby=symbol&match=ABCB4) |  | [171060](http://www.ncbi.nlm.nih.gov/entrez/dispomim.cgi?id=171060) | 1.3 |
| 1449823_AT | dachshund 2 (Drosophila) | [DACH2](http://www.gene.ucl.ac.uk/cgi-bin/nomenclature/searchgenes.pl?field=symbol&anchor=equals&symbol_search=Search&number=100&format=html&sortby=symbol&match=DACH2) |  |  | -2.09 |
| 1449851_AT | period homolog 1 (Drosophila) | [PER1](http://www.gene.ucl.ac.uk/cgi-bin/nomenclature/searchgenes.pl?field=symbol&anchor=equals&symbol_search=Search&number=100&format=html&sortby=symbol&match=PER1) |  | [602260](http://www.ncbi.nlm.nih.gov/entrez/dispomim.cgi?id=602260) | 2.46 |
| 1449888_AT | endothelial PAS domain protein 1 | [EPAS1](http://www.gene.ucl.ac.uk/cgi-bin/nomenclature/searchgenes.pl?field=symbol&anchor=equals&symbol_search=Search&number=100&format=html&sortby=symbol&match=EPAS1) |  | [603349](http://www.ncbi.nlm.nih.gov/entrez/dispomim.cgi?id=603349) | -1.42 |
| 1449939_S_AT | delta-like 1 homolog (Drosophila) | [DLK1](http://www.gene.ucl.ac.uk/cgi-bin/nomenclature/searchgenes.pl?field=symbol&anchor=equals&symbol_search=Search&number=100&format=html&sortby=symbol&match=DLK1) |  | [176290](http://www.ncbi.nlm.nih.gov/entrez/dispomim.cgi?id=176290) | -2.81 |
| 1449984_AT | chemokine (C-X-C motif) ligand 2 | [CXCL2](http://www.gene.ucl.ac.uk/cgi-bin/nomenclature/searchgenes.pl?field=symbol&anchor=equals&symbol_search=Search&number=100&format=html&sortby=symbol&match=CXCL2) |  |  | 2.9 |
| 1450079_AT | Nik related kinase | [NRK](http://www.gene.ucl.ac.uk/cgi-bin/nomenclature/searchgenes.pl?field=symbol&anchor=equals&symbol_search=Search&number=100&format=html&sortby=symbol&match=NRK) |  |  | -1.86 |
| 1450089_A_AT | signal recognition particle receptor, B subunit | [SRPRB](http://www.gene.ucl.ac.uk/cgi-bin/nomenclature/searchgenes.pl?field=symbol&anchor=equals&symbol_search=Search&number=100&format=html&sortby=symbol&match=SRPRB) |  |  | -1.37 |
| 1450138_A_AT | serine (or cysteine) proteinase inhibitor, clade B, member 6a | [SERPINB6A](http://www.gene.ucl.ac.uk/cgi-bin/nomenclature/searchgenes.pl?field=symbol&anchor=equals&symbol_search=Search&number=100&format=html&sortby=symbol&match=SERPINB6A) |  |  | 1.33 |
| 1450311_AT | solute carrier family 8 (sodium/calcium exchanger), member 3 | [SLC8A3](http://www.gene.ucl.ac.uk/cgi-bin/nomenclature/searchgenes.pl?field=symbol&anchor=equals&symbol_search=Search&number=100&format=html&sortby=symbol&match=SLC8A3) |  | [607991](http://www.ncbi.nlm.nih.gov/entrez/dispomim.cgi?id=607991) | 1.71 |
| 1450405_AT | mitochondrial ribosomal protein L19 | [MRPL19](http://www.gene.ucl.ac.uk/cgi-bin/nomenclature/searchgenes.pl?field=symbol&anchor=equals&symbol_search=Search&number=100&format=html&sortby=symbol&match=MRPL19) |  |  | -1.21 |
| 1450414_AT | platelet derived growth factor, B polypeptide | [PDGFB](http://www.gene.ucl.ac.uk/cgi-bin/nomenclature/searchgenes.pl?field=symbol&anchor=equals&symbol_search=Search&number=100&format=html&sortby=symbol&match=PDGFB) |  | [190040](http://www.ncbi.nlm.nih.gov/entrez/dispomim.cgi?id=190040) | -1.36 |
| 1450644_AT | zinc finger protein 36, C3H type-like 1 | [ZFP36L1](http://www.gene.ucl.ac.uk/cgi-bin/nomenclature/searchgenes.pl?field=symbol&anchor=equals&symbol_search=Search&number=100&format=html&sortby=symbol&match=ZFP36L1) |  | [601064](http://www.ncbi.nlm.nih.gov/entrez/dispomim.cgi?id=601064) | 1.22 |
| 1450767_AT | neural precursor cell expressed, developmentally down-regulated gene 9 | [NEDD9](http://www.gene.ucl.ac.uk/cgi-bin/nomenclature/searchgenes.pl?field=symbol&anchor=equals&symbol_search=Search&number=100&format=html&sortby=symbol&match=NEDD9) |  | [602265](http://www.ncbi.nlm.nih.gov/entrez/dispomim.cgi?id=602265) | -1.39 |
| 1451038_AT | apelin | [APLN](http://www.gene.ucl.ac.uk/cgi-bin/nomenclature/searchgenes.pl?field=symbol&anchor=equals&symbol_search=Search&number=100&format=html&sortby=symbol&match=APLN) |  | [300297](http://www.ncbi.nlm.nih.gov/entrez/dispomim.cgi?id=300297) | -1.46 |
| 1451069_AT | proviral integration site 3 | [PIM3](http://www.gene.ucl.ac.uk/cgi-bin/nomenclature/searchgenes.pl?field=symbol&anchor=equals&symbol_search=Search&number=100&format=html&sortby=symbol&match=PIM3) |  |  | 1.5 |
| 1451091_AT | thioredoxin domain containing 5 | [TXNDC5](http://www.gene.ucl.ac.uk/cgi-bin/nomenclature/searchgenes.pl?field=symbol&anchor=equals&symbol_search=Search&number=100&format=html&sortby=symbol&match=TXNDC5) |  | [607289](http://www.ncbi.nlm.nih.gov/entrez/dispomim.cgi?id=607289) | -1.26 |
| 1451241_AT | laminin B1 subunit 1 | [LAMB1-1](http://www.gene.ucl.ac.uk/cgi-bin/nomenclature/searchgenes.pl?field=symbol&anchor=equals&symbol_search=Search&number=100&format=html&sortby=symbol&match=LAMB1-1) |  |  | -2.06 |
| 1451427_A_AT | EGF-like domain 7 | [EGFL7](http://www.gene.ucl.ac.uk/cgi-bin/nomenclature/searchgenes.pl?field=symbol&anchor=equals&symbol_search=Search&number=100&format=html&sortby=symbol&match=EGFL7) |  | [608582](http://www.ncbi.nlm.nih.gov/entrez/dispomim.cgi?id=608582) | -1.48 |
| 1451428_X_AT | EGF-like domain 7 | [EGFL7](http://www.gene.ucl.ac.uk/cgi-bin/nomenclature/searchgenes.pl?field=symbol&anchor=equals&symbol_search=Search&number=100&format=html&sortby=symbol&match=EGFL7) |  | [608582](http://www.ncbi.nlm.nih.gov/entrez/dispomim.cgi?id=608582) | -1.43 |
| 1451500_AT | Usher syndrome 1C binding protein 1 | [USHBP1](http://www.gene.ucl.ac.uk/cgi-bin/nomenclature/searchgenes.pl?field=symbol&anchor=equals&symbol_search=Search&number=100&format=html&sortby=symbol&match=USHBP1) |  |  | -1.46 |
| 1451506_AT | myocyte enhancer factor 2C | [MEF2C](http://www.gene.ucl.ac.uk/cgi-bin/nomenclature/searchgenes.pl?field=symbol&anchor=equals&symbol_search=Search&number=100&format=html&sortby=symbol&match=MEF2C) |  | [600662](http://www.ncbi.nlm.nih.gov/entrez/dispomim.cgi?id=600662) | 1.28 |
| 1451553_AT | ADP-ribosyltransferase 5 | [ART5](http://www.gene.ucl.ac.uk/cgi-bin/nomenclature/searchgenes.pl?field=symbol&anchor=equals&symbol_search=Search&number=100&format=html&sortby=symbol&match=ART5) |  |  | 1.32 |
| 1451612_AT | gb:BC027262.1 /DB_XREF=gi:20072672 /FEA=FLmRNA /CN |  |  |  | 15.11 |
| 1451715_AT | v-maf musculoaponeurotic fibrosarcoma oncogene family, protein B (avian) | [MAFB](http://www.gene.ucl.ac.uk/cgi-bin/nomenclature/searchgenes.pl?field=symbol&anchor=equals&symbol_search=Search&number=100&format=html&sortby=symbol&match=MAFB) |  | [608968](http://www.ncbi.nlm.nih.gov/entrez/dispomim.cgi?id=608968) | 1.93 |
| 1452049_AT | ribosomal protein L7-like 1 | [RPL7L1](http://www.gene.ucl.ac.uk/cgi-bin/nomenclature/searchgenes.pl?field=symbol&anchor=equals&symbol_search=Search&number=100&format=html&sortby=symbol&match=RPL7L1) |  |  | -1.33 |
| 1452057_AT | ARP1 actin-related protein 1 homolog B (yeast) | [ACTR1B](http://www.gene.ucl.ac.uk/cgi-bin/nomenclature/searchgenes.pl?field=symbol&anchor=equals&symbol_search=Search&number=100&format=html&sortby=symbol&match=ACTR1B) |  | [605144](http://www.ncbi.nlm.nih.gov/entrez/dispomim.cgi?id=605144) | -1.25 |
| 1452072_AT | myc target 1 | [MYCT1](http://www.gene.ucl.ac.uk/cgi-bin/nomenclature/searchgenes.pl?field=symbol&anchor=equals&symbol_search=Search&number=100&format=html&sortby=symbol&match=MYCT1) |  |  | -1.55 |
| 1452124_AT | RIKEN cDNA 2900054D09 gene | [2900054D09RIK](http://www.gene.ucl.ac.uk/cgi-bin/nomenclature/searchgenes.pl?field=symbol&anchor=equals&symbol_search=Search&number=100&format=html&sortby=symbol&match=2900054D09RIK) |  |  | 1.35 |
| 1452161_AT | TCDD-inducible poly(ADP-ribose) polymerase | [TIPARP](http://www.gene.ucl.ac.uk/cgi-bin/nomenclature/searchgenes.pl?field=symbol&anchor=equals&symbol_search=Search&number=100&format=html&sortby=symbol&match=TIPARP) |  |  | 1.43 |
| 1452187_AT | RNA binding motif protein 5 | [RBM5](http://www.gene.ucl.ac.uk/cgi-bin/nomenclature/searchgenes.pl?field=symbol&anchor=equals&symbol_search=Search&number=100&format=html&sortby=symbol&match=RBM5) |  | [606884](http://www.ncbi.nlm.nih.gov/entrez/dispomim.cgi?id=606884) | -1.33 |
| 1452239_AT | gene trap ROSA 26, Philippe Soriano | [GT(ROSA)26SOR](http://www.gene.ucl.ac.uk/cgi-bin/nomenclature/searchgenes.pl?field=symbol&anchor=equals&symbol_search=Search&number=100&format=html&sortby=symbol&match=GT(ROSA)26SOR) |  |  | -1.63 |
| 1452299_AT | WW domain containing E3 ubiquitin protein ligase 1 | [WWP1](http://www.gene.ucl.ac.uk/cgi-bin/nomenclature/searchgenes.pl?field=symbol&anchor=equals&symbol_search=Search&number=100&format=html&sortby=symbol&match=WWP1) |  | [602307](http://www.ncbi.nlm.nih.gov/entrez/dispomim.cgi?id=602307) | 1.28 |
| 1452349_X_AT | interferon activated gene 205 | [IFI205](http://www.gene.ucl.ac.uk/cgi-bin/nomenclature/searchgenes.pl?field=symbol&anchor=equals&symbol_search=Search&number=100&format=html&sortby=symbol&match=IFI205) |  |  | 1.66 |
| 1452418_AT | RIKEN cDNA 1200016E24 gene | [1200016E24RIK](http://www.gene.ucl.ac.uk/cgi-bin/nomenclature/searchgenes.pl?field=symbol&anchor=equals&symbol_search=Search&number=100&format=html&sortby=symbol&match=1200016E24RIK) |  |  | 1.44 |
| 1452430_S_AT | splicing factor, arginine/serine-rich 1 (ASF/SF2) | [SFRS1](http://www.gene.ucl.ac.uk/cgi-bin/nomenclature/searchgenes.pl?field=symbol&anchor=equals&symbol_search=Search&number=100&format=html&sortby=symbol&match=SFRS1) |  | [600812](http://www.ncbi.nlm.nih.gov/entrez/dispomim.cgi?id=600812) | -1.23 |
| 1452519_A_AT | zinc finger protein 36 | [ZFP36](http://www.gene.ucl.ac.uk/cgi-bin/nomenclature/searchgenes.pl?field=symbol&anchor=equals&symbol_search=Search&number=100&format=html&sortby=symbol&match=ZFP36) |  | [190700](http://www.ncbi.nlm.nih.gov/entrez/dispomim.cgi?id=190700) | 3 |
| 1452670_AT | Myosin, light polypeptide 9, regulatory | [MYL9](http://www.gene.ucl.ac.uk/cgi-bin/nomenclature/searchgenes.pl?field=symbol&anchor=equals&symbol_search=Search&number=100&format=html&sortby=symbol&match=MYL9) |  |  | -1.95 |
| 1452730_AT | RIKEN cDNA 1110033J19 gene | [1110033J19RIK](http://www.gene.ucl.ac.uk/cgi-bin/nomenclature/searchgenes.pl?field=symbol&anchor=equals&symbol_search=Search&number=100&format=html&sortby=symbol&match=1110033J19RIK) |  |  | -1.65 |
| 1452899_AT | RNA imprinted and accumulated in nucleus | [RIAN](http://www.gene.ucl.ac.uk/cgi-bin/nomenclature/searchgenes.pl?field=symbol&anchor=equals&symbol_search=Search&number=100&format=html&sortby=symbol&match=RIAN) |  |  | -2.52 |
| 1452976_A_AT | solute carrier family 9 (sodium/hydrogen exchanger), isoform 3 regulator 2 | [SLC9A3R2](http://www.gene.ucl.ac.uk/cgi-bin/nomenclature/searchgenes.pl?field=symbol&anchor=equals&symbol_search=Search&number=100&format=html&sortby=symbol&match=SLC9A3R2) |  | [606553](http://www.ncbi.nlm.nih.gov/entrez/dispomim.cgi?id=606553) | -1.43 |
| 1452991_AT | RIKEN cDNA 2810013C04 gene | [2810013C04RIK](http://www.gene.ucl.ac.uk/cgi-bin/nomenclature/searchgenes.pl?field=symbol&anchor=equals&symbol_search=Search&number=100&format=html&sortby=symbol&match=2810013C04RIK) |  |  | -1.8 |
| 1453014_A_AT | SEC31-like 1 (S. cerevisiae) | [SEC31L1](http://www.gene.ucl.ac.uk/cgi-bin/nomenclature/searchgenes.pl?field=symbol&anchor=equals&symbol_search=Search&number=100&format=html&sortby=symbol&match=SEC31L1) |  |  | -1.25 |
| 1453238_S_AT | RIKEN cDNA 1200016E24 gene | [1200016E24RIK](http://www.gene.ucl.ac.uk/cgi-bin/nomenclature/searchgenes.pl?field=symbol&anchor=equals&symbol_search=Search&number=100&format=html&sortby=symbol&match=1200016E24RIK) |  |  | 1.49 |
| 1453424_AT | FYVE and coiled-coil domain containing 1 | [FYCO1](http://www.gene.ucl.ac.uk/cgi-bin/nomenclature/searchgenes.pl?field=symbol&anchor=equals&symbol_search=Search&number=100&format=html&sortby=symbol&match=FYCO1) |  | [607182](http://www.ncbi.nlm.nih.gov/entrez/dispomim.cgi?id=607182) | -1.32 |
| 1453530_AT | RIKEN cDNA 4833444C15 gene | [4833444C15RIK](http://www.gene.ucl.ac.uk/cgi-bin/nomenclature/searchgenes.pl?field=symbol&anchor=equals&symbol_search=Search&number=100&format=html&sortby=symbol&match=4833444C15RIK) |  |  | -1.7 |
| 1453744_A_AT | RIKEN cDNA 5530600A18 gene | [5530600A18RIK](http://www.gene.ucl.ac.uk/cgi-bin/nomenclature/searchgenes.pl?field=symbol&anchor=equals&symbol_search=Search&number=100&format=html&sortby=symbol&match=5530600A18RIK) |  |  | -1.27 |
| 1453851_A_AT | growth arrest and DNA-damage-inducible 45 gamma | [GADD45G](http://www.gene.ucl.ac.uk/cgi-bin/nomenclature/searchgenes.pl?field=symbol&anchor=equals&symbol_search=Search&number=100&format=html&sortby=symbol&match=GADD45G) |  | [604949](http://www.ncbi.nlm.nih.gov/entrez/dispomim.cgi?id=604949) | 3.57 |
| 1454034_A_AT | ubiquitin specific protease 21 | [USP21](http://www.gene.ucl.ac.uk/cgi-bin/nomenclature/searchgenes.pl?field=symbol&anchor=equals&symbol_search=Search&number=100&format=html&sortby=symbol&match=USP21) |  | [604729](http://www.ncbi.nlm.nih.gov/entrez/dispomim.cgi?id=604729) | -1.37 |
| 1454141_AT | RIKEN cDNA 5730416O20 gene | [5730416O20RIK](http://www.gene.ucl.ac.uk/cgi-bin/nomenclature/searchgenes.pl?field=symbol&anchor=equals&symbol_search=Search&number=100&format=html&sortby=symbol&match=5730416O20RIK) |  |  | 2.41 |
| 1454551_AT | RIKEN cDNA 9530034D02 gene | [9530034D02RIK](http://www.gene.ucl.ac.uk/cgi-bin/nomenclature/searchgenes.pl?field=symbol&anchor=equals&symbol_search=Search&number=100&format=html&sortby=symbol&match=9530034D02RIK) |  |  | 1.52 |
| 1454617_AT | arrestin domain containing 3 | [ARRDC3](http://www.gene.ucl.ac.uk/cgi-bin/nomenclature/searchgenes.pl?field=symbol&anchor=equals&symbol_search=Search&number=100&format=html&sortby=symbol&match=ARRDC3) |  |  | 1.56 |
| 1454708_AT | actin-binding LIM protein 1 | [ABLIM1](http://www.gene.ucl.ac.uk/cgi-bin/nomenclature/searchgenes.pl?field=symbol&anchor=equals&symbol_search=Search&number=100&format=html&sortby=symbol&match=ABLIM1) |  | [602330](http://www.ncbi.nlm.nih.gov/entrez/dispomim.cgi?id=602330) | 1.52 |
| 1455056_AT | LIM domain only 7 | [LMO7](http://www.gene.ucl.ac.uk/cgi-bin/nomenclature/searchgenes.pl?field=symbol&anchor=equals&symbol_search=Search&number=100&format=html&sortby=symbol&match=LMO7) |  | [604362](http://www.ncbi.nlm.nih.gov/entrez/dispomim.cgi?id=604362) | 1.25 |
| 1455160_AT | RIKEN cDNA 2610203C20 gene | [2610203C20RIK](http://www.gene.ucl.ac.uk/cgi-bin/nomenclature/searchgenes.pl?field=symbol&anchor=equals&symbol_search=Search&number=100&format=html&sortby=symbol&match=2610203C20RIK) |  |  | -1.56 |
| 1455214_AT | microphthalmia-associated transcription factor | [MITF](http://www.gene.ucl.ac.uk/cgi-bin/nomenclature/searchgenes.pl?field=symbol&anchor=equals&symbol_search=Search&number=100&format=html&sortby=symbol&match=MITF) |  | [156845](http://www.ncbi.nlm.nih.gov/entrez/dispomim.cgi?id=156845) | 1.23 |
| 1455334_AT | gb:BG067299 /DB_XREF=gi:12549868 /DB_XREF=H3052G10 |  |  |  | -1.44 |
| 1455377_AT | RIKEN cDNA 4921517B04 gene | [4921517B04RIK](http://www.gene.ucl.ac.uk/cgi-bin/nomenclature/searchgenes.pl?field=symbol&anchor=equals&symbol_search=Search&number=100&format=html&sortby=symbol&match=4921517B04RIK) |  |  | 1.23 |
| 1455487_AT | RIKEN cDNA 2600014M03 gene | [2600014M03RIK](http://www.gene.ucl.ac.uk/cgi-bin/nomenclature/searchgenes.pl?field=symbol&anchor=equals&symbol_search=Search&number=100&format=html&sortby=symbol&match=2600014M03RIK) |  |  | -1.26 |
| 1455522_AT | RIKEN cDNA D530030K12 gene | [ARHGEF15](http://www.gene.ucl.ac.uk/cgi-bin/nomenclature/searchgenes.pl?field=symbol&anchor=equals&symbol_search=Search&number=100&format=html&sortby=symbol&match=ARHGEF15) |  |  | -1.48 |
| 1455725_A_AT | H3 histone, family 3B | [H3F3B](http://www.gene.ucl.ac.uk/cgi-bin/nomenclature/searchgenes.pl?field=symbol&anchor=equals&symbol_search=Search&number=100&format=html&sortby=symbol&match=H3F3B) |  | [601058](http://www.ncbi.nlm.nih.gov/entrez/dispomim.cgi?id=601058) | 1.2 |
| 1455773_AT | gb:BG797225 /DB_XREF=gi:14143794 /DB_XREF=ic12d08. |  |  |  | 1.39 |
| 1455967_AT | Sorbin and SH3 domain containing 1 | [SORBS1](http://www.gene.ucl.ac.uk/cgi-bin/nomenclature/searchgenes.pl?field=symbol&anchor=equals&symbol_search=Search&number=100&format=html&sortby=symbol&match=SORBS1) |  | [605264](http://www.ncbi.nlm.nih.gov/entrez/dispomim.cgi?id=605264) | 2.86 |
| 1456046_AT | complement component 1, q subcomponent, receptor 1 | [C1QR1](http://www.gene.ucl.ac.uk/cgi-bin/nomenclature/searchgenes.pl?field=symbol&anchor=equals&symbol_search=Search&number=100&format=html&sortby=symbol&match=C1QR1) |  | [120577](http://www.ncbi.nlm.nih.gov/entrez/dispomim.cgi?id=120577) | -1.44 |
| 1456060_AT | avian musculoaponeurotic fibrosarcoma (v-maf) AS42 oncogene homolog | [MAF](http://www.gene.ucl.ac.uk/cgi-bin/nomenclature/searchgenes.pl?field=symbol&anchor=equals&symbol_search=Search&number=100&format=html&sortby=symbol&match=MAF) |  | [177075](http://www.ncbi.nlm.nih.gov/entrez/dispomim.cgi?id=177075) | 1.25 |
| 1456084_X_AT | fibromodulin | [FMOD](http://www.gene.ucl.ac.uk/cgi-bin/nomenclature/searchgenes.pl?field=symbol&anchor=equals&symbol_search=Search&number=100&format=html&sortby=symbol&match=FMOD) |  | [600245](http://www.ncbi.nlm.nih.gov/entrez/dispomim.cgi?id=600245) | -1.72 |
| 1456341_A_AT | basic transcription element binding protein 1 | [BTEB1](http://www.gene.ucl.ac.uk/cgi-bin/nomenclature/searchgenes.pl?field=symbol&anchor=equals&symbol_search=Search&number=100&format=html&sortby=symbol&match=BTEB1) |  | [602902](http://www.ncbi.nlm.nih.gov/entrez/dispomim.cgi?id=602902) | 1.68 |
| 1456500_AT | hypothetical protein 4632417K02 | [APH1B](http://www.gene.ucl.ac.uk/cgi-bin/nomenclature/searchgenes.pl?field=symbol&anchor=equals&symbol_search=Search&number=100&format=html&sortby=symbol&match=APH1B) |  |  | 1.33 |
| 1456530_X_AT | elongation of very long chain fatty acids (FEN1/Elo2, SUR4/Elo3, yeast)-like 1 | [ELOVL1](http://www.gene.ucl.ac.uk/cgi-bin/nomenclature/searchgenes.pl?field=symbol&anchor=equals&symbol_search=Search&number=100&format=html&sortby=symbol&match=ELOVL1) |  |  | -1.26 |
| 1456547_AT | gb:BM119402 /DB_XREF=gi:17087428 /DB_XREF=L0924H05 |  |  |  | -1.65 |
| 1456762_AT | gb:BM237714 /DB_XREF=gi:17872984 /DB_XREF=K0505H05 |  |  |  | 1.68 |
| 1456880_AT | gb:BE956180 /DB_XREF=gi:10600466 /DB_XREF=UI-M-BH4 |  |  |  | -1.35 |
| 1456918_AT | RIKEN cDNA 9430025M21 gene | [9430025M21RIK](http://www.gene.ucl.ac.uk/cgi-bin/nomenclature/searchgenes.pl?field=symbol&anchor=equals&symbol_search=Search&number=100&format=html&sortby=symbol&match=9430025M21RIK) |  |  | 1.45 |
| 1456955_AT | RIKEN cDNA 5830417C01 gene | [5830417C01RIK](http://www.gene.ucl.ac.uk/cgi-bin/nomenclature/searchgenes.pl?field=symbol&anchor=equals&symbol_search=Search&number=100&format=html&sortby=symbol&match=5830417C01RIK) |  |  | -1.79 |
| 1457030_AT | miRNA containing gene | [MIRG](http://www.gene.ucl.ac.uk/cgi-bin/nomenclature/searchgenes.pl?field=symbol&anchor=equals&symbol_search=Search&number=100&format=html&sortby=symbol&match=MIRG) |  |  | -2.51 |
| 1457035_AT | Expressed sequence AI607873 | [AI607873](http://www.gene.ucl.ac.uk/cgi-bin/nomenclature/searchgenes.pl?field=symbol&anchor=equals&symbol_search=Search&number=100&format=html&sortby=symbol&match=AI607873) |  |  | 2.13 |
| 1457094_AT | gb:AV229195 /DB_XREF=gi:16385629 /DB_XREF=AV229195 |  |  |  | -1.24 |
| 1457172_AT | hypothetical protein D130071N09 | [D130071N09](http://www.gene.ucl.ac.uk/cgi-bin/nomenclature/searchgenes.pl?field=symbol&anchor=equals&symbol_search=Search&number=100&format=html&sortby=symbol&match=D130071N09) |  |  | -1.55 |
| 1457188_AT | Rho guanine nucleotide exchange factor (GEF) 11 | [ARHGEF11](http://www.gene.ucl.ac.uk/cgi-bin/nomenclature/searchgenes.pl?field=symbol&anchor=equals&symbol_search=Search&number=100&format=html&sortby=symbol&match=ARHGEF11) |  | [605708](http://www.ncbi.nlm.nih.gov/entrez/dispomim.cgi?id=605708) | -1.4 |
| 1457198_AT | gb:AV291009 /DB_XREF=gi:16392354 /DB_XREF=AV291009 |  |  |  | -1.86 |
| 1457898_AT | gb:BM243944 /DB_XREF=gi:17879214 /DB_XREF=K0704C08 |  |  |  | 1.47 |
| 1458089_AT | RIKEN cDNA 6030422H21 gene | [FKBP5](http://www.gene.ucl.ac.uk/cgi-bin/nomenclature/searchgenes.pl?field=symbol&anchor=equals&symbol_search=Search&number=100&format=html&sortby=symbol&match=FKBP5) |  | [602623](http://www.ncbi.nlm.nih.gov/entrez/dispomim.cgi?id=602623) | 1.96 |
| 1458099_AT | gb:BB291417 /DB_XREF=gi:16401463 /DB_XREF=BB291417 |  |  |  | 1.36 |
| 1458369_AT | Ataxia, cerebellar, Cayman type homolog (human) | [ATCAY](http://www.gene.ucl.ac.uk/cgi-bin/nomenclature/searchgenes.pl?field=symbol&anchor=equals&symbol_search=Search&number=100&format=html&sortby=symbol&match=ATCAY) |  | [608179](http://www.ncbi.nlm.nih.gov/entrez/dispomim.cgi?id=608179) | 1.64 |
| 1458426_AT | gb:BM941075 /DB_XREF=gi:19400227 /DB_XREF=UI-M-BZ1 |  |  |  | -1.56 |
| 1458469_AT | Casitas B-lineage lymphoma b | [CBLB](http://www.gene.ucl.ac.uk/cgi-bin/nomenclature/searchgenes.pl?field=symbol&anchor=equals&symbol_search=Search&number=100&format=html&sortby=symbol&match=CBLB) |  | [604491](http://www.ncbi.nlm.nih.gov/entrez/dispomim.cgi?id=604491) | 1.59 |
| 1458492_X_AT | neurotrimin | [HNT](http://www.gene.ucl.ac.uk/cgi-bin/nomenclature/searchgenes.pl?field=symbol&anchor=equals&symbol_search=Search&number=100&format=html&sortby=symbol&match=HNT) |  | [607938](http://www.ncbi.nlm.nih.gov/entrez/dispomim.cgi?id=607938) | 1.62 |
| 1459005_AT | RIKEN cDNA D030051N19 gene | [D030051N19RIK](http://www.gene.ucl.ac.uk/cgi-bin/nomenclature/searchgenes.pl?field=symbol&anchor=equals&symbol_search=Search&number=100&format=html&sortby=symbol&match=D030051N19RIK) |  |  | 1.48 |
| 1459187_AT | gb:BG075096 /DB_XREF=gi:12557665 /DB_XREF=H3143C04 |  |  |  | -1.8 |
| 1459238_AT | gb:AV319481 /DB_XREF=gi:16394866 /DB_XREF=AV319481 |  |  |  | 1.94 |
| 1459253_AT | gb:AW556597 /DB_XREF=gi:7202026 /DB_XREF=L0270F09- |  |  |  | 1.75 |
| 1459601_AT | SNF1-like kinase | [SNF1LK](http://www.gene.ucl.ac.uk/cgi-bin/nomenclature/searchgenes.pl?field=symbol&anchor=equals&symbol_search=Search&number=100&format=html&sortby=symbol&match=SNF1LK) |  | [605705](http://www.ncbi.nlm.nih.gov/entrez/dispomim.cgi?id=605705) | 2.69 |
| 1459718_X_AT | gb:C86813 /DB_XREF=gi:2918770 /DB_XREF=C86813 /CLO |  |  |  | 1.91 |
| 1460330_AT | annexin A3 | [ANXA3](http://www.gene.ucl.ac.uk/cgi-bin/nomenclature/searchgenes.pl?field=symbol&anchor=equals&symbol_search=Search&number=100&format=html&sortby=symbol&match=ANXA3) |  | [106490](http://www.ncbi.nlm.nih.gov/entrez/dispomim.cgi?id=106490) | -1.31 |
| 1460342_S_AT | expressed sequence AA536749 | [AA536749](http://www.gene.ucl.ac.uk/cgi-bin/nomenclature/searchgenes.pl?field=symbol&anchor=equals&symbol_search=Search&number=100&format=html&sortby=symbol&match=AA536749) |  |  | -1.22 |
| 1460356_AT | endothelial cell-specific adhesion molecule | [ESAM1](http://www.gene.ucl.ac.uk/cgi-bin/nomenclature/searchgenes.pl?field=symbol&anchor=equals&symbol_search=Search&number=100&format=html&sortby=symbol&match=ESAM1) |  |  | -1.26 |
| 1460412_AT | RIKEN cDNA 1600015H20 gene | [1600015H20RIK](http://www.gene.ucl.ac.uk/cgi-bin/nomenclature/searchgenes.pl?field=symbol&anchor=equals&symbol_search=Search&number=100&format=html&sortby=symbol&match=1600015H20RIK) |  |  | 2.62 |
| 1460557_AT | suppressor of var1, 3-like 1 (S. cerevisiae) | [SUPV3L1](http://www.gene.ucl.ac.uk/cgi-bin/nomenclature/searchgenes.pl?field=symbol&anchor=equals&symbol_search=Search&number=100&format=html&sortby=symbol&match=SUPV3L1) |  | [605122](http://www.ncbi.nlm.nih.gov/entrez/dispomim.cgi?id=605122) | -1.29 |
| 1460740_AT | clathrin, light polypeptide (Lcb) | [CLTB](http://www.gene.ucl.ac.uk/cgi-bin/nomenclature/searchgenes.pl?field=symbol&anchor=equals&symbol_search=Search&number=100&format=html&sortby=symbol&match=CLTB) |  | [118970](http://www.ncbi.nlm.nih.gov/entrez/dispomim.cgi?id=118970) | 1.21 |
