# Supplementary material for: Corticortophin releasing factor 2 receptor agonist treatment significantly slows disease progression in mdx mice
Source: BMC Med. 2007 Jul 12;5:18. doi: 10.1186/1741-7015-5-18 (PMC1936998; doi:10.1186/1741-7015-5-18)
Supplement: Additional file 4 — C57BL10 vehicle versus mdx vehicle. All differential genes showed statistically significant differences in expression (NLogP = 4.0). [file 1741-7015-5-18-S4.doc]

### Additional file 2 – C57BL10 vehicle versus mdx vehicle. All differential genes demonstrated statistically significant differences in expression (NLogP=4.0).

| **AffyID** | **NetAffx Title** | **Acronym** | **Functional Classification** | **OMIM** | **C57BL10 vehicle verus MDX Vehicle Fold Change** |
| --- | --- | --- | --- | --- | --- |
| 1415678_AT | protein phosphatase 1A, magnesium dependent, alpha isoform | [PPM1A](http://www.gene.ucl.ac.uk/cgi-bin/nomenclature/searchgenes.pl?field=symbol&anchor=equals&symbol_search=Search&number=100&format=html&sortby=symbol&match=PPM1A) | (alpha-N-acetylneuraminyl-2,3-beta-galactosyl-1,3)-N-acetyl-galactosaminide 6-alpha-sialyltransferase activity;transferase activity, transferring glycosyl groups;sialyltransferase activity;transferase activity | [606108](http://www.ncbi.nlm.nih.gov/entrez/dispomim.cgi?id=606108) | **1.5** |
| 1415691_AT | discs, large homolog 1 (Drosophila) | [DLGH1](http://www.gene.ucl.ac.uk/cgi-bin/nomenclature/searchgenes.pl?field=symbol&anchor=equals&symbol_search=Search&number=100&format=html&sortby=symbol&match=DLGH1) | (alpha-N-acetylneuraminyl-2,3-beta-galactosyl-1,3)-N-acetyl-galactosaminide 6-alpha-sialyltransferase activity;transferase activity, transferring glycosyl groups;transferase activity;sialyltransferase activity |  | **1.39** |
| 1415692_S_AT | calnexin | [CANX](http://www.gene.ucl.ac.uk/cgi-bin/nomenclature/searchgenes.pl?field=symbol&anchor=equals&symbol_search=Search&number=100&format=html&sortby=symbol&match=CANX) | [pyruvate dehydrogenase (lipoamide)] kinase activity;kinase activity;protein-histidine kinase activity;ATP binding;transferase activity | [114217](http://www.ncbi.nlm.nih.gov/entrez/dispomim.cgi?id=114217) | **-1.29** |
| 1415693_AT | Der1-like domain family, member 1 | [DERL1](http://www.gene.ucl.ac.uk/cgi-bin/nomenclature/searchgenes.pl?field=symbol&anchor=equals&symbol_search=Search&number=100&format=html&sortby=symbol&match=DERL1) | 1,4-alpha-glucan branching enzyme activity;transferase activity, transferring glycosyl groups;alpha-amylase activity;hydrolase activity, hydrolyzing O-glycosyl compounds;transferase activity | [608813](http://www.ncbi.nlm.nih.gov/entrez/dispomim.cgi?id=608813) | **-1.2** |
| 1415694_AT | gb:AK004541.1 /DB_XREF=gi:12835777 /GEN=Wars /FEA= |  | 1-alkyl-2-acetylglycerophosphocholine esterase activity;catalytic activity;hydrolase activity |  | **1.31** |
| 1415711_AT | RIKEN cDNA D130059B05 gene | [ARFGEF1](http://www.gene.ucl.ac.uk/cgi-bin/nomenclature/searchgenes.pl?field=symbol&anchor=equals&symbol_search=Search&number=100&format=html&sortby=symbol&match=ARFGEF1) | 3',5'-cyclic-nucleotide phosphodiesterase activity;cAMP-specific phosphodiesterase activity;magnesium ion binding;manganese ion binding;signal transducer activity;hydrolase activity | [604141](http://www.ncbi.nlm.nih.gov/entrez/dispomim.cgi?id=604141) | **1.53** |
| 1415718_AT | RIKEN cDNA 2310079P12 gene | [2310079P12RIK](http://www.gene.ucl.ac.uk/cgi-bin/nomenclature/searchgenes.pl?field=symbol&anchor=equals&symbol_search=Search&number=100&format=html&sortby=symbol&match=2310079P12RIK) | 3-hydroxyacyl-CoA dehydrogenase activity;oxidoreductase activity |  | **1.33** |
| 1415729_AT | 3-phosphoinositide dependent protein kinase-1 | [PDPK1](http://www.gene.ucl.ac.uk/cgi-bin/nomenclature/searchgenes.pl?field=symbol&anchor=equals&symbol_search=Search&number=100&format=html&sortby=symbol&match=PDPK1) | 3-hydroxyacyl-CoA dehydrogenase activity;oxidoreductase activity | [605213](http://www.ncbi.nlm.nih.gov/entrez/dispomim.cgi?id=605213) | **-1.24** |
| 1415732_AT | HLA-B associated transcript 5 | [BAT5](http://www.gene.ucl.ac.uk/cgi-bin/nomenclature/searchgenes.pl?field=symbol&anchor=equals&symbol_search=Search&number=100&format=html&sortby=symbol&match=BAT5) | 3-hydroxyacyl-CoA dehydrogenase activity;oxidoreductase activity | [142620](http://www.ncbi.nlm.nih.gov/entrez/dispomim.cgi?id=142620) | **1.33** |
| 1415735_AT | damage specific DNA binding protein 1 | [DDB1](http://www.gene.ucl.ac.uk/cgi-bin/nomenclature/searchgenes.pl?field=symbol&anchor=equals&symbol_search=Search&number=100&format=html&sortby=symbol&match=DDB1) | 3-oxoacyl-[acyl-carrier protein] reductase activity;oxidoreductase activity | [600045](http://www.ncbi.nlm.nih.gov/entrez/dispomim.cgi?id=600045) | **-1.63** |
| 1415741_AT | TPA regulated locus | [TPARL](http://www.gene.ucl.ac.uk/cgi-bin/nomenclature/searchgenes.pl?field=symbol&anchor=equals&symbol_search=Search&number=100&format=html&sortby=symbol&match=TPARL) | 4-diphosphocytidyl-2C-methyl-D-erythritol synthase activity;hydrolase activity |  | **-1.2** |
| 1415743_AT | histone deacetylase 5 | [HDAC5](http://www.gene.ucl.ac.uk/cgi-bin/nomenclature/searchgenes.pl?field=symbol&anchor=equals&symbol_search=Search&number=100&format=html&sortby=symbol&match=HDAC5) | 4-diphosphocytidyl-2C-methyl-D-erythritol synthase activity;hydrolase activity | [605315](http://www.ncbi.nlm.nih.gov/entrez/dispomim.cgi?id=605315) | **1.39** |
| 1415745_A_AT | Down syndrome critical region gene 3 | [DSCR3](http://www.gene.ucl.ac.uk/cgi-bin/nomenclature/searchgenes.pl?field=symbol&anchor=equals&symbol_search=Search&number=100&format=html&sortby=symbol&match=DSCR3) | 5'-nucleotidase activity | [605298](http://www.ncbi.nlm.nih.gov/entrez/dispomim.cgi?id=605298) | **-1.32** |
| 1415760_S_AT | ATX1 (antioxidant protein 1) homolog 1 (yeast) | [ATOX1](http://www.gene.ucl.ac.uk/cgi-bin/nomenclature/searchgenes.pl?field=symbol&anchor=equals&symbol_search=Search&number=100&format=html&sortby=symbol&match=ATOX1) | 5'-nucleotidase activity | [602270](http://www.ncbi.nlm.nih.gov/entrez/dispomim.cgi?id=602270) | **-1.53** |
| 1415761_AT | mitochondrial ribosomal protein L52 | [MRPL52](http://www.gene.ucl.ac.uk/cgi-bin/nomenclature/searchgenes.pl?field=symbol&anchor=equals&symbol_search=Search&number=100&format=html&sortby=symbol&match=MRPL52) | 5'-nucleotidase activity;magnesium ion binding;nucleotide binding;hydrolase activity |  | **-1.23** |
| 1415762_X_AT | mitochondrial ribosomal protein L52 | [MRPL52](http://www.gene.ucl.ac.uk/cgi-bin/nomenclature/searchgenes.pl?field=symbol&anchor=equals&symbol_search=Search&number=100&format=html&sortby=symbol&match=MRPL52) | 5-oxoprolinase (ATP-hydrolyzing) activity;catalytic activity;hydrolase activity |  | **-1.29** |
| 1415768_A_AT | ubiquitin-conjugating enzyme E2R 2 | [UBE2R2](http://www.gene.ucl.ac.uk/cgi-bin/nomenclature/searchgenes.pl?field=symbol&anchor=equals&symbol_search=Search&number=100&format=html&sortby=symbol&match=UBE2R2) | acetoacetate-CoA ligase activity;CoA-ligase activity;catalytic activity |  | **1.33** |
| 1415769_AT | Itchy | [ITCH](http://www.gene.ucl.ac.uk/cgi-bin/nomenclature/searchgenes.pl?field=symbol&anchor=equals&symbol_search=Search&number=100&format=html&sortby=symbol&match=ITCH) | acetyl-CoA C-acetyltransferase activity;acetyl-CoA C-acyltransferase activity;acyltransferase activity;transferase activity | [606409](http://www.ncbi.nlm.nih.gov/entrez/dispomim.cgi?id=606409) | **-1.24** |
| 1415771_AT | nucleolin | [NCL](http://www.gene.ucl.ac.uk/cgi-bin/nomenclature/searchgenes.pl?field=symbol&anchor=equals&symbol_search=Search&number=100&format=html&sortby=symbol&match=NCL) | acetyl-CoA C-acetyltransferase activity;acetyl-CoA C-acyltransferase activity;acyltransferase activity;transferase activity | [164035](http://www.ncbi.nlm.nih.gov/entrez/dispomim.cgi?id=164035) | **-1.2** |
| 1415778_AT | mortality factor 4 like 2 | [MORF4L2](http://www.gene.ucl.ac.uk/cgi-bin/nomenclature/searchgenes.pl?field=symbol&anchor=equals&symbol_search=Search&number=100&format=html&sortby=symbol&match=MORF4L2) | acetyl-CoA C-acetyltransferase activity;acyltransferase activity;transferase activity | [300409](http://www.ncbi.nlm.nih.gov/entrez/dispomim.cgi?id=300409) | **-1.55** |
| 1415780_A_AT | armadillo repeat containing, X-linked 2 | [ARMCX2](http://www.gene.ucl.ac.uk/cgi-bin/nomenclature/searchgenes.pl?field=symbol&anchor=equals&symbol_search=Search&number=100&format=html&sortby=symbol&match=ARMCX2) | acetyl-CoA C-acetyltransferase activity;acyltransferase activity;transferase activity | [300363](http://www.ncbi.nlm.nih.gov/entrez/dispomim.cgi?id=300363) | **-1.82** |
| 1415785_A_AT | chaperonin subunit 8 (theta) | [CCT8](http://www.gene.ucl.ac.uk/cgi-bin/nomenclature/searchgenes.pl?field=symbol&anchor=equals&symbol_search=Search&number=100&format=html&sortby=symbol&match=CCT8) | acetyl-CoA C-acetyltransferase activity;acyltransferase activity;transferase activity |  | **-1.3** |
| 1415787_AT | alpha glucosidase 2 alpha neutral subunit | [GANAB](http://www.gene.ucl.ac.uk/cgi-bin/nomenclature/searchgenes.pl?field=symbol&anchor=equals&symbol_search=Search&number=100&format=html&sortby=symbol&match=GANAB) | acetyl-CoA C-acyltransferase activity;acyltransferase activity;transferase activity | [104160](http://www.ncbi.nlm.nih.gov/entrez/dispomim.cgi?id=104160) | **-1.33** |
| 1415801_AT | gap junction membrane channel protein alpha 1 | [GJA1](http://www.gene.ucl.ac.uk/cgi-bin/nomenclature/searchgenes.pl?field=symbol&anchor=equals&symbol_search=Search&number=100&format=html&sortby=symbol&match=GJA1) | acetyl-CoA C-acyltransferase activity;acyltransferase activity;transferase activity | [121014](http://www.ncbi.nlm.nih.gov/entrez/dispomim.cgi?id=121014) | **1.71** |
| 1415802_AT | solute carrier family 16 (monocarboxylic acid transporters), member 1 | [SLC16A1](http://www.gene.ucl.ac.uk/cgi-bin/nomenclature/searchgenes.pl?field=symbol&anchor=equals&symbol_search=Search&number=100&format=html&sortby=symbol&match=SLC16A1) | acetyl-CoA C-acyltransferase activity;acyltransferase activity;transferase activity | [600682](http://www.ncbi.nlm.nih.gov/entrez/dispomim.cgi?id=600682) | **1.5** |
| 1415803_AT | chemokine (C-X3-C motif) ligand 1 | [CX3CL1](http://www.gene.ucl.ac.uk/cgi-bin/nomenclature/searchgenes.pl?field=symbol&anchor=equals&symbol_search=Search&number=100&format=html&sortby=symbol&match=CX3CL1) | acetyl-CoA hydrolase activity | [601880](http://www.ncbi.nlm.nih.gov/entrez/dispomim.cgi?id=601880) | **-1.61** |
| 1415810_AT | ubiquitin-like, containing PHD and RING finger domains, 1 | [UHRF1](http://www.gene.ucl.ac.uk/cgi-bin/nomenclature/searchgenes.pl?field=symbol&anchor=equals&symbol_search=Search&number=100&format=html&sortby=symbol&match=UHRF1) | acid phosphatase activity | [607990](http://www.ncbi.nlm.nih.gov/entrez/dispomim.cgi?id=607990) | **-3.45** |
| 1415813_AT | gb:NM_007466.1 /DB_XREF=gi:6671566 /GEN=Api5 /FEA= |  | acid phosphatase activity |  | **-1.28** |
| 1415820_X_AT | gb:NM_023144.1 /DB_XREF=gi:12963530 /GEN=Nono /FEA |  | acid phosphatase activity;hydrolase activity |  | **-1.36** |
| 1415826_AT | ATPase, H+ transporting, lysosomal 50/57kDa, V1 subunit H | [ATP6V1H](http://www.gene.ucl.ac.uk/cgi-bin/nomenclature/searchgenes.pl?field=symbol&anchor=equals&symbol_search=Search&number=100&format=html&sortby=symbol&match=ATP6V1H) | actin binding | [608861](http://www.ncbi.nlm.nih.gov/entrez/dispomim.cgi?id=608861) | **-1.36** |
| 1415827_A_AT | DNA segment, Chr 3, University of California at Los Angeles 1 | [D3UCLA1](http://www.gene.ucl.ac.uk/cgi-bin/nomenclature/searchgenes.pl?field=symbol&anchor=equals&symbol_search=Search&number=100&format=html&sortby=symbol&match=D3UCLA1) | actin binding |  | **-1.77** |
| 1415828_A_AT | DNA segment, Chr 3, University of California at Los Angeles 1 | [D3UCLA1](http://www.gene.ucl.ac.uk/cgi-bin/nomenclature/searchgenes.pl?field=symbol&anchor=equals&symbol_search=Search&number=100&format=html&sortby=symbol&match=D3UCLA1) | actin binding |  | **-1.53** |
| 1415829_AT | lamin B receptor | [LBR](http://www.gene.ucl.ac.uk/cgi-bin/nomenclature/searchgenes.pl?field=symbol&anchor=equals&symbol_search=Search&number=100&format=html&sortby=symbol&match=LBR) | actin binding | [600024](http://www.ncbi.nlm.nih.gov/entrez/dispomim.cgi?id=600024) | **1.28** |
| 1415836_AT | pyrroline-5-carboxylate synthetase (glutamate gamma-semialdehyde synthetase) | [PYCS](http://www.gene.ucl.ac.uk/cgi-bin/nomenclature/searchgenes.pl?field=symbol&anchor=equals&symbol_search=Search&number=100&format=html&sortby=symbol&match=PYCS) | actin binding | [138250](http://www.ncbi.nlm.nih.gov/entrez/dispomim.cgi?id=138250) | **-1.52** |
| 1415839_A_AT | nucleophosmin 1 | [NPM1](http://www.gene.ucl.ac.uk/cgi-bin/nomenclature/searchgenes.pl?field=symbol&anchor=equals&symbol_search=Search&number=100&format=html&sortby=symbol&match=NPM1) | actin binding | [164040](http://www.ncbi.nlm.nih.gov/entrez/dispomim.cgi?id=164040) | **-1.48** |
| 1415850_AT | RAS p21 protein activator 3 | [RASA3](http://www.gene.ucl.ac.uk/cgi-bin/nomenclature/searchgenes.pl?field=symbol&anchor=equals&symbol_search=Search&number=100&format=html&sortby=symbol&match=RASA3) | actin binding | [605182](http://www.ncbi.nlm.nih.gov/entrez/dispomim.cgi?id=605182) | **-1.46** |
| 1415854_AT | kit ligand | [KITL](http://www.gene.ucl.ac.uk/cgi-bin/nomenclature/searchgenes.pl?field=symbol&anchor=equals&symbol_search=Search&number=100&format=html&sortby=symbol&match=KITL) | actin binding |  | **1.83** |
| 1415855_AT | kit ligand | [KITL](http://www.gene.ucl.ac.uk/cgi-bin/nomenclature/searchgenes.pl?field=symbol&anchor=equals&symbol_search=Search&number=100&format=html&sortby=symbol&match=KITL) | actin binding |  | **2.14** |
| 1415860_AT | karyopherin (importin) alpha 2 | [KPNA2](http://www.gene.ucl.ac.uk/cgi-bin/nomenclature/searchgenes.pl?field=symbol&anchor=equals&symbol_search=Search&number=100&format=html&sortby=symbol&match=KPNA2) | actin binding | [600685](http://www.ncbi.nlm.nih.gov/entrez/dispomim.cgi?id=600685) | **-1.82** |
| 1415864_AT | 2,3-bisphosphoglycerate mutase | [BPGM](http://www.gene.ucl.ac.uk/cgi-bin/nomenclature/searchgenes.pl?field=symbol&anchor=equals&symbol_search=Search&number=100&format=html&sortby=symbol&match=BPGM) | actin binding | [222800](http://www.ncbi.nlm.nih.gov/entrez/dispomim.cgi?id=222800) | **1.82** |
| 1415865_S_AT | 2,3-bisphosphoglycerate mutase | [BPGM](http://www.gene.ucl.ac.uk/cgi-bin/nomenclature/searchgenes.pl?field=symbol&anchor=equals&symbol_search=Search&number=100&format=html&sortby=symbol&match=BPGM) | actin binding | [222800](http://www.ncbi.nlm.nih.gov/entrez/dispomim.cgi?id=222800) | **1.64** |
| 1415867_AT | chaperonin subunit 4 (delta) | [CCT4](http://www.gene.ucl.ac.uk/cgi-bin/nomenclature/searchgenes.pl?field=symbol&anchor=equals&symbol_search=Search&number=100&format=html&sortby=symbol&match=CCT4) | actin binding | [605142](http://www.ncbi.nlm.nih.gov/entrez/dispomim.cgi?id=605142) | **-1.31** |
| 1415869_A_AT | tripartite motif protein 28 | [TRIM28](http://www.gene.ucl.ac.uk/cgi-bin/nomenclature/searchgenes.pl?field=symbol&anchor=equals&symbol_search=Search&number=100&format=html&sortby=symbol&match=TRIM28) | actin binding | [601742](http://www.ncbi.nlm.nih.gov/entrez/dispomim.cgi?id=601742) | **-1.33** |
| 1415870_AT | gb:NM_007594.1 /DB_XREF=gi:6680839 /GEN=Calu /FEA= |  | actin binding;actin filament binding;protein binding, bridging |  | **-1.29** |
| 1415871_AT | gb:NM_009369.1 /DB_XREF=gi:6678320 /GEN=Tgfbi /FEA |  | actin binding;actin monomer binding |  | **-2.07** |
| 1415876_A_AT | ribosomal protein S26 | [RPS26](http://www.gene.ucl.ac.uk/cgi-bin/nomenclature/searchgenes.pl?field=symbol&anchor=equals&symbol_search=Search&number=100&format=html&sortby=symbol&match=RPS26) | actin binding;actin monomer binding | [603701](http://www.ncbi.nlm.nih.gov/entrez/dispomim.cgi?id=603701) | **-1.31** |
| 1415877_AT | dihydropyrimidinase-like 3 | [DPYSL3](http://www.gene.ucl.ac.uk/cgi-bin/nomenclature/searchgenes.pl?field=symbol&anchor=equals&symbol_search=Search&number=100&format=html&sortby=symbol&match=DPYSL3) | actin binding;actin monomer binding | [601168](http://www.ncbi.nlm.nih.gov/entrez/dispomim.cgi?id=601168) | **-1.99** |
| 1415881_AT | growth hormone inducible transmembrane protein | [GHITM](http://www.gene.ucl.ac.uk/cgi-bin/nomenclature/searchgenes.pl?field=symbol&anchor=equals&symbol_search=Search&number=100&format=html&sortby=symbol&match=GHITM) | actin binding;calcium ion binding;actin filament binding;protein binding, bridging |  | **1.24** |
| 1415882_AT | growth hormone inducible transmembrane protein | [GHITM](http://www.gene.ucl.ac.uk/cgi-bin/nomenclature/searchgenes.pl?field=symbol&anchor=equals&symbol_search=Search&number=100&format=html&sortby=symbol&match=GHITM) | actin binding;calcium ion binding;microtubule binding |  | **1.26** |
| 1415886_AT | SH2 domain containing 3C | [SH2D3C](http://www.gene.ucl.ac.uk/cgi-bin/nomenclature/searchgenes.pl?field=symbol&anchor=equals&symbol_search=Search&number=100&format=html&sortby=symbol&match=SH2D3C) | actin binding;calmodulin binding | [604722](http://www.ncbi.nlm.nih.gov/entrez/dispomim.cgi?id=604722) | **1.77** |
| 1415889_A_AT | tumor rejection antigen gp96 | [TRA1](http://www.gene.ucl.ac.uk/cgi-bin/nomenclature/searchgenes.pl?field=symbol&anchor=equals&symbol_search=Search&number=100&format=html&sortby=symbol&match=TRA1) | actin binding;calmodulin binding | [191175](http://www.ncbi.nlm.nih.gov/entrez/dispomim.cgi?id=191175) | **-1.42** |
| 1415891_AT | succinate-CoA ligase, GDP-forming, alpha subunit | [SUCLG1](http://www.gene.ucl.ac.uk/cgi-bin/nomenclature/searchgenes.pl?field=symbol&anchor=equals&symbol_search=Search&number=100&format=html&sortby=symbol&match=SUCLG1) | actin binding;calmodulin binding |  | **1.48** |
| 1415892_AT | sphingosine phosphate lyase 1 | [SGPL1](http://www.gene.ucl.ac.uk/cgi-bin/nomenclature/searchgenes.pl?field=symbol&anchor=equals&symbol_search=Search&number=100&format=html&sortby=symbol&match=SGPL1) | actin binding;calmodulin binding | [603729](http://www.ncbi.nlm.nih.gov/entrez/dispomim.cgi?id=603729) | **-2.26** |
| 1415895_AT | small nuclear ribonucleoprotein N | [SNRPN](http://www.gene.ucl.ac.uk/cgi-bin/nomenclature/searchgenes.pl?field=symbol&anchor=equals&symbol_search=Search&number=100&format=html&sortby=symbol&match=SNRPN) | actin binding;calmodulin binding | [182279](http://www.ncbi.nlm.nih.gov/entrez/dispomim.cgi?id=182279) | **1.31** |
| 1415896_X_AT | small nuclear ribonucleoprotein N | [SNRPN](http://www.gene.ucl.ac.uk/cgi-bin/nomenclature/searchgenes.pl?field=symbol&anchor=equals&symbol_search=Search&number=100&format=html&sortby=symbol&match=SNRPN) | actin binding;calmodulin binding | [182279](http://www.ncbi.nlm.nih.gov/entrez/dispomim.cgi?id=182279) | **1.67** |
| 1415903_AT | solute carrier family 38, member 1 | [SLC38A1](http://www.gene.ucl.ac.uk/cgi-bin/nomenclature/searchgenes.pl?field=symbol&anchor=equals&symbol_search=Search&number=100&format=html&sortby=symbol&match=SLC38A1) | actin binding;calmodulin binding | [608490](http://www.ncbi.nlm.nih.gov/entrez/dispomim.cgi?id=608490) | **-1.45** |
| 1415904_AT | lipoprotein lipase | [LPL](http://www.gene.ucl.ac.uk/cgi-bin/nomenclature/searchgenes.pl?field=symbol&anchor=equals&symbol_search=Search&number=100&format=html&sortby=symbol&match=LPL) | actin binding;calmodulin binding | [238600](http://www.ncbi.nlm.nih.gov/entrez/dispomim.cgi?id=238600) | **1.39** |
| 1415906_AT | thymosin, beta 4, X chromosome | [TMSB4X](http://www.gene.ucl.ac.uk/cgi-bin/nomenclature/searchgenes.pl?field=symbol&anchor=equals&symbol_search=Search&number=100&format=html&sortby=symbol&match=TMSB4X) | actin binding;calmodulin binding | [300159](http://www.ncbi.nlm.nih.gov/entrez/dispomim.cgi?id=300159) | **-1.44** |
| 1415907_AT | cyclin D3 | [CCND3](http://www.gene.ucl.ac.uk/cgi-bin/nomenclature/searchgenes.pl?field=symbol&anchor=equals&symbol_search=Search&number=100&format=html&sortby=symbol&match=CCND3) | actin binding;glutamate-tRNA ligase activity;ligase activity;ATP binding;catalytic activity;tRNA ligase activity | [123834](http://www.ncbi.nlm.nih.gov/entrez/dispomim.cgi?id=123834) | **-1.51** |
| 1415910_S_AT | cytokine induced apoptosis inhibitor 1 | [CIAPIN1](http://www.gene.ucl.ac.uk/cgi-bin/nomenclature/searchgenes.pl?field=symbol&anchor=equals&symbol_search=Search&number=100&format=html&sortby=symbol&match=CIAPIN1) | actin binding;glutamate-tRNA ligase activity;ligase activity;catalytic activity;ATP binding;tRNA ligase activity | [608943](http://www.ncbi.nlm.nih.gov/entrez/dispomim.cgi?id=608943) | **1.49** |
| 1415914_AT | heterogeneous nuclear ribonucleoprotein A/B | [HNRPAB](http://www.gene.ucl.ac.uk/cgi-bin/nomenclature/searchgenes.pl?field=symbol&anchor=equals&symbol_search=Search&number=100&format=html&sortby=symbol&match=HNRPAB) | actin binding;microtubule binding;calcium ion binding | [602688](http://www.ncbi.nlm.nih.gov/entrez/dispomim.cgi?id=602688) | **-1.22** |
| 1415919_AT | neural proliferation, differentiation and control gene 1 | [NPDC1](http://www.gene.ucl.ac.uk/cgi-bin/nomenclature/searchgenes.pl?field=symbol&anchor=equals&symbol_search=Search&number=100&format=html&sortby=symbol&match=NPDC1) | actin binding;motor activity;ATP binding | [605798](http://www.ncbi.nlm.nih.gov/entrez/dispomim.cgi?id=605798) | **-1.3** |
| 1415927_AT | actin, alpha, cardiac | [ACTC1](http://www.gene.ucl.ac.uk/cgi-bin/nomenclature/searchgenes.pl?field=symbol&anchor=equals&symbol_search=Search&number=100&format=html&sortby=symbol&match=ACTC1) | actin binding;motor activity;ATP binding |  | **-1.82** |
| 1415929_AT | microtubule-associated protein 1 light chain 3 beta | [MAP1LC3B](http://www.gene.ucl.ac.uk/cgi-bin/nomenclature/searchgenes.pl?field=symbol&anchor=equals&symbol_search=Search&number=100&format=html&sortby=symbol&match=MAP1LC3B) | actin binding;motor activity;ATP binding;calmodulin binding |  | **1.27** |
| 1415931_AT | gb:M14951.1 /DB_XREF=gi:193484 /FEA=FLmRNA /CNT=32 |  | actin binding;motor activity;ATP binding;calmodulin binding |  | **-7.78** |
| 1415933_A_AT | cytochrome c oxidase, subunit Va | [COX5A](http://www.gene.ucl.ac.uk/cgi-bin/nomenclature/searchgenes.pl?field=symbol&anchor=equals&symbol_search=Search&number=100&format=html&sortby=symbol&match=COX5A) | actin binding;motor activity;ATP binding;calmodulin binding | [603773](http://www.ncbi.nlm.nih.gov/entrez/dispomim.cgi?id=603773) | **1.27** |
| 1415935_AT | SPARC related modular calcium binding 2 | [SMOC2](http://www.gene.ucl.ac.uk/cgi-bin/nomenclature/searchgenes.pl?field=symbol&anchor=equals&symbol_search=Search&number=100&format=html&sortby=symbol&match=SMOC2) | actin binding;motor activity;ATP binding;calmodulin binding | [607223](http://www.ncbi.nlm.nih.gov/entrez/dispomim.cgi?id=607223) | **-1.67** |
| 1415943_AT | syndecan 1 | [SDC1](http://www.gene.ucl.ac.uk/cgi-bin/nomenclature/searchgenes.pl?field=symbol&anchor=equals&symbol_search=Search&number=100&format=html&sortby=symbol&match=SDC1) | actin binding;motor activity;ATP binding;calmodulin binding | [186355](http://www.ncbi.nlm.nih.gov/entrez/dispomim.cgi?id=186355) | **-1.72** |
| 1415945_AT | minichromosome maintenance deficient 5, cell division cycle 46 (S. cerevisiae) | [MCM5](http://www.gene.ucl.ac.uk/cgi-bin/nomenclature/searchgenes.pl?field=symbol&anchor=equals&symbol_search=Search&number=100&format=html&sortby=symbol&match=MCM5) | actin binding;motor activity;GTPase activator activity;ATP binding;diacylglycerol binding;calmodulin binding | [602696](http://www.ncbi.nlm.nih.gov/entrez/dispomim.cgi?id=602696) | **-2.61** |
| 1415951_AT | FK506 binding protein 10 | [FKBP10](http://www.gene.ucl.ac.uk/cgi-bin/nomenclature/searchgenes.pl?field=symbol&anchor=equals&symbol_search=Search&number=100&format=html&sortby=symbol&match=FKBP10) | actin binding;myosin binding;signal transducer activity;calmodulin binding | [607063](http://www.ncbi.nlm.nih.gov/entrez/dispomim.cgi?id=607063) | **-1.48** |
| 1415958_AT | solute carrier family 2 (facilitated glucose transporter), member 4 | [SLC2A4](http://www.gene.ucl.ac.uk/cgi-bin/nomenclature/searchgenes.pl?field=symbol&anchor=equals&symbol_search=Search&number=100&format=html&sortby=symbol&match=SLC2A4) | actin binding;protein dimerization activity;structural constituent of ribosome | [138190](http://www.ncbi.nlm.nih.gov/entrez/dispomim.cgi?id=138190) | **1.89** |
| 1415959_AT | solute carrier family 2 (facilitated glucose transporter), member 4 | [SLC2A4](http://www.gene.ucl.ac.uk/cgi-bin/nomenclature/searchgenes.pl?field=symbol&anchor=equals&symbol_search=Search&number=100&format=html&sortby=symbol&match=SLC2A4) | actin binding;structural constituent of cytoskeleton | [138190](http://www.ncbi.nlm.nih.gov/entrez/dispomim.cgi?id=138190) | **2.12** |
| 1415961_AT | integral membrane protein 2C | [ITM2C](http://www.gene.ucl.ac.uk/cgi-bin/nomenclature/searchgenes.pl?field=symbol&anchor=equals&symbol_search=Search&number=100&format=html&sortby=symbol&match=ITM2C) | actin binding;structural constituent of cytoskeleton |  | **-1.69** |
| 1415963_AT | heterogeneous nuclear ribonucleoprotein H2 | [HNRPH2](http://www.gene.ucl.ac.uk/cgi-bin/nomenclature/searchgenes.pl?field=symbol&anchor=equals&symbol_search=Search&number=100&format=html&sortby=symbol&match=HNRPH2) | actin binding;structural constituent of cytoskeleton | [601036](http://www.ncbi.nlm.nih.gov/entrez/dispomim.cgi?id=601036) | **-1.26** |
| 1415964_AT | stearoyl-Coenzyme A desaturase 1 | [SCD1](http://www.gene.ucl.ac.uk/cgi-bin/nomenclature/searchgenes.pl?field=symbol&anchor=equals&symbol_search=Search&number=100&format=html&sortby=symbol&match=SCD1) | actin binding;structural constituent of cytoskeleton |  | **2.41** |
| 1415965_AT | stearoyl-Coenzyme A desaturase 1 | [SCD1](http://www.gene.ucl.ac.uk/cgi-bin/nomenclature/searchgenes.pl?field=symbol&anchor=equals&symbol_search=Search&number=100&format=html&sortby=symbol&match=SCD1) | actin binding;tropomyosin binding |  | **4.41** |
| 1415966_A_AT | gb:NM_133666.1 /DB_XREF=gi:19526813 /GEN=Ndufv1 /F |  | actin-dependent ATPase activity;actin filament binding;motor activity;ATP binding;microfilament motor activity |  | **1.33** |
| 1415967_AT | NADH dehydrogenase (ubiquinone) flavoprotein 1 | [NDUFV1](http://www.gene.ucl.ac.uk/cgi-bin/nomenclature/searchgenes.pl?field=symbol&anchor=equals&symbol_search=Search&number=100&format=html&sortby=symbol&match=NDUFV1) | acyl-CoA oxidase activity;oxidoreductase activity | [161015](http://www.ncbi.nlm.nih.gov/entrez/dispomim.cgi?id=161015) | **1.35** |
| 1415971_AT | myristoylated alanine rich protein kinase C substrate | [MARCKS](http://www.gene.ucl.ac.uk/cgi-bin/nomenclature/searchgenes.pl?field=symbol&anchor=equals&symbol_search=Search&number=100&format=html&sortby=symbol&match=MARCKS) | acyl-CoA oxidase activity;oxidoreductase activity | [177061](http://www.ncbi.nlm.nih.gov/entrez/dispomim.cgi?id=177061) | **-1.41** |
| 1415973_AT | Myristoylated alanine rich protein kinase C substrate | [MARCKS](http://www.gene.ucl.ac.uk/cgi-bin/nomenclature/searchgenes.pl?field=symbol&anchor=equals&symbol_search=Search&number=100&format=html&sortby=symbol&match=MARCKS) | acyl-CoA thioesterase activity;acyl-CoA thioesterase I activity;catalytic activity;serine esterase activity;hydrolase activity;palmitoyl-CoA hydrolase activity | [177061](http://www.ncbi.nlm.nih.gov/entrez/dispomim.cgi?id=177061) | **-1.45** |
| 1415979_X_AT | ribosomal protein L7 | [RPL7](http://www.gene.ucl.ac.uk/cgi-bin/nomenclature/searchgenes.pl?field=symbol&anchor=equals&symbol_search=Search&number=100&format=html&sortby=symbol&match=RPL7) | acyl-CoA thioesterase activity;acyl-CoA thioesterase I activity;catalytic activity;serine esterase activity;hydrolase activity;palmitoyl-CoA hydrolase activity | [604166](http://www.ncbi.nlm.nih.gov/entrez/dispomim.cgi?id=604166) | **-1.23** |
| 1415983_AT | lymphocyte cytosolic protein 1 | [LCP1](http://www.gene.ucl.ac.uk/cgi-bin/nomenclature/searchgenes.pl?field=symbol&anchor=equals&symbol_search=Search&number=100&format=html&sortby=symbol&match=LCP1) | acyl-CoA thioesterase activity;acyl-CoA thioesterase I activity;serine esterase activity;catalytic activity;hydrolase activity;palmitoyl-CoA hydrolase activity | [153430](http://www.ncbi.nlm.nih.gov/entrez/dispomim.cgi?id=153430) | **-4.13** |
| 1415984_AT | acetyl-Coenzyme A dehydrogenase, medium chain | [ACADM](http://www.gene.ucl.ac.uk/cgi-bin/nomenclature/searchgenes.pl?field=symbol&anchor=equals&symbol_search=Search&number=100&format=html&sortby=symbol&match=ACADM) | acyl-CoA thioesterase activity;serine esterase activity;hydrolase activity | [607008](http://www.ncbi.nlm.nih.gov/entrez/dispomim.cgi?id=607008) | **1.78** |
| 1415987_AT | high density lipoprotein (HDL) binding protein | [HDLBP](http://www.gene.ucl.ac.uk/cgi-bin/nomenclature/searchgenes.pl?field=symbol&anchor=equals&symbol_search=Search&number=100&format=html&sortby=symbol&match=HDLBP) | acyl-CoA thioesterase activity;serine esterase activity;hydrolase activity | [142695](http://www.ncbi.nlm.nih.gov/entrez/dispomim.cgi?id=142695) | **1.24** |
| 1415989_AT | vascular cell adhesion molecule 1 | [VCAM1](http://www.gene.ucl.ac.uk/cgi-bin/nomenclature/searchgenes.pl?field=symbol&anchor=equals&symbol_search=Search&number=100&format=html&sortby=symbol&match=VCAM1) | acylglycerol lipase activity;catalytic activity;aminopeptidase activity;serine esterase activity;hydrolase activity | [192225](http://www.ncbi.nlm.nih.gov/entrez/dispomim.cgi?id=192225) | **-2.41** |
| 1415998_AT | voltage-dependent anion channel 1 | [VDAC1](http://www.gene.ucl.ac.uk/cgi-bin/nomenclature/searchgenes.pl?field=symbol&anchor=equals&symbol_search=Search&number=100&format=html&sortby=symbol&match=VDAC1) | acylglycerol lipase activity;serine esterase activity;aminopeptidase activity;catalytic activity;hydrolase activity | [604492](http://www.ncbi.nlm.nih.gov/entrez/dispomim.cgi?id=604492) | **1.4** |
| 1416002_X_AT | coactosin-like 1 (Dictyostelium) | [COTL1](http://www.gene.ucl.ac.uk/cgi-bin/nomenclature/searchgenes.pl?field=symbol&anchor=equals&symbol_search=Search&number=100&format=html&sortby=symbol&match=COTL1) | acylglycerol lipase activity;serine esterase activity;catalytic activity;aminopeptidase activity;hydrolase activity | [606748](http://www.ncbi.nlm.nih.gov/entrez/dispomim.cgi?id=606748) | **-2.01** |
| 1416006_AT | midkine | [MDK](http://www.gene.ucl.ac.uk/cgi-bin/nomenclature/searchgenes.pl?field=symbol&anchor=equals&symbol_search=Search&number=100&format=html&sortby=symbol&match=MDK) | acylphosphatase activity;hydrolase activity | [162096](http://www.ncbi.nlm.nih.gov/entrez/dispomim.cgi?id=162096) | **-1.71** |
| 1416007_AT | special AT-rich sequence binding protein 1 | [SATB1](http://www.gene.ucl.ac.uk/cgi-bin/nomenclature/searchgenes.pl?field=symbol&anchor=equals&symbol_search=Search&number=100&format=html&sortby=symbol&match=SATB1) | acyltransferase activity;transferase activity | [602075](http://www.ncbi.nlm.nih.gov/entrez/dispomim.cgi?id=602075) | **1.55** |
| 1416008_AT | special AT-rich sequence binding protein 1 | [SATB1](http://www.gene.ucl.ac.uk/cgi-bin/nomenclature/searchgenes.pl?field=symbol&anchor=equals&symbol_search=Search&number=100&format=html&sortby=symbol&match=SATB1) | acyltransferase activity;transferase activity | [602075](http://www.ncbi.nlm.nih.gov/entrez/dispomim.cgi?id=602075) | **1.47** |
| 1416013_AT | phospholipase D3 | [PLD3](http://www.gene.ucl.ac.uk/cgi-bin/nomenclature/searchgenes.pl?field=symbol&anchor=equals&symbol_search=Search&number=100&format=html&sortby=symbol&match=PLD3) | acyltransferase activity;transferase activity |  | **-1.97** |
| 1416020_A_AT | ATP synthase, H+ transporting, mitochondrial F0 complex, subunit c (subunit 9), isoform 1 | [ATP5G1](http://www.gene.ucl.ac.uk/cgi-bin/nomenclature/searchgenes.pl?field=symbol&anchor=equals&symbol_search=Search&number=100&format=html&sortby=symbol&match=ATP5G1) | acyltransferase activity;transferase activity | [603192](http://www.ncbi.nlm.nih.gov/entrez/dispomim.cgi?id=603192) | **1.39** |
| 1416021_A_AT | fatty acid binding protein 5, epidermal | [FABP5](http://www.gene.ucl.ac.uk/cgi-bin/nomenclature/searchgenes.pl?field=symbol&anchor=equals&symbol_search=Search&number=100&format=html&sortby=symbol&match=FABP5) | adenosylmethionine decarboxylase activity | [605168](http://www.ncbi.nlm.nih.gov/entrez/dispomim.cgi?id=605168) | **-1.56** |
| 1416023_AT | fatty acid binding protein 3, muscle and heart | [FABP3](http://www.gene.ucl.ac.uk/cgi-bin/nomenclature/searchgenes.pl?field=symbol&anchor=equals&symbol_search=Search&number=100&format=html&sortby=symbol&match=FABP3) | adenosylmethionine decarboxylase activity;carboxy-lyase activity;lyase activity | [134651](http://www.ncbi.nlm.nih.gov/entrez/dispomim.cgi?id=134651) | **1.44** |
| 1416026_A_AT | gb:NM_009076.1 /DB_XREF=gi:6677768 /GEN=Rpl12 /FEA |  | adenosylmethionine decarboxylase activity;carboxy-lyase activity;lyase activity |  | **-1.42** |
| 1416028_A_AT | hematological and neurological expressed sequence 1 | [HN1](http://www.gene.ucl.ac.uk/cgi-bin/nomenclature/searchgenes.pl?field=symbol&anchor=equals&symbol_search=Search&number=100&format=html&sortby=symbol&match=HN1) | adenylate cyclase activity;magnesium ion binding;guanylate cyclase activity;calcium/calmodulin-responsive adenylate cyclase activity;lyase activity |  | **-2.95** |
| 1416033_AT | RIKEN cDNA 1110006I15 gene | [1110006I15RIK](http://www.gene.ucl.ac.uk/cgi-bin/nomenclature/searchgenes.pl?field=symbol&anchor=equals&symbol_search=Search&number=100&format=html&sortby=symbol&match=1110006I15RIK) | adenylate cyclase activity;magnesium ion binding;guanylate cyclase activity;lyase activity |  | **1.56** |
| 1416037_A_AT | chaperonin subunit 2 (beta) | [CCT2](http://www.gene.ucl.ac.uk/cgi-bin/nomenclature/searchgenes.pl?field=symbol&anchor=equals&symbol_search=Search&number=100&format=html&sortby=symbol&match=CCT2) | adenylate cyclase activity;magnesium ion binding;guanylate cyclase activity;lyase activity | [605139](http://www.ncbi.nlm.nih.gov/entrez/dispomim.cgi?id=605139) | **-1.27** |
| 1416048_AT | polyhomeotic-like 2 (Drosophila) | [PHC2](http://www.gene.ucl.ac.uk/cgi-bin/nomenclature/searchgenes.pl?field=symbol&anchor=equals&symbol_search=Search&number=100&format=html&sortby=symbol&match=PHC2) | adenylate cyclase activity;magnesium ion binding;guanylate cyclase activity;lyase activity | [602979](http://www.ncbi.nlm.nih.gov/entrez/dispomim.cgi?id=602979) | **-1.26** |
| 1416053_AT | leucine rich repeat protein 1, neuronal | [LRRN1](http://www.gene.ucl.ac.uk/cgi-bin/nomenclature/searchgenes.pl?field=symbol&anchor=equals&symbol_search=Search&number=100&format=html&sortby=symbol&match=LRRN1) | ADP-ribosylation factor binding;protein transporter activity |  | **-2.15** |
| 1416056_A_AT | nuclear protein 15.6 | [NP15](http://www.gene.ucl.ac.uk/cgi-bin/nomenclature/searchgenes.pl?field=symbol&anchor=equals&symbol_search=Search&number=100&format=html&sortby=symbol&match=NP15) | alanine-tRNA ligase activity;ligase activity;ATP binding;tRNA ligase activity |  | **1.21** |
| 1416057_AT | nuclear protein 15.6 | [NP15](http://www.gene.ucl.ac.uk/cgi-bin/nomenclature/searchgenes.pl?field=symbol&anchor=equals&symbol_search=Search&number=100&format=html&sortby=symbol&match=NP15) | alanine-tRNA ligase activity;nucleic acid binding;ligase activity;ATP binding;tRNA ligase activity |  | **1.21** |
| 1416058_S_AT | ATP synthase, H+ transporting, mitochondrial F1 complex, gamma polypeptide 1 | [ATP5C1](http://www.gene.ucl.ac.uk/cgi-bin/nomenclature/searchgenes.pl?field=symbol&anchor=equals&symbol_search=Search&number=100&format=html&sortby=symbol&match=ATP5C1) | alanine-tRNA ligase activity;nucleic acid binding;ligase activity;ATP binding;tRNA ligase activity | [108729](http://www.ncbi.nlm.nih.gov/entrez/dispomim.cgi?id=108729) | **1.28** |
| 1416059_AT | SEC23B (S. cerevisiae) | [SEC23B](http://www.gene.ucl.ac.uk/cgi-bin/nomenclature/searchgenes.pl?field=symbol&anchor=equals&symbol_search=Search&number=100&format=html&sortby=symbol&match=SEC23B) | alcohol dehydrogenase (NADP+) activity;oxidoreductase activity;aldehyde reductase activity |  | **-1.27** |
| 1416064_A_AT | heat shock 70kD protein 5 (glucose-regulated protein) | [HSPA5](http://www.gene.ucl.ac.uk/cgi-bin/nomenclature/searchgenes.pl?field=symbol&anchor=equals&symbol_search=Search&number=100&format=html&sortby=symbol&match=HSPA5) | alcohol dehydrogenase (NADP+) activity;oxidoreductase activity;aldehyde reductase activity | [138120](http://www.ncbi.nlm.nih.gov/entrez/dispomim.cgi?id=138120) | **-1.61** |
| 1416065_A_AT | ankyrin repeat domain 10 | [ANKRD10](http://www.gene.ucl.ac.uk/cgi-bin/nomenclature/searchgenes.pl?field=symbol&anchor=equals&symbol_search=Search&number=100&format=html&sortby=symbol&match=ANKRD10) | alcohol dehydrogenase (NADP+) activity;oxidoreductase activity;aldehyde reductase activity |  | **-2.54** |
| 1416066_AT | CD9 antigen | [CD9](http://www.gene.ucl.ac.uk/cgi-bin/nomenclature/searchgenes.pl?field=symbol&anchor=equals&symbol_search=Search&number=100&format=html&sortby=symbol&match=CD9) | alcohol dehydrogenase (NADP+) activity;oxidoreductase activity;aldehyde reductase activity | [143030](http://www.ncbi.nlm.nih.gov/entrez/dispomim.cgi?id=143030) | **-1.68** |
| 1416077_AT | adrenomedullin | [ADM](http://www.gene.ucl.ac.uk/cgi-bin/nomenclature/searchgenes.pl?field=symbol&anchor=equals&symbol_search=Search&number=100&format=html&sortby=symbol&match=ADM) | aldehyde dehydrogenase (NAD) activity;electron transporter activity;oxidoreductase activity;1-pyrroline-5-carboxylate dehydrogenase activity | [103275](http://www.ncbi.nlm.nih.gov/entrez/dispomim.cgi?id=103275) | **1.68** |
| 1416085_S_AT | zinc finger, A20 domain containing 2 | [ZA20D2](http://www.gene.ucl.ac.uk/cgi-bin/nomenclature/searchgenes.pl?field=symbol&anchor=equals&symbol_search=Search&number=100&format=html&sortby=symbol&match=ZA20D2) | aldehyde dehydrogenase (NAD) activity;oxidoreductase activity | [604761](http://www.ncbi.nlm.nih.gov/entrez/dispomim.cgi?id=604761) | **1.21** |
| 1416088_A_AT | ribosomal protein S15 | [RPS15](http://www.gene.ucl.ac.uk/cgi-bin/nomenclature/searchgenes.pl?field=symbol&anchor=equals&symbol_search=Search&number=100&format=html&sortby=symbol&match=RPS15) | aldehyde dehydrogenase (NAD) activity;oxidoreductase activity;aldehyde dehydrogenase activity | [180535](http://www.ncbi.nlm.nih.gov/entrez/dispomim.cgi?id=180535) | **-1.24** |
| 1416090_AT | pyruvate dehydrogenase (lipoamide) beta | [PDHB](http://www.gene.ucl.ac.uk/cgi-bin/nomenclature/searchgenes.pl?field=symbol&anchor=equals&symbol_search=Search&number=100&format=html&sortby=symbol&match=PDHB) | aldehyde dehydrogenase (NAD) activity;oxidoreductase activity;aldehyde dehydrogenase activity | [179060](http://www.ncbi.nlm.nih.gov/entrez/dispomim.cgi?id=179060) | **1.2** |
| 1416099_AT | ribosomal protein L27 | [RPL27](http://www.gene.ucl.ac.uk/cgi-bin/nomenclature/searchgenes.pl?field=symbol&anchor=equals&symbol_search=Search&number=100&format=html&sortby=symbol&match=RPL27) | alkaline phosphatase activity;magnesium ion binding;hydrolase activity | [607526](http://www.ncbi.nlm.nih.gov/entrez/dispomim.cgi?id=607526) | **-1.25** |
| 1416100_AT | eukaryotic translation initiation factor 3, subunit 7 (zeta) | [EIF3S7](http://www.gene.ucl.ac.uk/cgi-bin/nomenclature/searchgenes.pl?field=symbol&anchor=equals&symbol_search=Search&number=100&format=html&sortby=symbol&match=EIF3S7) | alpha-1,6-mannosylglycoprotein 2-beta-N-acetylglucosaminyltransferase activity;methane monooxygenase activity;transferase activity, transferring glycosyl groups;transferase activity | [603915](http://www.ncbi.nlm.nih.gov/entrez/dispomim.cgi?id=603915) | **-1.59** |
| 1416102_AT | tyrosine 3-monooxygenase/tryptophan 5-monooxygenase activation protein, zeta polypeptide | [YWHAZ](http://www.gene.ucl.ac.uk/cgi-bin/nomenclature/searchgenes.pl?field=symbol&anchor=equals&symbol_search=Search&number=100&format=html&sortby=symbol&match=YWHAZ) | alpha-amylase activity;calcium ion binding;hydrolase activity, acting on glycosyl bonds;hydrolase activity | [601288](http://www.ncbi.nlm.nih.gov/entrez/dispomim.cgi?id=601288) | **-1.29** |
| 1416105_AT | nicotinamide nucleotide transhydrogenase | [NNT](http://www.gene.ucl.ac.uk/cgi-bin/nomenclature/searchgenes.pl?field=symbol&anchor=equals&symbol_search=Search&number=100&format=html&sortby=symbol&match=NNT) | alpha-glucosidase activity;hydrolase activity, hydrolyzing O-glycosyl compounds;hydrolase activity, acting on glycosyl bonds;hydrolase activity | [607878](http://www.ncbi.nlm.nih.gov/entrez/dispomim.cgi?id=607878) | **1.7** |
| 1416108_A_AT | RIKEN cDNA 1200002G13 gene | [1200002G13RIK](http://www.gene.ucl.ac.uk/cgi-bin/nomenclature/searchgenes.pl?field=symbol&anchor=equals&symbol_search=Search&number=100&format=html&sortby=symbol&match=1200002G13RIK) | alpha-ketoacid dehydrogenase activity;transketolase activity;oxidoreductase activity;3-methyl-2-oxobutanoate dehydrogenase (2-methylpropanoyl-transferring) activity |  | **-1.77** |
| 1416109_AT | fucosidase, alpha-L- 1, tissue | [FUCA1](http://www.gene.ucl.ac.uk/cgi-bin/nomenclature/searchgenes.pl?field=symbol&anchor=equals&symbol_search=Search&number=100&format=html&sortby=symbol&match=FUCA1) | alpha-L-fucosidase activity | [230000](http://www.ncbi.nlm.nih.gov/entrez/dispomim.cgi?id=230000) | **-1.35** |
| 1416112_AT | gb:NM_007750.1 /DB_XREF=gi:6680992 /GEN=Cox8a /FEA |  | amidase activity;glutamyl-tRNA(Gln) amidotransferase activity |  | **-1.29** |
| 1416118_AT | gb:NM_025863.1 /DB_XREF=gi:13385345 /GEN=2310035M2 |  | amine oxidase activity;oxidoreductase activity |  | **-5.09** |
| 1416119_AT | thioredoxin 1 | [TXN1](http://www.gene.ucl.ac.uk/cgi-bin/nomenclature/searchgenes.pl?field=symbol&anchor=equals&symbol_search=Search&number=100&format=html&sortby=symbol&match=TXN1) | amino acid-polyamine transporter activity |  | **-1.54** |
| 1416121_AT | lysyl oxidase | [LOX](http://www.gene.ucl.ac.uk/cgi-bin/nomenclature/searchgenes.pl?field=symbol&anchor=equals&symbol_search=Search&number=100&format=html&sortby=symbol&match=LOX) | amino acid-polyamine transporter activity;L-glutamine transporter activity | [153455](http://www.ncbi.nlm.nih.gov/entrez/dispomim.cgi?id=153455) | **-2.73** |
| 1416135_AT | apurinic/apyrimidinic endonuclease 1 | [APEX1](http://www.gene.ucl.ac.uk/cgi-bin/nomenclature/searchgenes.pl?field=symbol&anchor=equals&symbol_search=Search&number=100&format=html&sortby=symbol&match=APEX1) | aminomethyltransferase activity | [107748](http://www.ncbi.nlm.nih.gov/entrez/dispomim.cgi?id=107748) | **-1.24** |
| 1416136_AT | matrix metalloproteinase 2 | [MMP2](http://www.gene.ucl.ac.uk/cgi-bin/nomenclature/searchgenes.pl?field=symbol&anchor=equals&symbol_search=Search&number=100&format=html&sortby=symbol&match=MMP2) | aminopeptidase activity | [120360](http://www.ncbi.nlm.nih.gov/entrez/dispomim.cgi?id=120360) | **-2.72** |
| 1416138_AT | annexin A7 | [ANXA7](http://www.gene.ucl.ac.uk/cgi-bin/nomenclature/searchgenes.pl?field=symbol&anchor=equals&symbol_search=Search&number=100&format=html&sortby=symbol&match=ANXA7) | anaphylatoxin receptor activity;G-protein coupled receptor activity;rhodopsin-like receptor activity;C5a anaphylatoxin receptor activity | [186360](http://www.ncbi.nlm.nih.gov/entrez/dispomim.cgi?id=186360) | **-1.78** |
| 1416142_AT | gb:AA103697 /DB_XREF=gi:1649860 /DB_XREF=mo40d06.r |  | anaphylatoxin receptor activity;receptor activity;C3a anaphylatoxin receptor activity;G-protein coupled receptor activity;rhodopsin-like receptor activity |  | **-1.88** |
| 1416143_AT | ATP synthase, H+ transporting, mitochondrial F0 complex, subunit F | [ATP5J](http://www.gene.ucl.ac.uk/cgi-bin/nomenclature/searchgenes.pl?field=symbol&anchor=equals&symbol_search=Search&number=100&format=html&sortby=symbol&match=ATP5J) | anaphylatoxin receptor activity;receptor activity;C3a anaphylatoxin receptor activity;G-protein coupled receptor activity;rhodopsin-like receptor activity | [603152](http://www.ncbi.nlm.nih.gov/entrez/dispomim.cgi?id=603152) | **1.23** |
| 1416146_AT | heat shock protein 4 | [HSPA4](http://www.gene.ucl.ac.uk/cgi-bin/nomenclature/searchgenes.pl?field=symbol&anchor=equals&symbol_search=Search&number=100&format=html&sortby=symbol&match=HSPA4) | angiotensin type II receptor activity;G-protein coupled receptor activity;rhodopsin-like receptor activity | [601113](http://www.ncbi.nlm.nih.gov/entrez/dispomim.cgi?id=601113) | **-1.28** |
| 1416156_AT | gb:BC008520.1 /DB_XREF=gi:14250199 /FEA=FLmRNA /CN |  | anion exchanger activity;antiporter activity;sulfate porter activity |  | **-1.38** |
| 1416163_AT | COP9 (constitutive photomorphogenic) homolog, subunit 4 (Arabidopsis thaliana) | [COPS4](http://www.gene.ucl.ac.uk/cgi-bin/nomenclature/searchgenes.pl?field=symbol&anchor=equals&symbol_search=Search&number=100&format=html&sortby=symbol&match=COPS4) | anion exchanger activity;inorganic anion exchanger activity;antiporter activity;carrier activity |  | **1.24** |
| 1416164_AT | fibulin 5 | [FBLN5](http://www.gene.ucl.ac.uk/cgi-bin/nomenclature/searchgenes.pl?field=symbol&anchor=equals&symbol_search=Search&number=100&format=html&sortby=symbol&match=FBLN5) | antioxidant activity | [604580](http://www.ncbi.nlm.nih.gov/entrez/dispomim.cgi?id=604580) | **1.44** |
| 1416165_AT | RIKEN cDNA 1700093E07 gene | [1700093E07RIK](http://www.gene.ucl.ac.uk/cgi-bin/nomenclature/searchgenes.pl?field=symbol&anchor=equals&symbol_search=Search&number=100&format=html&sortby=symbol&match=1700093E07RIK) | antioxidant activity;oxidoreductase activity;catalytic activity;hydrolase activity;peroxidase activity |  | **-2.7** |
| 1416167_AT | gb:NM_016764.1 /DB_XREF=gi:7948998 /GEN=Prdx4 /FEA |  | apoptotic protease activator activity |  | **-2.08** |
| 1416168_AT | serine (or cysteine) proteinase inhibitor, clade F, member 1 | [SERPINF1](http://www.gene.ucl.ac.uk/cgi-bin/nomenclature/searchgenes.pl?field=symbol&anchor=equals&symbol_search=Search&number=100&format=html&sortby=symbol&match=SERPINF1) | apyrase activity;magnesium ion binding;ATPase activity, uncoupled;hydrolase activity | [172860](http://www.ncbi.nlm.nih.gov/entrez/dispomim.cgi?id=172860) | **-1.76** |
| 1416175_A_AT | voltage-dependent anion channel 3 | [VDAC3](http://www.gene.ucl.ac.uk/cgi-bin/nomenclature/searchgenes.pl?field=symbol&anchor=equals&symbol_search=Search&number=100&format=html&sortby=symbol&match=VDAC3) | ARF guanyl-nucleotide exchange factor activity |  | **1.37** |
| 1416177_AT | RNA binding motif protein, X chromosome retrogene | [RBMXRT](http://www.gene.ucl.ac.uk/cgi-bin/nomenclature/searchgenes.pl?field=symbol&anchor=equals&symbol_search=Search&number=100&format=html&sortby=symbol&match=RBMXRT) | arsenate reductase (thioredoxin) activity;electron transporter activity |  | **-1.29** |
| 1416178_A_AT | pleckstrin homology domain containing, family B (evectins) member 1 | [PLEKHB1](http://www.gene.ucl.ac.uk/cgi-bin/nomenclature/searchgenes.pl?field=symbol&anchor=equals&symbol_search=Search&number=100&format=html&sortby=symbol&match=PLEKHB1) | arsenite methyltransferase activity;methyltransferase activity;methylarsonite methyltransferase activity;S-adenosylmethionine-dependent methyltransferase activity;transferase activity | [607651](http://www.ncbi.nlm.nih.gov/entrez/dispomim.cgi?id=607651) | **2.1** |
| 1416183_A_AT | lactate dehydrogenase 2, B chain | [LDH2](http://www.gene.ucl.ac.uk/cgi-bin/nomenclature/searchgenes.pl?field=symbol&anchor=equals&symbol_search=Search&number=100&format=html&sortby=symbol&match=LDH2) | asparaginase activity |  | **1.57** |
| 1416186_AT | proline-rich nuclear receptor coactivator 2 | [PNRC2](http://www.gene.ucl.ac.uk/cgi-bin/nomenclature/searchgenes.pl?field=symbol&anchor=equals&symbol_search=Search&number=100&format=html&sortby=symbol&match=PNRC2) | asparaginase activity |  | **-1.37** |
| 1416189_A_AT | Sec61 alpha 1 subunit (S. cerevisiae) | [SEC61A1](http://www.gene.ucl.ac.uk/cgi-bin/nomenclature/searchgenes.pl?field=symbol&anchor=equals&symbol_search=Search&number=100&format=html&sortby=symbol&match=SEC61A1) | aspartate transaminase activity;transaminase activity;catalytic activity;transferase activity |  | **-2.04** |
| 1416191_AT | Sec61 alpha 1 subunit (S. cerevisiae) | [SEC61A1](http://www.gene.ucl.ac.uk/cgi-bin/nomenclature/searchgenes.pl?field=symbol&anchor=equals&symbol_search=Search&number=100&format=html&sortby=symbol&match=SEC61A1) | aspartate transaminase activity;transaminase activity;catalytic activity;transferase activity |  | **-1.36** |
| 1416194_AT | cytochrome P450, family 4, subfamily b, polypeptide 1 | [CYP4B1](http://www.gene.ucl.ac.uk/cgi-bin/nomenclature/searchgenes.pl?field=symbol&anchor=equals&symbol_search=Search&number=100&format=html&sortby=symbol&match=CYP4B1) | aspartate transaminase activity;transaminase activity;catalytic activity;transferase activity | [124075](http://www.ncbi.nlm.nih.gov/entrez/dispomim.cgi?id=124075) | **1.87** |
| 1416203_AT | aquaporin 1 | [AQP1](http://www.gene.ucl.ac.uk/cgi-bin/nomenclature/searchgenes.pl?field=symbol&anchor=equals&symbol_search=Search&number=100&format=html&sortby=symbol&match=AQP1) | aspartic-type endopeptidase activity | [107776](http://www.ncbi.nlm.nih.gov/entrez/dispomim.cgi?id=107776) | **2.56** |
| 1416204_AT | glycerol-3-phosphate dehydrogenase 1 (soluble) | [GPD1](http://www.gene.ucl.ac.uk/cgi-bin/nomenclature/searchgenes.pl?field=symbol&anchor=equals&symbol_search=Search&number=100&format=html&sortby=symbol&match=GPD1) | aspartic-type endopeptidase activity;pepsin A activity;aspartic-type signal peptidase activity;hydrolase activity;peptidase activity | [138420](http://www.ncbi.nlm.nih.gov/entrez/dispomim.cgi?id=138420) | **1.76** |
| 1416209_AT | glutamate dehydrogenase 1 | [GLUD1](http://www.gene.ucl.ac.uk/cgi-bin/nomenclature/searchgenes.pl?field=symbol&anchor=equals&symbol_search=Search&number=100&format=html&sortby=symbol&match=GLUD1) | ATP binding | [138130](http://www.ncbi.nlm.nih.gov/entrez/dispomim.cgi?id=138130) | **1.23** |
| 1416219_AT | ribosomal protein L19 | [RPL19](http://www.gene.ucl.ac.uk/cgi-bin/nomenclature/searchgenes.pl?field=symbol&anchor=equals&symbol_search=Search&number=100&format=html&sortby=symbol&match=RPL19) | ATP binding | [180466](http://www.ncbi.nlm.nih.gov/entrez/dispomim.cgi?id=180466) | **-1.25** |
| 1416220_AT | RIKEN cDNA 1810004F21 gene | [1810004F21RIK](http://www.gene.ucl.ac.uk/cgi-bin/nomenclature/searchgenes.pl?field=symbol&anchor=equals&symbol_search=Search&number=100&format=html&sortby=symbol&match=1810004F21RIK) | ATP binding |  | **-1.33** |
| 1416221_AT | follistatin-like 1 | [FSTL1](http://www.gene.ucl.ac.uk/cgi-bin/nomenclature/searchgenes.pl?field=symbol&anchor=equals&symbol_search=Search&number=100&format=html&sortby=symbol&match=FSTL1) | ATP binding | [605547](http://www.ncbi.nlm.nih.gov/entrez/dispomim.cgi?id=605547) | **-1.75** |
| 1416226_AT | actin related protein 2/3 complex, subunit 1B | [ARPC1B](http://www.gene.ucl.ac.uk/cgi-bin/nomenclature/searchgenes.pl?field=symbol&anchor=equals&symbol_search=Search&number=100&format=html&sortby=symbol&match=ARPC1B) | ATP binding;tRNA ligase activity | [604223](http://www.ncbi.nlm.nih.gov/entrez/dispomim.cgi?id=604223) | **-2.24** |
| 1416229_AT | riboflavin kinase | [RFK](http://www.gene.ucl.ac.uk/cgi-bin/nomenclature/searchgenes.pl?field=symbol&anchor=equals&symbol_search=Search&number=100&format=html&sortby=symbol&match=RFK) | ATP binding;tRNA ligase activity |  | **1.29** |
| 1416230_AT | riboflavin kinase | [RFK](http://www.gene.ucl.ac.uk/cgi-bin/nomenclature/searchgenes.pl?field=symbol&anchor=equals&symbol_search=Search&number=100&format=html&sortby=symbol&match=RFK) | ATP binding;tRNA ligase activity |  | **1.31** |
| 1416234_AT | expressed sequence AA959742 | [AA959742](http://www.gene.ucl.ac.uk/cgi-bin/nomenclature/searchgenes.pl?field=symbol&anchor=equals&symbol_search=Search&number=100&format=html&sortby=symbol&match=AA959742) | ATP binding;tRNA ligase activity |  | **-1.24** |
| 1416238_AT | tyrosine kinase receptor 1 | [TIE1](http://www.gene.ucl.ac.uk/cgi-bin/nomenclature/searchgenes.pl?field=symbol&anchor=equals&symbol_search=Search&number=100&format=html&sortby=symbol&match=TIE1) | ATPase activity, coupled to transmembrane movement of ions, phosphorylative mechanism;magnesium ion binding;ATPase activity;ATP binding;catalytic activity;hydrolase activity;hydrolase activity, acting on acid anhydrides, catalyzing transmembrane movement of substances | [600222](http://www.ncbi.nlm.nih.gov/entrez/dispomim.cgi?id=600222) | **1.8** |
| 1416243_A_AT | ribosomal protein L35 | [RPL35](http://www.gene.ucl.ac.uk/cgi-bin/nomenclature/searchgenes.pl?field=symbol&anchor=equals&symbol_search=Search&number=100&format=html&sortby=symbol&match=RPL35) | ATPase activity, coupled to transmembrane movement of ions, phosphorylative mechanism;sodium/potassium-exchanging ATPase activity;magnesium ion binding;cation-transporting ATPase activity;catalytic activity;ATP binding;hydrolase activity, acting on acid anhydrides, catalyzing transmembrane movement of substances;hydrolase activity |  | **-1.4** |
| 1416246_A_AT | coronin, actin binding protein 1A | [CORO1A](http://www.gene.ucl.ac.uk/cgi-bin/nomenclature/searchgenes.pl?field=symbol&anchor=equals&symbol_search=Search&number=100&format=html&sortby=symbol&match=CORO1A) | ATPase activity, coupled to transmembrane movement of ions, phosphorylative mechanism;sodium/potassium-exchanging ATPase activity;magnesium ion binding;cation-transporting ATPase activity;catalytic activity;ATP binding;hydrolase activity;hydrolase activity, acting on acid anhydrides, catalyzing transmembrane movement of substances | [605000](http://www.ncbi.nlm.nih.gov/entrez/dispomim.cgi?id=605000) | **-3.05** |
| 1416250_AT | B-cell translocation gene 2, anti-proliferative | [BTG2](http://www.gene.ucl.ac.uk/cgi-bin/nomenclature/searchgenes.pl?field=symbol&anchor=equals&symbol_search=Search&number=100&format=html&sortby=symbol&match=BTG2) | ATPase activity, coupled to transmembrane movement of substances;ATP binding | [601597](http://www.ncbi.nlm.nih.gov/entrez/dispomim.cgi?id=601597) | **1.32** |
| 1416251_AT | minichromosome maintenance deficient 6 (MIS5 homolog, S. pombe) (S. cerevisiae) | [MCM6](http://www.gene.ucl.ac.uk/cgi-bin/nomenclature/searchgenes.pl?field=symbol&anchor=equals&symbol_search=Search&number=100&format=html&sortby=symbol&match=MCM6) | ATPase activity, coupled to transmembrane movement of substances;ATP binding | [601806](http://www.ncbi.nlm.nih.gov/entrez/dispomim.cgi?id=601806) | **-2.38** |
| 1416252_AT | serine/threonine kinase 38 | [STK38](http://www.gene.ucl.ac.uk/cgi-bin/nomenclature/searchgenes.pl?field=symbol&anchor=equals&symbol_search=Search&number=100&format=html&sortby=symbol&match=STK38) | ATPase activity;ATP-binding cassette (ABC) transporter activity;ATPase activity, coupled to transmembrane movement of substances;ATP binding;permease activity | [606964](http://www.ncbi.nlm.nih.gov/entrez/dispomim.cgi?id=606964) | **-1.45** |
| 1416253_AT | cyclin-dependent kinase inhibitor 2D (p19, inhibits CDK4) | [CDKN2D](http://www.gene.ucl.ac.uk/cgi-bin/nomenclature/searchgenes.pl?field=symbol&anchor=equals&symbol_search=Search&number=100&format=html&sortby=symbol&match=CDKN2D) | ATPase inhibitor activity;calcium channel regulator activity | [600927](http://www.ncbi.nlm.nih.gov/entrez/dispomim.cgi?id=600927) | **-1.43** |
| 1416255_AT | gap junction membrane channel protein alpha 4 | [GJA4](http://www.gene.ucl.ac.uk/cgi-bin/nomenclature/searchgenes.pl?field=symbol&anchor=equals&symbol_search=Search&number=100&format=html&sortby=symbol&match=GJA4) | ATP-binding cassette (ABC) transporter activity;ATPase activity, coupled to transmembrane movement of substances;ATP binding | [121012](http://www.ncbi.nlm.nih.gov/entrez/dispomim.cgi?id=121012) | **1.71** |
| 1416256_A_AT | tubulin, beta 5 | [TUBB5](http://www.gene.ucl.ac.uk/cgi-bin/nomenclature/searchgenes.pl?field=symbol&anchor=equals&symbol_search=Search&number=100&format=html&sortby=symbol&match=TUBB5) | ATP-binding cassette (ABC) transporter activity;ATPase activity, coupled to transmembrane movement of substances;ATP binding | [602662](http://www.ncbi.nlm.nih.gov/entrez/dispomim.cgi?id=602662) | **-1.96** |
| 1416257_AT | calpain 2 | [CAPN2](http://www.gene.ucl.ac.uk/cgi-bin/nomenclature/searchgenes.pl?field=symbol&anchor=equals&symbol_search=Search&number=100&format=html&sortby=symbol&match=CAPN2) | ATP-dependent helicase activity;DNA binding;RNA binding;ATP binding | [114230](http://www.ncbi.nlm.nih.gov/entrez/dispomim.cgi?id=114230) | **-1.28** |
| 1416260_A_AT | sorting nexin 1 | [SNX1](http://www.gene.ucl.ac.uk/cgi-bin/nomenclature/searchgenes.pl?field=symbol&anchor=equals&symbol_search=Search&number=100&format=html&sortby=symbol&match=SNX1) | ATP-dependent helicase activity;DNA binding;RNA binding;ATP binding | [601272](http://www.ncbi.nlm.nih.gov/entrez/dispomim.cgi?id=601272) | **-1.33** |
| 1416268_AT | E26 avian leukemia oncogene 2, 3' domain | [ETS2](http://www.gene.ucl.ac.uk/cgi-bin/nomenclature/searchgenes.pl?field=symbol&anchor=equals&symbol_search=Search&number=100&format=html&sortby=symbol&match=ETS2) | basic amino acid permease activity;amino acid-polyamine transporter activity;carrier activity | [164740](http://www.ncbi.nlm.nih.gov/entrez/dispomim.cgi?id=164740) | **1.35** |
| 1416278_A_AT | ATP synthase, H+ transporting, mitochondrial F1 complex, O subunit | [ATP5O](http://www.gene.ucl.ac.uk/cgi-bin/nomenclature/searchgenes.pl?field=symbol&anchor=equals&symbol_search=Search&number=100&format=html&sortby=symbol&match=ATP5O) | beta-catenin binding | [600828](http://www.ncbi.nlm.nih.gov/entrez/dispomim.cgi?id=600828) | **1.22** |
| 1416284_AT | mitochondrial ribosomal protein L28 | [MRPL28](http://www.gene.ucl.ac.uk/cgi-bin/nomenclature/searchgenes.pl?field=symbol&anchor=equals&symbol_search=Search&number=100&format=html&sortby=symbol&match=MRPL28) | beta-catenin binding | [604853](http://www.ncbi.nlm.nih.gov/entrez/dispomim.cgi?id=604853) | **1.31** |
| 1416285_AT | NADH dehydrogenase (ubiquinone) 1, subcomplex unknown, 1 | [NDUFC1](http://www.gene.ucl.ac.uk/cgi-bin/nomenclature/searchgenes.pl?field=symbol&anchor=equals&symbol_search=Search&number=100&format=html&sortby=symbol&match=NDUFC1) | beta-catenin binding;structural constituent of ribosome;transcription regulator activity | [603844](http://www.ncbi.nlm.nih.gov/entrez/dispomim.cgi?id=603844) | **1.33** |
| 1416286_AT | regulator of G-protein signaling 4 | [RGS4](http://www.gene.ucl.ac.uk/cgi-bin/nomenclature/searchgenes.pl?field=symbol&anchor=equals&symbol_search=Search&number=100&format=html&sortby=symbol&match=RGS4) | beta-catenin binding;structural constituent of ribosome;transcription regulator activity |  | **1.67** |
| 1416291_AT | proteasome (prosome, macropain) 26S subunit, ATPase, 4 | [PSMC4](http://www.gene.ucl.ac.uk/cgi-bin/nomenclature/searchgenes.pl?field=symbol&anchor=equals&symbol_search=Search&number=100&format=html&sortby=symbol&match=PSMC4) | beta-catenin binding;structural constituent of ribosome;transcription regulator activity | [602707](http://www.ncbi.nlm.nih.gov/entrez/dispomim.cgi?id=602707) | **-1.33** |
| 1416292_AT | gb:NM_007452.1 /DB_XREF=gi:6680689 /GEN=Prdx3 /FEA |  | beta-mannosidase activity;hydrolase activity, hydrolyzing O-glycosyl compounds |  | **1.34** |
| 1416300_A_AT | solute carrier family 25 (mitochondrial carrier, phosphate carrier), member 3 | [SLC25A3](http://www.gene.ucl.ac.uk/cgi-bin/nomenclature/searchgenes.pl?field=symbol&anchor=equals&symbol_search=Search&number=100&format=html&sortby=symbol&match=SLC25A3) | beta-N-acetylhexosaminidase activity;hydrolase activity, acting on glycosyl bonds;hydrolase activity | [600370](http://www.ncbi.nlm.nih.gov/entrez/dispomim.cgi?id=600370) | **1.24** |
| 1416303_AT | LPS-induced TN factor | [LITAF](http://www.gene.ucl.ac.uk/cgi-bin/nomenclature/searchgenes.pl?field=symbol&anchor=equals&symbol_search=Search&number=100&format=html&sortby=symbol&match=LITAF) | beta-N-acetylhexosaminidase activity;hydrolase activity, acting on glycosyl bonds;hydrolase activity | [603795](http://www.ncbi.nlm.nih.gov/entrez/dispomim.cgi?id=603795) | **-1.5** |
| 1416304_AT | LPS-induced TN factor | [LITAF](http://www.gene.ucl.ac.uk/cgi-bin/nomenclature/searchgenes.pl?field=symbol&anchor=equals&symbol_search=Search&number=100&format=html&sortby=symbol&match=LITAF) | binding | [603795](http://www.ncbi.nlm.nih.gov/entrez/dispomim.cgi?id=603795) | **-1.81** |
| 1416312_AT | arginyl-tRNA synthetase | [RARS](http://www.gene.ucl.ac.uk/cgi-bin/nomenclature/searchgenes.pl?field=symbol&anchor=equals&symbol_search=Search&number=100&format=html&sortby=symbol&match=RARS) | binding | [107820](http://www.ncbi.nlm.nih.gov/entrez/dispomim.cgi?id=107820) | **-1.27** |
| 1416313_AT | expressed sequence AI839562 | [AI839562](http://www.gene.ucl.ac.uk/cgi-bin/nomenclature/searchgenes.pl?field=symbol&anchor=equals&symbol_search=Search&number=100&format=html&sortby=symbol&match=AI839562) | binding |  | **-2.22** |
| 1416318_AT | gb:AF426024.1 /DB_XREF=gi:18034363 /GEN=Serpinb1 / |  | binding |  | **-5.55** |
| 1416319_AT | adenosine kinase | [ADK](http://www.gene.ucl.ac.uk/cgi-bin/nomenclature/searchgenes.pl?field=symbol&anchor=equals&symbol_search=Search&number=100&format=html&sortby=symbol&match=ADK) | binding | [102750](http://www.ncbi.nlm.nih.gov/entrez/dispomim.cgi?id=102750) | **2.02** |
| 1416328_A_AT | ATPase, H+ transporting, V0 subunit | [ATP6V0E](http://www.gene.ucl.ac.uk/cgi-bin/nomenclature/searchgenes.pl?field=symbol&anchor=equals&symbol_search=Search&number=100&format=html&sortby=symbol&match=ATP6V0E) | binding | [603931](http://www.ncbi.nlm.nih.gov/entrez/dispomim.cgi?id=603931) | **-1.64** |
| 1416330_AT | CD 81 antigen | [CD81](http://www.gene.ucl.ac.uk/cgi-bin/nomenclature/searchgenes.pl?field=symbol&anchor=equals&symbol_search=Search&number=100&format=html&sortby=symbol&match=CD81) | binding;iron ion transporter activity | [186845](http://www.ncbi.nlm.nih.gov/entrez/dispomim.cgi?id=186845) | **-1.22** |
| 1416336_S_AT | small nuclear ribonucleoprotein D1 | [SNRPD1](http://www.gene.ucl.ac.uk/cgi-bin/nomenclature/searchgenes.pl?field=symbol&anchor=equals&symbol_search=Search&number=100&format=html&sortby=symbol&match=SNRPD1) | binding;iron ion transporter activity | [601063](http://www.ncbi.nlm.nih.gov/entrez/dispomim.cgi?id=601063) | **-1.26** |
| 1416337_AT | ubiquinol-cytochrome c reductase binding protein | [UQCRB](http://www.gene.ucl.ac.uk/cgi-bin/nomenclature/searchgenes.pl?field=symbol&anchor=equals&symbol_search=Search&number=100&format=html&sortby=symbol&match=UQCRB) | biotin binding;ligase activity;methylcrotonoyl-CoA carboxylase activity;ATP binding | [191330](http://www.ncbi.nlm.nih.gov/entrez/dispomim.cgi?id=191330) | **1.25** |
| 1416341_AT | polymerase (RNA) II (DNA directed) polypeptide C | [POLR2C](http://www.gene.ucl.ac.uk/cgi-bin/nomenclature/searchgenes.pl?field=symbol&anchor=equals&symbol_search=Search&number=100&format=html&sortby=symbol&match=POLR2C) | biotin carboxylase activity;acetyl-CoA carboxylase activity | [180663](http://www.ncbi.nlm.nih.gov/entrez/dispomim.cgi?id=180663) | **1.24** |
| 1416342_AT | tenascin C | [TNC](http://www.gene.ucl.ac.uk/cgi-bin/nomenclature/searchgenes.pl?field=symbol&anchor=equals&symbol_search=Search&number=100&format=html&sortby=symbol&match=TNC) | bisphosphoglycerate mutase activity;intramolecular transferase activity, phosphotransferases;isomerase activity;bisphosphoglycerate phosphatase activity;catalytic activity;hydrolase activity;phosphoglycerate mutase activity | [187380](http://www.ncbi.nlm.nih.gov/entrez/dispomim.cgi?id=187380) | **-23.15** |
| 1416343_A_AT | lysosomal membrane glycoprotein 2 | [LAMP2](http://www.gene.ucl.ac.uk/cgi-bin/nomenclature/searchgenes.pl?field=symbol&anchor=equals&symbol_search=Search&number=100&format=html&sortby=symbol&match=LAMP2) | bisphosphoglycerate mutase activity;intramolecular transferase activity, phosphotransferases;isomerase activity;bisphosphoglycerate phosphatase activity;catalytic activity;hydrolase activity;phosphoglycerate mutase activity | [309060](http://www.ncbi.nlm.nih.gov/entrez/dispomim.cgi?id=309060) | **-1.36** |
| 1416344_AT | lysosomal membrane glycoprotein 2 | [LAMP2](http://www.gene.ucl.ac.uk/cgi-bin/nomenclature/searchgenes.pl?field=symbol&anchor=equals&symbol_search=Search&number=100&format=html&sortby=symbol&match=LAMP2) | bisphosphoglycerate mutase activity;intramolecular transferase activity, phosphotransferases;isomerase activity;bisphosphoglycerate phosphatase activity;catalytic activity;hydrolase activity;phosphoglycerate mutase activity | [309060](http://www.ncbi.nlm.nih.gov/entrez/dispomim.cgi?id=309060) | **-1.85** |
| 1416349_AT | mitochondrial ribosomal protein L34 | [MRPL34](http://www.gene.ucl.ac.uk/cgi-bin/nomenclature/searchgenes.pl?field=symbol&anchor=equals&symbol_search=Search&number=100&format=html&sortby=symbol&match=MRPL34) | bleomycin hydrolase activity;cysteine-type endopeptidase activity;hydrolase activity;cysteine-type peptidase activity |  | **1.43** |
| 1416352_S_AT | glycoprotein, synaptic 2 | [GPSN2](http://www.gene.ucl.ac.uk/cgi-bin/nomenclature/searchgenes.pl?field=symbol&anchor=equals&symbol_search=Search&number=100&format=html&sortby=symbol&match=GPSN2) | bleomycin hydrolase activity;cysteine-type endopeptidase activity;hydrolase activity;cysteine-type peptidase activity |  | **1.38** |
| 1416362_A_AT | FK506 binding protein 4 | [FKBP4](http://www.gene.ucl.ac.uk/cgi-bin/nomenclature/searchgenes.pl?field=symbol&anchor=equals&symbol_search=Search&number=100&format=html&sortby=symbol&match=FKBP4) | branched-chain-amino-acid transaminase activity;transaminase activity;catalytic activity;transferase activity | [600611](http://www.ncbi.nlm.nih.gov/entrez/dispomim.cgi?id=600611) | **1.43** |
| 1416366_AT | gb:NM_024220.1 /DB_XREF=gi:18859596 /GEN=1810004I0 |  | C-8 sterol isomerase activity;isomerase activity;cholestenol delta-isomerase activity |  | **1.22** |
| 1416367_AT | RIKEN cDNA 1110001J03 gene | [1110001J03RIK](http://www.gene.ucl.ac.uk/cgi-bin/nomenclature/searchgenes.pl?field=symbol&anchor=equals&symbol_search=Search&number=100&format=html&sortby=symbol&match=1110001J03RIK) | calcium channel activity;cation channel activity;inositol 1,4,5-triphosphate-sensitive calcium-release channel activity;protein binding;inositol-1,4,5-triphosphate receptor activity;ion channel activity;receptor activity |  | **1.4** |
| 1416368_AT | glutathione S-transferase, alpha 4 | [GSTA4](http://www.gene.ucl.ac.uk/cgi-bin/nomenclature/searchgenes.pl?field=symbol&anchor=equals&symbol_search=Search&number=100&format=html&sortby=symbol&match=GSTA4) | calcium channel activity;cation channel activity;ion channel activity;voltage-gated ion channel activity;calcium ion binding;voltage-gated calcium channel activity | [605450](http://www.ncbi.nlm.nih.gov/entrez/dispomim.cgi?id=605450) | **2.45** |
| 1416373_AT | nitrogen fixation gene 1 (S. cerevisiae) | [NFS1](http://www.gene.ucl.ac.uk/cgi-bin/nomenclature/searchgenes.pl?field=symbol&anchor=equals&symbol_search=Search&number=100&format=html&sortby=symbol&match=NFS1) | calcium channel activity;protein binding;ion channel activity;receptor activity | [603485](http://www.ncbi.nlm.nih.gov/entrez/dispomim.cgi?id=603485) | **1.26** |
| 1416379_AT | pannexin 1 | [PANX1](http://www.gene.ucl.ac.uk/cgi-bin/nomenclature/searchgenes.pl?field=symbol&anchor=equals&symbol_search=Search&number=100&format=html&sortby=symbol&match=PANX1) | calcium channel activity;voltage-gated chloride channel activity;voltage-gated ion channel activity;ion channel activity | [608420](http://www.ncbi.nlm.nih.gov/entrez/dispomim.cgi?id=608420) | **-3.07** |
| 1416381_A_AT | peroxiredoxin 5 | [PRDX5](http://www.gene.ucl.ac.uk/cgi-bin/nomenclature/searchgenes.pl?field=symbol&anchor=equals&symbol_search=Search&number=100&format=html&sortby=symbol&match=PRDX5) | calcium channel activity;voltage-gated ion channel activity;ion channel activity | [606583](http://www.ncbi.nlm.nih.gov/entrez/dispomim.cgi?id=606583) | **1.33** |
| 1416382_AT | cathepsin C | [CTSC](http://www.gene.ucl.ac.uk/cgi-bin/nomenclature/searchgenes.pl?field=symbol&anchor=equals&symbol_search=Search&number=100&format=html&sortby=symbol&match=CTSC) | calcium ion binding | [602365](http://www.ncbi.nlm.nih.gov/entrez/dispomim.cgi?id=602365) | **-2.35** |
| 1416383_A_AT | pyruvate carboxylase | [PCX](http://www.gene.ucl.ac.uk/cgi-bin/nomenclature/searchgenes.pl?field=symbol&anchor=equals&symbol_search=Search&number=100&format=html&sortby=symbol&match=PCX) | calcium ion binding |  | **1.74** |
| 1416385_A_AT | mannose-6-phosphate receptor, cation dependent | [M6PR](http://www.gene.ucl.ac.uk/cgi-bin/nomenclature/searchgenes.pl?field=symbol&anchor=equals&symbol_search=Search&number=100&format=html&sortby=symbol&match=M6PR) | calcium ion binding | [154540](http://www.ncbi.nlm.nih.gov/entrez/dispomim.cgi?id=154540) | **-1.21** |
| 1416399_A_AT | heme oxygenase (decycling) 2 | [HMOX2](http://www.gene.ucl.ac.uk/cgi-bin/nomenclature/searchgenes.pl?field=symbol&anchor=equals&symbol_search=Search&number=100&format=html&sortby=symbol&match=HMOX2) | calcium ion binding | [141251](http://www.ncbi.nlm.nih.gov/entrez/dispomim.cgi?id=141251) | **1.25** |
| 1416401_AT | kangai 1 (suppression of tumorigenicity 6, prostate) | [KAI1](http://www.gene.ucl.ac.uk/cgi-bin/nomenclature/searchgenes.pl?field=symbol&anchor=equals&symbol_search=Search&number=100&format=html&sortby=symbol&match=KAI1) | calcium ion binding | [600623](http://www.ncbi.nlm.nih.gov/entrez/dispomim.cgi?id=600623) | **-1.51** |
| 1416402_AT | ATP-binding cassette, sub-family B (MDR/TAP), member 10 | [ABCB10](http://www.gene.ucl.ac.uk/cgi-bin/nomenclature/searchgenes.pl?field=symbol&anchor=equals&symbol_search=Search&number=100&format=html&sortby=symbol&match=ABCB10) | calcium ion binding | [605454](http://www.ncbi.nlm.nih.gov/entrez/dispomim.cgi?id=605454) | **1.21** |
| 1416403_AT | ATP-binding cassette, sub-family B (MDR/TAP), member 10 | [ABCB10](http://www.gene.ucl.ac.uk/cgi-bin/nomenclature/searchgenes.pl?field=symbol&anchor=equals&symbol_search=Search&number=100&format=html&sortby=symbol&match=ABCB10) | calcium ion binding | [605454](http://www.ncbi.nlm.nih.gov/entrez/dispomim.cgi?id=605454) | **1.42** |
| 1416404_S_AT | ribosomal protein S16 | [RPS16](http://www.gene.ucl.ac.uk/cgi-bin/nomenclature/searchgenes.pl?field=symbol&anchor=equals&symbol_search=Search&number=100&format=html&sortby=symbol&match=RPS16) | calcium ion binding | [603675](http://www.ncbi.nlm.nih.gov/entrez/dispomim.cgi?id=603675) | **-1.29** |
| 1416405_AT | biglycan | [BGN](http://www.gene.ucl.ac.uk/cgi-bin/nomenclature/searchgenes.pl?field=symbol&anchor=equals&symbol_search=Search&number=100&format=html&sortby=symbol&match=BGN) | calcium ion binding | [301870](http://www.ncbi.nlm.nih.gov/entrez/dispomim.cgi?id=301870) | **-2.02** |
| 1416408_AT | acyl-Coenzyme A oxidase 1, palmitoyl | [ACOX1](http://www.gene.ucl.ac.uk/cgi-bin/nomenclature/searchgenes.pl?field=symbol&anchor=equals&symbol_search=Search&number=100&format=html&sortby=symbol&match=ACOX1) | calcium ion binding | [264470](http://www.ncbi.nlm.nih.gov/entrez/dispomim.cgi?id=264470) | **1.72** |
| 1416409_AT | acyl-Coenzyme A oxidase 1, palmitoyl | [ACOX1](http://www.gene.ucl.ac.uk/cgi-bin/nomenclature/searchgenes.pl?field=symbol&anchor=equals&symbol_search=Search&number=100&format=html&sortby=symbol&match=ACOX1) | calcium ion binding | [264470](http://www.ncbi.nlm.nih.gov/entrez/dispomim.cgi?id=264470) | **1.51** |
| 1416411_AT | glutathione S-transferase, mu 2 | [GSTM2](http://www.gene.ucl.ac.uk/cgi-bin/nomenclature/searchgenes.pl?field=symbol&anchor=equals&symbol_search=Search&number=100&format=html&sortby=symbol&match=GSTM2) | calcium ion binding |  | **2.2** |
| 1416416_X_AT | glutathione S-transferase, mu 1 | [GSTM1](http://www.gene.ucl.ac.uk/cgi-bin/nomenclature/searchgenes.pl?field=symbol&anchor=equals&symbol_search=Search&number=100&format=html&sortby=symbol&match=GSTM1) | calcium ion binding |  | **2.07** |
| 1416424_AT | mannose-6-phosphate receptor binding protein 1 | [M6PRBP1](http://www.gene.ucl.ac.uk/cgi-bin/nomenclature/searchgenes.pl?field=symbol&anchor=equals&symbol_search=Search&number=100&format=html&sortby=symbol&match=M6PRBP1) | calcium ion binding | [602702](http://www.ncbi.nlm.nih.gov/entrez/dispomim.cgi?id=602702) | **1.74** |
| 1416425_AT | peroxisome biogenesis factor 19 | [PEX19](http://www.gene.ucl.ac.uk/cgi-bin/nomenclature/searchgenes.pl?field=symbol&anchor=equals&symbol_search=Search&number=100&format=html&sortby=symbol&match=PEX19) | calcium ion binding | [600279](http://www.ncbi.nlm.nih.gov/entrez/dispomim.cgi?id=600279) | **1.29** |
| 1416426_AT | RAB5A, member RAS oncogene family | [RAB5A](http://www.gene.ucl.ac.uk/cgi-bin/nomenclature/searchgenes.pl?field=symbol&anchor=equals&symbol_search=Search&number=100&format=html&sortby=symbol&match=RAB5A) | calcium ion binding | [179512](http://www.ncbi.nlm.nih.gov/entrez/dispomim.cgi?id=179512) | **-1.24** |
| 1416429_A_AT | catalase | [CAT](http://www.gene.ucl.ac.uk/cgi-bin/nomenclature/searchgenes.pl?field=symbol&anchor=equals&symbol_search=Search&number=100&format=html&sortby=symbol&match=CAT) | calcium ion binding | [115500](http://www.ncbi.nlm.nih.gov/entrez/dispomim.cgi?id=115500) | **1.46** |
| 1416430_AT | catalase | [CAT](http://www.gene.ucl.ac.uk/cgi-bin/nomenclature/searchgenes.pl?field=symbol&anchor=equals&symbol_search=Search&number=100&format=html&sortby=symbol&match=CAT) | calcium ion binding | [115500](http://www.ncbi.nlm.nih.gov/entrez/dispomim.cgi?id=115500) | **1.85** |
| 1416431_AT | RIKEN cDNA 2310057H16 gene | [TUBB6](http://www.gene.ucl.ac.uk/cgi-bin/nomenclature/searchgenes.pl?field=symbol&anchor=equals&symbol_search=Search&number=100&format=html&sortby=symbol&match=TUBB6) | calcium ion binding |  | **-8.13** |
| 1416436_A_AT | RIKEN cDNA 2410003P15 gene | [2410003P15RIK](http://www.gene.ucl.ac.uk/cgi-bin/nomenclature/searchgenes.pl?field=symbol&anchor=equals&symbol_search=Search&number=100&format=html&sortby=symbol&match=2410003P15RIK) | calcium ion binding |  | **1.75** |
| 1416440_AT | CD164 antigen | [CD164](http://www.gene.ucl.ac.uk/cgi-bin/nomenclature/searchgenes.pl?field=symbol&anchor=equals&symbol_search=Search&number=100&format=html&sortby=symbol&match=CD164) | calcium ion binding | [603356](http://www.ncbi.nlm.nih.gov/entrez/dispomim.cgi?id=603356) | **-1.3** |
| 1416443_A_AT | ubiquitin-like 1 (sentrin) activating enzyme E1A | [UBLE1A](http://www.gene.ucl.ac.uk/cgi-bin/nomenclature/searchgenes.pl?field=symbol&anchor=equals&symbol_search=Search&number=100&format=html&sortby=symbol&match=UBLE1A) | calcium ion binding |  | **-1.3** |
| 1416452_AT | ornithine aminotransferase | [OAT](http://www.gene.ucl.ac.uk/cgi-bin/nomenclature/searchgenes.pl?field=symbol&anchor=equals&symbol_search=Search&number=100&format=html&sortby=symbol&match=OAT) | calcium ion binding | [258870](http://www.ncbi.nlm.nih.gov/entrez/dispomim.cgi?id=258870) | **1.42** |
| 1416453_X_AT | ribosomal protein S12 | [RPS12](http://www.gene.ucl.ac.uk/cgi-bin/nomenclature/searchgenes.pl?field=symbol&anchor=equals&symbol_search=Search&number=100&format=html&sortby=symbol&match=RPS12) | calcium ion binding | [603660](http://www.ncbi.nlm.nih.gov/entrez/dispomim.cgi?id=603660) | **-1.56** |
| 1416454_S_AT | actin, alpha 2, smooth muscle, aorta | [ACTA2](http://www.gene.ucl.ac.uk/cgi-bin/nomenclature/searchgenes.pl?field=symbol&anchor=equals&symbol_search=Search&number=100&format=html&sortby=symbol&match=ACTA2) | calcium ion binding | [102620](http://www.ncbi.nlm.nih.gov/entrez/dispomim.cgi?id=102620) | **-1.74** |
| 1416455_A_AT | crystallin, alpha B | [CRYAB](http://www.gene.ucl.ac.uk/cgi-bin/nomenclature/searchgenes.pl?field=symbol&anchor=equals&symbol_search=Search&number=100&format=html&sortby=symbol&match=CRYAB) | calcium ion binding | [123590](http://www.ncbi.nlm.nih.gov/entrez/dispomim.cgi?id=123590) | **1.21** |
| 1416461_AT | GPI-anchored membrane protein 1 | [GPIAP1](http://www.gene.ucl.ac.uk/cgi-bin/nomenclature/searchgenes.pl?field=symbol&anchor=equals&symbol_search=Search&number=100&format=html&sortby=symbol&match=GPIAP1) | calcium ion binding |  | **-1.27** |
| 1416465_A_AT | vesicle-associated membrane protein, associated protein A | [VAPA](http://www.gene.ucl.ac.uk/cgi-bin/nomenclature/searchgenes.pl?field=symbol&anchor=equals&symbol_search=Search&number=100&format=html&sortby=symbol&match=VAPA) | calcium ion binding | [605703](http://www.ncbi.nlm.nih.gov/entrez/dispomim.cgi?id=605703) | **-1.25** |
| 1416468_AT | aldehyde dehydrogenase family 1, subfamily A1 | [ALDH1A1](http://www.gene.ucl.ac.uk/cgi-bin/nomenclature/searchgenes.pl?field=symbol&anchor=equals&symbol_search=Search&number=100&format=html&sortby=symbol&match=ALDH1A1) | calcium ion binding | [100640](http://www.ncbi.nlm.nih.gov/entrez/dispomim.cgi?id=100640) | **1.57** |
| 1416470_A_AT | ribophorin I | [RPN1](http://www.gene.ucl.ac.uk/cgi-bin/nomenclature/searchgenes.pl?field=symbol&anchor=equals&symbol_search=Search&number=100&format=html&sortby=symbol&match=RPN1) | calcium ion binding | [180470](http://www.ncbi.nlm.nih.gov/entrez/dispomim.cgi?id=180470) | **-1.43** |
| 1416473_A_AT | gb:NM_020043.1 /DB_XREF=gi:9910463 /GEN=Nope /FEA= |  | calcium ion binding |  | **-1.88** |
| 1416474_AT | neighbor of Punc E11 | [NOPE](http://www.gene.ucl.ac.uk/cgi-bin/nomenclature/searchgenes.pl?field=symbol&anchor=equals&symbol_search=Search&number=100&format=html&sortby=symbol&match=NOPE) | calcium ion binding |  | **-1.85** |
| 1416478_A_AT | malate dehydrogenase 2, NAD (mitochondrial) | [MDH2](http://www.gene.ucl.ac.uk/cgi-bin/nomenclature/searchgenes.pl?field=symbol&anchor=equals&symbol_search=Search&number=100&format=html&sortby=symbol&match=MDH2) | calcium ion binding | [154100](http://www.ncbi.nlm.nih.gov/entrez/dispomim.cgi?id=154100) | **1.25** |
| 1416483_AT | tetratricopeptide repeat domain 3 | [TTC3](http://www.gene.ucl.ac.uk/cgi-bin/nomenclature/searchgenes.pl?field=symbol&anchor=equals&symbol_search=Search&number=100&format=html&sortby=symbol&match=TTC3) | calcium ion binding | [602259](http://www.ncbi.nlm.nih.gov/entrez/dispomim.cgi?id=602259) | **-1.2** |
| 1416485_AT | translocase of inner mitochondrial membrane 23 homolog (yeast) | [MGC46821](http://www.gene.ucl.ac.uk/cgi-bin/nomenclature/searchgenes.pl?field=symbol&anchor=equals&symbol_search=Search&number=100&format=html&sortby=symbol&match=MGC46821) | calcium ion binding | [605034](http://www.ncbi.nlm.nih.gov/entrez/dispomim.cgi?id=605034) | **1.25** |
| 1416493_AT | dolichyl-di-phosphooligosaccharide-protein glycotransferase | [DDOST](http://www.gene.ucl.ac.uk/cgi-bin/nomenclature/searchgenes.pl?field=symbol&anchor=equals&symbol_search=Search&number=100&format=html&sortby=symbol&match=DDOST) | calcium ion binding | [602202](http://www.ncbi.nlm.nih.gov/entrez/dispomim.cgi?id=602202) | **-1.37** |
| 1416494_AT | gb:NM_134104.1 /DB_XREF=gi:19527333 /GEN=AI256693 |  | calcium ion binding |  | **1.23** |
| 1416497_AT | calcium binding protein, intestinal | [CAI](http://www.gene.ucl.ac.uk/cgi-bin/nomenclature/searchgenes.pl?field=symbol&anchor=equals&symbol_search=Search&number=100&format=html&sortby=symbol&match=CAI) | calcium ion binding |  | **-1.56** |
| 1416498_AT | peptidylprolyl isomerase C | [PPIC](http://www.gene.ucl.ac.uk/cgi-bin/nomenclature/searchgenes.pl?field=symbol&anchor=equals&symbol_search=Search&number=100&format=html&sortby=symbol&match=PPIC) | calcium ion binding | [123842](http://www.ncbi.nlm.nih.gov/entrez/dispomim.cgi?id=123842) | **-1.83** |
| 1416503_AT | latexin | [LXN](http://www.gene.ucl.ac.uk/cgi-bin/nomenclature/searchgenes.pl?field=symbol&anchor=equals&symbol_search=Search&number=100&format=html&sortby=symbol&match=LXN) | calcium ion binding |  | **-1.47** |
| 1416504_AT | Unc-51 like kinase 1 (C. elegans) | [ULK1](http://www.gene.ucl.ac.uk/cgi-bin/nomenclature/searchgenes.pl?field=symbol&anchor=equals&symbol_search=Search&number=100&format=html&sortby=symbol&match=ULK1) | calcium ion binding;acylphosphatase activity;hydrolase activity | [603168](http://www.ncbi.nlm.nih.gov/entrez/dispomim.cgi?id=603168) | **1.79** |
| 1416510_AT | mitochondrial ribosomal protein L4 | [MRPL4](http://www.gene.ucl.ac.uk/cgi-bin/nomenclature/searchgenes.pl?field=symbol&anchor=equals&symbol_search=Search&number=100&format=html&sortby=symbol&match=MRPL4) | calcium ion binding;binding |  | **1.24** |
| 1416514_A_AT | fascin homolog 1, actin bundling protein (Strongylocentrotus) purpuratus) | [FSCN1](http://www.gene.ucl.ac.uk/cgi-bin/nomenclature/searchgenes.pl?field=symbol&anchor=equals&symbol_search=Search&number=100&format=html&sortby=symbol&match=FSCN1) | calcium ion binding;binding | [602689](http://www.ncbi.nlm.nih.gov/entrez/dispomim.cgi?id=602689) | **-1.98** |
| 1416521_AT | selenoprotein W, muscle 1 | [SEPW1](http://www.gene.ucl.ac.uk/cgi-bin/nomenclature/searchgenes.pl?field=symbol&anchor=equals&symbol_search=Search&number=100&format=html&sortby=symbol&match=SEPW1) | calcium ion binding;cysteine-type endopeptidase activity;hydrolase activity, acting on glycosyl bonds;hydrolase activity, hydrolyzing O-glycosyl compounds;hydrolase activity;calpain activity;peptidase activity;cysteine-type peptidase activity | [603235](http://www.ncbi.nlm.nih.gov/entrez/dispomim.cgi?id=603235) | **1.43** |
| 1416525_AT | speckle-type POZ protein | [SPOP](http://www.gene.ucl.ac.uk/cgi-bin/nomenclature/searchgenes.pl?field=symbol&anchor=equals&symbol_search=Search&number=100&format=html&sortby=symbol&match=SPOP) | calcium ion binding;cysteine-type endopeptidase activity;hydrolase activity, acting on glycosyl bonds;hydrolase activity, hydrolyzing O-glycosyl compounds;hydrolase activity;peptidase activity;cysteine-type peptidase activity;calpain activity | [602650](http://www.ncbi.nlm.nih.gov/entrez/dispomim.cgi?id=602650) | **1.27** |
| 1416528_AT | SH3 domain binding glutamic acid-rich protein-like 3 | [SH3BGRL3](http://www.gene.ucl.ac.uk/cgi-bin/nomenclature/searchgenes.pl?field=symbol&anchor=equals&symbol_search=Search&number=100&format=html&sortby=symbol&match=SH3BGRL3) | calcium ion binding;cysteine-type endopeptidase activity;hydrolase activity;calpain activity;cysteine-type peptidase activity;peptidase activity |  | **-1.42** |
| 1416529_AT | epithelial membrane protein 1 | [EMP1](http://www.gene.ucl.ac.uk/cgi-bin/nomenclature/searchgenes.pl?field=symbol&anchor=equals&symbol_search=Search&number=100&format=html&sortby=symbol&match=EMP1) | calcium ion binding;cytokine activity;phospholipase A2 activity | [602333](http://www.ncbi.nlm.nih.gov/entrez/dispomim.cgi?id=602333) | **-2.02** |
| 1416532_AT | expressed sequence AI481500 | [TRRAP](http://www.gene.ucl.ac.uk/cgi-bin/nomenclature/searchgenes.pl?field=symbol&anchor=equals&symbol_search=Search&number=100&format=html&sortby=symbol&match=TRRAP) | calcium ion binding;diacylglycerol binding |  | **-1.29** |
| 1416541_AT | suppressor of K+ transport defect 3 | [SKD3](http://www.gene.ucl.ac.uk/cgi-bin/nomenclature/searchgenes.pl?field=symbol&anchor=equals&symbol_search=Search&number=100&format=html&sortby=symbol&match=SKD3) | calcium ion binding;electron transporter activity;nitric-oxide synthase activity;oxidoreductase activity;calmodulin binding;FMN binding |  | **1.71** |
| 1416544_AT | enhancer of zeste homolog 2 (Drosophila) | [EZH2](http://www.gene.ucl.ac.uk/cgi-bin/nomenclature/searchgenes.pl?field=symbol&anchor=equals&symbol_search=Search&number=100&format=html&sortby=symbol&match=EZH2) | calcium ion binding;glycerol-3-phosphate dehydrogenase activity;oxidoreductase activity | [601573](http://www.ncbi.nlm.nih.gov/entrez/dispomim.cgi?id=601573) | **-1.41** |
| 1416546_A_AT | ribosomal protein L6 | [RPL6](http://www.gene.ucl.ac.uk/cgi-bin/nomenclature/searchgenes.pl?field=symbol&anchor=equals&symbol_search=Search&number=100&format=html&sortby=symbol&match=RPL6) | calcium ion binding;G-protein coupled receptor activity | [603703](http://www.ncbi.nlm.nih.gov/entrez/dispomim.cgi?id=603703) | **-1.28** |
| 1416547_AT | NADH dehydrogenase (ubiquinone) 1 beta subcomplex 3 | [NDUFB3](http://www.gene.ucl.ac.uk/cgi-bin/nomenclature/searchgenes.pl?field=symbol&anchor=equals&symbol_search=Search&number=100&format=html&sortby=symbol&match=NDUFB3) | calcium ion binding;G-protein coupled receptor activity | [603839](http://www.ncbi.nlm.nih.gov/entrez/dispomim.cgi?id=603839) | **1.25** |
| 1416556_AT | sarcoma amplified sequence | [SAS](http://www.gene.ucl.ac.uk/cgi-bin/nomenclature/searchgenes.pl?field=symbol&anchor=equals&symbol_search=Search&number=100&format=html&sortby=symbol&match=SAS) | calcium ion binding;G-protein coupled receptor activity | [181035](http://www.ncbi.nlm.nih.gov/entrez/dispomim.cgi?id=181035) | **-1.39** |
| 1416563_AT | cytidine 5'-triphosphate synthase | [CTPS](http://www.gene.ucl.ac.uk/cgi-bin/nomenclature/searchgenes.pl?field=symbol&anchor=equals&symbol_search=Search&number=100&format=html&sortby=symbol&match=CTPS) | calcium ion binding;growth factor activity | [123860](http://www.ncbi.nlm.nih.gov/entrez/dispomim.cgi?id=123860) | **-1.55** |
| 1416564_AT | SRY-box containing gene 7 | [SOX7](http://www.gene.ucl.ac.uk/cgi-bin/nomenclature/searchgenes.pl?field=symbol&anchor=equals&symbol_search=Search&number=100&format=html&sortby=symbol&match=SOX7) | calcium ion binding;GTP binding |  | **2.84** |
| 1416567_S_AT | ATP synthase, H+ transporting, mitochondrial F1 complex, epsilon subunit | [ATP5E](http://www.gene.ucl.ac.uk/cgi-bin/nomenclature/searchgenes.pl?field=symbol&anchor=equals&symbol_search=Search&number=100&format=html&sortby=symbol&match=ATP5E) | calcium ion binding;GTPase activity;GTP binding;ATP binding | [606153](http://www.ncbi.nlm.nih.gov/entrez/dispomim.cgi?id=606153) | **1.21** |
| 1416570_S_AT | G elongation factor | [GFM1](http://www.gene.ucl.ac.uk/cgi-bin/nomenclature/searchgenes.pl?field=symbol&anchor=equals&symbol_search=Search&number=100&format=html&sortby=symbol&match=GFM1) | calcium ion binding;heparin binding | [606639](http://www.ncbi.nlm.nih.gov/entrez/dispomim.cgi?id=606639) | **1.36** |
| 1416572_AT | matrix metalloproteinase 14 (membrane-inserted) | [MMP14](http://www.gene.ucl.ac.uk/cgi-bin/nomenclature/searchgenes.pl?field=symbol&anchor=equals&symbol_search=Search&number=100&format=html&sortby=symbol&match=MMP14) | calcium ion binding;heparin binding | [600754](http://www.ncbi.nlm.nih.gov/entrez/dispomim.cgi?id=600754) | **-2.05** |
| 1416576_AT | suppressor of cytokine signaling 3 | [SOCS3](http://www.gene.ucl.ac.uk/cgi-bin/nomenclature/searchgenes.pl?field=symbol&anchor=equals&symbol_search=Search&number=100&format=html&sortby=symbol&match=SOCS3) | calcium ion binding;integrin binding | [604176](http://www.ncbi.nlm.nih.gov/entrez/dispomim.cgi?id=604176) | **-2.63** |
| 1416589_AT | secreted acidic cysteine rich glycoprotein | [SPARC](http://www.gene.ucl.ac.uk/cgi-bin/nomenclature/searchgenes.pl?field=symbol&anchor=equals&symbol_search=Search&number=100&format=html&sortby=symbol&match=SPARC) | calcium ion binding;phospholipase A2 activity;cytokine activity | [182120](http://www.ncbi.nlm.nih.gov/entrez/dispomim.cgi?id=182120) | **-1.86** |
| 1416590_A_AT | RAB34, member of RAS oncogene family | [RAB34](http://www.gene.ucl.ac.uk/cgi-bin/nomenclature/searchgenes.pl?field=symbol&anchor=equals&symbol_search=Search&number=100&format=html&sortby=symbol&match=RAB34) | calcium ion binding;phospholipase A2 activity;cytokine activity |  | **-1.65** |
| 1416591_AT | RAB34, member of RAS oncogene family | [RAB34](http://www.gene.ucl.ac.uk/cgi-bin/nomenclature/searchgenes.pl?field=symbol&anchor=equals&symbol_search=Search&number=100&format=html&sortby=symbol&match=RAB34) | calcium ion binding;phospholipase A2 activity;hydrolase activity |  | **-1.43** |
| 1416603_AT | ribosomal protein L22 | [RPL22](http://www.gene.ucl.ac.uk/cgi-bin/nomenclature/searchgenes.pl?field=symbol&anchor=equals&symbol_search=Search&number=100&format=html&sortby=symbol&match=RPL22) | calcium ion binding;protein-arginine deiminase activity;hydrolase activity | [180474](http://www.ncbi.nlm.nih.gov/entrez/dispomim.cgi?id=180474) | **-1.82** |
| 1416604_AT | cytochrome c-1 | [CYC1](http://www.gene.ucl.ac.uk/cgi-bin/nomenclature/searchgenes.pl?field=symbol&anchor=equals&symbol_search=Search&number=100&format=html&sortby=symbol&match=CYC1) | calcium ion binding;serine-type endopeptidase inhibitor activity | [123980](http://www.ncbi.nlm.nih.gov/entrez/dispomim.cgi?id=123980) | **1.27** |
| 1416609_AT | cDNA sequence BC004004 | [BC004004](http://www.gene.ucl.ac.uk/cgi-bin/nomenclature/searchgenes.pl?field=symbol&anchor=equals&symbol_search=Search&number=100&format=html&sortby=symbol&match=BC004004) | calcium ion binding;structural constituent of muscle |  | **1.27** |
| 1416610_A_AT | chloride channel 3 | [CLCN3](http://www.gene.ucl.ac.uk/cgi-bin/nomenclature/searchgenes.pl?field=symbol&anchor=equals&symbol_search=Search&number=100&format=html&sortby=symbol&match=CLCN3) | calcium-dependent phospholipid binding;calcium ion binding | [600580](http://www.ncbi.nlm.nih.gov/entrez/dispomim.cgi?id=600580) | **1.77** |
| 1416615_AT | caseinolytic protease, ATP-dependent, proteolytic subunit homolog (E. coli) | [CLPP](http://www.gene.ucl.ac.uk/cgi-bin/nomenclature/searchgenes.pl?field=symbol&anchor=equals&symbol_search=Search&number=100&format=html&sortby=symbol&match=CLPP) | calcium-dependent phospholipid binding;calcium ion binding | [601119](http://www.ncbi.nlm.nih.gov/entrez/dispomim.cgi?id=601119) | **1.34** |
| 1416617_AT | acetyl-Coenzyme A synthetase 2 (AMP forming)-like | [ACAS2L](http://www.gene.ucl.ac.uk/cgi-bin/nomenclature/searchgenes.pl?field=symbol&anchor=equals&symbol_search=Search&number=100&format=html&sortby=symbol&match=ACAS2L) | calcium-dependent phospholipid binding;protein binding;calcium ion binding |  | **2.24** |
| 1416623_AT | thrombospondin 3 | [THBS3](http://www.gene.ucl.ac.uk/cgi-bin/nomenclature/searchgenes.pl?field=symbol&anchor=equals&symbol_search=Search&number=100&format=html&sortby=symbol&match=THBS3) | calcium-dependent phospholipid binding;ubiquitin thiolesterase activity;calcium ion binding;cysteine-type endopeptidase activity;hydrolase activity;cysteine-type peptidase activity;peptidase activity | [188062](http://www.ncbi.nlm.nih.gov/entrez/dispomim.cgi?id=188062) | **-1.42** |
| 1416625_AT | serine (or cysteine) proteinase inhibitor, clade G, member 1 | [SERPING1](http://www.gene.ucl.ac.uk/cgi-bin/nomenclature/searchgenes.pl?field=symbol&anchor=equals&symbol_search=Search&number=100&format=html&sortby=symbol&match=SERPING1) | calcium-dependent phospholipid binding;ubiquitin thiolesterase activity;calcium ion binding;cysteine-type endopeptidase activity;hydrolase activity;cysteine-type peptidase activity;peptidase activity | [606860](http://www.ncbi.nlm.nih.gov/entrez/dispomim.cgi?id=606860) | **-1.3** |
| 1416632_AT | malic enzyme, supernatant | [MOD1](http://www.gene.ucl.ac.uk/cgi-bin/nomenclature/searchgenes.pl?field=symbol&anchor=equals&symbol_search=Search&number=100&format=html&sortby=symbol&match=MOD1) | calmodulin binding |  | **2.27** |
| 1416647_AT | branched chain ketoacid dehydrogenase E1, alpha polypeptide | [BCKDHA](http://www.gene.ucl.ac.uk/cgi-bin/nomenclature/searchgenes.pl?field=symbol&anchor=equals&symbol_search=Search&number=100&format=html&sortby=symbol&match=BCKDHA) | calmodulin binding | [608348](http://www.ncbi.nlm.nih.gov/entrez/dispomim.cgi?id=608348) | **1.47** |
| 1416648_AT | dynein, cytoplasmic, heavy chain 1 | [DNCHC1](http://www.gene.ucl.ac.uk/cgi-bin/nomenclature/searchgenes.pl?field=symbol&anchor=equals&symbol_search=Search&number=100&format=html&sortby=symbol&match=DNCHC1) | calmodulin binding |  | **-1.35** |
| 1416652_AT | asporin | [ASPN](http://www.gene.ucl.ac.uk/cgi-bin/nomenclature/searchgenes.pl?field=symbol&anchor=equals&symbol_search=Search&number=100&format=html&sortby=symbol&match=ASPN) | calpain inhibitor activity;cysteine protease inhibitor activity | [608135](http://www.ncbi.nlm.nih.gov/entrez/dispomim.cgi?id=608135) | **-2.52** |
| 1416653_AT | syntaxin binding protein 3 | [STXBP3](http://www.gene.ucl.ac.uk/cgi-bin/nomenclature/searchgenes.pl?field=symbol&anchor=equals&symbol_search=Search&number=100&format=html&sortby=symbol&match=STXBP3) | calpain inhibitor activity;cysteine protease inhibitor activity | [608339](http://www.ncbi.nlm.nih.gov/entrez/dispomim.cgi?id=608339) | **-1.28** |
| 1416655_AT | RIKEN cDNA 1500002I11 gene | [1500002I11RIK](http://www.gene.ucl.ac.uk/cgi-bin/nomenclature/searchgenes.pl?field=symbol&anchor=equals&symbol_search=Search&number=100&format=html&sortby=symbol&match=1500002I11RIK) | calpain inhibitor activity;cysteine protease inhibitor activity |  | **-1.64** |
| 1416656_AT | chloride intracellular channel 1 | [CLIC1](http://www.gene.ucl.ac.uk/cgi-bin/nomenclature/searchgenes.pl?field=symbol&anchor=equals&symbol_search=Search&number=100&format=html&sortby=symbol&match=CLIC1) | cAMP-dependent protein kinase inhibitor activity;protein kinase inhibitor activity | [602872](http://www.ncbi.nlm.nih.gov/entrez/dispomim.cgi?id=602872) | **-1.46** |
| 1416658_AT | frizzled-related protein | [FRZB](http://www.gene.ucl.ac.uk/cgi-bin/nomenclature/searchgenes.pl?field=symbol&anchor=equals&symbol_search=Search&number=100&format=html&sortby=symbol&match=FRZB) | cAMP-dependent protein kinase inhibitor activity;protein kinase inhibitor activity | [605083](http://www.ncbi.nlm.nih.gov/entrez/dispomim.cgi?id=605083) | **1.7** |
| 1416660_AT | eukaryotic translation initiation factor 3, subunit 10 (theta) | [EIF3S10](http://www.gene.ucl.ac.uk/cgi-bin/nomenclature/searchgenes.pl?field=symbol&anchor=equals&symbol_search=Search&number=100&format=html&sortby=symbol&match=EIF3S10) | cAMP-specific phosphodiesterase activity;3',5'-cyclic-nucleotide phosphodiesterase activity;catalytic activity;hydrolase activity | [602039](http://www.ncbi.nlm.nih.gov/entrez/dispomim.cgi?id=602039) | **-1.27** |
| 1416663_AT | NADH dehydrogenase (ubiquinone) 1 alpha subcomplex, 9 | [NDUFA9](http://www.gene.ucl.ac.uk/cgi-bin/nomenclature/searchgenes.pl?field=symbol&anchor=equals&symbol_search=Search&number=100&format=html&sortby=symbol&match=NDUFA9) | carbon-nitrogen ligase activity, with glutamine as amido-N-donor;integrin binding;heparin binding | [603834](http://www.ncbi.nlm.nih.gov/entrez/dispomim.cgi?id=603834) | **1.32** |
| 1416665_AT | demethyl-Q 7 | [COQ7](http://www.gene.ucl.ac.uk/cgi-bin/nomenclature/searchgenes.pl?field=symbol&anchor=equals&symbol_search=Search&number=100&format=html&sortby=symbol&match=COQ7) | carboxylesterase activity;carboxylic ester hydrolase activity;catalytic activity;serine esterase activity;hydrolase activity | [601683](http://www.ncbi.nlm.nih.gov/entrez/dispomim.cgi?id=601683) | **1.39** |
| 1416666_AT | serine (or cysteine) proteinase inhibitor, clade E, member 2 | [SERPINE2](http://www.gene.ucl.ac.uk/cgi-bin/nomenclature/searchgenes.pl?field=symbol&anchor=equals&symbol_search=Search&number=100&format=html&sortby=symbol&match=SERPINE2) | carboxylesterase activity;carboxylic ester hydrolase activity;serine esterase activity;catalytic activity;hydrolase activity | [177010](http://www.ncbi.nlm.nih.gov/entrez/dispomim.cgi?id=177010) | **1.77** |
| 1416667_AT | phenylalkylamine Ca2+ antagonist (emopamil) binding protein | [EBP](http://www.gene.ucl.ac.uk/cgi-bin/nomenclature/searchgenes.pl?field=symbol&anchor=equals&symbol_search=Search&number=100&format=html&sortby=symbol&match=EBP) | carboxylesterase activity;serine esterase activity;catalytic activity;hydrolase activity | [300205](http://www.ncbi.nlm.nih.gov/entrez/dispomim.cgi?id=300205) | **-1.4** |
| 1416679_AT | ATP-binding cassette, sub-family D (ALD), member 3 | [ABCD3](http://www.gene.ucl.ac.uk/cgi-bin/nomenclature/searchgenes.pl?field=symbol&anchor=equals&symbol_search=Search&number=100&format=html&sortby=symbol&match=ABCD3) | carboxylic ester hydrolase activity;catalytic activity;sterol esterase activity;hydrolase activity | [170995](http://www.ncbi.nlm.nih.gov/entrez/dispomim.cgi?id=170995) | **1.35** |
| 1416683_AT | plexin B2 | [PLXNB2](http://www.gene.ucl.ac.uk/cgi-bin/nomenclature/searchgenes.pl?field=symbol&anchor=equals&symbol_search=Search&number=100&format=html&sortby=symbol&match=PLXNB2) | carboxylic ester hydrolase activity;catalytic activity;sterol esterase activity;hydrolase activity | [604293](http://www.ncbi.nlm.nih.gov/entrez/dispomim.cgi?id=604293) | **-1.8** |
| 1416686_AT | procollagen lysine, 2-oxoglutarate 5-dioxygenase 2 | [PLOD2](http://www.gene.ucl.ac.uk/cgi-bin/nomenclature/searchgenes.pl?field=symbol&anchor=equals&symbol_search=Search&number=100&format=html&sortby=symbol&match=PLOD2) | carboxy-lyase activity;lyase activity;ornithine decarboxylase activity;catalytic activity | [601865](http://www.ncbi.nlm.nih.gov/entrez/dispomim.cgi?id=601865) | **-2.42** |
| 1416687_AT | procollagen lysine, 2-oxoglutarate 5-dioxygenase 2 | [PLOD2](http://www.gene.ucl.ac.uk/cgi-bin/nomenclature/searchgenes.pl?field=symbol&anchor=equals&symbol_search=Search&number=100&format=html&sortby=symbol&match=PLOD2) | carboxy-lyase activity;lyase activity;sphinganine-1-phosphate aldolase activity | [601865](http://www.ncbi.nlm.nih.gov/entrez/dispomim.cgi?id=601865) | **-2.4** |
| 1416695_AT | benzodiazepine receptor, peripheral | [BZRP](http://www.gene.ucl.ac.uk/cgi-bin/nomenclature/searchgenes.pl?field=symbol&anchor=equals&symbol_search=Search&number=100&format=html&sortby=symbol&match=BZRP) | carboxy-lyase activity;phosphatidylserine decarboxylase activity;lyase activity | [109610](http://www.ncbi.nlm.nih.gov/entrez/dispomim.cgi?id=109610) | **-1.99** |
| 1416698_A_AT | CDC28 protein kinase 1b | [CKS1B](http://www.gene.ucl.ac.uk/cgi-bin/nomenclature/searchgenes.pl?field=symbol&anchor=equals&symbol_search=Search&number=100&format=html&sortby=symbol&match=CKS1B) | carboxy-lyase activity;phosphatidylserine decarboxylase activity;lyase activity | [116900](http://www.ncbi.nlm.nih.gov/entrez/dispomim.cgi?id=116900) | **-2.11** |
| 1416703_AT | mitogen activated protein kinase 14 | [MAPK14](http://www.gene.ucl.ac.uk/cgi-bin/nomenclature/searchgenes.pl?field=symbol&anchor=equals&symbol_search=Search&number=100&format=html&sortby=symbol&match=MAPK14) | carboxy-lyase activity;phosphatidylserine decarboxylase activity;lyase activity | [600289](http://www.ncbi.nlm.nih.gov/entrez/dispomim.cgi?id=600289) | **1.28** |
| 1416704_AT | mitogen activated protein kinase 14 | [MAPK14](http://www.gene.ucl.ac.uk/cgi-bin/nomenclature/searchgenes.pl?field=symbol&anchor=equals&symbol_search=Search&number=100&format=html&sortby=symbol&match=MAPK14) | carboxy-lyase activity;phosphatidylserine decarboxylase activity;lyase activity | [600289](http://www.ncbi.nlm.nih.gov/entrez/dispomim.cgi?id=600289) | **1.52** |
| 1416714_AT | interferon consensus sequence binding protein 1 | [ICSBP1](http://www.gene.ucl.ac.uk/cgi-bin/nomenclature/searchgenes.pl?field=symbol&anchor=equals&symbol_search=Search&number=100&format=html&sortby=symbol&match=ICSBP1) | carboxy-lyase activity;phosphatidylserine decarboxylase activity;lyase activity | [601565](http://www.ncbi.nlm.nih.gov/entrez/dispomim.cgi?id=601565) | **-1.84** |
| 1416731_AT | topoisomerase (DNA) II beta | [TOP2B](http://www.gene.ucl.ac.uk/cgi-bin/nomenclature/searchgenes.pl?field=symbol&anchor=equals&symbol_search=Search&number=100&format=html&sortby=symbol&match=TOP2B) | carboxypeptidase A activity | [126431](http://www.ncbi.nlm.nih.gov/entrez/dispomim.cgi?id=126431) | **-1.22** |
| 1416735_AT | N-acylsphingosine amidohydrolase 1 | [ASAH1](http://www.gene.ucl.ac.uk/cgi-bin/nomenclature/searchgenes.pl?field=symbol&anchor=equals&symbol_search=Search&number=100&format=html&sortby=symbol&match=ASAH1) | carboxypeptidase activity;hydrolase activity | [228000](http://www.ncbi.nlm.nih.gov/entrez/dispomim.cgi?id=228000) | **-1.48** |
| 1416737_AT | glycogen synthase 3, brain | [GYS3](http://www.gene.ucl.ac.uk/cgi-bin/nomenclature/searchgenes.pl?field=symbol&anchor=equals&symbol_search=Search&number=100&format=html&sortby=symbol&match=GYS3) | carboxypeptidase activity;metallopeptidase activity;peptidase activity |  | **1.23** |
| 1416740_AT | procollagen, type V, alpha 1 | [COL5A1](http://www.gene.ucl.ac.uk/cgi-bin/nomenclature/searchgenes.pl?field=symbol&anchor=equals&symbol_search=Search&number=100&format=html&sortby=symbol&match=COL5A1) | carnitine O-acetyltransferase activity;acyltransferase activity;transferase activity | [120215](http://www.ncbi.nlm.nih.gov/entrez/dispomim.cgi?id=120215) | **-2.33** |
| 1416741_AT | procollagen, type V, alpha 1 | [COL5A1](http://www.gene.ucl.ac.uk/cgi-bin/nomenclature/searchgenes.pl?field=symbol&anchor=equals&symbol_search=Search&number=100&format=html&sortby=symbol&match=COL5A1) | carnitine O-acetyltransferase activity;acyltransferase activity;transferase activity | [120215](http://www.ncbi.nlm.nih.gov/entrez/dispomim.cgi?id=120215) | **-2.24** |
| 1416742_AT | craniofacial development protein 1 | [CFDP1](http://www.gene.ucl.ac.uk/cgi-bin/nomenclature/searchgenes.pl?field=symbol&anchor=equals&symbol_search=Search&number=100&format=html&sortby=symbol&match=CFDP1) | carnitine O-palmitoyltransferase activity;acyltransferase activity;transferase activity | [608108](http://www.ncbi.nlm.nih.gov/entrez/dispomim.cgi?id=608108) | **-1.21** |
| 1416752_AT | LIM domain binding 3 | [LDB3](http://www.gene.ucl.ac.uk/cgi-bin/nomenclature/searchgenes.pl?field=symbol&anchor=equals&symbol_search=Search&number=100&format=html&sortby=symbol&match=LDB3) | carnitine O-palmitoyltransferase activity;kinase activity;acyltransferase activity;transferase activity | [605906](http://www.ncbi.nlm.nih.gov/entrez/dispomim.cgi?id=605906) | **1.35** |
| 1416762_AT | S100 calcium binding protein A10 (calpactin) | [S100A10](http://www.gene.ucl.ac.uk/cgi-bin/nomenclature/searchgenes.pl?field=symbol&anchor=equals&symbol_search=Search&number=100&format=html&sortby=symbol&match=S100A10) | caspase activator activity | [114085](http://www.ncbi.nlm.nih.gov/entrez/dispomim.cgi?id=114085) | **-1.92** |
| 1416766_AT | RIKEN cDNA 2810484M10 gene | [2810484M10RIK](http://www.gene.ucl.ac.uk/cgi-bin/nomenclature/searchgenes.pl?field=symbol&anchor=equals&symbol_search=Search&number=100&format=html&sortby=symbol&match=2810484M10RIK) | catalytic activity |  | **1.24** |
| 1416772_AT | carnitine palmitoyltransferase 2 | [CPT2](http://www.gene.ucl.ac.uk/cgi-bin/nomenclature/searchgenes.pl?field=symbol&anchor=equals&symbol_search=Search&number=100&format=html&sortby=symbol&match=CPT2) | catalytic activity | [600650](http://www.ncbi.nlm.nih.gov/entrez/dispomim.cgi?id=600650) | **2.15** |
| 1416775_AT | RIKEN cDNA 2310004L02 gene | [2310004L02RIK](http://www.gene.ucl.ac.uk/cgi-bin/nomenclature/searchgenes.pl?field=symbol&anchor=equals&symbol_search=Search&number=100&format=html&sortby=symbol&match=2310004L02RIK) | catalytic activity |  | **1.34** |
| 1416776_AT | crystallin, mu | [CRYM](http://www.gene.ucl.ac.uk/cgi-bin/nomenclature/searchgenes.pl?field=symbol&anchor=equals&symbol_search=Search&number=100&format=html&sortby=symbol&match=CRYM) | catalytic activity | [123740](http://www.ncbi.nlm.nih.gov/entrez/dispomim.cgi?id=123740) | **2.12** |
| 1416778_AT | serum deprivation response | [SDPR](http://www.gene.ucl.ac.uk/cgi-bin/nomenclature/searchgenes.pl?field=symbol&anchor=equals&symbol_search=Search&number=100&format=html&sortby=symbol&match=SDPR) | catalytic activity | [606728](http://www.ncbi.nlm.nih.gov/entrez/dispomim.cgi?id=606728) | **1.63** |
| 1416779_AT | serum deprivation response | [SDPR](http://www.gene.ucl.ac.uk/cgi-bin/nomenclature/searchgenes.pl?field=symbol&anchor=equals&symbol_search=Search&number=100&format=html&sortby=symbol&match=SDPR) | catalytic activity | [606728](http://www.ncbi.nlm.nih.gov/entrez/dispomim.cgi?id=606728) | **1.73** |
| 1416780_AT | phosphofructokinase, muscle | [PFKM](http://www.gene.ucl.ac.uk/cgi-bin/nomenclature/searchgenes.pl?field=symbol&anchor=equals&symbol_search=Search&number=100&format=html&sortby=symbol&match=PFKM) | catalytic activity | [232800](http://www.ncbi.nlm.nih.gov/entrez/dispomim.cgi?id=232800) | **1.38** |
| 1416788_A_AT | isocitrate dehydrogenase 3 (NAD+), gamma | [IDH3G](http://www.gene.ucl.ac.uk/cgi-bin/nomenclature/searchgenes.pl?field=symbol&anchor=equals&symbol_search=Search&number=100&format=html&sortby=symbol&match=IDH3G) | catalytic activity | [300089](http://www.ncbi.nlm.nih.gov/entrez/dispomim.cgi?id=300089) | **1.28** |
| 1416789_AT | isocitrate dehydrogenase 3 (NAD+), gamma | [IDH3G](http://www.gene.ucl.ac.uk/cgi-bin/nomenclature/searchgenes.pl?field=symbol&anchor=equals&symbol_search=Search&number=100&format=html&sortby=symbol&match=IDH3G) | catalytic activity | [300089](http://www.ncbi.nlm.nih.gov/entrez/dispomim.cgi?id=300089) | **1.34** |
| 1416790_A_AT | thymine DNA glycosylase | [TDG](http://www.gene.ucl.ac.uk/cgi-bin/nomenclature/searchgenes.pl?field=symbol&anchor=equals&symbol_search=Search&number=100&format=html&sortby=symbol&match=TDG) | catalytic activity | [601423](http://www.ncbi.nlm.nih.gov/entrez/dispomim.cgi?id=601423) | **-1.32** |
| 1416794_AT | ADP-ribosylation factor-like 6 interacting protein 2 | [ARL6IP2](http://www.gene.ucl.ac.uk/cgi-bin/nomenclature/searchgenes.pl?field=symbol&anchor=equals&symbol_search=Search&number=100&format=html&sortby=symbol&match=ARL6IP2) | catalytic activity |  | **1.49** |
| 1416803_AT | FK506 binding protein 7 | [FKBP7](http://www.gene.ucl.ac.uk/cgi-bin/nomenclature/searchgenes.pl?field=symbol&anchor=equals&symbol_search=Search&number=100&format=html&sortby=symbol&match=FKBP7) | catalytic activity | [607062](http://www.ncbi.nlm.nih.gov/entrez/dispomim.cgi?id=607062) | **-1.45** |
| 1416807_AT | ribosomal protein L36a | [RPL36A](http://www.gene.ucl.ac.uk/cgi-bin/nomenclature/searchgenes.pl?field=symbol&anchor=equals&symbol_search=Search&number=100&format=html&sortby=symbol&match=RPL36A) | catalytic activity |  | **-1.9** |
| 1416808_AT | nidogen 1 | [NID1](http://www.gene.ucl.ac.uk/cgi-bin/nomenclature/searchgenes.pl?field=symbol&anchor=equals&symbol_search=Search&number=100&format=html&sortby=symbol&match=NID1) | catalytic activity |  | **-1.35** |
| 1416824_AT | RIKEN cDNA B230118H07 gene | [B230118H07RIK](http://www.gene.ucl.ac.uk/cgi-bin/nomenclature/searchgenes.pl?field=symbol&anchor=equals&symbol_search=Search&number=100&format=html&sortby=symbol&match=B230118H07RIK) | catalytic activity;cytoskeletal protein binding |  | **1.64** |
| 1416829_AT | ATP synthase, H+ transporting mitochondrial F1 complex, beta subunit | [ATP5B](http://www.gene.ucl.ac.uk/cgi-bin/nomenclature/searchgenes.pl?field=symbol&anchor=equals&symbol_search=Search&number=100&format=html&sortby=symbol&match=ATP5B) | catalytic activity;cytoskeletal protein binding | [102910](http://www.ncbi.nlm.nih.gov/entrez/dispomim.cgi?id=102910) | **1.23** |
| 1416832_AT | solute carrier family 39 (metal ion transporter), member 8 | [SLC39A8](http://www.gene.ucl.ac.uk/cgi-bin/nomenclature/searchgenes.pl?field=symbol&anchor=equals&symbol_search=Search&number=100&format=html&sortby=symbol&match=SLC39A8) | catalytic activity;cytoskeletal protein binding | [608732](http://www.ncbi.nlm.nih.gov/entrez/dispomim.cgi?id=608732) | **1.92** |
| 1416834_X_AT | NADH dehydrogenase (ubiquinone) 1 beta subcomplex, 2 | [NDUFB2](http://www.gene.ucl.ac.uk/cgi-bin/nomenclature/searchgenes.pl?field=symbol&anchor=equals&symbol_search=Search&number=100&format=html&sortby=symbol&match=NDUFB2) | catalytic activity;hydrolase activity | [603838](http://www.ncbi.nlm.nih.gov/entrez/dispomim.cgi?id=603838) | **1.35** |
| 1416835_S_AT | S-adenosylmethionine decarboxylase 1 | [AMD1](http://www.gene.ucl.ac.uk/cgi-bin/nomenclature/searchgenes.pl?field=symbol&anchor=equals&symbol_search=Search&number=100&format=html&sortby=symbol&match=AMD1) | catalytic activity;hydrolase activity | [180980](http://www.ncbi.nlm.nih.gov/entrez/dispomim.cgi?id=180980) | **1.49** |
| 1416837_AT | Bcl2-associated X protein | [BAX](http://www.gene.ucl.ac.uk/cgi-bin/nomenclature/searchgenes.pl?field=symbol&anchor=equals&symbol_search=Search&number=100&format=html&sortby=symbol&match=BAX) | catalytic activity;hydrolase activity | [600040](http://www.ncbi.nlm.nih.gov/entrez/dispomim.cgi?id=600040) | **-1.58** |
| 1416838_AT | gb:BC019175.1 /DB_XREF=gi:17512430 /FEA=FLmRNA /CN |  | catalytic activity;hydrolase activity |  | **1.45** |
| 1416842_AT | glutathione S-transferase, mu 5 | [GSTM5](http://www.gene.ucl.ac.uk/cgi-bin/nomenclature/searchgenes.pl?field=symbol&anchor=equals&symbol_search=Search&number=100&format=html&sortby=symbol&match=GSTM5) | catalytic activity;hydrolase activity |  | **1.32** |
| 1416845_AT | heat shock 70kDa protein 5 binding protein 1 | [HSPA5BP1](http://www.gene.ucl.ac.uk/cgi-bin/nomenclature/searchgenes.pl?field=symbol&anchor=equals&symbol_search=Search&number=100&format=html&sortby=symbol&match=HSPA5BP1) | catalytic activity;hydrolase activity |  | **-2.45** |
| 1416846_A_AT | PDZ domain containing RING finger 3 | [PDZRN3](http://www.gene.ucl.ac.uk/cgi-bin/nomenclature/searchgenes.pl?field=symbol&anchor=equals&symbol_search=Search&number=100&format=html&sortby=symbol&match=PDZRN3) | catalytic activity;hydrolase activity |  | **1.69** |
| 1416849_AT | DNA segment, Chr 10, ERATO Doi 214, expressed | [D10ERTD214E](http://www.gene.ucl.ac.uk/cgi-bin/nomenclature/searchgenes.pl?field=symbol&anchor=equals&symbol_search=Search&number=100&format=html&sortby=symbol&match=D10ERTD214E) | catalytic activity;hydrolase activity |  | **1.4** |
| 1416857_AT | stromal cell derived factor 2 | [SDF2](http://www.gene.ucl.ac.uk/cgi-bin/nomenclature/searchgenes.pl?field=symbol&anchor=equals&symbol_search=Search&number=100&format=html&sortby=symbol&match=SDF2) | catalytic activity;hydrolase activity;lysophospholipase activity | [602934](http://www.ncbi.nlm.nih.gov/entrez/dispomim.cgi?id=602934) | **-1.27** |
| 1416861_AT | signal transducing adaptor molecule (SH3 domain and ITAM motif) 1 | [STAM](http://www.gene.ucl.ac.uk/cgi-bin/nomenclature/searchgenes.pl?field=symbol&anchor=equals&symbol_search=Search&number=100&format=html&sortby=symbol&match=STAM) | catalytic activity;hydrolase activity;phospholipase D activity | [601899](http://www.ncbi.nlm.nih.gov/entrez/dispomim.cgi?id=601899) | **-1.47** |
| 1416871_AT | a disintegrin and metalloprotease domain 8 | [ADAM8](http://www.gene.ucl.ac.uk/cgi-bin/nomenclature/searchgenes.pl?field=symbol&anchor=equals&symbol_search=Search&number=100&format=html&sortby=symbol&match=ADAM8) | cathepsin E activity;aspartic-type endopeptidase activity;protein homodimerization activity;neutrophil collagenase activity;pepsin A activity;hydrolase activity | [602267](http://www.ncbi.nlm.nih.gov/entrez/dispomim.cgi?id=602267) | **-3.69** |
| 1416877_A_AT | mitochondrial ribosomal protein L51 | [MRPL51](http://www.gene.ucl.ac.uk/cgi-bin/nomenclature/searchgenes.pl?field=symbol&anchor=equals&symbol_search=Search&number=100&format=html&sortby=symbol&match=MRPL51) | cathepsin H activity;cysteine-type endopeptidase activity;hydrolase activity;cysteine-type peptidase activity;peptidase activity |  | **1.25** |
| 1416882_AT | regulator of G-protein signalling 10 | [RGS10](http://www.gene.ucl.ac.uk/cgi-bin/nomenclature/searchgenes.pl?field=symbol&anchor=equals&symbol_search=Search&number=100&format=html&sortby=symbol&match=RGS10) | cathepsin H activity;cysteine-type endopeptidase activity;hydrolase activity;peptidase activity;cysteine-type peptidase activity | [602856](http://www.ncbi.nlm.nih.gov/entrez/dispomim.cgi?id=602856) | **-1.93** |
| 1416884_AT | gb:BC018354.1 /DB_XREF=gi:17390827 /FEA=FLmRNA /CN |  | cathepsin K activity;cysteine-type endopeptidase activity;hydrolase activity;cysteine-type peptidase activity |  | **-1.27** |
| 1416885_AT | RIKEN cDNA 1110038F14 gene | [1110038F14RIK](http://www.gene.ucl.ac.uk/cgi-bin/nomenclature/searchgenes.pl?field=symbol&anchor=equals&symbol_search=Search&number=100&format=html&sortby=symbol&match=1110038F14RIK) | cathepsin S activity;cysteine-type endopeptidase activity;hydrolase activity;cysteine-type peptidase activity;peptidase activity |  | **-1.39** |
| 1416891_AT | gb:U70674.1 /DB_XREF=gi:1575755 /GEN=m-nb /FEA=FLm |  | cation channel activity;calcium channel activity;DNA binding;ion channel activity;receptor activity;transcription factor activity |  | **1.23** |
| 1416897_AT | poly (ADP-ribose) polymerase family, member 9 | [PARP9](http://www.gene.ucl.ac.uk/cgi-bin/nomenclature/searchgenes.pl?field=symbol&anchor=equals&symbol_search=Search&number=100&format=html&sortby=symbol&match=PARP9) | cation channel activity;ion channel activity |  | **-1.32** |
| 1416901_AT | Niemann Pick type C2 | [NPC2](http://www.gene.ucl.ac.uk/cgi-bin/nomenclature/searchgenes.pl?field=symbol&anchor=equals&symbol_search=Search&number=100&format=html&sortby=symbol&match=NPC2) | cation channel activity;potassium channel activity;ion channel activity;inward rectifier potassium channel activity | [601015](http://www.ncbi.nlm.nih.gov/entrez/dispomim.cgi?id=601015) | **-1.48** |
| 1416910_AT | DnaJ (Hsp40) homolog, subfamily D, member 1 | [DNAJD1](http://www.gene.ucl.ac.uk/cgi-bin/nomenclature/searchgenes.pl?field=symbol&anchor=equals&symbol_search=Search&number=100&format=html&sortby=symbol&match=DNAJD1) | cation channel activity;potassium channel activity;protein binding;ion channel activity;voltage-gated ion channel activity;voltage-gated potassium channel activity |  | **1.26** |
| 1416922_A_AT | BCL2/adenovirus E1B 19kDa-interacting protein 3-like | [BNIP3L](http://www.gene.ucl.ac.uk/cgi-bin/nomenclature/searchgenes.pl?field=symbol&anchor=equals&symbol_search=Search&number=100&format=html&sortby=symbol&match=BNIP3L) | cation channel activity;potassium channel activity;protein binding;kinase activity;ion channel activity;voltage-gated ion channel activity;voltage-gated potassium channel activity;cytoskeletal protein binding | [605368](http://www.ncbi.nlm.nih.gov/entrez/dispomim.cgi?id=605368) | **1.27** |
| 1416924_AT | brain protein I3 | [BRI3](http://www.gene.ucl.ac.uk/cgi-bin/nomenclature/searchgenes.pl?field=symbol&anchor=equals&symbol_search=Search&number=100&format=html&sortby=symbol&match=BRI3) | cation channel activity;potassium channel activity;protein binding;voltage-gated ion channel activity;ion channel activity;voltage-gated potassium channel activity |  | **1.39** |
| 1416925_AT | karyopherin (importin) beta 1 | [KPNB1](http://www.gene.ucl.ac.uk/cgi-bin/nomenclature/searchgenes.pl?field=symbol&anchor=equals&symbol_search=Search&number=100&format=html&sortby=symbol&match=KPNB1) | cation channel activity;protein binding;potassium channel activity;ion channel activity;voltage-gated potassium channel activity | [602738](http://www.ncbi.nlm.nih.gov/entrez/dispomim.cgi?id=602738) | **-1.33** |
| 1416926_AT | transformation related protein 53 inducible nuclear protein 1 | [TRP53INP1](http://www.gene.ucl.ac.uk/cgi-bin/nomenclature/searchgenes.pl?field=symbol&anchor=equals&symbol_search=Search&number=100&format=html&sortby=symbol&match=TRP53INP1) | cation channel activity;protein binding;potassium channel activity;kinase activity;ion channel activity;voltage-gated ion channel activity;voltage-gated potassium channel activity;cytoskeletal protein binding |  | **-1.57** |
| 1416938_AT | coiled-coil-helix-coiled-coil-helix domain containing 1 | [CHCHD1](http://www.gene.ucl.ac.uk/cgi-bin/nomenclature/searchgenes.pl?field=symbol&anchor=equals&symbol_search=Search&number=100&format=html&sortby=symbol&match=CHCHD1) | cation channel activity;voltage-gated ion channel activity;ion channel activity;voltage-gated sodium channel activity | [608842](http://www.ncbi.nlm.nih.gov/entrez/dispomim.cgi?id=608842) | **1.28** |
| 1416940_AT | peptidylprolyl isomerase F (cyclophilin F) | [PPIF](http://www.gene.ucl.ac.uk/cgi-bin/nomenclature/searchgenes.pl?field=symbol&anchor=equals&symbol_search=Search&number=100&format=html&sortby=symbol&match=PPIF) | cation channel activity;voltage-gated ion channel activity;ion channel activity;voltage-gated sodium channel activity | [604486](http://www.ncbi.nlm.nih.gov/entrez/dispomim.cgi?id=604486) | **1.74** |
| 1416950_AT | tumor necrosis factor, alpha-induced protein 8 | [TNFAIP8](http://www.gene.ucl.ac.uk/cgi-bin/nomenclature/searchgenes.pl?field=symbol&anchor=equals&symbol_search=Search&number=100&format=html&sortby=symbol&match=TNFAIP8) | cation transporter activity |  | **-1.48** |
| 1416953_AT | connective tissue growth factor | [CTGF](http://www.gene.ucl.ac.uk/cgi-bin/nomenclature/searchgenes.pl?field=symbol&anchor=equals&symbol_search=Search&number=100&format=html&sortby=symbol&match=CTGF) | cation transporter activity | [121009](http://www.ncbi.nlm.nih.gov/entrez/dispomim.cgi?id=121009) | **-1.54** |
| 1416974_AT | signal transducing adaptor molecule (SH3 domain and ITAM motif) 2 | [STAM2](http://www.gene.ucl.ac.uk/cgi-bin/nomenclature/searchgenes.pl?field=symbol&anchor=equals&symbol_search=Search&number=100&format=html&sortby=symbol&match=STAM2) | cation transporter activity | [606244](http://www.ncbi.nlm.nih.gov/entrez/dispomim.cgi?id=606244) | **-1.3** |
| 1416979_AT | RIKEN cDNA 2510048O06 gene | [2510048O06RIK](http://www.gene.ucl.ac.uk/cgi-bin/nomenclature/searchgenes.pl?field=symbol&anchor=equals&symbol_search=Search&number=100&format=html&sortby=symbol&match=2510048O06RIK) | cation transporter activity |  | **-1.24** |
| 1416984_AT | mitochondrial ribosomal protein S18A | [MRPS18A](http://www.gene.ucl.ac.uk/cgi-bin/nomenclature/searchgenes.pl?field=symbol&anchor=equals&symbol_search=Search&number=100&format=html&sortby=symbol&match=MRPS18A) | cation transporter activity;peroxidase activity |  | **1.58** |
| 1416985_AT | gb:D87968.1 /DB_XREF=gi:1864014 /FEA=FLmRNA /CNT=1 |  | cation transporter activity;peroxidase activity |  | **-2.4** |
| 1416996_AT | TBC1 domain family, member 8 | [TBC1D8](http://www.gene.ucl.ac.uk/cgi-bin/nomenclature/searchgenes.pl?field=symbol&anchor=equals&symbol_search=Search&number=100&format=html&sortby=symbol&match=TBC1D8) | CDP-diacylglycerol-inositol 3-phosphatidyltransferase activity;manganese ion binding;transferase activity |  | **-1.38** |
| 1417002_AT | RIKEN cDNA 0610012G03 gene | [0610012G03RIK](http://www.gene.ucl.ac.uk/cgi-bin/nomenclature/searchgenes.pl?field=symbol&anchor=equals&symbol_search=Search&number=100&format=html&sortby=symbol&match=0610012G03RIK) | CDP-diacylglycerol-inositol 3-phosphatidyltransferase activity;manganese ion binding;transferase activity |  | **1.42** |
| 1417005_AT | kinesin 2 | [KNS2](http://www.gene.ucl.ac.uk/cgi-bin/nomenclature/searchgenes.pl?field=symbol&anchor=equals&symbol_search=Search&number=100&format=html&sortby=symbol&match=KNS2) | ceramidase activity;hydrolase activity | [600025](http://www.ncbi.nlm.nih.gov/entrez/dispomim.cgi?id=600025) | **-1.86** |
| 1417007_A_AT | vacuolar protein sorting 4b (yeast) | [VPS4B](http://www.gene.ucl.ac.uk/cgi-bin/nomenclature/searchgenes.pl?field=symbol&anchor=equals&symbol_search=Search&number=100&format=html&sortby=symbol&match=VPS4B) | chaperone activity |  | **-1.59** |
| 1417008_AT | carnitine acetyltransferase | [CRAT](http://www.gene.ucl.ac.uk/cgi-bin/nomenclature/searchgenes.pl?field=symbol&anchor=equals&symbol_search=Search&number=100&format=html&sortby=symbol&match=CRAT) | chaperone activity | [600184](http://www.ncbi.nlm.nih.gov/entrez/dispomim.cgi?id=600184) | **1.54** |
| 1417010_AT | zinc finger protein 238 | [ZFP238](http://www.gene.ucl.ac.uk/cgi-bin/nomenclature/searchgenes.pl?field=symbol&anchor=equals&symbol_search=Search&number=100&format=html&sortby=symbol&match=ZFP238) | chaperone activity |  | **1.36** |
| 1417013_AT | heat shock 27kDa protein 8 | [HSPB8](http://www.gene.ucl.ac.uk/cgi-bin/nomenclature/searchgenes.pl?field=symbol&anchor=equals&symbol_search=Search&number=100&format=html&sortby=symbol&match=HSPB8) | chaperone activity | [608014](http://www.ncbi.nlm.nih.gov/entrez/dispomim.cgi?id=608014) | **-1.61** |
| 1417014_AT | heat shock 27kDa protein 8 | [HSPB8](http://www.gene.ucl.ac.uk/cgi-bin/nomenclature/searchgenes.pl?field=symbol&anchor=equals&symbol_search=Search&number=100&format=html&sortby=symbol&match=HSPB8) | chaperone activity | [608014](http://www.ncbi.nlm.nih.gov/entrez/dispomim.cgi?id=608014) | **-1.43** |
| 1417018_AT | epidermal growth factor-containing fibulin-like extracellular matrix protein 2 | [EFEMP2](http://www.gene.ucl.ac.uk/cgi-bin/nomenclature/searchgenes.pl?field=symbol&anchor=equals&symbol_search=Search&number=100&format=html&sortby=symbol&match=EFEMP2) | chaperone activity | [604633](http://www.ncbi.nlm.nih.gov/entrez/dispomim.cgi?id=604633) | **-1.41** |
| 1417023_A_AT | fatty acid binding protein 4, adipocyte | [FABP4](http://www.gene.ucl.ac.uk/cgi-bin/nomenclature/searchgenes.pl?field=symbol&anchor=equals&symbol_search=Search&number=100&format=html&sortby=symbol&match=FABP4) | chaperone activity | [600434](http://www.ncbi.nlm.nih.gov/entrez/dispomim.cgi?id=600434) | **1.47** |
| 1417024_AT | histidyl-tRNA synthetase | [HARS](http://www.gene.ucl.ac.uk/cgi-bin/nomenclature/searchgenes.pl?field=symbol&anchor=equals&symbol_search=Search&number=100&format=html&sortby=symbol&match=HARS) | chaperone activity | [142810](http://www.ncbi.nlm.nih.gov/entrez/dispomim.cgi?id=142810) | **-1.28** |
| 1417025_AT | histocompatibility 2, class II antigen E beta | [H2-EB1](http://www.gene.ucl.ac.uk/cgi-bin/nomenclature/searchgenes.pl?field=symbol&anchor=equals&symbol_search=Search&number=100&format=html&sortby=symbol&match=H2-EB1) | chaperone activity |  | **-2.44** |
| 1417038_AT | septin 9 | [SEPT9](http://www.gene.ucl.ac.uk/cgi-bin/nomenclature/searchgenes.pl?field=symbol&anchor=equals&symbol_search=Search&number=100&format=html&sortby=symbol&match=SEPT9) | chaperone activity |  | **-1.85** |
| 1417039_A_AT | Cullin 7 | [CUL7](http://www.gene.ucl.ac.uk/cgi-bin/nomenclature/searchgenes.pl?field=symbol&anchor=equals&symbol_search=Search&number=100&format=html&sortby=symbol&match=CUL7) | chaperone activity |  | **-1.72** |
| 1417040_A_AT | Bcl-2-related ovarian killer protein | [BOK](http://www.gene.ucl.ac.uk/cgi-bin/nomenclature/searchgenes.pl?field=symbol&anchor=equals&symbol_search=Search&number=100&format=html&sortby=symbol&match=BOK) | chaperone activity;ATP binding | [605404](http://www.ncbi.nlm.nih.gov/entrez/dispomim.cgi?id=605404) | **-1.66** |
| 1417054_A_AT | RIKEN cDNA 0610009D07 gene | [0610009D07RIK](http://www.gene.ucl.ac.uk/cgi-bin/nomenclature/searchgenes.pl?field=symbol&anchor=equals&symbol_search=Search&number=100&format=html&sortby=symbol&match=0610009D07RIK) | chaperone activity;ATP binding |  | **-1.48** |
| 1417056_AT | proteasome (prosome, macropain) 28 subunit, alpha | [PSME1](http://www.gene.ucl.ac.uk/cgi-bin/nomenclature/searchgenes.pl?field=symbol&anchor=equals&symbol_search=Search&number=100&format=html&sortby=symbol&match=PSME1) | chaperone activity;ATP binding | [600654](http://www.ncbi.nlm.nih.gov/entrez/dispomim.cgi?id=600654) | **1.28** |
| 1417061_AT | solute carrier family 40 (iron-regulated transporter), member 1 | [SLC40A1](http://www.gene.ucl.ac.uk/cgi-bin/nomenclature/searchgenes.pl?field=symbol&anchor=equals&symbol_search=Search&number=100&format=html&sortby=symbol&match=SLC40A1) | chaperone activity;ATP binding | [604653](http://www.ncbi.nlm.nih.gov/entrez/dispomim.cgi?id=604653) | **1.49** |
| 1417063_AT | complement component 1, q subcomponent, beta polypeptide | [C1QB](http://www.gene.ucl.ac.uk/cgi-bin/nomenclature/searchgenes.pl?field=symbol&anchor=equals&symbol_search=Search&number=100&format=html&sortby=symbol&match=C1QB) | chaperone activity;ATP binding | [120570](http://www.ncbi.nlm.nih.gov/entrez/dispomim.cgi?id=120570) | **-3.44** |
| 1417066_AT | chaperone, ABC1 activity of bc1 complex like (S. pombe) | [CABC1](http://www.gene.ucl.ac.uk/cgi-bin/nomenclature/searchgenes.pl?field=symbol&anchor=equals&symbol_search=Search&number=100&format=html&sortby=symbol&match=CABC1) | chaperone activity;ATP binding | [606980](http://www.ncbi.nlm.nih.gov/entrez/dispomim.cgi?id=606980) | **1.35** |
| 1417067_S_AT | gb:AK014605.1 /DB_XREF=gi:12852564 /FEA=FLmRNA /CN |  | chaperone activity;ATP binding |  | **1.58** |
| 1417068_A_AT | protein tyrosine phosphatase, non-receptor type 1 | [PTPN1](http://www.gene.ucl.ac.uk/cgi-bin/nomenclature/searchgenes.pl?field=symbol&anchor=equals&symbol_search=Search&number=100&format=html&sortby=symbol&match=PTPN1) | chaperone activity;calcium ion binding;ATP binding | [176885](http://www.ncbi.nlm.nih.gov/entrez/dispomim.cgi?id=176885) | **-1.71** |
| 1417073_A_AT | quaking | [QK](http://www.gene.ucl.ac.uk/cgi-bin/nomenclature/searchgenes.pl?field=symbol&anchor=equals&symbol_search=Search&number=100&format=html&sortby=symbol&match=QK) | chaperone activity;co-chaperonin activity;protein binding |  | **1.31** |
| 1417080_A_AT | signaling intermediate in Toll pathway-evolutionarily conserved | [SITPEC](http://www.gene.ucl.ac.uk/cgi-bin/nomenclature/searchgenes.pl?field=symbol&anchor=equals&symbol_search=Search&number=100&format=html&sortby=symbol&match=SITPEC) | chaperone activity;DNA binding;N-methyltransferase activity | [608388](http://www.ncbi.nlm.nih.gov/entrez/dispomim.cgi?id=608388) | **1.36** |
| 1417082_AT | acidic nuclear phosphoprotein 32 family, member B | [ANP32B](http://www.gene.ucl.ac.uk/cgi-bin/nomenclature/searchgenes.pl?field=symbol&anchor=equals&symbol_search=Search&number=100&format=html&sortby=symbol&match=ANP32B) | chaperone activity;metal ion binding;copper ion transporter activity |  | **-1.31** |
| 1417083_AT | Sec61 beta subunit | [SEC61B](http://www.gene.ucl.ac.uk/cgi-bin/nomenclature/searchgenes.pl?field=symbol&anchor=equals&symbol_search=Search&number=100&format=html&sortby=symbol&match=SEC61B) | chaperone activity;molecular_function unknown;prostaglandin-E synthase activity |  | **-1.8** |
| 1417084_AT | eukaryotic translation initiation factor 4E binding protein 2 | [EIF4EBP2](http://www.gene.ucl.ac.uk/cgi-bin/nomenclature/searchgenes.pl?field=symbol&anchor=equals&symbol_search=Search&number=100&format=html&sortby=symbol&match=EIF4EBP2) | chaperone activity;nucleic acid binding;single-stranded DNA binding;RNA binding | [602224](http://www.ncbi.nlm.nih.gov/entrez/dispomim.cgi?id=602224) | **1.5** |
| 1417090_AT | reticulocalbin 1 | [RCN1](http://www.gene.ucl.ac.uk/cgi-bin/nomenclature/searchgenes.pl?field=symbol&anchor=equals&symbol_search=Search&number=100&format=html&sortby=symbol&match=RCN1) | chaperone activity;nucleic acid binding;snoRNA binding;snoRNP binding | [602735](http://www.ncbi.nlm.nih.gov/entrez/dispomim.cgi?id=602735) | **-1.35** |
| 1417094_AT | brain acyl-CoA hydrolase | [BACH](http://www.gene.ucl.ac.uk/cgi-bin/nomenclature/searchgenes.pl?field=symbol&anchor=equals&symbol_search=Search&number=100&format=html&sortby=symbol&match=BACH) | chaperone activity;protein binding |  | **1.38** |
| 1417101_AT | heat shock protein 2 | [HSPA2](http://www.gene.ucl.ac.uk/cgi-bin/nomenclature/searchgenes.pl?field=symbol&anchor=equals&symbol_search=Search&number=100&format=html&sortby=symbol&match=HSPA2) | chaperone activity;protein binding;adenyl-nucleotide exchange factor activity;protein homodimerization activity;co-chaperone activity;Hsp70/Hsp90 organizing protein activity | [140560](http://www.ncbi.nlm.nih.gov/entrez/dispomim.cgi?id=140560) | **-1.77** |
| 1417102_A_AT | NADH dehydrogenase (ubiquinone) 1 beta subcomplex, 5 | [NDUFB5](http://www.gene.ucl.ac.uk/cgi-bin/nomenclature/searchgenes.pl?field=symbol&anchor=equals&symbol_search=Search&number=100&format=html&sortby=symbol&match=NDUFB5) | chaperone activity;protein binding;ATP binding | [603841](http://www.ncbi.nlm.nih.gov/entrez/dispomim.cgi?id=603841) | **1.29** |
| 1417104_AT | epithelial membrane protein 3 | [EMP3](http://www.gene.ucl.ac.uk/cgi-bin/nomenclature/searchgenes.pl?field=symbol&anchor=equals&symbol_search=Search&number=100&format=html&sortby=symbol&match=EMP3) | chaperone activity;protein binding;ATP binding | [602335](http://www.ncbi.nlm.nih.gov/entrez/dispomim.cgi?id=602335) | **-1.65** |
| 1417109_AT | lipocalin 7 | [LCN7](http://www.gene.ucl.ac.uk/cgi-bin/nomenclature/searchgenes.pl?field=symbol&anchor=equals&symbol_search=Search&number=100&format=html&sortby=symbol&match=LCN7) | chaperone activity;protein binding;ATP binding |  | **1.93** |
| 1417112_AT | ADP-ribosylation factor-like 2 binding protein | [ARL2BP](http://www.gene.ucl.ac.uk/cgi-bin/nomenclature/searchgenes.pl?field=symbol&anchor=equals&symbol_search=Search&number=100&format=html&sortby=symbol&match=ARL2BP) | chaperone activity;protein binding;cytokine binding |  | **-1.5** |
| 1417126_A_AT | RIKEN cDNA 3110001N18 gene | [3110001N18RIK](http://www.gene.ucl.ac.uk/cgi-bin/nomenclature/searchgenes.pl?field=symbol&anchor=equals&symbol_search=Search&number=100&format=html&sortby=symbol&match=3110001N18RIK) | chaperone activity;protein binding;MHC class II receptor activity;receptor activity |  | **-1.47** |
| 1417128_AT | RIKEN cDNA 2810052M02 gene | [2810052M02RIK](http://www.gene.ucl.ac.uk/cgi-bin/nomenclature/searchgenes.pl?field=symbol&anchor=equals&symbol_search=Search&number=100&format=html&sortby=symbol&match=2810052M02RIK) | chaperone activity;protein binding;nitric-oxide synthase regulator activity;heat shock protein activity;protein homodimerization activity;TPR domain binding;ATP binding |  | **-3.45** |
| 1417138_S_AT | polymerase (RNA) II (DNA directed) polypeptide E | [POLR2E](http://www.gene.ucl.ac.uk/cgi-bin/nomenclature/searchgenes.pl?field=symbol&anchor=equals&symbol_search=Search&number=100&format=html&sortby=symbol&match=POLR2E) | chaperone activity;protein binding;nitric-oxide synthase regulator activity;heat shock protein activity;protein homodimerization activity;TPR domain binding;ATP binding | [180664](http://www.ncbi.nlm.nih.gov/entrez/dispomim.cgi?id=180664) | **1.31** |
| 1417143_AT | endothelial differentiation, lysophosphatidic acid G-protein-coupled receptor, 2 | [EDG2](http://www.gene.ucl.ac.uk/cgi-bin/nomenclature/searchgenes.pl?field=symbol&anchor=equals&symbol_search=Search&number=100&format=html&sortby=symbol&match=EDG2) | chaperone activity;protein binding;nitric-oxide synthase regulator activity;heat shock protein activity;protein homodimerization activity;TPR domain binding;ATP binding | [602282](http://www.ncbi.nlm.nih.gov/entrez/dispomim.cgi?id=602282) | **-1.38** |
| 1417146_AT | RIKEN cDNA 2410018C20 gene | [2410018C20RIK](http://www.gene.ucl.ac.uk/cgi-bin/nomenclature/searchgenes.pl?field=symbol&anchor=equals&symbol_search=Search&number=100&format=html&sortby=symbol&match=2410018C20RIK) | chaperone activity;protein kinase activity;ATP binding |  | **1.33** |
| 1417147_AT | RIKEN cDNA B230317C12 gene | [B230317C12RIK](http://www.gene.ucl.ac.uk/cgi-bin/nomenclature/searchgenes.pl?field=symbol&anchor=equals&symbol_search=Search&number=100&format=html&sortby=symbol&match=B230317C12RIK) | chaperone activity;protein kinase activity;ATP binding |  | **2.11** |
| 1417156_AT | keratin complex 1, acidic, gene 19 | [KRT1-19](http://www.gene.ucl.ac.uk/cgi-bin/nomenclature/searchgenes.pl?field=symbol&anchor=equals&symbol_search=Search&number=100&format=html&sortby=symbol&match=KRT1-19) | chaperone activity;RNA binding;transcription factor activity | [148020](http://www.ncbi.nlm.nih.gov/entrez/dispomim.cgi?id=148020) | **2.34** |
| 1417162_AT | RIKEN cDNA 2310061B02 gene | [2310061B02RIK](http://www.gene.ucl.ac.uk/cgi-bin/nomenclature/searchgenes.pl?field=symbol&anchor=equals&symbol_search=Search&number=100&format=html&sortby=symbol&match=2310061B02RIK) | chaperone activity;small GTPase regulatory/interacting protein activity;co-chaperone activity |  | **-1.47** |
| 1417177_AT | galactokinase 1 | [GALK1](http://www.gene.ucl.ac.uk/cgi-bin/nomenclature/searchgenes.pl?field=symbol&anchor=equals&symbol_search=Search&number=100&format=html&sortby=symbol&match=GALK1) | chaperone activity;sugar binding;calcium ion binding;calcium ion storage activity | [604313](http://www.ncbi.nlm.nih.gov/entrez/dispomim.cgi?id=604313) | **-1.9** |
| 1417182_AT | DnaJ (Hsp40) homolog, subfamily A, member 2 | [DNAJA2](http://www.gene.ucl.ac.uk/cgi-bin/nomenclature/searchgenes.pl?field=symbol&anchor=equals&symbol_search=Search&number=100&format=html&sortby=symbol&match=DNAJA2) | chaperone activity;sugar binding;calcium ion binding;calcium ion storage activity |  | **1.45** |
| 1417184_S_AT | hemoglobin, beta adult major chain | [HBB-B1](http://www.gene.ucl.ac.uk/cgi-bin/nomenclature/searchgenes.pl?field=symbol&anchor=equals&symbol_search=Search&number=100&format=html&sortby=symbol&match=HBB-B1) | chaperone activity;sugar binding;calcium ion binding;calcium ion storage activity |  | **2.33** |
| 1417190_AT | pre-B-cell colony-enhancing factor 1 | [PBEF1](http://www.gene.ucl.ac.uk/cgi-bin/nomenclature/searchgenes.pl?field=symbol&anchor=equals&symbol_search=Search&number=100&format=html&sortby=symbol&match=PBEF1) | chaperone activity;tubulin-specific chaperone activity | [608764](http://www.ncbi.nlm.nih.gov/entrez/dispomim.cgi?id=608764) | **1.42** |
| 1417193_AT | superoxide dismutase 2, mitochondrial | [SOD2](http://www.gene.ucl.ac.uk/cgi-bin/nomenclature/searchgenes.pl?field=symbol&anchor=equals&symbol_search=Search&number=100&format=html&sortby=symbol&match=SOD2) | chemokine activity;cytokine activity | [147460](http://www.ncbi.nlm.nih.gov/entrez/dispomim.cgi?id=147460) | **1.39** |
| 1417194_AT | superoxide dismutase 2, mitochondrial | [SOD2](http://www.gene.ucl.ac.uk/cgi-bin/nomenclature/searchgenes.pl?field=symbol&anchor=equals&symbol_search=Search&number=100&format=html&sortby=symbol&match=SOD2) | chemokine activity;cytokine activity | [147460](http://www.ncbi.nlm.nih.gov/entrez/dispomim.cgi?id=147460) | **2.07** |
| 1417196_S_AT | DNA segment, Chr 8, ERATO Doi 594, expressed | [D8ERTD594E](http://www.gene.ucl.ac.uk/cgi-bin/nomenclature/searchgenes.pl?field=symbol&anchor=equals&symbol_search=Search&number=100&format=html&sortby=symbol&match=D8ERTD594E) | chemokine activity;cytokine activity |  | **1.44** |
| 1417200_AT | RIKEN cDNA 1300007B12 gene | [1300007B12RIK](http://www.gene.ucl.ac.uk/cgi-bin/nomenclature/searchgenes.pl?field=symbol&anchor=equals&symbol_search=Search&number=100&format=html&sortby=symbol&match=1300007B12RIK) | chemokine activity;cytokine activity |  | **-1.43** |
| 1417201_AT | 5'-nucleotidase, cytosolic II | [NT5C2](http://www.gene.ucl.ac.uk/cgi-bin/nomenclature/searchgenes.pl?field=symbol&anchor=equals&symbol_search=Search&number=100&format=html&sortby=symbol&match=NT5C2) | chemokine activity;cytokine activity | [600417](http://www.ncbi.nlm.nih.gov/entrez/dispomim.cgi?id=600417) | **-1.51** |
| 1417202_S_AT | ubiquitin-activating enzyme E1C | [UBE1C](http://www.gene.ucl.ac.uk/cgi-bin/nomenclature/searchgenes.pl?field=symbol&anchor=equals&symbol_search=Search&number=100&format=html&sortby=symbol&match=UBE1C) | chemokine activity;cytokine activity;heparin binding | [603172](http://www.ncbi.nlm.nih.gov/entrez/dispomim.cgi?id=603172) | **-1.39** |
| 1417203_AT | ethylmalonic encephalopathy 1 | [ETHE1](http://www.gene.ucl.ac.uk/cgi-bin/nomenclature/searchgenes.pl?field=symbol&anchor=equals&symbol_search=Search&number=100&format=html&sortby=symbol&match=ETHE1) | chemokine activity;growth factor activity;cytokine activity | [608451](http://www.ncbi.nlm.nih.gov/entrez/dispomim.cgi?id=608451) | **1.48** |
| 1417204_AT | KDEL (Lys-Asp-Glu-Leu) endoplasmic reticulum protein retention receptor 2 | [KDELR2](http://www.gene.ucl.ac.uk/cgi-bin/nomenclature/searchgenes.pl?field=symbol&anchor=equals&symbol_search=Search&number=100&format=html&sortby=symbol&match=KDELR2) | chitinase activity;sugar binding;beta-N-acetylhexosaminidase activity;chitin binding;hydrolase activity |  | **-1.29** |
| 1417205_AT | KDEL (Lys-Asp-Glu-Leu) endoplasmic reticulum protein retention receptor 2 | [KDELR2](http://www.gene.ucl.ac.uk/cgi-bin/nomenclature/searchgenes.pl?field=symbol&anchor=equals&symbol_search=Search&number=100&format=html&sortby=symbol&match=KDELR2) | choline-phosphate cytidylyltransferase activity;nucleotidyltransferase activity;transferase activity |  | **-1.47** |
| 1417206_AT | uroporphyrinogen decarboxylase | [UROD](http://www.gene.ucl.ac.uk/cgi-bin/nomenclature/searchgenes.pl?field=symbol&anchor=equals&symbol_search=Search&number=100&format=html&sortby=symbol&match=UROD) | choline-phosphate cytidylyltransferase activity;nucleotidyltransferase activity;transferase activity | [176100](http://www.ncbi.nlm.nih.gov/entrez/dispomim.cgi?id=176100) | **1.24** |
| 1417211_A_AT | RIKEN cDNA 1110032A03 gene | [1110032A03RIK](http://www.gene.ucl.ac.uk/cgi-bin/nomenclature/searchgenes.pl?field=symbol&anchor=equals&symbol_search=Search&number=100&format=html&sortby=symbol&match=1110032A03RIK) | CoA hydrolase activity;catalytic activity;serine esterase activity;hydrolase activity;palmitoyl-CoA hydrolase activity |  | **1.36** |
| 1417212_AT | RIKEN cDNA 9530058B02 gene | [9530058B02RIK](http://www.gene.ucl.ac.uk/cgi-bin/nomenclature/searchgenes.pl?field=symbol&anchor=equals&symbol_search=Search&number=100&format=html&sortby=symbol&match=9530058B02RIK) | CoA-transferase activity;3-oxoacid CoA-transferase activity;transferase activity |  | **1.72** |
| 1417219_S_AT | thymosin, beta 10 | [TMSB10](http://www.gene.ucl.ac.uk/cgi-bin/nomenclature/searchgenes.pl?field=symbol&anchor=equals&symbol_search=Search&number=100&format=html&sortby=symbol&match=TMSB10) | CoA-transferase activity;3-oxoacid CoA-transferase activity;transferase activity | [188399](http://www.ncbi.nlm.nih.gov/entrez/dispomim.cgi?id=188399) | **-5.25** |
| 1417225_AT | ADP-ribosylation factor-like 6 interacting protein 5 | [ARL6IP5](http://www.gene.ucl.ac.uk/cgi-bin/nomenclature/searchgenes.pl?field=symbol&anchor=equals&symbol_search=Search&number=100&format=html&sortby=symbol&match=ARL6IP5) | CoA-transferase activity;3-oxoacid CoA-transferase activity;transferase activity | [605709](http://www.ncbi.nlm.nih.gov/entrez/dispomim.cgi?id=605709) | **-1.47** |
| 1417226_AT | F-box and WD-40 domain protein 4 | [FBXW4](http://www.gene.ucl.ac.uk/cgi-bin/nomenclature/searchgenes.pl?field=symbol&anchor=equals&symbol_search=Search&number=100&format=html&sortby=symbol&match=FBXW4) | cob(I)yrinic acid a,c-diamide adenosyltransferase activity;transferase activity |  | **1.34** |
| 1417227_AT | methylcrotonoyl-Coenzyme A carboxylase 1 (alpha) | [MCCC1](http://www.gene.ucl.ac.uk/cgi-bin/nomenclature/searchgenes.pl?field=symbol&anchor=equals&symbol_search=Search&number=100&format=html&sortby=symbol&match=MCCC1) | cob(I)yrinic acid a,c-diamide adenosyltransferase activity;transferase activity | 210200 609010 | **1.26** |
| 1417239_AT | centrin 3 | [CETN3](http://www.gene.ucl.ac.uk/cgi-bin/nomenclature/searchgenes.pl?field=symbol&anchor=equals&symbol_search=Search&number=100&format=html&sortby=symbol&match=CETN3) | cyclin-dependent protein kinase activity | [602907](http://www.ncbi.nlm.nih.gov/entrez/dispomim.cgi?id=602907) | **-1.28** |
| 1417241_AT | EST X83328 | [X83328](http://www.gene.ucl.ac.uk/cgi-bin/nomenclature/searchgenes.pl?field=symbol&anchor=equals&symbol_search=Search&number=100&format=html&sortby=symbol&match=X83328) | cyclin-dependent protein kinase activity |  | **1.27** |
| 1417244_A_AT | interferon regulatory factor 7 | [IRF7](http://www.gene.ucl.ac.uk/cgi-bin/nomenclature/searchgenes.pl?field=symbol&anchor=equals&symbol_search=Search&number=100&format=html&sortby=symbol&match=IRF7) | cyclin-dependent protein kinase inhibitor activity;caspase activity;hydrolase activity;peptidase activity;cysteine-type peptidase activity | [605047](http://www.ncbi.nlm.nih.gov/entrez/dispomim.cgi?id=605047) | **-1.85** |
| 1417247_AT | expressed sequence AI597479 | [AI597479](http://www.gene.ucl.ac.uk/cgi-bin/nomenclature/searchgenes.pl?field=symbol&anchor=equals&symbol_search=Search&number=100&format=html&sortby=symbol&match=AI597479) | cyclin-dependent protein kinase inhibitor activity;hydrolase activity;caspase activity;cysteine-type peptidase activity;peptidase activity |  | **1.29** |
| 1417251_AT | palmdelphin | [PALMD](http://www.gene.ucl.ac.uk/cgi-bin/nomenclature/searchgenes.pl?field=symbol&anchor=equals&symbol_search=Search&number=100&format=html&sortby=symbol&match=PALMD) | cyclin-dependent protein kinase regulator activity |  | **1.66** |
| 1417252_AT | 5',3'-nucleotidase, cytosolic | [NT5C](http://www.gene.ucl.ac.uk/cgi-bin/nomenclature/searchgenes.pl?field=symbol&anchor=equals&symbol_search=Search&number=100&format=html&sortby=symbol&match=NT5C) | cyclin-dependent protein kinase regulator activity | [191720](http://www.ncbi.nlm.nih.gov/entrez/dispomim.cgi?id=191720) | **-1.84** |
| 1417264_AT | DNA segment, Chr 5, ERATO Doi 33, expressed | [D5ERTD33E](http://www.gene.ucl.ac.uk/cgi-bin/nomenclature/searchgenes.pl?field=symbol&anchor=equals&symbol_search=Search&number=100&format=html&sortby=symbol&match=D5ERTD33E) | cyclin-dependent protein kinase regulator activity |  | **1.43** |
| 1417265_S_AT | DNA segment, Chr 5, ERATO Doi 33, expressed | [D5ERTD33E](http://www.gene.ucl.ac.uk/cgi-bin/nomenclature/searchgenes.pl?field=symbol&anchor=equals&symbol_search=Search&number=100&format=html&sortby=symbol&match=D5ERTD33E) | cyclosporin A binding;isomerase activity;protein transporter activity;peptidyl-prolyl cis-trans isomerase activity;virion binding |  | **1.61** |
| 1417266_AT | chemokine (C-C motif) ligand 6 | [CCL6](http://www.gene.ucl.ac.uk/cgi-bin/nomenclature/searchgenes.pl?field=symbol&anchor=equals&symbol_search=Search&number=100&format=html&sortby=symbol&match=CCL6) | cysteine protease inhibitor activity |  | **-2.3** |
| 1417271_A_AT | endoglin | [ENG](http://www.gene.ucl.ac.uk/cgi-bin/nomenclature/searchgenes.pl?field=symbol&anchor=equals&symbol_search=Search&number=100&format=html&sortby=symbol&match=ENG) | cysteine-type endopeptidase activity;calpain activity | [131195](http://www.ncbi.nlm.nih.gov/entrez/dispomim.cgi?id=131195) | **1.85** |
| 1417272_AT | RIKEN cDNA 9130005N14 gene | [9130005N14RIK](http://www.gene.ucl.ac.uk/cgi-bin/nomenclature/searchgenes.pl?field=symbol&anchor=equals&symbol_search=Search&number=100&format=html&sortby=symbol&match=9130005N14RIK) | cysteine-type endopeptidase activity;cathepsin B activity;hydrolase activity;peroxidase activity;cysteine-type peptidase activity |  | **-1.34** |
| 1417275_AT | myelin and lymphocyte protein, T-cell differentiation protein | [MAL](http://www.gene.ucl.ac.uk/cgi-bin/nomenclature/searchgenes.pl?field=symbol&anchor=equals&symbol_search=Search&number=100&format=html&sortby=symbol&match=MAL) | cysteine-type endopeptidase activity;cathepsin B activity;hydrolase activity;peroxidase activity;cysteine-type peptidase activity | [188860](http://www.ncbi.nlm.nih.gov/entrez/dispomim.cgi?id=188860) | **2.27** |
| 1417279_AT | inositol 1,4,5-triphosphate receptor 1 | [ITPR1](http://www.gene.ucl.ac.uk/cgi-bin/nomenclature/searchgenes.pl?field=symbol&anchor=equals&symbol_search=Search&number=100&format=html&sortby=symbol&match=ITPR1) | cysteine-type endopeptidase activity;hydrolase activity;cysteine-type peptidase activity | [147265](http://www.ncbi.nlm.nih.gov/entrez/dispomim.cgi?id=147265) | **1.46** |
| 1417285_A_AT | NADH dehydrogenase (ubiquinone) 1 alpha subcomplex, 5 | [NDUFA5](http://www.gene.ucl.ac.uk/cgi-bin/nomenclature/searchgenes.pl?field=symbol&anchor=equals&symbol_search=Search&number=100&format=html&sortby=symbol&match=NDUFA5) | cysteine-type endopeptidase activity;hydrolase activity;cysteine-type peptidase activity | [601677](http://www.ncbi.nlm.nih.gov/entrez/dispomim.cgi?id=601677) | **1.25** |
| 1417286_AT | NADH dehydrogenase (ubiquinone) 1 alpha subcomplex, 5 | [NDUFA5](http://www.gene.ucl.ac.uk/cgi-bin/nomenclature/searchgenes.pl?field=symbol&anchor=equals&symbol_search=Search&number=100&format=html&sortby=symbol&match=NDUFA5) | cysteine-type endopeptidase activity;hydrolase activity;cysteine-type peptidase activity | [601677](http://www.ncbi.nlm.nih.gov/entrez/dispomim.cgi?id=601677) | **1.24** |
| 1417287_AT | histocompatibility 13 | [H13](http://www.gene.ucl.ac.uk/cgi-bin/nomenclature/searchgenes.pl?field=symbol&anchor=equals&symbol_search=Search&number=100&format=html&sortby=symbol&match=H13) | cysteine-type endopeptidase activity;peptidase activity |  | **-1.35** |
| 1417301_AT | frizzled homolog 6 (Drosophila) | [FZD6](http://www.gene.ucl.ac.uk/cgi-bin/nomenclature/searchgenes.pl?field=symbol&anchor=equals&symbol_search=Search&number=100&format=html&sortby=symbol&match=FZD6) | cytochrome-b5 reductase activity;oxidoreductase activity | [603409](http://www.ncbi.nlm.nih.gov/entrez/dispomim.cgi?id=603409) | **1.51** |
| 1417307_AT | dystrophin, muscular dystrophy | [DMD](http://www.gene.ucl.ac.uk/cgi-bin/nomenclature/searchgenes.pl?field=symbol&anchor=equals&symbol_search=Search&number=100&format=html&sortby=symbol&match=DMD) | cytochrome-b5 reductase activity;oxidoreductase activity | [300377](http://www.ncbi.nlm.nih.gov/entrez/dispomim.cgi?id=300377) | **3.33** |
| 1417311_AT | cysteine rich protein 2 | [CRIP2](http://www.gene.ucl.ac.uk/cgi-bin/nomenclature/searchgenes.pl?field=symbol&anchor=equals&symbol_search=Search&number=100&format=html&sortby=symbol&match=CRIP2) | cytochrome-c oxidase activity;electron transporter activity;oxidoreductase activity | [601183](http://www.ncbi.nlm.nih.gov/entrez/dispomim.cgi?id=601183) | **-1.2** |
| 1417316_AT | thioesterase superfamily member 2 | [THEM2](http://www.gene.ucl.ac.uk/cgi-bin/nomenclature/searchgenes.pl?field=symbol&anchor=equals&symbol_search=Search&number=100&format=html&sortby=symbol&match=THEM2) | cytochrome-c oxidase activity;oxidoreductase activity |  | **1.68** |
| 1417317_S_AT | ribosomal protein L35a | [RPL35A](http://www.gene.ucl.ac.uk/cgi-bin/nomenclature/searchgenes.pl?field=symbol&anchor=equals&symbol_search=Search&number=100&format=html&sortby=symbol&match=RPL35A) | cytochrome-c oxidase activity;oxidoreductase activity | [180468](http://www.ncbi.nlm.nih.gov/entrez/dispomim.cgi?id=180468) | **-1.35** |
| 1417339_A_AT | dynein, cytoplasmic, light chain 1 | [DNCLC1](http://www.gene.ucl.ac.uk/cgi-bin/nomenclature/searchgenes.pl?field=symbol&anchor=equals&symbol_search=Search&number=100&format=html&sortby=symbol&match=DNCLC1) | cytochrome-c oxidase activity;oxidoreductase activity |  | **-2.49** |
| 1417342_AT | gb:AV236660 /DB_XREF=gi:15405111 /DB_XREF=AV236660 |  | cytochrome-c oxidase activity;oxidoreductase activity |  | **1.36** |
| 1417343_AT | FXYD domain-containing ion transport regulator 6 | [FXYD6](http://www.gene.ucl.ac.uk/cgi-bin/nomenclature/searchgenes.pl?field=symbol&anchor=equals&symbol_search=Search&number=100&format=html&sortby=symbol&match=FXYD6) | cytochrome-c oxidase activity;oxidoreductase activity | [606683](http://www.ncbi.nlm.nih.gov/entrez/dispomim.cgi?id=606683) | **1.56** |
| 1417346_AT | PYD and CARD domain containing | [PYCARD](http://www.gene.ucl.ac.uk/cgi-bin/nomenclature/searchgenes.pl?field=symbol&anchor=equals&symbol_search=Search&number=100&format=html&sortby=symbol&match=PYCARD) | cytochrome-c oxidase activity;oxidoreductase activity | [606838](http://www.ncbi.nlm.nih.gov/entrez/dispomim.cgi?id=606838) | **-3.09** |
| 1417355_AT | Paternally expressed 3 | [PEG3](http://www.gene.ucl.ac.uk/cgi-bin/nomenclature/searchgenes.pl?field=symbol&anchor=equals&symbol_search=Search&number=100&format=html&sortby=symbol&match=PEG3) | cytochrome-c oxidase activity;oxidoreductase activity | [601483](http://www.ncbi.nlm.nih.gov/entrez/dispomim.cgi?id=601483) | **-6.12** |
| 1417356_AT | paternally expressed 3 | [PEG3](http://www.gene.ucl.ac.uk/cgi-bin/nomenclature/searchgenes.pl?field=symbol&anchor=equals&symbol_search=Search&number=100&format=html&sortby=symbol&match=PEG3) | cytochrome-c oxidase activity;receptor activity | [601483](http://www.ncbi.nlm.nih.gov/entrez/dispomim.cgi?id=601483) | **-6.23** |
| 1417358_S_AT | sorbin and SH3 domain containing 1 | [SORBS1](http://www.gene.ucl.ac.uk/cgi-bin/nomenclature/searchgenes.pl?field=symbol&anchor=equals&symbol_search=Search&number=100&format=html&sortby=symbol&match=SORBS1) | cytokine activity | [605264](http://www.ncbi.nlm.nih.gov/entrez/dispomim.cgi?id=605264) | **1.45** |
| 1417369_AT | hydroxysteroid (17-beta) dehydrogenase 4 | [HSD17B4](http://www.gene.ucl.ac.uk/cgi-bin/nomenclature/searchgenes.pl?field=symbol&anchor=equals&symbol_search=Search&number=100&format=html&sortby=symbol&match=HSD17B4) | cytokine activity | [601860](http://www.ncbi.nlm.nih.gov/entrez/dispomim.cgi?id=601860) | **1.57** |
| 1417373_A_AT | tubulin, alpha 4 | [TUBA4](http://www.gene.ucl.ac.uk/cgi-bin/nomenclature/searchgenes.pl?field=symbol&anchor=equals&symbol_search=Search&number=100&format=html&sortby=symbol&match=TUBA4) | cytokine activity;hematopoietin/interferon-class (D200-domain) cytokine receptor binding |  | **1.2** |
| 1417378_AT | immunoglobulin superfamily, member 4A | [IGSF4A](http://www.gene.ucl.ac.uk/cgi-bin/nomenclature/searchgenes.pl?field=symbol&anchor=equals&symbol_search=Search&number=100&format=html&sortby=symbol&match=IGSF4A) | cytoskeletal protein binding |  | **-1.45** |
| 1417379_AT | IQ motif containing GTPase activating protein 1 | [IQGAP1](http://www.gene.ucl.ac.uk/cgi-bin/nomenclature/searchgenes.pl?field=symbol&anchor=equals&symbol_search=Search&number=100&format=html&sortby=symbol&match=IQGAP1) | cytoskeletal protein binding | [603379](http://www.ncbi.nlm.nih.gov/entrez/dispomim.cgi?id=603379) | **-1.94** |
| 1417380_AT | IQ motif containing GTPase activating protein 1 | [IQGAP1](http://www.gene.ucl.ac.uk/cgi-bin/nomenclature/searchgenes.pl?field=symbol&anchor=equals&symbol_search=Search&number=100&format=html&sortby=symbol&match=IQGAP1) | cytoskeletal regulatory protein binding | [603379](http://www.ncbi.nlm.nih.gov/entrez/dispomim.cgi?id=603379) | **-1.59** |
| 1417381_AT | complement component 1, q subcomponent, alpha polypeptide | [C1QA](http://www.gene.ucl.ac.uk/cgi-bin/nomenclature/searchgenes.pl?field=symbol&anchor=equals&symbol_search=Search&number=100&format=html&sortby=symbol&match=C1QA) | D-alanyl-D-alanine endopeptidase activity;hydrolase activity;peptidase activity | [120550](http://www.ncbi.nlm.nih.gov/entrez/dispomim.cgi?id=120550) | **-3.71** |
| 1417383_AT | gb:BB810113 /DB_XREF=gi:16982742 /DB_XREF=BB810113 |  | D-aspartate oxidase activity;oxidoreductase activity;D-amino-acid oxidase activity |  | **1.82** |
| 1417384_AT | ectonucleoside triphosphate diphosphohydrolase 5 | [ENTPD5](http://www.gene.ucl.ac.uk/cgi-bin/nomenclature/searchgenes.pl?field=symbol&anchor=equals&symbol_search=Search&number=100&format=html&sortby=symbol&match=ENTPD5) | deaminase activity | [603162](http://www.ncbi.nlm.nih.gov/entrez/dispomim.cgi?id=603162) | **1.43** |
| 1417389_AT | glypican 1 | [GPC1](http://www.gene.ucl.ac.uk/cgi-bin/nomenclature/searchgenes.pl?field=symbol&anchor=equals&symbol_search=Search&number=100&format=html&sortby=symbol&match=GPC1) | deaminase activity;adenosine deaminase activity;hydrolase activity | [600395](http://www.ncbi.nlm.nih.gov/entrez/dispomim.cgi?id=600395) | **-1.39** |
| 1417392_A_AT | solute carrier family 7 (cationic amino acid transporter, y+ system), member 7 | [SLC7A7](http://www.gene.ucl.ac.uk/cgi-bin/nomenclature/searchgenes.pl?field=symbol&anchor=equals&symbol_search=Search&number=100&format=html&sortby=symbol&match=SLC7A7) | deaminase activity;AMP deaminase activity;hydrolase activity | [603593](http://www.ncbi.nlm.nih.gov/entrez/dispomim.cgi?id=603593) | **-2.02** |
| 1417394_AT | Kruppel-like factor 4 (gut) | [KLF4](http://www.gene.ucl.ac.uk/cgi-bin/nomenclature/searchgenes.pl?field=symbol&anchor=equals&symbol_search=Search&number=100&format=html&sortby=symbol&match=KLF4) | diacylglycerol cholinephosphotransferase activity;diacylglycerol binding;transferase activity | [602253](http://www.ncbi.nlm.nih.gov/entrez/dispomim.cgi?id=602253) | **1.66** |
| 1417395_AT | Kruppel-like factor 4 (gut) | [KLF4](http://www.gene.ucl.ac.uk/cgi-bin/nomenclature/searchgenes.pl?field=symbol&anchor=equals&symbol_search=Search&number=100&format=html&sortby=symbol&match=KLF4) | diacylglycerol cholinephosphotransferase activity;diacylglycerol binding;transferase activity | [602253](http://www.ncbi.nlm.nih.gov/entrez/dispomim.cgi?id=602253) | **1.75** |
| 1417398_AT | related RAS viral (r-ras) oncogene homolog 2 | [RRAS2](http://www.gene.ucl.ac.uk/cgi-bin/nomenclature/searchgenes.pl?field=symbol&anchor=equals&symbol_search=Search&number=100&format=html&sortby=symbol&match=RRAS2) | diacylglycerol cholinephosphotransferase activity;diacylglycerol binding;transferase activity | [600098](http://www.ncbi.nlm.nih.gov/entrez/dispomim.cgi?id=600098) | **-1.83** |
| 1417400_AT | retinoic acid induced 14 | [RAI14](http://www.gene.ucl.ac.uk/cgi-bin/nomenclature/searchgenes.pl?field=symbol&anchor=equals&symbol_search=Search&number=100&format=html&sortby=symbol&match=RAI14) | diacylglycerol cholinephosphotransferase activity;protein binding;diacylglycerol binding | [606586](http://www.ncbi.nlm.nih.gov/entrez/dispomim.cgi?id=606586) | **-1.7** |
| 1417403_AT | ELOVL family member 6, elongation of long chain fatty acids (yeast) | [ELOVL6](http://www.gene.ucl.ac.uk/cgi-bin/nomenclature/searchgenes.pl?field=symbol&anchor=equals&symbol_search=Search&number=100&format=html&sortby=symbol&match=ELOVL6) | diacylglycerol kinase activity;kinase activity;diacylglycerol binding;transferase activity |  | **2.34** |
| 1417404_AT | ELOVL family member 6, elongation of long chain fatty acids (yeast) | [ELOVL6](http://www.gene.ucl.ac.uk/cgi-bin/nomenclature/searchgenes.pl?field=symbol&anchor=equals&symbol_search=Search&number=100&format=html&sortby=symbol&match=ELOVL6) | diacylglycerol O-acyltransferase activity;acyltransferase activity;transferase activity |  | **2.38** |
| 1417408_AT | coagulation factor III | [F3](http://www.gene.ucl.ac.uk/cgi-bin/nomenclature/searchgenes.pl?field=symbol&anchor=equals&symbol_search=Search&number=100&format=html&sortby=symbol&match=F3) | diacylglycerol O-acyltransferase activity;acyltransferase activity;transferase activity | [134390](http://www.ncbi.nlm.nih.gov/entrez/dispomim.cgi?id=134390) | **1.77** |
| 1417409_AT | Jun oncogene | [JUN](http://www.gene.ucl.ac.uk/cgi-bin/nomenclature/searchgenes.pl?field=symbol&anchor=equals&symbol_search=Search&number=100&format=html&sortby=symbol&match=JUN) | diamine N-acetyltransferase activity;N-acetyltransferase activity;acyltransferase activity;transferase activity | [165160](http://www.ncbi.nlm.nih.gov/entrez/dispomim.cgi?id=165160) | **1.46** |
| 1417417_A_AT | cytochrome c oxidase, subunit VI a, polypeptide 1 | [COX6A1](http://www.gene.ucl.ac.uk/cgi-bin/nomenclature/searchgenes.pl?field=symbol&anchor=equals&symbol_search=Search&number=100&format=html&sortby=symbol&match=COX6A1) | dihydrolipoyllysine-residue succinyltransferase activity;acyltransferase activity;transferase activity | [602072](http://www.ncbi.nlm.nih.gov/entrez/dispomim.cgi?id=602072) | **-1.34** |
| 1417419_AT | cyclin D1 | [CCND1](http://www.gene.ucl.ac.uk/cgi-bin/nomenclature/searchgenes.pl?field=symbol&anchor=equals&symbol_search=Search&number=100&format=html&sortby=symbol&match=CCND1) | dipeptidase activity | [168461](http://www.ncbi.nlm.nih.gov/entrez/dispomim.cgi?id=168461) | **-2.58** |
| 1417420_AT | cyclin D1 | [CCND1](http://www.gene.ucl.ac.uk/cgi-bin/nomenclature/searchgenes.pl?field=symbol&anchor=equals&symbol_search=Search&number=100&format=html&sortby=symbol&match=CCND1) | dipeptidase activity;metallopeptidase activity;carboxypeptidase activity;hydrolase activity | [168461](http://www.ncbi.nlm.nih.gov/entrez/dispomim.cgi?id=168461) | **-1.52** |
| 1417421_AT | S100 calcium binding protein A1 | [S100A1](http://www.gene.ucl.ac.uk/cgi-bin/nomenclature/searchgenes.pl?field=symbol&anchor=equals&symbol_search=Search&number=100&format=html&sortby=symbol&match=S100A1) | dipeptidyl-peptidase I activity;cysteine-type endopeptidase activity;hydrolase activity;cysteine-type peptidase activity;peptidase activity | [176940](http://www.ncbi.nlm.nih.gov/entrez/dispomim.cgi?id=176940) | **1.42** |
| 1417432_A_AT | guanine nucleotide binding protein, beta 1 | [GNB1](http://www.gene.ucl.ac.uk/cgi-bin/nomenclature/searchgenes.pl?field=symbol&anchor=equals&symbol_search=Search&number=100&format=html&sortby=symbol&match=GNB1) | dipeptidyl-peptidase III activity;metallopeptidase activity;aminopeptidase activity;hydrolase activity | [139380](http://www.ncbi.nlm.nih.gov/entrez/dispomim.cgi?id=139380) | **-1.54** |
| 1417438_AT | retinol dehydrogenase 14 (all-trans and 9-cis) | [RDH14](http://www.gene.ucl.ac.uk/cgi-bin/nomenclature/searchgenes.pl?field=symbol&anchor=equals&symbol_search=Search&number=100&format=html&sortby=symbol&match=RDH14) | disulfide oxidoreductase activity |  | **1.35** |
| 1417451_A_AT | peptidylprolyl isomerase A | [PPIA](http://www.gene.ucl.ac.uk/cgi-bin/nomenclature/searchgenes.pl?field=symbol&anchor=equals&symbol_search=Search&number=100&format=html&sortby=symbol&match=PPIA) | disulfide oxidoreductase activity;electron-transferring-flavoprotein dehydrogenase activity;oxidoreductase activity | [123840](http://www.ncbi.nlm.nih.gov/entrez/dispomim.cgi?id=123840) | **-1.68** |
| 1417452_A_AT | Finkel-Biskis-Reilly murine sarcoma virus (FBR-MuSV) ubiquitously expressed (fox derived) | [FAU](http://www.gene.ucl.ac.uk/cgi-bin/nomenclature/searchgenes.pl?field=symbol&anchor=equals&symbol_search=Search&number=100&format=html&sortby=symbol&match=FAU) | disulfide oxidoreductase activity;magnesium ion binding;dimethylaniline monooxygenase (N-oxide-forming) activity;oxidoreductase activity;monooxygenase activity | [134690](http://www.ncbi.nlm.nih.gov/entrez/dispomim.cgi?id=134690) | **-1.3** |
| 1417455_AT | transforming growth factor, beta 3 | [TGFB3](http://www.gene.ucl.ac.uk/cgi-bin/nomenclature/searchgenes.pl?field=symbol&anchor=equals&symbol_search=Search&number=100&format=html&sortby=symbol&match=TGFB3) | disulfide oxidoreductase activity;magnesium ion binding;dimethylaniline monooxygenase (N-oxide-forming) activity;oxidoreductase activity;monooxygenase activity | [190230](http://www.ncbi.nlm.nih.gov/entrez/dispomim.cgi?id=190230) | **-1.54** |
| 1417457_AT | CDC28 protein kinase regulatory subunit 2 | [CKS2](http://www.gene.ucl.ac.uk/cgi-bin/nomenclature/searchgenes.pl?field=symbol&anchor=equals&symbol_search=Search&number=100&format=html&sortby=symbol&match=CKS2) | disulfide oxidoreductase activity;magnesium ion binding;dimethylaniline monooxygenase (N-oxide-forming) activity;oxidoreductase activity;monooxygenase activity | [116901](http://www.ncbi.nlm.nih.gov/entrez/dispomim.cgi?id=116901) | **-5.61** |
| 1417458_S_AT | CDC28 protein kinase regulatory subunit 2 | [CKS2](http://www.gene.ucl.ac.uk/cgi-bin/nomenclature/searchgenes.pl?field=symbol&anchor=equals&symbol_search=Search&number=100&format=html&sortby=symbol&match=CKS2) | disulfide oxidoreductase activity;molecular_function unknown;oxidoreductase activity | [116901](http://www.ncbi.nlm.nih.gov/entrez/dispomim.cgi?id=116901) | **-3.07** |
| 1417460_AT | interferon induced transmembrane protein 2 | [IFITM2](http://www.gene.ucl.ac.uk/cgi-bin/nomenclature/searchgenes.pl?field=symbol&anchor=equals&symbol_search=Search&number=100&format=html&sortby=symbol&match=IFITM2) | disulfide oxidoreductase activity;molecular_function unknown;oxidoreductase activity |  | **-1.3** |
| 1417462_AT | CAP, adenylate cyclase-associated protein 1 (yeast) | [CAP1](http://www.gene.ucl.ac.uk/cgi-bin/nomenclature/searchgenes.pl?field=symbol&anchor=equals&symbol_search=Search&number=100&format=html&sortby=symbol&match=CAP1) | disulfide oxidoreductase activity;oxidoreductase activity |  | **-1.45** |
| 1417466_AT | gb:NM_133736.1 /DB_XREF=gi:19526933 /GEN=1110070A0 |  | disulfide oxidoreductase activity;oxidoreductase activity, acting on the CH-CH group of donors;oxidoreductase activity |  | **1.76** |
| 1417470_AT | apolipoprotein B editing complex 3 | [APOBEC3](http://www.gene.ucl.ac.uk/cgi-bin/nomenclature/searchgenes.pl?field=symbol&anchor=equals&symbol_search=Search&number=100&format=html&sortby=symbol&match=APOBEC3) | disulfide oxidoreductase activity;oxidoreductase activity, acting on the CH-CH group of donors;oxidoreductase activity |  | **-1.74** |
| 1417475_AT | ATPase type 13A | [ATP13A1](http://www.gene.ucl.ac.uk/cgi-bin/nomenclature/searchgenes.pl?field=symbol&anchor=equals&symbol_search=Search&number=100&format=html&sortby=symbol&match=ATP13A1) | DNA (cytosine-5-)-methyltransferase activity;protein binding;zinc ion binding;DNA binding;methyltransferase activity;transcription factor binding;catalytic activity;transferase activity |  | **-1.62** |
| 1417476_AT | F-box and WD-40 domain protein 5 | [FBXW5](http://www.gene.ucl.ac.uk/cgi-bin/nomenclature/searchgenes.pl?field=symbol&anchor=equals&symbol_search=Search&number=100&format=html&sortby=symbol&match=FBXW5) | DNA binding | [609072](http://www.ncbi.nlm.nih.gov/entrez/dispomim.cgi?id=609072) | **1.28** |
| 1417481_AT | receptor (calcitonin) activity modifying protein 1 | [RAMP1](http://www.gene.ucl.ac.uk/cgi-bin/nomenclature/searchgenes.pl?field=symbol&anchor=equals&symbol_search=Search&number=100&format=html&sortby=symbol&match=RAMP1) | DNA binding | [605153](http://www.ncbi.nlm.nih.gov/entrez/dispomim.cgi?id=605153) | **1.8** |
| 1417490_AT | cathepsin B | [CTSB](http://www.gene.ucl.ac.uk/cgi-bin/nomenclature/searchgenes.pl?field=symbol&anchor=equals&symbol_search=Search&number=100&format=html&sortby=symbol&match=CTSB) | DNA binding | [116810](http://www.ncbi.nlm.nih.gov/entrez/dispomim.cgi?id=116810) | **-1.54** |
| 1417502_AT | transmembrane 4 superfamily member 2 | [TM4SF2](http://www.gene.ucl.ac.uk/cgi-bin/nomenclature/searchgenes.pl?field=symbol&anchor=equals&symbol_search=Search&number=100&format=html&sortby=symbol&match=TM4SF2) | DNA binding | [300096](http://www.ncbi.nlm.nih.gov/entrez/dispomim.cgi?id=300096) | **1.65** |
| 1417506_AT | geminin | [GMNN](http://www.gene.ucl.ac.uk/cgi-bin/nomenclature/searchgenes.pl?field=symbol&anchor=equals&symbol_search=Search&number=100&format=html&sortby=symbol&match=GMNN) | DNA binding | [602842](http://www.ncbi.nlm.nih.gov/entrez/dispomim.cgi?id=602842) | **2.05** |
| 1417530_A_AT | signal recognition particle 9 | [SRP9](http://www.gene.ucl.ac.uk/cgi-bin/nomenclature/searchgenes.pl?field=symbol&anchor=equals&symbol_search=Search&number=100&format=html&sortby=symbol&match=SRP9) | DNA binding | [600707](http://www.ncbi.nlm.nih.gov/entrez/dispomim.cgi?id=600707) | **-1.25** |
| 1417542_AT | ribosomal protein S6 kinase, polypeptide 2 | [RPS6KA2](http://www.gene.ucl.ac.uk/cgi-bin/nomenclature/searchgenes.pl?field=symbol&anchor=equals&symbol_search=Search&number=100&format=html&sortby=symbol&match=RPS6KA2) | DNA binding | [601685](http://www.ncbi.nlm.nih.gov/entrez/dispomim.cgi?id=601685) | **1.65** |
| 1417543_AT | ribosomal protein S6 kinase, polypeptide 2 | [RPS6KA2](http://www.gene.ucl.ac.uk/cgi-bin/nomenclature/searchgenes.pl?field=symbol&anchor=equals&symbol_search=Search&number=100&format=html&sortby=symbol&match=RPS6KA2) | DNA binding | [601685](http://www.ncbi.nlm.nih.gov/entrez/dispomim.cgi?id=601685) | **1.88** |
| 1417552_AT | fibroblast activation protein | [FAP](http://www.gene.ucl.ac.uk/cgi-bin/nomenclature/searchgenes.pl?field=symbol&anchor=equals&symbol_search=Search&number=100&format=html&sortby=symbol&match=FAP) | DNA binding | [600403](http://www.ncbi.nlm.nih.gov/entrez/dispomim.cgi?id=600403) | **-1.93** |
| 1417557_AT | UBX domain containing 1 | [UBXD1](http://www.gene.ucl.ac.uk/cgi-bin/nomenclature/searchgenes.pl?field=symbol&anchor=equals&symbol_search=Search&number=100&format=html&sortby=symbol&match=UBXD1) | DNA binding |  | **1.26** |
| 1417565_AT | abhydrolase domain containing 5 | [ABHD5](http://www.gene.ucl.ac.uk/cgi-bin/nomenclature/searchgenes.pl?field=symbol&anchor=equals&symbol_search=Search&number=100&format=html&sortby=symbol&match=ABHD5) | DNA binding | [604780](http://www.ncbi.nlm.nih.gov/entrez/dispomim.cgi?id=604780) | **1.47** |
| 1417566_AT | abhydrolase domain containing 5 | [ABHD5](http://www.gene.ucl.ac.uk/cgi-bin/nomenclature/searchgenes.pl?field=symbol&anchor=equals&symbol_search=Search&number=100&format=html&sortby=symbol&match=ABHD5) | DNA binding | [604780](http://www.ncbi.nlm.nih.gov/entrez/dispomim.cgi?id=604780) | **1.41** |
| 1417574_AT | chemokine (C-X-C motif) ligand 12 | [CXCL12](http://www.gene.ucl.ac.uk/cgi-bin/nomenclature/searchgenes.pl?field=symbol&anchor=equals&symbol_search=Search&number=100&format=html&sortby=symbol&match=CXCL12) | DNA binding | [600835](http://www.ncbi.nlm.nih.gov/entrez/dispomim.cgi?id=600835) | **3.17** |
| 1417577_AT | transient receptor potential cation channel, subfamily C, member 3 | [TRPC3](http://www.gene.ucl.ac.uk/cgi-bin/nomenclature/searchgenes.pl?field=symbol&anchor=equals&symbol_search=Search&number=100&format=html&sortby=symbol&match=TRPC3) | DNA binding | [602345](http://www.ncbi.nlm.nih.gov/entrez/dispomim.cgi?id=602345) | **1.55** |
| 1417580_S_AT | selenium binding protein 1 | [SELENBP1](http://www.gene.ucl.ac.uk/cgi-bin/nomenclature/searchgenes.pl?field=symbol&anchor=equals&symbol_search=Search&number=100&format=html&sortby=symbol&match=SELENBP1) | DNA binding | [604188](http://www.ncbi.nlm.nih.gov/entrez/dispomim.cgi?id=604188) | **2.43** |
| 1417592_AT | FK506 binding protein 12-rapamycin associated protein 1 | [FRAP1](http://www.gene.ucl.ac.uk/cgi-bin/nomenclature/searchgenes.pl?field=symbol&anchor=equals&symbol_search=Search&number=100&format=html&sortby=symbol&match=FRAP1) | DNA binding | [601231](http://www.ncbi.nlm.nih.gov/entrez/dispomim.cgi?id=601231) | **1.25** |
| 1417593_AT | tumor suppressor candidate 2 | [TUSC2](http://www.gene.ucl.ac.uk/cgi-bin/nomenclature/searchgenes.pl?field=symbol&anchor=equals&symbol_search=Search&number=100&format=html&sortby=symbol&match=TUSC2) | DNA binding | [607052](http://www.ncbi.nlm.nih.gov/entrez/dispomim.cgi?id=607052) | **1.39** |
| 1417594_AT | G kinase anchoring protein 1 | [GKAP1](http://www.gene.ucl.ac.uk/cgi-bin/nomenclature/searchgenes.pl?field=symbol&anchor=equals&symbol_search=Search&number=100&format=html&sortby=symbol&match=GKAP1) | DNA binding |  | **1.26** |
| 1417595_AT | mesenchyme homeobox 1 | [MEOX1](http://www.gene.ucl.ac.uk/cgi-bin/nomenclature/searchgenes.pl?field=symbol&anchor=equals&symbol_search=Search&number=100&format=html&sortby=symbol&match=MEOX1) | DNA binding | [600147](http://www.ncbi.nlm.nih.gov/entrez/dispomim.cgi?id=600147) | **-2.06** |
| 1417599_AT | B7 homlog 3 | [B7H3](http://www.gene.ucl.ac.uk/cgi-bin/nomenclature/searchgenes.pl?field=symbol&anchor=equals&symbol_search=Search&number=100&format=html&sortby=symbol&match=B7H3) | DNA binding | [605715](http://www.ncbi.nlm.nih.gov/entrez/dispomim.cgi?id=605715) | **-6.35** |
| 1417601_AT | regulator of G-protein signaling 1 | [RGS1](http://www.gene.ucl.ac.uk/cgi-bin/nomenclature/searchgenes.pl?field=symbol&anchor=equals&symbol_search=Search&number=100&format=html&sortby=symbol&match=RGS1) | DNA binding | [600323](http://www.ncbi.nlm.nih.gov/entrez/dispomim.cgi?id=600323) | **-2.78** |
| 1417606_A_AT | calreticulin | [CALR](http://www.gene.ucl.ac.uk/cgi-bin/nomenclature/searchgenes.pl?field=symbol&anchor=equals&symbol_search=Search&number=100&format=html&sortby=symbol&match=CALR) | DNA binding | [109091](http://www.ncbi.nlm.nih.gov/entrez/dispomim.cgi?id=109091) | **-1.25** |
| 1417607_AT | cytochrome c oxidase, subunit VI a, polypeptide 2 | [COX6A2](http://www.gene.ucl.ac.uk/cgi-bin/nomenclature/searchgenes.pl?field=symbol&anchor=equals&symbol_search=Search&number=100&format=html&sortby=symbol&match=COX6A2) | DNA binding | [602009](http://www.ncbi.nlm.nih.gov/entrez/dispomim.cgi?id=602009) | **1.3** |
| 1417608_A_AT | ribosomal protein L13a | [RPL13A](http://www.gene.ucl.ac.uk/cgi-bin/nomenclature/searchgenes.pl?field=symbol&anchor=equals&symbol_search=Search&number=100&format=html&sortby=symbol&match=RPL13A) | DNA binding |  | **-1.35** |
| 1417611_AT | protein distantly related to to the gamma subunit family | [TMEM37](http://www.gene.ucl.ac.uk/cgi-bin/nomenclature/searchgenes.pl?field=symbol&anchor=equals&symbol_search=Search&number=100&format=html&sortby=symbol&match=TMEM37) | DNA binding |  | **-2.34** |
| 1417615_A_AT | ribosomal protein L11 | [RPL11](http://www.gene.ucl.ac.uk/cgi-bin/nomenclature/searchgenes.pl?field=symbol&anchor=equals&symbol_search=Search&number=100&format=html&sortby=symbol&match=RPL11) | DNA binding | [604175](http://www.ncbi.nlm.nih.gov/entrez/dispomim.cgi?id=604175) | **-1.33** |
| 1417619_AT | growth arrest and DNA-damage-inducible, gamma interacting protein 1 | [GADD45GIP1](http://www.gene.ucl.ac.uk/cgi-bin/nomenclature/searchgenes.pl?field=symbol&anchor=equals&symbol_search=Search&number=100&format=html&sortby=symbol&match=GADD45GIP1) | DNA binding | [605162](http://www.ncbi.nlm.nih.gov/entrez/dispomim.cgi?id=605162) | **1.21** |
| 1417620_AT | RAS-related C3 botulinum substrate 2 | [RAC2](http://www.gene.ucl.ac.uk/cgi-bin/nomenclature/searchgenes.pl?field=symbol&anchor=equals&symbol_search=Search&number=100&format=html&sortby=symbol&match=RAC2) | DNA binding | [602049](http://www.ncbi.nlm.nih.gov/entrez/dispomim.cgi?id=602049) | **-1.87** |
| 1417625_S_AT | chemokine orphan receptor 1 | [CMKOR1](http://www.gene.ucl.ac.uk/cgi-bin/nomenclature/searchgenes.pl?field=symbol&anchor=equals&symbol_search=Search&number=100&format=html&sortby=symbol&match=CMKOR1) | DNA binding |  | **1.73** |
| 1417633_AT | superoxide dismutase 3, extracellular | [SOD3](http://www.gene.ucl.ac.uk/cgi-bin/nomenclature/searchgenes.pl?field=symbol&anchor=equals&symbol_search=Search&number=100&format=html&sortby=symbol&match=SOD3) | DNA binding | [185490](http://www.ncbi.nlm.nih.gov/entrez/dispomim.cgi?id=185490) | **1.29** |
| 1417647_AT | sorting nexin 5 | [SNX5](http://www.gene.ucl.ac.uk/cgi-bin/nomenclature/searchgenes.pl?field=symbol&anchor=equals&symbol_search=Search&number=100&format=html&sortby=symbol&match=SNX5) | DNA binding | [605937](http://www.ncbi.nlm.nih.gov/entrez/dispomim.cgi?id=605937) | **-1.48** |
| 1417649_AT | cyclin-dependent kinase inhibitor 1C (P57) | [CDKN1C](http://www.gene.ucl.ac.uk/cgi-bin/nomenclature/searchgenes.pl?field=symbol&anchor=equals&symbol_search=Search&number=100&format=html&sortby=symbol&match=CDKN1C) | DNA binding | [600856](http://www.ncbi.nlm.nih.gov/entrez/dispomim.cgi?id=600856) | **-3.24** |
| 1417660_S_AT | vacuolar protein sorting 29 (S. pombe) | [VPS29](http://www.gene.ucl.ac.uk/cgi-bin/nomenclature/searchgenes.pl?field=symbol&anchor=equals&symbol_search=Search&number=100&format=html&sortby=symbol&match=VPS29) | DNA binding | [606932](http://www.ncbi.nlm.nih.gov/entrez/dispomim.cgi?id=606932) | **-1.27** |
| 1417661_AT | RAD52 homolog B (S. cerevisiae) | [RAD52B](http://www.gene.ucl.ac.uk/cgi-bin/nomenclature/searchgenes.pl?field=symbol&anchor=equals&symbol_search=Search&number=100&format=html&sortby=symbol&match=RAD52B) | DNA binding |  | **1.36** |
| 1417662_AT | gb:BI697630 /DB_XREF=gi:15660259 /DB_XREF=60334880 |  | DNA binding |  | **1.56** |
| 1417668_AT | reticulon 4 interacting protein 1 | [RTN4IP1](http://www.gene.ucl.ac.uk/cgi-bin/nomenclature/searchgenes.pl?field=symbol&anchor=equals&symbol_search=Search&number=100&format=html&sortby=symbol&match=RTN4IP1) | DNA binding |  | **1.46** |
| 1417673_AT | growth factor receptor bound protein 14 | [GRB14](http://www.gene.ucl.ac.uk/cgi-bin/nomenclature/searchgenes.pl?field=symbol&anchor=equals&symbol_search=Search&number=100&format=html&sortby=symbol&match=GRB14) | DNA binding | [601524](http://www.ncbi.nlm.nih.gov/entrez/dispomim.cgi?id=601524) | **3.08** |
| 1417674_S_AT | golgi autoantigen, golgin subfamily a, 4 | [GOLGA4](http://www.gene.ucl.ac.uk/cgi-bin/nomenclature/searchgenes.pl?field=symbol&anchor=equals&symbol_search=Search&number=100&format=html&sortby=symbol&match=GOLGA4) | DNA binding | [602509](http://www.ncbi.nlm.nih.gov/entrez/dispomim.cgi?id=602509) | **1.32** |
| 1417676_A_AT | protein tyrosine phosphatase, receptor type, O | [PTPRO](http://www.gene.ucl.ac.uk/cgi-bin/nomenclature/searchgenes.pl?field=symbol&anchor=equals&symbol_search=Search&number=100&format=html&sortby=symbol&match=PTPRO) | DNA binding | [600579](http://www.ncbi.nlm.nih.gov/entrez/dispomim.cgi?id=600579) | **-1.79** |
| 1417680_AT | potassium voltage-gated channel, shaker-related subfamily, member 5 | [KCNA5](http://www.gene.ucl.ac.uk/cgi-bin/nomenclature/searchgenes.pl?field=symbol&anchor=equals&symbol_search=Search&number=100&format=html&sortby=symbol&match=KCNA5) | DNA binding | [176267](http://www.ncbi.nlm.nih.gov/entrez/dispomim.cgi?id=176267) | **1.84** |
| 1417681_AT | cleavage and polyadenylation specific factor 5 | [CPSF5](http://www.gene.ucl.ac.uk/cgi-bin/nomenclature/searchgenes.pl?field=symbol&anchor=equals&symbol_search=Search&number=100&format=html&sortby=symbol&match=CPSF5) | DNA binding | [604978](http://www.ncbi.nlm.nih.gov/entrez/dispomim.cgi?id=604978) | **-1.22** |
| 1417688_AT | cDNA sequence BC004044 | [BC004044](http://www.gene.ucl.ac.uk/cgi-bin/nomenclature/searchgenes.pl?field=symbol&anchor=equals&symbol_search=Search&number=100&format=html&sortby=symbol&match=BC004044) | DNA binding |  | **-1.51** |
| 1417690_AT | protein kinase, AMP-activated, gamma 1 non-catalytic subunit | [PRKAG1](http://www.gene.ucl.ac.uk/cgi-bin/nomenclature/searchgenes.pl?field=symbol&anchor=equals&symbol_search=Search&number=100&format=html&sortby=symbol&match=PRKAG1) | DNA binding | [602742](http://www.ncbi.nlm.nih.gov/entrez/dispomim.cgi?id=602742) | **1.42** |
| 1417695_A_AT | sterol O-acyltransferase 1 | [SOAT1](http://www.gene.ucl.ac.uk/cgi-bin/nomenclature/searchgenes.pl?field=symbol&anchor=equals&symbol_search=Search&number=100&format=html&sortby=symbol&match=SOAT1) | DNA binding | [102642](http://www.ncbi.nlm.nih.gov/entrez/dispomim.cgi?id=102642) | **-2.78** |
| 1417696_AT | sterol O-acyltransferase 1 | [SOAT1](http://www.gene.ucl.ac.uk/cgi-bin/nomenclature/searchgenes.pl?field=symbol&anchor=equals&symbol_search=Search&number=100&format=html&sortby=symbol&match=SOAT1) | DNA binding | [102642](http://www.ncbi.nlm.nih.gov/entrez/dispomim.cgi?id=102642) | **-3.65** |
| 1417697_AT | sterol O-acyltransferase 1 | [SOAT1](http://www.gene.ucl.ac.uk/cgi-bin/nomenclature/searchgenes.pl?field=symbol&anchor=equals&symbol_search=Search&number=100&format=html&sortby=symbol&match=SOAT1) | DNA binding;actin binding;transcription factor activity;transcriptional repressor activity | [102642](http://www.ncbi.nlm.nih.gov/entrez/dispomim.cgi?id=102642) | **-1.63** |
| 1417702_A_AT | histamine N-methyltransferase | [HNMT](http://www.gene.ucl.ac.uk/cgi-bin/nomenclature/searchgenes.pl?field=symbol&anchor=equals&symbol_search=Search&number=100&format=html&sortby=symbol&match=HNMT) | DNA binding;acyltransferase activity;transferase activity | [605238](http://www.ncbi.nlm.nih.gov/entrez/dispomim.cgi?id=605238) | **1.99** |
| 1417714_X_AT | hemoglobin alpha, adult chain 1 | [HBA-A1](http://www.gene.ucl.ac.uk/cgi-bin/nomenclature/searchgenes.pl?field=symbol&anchor=equals&symbol_search=Search&number=100&format=html&sortby=symbol&match=HBA-A1) | DNA binding;ARF GTPase activator activity;GTPase activator activity;protein transporter activity | [141800](http://www.ncbi.nlm.nih.gov/entrez/dispomim.cgi?id=141800) | **5.05** |
| 1417715_A_AT | glutamate oxaloacetate transaminase 2, mitochondrial | [GOT2](http://www.gene.ucl.ac.uk/cgi-bin/nomenclature/searchgenes.pl?field=symbol&anchor=equals&symbol_search=Search&number=100&format=html&sortby=symbol&match=GOT2) | DNA binding;cation transporter activity | [138150](http://www.ncbi.nlm.nih.gov/entrez/dispomim.cgi?id=138150) | **1.27** |
| 1417716_AT | glutamate oxaloacetate transaminase 2, mitochondrial | [GOT2](http://www.gene.ucl.ac.uk/cgi-bin/nomenclature/searchgenes.pl?field=symbol&anchor=equals&symbol_search=Search&number=100&format=html&sortby=symbol&match=GOT2) | DNA binding;chromatin binding | [138150](http://www.ncbi.nlm.nih.gov/entrez/dispomim.cgi?id=138150) | **1.5** |
| 1417719_AT | sin3 associated polypeptide | [SAP30](http://www.gene.ucl.ac.uk/cgi-bin/nomenclature/searchgenes.pl?field=symbol&anchor=equals&symbol_search=Search&number=100&format=html&sortby=symbol&match=SAP30) | DNA binding;chromatin binding | [603378](http://www.ncbi.nlm.nih.gov/entrez/dispomim.cgi?id=603378) | **-3.95** |
| 1417721_S_AT | lysosomal-associated protein transmembrane 5 | [LAPTM5](http://www.gene.ucl.ac.uk/cgi-bin/nomenclature/searchgenes.pl?field=symbol&anchor=equals&symbol_search=Search&number=100&format=html&sortby=symbol&match=LAPTM5) | DNA binding;chromatin binding | [601476](http://www.ncbi.nlm.nih.gov/entrez/dispomim.cgi?id=601476) | **-1.98** |
| 1417724_AT | THO complex 4 | [THOC4](http://www.gene.ucl.ac.uk/cgi-bin/nomenclature/searchgenes.pl?field=symbol&anchor=equals&symbol_search=Search&number=100&format=html&sortby=symbol&match=THOC4) | DNA binding;chromatin binding | [604171](http://www.ncbi.nlm.nih.gov/entrez/dispomim.cgi?id=604171) | **-1.31** |
| 1417727_AT | splicing factor, arginine/serine rich 9 | [SFRS9](http://www.gene.ucl.ac.uk/cgi-bin/nomenclature/searchgenes.pl?field=symbol&anchor=equals&symbol_search=Search&number=100&format=html&sortby=symbol&match=SFRS9) | DNA binding;DNA topoisomerase (ATP-hydrolyzing) activity;GTP binding;ATP binding | [601943](http://www.ncbi.nlm.nih.gov/entrez/dispomim.cgi?id=601943) | **-1.22** |
| 1417730_AT | exostoses (multiple) 1 | [EXT1](http://www.gene.ucl.ac.uk/cgi-bin/nomenclature/searchgenes.pl?field=symbol&anchor=equals&symbol_search=Search&number=100&format=html&sortby=symbol&match=EXT1) | DNA binding;DNA-dependent ATPase activity;ATP binding | [608177](http://www.ncbi.nlm.nih.gov/entrez/dispomim.cgi?id=608177) | **-1.69** |
| 1417743_AT | CDC-like kinase 2 | [CLK2](http://www.gene.ucl.ac.uk/cgi-bin/nomenclature/searchgenes.pl?field=symbol&anchor=equals&symbol_search=Search&number=100&format=html&sortby=symbol&match=CLK2) | DNA binding;DNA-dependent ATPase activity;ATP binding | [602989](http://www.ncbi.nlm.nih.gov/entrez/dispomim.cgi?id=602989) | **-1.35** |
| 1417749_A_AT | tight junction protein 1 | [TJP1](http://www.gene.ucl.ac.uk/cgi-bin/nomenclature/searchgenes.pl?field=symbol&anchor=equals&symbol_search=Search&number=100&format=html&sortby=symbol&match=TJP1) | DNA binding;DNA-dependent ATPase activity;ATP binding | [601009](http://www.ncbi.nlm.nih.gov/entrez/dispomim.cgi?id=601009) | **1.39** |
| 1417756_A_AT | lymphocyte specific 1 | [LSP1](http://www.gene.ucl.ac.uk/cgi-bin/nomenclature/searchgenes.pl?field=symbol&anchor=equals&symbol_search=Search&number=100&format=html&sortby=symbol&match=LSP1) | DNA binding;DNA-dependent ATPase activity;ATP binding | [153432](http://www.ncbi.nlm.nih.gov/entrez/dispomim.cgi?id=153432) | **-2.46** |
| 1417763_AT | signal sequence receptor, alpha | [SSR1](http://www.gene.ucl.ac.uk/cgi-bin/nomenclature/searchgenes.pl?field=symbol&anchor=equals&symbol_search=Search&number=100&format=html&sortby=symbol&match=SSR1) | DNA binding;DNA-directed RNA polymerase activity;protein dimerization activity;transferase activity | [600868](http://www.ncbi.nlm.nih.gov/entrez/dispomim.cgi?id=600868) | **-1.4** |
| 1417765_A_AT | amylase 1, salivary | [AMY1](http://www.gene.ucl.ac.uk/cgi-bin/nomenclature/searchgenes.pl?field=symbol&anchor=equals&symbol_search=Search&number=100&format=html&sortby=symbol&match=AMY1) | DNA binding;DNA-directed RNA polymerase activity;transferase activity |  | **3.34** |
| 1417767_AT | RIKEN cDNA 1810044O22 gene | [1810044O22RIK](http://www.gene.ucl.ac.uk/cgi-bin/nomenclature/searchgenes.pl?field=symbol&anchor=equals&symbol_search=Search&number=100&format=html&sortby=symbol&match=1810044O22RIK) | DNA binding;GTPase activator activity |  | **1.31** |
| 1417768_AT | RIKEN cDNA 1200006O19 gene | [1200006O19RIK](http://www.gene.ucl.ac.uk/cgi-bin/nomenclature/searchgenes.pl?field=symbol&anchor=equals&symbol_search=Search&number=100&format=html&sortby=symbol&match=1200006O19RIK) | DNA binding;GTPase activity;GTP binding;protein transporter activity |  | **1.34** |
| 1417773_AT | N-acetylneuraminic acid synthase (sialic acid synthase) | [NANS](http://www.gene.ucl.ac.uk/cgi-bin/nomenclature/searchgenes.pl?field=symbol&anchor=equals&symbol_search=Search&number=100&format=html&sortby=symbol&match=NANS) | DNA binding;GTPase activity;GTP binding;protein transporter activity | [605202](http://www.ncbi.nlm.nih.gov/entrez/dispomim.cgi?id=605202) | **-1.62** |
| 1417774_AT | N-acetylneuraminic acid synthase (sialic acid synthase) | [NANS](http://www.gene.ucl.ac.uk/cgi-bin/nomenclature/searchgenes.pl?field=symbol&anchor=equals&symbol_search=Search&number=100&format=html&sortby=symbol&match=NANS) | DNA binding;hydrolase activity | [605202](http://www.ncbi.nlm.nih.gov/entrez/dispomim.cgi?id=605202) | **-1.56** |
| 1417777_AT | leukotriene B4 12-hydroxydehydrogenase | [LTB4DH](http://www.gene.ucl.ac.uk/cgi-bin/nomenclature/searchgenes.pl?field=symbol&anchor=equals&symbol_search=Search&number=100&format=html&sortby=symbol&match=LTB4DH) | DNA binding;hydrolase activity | [601274](http://www.ncbi.nlm.nih.gov/entrez/dispomim.cgi?id=601274) | **-1.78** |
| 1417786_A_AT | regulator of G-protein signaling 19 | [RGS19](http://www.gene.ucl.ac.uk/cgi-bin/nomenclature/searchgenes.pl?field=symbol&anchor=equals&symbol_search=Search&number=100&format=html&sortby=symbol&match=RGS19) | DNA binding;hydrolase activity | [605071](http://www.ncbi.nlm.nih.gov/entrez/dispomim.cgi?id=605071) | **-1.78** |
| 1417789_AT | small chemokine (C-C motif) ligand 11 | [CCL11](http://www.gene.ucl.ac.uk/cgi-bin/nomenclature/searchgenes.pl?field=symbol&anchor=equals&symbol_search=Search&number=100&format=html&sortby=symbol&match=CCL11) | DNA binding;hydrolase activity | [601156](http://www.ncbi.nlm.nih.gov/entrez/dispomim.cgi?id=601156) | **3** |
| 1417793_AT | expressed sequence AI481100 | [IIGP2](http://www.gene.ucl.ac.uk/cgi-bin/nomenclature/searchgenes.pl?field=symbol&anchor=equals&symbol_search=Search&number=100&format=html&sortby=symbol&match=IIGP2) | DNA binding;hydrolase activity |  | **-1.3** |
| 1417796_AT | G protein pathway suppressor 2 | [GPS2](http://www.gene.ucl.ac.uk/cgi-bin/nomenclature/searchgenes.pl?field=symbol&anchor=equals&symbol_search=Search&number=100&format=html&sortby=symbol&match=GPS2) | DNA binding;integrase activity |  | **1.37** |
| 1417804_AT | RAS, guanyl releasing protein 2 | [RASGRP2](http://www.gene.ucl.ac.uk/cgi-bin/nomenclature/searchgenes.pl?field=symbol&anchor=equals&symbol_search=Search&number=100&format=html&sortby=symbol&match=RASGRP2) | DNA binding;kinase activity;cyclin-dependent protein kinase inhibitor activity;transcription factor activity | [605577](http://www.ncbi.nlm.nih.gov/entrez/dispomim.cgi?id=605577) | **1.87** |
| 1417810_A_AT | protein kinase C and casein kinase substrate in neurons 2 | [KCNB1](http://www.gene.ucl.ac.uk/cgi-bin/nomenclature/searchgenes.pl?field=symbol&anchor=equals&symbol_search=Search&number=100&format=html&sortby=symbol&match=KCNB1) | DNA binding;kinase activity;transcription factor activity;GTP binding;ATP binding;transferase activity | [600397](http://www.ncbi.nlm.nih.gov/entrez/dispomim.cgi?id=600397) | **1.24** |
| 1417811_AT | solute carrier family 24 (sodium/potassium/calcium exchanger), member 6 | [SLC24A6](http://www.gene.ucl.ac.uk/cgi-bin/nomenclature/searchgenes.pl?field=symbol&anchor=equals&symbol_search=Search&number=100&format=html&sortby=symbol&match=SLC24A6) | DNA binding;lambda DNA polymerase activity;beta DNA polymerase activity;nucleotidyltransferase activity;manganese ion binding;lyase activity;DNA-directed DNA polymerase activity;transferase activity |  | **-1.36** |
| 1417821_AT | DNA segment, Chr 17, human D6S56E 5 | [D17H6S56E-5](http://www.gene.ucl.ac.uk/cgi-bin/nomenclature/searchgenes.pl?field=symbol&anchor=equals&symbol_search=Search&number=100&format=html&sortby=symbol&match=D17H6S56E-5) | DNA binding;N-methyltransferase activity |  | **-2.46** |
| 1417822_AT | DNA segment, Chr 17, human D6S56E 5 | [D17H6S56E-5](http://www.gene.ucl.ac.uk/cgi-bin/nomenclature/searchgenes.pl?field=symbol&anchor=equals&symbol_search=Search&number=100&format=html&sortby=symbol&match=D17H6S56E-5) | DNA binding;nucleic acid binding;damaged DNA binding |  | **-2.05** |
| 1417823_AT | glycine C-acetyltransferase (2-amino-3-ketobutyrate-coenzyme A ligase) | [GCAT](http://www.gene.ucl.ac.uk/cgi-bin/nomenclature/searchgenes.pl?field=symbol&anchor=equals&symbol_search=Search&number=100&format=html&sortby=symbol&match=GCAT) | DNA binding;nucleic acid binding;RNA binding | [607422](http://www.ncbi.nlm.nih.gov/entrez/dispomim.cgi?id=607422) | **2.18** |
| 1417825_AT | esterase D/formylglutathione hydrolase | [ESD](http://www.gene.ucl.ac.uk/cgi-bin/nomenclature/searchgenes.pl?field=symbol&anchor=equals&symbol_search=Search&number=100&format=html&sortby=symbol&match=ESD) | DNA binding;nucleic acid binding;transcription factor activity | [133280](http://www.ncbi.nlm.nih.gov/entrez/dispomim.cgi?id=133280) | **-1.33** |
| 1417830_AT | SMC (structural maintenance of chromosomes 1)-like 1 (S. cerevisiae) | [SMC1L1](http://www.gene.ucl.ac.uk/cgi-bin/nomenclature/searchgenes.pl?field=symbol&anchor=equals&symbol_search=Search&number=100&format=html&sortby=symbol&match=SMC1L1) | DNA binding;nucleotide binding;ATP binding | [300040](http://www.ncbi.nlm.nih.gov/entrez/dispomim.cgi?id=300040) | **-1.33** |
| 1417839_AT | claudin 5 | [CLDN5](http://www.gene.ucl.ac.uk/cgi-bin/nomenclature/searchgenes.pl?field=symbol&anchor=equals&symbol_search=Search&number=100&format=html&sortby=symbol&match=CLDN5) | DNA binding;nucleotide binding;DNA-dependent ATPase activity;ATP binding | [602101](http://www.ncbi.nlm.nih.gov/entrez/dispomim.cgi?id=602101) | **1.58** |
| 1417841_AT | gb:AF309644.1 /DB_XREF=gi:10954088 /GEN=Pxmp2 /FEA |  | DNA binding;nucleotide binding;DNA-dependent ATPase activity;ATP binding;nucleoside-triphosphatase activity |  | **1.46** |
| 1417846_AT | Unc-51 like kinase 2 (C. elegans) | [ULK2](http://www.gene.ucl.ac.uk/cgi-bin/nomenclature/searchgenes.pl?field=symbol&anchor=equals&symbol_search=Search&number=100&format=html&sortby=symbol&match=ULK2) | DNA binding;nucleotide binding;DNA-dependent ATPase activity;nucleoside-triphosphatase activity;ATP binding | [608650](http://www.ncbi.nlm.nih.gov/entrez/dispomim.cgi?id=608650) | **1.68** |
| 1417847_AT | Unc-51 like kinase 2 (C. elegans) | [ULK2](http://www.gene.ucl.ac.uk/cgi-bin/nomenclature/searchgenes.pl?field=symbol&anchor=equals&symbol_search=Search&number=100&format=html&sortby=symbol&match=ULK2) | DNA binding;nucleotide binding;transcription factor activity;ATP binding;nucleoside-triphosphatase activity | [608650](http://www.ncbi.nlm.nih.gov/entrez/dispomim.cgi?id=608650) | **1.35** |
| 1417848_AT | glucocorticoid induced gene 1 | [GIG1](http://www.gene.ucl.ac.uk/cgi-bin/nomenclature/searchgenes.pl?field=symbol&anchor=equals&symbol_search=Search&number=100&format=html&sortby=symbol&match=GIG1) | DNA binding;nucleotide binding;transcription factor activity;ATP binding;nucleoside-triphosphatase activity |  | **-1.37** |
| 1417850_AT | retinoblastoma 1 | [RB1](http://www.gene.ucl.ac.uk/cgi-bin/nomenclature/searchgenes.pl?field=symbol&anchor=equals&symbol_search=Search&number=100&format=html&sortby=symbol&match=RB1) | DNA binding;receptor activity;transcription factor activity | [180200](http://www.ncbi.nlm.nih.gov/entrez/dispomim.cgi?id=180200) | **-1.59** |
| 1417865_AT | tumor necrosis factor, alpha-induced protein 1 (endothelial) | [TNFAIP1](http://www.gene.ucl.ac.uk/cgi-bin/nomenclature/searchgenes.pl?field=symbol&anchor=equals&symbol_search=Search&number=100&format=html&sortby=symbol&match=TNFAIP1) | DNA binding;RNA binding | [191161](http://www.ncbi.nlm.nih.gov/entrez/dispomim.cgi?id=191161) | **1.3** |
| 1417866_AT | tumor necrosis factor, alpha-induced protein 1 (endothelial) | [TNFAIP1](http://www.gene.ucl.ac.uk/cgi-bin/nomenclature/searchgenes.pl?field=symbol&anchor=equals&symbol_search=Search&number=100&format=html&sortby=symbol&match=TNFAIP1) | DNA binding;RNA binding | [191161](http://www.ncbi.nlm.nih.gov/entrez/dispomim.cgi?id=191161) | **1.27** |
| 1417868_A_AT | cathepsin Z | [CTSZ](http://www.gene.ucl.ac.uk/cgi-bin/nomenclature/searchgenes.pl?field=symbol&anchor=equals&symbol_search=Search&number=100&format=html&sortby=symbol&match=CTSZ) | DNA binding;RNA binding | [603169](http://www.ncbi.nlm.nih.gov/entrez/dispomim.cgi?id=603169) | **-2.6** |
| 1417869_S_AT | cathepsin Z | [CTSZ](http://www.gene.ucl.ac.uk/cgi-bin/nomenclature/searchgenes.pl?field=symbol&anchor=equals&symbol_search=Search&number=100&format=html&sortby=symbol&match=CTSZ) | DNA binding;RNA binding | [603169](http://www.ncbi.nlm.nih.gov/entrez/dispomim.cgi?id=603169) | **-2.64** |
| 1417870_X_AT | cathepsin Z | [CTSZ](http://www.gene.ucl.ac.uk/cgi-bin/nomenclature/searchgenes.pl?field=symbol&anchor=equals&symbol_search=Search&number=100&format=html&sortby=symbol&match=CTSZ) | DNA binding;RNA binding | [603169](http://www.ncbi.nlm.nih.gov/entrez/dispomim.cgi?id=603169) | **-2.3** |
| 1417876_AT | Fc receptor, IgG, high affinity I | [FCGR1](http://www.gene.ucl.ac.uk/cgi-bin/nomenclature/searchgenes.pl?field=symbol&anchor=equals&symbol_search=Search&number=100&format=html&sortby=symbol&match=FCGR1) | DNA binding;RNA binding |  | **-3.24** |
| 1417877_AT | RIKEN cDNA 2310005P05 gene | [2310005P05RIK](http://www.gene.ucl.ac.uk/cgi-bin/nomenclature/searchgenes.pl?field=symbol&anchor=equals&symbol_search=Search&number=100&format=html&sortby=symbol&match=2310005P05RIK) | DNA binding;RNA polymerase II transcription factor activity |  | **1.84** |
| 1417881_AT | solute carrier family 39 (zinc transporter), member 3 | [SLC39A3](http://www.gene.ucl.ac.uk/cgi-bin/nomenclature/searchgenes.pl?field=symbol&anchor=equals&symbol_search=Search&number=100&format=html&sortby=symbol&match=SLC39A3) | DNA binding;RNA polymerase II transcription factor activity, enhancer binding |  | **1.42** |
| 1417886_AT | RIKEN cDNA 1810009A15 gene | [1810009A15RIK](http://www.gene.ucl.ac.uk/cgi-bin/nomenclature/searchgenes.pl?field=symbol&anchor=equals&symbol_search=Search&number=100&format=html&sortby=symbol&match=1810009A15RIK) | DNA binding;structural constituent of ribosome |  | **-1.36** |
| 1417911_AT | cyclin A2 | [CCNA2](http://www.gene.ucl.ac.uk/cgi-bin/nomenclature/searchgenes.pl?field=symbol&anchor=equals&symbol_search=Search&number=100&format=html&sortby=symbol&match=CCNA2) | DNA binding;structural constituent of ribosome | [123835](http://www.ncbi.nlm.nih.gov/entrez/dispomim.cgi?id=123835) | **-3.02** |
| 1417914_AT | RAP2B, member of RAS oncogene family | [RAP2B](http://www.gene.ucl.ac.uk/cgi-bin/nomenclature/searchgenes.pl?field=symbol&anchor=equals&symbol_search=Search&number=100&format=html&sortby=symbol&match=RAP2B) | DNA binding;structural constituent of ribosome | [179541](http://www.ncbi.nlm.nih.gov/entrez/dispomim.cgi?id=179541) | **-1.88** |
| 1417916_A_AT | fractured callus expressed transcript 1 | [FXC1](http://www.gene.ucl.ac.uk/cgi-bin/nomenclature/searchgenes.pl?field=symbol&anchor=equals&symbol_search=Search&number=100&format=html&sortby=symbol&match=FXC1) | DNA binding;sugar binding;transcription factor activity;catalytic activity | [607388](http://www.ncbi.nlm.nih.gov/entrez/dispomim.cgi?id=607388) | **-1.27** |
| 1417921_AT | RIKEN cDNA 2610029G23 gene | [2610029G23RIK](http://www.gene.ucl.ac.uk/cgi-bin/nomenclature/searchgenes.pl?field=symbol&anchor=equals&symbol_search=Search&number=100&format=html&sortby=symbol&match=2610029G23RIK) | DNA binding;transcription corepressor activity;transcription factor activity |  | **-1.58** |
| 1417926_AT | RIKEN cDNA 5830426I05 gene | [5830426I05RIK](http://www.gene.ucl.ac.uk/cgi-bin/nomenclature/searchgenes.pl?field=symbol&anchor=equals&symbol_search=Search&number=100&format=html&sortby=symbol&match=5830426I05RIK) | DNA binding;transcription corepressor activity;transcription factor activity |  | **-2.09** |
| 1417928_AT | PDZ and LIM domain 4 | [PDLIM4](http://www.gene.ucl.ac.uk/cgi-bin/nomenclature/searchgenes.pl?field=symbol&anchor=equals&symbol_search=Search&number=100&format=html&sortby=symbol&match=PDLIM4) | DNA binding;transcription factor activity | [603422](http://www.ncbi.nlm.nih.gov/entrez/dispomim.cgi?id=603422) | **-2.17** |
| 1417930_AT | gb:NM_008668.1 /DB_XREF=gi:6679003 /GEN=Nab2 /FEA= |  | DNA binding;transcription factor activity |  | **-1.46** |
| 1417933_AT | insulin-like growth factor binding protein 6 | [IGFBP6](http://www.gene.ucl.ac.uk/cgi-bin/nomenclature/searchgenes.pl?field=symbol&anchor=equals&symbol_search=Search&number=100&format=html&sortby=symbol&match=IGFBP6) | DNA binding;transcription factor activity | [146735](http://www.ncbi.nlm.nih.gov/entrez/dispomim.cgi?id=146735) | **1.3** |
| 1417935_AT | makorin, ring finger protein, 2 | [LOC212815](http://www.gene.ucl.ac.uk/cgi-bin/nomenclature/searchgenes.pl?field=symbol&anchor=equals&symbol_search=Search&number=100&format=html&sortby=symbol&match=LOC212815) | DNA binding;transcription factor activity | [608426](http://www.ncbi.nlm.nih.gov/entrez/dispomim.cgi?id=608426) | **1.28** |
| 1417936_AT | gb:NM_011338.1 /DB_XREF=gi:6755433 /GEN=Scya9 /FEA |  | DNA binding;transcription factor activity |  | **-2.61** |
| 1417948_S_AT | interleukin enhancer binding factor 2 | [ILF2](http://www.gene.ucl.ac.uk/cgi-bin/nomenclature/searchgenes.pl?field=symbol&anchor=equals&symbol_search=Search&number=100&format=html&sortby=symbol&match=ILF2) | DNA binding;transcription factor activity | [603181](http://www.ncbi.nlm.nih.gov/entrez/dispomim.cgi?id=603181) | **-1.43** |
| 1417956_AT | cell death-inducing DNA fragmentation factor, alpha subunit-like effector A | [CIDEA](http://www.gene.ucl.ac.uk/cgi-bin/nomenclature/searchgenes.pl?field=symbol&anchor=equals&symbol_search=Search&number=100&format=html&sortby=symbol&match=CIDEA) | DNA binding;transcription factor activity | [604440](http://www.ncbi.nlm.nih.gov/entrez/dispomim.cgi?id=604440) | **1.71** |
| 1417969_AT | F-box only protein 31 | [FBXO31](http://www.gene.ucl.ac.uk/cgi-bin/nomenclature/searchgenes.pl?field=symbol&anchor=equals&symbol_search=Search&number=100&format=html&sortby=symbol&match=FBXO31) | DNA binding;transcription factor activity | [609102](http://www.ncbi.nlm.nih.gov/entrez/dispomim.cgi?id=609102) | **1.72** |
| 1417970_AT | ATP synthase, H+ transporting, mitochondrial F0 complex, subunit s | [ATP5S](http://www.gene.ucl.ac.uk/cgi-bin/nomenclature/searchgenes.pl?field=symbol&anchor=equals&symbol_search=Search&number=100&format=html&sortby=symbol&match=ATP5S) | DNA binding;transcription factor activity |  | **1.46** |
| 1417976_AT | adenosine deaminase | [ADA](http://www.gene.ucl.ac.uk/cgi-bin/nomenclature/searchgenes.pl?field=symbol&anchor=equals&symbol_search=Search&number=100&format=html&sortby=symbol&match=ADA) | DNA binding;transcription factor activity | 608958 102700 | **-1.84** |
| 1417979_AT | tenomodulin | [TNMD](http://www.gene.ucl.ac.uk/cgi-bin/nomenclature/searchgenes.pl?field=symbol&anchor=equals&symbol_search=Search&number=100&format=html&sortby=symbol&match=TNMD) | DNA binding;transcription factor activity | [300459](http://www.ncbi.nlm.nih.gov/entrez/dispomim.cgi?id=300459) | **-2.54** |
| 1417998_AT | telomerase binding protein, p23 | [TEBP](http://www.gene.ucl.ac.uk/cgi-bin/nomenclature/searchgenes.pl?field=symbol&anchor=equals&symbol_search=Search&number=100&format=html&sortby=symbol&match=TEBP) | DNA binding;transcription factor activity | [607061](http://www.ncbi.nlm.nih.gov/entrez/dispomim.cgi?id=607061) | **-1.25** |
| 1418002_AT | RIKEN cDNA 2010110M21 gene | [2010110M21RIK](http://www.gene.ucl.ac.uk/cgi-bin/nomenclature/searchgenes.pl?field=symbol&anchor=equals&symbol_search=Search&number=100&format=html&sortby=symbol&match=2010110M21RIK) | DNA binding;transcription factor activity |  | **1.25** |
| 1418004_A_AT | RIKEN cDNA 1810009M01 gene | [1810009M01RIK](http://www.gene.ucl.ac.uk/cgi-bin/nomenclature/searchgenes.pl?field=symbol&anchor=equals&symbol_search=Search&number=100&format=html&sortby=symbol&match=1810009M01RIK) | DNA binding;transcription factor activity |  | **-1.92** |
| 1418005_AT | succinate dehydrogenase complex, subunit B, iron sulfur (Ip) | [SDHB](http://www.gene.ucl.ac.uk/cgi-bin/nomenclature/searchgenes.pl?field=symbol&anchor=equals&symbol_search=Search&number=100&format=html&sortby=symbol&match=SDHB) | DNA binding;transcription factor activity | [185470](http://www.ncbi.nlm.nih.gov/entrez/dispomim.cgi?id=185470) | **1.38** |
| 1418020_S_AT | carboxypeptidase D | [CPD](http://www.gene.ucl.ac.uk/cgi-bin/nomenclature/searchgenes.pl?field=symbol&anchor=equals&symbol_search=Search&number=100&format=html&sortby=symbol&match=CPD) | DNA binding;transcription factor activity | [603102](http://www.ncbi.nlm.nih.gov/entrez/dispomim.cgi?id=603102) | **-1.3** |
| 1418031_AT | myosin IXb | [MYO9B](http://www.gene.ucl.ac.uk/cgi-bin/nomenclature/searchgenes.pl?field=symbol&anchor=equals&symbol_search=Search&number=100&format=html&sortby=symbol&match=MYO9B) | DNA binding;transcription factor activity | [602129](http://www.ncbi.nlm.nih.gov/entrez/dispomim.cgi?id=602129) | **-1.76** |
| 1418034_AT | mitochondrial ribosomal protein S9 | [MRPS9](http://www.gene.ucl.ac.uk/cgi-bin/nomenclature/searchgenes.pl?field=symbol&anchor=equals&symbol_search=Search&number=100&format=html&sortby=symbol&match=MRPS9) | DNA binding;transcription factor activity |  | **1.34** |
| 1418040_AT | RIKEN cDNA 4432406C05 gene | [4432406C05RIK](http://www.gene.ucl.ac.uk/cgi-bin/nomenclature/searchgenes.pl?field=symbol&anchor=equals&symbol_search=Search&number=100&format=html&sortby=symbol&match=4432406C05RIK) | DNA binding;transcription factor activity |  | **1.47** |
| 1418058_AT | EGF, latrophilin seven transmembrane domain containing 1 | [ELTD1](http://www.gene.ucl.ac.uk/cgi-bin/nomenclature/searchgenes.pl?field=symbol&anchor=equals&symbol_search=Search&number=100&format=html&sortby=symbol&match=ELTD1) | DNA binding;transcription factor activity |  | **2.02** |
| 1418059_AT | EGF, latrophilin seven transmembrane domain containing 1 | [ELTD1](http://www.gene.ucl.ac.uk/cgi-bin/nomenclature/searchgenes.pl?field=symbol&anchor=equals&symbol_search=Search&number=100&format=html&sortby=symbol&match=ELTD1) | DNA binding;transcription factor activity |  | **2.29** |
| 1418061_AT | latent transforming growth factor beta binding protein 2 | [LTBP2](http://www.gene.ucl.ac.uk/cgi-bin/nomenclature/searchgenes.pl?field=symbol&anchor=equals&symbol_search=Search&number=100&format=html&sortby=symbol&match=LTBP2) | DNA binding;transcription factor activity | [602091](http://www.ncbi.nlm.nih.gov/entrez/dispomim.cgi?id=602091) | **-1.69** |
| 1418062_AT | eukaryotic translation elongation factor 1 alpha 2 | [EEF1A2](http://www.gene.ucl.ac.uk/cgi-bin/nomenclature/searchgenes.pl?field=symbol&anchor=equals&symbol_search=Search&number=100&format=html&sortby=symbol&match=EEF1A2) | DNA binding;transcription factor activity | [602959](http://www.ncbi.nlm.nih.gov/entrez/dispomim.cgi?id=602959) | **1.24** |
| 1418068_AT | gb:BQ176221 /DB_XREF=gi:20351713 /DB_XREF=UI-M-DJ2 |  | DNA binding;transcription factor activity |  | **1.62** |
| 1418073_AT | acyl-Coenzyme A thioesterase 2, mitochondrial | [ACATE2](http://www.gene.ucl.ac.uk/cgi-bin/nomenclature/searchgenes.pl?field=symbol&anchor=equals&symbol_search=Search&number=100&format=html&sortby=symbol&match=ACATE2) | DNA binding;transcription factor activity |  | **-1.69** |
| 1418074_AT | sialyltransferase 7 ((alpha-N-acetylneuraminyl 2,3-betagalactosyl-1,3)-N-acetyl galactosaminide alpha-2,6-sialyltransferase) D | [SIAT7D](http://www.gene.ucl.ac.uk/cgi-bin/nomenclature/searchgenes.pl?field=symbol&anchor=equals&symbol_search=Search&number=100&format=html&sortby=symbol&match=SIAT7D) | DNA binding;transcription factor activity | [606378](http://www.ncbi.nlm.nih.gov/entrez/dispomim.cgi?id=606378) | **-1.61** |
| 1418075_AT | sialyltransferase 7 ((alpha-N-acetylneuraminyl 2,3-betagalactosyl-1,3)-N-acetyl galactosaminide alpha-2,6-sialyltransferase) D | [SIAT7D](http://www.gene.ucl.ac.uk/cgi-bin/nomenclature/searchgenes.pl?field=symbol&anchor=equals&symbol_search=Search&number=100&format=html&sortby=symbol&match=SIAT7D) | DNA binding;transcription factor activity | [606378](http://www.ncbi.nlm.nih.gov/entrez/dispomim.cgi?id=606378) | **-1.44** |
| 1418081_AT | Williams-Beuren syndrome chromosome region 18 homolog (human) | [WBSCR18](http://www.gene.ucl.ac.uk/cgi-bin/nomenclature/searchgenes.pl?field=symbol&anchor=equals&symbol_search=Search&number=100&format=html&sortby=symbol&match=WBSCR18) | DNA binding;transcription factor activity |  | **1.27** |
| 1418084_AT | neuropilin 1 | [NRP1](http://www.gene.ucl.ac.uk/cgi-bin/nomenclature/searchgenes.pl?field=symbol&anchor=equals&symbol_search=Search&number=100&format=html&sortby=symbol&match=NRP1) | DNA binding;transcription factor activity | [602069](http://www.ncbi.nlm.nih.gov/entrez/dispomim.cgi?id=602069) | **1.37** |
| 1418092_S_AT | thyroid hormone receptor interactor 10 | [TRIP10](http://www.gene.ucl.ac.uk/cgi-bin/nomenclature/searchgenes.pl?field=symbol&anchor=equals&symbol_search=Search&number=100&format=html&sortby=symbol&match=TRIP10) | DNA binding;transcription factor activity | [604504](http://www.ncbi.nlm.nih.gov/entrez/dispomim.cgi?id=604504) | **1.61** |
| 1418093_A_AT | epidermal growth factor | [EGF](http://www.gene.ucl.ac.uk/cgi-bin/nomenclature/searchgenes.pl?field=symbol&anchor=equals&symbol_search=Search&number=100&format=html&sortby=symbol&match=EGF) | DNA binding;transcription factor activity | [131530](http://www.ncbi.nlm.nih.gov/entrez/dispomim.cgi?id=131530) | **1.43** |
| 1418094_S_AT | carbonic anhydrase 4 | [CAR4](http://www.gene.ucl.ac.uk/cgi-bin/nomenclature/searchgenes.pl?field=symbol&anchor=equals&symbol_search=Search&number=100&format=html&sortby=symbol&match=CAR4) | DNA binding;transcription factor activity |  | **2.23** |
| 1418097_A_AT | thymic stromal-derived lymphopoietin, receptor | [TSLPR](http://www.gene.ucl.ac.uk/cgi-bin/nomenclature/searchgenes.pl?field=symbol&anchor=equals&symbol_search=Search&number=100&format=html&sortby=symbol&match=TSLPR) | DNA binding;transcription factor activity |  | **-1.71** |
| 1418098_AT | adenylate cyclase 4 | [ADCY4](http://www.gene.ucl.ac.uk/cgi-bin/nomenclature/searchgenes.pl?field=symbol&anchor=equals&symbol_search=Search&number=100&format=html&sortby=symbol&match=ADCY4) | DNA binding;transcription factor activity | [600292](http://www.ncbi.nlm.nih.gov/entrez/dispomim.cgi?id=600292) | **1.48** |
| 1418099_AT | tumor necrosis factor receptor superfamily, member 1b | [TNFRSF1B](http://www.gene.ucl.ac.uk/cgi-bin/nomenclature/searchgenes.pl?field=symbol&anchor=equals&symbol_search=Search&number=100&format=html&sortby=symbol&match=TNFRSF1B) | DNA binding;transcription factor activity | [191191](http://www.ncbi.nlm.nih.gov/entrez/dispomim.cgi?id=191191) | **-1.56** |
| 1418117_AT | NADH dehydrogenase (ubiquinone) Fe-S protein 4 | [NDUFS4](http://www.gene.ucl.ac.uk/cgi-bin/nomenclature/searchgenes.pl?field=symbol&anchor=equals&symbol_search=Search&number=100&format=html&sortby=symbol&match=NDUFS4) | DNA binding;transcription factor activity | [602694](http://www.ncbi.nlm.nih.gov/entrez/dispomim.cgi?id=602694) | **1.42** |
| 1418119_AT | RNA binding motif protein 8a | [RBM8A](http://www.gene.ucl.ac.uk/cgi-bin/nomenclature/searchgenes.pl?field=symbol&anchor=equals&symbol_search=Search&number=100&format=html&sortby=symbol&match=RBM8A) | DNA binding;transcription factor activity | [605313](http://www.ncbi.nlm.nih.gov/entrez/dispomim.cgi?id=605313) | **-1.23** |
| 1418120_AT | RNA binding motif protein 8a | [RBM8A](http://www.gene.ucl.ac.uk/cgi-bin/nomenclature/searchgenes.pl?field=symbol&anchor=equals&symbol_search=Search&number=100&format=html&sortby=symbol&match=RBM8A) | DNA binding;transcription factor activity | [605313](http://www.ncbi.nlm.nih.gov/entrez/dispomim.cgi?id=605313) | **-1.54** |
| 1418127_A_AT | programmed cell death 8 | [PDCD8](http://www.gene.ucl.ac.uk/cgi-bin/nomenclature/searchgenes.pl?field=symbol&anchor=equals&symbol_search=Search&number=100&format=html&sortby=symbol&match=PDCD8) | DNA binding;transcription factor activity | [300169](http://www.ncbi.nlm.nih.gov/entrez/dispomim.cgi?id=300169) | **1.21** |
| 1418133_AT | B-cell leukemia/lymphoma 3 | [BCL3](http://www.gene.ucl.ac.uk/cgi-bin/nomenclature/searchgenes.pl?field=symbol&anchor=equals&symbol_search=Search&number=100&format=html&sortby=symbol&match=BCL3) | DNA binding;transcription factor activity | [109560](http://www.ncbi.nlm.nih.gov/entrez/dispomim.cgi?id=109560) | **-2.14** |
| 1418142_AT | potassium inwardly-rectifying channel, subfamily J, member 8 | [KCNJ8](http://www.gene.ucl.ac.uk/cgi-bin/nomenclature/searchgenes.pl?field=symbol&anchor=equals&symbol_search=Search&number=100&format=html&sortby=symbol&match=KCNJ8) | DNA binding;transcription factor activity | [600935](http://www.ncbi.nlm.nih.gov/entrez/dispomim.cgi?id=600935) | **1.95** |
| 1418146_A_AT | retinoblastoma-like 2 | [RBL2](http://www.gene.ucl.ac.uk/cgi-bin/nomenclature/searchgenes.pl?field=symbol&anchor=equals&symbol_search=Search&number=100&format=html&sortby=symbol&match=RBL2) | DNA binding;transcription factor activity | [180203](http://www.ncbi.nlm.nih.gov/entrez/dispomim.cgi?id=180203) | **1.53** |
| 1418154_AT | cDNA sequence BC004022 | [BC004022](http://www.gene.ucl.ac.uk/cgi-bin/nomenclature/searchgenes.pl?field=symbol&anchor=equals&symbol_search=Search&number=100&format=html&sortby=symbol&match=BC004022) | DNA binding;transcription factor activity |  | **1.35** |
| 1418162_AT | toll-like receptor 4 | [TLR4](http://www.gene.ucl.ac.uk/cgi-bin/nomenclature/searchgenes.pl?field=symbol&anchor=equals&symbol_search=Search&number=100&format=html&sortby=symbol&match=TLR4) | DNA binding;transcription factor activity | [603030](http://www.ncbi.nlm.nih.gov/entrez/dispomim.cgi?id=603030) | **-1.89** |
| 1418169_AT | zinc finger, CCHC domain containing 14 | [ZCCHC14](http://www.gene.ucl.ac.uk/cgi-bin/nomenclature/searchgenes.pl?field=symbol&anchor=equals&symbol_search=Search&number=100&format=html&sortby=symbol&match=ZCCHC14) | DNA binding;transcription factor activity |  | **1.31** |
| 1418170_A_AT | zinc finger, CCHC domain containing 14 | [ZCCHC14](http://www.gene.ucl.ac.uk/cgi-bin/nomenclature/searchgenes.pl?field=symbol&anchor=equals&symbol_search=Search&number=100&format=html&sortby=symbol&match=ZCCHC14) | DNA binding;transcription factor activity |  | **1.5** |
| 1418171_AT | transcription elongation factor A (SII)-like 8 | [TCEAL8](http://www.gene.ucl.ac.uk/cgi-bin/nomenclature/searchgenes.pl?field=symbol&anchor=equals&symbol_search=Search&number=100&format=html&sortby=symbol&match=TCEAL8) | DNA binding;transcription factor activity |  | **-2.09** |
| 1418181_AT | protein tyrosine phosphatase 4a3 | [PTP4A3](http://www.gene.ucl.ac.uk/cgi-bin/nomenclature/searchgenes.pl?field=symbol&anchor=equals&symbol_search=Search&number=100&format=html&sortby=symbol&match=PTP4A3) | DNA binding;transcription factor activity | [606449](http://www.ncbi.nlm.nih.gov/entrez/dispomim.cgi?id=606449) | **1.88** |
| 1418183_A_AT | pleckstrin homology, Sec7 and coiled-coil domains 1 | [PSCD1](http://www.gene.ucl.ac.uk/cgi-bin/nomenclature/searchgenes.pl?field=symbol&anchor=equals&symbol_search=Search&number=100&format=html&sortby=symbol&match=PSCD1) | DNA binding;transcription factor activity | [182115](http://www.ncbi.nlm.nih.gov/entrez/dispomim.cgi?id=182115) | **1.36** |
| 1418185_AT | RIKEN cDNA 4733401H18 gene | [4733401H18RIK](http://www.gene.ucl.ac.uk/cgi-bin/nomenclature/searchgenes.pl?field=symbol&anchor=equals&symbol_search=Search&number=100&format=html&sortby=symbol&match=4733401H18RIK) | DNA binding;transcription factor activity |  | **1.28** |
| 1418187_AT | receptor (calcitonin) activity modifying protein 2 | [RAMP2](http://www.gene.ucl.ac.uk/cgi-bin/nomenclature/searchgenes.pl?field=symbol&anchor=equals&symbol_search=Search&number=100&format=html&sortby=symbol&match=RAMP2) | DNA binding;transcription factor activity | [605154](http://www.ncbi.nlm.nih.gov/entrez/dispomim.cgi?id=605154) | **1.47** |
| 1418196_AT | telomerase associated protein 1 | [TEP1](http://www.gene.ucl.ac.uk/cgi-bin/nomenclature/searchgenes.pl?field=symbol&anchor=equals&symbol_search=Search&number=100&format=html&sortby=symbol&match=TEP1) | DNA binding;transcription factor activity | [601686](http://www.ncbi.nlm.nih.gov/entrez/dispomim.cgi?id=601686) | **-1.81** |
| 1418198_A_AT | transmembrane 9 superfamily member 1 | [TM9SF1](http://www.gene.ucl.ac.uk/cgi-bin/nomenclature/searchgenes.pl?field=symbol&anchor=equals&symbol_search=Search&number=100&format=html&sortby=symbol&match=TM9SF1) | DNA binding;transcription factor activity |  | **-1.24** |
| 1418204_S_AT | allograft inflammatory factor 1 | [AIF1](http://www.gene.ucl.ac.uk/cgi-bin/nomenclature/searchgenes.pl?field=symbol&anchor=equals&symbol_search=Search&number=100&format=html&sortby=symbol&match=AIF1) | DNA binding;transcription factor activity | [601833](http://www.ncbi.nlm.nih.gov/entrez/dispomim.cgi?id=601833) | **-5.15** |
| 1418219_AT | interleukin 15 | [IL15](http://www.gene.ucl.ac.uk/cgi-bin/nomenclature/searchgenes.pl?field=symbol&anchor=equals&symbol_search=Search&number=100&format=html&sortby=symbol&match=IL15) | DNA binding;transcription factor activity | [600554](http://www.ncbi.nlm.nih.gov/entrez/dispomim.cgi?id=600554) | **2.95** |
| 1418223_AT | signal peptidase complex | [SEC11L1](http://www.gene.ucl.ac.uk/cgi-bin/nomenclature/searchgenes.pl?field=symbol&anchor=equals&symbol_search=Search&number=100&format=html&sortby=symbol&match=SEC11L1) | DNA binding;transcription factor activity |  | **-1.37** |
| 1418238_AT | isovaleryl coenzyme A dehydrogenase | [IVD](http://www.gene.ucl.ac.uk/cgi-bin/nomenclature/searchgenes.pl?field=symbol&anchor=equals&symbol_search=Search&number=100&format=html&sortby=symbol&match=IVD) | DNA binding;transcription factor activity | [607036](http://www.ncbi.nlm.nih.gov/entrez/dispomim.cgi?id=607036) | **1.83** |
| 1418241_AT | upstream transcription factor 2 | [USF2](http://www.gene.ucl.ac.uk/cgi-bin/nomenclature/searchgenes.pl?field=symbol&anchor=equals&symbol_search=Search&number=100&format=html&sortby=symbol&match=USF2) | DNA binding;transcription factor activity | [600390](http://www.ncbi.nlm.nih.gov/entrez/dispomim.cgi?id=600390) | **1.26** |
| 1418252_AT | peptidyl arginine deiminase, type II | [PADI2](http://www.gene.ucl.ac.uk/cgi-bin/nomenclature/searchgenes.pl?field=symbol&anchor=equals&symbol_search=Search&number=100&format=html&sortby=symbol&match=PADI2) | DNA binding;transcription factor activity | [607935](http://www.ncbi.nlm.nih.gov/entrez/dispomim.cgi?id=607935) | **1.76** |
| 1418256_AT | serum response factor | [SRF](http://www.gene.ucl.ac.uk/cgi-bin/nomenclature/searchgenes.pl?field=symbol&anchor=equals&symbol_search=Search&number=100&format=html&sortby=symbol&match=SRF) | DNA binding;transcription factor activity;receptor activity | [600589](http://www.ncbi.nlm.nih.gov/entrez/dispomim.cgi?id=600589) | **1.36** |
| 1418258_S_AT | RIKEN cDNA 6720463E02 gene | [6720463E02RIK](http://www.gene.ucl.ac.uk/cgi-bin/nomenclature/searchgenes.pl?field=symbol&anchor=equals&symbol_search=Search&number=100&format=html&sortby=symbol&match=6720463E02RIK) | DNA binding;transcription factor activity;ribulose-bisphosphate carboxylase activity |  | **1.34** |
| 1418259_A_AT | ectonucleoside triphosphate diphosphohydrolase 2 | [ENTPD2](http://www.gene.ucl.ac.uk/cgi-bin/nomenclature/searchgenes.pl?field=symbol&anchor=equals&symbol_search=Search&number=100&format=html&sortby=symbol&match=ENTPD2) | DNA binding;transcription factor activity;RNA polymerase II transcription factor activity | [602012](http://www.ncbi.nlm.nih.gov/entrez/dispomim.cgi?id=602012) | **-1.49** |
| 1418265_S_AT | interferon regulatory factor 2 | [IRF2](http://www.gene.ucl.ac.uk/cgi-bin/nomenclature/searchgenes.pl?field=symbol&anchor=equals&symbol_search=Search&number=100&format=html&sortby=symbol&match=IRF2) | DNA binding;transcription factor activity;RNA polymerase II transcription factor activity, enhancer binding | [147576](http://www.ncbi.nlm.nih.gov/entrez/dispomim.cgi?id=147576) | **1.29** |
| 1418277_AT | retinitis pigmentosa 9 homolog (human) | [RP9H](http://www.gene.ucl.ac.uk/cgi-bin/nomenclature/searchgenes.pl?field=symbol&anchor=equals&symbol_search=Search&number=100&format=html&sortby=symbol&match=RP9H) | DNA binding;transcription factor activity;signal transducer activity |  | **1.21** |
| 1418279_A_AT | A kinase (PRKA) anchor protein 1 | [AKAP1](http://www.gene.ucl.ac.uk/cgi-bin/nomenclature/searchgenes.pl?field=symbol&anchor=equals&symbol_search=Search&number=100&format=html&sortby=symbol&match=AKAP1) | DNA binding;transcription factor activity;signal transducer activity | [602449](http://www.ncbi.nlm.nih.gov/entrez/dispomim.cgi?id=602449) | **1.6** |
| 1418286_A_AT | ephrin B1 | [EFNB1](http://www.gene.ucl.ac.uk/cgi-bin/nomenclature/searchgenes.pl?field=symbol&anchor=equals&symbol_search=Search&number=100&format=html&sortby=symbol&match=EFNB1) | DNA binding;transcription factor activity;signal transducer activity | [300035](http://www.ncbi.nlm.nih.gov/entrez/dispomim.cgi?id=300035) | **1.44** |
| 1418288_AT | gb:NM_015763.1 /DB_XREF=gi:7656874 /GEN=Lpin1 /FEA |  | DNA binding;transcription factor activity;signal transducer activity;histone acetyltransferase binding |  | **1.99** |
| 1418289_AT | nestin | [NES](http://www.gene.ucl.ac.uk/cgi-bin/nomenclature/searchgenes.pl?field=symbol&anchor=equals&symbol_search=Search&number=100&format=html&sortby=symbol&match=NES) | DNA binding;transcription factor activity;transcription cofactor activity | [600915](http://www.ncbi.nlm.nih.gov/entrez/dispomim.cgi?id=600915) | **-1.73** |
| 1418293_AT | interferon-induced protein with tetratricopeptide repeats 2 | [IFIT2](http://www.gene.ucl.ac.uk/cgi-bin/nomenclature/searchgenes.pl?field=symbol&anchor=equals&symbol_search=Search&number=100&format=html&sortby=symbol&match=IFIT2) | DNA binding;transcription factor activity;transcriptional activator activity | [147040](http://www.ncbi.nlm.nih.gov/entrez/dispomim.cgi?id=147040) | **-1.6** |
| 1418296_AT | FXYD domain-containing ion transport regulator 5 | [FXYD5](http://www.gene.ucl.ac.uk/cgi-bin/nomenclature/searchgenes.pl?field=symbol&anchor=equals&symbol_search=Search&number=100&format=html&sortby=symbol&match=FXYD5) | DNA binding;transcription factor activity;transcriptional activator activity | [606669](http://www.ncbi.nlm.nih.gov/entrez/dispomim.cgi?id=606669) | **-1.77** |
| 1418321_AT | dodecenoyl-Coenzyme A delta isomerase (3,2 trans-enoyl-Coenyme A isomerase) | [DCI](http://www.gene.ucl.ac.uk/cgi-bin/nomenclature/searchgenes.pl?field=symbol&anchor=equals&symbol_search=Search&number=100&format=html&sortby=symbol&match=DCI) | DNA binding;transcription factor activity;transcriptional repressor activity | [600305](http://www.ncbi.nlm.nih.gov/entrez/dispomim.cgi?id=600305) | **1.87** |
| 1418323_AT | feminization 1 homolog b (C. elegans) | [FEM1B](http://www.gene.ucl.ac.uk/cgi-bin/nomenclature/searchgenes.pl?field=symbol&anchor=equals&symbol_search=Search&number=100&format=html&sortby=symbol&match=FEM1B) | DNA binding;transcription factor activity;transcriptional repressor activity |  | **-1.95** |
| 1418324_AT | feminization 1 homolog b (C. elegans) | [FEM1B](http://www.gene.ucl.ac.uk/cgi-bin/nomenclature/searchgenes.pl?field=symbol&anchor=equals&symbol_search=Search&number=100&format=html&sortby=symbol&match=FEM1B) | DNA binding;transcriptional activator activity;transcription factor activity |  | **-1.36** |
| 1418327_AT | RIKEN cDNA 1110058L19 gene | [1110058L19RIK](http://www.gene.ucl.ac.uk/cgi-bin/nomenclature/searchgenes.pl?field=symbol&anchor=equals&symbol_search=Search&number=100&format=html&sortby=symbol&match=1110058L19RIK) | DNA topoisomerase activity;DNA binding;isomerase activity;DNA topoisomerase (ATP-hydrolyzing) activity;ATP binding |  | **1.31** |
| 1418328_AT | carnitine palmitoyltransferase 1b, muscle | [CPT1B](http://www.gene.ucl.ac.uk/cgi-bin/nomenclature/searchgenes.pl?field=symbol&anchor=equals&symbol_search=Search&number=100&format=html&sortby=symbol&match=CPT1B) | DNA topoisomerase activity;DNA binding;isomerase activity;DNA topoisomerase (ATP-hydrolyzing) activity;ATP binding | [601987](http://www.ncbi.nlm.nih.gov/entrez/dispomim.cgi?id=601987) | **1.72** |
| 1418340_AT | Fc receptor, IgE, high affinity I, gamma polypeptide | [FCER1G](http://www.gene.ucl.ac.uk/cgi-bin/nomenclature/searchgenes.pl?field=symbol&anchor=equals&symbol_search=Search&number=100&format=html&sortby=symbol&match=FCER1G) | DNA topoisomerase activity;DNA binding;isomerase activity;DNA topoisomerase type I activity | [147139](http://www.ncbi.nlm.nih.gov/entrez/dispomim.cgi?id=147139) | **-4** |
| 1418341_AT | RAB4A, member RAS oncogene family | [RAB4A](http://www.gene.ucl.ac.uk/cgi-bin/nomenclature/searchgenes.pl?field=symbol&anchor=equals&symbol_search=Search&number=100&format=html&sortby=symbol&match=RAB4A) | DNA-directed RNA polymerase activity | [179511](http://www.ncbi.nlm.nih.gov/entrez/dispomim.cgi?id=179511) | **1.28** |
| 1418347_AT | DNA segment, Chr X, Immunex 40, expressed | [DXIMX40E](http://www.gene.ucl.ac.uk/cgi-bin/nomenclature/searchgenes.pl?field=symbol&anchor=equals&symbol_search=Search&number=100&format=html&sortby=symbol&match=DXIMX40E) | dolichyl-diphosphooligosaccharide-protein glycotransferase activity;transferase activity |  | **-1.51** |
| 1418350_AT | diphtheria toxin receptor | [HBEGF](http://www.gene.ucl.ac.uk/cgi-bin/nomenclature/searchgenes.pl?field=symbol&anchor=equals&symbol_search=Search&number=100&format=html&sortby=symbol&match=HBEGF) | dolichyl-diphosphooligosaccharide-protein glycotransferase activity;transferase activity | [126150](http://www.ncbi.nlm.nih.gov/entrez/dispomim.cgi?id=126150) | **-1.4** |
| 1418356_AT | mercaptopyruvate sulfurtransferase | [MPST](http://www.gene.ucl.ac.uk/cgi-bin/nomenclature/searchgenes.pl?field=symbol&anchor=equals&symbol_search=Search&number=100&format=html&sortby=symbol&match=MPST) | dolichyl-diphosphooligosaccharide-protein glycotransferase activity;transferase activity | [602496](http://www.ncbi.nlm.nih.gov/entrez/dispomim.cgi?id=602496) | **1.65** |
| 1418364_A_AT | ferritin light chain 1 | [FTL1](http://www.gene.ucl.ac.uk/cgi-bin/nomenclature/searchgenes.pl?field=symbol&anchor=equals&symbol_search=Search&number=100&format=html&sortby=symbol&match=FTL1) | dolichyl-diphosphooligosaccharide-protein glycotransferase activity;transferase activity |  | **-2.03** |
| 1418365_AT | cathepsin H | [CTSH](http://www.gene.ucl.ac.uk/cgi-bin/nomenclature/searchgenes.pl?field=symbol&anchor=equals&symbol_search=Search&number=100&format=html&sortby=symbol&match=CTSH) | dolichyl-diphosphooligosaccharide-protein glycotransferase activity;transferase activity | [116820](http://www.ncbi.nlm.nih.gov/entrez/dispomim.cgi?id=116820) | **-1.91** |
| 1418372_AT | gb:NM_009634.1 /DB_XREF=gi:6752995 /GEN=Adsl /FEA= |  | dolichyl-diphosphooligosaccharide-protein glycotransferase activity;transferase activity |  | **1.4** |
| 1418379_S_AT | G protein-coupled receptor 124 | [GPR124](http://www.gene.ucl.ac.uk/cgi-bin/nomenclature/searchgenes.pl?field=symbol&anchor=equals&symbol_search=Search&number=100&format=html&sortby=symbol&match=GPR124) | dolichyl-phosphate beta-glucosyltransferase activity;transferase activity, transferring glycosyl groups;transferase activity | [606823](http://www.ncbi.nlm.nih.gov/entrez/dispomim.cgi?id=606823) | **-1.56** |
| 1418389_AT | RIKEN cDNA 2810453I06 gene | [2810453I06RIK](http://www.gene.ucl.ac.uk/cgi-bin/nomenclature/searchgenes.pl?field=symbol&anchor=equals&symbol_search=Search&number=100&format=html&sortby=symbol&match=2810453I06RIK) | double-stranded DNA binding |  | **1.34** |
| 1418393_A_AT | integrin alpha 7 | [ITGA7](http://www.gene.ucl.ac.uk/cgi-bin/nomenclature/searchgenes.pl?field=symbol&anchor=equals&symbol_search=Search&number=100&format=html&sortby=symbol&match=ITGA7) | electron acceptor activity;electron-transferring-flavoprotein dehydrogenase activity;oxidoreductase activity | [600536](http://www.ncbi.nlm.nih.gov/entrez/dispomim.cgi?id=600536) | **-1.47** |
| 1418397_AT | Zinc finger protein 275 | [ZFP275](http://www.gene.ucl.ac.uk/cgi-bin/nomenclature/searchgenes.pl?field=symbol&anchor=equals&symbol_search=Search&number=100&format=html&sortby=symbol&match=ZFP275) | electron carrier activity |  | **-1.48** |
| 1418399_AT | potassium channel tetramerisation domain containing 9 | [KCTD9](http://www.gene.ucl.ac.uk/cgi-bin/nomenclature/searchgenes.pl?field=symbol&anchor=equals&symbol_search=Search&number=100&format=html&sortby=symbol&match=KCTD9) | electron carrier activity;oxidoreductase activity |  | **1.41** |
| 1418402_AT | a disintegrin and metalloproteinase domain 19 (meltrin beta) | [ADAM19](http://www.gene.ucl.ac.uk/cgi-bin/nomenclature/searchgenes.pl?field=symbol&anchor=equals&symbol_search=Search&number=100&format=html&sortby=symbol&match=ADAM19) | electron transporter activity | [603640](http://www.ncbi.nlm.nih.gov/entrez/dispomim.cgi?id=603640) | **-2.38** |
| 1418403_AT | gb:NM_009616.1 /DB_XREF=gi:6752965 /GEN=Adam19 /FE |  | electron transporter activity |  | **-2.3** |
| 1418406_AT | phosphodiesterase 8A | [PDE8A](http://www.gene.ucl.ac.uk/cgi-bin/nomenclature/searchgenes.pl?field=symbol&anchor=equals&symbol_search=Search&number=100&format=html&sortby=symbol&match=PDE8A) | electron transporter activity | [602972](http://www.ncbi.nlm.nih.gov/entrez/dispomim.cgi?id=602972) | **1.6** |
| 1418413_AT | caveolin 3 | [CAV3](http://www.gene.ucl.ac.uk/cgi-bin/nomenclature/searchgenes.pl?field=symbol&anchor=equals&symbol_search=Search&number=100&format=html&sortby=symbol&match=CAV3) | electron transporter activity | [601253](http://www.ncbi.nlm.nih.gov/entrez/dispomim.cgi?id=601253) | **-2.02** |
| 1418417_AT | musculin | [MSC](http://www.gene.ucl.ac.uk/cgi-bin/nomenclature/searchgenes.pl?field=symbol&anchor=equals&symbol_search=Search&number=100&format=html&sortby=symbol&match=MSC) | electron transporter activity | [603628](http://www.ncbi.nlm.nih.gov/entrez/dispomim.cgi?id=603628) | **-2.1** |
| 1418420_AT | myogenic differentiation 1 | [MYOD1](http://www.gene.ucl.ac.uk/cgi-bin/nomenclature/searchgenes.pl?field=symbol&anchor=equals&symbol_search=Search&number=100&format=html&sortby=symbol&match=MYOD1) | electron transporter activity | [159970](http://www.ncbi.nlm.nih.gov/entrez/dispomim.cgi?id=159970) | **-2.91** |
| 1418431_AT | kinesin family member 5B | [KIF5B](http://www.gene.ucl.ac.uk/cgi-bin/nomenclature/searchgenes.pl?field=symbol&anchor=equals&symbol_search=Search&number=100&format=html&sortby=symbol&match=KIF5B) | electron transporter activity | [602809](http://www.ncbi.nlm.nih.gov/entrez/dispomim.cgi?id=602809) | **-1.5** |
| 1418433_AT | calcium binding protein 39 | [CAB39](http://www.gene.ucl.ac.uk/cgi-bin/nomenclature/searchgenes.pl?field=symbol&anchor=equals&symbol_search=Search&number=100&format=html&sortby=symbol&match=CAB39) | electron transporter activity;electron carrier activity |  | **1.21** |
| 1418439_AT | DNA segment, Chr 10, ERATO Doi 322, expressed | [D10ERTD322E](http://www.gene.ucl.ac.uk/cgi-bin/nomenclature/searchgenes.pl?field=symbol&anchor=equals&symbol_search=Search&number=100&format=html&sortby=symbol&match=D10ERTD322E) | electron transporter activity;electron carrier activity |  | **1.31** |
| 1418440_AT | procollagen, type VIII, alpha 1 | [COL8A1](http://www.gene.ucl.ac.uk/cgi-bin/nomenclature/searchgenes.pl?field=symbol&anchor=equals&symbol_search=Search&number=100&format=html&sortby=symbol&match=COL8A1) | electron transporter activity;oxidoreductase activity;copper ion binding;protein-lysine 6-oxidase activity;oxidoreductase activity, acting on the CH-NH2 group of donors, oxygen as acceptor | [120251](http://www.ncbi.nlm.nih.gov/entrez/dispomim.cgi?id=120251) | **-2.39** |
| 1418445_AT | gb:NM_009197.1 /DB_XREF=gi:6677996 /GEN=Slc16a2 /F |  | electron transporter activity;oxidoreductase activity;oxidoreductase activity, acting on NADH or NADPH |  | **-1.82** |
| 1418446_AT | solute carrier family 16 (monocarboxylic acid transporters), member 2 | [SLC16A2](http://www.gene.ucl.ac.uk/cgi-bin/nomenclature/searchgenes.pl?field=symbol&anchor=equals&symbol_search=Search&number=100&format=html&sortby=symbol&match=SLC16A2) | endonuclease activity;nuclease activity;nucleic acid binding;magnesium ion binding;hydrolase activity | [300095](http://www.ncbi.nlm.nih.gov/entrez/dispomim.cgi?id=300095) | **-1.78** |
| 1418448_AT | Harvey rat sarcoma oncogene, subgroup R | [RRAS](http://www.gene.ucl.ac.uk/cgi-bin/nomenclature/searchgenes.pl?field=symbol&anchor=equals&symbol_search=Search&number=100&format=html&sortby=symbol&match=RRAS) | endonuclease activity;nuclease activity;RNA binding;hydrolase activity | [165090](http://www.ncbi.nlm.nih.gov/entrez/dispomim.cgi?id=165090) | **-1.26** |
| 1418453_A_AT | ATPase, Na+/K+ transporting, beta 1 polypeptide | [ATP1B1](http://www.gene.ucl.ac.uk/cgi-bin/nomenclature/searchgenes.pl?field=symbol&anchor=equals&symbol_search=Search&number=100&format=html&sortby=symbol&match=ATP1B1) | endonuclease activity;nucleotide diphosphatase activity;nucleic acid binding;catalytic activity;hydrolase activity;phosphodiesterase I activity | [182330](http://www.ncbi.nlm.nih.gov/entrez/dispomim.cgi?id=182330) | **1.37** |
| 1418454_AT | microfibrillar associated protein 5 | [MFAP5](http://www.gene.ucl.ac.uk/cgi-bin/nomenclature/searchgenes.pl?field=symbol&anchor=equals&symbol_search=Search&number=100&format=html&sortby=symbol&match=MFAP5) | endopeptidase activity | [601103](http://www.ncbi.nlm.nih.gov/entrez/dispomim.cgi?id=601103) | **-2.7** |
| 1418459_AT | RIKEN cDNA 1810060J02 gene | [1810060J02RIK](http://www.gene.ucl.ac.uk/cgi-bin/nomenclature/searchgenes.pl?field=symbol&anchor=equals&symbol_search=Search&number=100&format=html&sortby=symbol&match=1810060J02RIK) | endopeptidase activity |  | **1.34** |
| 1418460_AT | SH3 domain protein D19 | [SH3D19](http://www.gene.ucl.ac.uk/cgi-bin/nomenclature/searchgenes.pl?field=symbol&anchor=equals&symbol_search=Search&number=100&format=html&sortby=symbol&match=SH3D19) | endopeptidase activity;hydrolase activity;peptidase activity |  | **-1.73** |
| 1418467_AT | SWI/SNF related, matrix associated, actin dependent regulator of chromatin, subfamily d, member 3 | [SMARCD3](http://www.gene.ucl.ac.uk/cgi-bin/nomenclature/searchgenes.pl?field=symbol&anchor=equals&symbol_search=Search&number=100&format=html&sortby=symbol&match=SMARCD3) | endopeptidase inhibitor activity;cysteine protease inhibitor activity | [601737](http://www.ncbi.nlm.nih.gov/entrez/dispomim.cgi?id=601737) | **1.26** |
| 1418469_AT | gb:AV272221 /DB_XREF=gi:16390145 /DB_XREF=AV272221 |  | endopeptidase inhibitor activity;cysteine protease inhibitor activity |  | **-1.45** |
| 1418474_AT | RIKEN cDNA 1500005A01 gene | [1500005A01RIK](http://www.gene.ucl.ac.uk/cgi-bin/nomenclature/searchgenes.pl?field=symbol&anchor=equals&symbol_search=Search&number=100&format=html&sortby=symbol&match=1500005A01RIK) | enoyl-CoA hydratase activity;isomerase activity;lyase activity;long-chain-3-hydroxyacyl-CoA dehydrogenase activity;oxidoreductase activity;catalytic activity |  | **1.21** |
| 1418485_AT | solute carrier family 4 (anion exchanger), member 3 | [SLC4A3](http://www.gene.ucl.ac.uk/cgi-bin/nomenclature/searchgenes.pl?field=symbol&anchor=equals&symbol_search=Search&number=100&format=html&sortby=symbol&match=SLC4A3) | enoyl-CoA hydratase activity;lyase activity;long-chain-enoyl-CoA hydratase activity;catalytic activity | [106195](http://www.ncbi.nlm.nih.gov/entrez/dispomim.cgi?id=106195) | **-1.72** |
| 1418501_A_AT | oxidation resistance 1 | [OXR1](http://www.gene.ucl.ac.uk/cgi-bin/nomenclature/searchgenes.pl?field=symbol&anchor=equals&symbol_search=Search&number=100&format=html&sortby=symbol&match=OXR1) | enoyl-CoA hydratase activity;RNA binding;lyase activity;catalytic activity;methylglutaconyl-CoA hydratase activity | [605609](http://www.ncbi.nlm.nih.gov/entrez/dispomim.cgi?id=605609) | **1.23** |
| 1418503_AT | heat shock protein, A | [HSPA9A](http://www.gene.ucl.ac.uk/cgi-bin/nomenclature/searchgenes.pl?field=symbol&anchor=equals&symbol_search=Search&number=100&format=html&sortby=symbol&match=HSPA9A) | enzyme activator activity |  | **1.3** |
| 1418504_AT | heat shock protein, A | [HSPA9A](http://www.gene.ucl.ac.uk/cgi-bin/nomenclature/searchgenes.pl?field=symbol&anchor=equals&symbol_search=Search&number=100&format=html&sortby=symbol&match=HSPA9A) | enzyme activator activity;metalloendopeptidase inhibitor activity |  | **1.33** |
| 1418510_S_AT | F-box only protein 8 | [FBXO8](http://www.gene.ucl.ac.uk/cgi-bin/nomenclature/searchgenes.pl?field=symbol&anchor=equals&symbol_search=Search&number=100&format=html&sortby=symbol&match=FBXO8) | enzyme activator activity;metalloendopeptidase inhibitor activity | [605649](http://www.ncbi.nlm.nih.gov/entrez/dispomim.cgi?id=605649) | **1.21** |
| 1418511_AT | dermatopontin | [DPT](http://www.gene.ucl.ac.uk/cgi-bin/nomenclature/searchgenes.pl?field=symbol&anchor=equals&symbol_search=Search&number=100&format=html&sortby=symbol&match=DPT) | enzyme inhibitor activity | [125597](http://www.ncbi.nlm.nih.gov/entrez/dispomim.cgi?id=125597) | **-1.96** |
| 1418538_AT | KDEL (Lys-Asp-Glu-Leu) endoplasmic reticulum protein retention receptor 3 | [KDELR3](http://www.gene.ucl.ac.uk/cgi-bin/nomenclature/searchgenes.pl?field=symbol&anchor=equals&symbol_search=Search&number=100&format=html&sortby=symbol&match=KDELR3) | ephrin receptor binding;protein binding |  | **-1.96** |
| 1418539_A_AT | protein tyrosine phosphatase, receptor type, E | [PTPRE](http://www.gene.ucl.ac.uk/cgi-bin/nomenclature/searchgenes.pl?field=symbol&anchor=equals&symbol_search=Search&number=100&format=html&sortby=symbol&match=PTPRE) | ephrin receptor binding;protein binding | [600926](http://www.ncbi.nlm.nih.gov/entrez/dispomim.cgi?id=600926) | **-1.82** |
| 1418540_A_AT | protein tyrosine phosphatase, receptor type, E | [PTPRE](http://www.gene.ucl.ac.uk/cgi-bin/nomenclature/searchgenes.pl?field=symbol&anchor=equals&symbol_search=Search&number=100&format=html&sortby=symbol&match=PTPRE) | epoxide hydrolase activity;aminopeptidase activity;catalytic activity;hydrolase activity | [600926](http://www.ncbi.nlm.nih.gov/entrez/dispomim.cgi?id=600926) | **-2** |
| 1418547_AT | tissue factor pathway inhibitor 2 | [TFPI2](http://www.gene.ucl.ac.uk/cgi-bin/nomenclature/searchgenes.pl?field=symbol&anchor=equals&symbol_search=Search&number=100&format=html&sortby=symbol&match=TFPI2) | epoxide hydrolase activity;catalytic activity;aminopeptidase activity;hydrolase activity | [600033](http://www.ncbi.nlm.nih.gov/entrez/dispomim.cgi?id=600033) | **-2.13** |
| 1418560_AT | pyruvate dehydrogenase E1 alpha 1 | [PDHA1](http://www.gene.ucl.ac.uk/cgi-bin/nomenclature/searchgenes.pl?field=symbol&anchor=equals&symbol_search=Search&number=100&format=html&sortby=symbol&match=PDHA1) | epoxide hydrolase activity;catalytic activity;hydrolase activity | [300502](http://www.ncbi.nlm.nih.gov/entrez/dispomim.cgi?id=300502) | **1.27** |
| 1418563_AT | RIKEN cDNA 1200009K13 gene | [1200009K13RIK](http://www.gene.ucl.ac.uk/cgi-bin/nomenclature/searchgenes.pl?field=symbol&anchor=equals&symbol_search=Search&number=100&format=html&sortby=symbol&match=1200009K13RIK) | extracellular matrix constituent conferring elasticity |  | **1.3** |
| 1418566_S_AT | DNA segment, Chr 11, ERATO Doi 603, expressed | [D11ERTD603E](http://www.gene.ucl.ac.uk/cgi-bin/nomenclature/searchgenes.pl?field=symbol&anchor=equals&symbol_search=Search&number=100&format=html&sortby=symbol&match=D11ERTD603E) | extracellular matrix constituent conferring elasticity |  | **-1.55** |
| 1418567_A_AT | signal recognition particle 14 | [SRP14](http://www.gene.ucl.ac.uk/cgi-bin/nomenclature/searchgenes.pl?field=symbol&anchor=equals&symbol_search=Search&number=100&format=html&sortby=symbol&match=SRP14) | extracellular matrix structural constituent;extracellular matrix structural constituent conferring tensile strength | [600708](http://www.ncbi.nlm.nih.gov/entrez/dispomim.cgi?id=600708) | **-1.25** |
| 1418568_X_AT | signal recognition particle 14 | [SRP14](http://www.gene.ucl.ac.uk/cgi-bin/nomenclature/searchgenes.pl?field=symbol&anchor=equals&symbol_search=Search&number=100&format=html&sortby=symbol&match=SRP14) | ferric iron binding | [600708](http://www.ncbi.nlm.nih.gov/entrez/dispomim.cgi?id=600708) | **-1.24** |
| 1418571_AT | tumor necrosis factor receptor superfamily, member 12a | [TNFRSF12A](http://www.gene.ucl.ac.uk/cgi-bin/nomenclature/searchgenes.pl?field=symbol&anchor=equals&symbol_search=Search&number=100&format=html&sortby=symbol&match=TNFRSF12A) | ferric iron binding;binding | [605914](http://www.ncbi.nlm.nih.gov/entrez/dispomim.cgi?id=605914) | **-2.95** |
| 1418576_AT | RIKEN cDNA 2610311I19 gene | [2610311I19RIK](http://www.gene.ucl.ac.uk/cgi-bin/nomenclature/searchgenes.pl?field=symbol&anchor=equals&symbol_search=Search&number=100&format=html&sortby=symbol&match=2610311I19RIK) | ferric iron binding;binding |  | **-1.58** |
| 1418577_AT | tripartite motif protein 8 | [TRIM8](http://www.gene.ucl.ac.uk/cgi-bin/nomenclature/searchgenes.pl?field=symbol&anchor=equals&symbol_search=Search&number=100&format=html&sortby=symbol&match=TRIM8) | fibroblast growth factor binding | [606125](http://www.ncbi.nlm.nih.gov/entrez/dispomim.cgi?id=606125) | **-1.27** |
| 1418583_AT | histidine triad nucleotide binding protein 3 | [HINT3](http://www.gene.ucl.ac.uk/cgi-bin/nomenclature/searchgenes.pl?field=symbol&anchor=equals&symbol_search=Search&number=100&format=html&sortby=symbol&match=HINT3) | fibroblast growth factor binding |  | **1.43** |
| 1418587_AT | Tnf receptor-associated factor 3 | [TRAF3](http://www.gene.ucl.ac.uk/cgi-bin/nomenclature/searchgenes.pl?field=symbol&anchor=equals&symbol_search=Search&number=100&format=html&sortby=symbol&match=TRAF3) | GABA receptor binding | [601896](http://www.ncbi.nlm.nih.gov/entrez/dispomim.cgi?id=601896) | **-1.5** |
| 1418591_AT | DnaJ (Hsp40) homolog, subfamily A, member 4 | [DNAJA4](http://www.gene.ucl.ac.uk/cgi-bin/nomenclature/searchgenes.pl?field=symbol&anchor=equals&symbol_search=Search&number=100&format=html&sortby=symbol&match=DNAJA4) | GABA receptor binding |  | **1.46** |
| 1418592_AT | DnaJ (Hsp40) homolog, subfamily A, member 4 | [DNAJA4](http://www.gene.ucl.ac.uk/cgi-bin/nomenclature/searchgenes.pl?field=symbol&anchor=equals&symbol_search=Search&number=100&format=html&sortby=symbol&match=DNAJA4) | gamma-glutamyl hydrolase activity;catalytic activity;hydrolase activity |  | **1.58** |
| 1418593_AT | TAF6 RNA polymerase II, TATA box binding protein (TBP)-associated factor | [TAF6](http://www.gene.ucl.ac.uk/cgi-bin/nomenclature/searchgenes.pl?field=symbol&anchor=equals&symbol_search=Search&number=100&format=html&sortby=symbol&match=TAF6) | gap-junction forming channel activity;connexon channel activity | [602955](http://www.ncbi.nlm.nih.gov/entrez/dispomim.cgi?id=602955) | **1.28** |
| 1418595_AT | plasma membrane associated protein, S3-12 | [S3-12](http://www.gene.ucl.ac.uk/cgi-bin/nomenclature/searchgenes.pl?field=symbol&anchor=equals&symbol_search=Search&number=100&format=html&sortby=symbol&match=S3-12) | gap-junction forming channel activity;connexon channel activity |  | **1.47** |
| 1418596_AT | gb:NM_008011.1 /DB_XREF=gi:6679788 /GEN=Fgfr4 /FEA |  | gap-junction forming channel activity;connexon channel activity |  | **-1.72** |
| 1418602_AT | cadherin 15 | [CDH15](http://www.gene.ucl.ac.uk/cgi-bin/nomenclature/searchgenes.pl?field=symbol&anchor=equals&symbol_search=Search&number=100&format=html&sortby=symbol&match=CDH15) | glucosamine-6-phosphate deaminase activity;hydrolase activity | [114019](http://www.ncbi.nlm.nih.gov/entrez/dispomim.cgi?id=114019) | **-1.81** |
| 1418638_AT | gb:NM_010387.1 /DB_XREF=gi:6754121 /GEN=H2-DMb1 /F |  | glucosylceramidase activity;hydrolase activity, acting on glycosyl bonds;hydrolase activity |  | **-1.68** |
| 1418648_AT | EGL nine homolog 3 (C. elegans) | [EGLN3](http://www.gene.ucl.ac.uk/cgi-bin/nomenclature/searchgenes.pl?field=symbol&anchor=equals&symbol_search=Search&number=100&format=html&sortby=symbol&match=EGLN3) | glucuronosyl-N-acetylgalactosaminyl-proteoglycan 4-beta-N-acetylgalactosaminyltransferase activity | [606426](http://www.ncbi.nlm.nih.gov/entrez/dispomim.cgi?id=606426) | **1.93** |
| 1418649_AT | EGL nine homolog 3 (C. elegans) | [EGLN3](http://www.gene.ucl.ac.uk/cgi-bin/nomenclature/searchgenes.pl?field=symbol&anchor=equals&symbol_search=Search&number=100&format=html&sortby=symbol&match=EGLN3) | glutamate carboxypeptidase activity;dipeptidase activity;metallopeptidase activity;carboxypeptidase activity;dipeptidase E activity;catalytic activity;hydrolase activity;peptidase activity | [606426](http://www.ncbi.nlm.nih.gov/entrez/dispomim.cgi?id=606426) | **1.75** |
| 1418658_AT | RIKEN cDNA 2410005O16 gene | [2410005O16RIK](http://www.gene.ucl.ac.uk/cgi-bin/nomenclature/searchgenes.pl?field=symbol&anchor=equals&symbol_search=Search&number=100&format=html&sortby=symbol&match=2410005O16RIK) | glutamate dehydrogenase [NAD(P)+] activity;GTP binding;oxidoreductase activity;ATP binding |  | **1.62** |
| 1418663_AT | multiple PDZ domain protein | [MPDZ](http://www.gene.ucl.ac.uk/cgi-bin/nomenclature/searchgenes.pl?field=symbol&anchor=equals&symbol_search=Search&number=100&format=html&sortby=symbol&match=MPDZ) | glutamate-5-semialdehyde dehydrogenase activity;kinase activity;oxidoreductase activity;catalytic activity;glutamate 5-kinase activity;transferase activity | [603785](http://www.ncbi.nlm.nih.gov/entrez/dispomim.cgi?id=603785) | **1.39** |
| 1418664_AT | Multiple PDZ domain protein | [MPDZ](http://www.gene.ucl.ac.uk/cgi-bin/nomenclature/searchgenes.pl?field=symbol&anchor=equals&symbol_search=Search&number=100&format=html&sortby=symbol&match=MPDZ) | glutamate-5-semialdehyde dehydrogenase activity;kinase activity;oxidoreductase activity;catalytic activity;glutamate 5-kinase activity;transferase activity | [603785](http://www.ncbi.nlm.nih.gov/entrez/dispomim.cgi?id=603785) | **1.23** |
| 1418666_AT | pentaxin related gene | [PTX3](http://www.gene.ucl.ac.uk/cgi-bin/nomenclature/searchgenes.pl?field=symbol&anchor=equals&symbol_search=Search&number=100&format=html&sortby=symbol&match=PTX3) | glutamate-5-semialdehyde dehydrogenase activity;kinase activity;oxidoreductase activity;catalytic activity;glutamate 5-kinase activity;transferase activity | [602492](http://www.ncbi.nlm.nih.gov/entrez/dispomim.cgi?id=602492) | **-2.47** |
| 1418670_S_AT | gb:NM_008305.1 /DB_XREF=gi:6680310 /GEN=Hspg2 /FEA |  | glutamate-tRNA ligase activity;actin binding;ligase activity;catalytic activity;ATP binding;tRNA ligase activity |  | **-1.3** |
| 1418674_AT | oncostatin M receptor | [OSMR](http://www.gene.ucl.ac.uk/cgi-bin/nomenclature/searchgenes.pl?field=symbol&anchor=equals&symbol_search=Search&number=100&format=html&sortby=symbol&match=OSMR) | glutaryl-CoA dehydrogenase activity;transcription factor activity;oxidoreductase activity;acyl-CoA dehydrogenase activity | [601743](http://www.ncbi.nlm.nih.gov/entrez/dispomim.cgi?id=601743) | **-1.42** |
| 1418677_AT | actinin alpha 3 | [ACTN3](http://www.gene.ucl.ac.uk/cgi-bin/nomenclature/searchgenes.pl?field=symbol&anchor=equals&symbol_search=Search&number=100&format=html&sortby=symbol&match=ACTN3) | glutathione peroxidase activity;oxidoreductase activity;peroxidase activity | [102574](http://www.ncbi.nlm.nih.gov/entrez/dispomim.cgi?id=102574) | **-1.58** |
| 1418694_AT | potassium channel modulatory factor 1 | [KCMF1](http://www.gene.ucl.ac.uk/cgi-bin/nomenclature/searchgenes.pl?field=symbol&anchor=equals&symbol_search=Search&number=100&format=html&sortby=symbol&match=KCMF1) | glutathione transferase activity;transferase activity |  | **1.56** |
| 1418699_S_AT | ferrochelatase | [FECH](http://www.gene.ucl.ac.uk/cgi-bin/nomenclature/searchgenes.pl?field=symbol&anchor=equals&symbol_search=Search&number=100&format=html&sortby=symbol&match=FECH) | glutathione transferase activity;transferase activity | [177000](http://www.ncbi.nlm.nih.gov/entrez/dispomim.cgi?id=177000) | **1.23** |
| 1418704_AT | S100 calcium binding protein A13 | [S100A13](http://www.gene.ucl.ac.uk/cgi-bin/nomenclature/searchgenes.pl?field=symbol&anchor=equals&symbol_search=Search&number=100&format=html&sortby=symbol&match=S100A13) | glutathione transferase activity;transferase activity | [601989](http://www.ncbi.nlm.nih.gov/entrez/dispomim.cgi?id=601989) | **-1.38** |
| 1418709_AT | cytochrome c oxidase, subunit VIIa 1 | [COX7A1](http://www.gene.ucl.ac.uk/cgi-bin/nomenclature/searchgenes.pl?field=symbol&anchor=equals&symbol_search=Search&number=100&format=html&sortby=symbol&match=COX7A1) | glutathione transferase activity;transferase activity | [123995](http://www.ncbi.nlm.nih.gov/entrez/dispomim.cgi?id=123995) | **1.46** |
| 1418710_AT | CD59a antigen | [CD59A](http://www.gene.ucl.ac.uk/cgi-bin/nomenclature/searchgenes.pl?field=symbol&anchor=equals&symbol_search=Search&number=100&format=html&sortby=symbol&match=CD59A) | glutathione transferase activity;transferase activity |  | **1.61** |
| 1418714_AT | dual specificity phosphatase 8 | [DUSP8](http://www.gene.ucl.ac.uk/cgi-bin/nomenclature/searchgenes.pl?field=symbol&anchor=equals&symbol_search=Search&number=100&format=html&sortby=symbol&match=DUSP8) | glutathione transferase activity;transferase activity | [602038](http://www.ncbi.nlm.nih.gov/entrez/dispomim.cgi?id=602038) | **1.62** |
| 1418716_AT | mitochondrial ribosomal protein S25 | [MRPS25](http://www.gene.ucl.ac.uk/cgi-bin/nomenclature/searchgenes.pl?field=symbol&anchor=equals&symbol_search=Search&number=100&format=html&sortby=symbol&match=MRPS25) | glutathione transferase activity;transferase activity |  | **1.47** |
| 1418718_AT | chemokine (C-X-C motif) ligand 16 | [CXCL16](http://www.gene.ucl.ac.uk/cgi-bin/nomenclature/searchgenes.pl?field=symbol&anchor=equals&symbol_search=Search&number=100&format=html&sortby=symbol&match=CXCL16) | glycerol-3-phosphate O-acyltransferase activity;acyltransferase activity;transferase activity | [605398](http://www.ncbi.nlm.nih.gov/entrez/dispomim.cgi?id=605398) | **-2.13** |
| 1418726_A_AT | troponin T2, cardiac | [TNNT2](http://www.gene.ucl.ac.uk/cgi-bin/nomenclature/searchgenes.pl?field=symbol&anchor=equals&symbol_search=Search&number=100&format=html&sortby=symbol&match=TNNT2) | glycerophosphodiester phosphodiesterase activity;catalytic activity;hydrolase activity | [191045](http://www.ncbi.nlm.nih.gov/entrez/dispomim.cgi?id=191045) | **-29.98** |
| 1418747_AT | SFFV proviral integration 1 | [SFPI1](http://www.gene.ucl.ac.uk/cgi-bin/nomenclature/searchgenes.pl?field=symbol&anchor=equals&symbol_search=Search&number=100&format=html&sortby=symbol&match=SFPI1) | glycerophosphodiester phosphodiesterase activity;catalytic activity;hydrolase activity |  | **-1.78** |
| 1418768_AT | optic atrophy 1 homolog (human) | [OPA1](http://www.gene.ucl.ac.uk/cgi-bin/nomenclature/searchgenes.pl?field=symbol&anchor=equals&symbol_search=Search&number=100&format=html&sortby=symbol&match=OPA1) | glycogen phosphorylase activity;phosphorylase activity;transferase activity, transferring glycosyl groups;catalytic activity;transferase activity | [605290](http://www.ncbi.nlm.nih.gov/entrez/dispomim.cgi?id=605290) | **1.75** |
| 1418778_AT | RIKEN cDNA 9030408N13 gene | [9030408N13RIK](http://www.gene.ucl.ac.uk/cgi-bin/nomenclature/searchgenes.pl?field=symbol&anchor=equals&symbol_search=Search&number=100&format=html&sortby=symbol&match=9030408N13RIK) | GMP reductase activity;oxidoreductase activity;catalytic activity |  | **-1.79** |
| 1418782_AT | retinoid X receptor gamma | [RXRG](http://www.gene.ucl.ac.uk/cgi-bin/nomenclature/searchgenes.pl?field=symbol&anchor=equals&symbol_search=Search&number=100&format=html&sortby=symbol&match=RXRG) | G-protein coupled receptor activity, unknown ligand;receptor activity;G-protein coupled receptor activity | [180247](http://www.ncbi.nlm.nih.gov/entrez/dispomim.cgi?id=180247) | **1.62** |
| 1418788_AT | endothelial-specific receptor tyrosine kinase | [TEK](http://www.gene.ucl.ac.uk/cgi-bin/nomenclature/searchgenes.pl?field=symbol&anchor=equals&symbol_search=Search&number=100&format=html&sortby=symbol&match=TEK) | G-protein coupled receptor activity, unknown ligand;receptor activity;G-protein coupled receptor activity | [600221](http://www.ncbi.nlm.nih.gov/entrez/dispomim.cgi?id=600221) | **2.02** |
| 1418796_AT | stem cell growth factor | [SCGF](http://www.gene.ucl.ac.uk/cgi-bin/nomenclature/searchgenes.pl?field=symbol&anchor=equals&symbol_search=Search&number=100&format=html&sortby=symbol&match=SCGF) | G-protein coupled receptor activity, unknown ligand;receptor activity;G-protein coupled receptor activity;catalytic activity | [604713](http://www.ncbi.nlm.nih.gov/entrez/dispomim.cgi?id=604713) | **-1.85** |
| 1418798_S_AT | serine/threonine kinase 23 | [STK23](http://www.gene.ucl.ac.uk/cgi-bin/nomenclature/searchgenes.pl?field=symbol&anchor=equals&symbol_search=Search&number=100&format=html&sortby=symbol&match=STK23) | G-protein coupled receptor activity;latrotoxin receptor activity |  | **-1.62** |
| 1418814_S_AT | RIKEN cDNA 2410011G03 gene | [2410011G03RIK](http://www.gene.ucl.ac.uk/cgi-bin/nomenclature/searchgenes.pl?field=symbol&anchor=equals&symbol_search=Search&number=100&format=html&sortby=symbol&match=2410011G03RIK) | G-protein coupled receptor activity;latrotoxin receptor activity |  | **1.32** |
| 1418815_AT | cadherin 2 | [CDH2](http://www.gene.ucl.ac.uk/cgi-bin/nomenclature/searchgenes.pl?field=symbol&anchor=equals&symbol_search=Search&number=100&format=html&sortby=symbol&match=CDH2) | G-protein coupled receptor activity;lysosphingolipid and lysophosphatidic acid receptor activity;rhodopsin-like receptor activity | [114020](http://www.ncbi.nlm.nih.gov/entrez/dispomim.cgi?id=114020) | **-3.15** |
| 1418822_A_AT | ADP-ribosylation factor 6 | [ARF6](http://www.gene.ucl.ac.uk/cgi-bin/nomenclature/searchgenes.pl?field=symbol&anchor=equals&symbol_search=Search&number=100&format=html&sortby=symbol&match=ARF6) | G-protein coupled receptor activity;rhodopsin-like receptor activity;C-C chemokine receptor activity | [600464](http://www.ncbi.nlm.nih.gov/entrez/dispomim.cgi?id=600464) | **-1.78** |
| 1418823_AT | ADP-ribosylation factor 6 | [ARF6](http://www.gene.ucl.ac.uk/cgi-bin/nomenclature/searchgenes.pl?field=symbol&anchor=equals&symbol_search=Search&number=100&format=html&sortby=symbol&match=ARF6) | growth factor activity;chemokine activity;cytokine activity | [600464](http://www.ncbi.nlm.nih.gov/entrez/dispomim.cgi?id=600464) | **-1.61** |
| 1418826_AT | membrane-spanning 4-domains, subfamily A, member 6B | [MS4A6B](http://www.gene.ucl.ac.uk/cgi-bin/nomenclature/searchgenes.pl?field=symbol&anchor=equals&symbol_search=Search&number=100&format=html&sortby=symbol&match=MS4A6B) | growth factor activity;fibroblast growth factor receptor binding |  | **-3.54** |
| 1418835_AT | pleckstrin homology-like domain, family A, member 1 | [PHLDA1](http://www.gene.ucl.ac.uk/cgi-bin/nomenclature/searchgenes.pl?field=symbol&anchor=equals&symbol_search=Search&number=100&format=html&sortby=symbol&match=PHLDA1) | growth factor activity;fibroblast growth factor receptor binding;heparin binding | [605335](http://www.ncbi.nlm.nih.gov/entrez/dispomim.cgi?id=605335) | **-1.5** |
| 1418843_AT | solute carrier family 30 (zinc transporter), member 4 | [SLC30A4](http://www.gene.ucl.ac.uk/cgi-bin/nomenclature/searchgenes.pl?field=symbol&anchor=equals&symbol_search=Search&number=100&format=html&sortby=symbol&match=SLC30A4) | growth factor activity;fibroblast growth factor receptor binding;heparin binding | [602095](http://www.ncbi.nlm.nih.gov/entrez/dispomim.cgi?id=602095) | **-1.51** |
| 1418849_X_AT | aquaporin 7 | [AQP7](http://www.gene.ucl.ac.uk/cgi-bin/nomenclature/searchgenes.pl?field=symbol&anchor=equals&symbol_search=Search&number=100&format=html&sortby=symbol&match=AQP7) | growth factor activity;heparin binding | [602974](http://www.ncbi.nlm.nih.gov/entrez/dispomim.cgi?id=602974) | **2.22** |
| 1418852_AT | cholinergic receptor, nicotinic, alpha polypeptide 1 (muscle) | [CHRNA1](http://www.gene.ucl.ac.uk/cgi-bin/nomenclature/searchgenes.pl?field=symbol&anchor=equals&symbol_search=Search&number=100&format=html&sortby=symbol&match=CHRNA1) | growth factor activity;heparin binding | [100690](http://www.ncbi.nlm.nih.gov/entrez/dispomim.cgi?id=100690) | **-3.36** |
| 1418862_AT | enoyl Coenzyme A hydratase domain containing 3 | [ECHDC3](http://www.gene.ucl.ac.uk/cgi-bin/nomenclature/searchgenes.pl?field=symbol&anchor=equals&symbol_search=Search&number=100&format=html&sortby=symbol&match=ECHDC3) | growth factor activity;heparin binding |  | **1.45** |
| 1418883_A_AT | poly A binding protein, cytoplasmic 1 | [PABPC1](http://www.gene.ucl.ac.uk/cgi-bin/nomenclature/searchgenes.pl?field=symbol&anchor=equals&symbol_search=Search&number=100&format=html&sortby=symbol&match=PABPC1) | growth factor activity;heparin binding | [604679](http://www.ncbi.nlm.nih.gov/entrez/dispomim.cgi?id=604679) | **-1.59** |
| 1418884_X_AT | tubulin, alpha 1 | [TUBA1](http://www.gene.ucl.ac.uk/cgi-bin/nomenclature/searchgenes.pl?field=symbol&anchor=equals&symbol_search=Search&number=100&format=html&sortby=symbol&match=TUBA1) | growth factor activity;heparin binding | [191110](http://www.ncbi.nlm.nih.gov/entrez/dispomim.cgi?id=191110) | **-2.91** |
| 1418885_A_AT | isocitrate dehydrogenase 3 (NAD+) beta | [IDH3B](http://www.gene.ucl.ac.uk/cgi-bin/nomenclature/searchgenes.pl?field=symbol&anchor=equals&symbol_search=Search&number=100&format=html&sortby=symbol&match=IDH3B) | growth factor activity;transforming growth factor beta receptor binding | [604526](http://www.ncbi.nlm.nih.gov/entrez/dispomim.cgi?id=604526) | **-1.36** |
| 1418886_S_AT | isocitrate dehydrogenase 3 (NAD+) beta | [IDH3B](http://www.gene.ucl.ac.uk/cgi-bin/nomenclature/searchgenes.pl?field=symbol&anchor=equals&symbol_search=Search&number=100&format=html&sortby=symbol&match=IDH3B) | growth factor binding;calcium ion binding | [604526](http://www.ncbi.nlm.nih.gov/entrez/dispomim.cgi?id=604526) | **1.32** |
| 1418888_A_AT | selenoprotein X 1 | [SEPX1](http://www.gene.ucl.ac.uk/cgi-bin/nomenclature/searchgenes.pl?field=symbol&anchor=equals&symbol_search=Search&number=100&format=html&sortby=symbol&match=SEPX1) | growth factor binding;calcium ion binding | [606216](http://www.ncbi.nlm.nih.gov/entrez/dispomim.cgi?id=606216) | **-1.24** |
| 1418893_AT | pre B-cell leukemia transcription factor 2 | [PBX2](http://www.gene.ucl.ac.uk/cgi-bin/nomenclature/searchgenes.pl?field=symbol&anchor=equals&symbol_search=Search&number=100&format=html&sortby=symbol&match=PBX2) | growth factor binding;calcium ion binding;chitin binding | [176311](http://www.ncbi.nlm.nih.gov/entrez/dispomim.cgi?id=176311) | **-1.33** |
| 1418895_AT | src family associated phosphoprotein 2 | [SCAP2](http://www.gene.ucl.ac.uk/cgi-bin/nomenclature/searchgenes.pl?field=symbol&anchor=equals&symbol_search=Search&number=100&format=html&sortby=symbol&match=SCAP2) | growth factor binding;calcium ion binding;oxidoreductase activity | [605215](http://www.ncbi.nlm.nih.gov/entrez/dispomim.cgi?id=605215) | **-1.48** |
| 1418896_A_AT | ribophorin II | [RPN2](http://www.gene.ucl.ac.uk/cgi-bin/nomenclature/searchgenes.pl?field=symbol&anchor=equals&symbol_search=Search&number=100&format=html&sortby=symbol&match=RPN2) | growth factor binding;insulin-like growth factor binding | [180490](http://www.ncbi.nlm.nih.gov/entrez/dispomim.cgi?id=180490) | **-1.49** |
| 1418911_S_AT | acyl-CoA synthetase long-chain family member 4 | [ACSL4](http://www.gene.ucl.ac.uk/cgi-bin/nomenclature/searchgenes.pl?field=symbol&anchor=equals&symbol_search=Search&number=100&format=html&sortby=symbol&match=ACSL4) | growth factor binding;protein binding;calcium ion binding;integrin binding;glycosaminoglycan binding | [300157](http://www.ncbi.nlm.nih.gov/entrez/dispomim.cgi?id=300157) | **-1.41** |
| 1418915_AT | RIKEN cDNA 1810037K07 gene | [1810037K07RIK](http://www.gene.ucl.ac.uk/cgi-bin/nomenclature/searchgenes.pl?field=symbol&anchor=equals&symbol_search=Search&number=100&format=html&sortby=symbol&match=1810037K07RIK) | growth factor binding;protein binding;insulin-like growth factor binding;heparin binding |  | **1.43** |
| 1418920_AT | claudin 15 | [CLDN15](http://www.gene.ucl.ac.uk/cgi-bin/nomenclature/searchgenes.pl?field=symbol&anchor=equals&symbol_search=Search&number=100&format=html&sortby=symbol&match=CLDN15) | GTP binding |  | **1.77** |
| 1418930_AT | chemokine (C-X-C motif) ligand 10 | [CXCL10](http://www.gene.ucl.ac.uk/cgi-bin/nomenclature/searchgenes.pl?field=symbol&anchor=equals&symbol_search=Search&number=100&format=html&sortby=symbol&match=CXCL10) | GTP binding | [147310](http://www.ncbi.nlm.nih.gov/entrez/dispomim.cgi?id=147310) | **-3.31** |
| 1418937_AT | deiodinase, iodothyronine, type II | [DIO2](http://www.gene.ucl.ac.uk/cgi-bin/nomenclature/searchgenes.pl?field=symbol&anchor=equals&symbol_search=Search&number=100&format=html&sortby=symbol&match=DIO2) | GTP binding | [601413](http://www.ncbi.nlm.nih.gov/entrez/dispomim.cgi?id=601413) | **-5.08** |
| 1418942_AT | coiled-coil domain containing 2 | [CCDC2](http://www.gene.ucl.ac.uk/cgi-bin/nomenclature/searchgenes.pl?field=symbol&anchor=equals&symbol_search=Search&number=100&format=html&sortby=symbol&match=CCDC2) | GTP binding | [608040](http://www.ncbi.nlm.nih.gov/entrez/dispomim.cgi?id=608040) | **-1.43** |
| 1418945_AT | matrix metalloproteinase 3 | [MMP3](http://www.gene.ucl.ac.uk/cgi-bin/nomenclature/searchgenes.pl?field=symbol&anchor=equals&symbol_search=Search&number=100&format=html&sortby=symbol&match=MMP3) | GTP binding | [185250](http://www.ncbi.nlm.nih.gov/entrez/dispomim.cgi?id=185250) | **-5** |
| 1418951_AT | RIKEN cDNA 2310001N14 gene | [2310001N14RIK](http://www.gene.ucl.ac.uk/cgi-bin/nomenclature/searchgenes.pl?field=symbol&anchor=equals&symbol_search=Search&number=100&format=html&sortby=symbol&match=2310001N14RIK) | GTP binding |  | **1.71** |
| 1418952_AT | RIKEN cDNA 2310001N14 gene | [2310001N14RIK](http://www.gene.ucl.ac.uk/cgi-bin/nomenclature/searchgenes.pl?field=symbol&anchor=equals&symbol_search=Search&number=100&format=html&sortby=symbol&match=2310001N14RIK) | GTP binding |  | **1.8** |
| 1418968_AT | RB1-inducible coiled-coil 1 | [RB1CC1](http://www.gene.ucl.ac.uk/cgi-bin/nomenclature/searchgenes.pl?field=symbol&anchor=equals&symbol_search=Search&number=100&format=html&sortby=symbol&match=RB1CC1) | GTP binding | [606837](http://www.ncbi.nlm.nih.gov/entrez/dispomim.cgi?id=606837) | **1.3** |
| 1418988_AT | peroxisome biogenesis factor 7 | [PEX7](http://www.gene.ucl.ac.uk/cgi-bin/nomenclature/searchgenes.pl?field=symbol&anchor=equals&symbol_search=Search&number=100&format=html&sortby=symbol&match=PEX7) | GTP binding | [601757](http://www.ncbi.nlm.nih.gov/entrez/dispomim.cgi?id=601757) | **1.37** |
| 1418991_AT | BCL2-antagonist/killer 1 | [BAK1](http://www.gene.ucl.ac.uk/cgi-bin/nomenclature/searchgenes.pl?field=symbol&anchor=equals&symbol_search=Search&number=100&format=html&sortby=symbol&match=BAK1) | GTP binding | [600516](http://www.ncbi.nlm.nih.gov/entrez/dispomim.cgi?id=600516) | **-2.26** |
| 1418996_A_AT | RIKEN cDNA 4930469P12 gene | [4930469P12RIK](http://www.gene.ucl.ac.uk/cgi-bin/nomenclature/searchgenes.pl?field=symbol&anchor=equals&symbol_search=Search&number=100&format=html&sortby=symbol&match=4930469P12RIK) | GTP binding |  | **1.43** |
| 1419004_S_AT | B-cell leukemia/lymphoma 2 related protein A1d | [BCL2A1A](http://www.gene.ucl.ac.uk/cgi-bin/nomenclature/searchgenes.pl?field=symbol&anchor=equals&symbol_search=Search&number=100&format=html&sortby=symbol&match=BCL2A1A) | GTP binding |  | **-12.24** |
| 1419022_A_AT | enolase 1, alpha non-neuron | [ENO1](http://www.gene.ucl.ac.uk/cgi-bin/nomenclature/searchgenes.pl?field=symbol&anchor=equals&symbol_search=Search&number=100&format=html&sortby=symbol&match=ENO1) | GTP binding | [172430](http://www.ncbi.nlm.nih.gov/entrez/dispomim.cgi?id=172430) | **-1.34** |
| 1419023_X_AT | enolase 1, alpha non-neuron | [ENO1](http://www.gene.ucl.ac.uk/cgi-bin/nomenclature/searchgenes.pl?field=symbol&anchor=equals&symbol_search=Search&number=100&format=html&sortby=symbol&match=ENO1) | GTP binding | [172430](http://www.ncbi.nlm.nih.gov/entrez/dispomim.cgi?id=172430) | **-1.66** |
| 1419027_S_AT | glycolipid transfer protein | [GLTP](http://www.gene.ucl.ac.uk/cgi-bin/nomenclature/searchgenes.pl?field=symbol&anchor=equals&symbol_search=Search&number=100&format=html&sortby=symbol&match=GLTP) | GTP binding | [608949](http://www.ncbi.nlm.nih.gov/entrez/dispomim.cgi?id=608949) | **-1.4** |
| 1419028_AT | cyclic AMP-regulated phosphoprotein, 21 | [ARPP21](http://www.gene.ucl.ac.uk/cgi-bin/nomenclature/searchgenes.pl?field=symbol&anchor=equals&symbol_search=Search&number=100&format=html&sortby=symbol&match=ARPP21) | GTP binding |  | **-2.84** |
| 1419032_AT | RIKEN cDNA 2610018G03 gene | [2610018G03RIK](http://www.gene.ucl.ac.uk/cgi-bin/nomenclature/searchgenes.pl?field=symbol&anchor=equals&symbol_search=Search&number=100&format=html&sortby=symbol&match=2610018G03RIK) | GTP binding |  | **1.6** |
| 1419033_AT | RIKEN cDNA 2610018G03 gene | [2610018G03RIK](http://www.gene.ucl.ac.uk/cgi-bin/nomenclature/searchgenes.pl?field=symbol&anchor=equals&symbol_search=Search&number=100&format=html&sortby=symbol&match=2610018G03RIK) | GTP binding;protein transporter activity |  | **1.82** |
| 1419039_AT | cytochrome P450, family 2, subfamily d, polypeptide 22 | [CYP2D22](http://www.gene.ucl.ac.uk/cgi-bin/nomenclature/searchgenes.pl?field=symbol&anchor=equals&symbol_search=Search&number=100&format=html&sortby=symbol&match=CYP2D22) | GTP binding;protein transporter activity |  | **1.57** |
| 1419040_AT | cytochrome P450, family 2, subfamily d, polypeptide 22 | [CYP2D22](http://www.gene.ucl.ac.uk/cgi-bin/nomenclature/searchgenes.pl?field=symbol&anchor=equals&symbol_search=Search&number=100&format=html&sortby=symbol&match=CYP2D22) | GTPase activator activity |  | **1.44** |
| 1419045_AT | RIKEN cDNA 2310067G05 gene | [2310067G05RIK](http://www.gene.ucl.ac.uk/cgi-bin/nomenclature/searchgenes.pl?field=symbol&anchor=equals&symbol_search=Search&number=100&format=html&sortby=symbol&match=2310067G05RIK) | GTPase activator activity |  | **1.33** |
| 1419047_AT | pecanex homolog (Drosophila) | [PCNX](http://www.gene.ucl.ac.uk/cgi-bin/nomenclature/searchgenes.pl?field=symbol&anchor=equals&symbol_search=Search&number=100&format=html&sortby=symbol&match=PCNX) | GTPase activator activity |  | **1.38** |
| 1419050_AT | RIKEN cDNA 1110002H13 gene | [1110002H13RIK](http://www.gene.ucl.ac.uk/cgi-bin/nomenclature/searchgenes.pl?field=symbol&anchor=equals&symbol_search=Search&number=100&format=html&sortby=symbol&match=1110002H13RIK) | GTPase activator activity |  | **-42.74** |
| 1419056_AT | reticulon 2 (Z-band associated protein) | [RTN2](http://www.gene.ucl.ac.uk/cgi-bin/nomenclature/searchgenes.pl?field=symbol&anchor=equals&symbol_search=Search&number=100&format=html&sortby=symbol&match=RTN2) | GTPase activator activity | [603183](http://www.ncbi.nlm.nih.gov/entrez/dispomim.cgi?id=603183) | **1.26** |
| 1419072_AT | RIKEN cDNA 0610005A07 gene | [GSTM7](http://www.gene.ucl.ac.uk/cgi-bin/nomenclature/searchgenes.pl?field=symbol&anchor=equals&symbol_search=Search&number=100&format=html&sortby=symbol&match=GSTM7) | GTPase activator activity |  | **2.29** |
| 1419074_AT | RIKEN cDNA 2510006C20 gene | [2510006C20RIK](http://www.gene.ucl.ac.uk/cgi-bin/nomenclature/searchgenes.pl?field=symbol&anchor=equals&symbol_search=Search&number=100&format=html&sortby=symbol&match=2510006C20RIK) | GTPase activator activity |  | **1.58** |
| 1419081_AT | gb:NM_025770.1 /DB_XREF=gi:13385237 /GEN=5430428K1 |  | GTPase activator activity |  | **1.55** |
| 1419088_AT | tissue inhibitor of metalloproteinase 3 | [TIMP3](http://www.gene.ucl.ac.uk/cgi-bin/nomenclature/searchgenes.pl?field=symbol&anchor=equals&symbol_search=Search&number=100&format=html&sortby=symbol&match=TIMP3) | GTPase activator activity | [188826](http://www.ncbi.nlm.nih.gov/entrez/dispomim.cgi?id=188826) | **2.16** |
| 1419089_AT | tissue inhibitor of metalloproteinase 3 | [TIMP3](http://www.gene.ucl.ac.uk/cgi-bin/nomenclature/searchgenes.pl?field=symbol&anchor=equals&symbol_search=Search&number=100&format=html&sortby=symbol&match=TIMP3) | GTPase activator activity;diacylglycerol binding | [188826](http://www.ncbi.nlm.nih.gov/entrez/dispomim.cgi?id=188826) | **1.97** |
| 1419091_A_AT | annexin A2 | [ANXA2](http://www.gene.ucl.ac.uk/cgi-bin/nomenclature/searchgenes.pl?field=symbol&anchor=equals&symbol_search=Search&number=100&format=html&sortby=symbol&match=ANXA2) | GTPase activator activity;diacylglycerol binding | [151740](http://www.ncbi.nlm.nih.gov/entrez/dispomim.cgi?id=151740) | **-1.83** |
| 1419097_A_AT | erythrocyte protein band 7.2 | [STOM](http://www.gene.ucl.ac.uk/cgi-bin/nomenclature/searchgenes.pl?field=symbol&anchor=equals&symbol_search=Search&number=100&format=html&sortby=symbol&match=STOM) | GTPase activator activity;RAB GDP-dissociation inhibitor activity;Rab GTPase activator activity | [133090](http://www.ncbi.nlm.nih.gov/entrez/dispomim.cgi?id=133090) | **1.34** |
| 1419098_AT | erythrocyte protein band 7.2 | [STOM](http://www.gene.ucl.ac.uk/cgi-bin/nomenclature/searchgenes.pl?field=symbol&anchor=equals&symbol_search=Search&number=100&format=html&sortby=symbol&match=STOM) | GTPase activator activity;RAB GDP-dissociation inhibitor activity;Rab GTPase activator activity | [133090](http://www.ncbi.nlm.nih.gov/entrez/dispomim.cgi?id=133090) | **1.71** |
| 1419099_X_AT | erythrocyte protein band 7.2 | [STOM](http://www.gene.ucl.ac.uk/cgi-bin/nomenclature/searchgenes.pl?field=symbol&anchor=equals&symbol_search=Search&number=100&format=html&sortby=symbol&match=STOM) | GTPase activator activity;signal transducer activity | [133090](http://www.ncbi.nlm.nih.gov/entrez/dispomim.cgi?id=133090) | **1.6** |
| 1419100_AT | serine (or cysteine) proteinase inhibitor, clade A, member 3N | [SERPINA3N](http://www.gene.ucl.ac.uk/cgi-bin/nomenclature/searchgenes.pl?field=symbol&anchor=equals&symbol_search=Search&number=100&format=html&sortby=symbol&match=SERPINA3N) | GTPase activity |  | **-24.27** |
| 1419103_A_AT | abhydrolase domain containing 6 | [ABHD6](http://www.gene.ucl.ac.uk/cgi-bin/nomenclature/searchgenes.pl?field=symbol&anchor=equals&symbol_search=Search&number=100&format=html&sortby=symbol&match=ABHD6) | GTPase activity;GTP binding |  | **1.95** |
| 1419109_AT | histidine rich calcium binding protein | [HRC](http://www.gene.ucl.ac.uk/cgi-bin/nomenclature/searchgenes.pl?field=symbol&anchor=equals&symbol_search=Search&number=100&format=html&sortby=symbol&match=HRC) | GTPase activity;GTP binding | [142705](http://www.ncbi.nlm.nih.gov/entrez/dispomim.cgi?id=142705) | **1.25** |
| 1419128_AT | integrin alpha X | [ITGAX](http://www.gene.ucl.ac.uk/cgi-bin/nomenclature/searchgenes.pl?field=symbol&anchor=equals&symbol_search=Search&number=100&format=html&sortby=symbol&match=ITGAX) | GTPase activity;GTP binding | [151510](http://www.ncbi.nlm.nih.gov/entrez/dispomim.cgi?id=151510) | **-3.53** |
| 1419132_AT | toll-like receptor 2 | [TLR2](http://www.gene.ucl.ac.uk/cgi-bin/nomenclature/searchgenes.pl?field=symbol&anchor=equals&symbol_search=Search&number=100&format=html&sortby=symbol&match=TLR2) | GTPase activity;GTP binding | [603028](http://www.ncbi.nlm.nih.gov/entrez/dispomim.cgi?id=603028) | **-1.69** |
| 1419137_AT | SH3/ankyrin domain gene 3 | [SHANK3](http://www.gene.ucl.ac.uk/cgi-bin/nomenclature/searchgenes.pl?field=symbol&anchor=equals&symbol_search=Search&number=100&format=html&sortby=symbol&match=SHANK3) | GTPase activity;GTP binding | [606230](http://www.ncbi.nlm.nih.gov/entrez/dispomim.cgi?id=606230) | **1.9** |
| 1419144_AT | CD163 antigen | [CD163](http://www.gene.ucl.ac.uk/cgi-bin/nomenclature/searchgenes.pl?field=symbol&anchor=equals&symbol_search=Search&number=100&format=html&sortby=symbol&match=CD163) | GTPase activity;GTP binding | [605545](http://www.ncbi.nlm.nih.gov/entrez/dispomim.cgi?id=605545) | **3.17** |
| 1419145_AT | RIKEN cDNA 1110030K22 gene | [1110030K22RIK](http://www.gene.ucl.ac.uk/cgi-bin/nomenclature/searchgenes.pl?field=symbol&anchor=equals&symbol_search=Search&number=100&format=html&sortby=symbol&match=1110030K22RIK) | GTPase activity;GTP binding |  | **1.49** |
| 1419153_AT | RIKEN cDNA 2810417H13 gene | [2810417H13RIK](http://www.gene.ucl.ac.uk/cgi-bin/nomenclature/searchgenes.pl?field=symbol&anchor=equals&symbol_search=Search&number=100&format=html&sortby=symbol&match=2810417H13RIK) | GTPase activity;GTP binding |  | **-2.1** |
| 1419155_A_AT | SRY-box containing gene 4 | [SOX4](http://www.gene.ucl.ac.uk/cgi-bin/nomenclature/searchgenes.pl?field=symbol&anchor=equals&symbol_search=Search&number=100&format=html&sortby=symbol&match=SOX4) | GTPase activity;GTP binding;calmodulin binding | [184430](http://www.ncbi.nlm.nih.gov/entrez/dispomim.cgi?id=184430) | **-2.18** |
| 1419165_AT | zinc finger protein 260 | [ZFP260](http://www.gene.ucl.ac.uk/cgi-bin/nomenclature/searchgenes.pl?field=symbol&anchor=equals&symbol_search=Search&number=100&format=html&sortby=symbol&match=ZFP260) | GTPase activity;GTP binding;protein transporter activity | [601505](http://www.ncbi.nlm.nih.gov/entrez/dispomim.cgi?id=601505) | **-1.39** |
| 1419169_AT | mitogen-activated protein kinase 6 | [MAPK6](http://www.gene.ucl.ac.uk/cgi-bin/nomenclature/searchgenes.pl?field=symbol&anchor=equals&symbol_search=Search&number=100&format=html&sortby=symbol&match=MAPK6) | GTPase activity;GTP binding;signal transducer activity | [602904](http://www.ncbi.nlm.nih.gov/entrez/dispomim.cgi?id=602904) | **-1.58** |
| 1419191_AT | homeodomain interacting protein kinase 3 | [HIPK3](http://www.gene.ucl.ac.uk/cgi-bin/nomenclature/searchgenes.pl?field=symbol&anchor=equals&symbol_search=Search&number=100&format=html&sortby=symbol&match=HIPK3) | GTPase activity;GTP binding;signal transducer activity | [604424](http://www.ncbi.nlm.nih.gov/entrez/dispomim.cgi?id=604424) | **1.49** |
| 1419204_AT | delta-like 1 (Drosophila) | [DLL1](http://www.gene.ucl.ac.uk/cgi-bin/nomenclature/searchgenes.pl?field=symbol&anchor=equals&symbol_search=Search&number=100&format=html&sortby=symbol&match=DLL1) | GTPase activity;GTP binding;signal transducer activity | [606582](http://www.ncbi.nlm.nih.gov/entrez/dispomim.cgi?id=606582) | **-1.36** |
| 1419209_AT | chemokine (C-X-C motif) ligand 1 | [CXCL1](http://www.gene.ucl.ac.uk/cgi-bin/nomenclature/searchgenes.pl?field=symbol&anchor=equals&symbol_search=Search&number=100&format=html&sortby=symbol&match=CXCL1) | GTPase activity;GTP binding;signal transducer activity | [155730](http://www.ncbi.nlm.nih.gov/entrez/dispomim.cgi?id=155730) | **2.14** |
| 1419249_AT | PFTAIRE protein kinase 1 | [PFTK1](http://www.gene.ucl.ac.uk/cgi-bin/nomenclature/searchgenes.pl?field=symbol&anchor=equals&symbol_search=Search&number=100&format=html&sortby=symbol&match=PFTK1) | GTPase activity;GTP binding;signal transducer activity |  | **-1.91** |
| 1419253_AT | methylenetetrahydrofolate dehydrogenase (NAD+ dependent), methenyltetrahydrofolate cyclohydrolase | [MTHFD2](http://www.gene.ucl.ac.uk/cgi-bin/nomenclature/searchgenes.pl?field=symbol&anchor=equals&symbol_search=Search&number=100&format=html&sortby=symbol&match=MTHFD2) | GTPase activity;GTP binding;signal transducer activity | [604887](http://www.ncbi.nlm.nih.gov/entrez/dispomim.cgi?id=604887) | **-2.47** |
| 1419254_AT | methylenetetrahydrofolate dehydrogenase (NAD+ dependent), methenyltetrahydrofolate cyclohydrolase | [MTHFD2](http://www.gene.ucl.ac.uk/cgi-bin/nomenclature/searchgenes.pl?field=symbol&anchor=equals&symbol_search=Search&number=100&format=html&sortby=symbol&match=MTHFD2) | GTPase activity;guanyl nucleotide binding;GTP binding;protein transporter activity;catalytic activity | [604887](http://www.ncbi.nlm.nih.gov/entrez/dispomim.cgi?id=604887) | **-2.81** |
| 1419259_AT | gb:NM_009105.1 /DB_XREF=gi:6677824 /GEN=Rsu1 /FEA= |  | GTPase activity;signal transducer activity |  | **-1.35** |
| 1419272_AT | myeloid differentiation primary response gene 88 | [MYD88](http://www.gene.ucl.ac.uk/cgi-bin/nomenclature/searchgenes.pl?field=symbol&anchor=equals&symbol_search=Search&number=100&format=html&sortby=symbol&match=MYD88) | GTPase inhibitor activity | [602170](http://www.ncbi.nlm.nih.gov/entrez/dispomim.cgi?id=602170) | **-1.78** |
| 1419274_AT | expressed sequence C80913 | [C80913](http://www.gene.ucl.ac.uk/cgi-bin/nomenclature/searchgenes.pl?field=symbol&anchor=equals&symbol_search=Search&number=100&format=html&sortby=symbol&match=C80913) | guanidinoacetate N-methyltransferase activity;methyltransferase activity;transferase activity |  | **1.31** |
| 1419279_AT | phosphatidylinositol-4-phosphate 5-kinase, type II, alpha | [PIP5K2A](http://www.gene.ucl.ac.uk/cgi-bin/nomenclature/searchgenes.pl?field=symbol&anchor=equals&symbol_search=Search&number=100&format=html&sortby=symbol&match=PIP5K2A) | guanyl nucleotide binding;GTPase activity;GTP binding;protein transporter activity | [603140](http://www.ncbi.nlm.nih.gov/entrez/dispomim.cgi?id=603140) | **-2.22** |
| 1419280_AT | phosphatidylinositol-4-phosphate 5-kinase, type II, alpha | [PIP5K2A](http://www.gene.ucl.ac.uk/cgi-bin/nomenclature/searchgenes.pl?field=symbol&anchor=equals&symbol_search=Search&number=100&format=html&sortby=symbol&match=PIP5K2A) | guanyl nucleotide binding;GTPase activity;GTP binding;protein transporter activity | [603140](http://www.ncbi.nlm.nih.gov/entrez/dispomim.cgi?id=603140) | **-2.6** |
| 1419282_AT | chemokine (C-C motif) ligand 12 | [CCL12](http://www.gene.ucl.ac.uk/cgi-bin/nomenclature/searchgenes.pl?field=symbol&anchor=equals&symbol_search=Search&number=100&format=html&sortby=symbol&match=CCL12) | guanylate cyclase activity;receptor activity;lyase activity |  | **-24.75** |
| 1419283_S_AT | tensin | [TNS](http://www.gene.ucl.ac.uk/cgi-bin/nomenclature/searchgenes.pl?field=symbol&anchor=equals&symbol_search=Search&number=100&format=html&sortby=symbol&match=TNS) | guanyl-nucleotide exchange factor activity | [600076](http://www.ncbi.nlm.nih.gov/entrez/dispomim.cgi?id=600076) | **1.36** |
| 1419287_AT | HSPC171 protein | [HSPC171](http://www.gene.ucl.ac.uk/cgi-bin/nomenclature/searchgenes.pl?field=symbol&anchor=equals&symbol_search=Search&number=100&format=html&sortby=symbol&match=HSPC171) | guanyl-nucleotide exchange factor activity |  | **-1.4** |
| 1419288_AT | junction adhesion molecule 2 | [JAM2](http://www.gene.ucl.ac.uk/cgi-bin/nomenclature/searchgenes.pl?field=symbol&anchor=equals&symbol_search=Search&number=100&format=html&sortby=symbol&match=JAM2) | guanyl-nucleotide exchange factor activity | [606870](http://www.ncbi.nlm.nih.gov/entrez/dispomim.cgi?id=606870) | **1.71** |
| 1419289_A_AT | synaptogyrin 1 | [SYNGR1](http://www.gene.ucl.ac.uk/cgi-bin/nomenclature/searchgenes.pl?field=symbol&anchor=equals&symbol_search=Search&number=100&format=html&sortby=symbol&match=SYNGR1) | guanyl-nucleotide exchange factor activity | [603925](http://www.ncbi.nlm.nih.gov/entrez/dispomim.cgi?id=603925) | **2.02** |
| 1419291_X_AT | growth arrest specific 5 | [GAS5](http://www.gene.ucl.ac.uk/cgi-bin/nomenclature/searchgenes.pl?field=symbol&anchor=equals&symbol_search=Search&number=100&format=html&sortby=symbol&match=GAS5) | guanyl-nucleotide exchange factor activity;3',5'-cAMP binding;cAMP-dependent protein kinase regulator activity |  | **-1.72** |
| 1419292_AT | serine protease HTRA3 | [HTRA3](http://www.gene.ucl.ac.uk/cgi-bin/nomenclature/searchgenes.pl?field=symbol&anchor=equals&symbol_search=Search&number=100&format=html&sortby=symbol&match=HTRA3) | guanyl-nucleotide exchange factor activity;3',5'-cAMP binding;cAMP-dependent protein kinase regulator activity | [608785](http://www.ncbi.nlm.nih.gov/entrez/dispomim.cgi?id=608785) | **-1.3** |
| 1419300_AT | FMS-like tyrosine kinase 1 | [FLT1](http://www.gene.ucl.ac.uk/cgi-bin/nomenclature/searchgenes.pl?field=symbol&anchor=equals&symbol_search=Search&number=100&format=html&sortby=symbol&match=FLT1) | guanyl-nucleotide exchange factor activity;GTP binding;GTPase binding | [165070](http://www.ncbi.nlm.nih.gov/entrez/dispomim.cgi?id=165070) | **2.11** |
| 1419309_AT | glycoprotein 38 | [GP38](http://www.gene.ucl.ac.uk/cgi-bin/nomenclature/searchgenes.pl?field=symbol&anchor=equals&symbol_search=Search&number=100&format=html&sortby=symbol&match=GP38) | guanyl-nucleotide exchange factor activity;GTP binding;GTPase binding |  | **-1.93** |
| 1419315_AT | SLAM family member 9 | [SLAMF9](http://www.gene.ucl.ac.uk/cgi-bin/nomenclature/searchgenes.pl?field=symbol&anchor=equals&symbol_search=Search&number=100&format=html&sortby=symbol&match=SLAMF9) | guanyl-nucleotide exchange factor activity;GTP binding;GTPase binding | [608589](http://www.ncbi.nlm.nih.gov/entrez/dispomim.cgi?id=608589) | **-2.24** |
| 1419351_A_AT | RIKEN cDNA 0610007P06 gene | [0610007P06RIK](http://www.gene.ucl.ac.uk/cgi-bin/nomenclature/searchgenes.pl?field=symbol&anchor=equals&symbol_search=Search&number=100&format=html&sortby=symbol&match=0610007P06RIK) | guanyl-nucleotide exchange factor activity;GTP binding;GTPase binding |  | **1.33** |
| 1419352_AT | RIKEN cDNA 0610007P06 gene | [0610007P06RIK](http://www.gene.ucl.ac.uk/cgi-bin/nomenclature/searchgenes.pl?field=symbol&anchor=equals&symbol_search=Search&number=100&format=html&sortby=symbol&match=0610007P06RIK) | guanyl-nucleotide exchange factor activity;GTPase activator activity |  | **1.34** |
| 1419364_A_AT | ribosomal protein S7 | [RPS7](http://www.gene.ucl.ac.uk/cgi-bin/nomenclature/searchgenes.pl?field=symbol&anchor=equals&symbol_search=Search&number=100&format=html&sortby=symbol&match=RPS7) | guanyl-nucleotide exchange factor activity;phosphatidylinositol binding;Rho guanyl-nucleotide exchange factor activity | [603658](http://www.ncbi.nlm.nih.gov/entrez/dispomim.cgi?id=603658) | **-1.24** |
| 1419365_AT | peroxisomal biogenesis factor 11a | [PEX11A](http://www.gene.ucl.ac.uk/cgi-bin/nomenclature/searchgenes.pl?field=symbol&anchor=equals&symbol_search=Search&number=100&format=html&sortby=symbol&match=PEX11A) | guanyl-nucleotide exchange factor activity;receptor activity;transmembrane receptor protein tyrosine kinase adaptor protein activity | [603866](http://www.ncbi.nlm.nih.gov/entrez/dispomim.cgi?id=603866) | **1.71** |
| 1419367_AT | 2,4-dienoyl CoA reductase 1, mitochondrial | [DECR1](http://www.gene.ucl.ac.uk/cgi-bin/nomenclature/searchgenes.pl?field=symbol&anchor=equals&symbol_search=Search&number=100&format=html&sortby=symbol&match=DECR1) | heat shock protein activity | [222745](http://www.ncbi.nlm.nih.gov/entrez/dispomim.cgi?id=222745) | **1.86** |
| 1419376_AT | RIKEN cDNA 1110018M03 gene | [1110018M03RIK](http://www.gene.ucl.ac.uk/cgi-bin/nomenclature/searchgenes.pl?field=symbol&anchor=equals&symbol_search=Search&number=100&format=html&sortby=symbol&match=1110018M03RIK) | heat shock protein activity |  | **-1.49** |
| 1419380_AT | zinc finger protein 423 | [ZFP423](http://www.gene.ucl.ac.uk/cgi-bin/nomenclature/searchgenes.pl?field=symbol&anchor=equals&symbol_search=Search&number=100&format=html&sortby=symbol&match=ZFP423) | heat shock protein activity;ATP binding |  | **1.81** |
| 1419382_A_AT | dehydrogenase/reductase (SDR family) member 4 | [DHRS4](http://www.gene.ucl.ac.uk/cgi-bin/nomenclature/searchgenes.pl?field=symbol&anchor=equals&symbol_search=Search&number=100&format=html&sortby=symbol&match=DHRS4) | helicase activity;ATP binding;hydrolase activity |  | **2.26** |
| 1419391_AT | myogenin | [MYOG](http://www.gene.ucl.ac.uk/cgi-bin/nomenclature/searchgenes.pl?field=symbol&anchor=equals&symbol_search=Search&number=100&format=html&sortby=symbol&match=MYOG) | helicase activity;ATP-dependent helicase activity;nucleic acid binding;DNA binding;ATP binding | [159980](http://www.ncbi.nlm.nih.gov/entrez/dispomim.cgi?id=159980) | **-12.59** |
| 1419398_A_AT | deleted in polyposis 1 | [DP1](http://www.gene.ucl.ac.uk/cgi-bin/nomenclature/searchgenes.pl?field=symbol&anchor=equals&symbol_search=Search&number=100&format=html&sortby=symbol&match=DP1) | helicase activity;ATP-dependent RNA helicase activity;ATP-dependent helicase activity;nucleic acid binding;ATPase activity;RNA binding;ATP binding;hydrolase activity | [125265](http://www.ncbi.nlm.nih.gov/entrez/dispomim.cgi?id=125265) | **-1.34** |
| 1419428_A_AT | glucosidase, alpha, acid | [GAA](http://www.gene.ucl.ac.uk/cgi-bin/nomenclature/searchgenes.pl?field=symbol&anchor=equals&symbol_search=Search&number=100&format=html&sortby=symbol&match=GAA) | helicase activity;ATP-dependent RNA helicase activity;ATP-dependent helicase activity;nucleic acid binding;ATPase activity;RNA binding;ATP binding;hydrolase activity | [606800](http://www.ncbi.nlm.nih.gov/entrez/dispomim.cgi?id=606800) | **1.31** |
| 1419435_AT | aldehyde oxidase 1 | [AOX1](http://www.gene.ucl.ac.uk/cgi-bin/nomenclature/searchgenes.pl?field=symbol&anchor=equals&symbol_search=Search&number=100&format=html&sortby=symbol&match=AOX1) | helicase activity;ATP-dependent RNA helicase activity;ATP-dependent helicase activity;nucleic acid binding;ATPase activity;RNA binding;ATP binding;hydrolase activity | [602841](http://www.ncbi.nlm.nih.gov/entrez/dispomim.cgi?id=602841) | **1.36** |
| 1419440_AT | ring finger protein 30 | [RNF30](http://www.gene.ucl.ac.uk/cgi-bin/nomenclature/searchgenes.pl?field=symbol&anchor=equals&symbol_search=Search&number=100&format=html&sortby=symbol&match=RNF30) | helicase activity;ATP-dependent RNA helicase activity;ATP-dependent helicase activity;nucleic acid binding;RNA binding;ATPase activity;ATP binding;hydrolase activity | [606474](http://www.ncbi.nlm.nih.gov/entrez/dispomim.cgi?id=606474) | **1.81** |
| 1419442_AT | matrilin 2 | [MATN2](http://www.gene.ucl.ac.uk/cgi-bin/nomenclature/searchgenes.pl?field=symbol&anchor=equals&symbol_search=Search&number=100&format=html&sortby=symbol&match=MATN2) | helicase activity;translation initiation factor activity;nucleic acid binding;ATP-dependent helicase activity;DNA binding;RNA binding;ATP binding;hydrolase activity | [602108](http://www.ncbi.nlm.nih.gov/entrez/dispomim.cgi?id=602108) | **-1.87** |
| 1419443_AT | Sin3-associated polypeptide 18 | [SAP18](http://www.gene.ucl.ac.uk/cgi-bin/nomenclature/searchgenes.pl?field=symbol&anchor=equals&symbol_search=Search&number=100&format=html&sortby=symbol&match=SAP18) | hematopoietin/interferon-class (D200-domain) cytokine receptor activity | [602949](http://www.ncbi.nlm.nih.gov/entrez/dispomim.cgi?id=602949) | **1.3** |
| 1419460_AT | ribonuclease P 14 subunit (human) | [RPP14](http://www.gene.ucl.ac.uk/cgi-bin/nomenclature/searchgenes.pl?field=symbol&anchor=equals&symbol_search=Search&number=100&format=html&sortby=symbol&match=RPP14) | hematopoietin/interferon-class (D200-domain) cytokine receptor activity;receptor activity | [606112](http://www.ncbi.nlm.nih.gov/entrez/dispomim.cgi?id=606112) | **1.24** |
| 1419462_S_AT | gene trap locus 3 | [GTL3](http://www.gene.ucl.ac.uk/cgi-bin/nomenclature/searchgenes.pl?field=symbol&anchor=equals&symbol_search=Search&number=100&format=html&sortby=symbol&match=GTL3) | hematopoietin/interferon-class (D200-domain) cytokine receptor activity;receptor activity |  | **-1.25** |
| 1419467_AT | RIKEN cDNA 1200003C23 gene | [1200003C23RIK](http://www.gene.ucl.ac.uk/cgi-bin/nomenclature/searchgenes.pl?field=symbol&anchor=equals&symbol_search=Search&number=100&format=html&sortby=symbol&match=1200003C23RIK) | hematopoietin/interferon-class (D200-domain) cytokine receptor activity;receptor activity;cytokine binding |  | **1.8** |
| 1419482_AT | complement component 3a receptor 1 | [C3AR1](http://www.gene.ucl.ac.uk/cgi-bin/nomenclature/searchgenes.pl?field=symbol&anchor=equals&symbol_search=Search&number=100&format=html&sortby=symbol&match=C3AR1) | heme binding | [605246](http://www.ncbi.nlm.nih.gov/entrez/dispomim.cgi?id=605246) | **-2.54** |
| 1419483_AT | complement component 3a receptor 1 | [C3AR1](http://www.gene.ucl.ac.uk/cgi-bin/nomenclature/searchgenes.pl?field=symbol&anchor=equals&symbol_search=Search&number=100&format=html&sortby=symbol&match=C3AR1) | heme binding | [605246](http://www.ncbi.nlm.nih.gov/entrez/dispomim.cgi?id=605246) | **-2.84** |
| 1419484_A_AT | glioblastoma amplified sequence | [GBAS](http://www.gene.ucl.ac.uk/cgi-bin/nomenclature/searchgenes.pl?field=symbol&anchor=equals&symbol_search=Search&number=100&format=html&sortby=symbol&match=GBAS) | heme binding;electron transporter activity | [603004](http://www.ncbi.nlm.nih.gov/entrez/dispomim.cgi?id=603004) | **1.24** |
| 1419487_AT | myosin binding protein H | [MYBPH](http://www.gene.ucl.ac.uk/cgi-bin/nomenclature/searchgenes.pl?field=symbol&anchor=equals&symbol_search=Search&number=100&format=html&sortby=symbol&match=MYBPH) | heme binding;electron transporter activity | [160795](http://www.ncbi.nlm.nih.gov/entrez/dispomim.cgi?id=160795) | **-47.03** |
| 1419495_AT | inner mitochondrial membrane peptidase 2-like (S. cerevisiae) | [IMMP2L](http://www.gene.ucl.ac.uk/cgi-bin/nomenclature/searchgenes.pl?field=symbol&anchor=equals&symbol_search=Search&number=100&format=html&sortby=symbol&match=IMMP2L) | heme binding;electron transporter activity | [605977](http://www.ncbi.nlm.nih.gov/entrez/dispomim.cgi?id=605977) | **1.29** |
| 1419499_AT | gb:NM_008149.1 /DB_XREF=gi:6680056 /GEN=Gpam /FEA= |  | heme binding;electron transporter activity |  | **1.47** |
| 1419518_AT | tubulin, alpha 8 | [TUBA8](http://www.gene.ucl.ac.uk/cgi-bin/nomenclature/searchgenes.pl?field=symbol&anchor=equals&symbol_search=Search&number=100&format=html&sortby=symbol&match=TUBA8) | heme binding;ligase activity;electron transporter activity;leucine-tRNA ligase activity;ATP binding;tRNA ligase activity | [605742](http://www.ncbi.nlm.nih.gov/entrez/dispomim.cgi?id=605742) | **1.67** |
| 1419519_AT | insulin-like growth factor 1 | [IGF1](http://www.gene.ucl.ac.uk/cgi-bin/nomenclature/searchgenes.pl?field=symbol&anchor=equals&symbol_search=Search&number=100&format=html&sortby=symbol&match=IGF1) | heme binding;oxidoreductase activity | [147440](http://www.ncbi.nlm.nih.gov/entrez/dispomim.cgi?id=147440) | **-2.03** |
| 1419543_A_AT | splicing factor, arginine/serine-rich 10 (transformer 2 homolog, Drosophila) | [SFRS10](http://www.gene.ucl.ac.uk/cgi-bin/nomenclature/searchgenes.pl?field=symbol&anchor=equals&symbol_search=Search&number=100&format=html&sortby=symbol&match=SFRS10) | heme oxygenase (decyclizing) activity;oxidoreductase activity | [602719](http://www.ncbi.nlm.nih.gov/entrez/dispomim.cgi?id=602719) | **-1.26** |
| 1419545_A_AT | gb:AA987147 /DB_XREF=gi:3167910 /DB_XREF=uc80h04.x |  | heparin-sulfate 2-sulfotransferase activity;transferase activity |  | **-1.45** |
| 1419551_S_AT | serine/threonine kinase 39, STE20/SPS1 homolog (yeast) | [STK39](http://www.gene.ucl.ac.uk/cgi-bin/nomenclature/searchgenes.pl?field=symbol&anchor=equals&symbol_search=Search&number=100&format=html&sortby=symbol&match=STK39) | hexokinase activity;kinase activity;ATP binding;catalytic activity;transferase activity | [607648](http://www.ncbi.nlm.nih.gov/entrez/dispomim.cgi?id=607648) | **1.54** |
| 1419552_AT | enoyl Coenzyme A hydratase domain containing 1 | [ECHDC1](http://www.gene.ucl.ac.uk/cgi-bin/nomenclature/searchgenes.pl?field=symbol&anchor=equals&symbol_search=Search&number=100&format=html&sortby=symbol&match=ECHDC1) | hexokinase activity;kinase activity;catalytic activity;ATP binding;transferase activity |  | **1.5** |
| 1419553_A_AT | RAB geranylgeranyl transferase, b subunit | [RABGGTB](http://www.gene.ucl.ac.uk/cgi-bin/nomenclature/searchgenes.pl?field=symbol&anchor=equals&symbol_search=Search&number=100&format=html&sortby=symbol&match=RABGGTB) | hexokinase activity;kinase activity;catalytic activity;ATP binding;transferase activity | [179080](http://www.ncbi.nlm.nih.gov/entrez/dispomim.cgi?id=179080) | **-1.27** |
| 1419573_A_AT | lectin, galactose binding, soluble 1 | [LGALS1](http://www.gene.ucl.ac.uk/cgi-bin/nomenclature/searchgenes.pl?field=symbol&anchor=equals&symbol_search=Search&number=100&format=html&sortby=symbol&match=LGALS1) | hexokinase activity;kinase activity;catalytic activity;ATP binding;transferase activity | [150570](http://www.ncbi.nlm.nih.gov/entrez/dispomim.cgi?id=150570) | **-1.69** |
| 1419595_A_AT | gamma-glutamyl hydrolase | [GGH](http://www.gene.ucl.ac.uk/cgi-bin/nomenclature/searchgenes.pl?field=symbol&anchor=equals&symbol_search=Search&number=100&format=html&sortby=symbol&match=GGH) | hexokinase activity;kinase activity;phosphotransferase activity, alcohol group as acceptor;ATP binding;galactokinase activity;transferase activity | [601509](http://www.ncbi.nlm.nih.gov/entrez/dispomim.cgi?id=601509) | **-1.57** |
| 1419598_AT | membrane-spanning 4-domains, subfamily A, member 6D | [MS4A6D](http://www.gene.ucl.ac.uk/cgi-bin/nomenclature/searchgenes.pl?field=symbol&anchor=equals&symbol_search=Search&number=100&format=html&sortby=symbol&match=MS4A6D) | high-density lipoprotein binding |  | **-19.63** |
| 1419599_S_AT | membrane-spanning 4-domains, subfamily A, member 11 | [MS4A11](http://www.gene.ucl.ac.uk/cgi-bin/nomenclature/searchgenes.pl?field=symbol&anchor=equals&symbol_search=Search&number=100&format=html&sortby=symbol&match=MS4A11) | histamine N-methyltransferase activity;methyltransferase activity;transferase activity;N-methyltransferase activity |  | **-5.53** |
| 1419603_AT | myeloid cell nuclear differentiation antigen | [IFI204](http://www.gene.ucl.ac.uk/cgi-bin/nomenclature/searchgenes.pl?field=symbol&anchor=equals&symbol_search=Search&number=100&format=html&sortby=symbol&match=IFI204) | histidine-tRNA ligase activity;ligase activity;ATP binding;tRNA ligase activity | [147586](http://www.ncbi.nlm.nih.gov/entrez/dispomim.cgi?id=147586) | **-4.12** |
| 1419606_A_AT | troponin T1, skeletal, slow | [TNNT1](http://www.gene.ucl.ac.uk/cgi-bin/nomenclature/searchgenes.pl?field=symbol&anchor=equals&symbol_search=Search&number=100&format=html&sortby=symbol&match=TNNT1) | histone acetyltransferase activity;protein binding;N-acetyltransferase activity;transferase activity | [191041](http://www.ncbi.nlm.nih.gov/entrez/dispomim.cgi?id=191041) | **-1.25** |
| 1419613_AT | procollagen, type VII, alpha 1 | [COL7A1](http://www.gene.ucl.ac.uk/cgi-bin/nomenclature/searchgenes.pl?field=symbol&anchor=equals&symbol_search=Search&number=100&format=html&sortby=symbol&match=COL7A1) | histone deacetylase activity;protein binding;transcription corepressor activity;transcription factor binding;transcriptional repressor activity;hydrolase activity;specific transcriptional repressor activity | [120120](http://www.ncbi.nlm.nih.gov/entrez/dispomim.cgi?id=120120) | **-1.52** |
| 1419627_S_AT | C-type (calcium dependent, carbohydrate recognition domain) lectin, superfamily member 10 | [CLECSF10](http://www.gene.ucl.ac.uk/cgi-bin/nomenclature/searchgenes.pl?field=symbol&anchor=equals&symbol_search=Search&number=100&format=html&sortby=symbol&match=CLECSF10) | histone methyltransferase activity;protein binding;DNA binding;chromatin binding |  | **-5.27** |
| 1419631_AT | Wiskott-Aldrich syndrome homolog (human) | [WAS](http://www.gene.ucl.ac.uk/cgi-bin/nomenclature/searchgenes.pl?field=symbol&anchor=equals&symbol_search=Search&number=100&format=html&sortby=symbol&match=WAS) | holocytochrome-c synthase activity;lyase activity | [300392](http://www.ncbi.nlm.nih.gov/entrez/dispomim.cgi?id=300392) | **-1.98** |
| 1419639_AT | gb:BB452953 /DB_XREF=gi:16425729 /DB_XREF=BB452953 |  | holocytochrome-c synthase activity;lyase activity |  | **1.44** |
| 1419642_AT | Purine rich element binding protein B | [PURB](http://www.gene.ucl.ac.uk/cgi-bin/nomenclature/searchgenes.pl?field=symbol&anchor=equals&symbol_search=Search&number=100&format=html&sortby=symbol&match=PURB) | hormone activity | [608887](http://www.ncbi.nlm.nih.gov/entrez/dispomim.cgi?id=608887) | **-1.39** |
| 1419646_A_AT | myelin basic protein | [MBP](http://www.gene.ucl.ac.uk/cgi-bin/nomenclature/searchgenes.pl?field=symbol&anchor=equals&symbol_search=Search&number=100&format=html&sortby=symbol&match=MBP) | hormone activity | [159430](http://www.ncbi.nlm.nih.gov/entrez/dispomim.cgi?id=159430) | **2.13** |
| 1419651_AT | RIKEN cDNA 2610200G18 gene | [2610200G18RIK](http://www.gene.ucl.ac.uk/cgi-bin/nomenclature/searchgenes.pl?field=symbol&anchor=equals&symbol_search=Search&number=100&format=html&sortby=symbol&match=2610200G18RIK) | hormone activity;neuropeptide hormone activity |  | **1.44** |
| 1419659_S_AT | cysteine-rich hydrophobic domain 2 | [CHIC2](http://www.gene.ucl.ac.uk/cgi-bin/nomenclature/searchgenes.pl?field=symbol&anchor=equals&symbol_search=Search&number=100&format=html&sortby=symbol&match=CHIC2) | hormone binding;protein binding;protein heterodimerization activity;protein self binding;receptor activity | [604332](http://www.ncbi.nlm.nih.gov/entrez/dispomim.cgi?id=604332) | **-1.34** |
| 1419665_A_AT | nuclear protein 1 | [NUPR1](http://www.gene.ucl.ac.uk/cgi-bin/nomenclature/searchgenes.pl?field=symbol&anchor=equals&symbol_search=Search&number=100&format=html&sortby=symbol&match=NUPR1) | hormone binding;protein heterodimerization activity;protein binding;protein self binding;receptor activity |  | **-1.43** |
| 1419666_X_AT | nuclear protein 1 | [NUPR1](http://www.gene.ucl.ac.uk/cgi-bin/nomenclature/searchgenes.pl?field=symbol&anchor=equals&symbol_search=Search&number=100&format=html&sortby=symbol&match=NUPR1) | hormone binding;protein heterodimerization activity;protein binding;protein self binding;receptor activity |  | **-1.41** |
| 1419684_AT | chemokine (C-C motif) ligand 8 | [CCL8](http://www.gene.ucl.ac.uk/cgi-bin/nomenclature/searchgenes.pl?field=symbol&anchor=equals&symbol_search=Search&number=100&format=html&sortby=symbol&match=CCL8) | hydrogen ion transporter activity | [602283](http://www.ncbi.nlm.nih.gov/entrez/dispomim.cgi?id=602283) | **-3.98** |
| 1419687_AT | RIKEN cDNA D930010J01 gene | [D930010J01RIK](http://www.gene.ucl.ac.uk/cgi-bin/nomenclature/searchgenes.pl?field=symbol&anchor=equals&symbol_search=Search&number=100&format=html&sortby=symbol&match=D930010J01RIK) | hydrogen ion transporter activity |  | **1.41** |
| 1419703_AT | procollagen, type V, alpha 3 | [COL5A3](http://www.gene.ucl.ac.uk/cgi-bin/nomenclature/searchgenes.pl?field=symbol&anchor=equals&symbol_search=Search&number=100&format=html&sortby=symbol&match=COL5A3) | hydrogen ion transporter activity;hydrolase activity | [120216](http://www.ncbi.nlm.nih.gov/entrez/dispomim.cgi?id=120216) | **-2.23** |
| 1419706_A_AT | A kinase (PRKA) anchor protein (gravin) 12 | [AKAP12](http://www.gene.ucl.ac.uk/cgi-bin/nomenclature/searchgenes.pl?field=symbol&anchor=equals&symbol_search=Search&number=100&format=html&sortby=symbol&match=AKAP12) | hydrogen ion transporter activity;hydrolase activity | [604698](http://www.ncbi.nlm.nih.gov/entrez/dispomim.cgi?id=604698) | **1.4** |
| 1419736_A_AT | eukaryotic translation initiation factor 1A, Y-linked | [EIF1AY](http://www.gene.ucl.ac.uk/cgi-bin/nomenclature/searchgenes.pl?field=symbol&anchor=equals&symbol_search=Search&number=100&format=html&sortby=symbol&match=EIF1AY) | hydrogen ion transporter activity;hydrolase activity | [400014](http://www.ncbi.nlm.nih.gov/entrez/dispomim.cgi?id=400014) | **-1.28** |
| 1419739_AT | tropomyosin 2, beta | [TPM2](http://www.gene.ucl.ac.uk/cgi-bin/nomenclature/searchgenes.pl?field=symbol&anchor=equals&symbol_search=Search&number=100&format=html&sortby=symbol&match=TPM2) | hydrogen-exporting ATPase activity, phosphorylative mechanism | [190990](http://www.ncbi.nlm.nih.gov/entrez/dispomim.cgi?id=190990) | **1.24** |
| 1419741_AT | suppressor of Ty 16 homolog (S. cerevisiae) | [SUPT16H](http://www.gene.ucl.ac.uk/cgi-bin/nomenclature/searchgenes.pl?field=symbol&anchor=equals&symbol_search=Search&number=100&format=html&sortby=symbol&match=SUPT16H) | hydrogen-exporting ATPase activity, phosphorylative mechanism;ATP binding | [605012](http://www.ncbi.nlm.nih.gov/entrez/dispomim.cgi?id=605012) | **-1.24** |
| 1419743_S_AT | coactivator-associated arginine methyltransferase 1 | [CARM1](http://www.gene.ucl.ac.uk/cgi-bin/nomenclature/searchgenes.pl?field=symbol&anchor=equals&symbol_search=Search&number=100&format=html&sortby=symbol&match=CARM1) | hydrogen-exporting ATPase activity, phosphorylative mechanism;hydrogen-transporting ATP synthase activity, rotational mechanism;hydrogen ion transporter activity;ATP binding;hydrogen-transporting ATPase activity, rotational mechanism;hydrolase activity | [603934](http://www.ncbi.nlm.nih.gov/entrez/dispomim.cgi?id=603934) | **1.22** |
| 1419748_AT | ATP-binding cassette, sub-family D (ALD), member 2 | [ABCD2](http://www.gene.ucl.ac.uk/cgi-bin/nomenclature/searchgenes.pl?field=symbol&anchor=equals&symbol_search=Search&number=100&format=html&sortby=symbol&match=ABCD2) | hydrogen-exporting ATPase activity, phosphorylative mechanism;hydrogen-transporting ATP synthase activity, rotational mechanism;hydrogen ion transporter activity;ATP binding;hydrogen-transporting ATPase activity, rotational mechanism;hydrolase activity;hydrolase activity, acting on acid anhydrides, catalyzing transmembrane movement of substances | [601081](http://www.ncbi.nlm.nih.gov/entrez/dispomim.cgi?id=601081) | **1.51** |
| 1419754_AT | myosin Va | [MYO5A](http://www.gene.ucl.ac.uk/cgi-bin/nomenclature/searchgenes.pl?field=symbol&anchor=equals&symbol_search=Search&number=100&format=html&sortby=symbol&match=MYO5A) | hydrogen-exporting ATPase activity, phosphorylative mechanism;hydrogen-transporting ATP synthase activity, rotational mechanism;hydrogen ion transporter activity;hydrogen-transporting ATPase activity, rotational mechanism;ATP binding;hydrolase activity, acting on acid anhydrides, catalyzing transmembrane movement of substances;hydrolase activity | [160777](http://www.ncbi.nlm.nih.gov/entrez/dispomim.cgi?id=160777) | **-1.88** |
| 1419757_AT | phosphatidylinositol transfer protein, membrane-associated 2 | [PITPNM2](http://www.gene.ucl.ac.uk/cgi-bin/nomenclature/searchgenes.pl?field=symbol&anchor=equals&symbol_search=Search&number=100&format=html&sortby=symbol&match=PITPNM2) | hydrogen-exporting ATPase activity, phosphorylative mechanism;hydrogen-transporting ATP synthase activity, rotational mechanism;hydrogen ion transporter activity;hydrogen-transporting ATPase activity, rotational mechanism;ATP binding;hydrolase activity;hydrolase activity, acting on acid anhydrides, catalyzing transmembrane movement of substances | [608920](http://www.ncbi.nlm.nih.gov/entrez/dispomim.cgi?id=608920) | **1.28** |
| 1419758_AT | ATP-binding cassette, sub-family B (MDR/TAP), member 1A | [ABCB1A](http://www.gene.ucl.ac.uk/cgi-bin/nomenclature/searchgenes.pl?field=symbol&anchor=equals&symbol_search=Search&number=100&format=html&sortby=symbol&match=ABCB1A) | hydrogen-exporting ATPase activity, phosphorylative mechanism;hydrogen-transporting ATP synthase activity, rotational mechanism;hydrogen ion transporter activity;hydrogen-transporting ATPase activity, rotational mechanism;ATP binding;hydrolase activity;hydrolase activity, acting on acid anhydrides, catalyzing transmembrane movement of substances |  | **2.95** |
| 1419759_AT | ATP-binding cassette, sub-family B (MDR/TAP), member 1A | [ABCB1A](http://www.gene.ucl.ac.uk/cgi-bin/nomenclature/searchgenes.pl?field=symbol&anchor=equals&symbol_search=Search&number=100&format=html&sortby=symbol&match=ABCB1A) | hydrogen-exporting ATPase activity, phosphorylative mechanism;hydrogen-transporting ATP synthase activity, rotational mechanism;hydrogen ion transporter activity;hydrogen-transporting ATPase activity, rotational mechanism;hydrolase activity |  | **2.34** |
| 1419764_AT | chitinase 3-like 3 | [CHI3L3](http://www.gene.ucl.ac.uk/cgi-bin/nomenclature/searchgenes.pl?field=symbol&anchor=equals&symbol_search=Search&number=100&format=html&sortby=symbol&match=CHI3L3) | hydrogen-exporting ATPase activity, phosphorylative mechanism;hydrogen-transporting ATP synthase activity, rotational mechanism;hydrogen ion transporter activity;hydrogen-transporting ATPase activity, rotational mechanism;hydrolase activity |  | **-22.75** |
| 1419814_S_AT | S100 calcium binding protein A1 | [S100A1](http://www.gene.ucl.ac.uk/cgi-bin/nomenclature/searchgenes.pl?field=symbol&anchor=equals&symbol_search=Search&number=100&format=html&sortby=symbol&match=S100A1) | hydrogen-exporting ATPase activity, phosphorylative mechanism;hydrogen-transporting ATP synthase activity, rotational mechanism;hydrogen-transporting ATPase activity, rotational mechanism | [176940](http://www.ncbi.nlm.nih.gov/entrez/dispomim.cgi?id=176940) | **1.46** |
| 1419829_A_AT | gb:AW049055 /DB_XREF=gi:5909584 /DB_XREF=UI-M-BH1- |  | hydrogen-exporting ATPase activity, phosphorylative mechanism;hydrogen-transporting ATP synthase activity, rotational mechanism;hydrogen-transporting ATPase activity, rotational mechanism |  | **-1.51** |
| 1419833_S_AT | RIKEN cDNA E030006K04 gene | [E030006K04RIK](http://www.gene.ucl.ac.uk/cgi-bin/nomenclature/searchgenes.pl?field=symbol&anchor=equals&symbol_search=Search&number=100&format=html&sortby=symbol&match=E030006K04RIK) | hydrogen-exporting ATPase activity, phosphorylative mechanism;hydrogen-transporting ATP synthase activity, rotational mechanism;lipid binding;hydrogen ion transporter activity;hydrogen-transporting ATPase activity, rotational mechanism |  | **1.56** |
| 1419834_X_AT | MAP/microtubule affinity-regulating kinase 1 | [MARK1](http://www.gene.ucl.ac.uk/cgi-bin/nomenclature/searchgenes.pl?field=symbol&anchor=equals&symbol_search=Search&number=100&format=html&sortby=symbol&match=MARK1) | hydrogen-exporting ATPase activity, phosphorylative mechanism;nucleotide binding;hydrogen-transporting ATP synthase activity, rotational mechanism;hydrogen ion transporter activity;ATP binding;hydrogen-transporting ATPase activity, rotational mechanism;hydrolase activity, acting on acid anhydrides, catalyzing transmembrane movement of substances;hydrolase activity | [606511](http://www.ncbi.nlm.nih.gov/entrez/dispomim.cgi?id=606511) | **1.26** |
| 1419869_S_AT | high density lipoprotein (HDL) binding protein | [HDLBP](http://www.gene.ucl.ac.uk/cgi-bin/nomenclature/searchgenes.pl?field=symbol&anchor=equals&symbol_search=Search&number=100&format=html&sortby=symbol&match=HDLBP) | hydrogen-transporting ATP synthase activity, rotational mechanism;hydrogen ion transporter activity;hydrogen-transporting ATPase activity, rotational mechanism | [142695](http://www.ncbi.nlm.nih.gov/entrez/dispomim.cgi?id=142695) | **1.36** |
| 1419872_AT | gb:AI323359 /DB_XREF=gi:4057788 /DB_XREF=mi30h09.x |  | hydrogen-transporting ATP synthase activity, rotational mechanism;hydrogen ion transporter activity;hydrogen-transporting ATPase activity, rotational mechanism |  | **-1.68** |
| 1419873_S_AT | colony stimulating factor 1 receptor | [CSF1R](http://www.gene.ucl.ac.uk/cgi-bin/nomenclature/searchgenes.pl?field=symbol&anchor=equals&symbol_search=Search&number=100&format=html&sortby=symbol&match=CSF1R) | hydrogen-transporting ATP synthase activity, rotational mechanism;hydrogen ion transporter activity;hydrogen-transporting ATPase activity, rotational mechanism | [164770](http://www.ncbi.nlm.nih.gov/entrez/dispomim.cgi?id=164770) | **-1.85** |
| 1419879_S_AT | tripartite motif protein 25 | [TRIM25](http://www.gene.ucl.ac.uk/cgi-bin/nomenclature/searchgenes.pl?field=symbol&anchor=equals&symbol_search=Search&number=100&format=html&sortby=symbol&match=TRIM25) | hydrogen-transporting ATP synthase activity, rotational mechanism;hydrogen ion transporter activity;hydrogen-transporting ATPase activity, rotational mechanism;hydrolase activity | [600453](http://www.ncbi.nlm.nih.gov/entrez/dispomim.cgi?id=600453) | **-1.57** |
| 1419917_S_AT | RIKEN cDNA 3930401E15 gene | [TMED7](http://www.gene.ucl.ac.uk/cgi-bin/nomenclature/searchgenes.pl?field=symbol&anchor=equals&symbol_search=Search&number=100&format=html&sortby=symbol&match=TMED7) | hydrogen-transporting ATP synthase activity, rotational mechanism;hydrogen-transporting ATPase activity, rotational mechanism |  | **-1.28** |
| 1419918_AT | gb:AW545765 /DB_XREF=gi:7188182 /DB_XREF=C0198A06- |  | hydrolase activity |  | **-1.43** |
| 1419925_S_AT | gb:AV259382 /DB_XREF=gi:15405370 /DB_XREF=AV259382 |  | hydrolase activity |  | **-1.4** |
| 1419954_S_AT | testis expressed gene 27 | [TEX27](http://www.gene.ucl.ac.uk/cgi-bin/nomenclature/searchgenes.pl?field=symbol&anchor=equals&symbol_search=Search&number=100&format=html&sortby=symbol&match=TEX27) | hydrolase activity | [607455](http://www.ncbi.nlm.nih.gov/entrez/dispomim.cgi?id=607455) | **1.26** |
| 1419978_S_AT | DNA segment, Chr 10, ERATO Doi 610, expressed | [D10ERTD610E](http://www.gene.ucl.ac.uk/cgi-bin/nomenclature/searchgenes.pl?field=symbol&anchor=equals&symbol_search=Search&number=100&format=html&sortby=symbol&match=D10ERTD610E) | hydrolase activity |  | **-1.35** |
| 1420028_S_AT | minichromosome maintenance deficient 3 (S. cerevisiae) | [MCM3](http://www.gene.ucl.ac.uk/cgi-bin/nomenclature/searchgenes.pl?field=symbol&anchor=equals&symbol_search=Search&number=100&format=html&sortby=symbol&match=MCM3) | hydrolase activity | [602693](http://www.ncbi.nlm.nih.gov/entrez/dispomim.cgi?id=602693) | **-1.58** |
| 1420058_S_AT | RIKEN cDNA 2410166I05 gene | [2410166I05RIK](http://www.gene.ucl.ac.uk/cgi-bin/nomenclature/searchgenes.pl?field=symbol&anchor=equals&symbol_search=Search&number=100&format=html&sortby=symbol&match=2410166I05RIK) | hydrolase activity |  | **1.31** |
| 1420091_S_AT | zinc finger, CW-type with coiled-coil domain 3 | [ZCWCC3](http://www.gene.ucl.ac.uk/cgi-bin/nomenclature/searchgenes.pl?field=symbol&anchor=equals&symbol_search=Search&number=100&format=html&sortby=symbol&match=ZCWCC3) | hydrolase activity |  | **-1.52** |
| 1420124_S_AT | T-cell leukemia translocation altered gene | [TCTA](http://www.gene.ucl.ac.uk/cgi-bin/nomenclature/searchgenes.pl?field=symbol&anchor=equals&symbol_search=Search&number=100&format=html&sortby=symbol&match=TCTA) | hydrolase activity | [600690](http://www.ncbi.nlm.nih.gov/entrez/dispomim.cgi?id=600690) | **1.36** |
| 1420142_S_AT | proliferation-associated 2G4 | [PA2G4](http://www.gene.ucl.ac.uk/cgi-bin/nomenclature/searchgenes.pl?field=symbol&anchor=equals&symbol_search=Search&number=100&format=html&sortby=symbol&match=PA2G4) | hydrolase activity | [602145](http://www.ncbi.nlm.nih.gov/entrez/dispomim.cgi?id=602145) | **-1.38** |
| 1420150_AT | RIKEN cDNA 4930422J18 gene | [4930422J18RIK](http://www.gene.ucl.ac.uk/cgi-bin/nomenclature/searchgenes.pl?field=symbol&anchor=equals&symbol_search=Search&number=100&format=html&sortby=symbol&match=4930422J18RIK) | hydrolase activity |  | **-1.32** |
| 1420196_S_AT | TBC1 domain family, member 14 | [TBC1D14](http://www.gene.ucl.ac.uk/cgi-bin/nomenclature/searchgenes.pl?field=symbol&anchor=equals&symbol_search=Search&number=100&format=html&sortby=symbol&match=TBC1D14) | hydrolase activity |  | **-1.46** |
| 1420249_S_AT | chemokine (C-C motif) ligand 6 | [CCL6](http://www.gene.ucl.ac.uk/cgi-bin/nomenclature/searchgenes.pl?field=symbol&anchor=equals&symbol_search=Search&number=100&format=html&sortby=symbol&match=CCL6) | hydrolase activity |  | **-2.12** |
| 1420361_AT | solute carrier family 11 (proton-coupled divalent metal ion transporters), member 1 | [SLC11A1](http://www.gene.ucl.ac.uk/cgi-bin/nomenclature/searchgenes.pl?field=symbol&anchor=equals&symbol_search=Search&number=100&format=html&sortby=symbol&match=SLC11A1) | hydrolase activity | [600266](http://www.ncbi.nlm.nih.gov/entrez/dispomim.cgi?id=600266) | **-1.88** |
| 1420377_AT | sialyltransferase 8 (alpha-2, 8-sialyltransferase) B | [SIAT8B](http://www.gene.ucl.ac.uk/cgi-bin/nomenclature/searchgenes.pl?field=symbol&anchor=equals&symbol_search=Search&number=100&format=html&sortby=symbol&match=SIAT8B) | hydrolase activity, acting on acid anhydrides, catalyzing transmembrane movement of substances;hydrolase activity;copper ion transporter activity;ATP binding;metal ion binding;ATPase activity, coupled to transmembrane movement of ions, phosphorylative mechanism;copper ion binding;copper-exporting ATPase activity;metal ion transporter activity;magnesium ion binding;hydrogen/potassium-exchanging ATPase activity;catalytic activity | [602546](http://www.ncbi.nlm.nih.gov/entrez/dispomim.cgi?id=602546) | **-1.72** |
| 1420381_A_AT | ribosomal protein L31 | [RPL31](http://www.gene.ucl.ac.uk/cgi-bin/nomenclature/searchgenes.pl?field=symbol&anchor=equals&symbol_search=Search&number=100&format=html&sortby=symbol&match=RPL31) | hydrolase activity, acting on glycosyl bonds;hydrolase activity |  | **-1.46** |
| 1420394_S_AT | glycoprotein 49 A | [GP49A](http://www.gene.ucl.ac.uk/cgi-bin/nomenclature/searchgenes.pl?field=symbol&anchor=equals&symbol_search=Search&number=100&format=html&sortby=symbol&match=GP49A) | hydrolase activity, acting on glycosyl bonds;hydrolase activity |  | **-5.57** |
| 1420398_AT | regulator of G-protein signaling 18 | [RGS18](http://www.gene.ucl.ac.uk/cgi-bin/nomenclature/searchgenes.pl?field=symbol&anchor=equals&symbol_search=Search&number=100&format=html&sortby=symbol&match=RGS18) | hydrolase activity, acting on glycosyl bonds;hydrolase activity | [607192](http://www.ncbi.nlm.nih.gov/entrez/dispomim.cgi?id=607192) | **-3.54** |
| 1420408_A_AT | ATP-binding cassette, sub-family C (CFTR/MRP), member 9 | [ABCC9](http://www.gene.ucl.ac.uk/cgi-bin/nomenclature/searchgenes.pl?field=symbol&anchor=equals&symbol_search=Search&number=100&format=html&sortby=symbol&match=ABCC9) | hydrolase activity;acylphosphatase activity | [601439](http://www.ncbi.nlm.nih.gov/entrez/dispomim.cgi?id=601439) | **1.72** |
| 1420442_AT | calcium channel, voltage-dependent, L type, alpha 1S subunit | [CACNA1S](http://www.gene.ucl.ac.uk/cgi-bin/nomenclature/searchgenes.pl?field=symbol&anchor=equals&symbol_search=Search&number=100&format=html&sortby=symbol&match=CACNA1S) | hydrolase activity;ATP binding;ATP-dependent helicase activity;estrogen receptor binding;nucleic acid binding;ATP-dependent RNA helicase activity;RNA binding;transcription corepressor activity;helicase activity;receptor binding | [114208](http://www.ncbi.nlm.nih.gov/entrez/dispomim.cgi?id=114208) | **1.55** |
| 1420464_S_AT | paired-Ig-like receptor A4 | [PIRA1](http://www.gene.ucl.ac.uk/cgi-bin/nomenclature/searchgenes.pl?field=symbol&anchor=equals&symbol_search=Search&number=100&format=html&sortby=symbol&match=PIRA1) | hydrolase activity;cofactor binding;zinc ion binding;transferase activity;[acyl-carrier protein] S-malonyltransferase activity;oxidoreductase activity;oleoyl-[acyl-carrier protein] hydrolase activity;hydrolase activity, acting on ester bonds;lyase activity;alcohol dehydrogenase activity, zinc-dependent;S-adenosylmethionine-dependent methyltransferase activity;catalytic activity;fatty-acid synthase activity;acyltransferase activity |  | **-2.79** |
| 1420473_AT | myotrophin | [MTPN](http://www.gene.ucl.ac.uk/cgi-bin/nomenclature/searchgenes.pl?field=symbol&anchor=equals&symbol_search=Search&number=100&format=html&sortby=symbol&match=MTPN) | hydrolase activity;diphosphoinositol-polyphosphate diphosphatase activity | [606484](http://www.ncbi.nlm.nih.gov/entrez/dispomim.cgi?id=606484) | **-1.34** |
| 1420474_AT | myotrophin | [MTPN](http://www.gene.ucl.ac.uk/cgi-bin/nomenclature/searchgenes.pl?field=symbol&anchor=equals&symbol_search=Search&number=100&format=html&sortby=symbol&match=MTPN) | hydrolase activity;endopeptidase activity;peptidase activity | [606484](http://www.ncbi.nlm.nih.gov/entrez/dispomim.cgi?id=606484) | **-1.47** |
| 1420475_AT | myotrophin | [MTPN](http://www.gene.ucl.ac.uk/cgi-bin/nomenclature/searchgenes.pl?field=symbol&anchor=equals&symbol_search=Search&number=100&format=html&sortby=symbol&match=MTPN) | hydrolase activity;ferric iron binding;carboxypeptidase activity;zinc ion binding;peptidase activity;carboxypeptidase A activity;metallocarboxypeptidase activity;catalytic activity;metallopeptidase activity | [606484](http://www.ncbi.nlm.nih.gov/entrez/dispomim.cgi?id=606484) | **-1.37** |
| 1420476_A_AT | nucleosome assembly protein 1-like 1 | [NAP1L1](http://www.gene.ucl.ac.uk/cgi-bin/nomenclature/searchgenes.pl?field=symbol&anchor=equals&symbol_search=Search&number=100&format=html&sortby=symbol&match=NAP1L1) | hydrolase activity;growth factor activity;zinc ion binding;procollagen C-endopeptidase activity;astacin activity;cytokine activity;calcium ion binding;metalloendopeptidase activity;metallopeptidase activity | [164060](http://www.ncbi.nlm.nih.gov/entrez/dispomim.cgi?id=164060) | **-1.44** |
| 1420478_AT | nucleosome assembly protein 1-like 1 | [NAP1L1](http://www.gene.ucl.ac.uk/cgi-bin/nomenclature/searchgenes.pl?field=symbol&anchor=equals&symbol_search=Search&number=100&format=html&sortby=symbol&match=NAP1L1) | hydrolase activity;hydroxyacylglutathione hydrolase activity | [164060](http://www.ncbi.nlm.nih.gov/entrez/dispomim.cgi?id=164060) | **-1.4** |
| 1420479_A_AT | nucleosome assembly protein 1-like 1 | [NAP1L1](http://www.gene.ucl.ac.uk/cgi-bin/nomenclature/searchgenes.pl?field=symbol&anchor=equals&symbol_search=Search&number=100&format=html&sortby=symbol&match=NAP1L1) | hydrolase activity;peptidase activity | [164060](http://www.ncbi.nlm.nih.gov/entrez/dispomim.cgi?id=164060) | **-1.47** |
| 1420502_AT | spermidine/spermine N1-acetyl transferase 1 | [SAT1](http://www.gene.ucl.ac.uk/cgi-bin/nomenclature/searchgenes.pl?field=symbol&anchor=equals&symbol_search=Search&number=100&format=html&sortby=symbol&match=SAT1) | hydrolase activity;peptidase activity |  | **-1.7** |
| 1420519_A_AT | Era (G-protein)-like 1 (E. coli) | [ERAL1](http://www.gene.ucl.ac.uk/cgi-bin/nomenclature/searchgenes.pl?field=symbol&anchor=equals&symbol_search=Search&number=100&format=html&sortby=symbol&match=ERAL1) | hydrolase activity;protein phosphatase inhibitor activity;endonuclease activity;type 1 serine/threonine specific protein phosphatase inhibitor activity;protein binding;protein phosphatase type 1 regulator activity;ribonuclease E activity;nuclease activity;magnesium ion binding;RNA binding | [607435](http://www.ncbi.nlm.nih.gov/entrez/dispomim.cgi?id=607435) | **1.31** |
| 1420533_AT | guanylate cyclase 1, soluble, alpha 3 | [GUCY1A3](http://www.gene.ucl.ac.uk/cgi-bin/nomenclature/searchgenes.pl?field=symbol&anchor=equals&symbol_search=Search&number=100&format=html&sortby=symbol&match=GUCY1A3) | hydrolase activity;protein phosphatase type 2A activity;CTD phosphatase activity;protein phosphatase type 1 activity;manganese ion binding;protein serine/threonine phosphatase activity;phosphoprotein phosphatase activity;myosin phosphatase activity;protein phosphatase type 2C activity | [139396](http://www.ncbi.nlm.nih.gov/entrez/dispomim.cgi?id=139396) | **1.37** |
| 1420534_AT | guanylate cyclase 1, soluble, alpha 3 | [GUCY1A3](http://www.gene.ucl.ac.uk/cgi-bin/nomenclature/searchgenes.pl?field=symbol&anchor=equals&symbol_search=Search&number=100&format=html&sortby=symbol&match=GUCY1A3) | hydrolase activity;protein phosphatase type 2A activity;protein binding;CTD phosphatase activity;protein phosphatase type 1 activity;manganese ion binding;protein serine/threonine phosphatase activity;phosphoprotein phosphatase activity;myosin phosphatase activity;protein phosphatase type 2C activity | [139396](http://www.ncbi.nlm.nih.gov/entrez/dispomim.cgi?id=139396) | **1.6** |
| 1420542_AT | open reading frame 28 | [ORF28](http://www.gene.ucl.ac.uk/cgi-bin/nomenclature/searchgenes.pl?field=symbol&anchor=equals&symbol_search=Search&number=100&format=html&sortby=symbol&match=ORF28) | hydrolase activity;protein phosphatase type 2A activity;protein binding;CTD phosphatase activity;protein phosphatase type 1 activity;manganese ion binding;protein serine/threonine phosphatase activity;phosphoprotein phosphatase activity;myosin phosphatase activity;protein phosphatase type 2C activity |  | **2.11** |
| 1420543_AT | open reading frame 28 | [ORF28](http://www.gene.ucl.ac.uk/cgi-bin/nomenclature/searchgenes.pl?field=symbol&anchor=equals&symbol_search=Search&number=100&format=html&sortby=symbol&match=ORF28) | hydrolase activity;zinc ion binding;ATP binding;ATP-dependent helicase activity;nucleic acid binding;ubiquitin-protein ligase activity;chromatin binding;DNA binding;helicase activity |  | **1.73** |
| 1420619_A_AT | amino-terminal enhancer of split | [AES](http://www.gene.ucl.ac.uk/cgi-bin/nomenclature/searchgenes.pl?field=symbol&anchor=equals&symbol_search=Search&number=100&format=html&sortby=symbol&match=AES) | hydroxymethyl-, formyl- and related transferase activity;formyltetrahydrofolate dehydrogenase activity;cofactor binding;oxidoreductase activity | [600188](http://www.ncbi.nlm.nih.gov/entrez/dispomim.cgi?id=600188) | **1.34** |
| 1420629_A_AT | DnaJ (Hsp40) homolog, subfamily A, member 3 | [DNAJA3](http://www.gene.ucl.ac.uk/cgi-bin/nomenclature/searchgenes.pl?field=symbol&anchor=equals&symbol_search=Search&number=100&format=html&sortby=symbol&match=DNAJA3) | hypoxanthine phosphoribosyltransferase activity;transferase activity, transferring glycosyl groups;magnesium ion binding;transferase activity | [608382](http://www.ncbi.nlm.nih.gov/entrez/dispomim.cgi?id=608382) | **1.56** |
| 1420631_A_AT | bladder cancer associated protein homolog (human) | [BLCAP](http://www.gene.ucl.ac.uk/cgi-bin/nomenclature/searchgenes.pl?field=symbol&anchor=equals&symbol_search=Search&number=100&format=html&sortby=symbol&match=BLCAP) | ice binding;N-acetylneuraminic acid phosphate synthase activity |  | **1.63** |
| 1420635_A_AT | T-cell, immune regulator 1 | [TCIRG1](http://www.gene.ucl.ac.uk/cgi-bin/nomenclature/searchgenes.pl?field=symbol&anchor=equals&symbol_search=Search&number=100&format=html&sortby=symbol&match=TCIRG1) | ice binding;N-acetylneuraminic acid phosphate synthase activity | [604592](http://www.ncbi.nlm.nih.gov/entrez/dispomim.cgi?id=604592) | **-1.85** |
| 1420641_A_AT | sulfide quinone reductase-like (yeast) | [SQRDL](http://www.gene.ucl.ac.uk/cgi-bin/nomenclature/searchgenes.pl?field=symbol&anchor=equals&symbol_search=Search&number=100&format=html&sortby=symbol&match=SQRDL) | IgG binding;receptor activity;IgG receptor activity |  | **1.67** |
| 1420653_AT | transforming growth factor, beta 1 | [TGFB1](http://www.gene.ucl.ac.uk/cgi-bin/nomenclature/searchgenes.pl?field=symbol&anchor=equals&symbol_search=Search&number=100&format=html&sortby=symbol&match=TGFB1) | IgG binding;receptor activity;IgG receptor activity | [190180](http://www.ncbi.nlm.nih.gov/entrez/dispomim.cgi?id=190180) | **-1.58** |
| 1420654_A_AT | glucan (1,4-alpha-), branching enzyme 1 | [GBE1](http://www.gene.ucl.ac.uk/cgi-bin/nomenclature/searchgenes.pl?field=symbol&anchor=equals&symbol_search=Search&number=100&format=html&sortby=symbol&match=GBE1) | IgG binding;receptor activity;receptor signaling protein activity | [607839](http://www.ncbi.nlm.nih.gov/entrez/dispomim.cgi?id=607839) | **1.88** |
| 1420661_A_AT | RIKEN cDNA 4933439F18 gene | [4933439F18RIK](http://www.gene.ucl.ac.uk/cgi-bin/nomenclature/searchgenes.pl?field=symbol&anchor=equals&symbol_search=Search&number=100&format=html&sortby=symbol&match=4933439F18RIK) | IgG binding;receptor activity;receptor signaling protein activity |  | **1.53** |
| 1420682_AT | cholinergic receptor, nicotinic, beta polypeptide 1 (muscle) | [CHRNB1](http://www.gene.ucl.ac.uk/cgi-bin/nomenclature/searchgenes.pl?field=symbol&anchor=equals&symbol_search=Search&number=100&format=html&sortby=symbol&match=CHRNB1) | IgG binding;receptor activity;receptor signaling protein activity | [100710](http://www.ncbi.nlm.nih.gov/entrez/dispomim.cgi?id=100710) | **-2.47** |
| 1420686_AT | crystallin, beta A4 | [CRYBA4](http://www.gene.ucl.ac.uk/cgi-bin/nomenclature/searchgenes.pl?field=symbol&anchor=equals&symbol_search=Search&number=100&format=html&sortby=symbol&match=CRYBA4) | IgG binding;receptor signaling protein activity;receptor activity | [123631](http://www.ncbi.nlm.nih.gov/entrez/dispomim.cgi?id=123631) | **3.43** |
| 1420693_AT | myomesin 1 | [MYOM1](http://www.gene.ucl.ac.uk/cgi-bin/nomenclature/searchgenes.pl?field=symbol&anchor=equals&symbol_search=Search&number=100&format=html&sortby=symbol&match=MYOM1) | inorganic anion exchanger activity | [603508](http://www.ncbi.nlm.nih.gov/entrez/dispomim.cgi?id=603508) | **1.21** |
| 1420697_AT | solute carrier family 15, member 3 | [SLC15A3](http://www.gene.ucl.ac.uk/cgi-bin/nomenclature/searchgenes.pl?field=symbol&anchor=equals&symbol_search=Search&number=100&format=html&sortby=symbol&match=SLC15A3) | inositol/phosphatidylinositol phosphatase activity;inositol-polyphosphate 5-phosphatase activity;hydrolase activity |  | **-4.65** |
| 1420699_AT | C-type (calcium dependent, carbohydrate recognition domain) lectin, superfamily member 12 | [CLECSF12](http://www.gene.ucl.ac.uk/cgi-bin/nomenclature/searchgenes.pl?field=symbol&anchor=equals&symbol_search=Search&number=100&format=html&sortby=symbol&match=CLECSF12) | inositol/phosphatidylinositol phosphatase activity;RNA binding;polyphosphoinositide phosphatase activity;hydrolase activity | [606264](http://www.ncbi.nlm.nih.gov/entrez/dispomim.cgi?id=606264) | **-5.57** |
| 1420703_AT | colony stimulating factor 2 receptor, alpha, low-affinity (granulocyte-macrophage) | [CSF2RA](http://www.gene.ucl.ac.uk/cgi-bin/nomenclature/searchgenes.pl?field=symbol&anchor=equals&symbol_search=Search&number=100&format=html&sortby=symbol&match=CSF2RA) | insulin-like growth factor binding;protein binding | 425000 306250 | **-2.07** |
| 1420745_A_AT | cyclin D-type binding-protein 1 | [CCNDBP1](http://www.gene.ucl.ac.uk/cgi-bin/nomenclature/searchgenes.pl?field=symbol&anchor=equals&symbol_search=Search&number=100&format=html&sortby=symbol&match=CCNDBP1) | insulin-like growth factor binding;protein binding;integrin binding;heparin binding | [607089](http://www.ncbi.nlm.nih.gov/entrez/dispomim.cgi?id=607089) | **-1.33** |
| 1420757_AT | myogenic factor 5 | [MYF5](http://www.gene.ucl.ac.uk/cgi-bin/nomenclature/searchgenes.pl?field=symbol&anchor=equals&symbol_search=Search&number=100&format=html&sortby=symbol&match=MYF5) | insulin-like growth factor binding;serine-type endopeptidase inhibitor activity | [159990](http://www.ncbi.nlm.nih.gov/entrez/dispomim.cgi?id=159990) | **-3.56** |
| 1420760_S_AT | N-myc downstream regulated-like | [NDRL](http://www.gene.ucl.ac.uk/cgi-bin/nomenclature/searchgenes.pl?field=symbol&anchor=equals&symbol_search=Search&number=100&format=html&sortby=symbol&match=NDRL) | insulin-like growth factor binding;serine-type endopeptidase inhibitor activity |  | **1.34** |
| 1420762_A_AT | Y box protein 2 | [YBX2](http://www.gene.ucl.ac.uk/cgi-bin/nomenclature/searchgenes.pl?field=symbol&anchor=equals&symbol_search=Search&number=100&format=html&sortby=symbol&match=YBX2) | insulin-like growth factor binding;transporter activity;receptor activity |  | **2.41** |
| 1420776_A_AT | AU RNA binding protein/enoyl-coenzyme A hydratase | [AUH](http://www.gene.ucl.ac.uk/cgi-bin/nomenclature/searchgenes.pl?field=symbol&anchor=equals&symbol_search=Search&number=100&format=html&sortby=symbol&match=AUH) | insulin-like growth factor binding;transporter activity;receptor activity | [600529](http://www.ncbi.nlm.nih.gov/entrez/dispomim.cgi?id=600529) | **1.51** |
| 1420804_S_AT | C-type (calcium dependent, carbohydrate recognition domain) lectin, superfamily member 8 | [CLECSF8](http://www.gene.ucl.ac.uk/cgi-bin/nomenclature/searchgenes.pl?field=symbol&anchor=equals&symbol_search=Search&number=100&format=html&sortby=symbol&match=CLECSF8) | insulysin activity;metallopeptidase activity;metalloendopeptidase activity;hydrolase activity |  | **-4.32** |
| 1420808_AT | nuclear receptor coactivator 4 | [NCOA4](http://www.gene.ucl.ac.uk/cgi-bin/nomenclature/searchgenes.pl?field=symbol&anchor=equals&symbol_search=Search&number=100&format=html&sortby=symbol&match=NCOA4) | interleukin receptor activity;hematopoietin/interferon-class (D200-domain) cytokine receptor activity;receptor activity | [601984](http://www.ncbi.nlm.nih.gov/entrez/dispomim.cgi?id=601984) | **1.38** |
| 1420812_AT | histone deacetylase 7A | [HDAC7A](http://www.gene.ucl.ac.uk/cgi-bin/nomenclature/searchgenes.pl?field=symbol&anchor=equals&symbol_search=Search&number=100&format=html&sortby=symbol&match=HDAC7A) | interleukin receptor activity;receptor activity | [606542](http://www.ncbi.nlm.nih.gov/entrez/dispomim.cgi?id=606542) | **1.48** |
| 1420813_AT | histone deacetylase 7A | [HDAC7A](http://www.gene.ucl.ac.uk/cgi-bin/nomenclature/searchgenes.pl?field=symbol&anchor=equals&symbol_search=Search&number=100&format=html&sortby=symbol&match=HDAC7A) | interleukin receptor activity;receptor activity;hematopoietin/interferon-class (D200-domain) cytokine receptor activity | [606542](http://www.ncbi.nlm.nih.gov/entrez/dispomim.cgi?id=606542) | **1.34** |
| 1420826_AT | leucine zipper-EF-hand containing transmembrane protein 1 | [LETM1](http://www.gene.ucl.ac.uk/cgi-bin/nomenclature/searchgenes.pl?field=symbol&anchor=equals&symbol_search=Search&number=100&format=html&sortby=symbol&match=LETM1) | interleukin receptor activity;receptor activity;hematopoietin/interferon-class (D200-domain) cytokine receptor activity | [604407](http://www.ncbi.nlm.nih.gov/entrez/dispomim.cgi?id=604407) | **1.25** |
| 1420827_A_AT | gb:BG085921 /DB_XREF=gi:12568485 /DB_XREF=H3119F08 |  | interleukin receptor activity;receptor activity;hematopoietin/interferon-class (D200-domain) cytokine receptor activity |  | **1.31** |
| 1420828_S_AT | tyrosine 3-monooxygenase/tryptophan 5-monooxygenase activation protein, theta polypeptide | [YWHAQ](http://www.gene.ucl.ac.uk/cgi-bin/nomenclature/searchgenes.pl?field=symbol&anchor=equals&symbol_search=Search&number=100&format=html&sortby=symbol&match=YWHAQ) | interleukin receptor activity;receptor activity;hematopoietin/interferon-class (D200-domain) cytokine receptor activity | [609009](http://www.ncbi.nlm.nih.gov/entrez/dispomim.cgi?id=609009) | **-1.24** |
| 1420830_X_AT | gb:AW536266 /DB_XREF=gi:7178683 /DB_XREF=G0102D11- |  | interleukin-1 receptor binding;receptor activity;cytokine activity;interleukin-1 receptor antagonist activity |  | **-1.25** |
| 1420858_AT | protein kinase inhibitor, alpha | [PKIA](http://www.gene.ucl.ac.uk/cgi-bin/nomenclature/searchgenes.pl?field=symbol&anchor=equals&symbol_search=Search&number=100&format=html&sortby=symbol&match=PKIA) | ion channel activity | [606059](http://www.ncbi.nlm.nih.gov/entrez/dispomim.cgi?id=606059) | **1.58** |
| 1420872_AT | guanylate cyclase 1, soluble, beta 3 | [GUCY1B3](http://www.gene.ucl.ac.uk/cgi-bin/nomenclature/searchgenes.pl?field=symbol&anchor=equals&symbol_search=Search&number=100&format=html&sortby=symbol&match=GUCY1B3) | ion channel activity | [139397](http://www.ncbi.nlm.nih.gov/entrez/dispomim.cgi?id=139397) | **1.35** |
| 1420889_AT | holocytochrome c synthetase | [HCCS](http://www.gene.ucl.ac.uk/cgi-bin/nomenclature/searchgenes.pl?field=symbol&anchor=equals&symbol_search=Search&number=100&format=html&sortby=symbol&match=HCCS) | ion channel activity;receptor activity;ATP binding;ATP-gated cation channel activity | [300056](http://www.ncbi.nlm.nih.gov/entrez/dispomim.cgi?id=300056) | **1.33** |
| 1420890_AT | holocytochrome c synthetase | [HCCS](http://www.gene.ucl.ac.uk/cgi-bin/nomenclature/searchgenes.pl?field=symbol&anchor=equals&symbol_search=Search&number=100&format=html&sortby=symbol&match=HCCS) | ion channel activity;receptor activity;ATP binding;ATP-gated cation channel activity | [300056](http://www.ncbi.nlm.nih.gov/entrez/dispomim.cgi?id=300056) | **1.22** |
| 1420895_AT | transforming growth factor, beta receptor I | [TGFBR1](http://www.gene.ucl.ac.uk/cgi-bin/nomenclature/searchgenes.pl?field=symbol&anchor=equals&symbol_search=Search&number=100&format=html&sortby=symbol&match=TGFBR1) | ion channel activity;voltage-gated ion channel activity;oxidoreductase activity | [190181](http://www.ncbi.nlm.nih.gov/entrez/dispomim.cgi?id=190181) | **-1.81** |
| 1420901_A_AT | hexokinase 1 | [HK1](http://www.gene.ucl.ac.uk/cgi-bin/nomenclature/searchgenes.pl?field=symbol&anchor=equals&symbol_search=Search&number=100&format=html&sortby=symbol&match=HK1) | ion channel activity;voltage-gated ion channel activity;oxidoreductase activity | [142600](http://www.ncbi.nlm.nih.gov/entrez/dispomim.cgi?id=142600) | **-1.94** |
| 1420903_AT | sialyltransferase 7 ((alpha-N-acetylneuraminyl 2,3-betagalactosyl-1,3)-N-acetyl galactosaminide alpha-2,6-sialyltransferase) C | [SIAT7C](http://www.gene.ucl.ac.uk/cgi-bin/nomenclature/searchgenes.pl?field=symbol&anchor=equals&symbol_search=Search&number=100&format=html&sortby=symbol&match=SIAT7C) | iron ion binding;aconitate hydratase activity;hydro-lyase activity;lyase activity |  | **1.92** |
| 1420904_AT | interleukin 17 receptor | [IL17R](http://www.gene.ucl.ac.uk/cgi-bin/nomenclature/searchgenes.pl?field=symbol&anchor=equals&symbol_search=Search&number=100&format=html&sortby=symbol&match=IL17R) | iron ion binding;aconitate hydratase activity;lyase activity;hydro-lyase activity | [605461](http://www.ncbi.nlm.nih.gov/entrez/dispomim.cgi?id=605461) | **-1.86** |
| 1420909_AT | vascular endothelial growth factor A | [VEGFA](http://www.gene.ucl.ac.uk/cgi-bin/nomenclature/searchgenes.pl?field=symbol&anchor=equals&symbol_search=Search&number=100&format=html&sortby=symbol&match=VEGFA) | iron ion binding;electron transporter activity;electron carrier activity;oxidoreductase activity;succinate dehydrogenase (ubiquinone) activity |  | **1.43** |
| 1420940_X_AT | regulator of G-protein signaling 5 | [RGS5](http://www.gene.ucl.ac.uk/cgi-bin/nomenclature/searchgenes.pl?field=symbol&anchor=equals&symbol_search=Search&number=100&format=html&sortby=symbol&match=RGS5) | iron ion binding;oxidoreductase activity;methanol dehydrogenase activity | [603276](http://www.ncbi.nlm.nih.gov/entrez/dispomim.cgi?id=603276) | **1.94** |
| 1420941_AT | regulator of G-protein signaling 5 | [RGS5](http://www.gene.ucl.ac.uk/cgi-bin/nomenclature/searchgenes.pl?field=symbol&anchor=equals&symbol_search=Search&number=100&format=html&sortby=symbol&match=RGS5) | iron ion binding;oxidoreductase activity;methanol dehydrogenase activity | [603276](http://www.ncbi.nlm.nih.gov/entrez/dispomim.cgi?id=603276) | **2.07** |
| 1420942_S_AT | gb:BF585144 /DB_XREF=gi:11658862 /DB_XREF=60210188 |  | iron ion binding;stearoyl-CoA 9-desaturase activity;oxidoreductase activity |  | **2.75** |
| 1420950_AT | zinc and ring finger 1 | [ZNRF1](http://www.gene.ucl.ac.uk/cgi-bin/nomenclature/searchgenes.pl?field=symbol&anchor=equals&symbol_search=Search&number=100&format=html&sortby=symbol&match=ZNRF1) | iron ion binding;stearoyl-CoA 9-desaturase activity;oxidoreductase activity |  | **1.44** |
| 1420954_A_AT | adducin 1 (alpha) | [ADD1](http://www.gene.ucl.ac.uk/cgi-bin/nomenclature/searchgenes.pl?field=symbol&anchor=equals&symbol_search=Search&number=100&format=html&sortby=symbol&match=ADD1) | iron ion binding;transmembrane receptor protein tyrosine kinase activity;electron transporter activity;ATP binding;heparin binding | [102680](http://www.ncbi.nlm.nih.gov/entrez/dispomim.cgi?id=102680) | **1.26** |
| 1420965_A_AT | ectodermal-neural cortex 1 | [ENC1](http://www.gene.ucl.ac.uk/cgi-bin/nomenclature/searchgenes.pl?field=symbol&anchor=equals&symbol_search=Search&number=100&format=html&sortby=symbol&match=ENC1) | isocitrate dehydrogenase (NADP+) activity;oxidoreductase activity | [605173](http://www.ncbi.nlm.nih.gov/entrez/dispomim.cgi?id=605173) | **-1.66** |
| 1420991_AT | ankyrin repeat domain 1 (cardiac muscle) | [ANKRD1](http://www.gene.ucl.ac.uk/cgi-bin/nomenclature/searchgenes.pl?field=symbol&anchor=equals&symbol_search=Search&number=100&format=html&sortby=symbol&match=ANKRD1) | isoleucine-tRNA ligase activity;ATP binding;tRNA ligase activity |  | **-24.35** |
| 1420992_AT | ankyrin repeat domain 1 (cardiac muscle) | [ANKRD1](http://www.gene.ucl.ac.uk/cgi-bin/nomenclature/searchgenes.pl?field=symbol&anchor=equals&symbol_search=Search&number=100&format=html&sortby=symbol&match=ANKRD1) | isomerase activity |  | **-22.42** |
| 1420994_AT | UDP-GlcNAc:betaGal beta-1,3-N-acetylglucosaminyltransferase 5 | [B3GNT5](http://www.gene.ucl.ac.uk/cgi-bin/nomenclature/searchgenes.pl?field=symbol&anchor=equals&symbol_search=Search&number=100&format=html&sortby=symbol&match=B3GNT5) | isomerase activity |  | **-2.75** |
| 1421022_X_AT | acylphosphatase 1, erythrocyte (common) type | [ACYP1](http://www.gene.ucl.ac.uk/cgi-bin/nomenclature/searchgenes.pl?field=symbol&anchor=equals&symbol_search=Search&number=100&format=html&sortby=symbol&match=ACYP1) | isomerase activity;3-hydroxyacyl-CoA dehydrogenase activity;oxidoreductase activity;lyase activity;catalytic activity;dodecenoyl-CoA delta-isomerase activity | [600875](http://www.ncbi.nlm.nih.gov/entrez/dispomim.cgi?id=600875) | **1.39** |
| 1421026_AT | guanine nucleotide binding protein, alpha 12 | [GNA12](http://www.gene.ucl.ac.uk/cgi-bin/nomenclature/searchgenes.pl?field=symbol&anchor=equals&symbol_search=Search&number=100&format=html&sortby=symbol&match=GNA12) | isomerase activity;acyl-CoA binding;catalytic activity;dodecenoyl-CoA delta-isomerase activity | [604394](http://www.ncbi.nlm.nih.gov/entrez/dispomim.cgi?id=604394) | **1.52** |
| 1421027_A_AT | myocyte enhancer factor 2C | [MEF2C](http://www.gene.ucl.ac.uk/cgi-bin/nomenclature/searchgenes.pl?field=symbol&anchor=equals&symbol_search=Search&number=100&format=html&sortby=symbol&match=MEF2C) | isomerase activity;calcium ion binding;electron transporter activity;protein disulfide isomerase activity;calcium ion storage activity | [600662](http://www.ncbi.nlm.nih.gov/entrez/dispomim.cgi?id=600662) | **-1.27** |
| 1421028_A_AT | myocyte enhancer factor 2C | [MEF2C](http://www.gene.ucl.ac.uk/cgi-bin/nomenclature/searchgenes.pl?field=symbol&anchor=equals&symbol_search=Search&number=100&format=html&sortby=symbol&match=MEF2C) | isomerase activity;calcium ion binding;peptidyl-prolyl cis-trans isomerase activity | [600662](http://www.ncbi.nlm.nih.gov/entrez/dispomim.cgi?id=600662) | **-1.22** |
| 1421042_AT | rho/rac guanine nucleotide exchange factor (GEF) 2 | [ARHGEF2](http://www.gene.ucl.ac.uk/cgi-bin/nomenclature/searchgenes.pl?field=symbol&anchor=equals&symbol_search=Search&number=100&format=html&sortby=symbol&match=ARHGEF2) | isomerase activity;calcium ion binding;peptidyl-prolyl cis-trans isomerase activity | [607560](http://www.ncbi.nlm.nih.gov/entrez/dispomim.cgi?id=607560) | **-1.4** |
| 1421043_S_AT | rho/rac guanine nucleotide exchange factor (GEF) 2 | [ARHGEF2](http://www.gene.ucl.ac.uk/cgi-bin/nomenclature/searchgenes.pl?field=symbol&anchor=equals&symbol_search=Search&number=100&format=html&sortby=symbol&match=ARHGEF2) | isomerase activity;calcium ion binding;peptidyl-prolyl cis-trans isomerase activity | [607560](http://www.ncbi.nlm.nih.gov/entrez/dispomim.cgi?id=607560) | **-1.35** |
| 1421045_AT | mannose receptor, C type 2 | [MRC2](http://www.gene.ucl.ac.uk/cgi-bin/nomenclature/searchgenes.pl?field=symbol&anchor=equals&symbol_search=Search&number=100&format=html&sortby=symbol&match=MRC2) | isomerase activity;calcium ion binding;peptidyl-prolyl cis-trans isomerase activity |  | **-1.82** |
| 1421046_A_AT | poly A binding protein, cytoplasmic 4 | [PABPC4](http://www.gene.ucl.ac.uk/cgi-bin/nomenclature/searchgenes.pl?field=symbol&anchor=equals&symbol_search=Search&number=100&format=html&sortby=symbol&match=PABPC4) | isomerase activity;catalytic activity | [603407](http://www.ncbi.nlm.nih.gov/entrez/dispomim.cgi?id=603407) | **1.23** |
| 1421052_A_AT | spermine synthase | [SMS](http://www.gene.ucl.ac.uk/cgi-bin/nomenclature/searchgenes.pl?field=symbol&anchor=equals&symbol_search=Search&number=100&format=html&sortby=symbol&match=SMS) | isomerase activity;catalytic activity | [300105](http://www.ncbi.nlm.nih.gov/entrez/dispomim.cgi?id=300105) | **-1.48** |
| 1421062_S_AT | clathrin, light polypeptide (Lca) | [CLTA](http://www.gene.ucl.ac.uk/cgi-bin/nomenclature/searchgenes.pl?field=symbol&anchor=equals&symbol_search=Search&number=100&format=html&sortby=symbol&match=CLTA) | isomerase activity;catalytic activity;dodecenoyl-CoA delta-isomerase activity | [118960](http://www.ncbi.nlm.nih.gov/entrez/dispomim.cgi?id=118960) | **-1.4** |
| 1421063_S_AT | SNRPN upstream reading frame | [SNRPN](http://www.gene.ucl.ac.uk/cgi-bin/nomenclature/searchgenes.pl?field=symbol&anchor=equals&symbol_search=Search&number=100&format=html&sortby=symbol&match=SNRPN) | isomerase activity;cobalt ion binding;methylmalonyl-CoA mutase activity | [182279](http://www.ncbi.nlm.nih.gov/entrez/dispomim.cgi?id=182279) | **1.77** |
| 1421097_AT | endonuclease G | [ENDOG](http://www.gene.ucl.ac.uk/cgi-bin/nomenclature/searchgenes.pl?field=symbol&anchor=equals&symbol_search=Search&number=100&format=html&sortby=symbol&match=ENDOG) | isomerase activity;intramolecular transferase activity, phosphotransferases;magnesium ion binding;phosphoglucomutase activity | [600440](http://www.ncbi.nlm.nih.gov/entrez/dispomim.cgi?id=600440) | **1.32** |
| 1421118_A_AT | G protein-coupled receptor 56 | [GPR56](http://www.gene.ucl.ac.uk/cgi-bin/nomenclature/searchgenes.pl?field=symbol&anchor=equals&symbol_search=Search&number=100&format=html&sortby=symbol&match=GPR56) | isomerase activity;peptidyl-prolyl cis-trans isomerase activity | [604110](http://www.ncbi.nlm.nih.gov/entrez/dispomim.cgi?id=604110) | **2.03** |
| 1421144_AT | retinitis pigmentosa GTPase regulator interacting protein 1 | [RPGRIP1](http://www.gene.ucl.ac.uk/cgi-bin/nomenclature/searchgenes.pl?field=symbol&anchor=equals&symbol_search=Search&number=100&format=html&sortby=symbol&match=RPGRIP1) | isomerase activity;peptidyl-prolyl cis-trans isomerase activity | [605446](http://www.ncbi.nlm.nih.gov/entrez/dispomim.cgi?id=605446) | **-1.33** |
| 1421146_AT | Rap guanine nucleotide exchange factor (GEF) 1 | [RAPGEF1](http://www.gene.ucl.ac.uk/cgi-bin/nomenclature/searchgenes.pl?field=symbol&anchor=equals&symbol_search=Search&number=100&format=html&sortby=symbol&match=RAPGEF1) | isomerase activity;peptidyl-prolyl cis-trans isomerase activity | [600303](http://www.ncbi.nlm.nih.gov/entrez/dispomim.cgi?id=600303) | **1.72** |
| 1421186_AT | chemokine (C-C motif) receptor 2 | [CCR2](http://www.gene.ucl.ac.uk/cgi-bin/nomenclature/searchgenes.pl?field=symbol&anchor=equals&symbol_search=Search&number=100&format=html&sortby=symbol&match=CCR2) | isomerase activity;peptidyl-prolyl cis-trans isomerase activity | [601267](http://www.ncbi.nlm.nih.gov/entrez/dispomim.cgi?id=601267) | **-3.09** |
| 1421187_AT | chemokine (C-C motif) receptor 2 | [CCR2](http://www.gene.ucl.ac.uk/cgi-bin/nomenclature/searchgenes.pl?field=symbol&anchor=equals&symbol_search=Search&number=100&format=html&sortby=symbol&match=CCR2) | isomerase activity;peptidyl-prolyl cis-trans isomerase activity | [601267](http://www.ncbi.nlm.nih.gov/entrez/dispomim.cgi?id=601267) | **-2.06** |
| 1421203_AT | cholinergic receptor, nicotinic, alpha polypeptide 4 | [CHRNA4](http://www.gene.ucl.ac.uk/cgi-bin/nomenclature/searchgenes.pl?field=symbol&anchor=equals&symbol_search=Search&number=100&format=html&sortby=symbol&match=CHRNA4) | isomerase activity;peptidyl-prolyl cis-trans isomerase activity | [118504](http://www.ncbi.nlm.nih.gov/entrez/dispomim.cgi?id=118504) | **1.87** |
| 1421215_A_AT | sarcolemma associated protein | [SLMAP](http://www.gene.ucl.ac.uk/cgi-bin/nomenclature/searchgenes.pl?field=symbol&anchor=equals&symbol_search=Search&number=100&format=html&sortby=symbol&match=SLMAP) | isomerase activity;peptidyl-prolyl cis-trans isomerase activity | [602701](http://www.ncbi.nlm.nih.gov/entrez/dispomim.cgi?id=602701) | **1.38** |
| 1421223_A_AT | annexin A4 | [ANXA4](http://www.gene.ucl.ac.uk/cgi-bin/nomenclature/searchgenes.pl?field=symbol&anchor=equals&symbol_search=Search&number=100&format=html&sortby=symbol&match=ANXA4) | isomerase activity;peptidyl-prolyl cis-trans isomerase activity | [106491](http://www.ncbi.nlm.nih.gov/entrez/dispomim.cgi?id=106491) | **-1.62** |
| 1421253_AT | nebulin-related anchoring protein | [NRAP](http://www.gene.ucl.ac.uk/cgi-bin/nomenclature/searchgenes.pl?field=symbol&anchor=equals&symbol_search=Search&number=100&format=html&sortby=symbol&match=NRAP) | isomerase activity;procollagen-proline 4-dioxygenase activity;electron transporter activity;protein disulfide isomerase activity | [602873](http://www.ncbi.nlm.nih.gov/entrez/dispomim.cgi?id=602873) | **1.47** |
| 1421265_A_AT | RNA-binding region (RNP1, RRM) containing 1 | [RNPC1](http://www.gene.ucl.ac.uk/cgi-bin/nomenclature/searchgenes.pl?field=symbol&anchor=equals&symbol_search=Search&number=100&format=html&sortby=symbol&match=RNPC1) | isomerase activity;protein disulfide isomerase activity;electron transporter activity;peptidase activity |  | **1.58** |
| 1421269_AT | UDP-glucose ceramide glucosyltransferase | [UGCG](http://www.gene.ucl.ac.uk/cgi-bin/nomenclature/searchgenes.pl?field=symbol&anchor=equals&symbol_search=Search&number=100&format=html&sortby=symbol&match=UGCG) | isovaleryl-CoA dehydrogenase activity;oxidoreductase activity;acyl-CoA dehydrogenase activity | [602874](http://www.ncbi.nlm.nih.gov/entrez/dispomim.cgi?id=602874) | **-1.87** |
| 1421276_A_AT | dystonin | [DST](http://www.gene.ucl.ac.uk/cgi-bin/nomenclature/searchgenes.pl?field=symbol&anchor=equals&symbol_search=Search&number=100&format=html&sortby=symbol&match=DST) | isovaleryl-CoA dehydrogenase activity;oxidoreductase activity;acyl-CoA dehydrogenase activity |  | **1.27** |
| 1421287_A_AT | platelet/endothelial cell adhesion molecule 1 | [PECAM1](http://www.gene.ucl.ac.uk/cgi-bin/nomenclature/searchgenes.pl?field=symbol&anchor=equals&symbol_search=Search&number=100&format=html&sortby=symbol&match=PECAM1) | KDEL sequence binding;receptor activity;protein transporter activity | [173445](http://www.ncbi.nlm.nih.gov/entrez/dispomim.cgi?id=173445) | **1.6** |
| 1421321_A_AT | neuroepithelial cell transforming gene 1 | [NET1](http://www.gene.ucl.ac.uk/cgi-bin/nomenclature/searchgenes.pl?field=symbol&anchor=equals&symbol_search=Search&number=100&format=html&sortby=symbol&match=NET1) | KDEL sequence binding;receptor activity;protein transporter activity | [606450](http://www.ncbi.nlm.nih.gov/entrez/dispomim.cgi?id=606450) | **-1.36** |
| 1421326_AT | colony stimulating factor 2 receptor, beta 1, low-affinity (granulocyte-macrophage) | [CSF2RB1](http://www.gene.ucl.ac.uk/cgi-bin/nomenclature/searchgenes.pl?field=symbol&anchor=equals&symbol_search=Search&number=100&format=html&sortby=symbol&match=CSF2RB1) | kinase activity |  | **-3.71** |
| 1421365_AT | follistatin | [FST](http://www.gene.ucl.ac.uk/cgi-bin/nomenclature/searchgenes.pl?field=symbol&anchor=equals&symbol_search=Search&number=100&format=html&sortby=symbol&match=FST) | kinase activity | [136470](http://www.ncbi.nlm.nih.gov/entrez/dispomim.cgi?id=136470) | **-3.52** |
| 1421375_A_AT | S100 calcium binding protein A6 (calcyclin) | [S100A6](http://www.gene.ucl.ac.uk/cgi-bin/nomenclature/searchgenes.pl?field=symbol&anchor=equals&symbol_search=Search&number=100&format=html&sortby=symbol&match=S100A6) | kinase activity | [114110](http://www.ncbi.nlm.nih.gov/entrez/dispomim.cgi?id=114110) | **-1.67** |
| 1421408_AT | immunoglobulin superfamily, member 6 | [IGSF6](http://www.gene.ucl.ac.uk/cgi-bin/nomenclature/searchgenes.pl?field=symbol&anchor=equals&symbol_search=Search&number=100&format=html&sortby=symbol&match=IGSF6) | kinase activity | [606222](http://www.ncbi.nlm.nih.gov/entrez/dispomim.cgi?id=606222) | **-13.17** |
| 1421424_A_AT | alanyl (membrane) aminopeptidase | [ANPEP](http://www.gene.ucl.ac.uk/cgi-bin/nomenclature/searchgenes.pl?field=symbol&anchor=equals&symbol_search=Search&number=100&format=html&sortby=symbol&match=ANPEP) | kinase activity | [151530](http://www.ncbi.nlm.nih.gov/entrez/dispomim.cgi?id=151530) | **-2.11** |
| 1421425_A_AT | Down syndrome critical region gene 1-like 1 | [DSCR1L1](http://www.gene.ucl.ac.uk/cgi-bin/nomenclature/searchgenes.pl?field=symbol&anchor=equals&symbol_search=Search&number=100&format=html&sortby=symbol&match=DSCR1L1) | kinase activity;[pyruvate dehydrogenase (lipoamide)] kinase activity;ATP binding;transferase activity | [604876](http://www.ncbi.nlm.nih.gov/entrez/dispomim.cgi?id=604876) | **1.45** |
| 1421431_AT | polymerase I and transcript release factor | [PTRF](http://www.gene.ucl.ac.uk/cgi-bin/nomenclature/searchgenes.pl?field=symbol&anchor=equals&symbol_search=Search&number=100&format=html&sortby=symbol&match=PTRF) | kinase activity;[pyruvate dehydrogenase (lipoamide)] kinase activity;protein-histidine kinase activity;ATP binding;transferase activity | [603198](http://www.ncbi.nlm.nih.gov/entrez/dispomim.cgi?id=603198) | **1.62** |
| 1421448_AT | GTPase activating RANGAP domain-like 1 | [GARNL1](http://www.gene.ucl.ac.uk/cgi-bin/nomenclature/searchgenes.pl?field=symbol&anchor=equals&symbol_search=Search&number=100&format=html&sortby=symbol&match=GARNL1) | kinase activity;1-phosphatidylinositol-4-phosphate 5-kinase activity;1-phosphatidylinositol-5-phosphate 4-kinase activity;transferase activity | [608884](http://www.ncbi.nlm.nih.gov/entrez/dispomim.cgi?id=608884) | **1.41** |
| 1421466_AT | ankyrin repeat and SOCS box-containing protein 10 | [ASB10](http://www.gene.ucl.ac.uk/cgi-bin/nomenclature/searchgenes.pl?field=symbol&anchor=equals&symbol_search=Search&number=100&format=html&sortby=symbol&match=ASB10) | kinase activity;1-phosphatidylinositol-4-phosphate 5-kinase activity;transferase activity;1-phosphatidylinositol-5-phosphate 4-kinase activity |  | **1.64** |
| 1421491_A_AT | RIKEN cDNA 4930579A11 gene | [4930579A11RIK](http://www.gene.ucl.ac.uk/cgi-bin/nomenclature/searchgenes.pl?field=symbol&anchor=equals&symbol_search=Search&number=100&format=html&sortby=symbol&match=4930579A11RIK) | kinase activity;1-phosphatidylinositol-4-phosphate 5-kinase activity;transferase activity;1-phosphatidylinositol-5-phosphate 4-kinase activity |  | **1.33** |
| 1421525_A_AT | baculoviral IAP repeat-containing 1e | [BIRC1E](http://www.gene.ucl.ac.uk/cgi-bin/nomenclature/searchgenes.pl?field=symbol&anchor=equals&symbol_search=Search&number=100&format=html&sortby=symbol&match=BIRC1E) | kinase activity;adenylate kinase activity;ATP binding;phosphotransferase activity, phosphate group as acceptor;transferase activity |  | **-3.84** |
| 1421535_A_AT | phosphodiesterase 4A, cAMP specific | [PDE4A](http://www.gene.ucl.ac.uk/cgi-bin/nomenclature/searchgenes.pl?field=symbol&anchor=equals&symbol_search=Search&number=100&format=html&sortby=symbol&match=PDE4A) | kinase activity;adenylate kinase activity;ATP binding;transferase activity | [600126](http://www.ncbi.nlm.nih.gov/entrez/dispomim.cgi?id=600126) | **1.48** |
| 1421557_X_AT | thioredoxin 2 | [TXN2](http://www.gene.ucl.ac.uk/cgi-bin/nomenclature/searchgenes.pl?field=symbol&anchor=equals&symbol_search=Search&number=100&format=html&sortby=symbol&match=TXN2) | kinase activity;adenylate kinase activity;GTP binding;shikimate kinase activity;ATP binding;phosphotransferase activity, phosphate group as acceptor;transferase activity | [609063](http://www.ncbi.nlm.nih.gov/entrez/dispomim.cgi?id=609063) | **1.27** |
| 1421571_A_AT | lymphocyte antigen 6 complex, locus C | [LY6C](http://www.gene.ucl.ac.uk/cgi-bin/nomenclature/searchgenes.pl?field=symbol&anchor=equals&symbol_search=Search&number=100&format=html&sortby=symbol&match=LY6C) | kinase activity;adenylate kinase activity;shikimate kinase activity;GTP binding;ATP binding;phosphotransferase activity, phosphate group as acceptor;transferase activity |  | **1.52** |
| 1421594_A_AT | synaptotagmin-like 2 | [SYTL2](http://www.gene.ucl.ac.uk/cgi-bin/nomenclature/searchgenes.pl?field=symbol&anchor=equals&symbol_search=Search&number=100&format=html&sortby=symbol&match=SYTL2) | kinase activity;adenylate kinase activity;shikimate kinase activity;GTP binding;phosphotransferase activity, phosphate group as acceptor;ATP binding;transferase activity |  | **-2.07** |
| 1421604_A_AT | Kruppel-like factor 3 (basic) | [KLF3](http://www.gene.ucl.ac.uk/cgi-bin/nomenclature/searchgenes.pl?field=symbol&anchor=equals&symbol_search=Search&number=100&format=html&sortby=symbol&match=KLF3) | kinase activity;calcium ion binding;guanyl-nucleotide exchange factor activity;diacylglycerol binding |  | **1.3** |
| 1421624_A_AT | enabled homolog (Drosophila) | [ENAH](http://www.gene.ucl.ac.uk/cgi-bin/nomenclature/searchgenes.pl?field=symbol&anchor=equals&symbol_search=Search&number=100&format=html&sortby=symbol&match=ENAH) | kinase activity;cGMP-dependent protein kinase activity;protein serine/threonine kinase activity;ATP binding;transferase activity;3',5'-cGMP binding;cAMP-dependent protein kinase regulator activity;protein binding;protein kinase activity |  | **-2.16** |
| 1421654_A_AT | lamin A | [LMNA](http://www.gene.ucl.ac.uk/cgi-bin/nomenclature/searchgenes.pl?field=symbol&anchor=equals&symbol_search=Search&number=100&format=html&sortby=symbol&match=LMNA) | kinase activity;creatine kinase activity;transferase activity, transferring phosphorus-containing groups;transferase activity | [150330](http://www.ncbi.nlm.nih.gov/entrez/dispomim.cgi?id=150330) | **-1.89** |
| 1421657_A_AT | SRY-box containing gene 17 | [SOX17](http://www.gene.ucl.ac.uk/cgi-bin/nomenclature/searchgenes.pl?field=symbol&anchor=equals&symbol_search=Search&number=100&format=html&sortby=symbol&match=SOX17) | kinase activity;creatine kinase activity;transferase activity, transferring phosphorus-containing groups;transferase activity |  | **2.14** |
| 1421662_A_AT | tumor suppressor candidate 3 | [TUSC3](http://www.gene.ucl.ac.uk/cgi-bin/nomenclature/searchgenes.pl?field=symbol&anchor=equals&symbol_search=Search&number=100&format=html&sortby=symbol&match=TUSC3) | kinase activity;cyclin-dependent protein kinase activity | [601385](http://www.ncbi.nlm.nih.gov/entrez/dispomim.cgi?id=601385) | **-1.53** |
| 1421679_A_AT | cyclin-dependent kinase inhibitor 1A (P21) | [CDKN1A](http://www.gene.ucl.ac.uk/cgi-bin/nomenclature/searchgenes.pl?field=symbol&anchor=equals&symbol_search=Search&number=100&format=html&sortby=symbol&match=CDKN1A) | kinase activity;cyclin-dependent protein kinase activity | [116899](http://www.ncbi.nlm.nih.gov/entrez/dispomim.cgi?id=116899) | **-3.13** |
| 1421735_A_AT | sialyltransferase 8 (alpha-2, 8-sialyltransferase) E | [SIAT8E](http://www.gene.ucl.ac.uk/cgi-bin/nomenclature/searchgenes.pl?field=symbol&anchor=equals&symbol_search=Search&number=100&format=html&sortby=symbol&match=SIAT8E) | kinase activity;fructose-2,6-bisphosphate 2-phosphatase activity;ATP binding;catalytic activity;6-phosphofructo-2-kinase activity;hydrolase activity;transferase activity | [607162](http://www.ncbi.nlm.nih.gov/entrez/dispomim.cgi?id=607162) | **1.82** |
| 1421751_A_AT | proteasome (prosome, macropain) 26S subunit, non-ATPase, 14 | [PSMD14](http://www.gene.ucl.ac.uk/cgi-bin/nomenclature/searchgenes.pl?field=symbol&anchor=equals&symbol_search=Search&number=100&format=html&sortby=symbol&match=PSMD14) | kinase activity;G-protein coupled photoreceptor activity;ATP binding | [607173](http://www.ncbi.nlm.nih.gov/entrez/dispomim.cgi?id=607173) | **-1.31** |
| 1421756_A_AT | G protein-coupled receptor 19 | [GPR19](http://www.gene.ucl.ac.uk/cgi-bin/nomenclature/searchgenes.pl?field=symbol&anchor=equals&symbol_search=Search&number=100&format=html&sortby=symbol&match=GPR19) | kinase activity;growth factor activity;receptor activity | [602927](http://www.ncbi.nlm.nih.gov/entrez/dispomim.cgi?id=602927) | **1.32** |
| 1421814_AT | moesin | [MSN](http://www.gene.ucl.ac.uk/cgi-bin/nomenclature/searchgenes.pl?field=symbol&anchor=equals&symbol_search=Search&number=100&format=html&sortby=symbol&match=MSN) | kinase activity;guanyl-nucleotide exchange factor activity;calcium ion binding;diacylglycerol binding | [309845](http://www.ncbi.nlm.nih.gov/entrez/dispomim.cgi?id=309845) | **-1.44** |
| 1421815_AT | expressed sequence AU040950 | [AU040950](http://www.gene.ucl.ac.uk/cgi-bin/nomenclature/searchgenes.pl?field=symbol&anchor=equals&symbol_search=Search&number=100&format=html&sortby=symbol&match=AU040950) | kinase activity;inositol-trisphosphate 3-kinase activity |  | **1.5** |
| 1421821_AT | Low density lipoprotein receptor | [LDLR](http://www.gene.ucl.ac.uk/cgi-bin/nomenclature/searchgenes.pl?field=symbol&anchor=equals&symbol_search=Search&number=100&format=html&sortby=symbol&match=LDLR) | kinase activity;inositol-trisphosphate 3-kinase activity | [606945](http://www.ncbi.nlm.nih.gov/entrez/dispomim.cgi?id=606945) | **-1.61** |
| 1421830_AT | gb:NM_009647.1 /DB_XREF=gi:6753021 /GEN=Ak4 /FEA=F |  | kinase activity;inositol-trisphosphate 3-kinase activity |  | **2.72** |
| 1421836_AT | gb:AK004303.1 /DB_XREF=gi:12835438 /FEA=FLmRNA /CN |  | kinase activity;inositol-trisphosphate 3-kinase activity;inositol/phosphatidylinositol kinase activity;transferase activity |  | **1.59** |
| 1421840_AT | ATP-binding cassette, sub-family A (ABC1), member 1 | [ABCA1](http://www.gene.ucl.ac.uk/cgi-bin/nomenclature/searchgenes.pl?field=symbol&anchor=equals&symbol_search=Search&number=100&format=html&sortby=symbol&match=ABCA1) | kinase activity;magnesium ion binding;adenosine kinase activity;transferase activity | [600046](http://www.ncbi.nlm.nih.gov/entrez/dispomim.cgi?id=600046) | **-1.3** |
| 1421848_AT | solute carrier family 22 (organic cation transporter), member 5 | [SLC22A5](http://www.gene.ucl.ac.uk/cgi-bin/nomenclature/searchgenes.pl?field=symbol&anchor=equals&symbol_search=Search&number=100&format=html&sortby=symbol&match=SLC22A5) | kinase activity;magnesium ion binding;adenosine kinase activity;transferase activity | [603377](http://www.ncbi.nlm.nih.gov/entrez/dispomim.cgi?id=603377) | **1.57** |
| 1421851_AT | DEAD/H (Asp-Glu-Ala-Asp/His) box polypeptide 26 | [DDX26](http://www.gene.ucl.ac.uk/cgi-bin/nomenclature/searchgenes.pl?field=symbol&anchor=equals&symbol_search=Search&number=100&format=html&sortby=symbol&match=DDX26) | kinase activity;magnesium ion binding;catalytic activity;ATP binding;6-phosphofructokinase activity;transferase activity | [604331](http://www.ncbi.nlm.nih.gov/entrez/dispomim.cgi?id=604331) | **-2.43** |
| 1421854_AT | fibrinogen-like protein 2 | [FGL2](http://www.gene.ucl.ac.uk/cgi-bin/nomenclature/searchgenes.pl?field=symbol&anchor=equals&symbol_search=Search&number=100&format=html&sortby=symbol&match=FGL2) | kinase activity;magnesium ion binding;phosphofructokinase activity;catalytic activity;ATP binding;6-phosphofructokinase activity;transferase activity | [605351](http://www.ncbi.nlm.nih.gov/entrez/dispomim.cgi?id=605351) | **-1.68** |
| 1421855_AT | fibrinogen-like protein 2 | [FGL2](http://www.gene.ucl.ac.uk/cgi-bin/nomenclature/searchgenes.pl?field=symbol&anchor=equals&symbol_search=Search&number=100&format=html&sortby=symbol&match=FGL2) | kinase activity;magnesium ion binding;riboflavin kinase activity;transferase activity | [605351](http://www.ncbi.nlm.nih.gov/entrez/dispomim.cgi?id=605351) | **-1.6** |
| 1421858_AT | gb:BE980579 /DB_XREF=gi:10648718 /DB_XREF=UI-M-BG2 |  | kinase activity;magnesium ion binding;riboflavin kinase activity;transferase activity |  | **-1.23** |
| 1421859_AT | gb:C76813 /DB_XREF=gi:2517143 /DB_XREF=C76813 /CLO |  | kinase activity;nucleic acid binding;RNA binding |  | **-1.25** |
| 1421861_AT | calsyntenin 1 | [CLSTN1](http://www.gene.ucl.ac.uk/cgi-bin/nomenclature/searchgenes.pl?field=symbol&anchor=equals&symbol_search=Search&number=100&format=html&sortby=symbol&match=CLSTN1) | kinase activity;nucleotidyltransferase activity;transcription factor activity;UTP-glucose-1-phosphate uridylyltransferase activity;transferase activity |  | **1.29** |
| 1421871_AT | SH3-binding domain glutamic acid-rich protein like | [SH3BGRL](http://www.gene.ucl.ac.uk/cgi-bin/nomenclature/searchgenes.pl?field=symbol&anchor=equals&symbol_search=Search&number=100&format=html&sortby=symbol&match=SH3BGRL) | kinase activity;nucleotidyltransferase activity;transcription factor activity;UTP-glucose-1-phosphate uridylyltransferase activity;transferase activity | [300190](http://www.ncbi.nlm.nih.gov/entrez/dispomim.cgi?id=300190) | **-1.44** |
| 1421887_A_AT | amyloid beta (A4) precursor-like protein 2 | [APLP2](http://www.gene.ucl.ac.uk/cgi-bin/nomenclature/searchgenes.pl?field=symbol&anchor=equals&symbol_search=Search&number=100&format=html&sortby=symbol&match=APLP2) | kinase activity;nucleotidyltransferase activity;transcription factor activity;UTP-glucose-1-phosphate uridylyltransferase activity;transferase activity | [104776](http://www.ncbi.nlm.nih.gov/entrez/dispomim.cgi?id=104776) | **1.26** |
| 1421889_A_AT | amyloid beta (A4) precursor-like protein 2 | [APLP2](http://www.gene.ucl.ac.uk/cgi-bin/nomenclature/searchgenes.pl?field=symbol&anchor=equals&symbol_search=Search&number=100&format=html&sortby=symbol&match=APLP2) | kinase activity;nucleotidyltransferase activity;transcription factor activity;UTP-glucose-1-phosphate uridylyltransferase activity;transferase activity | [104776](http://www.ncbi.nlm.nih.gov/entrez/dispomim.cgi?id=104776) | **1.29** |
| 1421917_AT | platelet derived growth factor receptor, alpha polypeptide | [PDGFRA](http://www.gene.ucl.ac.uk/cgi-bin/nomenclature/searchgenes.pl?field=symbol&anchor=equals&symbol_search=Search&number=100&format=html&sortby=symbol&match=PDGFRA) | kinase activity;phosphorylase kinase activity;calmodulin binding;transferase activity | [173490](http://www.ncbi.nlm.nih.gov/entrez/dispomim.cgi?id=173490) | **-1.6** |
| 1421918_AT | acidic (leucine-rich) nuclear phosphoprotein 32 family, member A | [ANP32A](http://www.gene.ucl.ac.uk/cgi-bin/nomenclature/searchgenes.pl?field=symbol&anchor=equals&symbol_search=Search&number=100&format=html&sortby=symbol&match=ANP32A) | kinase activity;phosphorylase kinase activity;calmodulin binding;transferase activity | [600832](http://www.ncbi.nlm.nih.gov/entrez/dispomim.cgi?id=600832) | **1.35** |
| 1421922_AT | gb:BC018237.1 /DB_XREF=gi:17390546 /FEA=FLmRNA /CN |  | kinase activity;phosphotransferase activity, alcohol group as acceptor;ATP binding;galactokinase activity;transferase activity |  | **1.49** |
| 1421962_AT | DnaJ (Hsp40) homolog, subfamily B, member 5 | [DNAJB5](http://www.gene.ucl.ac.uk/cgi-bin/nomenclature/searchgenes.pl?field=symbol&anchor=equals&symbol_search=Search&number=100&format=html&sortby=symbol&match=DNAJB5) | kinase activity;phosphotransferase activity, alcohol group as acceptor;deoxycytidine kinase activity;ATP binding;transferase activity |  | **1.33** |
| 1421964_AT | Notch gene homolog 3 (Drosophila) | [NOTCH3](http://www.gene.ucl.ac.uk/cgi-bin/nomenclature/searchgenes.pl?field=symbol&anchor=equals&symbol_search=Search&number=100&format=html&sortby=symbol&match=NOTCH3) | kinase activity;phosphotransferase activity, alcohol group as acceptor;inositol/phosphatidylinositol kinase activity;transferase activity | [600276](http://www.ncbi.nlm.nih.gov/entrez/dispomim.cgi?id=600276) | **1.59** |
| 1421965_S_AT | Notch gene homolog 3 (Drosophila) | [NOTCH3](http://www.gene.ucl.ac.uk/cgi-bin/nomenclature/searchgenes.pl?field=symbol&anchor=equals&symbol_search=Search&number=100&format=html&sortby=symbol&match=NOTCH3) | kinase activity;protein domain specific binding | [600276](http://www.ncbi.nlm.nih.gov/entrez/dispomim.cgi?id=600276) | **1.36** |
| 1421985_A_AT | eukaryotic translation initiation factor 4E member 2 | [EIF4E2](http://www.gene.ucl.ac.uk/cgi-bin/nomenclature/searchgenes.pl?field=symbol&anchor=equals&symbol_search=Search&number=100&format=html&sortby=symbol&match=EIF4E2) | kinase activity;protein domain specific binding | [605895](http://www.ncbi.nlm.nih.gov/entrez/dispomim.cgi?id=605895) | **-1.41** |
| 1422013_AT | C-type (calcium dependent, carbohydrate recognition domain) lectin, superfamily member 6 | [CLECSF6](http://www.gene.ucl.ac.uk/cgi-bin/nomenclature/searchgenes.pl?field=symbol&anchor=equals&symbol_search=Search&number=100&format=html&sortby=symbol&match=CLECSF6) | kinase activity;protein domain specific binding | [605306](http://www.ncbi.nlm.nih.gov/entrez/dispomim.cgi?id=605306) | **-3.35** |
| 1422015_A_AT | RIKEN cDNA 4833412N02 gene | [ABCB8](http://www.gene.ucl.ac.uk/cgi-bin/nomenclature/searchgenes.pl?field=symbol&anchor=equals&symbol_search=Search&number=100&format=html&sortby=symbol&match=ABCB8) | kinase activity;protein domain specific binding | [605464](http://www.ncbi.nlm.nih.gov/entrez/dispomim.cgi?id=605464) | **1.7** |
| 1422017_S_AT | RIKEN cDNA 4833439L19 gene | [4833439L19RIK](http://www.gene.ucl.ac.uk/cgi-bin/nomenclature/searchgenes.pl?field=symbol&anchor=equals&symbol_search=Search&number=100&format=html&sortby=symbol&match=4833439L19RIK) | kinase activity;protein kinase A binding |  | **1.72** |
| 1422024_AT | Friend leukemia integration 1 | [FLI1](http://www.gene.ucl.ac.uk/cgi-bin/nomenclature/searchgenes.pl?field=symbol&anchor=equals&symbol_search=Search&number=100&format=html&sortby=symbol&match=FLI1) | kinase activity;protein kinase activity;actin binding;magnesium ion binding;protein serine/threonine kinase activity;ATP binding;transferase activity | [193067](http://www.ncbi.nlm.nih.gov/entrez/dispomim.cgi?id=193067) | **1.32** |
| 1422045_A_AT | protein tyrosine phosphatase, non-receptor type 12 | [PTPN12](http://www.gene.ucl.ac.uk/cgi-bin/nomenclature/searchgenes.pl?field=symbol&anchor=equals&symbol_search=Search&number=100&format=html&sortby=symbol&match=PTPN12) | kinase activity;protein kinase activity;ATP binding;transferase activity | [600079](http://www.ncbi.nlm.nih.gov/entrez/dispomim.cgi?id=600079) | **-1.56** |
| 1422046_AT | integrin alpha M | [ITGAM](http://www.gene.ucl.ac.uk/cgi-bin/nomenclature/searchgenes.pl?field=symbol&anchor=equals&symbol_search=Search&number=100&format=html&sortby=symbol&match=ITGAM) | kinase activity;protein kinase activity;ATP binding;transferase activity | [120980](http://www.ncbi.nlm.nih.gov/entrez/dispomim.cgi?id=120980) | **-2.51** |
| 1422047_AT | cadherin 5 | [CDH5](http://www.gene.ucl.ac.uk/cgi-bin/nomenclature/searchgenes.pl?field=symbol&anchor=equals&symbol_search=Search&number=100&format=html&sortby=symbol&match=CDH5) | kinase activity;protein kinase activity;ephrin receptor activity;protein serine/threonine kinase activity;receptor activity;protein-tyrosine kinase activity;ATP binding;transferase activity | [601120](http://www.ncbi.nlm.nih.gov/entrez/dispomim.cgi?id=601120) | **2.65** |
| 1422079_AT | protein kinase C, eta | [PRKCH](http://www.gene.ucl.ac.uk/cgi-bin/nomenclature/searchgenes.pl?field=symbol&anchor=equals&symbol_search=Search&number=100&format=html&sortby=symbol&match=PRKCH) | kinase activity;protein kinase activity;ephrin receptor activity;protein serine/threonine kinase activity;receptor activity;protein-tyrosine kinase activity;ATP binding;transferase activity | [605437](http://www.ncbi.nlm.nih.gov/entrez/dispomim.cgi?id=605437) | **1.92** |
| 1422101_AT | tumor necrosis factor receptor superfamily, member 23 | [TNFRSF23](http://www.gene.ucl.ac.uk/cgi-bin/nomenclature/searchgenes.pl?field=symbol&anchor=equals&symbol_search=Search&number=100&format=html&sortby=symbol&match=TNFRSF23) | kinase activity;protein kinase activity;magnesium ion binding;protein serine/threonine kinase activity;protein-tyrosine kinase activity;ATP binding;transferase activity |  | **-2.87** |
| 1422102_A_AT | signal transducer and activator of transcription 5B | [STAT5B](http://www.gene.ucl.ac.uk/cgi-bin/nomenclature/searchgenes.pl?field=symbol&anchor=equals&symbol_search=Search&number=100&format=html&sortby=symbol&match=STAT5B) | kinase activity;protein kinase activity;protein serine/threonine kinase activity;ATP binding;protein-tyrosine kinase activity;diacylglycerol binding;transferase activity | [604260](http://www.ncbi.nlm.nih.gov/entrez/dispomim.cgi?id=604260) | **1.65** |
| 1422103_A_AT | signal transducer and activator of transcription 5B | [STAT5B](http://www.gene.ucl.ac.uk/cgi-bin/nomenclature/searchgenes.pl?field=symbol&anchor=equals&symbol_search=Search&number=100&format=html&sortby=symbol&match=STAT5B) | kinase activity;protein kinase activity;protein serine/threonine kinase activity;ATP binding;protein-tyrosine kinase activity;protein kinase CK2 activity;transferase activity | [604260](http://www.ncbi.nlm.nih.gov/entrez/dispomim.cgi?id=604260) | **1.28** |
| 1422123_S_AT | CEA-related cell adhesion molecule 2 | [CEACAM1](http://www.gene.ucl.ac.uk/cgi-bin/nomenclature/searchgenes.pl?field=symbol&anchor=equals&symbol_search=Search&number=100&format=html&sortby=symbol&match=CEACAM1) | kinase activity;protein kinase activity;protein serine/threonine kinase activity;ATP binding;protein-tyrosine kinase activity;transferase activity | [109770](http://www.ncbi.nlm.nih.gov/entrez/dispomim.cgi?id=109770) | **1.64** |
| 1422124_A_AT | protein tyrosine phosphatase, receptor type, C | [PTPRC](http://www.gene.ucl.ac.uk/cgi-bin/nomenclature/searchgenes.pl?field=symbol&anchor=equals&symbol_search=Search&number=100&format=html&sortby=symbol&match=PTPRC) | kinase activity;protein kinase activity;protein serine/threonine kinase activity;ATP binding;protein-tyrosine kinase activity;transferase activity | [151460](http://www.ncbi.nlm.nih.gov/entrez/dispomim.cgi?id=151460) | **-3.62** |
| 1422126_A_AT | nudix (nucleoside diphosphate linked moiety X)-type motif 13 | [NUDT13](http://www.gene.ucl.ac.uk/cgi-bin/nomenclature/searchgenes.pl?field=symbol&anchor=equals&symbol_search=Search&number=100&format=html&sortby=symbol&match=NUDT13) | kinase activity;protein kinase activity;protein serine/threonine kinase activity;ATP binding;protein-tyrosine kinase activity;transferase activity |  | **1.28** |
| 1422153_A_AT | ankyrin repeat and SOCS box-containing protein 11 | [ASB11](http://www.gene.ucl.ac.uk/cgi-bin/nomenclature/searchgenes.pl?field=symbol&anchor=equals&symbol_search=Search&number=100&format=html&sortby=symbol&match=ASB11) | kinase activity;protein kinase activity;protein serine/threonine kinase activity;ATP binding;protein-tyrosine kinase activity;transferase activity |  | **1.61** |
| 1422180_A_AT | mast cell protease 6 | [MCPT6](http://www.gene.ucl.ac.uk/cgi-bin/nomenclature/searchgenes.pl?field=symbol&anchor=equals&symbol_search=Search&number=100&format=html&sortby=symbol&match=MCPT6) | kinase activity;protein kinase activity;protein serine/threonine kinase activity;ATP binding;protein-tyrosine kinase activity;transferase activity |  | **1.93** |
| 1422184_A_AT | adenylate kinase 1 | [AK1](http://www.gene.ucl.ac.uk/cgi-bin/nomenclature/searchgenes.pl?field=symbol&anchor=equals&symbol_search=Search&number=100&format=html&sortby=symbol&match=AK1) | kinase activity;protein kinase activity;protein serine/threonine kinase activity;ATP binding;structural constituent of ribosome;transferase activity | [103000](http://www.ncbi.nlm.nih.gov/entrez/dispomim.cgi?id=103000) | **1.22** |
| 1422185_A_AT | diaphorase 1 (NADH) | [DIA1](http://www.gene.ucl.ac.uk/cgi-bin/nomenclature/searchgenes.pl?field=symbol&anchor=equals&symbol_search=Search&number=100&format=html&sortby=symbol&match=DIA1) | kinase activity;protein kinase activity;protein serine/threonine kinase activity;ATP binding;transferase activity | [250800](http://www.ncbi.nlm.nih.gov/entrez/dispomim.cgi?id=250800) | **-1.37** |
| 1422186_S_AT | diaphorase 1 (NADH) | [DIA1](http://www.gene.ucl.ac.uk/cgi-bin/nomenclature/searchgenes.pl?field=symbol&anchor=equals&symbol_search=Search&number=100&format=html&sortby=symbol&match=DIA1) | kinase activity;protein kinase activity;protein serine/threonine kinase activity;ATP binding;transferase activity | [250800](http://www.ncbi.nlm.nih.gov/entrez/dispomim.cgi?id=250800) | **-1.55** |
| 1422190_AT | complement component 5, receptor 1 | [C5R1](http://www.gene.ucl.ac.uk/cgi-bin/nomenclature/searchgenes.pl?field=symbol&anchor=equals&symbol_search=Search&number=100&format=html&sortby=symbol&match=C5R1) | kinase activity;protein kinase activity;protein serine/threonine kinase activity;ATP binding;transferase activity | [113995](http://www.ncbi.nlm.nih.gov/entrez/dispomim.cgi?id=113995) | **-2.49** |
| 1422241_A_AT | NADH dehydrogenase (ubiquinone) 1 alpha subcomplex, 1 | [NDUFA1](http://www.gene.ucl.ac.uk/cgi-bin/nomenclature/searchgenes.pl?field=symbol&anchor=equals&symbol_search=Search&number=100&format=html&sortby=symbol&match=NDUFA1) | kinase activity;protein kinase activity;protein serine/threonine kinase activity;ATP binding;transferase activity | [300078](http://www.ncbi.nlm.nih.gov/entrez/dispomim.cgi?id=300078) | **1.23** |
| 1422302_S_AT | ferritin light chain 1 | [FTL1](http://www.gene.ucl.ac.uk/cgi-bin/nomenclature/searchgenes.pl?field=symbol&anchor=equals&symbol_search=Search&number=100&format=html&sortby=symbol&match=FTL1) | kinase activity;protein kinase activity;protein serine/threonine kinase activity;ATP binding;transferase activity |  | **-1.55** |
| 1422315_X_AT | phosphorylase kinase gamma 1 | [PHKG1](http://www.gene.ucl.ac.uk/cgi-bin/nomenclature/searchgenes.pl?field=symbol&anchor=equals&symbol_search=Search&number=100&format=html&sortby=symbol&match=PHKG1) | kinase activity;protein kinase activity;protein serine/threonine kinase activity;ATP binding;transferase activity | [172470](http://www.ncbi.nlm.nih.gov/entrez/dispomim.cgi?id=172470) | **1.63** |
| 1422329_A_AT | neurotrophic tyrosine kinase, receptor, type 3 | [NTRK3](http://www.gene.ucl.ac.uk/cgi-bin/nomenclature/searchgenes.pl?field=symbol&anchor=equals&symbol_search=Search&number=100&format=html&sortby=symbol&match=NTRK3) | kinase activity;protein kinase activity;protein serine/threonine kinase activity;diacylglycerol binding;protein-tyrosine kinase activity;ATP binding;transferase activity | [191316](http://www.ncbi.nlm.nih.gov/entrez/dispomim.cgi?id=191316) | **1.73** |
| 1422407_S_AT | Harvey rat sarcoma virus oncogene 1 | [HRAS1](http://www.gene.ucl.ac.uk/cgi-bin/nomenclature/searchgenes.pl?field=symbol&anchor=equals&symbol_search=Search&number=100&format=html&sortby=symbol&match=HRAS1) | kinase activity;protein kinase activity;protein serine/threonine kinase activity;magnesium ion binding;ATP binding;transferase activity |  | **-1.57** |
| 1422411_S_AT | eosinophil-associated, ribonuclease A family, member 3 | [EAR1](http://www.gene.ucl.ac.uk/cgi-bin/nomenclature/searchgenes.pl?field=symbol&anchor=equals&symbol_search=Search&number=100&format=html&sortby=symbol&match=EAR1) | kinase activity;protein kinase activity;protein serine/threonine kinase activity;protein-tyrosine kinase activity;ATP binding;transferase activity |  | **-3.59** |
| 1422437_AT | gb:NM_007737.1 /DB_XREF=gi:6680969 /GEN=Col5a2 /FE |  | kinase activity;protein kinase activity;protein serine/threonine kinase activity;protein-tyrosine kinase activity;ATP binding;transferase activity |  | **-3.58** |
| 1422438_AT | epoxide hydrolase 1, microsomal | [EPHX1](http://www.gene.ucl.ac.uk/cgi-bin/nomenclature/searchgenes.pl?field=symbol&anchor=equals&symbol_search=Search&number=100&format=html&sortby=symbol&match=EPHX1) | kinase activity;protein kinase activity;protein serine/threonine kinase activity;receptor activity;ATP binding;protein-tyrosine kinase activity;transferase activity | [132810](http://www.ncbi.nlm.nih.gov/entrez/dispomim.cgi?id=132810) | **1.61** |
| 1422439_A_AT | cyclin-dependent kinase 4 | [CDK4](http://www.gene.ucl.ac.uk/cgi-bin/nomenclature/searchgenes.pl?field=symbol&anchor=equals&symbol_search=Search&number=100&format=html&sortby=symbol&match=CDK4) | kinase activity;protein kinase activity;protein serine/threonine kinase activity;SNF1A/AMP-activated protein kinase activity;ATP binding;transferase activity | [123829](http://www.ncbi.nlm.nih.gov/entrez/dispomim.cgi?id=123829) | **-1.44** |
| 1422440_AT | cyclin-dependent kinase 4 | [CDK4](http://www.gene.ucl.ac.uk/cgi-bin/nomenclature/searchgenes.pl?field=symbol&anchor=equals&symbol_search=Search&number=100&format=html&sortby=symbol&match=CDK4) | kinase activity;protein serine/threonine kinase activity;ankyrin binding;ATP binding;transferase activity | [123829](http://www.ncbi.nlm.nih.gov/entrez/dispomim.cgi?id=123829) | **-1.45** |
| 1422441_X_AT | cyclin-dependent kinase 4 | [CDK4](http://www.gene.ucl.ac.uk/cgi-bin/nomenclature/searchgenes.pl?field=symbol&anchor=equals&symbol_search=Search&number=100&format=html&sortby=symbol&match=CDK4) | kinase activity;protein serine/threonine kinase activity;ATP binding | [123829](http://www.ncbi.nlm.nih.gov/entrez/dispomim.cgi?id=123829) | **-1.54** |
| 1422445_AT | integrin alpha 6 | [ITGA6](http://www.gene.ucl.ac.uk/cgi-bin/nomenclature/searchgenes.pl?field=symbol&anchor=equals&symbol_search=Search&number=100&format=html&sortby=symbol&match=ITGA6) | kinase activity;protein serine/threonine kinase activity;ATP binding;transferase activity | [147556](http://www.ncbi.nlm.nih.gov/entrez/dispomim.cgi?id=147556) | **1.57** |
| 1422459_A_AT | proteasome (prosome, macropain) 26S subunit, non-ATPase, 13 | [PSMD13](http://www.gene.ucl.ac.uk/cgi-bin/nomenclature/searchgenes.pl?field=symbol&anchor=equals&symbol_search=Search&number=100&format=html&sortby=symbol&match=PSMD13) | kinase activity;protein serine/threonine kinase activity;ATP binding;transferase activity | [603481](http://www.ncbi.nlm.nih.gov/entrez/dispomim.cgi?id=603481) | **-1.3** |
| 1422463_A_AT | mitochondrial ribosomal protein L3 | [MRPL3](http://www.gene.ucl.ac.uk/cgi-bin/nomenclature/searchgenes.pl?field=symbol&anchor=equals&symbol_search=Search&number=100&format=html&sortby=symbol&match=MRPL3) | kinase activity;protein serine/threonine kinase activity;ATP binding;transferase activity;protein kinase activity;calmodulin binding;calcium/calmodulin-dependent protein kinase activity;protein-tyrosine kinase activity;calcium ion binding | [607118](http://www.ncbi.nlm.nih.gov/entrez/dispomim.cgi?id=607118) | **1.29** |
| 1422464_AT | mitochondrial ribosomal protein L3 | [MRPL3](http://www.gene.ucl.ac.uk/cgi-bin/nomenclature/searchgenes.pl?field=symbol&anchor=equals&symbol_search=Search&number=100&format=html&sortby=symbol&match=MRPL3) | kinase activity;protein serine/threonine kinase activity;cGMP-dependent protein kinase activity;ATP binding;transferase activity;3',5'-cGMP binding;cAMP-dependent protein kinase regulator activity;protein binding;protein kinase activity | [607118](http://www.ncbi.nlm.nih.gov/entrez/dispomim.cgi?id=607118) | **1.26** |
| 1422470_AT | BCL2/adenovirus E1B 19kDa-interacting protein 1, NIP3 | [BNIP3](http://www.gene.ucl.ac.uk/cgi-bin/nomenclature/searchgenes.pl?field=symbol&anchor=equals&symbol_search=Search&number=100&format=html&sortby=symbol&match=BNIP3) | kinase activity;receptor activity;protein serine/threonine kinase activity;ATP binding;transferase activity;protein binding;protein kinase activity;protein-tyrosine kinase activity;transmembrane receptor protein tyrosine kinase activity | [603293](http://www.ncbi.nlm.nih.gov/entrez/dispomim.cgi?id=603293) | **1.31** |
| 1422476_AT | interferon gamma inducible protein 30 | [IFI30](http://www.gene.ucl.ac.uk/cgi-bin/nomenclature/searchgenes.pl?field=symbol&anchor=equals&symbol_search=Search&number=100&format=html&sortby=symbol&match=IFI30) | kinase activity;receptor activity;protein serine/threonine kinase activity;ATP binding;transferase activity;vascular endothelial growth factor receptor activity;protein kinase activity;protein-tyrosine kinase activity;transmembrane receptor protein tyrosine kinase activity | [604664](http://www.ncbi.nlm.nih.gov/entrez/dispomim.cgi?id=604664) | **-3.23** |
| 1422477_AT | DNA segment, Chr 18, ERATO Doi 289, expressed | [CABLES1](http://www.gene.ucl.ac.uk/cgi-bin/nomenclature/searchgenes.pl?field=symbol&anchor=equals&symbol_search=Search&number=100&format=html&sortby=symbol&match=CABLES1) | kinase activity;receptor activity;protein serine/threonine kinase activity;transmembrane receptor protein serine/threonine kinase activity;ATP binding;transforming growth factor beta receptor activity;transferase activity;type II transforming growth factor beta receptor activity;protein kinase activity;transmembrane receptor protein serine/threonine kinase signaling protein activity;protein-tyrosine kinase activity;hormone binding |  | **1.49** |
| 1422478_A_AT | acetyl-Coenzyme A synthetase 2 (ADP forming) | [ACAS2](http://www.gene.ucl.ac.uk/cgi-bin/nomenclature/searchgenes.pl?field=symbol&anchor=equals&symbol_search=Search&number=100&format=html&sortby=symbol&match=ACAS2) | kinase activity;receptor signaling complex scaffold activity;protein transporter activity | [605832](http://www.ncbi.nlm.nih.gov/entrez/dispomim.cgi?id=605832) | **1.6** |
| 1422479_AT | acetyl-Coenzyme A synthetase 2 (ADP forming) | [ACAS2](http://www.gene.ucl.ac.uk/cgi-bin/nomenclature/searchgenes.pl?field=symbol&anchor=equals&symbol_search=Search&number=100&format=html&sortby=symbol&match=ACAS2) | kinase activity;SNF1A/AMP-activated protein kinase activity | [605832](http://www.ncbi.nlm.nih.gov/entrez/dispomim.cgi?id=605832) | **1.52** |
| 1422483_A_AT | cytochrome c, somatic | [CYCS](http://www.gene.ucl.ac.uk/cgi-bin/nomenclature/searchgenes.pl?field=symbol&anchor=equals&symbol_search=Search&number=100&format=html&sortby=symbol&match=CYCS) | kinase activity;structural constituent of ribosome;Ras interactor activity | [123970](http://www.ncbi.nlm.nih.gov/entrez/dispomim.cgi?id=123970) | **1.24** |
| 1422484_AT | cytochrome c, somatic | [CYCS](http://www.gene.ucl.ac.uk/cgi-bin/nomenclature/searchgenes.pl?field=symbol&anchor=equals&symbol_search=Search&number=100&format=html&sortby=symbol&match=CYCS) | kinase activity;thymidylate kinase activity;ATP binding;transferase activity | [123970](http://www.ncbi.nlm.nih.gov/entrez/dispomim.cgi?id=123970) | **1.44** |
| 1422492_AT | coproporphyrinogen oxidase | [CPOX](http://www.gene.ucl.ac.uk/cgi-bin/nomenclature/searchgenes.pl?field=symbol&anchor=equals&symbol_search=Search&number=100&format=html&sortby=symbol&match=CPOX) | kinase activity;uridine kinase activity;ATP binding;transferase activity | [121300](http://www.ncbi.nlm.nih.gov/entrez/dispomim.cgi?id=121300) | **1.54** |
| 1422493_AT | coproporphyrinogen oxidase | [CPOX](http://www.gene.ucl.ac.uk/cgi-bin/nomenclature/searchgenes.pl?field=symbol&anchor=equals&symbol_search=Search&number=100&format=html&sortby=symbol&match=CPOX) | kinase activity;uridine kinase activity;cytidylate kinase activity;phosphotransferase activity, phosphate group as acceptor;ATP binding;transferase activity | [121300](http://www.ncbi.nlm.nih.gov/entrez/dispomim.cgi?id=121300) | **1.36** |
| 1422495_A_AT | high mobility group nucleosomal binding domain 1 | [HMGN1](http://www.gene.ucl.ac.uk/cgi-bin/nomenclature/searchgenes.pl?field=symbol&anchor=equals&symbol_search=Search&number=100&format=html&sortby=symbol&match=HMGN1) | latrotoxin receptor activity | [163920](http://www.ncbi.nlm.nih.gov/entrez/dispomim.cgi?id=163920) | **-1.3** |
| 1422497_AT | solute carrier family 30 (zinc transporter), member 5 | [SLC30A5](http://www.gene.ucl.ac.uk/cgi-bin/nomenclature/searchgenes.pl?field=symbol&anchor=equals&symbol_search=Search&number=100&format=html&sortby=symbol&match=SLC30A5) | latrotoxin receptor activity | [607819](http://www.ncbi.nlm.nih.gov/entrez/dispomim.cgi?id=607819) | **-1.26** |
| 1422500_AT | isocitrate dehydrogenase 3 (NAD+) alpha | [IDH3A](http://www.gene.ucl.ac.uk/cgi-bin/nomenclature/searchgenes.pl?field=symbol&anchor=equals&symbol_search=Search&number=100&format=html&sortby=symbol&match=IDH3A) | legumain activity;cysteine-type endopeptidase activity;hydrolase activity;cysteine-type peptidase activity | [601149](http://www.ncbi.nlm.nih.gov/entrez/dispomim.cgi?id=601149) | **1.45** |
| 1422501_S_AT | isocitrate dehydrogenase 3 (NAD+) alpha | [IDH3A](http://www.gene.ucl.ac.uk/cgi-bin/nomenclature/searchgenes.pl?field=symbol&anchor=equals&symbol_search=Search&number=100&format=html&sortby=symbol&match=IDH3A) | ligand-dependent nuclear receptor activity;protein binding;steroid hormone receptor activity;DNA binding;transcription factor activity;receptor activity;transcriptional activator activity | [601149](http://www.ncbi.nlm.nih.gov/entrez/dispomim.cgi?id=601149) | **1.56** |
| 1422506_A_AT | cystatin B | [CSTB](http://www.gene.ucl.ac.uk/cgi-bin/nomenclature/searchgenes.pl?field=symbol&anchor=equals&symbol_search=Search&number=100&format=html&sortby=symbol&match=CSTB) | ligand-dependent nuclear receptor activity;steroid hormone receptor activity;DNA binding;AF-2 domain binding;receptor activity;transcriptional activator activity;transcription factor activity;calmodulin binding | [601145](http://www.ncbi.nlm.nih.gov/entrez/dispomim.cgi?id=601145) | **-2.92** |
| 1422507_AT | cystatin B | [CSTB](http://www.gene.ucl.ac.uk/cgi-bin/nomenclature/searchgenes.pl?field=symbol&anchor=equals&symbol_search=Search&number=100&format=html&sortby=symbol&match=CSTB) | ligand-dependent nuclear receptor activity;steroid hormone receptor activity;DNA binding;receptor activity;transcription factor activity;steroid binding | [601145](http://www.ncbi.nlm.nih.gov/entrez/dispomim.cgi?id=601145) | **-2.76** |
| 1422508_AT | ATPase, H+ transporting, V1 subunit A, isoform 1 | [ATP6V1A1](http://www.gene.ucl.ac.uk/cgi-bin/nomenclature/searchgenes.pl?field=symbol&anchor=equals&symbol_search=Search&number=100&format=html&sortby=symbol&match=ATP6V1A1) | ligand-dependent nuclear receptor activity;steroid hormone receptor activity;DNA binding;transcription factor activity;receptor activity |  | **-1.67** |
| 1422509_AT | U2 small nuclear ribonucleoprotein auxiliary factor (U2AF) 1 | [U2AF1](http://www.gene.ucl.ac.uk/cgi-bin/nomenclature/searchgenes.pl?field=symbol&anchor=equals&symbol_search=Search&number=100&format=html&sortby=symbol&match=U2AF1) | ligand-dependent nuclear receptor activity;steroid hormone receptor activity;DNA binding;transcription factor activity;receptor activity;steroid binding | [191317](http://www.ncbi.nlm.nih.gov/entrez/dispomim.cgi?id=191317) | **-1.3** |
| 1422522_AT | gb:NM_011814.1 /DB_XREF=gi:6753925 /GEN=Fxr2h /FEA |  | ligand-dependent nuclear receptor activity;steroid hormone receptor activity;DNA binding;transcription factor activity;receptor activity;steroid binding |  | **1.39** |
| 1422526_AT | acyl-CoA synthetase long-chain family member 1 | [ACSL1](http://www.gene.ucl.ac.uk/cgi-bin/nomenclature/searchgenes.pl?field=symbol&anchor=equals&symbol_search=Search&number=100&format=html&sortby=symbol&match=ACSL1) | ligand-regulated transcription factor activity;protein binding;DNA binding;calcium ion binding;receptor activity;transcription factor activity;transcription regulator activity | [152425](http://www.ncbi.nlm.nih.gov/entrez/dispomim.cgi?id=152425) | **2.03** |
| 1422527_AT | histocompatibility 2, class II, locus DMa | [H2-DMA](http://www.gene.ucl.ac.uk/cgi-bin/nomenclature/searchgenes.pl?field=symbol&anchor=equals&symbol_search=Search&number=100&format=html&sortby=symbol&match=H2-DMA) | ligase activity |  | **-1.76** |
| 1422537_A_AT | inhibitor of DNA binding 2 | [IDB2](http://www.gene.ucl.ac.uk/cgi-bin/nomenclature/searchgenes.pl?field=symbol&anchor=equals&symbol_search=Search&number=100&format=html&sortby=symbol&match=IDB2) | ligase activity;acyltransferase activity;catalytic activity;gamma-glutamyltransferase activity;acetate-CoA ligase activity;transferase activity |  | **-3.07** |
| 1422541_AT | protein tyrosine phosphatase, receptor type, M | [PTPRM](http://www.gene.ucl.ac.uk/cgi-bin/nomenclature/searchgenes.pl?field=symbol&anchor=equals&symbol_search=Search&number=100&format=html&sortby=symbol&match=PTPRM) | ligase activity;arginine-tRNA ligase activity;ATP binding;tRNA ligase activity | [176888](http://www.ncbi.nlm.nih.gov/entrez/dispomim.cgi?id=176888) | **1.51** |
| 1422547_AT | RAN binding protein 1 | [RANBP1](http://www.gene.ucl.ac.uk/cgi-bin/nomenclature/searchgenes.pl?field=symbol&anchor=equals&symbol_search=Search&number=100&format=html&sortby=symbol&match=RANBP1) | ligase activity;asparagine synthase (glutamine-hydrolyzing) activity | [601180](http://www.ncbi.nlm.nih.gov/entrez/dispomim.cgi?id=601180) | **-1.42** |
| 1422555_S_AT | guanine nucleotide binding protein, alpha 13 | [GNA13](http://www.gene.ucl.ac.uk/cgi-bin/nomenclature/searchgenes.pl?field=symbol&anchor=equals&symbol_search=Search&number=100&format=html&sortby=symbol&match=GNA13) | ligase activity;asparagine synthase (glutamine-hydrolyzing) activity | [604406](http://www.ncbi.nlm.nih.gov/entrez/dispomim.cgi?id=604406) | **-1.28** |
| 1422556_AT | guanine nucleotide binding protein, alpha 13 | [GNA13](http://www.gene.ucl.ac.uk/cgi-bin/nomenclature/searchgenes.pl?field=symbol&anchor=equals&symbol_search=Search&number=100&format=html&sortby=symbol&match=GNA13) | ligase activity;ATP binding;phenylalanine-tRNA ligase activity;tRNA ligase activity | [604406](http://www.ncbi.nlm.nih.gov/entrez/dispomim.cgi?id=604406) | **-1.25** |
| 1422557_S_AT | metallothionein 1 | [MT1](http://www.gene.ucl.ac.uk/cgi-bin/nomenclature/searchgenes.pl?field=symbol&anchor=equals&symbol_search=Search&number=100&format=html&sortby=symbol&match=MT1) | ligase activity;ATP binding;phenylalanine-tRNA ligase activity;tRNA ligase activity |  | **-2** |
| 1422558_AT | guanidinoacetate methyltransferase | [GAMT](http://www.gene.ucl.ac.uk/cgi-bin/nomenclature/searchgenes.pl?field=symbol&anchor=equals&symbol_search=Search&number=100&format=html&sortby=symbol&match=GAMT) | ligase activity;ATP binding;transferase activity, transferring acyl groups, acyl groups converted into alkyl on transfer;transferase activity;citrate (Si)-synthase activity;lyase activity;magnesium ion binding;ATP citrate synthase activity;catalytic activity | [601240](http://www.ncbi.nlm.nih.gov/entrez/dispomim.cgi?id=601240) | **-3.72** |
| 1422562_AT | Ras-related associated with diabetes | [RRAD](http://www.gene.ucl.ac.uk/cgi-bin/nomenclature/searchgenes.pl?field=symbol&anchor=equals&symbol_search=Search&number=100&format=html&sortby=symbol&match=RRAD) | ligase activity;catalytic activity;acetate-CoA ligase activity | [179503](http://www.ncbi.nlm.nih.gov/entrez/dispomim.cgi?id=179503) | **-6.7** |
| 1422566_AT | transcription factor EB | [TCFEB](http://www.gene.ucl.ac.uk/cgi-bin/nomenclature/searchgenes.pl?field=symbol&anchor=equals&symbol_search=Search&number=100&format=html&sortby=symbol&match=TCFEB) | ligase activity;catalytic activity;acyltransferase activity;gamma-glutamyltransferase activity;acetate-CoA ligase activity;transferase activity |  | **1.38** |
| 1422572_AT | ras homolog gene family, member G | [RHOG](http://www.gene.ucl.ac.uk/cgi-bin/nomenclature/searchgenes.pl?field=symbol&anchor=equals&symbol_search=Search&number=100&format=html&sortby=symbol&match=RHOG) | ligase activity;CTP synthase activity;catalytic activity | [179505](http://www.ncbi.nlm.nih.gov/entrez/dispomim.cgi?id=179505) | **-1.4** |
| 1422573_AT | gb:D85596.1 /DB_XREF=gi:2094764 /GEN=AMPD3 /FEA=FL |  | ligase activity;GTP binding;catalytic activity;succinate-CoA ligase (GDP-forming) activity |  | **-2.5** |
| 1422576_AT | spinocerebellar ataxia 10 homolog (human) | [SCA10](http://www.gene.ucl.ac.uk/cgi-bin/nomenclature/searchgenes.pl?field=symbol&anchor=equals&symbol_search=Search&number=100&format=html&sortby=symbol&match=SCA10) | ligase activity;ubiquitin-protein ligase activity |  | **-1.35** |
| 1422577_AT | citrate synthase | [CS](http://www.gene.ucl.ac.uk/cgi-bin/nomenclature/searchgenes.pl?field=symbol&anchor=equals&symbol_search=Search&number=100&format=html&sortby=symbol&match=CS) | ligase activity;ubiquitin-protein ligase activity | [118950](http://www.ncbi.nlm.nih.gov/entrez/dispomim.cgi?id=118950) | **1.46** |
| 1422578_AT | citrate synthase | [CS](http://www.gene.ucl.ac.uk/cgi-bin/nomenclature/searchgenes.pl?field=symbol&anchor=equals&symbol_search=Search&number=100&format=html&sortby=symbol&match=CS) | ligase activity;ubiquitin-protein ligase activity | [118950](http://www.ncbi.nlm.nih.gov/entrez/dispomim.cgi?id=118950) | **1.28** |
| 1422580_AT | myosin, light polypeptide 4 | [MYL4](http://www.gene.ucl.ac.uk/cgi-bin/nomenclature/searchgenes.pl?field=symbol&anchor=equals&symbol_search=Search&number=100&format=html&sortby=symbol&match=MYL4) | ligase activity;ubiquitin-protein ligase activity;ubiquitin conjugating enzyme activity | [160770](http://www.ncbi.nlm.nih.gov/entrez/dispomim.cgi?id=160770) | **-67.93** |
| 1422587_AT | RIKEN cDNA C630002M10 gene | [C630002M10RIK](http://www.gene.ucl.ac.uk/cgi-bin/nomenclature/searchgenes.pl?field=symbol&anchor=equals&symbol_search=Search&number=100&format=html&sortby=symbol&match=C630002M10RIK) | ligase activity;ubiquitin-protein ligase activity;ubiquitin conjugating enzyme activity |  | **-2.53** |
| 1422593_AT | adaptor-related protein complex 3, sigma 1 subunit | [AP3S1](http://www.gene.ucl.ac.uk/cgi-bin/nomenclature/searchgenes.pl?field=symbol&anchor=equals&symbol_search=Search&number=100&format=html&sortby=symbol&match=AP3S1) | ligase activity;ubiquitin-protein ligase activity;ubiquitin conjugating enzyme activity | [601507](http://www.ncbi.nlm.nih.gov/entrez/dispomim.cgi?id=601507) | **-1.29** |
| 1422594_AT | RIKEN cDNA 5730470L24 gene | [5730470L24RIK](http://www.gene.ucl.ac.uk/cgi-bin/nomenclature/searchgenes.pl?field=symbol&anchor=equals&symbol_search=Search&number=100&format=html&sortby=symbol&match=5730470L24RIK) | ligase activity;ubiquitin-protein ligase activity;ubiquitin conjugating enzyme activity |  | **-1.37** |
| 1422595_S_AT | RIKEN cDNA 5730470L24 gene | [5730470L24RIK](http://www.gene.ucl.ac.uk/cgi-bin/nomenclature/searchgenes.pl?field=symbol&anchor=equals&symbol_search=Search&number=100&format=html&sortby=symbol&match=5730470L24RIK) | ligase activity;ubiquitin-protein ligase activity;ubiquitin conjugating enzyme activity |  | **-1.32** |
| 1422598_AT | gb:NM_009813.1 /DB_XREF=gi:6753289 /GEN=Casq1 /FEA |  | lipase activity;catalytic activity;hydrolase activity |  | **-1.21** |
| 1422601_AT | serine (or cysteine) proteinase inhibitor, clade B, member 9 | [SERPINB9](http://www.gene.ucl.ac.uk/cgi-bin/nomenclature/searchgenes.pl?field=symbol&anchor=equals&symbol_search=Search&number=100&format=html&sortby=symbol&match=SERPINB9) | lipid binding | [601799](http://www.ncbi.nlm.nih.gov/entrez/dispomim.cgi?id=601799) | **-1.37** |
| 1422605_AT | protein phosphatase 1, regulatory (inhibitor) subunit 1A | [PPP1R1A](http://www.gene.ucl.ac.uk/cgi-bin/nomenclature/searchgenes.pl?field=symbol&anchor=equals&symbol_search=Search&number=100&format=html&sortby=symbol&match=PPP1R1A) | lipid binding |  | **9.13** |
| 1422606_AT | C1q and tumor necrosis factor related protein 3 | [C1QTNF3](http://www.gene.ucl.ac.uk/cgi-bin/nomenclature/searchgenes.pl?field=symbol&anchor=equals&symbol_search=Search&number=100&format=html&sortby=symbol&match=C1QTNF3) | lipid binding;phospholipid binding |  | **-28.42** |
| 1422612_AT | hexokinase 2 | [HK2](http://www.gene.ucl.ac.uk/cgi-bin/nomenclature/searchgenes.pl?field=symbol&anchor=equals&symbol_search=Search&number=100&format=html&sortby=symbol&match=HK2) | lipid phosphatase activity;phosphatidate phosphatase activity;hydrolase activity | [601125](http://www.ncbi.nlm.nih.gov/entrez/dispomim.cgi?id=601125) | **1.65** |
| 1422615_AT | mitogen-activated protein kinase kinase kinase kinase 4 | [MAP4K4](http://www.gene.ucl.ac.uk/cgi-bin/nomenclature/searchgenes.pl?field=symbol&anchor=equals&symbol_search=Search&number=100&format=html&sortby=symbol&match=MAP4K4) | lipid phosphatase activity;phosphatidate phosphatase activity;hydrolase activity | [604666](http://www.ncbi.nlm.nih.gov/entrez/dispomim.cgi?id=604666) | **-1.45** |
| 1422619_AT | gb:NM_008903.1 /DB_XREF=gi:6679430 /GEN=Ppap2a /FE |  | lipid transporter activity;calcium ion binding;receptor activity |  | **-1.45** |
| 1422620_S_AT | phosphatidic acid phosphatase 2a | [PPAP2A](http://www.gene.ucl.ac.uk/cgi-bin/nomenclature/searchgenes.pl?field=symbol&anchor=equals&symbol_search=Search&number=100&format=html&sortby=symbol&match=PPAP2A) | lipid transporter activity;calcium ion binding;receptor activity | [607124](http://www.ncbi.nlm.nih.gov/entrez/dispomim.cgi?id=607124) | **-1.56** |
| 1422621_AT | RAN binding protein 2 | [RANBP2](http://www.gene.ucl.ac.uk/cgi-bin/nomenclature/searchgenes.pl?field=symbol&anchor=equals&symbol_search=Search&number=100&format=html&sortby=symbol&match=RANBP2) | lipid transporter activity;calcium ion binding;receptor activity |  | **-1.23** |
| 1422622_AT | nitric oxide synthase 3, endothelial cell | [NOS3](http://www.gene.ucl.ac.uk/cgi-bin/nomenclature/searchgenes.pl?field=symbol&anchor=equals&symbol_search=Search&number=100&format=html&sortby=symbol&match=NOS3) | lipid transporter activity;calcium ion binding;receptor activity;lipoprotein binding | [163729](http://www.ncbi.nlm.nih.gov/entrez/dispomim.cgi?id=163729) | **1.91** |
| 1422627_A_AT | McKusick-Kaufman syndrome protein | [MKKS](http://www.gene.ucl.ac.uk/cgi-bin/nomenclature/searchgenes.pl?field=symbol&anchor=equals&symbol_search=Search&number=100&format=html&sortby=symbol&match=MKKS) | lipid transporter activity;lipoprotein lipase activity;catalytic activity;heparin binding;hydrolase activity | [604896](http://www.ncbi.nlm.nih.gov/entrez/dispomim.cgi?id=604896) | **1.44** |
| 1422638_S_AT | Ras association (RalGDS/AF-6) domain family 5 | [RASSF5](http://www.gene.ucl.ac.uk/cgi-bin/nomenclature/searchgenes.pl?field=symbol&anchor=equals&symbol_search=Search&number=100&format=html&sortby=symbol&match=RASSF5) | lipid transporter activity;lipoprotein lipase activity;catalytic activity;hydrolase activity;heparin binding | [607020](http://www.ncbi.nlm.nih.gov/entrez/dispomim.cgi?id=607020) | **-2.03** |
| 1422645_AT | hemochromatosis | [HFE](http://www.gene.ucl.ac.uk/cgi-bin/nomenclature/searchgenes.pl?field=symbol&anchor=equals&symbol_search=Search&number=100&format=html&sortby=symbol&match=HFE) | lipid transporter activity;nucleic acid binding;RNA binding | [235200](http://www.ncbi.nlm.nih.gov/entrez/dispomim.cgi?id=235200) | **1.28** |
| 1422654_AT | sarcoglycan, alpha (dystrophin-associated glycoprotein) | [SGCA](http://www.gene.ucl.ac.uk/cgi-bin/nomenclature/searchgenes.pl?field=symbol&anchor=equals&symbol_search=Search&number=100&format=html&sortby=symbol&match=SGCA) | lipid transporter activity;nucleic acid binding;RNA binding | [600119](http://www.ncbi.nlm.nih.gov/entrez/dispomim.cgi?id=600119) | **1.27** |
| 1422659_AT | calcium/calmodulin-dependent protein kinase II, delta | [CAMK2D](http://www.gene.ucl.ac.uk/cgi-bin/nomenclature/searchgenes.pl?field=symbol&anchor=equals&symbol_search=Search&number=100&format=html&sortby=symbol&match=CAMK2D) | lipid transporter activity;receptor activity;electron transporter activity;copper ion binding | [607708](http://www.ncbi.nlm.nih.gov/entrez/dispomim.cgi?id=607708) | **-2.9** |
| 1422660_AT | RNA binding motif protein 3 | [RBM3](http://www.gene.ucl.ac.uk/cgi-bin/nomenclature/searchgenes.pl?field=symbol&anchor=equals&symbol_search=Search&number=100&format=html&sortby=symbol&match=RBM3) | lipid transporter activity;receptor activity;electron transporter activity;copper ion binding | [300027](http://www.ncbi.nlm.nih.gov/entrez/dispomim.cgi?id=300027) | **-1.64** |
| 1422669_AT | estrogen receptor-binding fragment-associated gene 9 | [EBAG9](http://www.gene.ucl.ac.uk/cgi-bin/nomenclature/searchgenes.pl?field=symbol&anchor=equals&symbol_search=Search&number=100&format=html&sortby=symbol&match=EBAG9) | lipid transporter activity;scavenger receptor activity;receptor activity | [605772](http://www.ncbi.nlm.nih.gov/entrez/dispomim.cgi?id=605772) | **1.2** |
| 1422671_S_AT | N-acetylated alpha-linked acidic dipeptidase 2 | [NAALAD2](http://www.gene.ucl.ac.uk/cgi-bin/nomenclature/searchgenes.pl?field=symbol&anchor=equals&symbol_search=Search&number=100&format=html&sortby=symbol&match=NAALAD2) | lipopolysaccharide N-acetylglucosaminyltransferase activity;transferase activity, transferring glycosyl groups;beta-galactosyl-N-acetylglucosaminylgalactosylglucosyl-ceramide beta-1,3-acetylglucosaminyltransferase activity;galactosyltransferase activity;transferase activity |  | **-1.52** |
| 1422675_AT | SWI/SNF related, matrix associated, actin dependent regulator of chromatin, subfamily e, member 1 | [SMARCE1](http://www.gene.ucl.ac.uk/cgi-bin/nomenclature/searchgenes.pl?field=symbol&anchor=equals&symbol_search=Search&number=100&format=html&sortby=symbol&match=SMARCE1) | L-lactate dehydrogenase activity;oxidoreductase activity | [603111](http://www.ncbi.nlm.nih.gov/entrez/dispomim.cgi?id=603111) | **-1.32** |
| 1422676_AT | SWI/SNF related, matrix associated, actin dependent regulator of chromatin, subfamily e, member 1 | [SMARCE1](http://www.gene.ucl.ac.uk/cgi-bin/nomenclature/searchgenes.pl?field=symbol&anchor=equals&symbol_search=Search&number=100&format=html&sortby=symbol&match=SMARCE1) | L-lactate dehydrogenase activity;oxidoreductase activity | [603111](http://www.ncbi.nlm.nih.gov/entrez/dispomim.cgi?id=603111) | **-1.25** |
| 1422677_AT | diacylglycerol O-acyltransferase 2 | [DGAT2](http://www.gene.ucl.ac.uk/cgi-bin/nomenclature/searchgenes.pl?field=symbol&anchor=equals&symbol_search=Search&number=100&format=html&sortby=symbol&match=DGAT2) | L-lactate dehydrogenase activity;oxidoreductase activity | [606983](http://www.ncbi.nlm.nih.gov/entrez/dispomim.cgi?id=606983) | **2.66** |
| 1422678_AT | diacylglycerol O-acyltransferase 2 | [DGAT2](http://www.gene.ucl.ac.uk/cgi-bin/nomenclature/searchgenes.pl?field=symbol&anchor=equals&symbol_search=Search&number=100&format=html&sortby=symbol&match=DGAT2) | L-lactate dehydrogenase activity;oxidoreductase activity | [606983](http://www.ncbi.nlm.nih.gov/entrez/dispomim.cgi?id=606983) | **2.55** |
| 1422687_AT | gb:NM_010937.1 /DB_XREF=gi:7242161 /GEN=Nras /FEA= |  | L-malate dehydrogenase activity;malic enzyme activity;malate dehydrogenase activity;oxidoreductase activity |  | **-1.39** |
| 1422688_A_AT | neuroblastoma ras oncogene | [NRAS](http://www.gene.ucl.ac.uk/cgi-bin/nomenclature/searchgenes.pl?field=symbol&anchor=equals&symbol_search=Search&number=100&format=html&sortby=symbol&match=NRAS) | L-malate dehydrogenase activity;malic enzyme activity;malate dehydrogenase activity;oxidoreductase activity | [164790](http://www.ncbi.nlm.nih.gov/entrez/dispomim.cgi?id=164790) | **-1.3** |
| 1422693_A_AT | RNA polymerase II transcriptional coactivator | [RPO2TC1](http://www.gene.ucl.ac.uk/cgi-bin/nomenclature/searchgenes.pl?field=symbol&anchor=equals&symbol_search=Search&number=100&format=html&sortby=symbol&match=RPO2TC1) | long-chain-fatty-acid-CoA ligase activity;magnesium ion binding;ligase activity;catalytic activity |  | **-1.26** |
| 1422702_AT | ornithine decarboxylase antizyme inhibitor | [OAZIN](http://www.gene.ucl.ac.uk/cgi-bin/nomenclature/searchgenes.pl?field=symbol&anchor=equals&symbol_search=Search&number=100&format=html&sortby=symbol&match=OAZIN) | long-chain-fatty-acid-CoA ligase activity;magnesium ion binding;ligase activity;catalytic activity | [607909](http://www.ncbi.nlm.nih.gov/entrez/dispomim.cgi?id=607909) | **-1.76** |
| 1422705_AT | transmembrane, prostate androgen induced RNA | [TMEPAI](http://www.gene.ucl.ac.uk/cgi-bin/nomenclature/searchgenes.pl?field=symbol&anchor=equals&symbol_search=Search&number=100&format=html&sortby=symbol&match=TMEPAI) | long-chain-fatty-acid-CoA ligase activity;magnesium ion binding;ligase activity;catalytic activity | [606564](http://www.ncbi.nlm.nih.gov/entrez/dispomim.cgi?id=606564) | **-1.76** |
| 1422706_AT | Transmembrane, prostate androgen induced RNA | [TMEPAI](http://www.gene.ucl.ac.uk/cgi-bin/nomenclature/searchgenes.pl?field=symbol&anchor=equals&symbol_search=Search&number=100&format=html&sortby=symbol&match=TMEPAI) | long-chain-fatty-acid-CoA ligase activity;magnesium ion binding;ligase activity;catalytic activity | [606564](http://www.ncbi.nlm.nih.gov/entrez/dispomim.cgi?id=606564) | **-1.82** |
| 1422732_AT | polymerase (DNA-directed), delta interacting protein 2 | [POLDIP2](http://www.gene.ucl.ac.uk/cgi-bin/nomenclature/searchgenes.pl?field=symbol&anchor=equals&symbol_search=Search&number=100&format=html&sortby=symbol&match=POLDIP2) | long-chain-fatty-acid-CoA ligase activity;magnesium ion binding;ligase activity;catalytic activity;acetate-CoA ligase activity |  | **1.2** |
| 1422738_AT | discoidin domain receptor family, member 2 | [DDR2](http://www.gene.ucl.ac.uk/cgi-bin/nomenclature/searchgenes.pl?field=symbol&anchor=equals&symbol_search=Search&number=100&format=html&sortby=symbol&match=DDR2) | long-chain-fatty-acid-CoA ligase activity;magnesium ion binding;ligase activity;catalytic activity;acetate-CoA ligase activity | [191311](http://www.ncbi.nlm.nih.gov/entrez/dispomim.cgi?id=191311) | **-1.59** |
| 1422743_AT | phosphorylase kinase alpha 1 | [PHKA1](http://www.gene.ucl.ac.uk/cgi-bin/nomenclature/searchgenes.pl?field=symbol&anchor=equals&symbol_search=Search&number=100&format=html&sortby=symbol&match=PHKA1) | long-chain-fatty-acid-CoA ligase activity;magnesium ion binding;ligase activity;catalytic activity;acetate-CoA ligase activity | [311870](http://www.ncbi.nlm.nih.gov/entrez/dispomim.cgi?id=311870) | **1.58** |
| 1422744_AT | phosphorylase kinase alpha 1 | [PHKA1](http://www.gene.ucl.ac.uk/cgi-bin/nomenclature/searchgenes.pl?field=symbol&anchor=equals&symbol_search=Search&number=100&format=html&sortby=symbol&match=PHKA1) | long-chain-fatty-acyl-CoA reductase activity | [311870](http://www.ncbi.nlm.nih.gov/entrez/dispomim.cgi?id=311870) | **1.79** |
| 1422746_S_AT | gb:BI250512 /DB_XREF=gi:14798930 /DB_XREF=60299356 |  | long-chain-fatty-acyl-CoA reductase activity |  | **-1.24** |
| 1422771_AT | gb:AF010133.1 /DB_XREF=gi:2507639 /GEN=mSmad6 /FEA |  | long-chain-fatty-acyl-CoA reductase activity |  | **1.91** |
| 1422778_AT | TAF9 RNA polymerase II, TATA box binding protein (TBP)-associated factor | [TAF9](http://www.gene.ucl.ac.uk/cgi-bin/nomenclature/searchgenes.pl?field=symbol&anchor=equals&symbol_search=Search&number=100&format=html&sortby=symbol&match=TAF9) | low-density lipoprotein receptor activity;protein binding;lipid transporter activity;calcium ion binding;receptor activity | [600822](http://www.ncbi.nlm.nih.gov/entrez/dispomim.cgi?id=600822) | **-1.31** |
| 1422780_AT | peroxisomal membrane protein 4 | [PXMP4](http://www.gene.ucl.ac.uk/cgi-bin/nomenclature/searchgenes.pl?field=symbol&anchor=equals&symbol_search=Search&number=100&format=html&sortby=symbol&match=PXMP4) | low-density lipoprotein receptor activity;scavenger receptor activity;chemokine activity;cytokine activity |  | **1.36** |
| 1422785_AT | gb:NM_053268.1 /DB_XREF=gi:16716600 /GEN=Rasa2 /FE |  | low-density lipoprotein receptor activity;scavenger receptor activity;chemokine activity;cytokine activity |  | **-1.47** |
| 1422789_AT | aldehyde dehydrogenase family 1, subfamily A2 | [ALDH1A2](http://www.gene.ucl.ac.uk/cgi-bin/nomenclature/searchgenes.pl?field=symbol&anchor=equals&symbol_search=Search&number=100&format=html&sortby=symbol&match=ALDH1A2) | lyase activity;carbon-carbon lyase activity;transferase activity | [603687](http://www.ncbi.nlm.nih.gov/entrez/dispomim.cgi?id=603687) | **1.82** |
| 1422795_AT | cullin 3 | [CUL3](http://www.gene.ucl.ac.uk/cgi-bin/nomenclature/searchgenes.pl?field=symbol&anchor=equals&symbol_search=Search&number=100&format=html&sortby=symbol&match=CUL3) | lyase activity;ferrochelatase activity | [603136](http://www.ncbi.nlm.nih.gov/entrez/dispomim.cgi?id=603136) | **1.59** |
| 1422804_AT | serine (or cysteine) proteinase inhibitor, clade B, member 6b | [SERPINB6B](http://www.gene.ucl.ac.uk/cgi-bin/nomenclature/searchgenes.pl?field=symbol&anchor=equals&symbol_search=Search&number=100&format=html&sortby=symbol&match=SERPINB6B) | lyase activity;ferrochelatase activity |  | **-1.85** |
| 1422811_AT | solute carrier family 27 (fatty acid transporter), member 1 | [SLC27A1](http://www.gene.ucl.ac.uk/cgi-bin/nomenclature/searchgenes.pl?field=symbol&anchor=equals&symbol_search=Search&number=100&format=html&sortby=symbol&match=SLC27A1) | lyase activity;lactoylglutathione lyase activity | [600691](http://www.ncbi.nlm.nih.gov/entrez/dispomim.cgi?id=600691) | **1.8** |
| 1422813_AT | gb:NM_007582.1 /DB_XREF=gi:6671657 /GEN=Cacng1 /FE |  | lyase activity;lactoylglutathione lyase activity |  | **-2.55** |
| 1422820_AT | lipase, hormone sensitive | [LIPE](http://www.gene.ucl.ac.uk/cgi-bin/nomenclature/searchgenes.pl?field=symbol&anchor=equals&symbol_search=Search&number=100&format=html&sortby=symbol&match=LIPE) | lysosphingolipid and lysophosphatidic acid receptor activity;G-protein coupled receptor activity;rhodopsin-like receptor activity | [151750](http://www.ncbi.nlm.nih.gov/entrez/dispomim.cgi?id=151750) | **1.75** |
| 1422848_A_AT | poly(A) binding protein, nuclear 1 | [PABPN1](http://www.gene.ucl.ac.uk/cgi-bin/nomenclature/searchgenes.pl?field=symbol&anchor=equals&symbol_search=Search&number=100&format=html&sortby=symbol&match=PABPN1) | lysozyme activity;catalytic activity;hydrolase activity, acting on glycosyl bonds;hydrolase activity | [602279](http://www.ncbi.nlm.nih.gov/entrez/dispomim.cgi?id=602279) | **-1.4** |
| 1422849_A_AT | poly(A) binding protein, nuclear 1 | [PABPN1](http://www.gene.ucl.ac.uk/cgi-bin/nomenclature/searchgenes.pl?field=symbol&anchor=equals&symbol_search=Search&number=100&format=html&sortby=symbol&match=PABPN1) | lysozyme activity;catalytic activity;hydrolase activity, acting on glycosyl bonds;hydrolase activity | [602279](http://www.ncbi.nlm.nih.gov/entrez/dispomim.cgi?id=602279) | **-1.63** |
| 1422864_AT | runt related transcription factor 1 | [RUNX1](http://www.gene.ucl.ac.uk/cgi-bin/nomenclature/searchgenes.pl?field=symbol&anchor=equals&symbol_search=Search&number=100&format=html&sortby=symbol&match=RUNX1) | lysozyme activity;hydrolase activity, acting on glycosyl bonds;catalytic activity;hydrolase activity | [151385](http://www.ncbi.nlm.nih.gov/entrez/dispomim.cgi?id=151385) | **-7.52** |
| 1422865_AT | runt related transcription factor 1 | [RUNX1](http://www.gene.ucl.ac.uk/cgi-bin/nomenclature/searchgenes.pl?field=symbol&anchor=equals&symbol_search=Search&number=100&format=html&sortby=symbol&match=RUNX1) | magnesium ion binding;farnesyl-diphosphate farnesyltransferase activity;oxidoreductase activity;catalytic activity;transferase activity | [151385](http://www.ncbi.nlm.nih.gov/entrez/dispomim.cgi?id=151385) | **-4.89** |
| 1422875_AT | CD84 antigen | [CD84](http://www.gene.ucl.ac.uk/cgi-bin/nomenclature/searchgenes.pl?field=symbol&anchor=equals&symbol_search=Search&number=100&format=html&sortby=symbol&match=CD84) | magnesium ion binding;farnesyl-diphosphate farnesyltransferase activity;oxidoreductase activity;catalytic activity;transferase activity | [604513](http://www.ncbi.nlm.nih.gov/entrez/dispomim.cgi?id=604513) | **-3.74** |
| 1422903_AT | lymphocyte antigen 86 | [LY86](http://www.gene.ucl.ac.uk/cgi-bin/nomenclature/searchgenes.pl?field=symbol&anchor=equals&symbol_search=Search&number=100&format=html&sortby=symbol&match=LY86) | magnesium ion binding;GTP binding | [605241](http://www.ncbi.nlm.nih.gov/entrez/dispomim.cgi?id=605241) | **-4.3** |
| 1422904_AT | flavin containing monooxygenase 2 | [FMO2](http://www.gene.ucl.ac.uk/cgi-bin/nomenclature/searchgenes.pl?field=symbol&anchor=equals&symbol_search=Search&number=100&format=html&sortby=symbol&match=FMO2) | magnesium ion binding;GTPase activity;motor activity;GTP binding | [603955](http://www.ncbi.nlm.nih.gov/entrez/dispomim.cgi?id=603955) | **1.76** |
| 1422908_AT | ATPase, (Na+)/K+ transporting, beta 4 polypeptide | [ATP1B4](http://www.gene.ucl.ac.uk/cgi-bin/nomenclature/searchgenes.pl?field=symbol&anchor=equals&symbol_search=Search&number=100&format=html&sortby=symbol&match=ATP1B4) | magnesium ion binding;GTPase activity;motor activity;GTP binding |  | **-3.09** |
| 1422919_AT | HRAS-like suppressor | [HRASLS](http://www.gene.ucl.ac.uk/cgi-bin/nomenclature/searchgenes.pl?field=symbol&anchor=equals&symbol_search=Search&number=100&format=html&sortby=symbol&match=HRASLS) | magnesium ion binding;lyase activity;6-pyruvoyltetrahydropterin synthase activity | [606487](http://www.ncbi.nlm.nih.gov/entrez/dispomim.cgi?id=606487) | **2.28** |
| 1422927_AT | RIKEN cDNA 2310016N21 gene | [2310016N21RIK](http://www.gene.ucl.ac.uk/cgi-bin/nomenclature/searchgenes.pl?field=symbol&anchor=equals&symbol_search=Search&number=100&format=html&sortby=symbol&match=2310016N21RIK) | magnesium ion binding;metallopeptidase activity;metalloendopeptidase activity;manganese ion binding;mitochondrial intermediate peptidase activity;hydrolase activity |  | **1.28** |
| 1422946_A_AT | DNA methyltransferase (cytosine-5) 1 | [DNMT1](http://www.gene.ucl.ac.uk/cgi-bin/nomenclature/searchgenes.pl?field=symbol&anchor=equals&symbol_search=Search&number=100&format=html&sortby=symbol&match=DNMT1) | magnesium ion binding;methionine adenosyltransferase activity;ATP binding;transferase activity | [126375](http://www.ncbi.nlm.nih.gov/entrez/dispomim.cgi?id=126375) | **-1.42** |
| 1422948_S_AT | histone 1, H4j | [HIST1H4C](http://www.gene.ucl.ac.uk/cgi-bin/nomenclature/searchgenes.pl?field=symbol&anchor=equals&symbol_search=Search&number=100&format=html&sortby=symbol&match=HIST1H4C) | magnesium ion binding;methionine adenosyltransferase activity;ATP binding;transferase activity |  | **1.66** |
| 1422949_AT | nitric oxide synthase 1, neuronal | [NOS1](http://www.gene.ucl.ac.uk/cgi-bin/nomenclature/searchgenes.pl?field=symbol&anchor=equals&symbol_search=Search&number=100&format=html&sortby=symbol&match=NOS1) | magnesium ion binding;methionine adenosyltransferase activity;ATP binding;transferase activity | [163731](http://www.ncbi.nlm.nih.gov/entrez/dispomim.cgi?id=163731) | **2.88** |
| 1422966_A_AT | transferrin receptor | [TFRC](http://www.gene.ucl.ac.uk/cgi-bin/nomenclature/searchgenes.pl?field=symbol&anchor=equals&symbol_search=Search&number=100&format=html&sortby=symbol&match=TFRC) | magnesium ion binding;methionine adenosyltransferase activity;ATP binding;transferase activity | [190010](http://www.ncbi.nlm.nih.gov/entrez/dispomim.cgi?id=190010) | **-2.52** |
| 1422973_A_AT | thyroid hormone responsive SPOT14 homolog (Rattus) | [THRSP](http://www.gene.ucl.ac.uk/cgi-bin/nomenclature/searchgenes.pl?field=symbol&anchor=equals&symbol_search=Search&number=100&format=html&sortby=symbol&match=THRSP) | magnesium ion binding;nucleoside-diphosphatase activity;hydrolase activity | [601926](http://www.ncbi.nlm.nih.gov/entrez/dispomim.cgi?id=601926) | **2.38** |
| 1422978_AT | cytochrome b-245, beta polypeptide | [CYBB](http://www.gene.ucl.ac.uk/cgi-bin/nomenclature/searchgenes.pl?field=symbol&anchor=equals&symbol_search=Search&number=100&format=html&sortby=symbol&match=CYBB) | magnesium ion binding;nucleoside-diphosphatase activity;hydrolase activity | [300481](http://www.ncbi.nlm.nih.gov/entrez/dispomim.cgi?id=300481) | **-2.17** |
| 1422986_AT | estrogen related receptor, beta | [ESRRB](http://www.gene.ucl.ac.uk/cgi-bin/nomenclature/searchgenes.pl?field=symbol&anchor=equals&symbol_search=Search&number=100&format=html&sortby=symbol&match=ESRRB) | magnesium ion binding;nucleoside-diphosphatase activity;hydrolase activity | [602167](http://www.ncbi.nlm.nih.gov/entrez/dispomim.cgi?id=602167) | **2.22** |
| 1422989_A_AT | gb:NM_032418.1 /DB_XREF=gi:14192944 /GEN=Dm15 /FEA |  | magnesium ion binding;nucleoside-diphosphatase activity;hydrolase activity |  | **1.63** |
| 1422996_AT | mitochondrial acyl-CoA thioesterase 1 | [MTE1](http://www.gene.ucl.ac.uk/cgi-bin/nomenclature/searchgenes.pl?field=symbol&anchor=equals&symbol_search=Search&number=100&format=html&sortby=symbol&match=MTE1) | magnesium ion binding;nucleoside-diphosphatase activity;hydrolase activity |  | **1.94** |
| 1422997_S_AT | mitochondrial acyl-CoA thioesterase 1 | [MTE1](http://www.gene.ucl.ac.uk/cgi-bin/nomenclature/searchgenes.pl?field=symbol&anchor=equals&symbol_search=Search&number=100&format=html&sortby=symbol&match=MTE1) | magnesium ion binding;nucleotidyltransferase activity;transferase activity |  | **2.04** |
| 1423025_A_AT | schwannomin interacting protein 1 | [SCHIP1](http://www.gene.ucl.ac.uk/cgi-bin/nomenclature/searchgenes.pl?field=symbol&anchor=equals&symbol_search=Search&number=100&format=html&sortby=symbol&match=SCHIP1) | malate dehydrogenase (oxaloacetate-decarboxylating) (NADP+) activity;malate dehydrogenase (decarboxylating) activity;malic enzyme activity;oxidoreductase activity |  | **1.25** |
| 1423035_S_AT | thioredoxin-like 5 | [TXNL5](http://www.gene.ucl.ac.uk/cgi-bin/nomenclature/searchgenes.pl?field=symbol&anchor=equals&symbol_search=Search&number=100&format=html&sortby=symbol&match=TXNL5) | malate dehydrogenase (oxaloacetate-decarboxylating) (NADP+) activity;malate dehydrogenase (decarboxylating) activity;malic enzyme activity;oxidoreductase activity |  | **-1.4** |
| 1423040_AT | basic leucine zipper and W2 domains 1 | [BZW1](http://www.gene.ucl.ac.uk/cgi-bin/nomenclature/searchgenes.pl?field=symbol&anchor=equals&symbol_search=Search&number=100&format=html&sortby=symbol&match=BZW1) | malate dehydrogenase (oxaloacetate-decarboxylating) activity;malate dehydrogenase (decarboxylating) activity;malic enzyme activity;electron transporter activity;oxidoreductase activity |  | **-1.39** |
| 1423041_A_AT | basic leucine zipper and W2 domains 1 | [BZW1](http://www.gene.ucl.ac.uk/cgi-bin/nomenclature/searchgenes.pl?field=symbol&anchor=equals&symbol_search=Search&number=100&format=html&sortby=symbol&match=BZW1) | malic enzyme activity;L-malate dehydrogenase activity;malate dehydrogenase activity;oxidoreductase activity |  | **-1.3** |
| 1423044_AT | gb:AV072100 /DB_XREF=gi:16380960 /DB_XREF=AV072100 |  | malic enzyme activity;L-malate dehydrogenase activity;malate dehydrogenase activity;oxidoreductase activity |  | **1.21** |
| 1423051_AT | heterogeneous nuclear ribonucleoprotein U | [HNRPU](http://www.gene.ucl.ac.uk/cgi-bin/nomenclature/searchgenes.pl?field=symbol&anchor=equals&symbol_search=Search&number=100&format=html&sortby=symbol&match=HNRPU) | malonyl-CoA decarboxylase activity;carboxy-lyase activity;methylmalonyl-CoA decarboxylase activity;lyase activity | [602869](http://www.ncbi.nlm.nih.gov/entrez/dispomim.cgi?id=602869) | **-1.27** |
| 1423052_AT | ADP-ribosylation factor 4 | [ARF4](http://www.gene.ucl.ac.uk/cgi-bin/nomenclature/searchgenes.pl?field=symbol&anchor=equals&symbol_search=Search&number=100&format=html&sortby=symbol&match=ARF4) | mannose binding;sugar binding | [601177](http://www.ncbi.nlm.nih.gov/entrez/dispomim.cgi?id=601177) | **-1.47** |
| 1423053_AT | ADP-ribosylation factor 4 | [ARF4](http://www.gene.ucl.ac.uk/cgi-bin/nomenclature/searchgenes.pl?field=symbol&anchor=equals&symbol_search=Search&number=100&format=html&sortby=symbol&match=ARF4) | mannose binding;sugar binding;receptor activity | [601177](http://www.ncbi.nlm.nih.gov/entrez/dispomim.cgi?id=601177) | **-1.33** |
| 1423059_AT | PTK2 protein tyrosine kinase 2 | [PTK2](http://www.gene.ucl.ac.uk/cgi-bin/nomenclature/searchgenes.pl?field=symbol&anchor=equals&symbol_search=Search&number=100&format=html&sortby=symbol&match=PTK2) | mannosyl-oligosaccharide 1,3-1,6-alpha-mannosidase activity;hydrolase activity, acting on glycosyl bonds;alpha-mannosidase activity;hydrolase activity | [600758](http://www.ncbi.nlm.nih.gov/entrez/dispomim.cgi?id=600758) | **1.25** |
| 1423060_AT | proliferation-associated 2G4 | [PA2G4](http://www.gene.ucl.ac.uk/cgi-bin/nomenclature/searchgenes.pl?field=symbol&anchor=equals&symbol_search=Search&number=100&format=html&sortby=symbol&match=PA2G4) | MAP kinase activity;kinase activity;protein kinase activity;protein serine/threonine kinase activity;MP kinase activity;protein-tyrosine kinase activity;ATP binding;transferase activity | [602145](http://www.ncbi.nlm.nih.gov/entrez/dispomim.cgi?id=602145) | **-1.26** |
| 1423068_AT | intraflagellar transport 172 | [IFT172](http://www.gene.ucl.ac.uk/cgi-bin/nomenclature/searchgenes.pl?field=symbol&anchor=equals&symbol_search=Search&number=100&format=html&sortby=symbol&match=IFT172) | MAP kinase activity;protein kinase activity;kinase activity;protein serine/threonine kinase activity;ATP binding;transferase activity |  | **1.27** |
| 1423071_X_AT | gb:AW549928 /DB_XREF=gi:7195356 /DB_XREF=L0057E09- |  | MAP kinase activity;protein kinase activity;kinase activity;protein serine/threonine kinase activity;protein-tyrosine kinase activity;ATP binding;MP kinase activity;transferase activity |  | **2.55** |
| 1423072_AT | RIKEN cDNA 6720475J19 gene | [6720475J19RIK](http://www.gene.ucl.ac.uk/cgi-bin/nomenclature/searchgenes.pl?field=symbol&anchor=equals&symbol_search=Search&number=100&format=html&sortby=symbol&match=6720475J19RIK) | melanin-concentrating hormone receptor activity;G-protein coupled receptor activity;rhodopsin-like receptor activity;protein-hormone receptor activity |  | **2.72** |
| 1423073_AT | RIKEN cDNA 0610011D08 gene | [0610011D08RIK](http://www.gene.ucl.ac.uk/cgi-bin/nomenclature/searchgenes.pl?field=symbol&anchor=equals&symbol_search=Search&number=100&format=html&sortby=symbol&match=0610011D08RIK) | metabotropic glutamate, GABA-B-like receptor activity;G-protein coupled receptor activity |  | **-1.41** |
| 1423082_AT | Der1-like domain family, member 1 | [DERL1](http://www.gene.ucl.ac.uk/cgi-bin/nomenclature/searchgenes.pl?field=symbol&anchor=equals&symbol_search=Search&number=100&format=html&sortby=symbol&match=DERL1) | metabotropic glutamate, GABA-B-like receptor activity;G-protein coupled receptor activity;sevenless binding | [608813](http://www.ncbi.nlm.nih.gov/entrez/dispomim.cgi?id=608813) | **-1.31** |
| 1423084_AT | UDP-Gal:betaGlcNAc beta 1,3-galactosyltransferase, polypeptide 2 | [B3GALT2](http://www.gene.ucl.ac.uk/cgi-bin/nomenclature/searchgenes.pl?field=symbol&anchor=equals&symbol_search=Search&number=100&format=html&sortby=symbol&match=B3GALT2) | metal ion binding;antioxidant activity;oxidoreductase activity;superoxide dismutase activity;copper, zinc superoxide dismutase activity | [603018](http://www.ncbi.nlm.nih.gov/entrez/dispomim.cgi?id=603018) | **-1.73** |
| 1423091_A_AT | glycoprotein m6b | [GPM6B](http://www.gene.ucl.ac.uk/cgi-bin/nomenclature/searchgenes.pl?field=symbol&anchor=equals&symbol_search=Search&number=100&format=html&sortby=symbol&match=GPM6B) | metal ion binding;copper, zinc superoxide dismutase activity | [300051](http://www.ncbi.nlm.nih.gov/entrez/dispomim.cgi?id=300051) | **-2.8** |
| 1423098_AT | calpain 7 | [CAPN7](http://www.gene.ucl.ac.uk/cgi-bin/nomenclature/searchgenes.pl?field=symbol&anchor=equals&symbol_search=Search&number=100&format=html&sortby=symbol&match=CAPN7) | metal ion binding;DNA binding;transcription factor activity | [606400](http://www.ncbi.nlm.nih.gov/entrez/dispomim.cgi?id=606400) | **1.28** |
| 1423101_AT | progestin and adipoQ receptor family member IV | [PAQR4](http://www.gene.ucl.ac.uk/cgi-bin/nomenclature/searchgenes.pl?field=symbol&anchor=equals&symbol_search=Search&number=100&format=html&sortby=symbol&match=PAQR4) | metal ion binding;manganese superoxide dismutase activity;manganese ion binding;oxidoreductase activity;superoxide dismutase activity |  | **1.4** |
| 1423106_AT | ubiquitin-conjugating enzyme E2B, RAD6 homology (S. cerevisiae) | [UBE2B](http://www.gene.ucl.ac.uk/cgi-bin/nomenclature/searchgenes.pl?field=symbol&anchor=equals&symbol_search=Search&number=100&format=html&sortby=symbol&match=UBE2B) | metal ion binding;manganese superoxide dismutase activity;manganese ion binding;oxidoreductase activity;superoxide dismutase activity | [179095](http://www.ncbi.nlm.nih.gov/entrez/dispomim.cgi?id=179095) | **1.58** |
| 1423107_AT | ubiquitin-conjugating enzyme E2B, RAD6 homology (S. cerevisiae) | [UBE2B](http://www.gene.ucl.ac.uk/cgi-bin/nomenclature/searchgenes.pl?field=symbol&anchor=equals&symbol_search=Search&number=100&format=html&sortby=symbol&match=UBE2B) | metal ion binding;manganese superoxide dismutase activity;manganese ion binding;oxidoreductase activity;superoxide dismutase activity | [179095](http://www.ncbi.nlm.nih.gov/entrez/dispomim.cgi?id=179095) | **1.36** |
| 1423108_AT | solute carrier family 25 (mitochondrial carnitine/acylcarnitine translocase), member 20 | [SLC25A20](http://www.gene.ucl.ac.uk/cgi-bin/nomenclature/searchgenes.pl?field=symbol&anchor=equals&symbol_search=Search&number=100&format=html&sortby=symbol&match=SLC25A20) | metal ion binding;metal ion transporter activity;copper, zinc superoxide dismutase activity | [212138](http://www.ncbi.nlm.nih.gov/entrez/dispomim.cgi?id=212138) | **2** |
| 1423109_S_AT | solute carrier family 25 (mitochondrial carnitine/acylcarnitine translocase), member 20 | [SLC25A20](http://www.gene.ucl.ac.uk/cgi-bin/nomenclature/searchgenes.pl?field=symbol&anchor=equals&symbol_search=Search&number=100&format=html&sortby=symbol&match=SLC25A20) | metal ion binding;oxidoreductase activity;antioxidant activity;superoxide dismutase activity;copper, zinc superoxide dismutase activity | [212138](http://www.ncbi.nlm.nih.gov/entrez/dispomim.cgi?id=212138) | **2.11** |
| 1423110_AT | procollagen, type I, alpha 2 | [COL1A2](http://www.gene.ucl.ac.uk/cgi-bin/nomenclature/searchgenes.pl?field=symbol&anchor=equals&symbol_search=Search&number=100&format=html&sortby=symbol&match=COL1A2) | metal ion binding;peptidoglycan glycosyltransferase activity;glucuronosyltransferase activity;acetylgalactosaminyltransferase activity;glucuronosyl-N-acetylgalactosaminyl-proteoglycan 4-beta-N-acetylgalactosaminyltransferase activity;glucuronylgalactosylproteoglycan 4-beta-N-acetylgalactosaminyltransferase activity;transferase activity | [120160](http://www.ncbi.nlm.nih.gov/entrez/dispomim.cgi?id=120160) | **-5.3** |
| 1423111_AT | ATP synthase, H+ transporting, mitochondrial F1 complex, alpha subunit, isoform 1 | [ATP5A1](http://www.gene.ucl.ac.uk/cgi-bin/nomenclature/searchgenes.pl?field=symbol&anchor=equals&symbol_search=Search&number=100&format=html&sortby=symbol&match=ATP5A1) | metal ion binding;phospholipid binding | [164360](http://www.ncbi.nlm.nih.gov/entrez/dispomim.cgi?id=164360) | **1.27** |
| 1423112_AT | ubiquitin-conjugating enzyme E2D 3 (UBC4/5 homolog, yeast) | [UBE2D3](http://www.gene.ucl.ac.uk/cgi-bin/nomenclature/searchgenes.pl?field=symbol&anchor=equals&symbol_search=Search&number=100&format=html&sortby=symbol&match=UBE2D3) | metal ion transporter activity | [602963](http://www.ncbi.nlm.nih.gov/entrez/dispomim.cgi?id=602963) | **1.4** |
| 1423114_AT | ubiquitin-conjugating enzyme E2D 3 (UBC4/5 homolog, yeast) | [UBE2D3](http://www.gene.ucl.ac.uk/cgi-bin/nomenclature/searchgenes.pl?field=symbol&anchor=equals&symbol_search=Search&number=100&format=html&sortby=symbol&match=UBE2D3) | metal ion transporter activity | [602963](http://www.ncbi.nlm.nih.gov/entrez/dispomim.cgi?id=602963) | **1.3** |
| 1423119_AT | radial spokehead-like 2 | [RSHL2](http://www.gene.ucl.ac.uk/cgi-bin/nomenclature/searchgenes.pl?field=symbol&anchor=equals&symbol_search=Search&number=100&format=html&sortby=symbol&match=RSHL2) | metal ion transporter activity |  | **1.23** |
| 1423122_AT | arginine vasopressin-induced 1 | [AVPI1](http://www.gene.ucl.ac.uk/cgi-bin/nomenclature/searchgenes.pl?field=symbol&anchor=equals&symbol_search=Search&number=100&format=html&sortby=symbol&match=AVPI1) | metalloendopeptidase activity;oxidoreductase activity;ubiquinol-cytochrome-c reductase activity |  | **-1.57** |
| 1423126_AT | ATPase, Na+/K+ transporting, beta 3 polypeptide | [ATP1B3](http://www.gene.ucl.ac.uk/cgi-bin/nomenclature/searchgenes.pl?field=symbol&anchor=equals&symbol_search=Search&number=100&format=html&sortby=symbol&match=ATP1B3) | metalloendopeptidase activity;oxidoreductase activity;ubiquinol-cytochrome-c reductase activity | [601867](http://www.ncbi.nlm.nih.gov/entrez/dispomim.cgi?id=601867) | **-1.41** |
| 1423127_AT | inositol (myo)-1(or 4)-monophosphatase 1 | [IMPA1](http://www.gene.ucl.ac.uk/cgi-bin/nomenclature/searchgenes.pl?field=symbol&anchor=equals&symbol_search=Search&number=100&format=html&sortby=symbol&match=IMPA1) | metalloendopeptidase inhibitor activity | [602064](http://www.ncbi.nlm.nih.gov/entrez/dispomim.cgi?id=602064) | **-1.26** |
| 1423132_A_AT | RIKEN cDNA 5730427N09 gene | [5730427N09RIK](http://www.gene.ucl.ac.uk/cgi-bin/nomenclature/searchgenes.pl?field=symbol&anchor=equals&symbol_search=Search&number=100&format=html&sortby=symbol&match=5730427N09RIK) | metalloendopeptidase inhibitor activity |  | **-1.47** |
| 1423134_AT | cDNA sequence BC003324 | [BC003324](http://www.gene.ucl.ac.uk/cgi-bin/nomenclature/searchgenes.pl?field=symbol&anchor=equals&symbol_search=Search&number=100&format=html&sortby=symbol&match=BC003324) | metalloendopeptidase inhibitor activity |  | **-1.32** |
| 1423136_AT | fibroblast growth factor 1 | [FGF1](http://www.gene.ucl.ac.uk/cgi-bin/nomenclature/searchgenes.pl?field=symbol&anchor=equals&symbol_search=Search&number=100&format=html&sortby=symbol&match=FGF1) | metalloendopeptidase inhibitor activity | [131220](http://www.ncbi.nlm.nih.gov/entrez/dispomim.cgi?id=131220) | **1.97** |
| 1423137_AT | v-ral simian leukemia viral oncogene homolog A (ras related) | [RALA](http://www.gene.ucl.ac.uk/cgi-bin/nomenclature/searchgenes.pl?field=symbol&anchor=equals&symbol_search=Search&number=100&format=html&sortby=symbol&match=RALA) | metalloendopeptidase inhibitor activity | [179550](http://www.ncbi.nlm.nih.gov/entrez/dispomim.cgi?id=179550) | **-1.29** |
| 1423141_AT | lysosomal acid lipase 1 | [LIP1](http://www.gene.ucl.ac.uk/cgi-bin/nomenclature/searchgenes.pl?field=symbol&anchor=equals&symbol_search=Search&number=100&format=html&sortby=symbol&match=LIP1) | metalloendopeptidase inhibitor activity;enzyme inhibitor activity |  | **-1.59** |
| 1423145_A_AT | titin-cap | [TCAP](http://www.gene.ucl.ac.uk/cgi-bin/nomenclature/searchgenes.pl?field=symbol&anchor=equals&symbol_search=Search&number=100&format=html&sortby=symbol&match=TCAP) | metallopeptidase activity;hydrolase activity;peptidase activity | [604488](http://www.ncbi.nlm.nih.gov/entrez/dispomim.cgi?id=604488) | **1.39** |
| 1423151_AT | DnaJ (Hsp40) homolog, subfamily B, member 11 | [DNAJB11](http://www.gene.ucl.ac.uk/cgi-bin/nomenclature/searchgenes.pl?field=symbol&anchor=equals&symbol_search=Search&number=100&format=html&sortby=symbol&match=DNAJB11) | metallopeptidase activity;metalloendopeptidase activity;nardilysin activity;hydrolase activity |  | **-1.58** |
| 1423159_AT | gb:AI647805 /DB_XREF=gi:4726483 /DB_XREF=uk43d04.x |  | metallopeptidase activity;metalloendopeptidase activity;peptidase activity |  | **1.27** |
| 1423160_AT | sprouty protein with EVH-1 domain 1, related sequence | [SPRED1](http://www.gene.ucl.ac.uk/cgi-bin/nomenclature/searchgenes.pl?field=symbol&anchor=equals&symbol_search=Search&number=100&format=html&sortby=symbol&match=SPRED1) | methionine adenosyltransferase activity;magnesium ion binding;ATP binding;transferase activity |  | **-1.66** |
| 1423161_S_AT | sprouty protein with EVH-1 domain 1, related sequence | [SPRED1](http://www.gene.ucl.ac.uk/cgi-bin/nomenclature/searchgenes.pl?field=symbol&anchor=equals&symbol_search=Search&number=100&format=html&sortby=symbol&match=SPRED1) | methionyl aminopeptidase activity;metalloexopeptidase activity;aminopeptidase activity;hydrolase activity;peptidase activity |  | **-1.57** |
| 1423162_S_AT | sprouty protein with EVH-1 domain 1, related sequence | [SPRED1](http://www.gene.ucl.ac.uk/cgi-bin/nomenclature/searchgenes.pl?field=symbol&anchor=equals&symbol_search=Search&number=100&format=html&sortby=symbol&match=SPRED1) | methylated-DNA-[protein]-cysteine S-methyltransferase activity;protein binding |  | **-1.58** |
| 1423166_AT | CD36 antigen | [CD36](http://www.gene.ucl.ac.uk/cgi-bin/nomenclature/searchgenes.pl?field=symbol&anchor=equals&symbol_search=Search&number=100&format=html&sortby=symbol&match=CD36) | methylenetetrahydrofolate dehydrogenase (NADP+) activity;magnesium ion binding;methenyltetrahydrofolate cyclohydrolase activity;oxidoreductase activity;catalytic activity;methylenetetrahydrofolate dehydrogenase (NAD+) activity;hydrolase activity | [173510](http://www.ncbi.nlm.nih.gov/entrez/dispomim.cgi?id=173510) | **1.8** |
| 1423179_AT | potassium voltage gated channel, Shab-related subfamily, member 1 | [KCNB1](http://www.gene.ucl.ac.uk/cgi-bin/nomenclature/searchgenes.pl?field=symbol&anchor=equals&symbol_search=Search&number=100&format=html&sortby=symbol&match=KCNB1) | methylenetetrahydrofolate dehydrogenase (NADP+) activity;magnesium ion binding;methenyltetrahydrofolate cyclohydrolase activity;oxidoreductase activity;catalytic activity;methylenetetrahydrofolate dehydrogenase (NAD+) activity;hydrolase activity | [600397](http://www.ncbi.nlm.nih.gov/entrez/dispomim.cgi?id=600397) | **1.44** |
| 1423181_S_AT | chloride channel, nucleotide-sensitive, 1A | [CLNS1A](http://www.gene.ucl.ac.uk/cgi-bin/nomenclature/searchgenes.pl?field=symbol&anchor=equals&symbol_search=Search&number=100&format=html&sortby=symbol&match=CLNS1A) | methylmalonate-semialdehyde dehydrogenase (acylating) activity;oxidoreductase activity | [602158](http://www.ncbi.nlm.nih.gov/entrez/dispomim.cgi?id=602158) | **-1.28** |
| 1423183_AT | leucine-rich repeat LGI family, member 1 | [LGI1](http://www.gene.ucl.ac.uk/cgi-bin/nomenclature/searchgenes.pl?field=symbol&anchor=equals&symbol_search=Search&number=100&format=html&sortby=symbol&match=LGI1) | methyltransferase activity;S-adenosylmethionine-dependent methyltransferase activity;transferase activity | [604619](http://www.ncbi.nlm.nih.gov/entrez/dispomim.cgi?id=604619) | **3.51** |
| 1423206_S_AT | RIKEN cDNA 2310003F16 gene | [2310003F16RIK](http://www.gene.ucl.ac.uk/cgi-bin/nomenclature/searchgenes.pl?field=symbol&anchor=equals&symbol_search=Search&number=100&format=html&sortby=symbol&match=2310003F16RIK) | methyltransferase activity;S-adenosylmethionine-dependent methyltransferase activity;transferase activity |  | **-1.25** |
| 1423213_AT | plexin C1 | [PLXNC1](http://www.gene.ucl.ac.uk/cgi-bin/nomenclature/searchgenes.pl?field=symbol&anchor=equals&symbol_search=Search&number=100&format=html&sortby=symbol&match=PLXNC1) | methyltransferase activity;S-adenosylmethionine-dependent methyltransferase activity;transferase activity | [604259](http://www.ncbi.nlm.nih.gov/entrez/dispomim.cgi?id=604259) | **-3.84** |
| 1423215_AT | RIKEN cDNA 5730406I15 gene | [5730406I15RIK](http://www.gene.ucl.ac.uk/cgi-bin/nomenclature/searchgenes.pl?field=symbol&anchor=equals&symbol_search=Search&number=100&format=html&sortby=symbol&match=5730406I15RIK) | methyltransferase activity;transferase activity |  | **-1.34** |
| 1423216_A_AT | RIKEN cDNA 2510049I19 gene | [2510049I19RIK](http://www.gene.ucl.ac.uk/cgi-bin/nomenclature/searchgenes.pl?field=symbol&anchor=equals&symbol_search=Search&number=100&format=html&sortby=symbol&match=2510049I19RIK) | methyltransferase activity;transferase activity |  | **-1.31** |
| 1423220_AT | eukaryotic translation initiation factor 4E | [EIF4E](http://www.gene.ucl.ac.uk/cgi-bin/nomenclature/searchgenes.pl?field=symbol&anchor=equals&symbol_search=Search&number=100&format=html&sortby=symbol&match=EIF4E) | methyltransferase activity;transferase activity | [133440](http://www.ncbi.nlm.nih.gov/entrez/dispomim.cgi?id=133440) | **1.65** |
| 1423223_A_AT | peroxiredoxin 6 | [PRDX6](http://www.gene.ucl.ac.uk/cgi-bin/nomenclature/searchgenes.pl?field=symbol&anchor=equals&symbol_search=Search&number=100&format=html&sortby=symbol&match=PRDX6) | MHC class I receptor activity | [602316](http://www.ncbi.nlm.nih.gov/entrez/dispomim.cgi?id=602316) | **1.52** |
| 1423234_AT | proteasome (prosome, macropain) 26S subunit, non-ATPase, 5 | [PSMD5](http://www.gene.ucl.ac.uk/cgi-bin/nomenclature/searchgenes.pl?field=symbol&anchor=equals&symbol_search=Search&number=100&format=html&sortby=symbol&match=PSMD5) | MHC class I receptor activity | [604452](http://www.ncbi.nlm.nih.gov/entrez/dispomim.cgi?id=604452) | **-1.36** |
| 1423253_AT | myelin protein zero | [MPZ](http://www.gene.ucl.ac.uk/cgi-bin/nomenclature/searchgenes.pl?field=symbol&anchor=equals&symbol_search=Search&number=100&format=html&sortby=symbol&match=MPZ) | MHC class I receptor activity | [159440](http://www.ncbi.nlm.nih.gov/entrez/dispomim.cgi?id=159440) | **2.72** |
| 1423264_AT | block of proliferation 1 | [BOP1](http://www.gene.ucl.ac.uk/cgi-bin/nomenclature/searchgenes.pl?field=symbol&anchor=equals&symbol_search=Search&number=100&format=html&sortby=symbol&match=BOP1) | MHC class I receptor activity;receptor activity |  | **-1.33** |
| 1423265_AT | multiple inositol polyphosphate histidine phosphatase 1 | [MINPP1](http://www.gene.ucl.ac.uk/cgi-bin/nomenclature/searchgenes.pl?field=symbol&anchor=equals&symbol_search=Search&number=100&format=html&sortby=symbol&match=MINPP1) | MHC class I receptor activity;receptor activity | [605391](http://www.ncbi.nlm.nih.gov/entrez/dispomim.cgi?id=605391) | **-1.39** |
| 1423267_S_AT | integrin alpha 5 (fibronectin receptor alpha) | [ITGA5](http://www.gene.ucl.ac.uk/cgi-bin/nomenclature/searchgenes.pl?field=symbol&anchor=equals&symbol_search=Search&number=100&format=html&sortby=symbol&match=ITGA5) | microtubule binding | [135620](http://www.ncbi.nlm.nih.gov/entrez/dispomim.cgi?id=135620) | **-1.82** |
| 1423274_AT | gb:BB458655 /DB_XREF=gi:16426294 /DB_XREF=BB458655 |  | microtubule motor activity;motor activity;ATP binding |  | **-3.59** |
| 1423281_AT | stathmin-like 2 | [STMN2](http://www.gene.ucl.ac.uk/cgi-bin/nomenclature/searchgenes.pl?field=symbol&anchor=equals&symbol_search=Search&number=100&format=html&sortby=symbol&match=STMN2) | microtubule motor activity;motor activity;ATP binding | [600621](http://www.ncbi.nlm.nih.gov/entrez/dispomim.cgi?id=600621) | **1.84** |
| 1423283_AT | phosphatidylinositol transfer protein | [PITPN](http://www.gene.ucl.ac.uk/cgi-bin/nomenclature/searchgenes.pl?field=symbol&anchor=equals&symbol_search=Search&number=100&format=html&sortby=symbol&match=PITPN) | microtubule motor activity;motor activity;ATP binding | [600174](http://www.ncbi.nlm.nih.gov/entrez/dispomim.cgi?id=600174) | **-1.22** |
| 1423294_AT | mesoderm specific transcript | [MEST](http://www.gene.ucl.ac.uk/cgi-bin/nomenclature/searchgenes.pl?field=symbol&anchor=equals&symbol_search=Search&number=100&format=html&sortby=symbol&match=MEST) | microtubule motor activity;motor activity;ATP binding | [601029](http://www.ncbi.nlm.nih.gov/entrez/dispomim.cgi?id=601029) | **-3.19** |
| 1423296_AT | proteasome (prosome, macropain) 26S subunit, non-ATPase, 8 | [PSMD8](http://www.gene.ucl.ac.uk/cgi-bin/nomenclature/searchgenes.pl?field=symbol&anchor=equals&symbol_search=Search&number=100&format=html&sortby=symbol&match=PSMD8) | mitochondrial processing peptidase activity;metallopeptidase activity;metalloendopeptidase activity;hydrolase activity |  | **-1.38** |
| 1423297_AT | adducin 3 (gamma) | [ADD3](http://www.gene.ucl.ac.uk/cgi-bin/nomenclature/searchgenes.pl?field=symbol&anchor=equals&symbol_search=Search&number=100&format=html&sortby=symbol&match=ADD3) | molecular_function unknown | [601568](http://www.ncbi.nlm.nih.gov/entrez/dispomim.cgi?id=601568) | **1.39** |
| 1423306_AT | RIKEN cDNA 2010002N04 gene | [2010002N04RIK](http://www.gene.ucl.ac.uk/cgi-bin/nomenclature/searchgenes.pl?field=symbol&anchor=equals&symbol_search=Search&number=100&format=html&sortby=symbol&match=2010002N04RIK) | molecular_function unknown |  | **-1.55** |
| 1423331_A_AT | poliovirus receptor-related 3 | [PVRL3](http://www.gene.ucl.ac.uk/cgi-bin/nomenclature/searchgenes.pl?field=symbol&anchor=equals&symbol_search=Search&number=100&format=html&sortby=symbol&match=PVRL3) | molecular_function unknown | [607147](http://www.ncbi.nlm.nih.gov/entrez/dispomim.cgi?id=607147) | **-1.52** |
| 1423332_AT | syndecan binding protein | [SDCBP](http://www.gene.ucl.ac.uk/cgi-bin/nomenclature/searchgenes.pl?field=symbol&anchor=equals&symbol_search=Search&number=100&format=html&sortby=symbol&match=SDCBP) | molecular_function unknown | [602217](http://www.ncbi.nlm.nih.gov/entrez/dispomim.cgi?id=602217) | **-1.64** |
| 1423346_AT | degenerative spermatocyte homolog (Drosophila) | [DEGS1](http://www.gene.ucl.ac.uk/cgi-bin/nomenclature/searchgenes.pl?field=symbol&anchor=equals&symbol_search=Search&number=100&format=html&sortby=symbol&match=DEGS1) | molecular_function unknown |  | **-1.27** |
| 1423359_AT | phospholamban | [PLN](http://www.gene.ucl.ac.uk/cgi-bin/nomenclature/searchgenes.pl?field=symbol&anchor=equals&symbol_search=Search&number=100&format=html&sortby=symbol&match=PLN) | molecular_function unknown | [172405](http://www.ncbi.nlm.nih.gov/entrez/dispomim.cgi?id=172405) | **1.73** |
| 1423362_AT | sortilin 1 | [SORT1](http://www.gene.ucl.ac.uk/cgi-bin/nomenclature/searchgenes.pl?field=symbol&anchor=equals&symbol_search=Search&number=100&format=html&sortby=symbol&match=SORT1) | molecular_function unknown | [602458](http://www.ncbi.nlm.nih.gov/entrez/dispomim.cgi?id=602458) | **-1.39** |
| 1423374_AT | nuclear receptor coactivator 6 | [NCOA6](http://www.gene.ucl.ac.uk/cgi-bin/nomenclature/searchgenes.pl?field=symbol&anchor=equals&symbol_search=Search&number=100&format=html&sortby=symbol&match=NCOA6) | molecular_function unknown | [605299](http://www.ncbi.nlm.nih.gov/entrez/dispomim.cgi?id=605299) | **-1.21** |
| 1423384_S_AT | testis expressed gene 261 | [TEX261](http://www.gene.ucl.ac.uk/cgi-bin/nomenclature/searchgenes.pl?field=symbol&anchor=equals&symbol_search=Search&number=100&format=html&sortby=symbol&match=TEX261) | molecular_function unknown |  | **1.26** |
| 1423389_AT | MAD homolog 7 (Drosophila) | [SMAD7](http://www.gene.ucl.ac.uk/cgi-bin/nomenclature/searchgenes.pl?field=symbol&anchor=equals&symbol_search=Search&number=100&format=html&sortby=symbol&match=SMAD7) | molecular_function unknown | [602932](http://www.ncbi.nlm.nih.gov/entrez/dispomim.cgi?id=602932) | **1.49** |
| 1423395_AT | translin-associated factor X | [TSNAX](http://www.gene.ucl.ac.uk/cgi-bin/nomenclature/searchgenes.pl?field=symbol&anchor=equals&symbol_search=Search&number=100&format=html&sortby=symbol&match=TSNAX) | molecular_function unknown | [602964](http://www.ncbi.nlm.nih.gov/entrez/dispomim.cgi?id=602964) | **1.32** |
| 1423399_A_AT | YY1 associated factor 2 | [YAF2](http://www.gene.ucl.ac.uk/cgi-bin/nomenclature/searchgenes.pl?field=symbol&anchor=equals&symbol_search=Search&number=100&format=html&sortby=symbol&match=YAF2) | molecular_function unknown | [607534](http://www.ncbi.nlm.nih.gov/entrez/dispomim.cgi?id=607534) | **1.35** |
| 1423405_AT | tissue inhibitor of metalloproteinase 4 | [TIMP4](http://www.gene.ucl.ac.uk/cgi-bin/nomenclature/searchgenes.pl?field=symbol&anchor=equals&symbol_search=Search&number=100&format=html&sortby=symbol&match=TIMP4) | molecular_function unknown | [601915](http://www.ncbi.nlm.nih.gov/entrez/dispomim.cgi?id=601915) | **2.18** |
| 1423407_A_AT | fibulin 2 | [FBLN2](http://www.gene.ucl.ac.uk/cgi-bin/nomenclature/searchgenes.pl?field=symbol&anchor=equals&symbol_search=Search&number=100&format=html&sortby=symbol&match=FBLN2) | molecular_function unknown | [135821](http://www.ncbi.nlm.nih.gov/entrez/dispomim.cgi?id=135821) | **1.56** |
| 1423413_AT | N-myc downstream regulated gene 1 | [NDRG1](http://www.gene.ucl.ac.uk/cgi-bin/nomenclature/searchgenes.pl?field=symbol&anchor=equals&symbol_search=Search&number=100&format=html&sortby=symbol&match=NDRG1) | molecular_function unknown | [605262](http://www.ncbi.nlm.nih.gov/entrez/dispomim.cgi?id=605262) | **1.46** |
| 1423416_AT | SWI/SNF related, matrix associated, actin dependent regulator of chromatin, subfamily c, member 1 | [SMARCC1](http://www.gene.ucl.ac.uk/cgi-bin/nomenclature/searchgenes.pl?field=symbol&anchor=equals&symbol_search=Search&number=100&format=html&sortby=symbol&match=SMARCC1) | molecular_function unknown | [601732](http://www.ncbi.nlm.nih.gov/entrez/dispomim.cgi?id=601732) | **-1.53** |
| 1423422_AT | ankyrin repeat and SOCS box-containing protein 4 | [ASB4](http://www.gene.ucl.ac.uk/cgi-bin/nomenclature/searchgenes.pl?field=symbol&anchor=equals&symbol_search=Search&number=100&format=html&sortby=symbol&match=ASB4) | molecular_function unknown | [605761](http://www.ncbi.nlm.nih.gov/entrez/dispomim.cgi?id=605761) | **2.13** |
| 1423423_AT | glucose regulated protein | [GRP58](http://www.gene.ucl.ac.uk/cgi-bin/nomenclature/searchgenes.pl?field=symbol&anchor=equals&symbol_search=Search&number=100&format=html&sortby=symbol&match=GRP58) | molecular_function unknown | [602046](http://www.ncbi.nlm.nih.gov/entrez/dispomim.cgi?id=602046) | **-1.52** |
| 1423430_AT | MYB binding protein (P160) 1a | [MYBBP1A](http://www.gene.ucl.ac.uk/cgi-bin/nomenclature/searchgenes.pl?field=symbol&anchor=equals&symbol_search=Search&number=100&format=html&sortby=symbol&match=MYBBP1A) | molecular_function unknown | [604885](http://www.ncbi.nlm.nih.gov/entrez/dispomim.cgi?id=604885) | **1.25** |
| 1423432_AT | Pleckstrin homology domain interacting protein | [PHIP](http://www.gene.ucl.ac.uk/cgi-bin/nomenclature/searchgenes.pl?field=symbol&anchor=equals&symbol_search=Search&number=100&format=html&sortby=symbol&match=PHIP) | molecular_function unknown |  | **-1.25** |
| 1423441_AT | transcription factor B2, mitochondrial | [TFB2M](http://www.gene.ucl.ac.uk/cgi-bin/nomenclature/searchgenes.pl?field=symbol&anchor=equals&symbol_search=Search&number=100&format=html&sortby=symbol&match=TFB2M) | molecular_function unknown | [607055](http://www.ncbi.nlm.nih.gov/entrez/dispomim.cgi?id=607055) | **1.34** |
| 1423444_AT | Rho-associated coiled-coil forming kinase 1 | [ROCK1](http://www.gene.ucl.ac.uk/cgi-bin/nomenclature/searchgenes.pl?field=symbol&anchor=equals&symbol_search=Search&number=100&format=html&sortby=symbol&match=ROCK1) | molecular_function unknown | [601702](http://www.ncbi.nlm.nih.gov/entrez/dispomim.cgi?id=601702) | **-1.43** |
| 1423452_AT | serine/threonine kinase 17b (apoptosis-inducing) | [STK17B](http://www.gene.ucl.ac.uk/cgi-bin/nomenclature/searchgenes.pl?field=symbol&anchor=equals&symbol_search=Search&number=100&format=html&sortby=symbol&match=STK17B) | molecular_function unknown | [604727](http://www.ncbi.nlm.nih.gov/entrez/dispomim.cgi?id=604727) | **-2.12** |
| 1423455_AT | prothymosin alpha | [PTMA](http://www.gene.ucl.ac.uk/cgi-bin/nomenclature/searchgenes.pl?field=symbol&anchor=equals&symbol_search=Search&number=100&format=html&sortby=symbol&match=PTMA) | molecular_function unknown | [188390](http://www.ncbi.nlm.nih.gov/entrez/dispomim.cgi?id=188390) | **-1.38** |
| 1423456_AT | basic leucine zipper and W2 domains 2 | [BZW2](http://www.gene.ucl.ac.uk/cgi-bin/nomenclature/searchgenes.pl?field=symbol&anchor=equals&symbol_search=Search&number=100&format=html&sortby=symbol&match=BZW2) | molecular_function unknown |  | **1.31** |
| 1423459_AT | COP9 (constitutive photomorphogenic) homolog, subunit 2 (Arabidopsis thaliana) | [COPS2](http://www.gene.ucl.ac.uk/cgi-bin/nomenclature/searchgenes.pl?field=symbol&anchor=equals&symbol_search=Search&number=100&format=html&sortby=symbol&match=COPS2) | molecular_function unknown | [604508](http://www.ncbi.nlm.nih.gov/entrez/dispomim.cgi?id=604508) | **-1.3** |
| 1423462_AT | mitogen-activated protein kinase kinase kinase 7 interacting protein 2 | [MAP3K7IP2](http://www.gene.ucl.ac.uk/cgi-bin/nomenclature/searchgenes.pl?field=symbol&anchor=equals&symbol_search=Search&number=100&format=html&sortby=symbol&match=MAP3K7IP2) | molecular_function unknown | [605101](http://www.ncbi.nlm.nih.gov/entrez/dispomim.cgi?id=605101) | **1.26** |
| 1423472_AT | septin 2 | [SEPT2](http://www.gene.ucl.ac.uk/cgi-bin/nomenclature/searchgenes.pl?field=symbol&anchor=equals&symbol_search=Search&number=100&format=html&sortby=symbol&match=SEPT2) | molecular_function unknown |  | **-1.2** |
| 1423474_AT | topoisomerase (DNA) I | [TOP1](http://www.gene.ucl.ac.uk/cgi-bin/nomenclature/searchgenes.pl?field=symbol&anchor=equals&symbol_search=Search&number=100&format=html&sortby=symbol&match=TOP1) | molecular_function unknown | [126420](http://www.ncbi.nlm.nih.gov/entrez/dispomim.cgi?id=126420) | **-1.34** |
| 1423484_AT | bicaudal C homolog 1 (Drosophila) | [BICC1](http://www.gene.ucl.ac.uk/cgi-bin/nomenclature/searchgenes.pl?field=symbol&anchor=equals&symbol_search=Search&number=100&format=html&sortby=symbol&match=BICC1) | molecular_function unknown |  | **-1.56** |
| 1423492_AT | mitochondrial ribosomal protein L45 | [MRPL45](http://www.gene.ucl.ac.uk/cgi-bin/nomenclature/searchgenes.pl?field=symbol&anchor=equals&symbol_search=Search&number=100&format=html&sortby=symbol&match=MRPL45) | molecular_function unknown |  | **1.28** |
| 1423505_AT | transgelin | [SIDT2](http://www.gene.ucl.ac.uk/cgi-bin/nomenclature/searchgenes.pl?field=symbol&anchor=equals&symbol_search=Search&number=100&format=html&sortby=symbol&match=SIDT2) | molecular_function unknown | [600818](http://www.ncbi.nlm.nih.gov/entrez/dispomim.cgi?id=600818) | **1.61** |
| 1423507_A_AT | sirtuin 2 (silent mating type information regulation 2, homolog) 2 (S. cerevisiae) | [SIRT2](http://www.gene.ucl.ac.uk/cgi-bin/nomenclature/searchgenes.pl?field=symbol&anchor=equals&symbol_search=Search&number=100&format=html&sortby=symbol&match=SIRT2) | molecular_function unknown | [604480](http://www.ncbi.nlm.nih.gov/entrez/dispomim.cgi?id=604480) | **1.23** |
| 1423531_A_AT | heterogeneous nuclear ribonucleoprotein A1 | [HNRPA1](http://www.gene.ucl.ac.uk/cgi-bin/nomenclature/searchgenes.pl?field=symbol&anchor=equals&symbol_search=Search&number=100&format=html&sortby=symbol&match=HNRPA1) | molecular_function unknown | [164017](http://www.ncbi.nlm.nih.gov/entrez/dispomim.cgi?id=164017) | **-1.49** |
| 1423540_AT | RNA binding motif, single stranded interacting protein 2 | [RBMS2](http://www.gene.ucl.ac.uk/cgi-bin/nomenclature/searchgenes.pl?field=symbol&anchor=equals&symbol_search=Search&number=100&format=html&sortby=symbol&match=RBMS2) | molecular_function unknown | [602387](http://www.ncbi.nlm.nih.gov/entrez/dispomim.cgi?id=602387) | **1.39** |
| 1423545_A_AT | zinc finger protein 207 | [ZFP207](http://www.gene.ucl.ac.uk/cgi-bin/nomenclature/searchgenes.pl?field=symbol&anchor=equals&symbol_search=Search&number=100&format=html&sortby=symbol&match=ZFP207) | molecular_function unknown |  | **-1.3** |
| 1423547_AT | lysozyme | [LYZS](http://www.gene.ucl.ac.uk/cgi-bin/nomenclature/searchgenes.pl?field=symbol&anchor=equals&symbol_search=Search&number=100&format=html&sortby=symbol&match=LYZS) | molecular_function unknown |  | **-3.99** |
| 1423551_AT | cadherin 13 | [CDH13](http://www.gene.ucl.ac.uk/cgi-bin/nomenclature/searchgenes.pl?field=symbol&anchor=equals&symbol_search=Search&number=100&format=html&sortby=symbol&match=CDH13) | molecular_function unknown | [601364](http://www.ncbi.nlm.nih.gov/entrez/dispomim.cgi?id=601364) | **-1.34** |
| 1423552_AT | leptin receptor overlapping transcript-like 1 | [LEPROTL1](http://www.gene.ucl.ac.uk/cgi-bin/nomenclature/searchgenes.pl?field=symbol&anchor=equals&symbol_search=Search&number=100&format=html&sortby=symbol&match=LEPROTL1) | molecular_function unknown | [607338](http://www.ncbi.nlm.nih.gov/entrez/dispomim.cgi?id=607338) | **-1.24** |
| 1423569_AT | glycine amidinotransferase (L-arginine:glycine amidinotransferase) | [GATM](http://www.gene.ucl.ac.uk/cgi-bin/nomenclature/searchgenes.pl?field=symbol&anchor=equals&symbol_search=Search&number=100&format=html&sortby=symbol&match=GATM) | molecular_function unknown | [602360](http://www.ncbi.nlm.nih.gov/entrez/dispomim.cgi?id=602360) | **-3.85** |
| 1423570_AT | ATP-binding cassette, sub-family G (WHITE), member 1 | [ABCG1](http://www.gene.ucl.ac.uk/cgi-bin/nomenclature/searchgenes.pl?field=symbol&anchor=equals&symbol_search=Search&number=100&format=html&sortby=symbol&match=ABCG1) | molecular_function unknown | [603076](http://www.ncbi.nlm.nih.gov/entrez/dispomim.cgi?id=603076) | **-1.56** |
| 1423571_AT | gb:BB133079 /DB_XREF=gi:8787469 /DB_XREF=BB133079 |  | molecular_function unknown |  | **1.91** |
| 1423583_AT | feminization 1 homolog a (C. elegans) | [FEM1A](http://www.gene.ucl.ac.uk/cgi-bin/nomenclature/searchgenes.pl?field=symbol&anchor=equals&symbol_search=Search&number=100&format=html&sortby=symbol&match=FEM1A) | molecular_function unknown |  | **1.44** |
| 1423586_AT | AXL receptor tyrosine kinase | [AXL](http://www.gene.ucl.ac.uk/cgi-bin/nomenclature/searchgenes.pl?field=symbol&anchor=equals&symbol_search=Search&number=100&format=html&sortby=symbol&match=AXL) | molecular_function unknown | [109135](http://www.ncbi.nlm.nih.gov/entrez/dispomim.cgi?id=109135) | **-1.21** |
| 1423593_A_AT | colony stimulating factor 1 receptor | [CSF1R](http://www.gene.ucl.ac.uk/cgi-bin/nomenclature/searchgenes.pl?field=symbol&anchor=equals&symbol_search=Search&number=100&format=html&sortby=symbol&match=CSF1R) | molecular_function unknown | [164770](http://www.ncbi.nlm.nih.gov/entrez/dispomim.cgi?id=164770) | **-1.43** |
| 1423596_AT | NIMA (never in mitosis gene a)-related expressed kinase 6 | [NEK6](http://www.gene.ucl.ac.uk/cgi-bin/nomenclature/searchgenes.pl?field=symbol&anchor=equals&symbol_search=Search&number=100&format=html&sortby=symbol&match=NEK6) | molecular_function unknown | [604884](http://www.ncbi.nlm.nih.gov/entrez/dispomim.cgi?id=604884) | **-1.82** |
| 1423599_A_AT | phosducin-like | [PDCL](http://www.gene.ucl.ac.uk/cgi-bin/nomenclature/searchgenes.pl?field=symbol&anchor=equals&symbol_search=Search&number=100&format=html&sortby=symbol&match=PDCL) | molecular_function unknown | [604421](http://www.ncbi.nlm.nih.gov/entrez/dispomim.cgi?id=604421) | **-1.43** |
| 1423600_A_AT | Treacher Collins Franceschetti syndrome 1, homolog | [TCOF1](http://www.gene.ucl.ac.uk/cgi-bin/nomenclature/searchgenes.pl?field=symbol&anchor=equals&symbol_search=Search&number=100&format=html&sortby=symbol&match=TCOF1) | molecular_function unknown | [606847](http://www.ncbi.nlm.nih.gov/entrez/dispomim.cgi?id=606847) | **1.35** |
| 1423605_A_AT | transformed mouse 3T3 cell double minute 2 | [MDM2](http://www.gene.ucl.ac.uk/cgi-bin/nomenclature/searchgenes.pl?field=symbol&anchor=equals&symbol_search=Search&number=100&format=html&sortby=symbol&match=MDM2) | molecular_function unknown | [164785](http://www.ncbi.nlm.nih.gov/entrez/dispomim.cgi?id=164785) | **-1.63** |
| 1423606_AT | periostin, osteoblast specific factor | [POSTN](http://www.gene.ucl.ac.uk/cgi-bin/nomenclature/searchgenes.pl?field=symbol&anchor=equals&symbol_search=Search&number=100&format=html&sortby=symbol&match=POSTN) | molecular_function unknown | [608777](http://www.ncbi.nlm.nih.gov/entrez/dispomim.cgi?id=608777) | **-15.27** |
| 1423607_AT | lumican | [LUM](http://www.gene.ucl.ac.uk/cgi-bin/nomenclature/searchgenes.pl?field=symbol&anchor=equals&symbol_search=Search&number=100&format=html&sortby=symbol&match=LUM) | molecular_function unknown | [600616](http://www.ncbi.nlm.nih.gov/entrez/dispomim.cgi?id=600616) | **-2.5** |
| 1423608_AT | integral membrane protein 2A | [ITM2A](http://www.gene.ucl.ac.uk/cgi-bin/nomenclature/searchgenes.pl?field=symbol&anchor=equals&symbol_search=Search&number=100&format=html&sortby=symbol&match=ITM2A) | molecular_function unknown | [300222](http://www.ncbi.nlm.nih.gov/entrez/dispomim.cgi?id=300222) | **-1.51** |
| 1423611_AT | alkaline phosphatase 2, liver | [AKP2](http://www.gene.ucl.ac.uk/cgi-bin/nomenclature/searchgenes.pl?field=symbol&anchor=equals&symbol_search=Search&number=100&format=html&sortby=symbol&match=AKP2) | molecular_function unknown |  | **1.87** |
| 1423614_AT | RIKEN cDNA E430036I04 gene | [E430036I04RIK](http://www.gene.ucl.ac.uk/cgi-bin/nomenclature/searchgenes.pl?field=symbol&anchor=equals&symbol_search=Search&number=100&format=html&sortby=symbol&match=E430036I04RIK) | molecular_function unknown |  | **1.79** |
| 1423617_AT | RIKEN cDNA 2610019N19 gene | [2610019N19RIK](http://www.gene.ucl.ac.uk/cgi-bin/nomenclature/searchgenes.pl?field=symbol&anchor=equals&symbol_search=Search&number=100&format=html&sortby=symbol&match=2610019N19RIK) | molecular_function unknown |  | **1.23** |
| 1423619_AT | RAS, dexamethasone-induced 1 | [RASD1](http://www.gene.ucl.ac.uk/cgi-bin/nomenclature/searchgenes.pl?field=symbol&anchor=equals&symbol_search=Search&number=100&format=html&sortby=symbol&match=RASD1) | molecular_function unknown | [605550](http://www.ncbi.nlm.nih.gov/entrez/dispomim.cgi?id=605550) | **3.37** |
| 1423625_A_AT | RIKEN cDNA 1810055D05 gene | [1810055D05RIK](http://www.gene.ucl.ac.uk/cgi-bin/nomenclature/searchgenes.pl?field=symbol&anchor=equals&symbol_search=Search&number=100&format=html&sortby=symbol&match=1810055D05RIK) | molecular_function unknown |  | **1.25** |
| 1423627_AT | NAD(P)H dehydrogenase, quinone 1 | [NQO1](http://www.gene.ucl.ac.uk/cgi-bin/nomenclature/searchgenes.pl?field=symbol&anchor=equals&symbol_search=Search&number=100&format=html&sortby=symbol&match=NQO1) | molecular_function unknown | [125860](http://www.ncbi.nlm.nih.gov/entrez/dispomim.cgi?id=125860) | **1.86** |
| 1423641_S_AT | CCR4-NOT transcription complex, subunit 7 | [CNOT7](http://www.gene.ucl.ac.uk/cgi-bin/nomenclature/searchgenes.pl?field=symbol&anchor=equals&symbol_search=Search&number=100&format=html&sortby=symbol&match=CNOT7) | molecular_function unknown | [604913](http://www.ncbi.nlm.nih.gov/entrez/dispomim.cgi?id=604913) | **1.23** |
| 1423643_AT | DEAD (Asp-Glu-Ala-Asp) box polypeptide 39 | [DDX39](http://www.gene.ucl.ac.uk/cgi-bin/nomenclature/searchgenes.pl?field=symbol&anchor=equals&symbol_search=Search&number=100&format=html&sortby=symbol&match=DDX39) | molecular_function unknown |  | **-1.62** |
| 1423649_AT | RIKEN cDNA 2010300G19 gene | [2010300G19RIK](http://www.gene.ucl.ac.uk/cgi-bin/nomenclature/searchgenes.pl?field=symbol&anchor=equals&symbol_search=Search&number=100&format=html&sortby=symbol&match=2010300G19RIK) | molecular_function unknown |  | **-1.29** |
| 1423651_AT | HESB like domain containing 2 | [HBLD2](http://www.gene.ucl.ac.uk/cgi-bin/nomenclature/searchgenes.pl?field=symbol&anchor=equals&symbol_search=Search&number=100&format=html&sortby=symbol&match=HBLD2) | molecular_function unknown |  | **1.43** |
| 1423652_AT | HESB like domain containing 2 | [HBLD2](http://www.gene.ucl.ac.uk/cgi-bin/nomenclature/searchgenes.pl?field=symbol&anchor=equals&symbol_search=Search&number=100&format=html&sortby=symbol&match=HBLD2) | molecular_function unknown |  | **1.7** |
| 1423654_A_AT | ring finger protein 4 | [RNF4](http://www.gene.ucl.ac.uk/cgi-bin/nomenclature/searchgenes.pl?field=symbol&anchor=equals&symbol_search=Search&number=100&format=html&sortby=symbol&match=RNF4) | molecular_function unknown | [602850](http://www.ncbi.nlm.nih.gov/entrez/dispomim.cgi?id=602850) | **-1.42** |
| 1423657_AT | CDP-diacylglycerol--inositol 3-phosphatidyltransferase (phosphatidylinositol synthase) | [CDIPT](http://www.gene.ucl.ac.uk/cgi-bin/nomenclature/searchgenes.pl?field=symbol&anchor=equals&symbol_search=Search&number=100&format=html&sortby=symbol&match=CDIPT) | molecular_function unknown | [605893](http://www.ncbi.nlm.nih.gov/entrez/dispomim.cgi?id=605893) | **-1.26** |
| 1423662_AT | ATPase, H+ transporting, lysosomal accessory protein 2 | [ATP6AP2](http://www.gene.ucl.ac.uk/cgi-bin/nomenclature/searchgenes.pl?field=symbol&anchor=equals&symbol_search=Search&number=100&format=html&sortby=symbol&match=ATP6AP2) | molecular_function unknown |  | **-1.59** |
| 1423666_S_AT | ribosomal protein L5 | [RPL5](http://www.gene.ucl.ac.uk/cgi-bin/nomenclature/searchgenes.pl?field=symbol&anchor=equals&symbol_search=Search&number=100&format=html&sortby=symbol&match=RPL5) | molecular_function unknown | [603634](http://www.ncbi.nlm.nih.gov/entrez/dispomim.cgi?id=603634) | **-1.21** |
| 1423669_AT | procollagen, type I, alpha 1 | [COL1A1](http://www.gene.ucl.ac.uk/cgi-bin/nomenclature/searchgenes.pl?field=symbol&anchor=equals&symbol_search=Search&number=100&format=html&sortby=symbol&match=COL1A1) | molecular_function unknown | [120150](http://www.ncbi.nlm.nih.gov/entrez/dispomim.cgi?id=120150) | **-4.58** |
| 1423670_A_AT | signal recognition particle receptor ('docking protein') | [SRPR](http://www.gene.ucl.ac.uk/cgi-bin/nomenclature/searchgenes.pl?field=symbol&anchor=equals&symbol_search=Search&number=100&format=html&sortby=symbol&match=SRPR) | molecular_function unknown | [182180](http://www.ncbi.nlm.nih.gov/entrez/dispomim.cgi?id=182180) | **1.25** |
| 1423672_AT | RIKEN cDNA 2510042P03 gene | [2510042P03RIK](http://www.gene.ucl.ac.uk/cgi-bin/nomenclature/searchgenes.pl?field=symbol&anchor=equals&symbol_search=Search&number=100&format=html&sortby=symbol&match=2510042P03RIK) | molecular_function unknown |  | **1.52** |
| 1423676_AT | ATP synthase, H+ transporting, mitochondrial F0 complex, subunit d | [ATP5H](http://www.gene.ucl.ac.uk/cgi-bin/nomenclature/searchgenes.pl?field=symbol&anchor=equals&symbol_search=Search&number=100&format=html&sortby=symbol&match=ATP5H) | molecular_function unknown |  | **1.24** |
| 1423682_A_AT | cell division cycle associated 4 | [CDCA4](http://www.gene.ucl.ac.uk/cgi-bin/nomenclature/searchgenes.pl?field=symbol&anchor=equals&symbol_search=Search&number=100&format=html&sortby=symbol&match=CDCA4) | molecular_function unknown |  | **-1.77** |
| 1423685_AT | alanyl-tRNA synthetase | [AARS](http://www.gene.ucl.ac.uk/cgi-bin/nomenclature/searchgenes.pl?field=symbol&anchor=equals&symbol_search=Search&number=100&format=html&sortby=symbol&match=AARS) | molecular_function unknown | [601065](http://www.ncbi.nlm.nih.gov/entrez/dispomim.cgi?id=601065) | **1.42** |
| 1423692_AT | NADH dehydrogenase (ubiquinone) 1 alpha subcomplex, 8 | [NDUFA8](http://www.gene.ucl.ac.uk/cgi-bin/nomenclature/searchgenes.pl?field=symbol&anchor=equals&symbol_search=Search&number=100&format=html&sortby=symbol&match=NDUFA8) | molecular_function unknown | [603359](http://www.ncbi.nlm.nih.gov/entrez/dispomim.cgi?id=603359) | **1.36** |
| 1423704_AT | lysophospholipase 3 | [LYPLA3](http://www.gene.ucl.ac.uk/cgi-bin/nomenclature/searchgenes.pl?field=symbol&anchor=equals&symbol_search=Search&number=100&format=html&sortby=symbol&match=LYPLA3) | molecular_function unknown |  | **-1.5** |
| 1423707_AT | RIKEN cDNA B230114J08 gene | [B230114J08RIK](http://www.gene.ucl.ac.uk/cgi-bin/nomenclature/searchgenes.pl?field=symbol&anchor=equals&symbol_search=Search&number=100&format=html&sortby=symbol&match=B230114J08RIK) | molecular_function unknown |  | **1.39** |
| 1423710_AT | dihydrolipoamide S-succinyltransferase (E2 component of 2-oxo-glutarate complex) | [DLST](http://www.gene.ucl.ac.uk/cgi-bin/nomenclature/searchgenes.pl?field=symbol&anchor=equals&symbol_search=Search&number=100&format=html&sortby=symbol&match=DLST) | molecular_function unknown | [126063](http://www.ncbi.nlm.nih.gov/entrez/dispomim.cgi?id=126063) | **1.32** |
| 1423711_AT | NADH dehydrogenase (ubiquinone) 1 alpha subcomplex, assembly factor 1 | [NDUFAF1](http://www.gene.ucl.ac.uk/cgi-bin/nomenclature/searchgenes.pl?field=symbol&anchor=equals&symbol_search=Search&number=100&format=html&sortby=symbol&match=NDUFAF1) | molecular_function unknown | [606934](http://www.ncbi.nlm.nih.gov/entrez/dispomim.cgi?id=606934) | **1.24** |
| 1423713_AT | RIKEN cDNA 4833412N02 gene | [ABCB8](http://www.gene.ucl.ac.uk/cgi-bin/nomenclature/searchgenes.pl?field=symbol&anchor=equals&symbol_search=Search&number=100&format=html&sortby=symbol&match=ABCB8) | molecular_function unknown | [605464](http://www.ncbi.nlm.nih.gov/entrez/dispomim.cgi?id=605464) | **1.49** |
| 1423716_S_AT | ATP synthase, H+ transporting, mitochondrial F1 complex, delta subunit | [ATP5D](http://www.gene.ucl.ac.uk/cgi-bin/nomenclature/searchgenes.pl?field=symbol&anchor=equals&symbol_search=Search&number=100&format=html&sortby=symbol&match=ATP5D) | molecular_function unknown | [603150](http://www.ncbi.nlm.nih.gov/entrez/dispomim.cgi?id=603150) | **1.24** |
| 1423717_AT | adenylate kinase 3 alpha-like 1 | [AK3L1](http://www.gene.ucl.ac.uk/cgi-bin/nomenclature/searchgenes.pl?field=symbol&anchor=equals&symbol_search=Search&number=100&format=html&sortby=symbol&match=AK3L1) | molecular_function unknown |  | **1.33** |
| 1423718_AT | adenylate kinase 3 alpha-like 1 | [AK3L1](http://www.gene.ucl.ac.uk/cgi-bin/nomenclature/searchgenes.pl?field=symbol&anchor=equals&symbol_search=Search&number=100&format=html&sortby=symbol&match=AK3L1) | molecular_function unknown |  | **1.56** |
| 1423722_AT | RIKEN cDNA 4930579A11 gene | [4930579A11RIK](http://www.gene.ucl.ac.uk/cgi-bin/nomenclature/searchgenes.pl?field=symbol&anchor=equals&symbol_search=Search&number=100&format=html&sortby=symbol&match=4930579A11RIK) | molecular_function unknown |  | **-1.46** |
| 1423723_S_AT | TAR DNA binding protein | [TARDBP](http://www.gene.ucl.ac.uk/cgi-bin/nomenclature/searchgenes.pl?field=symbol&anchor=equals&symbol_search=Search&number=100&format=html&sortby=symbol&match=TARDBP) | molecular_function unknown | [605078](http://www.ncbi.nlm.nih.gov/entrez/dispomim.cgi?id=605078) | **-1.42** |
| 1423726_AT | vesicle amine transport protein 1 homolog (T californica) | [VAT1](http://www.gene.ucl.ac.uk/cgi-bin/nomenclature/searchgenes.pl?field=symbol&anchor=equals&symbol_search=Search&number=100&format=html&sortby=symbol&match=VAT1) | molecular_function unknown | [604631](http://www.ncbi.nlm.nih.gov/entrez/dispomim.cgi?id=604631) | **-1.56** |
| 1423728_AT | eukaryotic translation initiation factor 3, subunit 6 interacting protein | [EIF3S6IP](http://www.gene.ucl.ac.uk/cgi-bin/nomenclature/searchgenes.pl?field=symbol&anchor=equals&symbol_search=Search&number=100&format=html&sortby=symbol&match=EIF3S6IP) | molecular_function unknown |  | **-1.26** |
| 1423730_AT | RIKEN cDNA C130052I12 gene | [C130052I12RIK](http://www.gene.ucl.ac.uk/cgi-bin/nomenclature/searchgenes.pl?field=symbol&anchor=equals&symbol_search=Search&number=100&format=html&sortby=symbol&match=C130052I12RIK) | molecular_function unknown |  | **-1.31** |
| 1423732_AT | translocating chain-associating membrane protein 1 | [TRAM1](http://www.gene.ucl.ac.uk/cgi-bin/nomenclature/searchgenes.pl?field=symbol&anchor=equals&symbol_search=Search&number=100&format=html&sortby=symbol&match=TRAM1) | molecular_function unknown | [605190](http://www.ncbi.nlm.nih.gov/entrez/dispomim.cgi?id=605190) | **-1.52** |
| 1423735_A_AT | WD repeat domain 36 | [WDR36](http://www.gene.ucl.ac.uk/cgi-bin/nomenclature/searchgenes.pl?field=symbol&anchor=equals&symbol_search=Search&number=100&format=html&sortby=symbol&match=WDR36) | molecular_function unknown |  | **-1.46** |
| 1423736_A_AT | RIKEN cDNA 4933427L07 gene | [4933427L07RIK](http://www.gene.ucl.ac.uk/cgi-bin/nomenclature/searchgenes.pl?field=symbol&anchor=equals&symbol_search=Search&number=100&format=html&sortby=symbol&match=4933427L07RIK) | molecular_function unknown |  | **2.02** |
| 1423737_AT | NADH dehydrogenase (ubiquinone) Fe-S protein 3 | [NDUFS3](http://www.gene.ucl.ac.uk/cgi-bin/nomenclature/searchgenes.pl?field=symbol&anchor=equals&symbol_search=Search&number=100&format=html&sortby=symbol&match=NDUFS3) | molecular_function unknown | [603846](http://www.ncbi.nlm.nih.gov/entrez/dispomim.cgi?id=603846) | **1.34** |
| 1423739_X_AT | gb:U15571.1 /DB_XREF=gi:558467 /GEN=APLP2 /FEA=FLm |  | molecular_function unknown |  | **1.24** |
| 1423740_A_AT | RNA binding motif protein 10 | [RBM10](http://www.gene.ucl.ac.uk/cgi-bin/nomenclature/searchgenes.pl?field=symbol&anchor=equals&symbol_search=Search&number=100&format=html&sortby=symbol&match=RBM10) | molecular_function unknown | [300080](http://www.ncbi.nlm.nih.gov/entrez/dispomim.cgi?id=300080) | **-1.36** |
| 1423746_AT | thioredoxin domain containing 5 | [TXNDC5](http://www.gene.ucl.ac.uk/cgi-bin/nomenclature/searchgenes.pl?field=symbol&anchor=equals&symbol_search=Search&number=100&format=html&sortby=symbol&match=TXNDC5) | molecular_function unknown | [607289](http://www.ncbi.nlm.nih.gov/entrez/dispomim.cgi?id=607289) | **1.43** |
| 1423747_A_AT | pyruvate dehydrogenase kinase, isoenzyme 1 | [PDK1](http://www.gene.ucl.ac.uk/cgi-bin/nomenclature/searchgenes.pl?field=symbol&anchor=equals&symbol_search=Search&number=100&format=html&sortby=symbol&match=PDK1) | molecular_function unknown | [602524](http://www.ncbi.nlm.nih.gov/entrez/dispomim.cgi?id=602524) | **1.6** |
| 1423748_AT | pyruvate dehydrogenase kinase, isoenzyme 1 | [PDK1](http://www.gene.ucl.ac.uk/cgi-bin/nomenclature/searchgenes.pl?field=symbol&anchor=equals&symbol_search=Search&number=100&format=html&sortby=symbol&match=PDK1) | molecular_function unknown | [602524](http://www.ncbi.nlm.nih.gov/entrez/dispomim.cgi?id=602524) | **1.75** |
| 1423754_AT | interferon induced transmembrane protein 3 | [IFITM3](http://www.gene.ucl.ac.uk/cgi-bin/nomenclature/searchgenes.pl?field=symbol&anchor=equals&symbol_search=Search&number=100&format=html&sortby=symbol&match=IFITM3) | molecular_function unknown | [605579](http://www.ncbi.nlm.nih.gov/entrez/dispomim.cgi?id=605579) | **-1.41** |
| 1423760_AT | CD44 antigen | [CD44](http://www.gene.ucl.ac.uk/cgi-bin/nomenclature/searchgenes.pl?field=symbol&anchor=equals&symbol_search=Search&number=100&format=html&sortby=symbol&match=CD44) | molecular_function unknown | [107269](http://www.ncbi.nlm.nih.gov/entrez/dispomim.cgi?id=107269) | **-4.06** |
| 1423762_AT | aarF domain containing kinase 1 | [ADCK1](http://www.gene.ucl.ac.uk/cgi-bin/nomenclature/searchgenes.pl?field=symbol&anchor=equals&symbol_search=Search&number=100&format=html&sortby=symbol&match=ADCK1) | molecular_function unknown |  | **1.55** |
| 1423772_X_AT | solute carrier family 25 (mitochondrial carrier, adenine nucleotide translocator), member 5 | [SLC25A5](http://www.gene.ucl.ac.uk/cgi-bin/nomenclature/searchgenes.pl?field=symbol&anchor=equals&symbol_search=Search&number=100&format=html&sortby=symbol&match=SLC25A5) | molecular_function unknown | [300150](http://www.ncbi.nlm.nih.gov/entrez/dispomim.cgi?id=300150) | **-2.08** |
| 1423777_AT | ubiquitin specific protease 20 | [USP20](http://www.gene.ucl.ac.uk/cgi-bin/nomenclature/searchgenes.pl?field=symbol&anchor=equals&symbol_search=Search&number=100&format=html&sortby=symbol&match=USP20) | molecular_function unknown |  | **-1.74** |
| 1423780_AT | 3-hydroxyisobutyrate dehydrogenase | [HIBADH](http://www.gene.ucl.ac.uk/cgi-bin/nomenclature/searchgenes.pl?field=symbol&anchor=equals&symbol_search=Search&number=100&format=html&sortby=symbol&match=HIBADH) | molecular_function unknown | [608475](http://www.ncbi.nlm.nih.gov/entrez/dispomim.cgi?id=608475) | **1.49** |
| 1423782_AT | gb:BC011285.1 /DB_XREF=gi:15277450 /FEA=FLmRNA /CN |  | molecular_function unknown |  | **-1.53** |
| 1423783_AT | torsin family 2, member A | [TOR2A](http://www.gene.ucl.ac.uk/cgi-bin/nomenclature/searchgenes.pl?field=symbol&anchor=equals&symbol_search=Search&number=100&format=html&sortby=symbol&match=TOR2A) | molecular_function unknown | [608052](http://www.ncbi.nlm.nih.gov/entrez/dispomim.cgi?id=608052) | **-1.36** |
| 1423785_AT | EGL nine homolog 1 (C. elegans) | [EGLN1](http://www.gene.ucl.ac.uk/cgi-bin/nomenclature/searchgenes.pl?field=symbol&anchor=equals&symbol_search=Search&number=100&format=html&sortby=symbol&match=EGLN1) | molecular_function unknown | [606425](http://www.ncbi.nlm.nih.gov/entrez/dispomim.cgi?id=606425) | **1.89** |
| 1423790_AT | death-associated protein | [DAP](http://www.gene.ucl.ac.uk/cgi-bin/nomenclature/searchgenes.pl?field=symbol&anchor=equals&symbol_search=Search&number=100&format=html&sortby=symbol&match=DAP) | molecular_function unknown | [600954](http://www.ncbi.nlm.nih.gov/entrez/dispomim.cgi?id=600954) | **-1.7** |
| 1423791_AT | IK cytokine | [IK](http://www.gene.ucl.ac.uk/cgi-bin/nomenclature/searchgenes.pl?field=symbol&anchor=equals&symbol_search=Search&number=100&format=html&sortby=symbol&match=IK) | molecular_function unknown | [600549](http://www.ncbi.nlm.nih.gov/entrez/dispomim.cgi?id=600549) | **-1.35** |
| 1423796_AT | splicing factor proline/glutamine rich (polypyrimidine tract binding protein associated) | [SFPQ](http://www.gene.ucl.ac.uk/cgi-bin/nomenclature/searchgenes.pl?field=symbol&anchor=equals&symbol_search=Search&number=100&format=html&sortby=symbol&match=SFPQ) | molecular_function unknown | [605199](http://www.ncbi.nlm.nih.gov/entrez/dispomim.cgi?id=605199) | **-1.57** |
| 1423799_AT | suppressor of initiator codon mutations, related sequence 1 (S. cerevisiae) | [SUI1-RS1](http://www.gene.ucl.ac.uk/cgi-bin/nomenclature/searchgenes.pl?field=symbol&anchor=equals&symbol_search=Search&number=100&format=html&sortby=symbol&match=SUI1-RS1) | molecular_function unknown |  | **1.39** |
| 1423819_S_AT | ADP-ribosylation factor-like 6 interacting protein 1 | [ARL6IP1](http://www.gene.ucl.ac.uk/cgi-bin/nomenclature/searchgenes.pl?field=symbol&anchor=equals&symbol_search=Search&number=100&format=html&sortby=symbol&match=ARL6IP1) | molecular_function unknown |  | **-1.37** |
| 1423828_AT | fatty acid synthase | [FASN](http://www.gene.ucl.ac.uk/cgi-bin/nomenclature/searchgenes.pl?field=symbol&anchor=equals&symbol_search=Search&number=100&format=html&sortby=symbol&match=FASN) | molecular_function unknown | [600212](http://www.ncbi.nlm.nih.gov/entrez/dispomim.cgi?id=600212) | **2.59** |
| 1423829_AT | RIKEN cDNA 0910001A06 gene | [0910001A06RIK](http://www.gene.ucl.ac.uk/cgi-bin/nomenclature/searchgenes.pl?field=symbol&anchor=equals&symbol_search=Search&number=100&format=html&sortby=symbol&match=0910001A06RIK) | molecular_function unknown |  | **-1.49** |
| 1423833_A_AT | RIKEN cDNA 2610205H19 gene | [2610205H19RIK](http://www.gene.ucl.ac.uk/cgi-bin/nomenclature/searchgenes.pl?field=symbol&anchor=equals&symbol_search=Search&number=100&format=html&sortby=symbol&match=2610205H19RIK) | molecular_function unknown |  | **1.34** |
| 1423838_S_AT | RIKEN cDNA 2400003C14 gene | [2400003C14RIK](http://www.gene.ucl.ac.uk/cgi-bin/nomenclature/searchgenes.pl?field=symbol&anchor=equals&symbol_search=Search&number=100&format=html&sortby=symbol&match=2400003C14RIK) | molecular_function unknown |  | **-1.21** |
| 1423846_X_AT | tubulin, alpha 2 | [TUBA2](http://www.gene.ucl.ac.uk/cgi-bin/nomenclature/searchgenes.pl?field=symbol&anchor=equals&symbol_search=Search&number=100&format=html&sortby=symbol&match=TUBA2) | molecular_function unknown | [602528](http://www.ncbi.nlm.nih.gov/entrez/dispomim.cgi?id=602528) | **-2.65** |
| 1423855_X_AT | ribosomal protein L17 | [RPL17](http://www.gene.ucl.ac.uk/cgi-bin/nomenclature/searchgenes.pl?field=symbol&anchor=equals&symbol_search=Search&number=100&format=html&sortby=symbol&match=RPL17) | molecular_function unknown | [603661](http://www.ncbi.nlm.nih.gov/entrez/dispomim.cgi?id=603661) | **-1.46** |
| 1423856_AT | popeye domain containing 3 | [POPDC3](http://www.gene.ucl.ac.uk/cgi-bin/nomenclature/searchgenes.pl?field=symbol&anchor=equals&symbol_search=Search&number=100&format=html&sortby=symbol&match=POPDC3) | molecular_function unknown | [605824](http://www.ncbi.nlm.nih.gov/entrez/dispomim.cgi?id=605824) | **-1.73** |
| 1423857_AT | mitochondrial ribosomal protein L30 | [MRPL30](http://www.gene.ucl.ac.uk/cgi-bin/nomenclature/searchgenes.pl?field=symbol&anchor=equals&symbol_search=Search&number=100&format=html&sortby=symbol&match=MRPL30) | molecular_function unknown |  | **1.26** |
| 1423859_A_AT | prostaglandin D2 synthase (brain) | [PTGDS](http://www.gene.ucl.ac.uk/cgi-bin/nomenclature/searchgenes.pl?field=symbol&anchor=equals&symbol_search=Search&number=100&format=html&sortby=symbol&match=PTGDS) | molecular_function unknown | [176803](http://www.ncbi.nlm.nih.gov/entrez/dispomim.cgi?id=176803) | **-4** |
| 1423865_AT | CDW92 antigen | [CDW92](http://www.gene.ucl.ac.uk/cgi-bin/nomenclature/searchgenes.pl?field=symbol&anchor=equals&symbol_search=Search&number=100&format=html&sortby=symbol&match=CDW92) | molecular_function unknown | [606105](http://www.ncbi.nlm.nih.gov/entrez/dispomim.cgi?id=606105) | **-1.25** |
| 1423872_A_AT | dystroglycan 1 | [DAG1](http://www.gene.ucl.ac.uk/cgi-bin/nomenclature/searchgenes.pl?field=symbol&anchor=equals&symbol_search=Search&number=100&format=html&sortby=symbol&match=DAG1) | molecular_function unknown | [128239](http://www.ncbi.nlm.nih.gov/entrez/dispomim.cgi?id=128239) | **-1.65** |
| 1423882_AT | cDNA sequence BC027246 | [BC027246](http://www.gene.ucl.ac.uk/cgi-bin/nomenclature/searchgenes.pl?field=symbol&anchor=equals&symbol_search=Search&number=100&format=html&sortby=symbol&match=BC027246) | molecular_function unknown;acyl-CoA binding |  | **-1.47** |
| 1423883_AT | Acyl-CoA synthetase long-chain family member 1 | [ACSL1](http://www.gene.ucl.ac.uk/cgi-bin/nomenclature/searchgenes.pl?field=symbol&anchor=equals&symbol_search=Search&number=100&format=html&sortby=symbol&match=ACSL1) | molecular_function unknown;DNA binding | [152425](http://www.ncbi.nlm.nih.gov/entrez/dispomim.cgi?id=152425) | **1.67** |
| 1423890_X_AT | ATPase, Na+/K+ transporting, beta 1 polypeptide | [ATP1B1](http://www.gene.ucl.ac.uk/cgi-bin/nomenclature/searchgenes.pl?field=symbol&anchor=equals&symbol_search=Search&number=100&format=html&sortby=symbol&match=ATP1B1) | molecular_function unknown;DNA binding;nucleic acid binding | [182330](http://www.ncbi.nlm.nih.gov/entrez/dispomim.cgi?id=182330) | **1.33** |
| 1423895_A_AT | CUG triplet repeat,RNA binding protein 2 | [CUGBP2](http://www.gene.ucl.ac.uk/cgi-bin/nomenclature/searchgenes.pl?field=symbol&anchor=equals&symbol_search=Search&number=100&format=html&sortby=symbol&match=CUGBP2) | molecular_function unknown;dolichyl-diphosphooligosaccharide-protein glycotransferase activity | [602538](http://www.ncbi.nlm.nih.gov/entrez/dispomim.cgi?id=602538) | **-1.48** |
| 1423898_A_AT | thyroid hormone receptor interactor 12 | [TRIP12](http://www.gene.ucl.ac.uk/cgi-bin/nomenclature/searchgenes.pl?field=symbol&anchor=equals&symbol_search=Search&number=100&format=html&sortby=symbol&match=TRIP12) | molecular_function unknown;GTP binding | [604506](http://www.ncbi.nlm.nih.gov/entrez/dispomim.cgi?id=604506) | **-1.23** |
| 1423903_AT | DNA segment, Chr 7, ERATO Doi 458, expressed | [D7ERTD458E](http://www.gene.ucl.ac.uk/cgi-bin/nomenclature/searchgenes.pl?field=symbol&anchor=equals&symbol_search=Search&number=100&format=html&sortby=symbol&match=D7ERTD458E) | molecular_function unknown;GTP binding |  | **-1.54** |
| 1423905_AT | DNA segment, Chr 7, ERATO Doi 458, expressed | [D7ERTD458E](http://www.gene.ucl.ac.uk/cgi-bin/nomenclature/searchgenes.pl?field=symbol&anchor=equals&symbol_search=Search&number=100&format=html&sortby=symbol&match=D7ERTD458E) | molecular_function unknown;ion channel activity |  | **-1.76** |
| 1423907_A_AT | NADH dehydrogenase (ubiquinone) Fe-S protein 8 | [NDUFS8](http://www.gene.ucl.ac.uk/cgi-bin/nomenclature/searchgenes.pl?field=symbol&anchor=equals&symbol_search=Search&number=100&format=html&sortby=symbol&match=NDUFS8) | molecular_function unknown;iron ion binding;electron transporter activity | [602141](http://www.ncbi.nlm.nih.gov/entrez/dispomim.cgi?id=602141) | **1.29** |
| 1423908_AT | NADH dehydrogenase (ubiquinone) Fe-S protein 8 | [NDUFS8](http://www.gene.ucl.ac.uk/cgi-bin/nomenclature/searchgenes.pl?field=symbol&anchor=equals&symbol_search=Search&number=100&format=html&sortby=symbol&match=NDUFS8) | molecular_function unknown;ligase activity;ubiquitin-protein ligase activity;ubiquitin conjugating enzyme activity | [602141](http://www.ncbi.nlm.nih.gov/entrez/dispomim.cgi?id=602141) | **1.27** |
| 1423909_AT | RIKEN cDNA 0610011I04 gene | [0610011I04RIK](http://www.gene.ucl.ac.uk/cgi-bin/nomenclature/searchgenes.pl?field=symbol&anchor=equals&symbol_search=Search&number=100&format=html&sortby=symbol&match=0610011I04RIK) | molecular_function unknown;nucleic acid binding |  | **-2.07** |
| 1423915_AT | olfactomedin-like 2B | [OLFML2B](http://www.gene.ucl.ac.uk/cgi-bin/nomenclature/searchgenes.pl?field=symbol&anchor=equals&symbol_search=Search&number=100&format=html&sortby=symbol&match=OLFML2B) | molecular_function unknown;nucleic acid binding |  | **-2.04** |
| 1423917_A_AT | gb:BC011434.1 /DB_XREF=gi:15030314 /FEA=FLmRNA /CN |  | molecular_function unknown;nucleic acid binding;DNA binding |  | **-1.56** |
| 1423930_AT | anaphase promoting complex subunit 4 | [ANAPC4](http://www.gene.ucl.ac.uk/cgi-bin/nomenclature/searchgenes.pl?field=symbol&anchor=equals&symbol_search=Search&number=100&format=html&sortby=symbol&match=ANAPC4) | molecular_function unknown;phosphoric monoester hydrolase activity | [606947](http://www.ncbi.nlm.nih.gov/entrez/dispomim.cgi?id=606947) | **-1.33** |
| 1423937_AT | potassium channel tetramerisation domain containing 5 | [KCTD5](http://www.gene.ucl.ac.uk/cgi-bin/nomenclature/searchgenes.pl?field=symbol&anchor=equals&symbol_search=Search&number=100&format=html&sortby=symbol&match=KCTD5) | molecular_function unknown;protein binding |  | **-1.36** |
| 1423947_AT | RIKEN cDNA 1110008P14 gene | [1110008P14RIK](http://www.gene.ucl.ac.uk/cgi-bin/nomenclature/searchgenes.pl?field=symbol&anchor=equals&symbol_search=Search&number=100&format=html&sortby=symbol&match=1110008P14RIK) | molecular_function unknown;protein binding |  | **-1.39** |
| 1423949_AT | DNA segment, Chr 11, KL Mohlke 35 | [D11MOH35](http://www.gene.ucl.ac.uk/cgi-bin/nomenclature/searchgenes.pl?field=symbol&anchor=equals&symbol_search=Search&number=100&format=html&sortby=symbol&match=D11MOH35) | molecular_function unknown;protein binding |  | **-1.22** |
| 1423955_A_AT | longevity assurance homolog 2 (S. cerevisiae) | [LASS2](http://www.gene.ucl.ac.uk/cgi-bin/nomenclature/searchgenes.pl?field=symbol&anchor=equals&symbol_search=Search&number=100&format=html&sortby=symbol&match=LASS2) | molecular_function unknown;protein binding | [606920](http://www.ncbi.nlm.nih.gov/entrez/dispomim.cgi?id=606920) | **-1.27** |
| 1423961_AT | WD repeat domain 26 | [WDR26](http://www.gene.ucl.ac.uk/cgi-bin/nomenclature/searchgenes.pl?field=symbol&anchor=equals&symbol_search=Search&number=100&format=html&sortby=symbol&match=WDR26) | molecular_function unknown;protein binding |  | **-1.25** |
| 1423972_AT | electron transferring flavoprotein, alpha polypeptide | [ETFA](http://www.gene.ucl.ac.uk/cgi-bin/nomenclature/searchgenes.pl?field=symbol&anchor=equals&symbol_search=Search&number=100&format=html&sortby=symbol&match=ETFA) | molecular_function unknown;protein transporter activity;hydrolase activity | [608053](http://www.ncbi.nlm.nih.gov/entrez/dispomim.cgi?id=608053) | **1.59** |
| 1423982_AT | FUS interacting protein (serine-arginine rich) 1 | [FUSIP1](http://www.gene.ucl.ac.uk/cgi-bin/nomenclature/searchgenes.pl?field=symbol&anchor=equals&symbol_search=Search&number=100&format=html&sortby=symbol&match=FUSIP1) | molecular_function unknown;receptor activity | [605221](http://www.ncbi.nlm.nih.gov/entrez/dispomim.cgi?id=605221) | **-1.3** |
| 1423985_AT | guanine nucleotide binding protein (G protein), gamma 5 subunit | [GNG5](http://www.gene.ucl.ac.uk/cgi-bin/nomenclature/searchgenes.pl?field=symbol&anchor=equals&symbol_search=Search&number=100&format=html&sortby=symbol&match=GNG5) | molecular_function unknown;receptor activity |  | **1.25** |
| 1423986_A_AT | scotin gene | [SCOTIN](http://www.gene.ucl.ac.uk/cgi-bin/nomenclature/searchgenes.pl?field=symbol&anchor=equals&symbol_search=Search&number=100&format=html&sortby=symbol&match=SCOTIN) | molecular_function unknown;signal peptidase activity;hydrolase activity;peptidase activity | [607290](http://www.ncbi.nlm.nih.gov/entrez/dispomim.cgi?id=607290) | **-1.4** |
| 1423990_AT | RAB28, member RAS oncogene family | [RAB28](http://www.gene.ucl.ac.uk/cgi-bin/nomenclature/searchgenes.pl?field=symbol&anchor=equals&symbol_search=Search&number=100&format=html&sortby=symbol&match=RAB28) | molecular_function unknown;signal peptidase activity;hydrolase activity;peptidase activity |  | **1.29** |
| 1423997_AT | DNA segment, Chr 3, MJeffers 1 | [D3JFR1](http://www.gene.ucl.ac.uk/cgi-bin/nomenclature/searchgenes.pl?field=symbol&anchor=equals&symbol_search=Search&number=100&format=html&sortby=symbol&match=D3JFR1) | molecular_function unknown;zinc ion binding;nucleic acid binding |  | **1.31** |
| 1424000_A_AT | ribosomal protein S11 | [RPS11](http://www.gene.ucl.ac.uk/cgi-bin/nomenclature/searchgenes.pl?field=symbol&anchor=equals&symbol_search=Search&number=100&format=html&sortby=symbol&match=RPS11) | molecular_function unknown;zinc ion binding;ubiquitin-protein ligase activity | [180471](http://www.ncbi.nlm.nih.gov/entrez/dispomim.cgi?id=180471) | **-1.29** |
| 1424002_AT | phosducin-like 3 | [PDCL3](http://www.gene.ucl.ac.uk/cgi-bin/nomenclature/searchgenes.pl?field=symbol&anchor=equals&symbol_search=Search&number=100&format=html&sortby=symbol&match=PDCL3) | molybdenum ion binding;aldehyde oxidase activity;electron transporter activity;oxidoreductase activity |  | **-1.22** |
| 1424006_AT | RIKEN cDNA 2310044P18 gene | [2310044P18RIK](http://www.gene.ucl.ac.uk/cgi-bin/nomenclature/searchgenes.pl?field=symbol&anchor=equals&symbol_search=Search&number=100&format=html&sortby=symbol&match=2310044P18RIK) | motor activity |  | **1.34** |
| 1424010_AT | microfibrillar-associated protein 4 | [MFAP4](http://www.gene.ucl.ac.uk/cgi-bin/nomenclature/searchgenes.pl?field=symbol&anchor=equals&symbol_search=Search&number=100&format=html&sortby=symbol&match=MFAP4) | motor activity | [600596](http://www.ncbi.nlm.nih.gov/entrez/dispomim.cgi?id=600596) | **-7.11** |
| 1424025_AT | cDNA sequence BC013529 | [BC013529](http://www.gene.ucl.ac.uk/cgi-bin/nomenclature/searchgenes.pl?field=symbol&anchor=equals&symbol_search=Search&number=100&format=html&sortby=symbol&match=BC013529) | motor activity;ATP binding |  | **1.46** |
| 1424026_S_AT | cDNA sequence BC013529 | [BC013529](http://www.gene.ucl.ac.uk/cgi-bin/nomenclature/searchgenes.pl?field=symbol&anchor=equals&symbol_search=Search&number=100&format=html&sortby=symbol&match=BC013529) | motor activity;microtubule motor activity |  | **1.4** |
| 1424039_AT | RIKEN cDNA 1810045K07 gene | [1810045K07RIK](http://www.gene.ucl.ac.uk/cgi-bin/nomenclature/searchgenes.pl?field=symbol&anchor=equals&symbol_search=Search&number=100&format=html&sortby=symbol&match=1810045K07RIK) | motor activity;microtubule motor activity |  | **1.23** |
| 1424041_S_AT | complement component 1, s subcomponent | [C1S](http://www.gene.ucl.ac.uk/cgi-bin/nomenclature/searchgenes.pl?field=symbol&anchor=equals&symbol_search=Search&number=100&format=html&sortby=symbol&match=C1S) | motor activity;microtubule motor activity;ATP binding | [120580](http://www.ncbi.nlm.nih.gov/entrez/dispomim.cgi?id=120580) | **-1.34** |
| 1424045_AT | RIKEN cDNA 5730437N04 gene | [5730437N04RIK](http://www.gene.ucl.ac.uk/cgi-bin/nomenclature/searchgenes.pl?field=symbol&anchor=equals&symbol_search=Search&number=100&format=html&sortby=symbol&match=5730437N04RIK) | mRNA binding;RNA binding |  | **1.32** |
| 1424048_A_AT | NAD(P)H:quinone oxidoreductase type 3, polypeptide A2 | [NQO3A2](http://www.gene.ucl.ac.uk/cgi-bin/nomenclature/searchgenes.pl?field=symbol&anchor=equals&symbol_search=Search&number=100&format=html&sortby=symbol&match=NQO3A2) | mRNA binding;RNA binding | [608341](http://www.ncbi.nlm.nih.gov/entrez/dispomim.cgi?id=608341) | **-1.27** |
| 1424052_AT | THAP domain containing 4 | [THAP4](http://www.gene.ucl.ac.uk/cgi-bin/nomenclature/searchgenes.pl?field=symbol&anchor=equals&symbol_search=Search&number=100&format=html&sortby=symbol&match=THAP4) | multidrug efflux pump activity |  | **1.37** |
| 1424064_AT | RAB1B, member RAS oncogene family | [RAB1B](http://www.gene.ucl.ac.uk/cgi-bin/nomenclature/searchgenes.pl?field=symbol&anchor=equals&symbol_search=Search&number=100&format=html&sortby=symbol&match=RAB1B) | myosin II binding;actin binding;GTP-Rho binding |  | **1.3** |
| 1424072_AT | RIKEN cDNA 2010107G23 gene | [2010107G23RIK](http://www.gene.ucl.ac.uk/cgi-bin/nomenclature/searchgenes.pl?field=symbol&anchor=equals&symbol_search=Search&number=100&format=html&sortby=symbol&match=2010107G23RIK) | N-acetylglucosamine-6-phosphate deacetylase activity;hydrolase activity |  | **1.79** |
| 1424078_S_AT | peroxisomal biogenesis factor 6 | [PEX6](http://www.gene.ucl.ac.uk/cgi-bin/nomenclature/searchgenes.pl?field=symbol&anchor=equals&symbol_search=Search&number=100&format=html&sortby=symbol&match=PEX6) | N-acetylglucosamine-6-sulfatase activity;hydrolase activity;sulfuric ester hydrolase activity | [601498](http://www.ncbi.nlm.nih.gov/entrez/dispomim.cgi?id=601498) | **1.4** |
| 1424092_AT | erythrocyte protein band 4.1 | [EPB4.1](http://www.gene.ucl.ac.uk/cgi-bin/nomenclature/searchgenes.pl?field=symbol&anchor=equals&symbol_search=Search&number=100&format=html&sortby=symbol&match=EPB4.1) | NAD(P)+ transhydrogenase (B-specific) activity;NAD(P) transhydrogenase activity;NAD(P)+ transhydrogenase (AB-specific) activity;oxidoreductase activity |  | **1.77** |
| 1424094_AT | NIMA (never in mitosis gene a)-related expressed kinase 9 | [NEK9](http://www.gene.ucl.ac.uk/cgi-bin/nomenclature/searchgenes.pl?field=symbol&anchor=equals&symbol_search=Search&number=100&format=html&sortby=symbol&match=NEK9) | NAD(P)+ transhydrogenase (B-specific) activity;NAD(P) transhydrogenase activity;NAD(P)+ transhydrogenase (AB-specific) activity;oxidoreductase activity |  | **1.36** |
| 1424099_AT | RIKEN cDNA 2310016C16 gene | [2310016C16RIK](http://www.gene.ucl.ac.uk/cgi-bin/nomenclature/searchgenes.pl?field=symbol&anchor=equals&symbol_search=Search&number=100&format=html&sortby=symbol&match=2310016C16RIK) | NAD(P)+ transhydrogenase (B-specific) activity;NAD(P)+ transhydrogenase (AB-specific) activity;NAD(P) transhydrogenase activity;oxidoreductase activity |  | **-2.28** |
| 1424106_AT | RIKEN cDNA 1200003C05 gene | [1200003C05RIK](http://www.gene.ucl.ac.uk/cgi-bin/nomenclature/searchgenes.pl?field=symbol&anchor=equals&symbol_search=Search&number=100&format=html&sortby=symbol&match=1200003C05RIK) | NAD(P)H dehydrogenase (quinone) activity;oxidoreductase activity |  | **-1.29** |
| 1424108_AT | glyoxalase 1 | [GLO1](http://www.gene.ucl.ac.uk/cgi-bin/nomenclature/searchgenes.pl?field=symbol&anchor=equals&symbol_search=Search&number=100&format=html&sortby=symbol&match=GLO1) | NADH dehydrogenase (ubiquinone) activity;calcium ion binding;cofactor binding;acyl carrier activity;oxidoreductase activity | [138750](http://www.ncbi.nlm.nih.gov/entrez/dispomim.cgi?id=138750) | **1.26** |
| 1424111_AT | insulin-like growth factor 2 receptor | [IGF2R](http://www.gene.ucl.ac.uk/cgi-bin/nomenclature/searchgenes.pl?field=symbol&anchor=equals&symbol_search=Search&number=100&format=html&sortby=symbol&match=IGF2R) | NADH dehydrogenase (ubiquinone) activity;calcium ion binding;cofactor binding;oxidoreductase activity;acyl carrier activity | [147280](http://www.ncbi.nlm.nih.gov/entrez/dispomim.cgi?id=147280) | **-1.68** |
| 1424112_AT | insulin-like growth factor 2 receptor | [IGF2R](http://www.gene.ucl.ac.uk/cgi-bin/nomenclature/searchgenes.pl?field=symbol&anchor=equals&symbol_search=Search&number=100&format=html&sortby=symbol&match=IGF2R) | NADH dehydrogenase (ubiquinone) activity;electron transporter activity;oxidoreductase activity;NADH dehydrogenase activity | [147280](http://www.ncbi.nlm.nih.gov/entrez/dispomim.cgi?id=147280) | **-1.3** |
| 1424115_AT | protein phosphatase 5, catalytic subunit | [PPP5C](http://www.gene.ucl.ac.uk/cgi-bin/nomenclature/searchgenes.pl?field=symbol&anchor=equals&symbol_search=Search&number=100&format=html&sortby=symbol&match=PPP5C) | NADH dehydrogenase (ubiquinone) activity;electron transporter activity;oxidoreductase activity;NADH dehydrogenase activity | [600658](http://www.ncbi.nlm.nih.gov/entrez/dispomim.cgi?id=600658) | **1.34** |
| 1424129_AT | RIKEN cDNA 1200003O06 gene | [1200003O06RIK](http://www.gene.ucl.ac.uk/cgi-bin/nomenclature/searchgenes.pl?field=symbol&anchor=equals&symbol_search=Search&number=100&format=html&sortby=symbol&match=1200003O06RIK) | NADH dehydrogenase (ubiquinone) activity;electron transporter activity;oxidoreductase activity;NADH dehydrogenase activity |  | **-1.43** |
| 1424131_AT | procollagen, type VI, alpha 3 | [COL6A3](http://www.gene.ucl.ac.uk/cgi-bin/nomenclature/searchgenes.pl?field=symbol&anchor=equals&symbol_search=Search&number=100&format=html&sortby=symbol&match=COL6A3) | NADH dehydrogenase (ubiquinone) activity;iron ion binding;electron transporter activity;oxidoreductase activity;electron carrier activity;oxidoreductase activity, acting on NADH or NADPH;NADH dehydrogenase activity | [120250](http://www.ncbi.nlm.nih.gov/entrez/dispomim.cgi?id=120250) | **-3.1** |
| 1424134_AT | RIKEN cDNA 4930470D19 gene | [4930470D19RIK](http://www.gene.ucl.ac.uk/cgi-bin/nomenclature/searchgenes.pl?field=symbol&anchor=equals&symbol_search=Search&number=100&format=html&sortby=symbol&match=4930470D19RIK) | NADH dehydrogenase (ubiquinone) activity;oxidoreductase activity |  | **-1.35** |
| 1424135_AT | RIKEN cDNA 4930470D19 gene | [4930470D19RIK](http://www.gene.ucl.ac.uk/cgi-bin/nomenclature/searchgenes.pl?field=symbol&anchor=equals&symbol_search=Search&number=100&format=html&sortby=symbol&match=4930470D19RIK) | NADH dehydrogenase (ubiquinone) activity;oxidoreductase activity;electron carrier activity;oxidoreductase activity, acting on NADH or NADPH;NADH dehydrogenase activity |  | **-1.45** |
| 1424143_A_AT | retroviral integration site 2 | [RIS2](http://www.gene.ucl.ac.uk/cgi-bin/nomenclature/searchgenes.pl?field=symbol&anchor=equals&symbol_search=Search&number=100&format=html&sortby=symbol&match=RIS2) | NADH dehydrogenase (ubiquinone) activity;oxidoreductase activity;NADH dehydrogenase activity |  | **-1.86** |
| 1424154_A_AT | RIKEN cDNA 0710001C05 gene | [HBLD1](http://www.gene.ucl.ac.uk/cgi-bin/nomenclature/searchgenes.pl?field=symbol&anchor=equals&symbol_search=Search&number=100&format=html&sortby=symbol&match=HBLD1) | NADH dehydrogenase (ubiquinone) activity;oxidoreductase activity;NADH dehydrogenase activity |  | **1.25** |
| 1424155_AT | fatty acid binding protein 4, adipocyte | [FABP4](http://www.gene.ucl.ac.uk/cgi-bin/nomenclature/searchgenes.pl?field=symbol&anchor=equals&symbol_search=Search&number=100&format=html&sortby=symbol&match=FABP4) | NADH dehydrogenase (ubiquinone) activity;oxidoreductase activity;NADH dehydrogenase activity | [600434](http://www.ncbi.nlm.nih.gov/entrez/dispomim.cgi?id=600434) | **2** |
| 1424156_AT | retinoblastoma-like 1 (p107) | [RBL1](http://www.gene.ucl.ac.uk/cgi-bin/nomenclature/searchgenes.pl?field=symbol&anchor=equals&symbol_search=Search&number=100&format=html&sortby=symbol&match=RBL1) | NADH dehydrogenase (ubiquinone) activity;oxidoreductase activity;NADH dehydrogenase activity | [116957](http://www.ncbi.nlm.nih.gov/entrez/dispomim.cgi?id=116957) | **-1.55** |
| 1424158_AT | EH-domain containing 2 | [EHD2](http://www.gene.ucl.ac.uk/cgi-bin/nomenclature/searchgenes.pl?field=symbol&anchor=equals&symbol_search=Search&number=100&format=html&sortby=symbol&match=EHD2) | NADH dehydrogenase (ubiquinone) activity;oxidoreductase activity;NADH dehydrogenase activity | [605890](http://www.ncbi.nlm.nih.gov/entrez/dispomim.cgi?id=605890) | **2.09** |
| 1424160_AT | asparagine-linked glycosylation 5 homolog (yeast, dolichyl-phosphate beta-glucosyltransferase) | [ALG5](http://www.gene.ucl.ac.uk/cgi-bin/nomenclature/searchgenes.pl?field=symbol&anchor=equals&symbol_search=Search&number=100&format=html&sortby=symbol&match=ALG5) | NADH dehydrogenase (ubiquinone) activity;oxidoreductase activity;NADH dehydrogenase activity | [604565](http://www.ncbi.nlm.nih.gov/entrez/dispomim.cgi?id=604565) | **-1.32** |
| 1424168_A_AT | gb:U10406.1 /DB_XREF=gi:500746 /FEA=FLmRNA /CNT=11 |  | NADH dehydrogenase (ubiquinone) activity;oxidoreductase activity;NADH dehydrogenase activity |  | **-1.21** |
| 1424172_AT | hydroxyacyl glutathione hydrolase | [HAGH](http://www.gene.ucl.ac.uk/cgi-bin/nomenclature/searchgenes.pl?field=symbol&anchor=equals&symbol_search=Search&number=100&format=html&sortby=symbol&match=HAGH) | NADH dehydrogenase (ubiquinone) activity;oxidoreductase activity;NADH dehydrogenase activity | [138760](http://www.ncbi.nlm.nih.gov/entrez/dispomim.cgi?id=138760) | **1.24** |
| 1424176_A_AT | annexin A4 | [ANXA4](http://www.gene.ucl.ac.uk/cgi-bin/nomenclature/searchgenes.pl?field=symbol&anchor=equals&symbol_search=Search&number=100&format=html&sortby=symbol&match=ANXA4) | NADH dehydrogenase (ubiquinone) activity;oxidoreductase activity;oxidoreductase activity, acting on NADH or NADPH | [106491](http://www.ncbi.nlm.nih.gov/entrez/dispomim.cgi?id=106491) | **-2.69** |
| 1424177_AT | RIKEN cDNA 1110001E17 gene | [TMEM38A](http://www.gene.ucl.ac.uk/cgi-bin/nomenclature/searchgenes.pl?field=symbol&anchor=equals&symbol_search=Search&number=100&format=html&sortby=symbol&match=TMEM38A) | NADH dehydrogenase (ubiquinone) activity;oxidoreductase activity;oxidoreductase activity, acting on NADH or NADPH |  | **1.29** |
| 1424178_AT | RIKEN cDNA 1110001E17 gene | [TMEM38A](http://www.gene.ucl.ac.uk/cgi-bin/nomenclature/searchgenes.pl?field=symbol&anchor=equals&symbol_search=Search&number=100&format=html&sortby=symbol&match=TMEM38A) | NADH dehydrogenase (ubiquinone) activity;oxidoreductase activity;oxidoreductase activity, acting on NADH or NADPH;NADH dehydrogenase activity |  | **1.6** |
| 1424179_AT | pleckstrin homology domain containing, family J member 1 | [PLEKHJ1](http://www.gene.ucl.ac.uk/cgi-bin/nomenclature/searchgenes.pl?field=symbol&anchor=equals&symbol_search=Search&number=100&format=html&sortby=symbol&match=PLEKHJ1) | NADH dehydrogenase (ubiquinone) activity;oxidoreductase activity;oxidoreductase activity, acting on NADH or NADPH;NADH dehydrogenase activity |  | **-1.3** |
| 1424180_A_AT | thyroid hormone receptor associated protein 4 | [THRAP4](http://www.gene.ucl.ac.uk/cgi-bin/nomenclature/searchgenes.pl?field=symbol&anchor=equals&symbol_search=Search&number=100&format=html&sortby=symbol&match=THRAP4) | NADH dehydrogenase (ubiquinone) activity;oxidoreductase activity;oxidoreductase activity, acting on NADH or NADPH;NADH dehydrogenase activity | [607000](http://www.ncbi.nlm.nih.gov/entrez/dispomim.cgi?id=607000) | **1.51** |
| 1424182_AT | acetyl-Coenzyme A acetyltransferase 1 | [ACAT1](http://www.gene.ucl.ac.uk/cgi-bin/nomenclature/searchgenes.pl?field=symbol&anchor=equals&symbol_search=Search&number=100&format=html&sortby=symbol&match=ACAT1) | NADH dehydrogenase (ubiquinone) activity;oxidoreductase activity;oxidoreductase activity, acting on NADH or NADPH;NADH dehydrogenase activity | [607809](http://www.ncbi.nlm.nih.gov/entrez/dispomim.cgi?id=607809) | **2.04** |
| 1424183_AT | acetyl-Coenzyme A acetyltransferase 1 | [ACAT1](http://www.gene.ucl.ac.uk/cgi-bin/nomenclature/searchgenes.pl?field=symbol&anchor=equals&symbol_search=Search&number=100&format=html&sortby=symbol&match=ACAT1) | natriuretic peptide receptor activity;protein kinase activity;protein self binding;receptor activity;guanylate cyclase activity;peptide receptor activity, G-protein coupled;lyase activity;ATP binding | [607809](http://www.ncbi.nlm.nih.gov/entrez/dispomim.cgi?id=607809) | **1.93** |
| 1424184_AT | acyl-Coenzyme A dehydrogenase, very long chain | [ACADVL](http://www.gene.ucl.ac.uk/cgi-bin/nomenclature/searchgenes.pl?field=symbol&anchor=equals&symbol_search=Search&number=100&format=html&sortby=symbol&match=ACADVL) | neurotransmitter receptor activity;ion channel activity;receptor activity;extracellular ligand-gated ion channel activity;nicotinic acetylcholine-activated cation-selective channel activity | [201475](http://www.ncbi.nlm.nih.gov/entrez/dispomim.cgi?id=201475) | **1.54** |
| 1424186_AT | RIKEN cDNA 2610001E17 gene | [2610001E17RIK](http://www.gene.ucl.ac.uk/cgi-bin/nomenclature/searchgenes.pl?field=symbol&anchor=equals&symbol_search=Search&number=100&format=html&sortby=symbol&match=2610001E17RIK) | neurotransmitter receptor activity;ion channel activity;receptor activity;extracellular ligand-gated ion channel activity;nicotinic acetylcholine-activated cation-selective channel activity |  | **-1.68** |
| 1424187_AT | RIKEN cDNA 2610001E17 gene | [2610001E17RIK](http://www.gene.ucl.ac.uk/cgi-bin/nomenclature/searchgenes.pl?field=symbol&anchor=equals&symbol_search=Search&number=100&format=html&sortby=symbol&match=2610001E17RIK) | neurotransmitter receptor activity;ion channel activity;receptor activity;extracellular ligand-gated ion channel activity;nicotinic acetylcholine-activated cation-selective channel activity |  | **-2.43** |
| 1424189_AT | phosphatidylinositol glycan, classC | [PIGC](http://www.gene.ucl.ac.uk/cgi-bin/nomenclature/searchgenes.pl?field=symbol&anchor=equals&symbol_search=Search&number=100&format=html&sortby=symbol&match=PIGC) | neurotransmitter receptor activity;ion channel activity;receptor activity;extracellular ligand-gated ion channel activity;nicotinic acetylcholine-activated cation-selective channel activity | [601730](http://www.ncbi.nlm.nih.gov/entrez/dispomim.cgi?id=601730) | **-1.23** |
| 1424194_AT | cDNA sequence BC025872 | [BC025872](http://www.gene.ucl.ac.uk/cgi-bin/nomenclature/searchgenes.pl?field=symbol&anchor=equals&symbol_search=Search&number=100&format=html&sortby=symbol&match=BC025872) | NF-kappaB binding;protein binding;nucleic acid binding;RNA binding |  | **1.27** |
| 1424210_AT | similar to Caenorhabditis elegans protein C42C1.9 | [SPFH1](http://www.gene.ucl.ac.uk/cgi-bin/nomenclature/searchgenes.pl?field=symbol&anchor=equals&symbol_search=Search&number=100&format=html&sortby=symbol&match=SPFH1) | NF-kappaB binding;protein binding;nucleic acid binding;RNA binding |  | **-1.36** |
| 1424215_AT | FUN14 domain containing 1 | [FUNDC1](http://www.gene.ucl.ac.uk/cgi-bin/nomenclature/searchgenes.pl?field=symbol&anchor=equals&symbol_search=Search&number=100&format=html&sortby=symbol&match=FUNDC1) | non-tyrosine kinase fibroblast growth factor receptor activity;protein binding;receptor activity |  | **1.32** |
| 1424219_AT | RIKEN cDNA A530065E19 gene | [A530065E19RIK](http://www.gene.ucl.ac.uk/cgi-bin/nomenclature/searchgenes.pl?field=symbol&anchor=equals&symbol_search=Search&number=100&format=html&sortby=symbol&match=A530065E19RIK) | non-tyrosine kinase fibroblast growth factor receptor activity;protein binding;receptor activity |  | **1.35** |
| 1424223_AT | RIKEN cDNA 1700020C11 gene | [1700020C11RIK](http://www.gene.ucl.ac.uk/cgi-bin/nomenclature/searchgenes.pl?field=symbol&anchor=equals&symbol_search=Search&number=100&format=html&sortby=symbol&match=1700020C11RIK) | Notch binding;calcium ion binding;growth factor activity |  | **1.71** |
| 1424224_AT | ankyrin repeat and SOCS box-containing protein 8 | [ASB8](http://www.gene.ucl.ac.uk/cgi-bin/nomenclature/searchgenes.pl?field=symbol&anchor=equals&symbol_search=Search&number=100&format=html&sortby=symbol&match=ASB8) | Notch binding;protein binding;calcium ion binding |  | **1.21** |
| 1424225_AT | ankyrin repeat and SOCS box-containing protein 8 | [ASB8](http://www.gene.ucl.ac.uk/cgi-bin/nomenclature/searchgenes.pl?field=symbol&anchor=equals&symbol_search=Search&number=100&format=html&sortby=symbol&match=ASB8) | nuclease activity;endonuclease activity;nucleic acid binding;magnesium ion binding;hydrolase activity |  | **1.34** |
| 1424226_AT | RIKEN cDNA 9030617O03 gene | [9030617O03RIK](http://www.gene.ucl.ac.uk/cgi-bin/nomenclature/searchgenes.pl?field=symbol&anchor=equals&symbol_search=Search&number=100&format=html&sortby=symbol&match=9030617O03RIK) | nuclease activity;endonuclease activity;nucleic acid binding;pancreatic ribonuclease activity;hydrolase activity |  | **2.29** |
| 1424231_S_AT | SEC15-like 1 (S. cerevisiae) | [SEC15L1](http://www.gene.ucl.ac.uk/cgi-bin/nomenclature/searchgenes.pl?field=symbol&anchor=equals&symbol_search=Search&number=100&format=html&sortby=symbol&match=SEC15L1) | nuclease activity;endonuclease activity;nucleic acid binding;pancreatic ribonuclease activity;hydrolase activity |  | **1.47** |
| 1424234_S_AT | mesenchyme homeobox 2 | [MEOX2](http://www.gene.ucl.ac.uk/cgi-bin/nomenclature/searchgenes.pl?field=symbol&anchor=equals&symbol_search=Search&number=100&format=html&sortby=symbol&match=MEOX2) | nuclease activity;kinase activity;DNA binding;protein kinase activity;protein serine/threonine kinase activity;ATP binding;protein-tyrosine kinase activity;transferase activity | [600535](http://www.ncbi.nlm.nih.gov/entrez/dispomim.cgi?id=600535) | **1.55** |
| 1424235_AT | ORM1-like 2 (S. cerevisiae) | [ORMDL2](http://www.gene.ucl.ac.uk/cgi-bin/nomenclature/searchgenes.pl?field=symbol&anchor=equals&symbol_search=Search&number=100&format=html&sortby=symbol&match=ORMDL2) | nuclease activity;magnesium ion binding;RNA binding;hydrolase activity;exonuclease activity |  | **-1.55** |
| 1424236_AT | RIKEN cDNA 1110003P22 gene | [1110003P22RIK](http://www.gene.ucl.ac.uk/cgi-bin/nomenclature/searchgenes.pl?field=symbol&anchor=equals&symbol_search=Search&number=100&format=html&sortby=symbol&match=1110003P22RIK) | nuclease activity;magnesium ion binding;RNA binding;hydrolase activity;exonuclease activity |  | **-1.31** |
| 1424238_AT | sirtuin 7 (silent mating type information regulation 2, homolog) 7 (S. cerevisiae) | [SIRT7](http://www.gene.ucl.ac.uk/cgi-bin/nomenclature/searchgenes.pl?field=symbol&anchor=equals&symbol_search=Search&number=100&format=html&sortby=symbol&match=SIRT7) | nucleic acid binding | [606212](http://www.ncbi.nlm.nih.gov/entrez/dispomim.cgi?id=606212) | **-1.28** |
| 1424239_AT | RIKEN cDNA 2310066E14 gene | [2310066E14RIK](http://www.gene.ucl.ac.uk/cgi-bin/nomenclature/searchgenes.pl?field=symbol&anchor=equals&symbol_search=Search&number=100&format=html&sortby=symbol&match=2310066E14RIK) | nucleic acid binding |  | **1.27** |
| 1424248_AT | RIKEN cDNA 0710001E13 gene | [0710001E13RIK](http://www.gene.ucl.ac.uk/cgi-bin/nomenclature/searchgenes.pl?field=symbol&anchor=equals&symbol_search=Search&number=100&format=html&sortby=symbol&match=0710001E13RIK) | nucleic acid binding |  | **-5.59** |
| 1424252_AT | heterogeneous nuclear ribonucleoprotein D-like | [HNRPDL](http://www.gene.ucl.ac.uk/cgi-bin/nomenclature/searchgenes.pl?field=symbol&anchor=equals&symbol_search=Search&number=100&format=html&sortby=symbol&match=HNRPDL) | nucleic acid binding | [607137](http://www.ncbi.nlm.nih.gov/entrez/dispomim.cgi?id=607137) | **-1.36** |
| 1424253_AT | RIKEN cDNA 1810073G14 gene | [1810073G14RIK](http://www.gene.ucl.ac.uk/cgi-bin/nomenclature/searchgenes.pl?field=symbol&anchor=equals&symbol_search=Search&number=100&format=html&sortby=symbol&match=1810073G14RIK) | nucleic acid binding |  | **1.28** |
| 1424263_AT | RIKEN cDNA 2810003C17 gene | [2810003C17RIK](http://www.gene.ucl.ac.uk/cgi-bin/nomenclature/searchgenes.pl?field=symbol&anchor=equals&symbol_search=Search&number=100&format=html&sortby=symbol&match=2810003C17RIK) | nucleic acid binding |  | **-3.74** |
| 1424269_A_AT | myosin, light polypeptide 6, alkali, smooth muscle and non-muscle | [MYL6](http://www.gene.ucl.ac.uk/cgi-bin/nomenclature/searchgenes.pl?field=symbol&anchor=equals&symbol_search=Search&number=100&format=html&sortby=symbol&match=MYL6) | nucleic acid binding |  | **-1.45** |
| 1424270_AT | Double cortin and calcium/calmodulin-dependent protein kinase-like 1 | [DCAMKL1](http://www.gene.ucl.ac.uk/cgi-bin/nomenclature/searchgenes.pl?field=symbol&anchor=equals&symbol_search=Search&number=100&format=html&sortby=symbol&match=DCAMKL1) | nucleic acid binding | [604742](http://www.ncbi.nlm.nih.gov/entrez/dispomim.cgi?id=604742) | **-22.86** |
| 1424271_AT | double cortin and calcium/calmodulin-dependent protein kinase-like 1 | [DCAMKL1](http://www.gene.ucl.ac.uk/cgi-bin/nomenclature/searchgenes.pl?field=symbol&anchor=equals&symbol_search=Search&number=100&format=html&sortby=symbol&match=DCAMKL1) | nucleic acid binding | [604742](http://www.ncbi.nlm.nih.gov/entrez/dispomim.cgi?id=604742) | **-8.89** |
| 1424275_S_AT | tripartite motif-containing 41 | [TRIM41](http://www.gene.ucl.ac.uk/cgi-bin/nomenclature/searchgenes.pl?field=symbol&anchor=equals&symbol_search=Search&number=100&format=html&sortby=symbol&match=TRIM41) | nucleic acid binding |  | **-1.26** |
| 1424282_AT | PET112-like (yeast) | [PET112L](http://www.gene.ucl.ac.uk/cgi-bin/nomenclature/searchgenes.pl?field=symbol&anchor=equals&symbol_search=Search&number=100&format=html&sortby=symbol&match=PET112L) | nucleic acid binding | [603645](http://www.ncbi.nlm.nih.gov/entrez/dispomim.cgi?id=603645) | **1.44** |
| 1424285_S_AT | ADP-ribosylation factor-like 6 interacting protein 4 | [ARL6IP4](http://www.gene.ucl.ac.uk/cgi-bin/nomenclature/searchgenes.pl?field=symbol&anchor=equals&symbol_search=Search&number=100&format=html&sortby=symbol&match=ARL6IP4) | nucleic acid binding | [607668](http://www.ncbi.nlm.nih.gov/entrez/dispomim.cgi?id=607668) | **-1.42** |
| 1424289_AT | cDNA sequence BC010311 | [BC010311](http://www.gene.ucl.ac.uk/cgi-bin/nomenclature/searchgenes.pl?field=symbol&anchor=equals&symbol_search=Search&number=100&format=html&sortby=symbol&match=BC010311) | nucleic acid binding |  | **-1.4** |
| 1424290_AT | cDNA sequence BC010311 | [BC010311](http://www.gene.ucl.ac.uk/cgi-bin/nomenclature/searchgenes.pl?field=symbol&anchor=equals&symbol_search=Search&number=100&format=html&sortby=symbol&match=BC010311) | nucleic acid binding |  | **-1.53** |
| 1424291_AT | nucleoporin 93 | [NUP93](http://www.gene.ucl.ac.uk/cgi-bin/nomenclature/searchgenes.pl?field=symbol&anchor=equals&symbol_search=Search&number=100&format=html&sortby=symbol&match=NUP93) | nucleic acid binding |  | **-1.26** |
| 1424293_S_AT | RIKEN cDNA 2610319K07 gene | [2610319K07RIK](http://www.gene.ucl.ac.uk/cgi-bin/nomenclature/searchgenes.pl?field=symbol&anchor=equals&symbol_search=Search&number=100&format=html&sortby=symbol&match=2610319K07RIK) | nucleic acid binding |  | **-1.21** |
| 1424299_AT | RIKEN cDNA 2010001O09 gene | [OMA1](http://www.gene.ucl.ac.uk/cgi-bin/nomenclature/searchgenes.pl?field=symbol&anchor=equals&symbol_search=Search&number=100&format=html&sortby=symbol&match=OMA1) | nucleic acid binding |  | **1.57** |
| 1424302_AT | paired-Ig-like receptor B | [PIRB](http://www.gene.ucl.ac.uk/cgi-bin/nomenclature/searchgenes.pl?field=symbol&anchor=equals&symbol_search=Search&number=100&format=html&sortby=symbol&match=PIRB) | nucleic acid binding |  | **-12.47** |
| 1424313_A_AT | gb:BC013503.1 /DB_XREF=gi:15488720 /FEA=FLmRNA /CN |  | nucleic acid binding |  | **1.41** |
| 1424318_AT | RIKEN cDNA 1110067D22 gene | [1110067D22RIK](http://www.gene.ucl.ac.uk/cgi-bin/nomenclature/searchgenes.pl?field=symbol&anchor=equals&symbol_search=Search&number=100&format=html&sortby=symbol&match=1110067D22RIK) | nucleic acid binding |  | **2.13** |
| 1424321_AT | replication factor C (activator 1) 4 | [RFC4](http://www.gene.ucl.ac.uk/cgi-bin/nomenclature/searchgenes.pl?field=symbol&anchor=equals&symbol_search=Search&number=100&format=html&sortby=symbol&match=RFC4) | nucleic acid binding;ATPase activity;ATP-binding cassette (ABC) transporter activity;ATPase activity, coupled to transmembrane movement of substances;ATP binding | [102577](http://www.ncbi.nlm.nih.gov/entrez/dispomim.cgi?id=102577) | **1.37** |
| 1424343_A_AT | eukaryotic translation initiation factor 1A | [EIF1A](http://www.gene.ucl.ac.uk/cgi-bin/nomenclature/searchgenes.pl?field=symbol&anchor=equals&symbol_search=Search&number=100&format=html&sortby=symbol&match=EIF1A) | nucleic acid binding;ATP-dependent helicase activity;calcium ion binding;ATP binding | [300186](http://www.ncbi.nlm.nih.gov/entrez/dispomim.cgi?id=300186) | **-2.18** |
| 1424347_AT | protein phosphatase 6, catalytic subunit | [PPP6C](http://www.gene.ucl.ac.uk/cgi-bin/nomenclature/searchgenes.pl?field=symbol&anchor=equals&symbol_search=Search&number=100&format=html&sortby=symbol&match=PPP6C) | nucleic acid binding;DNA binding | [300141](http://www.ncbi.nlm.nih.gov/entrez/dispomim.cgi?id=300141) | **1.23** |
| 1424353_AT | leucine-rich PPR-motif containing | [LRPPRC](http://www.gene.ucl.ac.uk/cgi-bin/nomenclature/searchgenes.pl?field=symbol&anchor=equals&symbol_search=Search&number=100&format=html&sortby=symbol&match=LRPPRC) | nucleic acid binding;DNA binding;pre-mRNA splicing factor activity;RNA binding | [607544](http://www.ncbi.nlm.nih.gov/entrez/dispomim.cgi?id=607544) | **1.32** |
| 1424355_A_AT | transcriptional regulator, SIN3B (yeast) | [SIN3B](http://www.gene.ucl.ac.uk/cgi-bin/nomenclature/searchgenes.pl?field=symbol&anchor=equals&symbol_search=Search&number=100&format=html&sortby=symbol&match=SIN3B) | nucleic acid binding;DNA binding;receptor activity | [607777](http://www.ncbi.nlm.nih.gov/entrez/dispomim.cgi?id=607777) | **-1.2** |
| 1424356_A_AT | cDNA sequence BC019776 | [METRNL](http://www.gene.ucl.ac.uk/cgi-bin/nomenclature/searchgenes.pl?field=symbol&anchor=equals&symbol_search=Search&number=100&format=html&sortby=symbol&match=METRNL) | nucleic acid binding;DNA binding;RNA binding |  | **-1.34** |
| 1424359_AT | 5-oxoprolinase (ATP-hydrolysing) | [OPLAH](http://www.gene.ucl.ac.uk/cgi-bin/nomenclature/searchgenes.pl?field=symbol&anchor=equals&symbol_search=Search&number=100&format=html&sortby=symbol&match=OPLAH) | nucleic acid binding;DNA binding;RNA binding;translation activator activity |  | **2.56** |
| 1424364_A_AT | RIKEN cDNA 1110020P15 gene | [1110020P15RIK](http://www.gene.ucl.ac.uk/cgi-bin/nomenclature/searchgenes.pl?field=symbol&anchor=equals&symbol_search=Search&number=100&format=html&sortby=symbol&match=1110020P15RIK) | nucleic acid binding;DNA binding;transcription factor activity |  | **1.3** |
| 1424367_A_AT | homer homolog 2 (Drosophila) | [HOMER2](http://www.gene.ucl.ac.uk/cgi-bin/nomenclature/searchgenes.pl?field=symbol&anchor=equals&symbol_search=Search&number=100&format=html&sortby=symbol&match=HOMER2) | nucleic acid binding;lipid transporter activity;RNA binding | [604799](http://www.ncbi.nlm.nih.gov/entrez/dispomim.cgi?id=604799) | **1.79** |
| 1424373_AT | armadillo repeat containing, X-linked 3 | [ARMCX3](http://www.gene.ucl.ac.uk/cgi-bin/nomenclature/searchgenes.pl?field=symbol&anchor=equals&symbol_search=Search&number=100&format=html&sortby=symbol&match=ARMCX3) | nucleic acid binding;poly(A) binding;RNA binding | [300364](http://www.ncbi.nlm.nih.gov/entrez/dispomim.cgi?id=300364) | **-1.42** |
| 1424376_AT | CDC42 effector protein (Rho GTPase binding) 1 | [CDC42EP1](http://www.gene.ucl.ac.uk/cgi-bin/nomenclature/searchgenes.pl?field=symbol&anchor=equals&symbol_search=Search&number=100&format=html&sortby=symbol&match=CDC42EP1) | nucleic acid binding;poly(A) binding;RNA binding | [606084](http://www.ncbi.nlm.nih.gov/entrez/dispomim.cgi?id=606084) | **1.59** |
| 1424382_AT | reticulocalbin 3, EF-hand calcium binding domain | [RCN3](http://www.gene.ucl.ac.uk/cgi-bin/nomenclature/searchgenes.pl?field=symbol&anchor=equals&symbol_search=Search&number=100&format=html&sortby=symbol&match=RCN3) | nucleic acid binding;pre-mRNA splicing factor activity |  | **-1.66** |
| 1424383_AT | cDNA sequence BC003277 | [BC003277](http://www.gene.ucl.ac.uk/cgi-bin/nomenclature/searchgenes.pl?field=symbol&anchor=equals&symbol_search=Search&number=100&format=html&sortby=symbol&match=BC003277) | nucleic acid binding;protein phosphatase type 1 regulator activity |  | **-1.39** |
| 1424389_AT | nucleoporin like 1 | [NUPL1](http://www.gene.ucl.ac.uk/cgi-bin/nomenclature/searchgenes.pl?field=symbol&anchor=equals&symbol_search=Search&number=100&format=html&sortby=symbol&match=NUPL1) | nucleic acid binding;protein phosphatase type 1 regulator activity | [607615](http://www.ncbi.nlm.nih.gov/entrez/dispomim.cgi?id=607615) | **-1.39** |
| 1424391_AT | nardilysin, N-arginine dibasic convertase, NRD convertase 1 | [NRD1](http://www.gene.ucl.ac.uk/cgi-bin/nomenclature/searchgenes.pl?field=symbol&anchor=equals&symbol_search=Search&number=100&format=html&sortby=symbol&match=NRD1) | nucleic acid binding;RNA binding | [602651](http://www.ncbi.nlm.nih.gov/entrez/dispomim.cgi?id=602651) | **1.27** |
| 1424392_AT | alcohol dehydrogenase, iron containing, 1 | [ADHFE1](http://www.gene.ucl.ac.uk/cgi-bin/nomenclature/searchgenes.pl?field=symbol&anchor=equals&symbol_search=Search&number=100&format=html&sortby=symbol&match=ADHFE1) | nucleic acid binding;RNA binding |  | **1.82** |
| 1424393_S_AT | alcohol dehydrogenase, iron containing, 1 | [ADHFE1](http://www.gene.ucl.ac.uk/cgi-bin/nomenclature/searchgenes.pl?field=symbol&anchor=equals&symbol_search=Search&number=100&format=html&sortby=symbol&match=ADHFE1) | nucleic acid binding;RNA binding |  | **2.12** |
| 1424395_AT | asparaginase like 1 | [ASRGL1](http://www.gene.ucl.ac.uk/cgi-bin/nomenclature/searchgenes.pl?field=symbol&anchor=equals&symbol_search=Search&number=100&format=html&sortby=symbol&match=ASRGL1) | nucleic acid binding;RNA binding |  | **1.68** |
| 1424396_A_AT | asparaginase like 1 | [ASRGL1](http://www.gene.ucl.ac.uk/cgi-bin/nomenclature/searchgenes.pl?field=symbol&anchor=equals&symbol_search=Search&number=100&format=html&sortby=symbol&match=ASRGL1) | nucleic acid binding;RNA binding |  | **1.45** |
| 1424408_AT | LIM and senescent cell antigen like domains 2 | [LIMS2](http://www.gene.ucl.ac.uk/cgi-bin/nomenclature/searchgenes.pl?field=symbol&anchor=equals&symbol_search=Search&number=100&format=html&sortby=symbol&match=LIMS2) | nucleic acid binding;RNA binding | [607908](http://www.ncbi.nlm.nih.gov/entrez/dispomim.cgi?id=607908) | **2.78** |
| 1424421_AT | RIKEN cDNA A930017E24 gene | [A930017E24RIK](http://www.gene.ucl.ac.uk/cgi-bin/nomenclature/searchgenes.pl?field=symbol&anchor=equals&symbol_search=Search&number=100&format=html&sortby=symbol&match=A930017E24RIK) | nucleic acid binding;RNA binding |  | **1.31** |
| 1424422_S_AT | RIKEN cDNA A930017E24 gene | [A930017E24RIK](http://www.gene.ucl.ac.uk/cgi-bin/nomenclature/searchgenes.pl?field=symbol&anchor=equals&symbol_search=Search&number=100&format=html&sortby=symbol&match=A930017E24RIK) | nucleic acid binding;RNA binding |  | **1.34** |
| 1424424_AT | solute carrier family 39 (zinc transporter), member 1 | [SLC39A1](http://www.gene.ucl.ac.uk/cgi-bin/nomenclature/searchgenes.pl?field=symbol&anchor=equals&symbol_search=Search&number=100&format=html&sortby=symbol&match=SLC39A1) | nucleic acid binding;RNA binding | [604740](http://www.ncbi.nlm.nih.gov/entrez/dispomim.cgi?id=604740) | **-1.43** |
| 1424433_AT | methionine sulfoxide reductase B | [MSRB2](http://www.gene.ucl.ac.uk/cgi-bin/nomenclature/searchgenes.pl?field=symbol&anchor=equals&symbol_search=Search&number=100&format=html&sortby=symbol&match=MSRB2) | nucleic acid binding;RNA binding |  | **2.28** |
| 1424434_AT | cDNA sequence BC024814 | [BC024814](http://www.gene.ucl.ac.uk/cgi-bin/nomenclature/searchgenes.pl?field=symbol&anchor=equals&symbol_search=Search&number=100&format=html&sortby=symbol&match=BC024814) | nucleic acid binding;RNA binding |  | **1.34** |
| 1424436_AT | phosphoribosylglycinamide formyltransferase | [GART](http://www.gene.ucl.ac.uk/cgi-bin/nomenclature/searchgenes.pl?field=symbol&anchor=equals&symbol_search=Search&number=100&format=html&sortby=symbol&match=GART) | nucleic acid binding;RNA binding | [138440](http://www.ncbi.nlm.nih.gov/entrez/dispomim.cgi?id=138440) | **1.39** |
| 1424440_AT | mitochondrial ribosomal protein S6 | [MRPS6](http://www.gene.ucl.ac.uk/cgi-bin/nomenclature/searchgenes.pl?field=symbol&anchor=equals&symbol_search=Search&number=100&format=html&sortby=symbol&match=MRPS6) | nucleic acid binding;RNA binding |  | **1.42** |
| 1424443_AT | transmembrane 6 superfamily member 1 | [TM6SF1](http://www.gene.ucl.ac.uk/cgi-bin/nomenclature/searchgenes.pl?field=symbol&anchor=equals&symbol_search=Search&number=100&format=html&sortby=symbol&match=TM6SF1) | nucleic acid binding;RNA binding | [606562](http://www.ncbi.nlm.nih.gov/entrez/dispomim.cgi?id=606562) | **-2.13** |
| 1424453_AT | phosphate cytidylyltransferase 1, choline, alpha isoform | [PCYT1A](http://www.gene.ucl.ac.uk/cgi-bin/nomenclature/searchgenes.pl?field=symbol&anchor=equals&symbol_search=Search&number=100&format=html&sortby=symbol&match=PCYT1A) | nucleic acid binding;RNA binding | [123695](http://www.ncbi.nlm.nih.gov/entrez/dispomim.cgi?id=123695) | **1.43** |
| 1424470_A_AT | Rap guanine nucleotide exchange factor (GEF) 3 | [RAPGEF3](http://www.gene.ucl.ac.uk/cgi-bin/nomenclature/searchgenes.pl?field=symbol&anchor=equals&symbol_search=Search&number=100&format=html&sortby=symbol&match=RAPGEF3) | nucleic acid binding;RNA binding | [606057](http://www.ncbi.nlm.nih.gov/entrez/dispomim.cgi?id=606057) | **1.74** |
| 1424471_AT | Rap guanine nucleotide exchange factor (GEF) 3 | [RAPGEF3](http://www.gene.ucl.ac.uk/cgi-bin/nomenclature/searchgenes.pl?field=symbol&anchor=equals&symbol_search=Search&number=100&format=html&sortby=symbol&match=RAPGEF3) | nucleic acid binding;RNA binding | [606057](http://www.ncbi.nlm.nih.gov/entrez/dispomim.cgi?id=606057) | **2.13** |
| 1424474_A_AT | calcium/calmodulin-dependent protein kinase kinase 2, beta | [CAMKK2](http://www.gene.ucl.ac.uk/cgi-bin/nomenclature/searchgenes.pl?field=symbol&anchor=equals&symbol_search=Search&number=100&format=html&sortby=symbol&match=CAMKK2) | nucleic acid binding;RNA binding |  | **-1.45** |
| 1424483_AT | MOB1, Mps One Binder kinase activator-like 1B (yeast) | [MOBK1B](http://www.gene.ucl.ac.uk/cgi-bin/nomenclature/searchgenes.pl?field=symbol&anchor=equals&symbol_search=Search&number=100&format=html&sortby=symbol&match=MOBK1B) | nucleic acid binding;RNA binding |  | **-1.37** |
| 1424505_AT | RIKEN cDNA 0610042C05 gene | [0610042C05RIK](http://www.gene.ucl.ac.uk/cgi-bin/nomenclature/searchgenes.pl?field=symbol&anchor=equals&symbol_search=Search&number=100&format=html&sortby=symbol&match=0610042C05RIK) | nucleic acid binding;RNA binding;pre-mRNA splicing factor activity |  | **1.59** |
| 1424507_AT | Ras and Rab interactor 1 | [RIN1](http://www.gene.ucl.ac.uk/cgi-bin/nomenclature/searchgenes.pl?field=symbol&anchor=equals&symbol_search=Search&number=100&format=html&sortby=symbol&match=RIN1) | nucleic acid binding;RNA binding;pre-mRNA splicing factor activity | [605965](http://www.ncbi.nlm.nih.gov/entrez/dispomim.cgi?id=605965) | **-3.12** |
| 1424510_AT | nudix (nucleoside diphosphate linked moiety X)-type motif 6 | [NUDT6](http://www.gene.ucl.ac.uk/cgi-bin/nomenclature/searchgenes.pl?field=symbol&anchor=equals&symbol_search=Search&number=100&format=html&sortby=symbol&match=NUDT6) | nucleic acid binding;RNA binding;pre-mRNA splicing factor activity | [606261](http://www.ncbi.nlm.nih.gov/entrez/dispomim.cgi?id=606261) | **1.68** |
| 1424513_AT | ATPase, H+ transporting, lysosomal V0 subunit a isoform 2 | [ATP6V0A2](http://www.gene.ucl.ac.uk/cgi-bin/nomenclature/searchgenes.pl?field=symbol&anchor=equals&symbol_search=Search&number=100&format=html&sortby=symbol&match=ATP6V0A2) | nucleic acid binding;RNA binding;structural constituent of ribosome |  | **1.34** |
| 1424516_AT | RIKEN cDNA B230354K17 gene | [B230354K17RIK](http://www.gene.ucl.ac.uk/cgi-bin/nomenclature/searchgenes.pl?field=symbol&anchor=equals&symbol_search=Search&number=100&format=html&sortby=symbol&match=B230354K17RIK) | nucleic acid binding;structural constituent of ribosome |  | **-1.6** |
| 1424519_AT | gb:BC027306.1 /DB_XREF=gi:20073168 /FEA=FLmRNA /CN |  | nucleic acid binding;sugar binding;growth factor activity |  | **1.25** |
| 1424520_AT | RIKEN cDNA 2010305A19 gene | [2010305A19RIK](http://www.gene.ucl.ac.uk/cgi-bin/nomenclature/searchgenes.pl?field=symbol&anchor=equals&symbol_search=Search&number=100&format=html&sortby=symbol&match=2010305A19RIK) | nucleic acid binding;transporter activity;pre-mRNA splicing factor activity;RNA binding |  | **1.49** |
| 1424524_AT | RIKEN cDNA 1200002N14 gene | [1200002N14RIK](http://www.gene.ucl.ac.uk/cgi-bin/nomenclature/searchgenes.pl?field=symbol&anchor=equals&symbol_search=Search&number=100&format=html&sortby=symbol&match=1200002N14RIK) | nucleic acid binding;tubulin-tyrosine ligase activity;ligase activity |  | **-1.87** |
| 1424527_AT | protein phosphatase 2, regulatory subunit B, delta isoform | [PPP2R2D](http://www.gene.ucl.ac.uk/cgi-bin/nomenclature/searchgenes.pl?field=symbol&anchor=equals&symbol_search=Search&number=100&format=html&sortby=symbol&match=PPP2R2D) | nucleoside transporter activity |  | **1.24** |
| 1424529_S_AT | Cell growth regulator with EF hand domain 1 | [CGREF1](http://www.gene.ucl.ac.uk/cgi-bin/nomenclature/searchgenes.pl?field=symbol&anchor=equals&symbol_search=Search&number=100&format=html&sortby=symbol&match=CGREF1) | nucleotide binding;ATP binding;hydrolase activity | [606137](http://www.ncbi.nlm.nih.gov/entrez/dispomim.cgi?id=606137) | **-2.07** |
| 1424531_A_AT | transcription elongation factor A (SII), 3 | [TCEA3](http://www.gene.ucl.ac.uk/cgi-bin/nomenclature/searchgenes.pl?field=symbol&anchor=equals&symbol_search=Search&number=100&format=html&sortby=symbol&match=TCEA3) | nucleotide binding;ATP binding;nucleoside-triphosphatase activity;endopeptidase inhibitor activity;cysteine protease inhibitor activity | [604128](http://www.ncbi.nlm.nih.gov/entrez/dispomim.cgi?id=604128) | **1.84** |
| 1424541_AT | RIKEN cDNA 1110020A09 gene | [1110020A09RIK](http://www.gene.ucl.ac.uk/cgi-bin/nomenclature/searchgenes.pl?field=symbol&anchor=equals&symbol_search=Search&number=100&format=html&sortby=symbol&match=1110020A09RIK) | nucleotide binding;ATPase activity;ATPase activity, coupled to transmembrane movement of substances;ATP binding;nucleoside-triphosphatase activity |  | **1.45** |
| 1424542_AT | S100 calcium binding protein A4 | [S100A4](http://www.gene.ucl.ac.uk/cgi-bin/nomenclature/searchgenes.pl?field=symbol&anchor=equals&symbol_search=Search&number=100&format=html&sortby=symbol&match=S100A4) | nucleotide binding;ATPase activity;ATPase activity, coupled to transmembrane movement of substances;ATPase activity, coupled;nucleoside-triphosphatase activity;ATP binding | [114210](http://www.ncbi.nlm.nih.gov/entrez/dispomim.cgi?id=114210) | **-7.58** |
| 1424544_AT | cDNA sequence BC011468 | [BC011468](http://www.gene.ucl.ac.uk/cgi-bin/nomenclature/searchgenes.pl?field=symbol&anchor=equals&symbol_search=Search&number=100&format=html&sortby=symbol&match=BC011468) | nucleotide binding;ATPase activity;ATPase activity, coupled to transmembrane movement of substances;ATPase activity, coupled;nucleoside-triphosphatase activity;ATP binding |  | **-2.01** |
| 1424554_AT | protein phosphatase 1, regulatory (inhibitor) subunit 8 | [PPP1R8](http://www.gene.ucl.ac.uk/cgi-bin/nomenclature/searchgenes.pl?field=symbol&anchor=equals&symbol_search=Search&number=100&format=html&sortby=symbol&match=PPP1R8) | nucleotide binding;ATPase activity;ATPase activity, coupled to transmembrane movement of substances;ATP-binding cassette (ABC) transporter activity;ATP binding | [602636](http://www.ncbi.nlm.nih.gov/entrez/dispomim.cgi?id=602636) | **-1.32** |
| 1424563_AT | solute carrier family 25 (mitochondrial carrier, adenine nucleotide translocator), member 4 | [SLC25A4](http://www.gene.ucl.ac.uk/cgi-bin/nomenclature/searchgenes.pl?field=symbol&anchor=equals&symbol_search=Search&number=100&format=html&sortby=symbol&match=SLC25A4) | nucleotide binding;ATPase activity;ATPase activity, coupled to transmembrane movement of substances;ATP-binding cassette (ABC) transporter activity;nucleoside-triphosphatase activity;ATP binding | [103220](http://www.ncbi.nlm.nih.gov/entrez/dispomim.cgi?id=103220) | **1.46** |
| 1424567_AT | RIKEN cDNA 6330415F13 gene | [6330415F13RIK](http://www.gene.ucl.ac.uk/cgi-bin/nomenclature/searchgenes.pl?field=symbol&anchor=equals&symbol_search=Search&number=100&format=html&sortby=symbol&match=6330415F13RIK) | nucleotide binding;ATPase activity;ATP-binding cassette (ABC) transporter activity;ATPase activity, coupled to transmembrane movement of substances;ATP binding |  | **1.73** |
| 1424577_AT | cDNA sequence BC008103 | [BC008103](http://www.gene.ucl.ac.uk/cgi-bin/nomenclature/searchgenes.pl?field=symbol&anchor=equals&symbol_search=Search&number=100&format=html&sortby=symbol&match=BC008103) | nucleotide binding;ATPase activity;ATP-binding cassette (ABC) transporter activity;ATPase activity, coupled to transmembrane movement of substances;ATP binding;nucleoside-triphosphatase activity |  | **-1.86** |
| 1424595_AT | F11 receptor | [F11R](http://www.gene.ucl.ac.uk/cgi-bin/nomenclature/searchgenes.pl?field=symbol&anchor=equals&symbol_search=Search&number=100&format=html&sortby=symbol&match=F11R) | nucleotide binding;ATPase activity;nucleoside-triphosphatase activity;ATP binding | [605721](http://www.ncbi.nlm.nih.gov/entrez/dispomim.cgi?id=605721) | **1.53** |
| 1424628_A_AT | gb:BC013640.1 /DB_XREF=gi:15489047 /FEA=FLmRNA /CN |  | nucleotide binding;ATPase activity;receptor activity;ATP-binding cassette (ABC) transporter activity;sulfonylurea receptor activity;ATPase activity, coupled to transmembrane movement of substances;ATP binding |  | **1.28** |
| 1424635_AT | eukaryotic translation elongation factor 1 alpha 1 | [EEF1A1](http://www.gene.ucl.ac.uk/cgi-bin/nomenclature/searchgenes.pl?field=symbol&anchor=equals&symbol_search=Search&number=100&format=html&sortby=symbol&match=EEF1A1) | nucleotide binding;ATPase activity;receptor activity;sulfonylurea receptor activity;ATPase activity, coupled to transmembrane movement of substances;ATP-binding cassette (ABC) transporter activity;ATP binding | [130590](http://www.ncbi.nlm.nih.gov/entrez/dispomim.cgi?id=130590) | **-1.39** |
| 1424638_AT | cyclin-dependent kinase inhibitor 1A (P21) | [CDKN1A](http://www.gene.ucl.ac.uk/cgi-bin/nomenclature/searchgenes.pl?field=symbol&anchor=equals&symbol_search=Search&number=100&format=html&sortby=symbol&match=CDKN1A) | nucleotide binding;microtubule motor activity;motor activity;cysteine-type endopeptidase activity;nucleoside-triphosphatase activity;ATP binding | [116899](http://www.ncbi.nlm.nih.gov/entrez/dispomim.cgi?id=116899) | **-2.41** |
| 1424639_A_AT | gb:BC025440.1 /DB_XREF=gi:19343555 /FEA=FLmRNA /CN |  | nucleotide binding;nucleoside-triphosphatase activity;ATP binding |  | **1.38** |
| 1424649_A_AT | transmembrane 4 superfamily member 3 | [TM4SF3](http://www.gene.ucl.ac.uk/cgi-bin/nomenclature/searchgenes.pl?field=symbol&anchor=equals&symbol_search=Search&number=100&format=html&sortby=symbol&match=TM4SF3) | nucleotide binding;nucleoside-triphosphatase activity;ATP binding | [600769](http://www.ncbi.nlm.nih.gov/entrez/dispomim.cgi?id=600769) | **2.27** |
| 1424654_AT | acid phosphatase 2, lysosomal | [ACP2](http://www.gene.ucl.ac.uk/cgi-bin/nomenclature/searchgenes.pl?field=symbol&anchor=equals&symbol_search=Search&number=100&format=html&sortby=symbol&match=ACP2) | nucleotide binding;nucleoside-triphosphatase activity;ATP binding;hydrolase activity | [171650](http://www.ncbi.nlm.nih.gov/entrez/dispomim.cgi?id=171650) | **-1.33** |
| 1424667_A_AT | cut-like 1 (Drosophila) | [CUTL1](http://www.gene.ucl.ac.uk/cgi-bin/nomenclature/searchgenes.pl?field=symbol&anchor=equals&symbol_search=Search&number=100&format=html&sortby=symbol&match=CUTL1) | nucleotide binding;RNA binding;receptor activity;GTP binding;nucleoside-triphosphatase activity | [116896](http://www.ncbi.nlm.nih.gov/entrez/dispomim.cgi?id=116896) | **1.25** |
| 1424670_S_AT | zinc finger, FYVE domain containing 21 | [ZFYVE21](http://www.gene.ucl.ac.uk/cgi-bin/nomenclature/searchgenes.pl?field=symbol&anchor=equals&symbol_search=Search&number=100&format=html&sortby=symbol&match=ZFYVE21) | nucleotide binding;serine-type endopeptidase activity;ATP binding;ATP-dependent peptidase activity;hydrolase activity;peptidase activity |  | **1.36** |
| 1424674_AT | solute carrier family 39 (metal ion transporter), member 6 | [SLC39A6](http://www.gene.ucl.ac.uk/cgi-bin/nomenclature/searchgenes.pl?field=symbol&anchor=equals&symbol_search=Search&number=100&format=html&sortby=symbol&match=SLC39A6) | nucleotidyltransferase activity;N-acylneuraminate cytidylyltransferase activity;transferase activity | [608731](http://www.ncbi.nlm.nih.gov/entrez/dispomim.cgi?id=608731) | **-1.87** |
| 1424704_AT | runt related transcription factor 2 | [RUNX2](http://www.gene.ucl.ac.uk/cgi-bin/nomenclature/searchgenes.pl?field=symbol&anchor=equals&symbol_search=Search&number=100&format=html&sortby=symbol&match=RUNX2) | nucleotidyltransferase activity;transferase activity;phosphatidate cytidylyltransferase activity | [600211](http://www.ncbi.nlm.nih.gov/entrez/dispomim.cgi?id=600211) | **-8.33** |
| 1424715_AT | RIKEN cDNA 0610039N19 gene | [0610039N19RIK](http://www.gene.ucl.ac.uk/cgi-bin/nomenclature/searchgenes.pl?field=symbol&anchor=equals&symbol_search=Search&number=100&format=html&sortby=symbol&match=0610039N19RIK) | nutrient reservoir activity;calcium-independent phospholipase A2 activity;catalytic activity;ATP binding |  | **2.24** |
| 1424716_AT | RIKEN cDNA 0610039N19 gene | [0610039N19RIK](http://www.gene.ucl.ac.uk/cgi-bin/nomenclature/searchgenes.pl?field=symbol&anchor=equals&symbol_search=Search&number=100&format=html&sortby=symbol&match=0610039N19RIK) | nutrient reservoir activity;catalytic activity |  | **2.14** |
| 1424727_AT | chemokine (C-C motif) receptor 5 | [CCR5](http://www.gene.ucl.ac.uk/cgi-bin/nomenclature/searchgenes.pl?field=symbol&anchor=equals&symbol_search=Search&number=100&format=html&sortby=symbol&match=CCR5) | oligosaccharyl transferase activity;transferase activity | [601373](http://www.ncbi.nlm.nih.gov/entrez/dispomim.cgi?id=601373) | **-3.95** |
| 1424729_AT | cDNA sequence BC054059 | [BC054059](http://www.gene.ucl.ac.uk/cgi-bin/nomenclature/searchgenes.pl?field=symbol&anchor=equals&symbol_search=Search&number=100&format=html&sortby=symbol&match=BC054059) | oligosaccharyl transferase activity;transferase activity |  | **5.5** |
| 1424737_AT | thyroid hormone responsive SPOT14 homolog (Rattus) | [THRSP](http://www.gene.ucl.ac.uk/cgi-bin/nomenclature/searchgenes.pl?field=symbol&anchor=equals&symbol_search=Search&number=100&format=html&sortby=symbol&match=THRSP) | omega peptidase activity;protein binding;ubiquitin thiolesterase activity;cysteine-type endopeptidase activity;hydrolase activity;ubiquitin binding;cysteine-type peptidase activity | [601926](http://www.ncbi.nlm.nih.gov/entrez/dispomim.cgi?id=601926) | **2.55** |
| 1424741_S_AT | cAMP responsive element binding protein 3 | [CREB3](http://www.gene.ucl.ac.uk/cgi-bin/nomenclature/searchgenes.pl?field=symbol&anchor=equals&symbol_search=Search&number=100&format=html&sortby=symbol&match=CREB3) | oncostatin-M receptor activity;hematopoietin/interferon-class (D200-domain) cytokine receptor activity;receptor activity | [606443](http://www.ncbi.nlm.nih.gov/entrez/dispomim.cgi?id=606443) | **-1.28** |
| 1424746_AT | Kinesin family member 1C | [KIF1C](http://www.gene.ucl.ac.uk/cgi-bin/nomenclature/searchgenes.pl?field=symbol&anchor=equals&symbol_search=Search&number=100&format=html&sortby=symbol&match=KIF1C) | opioid peptide activity | [603060](http://www.ncbi.nlm.nih.gov/entrez/dispomim.cgi?id=603060) | **1.35** |
| 1424753_AT | nudix (nucleoside diphosphate linked moiety X)-type motif 14 | [NUDT14](http://www.gene.ucl.ac.uk/cgi-bin/nomenclature/searchgenes.pl?field=symbol&anchor=equals&symbol_search=Search&number=100&format=html&sortby=symbol&match=NUDT14) | opioid peptide activity;zinc ion binding;nucleic acid binding;SH3 domain binding |  | **-1.36** |
| 1424754_AT | membrane-spanning 4-domains, subfamily A, member 7 | [MS4A7](http://www.gene.ucl.ac.uk/cgi-bin/nomenclature/searchgenes.pl?field=symbol&anchor=equals&symbol_search=Search&number=100&format=html&sortby=symbol&match=MS4A7) | opioid peptide activity;zinc ion binding;nucleic acid binding;SH3 domain binding | [606502](http://www.ncbi.nlm.nih.gov/entrez/dispomim.cgi?id=606502) | **-10.8** |
| 1424762_AT | membrane-type frizzled-related protein | [C1QTNF5](http://www.gene.ucl.ac.uk/cgi-bin/nomenclature/searchgenes.pl?field=symbol&anchor=equals&symbol_search=Search&number=100&format=html&sortby=symbol&match=C1QTNF5) | ornithine decarboxylase inhibitor activity;calcium ion binding;enzyme inhibitor activity | [608752](http://www.ncbi.nlm.nih.gov/entrez/dispomim.cgi?id=608752) | **-1.51** |
| 1424767_AT | cadherin 22 | [CDH22](http://www.gene.ucl.ac.uk/cgi-bin/nomenclature/searchgenes.pl?field=symbol&anchor=equals&symbol_search=Search&number=100&format=html&sortby=symbol&match=CDH22) | O-sialoglycoprotein endopeptidase activity;zinc ion binding;peptidase activity |  | **3** |
| 1424790_AT | RIKEN cDNA 2900084M01 gene | [2900084M01RIK](http://www.gene.ucl.ac.uk/cgi-bin/nomenclature/searchgenes.pl?field=symbol&anchor=equals&symbol_search=Search&number=100&format=html&sortby=symbol&match=2900084M01RIK) | oxidoreductase activity |  | **1.68** |
| 1424800_AT | enabled homolog (Drosophila) | [ENAH](http://www.gene.ucl.ac.uk/cgi-bin/nomenclature/searchgenes.pl?field=symbol&anchor=equals&symbol_search=Search&number=100&format=html&sortby=symbol&match=ENAH) | oxidoreductase activity |  | **-1.54** |
| 1424801_AT | enabled homolog (Drosophila) | [ENAH](http://www.gene.ucl.ac.uk/cgi-bin/nomenclature/searchgenes.pl?field=symbol&anchor=equals&symbol_search=Search&number=100&format=html&sortby=symbol&match=ENAH) | oxidoreductase activity |  | **-1.34** |
| 1424802_A_AT | RIKEN cDNA 3300001P08 gene | [3300001P08RIK](http://www.gene.ucl.ac.uk/cgi-bin/nomenclature/searchgenes.pl?field=symbol&anchor=equals&symbol_search=Search&number=100&format=html&sortby=symbol&match=3300001P08RIK) | oxidoreductase activity |  | **-1.69** |
| 1424820_A_AT | Nedd4 family interacting protein 1 | [NDFIP1](http://www.gene.ucl.ac.uk/cgi-bin/nomenclature/searchgenes.pl?field=symbol&anchor=equals&symbol_search=Search&number=100&format=html&sortby=symbol&match=NDFIP1) | oxidoreductase activity |  | **-1.26** |
| 1424826_S_AT | metastasis suppressor 1 | [MTSS1](http://www.gene.ucl.ac.uk/cgi-bin/nomenclature/searchgenes.pl?field=symbol&anchor=equals&symbol_search=Search&number=100&format=html&sortby=symbol&match=MTSS1) | oxidoreductase activity | [608486](http://www.ncbi.nlm.nih.gov/entrez/dispomim.cgi?id=608486) | **1.36** |
| 1424827_A_AT | casein kinase 1, alpha 1 | [CSNK1A1](http://www.gene.ucl.ac.uk/cgi-bin/nomenclature/searchgenes.pl?field=symbol&anchor=equals&symbol_search=Search&number=100&format=html&sortby=symbol&match=CSNK1A1) | oxidoreductase activity | [600505](http://www.ncbi.nlm.nih.gov/entrez/dispomim.cgi?id=600505) | **-1.32** |
| 1424830_AT | cyclin K | [CCNK](http://www.gene.ucl.ac.uk/cgi-bin/nomenclature/searchgenes.pl?field=symbol&anchor=equals&symbol_search=Search&number=100&format=html&sortby=symbol&match=CCNK) | oxidoreductase activity | [603544](http://www.ncbi.nlm.nih.gov/entrez/dispomim.cgi?id=603544) | **1.31** |
| 1424831_AT | copine II | [CPNE2](http://www.gene.ucl.ac.uk/cgi-bin/nomenclature/searchgenes.pl?field=symbol&anchor=equals&symbol_search=Search&number=100&format=html&sortby=symbol&match=CPNE2) | oxidoreductase activity | [604206](http://www.ncbi.nlm.nih.gov/entrez/dispomim.cgi?id=604206) | **-19.61** |
| 1424832_AT | RIKEN cDNA 4732429D16 gene | [4732429D16RIK](http://www.gene.ucl.ac.uk/cgi-bin/nomenclature/searchgenes.pl?field=symbol&anchor=equals&symbol_search=Search&number=100&format=html&sortby=symbol&match=4732429D16RIK) | oxidoreductase activity |  | **-1.49** |
| 1424846_AT | cDNA sequence BC027174 | [BC027174](http://www.gene.ucl.ac.uk/cgi-bin/nomenclature/searchgenes.pl?field=symbol&anchor=equals&symbol_search=Search&number=100&format=html&sortby=symbol&match=BC027174) | oxidoreductase activity |  | **1.33** |
| 1424854_AT | histone 1, H4f | [HIST1H4F](http://www.gene.ucl.ac.uk/cgi-bin/nomenclature/searchgenes.pl?field=symbol&anchor=equals&symbol_search=Search&number=100&format=html&sortby=symbol&match=HIST1H4F) | oxidoreductase activity | [602824](http://www.ncbi.nlm.nih.gov/entrez/dispomim.cgi?id=602824) | **1.52** |
| 1424858_AT | cDNA sequence BC016226 | [BC016226](http://www.gene.ucl.ac.uk/cgi-bin/nomenclature/searchgenes.pl?field=symbol&anchor=equals&symbol_search=Search&number=100&format=html&sortby=symbol&match=BC016226) | oxidoreductase activity |  | **1.61** |
| 1424877_A_AT | aminolevulinate, delta-, dehydratase | [ALAD](http://www.gene.ucl.ac.uk/cgi-bin/nomenclature/searchgenes.pl?field=symbol&anchor=equals&symbol_search=Search&number=100&format=html&sortby=symbol&match=ALAD) | oxidoreductase activity | [125270](http://www.ncbi.nlm.nih.gov/entrez/dispomim.cgi?id=125270) | **1.87** |
| 1424886_AT | Protein tyrosine phosphatase, receptor type, D | [PTPRD](http://www.gene.ucl.ac.uk/cgi-bin/nomenclature/searchgenes.pl?field=symbol&anchor=equals&symbol_search=Search&number=100&format=html&sortby=symbol&match=PTPRD) | oxidoreductase activity | [601598](http://www.ncbi.nlm.nih.gov/entrez/dispomim.cgi?id=601598) | **-1.58** |
| 1424890_AT | basonuclin 1 | [BNC1](http://www.gene.ucl.ac.uk/cgi-bin/nomenclature/searchgenes.pl?field=symbol&anchor=equals&symbol_search=Search&number=100&format=html&sortby=symbol&match=BNC1) | oxidoreductase activity | [601930](http://www.ncbi.nlm.nih.gov/entrez/dispomim.cgi?id=601930) | **1.7** |
| 1424895_AT | G-protein signalling modulator 2 (AGS3-like, C. elegans) | [GPSM2](http://www.gene.ucl.ac.uk/cgi-bin/nomenclature/searchgenes.pl?field=symbol&anchor=equals&symbol_search=Search&number=100&format=html&sortby=symbol&match=GPSM2) | oxidoreductase activity |  | **-7.27** |
| 1424902_AT | plexin domain containing 1 | [PLXDC1](http://www.gene.ucl.ac.uk/cgi-bin/nomenclature/searchgenes.pl?field=symbol&anchor=equals&symbol_search=Search&number=100&format=html&sortby=symbol&match=PLXDC1) | oxidoreductase activity | [606826](http://www.ncbi.nlm.nih.gov/entrez/dispomim.cgi?id=606826) | **1.4** |
| 1424904_AT | gb:BC019143.1 /DB_XREF=gi:17512347 /FEA=FLmRNA /CN |  | oxidoreductase activity |  | **1.45** |
| 1424915_S_AT | gb:AK009800.1 /DB_XREF=gi:12844819 /FEA=FLmRNA /CN |  | oxidoreductase activity |  | **1.3** |
| 1424927_AT | GLI pathogenesis-related 1 (glioma) | [GLIPR1](http://www.gene.ucl.ac.uk/cgi-bin/nomenclature/searchgenes.pl?field=symbol&anchor=equals&symbol_search=Search&number=100&format=html&sortby=symbol&match=GLIPR1) | oxidoreductase activity | [602692](http://www.ncbi.nlm.nih.gov/entrez/dispomim.cgi?id=602692) | **-5.28** |
| 1424932_AT | epidermal growth factor receptor | [EGFR](http://www.gene.ucl.ac.uk/cgi-bin/nomenclature/searchgenes.pl?field=symbol&anchor=equals&symbol_search=Search&number=100&format=html&sortby=symbol&match=EGFR) | oxidoreductase activity | [131550](http://www.ncbi.nlm.nih.gov/entrez/dispomim.cgi?id=131550) | **-2.2** |
| 1424937_AT | RIKEN cDNA 2310076L09 gene | [2310076L09RIK](http://www.gene.ucl.ac.uk/cgi-bin/nomenclature/searchgenes.pl?field=symbol&anchor=equals&symbol_search=Search&number=100&format=html&sortby=symbol&match=2310076L09RIK) | oxidoreductase activity |  | **2.04** |
| 1424942_A_AT | myelocytomatosis oncogene | [MYC](http://www.gene.ucl.ac.uk/cgi-bin/nomenclature/searchgenes.pl?field=symbol&anchor=equals&symbol_search=Search&number=100&format=html&sortby=symbol&match=MYC) | oxidoreductase activity, acting on CH-OH group of donors;oxidoreductase activity;glycerol-3-phosphate dehydrogenase (NAD+) activity | [190080](http://www.ncbi.nlm.nih.gov/entrez/dispomim.cgi?id=190080) | **-2.62** |
| 1424946_A_AT | RIKEN cDNA 1300002C08 gene | [1300002C08RIK](http://www.gene.ucl.ac.uk/cgi-bin/nomenclature/searchgenes.pl?field=symbol&anchor=equals&symbol_search=Search&number=100&format=html&sortby=symbol&match=1300002C08RIK) | oxidoreductase activity, acting on iron-sulfur proteins as donors |  | **1.55** |
| 1424951_AT | RIKEN cDNA 1300006M19 gene | [1300006M19RIK](http://www.gene.ucl.ac.uk/cgi-bin/nomenclature/searchgenes.pl?field=symbol&anchor=equals&symbol_search=Search&number=100&format=html&sortby=symbol&match=1300006M19RIK) | oxidoreductase activity, acting on NADH or NADPH |  | **-1.98** |
| 1424965_AT | leupaxin | [LPXN](http://www.gene.ucl.ac.uk/cgi-bin/nomenclature/searchgenes.pl?field=symbol&anchor=equals&symbol_search=Search&number=100&format=html&sortby=symbol&match=LPXN) | oxidoreductase activity, acting on paired donors, with incorporation or reduction of molecular oxygen, reduced flavin or flavoprotein as one donor, and incorporation of one atom of oxygen;oxidoreductase activity;monooxygenase activity | [605390](http://www.ncbi.nlm.nih.gov/entrez/dispomim.cgi?id=605390) | **-7.98** |
| 1424967_X_AT | troponin T2, cardiac | [TNNT2](http://www.gene.ucl.ac.uk/cgi-bin/nomenclature/searchgenes.pl?field=symbol&anchor=equals&symbol_search=Search&number=100&format=html&sortby=symbol&match=TNNT2) | oxidoreductase activity, acting on paired donors, with incorporation or reduction of molecular oxygen, reduced flavin or flavoprotein as one donor, and incorporation of one atom of oxygen;oxidoreductase activity;monooxygenase activity | [191045](http://www.ncbi.nlm.nih.gov/entrez/dispomim.cgi?id=191045) | **-29.45** |
| 1424987_AT | gb:BB128517 /DB_XREF=gi:16262436 /DB_XREF=BB128517 |  | oxidoreductase activity, acting on peroxide as acceptor;catalase activity;oxidoreductase activity;peroxidase activity |  | **-2.21** |
| 1424988_AT | myosin regulatory light chain interacting protein | [MYLIP](http://www.gene.ucl.ac.uk/cgi-bin/nomenclature/searchgenes.pl?field=symbol&anchor=equals&symbol_search=Search&number=100&format=html&sortby=symbol&match=MYLIP) | oxidoreductase activity, acting on peroxide as acceptor;catalase activity;oxidoreductase activity;peroxidase activity |  | **1.38** |
| 1425019_AT | UBX domain containing 4 | [UBXD4](http://www.gene.ucl.ac.uk/cgi-bin/nomenclature/searchgenes.pl?field=symbol&anchor=equals&symbol_search=Search&number=100&format=html&sortby=symbol&match=UBXD4) | oxidoreductase activity, acting on single donors with incorporation of molecular oxygen, incorporation of two atoms of oxygen;oxidoreductase activity |  | **1.78** |
| 1425020_AT | UBX domain containing 4 | [UBXD4](http://www.gene.ucl.ac.uk/cgi-bin/nomenclature/searchgenes.pl?field=symbol&anchor=equals&symbol_search=Search&number=100&format=html&sortby=symbol&match=UBXD4) | oxidoreductase activity, acting on single donors with incorporation of molecular oxygen, incorporation of two atoms of oxygen;oxidoreductase activity |  | **1.54** |
| 1425025_AT | cDNA sequence BC022145 | [BC022145](http://www.gene.ucl.ac.uk/cgi-bin/nomenclature/searchgenes.pl?field=symbol&anchor=equals&symbol_search=Search&number=100&format=html&sortby=symbol&match=BC022145) | oxidoreductase activity, acting on single donors with incorporation of molecular oxygen, incorporation of two atoms of oxygen;oxidoreductase activity, acting on paired donors, with incorporation or reduction of molecular oxygen, 2-oxoglutarate as one donor, and incorporation of one atom each of oxygen into both donors;oxidoreductase activity |  | **-1.52** |
| 1425028_A_AT | tropomyosin 2, beta | [TPM2](http://www.gene.ucl.ac.uk/cgi-bin/nomenclature/searchgenes.pl?field=symbol&anchor=equals&symbol_search=Search&number=100&format=html&sortby=symbol&match=TPM2) | oxidoreductase activity, acting on single donors with incorporation of molecular oxygen, incorporation of two atoms of oxygen;oxidoreductase activity, acting on paired donors, with incorporation or reduction of molecular oxygen, 2-oxoglutarate as one donor, and incorporation of one atom each of oxygen into both donors;oxidoreductase activity | [190990](http://www.ncbi.nlm.nih.gov/entrez/dispomim.cgi?id=190990) | **1.25** |
| 1425029_A_AT | O-acyltransferase (membrane bound) domain containing 2 | [OACT2](http://www.gene.ucl.ac.uk/cgi-bin/nomenclature/searchgenes.pl?field=symbol&anchor=equals&symbol_search=Search&number=100&format=html&sortby=symbol&match=OACT2) | oxidoreductase activity, acting on single donors with incorporation of molecular oxygen, incorporation of two atoms of oxygen;oxidoreductase activity, acting on paired donors, with incorporation or reduction of molecular oxygen, 2-oxoglutarate as one donor, and incorporation of one atom each of oxygen into both donors;oxidoreductase activity;endopeptidase inhibitor activity;procollagen-lysine 5-dioxygenase activity |  | **-1.96** |
| 1425039_AT | integrin, beta-like 1 | [ITGBL1](http://www.gene.ucl.ac.uk/cgi-bin/nomenclature/searchgenes.pl?field=symbol&anchor=equals&symbol_search=Search&number=100&format=html&sortby=symbol&match=ITGBL1) | oxidoreductase activity, acting on single donors with incorporation of molecular oxygen, incorporation of two atoms of oxygen;oxidoreductase activity, acting on paired donors, with incorporation or reduction of molecular oxygen, 2-oxoglutarate as one donor, and incorporation of one atom each of oxygen into both donors;oxidoreductase activity;endopeptidase inhibitor activity;procollagen-lysine 5-dioxygenase activity | [604234](http://www.ncbi.nlm.nih.gov/entrez/dispomim.cgi?id=604234) | **-2.42** |
| 1425052_AT | RIKEN cDNA 2610034N03 gene | [2610034N03RIK](http://www.gene.ucl.ac.uk/cgi-bin/nomenclature/searchgenes.pl?field=symbol&anchor=equals&symbol_search=Search&number=100&format=html&sortby=symbol&match=2610034N03RIK) | oxidoreductase activity, acting on the aldehyde or oxo group of donors, disulfide as acceptor;oxidoreductase activity;3-methyl-2-oxobutanoate dehydrogenase (2-methylpropanoyl-transferring) activity |  | **1.27** |
| 1425053_AT | RIKEN cDNA 2610034N03 gene | [2610034N03RIK](http://www.gene.ucl.ac.uk/cgi-bin/nomenclature/searchgenes.pl?field=symbol&anchor=equals&symbol_search=Search&number=100&format=html&sortby=symbol&match=2610034N03RIK) | oxidoreductase activity;2,4-dienoyl-CoA reductase (NADPH) activity |  | **1.77** |
| 1425076_AT | RIKEN cDNA 2700075B01 gene | [2700075B01RIK](http://www.gene.ucl.ac.uk/cgi-bin/nomenclature/searchgenes.pl?field=symbol&anchor=equals&symbol_search=Search&number=100&format=html&sortby=symbol&match=2700075B01RIK) | oxidoreductase activity;2,4-dienoyl-CoA reductase (NADPH) activity |  | **-1.44** |
| 1425097_A_AT | zinc finger protein 106 | [ZFP106](http://www.gene.ucl.ac.uk/cgi-bin/nomenclature/searchgenes.pl?field=symbol&anchor=equals&symbol_search=Search&number=100&format=html&sortby=symbol&match=ZFP106) | oxidoreductase activity;3-hydroxybutyrate dehydrogenase activity |  | **1.86** |
| 1425143_A_AT | NADH dehydrogenase (ubiquinone) Fe-S protein 1 | [NDUFS1](http://www.gene.ucl.ac.uk/cgi-bin/nomenclature/searchgenes.pl?field=symbol&anchor=equals&symbol_search=Search&number=100&format=html&sortby=symbol&match=NDUFS1) | oxidoreductase activity;3-hydroxybutyrate dehydrogenase activity | [157655](http://www.ncbi.nlm.nih.gov/entrez/dispomim.cgi?id=157655) | **1.31** |
| 1425148_A_AT | sorting nexin 6 | [SNX6](http://www.gene.ucl.ac.uk/cgi-bin/nomenclature/searchgenes.pl?field=symbol&anchor=equals&symbol_search=Search&number=100&format=html&sortby=symbol&match=SNX6) | oxidoreductase activity;acyl-CoA dehydrogenase activity | [606098](http://www.ncbi.nlm.nih.gov/entrez/dispomim.cgi?id=606098) | **-1.42** |
| 1425164_A_AT | phosphorylase kinase gamma 1 | [PHKG1](http://www.gene.ucl.ac.uk/cgi-bin/nomenclature/searchgenes.pl?field=symbol&anchor=equals&symbol_search=Search&number=100&format=html&sortby=symbol&match=PHKG1) | oxidoreductase activity;acyl-CoA dehydrogenase activity | [172470](http://www.ncbi.nlm.nih.gov/entrez/dispomim.cgi?id=172470) | **1.75** |
| 1425186_AT | RIKEN cDNA 0910001K20 gene | [0910001K20RIK](http://www.gene.ucl.ac.uk/cgi-bin/nomenclature/searchgenes.pl?field=symbol&anchor=equals&symbol_search=Search&number=100&format=html&sortby=symbol&match=0910001K20RIK) | oxidoreductase activity;acyl-CoA dehydrogenase activity |  | **1.48** |
| 1425194_A_AT | RIKEN cDNA 6330577E15 gene | [6330577E15RIK](http://www.gene.ucl.ac.uk/cgi-bin/nomenclature/searchgenes.pl?field=symbol&anchor=equals&symbol_search=Search&number=100&format=html&sortby=symbol&match=6330577E15RIK) | oxidoreductase activity;acyl-CoA dehydrogenase activity |  | **-1.27** |
| 1425196_A_AT | Histidine triad nucleotide binding protein 2 | [HINT2](http://www.gene.ucl.ac.uk/cgi-bin/nomenclature/searchgenes.pl?field=symbol&anchor=equals&symbol_search=Search&number=100&format=html&sortby=symbol&match=HINT2) | oxidoreductase activity;acyl-CoA dehydrogenase activity |  | **1.63** |
| 1425214_AT | pyrimidinergic receptor P2Y, G-protein coupled, 6 | [P2RY6](http://www.gene.ucl.ac.uk/cgi-bin/nomenclature/searchgenes.pl?field=symbol&anchor=equals&symbol_search=Search&number=100&format=html&sortby=symbol&match=P2RY6) | oxidoreductase activity;acyl-CoA dehydrogenase activity;long-chain-acyl-CoA dehydrogenase activity | [602451](http://www.ncbi.nlm.nih.gov/entrez/dispomim.cgi?id=602451) | **-3.74** |
| 1425225_AT | Fc receptor-like 3 | [FCRL3](http://www.gene.ucl.ac.uk/cgi-bin/nomenclature/searchgenes.pl?field=symbol&anchor=equals&symbol_search=Search&number=100&format=html&sortby=symbol&match=FCRL3) | oxidoreductase activity;aldehyde reductase activity |  | **-1.75** |
| 1425265_A_AT | prion protein interacting protein 1 | [PRNPIP1](http://www.gene.ucl.ac.uk/cgi-bin/nomenclature/searchgenes.pl?field=symbol&anchor=equals&symbol_search=Search&number=100&format=html&sortby=symbol&match=PRNPIP1) | oxidoreductase activity;ATP binding;isocitrate dehydrogenase (NAD+) activity |  | **1.25** |
| 1425282_AT | IBR domain containing 2 | [IBRDC2](http://www.gene.ucl.ac.uk/cgi-bin/nomenclature/searchgenes.pl?field=symbol&anchor=equals&symbol_search=Search&number=100&format=html&sortby=symbol&match=IBRDC2) | oxidoreductase activity;ATP binding;isocitrate dehydrogenase (NAD+) activity |  | **2.43** |
| 1425320_AT | RIKEN cDNA A830023I12 gene | [A830023I12RIK](http://www.gene.ucl.ac.uk/cgi-bin/nomenclature/searchgenes.pl?field=symbol&anchor=equals&symbol_search=Search&number=100&format=html&sortby=symbol&match=A830023I12RIK) | oxidoreductase activity;carbonyl reductase (NADPH) activity |  | **1.6** |
| 1425331_AT | zinc finger protein 106 | [ZFP106](http://www.gene.ucl.ac.uk/cgi-bin/nomenclature/searchgenes.pl?field=symbol&anchor=equals&symbol_search=Search&number=100&format=html&sortby=symbol&match=ZFP106) | oxidoreductase activity;copper ion binding;protein-lysine 6-oxidase activity |  | **1.47** |
| 1425344_AT | RIKEN cDNA 4430402O11 gene | [4430402O11RIK](http://www.gene.ucl.ac.uk/cgi-bin/nomenclature/searchgenes.pl?field=symbol&anchor=equals&symbol_search=Search&number=100&format=html&sortby=symbol&match=4430402O11RIK) | oxidoreductase activity;coproporphyrinogen oxidase activity |  | **1.54** |
| 1425407_S_AT | C-type (calcium dependent, carbohydrate recognition domain) lectin, superfamily member 6 | [CLECSF6](http://www.gene.ucl.ac.uk/cgi-bin/nomenclature/searchgenes.pl?field=symbol&anchor=equals&symbol_search=Search&number=100&format=html&sortby=symbol&match=CLECSF6) | oxidoreductase activity;coproporphyrinogen oxidase activity | [605306](http://www.ncbi.nlm.nih.gov/entrez/dispomim.cgi?id=605306) | **-2.76** |
| 1425415_A_AT | solute carrier family 1 (neuronal/epithelial high affinity glutamate transporter, system Xag), member 1 | [SLC1A1](http://www.gene.ucl.ac.uk/cgi-bin/nomenclature/searchgenes.pl?field=symbol&anchor=equals&symbol_search=Search&number=100&format=html&sortby=symbol&match=SLC1A1) | oxidoreductase activity;electron carrier activity | [133550](http://www.ncbi.nlm.nih.gov/entrez/dispomim.cgi?id=133550) | **1.76** |
| 1425439_A_AT | solute carrier family 41, member 3 | [SLC41A3](http://www.gene.ucl.ac.uk/cgi-bin/nomenclature/searchgenes.pl?field=symbol&anchor=equals&symbol_search=Search&number=100&format=html&sortby=symbol&match=SLC41A3) | oxidoreductase activity;isocitrate dehydrogenase (NAD+) activity |  | **1.8** |
| 1425455_A_AT | Churchill domain containing 1 | [CHURC1](http://www.gene.ucl.ac.uk/cgi-bin/nomenclature/searchgenes.pl?field=symbol&anchor=equals&symbol_search=Search&number=100&format=html&sortby=symbol&match=CHURC1) | oxidoreductase activity;isocitrate dehydrogenase (NAD+) activity | [608577](http://www.ncbi.nlm.nih.gov/entrez/dispomim.cgi?id=608577) | **1.2** |
| 1425457_A_AT | growth factor receptor bound protein 10 | [GRB10](http://www.gene.ucl.ac.uk/cgi-bin/nomenclature/searchgenes.pl?field=symbol&anchor=equals&symbol_search=Search&number=100&format=html&sortby=symbol&match=GRB10) | oxidoreductase activity;isocitrate dehydrogenase (NAD+) activity | [601523](http://www.ncbi.nlm.nih.gov/entrez/dispomim.cgi?id=601523) | **-1.74** |
| 1425458_A_AT | gb:AK017862.1 /DB_XREF=gi:12857332 /FEA=FLmRNA /CN |  | oxidoreductase activity;isocitrate dehydrogenase (NAD+) activity |  | **-2.28** |
| 1425472_A_AT | lamin A | [LMNA](http://www.gene.ucl.ac.uk/cgi-bin/nomenclature/searchgenes.pl?field=symbol&anchor=equals&symbol_search=Search&number=100&format=html&sortby=symbol&match=LMNA) | oxidoreductase activity;isocitrate dehydrogenase (NAD+) activity | [150330](http://www.ncbi.nlm.nih.gov/entrez/dispomim.cgi?id=150330) | **-1.72** |
| 1425476_AT | procollagen, type IV, alpha 5 | [COL4A5](http://www.gene.ucl.ac.uk/cgi-bin/nomenclature/searchgenes.pl?field=symbol&anchor=equals&symbol_search=Search&number=100&format=html&sortby=symbol&match=COL4A5) | oxidoreductase activity;monooxygenase activity | [303630](http://www.ncbi.nlm.nih.gov/entrez/dispomim.cgi?id=303630) | **-1.61** |
| 1425477_X_AT | histocompatibility 2, class II antigen A, beta 1 | [RMCS1](http://www.gene.ucl.ac.uk/cgi-bin/nomenclature/searchgenes.pl?field=symbol&anchor=equals&symbol_search=Search&number=100&format=html&sortby=symbol&match=RMCS1) | oxidoreductase activity;monooxygenase activity |  | **-2.61** |
| 1425480_AT | CCR4-NOT transcription complex, subunit 6-like | [CNOT6L](http://www.gene.ucl.ac.uk/cgi-bin/nomenclature/searchgenes.pl?field=symbol&anchor=equals&symbol_search=Search&number=100&format=html&sortby=symbol&match=CNOT6L) | oxidoreductase activity;NADH dehydrogenase activity |  | **-1.25** |
| 1425505_AT | myosin, light polypeptide kinase | [MYLK](http://www.gene.ucl.ac.uk/cgi-bin/nomenclature/searchgenes.pl?field=symbol&anchor=equals&symbol_search=Search&number=100&format=html&sortby=symbol&match=MYLK) | oxidoreductase activity;NADH dehydrogenase activity | [600922](http://www.ncbi.nlm.nih.gov/entrez/dispomim.cgi?id=600922) | **1.64** |
| 1425506_AT | myosin, light polypeptide kinase | [MYLK](http://www.gene.ucl.ac.uk/cgi-bin/nomenclature/searchgenes.pl?field=symbol&anchor=equals&symbol_search=Search&number=100&format=html&sortby=symbol&match=MYLK) | oxidoreductase activity;NADH dehydrogenase activity | [600922](http://www.ncbi.nlm.nih.gov/entrez/dispomim.cgi?id=600922) | **1.87** |
| 1425519_A_AT | Ia-associated invariant chain | [II](http://www.gene.ucl.ac.uk/cgi-bin/nomenclature/searchgenes.pl?field=symbol&anchor=equals&symbol_search=Search&number=100&format=html&sortby=symbol&match=II) | oxidoreductase activity;NADH dehydrogenase activity |  | **-2.31** |
| 1425525_A_AT | purinergic receptor P2X, ligand-gated ion channel 4 | [P2RX4](http://www.gene.ucl.ac.uk/cgi-bin/nomenclature/searchgenes.pl?field=symbol&anchor=equals&symbol_search=Search&number=100&format=html&sortby=symbol&match=P2RX4) | oxidoreductase activity;NADH dehydrogenase activity | [600846](http://www.ncbi.nlm.nih.gov/entrez/dispomim.cgi?id=600846) | **-1.73** |
| 1425533_A_AT | staufen (RNA binding protein) homolog 2 (Drosophila) | [STAU2](http://www.gene.ucl.ac.uk/cgi-bin/nomenclature/searchgenes.pl?field=symbol&anchor=equals&symbol_search=Search&number=100&format=html&sortby=symbol&match=STAU2) | oxidoreductase activity;NADH dehydrogenase activity | [605920](http://www.ncbi.nlm.nih.gov/entrez/dispomim.cgi?id=605920) | **1.37** |
| 1425534_AT | Staufen (RNA binding protein) homolog 2 (Drosophila) | [STAU2](http://www.gene.ucl.ac.uk/cgi-bin/nomenclature/searchgenes.pl?field=symbol&anchor=equals&symbol_search=Search&number=100&format=html&sortby=symbol&match=STAU2) | oxidoreductase activity;NADH dehydrogenase activity | [605920](http://www.ncbi.nlm.nih.gov/entrez/dispomim.cgi?id=605920) | **1.41** |
| 1425538_X_AT | CEA-related cell adhesion molecule 1 | [CEACAM1](http://www.gene.ucl.ac.uk/cgi-bin/nomenclature/searchgenes.pl?field=symbol&anchor=equals&symbol_search=Search&number=100&format=html&sortby=symbol&match=CEACAM1) | oxidoreductase activity;NADH dehydrogenase activity | [109770](http://www.ncbi.nlm.nih.gov/entrez/dispomim.cgi?id=109770) | **1.9** |
| 1425543_S_AT | pleckstrin homology domain containing, family A member 5 | [PLEKHA5](http://www.gene.ucl.ac.uk/cgi-bin/nomenclature/searchgenes.pl?field=symbol&anchor=equals&symbol_search=Search&number=100&format=html&sortby=symbol&match=PLEKHA5) | oxidoreductase activity;oxidoreductase activity, acting on NADH or NADPH;NADH dehydrogenase activity | [607770](http://www.ncbi.nlm.nih.gov/entrez/dispomim.cgi?id=607770) | **-1.81** |
| 1425546_A_AT | transferrin | [TRF](http://www.gene.ucl.ac.uk/cgi-bin/nomenclature/searchgenes.pl?field=symbol&anchor=equals&symbol_search=Search&number=100&format=html&sortby=symbol&match=TRF) | oxidoreductase activity;protein-lysine 6-oxidase activity;copper ion binding |  | **1.7** |
| 1425548_A_AT | leukocyte specific transcript 1 | [LST1](http://www.gene.ucl.ac.uk/cgi-bin/nomenclature/searchgenes.pl?field=symbol&anchor=equals&symbol_search=Search&number=100&format=html&sortby=symbol&match=LST1) | oxidoreductase activity;protein-methionine-R-oxide reductase activity |  | **-3.91** |
| 1425575_AT | Eph receptor A3 | [EPHA3](http://www.gene.ucl.ac.uk/cgi-bin/nomenclature/searchgenes.pl?field=symbol&anchor=equals&symbol_search=Search&number=100&format=html&sortby=symbol&match=EPHA3) | oxidoreductase activity;protein-methionine-R-oxide reductase activity | [179611](http://www.ncbi.nlm.nih.gov/entrez/dispomim.cgi?id=179611) | **-2.01** |
| 1425582_A_AT | endomucin | [EMCN](http://www.gene.ucl.ac.uk/cgi-bin/nomenclature/searchgenes.pl?field=symbol&anchor=equals&symbol_search=Search&number=100&format=html&sortby=symbol&match=EMCN) | oxidoreductase activity;transferase activity | [608350](http://www.ncbi.nlm.nih.gov/entrez/dispomim.cgi?id=608350) | **1.69** |
| 1425597_A_AT | quaking | [QK](http://www.gene.ucl.ac.uk/cgi-bin/nomenclature/searchgenes.pl?field=symbol&anchor=equals&symbol_search=Search&number=100&format=html&sortby=symbol&match=QK) | oxidoreductase activity;transferase activity |  | **1.51** |
| 1425603_AT | RIKEN cDNA 0610011I04 gene | [0610011I04RIK](http://www.gene.ucl.ac.uk/cgi-bin/nomenclature/searchgenes.pl?field=symbol&anchor=equals&symbol_search=Search&number=100&format=html&sortby=symbol&match=0610011I04RIK) | oxidoreductase activity;ubiquinol-cytochrome-c reductase activity |  | **-1.93** |
| 1425616_A_AT | RIKEN cDNA 2410005K17 gene | [2410005K17RIK](http://www.gene.ucl.ac.uk/cgi-bin/nomenclature/searchgenes.pl?field=symbol&anchor=equals&symbol_search=Search&number=100&format=html&sortby=symbol&match=2410005K17RIK) | oxidoreductase activity;ubiquinol-cytochrome-c reductase activity |  | **-1.99** |
| 1425620_AT | transforming growth factor, beta receptor III | [TGFBR3](http://www.gene.ucl.ac.uk/cgi-bin/nomenclature/searchgenes.pl?field=symbol&anchor=equals&symbol_search=Search&number=100&format=html&sortby=symbol&match=TGFBR3) | oxidoreductase activity;ubiquinol-cytochrome-c reductase activity | [600742](http://www.ncbi.nlm.nih.gov/entrez/dispomim.cgi?id=600742) | **1.65** |
| 1425627_X_AT | glutathione S-transferase, mu 1 | [GSTM1](http://www.gene.ucl.ac.uk/cgi-bin/nomenclature/searchgenes.pl?field=symbol&anchor=equals&symbol_search=Search&number=100&format=html&sortby=symbol&match=GSTM1) | oxidoreductase activity;ubiquinol-cytochrome-c reductase activity |  | **1.69** |
| 1425652_S_AT | RNA binding protein gene with multiple splicing | [RBPMS](http://www.gene.ucl.ac.uk/cgi-bin/nomenclature/searchgenes.pl?field=symbol&anchor=equals&symbol_search=Search&number=100&format=html&sortby=symbol&match=RBPMS) | oxidoreductase activity;ubiquinol-cytochrome-c reductase activity | [601558](http://www.ncbi.nlm.nih.gov/entrez/dispomim.cgi?id=601558) | **1.87** |
| 1425676_A_AT | elongation of very long chain fatty acids (FEN1/Elo2, SUR4/Elo3, yeast)-like 1 | [ELOVL1](http://www.gene.ucl.ac.uk/cgi-bin/nomenclature/searchgenes.pl?field=symbol&anchor=equals&symbol_search=Search&number=100&format=html&sortby=symbol&match=ELOVL1) | oxoglutarate dehydrogenase (succinyl-transferring) activity;oxidoreductase activity, acting on the aldehyde or oxo group of donors, disulfide as acceptor;oxidoreductase activity |  | **-1.25** |
| 1425678_A_AT | SNF related kinase | [SNRK](http://www.gene.ucl.ac.uk/cgi-bin/nomenclature/searchgenes.pl?field=symbol&anchor=equals&symbol_search=Search&number=100&format=html&sortby=symbol&match=SNRK) | oxygen transporter activity |  | **1.77** |
| 1425704_AT | cDNA sequence BC022224 | [BC022224](http://www.gene.ucl.ac.uk/cgi-bin/nomenclature/searchgenes.pl?field=symbol&anchor=equals&symbol_search=Search&number=100&format=html&sortby=symbol&match=BC022224) | oxygen transporter activity |  | **1.28** |
| 1425718_A_AT | influenza virus NS1A binding protein | [IVNS1ABP](http://www.gene.ucl.ac.uk/cgi-bin/nomenclature/searchgenes.pl?field=symbol&anchor=equals&symbol_search=Search&number=100&format=html&sortby=symbol&match=IVNS1ABP) | pantothenate kinase activity;molecular_function unknown;kinase activity |  | **1.47** |
| 1425730_AT | calcium channel, voltage-dependent, gamma subunit 6 | [CACNG6](http://www.gene.ucl.ac.uk/cgi-bin/nomenclature/searchgenes.pl?field=symbol&anchor=equals&symbol_search=Search&number=100&format=html&sortby=symbol&match=CACNG6) | PDZ domain binding | [606898](http://www.ncbi.nlm.nih.gov/entrez/dispomim.cgi?id=606898) | **1.39** |
| 1425732_A_AT | Max interacting protein 1 | [MXI1](http://www.gene.ucl.ac.uk/cgi-bin/nomenclature/searchgenes.pl?field=symbol&anchor=equals&symbol_search=Search&number=100&format=html&sortby=symbol&match=MXI1) | PDZ domain binding;protein heterodimerization activity;chloride channel activity;voltage-gated chloride channel activity;ion channel activity;voltage-gated ion channel activity;protein homodimerization activity | [600020](http://www.ncbi.nlm.nih.gov/entrez/dispomim.cgi?id=600020) | **1.44** |
| 1425742_A_AT | transforming growth factor beta 1 induced transcript 4 | [TGFB1I4](http://www.gene.ucl.ac.uk/cgi-bin/nomenclature/searchgenes.pl?field=symbol&anchor=equals&symbol_search=Search&number=100&format=html&sortby=symbol&match=TGFB1I4) | PDZ domain binding;protein heterodimerization activity;chloride channel activity;voltage-gated chloride channel activity;ion channel activity;voltage-gated ion channel activity;protein homodimerization activity | [607715](http://www.ncbi.nlm.nih.gov/entrez/dispomim.cgi?id=607715) | **-1.37** |
| 1425753_A_AT | uracil-DNA glycosylase | [UNG](http://www.gene.ucl.ac.uk/cgi-bin/nomenclature/searchgenes.pl?field=symbol&anchor=equals&symbol_search=Search&number=100&format=html&sortby=symbol&match=UNG) | phosphate transporter activity | [191525](http://www.ncbi.nlm.nih.gov/entrez/dispomim.cgi?id=191525) | **2.12** |
| 1425764_A_AT | branched chain aminotransferase 2, mitochondrial | [BCAT2](http://www.gene.ucl.ac.uk/cgi-bin/nomenclature/searchgenes.pl?field=symbol&anchor=equals&symbol_search=Search&number=100&format=html&sortby=symbol&match=BCAT2) | phosphatidate phosphatase activity;hydrolase activity | [113530](http://www.ncbi.nlm.nih.gov/entrez/dispomim.cgi?id=113530) | **1.82** |
| 1425767_A_AT | sine oculis-related homeobox 4 homolog (Drosophila) | [SIX4](http://www.gene.ucl.ac.uk/cgi-bin/nomenclature/searchgenes.pl?field=symbol&anchor=equals&symbol_search=Search&number=100&format=html&sortby=symbol&match=SIX4) | phosphatidylcholine-sterol O-acyltransferase activity;calcium-independent phospholipase A2 activity;acyltransferase activity;hydrolase activity;lysophospholipase activity;transferase activity | [606342](http://www.ncbi.nlm.nih.gov/entrez/dispomim.cgi?id=606342) | **1.25** |
| 1425773_S_AT | nicotinamide nucleotide adenylyltransferase 1 | [UBE4B](http://www.gene.ucl.ac.uk/cgi-bin/nomenclature/searchgenes.pl?field=symbol&anchor=equals&symbol_search=Search&number=100&format=html&sortby=symbol&match=UBE4B) | phosphatidylinositol transporter activity;phosphatidylinositol binding |  | **2.09** |
| 1425779_A_AT | T-box 1 | [TBX1](http://www.gene.ucl.ac.uk/cgi-bin/nomenclature/searchgenes.pl?field=symbol&anchor=equals&symbol_search=Search&number=100&format=html&sortby=symbol&match=TBX1) | phosphatidylinositol transporter activity;phosphatidylinositol binding | [602054](http://www.ncbi.nlm.nih.gov/entrez/dispomim.cgi?id=602054) | **1.64** |
| 1425780_A_AT | RIKEN cDNA 0610041E09 gene | [0610041E09RIK](http://www.gene.ucl.ac.uk/cgi-bin/nomenclature/searchgenes.pl?field=symbol&anchor=equals&symbol_search=Search&number=100&format=html&sortby=symbol&match=0610041E09RIK) | phosphatidylinositol transporter activity;phosphatidylinositol binding |  | **-1.56** |
| 1425784_A_AT | olfactomedin 1 | [OLFM1](http://www.gene.ucl.ac.uk/cgi-bin/nomenclature/searchgenes.pl?field=symbol&anchor=equals&symbol_search=Search&number=100&format=html&sortby=symbol&match=OLFM1) | phosphatidylinositol transporter activity;phosphatidylinositol binding | [605366](http://www.ncbi.nlm.nih.gov/entrez/dispomim.cgi?id=605366) | **-1.8** |
| 1425792_A_AT | RAR-related orphan receptor gamma | [RORC](http://www.gene.ucl.ac.uk/cgi-bin/nomenclature/searchgenes.pl?field=symbol&anchor=equals&symbol_search=Search&number=100&format=html&sortby=symbol&match=RORC) | phosphatidylinositol transporter activity;phosphatidylinositol binding | [602943](http://www.ncbi.nlm.nih.gov/entrez/dispomim.cgi?id=602943) | **2.78** |
| 1425809_AT | Fatty acid binding protein 4, adipocyte | [FABP4](http://www.gene.ucl.ac.uk/cgi-bin/nomenclature/searchgenes.pl?field=symbol&anchor=equals&symbol_search=Search&number=100&format=html&sortby=symbol&match=FABP4) | phosphogluconate dehydrogenase (decarboxylating) activity;3-hydroxyisobutyrate dehydrogenase activity;oxidoreductase activity | [600434](http://www.ncbi.nlm.nih.gov/entrez/dispomim.cgi?id=600434) | **1.82** |
| 1425810_A_AT | cysteine and glycine-rich protein 1 | [CSRP1](http://www.gene.ucl.ac.uk/cgi-bin/nomenclature/searchgenes.pl?field=symbol&anchor=equals&symbol_search=Search&number=100&format=html&sortby=symbol&match=CSRP1) | phosphogluconate dehydrogenase (decarboxylating) activity;oxidoreductase activity;3-hydroxyisobutyrate dehydrogenase activity | [123876](http://www.ncbi.nlm.nih.gov/entrez/dispomim.cgi?id=123876) | **1.35** |
| 1425834_A_AT | glycerol-3-phosphate acyltransferase, mitochondrial | [GPAM](http://www.gene.ucl.ac.uk/cgi-bin/nomenclature/searchgenes.pl?field=symbol&anchor=equals&symbol_search=Search&number=100&format=html&sortby=symbol&match=GPAM) | phosphoglycerate dehydrogenase activity;oxidoreductase activity;oxidoreductase activity, acting on the CH-OH group of donors, NAD or NADP as acceptor | [602395](http://www.ncbi.nlm.nih.gov/entrez/dispomim.cgi?id=602395) | **2.04** |
| 1425848_A_AT | RIKEN cDNA 2310043K02 gene | [2310043K02RIK](http://www.gene.ucl.ac.uk/cgi-bin/nomenclature/searchgenes.pl?field=symbol&anchor=equals&symbol_search=Search&number=100&format=html&sortby=symbol&match=2310043K02RIK) | phosphoglycerate dehydrogenase activity;oxidoreductase activity;oxidoreductase activity, acting on the CH-OH group of donors, NAD or NADP as acceptor |  | **6.87** |
| 1425891_A_AT | GH regulated TBC protein 1 | [GRTP1](http://www.gene.ucl.ac.uk/cgi-bin/nomenclature/searchgenes.pl?field=symbol&anchor=equals&symbol_search=Search&number=100&format=html&sortby=symbol&match=GRTP1) | phospholipase A2 inhibitor activity;protein binding;phospholipase inhibitor activity;calcium-dependent phospholipid binding;calcium ion binding |  | **2.2** |
| 1425895_A_AT | inhibitor of DNA binding 1 | [IDB1](http://www.gene.ucl.ac.uk/cgi-bin/nomenclature/searchgenes.pl?field=symbol&anchor=equals&symbol_search=Search&number=100&format=html&sortby=symbol&match=IDB1) | phospholipase activity;subtilase activity;phospholipase A2 activity;hydrolase activity;lysophospholipase activity |  | **1.84** |
| 1425896_A_AT | fibrillin 1 | [FBN1](http://www.gene.ucl.ac.uk/cgi-bin/nomenclature/searchgenes.pl?field=symbol&anchor=equals&symbol_search=Search&number=100&format=html&sortby=symbol&match=FBN1) | phospholipase inhibitor activity;protein binding;calcium-dependent phospholipid binding;calcium ion binding;cytoskeletal protein binding | [134797](http://www.ncbi.nlm.nih.gov/entrez/dispomim.cgi?id=134797) | **-1.48** |
| 1425902_A_AT | nuclear factor of kappa light polypeptide gene enhancer in B-cells 2, p49/p100 | [NFKB2](http://www.gene.ucl.ac.uk/cgi-bin/nomenclature/searchgenes.pl?field=symbol&anchor=equals&symbol_search=Search&number=100&format=html&sortby=symbol&match=NFKB2) | phospholipid binding | [164012](http://www.ncbi.nlm.nih.gov/entrez/dispomim.cgi?id=164012) | **-1.81** |
| 1425914_A_AT | armadillo repeat containing, X-linked 1 | [ARMCX1](http://www.gene.ucl.ac.uk/cgi-bin/nomenclature/searchgenes.pl?field=symbol&anchor=equals&symbol_search=Search&number=100&format=html&sortby=symbol&match=ARMCX1) | phosphoprotein phosphatase activity | [300362](http://www.ncbi.nlm.nih.gov/entrez/dispomim.cgi?id=300362) | **-1.56** |
| 1425921_A_AT | RIKEN cDNA 1810055G02 gene | [1810055G02RIK](http://www.gene.ucl.ac.uk/cgi-bin/nomenclature/searchgenes.pl?field=symbol&anchor=equals&symbol_search=Search&number=100&format=html&sortby=symbol&match=1810055G02RIK) | phosphoprotein phosphatase activity;MAP kinase phosphatase activity;protein tyrosine/serine/threonine phosphatase activity;protein tyrosine phosphatase activity;hydrolase activity |  | **-2.35** |
| 1425927_A_AT | activating transcription factor 5 | [ATF5](http://www.gene.ucl.ac.uk/cgi-bin/nomenclature/searchgenes.pl?field=symbol&anchor=equals&symbol_search=Search&number=100&format=html&sortby=symbol&match=ATF5) | phosphoprotein phosphatase activity;MAP kinase phosphatase activity;protein tyrosine/serine/threonine phosphatase activity;protein tyrosine phosphatase activity;hydrolase activity | [606398](http://www.ncbi.nlm.nih.gov/entrez/dispomim.cgi?id=606398) | **1.52** |
| 1425933_A_AT | 5'-nucleotidase, cytosolic II | [NT5C2](http://www.gene.ucl.ac.uk/cgi-bin/nomenclature/searchgenes.pl?field=symbol&anchor=equals&symbol_search=Search&number=100&format=html&sortby=symbol&match=NT5C2) | phosphoprotein phosphatase activity;phosphohistidine phosphatase activity;hydrolase activity | [600417](http://www.ncbi.nlm.nih.gov/entrez/dispomim.cgi?id=600417) | **-1.93** |
| 1425942_A_AT | glycoprotein m6b | [GPM6B](http://www.gene.ucl.ac.uk/cgi-bin/nomenclature/searchgenes.pl?field=symbol&anchor=equals&symbol_search=Search&number=100&format=html&sortby=symbol&match=GPM6B) | phosphoprotein phosphatase activity;protein binding;kinase activity;guanylate kinase activity;protein tyrosine/serine/threonine phosphatase activity;hydrolase activity | [300051](http://www.ncbi.nlm.nih.gov/entrez/dispomim.cgi?id=300051) | **-1.91** |
| 1425974_A_AT | tripartite motif protein 25 | [TRIM25](http://www.gene.ucl.ac.uk/cgi-bin/nomenclature/searchgenes.pl?field=symbol&anchor=equals&symbol_search=Search&number=100&format=html&sortby=symbol&match=TRIM25) | phosphoprotein phosphatase activity;protein binding;protein serine/threonine phosphatase activity;manganese ion binding;hydrolase activity | [600453](http://www.ncbi.nlm.nih.gov/entrez/dispomim.cgi?id=600453) | **-1.57** |
| 1425977_A_AT | gb:AF112855.1 /DB_XREF=gi:4741822 /GEN=SMAK /FEA=F |  | phosphoprotein phosphatase activity;protein binding;protein serine/threonine phosphatase activity;protein phosphatase type 2C activity;magnesium ion binding;manganese ion binding;catalytic activity;hydrolase activity |  | **-1.39** |
| 1425981_A_AT | retinoblastoma-like 2 | [RBL2](http://www.gene.ucl.ac.uk/cgi-bin/nomenclature/searchgenes.pl?field=symbol&anchor=equals&symbol_search=Search&number=100&format=html&sortby=symbol&match=RBL2) | phosphoprotein phosphatase activity;protein binding;receptor activity;protein tyrosine phosphatase activity;hydrolase activity | [180203](http://www.ncbi.nlm.nih.gov/entrez/dispomim.cgi?id=180203) | **1.28** |
| 1426024_A_AT | drebrin 1 | [DBN1](http://www.gene.ucl.ac.uk/cgi-bin/nomenclature/searchgenes.pl?field=symbol&anchor=equals&symbol_search=Search&number=100&format=html&sortby=symbol&match=DBN1) | phosphoprotein phosphatase activity;protein binding;receptor activity;protein tyrosine phosphatase activity;non-membrane spanning protein tyrosine phosphatase activity;hydrolase activity | [126660](http://www.ncbi.nlm.nih.gov/entrez/dispomim.cgi?id=126660) | **-1.45** |
| 1426025_S_AT | lysosomal-associated protein transmembrane 5 | [LAPTM5](http://www.gene.ucl.ac.uk/cgi-bin/nomenclature/searchgenes.pl?field=symbol&anchor=equals&symbol_search=Search&number=100&format=html&sortby=symbol&match=LAPTM5) | phosphoprotein phosphatase activity;protein homodimerization activity;protein tyrosine phosphatase activity;hydrolase activity | [601476](http://www.ncbi.nlm.nih.gov/entrez/dispomim.cgi?id=601476) | **-2.56** |
| 1426027_A_AT | Rho GTPase activating protein 10 | [ARHGAP10](http://www.gene.ucl.ac.uk/cgi-bin/nomenclature/searchgenes.pl?field=symbol&anchor=equals&symbol_search=Search&number=100&format=html&sortby=symbol&match=ARHGAP10) | phosphoprotein phosphatase activity;protein homodimerization activity;protein tyrosine phosphatase activity;hydrolase activity |  | **1.38** |
| 1426043_A_AT | calpain 3 | [CAPN3](http://www.gene.ucl.ac.uk/cgi-bin/nomenclature/searchgenes.pl?field=symbol&anchor=equals&symbol_search=Search&number=100&format=html&sortby=symbol&match=CAPN3) | phosphoprotein phosphatase activity;protein kinase activity;kinase activity;protein serine/threonine kinase activity;receptor activity;ATP binding;calmodulin binding;transferase activity | [114240](http://www.ncbi.nlm.nih.gov/entrez/dispomim.cgi?id=114240) | **1.41** |
| 1426063_A_AT | GTP binding protein (gene overexpressed in skeletal muscle) | [GEM](http://www.gene.ucl.ac.uk/cgi-bin/nomenclature/searchgenes.pl?field=symbol&anchor=equals&symbol_search=Search&number=100&format=html&sortby=symbol&match=GEM) | phosphoprotein phosphatase activity;protein kinase activity;kinase activity;protein serine/threonine kinase activity;receptor activity;ATP binding;calmodulin binding;transferase activity | [600164](http://www.ncbi.nlm.nih.gov/entrez/dispomim.cgi?id=600164) | **-1.73** |
| 1426081_A_AT | deiodinase, iodothyronine, type II | [DIO2](http://www.gene.ucl.ac.uk/cgi-bin/nomenclature/searchgenes.pl?field=symbol&anchor=equals&symbol_search=Search&number=100&format=html&sortby=symbol&match=DIO2) | phosphoprotein phosphatase activity;protein phosphatase type 2A regulator activity | [601413](http://www.ncbi.nlm.nih.gov/entrez/dispomim.cgi?id=601413) | **-2.37** |
| 1426083_A_AT | B-cell translocation gene 1, anti-proliferative | [BTG1](http://www.gene.ucl.ac.uk/cgi-bin/nomenclature/searchgenes.pl?field=symbol&anchor=equals&symbol_search=Search&number=100&format=html&sortby=symbol&match=BTG1) | phosphoprotein phosphatase activity;protein serine/threonine phosphatase activity;calmodulin binding;hydrolase activity | [109580](http://www.ncbi.nlm.nih.gov/entrez/dispomim.cgi?id=109580) | **-1.69** |
| 1426084_A_AT | hypothetical protein MGC6357 | [MGC6357](http://www.gene.ucl.ac.uk/cgi-bin/nomenclature/searchgenes.pl?field=symbol&anchor=equals&symbol_search=Search&number=100&format=html&sortby=symbol&match=MGC6357) | phosphoprotein phosphatase activity;protein serine/threonine phosphatase activity;magnesium ion binding;manganese ion binding;catalytic activity;hydrolase activity |  | **1.34** |
| 1426095_A_AT | tumor necrosis factor receptor superfamily, member 22 | [TNFRSF22](http://www.gene.ucl.ac.uk/cgi-bin/nomenclature/searchgenes.pl?field=symbol&anchor=equals&symbol_search=Search&number=100&format=html&sortby=symbol&match=TNFRSF22) | phosphoprotein phosphatase activity;protein serine/threonine phosphatase activity;magnesium ion binding;manganese ion binding;catalytic activity;hydrolase activity |  | **-2.13** |
| 1426098_A_AT | calpastatin | [CAST](http://www.gene.ucl.ac.uk/cgi-bin/nomenclature/searchgenes.pl?field=symbol&anchor=equals&symbol_search=Search&number=100&format=html&sortby=symbol&match=CAST) | phosphoprotein phosphatase activity;protein serine/threonine phosphatase activity;magnesium ion binding;manganese ion binding;catalytic activity;hydrolase activity | [114090](http://www.ncbi.nlm.nih.gov/entrez/dispomim.cgi?id=114090) | **1.27** |
| 1426112_A_AT | gb:BC003824.1 /DB_XREF=gi:13277905 /FEA=FLmRNA /CN |  | phosphoprotein phosphatase activity;protein serine/threonine phosphatase activity;magnesium ion binding;manganese ion binding;catalytic activity;hydrolase activity |  | **-2.31** |
| 1426116_AT | ankyrin repeat and SOCS box-containing 18 | [ASB18](http://www.gene.ucl.ac.uk/cgi-bin/nomenclature/searchgenes.pl?field=symbol&anchor=equals&symbol_search=Search&number=100&format=html&sortby=symbol&match=ASB18) | phosphoprotein phosphatase activity;protein tyrosine phosphatase activity;hydrolase activity |  | **1.77** |
| 1426122_A_AT | coronin, actin binding protein 6 | [CORO6](http://www.gene.ucl.ac.uk/cgi-bin/nomenclature/searchgenes.pl?field=symbol&anchor=equals&symbol_search=Search&number=100&format=html&sortby=symbol&match=CORO6) | phosphoprotein phosphatase activity;protein tyrosine/serine/threonine phosphatase activity;hydrolase activity |  | **1.56** |
| 1426146_A_AT | choline phosphotransferase 1 | [CHPT1](http://www.gene.ucl.ac.uk/cgi-bin/nomenclature/searchgenes.pl?field=symbol&anchor=equals&symbol_search=Search&number=100&format=html&sortby=symbol&match=CHPT1) | phosphoprotein phosphatase activity;protein tyrosine/serine/threonine phosphatase activity;hydrolase activity |  | **2.15** |
| 1426152_A_AT | kit ligand | [KITL](http://www.gene.ucl.ac.uk/cgi-bin/nomenclature/searchgenes.pl?field=symbol&anchor=equals&symbol_search=Search&number=100&format=html&sortby=symbol&match=KITL) | phosphoprotein phosphatase activity;protein tyrosine/serine/threonine phosphatase activity;hydrolase activity |  | **1.6** |
| 1426162_A_AT | ribosomal protein L7 | [RPL7](http://www.gene.ucl.ac.uk/cgi-bin/nomenclature/searchgenes.pl?field=symbol&anchor=equals&symbol_search=Search&number=100&format=html&sortby=symbol&match=RPL7) | phosphoprotein phosphatase activity;protein tyrosine/serine/threonine phosphatase activity;hydrolase activity | [604166](http://www.ncbi.nlm.nih.gov/entrez/dispomim.cgi?id=604166) | **-1.34** |
| 1426163_X_AT | ribosomal protein L7 | [RPL7](http://www.gene.ucl.ac.uk/cgi-bin/nomenclature/searchgenes.pl?field=symbol&anchor=equals&symbol_search=Search&number=100&format=html&sortby=symbol&match=RPL7) | phosphoprotein phosphatase activity;protein tyrosine/serine/threonine phosphatase activity;protein tyrosine phosphatase activity | [604166](http://www.ncbi.nlm.nih.gov/entrez/dispomim.cgi?id=604166) | **-1.39** |
| 1426165_A_AT | caspase 3, apoptosis related cysteine protease | [CASP3](http://www.gene.ucl.ac.uk/cgi-bin/nomenclature/searchgenes.pl?field=symbol&anchor=equals&symbol_search=Search&number=100&format=html&sortby=symbol&match=CASP3) | phosphoprotein phosphatase activity;receptor activity;protein tyrosine phosphatase activity;hydrolase activity | [600636](http://www.ncbi.nlm.nih.gov/entrez/dispomim.cgi?id=600636) | **-3.34** |
| 1426172_A_AT | CD209a antigen | [CD209A](http://www.gene.ucl.ac.uk/cgi-bin/nomenclature/searchgenes.pl?field=symbol&anchor=equals&symbol_search=Search&number=100&format=html&sortby=symbol&match=CD209A) | phosphoprotein phosphatase activity;receptor activity;protein tyrosine phosphatase activity;hydrolase activity |  | **-2.02** |
| 1426195_A_AT | cystatin C | [CST3](http://www.gene.ucl.ac.uk/cgi-bin/nomenclature/searchgenes.pl?field=symbol&anchor=equals&symbol_search=Search&number=100&format=html&sortby=symbol&match=CST3) | phosphoprotein phosphatase activity;receptor activity;protein tyrosine phosphatase activity;hydrolase activity | [604312](http://www.ncbi.nlm.nih.gov/entrez/dispomim.cgi?id=604312) | **-1.27** |
| 1426205_AT | protein phosphatase 1, catalytic subunit, beta isoform | [PPP1CB](http://www.gene.ucl.ac.uk/cgi-bin/nomenclature/searchgenes.pl?field=symbol&anchor=equals&symbol_search=Search&number=100&format=html&sortby=symbol&match=PPP1CB) | phosphoprotein phosphatase activity;receptor activity;protein tyrosine phosphatase activity;hydrolase activity | [600590](http://www.ncbi.nlm.nih.gov/entrez/dispomim.cgi?id=600590) | **1.21** |
| 1426206_AT | roundabout homolog 4 (Drosophila) | [ROBO4](http://www.gene.ucl.ac.uk/cgi-bin/nomenclature/searchgenes.pl?field=symbol&anchor=equals&symbol_search=Search&number=100&format=html&sortby=symbol&match=ROBO4) | phosphoprotein phosphatase activity;sugar binding;receptor activity;protein tyrosine phosphatase activity;hydrolase activity | [607528](http://www.ncbi.nlm.nih.gov/entrez/dispomim.cgi?id=607528) | **2.21** |
| 1426207_AT | inhibitor of kappaB kinase beta | [IKBKB](http://www.gene.ucl.ac.uk/cgi-bin/nomenclature/searchgenes.pl?field=symbol&anchor=equals&symbol_search=Search&number=100&format=html&sortby=symbol&match=IKBKB) | phosphoprotein phosphatase activity;sugar binding;receptor activity;protein tyrosine phosphatase activity;hydrolase activity | [603258](http://www.ncbi.nlm.nih.gov/entrez/dispomim.cgi?id=603258) | **-1.31** |
| 1426208_X_AT | pleiomorphic adenoma gene-like 1 | [PLAGL1](http://www.gene.ucl.ac.uk/cgi-bin/nomenclature/searchgenes.pl?field=symbol&anchor=equals&symbol_search=Search&number=100&format=html&sortby=symbol&match=PLAGL1) | phosphoprotein phosphatase activity;zinc ion binding;carbonate dehydratase activity;protein tyrosine phosphatase activity;hydrolase activity |  | **-3.24** |
| 1426209_AT | striatin, calmodulin binding protein 4 | [STRN4](http://www.gene.ucl.ac.uk/cgi-bin/nomenclature/searchgenes.pl?field=symbol&anchor=equals&symbol_search=Search&number=100&format=html&sortby=symbol&match=STRN4) | phosphoribosylformylglycinamidine cyclo-ligase activity;hydroxymethyl-, formyl- and related transferase activity;phosphoribosylglycinamide formyltransferase activity;ligase activity;phosphoribosylamine-glycine ligase activity;catalytic activity;transferase activity |  | **-1.32** |
| 1426215_AT | gb:AF071068.1 /DB_XREF=gi:3288844 /FEA=FLmRNA /CNT |  | phosphoric ester hydrolase activity;fructose-bisphosphatase activity;hydrolase activity |  | **-3.36** |
| 1426221_AT | loss of heterozygosity, 11, chromosomal region 2, gene A homolog (human) | [LOH11CR2A](http://www.gene.ucl.ac.uk/cgi-bin/nomenclature/searchgenes.pl?field=symbol&anchor=equals&symbol_search=Search&number=100&format=html&sortby=symbol&match=LOH11CR2A) | phosphoric ester hydrolase activity;inositol/phosphatidylinositol phosphatase activity;magnesium ion binding;inositol-1(or 4)-monophosphatase activity;hydrolase activity | [602929](http://www.ncbi.nlm.nih.gov/entrez/dispomim.cgi?id=602929) | **-1.57** |
| 1426238_AT | bone morphogenetic protein 1 | [BMP1](http://www.gene.ucl.ac.uk/cgi-bin/nomenclature/searchgenes.pl?field=symbol&anchor=equals&symbol_search=Search&number=100&format=html&sortby=symbol&match=BMP1) | phosphoserine transaminase activity;transaminase activity;transferase activity | [112264](http://www.ncbi.nlm.nih.gov/entrez/dispomim.cgi?id=112264) | **-1.63** |
| 1426239_S_AT | arrestin, beta 2 | [ARRB2](http://www.gene.ucl.ac.uk/cgi-bin/nomenclature/searchgenes.pl?field=symbol&anchor=equals&symbol_search=Search&number=100&format=html&sortby=symbol&match=ARRB2) | phosphoserine transaminase activity;transaminase activity;transferase activity | [107941](http://www.ncbi.nlm.nih.gov/entrez/dispomim.cgi?id=107941) | **-2.14** |
| 1426246_AT | protein S (alpha) | [PROS1](http://www.gene.ucl.ac.uk/cgi-bin/nomenclature/searchgenes.pl?field=symbol&anchor=equals&symbol_search=Search&number=100&format=html&sortby=symbol&match=PROS1) | phosphotransferase activity, alcohol group as acceptor;oxidoreductase activity;ATP binding;NADH dehydrogenase activity | [176880](http://www.ncbi.nlm.nih.gov/entrez/dispomim.cgi?id=176880) | **-1.45** |
| 1426248_AT | serine/threonine kinase 24 (STE20 homolog, yeast) | [STK24](http://www.gene.ucl.ac.uk/cgi-bin/nomenclature/searchgenes.pl?field=symbol&anchor=equals&symbol_search=Search&number=100&format=html&sortby=symbol&match=STK24) | platelet-derived growth factor receptor binding;protein binding;growth factor activity | [604984](http://www.ncbi.nlm.nih.gov/entrez/dispomim.cgi?id=604984) | **1.42** |
| 1426256_AT | translocator of inner mitochondrial membrane 17a | [TIMM17A](http://www.gene.ucl.ac.uk/cgi-bin/nomenclature/searchgenes.pl?field=symbol&anchor=equals&symbol_search=Search&number=100&format=html&sortby=symbol&match=TIMM17A) | platelet-derived growth factor receptor binding;protein binding;growth factor activity | [605057](http://www.ncbi.nlm.nih.gov/entrez/dispomim.cgi?id=605057) | **1.33** |
| 1426258_AT | sortilin-related receptor, LDLR class A repeats-containing | [SORL1](http://www.gene.ucl.ac.uk/cgi-bin/nomenclature/searchgenes.pl?field=symbol&anchor=equals&symbol_search=Search&number=100&format=html&sortby=symbol&match=SORL1) | poly(A) binding;RNA binding | [602005](http://www.ncbi.nlm.nih.gov/entrez/dispomim.cgi?id=602005) | **1.61** |
| 1426306_A_AT | melanoma antigen, family D, 2 | [MAGED2](http://www.gene.ucl.ac.uk/cgi-bin/nomenclature/searchgenes.pl?field=symbol&anchor=equals&symbol_search=Search&number=100&format=html&sortby=symbol&match=MAGED2) | poly(A) binding;RNA binding;poly(rC) binding;poly(U) binding | [300470](http://www.ncbi.nlm.nih.gov/entrez/dispomim.cgi?id=300470) | **-4.6** |
| 1426315_A_AT | RIKEN cDNA 6330416G13 gene | [6330416G13RIK](http://www.gene.ucl.ac.uk/cgi-bin/nomenclature/searchgenes.pl?field=symbol&anchor=equals&symbol_search=Search&number=100&format=html&sortby=symbol&match=6330416G13RIK) | poly(ADP-ribose) glycohydrolase activity;hydrolase activity |  | **1.7** |
| 1426319_AT | platelet-derived growth factor, D polypeptide | [PDGFD](http://www.gene.ucl.ac.uk/cgi-bin/nomenclature/searchgenes.pl?field=symbol&anchor=equals&symbol_search=Search&number=100&format=html&sortby=symbol&match=PDGFD) | poly-pyrimidine tract binding |  | **1.82** |
| 1426321_AT | T-box 4 | [TBX4](http://www.gene.ucl.ac.uk/cgi-bin/nomenclature/searchgenes.pl?field=symbol&anchor=equals&symbol_search=Search&number=100&format=html&sortby=symbol&match=TBX4) | poly-pyrimidine tract binding | [601719](http://www.ncbi.nlm.nih.gov/entrez/dispomim.cgi?id=601719) | **-1.95** |
| 1426345_AT | RIKEN cDNA D030028O16 gene | [D030028O16RIK](http://www.gene.ucl.ac.uk/cgi-bin/nomenclature/searchgenes.pl?field=symbol&anchor=equals&symbol_search=Search&number=100&format=html&sortby=symbol&match=D030028O16RIK) | polysaccharide binding;zymosan binding;zymosan receptor activity;sugar binding;opsonin binding |  | **-1.48** |
| 1426351_AT | heat shock protein 1 (chaperonin) | [HSPD1](http://www.gene.ucl.ac.uk/cgi-bin/nomenclature/searchgenes.pl?field=symbol&anchor=equals&symbol_search=Search&number=100&format=html&sortby=symbol&match=HSPD1) | porin activity | [118190](http://www.ncbi.nlm.nih.gov/entrez/dispomim.cgi?id=118190) | **1.29** |
| 1426353_AT | signal transducer and activator of transcription 6 | [STAT6](http://www.gene.ucl.ac.uk/cgi-bin/nomenclature/searchgenes.pl?field=symbol&anchor=equals&symbol_search=Search&number=100&format=html&sortby=symbol&match=STAT6) | porin activity | [601512](http://www.ncbi.nlm.nih.gov/entrez/dispomim.cgi?id=601512) | **-1.25** |
| 1426355_A_AT | RIKEN cDNA 1700001A24 gene | [1700001A24RIK](http://www.gene.ucl.ac.uk/cgi-bin/nomenclature/searchgenes.pl?field=symbol&anchor=equals&symbol_search=Search&number=100&format=html&sortby=symbol&match=1700001A24RIK) | porin activity;nucleocytoplasmic transporter activity;protein transporter activity |  | **1.31** |
| 1426356_AT | RIKEN cDNA 6330578E17 gene | [6330578E17RIK](http://www.gene.ucl.ac.uk/cgi-bin/nomenclature/searchgenes.pl?field=symbol&anchor=equals&symbol_search=Search&number=100&format=html&sortby=symbol&match=6330578E17RIK) | porin activity;voltage-dependent ion-selective channel activity |  | **1.33** |
| 1426369_AT | male sterility domain containing 2 | [MLSTD2](http://www.gene.ucl.ac.uk/cgi-bin/nomenclature/searchgenes.pl?field=symbol&anchor=equals&symbol_search=Search&number=100&format=html&sortby=symbol&match=MLSTD2) | porin activity;voltage-dependent ion-selective channel activity |  | **-1.59** |
| 1426370_AT | male sterility domain containing 2 | [MLSTD2](http://www.gene.ucl.ac.uk/cgi-bin/nomenclature/searchgenes.pl?field=symbol&anchor=equals&symbol_search=Search&number=100&format=html&sortby=symbol&match=MLSTD2) | porin activity;voltage-dependent ion-selective channel activity |  | **-1.4** |
| 1426371_AT | male sterility domain containing 2 | [MLSTD2](http://www.gene.ucl.ac.uk/cgi-bin/nomenclature/searchgenes.pl?field=symbol&anchor=equals&symbol_search=Search&number=100&format=html&sortby=symbol&match=MLSTD2) | porphobilinogen synthase activity;lyase activity |  | **-1.53** |
| 1426374_AT | RIKEN cDNA 2410166I05 gene | [2410166I05RIK](http://www.gene.ucl.ac.uk/cgi-bin/nomenclature/searchgenes.pl?field=symbol&anchor=equals&symbol_search=Search&number=100&format=html&sortby=symbol&match=2410166I05RIK) | potassium channel activity;inward rectifier potassium channel activity;ion channel activity;voltage-gated ion channel activity;ATP-activated inward rectifier potassium channel activity |  | **1.34** |
| 1426387_X_AT | RIKEN cDNA 4933439C20 gene | [MGC65558](http://www.gene.ucl.ac.uk/cgi-bin/nomenclature/searchgenes.pl?field=symbol&anchor=equals&symbol_search=Search&number=100&format=html&sortby=symbol&match=MGC65558) | potassium channel activity;ion channel activity;calcium-activated potassium channel activity;calmodulin binding;small conductance calcium-activated potassium channel activity |  | **-1.93** |
| 1426389_AT | calcium/calmodulin-dependent protein kinase ID | [CAMK1D](http://www.gene.ucl.ac.uk/cgi-bin/nomenclature/searchgenes.pl?field=symbol&anchor=equals&symbol_search=Search&number=100&format=html&sortby=symbol&match=CAMK1D) | potassium channel activity;ion channel activity;inward rectifier potassium channel activity;voltage-gated ion channel activity;ATP-activated inward rectifier potassium channel activity | [607957](http://www.ncbi.nlm.nih.gov/entrez/dispomim.cgi?id=607957) | **-2.08** |
| 1426392_A_AT | ARP3 actin-related protein 3 homolog (yeast) | [ACTR3](http://www.gene.ucl.ac.uk/cgi-bin/nomenclature/searchgenes.pl?field=symbol&anchor=equals&symbol_search=Search&number=100&format=html&sortby=symbol&match=ACTR3) | pre-mRNA splicing factor activity | [604222](http://www.ncbi.nlm.nih.gov/entrez/dispomim.cgi?id=604222) | **-1.76** |
| 1426404_A_AT | ring finger protein 11 | [RNF11](http://www.gene.ucl.ac.uk/cgi-bin/nomenclature/searchgenes.pl?field=symbol&anchor=equals&symbol_search=Search&number=100&format=html&sortby=symbol&match=RNF11) | pre-mRNA splicing factor activity |  | **1.28** |
| 1426409_AT | leucine zipper, putative tumor suppressor 2 | [LZTS2](http://www.gene.ucl.ac.uk/cgi-bin/nomenclature/searchgenes.pl?field=symbol&anchor=equals&symbol_search=Search&number=100&format=html&sortby=symbol&match=LZTS2) | pre-mRNA splicing factor activity |  | **1.22** |
| 1426419_AT | RIKEN cDNA 1700009P03 gene | [1700009P03RIK](http://www.gene.ucl.ac.uk/cgi-bin/nomenclature/searchgenes.pl?field=symbol&anchor=equals&symbol_search=Search&number=100&format=html&sortby=symbol&match=1700009P03RIK) | pre-mRNA splicing factor activity |  | **-1.39** |
| 1426427_AT | tubulin tyrosine ligase-like 1 | [TTLL1](http://www.gene.ucl.ac.uk/cgi-bin/nomenclature/searchgenes.pl?field=symbol&anchor=equals&symbol_search=Search&number=100&format=html&sortby=symbol&match=TTLL1) | pre-mRNA splicing factor activity;RNA binding | [608955](http://www.ncbi.nlm.nih.gov/entrez/dispomim.cgi?id=608955) | **-1.64** |
| 1426430_AT | jagged 2 | [JAG2](http://www.gene.ucl.ac.uk/cgi-bin/nomenclature/searchgenes.pl?field=symbol&anchor=equals&symbol_search=Search&number=100&format=html&sortby=symbol&match=JAG2) | pre-mRNA splicing factor activity;RNA binding | [602570](http://www.ncbi.nlm.nih.gov/entrez/dispomim.cgi?id=602570) | **1.38** |
| 1426434_AT | RIKEN cDNA 1200015A22 gene | [1200015A22RIK](http://www.gene.ucl.ac.uk/cgi-bin/nomenclature/searchgenes.pl?field=symbol&anchor=equals&symbol_search=Search&number=100&format=html&sortby=symbol&match=1200015A22RIK) | pre-mRNA splicing factor activity;RNA binding |  | **-1.5** |
| 1426440_AT | dehydrogenase/reductase (SDR family) member 7 | [DHRS7](http://www.gene.ucl.ac.uk/cgi-bin/nomenclature/searchgenes.pl?field=symbol&anchor=equals&symbol_search=Search&number=100&format=html&sortby=symbol&match=DHRS7) | prenyltransferase activity;catalytic activity;transferase activity |  | **-1.45** |
| 1426442_AT | glycoprotein m6a | [GPM6A](http://www.gene.ucl.ac.uk/cgi-bin/nomenclature/searchgenes.pl?field=symbol&anchor=equals&symbol_search=Search&number=100&format=html&sortby=symbol&match=GPM6A) | prenyltransferase activity;transferase activity | [601275](http://www.ncbi.nlm.nih.gov/entrez/dispomim.cgi?id=601275) | **2.27** |
| 1426460_A_AT | gb:AV370025 /DB_XREF=gi:16397786 /DB_XREF=AV370025 |  | profilin binding;actin binding |  | **1.44** |
| 1426461_AT | UDP-glucose pyrophosphorylase 2 | [UGP2](http://www.gene.ucl.ac.uk/cgi-bin/nomenclature/searchgenes.pl?field=symbol&anchor=equals&symbol_search=Search&number=100&format=html&sortby=symbol&match=UGP2) | propionyl-CoA carboxylase activity;biotin binding;ligase activity;ATP binding;catalytic activity | [191760](http://www.ncbi.nlm.nih.gov/entrez/dispomim.cgi?id=191760) | **1.4** |
| 1426462_AT | gephyrin | [GPHN](http://www.gene.ucl.ac.uk/cgi-bin/nomenclature/searchgenes.pl?field=symbol&anchor=equals&symbol_search=Search&number=100&format=html&sortby=symbol&match=GPHN) | propionyl-CoA carboxylase activity;ligase activity;biotin carboxylase activity;acetyl-CoA carboxylase activity | [603930](http://www.ncbi.nlm.nih.gov/entrez/dispomim.cgi?id=603930) | **1.25** |
| 1426463_AT | gephyrin | [GPHN](http://www.gene.ucl.ac.uk/cgi-bin/nomenclature/searchgenes.pl?field=symbol&anchor=equals&symbol_search=Search&number=100&format=html&sortby=symbol&match=GPHN) | prostaglandin E receptor activity;G-protein coupled receptor activity;prostaglandin receptor activity;rhodopsin-like receptor activity | [603930](http://www.ncbi.nlm.nih.gov/entrez/dispomim.cgi?id=603930) | **1.26** |
| 1426465_AT | discs, large homolog-associated protein 4 (Drosophila) | [DLGAP4](http://www.gene.ucl.ac.uk/cgi-bin/nomenclature/searchgenes.pl?field=symbol&anchor=equals&symbol_search=Search&number=100&format=html&sortby=symbol&match=DLGAP4) | prostaglandin-D synthase activity;transporter activity;retinoid binding;isomerase activity |  | **-1.7** |
| 1426476_AT | RAS p21 protein activator 1 | [RASA1](http://www.gene.ucl.ac.uk/cgi-bin/nomenclature/searchgenes.pl?field=symbol&anchor=equals&symbol_search=Search&number=100&format=html&sortby=symbol&match=RASA1) | proteasome activator activity | [139150](http://www.ncbi.nlm.nih.gov/entrez/dispomim.cgi?id=139150) | **-1.28** |
| 1426477_AT | RAS p21 protein activator 1 | [RASA1](http://www.gene.ucl.ac.uk/cgi-bin/nomenclature/searchgenes.pl?field=symbol&anchor=equals&symbol_search=Search&number=100&format=html&sortby=symbol&match=RASA1) | protein binding | [139150](http://www.ncbi.nlm.nih.gov/entrez/dispomim.cgi?id=139150) | **-1.49** |
| 1426482_AT | protein-kinase, interferon-inducible double stranded RNA dependent inhibitor, repressor of (P58 repressor) | [PRKRIR](http://www.gene.ucl.ac.uk/cgi-bin/nomenclature/searchgenes.pl?field=symbol&anchor=equals&symbol_search=Search&number=100&format=html&sortby=symbol&match=PRKRIR) | protein binding | [607374](http://www.ncbi.nlm.nih.gov/entrez/dispomim.cgi?id=607374) | **1.4** |
| 1426483_AT | protein-kinase, interferon-inducible double stranded RNA dependent inhibitor, repressor of (P58 repressor) | [PRKRIR](http://www.gene.ucl.ac.uk/cgi-bin/nomenclature/searchgenes.pl?field=symbol&anchor=equals&symbol_search=Search&number=100&format=html&sortby=symbol&match=PRKRIR) | protein binding | [607374](http://www.ncbi.nlm.nih.gov/entrez/dispomim.cgi?id=607374) | **1.37** |
| 1426486_AT | UBX domain containing 2 | [UBXD2](http://www.gene.ucl.ac.uk/cgi-bin/nomenclature/searchgenes.pl?field=symbol&anchor=equals&symbol_search=Search&number=100&format=html&sortby=symbol&match=UBXD2) | protein binding |  | **-1.4** |
| 1426502_S_AT | glutamic pyruvic transaminase 1, soluble | [GPT1](http://www.gene.ucl.ac.uk/cgi-bin/nomenclature/searchgenes.pl?field=symbol&anchor=equals&symbol_search=Search&number=100&format=html&sortby=symbol&match=GPT1) | protein binding |  | **1.34** |
| 1426516_A_AT | lipin 1 | [LPIN1](http://www.gene.ucl.ac.uk/cgi-bin/nomenclature/searchgenes.pl?field=symbol&anchor=equals&symbol_search=Search&number=100&format=html&sortby=symbol&match=LPIN1) | protein binding | [605518](http://www.ncbi.nlm.nih.gov/entrez/dispomim.cgi?id=605518) | **1.93** |
| 1426522_AT | hydroxyacyl-Coenzyme A dehydrogenase/3-ketoacyl-Coenzyme A thiolase/enoyl-Coenzyme A hydratase (trifunctional protein), beta subunit | [HADHB](http://www.gene.ucl.ac.uk/cgi-bin/nomenclature/searchgenes.pl?field=symbol&anchor=equals&symbol_search=Search&number=100&format=html&sortby=symbol&match=HADHB) | protein binding | [143450](http://www.ncbi.nlm.nih.gov/entrez/dispomim.cgi?id=143450) | **1.67** |
| 1426534_A_AT | ADP-ribosylation factor GTPase activating protein 3 | [ARFGAP3](http://www.gene.ucl.ac.uk/cgi-bin/nomenclature/searchgenes.pl?field=symbol&anchor=equals&symbol_search=Search&number=100&format=html&sortby=symbol&match=ARFGAP3) | protein binding |  | **-1.37** |
| 1426555_AT | serine caroboxypeptidase 1 | [SCPEP1](http://www.gene.ucl.ac.uk/cgi-bin/nomenclature/searchgenes.pl?field=symbol&anchor=equals&symbol_search=Search&number=100&format=html&sortby=symbol&match=SCPEP1) | protein binding |  | **-1.67** |
| 1426560_A_AT | gb:BB433705 /DB_XREF=gi:16424302 /DB_XREF=BB433705 |  | protein binding |  | **-3** |
| 1426561_A_AT | gb:AW553512 /DB_XREF=gi:7198935 /DB_XREF=L0228B11- |  | protein binding |  | **-2.04** |
| 1426566_S_AT | interleukin 17 receptor E | [IL17RE](http://www.gene.ucl.ac.uk/cgi-bin/nomenclature/searchgenes.pl?field=symbol&anchor=equals&symbol_search=Search&number=100&format=html&sortby=symbol&match=IL17RE) | protein binding |  | **1.93** |
| 1426572_AT | malic enzyme 2, NAD(+)-dependent, mitochondrial | [ME2](http://www.gene.ucl.ac.uk/cgi-bin/nomenclature/searchgenes.pl?field=symbol&anchor=equals&symbol_search=Search&number=100&format=html&sortby=symbol&match=ME2) | protein binding | [154270](http://www.ncbi.nlm.nih.gov/entrez/dispomim.cgi?id=154270) | **-1.83** |
| 1426577_A_AT | RIKEN cDNA 1810054G18 gene | [1810054G18RIK](http://www.gene.ucl.ac.uk/cgi-bin/nomenclature/searchgenes.pl?field=symbol&anchor=equals&symbol_search=Search&number=100&format=html&sortby=symbol&match=1810054G18RIK) | protein binding |  | **-1.49** |
| 1426584_A_AT | sorbitol dehydrogenase 1 | [SDH1](http://www.gene.ucl.ac.uk/cgi-bin/nomenclature/searchgenes.pl?field=symbol&anchor=equals&symbol_search=Search&number=100&format=html&sortby=symbol&match=SDH1) | protein binding |  | **2.4** |
| 1426586_AT | solute carrier family 25 (mitochondrial carrier oxoglutarate carrier), member 11 | [SLC25A11](http://www.gene.ucl.ac.uk/cgi-bin/nomenclature/searchgenes.pl?field=symbol&anchor=equals&symbol_search=Search&number=100&format=html&sortby=symbol&match=SLC25A11) | protein binding | [604165](http://www.ncbi.nlm.nih.gov/entrez/dispomim.cgi?id=604165) | **1.53** |
| 1426587_A_AT | signal transducer and activator of transcription 3 | [STAT3](http://www.gene.ucl.ac.uk/cgi-bin/nomenclature/searchgenes.pl?field=symbol&anchor=equals&symbol_search=Search&number=100&format=html&sortby=symbol&match=STAT3) | protein binding | [102582](http://www.ncbi.nlm.nih.gov/entrez/dispomim.cgi?id=102582) | **-1.31** |
| 1426590_AT | G elongation factor, mitochondrial 2 | [GFM2](http://www.gene.ucl.ac.uk/cgi-bin/nomenclature/searchgenes.pl?field=symbol&anchor=equals&symbol_search=Search&number=100&format=html&sortby=symbol&match=GFM2) | protein binding | [606544](http://www.ncbi.nlm.nih.gov/entrez/dispomim.cgi?id=606544) | **1.62** |
| 1426615_S_AT | N-myc downstream regulated gene 4 | [NDRG4](http://www.gene.ucl.ac.uk/cgi-bin/nomenclature/searchgenes.pl?field=symbol&anchor=equals&symbol_search=Search&number=100&format=html&sortby=symbol&match=NDRG4) | protein binding |  | **-3.11** |
| 1426625_AT | RIKEN cDNA 2610029D06 gene | [2610029D06RIK](http://www.gene.ucl.ac.uk/cgi-bin/nomenclature/searchgenes.pl?field=symbol&anchor=equals&symbol_search=Search&number=100&format=html&sortby=symbol&match=2610029D06RIK) | protein binding |  | **-1.3** |
| 1426628_AT | transmembrane protein 34 | [TMEM34](http://www.gene.ucl.ac.uk/cgi-bin/nomenclature/searchgenes.pl?field=symbol&anchor=equals&symbol_search=Search&number=100&format=html&sortby=symbol&match=TMEM34) | protein binding |  | **-1.68** |
| 1426641_AT | tribbles homolog 2 (Drosophila) | [TRIB2](http://www.gene.ucl.ac.uk/cgi-bin/nomenclature/searchgenes.pl?field=symbol&anchor=equals&symbol_search=Search&number=100&format=html&sortby=symbol&match=TRIB2) | protein binding |  | **1.7** |
| 1426642_AT | fibronectin 1 | [FN1](http://www.gene.ucl.ac.uk/cgi-bin/nomenclature/searchgenes.pl?field=symbol&anchor=equals&symbol_search=Search&number=100&format=html&sortby=symbol&match=FN1) | protein binding | [135600](http://www.ncbi.nlm.nih.gov/entrez/dispomim.cgi?id=135600) | **-1.6** |
| 1426645_AT | heat shock protein 1, alpha | [HSPCA](http://www.gene.ucl.ac.uk/cgi-bin/nomenclature/searchgenes.pl?field=symbol&anchor=equals&symbol_search=Search&number=100&format=html&sortby=symbol&match=HSPCA) | protein binding | [140571](http://www.ncbi.nlm.nih.gov/entrez/dispomim.cgi?id=140571) | **-1.64** |
| 1426648_AT | MAP kinase-activated protein kinase 2 | [MAPKAPK2](http://www.gene.ucl.ac.uk/cgi-bin/nomenclature/searchgenes.pl?field=symbol&anchor=equals&symbol_search=Search&number=100&format=html&sortby=symbol&match=MAPKAPK2) | protein binding | [602006](http://www.ncbi.nlm.nih.gov/entrez/dispomim.cgi?id=602006) | **1.53** |
| 1426650_AT | Myosin, heavy polypeptide 8, skeletal muscle, perinatal | [MYH8](http://www.gene.ucl.ac.uk/cgi-bin/nomenclature/searchgenes.pl?field=symbol&anchor=equals&symbol_search=Search&number=100&format=html&sortby=symbol&match=MYH8) | protein binding | [160741](http://www.ncbi.nlm.nih.gov/entrez/dispomim.cgi?id=160741) | **-134.72** |
| 1426661_AT | ribosomal protein L27a | [RPL27A](http://www.gene.ucl.ac.uk/cgi-bin/nomenclature/searchgenes.pl?field=symbol&anchor=equals&symbol_search=Search&number=100&format=html&sortby=symbol&match=RPL27A) | protein binding | [603637](http://www.ncbi.nlm.nih.gov/entrez/dispomim.cgi?id=603637) | **-1.34** |
| 1426662_AT | cytidine monophospho-N-acetylneuraminic acid synthetase | [CMAS](http://www.gene.ucl.ac.uk/cgi-bin/nomenclature/searchgenes.pl?field=symbol&anchor=equals&symbol_search=Search&number=100&format=html&sortby=symbol&match=CMAS) | protein binding | [603316](http://www.ncbi.nlm.nih.gov/entrez/dispomim.cgi?id=603316) | **-1.23** |
| 1426668_AT | solute carrier family 30 (zinc transporter), member 9 | [SLC30A9](http://www.gene.ucl.ac.uk/cgi-bin/nomenclature/searchgenes.pl?field=symbol&anchor=equals&symbol_search=Search&number=100&format=html&sortby=symbol&match=SLC30A9) | protein binding | [604604](http://www.ncbi.nlm.nih.gov/entrez/dispomim.cgi?id=604604) | **1.4** |
| 1426672_AT | expressed sequence AI604832 | [AI604832](http://www.gene.ucl.ac.uk/cgi-bin/nomenclature/searchgenes.pl?field=symbol&anchor=equals&symbol_search=Search&number=100&format=html&sortby=symbol&match=AI604832) | protein binding |  | **2.47** |
| 1426682_AT | RIKEN cDNA A230103N10 gene | [A230103N10RIK](http://www.gene.ucl.ac.uk/cgi-bin/nomenclature/searchgenes.pl?field=symbol&anchor=equals&symbol_search=Search&number=100&format=html&sortby=symbol&match=A230103N10RIK) | protein binding |  | **-1.23** |
| 1426688_AT | succinate dehydrogenase complex, subunit A, flavoprotein (Fp) | [SDHA](http://www.gene.ucl.ac.uk/cgi-bin/nomenclature/searchgenes.pl?field=symbol&anchor=equals&symbol_search=Search&number=100&format=html&sortby=symbol&match=SDHA) | protein binding | [600857](http://www.ncbi.nlm.nih.gov/entrez/dispomim.cgi?id=600857) | **1.29** |
| 1426689_S_AT | succinate dehydrogenase complex, subunit A, flavoprotein (Fp) | [SDHA](http://www.gene.ucl.ac.uk/cgi-bin/nomenclature/searchgenes.pl?field=symbol&anchor=equals&symbol_search=Search&number=100&format=html&sortby=symbol&match=SDHA) | protein binding | [600857](http://www.ncbi.nlm.nih.gov/entrez/dispomim.cgi?id=600857) | **1.51** |
| 1426690_A_AT | sterol regulatory element binding factor 1 | [SREBF1](http://www.gene.ucl.ac.uk/cgi-bin/nomenclature/searchgenes.pl?field=symbol&anchor=equals&symbol_search=Search&number=100&format=html&sortby=symbol&match=SREBF1) | protein binding | [184756](http://www.ncbi.nlm.nih.gov/entrez/dispomim.cgi?id=184756) | **1.39** |
| 1426691_AT | tight junction protein 4 (peripheral) | [TJP4](http://www.gene.ucl.ac.uk/cgi-bin/nomenclature/searchgenes.pl?field=symbol&anchor=equals&symbol_search=Search&number=100&format=html&sortby=symbol&match=TJP4) | protein binding |  | **-1.34** |
| 1426708_AT | anthrax toxin receptor 2 | [ANTXR2](http://www.gene.ucl.ac.uk/cgi-bin/nomenclature/searchgenes.pl?field=symbol&anchor=equals&symbol_search=Search&number=100&format=html&sortby=symbol&match=ANTXR2) | protein binding | [608041](http://www.ncbi.nlm.nih.gov/entrez/dispomim.cgi?id=608041) | **-1.38** |
| 1426713_S_AT | glutamyl-prolyl-tRNA synthetase | [EPRS](http://www.gene.ucl.ac.uk/cgi-bin/nomenclature/searchgenes.pl?field=symbol&anchor=equals&symbol_search=Search&number=100&format=html&sortby=symbol&match=EPRS) | protein binding | [138295](http://www.ncbi.nlm.nih.gov/entrez/dispomim.cgi?id=138295) | **1.21** |
| 1426719_AT | amyloid beta (A4) precursor protein-binding, family B, member 2 | [APBB2](http://www.gene.ucl.ac.uk/cgi-bin/nomenclature/searchgenes.pl?field=symbol&anchor=equals&symbol_search=Search&number=100&format=html&sortby=symbol&match=APBB2) | protein binding | [602710](http://www.ncbi.nlm.nih.gov/entrez/dispomim.cgi?id=602710) | **1.35** |
| 1426724_AT | calponin 3, acidic | [CNN3](http://www.gene.ucl.ac.uk/cgi-bin/nomenclature/searchgenes.pl?field=symbol&anchor=equals&symbol_search=Search&number=100&format=html&sortby=symbol&match=CNN3) | protein binding | [602374](http://www.ncbi.nlm.nih.gov/entrez/dispomim.cgi?id=602374) | **-1.73** |
| 1426729_AT | RIKEN cDNA 2900046G09 gene | [2900046G09RIK](http://www.gene.ucl.ac.uk/cgi-bin/nomenclature/searchgenes.pl?field=symbol&anchor=equals&symbol_search=Search&number=100&format=html&sortby=symbol&match=2900046G09RIK) | protein binding |  | **1.72** |
| 1426734_AT | cDNA sequence BC022623 | [BC022623](http://www.gene.ucl.ac.uk/cgi-bin/nomenclature/searchgenes.pl?field=symbol&anchor=equals&symbol_search=Search&number=100&format=html&sortby=symbol&match=BC022623) | protein binding |  | **1.4** |
| 1426735_AT | RIKEN cDNA 2010002H18 gene | [2010002H18RIK](http://www.gene.ucl.ac.uk/cgi-bin/nomenclature/searchgenes.pl?field=symbol&anchor=equals&symbol_search=Search&number=100&format=html&sortby=symbol&match=2010002H18RIK) | protein binding |  | **1.23** |
| 1426742_AT | ATP synthase, H+ transporting, mitochondrial F0 complex, subunit b, isoform 1 | [ATP5F1](http://www.gene.ucl.ac.uk/cgi-bin/nomenclature/searchgenes.pl?field=symbol&anchor=equals&symbol_search=Search&number=100&format=html&sortby=symbol&match=ATP5F1) | protein binding | [603270](http://www.ncbi.nlm.nih.gov/entrez/dispomim.cgi?id=603270) | **1.31** |
| 1426744_AT | Sterol regulatory element binding factor 2 | [SREBF2](http://www.gene.ucl.ac.uk/cgi-bin/nomenclature/searchgenes.pl?field=symbol&anchor=equals&symbol_search=Search&number=100&format=html&sortby=symbol&match=SREBF2) | protein binding | [600481](http://www.ncbi.nlm.nih.gov/entrez/dispomim.cgi?id=600481) | **-1.47** |
| 1426750_AT | filamin, beta | [FLNB](http://www.gene.ucl.ac.uk/cgi-bin/nomenclature/searchgenes.pl?field=symbol&anchor=equals&symbol_search=Search&number=100&format=html&sortby=symbol&match=FLNB) | protein binding | [603381](http://www.ncbi.nlm.nih.gov/entrez/dispomim.cgi?id=603381) | **1.56** |
| 1426754_X_AT | Cytoskeleton-associated protein 4 | [CKAP4](http://www.gene.ucl.ac.uk/cgi-bin/nomenclature/searchgenes.pl?field=symbol&anchor=equals&symbol_search=Search&number=100&format=html&sortby=symbol&match=CKAP4) | protein binding |  | **-1.48** |
| 1426755_AT | Cytoskeleton-associated protein 4 | [CKAP4](http://www.gene.ucl.ac.uk/cgi-bin/nomenclature/searchgenes.pl?field=symbol&anchor=equals&symbol_search=Search&number=100&format=html&sortby=symbol&match=CKAP4) | protein binding |  | **-2.29** |
| 1426759_AT | mitogen-activated protein kinase kinase kinase kinase 3 | [MAP4K3](http://www.gene.ucl.ac.uk/cgi-bin/nomenclature/searchgenes.pl?field=symbol&anchor=equals&symbol_search=Search&number=100&format=html&sortby=symbol&match=MAP4K3) | protein binding | [604921](http://www.ncbi.nlm.nih.gov/entrez/dispomim.cgi?id=604921) | **1.23** |
| 1426764_AT | ornithine decarboxylase antizyme 2 | [OAZ2](http://www.gene.ucl.ac.uk/cgi-bin/nomenclature/searchgenes.pl?field=symbol&anchor=equals&symbol_search=Search&number=100&format=html&sortby=symbol&match=OAZ2) | protein binding | [604152](http://www.ncbi.nlm.nih.gov/entrez/dispomim.cgi?id=604152) | **1.25** |
| 1426765_AT | COMM domain containing 7 | [COMMD7](http://www.gene.ucl.ac.uk/cgi-bin/nomenclature/searchgenes.pl?field=symbol&anchor=equals&symbol_search=Search&number=100&format=html&sortby=symbol&match=COMMD7) | protein binding |  | **-1.35** |
| 1426773_AT | mitofusin 1 | [MFN1](http://www.gene.ucl.ac.uk/cgi-bin/nomenclature/searchgenes.pl?field=symbol&anchor=equals&symbol_search=Search&number=100&format=html&sortby=symbol&match=MFN1) | protein binding | [608506](http://www.ncbi.nlm.nih.gov/entrez/dispomim.cgi?id=608506) | **1.91** |
| 1426778_AT | dystroglycan 1 | [DAG1](http://www.gene.ucl.ac.uk/cgi-bin/nomenclature/searchgenes.pl?field=symbol&anchor=equals&symbol_search=Search&number=100&format=html&sortby=symbol&match=DAG1) | protein binding | [128239](http://www.ncbi.nlm.nih.gov/entrez/dispomim.cgi?id=128239) | **-1.32** |
| 1426784_AT | tripartite motif protein 47 | [TRIM47](http://www.gene.ucl.ac.uk/cgi-bin/nomenclature/searchgenes.pl?field=symbol&anchor=equals&symbol_search=Search&number=100&format=html&sortby=symbol&match=TRIM47) | protein binding |  | **-1.62** |
| 1426785_S_AT | monoglyceride lipase | [MGLL](http://www.gene.ucl.ac.uk/cgi-bin/nomenclature/searchgenes.pl?field=symbol&anchor=equals&symbol_search=Search&number=100&format=html&sortby=symbol&match=MGLL) | protein binding |  | **2.06** |
| 1426791_AT | RUN and SH3 domain containing 2 | [RUSC2](http://www.gene.ucl.ac.uk/cgi-bin/nomenclature/searchgenes.pl?field=symbol&anchor=equals&symbol_search=Search&number=100&format=html&sortby=symbol&match=RUSC2) | protein binding |  | **3.24** |
| 1426793_A_AT | ribosomal protein L14 | [RPL14](http://www.gene.ucl.ac.uk/cgi-bin/nomenclature/searchgenes.pl?field=symbol&anchor=equals&symbol_search=Search&number=100&format=html&sortby=symbol&match=RPL14) | protein binding |  | **-1.29** |
| 1426796_AT | Taxilin | [TXLN](http://www.gene.ucl.ac.uk/cgi-bin/nomenclature/searchgenes.pl?field=symbol&anchor=equals&symbol_search=Search&number=100&format=html&sortby=symbol&match=TXLN) | protein binding |  | **1.23** |
| 1426798_A_AT | protein phosphatase 1, regulatory (inhibitor) subunit 15b | [PPP1R15B](http://www.gene.ucl.ac.uk/cgi-bin/nomenclature/searchgenes.pl?field=symbol&anchor=equals&symbol_search=Search&number=100&format=html&sortby=symbol&match=PPP1R15B) | protein binding |  | **-1.41** |
| 1426799_AT | RAB8B, member RAS oncogene family | [RAB8B](http://www.gene.ucl.ac.uk/cgi-bin/nomenclature/searchgenes.pl?field=symbol&anchor=equals&symbol_search=Search&number=100&format=html&sortby=symbol&match=RAB8B) | protein binding |  | **-1.52** |
| 1426808_AT | lectin, galactose binding, soluble 3 | [LGALS3](http://www.gene.ucl.ac.uk/cgi-bin/nomenclature/searchgenes.pl?field=symbol&anchor=equals&symbol_search=Search&number=100&format=html&sortby=symbol&match=LGALS3) | protein binding | [153619](http://www.ncbi.nlm.nih.gov/entrez/dispomim.cgi?id=153619) | **-12.94** |
| 1426812_A_AT | RIKEN cDNA 9130404D14 gene | [9130404D14RIK](http://www.gene.ucl.ac.uk/cgi-bin/nomenclature/searchgenes.pl?field=symbol&anchor=equals&symbol_search=Search&number=100&format=html&sortby=symbol&match=9130404D14RIK) | protein binding |  | **-1.6** |
| 1426817_AT | antigen identified by monoclonal antibody Ki 67 | [MKI67](http://www.gene.ucl.ac.uk/cgi-bin/nomenclature/searchgenes.pl?field=symbol&anchor=equals&symbol_search=Search&number=100&format=html&sortby=symbol&match=MKI67) | protein binding | [176741](http://www.ncbi.nlm.nih.gov/entrez/dispomim.cgi?id=176741) | **-5.67** |
| 1426822_AT | ras homolog gene family, member T2 | [RHOT2](http://www.gene.ucl.ac.uk/cgi-bin/nomenclature/searchgenes.pl?field=symbol&anchor=equals&symbol_search=Search&number=100&format=html&sortby=symbol&match=RHOT2) | protein binding |  | **1.73** |
| 1426833_AT | eukaryotic translation initiation factor 4 gamma, 3 | [EIF4G3](http://www.gene.ucl.ac.uk/cgi-bin/nomenclature/searchgenes.pl?field=symbol&anchor=equals&symbol_search=Search&number=100&format=html&sortby=symbol&match=EIF4G3) | protein binding | [603929](http://www.ncbi.nlm.nih.gov/entrez/dispomim.cgi?id=603929) | **1.21** |
| 1426850_A_AT | mitogen activated protein kinase kinase 6 | [MAP2K6](http://www.gene.ucl.ac.uk/cgi-bin/nomenclature/searchgenes.pl?field=symbol&anchor=equals&symbol_search=Search&number=100&format=html&sortby=symbol&match=MAP2K6) | protein binding | [601254](http://www.ncbi.nlm.nih.gov/entrez/dispomim.cgi?id=601254) | **1.49** |
| 1426856_AT | gb:AJ293845.1 /DB_XREF=gi:13397922 /FEA=mRNA /CNT= |  | protein binding |  | **1.99** |
| 1426857_A_AT | RIKEN cDNA 2610207I16 gene | [HSDL2](http://www.gene.ucl.ac.uk/cgi-bin/nomenclature/searchgenes.pl?field=symbol&anchor=equals&symbol_search=Search&number=100&format=html&sortby=symbol&match=HSDL2) | protein binding |  | **1.65** |
| 1426864_A_AT | neural cell adhesion molecule 1 | [NCAM1](http://www.gene.ucl.ac.uk/cgi-bin/nomenclature/searchgenes.pl?field=symbol&anchor=equals&symbol_search=Search&number=100&format=html&sortby=symbol&match=NCAM1) | protein binding | [116930](http://www.ncbi.nlm.nih.gov/entrez/dispomim.cgi?id=116930) | **-20.59** |
| 1426865_A_AT | neural cell adhesion molecule 1 | [NCAM1](http://www.gene.ucl.ac.uk/cgi-bin/nomenclature/searchgenes.pl?field=symbol&anchor=equals&symbol_search=Search&number=100&format=html&sortby=symbol&match=NCAM1) | protein binding | [116930](http://www.ncbi.nlm.nih.gov/entrez/dispomim.cgi?id=116930) | **-3.52** |
| 1426869_AT | biregional cell adhesion molecule-related/down-regulated by oncogenes (Cdon) binding protein | [BOC](http://www.gene.ucl.ac.uk/cgi-bin/nomenclature/searchgenes.pl?field=symbol&anchor=equals&symbol_search=Search&number=100&format=html&sortby=symbol&match=BOC) | protein binding | [608708](http://www.ncbi.nlm.nih.gov/entrez/dispomim.cgi?id=608708) | **-1.51** |
| 1426884_AT | RIKEN cDNA 1110007A06 gene | [1110007A06RIK](http://www.gene.ucl.ac.uk/cgi-bin/nomenclature/searchgenes.pl?field=symbol&anchor=equals&symbol_search=Search&number=100&format=html&sortby=symbol&match=1110007A06RIK) | protein binding |  | **1.39** |
| 1426891_AT | RNA polymerase II associated protein 1 | [RPAP1](http://www.gene.ucl.ac.uk/cgi-bin/nomenclature/searchgenes.pl?field=symbol&anchor=equals&symbol_search=Search&number=100&format=html&sortby=symbol&match=RPAP1) | protein binding |  | **1.59** |
| 1426897_AT | RIKEN cDNA 2610510H01 gene | [2610510H01RIK](http://www.gene.ucl.ac.uk/cgi-bin/nomenclature/searchgenes.pl?field=symbol&anchor=equals&symbol_search=Search&number=100&format=html&sortby=symbol&match=2610510H01RIK) | protein binding |  | **-1.48** |
| 1426901_S_AT | calmodulin binding transcription activator 2 | [CAMTA2](http://www.gene.ucl.ac.uk/cgi-bin/nomenclature/searchgenes.pl?field=symbol&anchor=equals&symbol_search=Search&number=100&format=html&sortby=symbol&match=CAMTA2) | protein binding |  | **1.23** |
| 1426906_AT | interferon activated gene 203 | [IFI203](http://www.gene.ucl.ac.uk/cgi-bin/nomenclature/searchgenes.pl?field=symbol&anchor=equals&symbol_search=Search&number=100&format=html&sortby=symbol&match=IFI203) | protein binding |  | **-1.55** |
| 1426915_AT | death associated protein kinase 1 | [DAPK1](http://www.gene.ucl.ac.uk/cgi-bin/nomenclature/searchgenes.pl?field=symbol&anchor=equals&symbol_search=Search&number=100&format=html&sortby=symbol&match=DAPK1) | protein binding | [600831](http://www.ncbi.nlm.nih.gov/entrez/dispomim.cgi?id=600831) | **-1.65** |
| 1426917_S_AT | secernin 3 | [SCRN3](http://www.gene.ucl.ac.uk/cgi-bin/nomenclature/searchgenes.pl?field=symbol&anchor=equals&symbol_search=Search&number=100&format=html&sortby=symbol&match=SCRN3) | protein binding |  | **1.42** |
| 1426918_AT | integrin beta 1 (fibronectin receptor beta) | [ITGB1](http://www.gene.ucl.ac.uk/cgi-bin/nomenclature/searchgenes.pl?field=symbol&anchor=equals&symbol_search=Search&number=100&format=html&sortby=symbol&match=ITGB1) | protein binding | [135630](http://www.ncbi.nlm.nih.gov/entrez/dispomim.cgi?id=135630) | **-1.41** |
| 1426923_AT | HIV-1 Rev binding protein | [HRB](http://www.gene.ucl.ac.uk/cgi-bin/nomenclature/searchgenes.pl?field=symbol&anchor=equals&symbol_search=Search&number=100&format=html&sortby=symbol&match=HRB) | protein binding | [600862](http://www.ncbi.nlm.nih.gov/entrez/dispomim.cgi?id=600862) | **-1.25** |
| 1426931_S_AT | DNA segment, Chr 19, Brigham & Women's Genetics 1357 expressed | [D19BWG1357E](http://www.gene.ucl.ac.uk/cgi-bin/nomenclature/searchgenes.pl?field=symbol&anchor=equals&symbol_search=Search&number=100&format=html&sortby=symbol&match=D19BWG1357E) | protein binding |  | **-1.35** |
| 1426934_AT | gb:BC013565.1 /DB_XREF=gi:15488868 /FEA=mRNA /CNT= |  | protein binding |  | **1.7** |
| 1426936_AT | cDNA sequence BC005512 | [BC005512](http://www.gene.ucl.ac.uk/cgi-bin/nomenclature/searchgenes.pl?field=symbol&anchor=equals&symbol_search=Search&number=100&format=html&sortby=symbol&match=BC005512) | protein binding |  | **-4.21** |
| 1426945_AT | karyopherin (importin) beta 3 | [RANBP5](http://www.gene.ucl.ac.uk/cgi-bin/nomenclature/searchgenes.pl?field=symbol&anchor=equals&symbol_search=Search&number=100&format=html&sortby=symbol&match=RANBP5) | protein binding | [602008](http://www.ncbi.nlm.nih.gov/entrez/dispomim.cgi?id=602008) | **1.29** |
| 1426946_AT | karyopherin (importin) beta 3 | [RANBP5](http://www.gene.ucl.ac.uk/cgi-bin/nomenclature/searchgenes.pl?field=symbol&anchor=equals&symbol_search=Search&number=100&format=html&sortby=symbol&match=RANBP5) | protein binding | [602008](http://www.ncbi.nlm.nih.gov/entrez/dispomim.cgi?id=602008) | **1.29** |
| 1426947_X_AT | procollagen, type VI, alpha 2 | [COL6A2](http://www.gene.ucl.ac.uk/cgi-bin/nomenclature/searchgenes.pl?field=symbol&anchor=equals&symbol_search=Search&number=100&format=html&sortby=symbol&match=COL6A2) | protein binding | [120240](http://www.ncbi.nlm.nih.gov/entrez/dispomim.cgi?id=120240) | **-1.74** |
| 1426951_AT | cysteine-rich motor neuron 1 | [CRIM1](http://www.gene.ucl.ac.uk/cgi-bin/nomenclature/searchgenes.pl?field=symbol&anchor=equals&symbol_search=Search&number=100&format=html&sortby=symbol&match=CRIM1) | protein binding | [606189](http://www.ncbi.nlm.nih.gov/entrez/dispomim.cgi?id=606189) | **2.06** |
| 1426956_A_AT | transformation related protein 53 binding protein 1 | [TRP53BP1](http://www.gene.ucl.ac.uk/cgi-bin/nomenclature/searchgenes.pl?field=symbol&anchor=equals&symbol_search=Search&number=100&format=html&sortby=symbol&match=TRP53BP1) | protein binding |  | **-1.38** |
| 1426959_AT | 3-hydroxybutyrate dehydrogenase (heart, mitochondrial) | [BDH](http://www.gene.ucl.ac.uk/cgi-bin/nomenclature/searchgenes.pl?field=symbol&anchor=equals&symbol_search=Search&number=100&format=html&sortby=symbol&match=BDH) | protein binding | [603063](http://www.ncbi.nlm.nih.gov/entrez/dispomim.cgi?id=603063) | **2.8** |
| 1426964_AT | RIKEN cDNA 3110003A17 gene | [3110003A17RIK](http://www.gene.ucl.ac.uk/cgi-bin/nomenclature/searchgenes.pl?field=symbol&anchor=equals&symbol_search=Search&number=100&format=html&sortby=symbol&match=3110003A17RIK) | protein binding |  | **-2.11** |
| 1426965_AT | RIKEN cDNA 5830461H18 gene | [5830461H18RIK](http://www.gene.ucl.ac.uk/cgi-bin/nomenclature/searchgenes.pl?field=symbol&anchor=equals&symbol_search=Search&number=100&format=html&sortby=symbol&match=5830461H18RIK) | protein binding |  | **-1.25** |
| 1426972_AT | SEC24 related gene family, member D (S. cerevisiae) | [SEC24D](http://www.gene.ucl.ac.uk/cgi-bin/nomenclature/searchgenes.pl?field=symbol&anchor=equals&symbol_search=Search&number=100&format=html&sortby=symbol&match=SEC24D) | protein binding | [607186](http://www.ncbi.nlm.nih.gov/entrez/dispomim.cgi?id=607186) | **-1.78** |
| 1426981_AT | Paired basic amino acid cleaving system 4 | [PACE4](http://www.gene.ucl.ac.uk/cgi-bin/nomenclature/searchgenes.pl?field=symbol&anchor=equals&symbol_search=Search&number=100&format=html&sortby=symbol&match=PACE4) | protein binding | [167405](http://www.ncbi.nlm.nih.gov/entrez/dispomim.cgi?id=167405) | **2.11** |
| 1426983_AT | Formin binding protein 1 | [FNBP1](http://www.gene.ucl.ac.uk/cgi-bin/nomenclature/searchgenes.pl?field=symbol&anchor=equals&symbol_search=Search&number=100&format=html&sortby=symbol&match=FNBP1) | protein binding | [606191](http://www.ncbi.nlm.nih.gov/entrez/dispomim.cgi?id=606191) | **-1.29** |
| 1426993_AT | xenotropic and polytropic retrovirus receptor 1 | [XPR1](http://www.gene.ucl.ac.uk/cgi-bin/nomenclature/searchgenes.pl?field=symbol&anchor=equals&symbol_search=Search&number=100&format=html&sortby=symbol&match=XPR1) | protein binding | [605237](http://www.ncbi.nlm.nih.gov/entrez/dispomim.cgi?id=605237) | **1.36** |
| 1426994_AT | RIKEN cDNA 9430090L19 gene | [PLEKHE1](http://www.gene.ucl.ac.uk/cgi-bin/nomenclature/searchgenes.pl?field=symbol&anchor=equals&symbol_search=Search&number=100&format=html&sortby=symbol&match=PLEKHE1) | protein binding |  | **1.38** |
| 1426998_AT | testis expressed gene 27 | [TEX27](http://www.gene.ucl.ac.uk/cgi-bin/nomenclature/searchgenes.pl?field=symbol&anchor=equals&symbol_search=Search&number=100&format=html&sortby=symbol&match=TEX27) | protein binding | [607455](http://www.ncbi.nlm.nih.gov/entrez/dispomim.cgi?id=607455) | **1.43** |
| 1427014_AT | CDNA sequence BC026996 | [BC026996](http://www.gene.ucl.ac.uk/cgi-bin/nomenclature/searchgenes.pl?field=symbol&anchor=equals&symbol_search=Search&number=100&format=html&sortby=symbol&match=BC026996) | protein binding |  | **1.62** |
| 1427032_AT | hect domain and RLD 4 | [HERC4](http://www.gene.ucl.ac.uk/cgi-bin/nomenclature/searchgenes.pl?field=symbol&anchor=equals&symbol_search=Search&number=100&format=html&sortby=symbol&match=HERC4) | protein binding |  | **-1.35** |
| 1427035_AT | solute carrier family 39 (zinc transporter), member 14 | [SLC39A14](http://www.gene.ucl.ac.uk/cgi-bin/nomenclature/searchgenes.pl?field=symbol&anchor=equals&symbol_search=Search&number=100&format=html&sortby=symbol&match=SLC39A14) | protein binding | [608736](http://www.ncbi.nlm.nih.gov/entrez/dispomim.cgi?id=608736) | **-1.76** |
| 1427038_AT | preproenkephalin 1 | [PENK1](http://www.gene.ucl.ac.uk/cgi-bin/nomenclature/searchgenes.pl?field=symbol&anchor=equals&symbol_search=Search&number=100&format=html&sortby=symbol&match=PENK1) | protein binding |  | **1.37** |
| 1427039_AT | epsin 1 | [EPN1](http://www.gene.ucl.ac.uk/cgi-bin/nomenclature/searchgenes.pl?field=symbol&anchor=equals&symbol_search=Search&number=100&format=html&sortby=symbol&match=EPN1) | protein binding | [607262](http://www.ncbi.nlm.nih.gov/entrez/dispomim.cgi?id=607262) | **1.29** |
| 1427040_AT | kidney cell line derived transcript 1 | [MDFID](http://www.gene.ucl.ac.uk/cgi-bin/nomenclature/searchgenes.pl?field=symbol&anchor=equals&symbol_search=Search&number=100&format=html&sortby=symbol&match=MDFID) | protein binding |  | **-1.23** |
| 1427041_AT | cDNA sequence BC013712 | [BC013712](http://www.gene.ucl.ac.uk/cgi-bin/nomenclature/searchgenes.pl?field=symbol&anchor=equals&symbol_search=Search&number=100&format=html&sortby=symbol&match=BC013712) | protein binding |  | **-1.66** |
| 1427052_AT | gb:BC022940.1 /DB_XREF=gi:18606145 /FEA=mRNA /CNT= |  | protein binding |  | **2.2** |
| 1427054_S_AT | RIKEN cDNA D930038M13 gene | [D930038M13RIK](http://www.gene.ucl.ac.uk/cgi-bin/nomenclature/searchgenes.pl?field=symbol&anchor=equals&symbol_search=Search&number=100&format=html&sortby=symbol&match=D930038M13RIK) | protein binding |  | **-1.33** |
| 1427056_AT | A disintegrin-like and metalloprotease (reprolysin type) with thrombospondin type 1 motif, 15 | [ADAMTS15](http://www.gene.ucl.ac.uk/cgi-bin/nomenclature/searchgenes.pl?field=symbol&anchor=equals&symbol_search=Search&number=100&format=html&sortby=symbol&match=ADAMTS15) | protein binding | [607509](http://www.ncbi.nlm.nih.gov/entrez/dispomim.cgi?id=607509) | **-1.45** |
| 1427058_AT | eukaryotic translation initiation factor 4A1 | [EIF4A1](http://www.gene.ucl.ac.uk/cgi-bin/nomenclature/searchgenes.pl?field=symbol&anchor=equals&symbol_search=Search&number=100&format=html&sortby=symbol&match=EIF4A1) | protein binding | [602641](http://www.ncbi.nlm.nih.gov/entrez/dispomim.cgi?id=602641) | **-1.43** |
| 1427064_A_AT | scribbled homolog (Drosophila) | [SCRIB](http://www.gene.ucl.ac.uk/cgi-bin/nomenclature/searchgenes.pl?field=symbol&anchor=equals&symbol_search=Search&number=100&format=html&sortby=symbol&match=SCRIB) | protein binding | [607733](http://www.ncbi.nlm.nih.gov/entrez/dispomim.cgi?id=607733) | **-1.95** |
| 1427065_AT | RIKEN cDNA 4933439F18 gene | [4933439F18RIK](http://www.gene.ucl.ac.uk/cgi-bin/nomenclature/searchgenes.pl?field=symbol&anchor=equals&symbol_search=Search&number=100&format=html&sortby=symbol&match=4933439F18RIK) | protein binding |  | **1.57** |
| 1427067_AT | RIKEN cDNA 4933439F18 gene | [4933439F18RIK](http://www.gene.ucl.ac.uk/cgi-bin/nomenclature/searchgenes.pl?field=symbol&anchor=equals&symbol_search=Search&number=100&format=html&sortby=symbol&match=4933439F18RIK) | protein binding |  | **1.61** |
| 1427068_X_AT | RIKEN cDNA 4933439F18 gene | [4933439F18RIK](http://www.gene.ucl.ac.uk/cgi-bin/nomenclature/searchgenes.pl?field=symbol&anchor=equals&symbol_search=Search&number=100&format=html&sortby=symbol&match=4933439F18RIK) | protein binding |  | **1.55** |
| 1427073_AT | lactation elevated 1 | [LACE1](http://www.gene.ucl.ac.uk/cgi-bin/nomenclature/searchgenes.pl?field=symbol&anchor=equals&symbol_search=Search&number=100&format=html&sortby=symbol&match=LACE1) | protein binding |  | **1.32** |
| 1427074_AT | RIKEN cDNA 5330414D10 gene | [5330414D10RIK](http://www.gene.ucl.ac.uk/cgi-bin/nomenclature/searchgenes.pl?field=symbol&anchor=equals&symbol_search=Search&number=100&format=html&sortby=symbol&match=5330414D10RIK) | protein binding |  | **1.23** |
| 1427076_AT | macrophage expressed gene 1 | [MPEG1](http://www.gene.ucl.ac.uk/cgi-bin/nomenclature/searchgenes.pl?field=symbol&anchor=equals&symbol_search=Search&number=100&format=html&sortby=symbol&match=MPEG1) | protein binding |  | **-10.57** |
| 1427080_AT | RIKEN cDNA 2610036D13 gene | [2610036D13RIK](http://www.gene.ucl.ac.uk/cgi-bin/nomenclature/searchgenes.pl?field=symbol&anchor=equals&symbol_search=Search&number=100&format=html&sortby=symbol&match=2610036D13RIK) | protein binding |  | **1.44** |
| 1427098_AT | WW domain containing E3 ubiquitin protein ligase 1 | [WWP1](http://www.gene.ucl.ac.uk/cgi-bin/nomenclature/searchgenes.pl?field=symbol&anchor=equals&symbol_search=Search&number=100&format=html&sortby=symbol&match=WWP1) | protein binding | [602307](http://www.ncbi.nlm.nih.gov/entrez/dispomim.cgi?id=602307) | **1.69** |
| 1427100_AT | meteorin | [METRN](http://www.gene.ucl.ac.uk/cgi-bin/nomenclature/searchgenes.pl?field=symbol&anchor=equals&symbol_search=Search&number=100&format=html&sortby=symbol&match=METRN) | protein binding |  | **1.93** |
| 1427108_AT | RIKEN cDNA 9530068E07 gene | [9530068E07RIK](http://www.gene.ucl.ac.uk/cgi-bin/nomenclature/searchgenes.pl?field=symbol&anchor=equals&symbol_search=Search&number=100&format=html&sortby=symbol&match=9530068E07RIK) | protein binding |  | **-1.26** |
| 1427115_AT | Myosin, heavy polypeptide 3, skeletal muscle, embryonic | [MYH3](http://www.gene.ucl.ac.uk/cgi-bin/nomenclature/searchgenes.pl?field=symbol&anchor=equals&symbol_search=Search&number=100&format=html&sortby=symbol&match=MYH3) | protein binding | [160720](http://www.ncbi.nlm.nih.gov/entrez/dispomim.cgi?id=160720) | **-130.28** |
| 1427151_AT | RIKEN cDNA 4732486I23 gene | [4732486I23RIK](http://www.gene.ucl.ac.uk/cgi-bin/nomenclature/searchgenes.pl?field=symbol&anchor=equals&symbol_search=Search&number=100&format=html&sortby=symbol&match=4732486I23RIK) | protein binding, bridging |  | **1.58** |
| 1427152_AT | RIKEN cDNA 4732486I23 gene | [4732486I23RIK](http://www.gene.ucl.ac.uk/cgi-bin/nomenclature/searchgenes.pl?field=symbol&anchor=equals&symbol_search=Search&number=100&format=html&sortby=symbol&match=4732486I23RIK) | protein binding, bridging |  | **1.45** |
| 1427153_AT | branched chain ketoacid dehydrogenase E1, beta polypeptide | [BCKDHB](http://www.gene.ucl.ac.uk/cgi-bin/nomenclature/searchgenes.pl?field=symbol&anchor=equals&symbol_search=Search&number=100&format=html&sortby=symbol&match=BCKDHB) | protein binding, bridging | [248611](http://www.ncbi.nlm.nih.gov/entrez/dispomim.cgi?id=248611) | **1.75** |
| 1427157_AT | gb:BB787946 /DB_XREF=gi:16956442 /DB_XREF=BB787946 |  | protein binding;2'-phosphotransferase activity;transferase activity |  | **2.4** |
| 1427163_AT | RIKEN cDNA E130209G04 gene | [E130209G04RIK](http://www.gene.ucl.ac.uk/cgi-bin/nomenclature/searchgenes.pl?field=symbol&anchor=equals&symbol_search=Search&number=100&format=html&sortby=symbol&match=E130209G04RIK) | protein binding;actin binding |  | **1.52** |
| 1427164_AT | interleukin 13 receptor, alpha 1 | [IL13RA1](http://www.gene.ucl.ac.uk/cgi-bin/nomenclature/searchgenes.pl?field=symbol&anchor=equals&symbol_search=Search&number=100&format=html&sortby=symbol&match=IL13RA1) | protein binding;actin binding | [300119](http://www.ncbi.nlm.nih.gov/entrez/dispomim.cgi?id=300119) | **-4.2** |
| 1427165_AT | interleukin 13 receptor, alpha 1 | [IL13RA1](http://www.gene.ucl.ac.uk/cgi-bin/nomenclature/searchgenes.pl?field=symbol&anchor=equals&symbol_search=Search&number=100&format=html&sortby=symbol&match=IL13RA1) | protein binding;actin binding | [300119](http://www.ncbi.nlm.nih.gov/entrez/dispomim.cgi?id=300119) | **-2.34** |
| 1427166_A_AT | receptor-like tyrosine kinase | [RYK](http://www.gene.ucl.ac.uk/cgi-bin/nomenclature/searchgenes.pl?field=symbol&anchor=equals&symbol_search=Search&number=100&format=html&sortby=symbol&match=RYK) | protein binding;actin binding | [600524](http://www.ncbi.nlm.nih.gov/entrez/dispomim.cgi?id=600524) | **1.25** |
| 1427167_AT | expressed sequence AI448196 | [AI448196](http://www.gene.ucl.ac.uk/cgi-bin/nomenclature/searchgenes.pl?field=symbol&anchor=equals&symbol_search=Search&number=100&format=html&sortby=symbol&match=AI448196) | protein binding;actin binding |  | **-1.77** |
| 1427168_A_AT | procollagen, type XIV, alpha 1 | [COL14A1](http://www.gene.ucl.ac.uk/cgi-bin/nomenclature/searchgenes.pl?field=symbol&anchor=equals&symbol_search=Search&number=100&format=html&sortby=symbol&match=COL14A1) | protein binding;actin binding | [120324](http://www.ncbi.nlm.nih.gov/entrez/dispomim.cgi?id=120324) | **-1.59** |
| 1427177_AT | FYVE and coiled-coil domain containing 1 | [FYCO1](http://www.gene.ucl.ac.uk/cgi-bin/nomenclature/searchgenes.pl?field=symbol&anchor=equals&symbol_search=Search&number=100&format=html&sortby=symbol&match=FYCO1) | protein binding;actin binding;calcium ion binding | [607182](http://www.ncbi.nlm.nih.gov/entrez/dispomim.cgi?id=607182) | **1.49** |
| 1427183_AT | epidermal growth factor-containing fibulin-like extracellular matrix protein 1 | [EFEMP1](http://www.gene.ucl.ac.uk/cgi-bin/nomenclature/searchgenes.pl?field=symbol&anchor=equals&symbol_search=Search&number=100&format=html&sortby=symbol&match=EFEMP1) | protein binding;actin binding;calcium ion binding | [601548](http://www.ncbi.nlm.nih.gov/entrez/dispomim.cgi?id=601548) | **2.07** |
| 1427191_AT | natriuretic peptide receptor 2 | [NPR2](http://www.gene.ucl.ac.uk/cgi-bin/nomenclature/searchgenes.pl?field=symbol&anchor=equals&symbol_search=Search&number=100&format=html&sortby=symbol&match=NPR2) | protein binding;actin binding;calcium ion binding | [108961](http://www.ncbi.nlm.nih.gov/entrez/dispomim.cgi?id=108961) | **1.63** |
| 1427201_AT | musculoskeletal, embryonic nuclear protein 1 | [MUSTN1](http://www.gene.ucl.ac.uk/cgi-bin/nomenclature/searchgenes.pl?field=symbol&anchor=equals&symbol_search=Search&number=100&format=html&sortby=symbol&match=MUSTN1) | protein binding;actin binding;calcium ion binding;actin filament binding |  | **-3.26** |
| 1427208_AT | zinc finger protein 451 | [ZFP451](http://www.gene.ucl.ac.uk/cgi-bin/nomenclature/searchgenes.pl?field=symbol&anchor=equals&symbol_search=Search&number=100&format=html&sortby=symbol&match=ZFP451) | protein binding;actin binding;calcium ion binding;actin filament binding |  | **-1.29** |
| 1427212_AT | mitogen-activated protein kinase associated protein 1 | [MAPKAP1](http://www.gene.ucl.ac.uk/cgi-bin/nomenclature/searchgenes.pl?field=symbol&anchor=equals&symbol_search=Search&number=100&format=html&sortby=symbol&match=MAPKAP1) | protein binding;actin binding;calmodulin binding |  | **1.3** |
| 1427213_AT | 6-phosphofructo-2-kinase/fructose-2,6-biphosphatase 1 | [PFKFB1](http://www.gene.ucl.ac.uk/cgi-bin/nomenclature/searchgenes.pl?field=symbol&anchor=equals&symbol_search=Search&number=100&format=html&sortby=symbol&match=PFKFB1) | protein binding;actin binding;calmodulin binding | [311790](http://www.ncbi.nlm.nih.gov/entrez/dispomim.cgi?id=311790) | **2.58** |
| 1427226_AT | epsin 2 | [EPN2](http://www.gene.ucl.ac.uk/cgi-bin/nomenclature/searchgenes.pl?field=symbol&anchor=equals&symbol_search=Search&number=100&format=html&sortby=symbol&match=EPN2) | protein binding;actin binding;clathrin binding;phospholipid binding | [607263](http://www.ncbi.nlm.nih.gov/entrez/dispomim.cgi?id=607263) | **1.46** |
| 1427228_AT | RIKEN cDNA 2410003B16 gene | [2410003B16RIK](http://www.gene.ucl.ac.uk/cgi-bin/nomenclature/searchgenes.pl?field=symbol&anchor=equals&symbol_search=Search&number=100&format=html&sortby=symbol&match=2410003B16RIK) | protein binding;actin binding;cysteine-type endopeptidase activity |  | **-1.54** |
| 1427239_AT | WD repeat domain 10 | [WDR10](http://www.gene.ucl.ac.uk/cgi-bin/nomenclature/searchgenes.pl?field=symbol&anchor=equals&symbol_search=Search&number=100&format=html&sortby=symbol&match=WDR10) | protein binding;actin binding;enzyme binding | [606045](http://www.ncbi.nlm.nih.gov/entrez/dispomim.cgi?id=606045) | **-1.68** |
| 1427250_AT | gb:BI248739 /DB_XREF=gi:14795427 /DB_XREF=60299270 |  | protein binding;actin binding;enzyme binding |  | **-1.22** |
| 1427256_AT | chondroitin sulfate proteoglycan 2 | [CSPG2](http://www.gene.ucl.ac.uk/cgi-bin/nomenclature/searchgenes.pl?field=symbol&anchor=equals&symbol_search=Search&number=100&format=html&sortby=symbol&match=CSPG2) | protein binding;actin binding;GKAP/Homer scaffold activity | [118661](http://www.ncbi.nlm.nih.gov/entrez/dispomim.cgi?id=118661) | **-2.91** |
| 1427258_AT | tripartite motif protein 24 | [TRIM24](http://www.gene.ucl.ac.uk/cgi-bin/nomenclature/searchgenes.pl?field=symbol&anchor=equals&symbol_search=Search&number=100&format=html&sortby=symbol&match=TRIM24) | protein binding;actin binding;motor activity;ATP binding |  | **1.34** |
| 1427275_AT | SMC4 structural maintenance of chromosomes 4-like 1 (yeast) | [SMC4L1](http://www.gene.ucl.ac.uk/cgi-bin/nomenclature/searchgenes.pl?field=symbol&anchor=equals&symbol_search=Search&number=100&format=html&sortby=symbol&match=SMC4L1) | protein binding;actin binding;motor activity;ATP binding;calmodulin binding | [605575](http://www.ncbi.nlm.nih.gov/entrez/dispomim.cgi?id=605575) | **-1.8** |
| 1427276_AT | SMC4 structural maintenance of chromosomes 4-like 1 (yeast) | [SMC4L1](http://www.gene.ucl.ac.uk/cgi-bin/nomenclature/searchgenes.pl?field=symbol&anchor=equals&symbol_search=Search&number=100&format=html&sortby=symbol&match=SMC4L1) | protein binding;actin binding;motor activity;ATP binding;calmodulin binding;microfilament motor activity | [605575](http://www.ncbi.nlm.nih.gov/entrez/dispomim.cgi?id=605575) | **-1.85** |
| 1427278_AT | RIKEN cDNA 1700024K14 gene | [RSNL2](http://www.gene.ucl.ac.uk/cgi-bin/nomenclature/searchgenes.pl?field=symbol&anchor=equals&symbol_search=Search&number=100&format=html&sortby=symbol&match=RSNL2) | protein binding;actin binding;motor activity;ATP binding;microfilament motor activity;calmodulin binding |  | **1.4** |
| 1427279_AT | RIKEN cDNA 1700024K14 gene | [RSNL2](http://www.gene.ucl.ac.uk/cgi-bin/nomenclature/searchgenes.pl?field=symbol&anchor=equals&symbol_search=Search&number=100&format=html&sortby=symbol&match=RSNL2) | protein binding;acyltransferase activity;dihydrolipoyllysine-residue acetyltransferase activity;transferase activity |  | **1.39** |
| 1427282_A_AT | Friedreich ataxia | [FXN](http://www.gene.ucl.ac.uk/cgi-bin/nomenclature/searchgenes.pl?field=symbol&anchor=equals&symbol_search=Search&number=100&format=html&sortby=symbol&match=FXN) | protein binding;acyltransferase activity;transferase activity | [606829](http://www.ncbi.nlm.nih.gov/entrez/dispomim.cgi?id=606829) | **1.39** |
| 1427298_AT | neoplastic progression 1 | [NPN1](http://www.gene.ucl.ac.uk/cgi-bin/nomenclature/searchgenes.pl?field=symbol&anchor=equals&symbol_search=Search&number=100&format=html&sortby=symbol&match=NPN1) | protein binding;amine oxidase activity;oxidoreductase activity;copper ion binding |  | **-3.06** |
| 1427299_AT | ribosomal protein S6 kinase polypeptide 3 | [RPS6KA3](http://www.gene.ucl.ac.uk/cgi-bin/nomenclature/searchgenes.pl?field=symbol&anchor=equals&symbol_search=Search&number=100&format=html&sortby=symbol&match=RPS6KA3) | protein binding;aminopeptidase activity;catalytic activity;hydrolase activity;peptidase activity | [300075](http://www.ncbi.nlm.nih.gov/entrez/dispomim.cgi?id=300075) | **-1.43** |
| 1427301_AT | CD48 antigen | [CD48](http://www.gene.ucl.ac.uk/cgi-bin/nomenclature/searchgenes.pl?field=symbol&anchor=equals&symbol_search=Search&number=100&format=html&sortby=symbol&match=CD48) | protein binding;apoptotic protease activator activity | [109530](http://www.ncbi.nlm.nih.gov/entrez/dispomim.cgi?id=109530) | **-4.76** |
| 1427312_AT | cardiomyopathy associated 5 | [CMYA5](http://www.gene.ucl.ac.uk/cgi-bin/nomenclature/searchgenes.pl?field=symbol&anchor=equals&symbol_search=Search&number=100&format=html&sortby=symbol&match=CMYA5) | protein binding;ATP binding |  | **1.37** |
| 1427318_S_AT | dysferlin | [FER1L3](http://www.gene.ucl.ac.uk/cgi-bin/nomenclature/searchgenes.pl?field=symbol&anchor=equals&symbol_search=Search&number=100&format=html&sortby=symbol&match=FER1L3) | protein binding;ATP binding;apoptotic protease activator activity | [604603](http://www.ncbi.nlm.nih.gov/entrez/dispomim.cgi?id=604603) | **-1.72** |
| 1427322_AT | WD repeat domain 9 | [WDR9](http://www.gene.ucl.ac.uk/cgi-bin/nomenclature/searchgenes.pl?field=symbol&anchor=equals&symbol_search=Search&number=100&format=html&sortby=symbol&match=WDR9) | protein binding;binding |  | **-1.24** |
| 1427334_S_AT | RIKEN cDNA 2810474O19 gene | [2810474O19RIK](http://www.gene.ucl.ac.uk/cgi-bin/nomenclature/searchgenes.pl?field=symbol&anchor=equals&symbol_search=Search&number=100&format=html&sortby=symbol&match=2810474O19RIK) | protein binding;binding |  | **-1.8** |
| 1427347_S_AT | gb:BC003475.1 /DB_XREF=gi:13097482 /FEA=mRNA /CNT= |  | protein binding;calcium ion binding |  | **-4.35** |
| 1427351_S_AT | immunoglobulin heavy chain 6 (heavy chain of IgM) | [IGH-6](http://www.gene.ucl.ac.uk/cgi-bin/nomenclature/searchgenes.pl?field=symbol&anchor=equals&symbol_search=Search&number=100&format=html&sortby=symbol&match=IGH-6) | protein binding;calcium ion binding |  | **-4.92** |
| 1427357_AT | Cytidine deaminase | [CDA](http://www.gene.ucl.ac.uk/cgi-bin/nomenclature/searchgenes.pl?field=symbol&anchor=equals&symbol_search=Search&number=100&format=html&sortby=symbol&match=CDA) | protein binding;calcium ion binding | [123920](http://www.ncbi.nlm.nih.gov/entrez/dispomim.cgi?id=123920) | **1.82** |
| 1427361_AT | homeo box C6 | [HOXC6](http://www.gene.ucl.ac.uk/cgi-bin/nomenclature/searchgenes.pl?field=symbol&anchor=equals&symbol_search=Search&number=100&format=html&sortby=symbol&match=HOXC6) | protein binding;calcium ion binding | [142972](http://www.ncbi.nlm.nih.gov/entrez/dispomim.cgi?id=142972) | **1.35** |
| 1427362_X_AT | homeo box C6 | [HOXC6](http://www.gene.ucl.ac.uk/cgi-bin/nomenclature/searchgenes.pl?field=symbol&anchor=equals&symbol_search=Search&number=100&format=html&sortby=symbol&match=HOXC6) | protein binding;calcium ion binding | [142972](http://www.ncbi.nlm.nih.gov/entrez/dispomim.cgi?id=142972) | **1.44** |
| 1427385_S_AT | Actinin, alpha 1 | [ACTN1](http://www.gene.ucl.ac.uk/cgi-bin/nomenclature/searchgenes.pl?field=symbol&anchor=equals&symbol_search=Search&number=100&format=html&sortby=symbol&match=ACTN1) | protein binding;calcium ion binding |  | **-1.44** |
| 1427388_AT | Leucine-rich repeat-containing 2 | [LRRC2](http://www.gene.ucl.ac.uk/cgi-bin/nomenclature/searchgenes.pl?field=symbol&anchor=equals&symbol_search=Search&number=100&format=html&sortby=symbol&match=LRRC2) | protein binding;calcium ion binding | [607180](http://www.ncbi.nlm.nih.gov/entrez/dispomim.cgi?id=607180) | **1.73** |
| 1427400_AT | lady bird-like homeobox 1 homolog (Drosophila) | [LBX1H](http://www.gene.ucl.ac.uk/cgi-bin/nomenclature/searchgenes.pl?field=symbol&anchor=equals&symbol_search=Search&number=100&format=html&sortby=symbol&match=LBX1H) | protein binding;calcium ion binding |  | **1.35** |
| 1427404_X_AT | enolase 1, alpha non-neuron | [ENO1](http://www.gene.ucl.ac.uk/cgi-bin/nomenclature/searchgenes.pl?field=symbol&anchor=equals&symbol_search=Search&number=100&format=html&sortby=symbol&match=ENO1) | protein binding;calcium ion binding | [172430](http://www.ncbi.nlm.nih.gov/entrez/dispomim.cgi?id=172430) | **-1.56** |
| 1427410_AT | deleted in lymphocytic leukemia, 2 | [DLEU2](http://www.gene.ucl.ac.uk/cgi-bin/nomenclature/searchgenes.pl?field=symbol&anchor=equals&symbol_search=Search&number=100&format=html&sortby=symbol&match=DLEU2) | protein binding;calcium ion binding |  | **-1.96** |
| 1427411_S_AT | deleted in lymphocytic leukemia, 2 | [DLEU2](http://www.gene.ucl.ac.uk/cgi-bin/nomenclature/searchgenes.pl?field=symbol&anchor=equals&symbol_search=Search&number=100&format=html&sortby=symbol&match=DLEU2) | protein binding;calcium ion binding |  | **-1.89** |
| 1427413_A_AT | CUG triplet repeat, RNA binding protein 1 | [CUGBP1](http://www.gene.ucl.ac.uk/cgi-bin/nomenclature/searchgenes.pl?field=symbol&anchor=equals&symbol_search=Search&number=100&format=html&sortby=symbol&match=CUGBP1) | protein binding;calcium ion binding | [601074](http://www.ncbi.nlm.nih.gov/entrez/dispomim.cgi?id=601074) | **-1.74** |
| 1427414_AT | protein kinase, cAMP dependent regulatory, type II alpha | [PRKAR2A](http://www.gene.ucl.ac.uk/cgi-bin/nomenclature/searchgenes.pl?field=symbol&anchor=equals&symbol_search=Search&number=100&format=html&sortby=symbol&match=PRKAR2A) | protein binding;calcium ion binding | [176910](http://www.ncbi.nlm.nih.gov/entrez/dispomim.cgi?id=176910) | **1.7** |
| 1427441_A_AT | succinate-Coenzyme A ligase, GDP-forming, beta subunit | [SUCLG2](http://www.gene.ucl.ac.uk/cgi-bin/nomenclature/searchgenes.pl?field=symbol&anchor=equals&symbol_search=Search&number=100&format=html&sortby=symbol&match=SUCLG2) | protein binding;calcium ion binding | [603922](http://www.ncbi.nlm.nih.gov/entrez/dispomim.cgi?id=603922) | **1.87** |
| 1427447_A_AT | TRIO and F-actin binding protein | [TRIOBP](http://www.gene.ucl.ac.uk/cgi-bin/nomenclature/searchgenes.pl?field=symbol&anchor=equals&symbol_search=Search&number=100&format=html&sortby=symbol&match=TRIOBP) | protein binding;calcium ion binding |  | **1.3** |
| 1427450_X_AT | gb:BI080370 /DB_XREF=gi:14498700 /DB_XREF=60287682 |  | protein binding;calcium ion binding |  | **1.53** |
| 1427465_AT | ATPase, Na+/K+ transporting, alpha 2 polypeptide | [ATP1A2](http://www.gene.ucl.ac.uk/cgi-bin/nomenclature/searchgenes.pl?field=symbol&anchor=equals&symbol_search=Search&number=100&format=html&sortby=symbol&match=ATP1A2) | protein binding;calcium ion binding;ATP-binding cassette (ABC) transporter activity;ATP binding | [182340](http://www.ncbi.nlm.nih.gov/entrez/dispomim.cgi?id=182340) | **1.33** |
| 1427468_AT | protein phosphatase 3, catalytic subunit, beta isoform | [PPP3CB](http://www.gene.ucl.ac.uk/cgi-bin/nomenclature/searchgenes.pl?field=symbol&anchor=equals&symbol_search=Search&number=100&format=html&sortby=symbol&match=PPP3CB) | protein binding;calcium ion binding;growth factor activity | [114106](http://www.ncbi.nlm.nih.gov/entrez/dispomim.cgi?id=114106) | **1.22** |
| 1427471_AT | F-box and leucine-rich repeat protein 3a | [FBXL3](http://www.gene.ucl.ac.uk/cgi-bin/nomenclature/searchgenes.pl?field=symbol&anchor=equals&symbol_search=Search&number=100&format=html&sortby=symbol&match=FBXL3) | protein binding;calcium ion binding;guanyl-nucleotide exchange factor activity;signal transducer activity;Rap guanyl-nucleotide exchange factor activity | [605653](http://www.ncbi.nlm.nih.gov/entrez/dispomim.cgi?id=605653) | **1.35** |
| 1427482_A_AT | carbonic anhydrase 8 | [CAR8](http://www.gene.ucl.ac.uk/cgi-bin/nomenclature/searchgenes.pl?field=symbol&anchor=equals&symbol_search=Search&number=100&format=html&sortby=symbol&match=CAR8) | protein binding;calcium ion binding;protein homodimerization activity |  | **2.4** |
| 1427483_AT | solute carrier family 25 (mitochondrial carrier, phosphate carrier), member 24 | [SLC25A24](http://www.gene.ucl.ac.uk/cgi-bin/nomenclature/searchgenes.pl?field=symbol&anchor=equals&symbol_search=Search&number=100&format=html&sortby=symbol&match=SLC25A24) | protein binding;catalytic activity;aminopeptidase activity;hydrolase activity;peptidase activity | [608744](http://www.ncbi.nlm.nih.gov/entrez/dispomim.cgi?id=608744) | **-2.79** |
| 1427486_AT | protein tyrosine phosphatase, receptor type, B | [PTPRB](http://www.gene.ucl.ac.uk/cgi-bin/nomenclature/searchgenes.pl?field=symbol&anchor=equals&symbol_search=Search&number=100&format=html&sortby=symbol&match=PTPRB) | protein binding;chemokine activity;cytokine activity | [176882](http://www.ncbi.nlm.nih.gov/entrez/dispomim.cgi?id=176882) | **2.33** |
| 1427490_AT | ATP-binding cassette, sub-family B (MDR/TAP), member 7 | [ABCB7](http://www.gene.ucl.ac.uk/cgi-bin/nomenclature/searchgenes.pl?field=symbol&anchor=equals&symbol_search=Search&number=100&format=html&sortby=symbol&match=ABCB7) | protein binding;chromatin binding | [300135](http://www.ncbi.nlm.nih.gov/entrez/dispomim.cgi?id=300135) | **1.28** |
| 1427505_A_AT | CASP2 and RIPK1 domain containing adaptor with death domain | [CRADD](http://www.gene.ucl.ac.uk/cgi-bin/nomenclature/searchgenes.pl?field=symbol&anchor=equals&symbol_search=Search&number=100&format=html&sortby=symbol&match=CRADD) | protein binding;cyclin binding;cyclin-dependent protein kinase inhibitor activity;protein kinase inhibitor activity | [603454](http://www.ncbi.nlm.nih.gov/entrez/dispomim.cgi?id=603454) | **1.36** |
| 1427515_AT | RIKEN cDNA A530088I07 gene | [A530088I07RIK](http://www.gene.ucl.ac.uk/cgi-bin/nomenclature/searchgenes.pl?field=symbol&anchor=equals&symbol_search=Search&number=100&format=html&sortby=symbol&match=A530088I07RIK) | protein binding;cyclin binding;cyclin-dependent protein kinase inhibitor activity;protein kinase inhibitor activity |  | **-2.07** |
| 1427535_S_AT | expressed sequence AW822216 | [AW822216](http://www.gene.ucl.ac.uk/cgi-bin/nomenclature/searchgenes.pl?field=symbol&anchor=equals&symbol_search=Search&number=100&format=html&sortby=symbol&match=AW822216) | protein binding;cyclin-dependent protein kinase 5 activator activity;kinase activity;cytoskeletal protein binding |  | **-1.38** |
| 1427539_A_AT | ZW10 interactor | [ZWINT](http://www.gene.ucl.ac.uk/cgi-bin/nomenclature/searchgenes.pl?field=symbol&anchor=equals&symbol_search=Search&number=100&format=html&sortby=symbol&match=ZWINT) | protein binding;cyclin-dependent protein kinase regulator activity |  | **-2.22** |
| 1427567_A_AT | tropomyosin 3, gamma | [TPM3](http://www.gene.ucl.ac.uk/cgi-bin/nomenclature/searchgenes.pl?field=symbol&anchor=equals&symbol_search=Search&number=100&format=html&sortby=symbol&match=TPM3) | protein binding;cyclin-dependent protein kinase regulator activity;cyclin-dependent protein kinase activity | [191030](http://www.ncbi.nlm.nih.gov/entrez/dispomim.cgi?id=191030) | **-1.57** |
| 1427578_A_AT | integrin beta 4 binding protein | [ITGB4BP](http://www.gene.ucl.ac.uk/cgi-bin/nomenclature/searchgenes.pl?field=symbol&anchor=equals&symbol_search=Search&number=100&format=html&sortby=symbol&match=ITGB4BP) | protein binding;cysteine-type endopeptidase activity;cathepsin L activity;hydrolase activity;actinidain activity;peptidase activity;cysteine-type peptidase activity | [602912](http://www.ncbi.nlm.nih.gov/entrez/dispomim.cgi?id=602912) | **-1.45** |
| 1427661_A_AT | tumor-suppressing subchromosomal transferable fragment 4 | [TSSC4](http://www.gene.ucl.ac.uk/cgi-bin/nomenclature/searchgenes.pl?field=symbol&anchor=equals&symbol_search=Search&number=100&format=html&sortby=symbol&match=TSSC4) | protein binding;damaged DNA binding;transcription factor binding | [603852](http://www.ncbi.nlm.nih.gov/entrez/dispomim.cgi?id=603852) | **-1.67** |
| 1427680_A_AT | nuclear factor I/B | [NFIB](http://www.gene.ucl.ac.uk/cgi-bin/nomenclature/searchgenes.pl?field=symbol&anchor=equals&symbol_search=Search&number=100&format=html&sortby=symbol&match=NFIB) | protein binding;diacylglycerol binding | [600728](http://www.ncbi.nlm.nih.gov/entrez/dispomim.cgi?id=600728) | **1.21** |
| 1427718_A_AT | transformed mouse 3T3 cell double minute 2 | [MDM2](http://www.gene.ucl.ac.uk/cgi-bin/nomenclature/searchgenes.pl?field=symbol&anchor=equals&symbol_search=Search&number=100&format=html&sortby=symbol&match=MDM2) | protein binding;DNA binding | [164785](http://www.ncbi.nlm.nih.gov/entrez/dispomim.cgi?id=164785) | **-1.27** |
| 1427763_A_AT | calcium/calmodulin-dependent protein kinase II, delta | [CAMK2D](http://www.gene.ucl.ac.uk/cgi-bin/nomenclature/searchgenes.pl?field=symbol&anchor=equals&symbol_search=Search&number=100&format=html&sortby=symbol&match=CAMK2D) | protein binding;DNA binding | [607708](http://www.ncbi.nlm.nih.gov/entrez/dispomim.cgi?id=607708) | **-2.05** |
| 1427768_S_AT | myosin, light polypeptide 3 | [MYL3](http://www.gene.ucl.ac.uk/cgi-bin/nomenclature/searchgenes.pl?field=symbol&anchor=equals&symbol_search=Search&number=100&format=html&sortby=symbol&match=MYL3) | protein binding;DNA binding;calcium ion binding;receptor activity;transcription factor activity;transcription regulator activity | [160790](http://www.ncbi.nlm.nih.gov/entrez/dispomim.cgi?id=160790) | **1.75** |
| 1427769_X_AT | gb:X67685.1 /DB_XREF=gi:53299 /GEN=Mylc /FEA=mRNA |  | protein binding;DNA binding;calcium ion binding;receptor activity;transcription factor activity;transcription regulator activity |  | **1.81** |
| 1427771_X_AT | integrin beta 1 (fibronectin receptor beta) | [ITGB1](http://www.gene.ucl.ac.uk/cgi-bin/nomenclature/searchgenes.pl?field=symbol&anchor=equals&symbol_search=Search&number=100&format=html&sortby=symbol&match=ITGB1) | protein binding;DNA binding;DNA-directed RNA polymerase activity;transferase activity | [135630](http://www.ncbi.nlm.nih.gov/entrez/dispomim.cgi?id=135630) | **-1.61** |
| 1427797_S_AT | RIKEN cDNA C920004C08 gene | [CTSE](http://www.gene.ucl.ac.uk/cgi-bin/nomenclature/searchgenes.pl?field=symbol&anchor=equals&symbol_search=Search&number=100&format=html&sortby=symbol&match=CTSE) | protein binding;DNA binding;DNA-directed RNA polymerase activity;transferase activity | [116890](http://www.ncbi.nlm.nih.gov/entrez/dispomim.cgi?id=116890) | **1.47** |
| 1427875_A_AT | RIKEN cDNA 1100001I22 gene | [1100001I22RIK](http://www.gene.ucl.ac.uk/cgi-bin/nomenclature/searchgenes.pl?field=symbol&anchor=equals&symbol_search=Search&number=100&format=html&sortby=symbol&match=1100001I22RIK) | protein binding;DNA binding;DNA-directed RNA polymerase activity;transferase activity |  | **-1.2** |
| 1427876_AT | RIKEN cDNA 2610312B22 gene | [2610312B22RIK](http://www.gene.ucl.ac.uk/cgi-bin/nomenclature/searchgenes.pl?field=symbol&anchor=equals&symbol_search=Search&number=100&format=html&sortby=symbol&match=2610312B22RIK) | protein binding;DNA binding;methionyl aminopeptidase activity;transcription factor activity;metalloexopeptidase activity;hydrolase activity |  | **-1.26** |
| 1427878_AT | RIKEN cDNA 0610010O12 gene | [0610010O12RIK](http://www.gene.ucl.ac.uk/cgi-bin/nomenclature/searchgenes.pl?field=symbol&anchor=equals&symbol_search=Search&number=100&format=html&sortby=symbol&match=0610010O12RIK) | protein binding;DNA binding;methionyl aminopeptidase activity;transcription factor activity;metalloexopeptidase activity;hydrolase activity |  | **1.34** |
| 1427883_A_AT | procollagen, type III, alpha 1 | [COL3A1](http://www.gene.ucl.ac.uk/cgi-bin/nomenclature/searchgenes.pl?field=symbol&anchor=equals&symbol_search=Search&number=100&format=html&sortby=symbol&match=COL3A1) | protein binding;DNA binding;methionyl aminopeptidase activity;transcription factor activity;metalloexopeptidase activity;hydrolase activity | [120180](http://www.ncbi.nlm.nih.gov/entrez/dispomim.cgi?id=120180) | **-1.98** |
| 1427884_AT | procollagen, type III, alpha 1 | [COL3A1](http://www.gene.ucl.ac.uk/cgi-bin/nomenclature/searchgenes.pl?field=symbol&anchor=equals&symbol_search=Search&number=100&format=html&sortby=symbol&match=COL3A1) | protein binding;DNA binding;nucleic acid binding;protein dimerization activity | [120180](http://www.ncbi.nlm.nih.gov/entrez/dispomim.cgi?id=120180) | **-4.16** |
| 1427891_AT | immune associated nucleotide 6 | [GIMAP6](http://www.gene.ucl.ac.uk/cgi-bin/nomenclature/searchgenes.pl?field=symbol&anchor=equals&symbol_search=Search&number=100&format=html&sortby=symbol&match=GIMAP6) | protein binding;DNA binding;nucleic acid binding;protein dimerization activity |  | **1.98** |
| 1427895_AT | RIKEN cDNA 2310004N24 gene | [2310004N24RIK](http://www.gene.ucl.ac.uk/cgi-bin/nomenclature/searchgenes.pl?field=symbol&anchor=equals&symbol_search=Search&number=100&format=html&sortby=symbol&match=2310004N24RIK) | protein binding;DNA binding;protein transporter activity |  | **-1.48** |
| 1427903_AT | Phosphohistidine phosphatase 1 | [PHPT1](http://www.gene.ucl.ac.uk/cgi-bin/nomenclature/searchgenes.pl?field=symbol&anchor=equals&symbol_search=Search&number=100&format=html&sortby=symbol&match=PHPT1) | protein binding;DNA binding;receptor activity |  | **1.22** |
| 1427912_AT | carbonyl reductase 3 | [CBR3](http://www.gene.ucl.ac.uk/cgi-bin/nomenclature/searchgenes.pl?field=symbol&anchor=equals&symbol_search=Search&number=100&format=html&sortby=symbol&match=CBR3) | protein binding;DNA binding;RNA binding;ATP binding;transferase activity | [603608](http://www.ncbi.nlm.nih.gov/entrez/dispomim.cgi?id=603608) | **-1.54** |
| 1427919_AT | sushi-repeat-containing protein, X-linked 2 | [SRPX2](http://www.gene.ucl.ac.uk/cgi-bin/nomenclature/searchgenes.pl?field=symbol&anchor=equals&symbol_search=Search&number=100&format=html&sortby=symbol&match=SRPX2) | protein binding;DNA binding;serine-type endopeptidase inhibitor activity;endopeptidase inhibitor activity;binding |  | **-5.07** |
| 1427927_AT | expressed sequence AW049829 | [AW049829](http://www.gene.ucl.ac.uk/cgi-bin/nomenclature/searchgenes.pl?field=symbol&anchor=equals&symbol_search=Search&number=100&format=html&sortby=symbol&match=AW049829) | protein binding;DNA binding;serine-type endopeptidase inhibitor activity;endopeptidase inhibitor activity;binding |  | **1.33** |
| 1427937_AT | RIKEN cDNA 2610030H06 gene | [2610030H06RIK](http://www.gene.ucl.ac.uk/cgi-bin/nomenclature/searchgenes.pl?field=symbol&anchor=equals&symbol_search=Search&number=100&format=html&sortby=symbol&match=2610030H06RIK) | protein binding;DNA binding;serine-type endopeptidase inhibitor activity;endopeptidase inhibitor activity;binding |  | **-1.36** |
| 1427943_AT | acylphosphatase 2, muscle type | [ACYP2](http://www.gene.ucl.ac.uk/cgi-bin/nomenclature/searchgenes.pl?field=symbol&anchor=equals&symbol_search=Search&number=100&format=html&sortby=symbol&match=ACYP2) | protein binding;DNA binding;transcription corepressor activity;ligand-dependent nuclear receptor interactor activity;receptor activity | [102595](http://www.ncbi.nlm.nih.gov/entrez/dispomim.cgi?id=102595) | **1.64** |
| 1427966_AT | Similar to LOC90268 protein | [LOC432940](http://www.gene.ucl.ac.uk/cgi-bin/nomenclature/searchgenes.pl?field=symbol&anchor=equals&symbol_search=Search&number=100&format=html&sortby=symbol&match=LOC432940) | protein binding;DNA binding;transcription factor activity |  | **-1.81** |
| 1427969_S_AT | RIKEN cDNA 1600021C16 gene | [1600021C16RIK](http://www.gene.ucl.ac.uk/cgi-bin/nomenclature/searchgenes.pl?field=symbol&anchor=equals&symbol_search=Search&number=100&format=html&sortby=symbol&match=1600021C16RIK) | protein binding;DNA binding;transcription factor activity |  | **1.43** |
| 1427982_S_AT | synaptic nuclear envelope 2 | [SYNE2](http://www.gene.ucl.ac.uk/cgi-bin/nomenclature/searchgenes.pl?field=symbol&anchor=equals&symbol_search=Search&number=100&format=html&sortby=symbol&match=SYNE2) | protein binding;DNA binding;transcription factor activity | [608442](http://www.ncbi.nlm.nih.gov/entrez/dispomim.cgi?id=608442) | **1.56** |
| 1427986_A_AT | procollagen, type XVI, alpha 1 | [COL16A1](http://www.gene.ucl.ac.uk/cgi-bin/nomenclature/searchgenes.pl?field=symbol&anchor=equals&symbol_search=Search&number=100&format=html&sortby=symbol&match=COL16A1) | protein binding;DNA binding;transcription factor activity | [120326](http://www.ncbi.nlm.nih.gov/entrez/dispomim.cgi?id=120326) | **-1.57** |
| 1427988_S_AT | scaffold attachment factor B2 | [SAFB2](http://www.gene.ucl.ac.uk/cgi-bin/nomenclature/searchgenes.pl?field=symbol&anchor=equals&symbol_search=Search&number=100&format=html&sortby=symbol&match=SAFB2) | protein binding;DNA binding;transcription factor activity | [608066](http://www.ncbi.nlm.nih.gov/entrez/dispomim.cgi?id=608066) | **-1.37** |
| 1427992_A_AT | RAB12, member RAS oncogene family | [RAB12](http://www.gene.ucl.ac.uk/cgi-bin/nomenclature/searchgenes.pl?field=symbol&anchor=equals&symbol_search=Search&number=100&format=html&sortby=symbol&match=RAB12) | protein binding;DNA binding;transcription factor activity |  | **1.37** |
| 1428011_A_AT | Erbb2 interacting protein | [ERBB2IP](http://www.gene.ucl.ac.uk/cgi-bin/nomenclature/searchgenes.pl?field=symbol&anchor=equals&symbol_search=Search&number=100&format=html&sortby=symbol&match=ERBB2IP) | protein binding;DNA binding;transcription factor activity | [606944](http://www.ncbi.nlm.nih.gov/entrez/dispomim.cgi?id=606944) | **-1.51** |
| 1428014_AT | histone 1, H4h | [HIST1H4H](http://www.gene.ucl.ac.uk/cgi-bin/nomenclature/searchgenes.pl?field=symbol&anchor=equals&symbol_search=Search&number=100&format=html&sortby=symbol&match=HIST1H4H) | protein binding;DNA binding;transcription factor activity | [602828](http://www.ncbi.nlm.nih.gov/entrez/dispomim.cgi?id=602828) | **1.62** |
| 1428016_A_AT | Ras interacting protein 1 | [RASIP1](http://www.gene.ucl.ac.uk/cgi-bin/nomenclature/searchgenes.pl?field=symbol&anchor=equals&symbol_search=Search&number=100&format=html&sortby=symbol&match=RASIP1) | protein binding;DNA binding;transcription factor activity |  | **1.86** |
| 1428018_A_AT | immunoglobulin superfamily, member 7 | [IGSF7](http://www.gene.ucl.ac.uk/cgi-bin/nomenclature/searchgenes.pl?field=symbol&anchor=equals&symbol_search=Search&number=100&format=html&sortby=symbol&match=IGSF7) | protein binding;DNA binding;transcription factor activity |  | **-2.32** |
| 1428021_AT | Methylcrotonoyl-Coenzyme A carboxylase 2 (beta) | [MCCC2](http://www.gene.ucl.ac.uk/cgi-bin/nomenclature/searchgenes.pl?field=symbol&anchor=equals&symbol_search=Search&number=100&format=html&sortby=symbol&match=MCCC2) | protein binding;DNA binding;transcription factor activity | 609014 210210 | **1.95** |
| 1428025_S_AT | phosphatidylinositol transfer protein, cytoplasmic 1 | [PITPNC1](http://www.gene.ucl.ac.uk/cgi-bin/nomenclature/searchgenes.pl?field=symbol&anchor=equals&symbol_search=Search&number=100&format=html&sortby=symbol&match=PITPNC1) | protein binding;DNA binding;transcription factor activity | [605134](http://www.ncbi.nlm.nih.gov/entrez/dispomim.cgi?id=605134) | **1.81** |
| 1428031_AT | G protein coupled receptor 24 | [GPR24](http://www.gene.ucl.ac.uk/cgi-bin/nomenclature/searchgenes.pl?field=symbol&anchor=equals&symbol_search=Search&number=100&format=html&sortby=symbol&match=GPR24) | protein binding;DNA binding;transcription factor activity | [601751](http://www.ncbi.nlm.nih.gov/entrez/dispomim.cgi?id=601751) | **2.23** |
| 1428064_AT | centaurin, delta 2 | [CENTD2](http://www.gene.ucl.ac.uk/cgi-bin/nomenclature/searchgenes.pl?field=symbol&anchor=equals&symbol_search=Search&number=100&format=html&sortby=symbol&match=CENTD2) | protein binding;DNA binding;transcription factor activity | [606646](http://www.ncbi.nlm.nih.gov/entrez/dispomim.cgi?id=606646) | **-1.47** |
| 1428068_AT | RIKEN cDNA 1110030L07 gene | [1110030L07RIK](http://www.gene.ucl.ac.uk/cgi-bin/nomenclature/searchgenes.pl?field=symbol&anchor=equals&symbol_search=Search&number=100&format=html&sortby=symbol&match=1110030L07RIK) | protein binding;DNA binding;transcription factor activity |  | **1.31** |
| 1428074_AT | RIKEN cDNA 2310037P21 gene | [2310037P21RIK](http://www.gene.ucl.ac.uk/cgi-bin/nomenclature/searchgenes.pl?field=symbol&anchor=equals&symbol_search=Search&number=100&format=html&sortby=symbol&match=2310037P21RIK) | protein binding;DNA binding;transcription factor activity;ATP binding |  | **-4.16** |
| 1428075_AT | NADH dehydrogenase (ubiquinone) 1 beta subcomplex 4 | [NDUFB4](http://www.gene.ucl.ac.uk/cgi-bin/nomenclature/searchgenes.pl?field=symbol&anchor=equals&symbol_search=Search&number=100&format=html&sortby=symbol&match=NDUFB4) | protein binding;DNA binding;transcription factor activity;ATP binding | [603840](http://www.ncbi.nlm.nih.gov/entrez/dispomim.cgi?id=603840) | **1.33** |
| 1428076_S_AT | NADH dehydrogenase (ubiquinone) 1 beta subcomplex 4 | [NDUFB4](http://www.gene.ucl.ac.uk/cgi-bin/nomenclature/searchgenes.pl?field=symbol&anchor=equals&symbol_search=Search&number=100&format=html&sortby=symbol&match=NDUFB4) | protein binding;DNA binding;transcription factor activity;ATP binding | [603840](http://www.ncbi.nlm.nih.gov/entrez/dispomim.cgi?id=603840) | **1.37** |
| 1428081_AT | RIKEN cDNA 1810045K06 gene | [1810045K06RIK](http://www.gene.ucl.ac.uk/cgi-bin/nomenclature/searchgenes.pl?field=symbol&anchor=equals&symbol_search=Search&number=100&format=html&sortby=symbol&match=1810045K06RIK) | protein binding;DNA binding;transcription factor activity;RNA polymerase II transcription factor activity, enhancer binding |  | **1.61** |
| 1428094_AT | lysosomal membrane glycoprotein 2 | [LAMP2](http://www.gene.ucl.ac.uk/cgi-bin/nomenclature/searchgenes.pl?field=symbol&anchor=equals&symbol_search=Search&number=100&format=html&sortby=symbol&match=LAMP2) | protein binding;DNA binding;transcription factor activity;transcriptional activator activity;protein dimerization activity;signal transducer activity | [309060](http://www.ncbi.nlm.nih.gov/entrez/dispomim.cgi?id=309060) | **-1.5** |
| 1428099_A_AT | splicing factor, arginine/serine-rich 1 (ASF/SF2) | [SFRS1](http://www.gene.ucl.ac.uk/cgi-bin/nomenclature/searchgenes.pl?field=symbol&anchor=equals&symbol_search=Search&number=100&format=html&sortby=symbol&match=SFRS1) | protein binding;DNA binding;transcriptional repressor activity | [600812](http://www.ncbi.nlm.nih.gov/entrez/dispomim.cgi?id=600812) | **-1.37** |
| 1428103_AT | gb:AV327574 /DB_XREF=gi:16395670 /DB_XREF=AV327574 |  | protein binding;DNA binding;transcriptional repressor activity |  | **-1.24** |
| 1428106_AT | RIKEN cDNA 1300001I01 gene | [1300001I01RIK](http://www.gene.ucl.ac.uk/cgi-bin/nomenclature/searchgenes.pl?field=symbol&anchor=equals&symbol_search=Search&number=100&format=html&sortby=symbol&match=1300001I01RIK) | protein binding;DNA binding;transcriptional repressor activity |  | **1.24** |
| 1428107_AT | SH3-binding domain glutamic acid-rich protein like | [SH3BGRL](http://www.gene.ucl.ac.uk/cgi-bin/nomenclature/searchgenes.pl?field=symbol&anchor=equals&symbol_search=Search&number=100&format=html&sortby=symbol&match=SH3BGRL) | protein binding;DNA binding;transcriptional repressor activity;DNA-directed DNA polymerase activity | [300190](http://www.ncbi.nlm.nih.gov/entrez/dispomim.cgi?id=300190) | **-1.39** |
| 1428108_X_AT | RIKEN cDNA 1110063G11 gene | [1110063G11RIK](http://www.gene.ucl.ac.uk/cgi-bin/nomenclature/searchgenes.pl?field=symbol&anchor=equals&symbol_search=Search&number=100&format=html&sortby=symbol&match=1110063G11RIK) | protein binding;electron transporter activity;acyltransferase activity;transferase activity |  | **1.56** |
| 1428112_AT | arginine-rich, mutated in early stage tumors | [ARMET](http://www.gene.ucl.ac.uk/cgi-bin/nomenclature/searchgenes.pl?field=symbol&anchor=equals&symbol_search=Search&number=100&format=html&sortby=symbol&match=ARMET) | protein binding;electron transporter activity;nitric-oxide synthase activity;oxidoreductase activity;FMN binding;calmodulin binding | [601916](http://www.ncbi.nlm.nih.gov/entrez/dispomim.cgi?id=601916) | **-1.79** |
| 1428116_A_AT | t-complex testis expressed 1 | [TCTEX1](http://www.gene.ucl.ac.uk/cgi-bin/nomenclature/searchgenes.pl?field=symbol&anchor=equals&symbol_search=Search&number=100&format=html&sortby=symbol&match=TCTEX1) | protein binding;eukaryotic initiation factor 4E binding |  | **1.69** |
| 1428125_AT | RIKEN cDNA 4921506J03 gene | [4921506J03RIK](http://www.gene.ucl.ac.uk/cgi-bin/nomenclature/searchgenes.pl?field=symbol&anchor=equals&symbol_search=Search&number=100&format=html&sortby=symbol&match=4921506J03RIK) | protein binding;glucosidase activity;hydrolase activity, hydrolyzing O-glycosyl compounds;hydrolase activity |  | **-1.26** |
| 1428128_AT | RIKEN cDNA 4921506J03 gene | [4921506J03RIK](http://www.gene.ucl.ac.uk/cgi-bin/nomenclature/searchgenes.pl?field=symbol&anchor=equals&symbol_search=Search&number=100&format=html&sortby=symbol&match=4921506J03RIK) | protein binding;G-protein coupled receptor activity;cytokine binding;rhodopsin-like receptor activity;C-C chemokine receptor activity |  | **-1.24** |
| 1428131_A_AT | CDC42 small effector 1 | [CDC42SE1](http://www.gene.ucl.ac.uk/cgi-bin/nomenclature/searchgenes.pl?field=symbol&anchor=equals&symbol_search=Search&number=100&format=html&sortby=symbol&match=CDC42SE1) | protein binding;G-protein coupled receptor activity;rhodopsin-like receptor activity;cytokine binding;C-C chemokine receptor activity |  | **-1.73** |
| 1428134_AT | RIKEN cDNA 2310005O14 gene | [2310005O14RIK](http://www.gene.ucl.ac.uk/cgi-bin/nomenclature/searchgenes.pl?field=symbol&anchor=equals&symbol_search=Search&number=100&format=html&sortby=symbol&match=2310005O14RIK) | protein binding;growth factor activity;integrin binding;cytokine activity |  | **1.64** |
| 1428136_AT | RIKEN cDNA 2210415K03 gene | [2210415K03RIK](http://www.gene.ucl.ac.uk/cgi-bin/nomenclature/searchgenes.pl?field=symbol&anchor=equals&symbol_search=Search&number=100&format=html&sortby=symbol&match=2210415K03RIK) | protein binding;growth factor activity;transforming growth factor beta receptor binding |  | **-2.06** |
| 1428140_AT | 3-oxoacid CoA transferase 1 | [OXCT1](http://www.gene.ucl.ac.uk/cgi-bin/nomenclature/searchgenes.pl?field=symbol&anchor=equals&symbol_search=Search&number=100&format=html&sortby=symbol&match=OXCT1) | protein binding;GTP binding | [245050](http://www.ncbi.nlm.nih.gov/entrez/dispomim.cgi?id=245050) | **1.26** |
| 1428141_AT | golgi associated, gamma adaptin ear containing, ARF binding protein 2 | [GGA2](http://www.gene.ucl.ac.uk/cgi-bin/nomenclature/searchgenes.pl?field=symbol&anchor=equals&symbol_search=Search&number=100&format=html&sortby=symbol&match=GGA2) | protein binding;GTP binding | [606005](http://www.ncbi.nlm.nih.gov/entrez/dispomim.cgi?id=606005) | **-1.69** |
| 1428143_A_AT | patatin-like phospholipase domain containing 2 | [PNPLA2](http://www.gene.ucl.ac.uk/cgi-bin/nomenclature/searchgenes.pl?field=symbol&anchor=equals&symbol_search=Search&number=100&format=html&sortby=symbol&match=PNPLA2) | protein binding;GTP binding | [609059](http://www.ncbi.nlm.nih.gov/entrez/dispomim.cgi?id=609059) | **1.49** |
| 1428145_AT | acetyl-Coenzyme A acyltransferase 2 (mitochondrial 3-oxoacyl-Coenzyme A thiolase) | [ACAA2](http://www.gene.ucl.ac.uk/cgi-bin/nomenclature/searchgenes.pl?field=symbol&anchor=equals&symbol_search=Search&number=100&format=html&sortby=symbol&match=ACAA2) | protein binding;GTP binding | [604770](http://www.ncbi.nlm.nih.gov/entrez/dispomim.cgi?id=604770) | **1.8** |
| 1428146_S_AT | acetyl-Coenzyme A acyltransferase 2 (mitochondrial 3-oxoacyl-Coenzyme A thiolase) | [ACAA2](http://www.gene.ucl.ac.uk/cgi-bin/nomenclature/searchgenes.pl?field=symbol&anchor=equals&symbol_search=Search&number=100&format=html&sortby=symbol&match=ACAA2) | protein binding;GTP binding;hydrolase activity | [604770](http://www.ncbi.nlm.nih.gov/entrez/dispomim.cgi?id=604770) | **1.56** |
| 1428148_S_AT | coronin 7 | [CORO7](http://www.gene.ucl.ac.uk/cgi-bin/nomenclature/searchgenes.pl?field=symbol&anchor=equals&symbol_search=Search&number=100&format=html&sortby=symbol&match=CORO7) | protein binding;GTP binding;hydrolase activity |  | **-1.36** |
| 1428158_AT | AKT1 substrate 1 (proline-rich) | [AKT1S1](http://www.gene.ucl.ac.uk/cgi-bin/nomenclature/searchgenes.pl?field=symbol&anchor=equals&symbol_search=Search&number=100&format=html&sortby=symbol&match=AKT1S1) | protein binding;GTP binding;hydrolase activity |  | **1.27** |
| 1428159_S_AT | NADH dehydrogenase (ubiquinone) 1, alpha/beta subcomplex, 1 | [NDUFAB1](http://www.gene.ucl.ac.uk/cgi-bin/nomenclature/searchgenes.pl?field=symbol&anchor=equals&symbol_search=Search&number=100&format=html&sortby=symbol&match=NDUFAB1) | protein binding;GTP binding;protein transporter activity | [603836](http://www.ncbi.nlm.nih.gov/entrez/dispomim.cgi?id=603836) | **1.37** |
| 1428163_AT | SAR1a gene homolog 2 (S. cerevisiae) | [SARA2](http://www.gene.ucl.ac.uk/cgi-bin/nomenclature/searchgenes.pl?field=symbol&anchor=equals&symbol_search=Search&number=100&format=html&sortby=symbol&match=SARA2) | protein binding;GTPase activator activity | [607690](http://www.ncbi.nlm.nih.gov/entrez/dispomim.cgi?id=607690) | **1.39** |
| 1428176_AT | endothelial differentiation, sphingolipid G-protein-coupled receptor, 5 | [EDG5](http://www.gene.ucl.ac.uk/cgi-bin/nomenclature/searchgenes.pl?field=symbol&anchor=equals&symbol_search=Search&number=100&format=html&sortby=symbol&match=EDG5) | protein binding;GTPase activator activity;SH3 domain binding | [605111](http://www.ncbi.nlm.nih.gov/entrez/dispomim.cgi?id=605111) | **-1.75** |
| 1428178_S_AT | trafficking protein particle complex 6B | [TRAPPC6B](http://www.gene.ucl.ac.uk/cgi-bin/nomenclature/searchgenes.pl?field=symbol&anchor=equals&symbol_search=Search&number=100&format=html&sortby=symbol&match=TRAPPC6B) | protein binding;GTPase activator activity;signal transducer activity |  | **1.3** |
| 1428179_AT | NADH dehydrogenase (ubiquinone) flavoprotein 2 | [NDUFV2](http://www.gene.ucl.ac.uk/cgi-bin/nomenclature/searchgenes.pl?field=symbol&anchor=equals&symbol_search=Search&number=100&format=html&sortby=symbol&match=NDUFV2) | protein binding;GTPase activator activity;signal transducer activity | [600532](http://www.ncbi.nlm.nih.gov/entrez/dispomim.cgi?id=600532) | **1.22** |
| 1428180_AT | RIKEN cDNA 2810422J05 gene | [2810422J05RIK](http://www.gene.ucl.ac.uk/cgi-bin/nomenclature/searchgenes.pl?field=symbol&anchor=equals&symbol_search=Search&number=100&format=html&sortby=symbol&match=2810422J05RIK) | protein binding;GTPase activity;GTP binding |  | **1.25** |
| 1428181_AT | electron transferring flavoprotein, beta polypeptide | [ETFB](http://www.gene.ucl.ac.uk/cgi-bin/nomenclature/searchgenes.pl?field=symbol&anchor=equals&symbol_search=Search&number=100&format=html&sortby=symbol&match=ETFB) | protein binding;GTPase activity;GTP binding | [130410](http://www.ncbi.nlm.nih.gov/entrez/dispomim.cgi?id=130410) | **1.45** |
| 1428183_AT | gb:BG793484 /DB_XREF=gi:14129054 /DB_XREF=UTSW_SM1 |  | protein binding;GTPase activity;GTP binding |  | **1.58** |
| 1428193_AT | ubiquitin specific protease 9, X chromosome | [USP9X](http://www.gene.ucl.ac.uk/cgi-bin/nomenclature/searchgenes.pl?field=symbol&anchor=equals&symbol_search=Search&number=100&format=html&sortby=symbol&match=USP9X) | protein binding;GTPase activity;GTP binding | [300072](http://www.ncbi.nlm.nih.gov/entrez/dispomim.cgi?id=300072) | **1.29** |
| 1428194_AT | ubiquitin specific protease 9, X chromosome | [USP9X](http://www.gene.ucl.ac.uk/cgi-bin/nomenclature/searchgenes.pl?field=symbol&anchor=equals&symbol_search=Search&number=100&format=html&sortby=symbol&match=USP9X) | protein binding;GTPase activity;GTP binding | [300072](http://www.ncbi.nlm.nih.gov/entrez/dispomim.cgi?id=300072) | **1.2** |
| 1428198_AT | RIKEN cDNA 4930578F03 gene | [4930578F03RIK](http://www.gene.ucl.ac.uk/cgi-bin/nomenclature/searchgenes.pl?field=symbol&anchor=equals&symbol_search=Search&number=100&format=html&sortby=symbol&match=4930578F03RIK) | protein binding;GTPase activity;GTP binding;hydrolase activity |  | **1.46** |
| 1428225_S_AT | heterogeneous nuclear ribonucleoprotein D-like | [HNRPDL](http://www.gene.ucl.ac.uk/cgi-bin/nomenclature/searchgenes.pl?field=symbol&anchor=equals&symbol_search=Search&number=100&format=html&sortby=symbol&match=HNRPDL) | protein binding;GTPase activity;GTP binding;hydrolase activity | [607137](http://www.ncbi.nlm.nih.gov/entrez/dispomim.cgi?id=607137) | **-1.45** |
| 1428232_AT | cleavage and polyadenylation specific factor 6 | [CPSF6](http://www.gene.ucl.ac.uk/cgi-bin/nomenclature/searchgenes.pl?field=symbol&anchor=equals&symbol_search=Search&number=100&format=html&sortby=symbol&match=CPSF6) | protein binding;GTPase activity;GTP binding;protein transporter activity | [604979](http://www.ncbi.nlm.nih.gov/entrez/dispomim.cgi?id=604979) | **-1.49** |
| 1428235_AT | succinate dehydrogenase complex, subunit D, integral membrane protein | [SDHD](http://www.gene.ucl.ac.uk/cgi-bin/nomenclature/searchgenes.pl?field=symbol&anchor=equals&symbol_search=Search&number=100&format=html&sortby=symbol&match=SDHD) | protein binding;GTPase activity;GTP binding;signal transducer activity | [602690](http://www.ncbi.nlm.nih.gov/entrez/dispomim.cgi?id=602690) | **1.26** |
| 1428242_AT | RIKEN cDNA 6330406L22 gene | [6330406L22RIK](http://www.gene.ucl.ac.uk/cgi-bin/nomenclature/searchgenes.pl?field=symbol&anchor=equals&symbol_search=Search&number=100&format=html&sortby=symbol&match=6330406L22RIK) | protein binding;GTPase inhibitor activity;RAN protein binding;protein transporter activity;nuclear localization sequence binding |  | **-1.51** |
| 1428255_AT | Luc7 homolog (S. cerevisiae)-like | [LUC7L](http://www.gene.ucl.ac.uk/cgi-bin/nomenclature/searchgenes.pl?field=symbol&anchor=equals&symbol_search=Search&number=100&format=html&sortby=symbol&match=LUC7L) | protein binding;GTPase inhibitor activity;RAN protein binding;protein transporter activity;nuclear localization sequence binding | [607782](http://www.ncbi.nlm.nih.gov/entrez/dispomim.cgi?id=607782) | **-1.45** |
| 1428257_S_AT | dynein, cytoplasmic, light chain 2A | [DNCL2A](http://www.gene.ucl.ac.uk/cgi-bin/nomenclature/searchgenes.pl?field=symbol&anchor=equals&symbol_search=Search&number=100&format=html&sortby=symbol&match=DNCL2A) | protein binding;guanyl-nucleotide exchange factor activity | [607167](http://www.ncbi.nlm.nih.gov/entrez/dispomim.cgi?id=607167) | **-1.37** |
| 1428261_AT | RIKEN cDNA 2310042L06 gene | [2310042L06RIK](http://www.gene.ucl.ac.uk/cgi-bin/nomenclature/searchgenes.pl?field=symbol&anchor=equals&symbol_search=Search&number=100&format=html&sortby=symbol&match=2310042L06RIK) | protein binding;guanyl-nucleotide exchange factor activity;SH3/SH2 adaptor protein activity;signal transducer activity |  | **-2.12** |
| 1428266_AT | myosin, light polypeptide 3 | [MYL3](http://www.gene.ucl.ac.uk/cgi-bin/nomenclature/searchgenes.pl?field=symbol&anchor=equals&symbol_search=Search&number=100&format=html&sortby=symbol&match=MYL3) | protein binding;guanyl-nucleotide exchange factor adaptor activity | [160790](http://www.ncbi.nlm.nih.gov/entrez/dispomim.cgi?id=160790) | **1.81** |
| 1428269_A_AT | RIKEN cDNA 2410004H05 gene | [2410004H05RIK](http://www.gene.ucl.ac.uk/cgi-bin/nomenclature/searchgenes.pl?field=symbol&anchor=equals&symbol_search=Search&number=100&format=html&sortby=symbol&match=2410004H05RIK) | protein binding;heparin binding |  | **-1.55** |
| 1428270_AT | RIKEN cDNA 2410004H05 gene | [2410004H05RIK](http://www.gene.ucl.ac.uk/cgi-bin/nomenclature/searchgenes.pl?field=symbol&anchor=equals&symbol_search=Search&number=100&format=html&sortby=symbol&match=2410004H05RIK) | protein binding;heparin binding |  | **-1.33** |
| 1428279_A_AT | ataxin 7-like 4 | [ATXN7L4](http://www.gene.ucl.ac.uk/cgi-bin/nomenclature/searchgenes.pl?field=symbol&anchor=equals&symbol_search=Search&number=100&format=html&sortby=symbol&match=ATXN7L4) | protein binding;heparin binding |  | **1.3** |
| 1428283_AT | cytochrome P450, family 2, subfamily s, polypeptide 1 | [CYP2S1](http://www.gene.ucl.ac.uk/cgi-bin/nomenclature/searchgenes.pl?field=symbol&anchor=equals&symbol_search=Search&number=100&format=html&sortby=symbol&match=CYP2S1) | protein binding;heparin binding |  | **1.75** |
| 1428290_AT | mitochondrial intermediate peptidase | [MIPEP](http://www.gene.ucl.ac.uk/cgi-bin/nomenclature/searchgenes.pl?field=symbol&anchor=equals&symbol_search=Search&number=100&format=html&sortby=symbol&match=MIPEP) | protein binding;heparin binding | [602241](http://www.ncbi.nlm.nih.gov/entrez/dispomim.cgi?id=602241) | **1.67** |
| 1428295_AT | synaptopodin 2-like | [SYNPO2L](http://www.gene.ucl.ac.uk/cgi-bin/nomenclature/searchgenes.pl?field=symbol&anchor=equals&symbol_search=Search&number=100&format=html&sortby=symbol&match=SYNPO2L) | protein binding;heparin binding |  | **-1.75** |
| 1428297_AT | mitogen activated protein kinase kinase kinase kinase 2 | [MAP4K2](http://www.gene.ucl.ac.uk/cgi-bin/nomenclature/searchgenes.pl?field=symbol&anchor=equals&symbol_search=Search&number=100&format=html&sortby=symbol&match=MAP4K2) | protein binding;heparin binding | [603166](http://www.ncbi.nlm.nih.gov/entrez/dispomim.cgi?id=603166) | **1.28** |
| 1428307_AT | zinc finger, DHHC domain containing 13 | [ZDHHC13](http://www.gene.ucl.ac.uk/cgi-bin/nomenclature/searchgenes.pl?field=symbol&anchor=equals&symbol_search=Search&number=100&format=html&sortby=symbol&match=ZDHHC13) | protein binding;heparin binding |  | **-1.59** |
| 1428308_AT | p53 and DNA damage regulated 1 | [PDRG1](http://www.gene.ucl.ac.uk/cgi-bin/nomenclature/searchgenes.pl?field=symbol&anchor=equals&symbol_search=Search&number=100&format=html&sortby=symbol&match=PDRG1) | protein binding;histone deacetylase activity;transcription corepressor activity;transcription factor binding;transcriptional repressor activity;hydrolase activity;specific transcriptional repressor activity |  | **1.25** |
| 1428315_AT | EBNA1 binding protein 2 | [EBNA1BP2](http://www.gene.ucl.ac.uk/cgi-bin/nomenclature/searchgenes.pl?field=symbol&anchor=equals&symbol_search=Search&number=100&format=html&sortby=symbol&match=EBNA1BP2) | protein binding;histone deacetylase activity;transcription corepressor activity;transcription factor binding;transcriptional repressor activity;hydrolase activity;specific transcriptional repressor activity |  | **-1.32** |
| 1428322_A_AT | NADH dehydrogenase (ubiquinone) 1 beta subcomplex, 10 | [NDUFB10](http://www.gene.ucl.ac.uk/cgi-bin/nomenclature/searchgenes.pl?field=symbol&anchor=equals&symbol_search=Search&number=100&format=html&sortby=symbol&match=NDUFB10) | protein binding;histone deacetylase activity;transcription factor activity;transcription factor binding;hydrolase activity | [603843](http://www.ncbi.nlm.nih.gov/entrez/dispomim.cgi?id=603843) | **1.24** |
| 1428323_AT | glycerol phosphate dehydrogenase 2, mitochondrial | [GPD2](http://www.gene.ucl.ac.uk/cgi-bin/nomenclature/searchgenes.pl?field=symbol&anchor=equals&symbol_search=Search&number=100&format=html&sortby=symbol&match=GPD2) | protein binding;hyaluronic acid binding;receptor activity | [138430](http://www.ncbi.nlm.nih.gov/entrez/dispomim.cgi?id=138430) | **1.6** |
| 1428325_AT | RIKEN cDNA 2610019P18 gene | [2610019P18RIK](http://www.gene.ucl.ac.uk/cgi-bin/nomenclature/searchgenes.pl?field=symbol&anchor=equals&symbol_search=Search&number=100&format=html&sortby=symbol&match=2610019P18RIK) | protein binding;hyaluronic acid binding;receptor activity |  | **-1.53** |
| 1428334_AT | osteopetrosis associated transmembrane protein 1 | [OSTM1](http://www.gene.ucl.ac.uk/cgi-bin/nomenclature/searchgenes.pl?field=symbol&anchor=equals&symbol_search=Search&number=100&format=html&sortby=symbol&match=OSTM1) | protein binding;hydrogen-exporting ATPase activity, phosphorylative mechanism;hydrogen-transporting ATP synthase activity, rotational mechanism;hydrogen ion transporter activity;hydrogen-transporting ATPase activity, rotational mechanism;hydrolase activity | [607649](http://www.ncbi.nlm.nih.gov/entrez/dispomim.cgi?id=607649) | **1.36** |
| 1428342_AT | REST corepressor 3 | [RCOR3](http://www.gene.ucl.ac.uk/cgi-bin/nomenclature/searchgenes.pl?field=symbol&anchor=equals&symbol_search=Search&number=100&format=html&sortby=symbol&match=RCOR3) | protein binding;hydrogen-exporting ATPase activity, phosphorylative mechanism;hydrogen-transporting ATP synthase activity, rotational mechanism;hydrogen ion transporter activity;hydrogen-transporting ATPase activity, rotational mechanism;hydrolase activity |  | **1.3** |
| 1428345_AT | RIKEN cDNA 4932443D16 gene | [4932443D16RIK](http://www.gene.ucl.ac.uk/cgi-bin/nomenclature/searchgenes.pl?field=symbol&anchor=equals&symbol_search=Search&number=100&format=html&sortby=symbol&match=4932443D16RIK) | protein binding;hydrolase activity;caspase activity;cysteine-type peptidase activity;peptidase activity |  | **2.23** |
| 1428347_AT | gb:AK005148.1 /DB_XREF=gi:12836871 /FEA=mRNA /CNT= |  | protein binding;insulin-like growth factor binding |  | **-6.84** |
| 1428361_X_AT | gb:AK011116.1 /DB_XREF=gi:12847032 /GEN=Hba-a1 /FE |  | protein binding;insulin-like growth factor receptor binding;growth factor activity;hormone activity |  | **2.48** |
| 1428365_A_AT | protease, serine, 15 | [PRSS15](http://www.gene.ucl.ac.uk/cgi-bin/nomenclature/searchgenes.pl?field=symbol&anchor=equals&symbol_search=Search&number=100&format=html&sortby=symbol&match=PRSS15) | protein binding;insulin-like growth factor receptor binding;growth factor activity;hormone activity | [605490](http://www.ncbi.nlm.nih.gov/entrez/dispomim.cgi?id=605490) | **1.22** |
| 1428382_AT | RIKEN cDNA 5930405J04 gene | [5930405J04RIK](http://www.gene.ucl.ac.uk/cgi-bin/nomenclature/searchgenes.pl?field=symbol&anchor=equals&symbol_search=Search&number=100&format=html&sortby=symbol&match=5930405J04RIK) | protein binding;insulin-like growth factor receptor binding;growth factor activity;hormone activity |  | **1.23** |
| 1428391_AT | RAB3A interacting protein (rabin3)-like 1 | [RAB3IL1](http://www.gene.ucl.ac.uk/cgi-bin/nomenclature/searchgenes.pl?field=symbol&anchor=equals&symbol_search=Search&number=100&format=html&sortby=symbol&match=RAB3IL1) | protein binding;ion channel activity;voltage-gated potassium channel activity |  | **-1.7** |
| 1428392_AT | Ras association (RalGDS/AF-6) domain family 2 | [RASSF2](http://www.gene.ucl.ac.uk/cgi-bin/nomenclature/searchgenes.pl?field=symbol&anchor=equals&symbol_search=Search&number=100&format=html&sortby=symbol&match=RASSF2) | protein binding;ion channel activity;voltage-gated potassium channel activity |  | **-1.7** |
| 1428394_AT | leucine rich repeat containing 8 | [LRRC8](http://www.gene.ucl.ac.uk/cgi-bin/nomenclature/searchgenes.pl?field=symbol&anchor=equals&symbol_search=Search&number=100&format=html&sortby=symbol&match=LRRC8) | protein binding;ion channel activity;voltage-gated potassium channel activity | [608360](http://www.ncbi.nlm.nih.gov/entrez/dispomim.cgi?id=608360) | **2.25** |
| 1428411_AT | RIKEN cDNA 1700020I14 gene | [1700020I14RIK](http://www.gene.ucl.ac.uk/cgi-bin/nomenclature/searchgenes.pl?field=symbol&anchor=equals&symbol_search=Search&number=100&format=html&sortby=symbol&match=1700020I14RIK) | protein binding;ion channel activity;voltage-gated potassium channel activity |  | **1.28** |
| 1428416_AT | RIKEN cDNA 3110050N22 gene | [3110050N22RIK](http://www.gene.ucl.ac.uk/cgi-bin/nomenclature/searchgenes.pl?field=symbol&anchor=equals&symbol_search=Search&number=100&format=html&sortby=symbol&match=3110050N22RIK) | protein binding;isomerase activity;electron transporter activity |  | **-3.12** |
| 1428417_AT | RIKEN cDNA 3110050N22 gene | [3110050N22RIK](http://www.gene.ucl.ac.uk/cgi-bin/nomenclature/searchgenes.pl?field=symbol&anchor=equals&symbol_search=Search&number=100&format=html&sortby=symbol&match=3110050N22RIK) | protein binding;isomerase activity;electron transporter activity |  | **-2.14** |
| 1428418_S_AT | RIKEN cDNA 3110050N22 gene | [3110050N22RIK](http://www.gene.ucl.ac.uk/cgi-bin/nomenclature/searchgenes.pl?field=symbol&anchor=equals&symbol_search=Search&number=100&format=html&sortby=symbol&match=3110050N22RIK) | protein binding;kinase activity |  | **-2.57** |
| 1428420_A_AT | RIKEN cDNA 1200009I06 gene | [1200009I06RIK](http://www.gene.ucl.ac.uk/cgi-bin/nomenclature/searchgenes.pl?field=symbol&anchor=equals&symbol_search=Search&number=100&format=html&sortby=symbol&match=1200009I06RIK) | protein binding;kinase activity;cyclin-dependent protein kinase inhibitor activity;protein kinase inhibitor activity |  | **-1.96** |
| 1428423_AT | ring finger protein 3 | [RNF3](http://www.gene.ucl.ac.uk/cgi-bin/nomenclature/searchgenes.pl?field=symbol&anchor=equals&symbol_search=Search&number=100&format=html&sortby=symbol&match=RNF3) | protein binding;kinase activity;protein kinase activity;cyclin-dependent protein kinase regulator activity |  | **-1.32** |
| 1428432_AT | RIKEN cDNA 2310047A01 gene | [2310047A01RIK](http://www.gene.ucl.ac.uk/cgi-bin/nomenclature/searchgenes.pl?field=symbol&anchor=equals&symbol_search=Search&number=100&format=html&sortby=symbol&match=2310047A01RIK) | protein binding;kinase activity;protein kinase activity;magnesium ion binding;protein serine/threonine kinase activity;ATP binding;protein-tyrosine kinase activity;transferase activity |  | **-1.29** |
| 1428440_AT | solute carrier family 25 (mitochondrial carrier, Aralar), member 12 | [SLC25A12](http://www.gene.ucl.ac.uk/cgi-bin/nomenclature/searchgenes.pl?field=symbol&anchor=equals&symbol_search=Search&number=100&format=html&sortby=symbol&match=SLC25A12) | protein binding;kinase activity;protein kinase activity;magnesium ion binding;protein serine/threonine kinase activity;protein-tyrosine kinase activity;ATP binding;transferase activity | [603667](http://www.ncbi.nlm.nih.gov/entrez/dispomim.cgi?id=603667) | **1.39** |
| 1428441_AT | RIKEN cDNA 1500009M05 gene | [1500009M05RIK](http://www.gene.ucl.ac.uk/cgi-bin/nomenclature/searchgenes.pl?field=symbol&anchor=equals&symbol_search=Search&number=100&format=html&sortby=symbol&match=1500009M05RIK) | protein binding;kinase activity;protein kinase activity;protein serine/threonine kinase activity;ATP binding;cyclin-dependent protein kinase activity;transferase activity |  | **1.4** |
| 1428444_AT | ankyrin repeat and SOCS box-containing protein 2 | [ASB2](http://www.gene.ucl.ac.uk/cgi-bin/nomenclature/searchgenes.pl?field=symbol&anchor=equals&symbol_search=Search&number=100&format=html&sortby=symbol&match=ASB2) | protein binding;kinase activity;protein kinase activity;protein serine/threonine kinase activity;ATP binding;cyclin-dependent protein kinase activity;transferase activity | [605759](http://www.ncbi.nlm.nih.gov/entrez/dispomim.cgi?id=605759) | **1.72** |
| 1428464_AT | NADH dehydrogenase (ubiquinone) 1 alpha subcomplex, 3 | [NDUFA3](http://www.gene.ucl.ac.uk/cgi-bin/nomenclature/searchgenes.pl?field=symbol&anchor=equals&symbol_search=Search&number=100&format=html&sortby=symbol&match=NDUFA3) | protein binding;kinase activity;protein kinase activity;protein serine/threonine kinase activity;ATP binding;protein-tyrosine kinase activity;transferase activity | [603832](http://www.ncbi.nlm.nih.gov/entrez/dispomim.cgi?id=603832) | **1.37** |
| 1428489_AT | RIKEN cDNA 2700088M22 gene | [2700088M22RIK](http://www.gene.ucl.ac.uk/cgi-bin/nomenclature/searchgenes.pl?field=symbol&anchor=equals&symbol_search=Search&number=100&format=html&sortby=symbol&match=2700088M22RIK) | protein binding;kinase activity;protein kinase activity;protein serine/threonine kinase activity;ATP binding;transferase activity |  | **1.37** |
| 1428495_AT | RIKEN cDNA 2410003K15 gene | [2410003K15RIK](http://www.gene.ucl.ac.uk/cgi-bin/nomenclature/searchgenes.pl?field=symbol&anchor=equals&symbol_search=Search&number=100&format=html&sortby=symbol&match=2410003K15RIK) | protein binding;kinase activity;protein kinase activity;protein serine/threonine kinase activity;ATP binding;transferase activity |  | **1.37** |
| 1428509_AT | myosin IE | [MYO1E](http://www.gene.ucl.ac.uk/cgi-bin/nomenclature/searchgenes.pl?field=symbol&anchor=equals&symbol_search=Search&number=100&format=html&sortby=symbol&match=MYO1E) | protein binding;kinase activity;receptor activity;ATP binding;protein-tyrosine kinase activity;transferase activity | [601479](http://www.ncbi.nlm.nih.gov/entrez/dispomim.cgi?id=601479) | **-1.49** |
| 1428512_AT | basic helix-loop-helix domain containing, class B9 | [BHLHB9](http://www.gene.ucl.ac.uk/cgi-bin/nomenclature/searchgenes.pl?field=symbol&anchor=equals&symbol_search=Search&number=100&format=html&sortby=symbol&match=BHLHB9) | protein binding;ligase activity;ubiquitin-protein ligase activity |  | **-1.52** |
| 1428513_AT | RIKEN cDNA 1810009B06 gene | [1810009B06RIK](http://www.gene.ucl.ac.uk/cgi-bin/nomenclature/searchgenes.pl?field=symbol&anchor=equals&symbol_search=Search&number=100&format=html&sortby=symbol&match=1810009B06RIK) | protein binding;ligase activity;ubiquitin-protein ligase activity |  | **1.3** |
| 1428515_AT | RIKEN cDNA 2410012H22 gene | [2410012H22RIK](http://www.gene.ucl.ac.uk/cgi-bin/nomenclature/searchgenes.pl?field=symbol&anchor=equals&symbol_search=Search&number=100&format=html&sortby=symbol&match=2410012H22RIK) | protein binding;ligase activity;ubiquitin-protein ligase activity |  | **1.67** |
| 1428516_A_AT | RIKEN cDNA 2310045B01 gene | [SPATA11](http://www.gene.ucl.ac.uk/cgi-bin/nomenclature/searchgenes.pl?field=symbol&anchor=equals&symbol_search=Search&number=100&format=html&sortby=symbol&match=SPATA11) | protein binding;ligase activity;ubiquitin-protein ligase activity;ubiquitin conjugating enzyme activity |  | **1.44** |
| 1428519_AT | RIKEN cDNA 2610528E23 gene | [2610528E23RIK](http://www.gene.ucl.ac.uk/cgi-bin/nomenclature/searchgenes.pl?field=symbol&anchor=equals&symbol_search=Search&number=100&format=html&sortby=symbol&match=2610528E23RIK) | protein binding;ligase activity;ubiquitin-protein ligase activity;ubiquitin conjugating enzyme activity |  | **1.62** |
| 1428527_AT | Sorting nexin 7 | [SNX7](http://www.gene.ucl.ac.uk/cgi-bin/nomenclature/searchgenes.pl?field=symbol&anchor=equals&symbol_search=Search&number=100&format=html&sortby=symbol&match=SNX7) | protein binding;magnesium ion binding;exonuclease activity |  | **-1.72** |
| 1428529_AT | RIKEN cDNA 2810026P18 gene | [2810026P18RIK](http://www.gene.ucl.ac.uk/cgi-bin/nomenclature/searchgenes.pl?field=symbol&anchor=equals&symbol_search=Search&number=100&format=html&sortby=symbol&match=2810026P18RIK) | protein binding;magnesium ion binding;ligase activity;GTP binding;adenylosuccinate synthase activity |  | **-1.69** |
| 1428535_AT | RIKEN cDNA 1110056P05 gene | [1110056P05RIK](http://www.gene.ucl.ac.uk/cgi-bin/nomenclature/searchgenes.pl?field=symbol&anchor=equals&symbol_search=Search&number=100&format=html&sortby=symbol&match=1110056P05RIK) | protein binding;magnesium ion binding;lyase activity;phosphopyruvate hydratase activity |  | **1.53** |
| 1428536_AT | potassium voltage-gated channel, subfamily G, member 4 | [KCNG4](http://www.gene.ucl.ac.uk/cgi-bin/nomenclature/searchgenes.pl?field=symbol&anchor=equals&symbol_search=Search&number=100&format=html&sortby=symbol&match=KCNG4) | protein binding;magnesium ion binding;lyase activity;phosphopyruvate hydratase activity | [607603](http://www.ncbi.nlm.nih.gov/entrez/dispomim.cgi?id=607603) | **2.52** |
| 1428538_S_AT | retinoic acid receptor responder (tazarotene induced) 2 | [RARRES2](http://www.gene.ucl.ac.uk/cgi-bin/nomenclature/searchgenes.pl?field=symbol&anchor=equals&symbol_search=Search&number=100&format=html&sortby=symbol&match=RARRES2) | protein binding;magnesium ion binding;lyase activity;phosphopyruvate hydratase activity | [601973](http://www.ncbi.nlm.nih.gov/entrez/dispomim.cgi?id=601973) | **1.57** |
| 1428540_AT | RIKEN cDNA 3321401G04 gene | [3321401G04RIK](http://www.gene.ucl.ac.uk/cgi-bin/nomenclature/searchgenes.pl?field=symbol&anchor=equals&symbol_search=Search&number=100&format=html&sortby=symbol&match=3321401G04RIK) | protein binding;magnesium ion binding;receptor activity |  | **-1.79** |
| 1428541_AT | RIKEN cDNA 3321401G04 gene | [3321401G04RIK](http://www.gene.ucl.ac.uk/cgi-bin/nomenclature/searchgenes.pl?field=symbol&anchor=equals&symbol_search=Search&number=100&format=html&sortby=symbol&match=3321401G04RIK) | protein binding;magnesium ion binding;receptor activity |  | **-2.85** |
| 1428556_AT | RIKEN cDNA 2610022G08 gene | [2610022G08RIK](http://www.gene.ucl.ac.uk/cgi-bin/nomenclature/searchgenes.pl?field=symbol&anchor=equals&symbol_search=Search&number=100&format=html&sortby=symbol&match=2610022G08RIK) | protein binding;MHC class II receptor activity;peptide antigen binding |  | **1.3** |
| 1428557_A_AT | O-sialoglycoprotein endopeptidase-like 1 | [OSGEPL1](http://www.gene.ucl.ac.uk/cgi-bin/nomenclature/searchgenes.pl?field=symbol&anchor=equals&symbol_search=Search&number=100&format=html&sortby=symbol&match=OSGEPL1) | protein binding;MHC class II receptor activity;peptide antigen binding |  | **1.26** |
| 1428558_AT | O-sialoglycoprotein endopeptidase-like 1 | [OSGEPL1](http://www.gene.ucl.ac.uk/cgi-bin/nomenclature/searchgenes.pl?field=symbol&anchor=equals&symbol_search=Search&number=100&format=html&sortby=symbol&match=OSGEPL1) | protein binding;MHC class II receptor activity;peptide antigen binding |  | **1.48** |
| 1428572_AT | brain abundant, membrane attached signal protein 1 | [BASP1](http://www.gene.ucl.ac.uk/cgi-bin/nomenclature/searchgenes.pl?field=symbol&anchor=equals&symbol_search=Search&number=100&format=html&sortby=symbol&match=BASP1) | protein binding;MHC class II receptor activity;receptor activity;peptide antigen binding | [605940](http://www.ncbi.nlm.nih.gov/entrez/dispomim.cgi?id=605940) | **-1.63** |
| 1428583_AT | RIKEN cDNA 9530056D24 gene | [9530056D24RIK](http://www.gene.ucl.ac.uk/cgi-bin/nomenclature/searchgenes.pl?field=symbol&anchor=equals&symbol_search=Search&number=100&format=html&sortby=symbol&match=9530056D24RIK) | protein binding;microtubule binding |  | **-1.34** |
| 1428585_AT | actinin, alpha 1 | [ACTN1](http://www.gene.ucl.ac.uk/cgi-bin/nomenclature/searchgenes.pl?field=symbol&anchor=equals&symbol_search=Search&number=100&format=html&sortby=symbol&match=ACTN1) | protein binding;microtubule motor activity;motor activity;ATP binding |  | **-1.83** |
| 1428600_AT | RIKEN cDNA 3110068G20 gene | [3110068G20RIK](http://www.gene.ucl.ac.uk/cgi-bin/nomenclature/searchgenes.pl?field=symbol&anchor=equals&symbol_search=Search&number=100&format=html&sortby=symbol&match=3110068G20RIK) | protein binding;microtubule motor activity;motor activity;enzyme inhibitor activity |  | **-1.65** |
| 1428604_AT | RIKEN cDNA 2610305D13 gene | [2610305D13RIK](http://www.gene.ucl.ac.uk/cgi-bin/nomenclature/searchgenes.pl?field=symbol&anchor=equals&symbol_search=Search&number=100&format=html&sortby=symbol&match=2610305D13RIK) | protein binding;molecular_function unknown |  | **1.76** |
| 1428613_AT | lactate dehydrogenase D | [LDHD](http://www.gene.ucl.ac.uk/cgi-bin/nomenclature/searchgenes.pl?field=symbol&anchor=equals&symbol_search=Search&number=100&format=html&sortby=symbol&match=LDHD) | protein binding;molecular_function unknown | [607490](http://www.ncbi.nlm.nih.gov/entrez/dispomim.cgi?id=607490) | **1.6** |
| 1428614_AT | gb:AW105779 /DB_XREF=gi:6076515 /DB_XREF=um32f07.x |  | protein binding;molecular_function unknown |  | **1.64** |
| 1428631_A_AT | ubiquinol cytochrome c reductase core protein 2 | [UQCRC2](http://www.gene.ucl.ac.uk/cgi-bin/nomenclature/searchgenes.pl?field=symbol&anchor=equals&symbol_search=Search&number=100&format=html&sortby=symbol&match=UQCRC2) | protein binding;motor activity | [191329](http://www.ncbi.nlm.nih.gov/entrez/dispomim.cgi?id=191329) | **1.27** |
| 1428645_AT | guanine nucleotide binding protein, alpha inhibiting 3 | [GNAI3](http://www.gene.ucl.ac.uk/cgi-bin/nomenclature/searchgenes.pl?field=symbol&anchor=equals&symbol_search=Search&number=100&format=html&sortby=symbol&match=GNAI3) | protein binding;motor activity | [139370](http://www.ncbi.nlm.nih.gov/entrez/dispomim.cgi?id=139370) | **-1.33** |
| 1428648_AT | DNA segment, Chr 10, ERATO Doi 516, expressed | [D10ERTD516E](http://www.gene.ucl.ac.uk/cgi-bin/nomenclature/searchgenes.pl?field=symbol&anchor=equals&symbol_search=Search&number=100&format=html&sortby=symbol&match=D10ERTD516E) | protein binding;motor activity;microtubule motor activity;enzyme inhibitor activity |  | **-1.34** |
| 1428650_AT | tensin | [TNS](http://www.gene.ucl.ac.uk/cgi-bin/nomenclature/searchgenes.pl?field=symbol&anchor=equals&symbol_search=Search&number=100&format=html&sortby=symbol&match=TNS) | protein binding;motor activity;microtubule motor activity;enzyme inhibitor activity | [600076](http://www.ncbi.nlm.nih.gov/entrez/dispomim.cgi?id=600076) | **1.37** |
| 1428656_AT | ethanol induced 2 | [ETOHI2](http://www.gene.ucl.ac.uk/cgi-bin/nomenclature/searchgenes.pl?field=symbol&anchor=equals&symbol_search=Search&number=100&format=html&sortby=symbol&match=ETOHI2) | protein binding;mRNA (guanine-N7-)-methyltransferase activity;methyltransferase activity;transferase activity |  | **1.24** |
| 1428659_AT | PHD finger protein 7 | [PHF7](http://www.gene.ucl.ac.uk/cgi-bin/nomenclature/searchgenes.pl?field=symbol&anchor=equals&symbol_search=Search&number=100&format=html&sortby=symbol&match=PHF7) | protein binding;neurexin binding |  | **1.54** |
| 1428670_AT | RIKEN cDNA 2610305J24 gene | [2610305J24RIK](http://www.gene.ucl.ac.uk/cgi-bin/nomenclature/searchgenes.pl?field=symbol&anchor=equals&symbol_search=Search&number=100&format=html&sortby=symbol&match=2610305J24RIK) | protein binding;neurotransmitter receptor activity;ion channel activity;GABA-A receptor activity;extracellular ligand-gated ion channel activity;nicotinic acetylcholine-activated cation-selective channel activity |  | **-2.65** |
| 1428674_AT | RIKEN cDNA 1110021E09 gene | [1110021E09RIK](http://www.gene.ucl.ac.uk/cgi-bin/nomenclature/searchgenes.pl?field=symbol&anchor=equals&symbol_search=Search&number=100&format=html&sortby=symbol&match=1110021E09RIK) | protein binding;nucleic acid binding;microtubule binding |  | **-1.36** |
| 1428681_AT | RIKEN cDNA 5530400K22 gene | [5530400K22RIK](http://www.gene.ucl.ac.uk/cgi-bin/nomenclature/searchgenes.pl?field=symbol&anchor=equals&symbol_search=Search&number=100&format=html&sortby=symbol&match=5530400K22RIK) | protein binding;nucleic acid binding;pre-mRNA splicing factor activity;RNA binding |  | **-1.59** |
| 1428693_AT | RIKEN cDNA 2610044O15 gene | [2610044O15RIK](http://www.gene.ucl.ac.uk/cgi-bin/nomenclature/searchgenes.pl?field=symbol&anchor=equals&symbol_search=Search&number=100&format=html&sortby=symbol&match=2610044O15RIK) | protein binding;oxidoreductase activity |  | **1.63** |
| 1428695_AT | RIKEN cDNA 9130227C08 gene | [9130227C08RIK](http://www.gene.ucl.ac.uk/cgi-bin/nomenclature/searchgenes.pl?field=symbol&anchor=equals&symbol_search=Search&number=100&format=html&sortby=symbol&match=9130227C08RIK) | protein binding;oxidoreductase activity;heparin binding |  | **1.6** |
| 1428697_AT | dipeptidylpeptidase 8 | [DPP8](http://www.gene.ucl.ac.uk/cgi-bin/nomenclature/searchgenes.pl?field=symbol&anchor=equals&symbol_search=Search&number=100&format=html&sortby=symbol&match=DPP8) | protein binding;PDZ domain binding;zinc ion binding;protein homooligomerization activity;ubiquitin-protein ligase activity | [606819](http://www.ncbi.nlm.nih.gov/entrez/dispomim.cgi?id=606819) | **1.27** |
| 1428706_AT | RIKEN cDNA 3110013H01 gene | [PRR6](http://www.gene.ucl.ac.uk/cgi-bin/nomenclature/searchgenes.pl?field=symbol&anchor=equals&symbol_search=Search&number=100&format=html&sortby=symbol&match=PRR6) | protein binding;phosphoprotein phosphatase activity;protein serine/threonine phosphatase activity;manganese ion binding;hydrolase activity | [608139](http://www.ncbi.nlm.nih.gov/entrez/dispomim.cgi?id=608139) | **1.43** |
| 1428722_AT | creatine kinase, mitochondrial 2 | [CKMT2](http://www.gene.ucl.ac.uk/cgi-bin/nomenclature/searchgenes.pl?field=symbol&anchor=equals&symbol_search=Search&number=100&format=html&sortby=symbol&match=CKMT2) | protein binding;phosphoprotein phosphatase activity;protein serine/threonine phosphatase activity;protein phosphatase type 2C activity;magnesium ion binding;manganese ion binding;catalytic activity;hydrolase activity | [123295](http://www.ncbi.nlm.nih.gov/entrez/dispomim.cgi?id=123295) | **1.37** |
| 1428739_AT | RIKEN cDNA 2310040A07 gene | [2310040A07RIK](http://www.gene.ucl.ac.uk/cgi-bin/nomenclature/searchgenes.pl?field=symbol&anchor=equals&symbol_search=Search&number=100&format=html&sortby=symbol&match=2310040A07RIK) | protein binding;phosphoprotein phosphatase activity;receptor activity;protein tyrosine phosphatase activity;hydrolase activity |  | **-1.77** |
| 1428745_A_AT | RIKEN cDNA 2310003L22 gene | [2310003L22RIK](http://www.gene.ucl.ac.uk/cgi-bin/nomenclature/searchgenes.pl?field=symbol&anchor=equals&symbol_search=Search&number=100&format=html&sortby=symbol&match=2310003L22RIK) | protein binding;phosphoprotein phosphatase activity;receptor activity;protein tyrosine phosphatase activity;hydrolase activity |  | **1.46** |
| 1428749_AT | RIKEN cDNA 6430411K14 gene | [6430411K14RIK](http://www.gene.ucl.ac.uk/cgi-bin/nomenclature/searchgenes.pl?field=symbol&anchor=equals&symbol_search=Search&number=100&format=html&sortby=symbol&match=6430411K14RIK) | protein binding;profilin binding;SH3 domain binding |  | **-1.34** |
| 1428754_AT | RIKEN cDNA 3300001M20 gene | [3300001M20RIK](http://www.gene.ucl.ac.uk/cgi-bin/nomenclature/searchgenes.pl?field=symbol&anchor=equals&symbol_search=Search&number=100&format=html&sortby=symbol&match=3300001M20RIK) | protein binding;profilin binding;SH3 domain binding |  | **-1.41** |
| 1428760_AT | small nuclear RNA activating complex, polypeptide 3 | [SNAPC3](http://www.gene.ucl.ac.uk/cgi-bin/nomenclature/searchgenes.pl?field=symbol&anchor=equals&symbol_search=Search&number=100&format=html&sortby=symbol&match=SNAPC3) | protein binding;profilin binding;SH3 domain binding | [602348](http://www.ncbi.nlm.nih.gov/entrez/dispomim.cgi?id=602348) | **-1.35** |
| 1428766_AT | RIKEN cDNA 4833420N02 gene | [RNMTL1](http://www.gene.ucl.ac.uk/cgi-bin/nomenclature/searchgenes.pl?field=symbol&anchor=equals&symbol_search=Search&number=100&format=html&sortby=symbol&match=RNMTL1) | protein binding;profilin binding;SH3 domain binding |  | **1.22** |
| 1428780_AT | RIKEN cDNA 1300017K07 gene | [1300017K07RIK](http://www.gene.ucl.ac.uk/cgi-bin/nomenclature/searchgenes.pl?field=symbol&anchor=equals&symbol_search=Search&number=100&format=html&sortby=symbol&match=1300017K07RIK) | protein binding;prostaglandin E receptor activity;receptor activity;prostaglandin receptor activity;G-protein coupled receptor activity;rhodopsin-like receptor activity |  | **1.54** |
| 1428782_A_AT | ubiquinol-cytochrome c reductase core protein 1 | [UQCRC1](http://www.gene.ucl.ac.uk/cgi-bin/nomenclature/searchgenes.pl?field=symbol&anchor=equals&symbol_search=Search&number=100&format=html&sortby=symbol&match=UQCRC1) | protein binding;protein heterodimerization activity;DNA binding;calcium ion binding;transcription factor activity;receptor activity;transcription regulator activity | [191328](http://www.ncbi.nlm.nih.gov/entrez/dispomim.cgi?id=191328) | **1.38** |
| 1428783_AT | protein kinase, cAMP dependent regulatory, type II alpha | [PRKAR2A](http://www.gene.ucl.ac.uk/cgi-bin/nomenclature/searchgenes.pl?field=symbol&anchor=equals&symbol_search=Search&number=100&format=html&sortby=symbol&match=PRKAR2A) | protein binding;protein heterodimerization activity;protein C-terminus binding;ubiquitin activating enzyme activity;ligase activity;catalytic activity | [176910](http://www.ncbi.nlm.nih.gov/entrez/dispomim.cgi?id=176910) | **1.36** |
| 1428786_AT | hematopoietic protein 1 | [HEMP1](http://www.gene.ucl.ac.uk/cgi-bin/nomenclature/searchgenes.pl?field=symbol&anchor=equals&symbol_search=Search&number=100&format=html&sortby=symbol&match=HEMP1) | protein binding;protein homodimerization activity;calcium ion binding |  | **-2.63** |
| 1428821_AT | gb:AK010891.1 /DB_XREF=gi:12846646 /FEA=mRNA /CNT= |  | protein binding;protein homodimerization activity;calcium ion binding |  | **1.95** |
| 1428831_AT | RIKEN cDNA 6230429P13 gene | [6230429P13RIK](http://www.gene.ucl.ac.uk/cgi-bin/nomenclature/searchgenes.pl?field=symbol&anchor=equals&symbol_search=Search&number=100&format=html&sortby=symbol&match=6230429P13RIK) | protein binding;protein homooligomerization activity |  | **-1.23** |
| 1428835_AT | myosin, heavy polypeptide 14 | [MYH14](http://www.gene.ucl.ac.uk/cgi-bin/nomenclature/searchgenes.pl?field=symbol&anchor=equals&symbol_search=Search&number=100&format=html&sortby=symbol&match=MYH14) | protein binding;protein kinase activity;calmodulin-dependent protein kinase I activity;protein serine/threonine kinase activity;calcium/calmodulin-dependent protein kinase activity;ATP binding;calmodulin binding;transferase activity | [608568](http://www.ncbi.nlm.nih.gov/entrez/dispomim.cgi?id=608568) | **1.57** |
| 1428847_A_AT | microtubule-actin crosslinking factor 1 | [MACF1](http://www.gene.ucl.ac.uk/cgi-bin/nomenclature/searchgenes.pl?field=symbol&anchor=equals&symbol_search=Search&number=100&format=html&sortby=symbol&match=MACF1) | protein binding;protein kinase activity;kinase activity;cyclin-dependent protein kinase regulator activity |  | **-1.69** |
| 1428848_A_AT | microtubule-actin crosslinking factor 1 | [MACF1](http://www.gene.ucl.ac.uk/cgi-bin/nomenclature/searchgenes.pl?field=symbol&anchor=equals&symbol_search=Search&number=100&format=html&sortby=symbol&match=MACF1) | protein binding;protein kinase activity;kinase activity;cyclin-dependent protein kinase regulator activity |  | **-1.64** |
| 1428850_X_AT | RIKEN cDNA 2410026K10 gene | [2410026K10RIK](http://www.gene.ucl.ac.uk/cgi-bin/nomenclature/searchgenes.pl?field=symbol&anchor=equals&symbol_search=Search&number=100&format=html&sortby=symbol&match=2410026K10RIK) | protein binding;protein kinase activity;kinase activity;protein serine/threonine kinase activity;ATP binding;cyclin-dependent protein kinase activity;transferase activity |  | **-1.33** |
| 1428853_AT | patched homolog 1 | [PTCH1](http://www.gene.ucl.ac.uk/cgi-bin/nomenclature/searchgenes.pl?field=symbol&anchor=equals&symbol_search=Search&number=100&format=html&sortby=symbol&match=PTCH1) | protein binding;protein kinase activity;kinase activity;protein serine/threonine kinase activity;ATP binding;protein-tyrosine kinase activity;receptor signaling protein serine/threonine kinase activity;transferase activity |  | **1.39** |
| 1428854_AT | RIKEN cDNA 6430595O10 gene | [TMED8](http://www.gene.ucl.ac.uk/cgi-bin/nomenclature/searchgenes.pl?field=symbol&anchor=equals&symbol_search=Search&number=100&format=html&sortby=symbol&match=TMED8) | protein binding;protein kinase activity;kinase activity;protein serine/threonine kinase activity;protein-tyrosine kinase activity;ATP binding;transferase activity |  | **-1.36** |
| 1428878_A_AT | phosphatidylinositol transfer protein, cytoplasmic 1 | [PITPNC1](http://www.gene.ucl.ac.uk/cgi-bin/nomenclature/searchgenes.pl?field=symbol&anchor=equals&symbol_search=Search&number=100&format=html&sortby=symbol&match=PITPNC1) | protein binding;protein kinase binding;insulin receptor binding | [605134](http://www.ncbi.nlm.nih.gov/entrez/dispomim.cgi?id=605134) | **1.45** |
| 1428880_AT | RIKEN cDNA 0610038F07 gene | [0610038F07RIK](http://www.gene.ucl.ac.uk/cgi-bin/nomenclature/searchgenes.pl?field=symbol&anchor=equals&symbol_search=Search&number=100&format=html&sortby=symbol&match=0610038F07RIK) | protein binding;protein phosphatase type 2C activity;GTP binding;catalytic activity |  | **1.21** |
| 1428896_AT | platelet-derived growth factor receptor-like | [PDGFRL](http://www.gene.ucl.ac.uk/cgi-bin/nomenclature/searchgenes.pl?field=symbol&anchor=equals&symbol_search=Search&number=100&format=html&sortby=symbol&match=PDGFRL) | protein binding;protein phosphatase type 2C activity;GTP binding;catalytic activity | [604584](http://www.ncbi.nlm.nih.gov/entrez/dispomim.cgi?id=604584) | **-3.12** |
| 1428908_AT | RIKEN cDNA 2600011C06 gene | [2600011C06RIK](http://www.gene.ucl.ac.uk/cgi-bin/nomenclature/searchgenes.pl?field=symbol&anchor=equals&symbol_search=Search&number=100&format=html&sortby=symbol&match=2600011C06RIK) | protein binding;protein self binding |  | **-1.29** |
| 1428909_AT | gb:BI683916 /DB_XREF=gi:15646544 /DB_XREF=60330673 |  | protein binding;protein transporter activity |  | **-1.89** |
| 1428910_AT | RIKEN cDNA 2310022B05 gene | [2310022B05RIK](http://www.gene.ucl.ac.uk/cgi-bin/nomenclature/searchgenes.pl?field=symbol&anchor=equals&symbol_search=Search&number=100&format=html&sortby=symbol&match=2310022B05RIK) | protein binding;protein transporter activity |  | **-1.52** |
| 1428911_AT | tubulin tyrosine ligase-like family, member 4 | [TTLL4](http://www.gene.ucl.ac.uk/cgi-bin/nomenclature/searchgenes.pl?field=symbol&anchor=equals&symbol_search=Search&number=100&format=html&sortby=symbol&match=TTLL4) | protein binding;protein transporter activity |  | **-1.33** |
| 1428913_AT | DNA segment, Chr 2, Brigham & Women's Genetics 1335 expressed | [D2BWG1335E](http://www.gene.ucl.ac.uk/cgi-bin/nomenclature/searchgenes.pl?field=symbol&anchor=equals&symbol_search=Search&number=100&format=html&sortby=symbol&match=D2BWG1335E) | protein binding;Rab guanyl-nucleotide exchange factor activity;Rab interactor activity |  | **1.33** |
| 1428914_AT | RIKEN cDNA 2310014D11 gene | [2310014D11RIK](http://www.gene.ucl.ac.uk/cgi-bin/nomenclature/searchgenes.pl?field=symbol&anchor=equals&symbol_search=Search&number=100&format=html&sortby=symbol&match=2310014D11RIK) | protein binding;Ran GTPase activator activity;GTPase activator activity |  | **-1.93** |
| 1428915_AT | sirtuin 5 (silent mating type information regulation 2 homolog) 5 (S. cerevisiae) | [SIRT5](http://www.gene.ucl.ac.uk/cgi-bin/nomenclature/searchgenes.pl?field=symbol&anchor=equals&symbol_search=Search&number=100&format=html&sortby=symbol&match=SIRT5) | protein binding;receptor activity | [604483](http://www.ncbi.nlm.nih.gov/entrez/dispomim.cgi?id=604483) | **1.35** |
| 1428926_AT | RIKEN cDNA 1110003O08 gene | [1110003O08RIK](http://www.gene.ucl.ac.uk/cgi-bin/nomenclature/searchgenes.pl?field=symbol&anchor=equals&symbol_search=Search&number=100&format=html&sortby=symbol&match=1110003O08RIK) | protein binding;receptor activity |  | **1.69** |
| 1428929_S_AT | solute carrier family 25 (mitochondrial carrier, phosphate carrier), member 26 | [SLC25A26](http://www.gene.ucl.ac.uk/cgi-bin/nomenclature/searchgenes.pl?field=symbol&anchor=equals&symbol_search=Search&number=100&format=html&sortby=symbol&match=SLC25A26) | protein binding;receptor activity |  | **1.61** |
| 1428942_AT | metallothionein 2 | [MT2](http://www.gene.ucl.ac.uk/cgi-bin/nomenclature/searchgenes.pl?field=symbol&anchor=equals&symbol_search=Search&number=100&format=html&sortby=symbol&match=MT2) | protein binding;receptor activity |  | **-4.89** |
| 1428954_AT | solute carrier family 9 (sodium/hydrogen exchanger), isoform 3 regulator 2 | [SLC9A3R2](http://www.gene.ucl.ac.uk/cgi-bin/nomenclature/searchgenes.pl?field=symbol&anchor=equals&symbol_search=Search&number=100&format=html&sortby=symbol&match=SLC9A3R2) | protein binding;receptor activity | [606553](http://www.ncbi.nlm.nih.gov/entrez/dispomim.cgi?id=606553) | **1.99** |
| 1428955_X_AT | solute carrier family 9 (sodium/hydrogen exchanger), isoform 3 regulator 2 | [SLC9A3R2](http://www.gene.ucl.ac.uk/cgi-bin/nomenclature/searchgenes.pl?field=symbol&anchor=equals&symbol_search=Search&number=100&format=html&sortby=symbol&match=SLC9A3R2) | protein binding;receptor activity | [606553](http://www.ncbi.nlm.nih.gov/entrez/dispomim.cgi?id=606553) | **1.93** |
| 1428982_AT | RIKEN cDNA D530031C13 gene | [D530031C13RIK](http://www.gene.ucl.ac.uk/cgi-bin/nomenclature/searchgenes.pl?field=symbol&anchor=equals&symbol_search=Search&number=100&format=html&sortby=symbol&match=D530031C13RIK) | protein binding;receptor activity |  | **-1.37** |
| 1428991_AT | HRAS-like suppressor | [HRASLS](http://www.gene.ucl.ac.uk/cgi-bin/nomenclature/searchgenes.pl?field=symbol&anchor=equals&symbol_search=Search&number=100&format=html&sortby=symbol&match=HRASLS) | protein binding;receptor activity | [606487](http://www.ncbi.nlm.nih.gov/entrez/dispomim.cgi?id=606487) | **2.21** |
| 1428996_AT | RIKEN cDNA 4833426J09 gene | [4833426J09RIK](http://www.gene.ucl.ac.uk/cgi-bin/nomenclature/searchgenes.pl?field=symbol&anchor=equals&symbol_search=Search&number=100&format=html&sortby=symbol&match=4833426J09RIK) | protein binding;receptor activity |  | **1.58** |
| 1429004_AT | pleckstrin homology domain interacting protein | [PHIP](http://www.gene.ucl.ac.uk/cgi-bin/nomenclature/searchgenes.pl?field=symbol&anchor=equals&symbol_search=Search&number=100&format=html&sortby=symbol&match=PHIP) | protein binding;receptor activity |  | **-1.34** |
| 1429021_AT | Eph receptor A4 | [EPHA4](http://www.gene.ucl.ac.uk/cgi-bin/nomenclature/searchgenes.pl?field=symbol&anchor=equals&symbol_search=Search&number=100&format=html&sortby=symbol&match=EPHA4) | protein binding;receptor activity | [602188](http://www.ncbi.nlm.nih.gov/entrez/dispomim.cgi?id=602188) | **1.74** |
| 1429028_AT | Dedicator of cytokinesis 11 | [DOCK11](http://www.gene.ucl.ac.uk/cgi-bin/nomenclature/searchgenes.pl?field=symbol&anchor=equals&symbol_search=Search&number=100&format=html&sortby=symbol&match=DOCK11) | protein binding;receptor activity |  | **-1.48** |
| 1429047_AT | gene trap locus 7 | [GTL7](http://www.gene.ucl.ac.uk/cgi-bin/nomenclature/searchgenes.pl?field=symbol&anchor=equals&symbol_search=Search&number=100&format=html&sortby=symbol&match=GTL7) | protein binding;receptor activity |  | **1.47** |
| 1429051_S_AT | RIKEN cDNA 6230403H02 gene | [6230403H02RIK](http://www.gene.ucl.ac.uk/cgi-bin/nomenclature/searchgenes.pl?field=symbol&anchor=equals&symbol_search=Search&number=100&format=html&sortby=symbol&match=6230403H02RIK) | protein binding;receptor activity |  | **-2.44** |
| 1429052_AT | protein tyrosine phosphatase, receptor type, D | [PTPRD](http://www.gene.ucl.ac.uk/cgi-bin/nomenclature/searchgenes.pl?field=symbol&anchor=equals&symbol_search=Search&number=100&format=html&sortby=symbol&match=PTPRD) | protein binding;receptor activity | [601598](http://www.ncbi.nlm.nih.gov/entrez/dispomim.cgi?id=601598) | **-1.61** |
| 1429062_AT | kinesin family member 16B | [KIF16B](http://www.gene.ucl.ac.uk/cgi-bin/nomenclature/searchgenes.pl?field=symbol&anchor=equals&symbol_search=Search&number=100&format=html&sortby=symbol&match=KIF16B) | protein binding;receptor activity |  | **1.58** |
| 1429063_S_AT | kinesin family member 16B | [KIF16B](http://www.gene.ucl.ac.uk/cgi-bin/nomenclature/searchgenes.pl?field=symbol&anchor=equals&symbol_search=Search&number=100&format=html&sortby=symbol&match=KIF16B) | protein binding;receptor activity |  | **1.45** |
| 1429065_AT | RIKEN cDNA 1700023M03 gene | [1700023M03RIK](http://www.gene.ucl.ac.uk/cgi-bin/nomenclature/searchgenes.pl?field=symbol&anchor=equals&symbol_search=Search&number=100&format=html&sortby=symbol&match=1700023M03RIK) | protein binding;receptor activity |  | **-1.83** |
| 1429071_AT | RIKEN cDNA 1700020C08 gene | [ME3](http://www.gene.ucl.ac.uk/cgi-bin/nomenclature/searchgenes.pl?field=symbol&anchor=equals&symbol_search=Search&number=100&format=html&sortby=symbol&match=ME3) | protein binding;receptor activity | [604626](http://www.ncbi.nlm.nih.gov/entrez/dispomim.cgi?id=604626) | **1.64** |
| 1429073_AT | RIKEN cDNA 2010005J08 gene | [2210015D19RIK](http://www.gene.ucl.ac.uk/cgi-bin/nomenclature/searchgenes.pl?field=symbol&anchor=equals&symbol_search=Search&number=100&format=html&sortby=symbol&match=2210015D19RIK) | protein binding;receptor activity |  | **1.59** |
| 1429077_X_AT | ribosomal protein L21 | [RPL21](http://www.gene.ucl.ac.uk/cgi-bin/nomenclature/searchgenes.pl?field=symbol&anchor=equals&symbol_search=Search&number=100&format=html&sortby=symbol&match=RPL21) | protein binding;receptor activity | [603636](http://www.ncbi.nlm.nih.gov/entrez/dispomim.cgi?id=603636) | **-1.2** |
| 1429081_AT | RIKEN cDNA 2600014C01 gene | [2600014C01RIK](http://www.gene.ucl.ac.uk/cgi-bin/nomenclature/searchgenes.pl?field=symbol&anchor=equals&symbol_search=Search&number=100&format=html&sortby=symbol&match=2600014C01RIK) | protein binding;receptor activity |  | **1.27** |
| 1429088_AT | limb-bud and heart | [LBH](http://www.gene.ucl.ac.uk/cgi-bin/nomenclature/searchgenes.pl?field=symbol&anchor=equals&symbol_search=Search&number=100&format=html&sortby=symbol&match=LBH) | protein binding;receptor activity |  | **-1.99** |
| 1429110_A_AT | RIKEN cDNA 2810405F18 gene | [2810405F18RIK](http://www.gene.ucl.ac.uk/cgi-bin/nomenclature/searchgenes.pl?field=symbol&anchor=equals&symbol_search=Search&number=100&format=html&sortby=symbol&match=2810405F18RIK) | protein binding;receptor activity;hedgehog receptor activity |  | **1.41** |
| 1429114_AT | SEC14 and spectrin domains 1 | [SESTD1](http://www.gene.ucl.ac.uk/cgi-bin/nomenclature/searchgenes.pl?field=symbol&anchor=equals&symbol_search=Search&number=100&format=html&sortby=symbol&match=SESTD1) | protein binding;receptor activity;hyaluronic acid binding |  | **-1.44** |
| 1429116_AT | solute carrier family 17 (anion/sugar transporter), member 5 | [SLC17A5](http://www.gene.ucl.ac.uk/cgi-bin/nomenclature/searchgenes.pl?field=symbol&anchor=equals&symbol_search=Search&number=100&format=html&sortby=symbol&match=SLC17A5) | protein binding;receptor activity;MHC class II receptor activity;peptide antigen binding | [604322](http://www.ncbi.nlm.nih.gov/entrez/dispomim.cgi?id=604322) | **-1.4** |
| 1429129_AT | RIKEN cDNA 1200008A14 gene | [1200008A14RIK](http://www.gene.ucl.ac.uk/cgi-bin/nomenclature/searchgenes.pl?field=symbol&anchor=equals&symbol_search=Search&number=100&format=html&sortby=symbol&match=1200008A14RIK) | protein binding;receptor activity;opsonin binding |  | **-2.41** |
| 1429141_AT | neuralized-like 2 (Drosophila) | [NEURL2](http://www.gene.ucl.ac.uk/cgi-bin/nomenclature/searchgenes.pl?field=symbol&anchor=equals&symbol_search=Search&number=100&format=html&sortby=symbol&match=NEURL2) | protein binding;receptor activity;oxidoreductase activity |  | **1.32** |
| 1429144_AT | RIKEN cDNA 2310032D16 gene | [2310032D16RIK](http://www.gene.ucl.ac.uk/cgi-bin/nomenclature/searchgenes.pl?field=symbol&anchor=equals&symbol_search=Search&number=100&format=html&sortby=symbol&match=2310032D16RIK) | protein binding;receptor activity;oxidoreductase activity |  | **1.8** |
| 1429146_AT | RIKEN cDNA 6620401M08 gene | [6620401M08RIK](http://www.gene.ucl.ac.uk/cgi-bin/nomenclature/searchgenes.pl?field=symbol&anchor=equals&symbol_search=Search&number=100&format=html&sortby=symbol&match=6620401M08RIK) | protein binding;receptor activity;oxidoreductase activity |  | **1.58** |
| 1429159_AT | RIKEN cDNA 4631408O11 gene | [4631408O11RIK](http://www.gene.ucl.ac.uk/cgi-bin/nomenclature/searchgenes.pl?field=symbol&anchor=equals&symbol_search=Search&number=100&format=html&sortby=symbol&match=4631408O11RIK) | protein binding;receptor binding;signal transducer activity |  | **-1.46** |
| 1429177_X_AT | SRY-box containing gene 17 | [SOX17](http://www.gene.ucl.ac.uk/cgi-bin/nomenclature/searchgenes.pl?field=symbol&anchor=equals&symbol_search=Search&number=100&format=html&sortby=symbol&match=SOX17) | protein binding;receptor signaling complex scaffold activity |  | **1.98** |
| 1429183_AT | plakophilin 2 | [PKP2](http://www.gene.ucl.ac.uk/cgi-bin/nomenclature/searchgenes.pl?field=symbol&anchor=equals&symbol_search=Search&number=100&format=html&sortby=symbol&match=PKP2) | protein binding;Rho GTPase activator activity;GTPase activator activity | [602861](http://www.ncbi.nlm.nih.gov/entrez/dispomim.cgi?id=602861) | **-1.73** |
| 1429192_AT | Sloan-Kettering viral oncogene homolog | [SKI](http://www.gene.ucl.ac.uk/cgi-bin/nomenclature/searchgenes.pl?field=symbol&anchor=equals&symbol_search=Search&number=100&format=html&sortby=symbol&match=SKI) | protein binding;ribonucleoside-diphosphate reductase activity;oxidoreductase activity | [164780](http://www.ncbi.nlm.nih.gov/entrez/dispomim.cgi?id=164780) | **1.22** |
| 1429195_AT | RIKEN cDNA 2310076O14 gene | [2310076O14RIK](http://www.gene.ucl.ac.uk/cgi-bin/nomenclature/searchgenes.pl?field=symbol&anchor=equals&symbol_search=Search&number=100&format=html&sortby=symbol&match=2310076O14RIK) | protein binding;ribonucleoside-diphosphate reductase activity;oxidoreductase activity |  | **1.38** |
| 1429205_AT | RIKEN cDNA 2610012I03 gene | [2610012I03RIK](http://www.gene.ucl.ac.uk/cgi-bin/nomenclature/searchgenes.pl?field=symbol&anchor=equals&symbol_search=Search&number=100&format=html&sortby=symbol&match=2610012I03RIK) | protein binding;ribosome binding;ATP binding |  | **1.45** |
| 1429206_AT | Rho-related BTB domain containing 1 | [RHOBTB1](http://www.gene.ucl.ac.uk/cgi-bin/nomenclature/searchgenes.pl?field=symbol&anchor=equals&symbol_search=Search&number=100&format=html&sortby=symbol&match=RHOBTB1) | protein binding;RNA binding;ATP binding | [607351](http://www.ncbi.nlm.nih.gov/entrez/dispomim.cgi?id=607351) | **1.57** |
| 1429213_AT | RIKEN cDNA 2310030N02 gene | [2310030N02RIK](http://www.gene.ucl.ac.uk/cgi-bin/nomenclature/searchgenes.pl?field=symbol&anchor=equals&symbol_search=Search&number=100&format=html&sortby=symbol&match=2310030N02RIK) | protein binding;RNA binding;structural constituent of ribosome |  | **1.67** |
| 1429219_AT | RIKEN cDNA 1200009F10 gene | [1200009F10RIK](http://www.gene.ucl.ac.uk/cgi-bin/nomenclature/searchgenes.pl?field=symbol&anchor=equals&symbol_search=Search&number=100&format=html&sortby=symbol&match=1200009F10RIK) | protein binding;scavenger receptor activity;isomerase activity |  | **-1.55** |
| 1429223_A_AT | RGM domain family, member C | [HFE2](http://www.gene.ucl.ac.uk/cgi-bin/nomenclature/searchgenes.pl?field=symbol&anchor=equals&symbol_search=Search&number=100&format=html&sortby=symbol&match=HFE2) | protein binding;semaphorin receptor activity;receptor activity | [608374](http://www.ncbi.nlm.nih.gov/entrez/dispomim.cgi?id=608374) | **1.26** |
| 1429227_X_AT | nucleosome assembly protein 1-like 1 | [NAP1L1](http://www.gene.ucl.ac.uk/cgi-bin/nomenclature/searchgenes.pl?field=symbol&anchor=equals&symbol_search=Search&number=100&format=html&sortby=symbol&match=NAP1L1) | protein binding;semaphorin receptor activity;receptor activity | [164060](http://www.ncbi.nlm.nih.gov/entrez/dispomim.cgi?id=164060) | **-1.47** |
| 1429237_AT | syntaxin 16 | [STX16](http://www.gene.ucl.ac.uk/cgi-bin/nomenclature/searchgenes.pl?field=symbol&anchor=equals&symbol_search=Search&number=100&format=html&sortby=symbol&match=STX16) | protein binding;semaphorin receptor activity;receptor activity | [603666](http://www.ncbi.nlm.nih.gov/entrez/dispomim.cgi?id=603666) | **-1.35** |
| 1429259_A_AT | RIKEN cDNA 1810014B01 gene | [1810014B01RIK](http://www.gene.ucl.ac.uk/cgi-bin/nomenclature/searchgenes.pl?field=symbol&anchor=equals&symbol_search=Search&number=100&format=html&sortby=symbol&match=1810014B01RIK) | protein binding;serine carboxypeptidase activity;carboxypeptidase activity;carboxypeptidase C activity;catalytic activity;hydrolase activity;peptidase activity |  | **1.56** |
| 1429260_AT | RIKEN cDNA 1810014B01 gene | [1810014B01RIK](http://www.gene.ucl.ac.uk/cgi-bin/nomenclature/searchgenes.pl?field=symbol&anchor=equals&symbol_search=Search&number=100&format=html&sortby=symbol&match=1810014B01RIK) | protein binding;serine-type endopeptidase inhibitor activity;endopeptidase inhibitor activity |  | **1.65** |
| 1429273_AT | BMP-binding endothelial regulator | [BMPER](http://www.gene.ucl.ac.uk/cgi-bin/nomenclature/searchgenes.pl?field=symbol&anchor=equals&symbol_search=Search&number=100&format=html&sortby=symbol&match=BMPER) | protein binding;serine-type endopeptidase inhibitor activity;endopeptidase inhibitor activity | [608699](http://www.ncbi.nlm.nih.gov/entrez/dispomim.cgi?id=608699) | **-2.14** |
| 1429304_AT | ankyrin repeat domain 10 | [ANKRD10](http://www.gene.ucl.ac.uk/cgi-bin/nomenclature/searchgenes.pl?field=symbol&anchor=equals&symbol_search=Search&number=100&format=html&sortby=symbol&match=ANKRD10) | protein binding;SH3/SH2 adaptor protein activity |  | **-3.11** |
| 1429306_AT | leucine zipper and CTNNBIP1 domain containing | [LZIC](http://www.gene.ucl.ac.uk/cgi-bin/nomenclature/searchgenes.pl?field=symbol&anchor=equals&symbol_search=Search&number=100&format=html&sortby=symbol&match=LZIC) | protein binding;SH3/SH2 adaptor protein activity |  | **-1.96** |
| 1429307_S_AT | leucine zipper and CTNNBIP1 domain containing | [LZIC](http://www.gene.ucl.ac.uk/cgi-bin/nomenclature/searchgenes.pl?field=symbol&anchor=equals&symbol_search=Search&number=100&format=html&sortby=symbol&match=LZIC) | protein binding;SH3/SH2 adaptor protein activity;receptor activity |  | **-1.67** |
| 1429321_AT | Ring finger protein 149 | [RNF149](http://www.gene.ucl.ac.uk/cgi-bin/nomenclature/searchgenes.pl?field=symbol&anchor=equals&symbol_search=Search&number=100&format=html&sortby=symbol&match=RNF149) | protein binding;signal transducer activity |  | **-1.6** |
| 1429329_AT | COX10 homolog, cytochrome c oxidase assembly protein, heme A: farnesyltransferase (yeast) | [COX10](http://www.gene.ucl.ac.uk/cgi-bin/nomenclature/searchgenes.pl?field=symbol&anchor=equals&symbol_search=Search&number=100&format=html&sortby=symbol&match=COX10) | protein binding;small GTPase regulatory/interacting protein activity;GTPase activator activity | [602125](http://www.ncbi.nlm.nih.gov/entrez/dispomim.cgi?id=602125) | **1.63** |
| 1429345_AT | DNA segment, Chr 2, ERATO Doi 435, expressed | [D2ERTD435E](http://www.gene.ucl.ac.uk/cgi-bin/nomenclature/searchgenes.pl?field=symbol&anchor=equals&symbol_search=Search&number=100&format=html&sortby=symbol&match=D2ERTD435E) | protein binding;stem cell factor receptor binding |  | **-1.8** |
| 1429359_S_AT | RNA binding protein gene with multiple splicing | [RBPMS](http://www.gene.ucl.ac.uk/cgi-bin/nomenclature/searchgenes.pl?field=symbol&anchor=equals&symbol_search=Search&number=100&format=html&sortby=symbol&match=RBPMS) | protein binding;stem cell factor receptor binding | [601558](http://www.ncbi.nlm.nih.gov/entrez/dispomim.cgi?id=601558) | **1.63** |
| 1429383_AT | casein kinase 1, gamma 3 | [CSNK1G3](http://www.gene.ucl.ac.uk/cgi-bin/nomenclature/searchgenes.pl?field=symbol&anchor=equals&symbol_search=Search&number=100&format=html&sortby=symbol&match=CSNK1G3) | protein binding;stem cell factor receptor binding | [604253](http://www.ncbi.nlm.nih.gov/entrez/dispomim.cgi?id=604253) | **-1.42** |
| 1429384_AT | casein kinase 1, gamma 3 | [CSNK1G3](http://www.gene.ucl.ac.uk/cgi-bin/nomenclature/searchgenes.pl?field=symbol&anchor=equals&symbol_search=Search&number=100&format=html&sortby=symbol&match=CSNK1G3) | protein binding;stem cell factor receptor binding | [604253](http://www.ncbi.nlm.nih.gov/entrez/dispomim.cgi?id=604253) | **-1.83** |
| 1429400_AT | gb:BB794830 /DB_XREF=gi:16964449 /DB_XREF=BB794830 |  | protein binding;stem cell factor receptor binding;growth factor activity |  | **-1.97** |
| 1429401_AT | RIKEN cDNA 4933409N07 gene | [4933409N07RIK](http://www.gene.ucl.ac.uk/cgi-bin/nomenclature/searchgenes.pl?field=symbol&anchor=equals&symbol_search=Search&number=100&format=html&sortby=symbol&match=4933409N07RIK) | protein binding;stem cell factor receptor binding;growth factor activity |  | **1.35** |
| 1429413_AT | carboxypeptidase M | [CPM](http://www.gene.ucl.ac.uk/cgi-bin/nomenclature/searchgenes.pl?field=symbol&anchor=equals&symbol_search=Search&number=100&format=html&sortby=symbol&match=CPM) | protein binding;stem cell factor receptor binding;growth factor activity | [114860](http://www.ncbi.nlm.nih.gov/entrez/dispomim.cgi?id=114860) | **2.2** |
| 1429430_AT | RIKEN cDNA A030012M09 gene | [A030012M09RIK](http://www.gene.ucl.ac.uk/cgi-bin/nomenclature/searchgenes.pl?field=symbol&anchor=equals&symbol_search=Search&number=100&format=html&sortby=symbol&match=A030012M09RIK) | protein binding;stem cell factor receptor binding;growth factor activity |  | **1.33** |
| 1429453_A_AT | mitochondrial ribosomal protein L55 | [MRPL55](http://www.gene.ucl.ac.uk/cgi-bin/nomenclature/searchgenes.pl?field=symbol&anchor=equals&symbol_search=Search&number=100&format=html&sortby=symbol&match=MRPL55) | protein binding;structural constituent of muscle;cytoskeletal protein binding |  | **1.35** |
| 1429458_AT | gb:AV083806 /DB_XREF=gi:15404348 /DB_XREF=AV083806 |  | protein binding;structural constituent of ribosome |  | **1.87** |
| 1429463_AT | protein kinase, AMP-activated, alpha 2 catalytic subunit | [PRKAA2](http://www.gene.ucl.ac.uk/cgi-bin/nomenclature/searchgenes.pl?field=symbol&anchor=equals&symbol_search=Search&number=100&format=html&sortby=symbol&match=PRKAA2) | protein binding;sugar binding | [600497](http://www.ncbi.nlm.nih.gov/entrez/dispomim.cgi?id=600497) | **1.37** |
| 1429467_S_AT | solute carrier family 26, member 3 | [SLC26A3](http://www.gene.ucl.ac.uk/cgi-bin/nomenclature/searchgenes.pl?field=symbol&anchor=equals&symbol_search=Search&number=100&format=html&sortby=symbol&match=SLC26A3) | protein binding;sugar binding | [126650](http://www.ncbi.nlm.nih.gov/entrez/dispomim.cgi?id=126650) | **1.93** |
| 1429497_S_AT | sorting nexin 6 | [SNX6](http://www.gene.ucl.ac.uk/cgi-bin/nomenclature/searchgenes.pl?field=symbol&anchor=equals&symbol_search=Search&number=100&format=html&sortby=symbol&match=SNX6) | protein binding;sugar binding;IgE binding | [606098](http://www.ncbi.nlm.nih.gov/entrez/dispomim.cgi?id=606098) | **-1.37** |
| 1429505_AT | RIKEN cDNA 2310076G13 gene | [2310076G13RIK](http://www.gene.ucl.ac.uk/cgi-bin/nomenclature/searchgenes.pl?field=symbol&anchor=equals&symbol_search=Search&number=100&format=html&sortby=symbol&match=2310076G13RIK) | protein binding;syndecan binding;interleukin-5 receptor binding;cytoskeletal adaptor activity;oxidoreductase activity;neurexin binding |  | **1.3** |
| 1429514_AT | phosphatidic acid phosphatase type 2B | [PPAP2B](http://www.gene.ucl.ac.uk/cgi-bin/nomenclature/searchgenes.pl?field=symbol&anchor=equals&symbol_search=Search&number=100&format=html&sortby=symbol&match=PPAP2B) | protein binding;syntaxin binding;protein transporter activity | [607125](http://www.ncbi.nlm.nih.gov/entrez/dispomim.cgi?id=607125) | **1.24** |
| 1429515_AT | RIKEN cDNA E130209G04 gene | [E130209G04RIK](http://www.gene.ucl.ac.uk/cgi-bin/nomenclature/searchgenes.pl?field=symbol&anchor=equals&symbol_search=Search&number=100&format=html&sortby=symbol&match=E130209G04RIK) | protein binding;transcription corepressor activity |  | **1.43** |
| 1429524_AT | myosin IF | [MYO1F](http://www.gene.ucl.ac.uk/cgi-bin/nomenclature/searchgenes.pl?field=symbol&anchor=equals&symbol_search=Search&number=100&format=html&sortby=symbol&match=MYO1F) | protein binding;transcription corepressor activity | [601480](http://www.ncbi.nlm.nih.gov/entrez/dispomim.cgi?id=601480) | **-2.94** |
| 1429525_S_AT | myosin IF | [MYO1F](http://www.gene.ucl.ac.uk/cgi-bin/nomenclature/searchgenes.pl?field=symbol&anchor=equals&symbol_search=Search&number=100&format=html&sortby=symbol&match=MYO1F) | protein binding;transcription corepressor activity | [601480](http://www.ncbi.nlm.nih.gov/entrez/dispomim.cgi?id=601480) | **-5.28** |
| 1429528_AT | RAE1 RNA export 1 homolog (S. pombe) | [RAE1](http://www.gene.ucl.ac.uk/cgi-bin/nomenclature/searchgenes.pl?field=symbol&anchor=equals&symbol_search=Search&number=100&format=html&sortby=symbol&match=RAE1) | protein binding;transcription corepressor activity | [603343](http://www.ncbi.nlm.nih.gov/entrez/dispomim.cgi?id=603343) | **-1.26** |
| 1429533_AT | inner membrane protein, mitochondrial | [IMMT](http://www.gene.ucl.ac.uk/cgi-bin/nomenclature/searchgenes.pl?field=symbol&anchor=equals&symbol_search=Search&number=100&format=html&sortby=symbol&match=IMMT) | protein binding;transcription corepressor activity;receptor activity;signal transducer activity | [600378](http://www.ncbi.nlm.nih.gov/entrez/dispomim.cgi?id=600378) | **1.54** |
| 1429534_A_AT | inner membrane protein, mitochondrial | [IMMT](http://www.gene.ucl.ac.uk/cgi-bin/nomenclature/searchgenes.pl?field=symbol&anchor=equals&symbol_search=Search&number=100&format=html&sortby=symbol&match=IMMT) | protein binding;transcription factor activity;RNA polymerase II transcription factor activity | [600378](http://www.ncbi.nlm.nih.gov/entrez/dispomim.cgi?id=600378) | **1.4** |
| 1429539_AT | BCL2-like 13 (apoptosis facilitator) | [BCL2L13](http://www.gene.ucl.ac.uk/cgi-bin/nomenclature/searchgenes.pl?field=symbol&anchor=equals&symbol_search=Search&number=100&format=html&sortby=symbol&match=BCL2L13) | protein binding;transcription factor activity;signal transducer activity;RNA polymerase II transcription factor activity |  | **1.34** |
| 1429543_AT | RIKEN cDNA 6230424C14 gene | [6230424C14RIK](http://www.gene.ucl.ac.uk/cgi-bin/nomenclature/searchgenes.pl?field=symbol&anchor=equals&symbol_search=Search&number=100&format=html&sortby=symbol&match=6230424C14RIK) | protein binding;transcription factor binding;histone acetyltransferase binding |  | **1.82** |
| 1429588_AT | RIKEN cDNA 6720435I21 gene | [6720435I21RIK](http://www.gene.ucl.ac.uk/cgi-bin/nomenclature/searchgenes.pl?field=symbol&anchor=equals&symbol_search=Search&number=100&format=html&sortby=symbol&match=6720435I21RIK) | protein binding;transcription regulator activity |  | **-1.8** |
| 1429598_AT | RIKEN cDNA 2310042D19 gene | [2310042D19RIK](http://www.gene.ucl.ac.uk/cgi-bin/nomenclature/searchgenes.pl?field=symbol&anchor=equals&symbol_search=Search&number=100&format=html&sortby=symbol&match=2310042D19RIK) | protein binding;transcription regulator activity |  | **1.92** |
| 1429607_AT | amyotrophic lateral sclerosis 2 (juvenile) chromosome region, candidate 3 homolog(human) | [ALS2CR3](http://www.gene.ucl.ac.uk/cgi-bin/nomenclature/searchgenes.pl?field=symbol&anchor=equals&symbol_search=Search&number=100&format=html&sortby=symbol&match=ALS2CR3) | protein binding;transcriptional activator activity | [607334](http://www.ncbi.nlm.nih.gov/entrez/dispomim.cgi?id=607334) | **1.79** |
| 1429618_AT | cylindromatosis (turban tumor syndrome) | [CYLD](http://www.gene.ucl.ac.uk/cgi-bin/nomenclature/searchgenes.pl?field=symbol&anchor=equals&symbol_search=Search&number=100&format=html&sortby=symbol&match=CYLD) | protein binding;transcriptional activator activity;transcriptional repressor activity | [605018](http://www.ncbi.nlm.nih.gov/entrez/dispomim.cgi?id=605018) | **-1.45** |
| 1429621_AT | RIKEN cDNA 2210404G23 gene | [2210404G23RIK](http://www.gene.ucl.ac.uk/cgi-bin/nomenclature/searchgenes.pl?field=symbol&anchor=equals&symbol_search=Search&number=100&format=html&sortby=symbol&match=2210404G23RIK) | protein binding;transcriptional activator activity;transcriptional repressor activity |  | **1.29** |
| 1429622_AT | RIKEN cDNA 2210404G23 gene | [2210404G23RIK](http://www.gene.ucl.ac.uk/cgi-bin/nomenclature/searchgenes.pl?field=symbol&anchor=equals&symbol_search=Search&number=100&format=html&sortby=symbol&match=2210404G23RIK) | protein binding;transferase activity;2'-phosphotransferase activity |  | **1.33** |
| 1429639_AT | RIKEN cDNA 2310032D16 gene | [2310032D16RIK](http://www.gene.ucl.ac.uk/cgi-bin/nomenclature/searchgenes.pl?field=symbol&anchor=equals&symbol_search=Search&number=100&format=html&sortby=symbol&match=2310032D16RIK) | protein binding;transforming growth factor beta receptor binding;growth factor activity |  | **1.72** |
| 1429650_AT | RIKEN cDNA 2310004N11 gene | [2310004N11RIK](http://www.gene.ucl.ac.uk/cgi-bin/nomenclature/searchgenes.pl?field=symbol&anchor=equals&symbol_search=Search&number=100&format=html&sortby=symbol&match=2310004N11RIK) | protein binding;transporter activity;kinase activity;protein serine/threonine kinase activity;receptor activity;3',5'-cAMP binding;cAMP-dependent protein kinase regulator activity |  | **1.6** |
| 1429656_AT | Rho-related BTB domain containing 1 | [RHOBTB1](http://www.gene.ucl.ac.uk/cgi-bin/nomenclature/searchgenes.pl?field=symbol&anchor=equals&symbol_search=Search&number=100&format=html&sortby=symbol&match=RHOBTB1) | protein binding;transporter activity;kinase activity;protein serine/threonine kinase activity;receptor activity;3',5'-cAMP binding;cAMP-dependent protein kinase regulator activity | [607351](http://www.ncbi.nlm.nih.gov/entrez/dispomim.cgi?id=607351) | **1.86** |
| 1429658_A_AT | SMC2 structural maintenance of chromosomes 2-like 1 (yeast) | [SMC2L1](http://www.gene.ucl.ac.uk/cgi-bin/nomenclature/searchgenes.pl?field=symbol&anchor=equals&symbol_search=Search&number=100&format=html&sortby=symbol&match=SMC2L1) | protein binding;transporter activity;kinase activity;protein serine/threonine kinase activity;receptor activity;3',5'-cAMP binding;cAMP-dependent protein kinase regulator activity | [605576](http://www.ncbi.nlm.nih.gov/entrez/dispomim.cgi?id=605576) | **-3.57** |
| 1429679_AT | leucine rich repeat containing 17 | [LRRC17](http://www.gene.ucl.ac.uk/cgi-bin/nomenclature/searchgenes.pl?field=symbol&anchor=equals&symbol_search=Search&number=100&format=html&sortby=symbol&match=LRRC17) | protein binding;trypsin activity;serine-type endopeptidase activity;hydrolase activity;peptidase activity |  | **-3** |
| 1429681_A_AT | glycoprotein, synaptic 2 | [GPSN2](http://www.gene.ucl.ac.uk/cgi-bin/nomenclature/searchgenes.pl?field=symbol&anchor=equals&symbol_search=Search&number=100&format=html&sortby=symbol&match=GPSN2) | protein binding;tumor necrosis factor receptor activity |  | **1.39** |
| 1429691_AT | RIKEN cDNA 5430405N12 gene | [5430405N12RIK](http://www.gene.ucl.ac.uk/cgi-bin/nomenclature/searchgenes.pl?field=symbol&anchor=equals&symbol_search=Search&number=100&format=html&sortby=symbol&match=5430405N12RIK) | protein binding;tumor necrosis factor receptor activity;receptor activity |  | **1.55** |
| 1429708_AT | NADH dehydrogenase (ubiquinone) 1 alpha subcomplex | [NDUFA11](http://www.gene.ucl.ac.uk/cgi-bin/nomenclature/searchgenes.pl?field=symbol&anchor=equals&symbol_search=Search&number=100&format=html&sortby=symbol&match=NDUFA11) | protein binding;vascular endothelial growth factor receptor binding;growth factor activity |  | **1.26** |
| 1429709_AT | peptidase (mitochondrial processing) beta | [PMPCB](http://www.gene.ucl.ac.uk/cgi-bin/nomenclature/searchgenes.pl?field=symbol&anchor=equals&symbol_search=Search&number=100&format=html&sortby=symbol&match=PMPCB) | protein binding;voltage-gated potassium channel activity | [603131](http://www.ncbi.nlm.nih.gov/entrez/dispomim.cgi?id=603131) | **1.37** |
| 1429728_AT | RIKEN cDNA 4930429M06Rik | [4930429M06RIK](http://www.gene.ucl.ac.uk/cgi-bin/nomenclature/searchgenes.pl?field=symbol&anchor=equals&symbol_search=Search&number=100&format=html&sortby=symbol&match=4930429M06RIK) | protein binding;voltage-gated potassium channel activity |  | **-1.59** |
| 1429735_AT | RIKEN cDNA 1110003F05 gene | [1110003F05RIK](http://www.gene.ucl.ac.uk/cgi-bin/nomenclature/searchgenes.pl?field=symbol&anchor=equals&symbol_search=Search&number=100&format=html&sortby=symbol&match=1110003F05RIK) | protein binding;zinc ion binding |  | **1.3** |
| 1429736_AT | RIKEN cDNA 1110003F05 gene | [1110003F05RIK](http://www.gene.ucl.ac.uk/cgi-bin/nomenclature/searchgenes.pl?field=symbol&anchor=equals&symbol_search=Search&number=100&format=html&sortby=symbol&match=1110003F05RIK) | protein binding;zinc ion binding |  | **1.36** |
| 1429752_X_AT | RIKEN cDNA 1700024K14 gene | [RSNL2](http://www.gene.ucl.ac.uk/cgi-bin/nomenclature/searchgenes.pl?field=symbol&anchor=equals&symbol_search=Search&number=100&format=html&sortby=symbol&match=RSNL2) | protein binding;zinc ion binding |  | **1.55** |
| 1429775_A_AT | transmembrane 7 superfamily member 1 | [TM7SF1](http://www.gene.ucl.ac.uk/cgi-bin/nomenclature/searchgenes.pl?field=symbol&anchor=equals&symbol_search=Search&number=100&format=html&sortby=symbol&match=TM7SF1) | protein binding;zinc ion binding;carbonate dehydratase activity | [604658](http://www.ncbi.nlm.nih.gov/entrez/dispomim.cgi?id=604658) | **-1.72** |
| 1429778_AT | optineurin | [OPTN](http://www.gene.ucl.ac.uk/cgi-bin/nomenclature/searchgenes.pl?field=symbol&anchor=equals&symbol_search=Search&number=100&format=html&sortby=symbol&match=OPTN) | protein binding;zinc ion binding;DNA binding;nucleic acid binding | [602432](http://www.ncbi.nlm.nih.gov/entrez/dispomim.cgi?id=602432) | **1.94** |
| 1429787_X_AT | ZW10 interactor | [ZWINT](http://www.gene.ucl.ac.uk/cgi-bin/nomenclature/searchgenes.pl?field=symbol&anchor=equals&symbol_search=Search&number=100&format=html&sortby=symbol&match=ZWINT) | protein binding;zinc ion binding;DNA binding;nucleic acid binding |  | **-1.34** |
| 1429819_AT | nicotinamide nucleotide adenylyltransferase 1 | [NMNAT1](http://www.gene.ucl.ac.uk/cgi-bin/nomenclature/searchgenes.pl?field=symbol&anchor=equals&symbol_search=Search&number=100&format=html&sortby=symbol&match=NMNAT1) | protein binding;zinc ion binding;ligase activity;ubiquitin-protein ligase activity | [608700](http://www.ncbi.nlm.nih.gov/entrez/dispomim.cgi?id=608700) | **1.73** |
| 1429830_A_AT | CD59a antigen | [CD59A](http://www.gene.ucl.ac.uk/cgi-bin/nomenclature/searchgenes.pl?field=symbol&anchor=equals&symbol_search=Search&number=100&format=html&sortby=symbol&match=CD59A) | protein binding;zinc ion binding;ligase activity;ubiquitin-protein ligase activity |  | **1.79** |
| 1429839_A_AT | gb:AK012787.1 /DB_XREF=gi:12849760 /FEA=mRNA /CNT= |  | protein binding;zinc ion binding;NADPH:quinone reductase activity;alcohol dehydrogenase activity, zinc-dependent |  | **1.41** |
| 1429841_AT | RIKEN cDNA 3000002B06 gene | [3000002B06RIK](http://www.gene.ucl.ac.uk/cgi-bin/nomenclature/searchgenes.pl?field=symbol&anchor=equals&symbol_search=Search&number=100&format=html&sortby=symbol&match=3000002B06RIK) | protein binding;zinc ion binding;nucleic acid binding;DNA binding |  | **-2.85** |
| 1429887_AT | RIKEN cDNA 2310005C01 gene | [2310005C01RIK](http://www.gene.ucl.ac.uk/cgi-bin/nomenclature/searchgenes.pl?field=symbol&anchor=equals&symbol_search=Search&number=100&format=html&sortby=symbol&match=2310005C01RIK) | protein binding;zinc ion binding;receptor activity;ligase activity;ubiquitin-protein ligase activity |  | **2.57** |
| 1429946_AT | RIKEN cDNA 2610301F02 gene | [2610301F02RIK](http://www.gene.ucl.ac.uk/cgi-bin/nomenclature/searchgenes.pl?field=symbol&anchor=equals&symbol_search=Search&number=100&format=html&sortby=symbol&match=2610301F02RIK) | protein binding;zinc ion binding;ubiquitin-protein ligase activity |  | **-2.14** |
| 1429951_AT | single-stranded DNA binding protein 2 | [SSBP2](http://www.gene.ucl.ac.uk/cgi-bin/nomenclature/searchgenes.pl?field=symbol&anchor=equals&symbol_search=Search&number=100&format=html&sortby=symbol&match=SSBP2) | protein carrier activity | [607389](http://www.ncbi.nlm.nih.gov/entrez/dispomim.cgi?id=607389) | **-1.63** |
| 1429954_AT | gb:AK014135.1 /DB_XREF=gi:12851811 /FEA=mRNA /CNT= |  | protein carrier activity |  | **-3.95** |
| 1430020_X_AT | heterogeneous nuclear ribonucleoprotein A1 | [HNRPA1](http://www.gene.ucl.ac.uk/cgi-bin/nomenclature/searchgenes.pl?field=symbol&anchor=equals&symbol_search=Search&number=100&format=html&sortby=symbol&match=HNRPA1) | protein disulfide oxidoreductase activity;glutathione peroxidase activity;glutathione transferase activity;transferase activity | [164017](http://www.ncbi.nlm.nih.gov/entrez/dispomim.cgi?id=164017) | **-1.31** |
| 1430029_A_AT | sarcoma amplified sequence | [SAS](http://www.gene.ucl.ac.uk/cgi-bin/nomenclature/searchgenes.pl?field=symbol&anchor=equals&symbol_search=Search&number=100&format=html&sortby=symbol&match=SAS) | protein domain specific binding | [181035](http://www.ncbi.nlm.nih.gov/entrez/dispomim.cgi?id=181035) | **-1.25** |
| 1430038_AT | gephyrin | [GPHN](http://www.gene.ucl.ac.uk/cgi-bin/nomenclature/searchgenes.pl?field=symbol&anchor=equals&symbol_search=Search&number=100&format=html&sortby=symbol&match=GPHN) | protein domain specific binding | [603930](http://www.ncbi.nlm.nih.gov/entrez/dispomim.cgi?id=603930) | **1.47** |
| 1430053_A_AT | RIKEN cDNA 2810409H07 gene | [2810409H07RIK](http://www.gene.ucl.ac.uk/cgi-bin/nomenclature/searchgenes.pl?field=symbol&anchor=equals&symbol_search=Search&number=100&format=html&sortby=symbol&match=2810409H07RIK) | protein domain specific binding;calcium ion binding;GTPase activity;GTP binding;ATP binding |  | **-1.4** |
| 1430123_A_AT | aldo-keto reductase family 1, member A4 (aldehyde reductase) | [AKR1A4](http://www.gene.ucl.ac.uk/cgi-bin/nomenclature/searchgenes.pl?field=symbol&anchor=equals&symbol_search=Search&number=100&format=html&sortby=symbol&match=AKR1A4) | protein domain specific binding;cysteine-type endopeptidase activity;hydrolase activity;cysteine-type peptidase activity;calpain activity |  | **-1.38** |
| 1430124_X_AT | aldo-keto reductase family 1, member A4 (aldehyde reductase) | [AKR1A4](http://www.gene.ucl.ac.uk/cgi-bin/nomenclature/searchgenes.pl?field=symbol&anchor=equals&symbol_search=Search&number=100&format=html&sortby=symbol&match=AKR1A4) | protein domain specific binding;GTPase activity;calcium ion binding;GTP binding;ATP binding |  | **-1.41** |
| 1430205_A_AT | cell division cycle 37 homolog (S. cerevisiae)-like 1 | [CDC37L1](http://www.gene.ucl.ac.uk/cgi-bin/nomenclature/searchgenes.pl?field=symbol&anchor=equals&symbol_search=Search&number=100&format=html&sortby=symbol&match=CDC37L1) | protein heterodimerization activity;ATPase activity;DNA supercoiling activity;ATPase activity, coupled to transmembrane movement of substances;ATP-binding cassette (ABC) transporter activity;ATP binding |  | **1.27** |
| 1430288_X_AT | ribosomal protein S21 | [RPS21](http://www.gene.ucl.ac.uk/cgi-bin/nomenclature/searchgenes.pl?field=symbol&anchor=equals&symbol_search=Search&number=100&format=html&sortby=symbol&match=RPS21) | protein heterodimerization activity;ATPase activity;DNA supercoiling activity;ATPase activity, coupled to transmembrane movement of substances;ATP-binding cassette (ABC) transporter activity;ATP binding | [180477](http://www.ncbi.nlm.nih.gov/entrez/dispomim.cgi?id=180477) | **-1.25** |
| 1430307_A_AT | malic enzyme, supernatant | [MOD1](http://www.gene.ucl.ac.uk/cgi-bin/nomenclature/searchgenes.pl?field=symbol&anchor=equals&symbol_search=Search&number=100&format=html&sortby=symbol&match=MOD1) | protein heterodimerization activity;ATPase activity;DNA supercoiling activity;ATPase activity, coupled to transmembrane movement of substances;ATP-binding cassette (ABC) transporter activity;ATP binding |  | **2.31** |
| 1430326_S_AT | RIKEN cDNA 1500040F11 gene | [1500040F11RIK](http://www.gene.ucl.ac.uk/cgi-bin/nomenclature/searchgenes.pl?field=symbol&anchor=equals&symbol_search=Search&number=100&format=html&sortby=symbol&match=1500040F11RIK) | protein heterodimerization activity;diacylglycerol kinase activity;protein homodimerization activity |  | **1.2** |
| 1430379_AT | RIKEN cDNA 5830411K21 gene | [5830411K21RIK](http://www.gene.ucl.ac.uk/cgi-bin/nomenclature/searchgenes.pl?field=symbol&anchor=equals&symbol_search=Search&number=100&format=html&sortby=symbol&match=5830411K21RIK) | protein heterodimerization activity;PDZ domain binding;voltage-gated chloride channel activity;chloride channel activity;ion channel activity;voltage-gated ion channel activity;protein homodimerization activity |  | **-2.08** |
| 1430447_A_AT | leukocyte-associated Ig-like receptor 1 | [LAIR1](http://www.gene.ucl.ac.uk/cgi-bin/nomenclature/searchgenes.pl?field=symbol&anchor=equals&symbol_search=Search&number=100&format=html&sortby=symbol&match=LAIR1) | protein heterodimerization activity;PDZ domain binding;voltage-gated chloride channel activity;chloride channel activity;voltage-gated ion channel activity;ion channel activity;protein homodimerization activity | [602992](http://www.ncbi.nlm.nih.gov/entrez/dispomim.cgi?id=602992) | **-1.67** |
| 1430462_AT | RIKEN cDNA 2310002L09 gene | [2310002L09RIK](http://www.gene.ucl.ac.uk/cgi-bin/nomenclature/searchgenes.pl?field=symbol&anchor=equals&symbol_search=Search&number=100&format=html&sortby=symbol&match=2310002L09RIK) | protein heterodimerization activity;protein binding;chromatin binding;microtubule motor activity;ATPase activity, coupled to transmembrane movement of substances;ATP-binding cassette (ABC) transporter activity;ATP binding |  | **2.01** |
| 1430474_A_AT | mitochondrial carrier homolog 2 (C. elegans) | [MTCH2](http://www.gene.ucl.ac.uk/cgi-bin/nomenclature/searchgenes.pl?field=symbol&anchor=equals&symbol_search=Search&number=100&format=html&sortby=symbol&match=MTCH2) | protein heterodimerization activity;protein binding;protein C-terminus binding;ubiquitin activating enzyme activity;ligase activity;catalytic activity |  | **1.27** |
| 1430500_S_AT | metaxin 2 | [MTX2](http://www.gene.ucl.ac.uk/cgi-bin/nomenclature/searchgenes.pl?field=symbol&anchor=equals&symbol_search=Search&number=100&format=html&sortby=symbol&match=MTX2) | protein kinase activity;kinase activity;ATP binding;transferase activity | [608555](http://www.ncbi.nlm.nih.gov/entrez/dispomim.cgi?id=608555) | **1.29** |
| 1430519_A_AT | CCR4-NOT transcription complex, subunit 7 | [CNOT7](http://www.gene.ucl.ac.uk/cgi-bin/nomenclature/searchgenes.pl?field=symbol&anchor=equals&symbol_search=Search&number=100&format=html&sortby=symbol&match=CNOT7) | protein kinase activity;kinase activity;ATP binding;transferase activity | [604913](http://www.ncbi.nlm.nih.gov/entrez/dispomim.cgi?id=604913) | **1.39** |
| 1430522_A_AT | vesicle-associated membrane protein 5 | [VAMP5](http://www.gene.ucl.ac.uk/cgi-bin/nomenclature/searchgenes.pl?field=symbol&anchor=equals&symbol_search=Search&number=100&format=html&sortby=symbol&match=VAMP5) | protein kinase activity;kinase activity;ATP binding;transferase activity | [607029](http://www.ncbi.nlm.nih.gov/entrez/dispomim.cgi?id=607029) | **1.33** |
| 1430527_A_AT | ring finger protein 167 | [RNF167](http://www.gene.ucl.ac.uk/cgi-bin/nomenclature/searchgenes.pl?field=symbol&anchor=equals&symbol_search=Search&number=100&format=html&sortby=symbol&match=RNF167) | protein kinase activity;kinase activity;protein serine/threonine kinase activity;ATP binding;protein-tyrosine kinase activity;transferase activity |  | **1.29** |
| 1430536_A_AT | enhancer of rudimentary homolog (Drosophila) | [ERH](http://www.gene.ucl.ac.uk/cgi-bin/nomenclature/searchgenes.pl?field=symbol&anchor=equals&symbol_search=Search&number=100&format=html&sortby=symbol&match=ERH) | protein kinase activity;kinase activity;protein serine/threonine kinase activity;ATP binding;protein-tyrosine kinase activity;transferase activity | [601191](http://www.ncbi.nlm.nih.gov/entrez/dispomim.cgi?id=601191) | **-1.39** |
| 1430542_A_AT | solute carrier family 25 (mitochondrial carrier, adenine nucleotide translocator), member 5 | [SLC25A5](http://www.gene.ucl.ac.uk/cgi-bin/nomenclature/searchgenes.pl?field=symbol&anchor=equals&symbol_search=Search&number=100&format=html&sortby=symbol&match=SLC25A5) | protein kinase activity;kinase activity;protein serine/threonine kinase activity;ATP binding;protein-tyrosine kinase activity;transferase activity | [300150](http://www.ncbi.nlm.nih.gov/entrez/dispomim.cgi?id=300150) | **-1.63** |
| 1430561_AT | RIKEN cDNA 5730496F10 gene | [5730496F10RIK](http://www.gene.ucl.ac.uk/cgi-bin/nomenclature/searchgenes.pl?field=symbol&anchor=equals&symbol_search=Search&number=100&format=html&sortby=symbol&match=5730496F10RIK) | protein kinase activity;kinase activity;protein serine/threonine kinase activity;ATP binding;protein-tyrosine kinase activity;transferase activity |  | **-1.49** |
| 1430596_S_AT | RIKEN cDNA 1700110N18 gene | [1700110N18RIK](http://www.gene.ucl.ac.uk/cgi-bin/nomenclature/searchgenes.pl?field=symbol&anchor=equals&symbol_search=Search&number=100&format=html&sortby=symbol&match=1700110N18RIK) | protein kinase activity;kinase activity;protein serine/threonine kinase activity;ATP binding;structural constituent of ribosome;transferase activity |  | **-1.53** |
| 1430656_A_AT | RIKEN cDNA 2210409M21 gene | [2210409M21RIK](http://www.gene.ucl.ac.uk/cgi-bin/nomenclature/searchgenes.pl?field=symbol&anchor=equals&symbol_search=Search&number=100&format=html&sortby=symbol&match=2210409M21RIK) | protein kinase activity;kinase activity;protein serine/threonine kinase activity;ATP binding;transferase activity |  | **1.24** |
| 1430697_AT | Alport syndrome, mental retardation, midface hypoplasia and elliptocytosis chromosomal region gene 1 homolog (human) | [AMMECR1](http://www.gene.ucl.ac.uk/cgi-bin/nomenclature/searchgenes.pl?field=symbol&anchor=equals&symbol_search=Search&number=100&format=html&sortby=symbol&match=AMMECR1) | protein kinase activity;kinase activity;protein serine/threonine kinase activity;ATP binding;transferase activity | [300195](http://www.ncbi.nlm.nih.gov/entrez/dispomim.cgi?id=300195) | **-4.94** |
| 1430700_A_AT | phospholipase A2, group VII (platelet-activating factor acetylhydrolase, plasma) | [PLA2G7](http://www.gene.ucl.ac.uk/cgi-bin/nomenclature/searchgenes.pl?field=symbol&anchor=equals&symbol_search=Search&number=100&format=html&sortby=symbol&match=PLA2G7) | protein kinase activity;kinase activity;protein serine/threonine kinase activity;ATP binding;transferase activity | [601690](http://www.ncbi.nlm.nih.gov/entrez/dispomim.cgi?id=601690) | **-2.36** |
| 1430769_S_AT | RIKEN cDNA 2900009I07 gene | [2900009I07RIK](http://www.gene.ucl.ac.uk/cgi-bin/nomenclature/searchgenes.pl?field=symbol&anchor=equals&symbol_search=Search&number=100&format=html&sortby=symbol&match=2900009I07RIK) | protein kinase activity;kinase activity;protein serine/threonine kinase activity;ATP binding;transferase activity |  | **1.22** |
| 1430849_A_AT | cell growth regulator with ring finger domain 1 | [CGRRF1](http://www.gene.ucl.ac.uk/cgi-bin/nomenclature/searchgenes.pl?field=symbol&anchor=equals&symbol_search=Search&number=100&format=html&sortby=symbol&match=CGRRF1) | protein kinase activity;kinase activity;protein serine/threonine kinase activity;ATP binding;transferase activity | [606138](http://www.ncbi.nlm.nih.gov/entrez/dispomim.cgi?id=606138) | **1.31** |
| 1430885_AT | RIKEN cDNA 1110012N22 gene | [1110012N22RIK](http://www.gene.ucl.ac.uk/cgi-bin/nomenclature/searchgenes.pl?field=symbol&anchor=equals&symbol_search=Search&number=100&format=html&sortby=symbol&match=1110012N22RIK) | protein kinase activity;kinase activity;protein serine/threonine kinase activity;ATP binding;transferase activity |  | **1.29** |
| 1430896_S_AT | nudix (nucleoside diphosphate linked moiety X)-type motif 7 | [NUDT7](http://www.gene.ucl.ac.uk/cgi-bin/nomenclature/searchgenes.pl?field=symbol&anchor=equals&symbol_search=Search&number=100&format=html&sortby=symbol&match=NUDT7) | protein kinase activity;kinase activity;protein serine/threonine kinase activity;ATP binding;transferase activity |  | **1.81** |
| 1430978_AT | ribosomal protein S25 | [RPS25](http://www.gene.ucl.ac.uk/cgi-bin/nomenclature/searchgenes.pl?field=symbol&anchor=equals&symbol_search=Search&number=100&format=html&sortby=symbol&match=RPS25) | protein kinase activity;kinase activity;protein serine/threonine kinase activity;ATP binding;transferase activity | [180465](http://www.ncbi.nlm.nih.gov/entrez/dispomim.cgi?id=180465) | **-1.52** |
| 1430992_S_AT | RIKEN cDNA 1500009M05 gene | [1500009M05RIK](http://www.gene.ucl.ac.uk/cgi-bin/nomenclature/searchgenes.pl?field=symbol&anchor=equals&symbol_search=Search&number=100&format=html&sortby=symbol&match=1500009M05RIK) | protein kinase activity;kinase activity;protein serine/threonine kinase activity;calcium/calmodulin-dependent protein kinase activity;ATP binding;calmodulin binding;transferase activity |  | **1.46** |
| 1431003_A_AT | RIKEN cDNA 2610002J02 gene | [2610002J02RIK](http://www.gene.ucl.ac.uk/cgi-bin/nomenclature/searchgenes.pl?field=symbol&anchor=equals&symbol_search=Search&number=100&format=html&sortby=symbol&match=2610002J02RIK) | protein kinase activity;kinase activity;protein serine/threonine kinase activity;calcium/calmodulin-dependent protein kinase activity;ATP binding;calmodulin binding;transferase activity |  | **-1.5** |
| 1431012_A_AT | peroxisomal delta3, delta2-enoyl-Coenzyme A isomerase | [PECI](http://www.gene.ucl.ac.uk/cgi-bin/nomenclature/searchgenes.pl?field=symbol&anchor=equals&symbol_search=Search&number=100&format=html&sortby=symbol&match=PECI) | protein kinase activity;kinase activity;protein serine/threonine kinase activity;calcium/calmodulin-dependent protein kinase activity;ATP binding;protein-tyrosine kinase activity;calmodulin binding;transferase activity | [608024](http://www.ncbi.nlm.nih.gov/entrez/dispomim.cgi?id=608024) | **1.24** |
| 1431043_AT | kelch repeat and BTB (POZ) domain containing 5 | [KBTBD5](http://www.gene.ucl.ac.uk/cgi-bin/nomenclature/searchgenes.pl?field=symbol&anchor=equals&symbol_search=Search&number=100&format=html&sortby=symbol&match=KBTBD5) | protein kinase activity;kinase activity;protein serine/threonine kinase activity;diacylglycerol binding;protein-tyrosine kinase activity;ATP binding;transferase activity |  | **-1.53** |
| 1431055_A_AT | sorting nexin 10 | [SNX10](http://www.gene.ucl.ac.uk/cgi-bin/nomenclature/searchgenes.pl?field=symbol&anchor=equals&symbol_search=Search&number=100&format=html&sortby=symbol&match=SNX10) | protein kinase activity;kinase activity;protein serine/threonine kinase activity;magnesium ion binding;3-phosphoinositide-dependent protein kinase activity;protein-tyrosine kinase activity;ATP binding;transferase activity |  | **-1.91** |
| 1431056_A_AT | lipoprotein lipase | [LPL](http://www.gene.ucl.ac.uk/cgi-bin/nomenclature/searchgenes.pl?field=symbol&anchor=equals&symbol_search=Search&number=100&format=html&sortby=symbol&match=LPL) | protein kinase activity;kinase activity;protein serine/threonine kinase activity;magnesium ion binding;ATP binding;transferase activity | [238600](http://www.ncbi.nlm.nih.gov/entrez/dispomim.cgi?id=238600) | **2.53** |
| 1431057_A_AT | RIKEN cDNA 2310046G15 gene | [2310046G15RIK](http://www.gene.ucl.ac.uk/cgi-bin/nomenclature/searchgenes.pl?field=symbol&anchor=equals&symbol_search=Search&number=100&format=html&sortby=symbol&match=2310046G15RIK) | protein kinase activity;kinase activity;protein serine/threonine kinase activity;protein-tyrosine kinase activity;ATP binding;transferase activity |  | **1.38** |
| 1431074_A_AT | phosphatidylinositol transfer protein, cytoplasmic 1 | [PITPNC1](http://www.gene.ucl.ac.uk/cgi-bin/nomenclature/searchgenes.pl?field=symbol&anchor=equals&symbol_search=Search&number=100&format=html&sortby=symbol&match=PITPNC1) | protein kinase activity;kinase activity;protein serine/threonine kinase activity;protein-tyrosine kinase activity;ATP binding;transferase activity | [605134](http://www.ncbi.nlm.nih.gov/entrez/dispomim.cgi?id=605134) | **1.49** |
| 1431142_S_AT | apoptosis-inducing factor (AIF)-like mitochondrion-associated inducer of death | [AMID](http://www.gene.ucl.ac.uk/cgi-bin/nomenclature/searchgenes.pl?field=symbol&anchor=equals&symbol_search=Search&number=100&format=html&sortby=symbol&match=AMID) | protein kinase activity;kinase activity;protein serine/threonine kinase activity;protein-tyrosine kinase activity;ATP binding;transferase activity |  | **1.87** |
| 1431143_X_AT | gb:AK017403.1 /DB_XREF=gi:12856624 /FEA=mRNA /CNT= |  | protein kinase activity;kinase activity;protein serine/threonine kinase activity;protein-tyrosine kinase activity;ATP binding;transferase activity |  | **1.75** |
| 1431177_A_AT | ribosomal protein L10A | [RPL10A](http://www.gene.ucl.ac.uk/cgi-bin/nomenclature/searchgenes.pl?field=symbol&anchor=equals&symbol_search=Search&number=100&format=html&sortby=symbol&match=RPL10A) | protein kinase activity;kinase activity;protein serine/threonine kinase activity;protein-tyrosine kinase activity;ATP binding;transferase activity |  | **-1.23** |
| 1431199_AT | RIKEN cDNA 0610031G08 gene | [0610031G08RIK](http://www.gene.ucl.ac.uk/cgi-bin/nomenclature/searchgenes.pl?field=symbol&anchor=equals&symbol_search=Search&number=100&format=html&sortby=symbol&match=0610031G08RIK) | protein kinase activity;kinase activity;protein serine/threonine kinase activity;protein-tyrosine kinase activity;diacylglycerol binding;ATP binding;transferase activity |  | **1.89** |
| 1431208_A_AT | solute carrier family 9 (sodium/hydrogen exchanger), isoform 3 regulator 2 | [SLC9A3R2](http://www.gene.ucl.ac.uk/cgi-bin/nomenclature/searchgenes.pl?field=symbol&anchor=equals&symbol_search=Search&number=100&format=html&sortby=symbol&match=SLC9A3R2) | protein kinase activity;kinase activity;protein serine/threonine kinase activity;receptor activity;protein-tyrosine kinase activity;ATP binding;transferase activity | [606553](http://www.ncbi.nlm.nih.gov/entrez/dispomim.cgi?id=606553) | **2.23** |
| 1431225_AT | RIKEN cDNA 1110038H03 gene | [6230403H02RIK](http://www.gene.ucl.ac.uk/cgi-bin/nomenclature/searchgenes.pl?field=symbol&anchor=equals&symbol_search=Search&number=100&format=html&sortby=symbol&match=6230403H02RIK) | protein kinase activity;kinase activity;protein serine/threonine kinase activity;small GTPase regulatory/interacting protein activity;ATP binding;transferase activity |  | **-7.39** |
| 1431261_AT | chloride intracellular channel 5 | [CLIC5](http://www.gene.ucl.ac.uk/cgi-bin/nomenclature/searchgenes.pl?field=symbol&anchor=equals&symbol_search=Search&number=100&format=html&sortby=symbol&match=CLIC5) | protein kinase activity;kinase activity;protein serine/threonine kinase activity;SNF1A/AMP-activated protein kinase activity;ATP binding;transferase activity | [607293](http://www.ncbi.nlm.nih.gov/entrez/dispomim.cgi?id=607293) | **1.89** |
| 1431274_A_AT | heat shock protein, A | [HSPA9A](http://www.gene.ucl.ac.uk/cgi-bin/nomenclature/searchgenes.pl?field=symbol&anchor=equals&symbol_search=Search&number=100&format=html&sortby=symbol&match=HSPA9A) | protein kinase activity;kinase activity;receptor activity;ATP binding;protein-tyrosine kinase activity;transferase activity |  | **1.81** |
| 1431285_AT | mahogunin, ring finger 1 | [MGRN1](http://www.gene.ucl.ac.uk/cgi-bin/nomenclature/searchgenes.pl?field=symbol&anchor=equals&symbol_search=Search&number=100&format=html&sortby=symbol&match=MGRN1) | protein kinase activity;protein serine/threonine kinase activity;ATP binding;protein-tyrosine kinase activity;phosphorylase kinase activity;calmodulin binding;transferase activity | [607559](http://www.ncbi.nlm.nih.gov/entrez/dispomim.cgi?id=607559) | **1.36** |
| 1431308_AT | RIKEN cDNA 5730557B15 gene | [5730557B15RIK](http://www.gene.ucl.ac.uk/cgi-bin/nomenclature/searchgenes.pl?field=symbol&anchor=equals&symbol_search=Search&number=100&format=html&sortby=symbol&match=5730557B15RIK) | protein kinase activity;protein serine/threonine kinase activity;ATP binding;protein-tyrosine kinase activity;transferase activity |  | **2.01** |
| 1431320_A_AT | myosin Va | [MYO5A](http://www.gene.ucl.ac.uk/cgi-bin/nomenclature/searchgenes.pl?field=symbol&anchor=equals&symbol_search=Search&number=100&format=html&sortby=symbol&match=MYO5A) | protein kinase activity;protein serine/threonine kinase activity;ATP binding;protein-tyrosine kinase activity;transferase activity | [160777](http://www.ncbi.nlm.nih.gov/entrez/dispomim.cgi?id=160777) | **-2.07** |
| 1431339_A_AT | EF hand domain containing 2 | [EFHD2](http://www.gene.ucl.ac.uk/cgi-bin/nomenclature/searchgenes.pl?field=symbol&anchor=equals&symbol_search=Search&number=100&format=html&sortby=symbol&match=EFHD2) | protein kinase activity;protein serine/threonine kinase activity;ATP binding;protein-tyrosine kinase activity;transferase activity |  | **-2.02** |
| 1431354_A_AT | phenylalanine-tRNA synthetase 1 (mitochondrial) | [FARS1](http://www.gene.ucl.ac.uk/cgi-bin/nomenclature/searchgenes.pl?field=symbol&anchor=equals&symbol_search=Search&number=100&format=html&sortby=symbol&match=FARS1) | protein kinase activity;protein serine/threonine kinase activity;ATP binding;transferase activity |  | **1.43** |
| 1431372_AT | serine/arginine-rich protein specific kinase 2 | [SRPK2](http://www.gene.ucl.ac.uk/cgi-bin/nomenclature/searchgenes.pl?field=symbol&anchor=equals&symbol_search=Search&number=100&format=html&sortby=symbol&match=SRPK2) | protein kinase activity;protein serine/threonine kinase activity;ATP binding;transferase activity | [602980](http://www.ncbi.nlm.nih.gov/entrez/dispomim.cgi?id=602980) | **-1.52** |
| 1431382_A_AT | RIKEN cDNA 1700024K14 gene | [RSNL2](http://www.gene.ucl.ac.uk/cgi-bin/nomenclature/searchgenes.pl?field=symbol&anchor=equals&symbol_search=Search&number=100&format=html&sortby=symbol&match=RSNL2) | protein kinase activity;protein serine/threonine kinase activity;calcium/calmodulin-dependent protein kinase activity;protein-tyrosine kinase activity;ATP binding;calmodulin binding;transferase activity |  | **1.58** |
| 1431390_A_AT | glutamate receptor, ionotropic, N-methyl D-aspartate-like 1A | [GRINL1A](http://www.gene.ucl.ac.uk/cgi-bin/nomenclature/searchgenes.pl?field=symbol&anchor=equals&symbol_search=Search&number=100&format=html&sortby=symbol&match=GRINL1A) | protein kinase activity;protein serine/threonine kinase activity;magnesium ion binding;protein-tyrosine kinase activity;ATP binding;transferase activity | [606485](http://www.ncbi.nlm.nih.gov/entrez/dispomim.cgi?id=606485) | **1.26** |
| 1431420_S_AT | RIKEN cDNA 2610524G07 gene | [2610524G07RIK](http://www.gene.ucl.ac.uk/cgi-bin/nomenclature/searchgenes.pl?field=symbol&anchor=equals&symbol_search=Search&number=100&format=html&sortby=symbol&match=2610524G07RIK) | protein kinase activity;protein serine/threonine kinase activity;protein-tyrosine kinase activity;ATP binding;calmodulin binding;phosphorylase kinase activity;transferase activity |  | **-1.51** |
| 1431422_A_AT | dual specificity phosphatase 14 | [DUSP14](http://www.gene.ucl.ac.uk/cgi-bin/nomenclature/searchgenes.pl?field=symbol&anchor=equals&symbol_search=Search&number=100&format=html&sortby=symbol&match=DUSP14) | protein kinase activity;protein serine/threonine kinase activity;protein-tyrosine kinase activity;ATP binding;transferase activity | [606618](http://www.ncbi.nlm.nih.gov/entrez/dispomim.cgi?id=606618) | **-1.8** |
| 1431431_A_AT | nitrogen fixation gene 1 (S. cerevisiae) | [NFS1](http://www.gene.ucl.ac.uk/cgi-bin/nomenclature/searchgenes.pl?field=symbol&anchor=equals&symbol_search=Search&number=100&format=html&sortby=symbol&match=NFS1) | protein kinase activity;protein serine/threonine kinase activity;receptor activity;transmembrane receptor protein tyrosine kinase activity;ATP binding;protein-tyrosine kinase activity;transferase activity | [603485](http://www.ncbi.nlm.nih.gov/entrez/dispomim.cgi?id=603485) | **1.6** |
| 1431558_AT | RIKEN cDNA 2310016D03 gene | [2310016D03RIK](http://www.gene.ucl.ac.uk/cgi-bin/nomenclature/searchgenes.pl?field=symbol&anchor=equals&symbol_search=Search&number=100&format=html&sortby=symbol&match=2310016D03RIK) | protein kinase activity;protein serine/threonine kinase activity;small GTPase regulatory/interacting protein activity;ATP binding;protein-tyrosine kinase activity;transferase activity |  | **1.92** |
| 1431591_S_AT | interferon, alpha-inducible protein | [G1P2](http://www.gene.ucl.ac.uk/cgi-bin/nomenclature/searchgenes.pl?field=symbol&anchor=equals&symbol_search=Search&number=100&format=html&sortby=symbol&match=G1P2) | protein kinase activity;protein serine/threonine kinase activity;small GTPase regulatory/interacting protein activity;ATP binding;protein-tyrosine kinase activity;transferase activity | [147571](http://www.ncbi.nlm.nih.gov/entrez/dispomim.cgi?id=147571) | **-1.53** |
| 1431619_A_AT | dystrobrevin binding protein 1 | [DTNBP1](http://www.gene.ucl.ac.uk/cgi-bin/nomenclature/searchgenes.pl?field=symbol&anchor=equals&symbol_search=Search&number=100&format=html&sortby=symbol&match=DTNBP1) | protein kinase activity;protein serine/threonine kinase activity;small GTPase regulatory/interacting protein activity;ATP binding;protein-tyrosine kinase activity;transferase activity | [607145](http://www.ncbi.nlm.nih.gov/entrez/dispomim.cgi?id=607145) | **1.92** |
| 1431645_A_AT | guanosine diphosphate (GDP) dissociation inhibitor 3 | [GDI3](http://www.gene.ucl.ac.uk/cgi-bin/nomenclature/searchgenes.pl?field=symbol&anchor=equals&symbol_search=Search&number=100&format=html&sortby=symbol&match=GDI3) | protein kinase activity;protein serine/threonine kinase activity;small GTPase regulatory/interacting protein activity;protein-tyrosine kinase activity;ATP binding;transferase activity | [600767](http://www.ncbi.nlm.nih.gov/entrez/dispomim.cgi?id=600767) | **-1.21** |
| 1431665_A_AT | translocase of inner mitochondrial membrane 8 homolog b (yeast) | [TIMM8B](http://www.gene.ucl.ac.uk/cgi-bin/nomenclature/searchgenes.pl?field=symbol&anchor=equals&symbol_search=Search&number=100&format=html&sortby=symbol&match=TIMM8B) | protein kinase inhibitor activity | [606659](http://www.ncbi.nlm.nih.gov/entrez/dispomim.cgi?id=606659) | **1.33** |
| 1431751_A_AT | RIKEN cDNA 2700082O15 gene | [2700082O15RIK](http://www.gene.ucl.ac.uk/cgi-bin/nomenclature/searchgenes.pl?field=symbol&anchor=equals&symbol_search=Search&number=100&format=html&sortby=symbol&match=2700082O15RIK) | protein methyltransferase activity;methyltransferase activity;histone-arginine N-methyltransferase activity;S-adenosylmethionine-dependent methyltransferase activity;[cytochrome c]-arginine N-methyltransferase activity;transferase activity |  | **1.68** |
| 1431760_A_AT | RIKEN cDNA C330016H24 gene | [C330016H24RIK](http://www.gene.ucl.ac.uk/cgi-bin/nomenclature/searchgenes.pl?field=symbol&anchor=equals&symbol_search=Search&number=100&format=html&sortby=symbol&match=C330016H24RIK) | protein phosphatase inhibitor activity |  | **1.88** |
| 1431777_A_AT | high mobility group nucleosomal binding domain 3 | [HMGN3](http://www.gene.ucl.ac.uk/cgi-bin/nomenclature/searchgenes.pl?field=symbol&anchor=equals&symbol_search=Search&number=100&format=html&sortby=symbol&match=HMGN3) | protein phosphatase inhibitor activity | [604502](http://www.ncbi.nlm.nih.gov/entrez/dispomim.cgi?id=604502) | **-1.98** |
| 1431805_A_AT | rhophilin, Rho GTPase binding protein 2 | [RHPN2](http://www.gene.ucl.ac.uk/cgi-bin/nomenclature/searchgenes.pl?field=symbol&anchor=equals&symbol_search=Search&number=100&format=html&sortby=symbol&match=RHPN2) | protein phosphatase type 2A regulator activity |  | **1.78** |
| 1431928_AT | titin | [TTN](http://www.gene.ucl.ac.uk/cgi-bin/nomenclature/searchgenes.pl?field=symbol&anchor=equals&symbol_search=Search&number=100&format=html&sortby=symbol&match=TTN) | protein prenyltransferase activity | [188840](http://www.ncbi.nlm.nih.gov/entrez/dispomim.cgi?id=188840) | **1.36** |
| 1431929_A_AT | syntaxin 17 | [STX17](http://www.gene.ucl.ac.uk/cgi-bin/nomenclature/searchgenes.pl?field=symbol&anchor=equals&symbol_search=Search&number=100&format=html&sortby=symbol&match=STX17) | protein prenyltransferase activity | [604204](http://www.ncbi.nlm.nih.gov/entrez/dispomim.cgi?id=604204) | **1.31** |
| 1431980_A_AT | arsenic (+3 oxidation state) methyltransferase | [AS3MT](http://www.gene.ucl.ac.uk/cgi-bin/nomenclature/searchgenes.pl?field=symbol&anchor=equals&symbol_search=Search&number=100&format=html&sortby=symbol&match=AS3MT) | protein prenyltransferase activity;asparagine synthase (glutamine-hydrolyzing) activity;transferase activity |  | **1.69** |
| 1432016_A_AT | isocitrate dehydrogenase 3 (NAD+) alpha | [IDH3A](http://www.gene.ucl.ac.uk/cgi-bin/nomenclature/searchgenes.pl?field=symbol&anchor=equals&symbol_search=Search&number=100&format=html&sortby=symbol&match=IDH3A) | protein self binding | [601149](http://www.ncbi.nlm.nih.gov/entrez/dispomim.cgi?id=601149) | **1.35** |
| 1432063_AT | RIKEN cDNA 4833427F10 gene | [4833427F10RIK](http://www.gene.ucl.ac.uk/cgi-bin/nomenclature/searchgenes.pl?field=symbol&anchor=equals&symbol_search=Search&number=100&format=html&sortby=symbol&match=4833427F10RIK) | protein serine/threonine kinase activity |  | **1.58** |
| 1432107_AT | RIKEN cDNA 2310010M20 gene | [2310010M20RIK](http://www.gene.ucl.ac.uk/cgi-bin/nomenclature/searchgenes.pl?field=symbol&anchor=equals&symbol_search=Search&number=100&format=html&sortby=symbol&match=2310010M20RIK) | protein translocase activity |  | **2.58** |
| 1432164_A_AT | RIKEN cDNA 5730591C18 gene | [GCSH](http://www.gene.ucl.ac.uk/cgi-bin/nomenclature/searchgenes.pl?field=symbol&anchor=equals&symbol_search=Search&number=100&format=html&sortby=symbol&match=GCSH) | protein translocase activity | [238330](http://www.ncbi.nlm.nih.gov/entrez/dispomim.cgi?id=238330) | **1.21** |
| 1432195_S_AT | cyclin L2 | [CCNL2](http://www.gene.ucl.ac.uk/cgi-bin/nomenclature/searchgenes.pl?field=symbol&anchor=equals&symbol_search=Search&number=100&format=html&sortby=symbol&match=CCNL2) | protein translocase activity |  | **-1.27** |
| 1432198_AT | gb:AK018172.1 /DB_XREF=gi:12857769 /FEA=mRNA /CNT= |  | protein translocase activity |  | **-6.31** |
| 1432280_AT | RIKEN cDNA 2310007L24 gene | [2310007L24RIK](http://www.gene.ucl.ac.uk/cgi-bin/nomenclature/searchgenes.pl?field=symbol&anchor=equals&symbol_search=Search&number=100&format=html&sortby=symbol&match=2310007L24RIK) | protein translocase activity;oxidoreductase activity;protein transporter activity |  | **1.47** |
| 1432332_A_AT | DNA segment, Chr 7, Roswell Park 2 complex, expressed | [D7RP2E](http://www.gene.ucl.ac.uk/cgi-bin/nomenclature/searchgenes.pl?field=symbol&anchor=equals&symbol_search=Search&number=100&format=html&sortby=symbol&match=D7RP2E) | protein translocase activity;protein transporter activity |  | **1.34** |
| 1432344_A_AT | amyloid beta (A4) precursor-like protein 2 | [APLP2](http://www.gene.ucl.ac.uk/cgi-bin/nomenclature/searchgenes.pl?field=symbol&anchor=equals&symbol_search=Search&number=100&format=html&sortby=symbol&match=APLP2) | protein translocase activity;protein transporter activity | [104776](http://www.ncbi.nlm.nih.gov/entrez/dispomim.cgi?id=104776) | **1.39** |
| 1432358_AT | RIKEN cDNA 1110008I14 gene | [1110008I14RIK](http://www.gene.ucl.ac.uk/cgi-bin/nomenclature/searchgenes.pl?field=symbol&anchor=equals&symbol_search=Search&number=100&format=html&sortby=symbol&match=1110008I14RIK) | protein translocase activity;protein transporter activity |  | **1.93** |
| 1432372_A_AT | sepiapterin reductase | [SPR](http://www.gene.ucl.ac.uk/cgi-bin/nomenclature/searchgenes.pl?field=symbol&anchor=equals&symbol_search=Search&number=100&format=html&sortby=symbol&match=SPR) | protein translocase activity;protein transporter activity | [182125](http://www.ncbi.nlm.nih.gov/entrez/dispomim.cgi?id=182125) | **1.52** |
| 1432385_A_AT | ATP/GTP binding protein 1 | [AGTPBP1](http://www.gene.ucl.ac.uk/cgi-bin/nomenclature/searchgenes.pl?field=symbol&anchor=equals&symbol_search=Search&number=100&format=html&sortby=symbol&match=AGTPBP1) | protein translocase activity;structural constituent of ribosome | [606830](http://www.ncbi.nlm.nih.gov/entrez/dispomim.cgi?id=606830) | **2.67** |
| 1432416_A_AT | nucleophosmin 1 | [NPM1](http://www.gene.ucl.ac.uk/cgi-bin/nomenclature/searchgenes.pl?field=symbol&anchor=equals&symbol_search=Search&number=100&format=html&sortby=symbol&match=NPM1) | protein transporter activity | [164040](http://www.ncbi.nlm.nih.gov/entrez/dispomim.cgi?id=164040) | **-1.49** |
| 1432419_A_AT | RIKEN cDNA 2700078K21 gene | [2700078K21RIK](http://www.gene.ucl.ac.uk/cgi-bin/nomenclature/searchgenes.pl?field=symbol&anchor=equals&symbol_search=Search&number=100&format=html&sortby=symbol&match=2700078K21RIK) | protein transporter activity |  | **1.34** |
| 1432420_A_AT | RIKEN cDNA 2310002L09 gene | [2310002L09RIK](http://www.gene.ucl.ac.uk/cgi-bin/nomenclature/searchgenes.pl?field=symbol&anchor=equals&symbol_search=Search&number=100&format=html&sortby=symbol&match=2310002L09RIK) | protein transporter activity |  | **1.97** |
| 1432431_S_AT | RIKEN cDNA 1110033L15 gene | [1110033L15RIK](http://www.gene.ucl.ac.uk/cgi-bin/nomenclature/searchgenes.pl?field=symbol&anchor=equals&symbol_search=Search&number=100&format=html&sortby=symbol&match=1110033L15RIK) | protein transporter activity |  | **1.48** |
| 1432436_A_AT | adenylate kinase 3 alpha-like 1 | [AK3L1](http://www.gene.ucl.ac.uk/cgi-bin/nomenclature/searchgenes.pl?field=symbol&anchor=equals&symbol_search=Search&number=100&format=html&sortby=symbol&match=AK3L1) | protein transporter activity |  | **1.71** |
| 1432444_A_AT | RIKEN cDNA 1810011O16 gene | [1810011O16RIK](http://www.gene.ucl.ac.uk/cgi-bin/nomenclature/searchgenes.pl?field=symbol&anchor=equals&symbol_search=Search&number=100&format=html&sortby=symbol&match=1810011O16RIK) | protein transporter activity |  | **-1.23** |
| 1432445_AT | RIKEN cDNA 2310016G11 gene | [2310016G11RIK](http://www.gene.ucl.ac.uk/cgi-bin/nomenclature/searchgenes.pl?field=symbol&anchor=equals&symbol_search=Search&number=100&format=html&sortby=symbol&match=2310016G11RIK) | protein transporter activity |  | **2.58** |
| 1432462_A_AT | cofactor required for Sp1 transcriptional activation, subunit 8 | [CRSP8](http://www.gene.ucl.ac.uk/cgi-bin/nomenclature/searchgenes.pl?field=symbol&anchor=equals&symbol_search=Search&number=100&format=html&sortby=symbol&match=CRSP8) | protein transporter activity | [605044](http://www.ncbi.nlm.nih.gov/entrez/dispomim.cgi?id=605044) | **1.28** |
| 1432466_A_AT | apolipoprotein E | [APOE](http://www.gene.ucl.ac.uk/cgi-bin/nomenclature/searchgenes.pl?field=symbol&anchor=equals&symbol_search=Search&number=100&format=html&sortby=symbol&match=APOE) | protein transporter activity | [107741](http://www.ncbi.nlm.nih.gov/entrez/dispomim.cgi?id=107741) | **-4.67** |
| 1432472_A_AT | methylcrotonoyl-Coenzyme A carboxylase 2 (beta) | [MCCC2](http://www.gene.ucl.ac.uk/cgi-bin/nomenclature/searchgenes.pl?field=symbol&anchor=equals&symbol_search=Search&number=100&format=html&sortby=symbol&match=MCCC2) | protein transporter activity | 609014 210210 | **1.65** |
| 1432478_A_AT | IBR domain containing 3 | [IBRDC3](http://www.gene.ucl.ac.uk/cgi-bin/nomenclature/searchgenes.pl?field=symbol&anchor=equals&symbol_search=Search&number=100&format=html&sortby=symbol&match=IBRDC3) | protein transporter activity |  | **-1.76** |
| 1432608_AT | nicotinamide nucleotide transhydrogenase | [NNT](http://www.gene.ucl.ac.uk/cgi-bin/nomenclature/searchgenes.pl?field=symbol&anchor=equals&symbol_search=Search&number=100&format=html&sortby=symbol&match=NNT) | protein transporter activity | [607878](http://www.ncbi.nlm.nih.gov/entrez/dispomim.cgi?id=607878) | **1.68** |
| 1432646_A_AT | RIKEN cDNA 2900097C17 gene | [2900097C17RIK](http://www.gene.ucl.ac.uk/cgi-bin/nomenclature/searchgenes.pl?field=symbol&anchor=equals&symbol_search=Search&number=100&format=html&sortby=symbol&match=2900097C17RIK) | protein transporter activity |  | **1.47** |
| 1432842_S_AT | tyrosine 3-monooxygenase/tryptophan 5-monooxygenase activation protein, theta polypeptide | [YWHAQ](http://www.gene.ucl.ac.uk/cgi-bin/nomenclature/searchgenes.pl?field=symbol&anchor=equals&symbol_search=Search&number=100&format=html&sortby=symbol&match=YWHAQ) | protein transporter activity | [609009](http://www.ncbi.nlm.nih.gov/entrez/dispomim.cgi?id=609009) | **-1.31** |
| 1432913_AT | RIKEN cDNA 4930413P14 gene | [4930413P14RIK](http://www.gene.ucl.ac.uk/cgi-bin/nomenclature/searchgenes.pl?field=symbol&anchor=equals&symbol_search=Search&number=100&format=html&sortby=symbol&match=4930413P14RIK) | protein transporter activity |  | **2.56** |
| 1433398_AT | RIKEN cDNA 5830461L01 gene | [5830461L01RIK](http://www.gene.ucl.ac.uk/cgi-bin/nomenclature/searchgenes.pl?field=symbol&anchor=equals&symbol_search=Search&number=100&format=html&sortby=symbol&match=5830461L01RIK) | protein transporter activity |  | **-2.78** |
| 1433434_AT | expressed sequence AW551984 | [AW551984](http://www.gene.ucl.ac.uk/cgi-bin/nomenclature/searchgenes.pl?field=symbol&anchor=equals&symbol_search=Search&number=100&format=html&sortby=symbol&match=AW551984) | protein transporter activity |  | **-8.36** |
| 1433447_X_AT | chaperonin subunit 4 (delta) | [CCT4](http://www.gene.ucl.ac.uk/cgi-bin/nomenclature/searchgenes.pl?field=symbol&anchor=equals&symbol_search=Search&number=100&format=html&sortby=symbol&match=CCT4) | protein transporter activity | [605142](http://www.ncbi.nlm.nih.gov/entrez/dispomim.cgi?id=605142) | **-1.25** |
| 1433451_AT | cyclin-dependent kinase 5, regulatory subunit (p35) 1 | [CDK5R1](http://www.gene.ucl.ac.uk/cgi-bin/nomenclature/searchgenes.pl?field=symbol&anchor=equals&symbol_search=Search&number=100&format=html&sortby=symbol&match=CDK5R1) | protein-L-isoaspartate (D-aspartate) O-methyltransferase activity;S-adenosylmethionine-dependent methyltransferase activity | [603460](http://www.ncbi.nlm.nih.gov/entrez/dispomim.cgi?id=603460) | **-5.84** |
| 1433457_S_AT | G-rich RNA sequence binding factor 1 | [GRSF1](http://www.gene.ucl.ac.uk/cgi-bin/nomenclature/searchgenes.pl?field=symbol&anchor=equals&symbol_search=Search&number=100&format=html&sortby=symbol&match=GRSF1) | protein-L-isoaspartate (D-aspartate) O-methyltransferase activity;S-adenosylmethionine-dependent methyltransferase activity | [604851](http://www.ncbi.nlm.nih.gov/entrez/dispomim.cgi?id=604851) | **1.26** |
| 1433461_AT | splicing factor 3b, subunit 2 | [SF3B2](http://www.gene.ucl.ac.uk/cgi-bin/nomenclature/searchgenes.pl?field=symbol&anchor=equals&symbol_search=Search&number=100&format=html&sortby=symbol&match=SF3B2) | protein-L-isoaspartate (D-aspartate) O-methyltransferase activity;S-adenosylmethionine-dependent methyltransferase activity | [605591](http://www.ncbi.nlm.nih.gov/entrez/dispomim.cgi?id=605591) | **-1.21** |
| 1433464_AT | importin 13 | [IPO13](http://www.gene.ucl.ac.uk/cgi-bin/nomenclature/searchgenes.pl?field=symbol&anchor=equals&symbol_search=Search&number=100&format=html&sortby=symbol&match=IPO13) | protein-L-isoaspartate (D-aspartate) O-methyltransferase activity;S-adenosylmethionine-dependent methyltransferase activity |  | **1.42** |
| 1433465_A_AT | expressed sequence AI467606 | [AI467606](http://www.gene.ucl.ac.uk/cgi-bin/nomenclature/searchgenes.pl?field=symbol&anchor=equals&symbol_search=Search&number=100&format=html&sortby=symbol&match=AI467606) | protein-L-isoaspartate (D-aspartate) O-methyltransferase activity;S-adenosylmethionine-dependent methyltransferase activity |  | **-1.68** |
| 1433470_A_AT | inner membrane protein, mitochondrial | [IMMT](http://www.gene.ucl.ac.uk/cgi-bin/nomenclature/searchgenes.pl?field=symbol&anchor=equals&symbol_search=Search&number=100&format=html&sortby=symbol&match=IMMT) | protein-methionine-S-oxide reductase activity;oxidoreductase activity | [600378](http://www.ncbi.nlm.nih.gov/entrez/dispomim.cgi?id=600378) | **1.38** |
| 1433476_AT | expressed sequence C78339 | [C78339](http://www.gene.ucl.ac.uk/cgi-bin/nomenclature/searchgenes.pl?field=symbol&anchor=equals&symbol_search=Search&number=100&format=html&sortby=symbol&match=C78339) | protoheme IX farnesyltransferase activity;farnesyltranstransferase activity;transferase activity |  | **-1.25** |
| 1433477_AT | active BCR-related gene | [ABR](http://www.gene.ucl.ac.uk/cgi-bin/nomenclature/searchgenes.pl?field=symbol&anchor=equals&symbol_search=Search&number=100&format=html&sortby=symbol&match=ABR) | purine nucleoside transporter activity;nucleoside:sodium symporter activity | [600365](http://www.ncbi.nlm.nih.gov/entrez/dispomim.cgi?id=600365) | **-1.51** |
| 1433480_AT | RIKEN cDNA 2900010J23 gene | [2900010J23RIK](http://www.gene.ucl.ac.uk/cgi-bin/nomenclature/searchgenes.pl?field=symbol&anchor=equals&symbol_search=Search&number=100&format=html&sortby=symbol&match=2900010J23RIK) | purinergic nucleotide receptor activity, G-protein coupled;chemokine receptor activity;G-protein coupled receptor activity;rhodopsin-like receptor activity;C-C chemokine receptor activity;C-X-C chemokine receptor activity |  | **1.25** |
| 1433481_AT | FK506 binding protein 14 | [FKBP14](http://www.gene.ucl.ac.uk/cgi-bin/nomenclature/searchgenes.pl?field=symbol&anchor=equals&symbol_search=Search&number=100&format=html&sortby=symbol&match=FKBP14) | purinergic nucleotide receptor activity, G-protein coupled;G-protein coupled receptor activity, unknown ligand;chemokine receptor activity;G-protein coupled receptor activity;rhodopsin-like receptor activity |  | **-1.48** |
| 1433482_A_AT | far upstream element (FUSE) binding protein 1 | [FUBP1](http://www.gene.ucl.ac.uk/cgi-bin/nomenclature/searchgenes.pl?field=symbol&anchor=equals&symbol_search=Search&number=100&format=html&sortby=symbol&match=FUBP1) | purinergic nucleotide receptor activity, G-protein coupled;G-protein coupled receptor activity;rhodopsin-like receptor activity | [603444](http://www.ncbi.nlm.nih.gov/entrez/dispomim.cgi?id=603444) | **-1.68** |
| 1433486_AT | chloride channel 3 | [CLCN3](http://www.gene.ucl.ac.uk/cgi-bin/nomenclature/searchgenes.pl?field=symbol&anchor=equals&symbol_search=Search&number=100&format=html&sortby=symbol&match=CLCN3) | purinergic nucleotide receptor activity, G-protein coupled;G-protein coupled receptor activity;rhodopsin-like receptor activity | [600580](http://www.ncbi.nlm.nih.gov/entrez/dispomim.cgi?id=600580) | **1.59** |
| 1433487_AT | chloride channel 3 | [CLCN3](http://www.gene.ucl.ac.uk/cgi-bin/nomenclature/searchgenes.pl?field=symbol&anchor=equals&symbol_search=Search&number=100&format=html&sortby=symbol&match=CLCN3) | purinergic nucleotide receptor activity, G-protein coupled;G-protein coupled receptor activity;rhodopsin-like receptor activity;thrombin receptor activity | [600580](http://www.ncbi.nlm.nih.gov/entrez/dispomim.cgi?id=600580) | **1.47** |
| 1433488_X_AT | gb:BB543167 /DB_XREF=gi:9614390 /DB_XREF=BB543167 |  | purinergic nucleotide receptor activity, G-protein coupled;receptor activity;G-protein coupled receptor activity;rhodopsin-like receptor activity |  | **-1.51** |
| 1433503_AT | gb:BB667090 /DB_XREF=gi:16398539 /DB_XREF=BB667090 |  | purinergic nucleotide receptor activity, G-protein coupled;receptor activity;G-protein coupled receptor activity;rhodopsin-like receptor activity |  | **1.48** |
| 1433513_X_AT | RIKEN cDNA 2410011G03 gene | [2410011G03RIK](http://www.gene.ucl.ac.uk/cgi-bin/nomenclature/searchgenes.pl?field=symbol&anchor=equals&symbol_search=Search&number=100&format=html&sortby=symbol&match=2410011G03RIK) | pyridoxal phosphate binding;ornithine-oxo-acid transaminase activity;transaminase activity;transferase activity |  | **1.33** |
| 1433529_AT | gb:AU067747 /DB_XREF=gi:4968484 /DB_XREF=AU067747 |  | pyruvate carboxylase activity;biotin binding;ligase activity;manganese ion binding;catalytic activity;ATP binding |  | **-1.67** |
| 1433531_AT | acyl-CoA synthetase long-chain family member 4 | [ACSL4](http://www.gene.ucl.ac.uk/cgi-bin/nomenclature/searchgenes.pl?field=symbol&anchor=equals&symbol_search=Search&number=100&format=html&sortby=symbol&match=ACSL4) | pyruvate dehydrogenase (acetyl-transferring) activity;oxidoreductase activity, acting on the aldehyde or oxo group of donors, disulfide as acceptor;oxidoreductase activity;pyruvate dehydrogenase activity | [300157](http://www.ncbi.nlm.nih.gov/entrez/dispomim.cgi?id=300157) | **-1.57** |
| 1433532_A_AT | myelin basic protein | [MBP](http://www.gene.ucl.ac.uk/cgi-bin/nomenclature/searchgenes.pl?field=symbol&anchor=equals&symbol_search=Search&number=100&format=html&sortby=symbol&match=MBP) | pyruvate dehydrogenase (acetyl-transferring) activity;oxidoreductase activity, acting on the aldehyde or oxo group of donors, disulfide as acceptor;oxidoreductase activity;pyruvate dehydrogenase activity | [159430](http://www.ncbi.nlm.nih.gov/entrez/dispomim.cgi?id=159430) | **2.89** |
| 1433535_X_AT | chaperonin subunit 2 (beta) | [CCT2](http://www.gene.ucl.ac.uk/cgi-bin/nomenclature/searchgenes.pl?field=symbol&anchor=equals&symbol_search=Search&number=100&format=html&sortby=symbol&match=CCT2) | pyruvate dehydrogenase (acetyl-transferring) activity;transketolase activity;oxidoreductase activity | [605139](http://www.ncbi.nlm.nih.gov/entrez/dispomim.cgi?id=605139) | **-1.32** |
| 1433536_AT | low density lipoprotein receptor-related protein 11 | [LRP11](http://www.gene.ucl.ac.uk/cgi-bin/nomenclature/searchgenes.pl?field=symbol&anchor=equals&symbol_search=Search&number=100&format=html&sortby=symbol&match=LRP11) | pyruvate dehydrogenase (acetyl-transferring) activity;transketolase activity;oxidoreductase activity |  | **-1.94** |
| 1433537_AT | gb:AV112912 /DB_XREF=gi:16382055 /DB_XREF=AV112912 |  | RAB GDP-dissociation inhibitor activity;GTPase activator activity;Rab GTPase activator activity |  | **-1.24** |
| 1433540_X_AT | protein phosphatase 1, catalytic subunit, beta isoform | [PPP1CB](http://www.gene.ucl.ac.uk/cgi-bin/nomenclature/searchgenes.pl?field=symbol&anchor=equals&symbol_search=Search&number=100&format=html&sortby=symbol&match=PPP1CB) | RAB GDP-dissociation inhibitor activity;GTPase activator activity;Rab GTPase activator activity | [600590](http://www.ncbi.nlm.nih.gov/entrez/dispomim.cgi?id=600590) | **1.26** |
| 1433544_AT | amyotrophic lateral sclerosis 2 (juvenile) chromosome region, candidate 2 homolog (human) | [ALS2CR2](http://www.gene.ucl.ac.uk/cgi-bin/nomenclature/searchgenes.pl?field=symbol&anchor=equals&symbol_search=Search&number=100&format=html&sortby=symbol&match=ALS2CR2) | RAN protein binding;guanyl-nucleotide exchange factor activity | [607333](http://www.ncbi.nlm.nih.gov/entrez/dispomim.cgi?id=607333) | **1.45** |
| 1433545_S_AT | RIKEN cDNA 5730439E10 gene | [5730439E10RIK](http://www.gene.ucl.ac.uk/cgi-bin/nomenclature/searchgenes.pl?field=symbol&anchor=equals&symbol_search=Search&number=100&format=html&sortby=symbol&match=5730439E10RIK) | RAN protein binding;guanyl-nucleotide exchange factor activity;binding |  | **2.16** |
| 1433546_AT | glucosamine (N-acetyl)-6-sulfatase | [GNS](http://www.gene.ucl.ac.uk/cgi-bin/nomenclature/searchgenes.pl?field=symbol&anchor=equals&symbol_search=Search&number=100&format=html&sortby=symbol&match=GNS) | Ras GTPase activator activity;calmodulin binding | [607664](http://www.ncbi.nlm.nih.gov/entrez/dispomim.cgi?id=607664) | **-1.29** |
| 1433549_X_AT | ribosomal protein S21 | [RPS21](http://www.gene.ucl.ac.uk/cgi-bin/nomenclature/searchgenes.pl?field=symbol&anchor=equals&symbol_search=Search&number=100&format=html&sortby=symbol&match=RPS21) | Ras GTPase activator activity;calmodulin binding | [180477](http://www.ncbi.nlm.nih.gov/entrez/dispomim.cgi?id=180477) | **-1.34** |
| 1433550_AT | checkpoint with forkhead and ring finger domains | [CHFR](http://www.gene.ucl.ac.uk/cgi-bin/nomenclature/searchgenes.pl?field=symbol&anchor=equals&symbol_search=Search&number=100&format=html&sortby=symbol&match=CHFR) | Ras GTPase activator activity;calmodulin binding | [605209](http://www.ncbi.nlm.nih.gov/entrez/dispomim.cgi?id=605209) | **-1.32** |
| 1433555_AT | ELL associated factor 1 | [EAF1](http://www.gene.ucl.ac.uk/cgi-bin/nomenclature/searchgenes.pl?field=symbol&anchor=equals&symbol_search=Search&number=100&format=html&sortby=symbol&match=EAF1) | Ras GTPase activator activity;GTPase activator activity | [608315](http://www.ncbi.nlm.nih.gov/entrez/dispomim.cgi?id=608315) | **-1.38** |
| 1433558_AT | disabled homolog 2 (Drosophila) interacting protein | [DAB2IP](http://www.gene.ucl.ac.uk/cgi-bin/nomenclature/searchgenes.pl?field=symbol&anchor=equals&symbol_search=Search&number=100&format=html&sortby=symbol&match=DAB2IP) | Ras GTPase activator activity;GTPase activator activity |  | **1.36** |
| 1433564_AT | Diacylglycerol kinase, delta | [DGKD](http://www.gene.ucl.ac.uk/cgi-bin/nomenclature/searchgenes.pl?field=symbol&anchor=equals&symbol_search=Search&number=100&format=html&sortby=symbol&match=DGKD) | receptor activity | [601826](http://www.ncbi.nlm.nih.gov/entrez/dispomim.cgi?id=601826) | **-1.29** |
| 1433565_AT | RIKEN cDNA 2410002M20 gene | [2410002M20RIK](http://www.gene.ucl.ac.uk/cgi-bin/nomenclature/searchgenes.pl?field=symbol&anchor=equals&symbol_search=Search&number=100&format=html&sortby=symbol&match=2410002M20RIK) | receptor activity |  | **-1.53** |
| 1433571_AT | RIKEN cDNA A130038L21 gene | [A130038L21RIK](http://www.gene.ucl.ac.uk/cgi-bin/nomenclature/searchgenes.pl?field=symbol&anchor=equals&symbol_search=Search&number=100&format=html&sortby=symbol&match=A130038L21RIK) | receptor activity |  | **-2.09** |
| 1433575_AT | SRY-box containing gene 4 | [SOX4](http://www.gene.ucl.ac.uk/cgi-bin/nomenclature/searchgenes.pl?field=symbol&anchor=equals&symbol_search=Search&number=100&format=html&sortby=symbol&match=SOX4) | receptor activity | [184430](http://www.ncbi.nlm.nih.gov/entrez/dispomim.cgi?id=184430) | **-1.92** |
| 1433576_AT | methionine adenosyltransferase II, alpha | [MAT2A](http://www.gene.ucl.ac.uk/cgi-bin/nomenclature/searchgenes.pl?field=symbol&anchor=equals&symbol_search=Search&number=100&format=html&sortby=symbol&match=MAT2A) | receptor activity | [601468](http://www.ncbi.nlm.nih.gov/entrez/dispomim.cgi?id=601468) | **-1.28** |
| 1433579_AT | transmembrane protein 30B | [TMEM30B](http://www.gene.ucl.ac.uk/cgi-bin/nomenclature/searchgenes.pl?field=symbol&anchor=equals&symbol_search=Search&number=100&format=html&sortby=symbol&match=TMEM30B) | receptor activity |  | **-3.5** |
| 1433581_AT | RIKEN cDNA 1190002N15 gene | [1190002N15RIK](http://www.gene.ucl.ac.uk/cgi-bin/nomenclature/searchgenes.pl?field=symbol&anchor=equals&symbol_search=Search&number=100&format=html&sortby=symbol&match=1190002N15RIK) | receptor activity |  | **-1.59** |
| 1433582_AT | RIKEN cDNA 1190002N15 gene | [1190002N15RIK](http://www.gene.ucl.ac.uk/cgi-bin/nomenclature/searchgenes.pl?field=symbol&anchor=equals&symbol_search=Search&number=100&format=html&sortby=symbol&match=1190002N15RIK) | receptor activity |  | **-1.37** |
| 1433583_AT | gb:AV327248 /DB_XREF=gi:16395612 /DB_XREF=AV327248 |  | receptor activity |  | **-3.71** |
| 1433599_AT | cDNA sequence BC065123 | [BC065123](http://www.gene.ucl.ac.uk/cgi-bin/nomenclature/searchgenes.pl?field=symbol&anchor=equals&symbol_search=Search&number=100&format=html&sortby=symbol&match=BC065123) | receptor activity |  | **-2.21** |
| 1433603_AT | CDNA sequence BC059730 | [NDUFS6](http://www.gene.ucl.ac.uk/cgi-bin/nomenclature/searchgenes.pl?field=symbol&anchor=equals&symbol_search=Search&number=100&format=html&sortby=symbol&match=NDUFS6) | receptor activity | [603848](http://www.ncbi.nlm.nih.gov/entrez/dispomim.cgi?id=603848) | **1.39** |
| 1433605_AT | inositol polyphosphate-5-phosphatase A | [INPP5A](http://www.gene.ucl.ac.uk/cgi-bin/nomenclature/searchgenes.pl?field=symbol&anchor=equals&symbol_search=Search&number=100&format=html&sortby=symbol&match=INPP5A) | receptor activity | [600106](http://www.ncbi.nlm.nih.gov/entrez/dispomim.cgi?id=600106) | **1.71** |
| 1433611_S_AT | gb:AV132191 /DB_XREF=gi:16382641 /DB_XREF=AV132191 |  | receptor activity |  | **-1.49** |
| 1433612_AT | adaptor-related protein complex 2, sigma 1 subunit | [AP2S1](http://www.gene.ucl.ac.uk/cgi-bin/nomenclature/searchgenes.pl?field=symbol&anchor=equals&symbol_search=Search&number=100&format=html&sortby=symbol&match=AP2S1) | receptor activity | [602242](http://www.ncbi.nlm.nih.gov/entrez/dispomim.cgi?id=602242) | **-1.4** |
| 1433628_AT | Gene model 1, (NCBI) | [LOC210582](http://www.gene.ucl.ac.uk/cgi-bin/nomenclature/searchgenes.pl?field=symbol&anchor=equals&symbol_search=Search&number=100&format=html&sortby=symbol&match=LOC210582) | receptor activity |  | **1.81** |
| 1433640_AT | gb:BG070740 /DB_XREF=gi:12553309 /DB_XREF=H3090F06 |  | receptor activity |  | **-1.55** |
| 1433652_AT | immunoglobulin superfamily, member 1 | [IGSF1](http://www.gene.ucl.ac.uk/cgi-bin/nomenclature/searchgenes.pl?field=symbol&anchor=equals&symbol_search=Search&number=100&format=html&sortby=symbol&match=IGSF1) | receptor activity | [300137](http://www.ncbi.nlm.nih.gov/entrez/dispomim.cgi?id=300137) | **-1.71** |
| 1433656_A_AT | EST C77032 | [GNL3](http://www.gene.ucl.ac.uk/cgi-bin/nomenclature/searchgenes.pl?field=symbol&anchor=equals&symbol_search=Search&number=100&format=html&sortby=symbol&match=GNL3) | receptor activity |  | **-1.49** |
| 1433661_AT | cDNA sequence BC034204 | [BC034204](http://www.gene.ucl.ac.uk/cgi-bin/nomenclature/searchgenes.pl?field=symbol&anchor=equals&symbol_search=Search&number=100&format=html&sortby=symbol&match=BC034204) | receptor activity |  | **1.24** |
| 1433662_S_AT | tissue inhibitor of metalloproteinase 2 | [TIMP2](http://www.gene.ucl.ac.uk/cgi-bin/nomenclature/searchgenes.pl?field=symbol&anchor=equals&symbol_search=Search&number=100&format=html&sortby=symbol&match=TIMP2) | receptor activity | [188825](http://www.ncbi.nlm.nih.gov/entrez/dispomim.cgi?id=188825) | **-1.21** |
| 1433663_S_AT | Nuclear cap binding protein subunit 1, 80kDa | [LOC433702](http://www.gene.ucl.ac.uk/cgi-bin/nomenclature/searchgenes.pl?field=symbol&anchor=equals&symbol_search=Search&number=100&format=html&sortby=symbol&match=LOC433702) | receptor activity |  | **-1.64** |
| 1433674_A_AT | RNA, U22 small nucleolar | [RNU22](http://www.gene.ucl.ac.uk/cgi-bin/nomenclature/searchgenes.pl?field=symbol&anchor=equals&symbol_search=Search&number=100&format=html&sortby=symbol&match=RNU22) | receptor activity |  | **-1.69** |
| 1433675_AT | RNA, U22 small nucleolar | [RNU22](http://www.gene.ucl.ac.uk/cgi-bin/nomenclature/searchgenes.pl?field=symbol&anchor=equals&symbol_search=Search&number=100&format=html&sortby=symbol&match=RNU22) | receptor activity |  | **-1.53** |
| 1433681_X_AT | calpain 3 | [CAPN3](http://www.gene.ucl.ac.uk/cgi-bin/nomenclature/searchgenes.pl?field=symbol&anchor=equals&symbol_search=Search&number=100&format=html&sortby=symbol&match=CAPN3) | receptor activity | [114240](http://www.ncbi.nlm.nih.gov/entrez/dispomim.cgi?id=114240) | **1.4** |
| 1433688_X_AT | ribosomal protein L14 | [RPL14](http://www.gene.ucl.ac.uk/cgi-bin/nomenclature/searchgenes.pl?field=symbol&anchor=equals&symbol_search=Search&number=100&format=html&sortby=symbol&match=RPL14) | receptor activity |  | **-1.32** |
| 1433695_AT | RIKEN cDNA 3110054C06 gene | [3110054C06RIK](http://www.gene.ucl.ac.uk/cgi-bin/nomenclature/searchgenes.pl?field=symbol&anchor=equals&symbol_search=Search&number=100&format=html&sortby=symbol&match=3110054C06RIK) | receptor activity |  | **-1.64** |
| 1433697_AT | expressed sequence AV312086 | [AV312086](http://www.gene.ucl.ac.uk/cgi-bin/nomenclature/searchgenes.pl?field=symbol&anchor=equals&symbol_search=Search&number=100&format=html&sortby=symbol&match=AV312086) | receptor activity |  | **1.32** |
| 1433700_AT | RIKEN cDNA 4933433P14 gene | [4933433P14RIK](http://www.gene.ucl.ac.uk/cgi-bin/nomenclature/searchgenes.pl?field=symbol&anchor=equals&symbol_search=Search&number=100&format=html&sortby=symbol&match=4933433P14RIK) | receptor activity |  | **-1.23** |
| 1433706_A_AT | expressed sequence AW742319 | [AW742319](http://www.gene.ucl.ac.uk/cgi-bin/nomenclature/searchgenes.pl?field=symbol&anchor=equals&symbol_search=Search&number=100&format=html&sortby=symbol&match=AW742319) | receptor activity |  | **-1.4** |
| 1433719_AT | gb:AI854429 /DB_XREF=gi:5498335 /DB_XREF=UI-M-BH0- |  | receptor activity |  | **-2.11** |
| 1433720_S_AT | Nur77 downstream gene 2 | [NDG2](http://www.gene.ucl.ac.uk/cgi-bin/nomenclature/searchgenes.pl?field=symbol&anchor=equals&symbol_search=Search&number=100&format=html&sortby=symbol&match=NDG2) | receptor activity |  | **1.43** |
| 1433722_AT | RIKEN cDNA 5730522G15 gene | [5730522G15RIK](http://www.gene.ucl.ac.uk/cgi-bin/nomenclature/searchgenes.pl?field=symbol&anchor=equals&symbol_search=Search&number=100&format=html&sortby=symbol&match=5730522G15RIK) | receptor activity |  | **-1.22** |
| 1433724_AT | DNA segment, Chr 15, ERATO Doi 621, expressed | [D15ERTD621E](http://www.gene.ucl.ac.uk/cgi-bin/nomenclature/searchgenes.pl?field=symbol&anchor=equals&symbol_search=Search&number=100&format=html&sortby=symbol&match=D15ERTD621E) | receptor activity |  | **-1.56** |
| 1433746_AT | WD repeat domain 3 | [WDR3](http://www.gene.ucl.ac.uk/cgi-bin/nomenclature/searchgenes.pl?field=symbol&anchor=equals&symbol_search=Search&number=100&format=html&sortby=symbol&match=WDR3) | receptor activity | [604737](http://www.ncbi.nlm.nih.gov/entrez/dispomim.cgi?id=604737) | **-1.49** |
| 1433751_AT | solute carrier family 39 (zinc transporter), member 10 | [SLC39A10](http://www.gene.ucl.ac.uk/cgi-bin/nomenclature/searchgenes.pl?field=symbol&anchor=equals&symbol_search=Search&number=100&format=html&sortby=symbol&match=SLC39A10) | receptor activity | [608733](http://www.ncbi.nlm.nih.gov/entrez/dispomim.cgi?id=608733) | **-1.36** |
| 1433753_X_AT | Era (G-protein)-like 1 (E. coli) | [ERAL1](http://www.gene.ucl.ac.uk/cgi-bin/nomenclature/searchgenes.pl?field=symbol&anchor=equals&symbol_search=Search&number=100&format=html&sortby=symbol&match=ERAL1) | receptor activity | [607435](http://www.ncbi.nlm.nih.gov/entrez/dispomim.cgi?id=607435) | **1.36** |
| 1433754_AT | muscleblind-like 2 | [MBNL2](http://www.gene.ucl.ac.uk/cgi-bin/nomenclature/searchgenes.pl?field=symbol&anchor=equals&symbol_search=Search&number=100&format=html&sortby=symbol&match=MBNL2) | receptor activity | [607327](http://www.ncbi.nlm.nih.gov/entrez/dispomim.cgi?id=607327) | **1.25** |
| 1433756_AT | RIKEN cDNA 4930429A08 gene | [4930429A08RIK](http://www.gene.ucl.ac.uk/cgi-bin/nomenclature/searchgenes.pl?field=symbol&anchor=equals&symbol_search=Search&number=100&format=html&sortby=symbol&match=4930429A08RIK) | receptor activity |  | **-1.37** |
| 1433757_A_AT | nischarin | [NISCH](http://www.gene.ucl.ac.uk/cgi-bin/nomenclature/searchgenes.pl?field=symbol&anchor=equals&symbol_search=Search&number=100&format=html&sortby=symbol&match=NISCH) | receptor activity;chemokine receptor activity;G-protein coupled receptor activity;rhodopsin-like receptor activity;C-C chemokine receptor activity |  | **-1.53** |
| 1433759_AT | RIKEN cDNA 1100001I19 gene | [1100001I19RIK](http://www.gene.ucl.ac.uk/cgi-bin/nomenclature/searchgenes.pl?field=symbol&anchor=equals&symbol_search=Search&number=100&format=html&sortby=symbol&match=1100001I19RIK) | receptor activity;G-protein coupled receptor activity |  | **-1.5** |
| 1433760_A_AT | RIKEN cDNA 5730411O18 gene | [5730411O18RIK](http://www.gene.ucl.ac.uk/cgi-bin/nomenclature/searchgenes.pl?field=symbol&anchor=equals&symbol_search=Search&number=100&format=html&sortby=symbol&match=5730411O18RIK) | receptor activity;G-protein coupled receptor activity |  | **1.26** |
| 1433768_AT | RIKEN cDNA 2410003B16 gene | [2410003B16RIK](http://www.gene.ucl.ac.uk/cgi-bin/nomenclature/searchgenes.pl?field=symbol&anchor=equals&symbol_search=Search&number=100&format=html&sortby=symbol&match=2410003B16RIK) | receptor activity;G-protein coupled receptor activity |  | **-1.58** |
| 1433769_AT | ALS2 C-terminal like | [ALS2CL](http://www.gene.ucl.ac.uk/cgi-bin/nomenclature/searchgenes.pl?field=symbol&anchor=equals&symbol_search=Search&number=100&format=html&sortby=symbol&match=ALS2CL) | receptor activity;G-protein coupled receptor activity;lysosphingolipid and lysophosphatidic acid receptor activity;rhodopsin-like receptor activity |  | **1.47** |
| 1433773_AT | ribonucleotide reductase M2 B (TP53 inducible) | [RRM2B](http://www.gene.ucl.ac.uk/cgi-bin/nomenclature/searchgenes.pl?field=symbol&anchor=equals&symbol_search=Search&number=100&format=html&sortby=symbol&match=RRM2B) | receptor activity;G-protein coupled receptor activity;rhodopsin-like receptor activity | [604712](http://www.ncbi.nlm.nih.gov/entrez/dispomim.cgi?id=604712) | **1.4** |
| 1433776_AT | RIKEN cDNA 2810489O06 gene | [2810489O06RIK](http://www.gene.ucl.ac.uk/cgi-bin/nomenclature/searchgenes.pl?field=symbol&anchor=equals&symbol_search=Search&number=100&format=html&sortby=symbol&match=2810489O06RIK) | receptor activity;G-protein coupled receptor activity;rhodopsin-like receptor activity;protein-hormone receptor activity |  | **-1.54** |
| 1433779_AT | RIKEN cDNA D130060C09 gene | [D130060C09RIK](http://www.gene.ucl.ac.uk/cgi-bin/nomenclature/searchgenes.pl?field=symbol&anchor=equals&symbol_search=Search&number=100&format=html&sortby=symbol&match=D130060C09RIK) | receptor activity;GTPase activator activity |  | **-1.4** |
| 1433806_X_AT | calreticulin | [CALR](http://www.gene.ucl.ac.uk/cgi-bin/nomenclature/searchgenes.pl?field=symbol&anchor=equals&symbol_search=Search&number=100&format=html&sortby=symbol&match=CALR) | receptor activity;guanylate cyclase activity;lyase activity | [109091](http://www.ncbi.nlm.nih.gov/entrez/dispomim.cgi?id=109091) | **-1.21** |
| 1433808_AT | RIKEN cDNA D330001F17 gene | [D330001F17RIK](http://www.gene.ucl.ac.uk/cgi-bin/nomenclature/searchgenes.pl?field=symbol&anchor=equals&symbol_search=Search&number=100&format=html&sortby=symbol&match=D330001F17RIK) | receptor activity;guanylate cyclase activity;lyase activity |  | **1.29** |
| 1433813_AT | gb:BG064731 /DB_XREF=gi:12547294 /DB_XREF=H3023C03 |  | receptor activity;guanylate cyclase activity;lyase activity;calmodulin binding |  | **1.28** |
| 1433824_X_AT | G-rich RNA sequence binding factor 1 | [GRSF1](http://www.gene.ucl.ac.uk/cgi-bin/nomenclature/searchgenes.pl?field=symbol&anchor=equals&symbol_search=Search&number=100&format=html&sortby=symbol&match=GRSF1) | receptor activity;hematopoietin/interferon-class (D200-domain) cytokine receptor activity | [604851](http://www.ncbi.nlm.nih.gov/entrez/dispomim.cgi?id=604851) | **1.27** |
| 1433829_A_AT | heterogeneous nuclear ribonucleoprotein A2/B1 | [HNRPA2B1](http://www.gene.ucl.ac.uk/cgi-bin/nomenclature/searchgenes.pl?field=symbol&anchor=equals&symbol_search=Search&number=100&format=html&sortby=symbol&match=HNRPA2B1) | receptor activity;hematopoietin/interferon-class (D200-domain) cytokine receptor activity | [600124](http://www.ncbi.nlm.nih.gov/entrez/dispomim.cgi?id=600124) | **1.24** |
| 1433833_AT | RIKEN cDNA 1600019O04 gene | [1600019O04RIK](http://www.gene.ucl.ac.uk/cgi-bin/nomenclature/searchgenes.pl?field=symbol&anchor=equals&symbol_search=Search&number=100&format=html&sortby=symbol&match=1600019O04RIK) | receptor activity;hematopoietin/interferon-class (D200-domain) cytokine receptor activity |  | **-1.44** |
| 1433834_AT | RIKEN cDNA F830029L24 gene | [F830029L24RIK](http://www.gene.ucl.ac.uk/cgi-bin/nomenclature/searchgenes.pl?field=symbol&anchor=equals&symbol_search=Search&number=100&format=html&sortby=symbol&match=F830029L24RIK) | receptor activity;integrin binding |  | **1.44** |
| 1433837_AT | RIKEN cDNA 8430408G22 gene | [8430408G22RIK](http://www.gene.ucl.ac.uk/cgi-bin/nomenclature/searchgenes.pl?field=symbol&anchor=equals&symbol_search=Search&number=100&format=html&sortby=symbol&match=8430408G22RIK) | receptor activity;kinase activity;protein serine/threonine kinase activity;ATP binding;transferase activity;protein binding;protein kinase activity;protein-tyrosine kinase activity;signal transducer activity;epidermal growth factor receptor activity;transmembrane receptor protein tyrosine kinase activity |  | **1.89** |
| 1433842_AT | gb:BG069059 /DB_XREF=gi:12551628 /DB_XREF=H3072C08 |  | receptor activity;kinase activity;protein serine/threonine kinase activity;ATP binding;transferase activity;protein binding;protein kinase activity;protein-tyrosine kinase activity;transmembrane receptor protein tyrosine kinase activity |  | **-2.23** |
| 1433850_AT | protein phosphatase 4, regulatory subunit 2 | [PPP4R2](http://www.gene.ucl.ac.uk/cgi-bin/nomenclature/searchgenes.pl?field=symbol&anchor=equals&symbol_search=Search&number=100&format=html&sortby=symbol&match=PPP4R2) | receptor activity;kinase activity;protein serine/threonine kinase activity;ATP binding;transferase activity;protein kinase activity;protein-tyrosine kinase activity;signal transducer activity;G-protein coupled receptor kinase activity |  | **-1.25** |
| 1433852_AT | RIKEN cDNA C330002I19 gene | [C330002I19RIK](http://www.gene.ucl.ac.uk/cgi-bin/nomenclature/searchgenes.pl?field=symbol&anchor=equals&symbol_search=Search&number=100&format=html&sortby=symbol&match=C330002I19RIK) | receptor activity;kinase activity;protein serine/threonine kinase activity;ATP binding;transferase activity;vascular endothelial growth factor receptor activity;protein kinase activity;protein-tyrosine kinase activity;transmembrane receptor protein tyrosine kinase activity |  | **-1.42** |
| 1433857_AT | fat tumor suppressor homolog (Drosophila) | [FATH](http://www.gene.ucl.ac.uk/cgi-bin/nomenclature/searchgenes.pl?field=symbol&anchor=equals&symbol_search=Search&number=100&format=html&sortby=symbol&match=FATH) | receptor activity;kinase activity;protein serine/threonine kinase activity;ATP binding;transferase activity;vascular endothelial growth factor receptor activity;protein kinase activity;protein-tyrosine kinase activity;transmembrane receptor protein tyrosine kinase activity |  | **-1.34** |
| 1433864_AT | low density lipoprotein-related protein 12 | [LRP12](http://www.gene.ucl.ac.uk/cgi-bin/nomenclature/searchgenes.pl?field=symbol&anchor=equals&symbol_search=Search&number=100&format=html&sortby=symbol&match=LRP12) | receptor activity;kinase activity;protein serine/threonine kinase activity;ATP binding;transferase activity;vascular endothelial growth factor receptor activity;protein kinase activity;protein-tyrosine kinase activity;transmembrane receptor protein tyrosine kinase activity |  | **-2.44** |
| 1433873_S_AT | pericentrin 2 | [PCNT2](http://www.gene.ucl.ac.uk/cgi-bin/nomenclature/searchgenes.pl?field=symbol&anchor=equals&symbol_search=Search&number=100&format=html&sortby=symbol&match=PCNT2) | receptor activity;kinase activity;protein serine/threonine kinase activity;ATP binding;transferase activity;vascular endothelial growth factor receptor activity;protein kinase activity;protein-tyrosine kinase activity;transmembrane receptor protein tyrosine kinase activity;platelet-derived growth factor binding | [605925](http://www.ncbi.nlm.nih.gov/entrez/dispomim.cgi?id=605925) | **1.41** |
| 1433875_AT | RIKEN cDNA 4732418C07 gene | [4732418C07RIK](http://www.gene.ucl.ac.uk/cgi-bin/nomenclature/searchgenes.pl?field=symbol&anchor=equals&symbol_search=Search&number=100&format=html&sortby=symbol&match=4732418C07RIK) | receptor activity;kinase activity;transmembrane receptor protein serine/threonine kinase activity;protein serine/threonine kinase activity;ATP binding;transforming growth factor beta receptor activity;transferase activity;receptor signaling protein serine/threonine kinase activity;protein binding;protein kinase activity;protein-tyrosine kinase activity |  | **1.28** |
| 1433879_A_AT | RIKEN cDNA C130032J12 gene | [C130032J12RIK](http://www.gene.ucl.ac.uk/cgi-bin/nomenclature/searchgenes.pl?field=symbol&anchor=equals&symbol_search=Search&number=100&format=html&sortby=symbol&match=C130032J12RIK) | receptor activity;laminin receptor activity;structural constituent of ribosome |  | **-1.23** |
| 1433891_AT | G protein-coupled receptor 48 | [GPR48](http://www.gene.ucl.ac.uk/cgi-bin/nomenclature/searchgenes.pl?field=symbol&anchor=equals&symbol_search=Search&number=100&format=html&sortby=symbol&match=GPR48) | receptor activity;MHC class II receptor activity | [606666](http://www.ncbi.nlm.nih.gov/entrez/dispomim.cgi?id=606666) | **1.73** |
| 1433899_X_AT | transforming growth factor beta 1 induced transcript 4 | [TGFB1I4](http://www.gene.ucl.ac.uk/cgi-bin/nomenclature/searchgenes.pl?field=symbol&anchor=equals&symbol_search=Search&number=100&format=html&sortby=symbol&match=TGFB1I4) | receptor activity;protein carrier activity | [607715](http://www.ncbi.nlm.nih.gov/entrez/dispomim.cgi?id=607715) | **-1.79** |
| 1433904_AT | gametogenetin binding protein 1 | [ZBTB9](http://www.gene.ucl.ac.uk/cgi-bin/nomenclature/searchgenes.pl?field=symbol&anchor=equals&symbol_search=Search&number=100&format=html&sortby=symbol&match=ZBTB9) | receptor activity;protein transporter activity |  | **1.57** |
| 1433907_AT | gb:BQ175824 /DB_XREF=gi:20351316 /DB_XREF=UI-M-DJ2 |  | receptor activity;protein transporter activity;coreceptor, soluble ligand activity |  | **-1.33** |
| 1433908_A_AT | gb:AV009769 /DB_XREF=gi:4786756 /DB_XREF=AV009769 |  | receptor activity;protein transporter activity;coreceptor, soluble ligand activity |  | **-1.74** |
| 1433924_AT | gb:BM200248 /DB_XREF=gi:17753448 /DB_XREF=C0207H01 |  | receptor activity;protein transporter activity;hydrolase activity, acting on glycosyl bonds;hydrolase activity;neurotensin receptor activity, G-protein coupled |  | **-6.74** |
| 1433928_A_AT | gb:BF146301 /DB_XREF=gi:11027696 /DB_XREF=uy27b03. |  | receptor activity;serine-type endopeptidase inhibitor activity |  | **-2.68** |
| 1433951_AT | ADP-ribosylation factor-like 5 | [ARL5](http://www.gene.ucl.ac.uk/cgi-bin/nomenclature/searchgenes.pl?field=symbol&anchor=equals&symbol_search=Search&number=100&format=html&sortby=symbol&match=ARL5) | receptor activity;serine-type endopeptidase inhibitor activity | [608960](http://www.ncbi.nlm.nih.gov/entrez/dispomim.cgi?id=608960) | **-1.23** |
| 1433956_AT | cadherin 5 | [CDH5](http://www.gene.ucl.ac.uk/cgi-bin/nomenclature/searchgenes.pl?field=symbol&anchor=equals&symbol_search=Search&number=100&format=html&sortby=symbol&match=CDH5) | receptor activity;ubiquitin-protein ligase activity | [601120](http://www.ncbi.nlm.nih.gov/entrez/dispomim.cgi?id=601120) | **2.01** |
| 1433963_A_AT | cDNA sequence BC032204 | [BC032204](http://www.gene.ucl.ac.uk/cgi-bin/nomenclature/searchgenes.pl?field=symbol&anchor=equals&symbol_search=Search&number=100&format=html&sortby=symbol&match=BC032204) | receptor binding |  | **-4.21** |
| 1433966_X_AT | asparagine synthetase | [ASNS](http://www.gene.ucl.ac.uk/cgi-bin/nomenclature/searchgenes.pl?field=symbol&anchor=equals&symbol_search=Search&number=100&format=html&sortby=symbol&match=ASNS) | receptor binding | [108370](http://www.ncbi.nlm.nih.gov/entrez/dispomim.cgi?id=108370) | **-2.17** |
| 1433970_AT | RIKEN cDNA 1810056O20 gene | [1810056O20RIK](http://www.gene.ucl.ac.uk/cgi-bin/nomenclature/searchgenes.pl?field=symbol&anchor=equals&symbol_search=Search&number=100&format=html&sortby=symbol&match=1810056O20RIK) | receptor binding;actin binding;actin monomer binding |  | **1.39** |
| 1433971_AT | calmodulin binding transcription activator 1 | [CAMTA1](http://www.gene.ucl.ac.uk/cgi-bin/nomenclature/searchgenes.pl?field=symbol&anchor=equals&symbol_search=Search&number=100&format=html&sortby=symbol&match=CAMTA1) | receptor binding;GTPase activator activity |  | **-1.47** |
| 1433979_AT | RNA binding motif, single stranded interacting protein 2 | [RBMS2](http://www.gene.ucl.ac.uk/cgi-bin/nomenclature/searchgenes.pl?field=symbol&anchor=equals&symbol_search=Search&number=100&format=html&sortby=symbol&match=RBMS2) | receptor binding;hormone activity | [602387](http://www.ncbi.nlm.nih.gov/entrez/dispomim.cgi?id=602387) | **1.2** |
| 1433982_AT | ubiquitin specific protease 28 | [USP28](http://www.gene.ucl.ac.uk/cgi-bin/nomenclature/searchgenes.pl?field=symbol&anchor=equals&symbol_search=Search&number=100&format=html&sortby=symbol&match=USP28) | receptor binding;protein kinase binding |  | **-1.51** |
| 1433984_A_AT | malate dehydrogenase 2, NAD (mitochondrial) | [MDH2](http://www.gene.ucl.ac.uk/cgi-bin/nomenclature/searchgenes.pl?field=symbol&anchor=equals&symbol_search=Search&number=100&format=html&sortby=symbol&match=MDH2) | receptor binding;protein transporter activity | [154100](http://www.ncbi.nlm.nih.gov/entrez/dispomim.cgi?id=154100) | **1.24** |
| 1433986_AT | cDNA sequence BC024659 | [BC024659](http://www.gene.ucl.ac.uk/cgi-bin/nomenclature/searchgenes.pl?field=symbol&anchor=equals&symbol_search=Search&number=100&format=html&sortby=symbol&match=BC024659) | regulator of G-protein signaling activity;GTPase activator activity;signal transducer activity |  | **1.28** |
| 1433993_AT | RIKEN cDNA 4931406P16 gene | [4931406P16RIK](http://www.gene.ucl.ac.uk/cgi-bin/nomenclature/searchgenes.pl?field=symbol&anchor=equals&symbol_search=Search&number=100&format=html&sortby=symbol&match=4931406P16RIK) | retinoid binding;transporter activity;retinol binding;lipid binding;binding |  | **1.31** |
| 1434002_AT | checkpoint supressor 1 | [CHES1](http://www.gene.ucl.ac.uk/cgi-bin/nomenclature/searchgenes.pl?field=symbol&anchor=equals&symbol_search=Search&number=100&format=html&sortby=symbol&match=CHES1) | rhodopsin-like receptor activity | [602628](http://www.ncbi.nlm.nih.gov/entrez/dispomim.cgi?id=602628) | **1.24** |
| 1434008_AT | Sodium channel, type IV, beta polypeptide | [SCN4B](http://www.gene.ucl.ac.uk/cgi-bin/nomenclature/searchgenes.pl?field=symbol&anchor=equals&symbol_search=Search&number=100&format=html&sortby=symbol&match=SCN4B) | ribonucleoprotein binding | [608256](http://www.ncbi.nlm.nih.gov/entrez/dispomim.cgi?id=608256) | **1.66** |
| 1434011_A_AT | RIKEN cDNA 1110055N21 gene | [1110055N21RIK](http://www.gene.ucl.ac.uk/cgi-bin/nomenclature/searchgenes.pl?field=symbol&anchor=equals&symbol_search=Search&number=100&format=html&sortby=symbol&match=1110055N21RIK) | ribonucleoside-diphosphate reductase activity |  | **-1.31** |
| 1434017_AT | DNA segment, Chr 6, ERATO Doi 365, expressed | [D6ERTD365E](http://www.gene.ucl.ac.uk/cgi-bin/nomenclature/searchgenes.pl?field=symbol&anchor=equals&symbol_search=Search&number=100&format=html&sortby=symbol&match=D6ERTD365E) | ribonucleoside-diphosphate reductase activity |  | **1.36** |
| 1434025_AT | gb:BG069607 /DB_XREF=gi:12552176 /DB_XREF=H3078B09 |  | RNA binding |  | **-6.96** |
| 1434037_S_AT | p300/CBP-associated factor | [PCAF](http://www.gene.ucl.ac.uk/cgi-bin/nomenclature/searchgenes.pl?field=symbol&anchor=equals&symbol_search=Search&number=100&format=html&sortby=symbol&match=PCAF) | RNA binding | [602303](http://www.ncbi.nlm.nih.gov/entrez/dispomim.cgi?id=602303) | **1.24** |
| 1434051_S_AT | gb:BQ177161 /DB_XREF=gi:20352653 /DB_XREF=UI-M-DJ2 |  | RNA binding |  | **1.58** |
| 1434057_AT | Gene model 137, (NCBI) | [LOC230075](http://www.gene.ucl.ac.uk/cgi-bin/nomenclature/searchgenes.pl?field=symbol&anchor=equals&symbol_search=Search&number=100&format=html&sortby=symbol&match=LOC230075) | RNA binding |  | **1.32** |
| 1434059_AT | RIKEN cDNA B230312A22 gene | [B230312A22RIK](http://www.gene.ucl.ac.uk/cgi-bin/nomenclature/searchgenes.pl?field=symbol&anchor=equals&symbol_search=Search&number=100&format=html&sortby=symbol&match=B230312A22RIK) | RNA binding |  | **-1.52** |
| 1434067_AT | expressed sequence AI662270 | [AI662270](http://www.gene.ucl.ac.uk/cgi-bin/nomenclature/searchgenes.pl?field=symbol&anchor=equals&symbol_search=Search&number=100&format=html&sortby=symbol&match=AI662270) | RNA binding |  | **-2.88** |
| 1434069_AT | cDNA sequence BC067047 | [BC067047](http://www.gene.ucl.ac.uk/cgi-bin/nomenclature/searchgenes.pl?field=symbol&anchor=equals&symbol_search=Search&number=100&format=html&sortby=symbol&match=BC067047) | RNA binding |  | **-1.69** |
| 1434072_AT | gb:AV299469 /DB_XREF=gi:16393162 /DB_XREF=AV299469 |  | RNA binding |  | **1.25** |
| 1434089_AT | synaptopodin | [SYNPO](http://www.gene.ucl.ac.uk/cgi-bin/nomenclature/searchgenes.pl?field=symbol&anchor=equals&symbol_search=Search&number=100&format=html&sortby=symbol&match=SYNPO) | RNA binding | [608155](http://www.ncbi.nlm.nih.gov/entrez/dispomim.cgi?id=608155) | **-1.64** |
| 1434102_AT | RIKEN cDNA E030026I10 gene | [E030026I10RIK](http://www.gene.ucl.ac.uk/cgi-bin/nomenclature/searchgenes.pl?field=symbol&anchor=equals&symbol_search=Search&number=100&format=html&sortby=symbol&match=E030026I10RIK) | RNA binding |  | **1.3** |
| 1434103_AT | Hypothetical protein 6030458H05 | [AA408278](http://www.gene.ucl.ac.uk/cgi-bin/nomenclature/searchgenes.pl?field=symbol&anchor=equals&symbol_search=Search&number=100&format=html&sortby=symbol&match=AA408278) | RNA binding |  | **1.41** |
| 1434104_AT | hypothetical protein 6030458H05 | [6030458H05](http://www.gene.ucl.ac.uk/cgi-bin/nomenclature/searchgenes.pl?field=symbol&anchor=equals&symbol_search=Search&number=100&format=html&sortby=symbol&match=6030458H05) | RNA binding |  | **1.4** |
| 1434111_AT | latrophilin 2 | [LPHN2](http://www.gene.ucl.ac.uk/cgi-bin/nomenclature/searchgenes.pl?field=symbol&anchor=equals&symbol_search=Search&number=100&format=html&sortby=symbol&match=LPHN2) | RNA binding | [607018](http://www.ncbi.nlm.nih.gov/entrez/dispomim.cgi?id=607018) | **1.39** |
| 1434112_AT | latrophilin 2 | [LPHN2](http://www.gene.ucl.ac.uk/cgi-bin/nomenclature/searchgenes.pl?field=symbol&anchor=equals&symbol_search=Search&number=100&format=html&sortby=symbol&match=LPHN2) | RNA binding | [607018](http://www.ncbi.nlm.nih.gov/entrez/dispomim.cgi?id=607018) | **1.46** |
| 1434113_A_AT | gene model 111, (NCBI) | [GM111](http://www.gene.ucl.ac.uk/cgi-bin/nomenclature/searchgenes.pl?field=symbol&anchor=equals&symbol_search=Search&number=100&format=html&sortby=symbol&match=GM111) | RNA binding | [602930](http://www.ncbi.nlm.nih.gov/entrez/dispomim.cgi?id=602930) | **-1.4** |
| 1434115_AT | cadherin 13 | [CDH13](http://www.gene.ucl.ac.uk/cgi-bin/nomenclature/searchgenes.pl?field=symbol&anchor=equals&symbol_search=Search&number=100&format=html&sortby=symbol&match=CDH13) | RNA binding | [601364](http://www.ncbi.nlm.nih.gov/entrez/dispomim.cgi?id=601364) | **-1.37** |
| 1434117_AT | transcription elongation factor B (SIII), polypeptide 3 | [TCEB3](http://www.gene.ucl.ac.uk/cgi-bin/nomenclature/searchgenes.pl?field=symbol&anchor=equals&symbol_search=Search&number=100&format=html&sortby=symbol&match=TCEB3) | RNA binding | [600786](http://www.ncbi.nlm.nih.gov/entrez/dispomim.cgi?id=600786) | **1.41** |
| 1434129_S_AT | lipoma HMGIC fusion partner-like 2 | [LHFPL2](http://www.gene.ucl.ac.uk/cgi-bin/nomenclature/searchgenes.pl?field=symbol&anchor=equals&symbol_search=Search&number=100&format=html&sortby=symbol&match=LHFPL2) | RNA binding |  | **-2.28** |
| 1434133_S_AT | WD repeat domain 42A | [B230205O20RIK](http://www.gene.ucl.ac.uk/cgi-bin/nomenclature/searchgenes.pl?field=symbol&anchor=equals&symbol_search=Search&number=100&format=html&sortby=symbol&match=B230205O20RIK) | RNA binding |  | **1.29** |
| 1434140_AT | mcf.2 transforming sequence-like | [MCF2L](http://www.gene.ucl.ac.uk/cgi-bin/nomenclature/searchgenes.pl?field=symbol&anchor=equals&symbol_search=Search&number=100&format=html&sortby=symbol&match=MCF2L) | RNA binding |  | **2.61** |
| 1434141_AT | guanylate cyclase 1, soluble, alpha 3 | [GUCY1A3](http://www.gene.ucl.ac.uk/cgi-bin/nomenclature/searchgenes.pl?field=symbol&anchor=equals&symbol_search=Search&number=100&format=html&sortby=symbol&match=GUCY1A3) | RNA binding | [139396](http://www.ncbi.nlm.nih.gov/entrez/dispomim.cgi?id=139396) | **1.59** |
| 1434153_AT | src homology 2 domain-containing transforming protein B | [SHB](http://www.gene.ucl.ac.uk/cgi-bin/nomenclature/searchgenes.pl?field=symbol&anchor=equals&symbol_search=Search&number=100&format=html&sortby=symbol&match=SHB) | RNA binding | [600314](http://www.ncbi.nlm.nih.gov/entrez/dispomim.cgi?id=600314) | **-1.4** |
| 1434155_A_AT | RIKEN cDNA 2310061I04 gene | [2310061I04RIK](http://www.gene.ucl.ac.uk/cgi-bin/nomenclature/searchgenes.pl?field=symbol&anchor=equals&symbol_search=Search&number=100&format=html&sortby=symbol&match=2310061I04RIK) | RNA binding |  | **1.26** |
| 1434159_AT | serine/threonine kinase 4 | [STK4](http://www.gene.ucl.ac.uk/cgi-bin/nomenclature/searchgenes.pl?field=symbol&anchor=equals&symbol_search=Search&number=100&format=html&sortby=symbol&match=STK4) | RNA binding;double-stranded RNA binding | [604965](http://www.ncbi.nlm.nih.gov/entrez/dispomim.cgi?id=604965) | **-1.42** |
| 1434173_S_AT | DNA segment, Chr 19, Brigham & Women's Genetics 1357 expressed | [D19BWG1357E](http://www.gene.ucl.ac.uk/cgi-bin/nomenclature/searchgenes.pl?field=symbol&anchor=equals&symbol_search=Search&number=100&format=html&sortby=symbol&match=D19BWG1357E) | RNA binding;double-stranded RNA binding |  | **-1.58** |
| 1434180_AT | pleckstrin homology domain containing, family C (with FERM domain) member 1 | [PLEKHC1](http://www.gene.ucl.ac.uk/cgi-bin/nomenclature/searchgenes.pl?field=symbol&anchor=equals&symbol_search=Search&number=100&format=html&sortby=symbol&match=PLEKHC1) | RNA binding;GTP binding | [607746](http://www.ncbi.nlm.nih.gov/entrez/dispomim.cgi?id=607746) | **1.21** |
| 1434181_AT | pleckstrin homology domain containing, family C (with FERM domain) member 1 | [PLEKHC1](http://www.gene.ucl.ac.uk/cgi-bin/nomenclature/searchgenes.pl?field=symbol&anchor=equals&symbol_search=Search&number=100&format=html&sortby=symbol&match=PLEKHC1) | RNA binding;GTP binding | [607746](http://www.ncbi.nlm.nih.gov/entrez/dispomim.cgi?id=607746) | **1.31** |
| 1434184_S_AT | RIKEN cDNA 9430080K19 gene | [9430080K19RIK](http://www.gene.ucl.ac.uk/cgi-bin/nomenclature/searchgenes.pl?field=symbol&anchor=equals&symbol_search=Search&number=100&format=html&sortby=symbol&match=9430080K19RIK) | RNA binding;GTP binding |  | **-2.12** |
| 1434185_AT | acetyl-Coenzyme A carboxylase | [ACAC](http://www.gene.ucl.ac.uk/cgi-bin/nomenclature/searchgenes.pl?field=symbol&anchor=equals&symbol_search=Search&number=100&format=html&sortby=symbol&match=ACAC) | RNA binding;heparin binding;structural constituent of ribosome | [200350](http://www.ncbi.nlm.nih.gov/entrez/dispomim.cgi?id=200350) | **1.67** |
| 1434186_AT | G protein-coupled receptor 23 | [GPR23](http://www.gene.ucl.ac.uk/cgi-bin/nomenclature/searchgenes.pl?field=symbol&anchor=equals&symbol_search=Search&number=100&format=html&sortby=symbol&match=GPR23) | RNA binding;heparin binding;structural constituent of ribosome | [300086](http://www.ncbi.nlm.nih.gov/entrez/dispomim.cgi?id=300086) | **-4.52** |
| 1434196_AT | DnaJ (Hsp40) homolog, subfamily A, member 4 | [DNAJA4](http://www.gene.ucl.ac.uk/cgi-bin/nomenclature/searchgenes.pl?field=symbol&anchor=equals&symbol_search=Search&number=100&format=html&sortby=symbol&match=DNAJA4) | RNA binding;ligase activity |  | **1.53** |
| 1434210_S_AT | leucine-rich repeats and immunoglobulin-like domains 1 | [LRIG1](http://www.gene.ucl.ac.uk/cgi-bin/nomenclature/searchgenes.pl?field=symbol&anchor=equals&symbol_search=Search&number=100&format=html&sortby=symbol&match=LRIG1) | RNA binding;pre-mRNA splicing factor activity | [608868](http://www.ncbi.nlm.nih.gov/entrez/dispomim.cgi?id=608868) | **1.52** |
| 1434213_X_AT | NADH dehydrogenase (ubiquinone) Fe-S protein 8 | [NDUFS8](http://www.gene.ucl.ac.uk/cgi-bin/nomenclature/searchgenes.pl?field=symbol&anchor=equals&symbol_search=Search&number=100&format=html&sortby=symbol&match=NDUFS8) | RNA binding;pre-mRNA splicing factor activity | [602141](http://www.ncbi.nlm.nih.gov/entrez/dispomim.cgi?id=602141) | **1.23** |
| 1434216_A_AT | DNA segment, Chr 7, Roswell Park 2 complex, expressed | [D7RP2E](http://www.gene.ucl.ac.uk/cgi-bin/nomenclature/searchgenes.pl?field=symbol&anchor=equals&symbol_search=Search&number=100&format=html&sortby=symbol&match=D7RP2E) | RNA binding;pre-mRNA splicing factor activity |  | **1.3** |
| 1434219_AT | Stromal interaction molecule 2 | [STIM2](http://www.gene.ucl.ac.uk/cgi-bin/nomenclature/searchgenes.pl?field=symbol&anchor=equals&symbol_search=Search&number=100&format=html&sortby=symbol&match=STIM2) | RNA binding;pre-mRNA splicing factor activity;translation repressor activity, nucleic acid binding;BRE binding |  | **-1.47** |
| 1434231_X_AT | ribosomal protein L35 | [RPL35](http://www.gene.ucl.ac.uk/cgi-bin/nomenclature/searchgenes.pl?field=symbol&anchor=equals&symbol_search=Search&number=100&format=html&sortby=symbol&match=RPL35) | RNA binding;ribonuclease activity;ribonuclease P activity;hydrolase activity |  | **-1.41** |
| 1434237_AT | uroplakin 3B | [UPK3B](http://www.gene.ucl.ac.uk/cgi-bin/nomenclature/searchgenes.pl?field=symbol&anchor=equals&symbol_search=Search&number=100&format=html&sortby=symbol&match=UPK3B) | RNA binding;structural constituent of ribosome |  | **2.43** |
| 1434239_AT | expressed sequence AA408556 | [AA408556](http://www.gene.ucl.ac.uk/cgi-bin/nomenclature/searchgenes.pl?field=symbol&anchor=equals&symbol_search=Search&number=100&format=html&sortby=symbol&match=AA408556) | RNA binding;structural constituent of ribosome |  | **-1.94** |
| 1434248_AT | protein kinase C, eta | [PRKCH](http://www.gene.ucl.ac.uk/cgi-bin/nomenclature/searchgenes.pl?field=symbol&anchor=equals&symbol_search=Search&number=100&format=html&sortby=symbol&match=PRKCH) | RNA binding;structural constituent of ribosome | [605437](http://www.ncbi.nlm.nih.gov/entrez/dispomim.cgi?id=605437) | **1.34** |
| 1434252_AT | gb:BG966711 /DB_XREF=gi:14354348 /DB_XREF=60283457 |  | RNA binding;structural constituent of ribosome |  | **1.61** |
| 1434253_S_AT | gb:BB711990 /DB_XREF=gi:16065159 /DB_XREF=BB711990 |  | RNA binding;structural constituent of ribosome |  | **1.51** |
| 1434272_AT | cytoplasmic polyadenylation element binding protein 2 | [CPEB2](http://www.gene.ucl.ac.uk/cgi-bin/nomenclature/searchgenes.pl?field=symbol&anchor=equals&symbol_search=Search&number=100&format=html&sortby=symbol&match=CPEB2) | RNA binding;structural constituent of ribosome |  | **-1.75** |
| 1434281_AT | RIKEN cDNA 1500034J01 gene | [1500034J01RIK](http://www.gene.ucl.ac.uk/cgi-bin/nomenclature/searchgenes.pl?field=symbol&anchor=equals&symbol_search=Search&number=100&format=html&sortby=symbol&match=1500034J01RIK) | RNA binding;structural constituent of ribosome |  | **-1.21** |
| 1434291_A_AT | small EDRK-rich factor 1 | [SERF1](http://www.gene.ucl.ac.uk/cgi-bin/nomenclature/searchgenes.pl?field=symbol&anchor=equals&symbol_search=Search&number=100&format=html&sortby=symbol&match=SERF1) | RNA binding;structural constituent of ribosome |  | **-2.51** |
| 1434301_AT | RIKEN cDNA D330050I23 gene | [D330050I23RIK](http://www.gene.ucl.ac.uk/cgi-bin/nomenclature/searchgenes.pl?field=symbol&anchor=equals&symbol_search=Search&number=100&format=html&sortby=symbol&match=D330050I23RIK) | RNA binding;structural constituent of ribosome |  | **1.56** |
| 1434306_AT | RAB3A interacting protein | [RAB3IP](http://www.gene.ucl.ac.uk/cgi-bin/nomenclature/searchgenes.pl?field=symbol&anchor=equals&symbol_search=Search&number=100&format=html&sortby=symbol&match=RAB3IP) | RNA binding;structural constituent of ribosome;transcription regulator activity | [608686](http://www.ncbi.nlm.nih.gov/entrez/dispomim.cgi?id=608686) | **1.29** |
| 1434312_AT | gb:AA958965 /DB_XREF=gi:3125195 /DB_XREF=ub59g03.s |  | RNA binding;structural constituent of ribosome;transcription regulator activity |  | **-2** |
| 1434316_AT | carbohydrate (chondroitin) synthase 1 | [CHSY1](http://www.gene.ucl.ac.uk/cgi-bin/nomenclature/searchgenes.pl?field=symbol&anchor=equals&symbol_search=Search&number=100&format=html&sortby=symbol&match=CHSY1) | RNA binding;transcription regulator activity;structural constituent of ribosome | [608183](http://www.ncbi.nlm.nih.gov/entrez/dispomim.cgi?id=608183) | **-1.88** |
| 1434320_AT | general transcription factor IIIC, polypeptide 4 | [GTF3C4](http://www.gene.ucl.ac.uk/cgi-bin/nomenclature/searchgenes.pl?field=symbol&anchor=equals&symbol_search=Search&number=100&format=html&sortby=symbol&match=GTF3C4) | RNA methyltransferase activity;methyltransferase activity;RNA binding;transferase activity | [604892](http://www.ncbi.nlm.nih.gov/entrez/dispomim.cgi?id=604892) | **-1.42** |
| 1434329_S_AT | adiponectin receptor 2 | [ADIPOR2](http://www.gene.ucl.ac.uk/cgi-bin/nomenclature/searchgenes.pl?field=symbol&anchor=equals&symbol_search=Search&number=100&format=html&sortby=symbol&match=ADIPOR2) | RNA methyltransferase activity;RNA binding | [607946](http://www.ncbi.nlm.nih.gov/entrez/dispomim.cgi?id=607946) | **1.46** |
| 1434333_A_AT | protein kinase D2 | [PRKD2](http://www.gene.ucl.ac.uk/cgi-bin/nomenclature/searchgenes.pl?field=symbol&anchor=equals&symbol_search=Search&number=100&format=html&sortby=symbol&match=PRKD2) | rRNA binding;5S rRNA binding;structural constituent of ribosome | [607074](http://www.ncbi.nlm.nih.gov/entrez/dispomim.cgi?id=607074) | **1.51** |
| 1434334_AT | protein kinase D2 | [PRKD2](http://www.gene.ucl.ac.uk/cgi-bin/nomenclature/searchgenes.pl?field=symbol&anchor=equals&symbol_search=Search&number=100&format=html&sortby=symbol&match=PRKD2) | rRNA binding;5S rRNA binding;structural constituent of ribosome | [607074](http://www.ncbi.nlm.nih.gov/entrez/dispomim.cgi?id=607074) | **1.78** |
| 1434339_AT | RIKEN cDNA 2610318I01 gene | [FNBP1L](http://www.gene.ucl.ac.uk/cgi-bin/nomenclature/searchgenes.pl?field=symbol&anchor=equals&symbol_search=Search&number=100&format=html&sortby=symbol&match=FNBP1L) | rRNA binding;nucleic acid binding;RNA binding;structural constituent of ribosome | [608848](http://www.ncbi.nlm.nih.gov/entrez/dispomim.cgi?id=608848) | **1.37** |
| 1434341_X_AT | RIKEN cDNA 1110020P15 gene | [1110020P15RIK](http://www.gene.ucl.ac.uk/cgi-bin/nomenclature/searchgenes.pl?field=symbol&anchor=equals&symbol_search=Search&number=100&format=html&sortby=symbol&match=1110020P15RIK) | rRNA binding;nucleic acid binding;RNA binding;structural constituent of ribosome |  | **1.41** |
| 1434348_AT | fasciculation and elongation protein zeta 2 (zygin II) | [FEZ2](http://www.gene.ucl.ac.uk/cgi-bin/nomenclature/searchgenes.pl?field=symbol&anchor=equals&symbol_search=Search&number=100&format=html&sortby=symbol&match=FEZ2) | rRNA binding;protein binding;rRNA primary transcript binding;Pol I transcription termination factor activity;RNA binding | [604826](http://www.ncbi.nlm.nih.gov/entrez/dispomim.cgi?id=604826) | **1.49** |
| 1434354_AT | monoamine oxidase B | [MAOB](http://www.gene.ucl.ac.uk/cgi-bin/nomenclature/searchgenes.pl?field=symbol&anchor=equals&symbol_search=Search&number=100&format=html&sortby=symbol&match=MAOB) | rRNA binding;RNA binding;structural constituent of ribosome | [309860](http://www.ncbi.nlm.nih.gov/entrez/dispomim.cgi?id=309860) | **2.95** |
| 1434356_A_AT | proteasome (prosome, macropain) subunit, alpha type 5 | [PSMA5](http://www.gene.ucl.ac.uk/cgi-bin/nomenclature/searchgenes.pl?field=symbol&anchor=equals&symbol_search=Search&number=100&format=html&sortby=symbol&match=PSMA5) | rRNA methyltransferase activity;rRNA (adenine-N6,N6-)-dimethyltransferase activity | [176844](http://www.ncbi.nlm.nih.gov/entrez/dispomim.cgi?id=176844) | **-1.33** |
| 1434358_X_AT | ribosomal protein S21 | [RPS21](http://www.gene.ucl.ac.uk/cgi-bin/nomenclature/searchgenes.pl?field=symbol&anchor=equals&symbol_search=Search&number=100&format=html&sortby=symbol&match=RPS21) | scavenger receptor activity;receptor activity | [180477](http://www.ncbi.nlm.nih.gov/entrez/dispomim.cgi?id=180477) | **-1.45** |
| 1434360_S_AT | protein tyrosine phosphatase, receptor type, G | [PTPRG](http://www.gene.ucl.ac.uk/cgi-bin/nomenclature/searchgenes.pl?field=symbol&anchor=equals&symbol_search=Search&number=100&format=html&sortby=symbol&match=PTPRG) | scavenger receptor activity;receptor activity | [176886](http://www.ncbi.nlm.nih.gov/entrez/dispomim.cgi?id=176886) | **1.37** |
| 1434366_X_AT | complement component 1, q subcomponent, beta polypeptide | [C1QB](http://www.gene.ucl.ac.uk/cgi-bin/nomenclature/searchgenes.pl?field=symbol&anchor=equals&symbol_search=Search&number=100&format=html&sortby=symbol&match=C1QB) | selenium binding | [120570](http://www.ncbi.nlm.nih.gov/entrez/dispomim.cgi?id=120570) | **-2.17** |
| 1434370_S_AT | Fas-associated factor 1 | [FAF1](http://www.gene.ucl.ac.uk/cgi-bin/nomenclature/searchgenes.pl?field=symbol&anchor=equals&symbol_search=Search&number=100&format=html&sortby=symbol&match=FAF1) | selenium binding | [604460](http://www.ncbi.nlm.nih.gov/entrez/dispomim.cgi?id=604460) | **1.26** |
| 1434376_AT | CD44 antigen | [CD44](http://www.gene.ucl.ac.uk/cgi-bin/nomenclature/searchgenes.pl?field=symbol&anchor=equals&symbol_search=Search&number=100&format=html&sortby=symbol&match=CD44) | selenium binding | [107269](http://www.ncbi.nlm.nih.gov/entrez/dispomim.cgi?id=107269) | **-4.26** |
| 1434378_A_AT | RIKEN cDNA 2810410A03 gene | [MXD4](http://www.gene.ucl.ac.uk/cgi-bin/nomenclature/searchgenes.pl?field=symbol&anchor=equals&symbol_search=Search&number=100&format=html&sortby=symbol&match=MXD4) | selenium binding |  | **-1.51** |
| 1434396_A_AT | myosin, light polypeptide 6, alkali, smooth muscle and non-muscle | [MYL6](http://www.gene.ucl.ac.uk/cgi-bin/nomenclature/searchgenes.pl?field=symbol&anchor=equals&symbol_search=Search&number=100&format=html&sortby=symbol&match=MYL6) | selenium binding;oxidoreductase activity |  | **-1.6** |
| 1434403_AT | sprouty protein with EVH-1 domain 2, related sequence | [SPRED2](http://www.gene.ucl.ac.uk/cgi-bin/nomenclature/searchgenes.pl?field=symbol&anchor=equals&symbol_search=Search&number=100&format=html&sortby=symbol&match=SPRED2) | sepiapterin reductase activity;oxidoreductase activity |  | **1.28** |
| 1434410_AT | cDNA sequence BC043118 | [BC043118](http://www.gene.ucl.ac.uk/cgi-bin/nomenclature/searchgenes.pl?field=symbol&anchor=equals&symbol_search=Search&number=100&format=html&sortby=symbol&match=BC043118) | serine carboxypeptidase activity;carboxypeptidase activity;aminopeptidase activity;catalytic activity;hydrolase activity |  | **1.95** |
| 1434413_AT | gb:BG092677 /DB_XREF=gi:12575240 /DB_XREF=mac15d01 |  | serine carboxypeptidase activity;carboxypeptidase activity;catalytic activity;aminopeptidase activity;hydrolase activity |  | **-1.79** |
| 1434418_AT | gb:BQ176664 /DB_XREF=gi:20352156 /DB_XREF=UI-M-DJ2 |  | serine-type endopeptidase activity;hydrolase activity;peptidase activity;endopeptidase Clp activity |  | **-2.4** |
| 1434436_AT | zinc finger, CW-type with coiled-coil domain 2 | [ZCWCC2](http://www.gene.ucl.ac.uk/cgi-bin/nomenclature/searchgenes.pl?field=symbol&anchor=equals&symbol_search=Search&number=100&format=html&sortby=symbol&match=ZCWCC2) | serine-type endopeptidase inhibitor activity |  | **-2.11** |
| 1434437_X_AT | ribonucleotide reductase M2 | [RRM2](http://www.gene.ucl.ac.uk/cgi-bin/nomenclature/searchgenes.pl?field=symbol&anchor=equals&symbol_search=Search&number=100&format=html&sortby=symbol&match=RRM2) | serine-type endopeptidase inhibitor activity | [180390](http://www.ncbi.nlm.nih.gov/entrez/dispomim.cgi?id=180390) | **-4.12** |
| 1434441_AT | Mus musculus, clone IMAGE:4015738, mRNA |  | serine-type endopeptidase inhibitor activity |  | **1.25** |
| 1434442_AT | DNA segment, Chr 5, ERATO Doi 593, expressed | [D5ERTD593E](http://www.gene.ucl.ac.uk/cgi-bin/nomenclature/searchgenes.pl?field=symbol&anchor=equals&symbol_search=Search&number=100&format=html&sortby=symbol&match=D5ERTD593E) | serine-type endopeptidase inhibitor activity;endopeptidase inhibitor activity;peptidase activity |  | **-2.2** |
| 1434454_AT | gb:BB795229 /DB_XREF=gi:16964848 /DB_XREF=BB795229 |  | serine-type endopeptidase inhibitor activity;endopeptidase inhibitor activity;peptidase activity |  | **1.37** |
| 1434458_AT | gb:BB444134 /DB_XREF=gi:16424930 /DB_XREF=BB444134 |  | serine-type endopeptidase inhibitor activity;endopeptidase inhibitor activity;peptidase activity |  | **-2.98** |
| 1434469_AT | DNA segment, Chr 8, ERATO Doi 69, expressed | [D8ERTD69E](http://www.gene.ucl.ac.uk/cgi-bin/nomenclature/searchgenes.pl?field=symbol&anchor=equals&symbol_search=Search&number=100&format=html&sortby=symbol&match=D8ERTD69E) | serine-type endopeptidase inhibitor activity;endopeptidase inhibitor activity;peptidase activity |  | **1.22** |
| 1434478_AT | RIKEN cDNA 2210037E17 gene | [HECA](http://www.gene.ucl.ac.uk/cgi-bin/nomenclature/searchgenes.pl?field=symbol&anchor=equals&symbol_search=Search&number=100&format=html&sortby=symbol&match=HECA) | serine-type endopeptidase inhibitor activity;extracellular matrix structural constituent conferring tensile strength;endopeptidase inhibitor activity | [607977](http://www.ncbi.nlm.nih.gov/entrez/dispomim.cgi?id=607977) | **1.27** |
| 1434479_AT | gb:AV246911 /DB_XREF=gi:16387161 /DB_XREF=AV246911 |  | serine-type endopeptidase inhibitor activity;heparin binding |  | **-2.86** |
| 1434480_AT | RIKEN cDNA 4930402E16 gene | [4930402E16RIK](http://www.gene.ucl.ac.uk/cgi-bin/nomenclature/searchgenes.pl?field=symbol&anchor=equals&symbol_search=Search&number=100&format=html&sortby=symbol&match=4930402E16RIK) | serine-type endopeptidase inhibitor activity;protease inhibitor activity |  | **1.7** |
| 1434484_AT | RIKEN cDNA 1100001G20 gene | [1100001G20RIK](http://www.gene.ucl.ac.uk/cgi-bin/nomenclature/searchgenes.pl?field=symbol&anchor=equals&symbol_search=Search&number=100&format=html&sortby=symbol&match=1100001G20RIK) | serine-type endopeptidase inhibitor activity;protease inhibitor activity |  | **-6.81** |
| 1434485_A_AT | UDP-glucose pyrophosphorylase 2 | [UGP2](http://www.gene.ucl.ac.uk/cgi-bin/nomenclature/searchgenes.pl?field=symbol&anchor=equals&symbol_search=Search&number=100&format=html&sortby=symbol&match=UGP2) | serine-type peptidase activity;dipeptidyl-peptidase IV activity;aminopeptidase activity;catalytic activity | [191760](http://www.ncbi.nlm.nih.gov/entrez/dispomim.cgi?id=191760) | **1.38** |
| 1434486_X_AT | UDP-glucose pyrophosphorylase 2 | [UGP2](http://www.gene.ucl.ac.uk/cgi-bin/nomenclature/searchgenes.pl?field=symbol&anchor=equals&symbol_search=Search&number=100&format=html&sortby=symbol&match=UGP2) | serine-type peptidase activity;dipeptidyl-peptidase IV activity;catalytic activity;aminopeptidase activity | [191760](http://www.ncbi.nlm.nih.gov/entrez/dispomim.cgi?id=191760) | **1.33** |
| 1434499_A_AT | lactate dehydrogenase 2, B chain | [LDH2](http://www.gene.ucl.ac.uk/cgi-bin/nomenclature/searchgenes.pl?field=symbol&anchor=equals&symbol_search=Search&number=100&format=html&sortby=symbol&match=LDH2) | serine-type peptidase activity;hydrolase activity;peptidase activity |  | **1.52** |
| 1434500_AT | gb:BF585303 /DB_XREF=gi:11658931 /DB_XREF=60210196 |  | serine-type peptidase activity;mitochondrial processing peptidase activity;hydrogen-exporting ATPase activity, phosphorylative mechanism;ATP binding;hydrolase activity;peptidase activity |  | **-1.88** |
| 1434503_S_AT | lysosomal membrane glycoprotein 2 | [LAMP2](http://www.gene.ucl.ac.uk/cgi-bin/nomenclature/searchgenes.pl?field=symbol&anchor=equals&symbol_search=Search&number=100&format=html&sortby=symbol&match=LAMP2) | serine-type peptidase activity;prolyl oligopeptidase activity;catalytic activity;serine-type endopeptidase activity;hydrolase activity;peptidase activity | [309060](http://www.ncbi.nlm.nih.gov/entrez/dispomim.cgi?id=309060) | **-1.92** |
| 1434507_AT | aminopeptidase-like 1 | [NPEPL1](http://www.gene.ucl.ac.uk/cgi-bin/nomenclature/searchgenes.pl?field=symbol&anchor=equals&symbol_search=Search&number=100&format=html&sortby=symbol&match=NPEPL1) | serine-type peptidase activity;prolyl oligopeptidase activity;dipeptidyl-peptidase IV activity;catalytic activity;serine-type endopeptidase activity;hydrolase activity;peptidase activity |  | **1.48** |
| 1434511_AT | phosphorylase kinase beta | [PHKB](http://www.gene.ucl.ac.uk/cgi-bin/nomenclature/searchgenes.pl?field=symbol&anchor=equals&symbol_search=Search&number=100&format=html&sortby=symbol&match=PHKB) | serine-type peptidase activity;subtilase activity;transmembrane receptor protein tyrosine kinase activity;ATP binding;serine-type endopeptidase activity;hydrolase activity;peptidase activity | [172490](http://www.ncbi.nlm.nih.gov/entrez/dispomim.cgi?id=172490) | **1.3** |
| 1434513_AT | RIKEN cDNA A130034M23 gene | [A130034M23RIK](http://www.gene.ucl.ac.uk/cgi-bin/nomenclature/searchgenes.pl?field=symbol&anchor=equals&symbol_search=Search&number=100&format=html&sortby=symbol&match=A130034M23RIK) | SH3/SH2 adaptor protein activity |  | **-2.14** |
| 1434516_AT | phosphoseryl-tRNA kinase | [PSTK](http://www.gene.ucl.ac.uk/cgi-bin/nomenclature/searchgenes.pl?field=symbol&anchor=equals&symbol_search=Search&number=100&format=html&sortby=symbol&match=PSTK) | short-branched-chain-acyl-CoA dehydrogenase activity;oxidoreductase activity;electron carrier activity;acyl-CoA dehydrogenase activity |  | **1.52** |
| 1434517_AT | WD repeat and FYVE domain containing 2 | [WDFY2](http://www.gene.ucl.ac.uk/cgi-bin/nomenclature/searchgenes.pl?field=symbol&anchor=equals&symbol_search=Search&number=100&format=html&sortby=symbol&match=WDFY2) | signal peptidase activity;hydrolase activity;peptidase activity |  | **-1.26** |
| 1434523_X_AT | eukaryotic translation initiation factor 3, subunit 6 | [EIF3S6](http://www.gene.ucl.ac.uk/cgi-bin/nomenclature/searchgenes.pl?field=symbol&anchor=equals&symbol_search=Search&number=100&format=html&sortby=symbol&match=EIF3S6) | signal sequence binding;receptor activity | [602210](http://www.ncbi.nlm.nih.gov/entrez/dispomim.cgi?id=602210) | **-1.21** |
| 1434537_AT | gb:BM237089 /DB_XREF=gi:17872359 /DB_XREF=K0446A08 |  | signal transducer activity |  | **2.32** |
| 1434540_A_AT | clathrin, light polypeptide (Lca) | [CLTA](http://www.gene.ucl.ac.uk/cgi-bin/nomenclature/searchgenes.pl?field=symbol&anchor=equals&symbol_search=Search&number=100&format=html&sortby=symbol&match=CLTA) | signal transducer activity | [118960](http://www.ncbi.nlm.nih.gov/entrez/dispomim.cgi?id=118960) | **-1.54** |
| 1434541_X_AT | gb:BF142112 /DB_XREF=gi:10981152 /DB_XREF=60179192 |  | signal transducer activity |  | **-1.28** |
| 1434542_AT | glutamic pyruvate transaminase (alanine aminotransferase) 2 | [GPT2](http://www.gene.ucl.ac.uk/cgi-bin/nomenclature/searchgenes.pl?field=symbol&anchor=equals&symbol_search=Search&number=100&format=html&sortby=symbol&match=GPT2) | signal transducer activity;GTPase activator activity | [607438](http://www.ncbi.nlm.nih.gov/entrez/dispomim.cgi?id=607438) | **1.78** |
| 1434543_A_AT | RIKEN cDNA 1110025L05 gene | [1110025L05RIK](http://www.gene.ucl.ac.uk/cgi-bin/nomenclature/searchgenes.pl?field=symbol&anchor=equals&symbol_search=Search&number=100&format=html&sortby=symbol&match=1110025L05RIK) | signal transducer activity;GTPase activator activity |  | **-1.9** |
| 1434545_X_AT | RIKEN cDNA 1110025L05 gene | [1110025L05RIK](http://www.gene.ucl.ac.uk/cgi-bin/nomenclature/searchgenes.pl?field=symbol&anchor=equals&symbol_search=Search&number=100&format=html&sortby=symbol&match=1110025L05RIK) | signal transducer activity;GTPase activator activity |  | **-2.44** |
| 1434547_AT | Carboxypeptidase D | [CPD](http://www.gene.ucl.ac.uk/cgi-bin/nomenclature/searchgenes.pl?field=symbol&anchor=equals&symbol_search=Search&number=100&format=html&sortby=symbol&match=CPD) | signal transducer activity;GTPase activator activity | [603102](http://www.ncbi.nlm.nih.gov/entrez/dispomim.cgi?id=603102) | **-1.42** |
| 1434556_AT | gb:BI437942 /DB_XREF=gi:15262632 /DB_XREF=ic82b07. |  | single-stranded DNA binding |  | **1.45** |
| 1434557_AT | huntingtin interacting protein 1 | [HIP1](http://www.gene.ucl.ac.uk/cgi-bin/nomenclature/searchgenes.pl?field=symbol&anchor=equals&symbol_search=Search&number=100&format=html&sortby=symbol&match=HIP1) | single-stranded DNA binding;DNA binding;double-stranded DNA binding;DNA bending activity | [601767](http://www.ncbi.nlm.nih.gov/entrez/dispomim.cgi?id=601767) | **-1.32** |
| 1434560_AT | WD and tetratricopeptide repeats 1 | [WDTC1](http://www.gene.ucl.ac.uk/cgi-bin/nomenclature/searchgenes.pl?field=symbol&anchor=equals&symbol_search=Search&number=100&format=html&sortby=symbol&match=WDTC1) | single-stranded DNA binding;DNA binding;double-stranded DNA binding;DNA bending activity |  | **1.68** |
| 1434565_AT | cell growth regulator with ring finger domain 1 | [CGRRF1](http://www.gene.ucl.ac.uk/cgi-bin/nomenclature/searchgenes.pl?field=symbol&anchor=equals&symbol_search=Search&number=100&format=html&sortby=symbol&match=CGRRF1) | single-stranded DNA binding;DNA binding;transcription regulator activity | [606138](http://www.ncbi.nlm.nih.gov/entrez/dispomim.cgi?id=606138) | **1.27** |
| 1434573_AT | RIKEN cDNA 6030423D04 gene | [6030423D04RIK](http://www.gene.ucl.ac.uk/cgi-bin/nomenclature/searchgenes.pl?field=symbol&anchor=equals&symbol_search=Search&number=100&format=html&sortby=symbol&match=6030423D04RIK) | SMAD binding |  | **-5.77** |
| 1434585_AT | gb:BB667130 /DB_XREF=gi:16398579 /DB_XREF=BB667130 |  | small GTPase regulatory/interacting protein activity |  | **-1.54** |
| 1434611_AT | ring finger protein 123 | [RNF123](http://www.gene.ucl.ac.uk/cgi-bin/nomenclature/searchgenes.pl?field=symbol&anchor=equals&symbol_search=Search&number=100&format=html&sortby=symbol&match=RNF123) | small GTPase regulatory/interacting protein activity;signal transducer activity |  | **1.3** |
| 1434620_S_AT | RIKEN cDNA 2610024E20 gene | [2610024E20RIK](http://www.gene.ucl.ac.uk/cgi-bin/nomenclature/searchgenes.pl?field=symbol&anchor=equals&symbol_search=Search&number=100&format=html&sortby=symbol&match=2610024E20RIK) | small GTPase regulatory/interacting protein activity;signal transducer activity |  | **-1.37** |
| 1434621_AT | cDNA sequence BC054438 | [BC054438](http://www.gene.ucl.ac.uk/cgi-bin/nomenclature/searchgenes.pl?field=symbol&anchor=equals&symbol_search=Search&number=100&format=html&sortby=symbol&match=BC054438) | SNAP receptor activity;protein transporter activity |  | **1.31** |
| 1434624_X_AT | ribosomal protein S9 | [RPS9](http://www.gene.ucl.ac.uk/cgi-bin/nomenclature/searchgenes.pl?field=symbol&anchor=equals&symbol_search=Search&number=100&format=html&sortby=symbol&match=RPS9) | SNAP receptor activity;protein transporter activity;t-SNARE activity | [603631](http://www.ncbi.nlm.nih.gov/entrez/dispomim.cgi?id=603631) | **-1.39** |
| 1434628_A_AT | rhophilin, Rho GTPase binding protein 2 | [RHPN2](http://www.gene.ucl.ac.uk/cgi-bin/nomenclature/searchgenes.pl?field=symbol&anchor=equals&symbol_search=Search&number=100&format=html&sortby=symbol&match=RHPN2) | sodium/potassium-exchanging ATPase activity |  | **1.95** |
| 1434637_X_AT | Transcriptional regulator, SIN3B (yeast) | [SIN3B](http://www.gene.ucl.ac.uk/cgi-bin/nomenclature/searchgenes.pl?field=symbol&anchor=equals&symbol_search=Search&number=100&format=html&sortby=symbol&match=SIN3B) | sodium/potassium-exchanging ATPase activity | [607777](http://www.ncbi.nlm.nih.gov/entrez/dispomim.cgi?id=607777) | **-1.32** |
| 1434642_AT | dehydrogenase/reductase (SDR family) member 8 | [DHRS8](http://www.gene.ucl.ac.uk/cgi-bin/nomenclature/searchgenes.pl?field=symbol&anchor=equals&symbol_search=Search&number=100&format=html&sortby=symbol&match=DHRS8) | sodium/potassium-exchanging ATPase activity |  | **-1.25** |
| 1434647_AT | expressed sequence AU040377 | [AU040377](http://www.gene.ucl.ac.uk/cgi-bin/nomenclature/searchgenes.pl?field=symbol&anchor=equals&symbol_search=Search&number=100&format=html&sortby=symbol&match=AU040377) | sodium/potassium-exchanging ATPase activity |  | **2.27** |
| 1434666_AT | RIKEN cDNA 1110054A01 gene | [1110054A01RIK](http://www.gene.ucl.ac.uk/cgi-bin/nomenclature/searchgenes.pl?field=symbol&anchor=equals&symbol_search=Search&number=100&format=html&sortby=symbol&match=1110054A01RIK) | sodium/potassium-exchanging ATPase activity |  | **1.28** |
| 1434667_AT | procollagen, type VIII, alpha 2 | [COL8A2](http://www.gene.ucl.ac.uk/cgi-bin/nomenclature/searchgenes.pl?field=symbol&anchor=equals&symbol_search=Search&number=100&format=html&sortby=symbol&match=COL8A2) | sodium/potassium-exchanging ATPase activity;ATPase activity, coupled to transmembrane movement of ions, phosphorylative mechanism;magnesium ion binding;cation-transporting ATPase activity;ATP binding;catalytic activity;hydrolase activity;hydrolase activity, acting on acid anhydrides, catalyzing transmembrane movement of substances | [120252](http://www.ncbi.nlm.nih.gov/entrez/dispomim.cgi?id=120252) | **-1.99** |
| 1434671_AT | RIKEN cDNA B230337E12 gene | [B230337E12RIK](http://www.gene.ucl.ac.uk/cgi-bin/nomenclature/searchgenes.pl?field=symbol&anchor=equals&symbol_search=Search&number=100&format=html&sortby=symbol&match=B230337E12RIK) | sodium/potassium-exchanging ATPase activity;hydrogen ion transporter activity |  | **1.37** |
| 1434682_AT | RIKEN cDNA 6430601A21 gene | [6430601A21RIK](http://www.gene.ucl.ac.uk/cgi-bin/nomenclature/searchgenes.pl?field=symbol&anchor=equals&symbol_search=Search&number=100&format=html&sortby=symbol&match=6430601A21RIK) | sodium/potassium-exchanging ATPase activity;protein binding |  | **1.48** |
| 1434694_AT | Leucine-rich repeat-containing 8 | [1700084E18RIK](http://www.gene.ucl.ac.uk/cgi-bin/nomenclature/searchgenes.pl?field=symbol&anchor=equals&symbol_search=Search&number=100&format=html&sortby=symbol&match=1700084E18RIK) | sodium:hydrogen antiporter activity;ion channel activity;solute:hydrogen antiporter activity |  | **1.43** |
| 1434702_AT | RIKEN cDNA 2600009E05 gene | [2600009E05RIK](http://www.gene.ucl.ac.uk/cgi-bin/nomenclature/searchgenes.pl?field=symbol&anchor=equals&symbol_search=Search&number=100&format=html&sortby=symbol&match=2600009E05RIK) | specific transcriptional repressor activity |  | **1.25** |
| 1434717_AT | gb:BM234467 /DB_XREF=gi:17869737 /DB_XREF=K0400F06 |  | spermine synthase activity;catalytic activity;transferase activity |  | **1.71** |
| 1434718_AT | gb:BM198837 /DB_XREF=gi:17751465 /DB_XREF=C0408D11 |  | sterol carrier activity;isomerase activity;3-hydroxyacyl-CoA dehydrogenase activity;oxidoreductase activity;lyase activity;catalytic activity;estradiol 17-beta-dehydrogenase activity |  | **1.53** |
| 1434738_AT | RIKEN cDNA A530046H20 gene | [TARSL2](http://www.gene.ucl.ac.uk/cgi-bin/nomenclature/searchgenes.pl?field=symbol&anchor=equals&symbol_search=Search&number=100&format=html&sortby=symbol&match=TARSL2) | sterol carrier activity;isomerase activity;3-hydroxyacyl-CoA dehydrogenase activity;oxidoreductase activity;lyase activity;catalytic activity;estradiol 17-beta-dehydrogenase activity |  | **1.61** |
| 1434740_AT | scavenger receptor class F, member 2 | [SCARF2](http://www.gene.ucl.ac.uk/cgi-bin/nomenclature/searchgenes.pl?field=symbol&anchor=equals&symbol_search=Search&number=100&format=html&sortby=symbol&match=SCARF2) | sterol carrier activity;oxidoreductase activity |  | **-1.99** |
| 1434745_AT | cyclin D2 | [CCND2](http://www.gene.ucl.ac.uk/cgi-bin/nomenclature/searchgenes.pl?field=symbol&anchor=equals&symbol_search=Search&number=100&format=html&sortby=symbol&match=CCND2) | sterol carrier activity;sterol carrier protein X-related thiolase activity;lipid binding | [123833](http://www.ncbi.nlm.nih.gov/entrez/dispomim.cgi?id=123833) | **1.31** |
| 1434754_AT | GTPase activating RANGAP domain-like 4 | [GARNL4](http://www.gene.ucl.ac.uk/cgi-bin/nomenclature/searchgenes.pl?field=symbol&anchor=equals&symbol_search=Search&number=100&format=html&sortby=symbol&match=GARNL4) | sterol O-acyltransferase activity;acyltransferase activity;transferase activity |  | **-2.58** |
| 1434766_AT | Protein kinase, AMP-activated, alpha 2 catalytic subunit | [PRKAA2](http://www.gene.ucl.ac.uk/cgi-bin/nomenclature/searchgenes.pl?field=symbol&anchor=equals&symbol_search=Search&number=100&format=html&sortby=symbol&match=PRKAA2) | sterol O-acyltransferase activity;acyltransferase activity;transferase activity | [600497](http://www.ncbi.nlm.nih.gov/entrez/dispomim.cgi?id=600497) | **1.38** |
| 1434798_AT | ATPase, H+ transporting, V0 subunit D, isoform 2 | [ATP6V0D2](http://www.gene.ucl.ac.uk/cgi-bin/nomenclature/searchgenes.pl?field=symbol&anchor=equals&symbol_search=Search&number=100&format=html&sortby=symbol&match=ATP6V0D2) | sterol O-acyltransferase activity;acyltransferase activity;transferase activity |  | **-2.52** |
| 1434801_X_AT | solute carrier family 25 (mitochondrial carrier, adenine nucleotide translocator), member 5 | [SLC25A5](http://www.gene.ucl.ac.uk/cgi-bin/nomenclature/searchgenes.pl?field=symbol&anchor=equals&symbol_search=Search&number=100&format=html&sortby=symbol&match=SLC25A5) | strictosidine synthase activity | [300150](http://www.ncbi.nlm.nih.gov/entrez/dispomim.cgi?id=300150) | **-1.87** |
| 1434820_S_AT | protein kinase inhibitor, gamma | [PKIG](http://www.gene.ucl.ac.uk/cgi-bin/nomenclature/searchgenes.pl?field=symbol&anchor=equals&symbol_search=Search&number=100&format=html&sortby=symbol&match=PKIG) | structural constituent of cytoskeleton | [604932](http://www.ncbi.nlm.nih.gov/entrez/dispomim.cgi?id=604932) | **-1.31** |
| 1434828_AT | RIKEN cDNA 1600010D10 gene | [1600010D10RIK](http://www.gene.ucl.ac.uk/cgi-bin/nomenclature/searchgenes.pl?field=symbol&anchor=equals&symbol_search=Search&number=100&format=html&sortby=symbol&match=1600010D10RIK) | structural constituent of cytoskeleton |  | **-1.4** |
| 1434833_AT | mitogen activated protein kinase kinase kinase kinase 2 | [MAP4K2](http://www.gene.ucl.ac.uk/cgi-bin/nomenclature/searchgenes.pl?field=symbol&anchor=equals&symbol_search=Search&number=100&format=html&sortby=symbol&match=MAP4K2) | structural constituent of cytoskeleton | [603166](http://www.ncbi.nlm.nih.gov/entrez/dispomim.cgi?id=603166) | **1.53** |
| 1434846_AT | RIKEN cDNA 1700065A05 gene | [1700065A05RIK](http://www.gene.ucl.ac.uk/cgi-bin/nomenclature/searchgenes.pl?field=symbol&anchor=equals&symbol_search=Search&number=100&format=html&sortby=symbol&match=1700065A05RIK) | structural constituent of cytoskeleton |  | **1.35** |
| 1434854_A_AT | ribosomal protein S10 | [RPS10](http://www.gene.ucl.ac.uk/cgi-bin/nomenclature/searchgenes.pl?field=symbol&anchor=equals&symbol_search=Search&number=100&format=html&sortby=symbol&match=RPS10) | structural constituent of cytoskeleton | [603632](http://www.ncbi.nlm.nih.gov/entrez/dispomim.cgi?id=603632) | **-1.41** |
| 1434855_AT | RIKEN cDNA 5730457F11 gene | [5730457F11RIK](http://www.gene.ucl.ac.uk/cgi-bin/nomenclature/searchgenes.pl?field=symbol&anchor=equals&symbol_search=Search&number=100&format=html&sortby=symbol&match=5730457F11RIK) | structural constituent of cytoskeleton;cysteine-type endopeptidase activity;RNA-directed DNA polymerase activity;transferase activity;peroxidase activity |  | **-1.36** |
| 1434863_AT | gb:AV229170 /DB_XREF=gi:16385625 /DB_XREF=AV229170 |  | structural constituent of eye lens |  | **-1.38** |
| 1434870_AT | RIKEN cDNA 0610039J04 gene | [0610039J04RIK](http://www.gene.ucl.ac.uk/cgi-bin/nomenclature/searchgenes.pl?field=symbol&anchor=equals&symbol_search=Search&number=100&format=html&sortby=symbol&match=0610039J04RIK) | structural constituent of eye lens |  | **1.31** |
| 1434872_X_AT | gb:AV170241 /DB_XREF=gi:5376678 /DB_XREF=AV170241 |  | structural constituent of eye lens |  | **-1.29** |
| 1434875_A_AT | high mobility group nucleosomal binding domain 3 | [HMGN3](http://www.gene.ucl.ac.uk/cgi-bin/nomenclature/searchgenes.pl?field=symbol&anchor=equals&symbol_search=Search&number=100&format=html&sortby=symbol&match=HMGN3) | structural constituent of muscle;structural constituent of cytoskeleton | [604502](http://www.ncbi.nlm.nih.gov/entrez/dispomim.cgi?id=604502) | **-2.82** |
| 1434879_AT | cell division cycle 34 homolog (S. cerevisiae) | [CDC34](http://www.gene.ucl.ac.uk/cgi-bin/nomenclature/searchgenes.pl?field=symbol&anchor=equals&symbol_search=Search&number=100&format=html&sortby=symbol&match=CDC34) | structural constituent of muscle;structural constituent of cytoskeleton | [116948](http://www.ncbi.nlm.nih.gov/entrez/dispomim.cgi?id=116948) | **1.2** |
| 1434882_AT | Metadherin | [MTDH](http://www.gene.ucl.ac.uk/cgi-bin/nomenclature/searchgenes.pl?field=symbol&anchor=equals&symbol_search=Search&number=100&format=html&sortby=symbol&match=MTDH) | structural constituent of ribosome |  | **-1.31** |
| 1434883_AT | Metadherin | [MTDH](http://www.gene.ucl.ac.uk/cgi-bin/nomenclature/searchgenes.pl?field=symbol&anchor=equals&symbol_search=Search&number=100&format=html&sortby=symbol&match=MTDH) | structural constituent of ribosome |  | **-1.33** |
| 1434887_AT | frequenin homolog (Drosophila) | [FREQ](http://www.gene.ucl.ac.uk/cgi-bin/nomenclature/searchgenes.pl?field=symbol&anchor=equals&symbol_search=Search&number=100&format=html&sortby=symbol&match=FREQ) | structural constituent of ribosome | [603315](http://www.ncbi.nlm.nih.gov/entrez/dispomim.cgi?id=603315) | **-4.06** |
| 1434888_A_AT | matrin 3 | [MATR3](http://www.gene.ucl.ac.uk/cgi-bin/nomenclature/searchgenes.pl?field=symbol&anchor=equals&symbol_search=Search&number=100&format=html&sortby=symbol&match=MATR3) | structural constituent of ribosome | [604706](http://www.ncbi.nlm.nih.gov/entrez/dispomim.cgi?id=604706) | **-1.23** |
| 1434890_AT | gb:BG072813 /DB_XREF=gi:12555382 /DB_XREF=H3115C07 |  | structural constituent of ribosome |  | **1.35** |
| 1434891_AT | prostaglandin F2 receptor negative regulator | [PTGFRN](http://www.gene.ucl.ac.uk/cgi-bin/nomenclature/searchgenes.pl?field=symbol&anchor=equals&symbol_search=Search&number=100&format=html&sortby=symbol&match=PTGFRN) | structural constituent of ribosome | [601204](http://www.ncbi.nlm.nih.gov/entrez/dispomim.cgi?id=601204) | **-1.5** |
| 1434892_X_AT | retinoblastoma binding protein 4 | [RBBP4](http://www.gene.ucl.ac.uk/cgi-bin/nomenclature/searchgenes.pl?field=symbol&anchor=equals&symbol_search=Search&number=100&format=html&sortby=symbol&match=RBBP4) | structural constituent of ribosome | [602923](http://www.ncbi.nlm.nih.gov/entrez/dispomim.cgi?id=602923) | **-1.34** |
| 1434893_AT | ATPase, Na+/K+ transporting, alpha 2 polypeptide | [ATP1A2](http://www.gene.ucl.ac.uk/cgi-bin/nomenclature/searchgenes.pl?field=symbol&anchor=equals&symbol_search=Search&number=100&format=html&sortby=symbol&match=ATP1A2) | structural constituent of ribosome | [182340](http://www.ncbi.nlm.nih.gov/entrez/dispomim.cgi?id=182340) | **1.43** |
| 1434895_S_AT | protein phosphatase 1, regulatory (inhibitor) subunit 13B | [PPP1R13B](http://www.gene.ucl.ac.uk/cgi-bin/nomenclature/searchgenes.pl?field=symbol&anchor=equals&symbol_search=Search&number=100&format=html&sortby=symbol&match=PPP1R13B) | structural constituent of ribosome | [606455](http://www.ncbi.nlm.nih.gov/entrez/dispomim.cgi?id=606455) | **1.45** |
| 1434897_A_AT | Solute carrier family 25 (mitochondrial carrier, adenine nucleotide translocator), member 4 | [SLC25A4](http://www.gene.ucl.ac.uk/cgi-bin/nomenclature/searchgenes.pl?field=symbol&anchor=equals&symbol_search=Search&number=100&format=html&sortby=symbol&match=SLC25A4) | structural constituent of ribosome | [103220](http://www.ncbi.nlm.nih.gov/entrez/dispomim.cgi?id=103220) | **1.26** |
| 1434903_S_AT | gb:BG073776 /DB_XREF=gi:12556345 /DB_XREF=H3127A01 |  | structural constituent of ribosome |  | **-1.55** |
| 1434918_AT | SRY-box containing gene 6 | [SOX6](http://www.gene.ucl.ac.uk/cgi-bin/nomenclature/searchgenes.pl?field=symbol&anchor=equals&symbol_search=Search&number=100&format=html&sortby=symbol&match=SOX6) | structural constituent of ribosome | [607257](http://www.ncbi.nlm.nih.gov/entrez/dispomim.cgi?id=607257) | **1.36** |
| 1434920_A_AT | Ena-vasodilator stimulated phosphoprotein | [EVL](http://www.gene.ucl.ac.uk/cgi-bin/nomenclature/searchgenes.pl?field=symbol&anchor=equals&symbol_search=Search&number=100&format=html&sortby=symbol&match=EVL) | structural constituent of ribosome |  | **-1.44** |
| 1434924_AT | PHD finger protein 2 | [PHF2](http://www.gene.ucl.ac.uk/cgi-bin/nomenclature/searchgenes.pl?field=symbol&anchor=equals&symbol_search=Search&number=100&format=html&sortby=symbol&match=PHF2) | structural constituent of ribosome | [604351](http://www.ncbi.nlm.nih.gov/entrez/dispomim.cgi?id=604351) | **1.24** |
| 1434928_AT | growth arrest-specific 2 like 1 | [GAS2L1](http://www.gene.ucl.ac.uk/cgi-bin/nomenclature/searchgenes.pl?field=symbol&anchor=equals&symbol_search=Search&number=100&format=html&sortby=symbol&match=GAS2L1) | structural constituent of ribosome | [602128](http://www.ncbi.nlm.nih.gov/entrez/dispomim.cgi?id=602128) | **-1.32** |
| 1434930_AT | two pore channel 1 | [TPCN1](http://www.gene.ucl.ac.uk/cgi-bin/nomenclature/searchgenes.pl?field=symbol&anchor=equals&symbol_search=Search&number=100&format=html&sortby=symbol&match=TPCN1) | structural constituent of ribosome |  | **1.22** |
| 1434934_AT | ATP synthase mitochondrial F1 complex assembly factor 1 | [ATPAF1](http://www.gene.ucl.ac.uk/cgi-bin/nomenclature/searchgenes.pl?field=symbol&anchor=equals&symbol_search=Search&number=100&format=html&sortby=symbol&match=ATPAF1) | structural constituent of ribosome | [608917](http://www.ncbi.nlm.nih.gov/entrez/dispomim.cgi?id=608917) | **1.92** |
| 1434940_X_AT | regulator of G-protein signaling 19 | [RGS19](http://www.gene.ucl.ac.uk/cgi-bin/nomenclature/searchgenes.pl?field=symbol&anchor=equals&symbol_search=Search&number=100&format=html&sortby=symbol&match=RGS19) | structural constituent of ribosome | [605071](http://www.ncbi.nlm.nih.gov/entrez/dispomim.cgi?id=605071) | **-1.7** |
| 1434944_AT | gb:AW108486 /DB_XREF=gi:6079286 /DB_XREF=um21h05.x |  | structural constituent of ribosome |  | **1.31** |
| 1434945_AT | hypothetical protein A330042H22 | [A330042H22](http://www.gene.ucl.ac.uk/cgi-bin/nomenclature/searchgenes.pl?field=symbol&anchor=equals&symbol_search=Search&number=100&format=html&sortby=symbol&match=A330042H22) | structural constituent of ribosome |  | **-2.01** |
| 1434946_AT | gb:BB303415 /DB_XREF=gi:15411232 /DB_XREF=BB303415 |  | structural constituent of ribosome |  | **2.11** |
| 1434967_AT | zinc finger, SWIM domain containing 6 | [ZSWIM6](http://www.gene.ucl.ac.uk/cgi-bin/nomenclature/searchgenes.pl?field=symbol&anchor=equals&symbol_search=Search&number=100&format=html&sortby=symbol&match=ZSWIM6) | structural constituent of ribosome |  | **-1.49** |
| 1434968_A_AT | ARP3 actin-related protein 3 homolog (yeast) | [ACTR3](http://www.gene.ucl.ac.uk/cgi-bin/nomenclature/searchgenes.pl?field=symbol&anchor=equals&symbol_search=Search&number=100&format=html&sortby=symbol&match=ACTR3) | structural constituent of ribosome | [604222](http://www.ncbi.nlm.nih.gov/entrez/dispomim.cgi?id=604222) | **-1.51** |
| 1434972_X_AT | splicing factor, arginine/serine-rich 1 (ASF/SF2) | [SFRS1](http://www.gene.ucl.ac.uk/cgi-bin/nomenclature/searchgenes.pl?field=symbol&anchor=equals&symbol_search=Search&number=100&format=html&sortby=symbol&match=SFRS1) | structural constituent of ribosome | [600812](http://www.ncbi.nlm.nih.gov/entrez/dispomim.cgi?id=600812) | **-1.24** |
| 1434975_X_AT | similar to phosphatidylserine decarboxylase | [MGC65558](http://www.gene.ucl.ac.uk/cgi-bin/nomenclature/searchgenes.pl?field=symbol&anchor=equals&symbol_search=Search&number=100&format=html&sortby=symbol&match=MGC65558) | structural constituent of ribosome |  | **-2.42** |
| 1434977_AT | RIKEN cDNA 4933403F05 gene | [4933403F05RIK](http://www.gene.ucl.ac.uk/cgi-bin/nomenclature/searchgenes.pl?field=symbol&anchor=equals&symbol_search=Search&number=100&format=html&sortby=symbol&match=4933403F05RIK) | structural constituent of ribosome |  | **1.45** |
| 1434978_AT | RIKEN cDNA 4933403F05 gene | [4933403F05RIK](http://www.gene.ucl.ac.uk/cgi-bin/nomenclature/searchgenes.pl?field=symbol&anchor=equals&symbol_search=Search&number=100&format=html&sortby=symbol&match=4933403F05RIK) | structural constituent of ribosome |  | **1.71** |
| 1434979_AT | RIKEN cDNA 4933403F05 gene | [4933403F05RIK](http://www.gene.ucl.ac.uk/cgi-bin/nomenclature/searchgenes.pl?field=symbol&anchor=equals&symbol_search=Search&number=100&format=html&sortby=symbol&match=4933403F05RIK) | structural constituent of ribosome |  | **1.43** |
| 1434980_AT | gb:AV230647 /DB_XREF=gi:15404968 /DB_XREF=AV230647 |  | structural constituent of ribosome |  | **-1.76** |
| 1434985_A_AT | eukaryotic translation initiation factor 4A1 | [EIF4A1](http://www.gene.ucl.ac.uk/cgi-bin/nomenclature/searchgenes.pl?field=symbol&anchor=equals&symbol_search=Search&number=100&format=html&sortby=symbol&match=EIF4A1) | structural constituent of ribosome | [602641](http://www.ncbi.nlm.nih.gov/entrez/dispomim.cgi?id=602641) | **-1.36** |
| 1434986_A_AT | Sec61 alpha 1 subunit (S. cerevisiae) | [SEC61A1](http://www.gene.ucl.ac.uk/cgi-bin/nomenclature/searchgenes.pl?field=symbol&anchor=equals&symbol_search=Search&number=100&format=html&sortby=symbol&match=SEC61A1) | structural constituent of ribosome |  | **-1.85** |
| 1434998_AT | IQ motif containing GTPase activating protein 1 | [IQGAP1](http://www.gene.ucl.ac.uk/cgi-bin/nomenclature/searchgenes.pl?field=symbol&anchor=equals&symbol_search=Search&number=100&format=html&sortby=symbol&match=IQGAP1) | structural constituent of ribosome | [603379](http://www.ncbi.nlm.nih.gov/entrez/dispomim.cgi?id=603379) | **-1.54** |
| 1435004_AT | pantothenate kinase 4 | [PANK4](http://www.gene.ucl.ac.uk/cgi-bin/nomenclature/searchgenes.pl?field=symbol&anchor=equals&symbol_search=Search&number=100&format=html&sortby=symbol&match=PANK4) | structural constituent of ribosome | [606162](http://www.ncbi.nlm.nih.gov/entrez/dispomim.cgi?id=606162) | **1.3** |
| 1435006_S_AT | ATP-binding cassette, sub-family B (MDR/TAP), member 7 | [ABCB7](http://www.gene.ucl.ac.uk/cgi-bin/nomenclature/searchgenes.pl?field=symbol&anchor=equals&symbol_search=Search&number=100&format=html&sortby=symbol&match=ABCB7) | structural constituent of ribosome | [300135](http://www.ncbi.nlm.nih.gov/entrez/dispomim.cgi?id=300135) | **1.32** |
| 1435011_X_AT | aldo-keto reductase family 1, member A4 (aldehyde reductase) | [AKR1A4](http://www.gene.ucl.ac.uk/cgi-bin/nomenclature/searchgenes.pl?field=symbol&anchor=equals&symbol_search=Search&number=100&format=html&sortby=symbol&match=AKR1A4) | structural constituent of ribosome |  | **-1.35** |
| 1435016_AT | amyotrophic lateral sclerosis 2 (juvenile) chromosome region, candidate 3 homolog(human) | [ALS2CR3](http://www.gene.ucl.ac.uk/cgi-bin/nomenclature/searchgenes.pl?field=symbol&anchor=equals&symbol_search=Search&number=100&format=html&sortby=symbol&match=ALS2CR3) | structural constituent of ribosome | [607334](http://www.ncbi.nlm.nih.gov/entrez/dispomim.cgi?id=607334) | **1.3** |
| 1435026_AT | sparc/osteonectin, cwcv and kazal-like domains proteoglycan 2 | [SPOCK2](http://www.gene.ucl.ac.uk/cgi-bin/nomenclature/searchgenes.pl?field=symbol&anchor=equals&symbol_search=Search&number=100&format=html&sortby=symbol&match=SPOCK2) | structural constituent of ribosome | [607988](http://www.ncbi.nlm.nih.gov/entrez/dispomim.cgi?id=607988) | **1.61** |
| 1435030_AT | UPF2 regulator of nonsense transcripts homolog (yeast) | [UPF2](http://www.gene.ucl.ac.uk/cgi-bin/nomenclature/searchgenes.pl?field=symbol&anchor=equals&symbol_search=Search&number=100&format=html&sortby=symbol&match=UPF2) | structural constituent of ribosome | [605529](http://www.ncbi.nlm.nih.gov/entrez/dispomim.cgi?id=605529) | **1.52** |
| 1435066_AT | phosphatidylinositol transfer protein, cytoplasmic 1 | [PITPNC1](http://www.gene.ucl.ac.uk/cgi-bin/nomenclature/searchgenes.pl?field=symbol&anchor=equals&symbol_search=Search&number=100&format=html&sortby=symbol&match=PITPNC1) | structural constituent of ribosome | [605134](http://www.ncbi.nlm.nih.gov/entrez/dispomim.cgi?id=605134) | **1.66** |
| 1435074_AT | RIKEN cDNA 2310036D22 gene | [2310036D22RIK](http://www.gene.ucl.ac.uk/cgi-bin/nomenclature/searchgenes.pl?field=symbol&anchor=equals&symbol_search=Search&number=100&format=html&sortby=symbol&match=2310036D22RIK) | structural constituent of ribosome |  | **1.21** |
| 1435085_AT | gb:AV234069 /DB_XREF=gi:15405067 /DB_XREF=AV234069 |  | structural constituent of ribosome |  | **1.63** |
| 1435088_AT | gb:BM945177 /DB_XREF=gi:19428762 /DB_XREF=UI-M-EM0 |  | structural constituent of ribosome |  | **-1.25** |
| 1435091_AT | hypothetical LOC243905 | [LOC243905](http://www.gene.ucl.ac.uk/cgi-bin/nomenclature/searchgenes.pl?field=symbol&anchor=equals&symbol_search=Search&number=100&format=html&sortby=symbol&match=LOC243905) | structural constituent of ribosome |  | **-3** |
| 1435096_AT | cDNA sequence BC051080 | [BC051080](http://www.gene.ucl.ac.uk/cgi-bin/nomenclature/searchgenes.pl?field=symbol&anchor=equals&symbol_search=Search&number=100&format=html&sortby=symbol&match=BC051080) | structural constituent of ribosome |  | **1.47** |
| 1435097_AT | methylmalonic aciduria (cobalamin deficiency) type B homolog (human) | [MMAB](http://www.gene.ucl.ac.uk/cgi-bin/nomenclature/searchgenes.pl?field=symbol&anchor=equals&symbol_search=Search&number=100&format=html&sortby=symbol&match=MMAB) | structural constituent of ribosome | [607568](http://www.ncbi.nlm.nih.gov/entrez/dispomim.cgi?id=607568) | **1.48** |
| 1435115_AT | fibronectin type III domain containing 5 | [FNDC5](http://www.gene.ucl.ac.uk/cgi-bin/nomenclature/searchgenes.pl?field=symbol&anchor=equals&symbol_search=Search&number=100&format=html&sortby=symbol&match=FNDC5) | structural constituent of ribosome |  | **1.32** |
| 1435117_A_AT | RIKEN cDNA C330021A05 gene | [C330021A05RIK](http://www.gene.ucl.ac.uk/cgi-bin/nomenclature/searchgenes.pl?field=symbol&anchor=equals&symbol_search=Search&number=100&format=html&sortby=symbol&match=C330021A05RIK) | structural constituent of ribosome |  | **1.92** |
| 1435127_A_AT | O-sialoglycoprotein endopeptidase-like 1 | [OSGEPL1](http://www.gene.ucl.ac.uk/cgi-bin/nomenclature/searchgenes.pl?field=symbol&anchor=equals&symbol_search=Search&number=100&format=html&sortby=symbol&match=OSGEPL1) | structural constituent of ribosome |  | **1.31** |
| 1435133_AT | UDP-glucose ceramide glucosyltransferase | [UGCG](http://www.gene.ucl.ac.uk/cgi-bin/nomenclature/searchgenes.pl?field=symbol&anchor=equals&symbol_search=Search&number=100&format=html&sortby=symbol&match=UGCG) | structural constituent of ribosome | [602874](http://www.ncbi.nlm.nih.gov/entrez/dispomim.cgi?id=602874) | **-1.7** |
| 1435134_AT | RIKEN cDNA B230106I24 gene | [B230106I24RIK](http://www.gene.ucl.ac.uk/cgi-bin/nomenclature/searchgenes.pl?field=symbol&anchor=equals&symbol_search=Search&number=100&format=html&sortby=symbol&match=B230106I24RIK) | structural constituent of ribosome |  | **1.62** |
| 1435144_AT | gb:BM243379 /DB_XREF=gi:17878649 /DB_XREF=K0645H01 |  | structural constituent of ribosome |  | **-4.82** |
| 1435148_AT | ATPase, Na+/K+ transporting, beta 2 polypeptide | [ATP1B2](http://www.gene.ucl.ac.uk/cgi-bin/nomenclature/searchgenes.pl?field=symbol&anchor=equals&symbol_search=Search&number=100&format=html&sortby=symbol&match=ATP1B2) | structural constituent of ribosome | [182331](http://www.ncbi.nlm.nih.gov/entrez/dispomim.cgi?id=182331) | **-2.13** |
| 1435151_A_AT | ribosomal protein S3 | [RPS3](http://www.gene.ucl.ac.uk/cgi-bin/nomenclature/searchgenes.pl?field=symbol&anchor=equals&symbol_search=Search&number=100&format=html&sortby=symbol&match=RPS3) | structural constituent of ribosome | [600454](http://www.ncbi.nlm.nih.gov/entrez/dispomim.cgi?id=600454) | **-1.21** |
| 1435153_AT | BTB (POZ) domain containing 6 | [BTBD6](http://www.gene.ucl.ac.uk/cgi-bin/nomenclature/searchgenes.pl?field=symbol&anchor=equals&symbol_search=Search&number=100&format=html&sortby=symbol&match=BTBD6) | structural constituent of ribosome |  | **1.28** |
| 1435164_S_AT | ubiquitin-activating enzyme E1C | [UBE1C](http://www.gene.ucl.ac.uk/cgi-bin/nomenclature/searchgenes.pl?field=symbol&anchor=equals&symbol_search=Search&number=100&format=html&sortby=symbol&match=UBE1C) | structural constituent of ribosome | [603172](http://www.ncbi.nlm.nih.gov/entrez/dispomim.cgi?id=603172) | **-1.37** |
| 1435176_A_AT | inhibitor of DNA binding 2 | [IDB2](http://www.gene.ucl.ac.uk/cgi-bin/nomenclature/searchgenes.pl?field=symbol&anchor=equals&symbol_search=Search&number=100&format=html&sortby=symbol&match=IDB2) | structural constituent of ribosome |  | **-3.3** |
| 1435184_AT | RIKEN cDNA B430320C24 gene | [B430320C24RIK](http://www.gene.ucl.ac.uk/cgi-bin/nomenclature/searchgenes.pl?field=symbol&anchor=equals&symbol_search=Search&number=100&format=html&sortby=symbol&match=B430320C24RIK) | structural constituent of ribosome |  | **2.46** |
| 1435187_AT | gb:BG064946 /DB_XREF=gi:12547509 /DB_XREF=H3025F06 |  | structural constituent of ribosome |  | **1.31** |
| 1435195_AT | RIKEN cDNA D930046M13 gene | [D930046M13RIK](http://www.gene.ucl.ac.uk/cgi-bin/nomenclature/searchgenes.pl?field=symbol&anchor=equals&symbol_search=Search&number=100&format=html&sortby=symbol&match=D930046M13RIK) | structural constituent of ribosome |  | **-1.26** |
| 1435208_AT | CDNA sequence BC023741 | [DTX3L](http://www.gene.ucl.ac.uk/cgi-bin/nomenclature/searchgenes.pl?field=symbol&anchor=equals&symbol_search=Search&number=100&format=html&sortby=symbol&match=DTX3L) | structural constituent of ribosome |  | **-1.28** |
| 1435213_AT | NHL repeat containing 1 | [NHLRC1](http://www.gene.ucl.ac.uk/cgi-bin/nomenclature/searchgenes.pl?field=symbol&anchor=equals&symbol_search=Search&number=100&format=html&sortby=symbol&match=NHLRC1) | structural constituent of ribosome | [608072](http://www.ncbi.nlm.nih.gov/entrez/dispomim.cgi?id=608072) | **1.51** |
| 1435220_S_AT | CDC42 small effector 2 | [CDC42SE2](http://www.gene.ucl.ac.uk/cgi-bin/nomenclature/searchgenes.pl?field=symbol&anchor=equals&symbol_search=Search&number=100&format=html&sortby=symbol&match=CDC42SE2) | structural constituent of ribosome |  | **-1.48** |
| 1435247_AT | ubiquitin-activating enzyme E1-domain containing 1 | [UBE1DC1](http://www.gene.ucl.ac.uk/cgi-bin/nomenclature/searchgenes.pl?field=symbol&anchor=equals&symbol_search=Search&number=100&format=html&sortby=symbol&match=UBE1DC1) | structural constituent of ribosome |  | **-1.48** |
| 1435248_A_AT | RIKEN cDNA E430027O22 gene | [E430027O22RIK](http://www.gene.ucl.ac.uk/cgi-bin/nomenclature/searchgenes.pl?field=symbol&anchor=equals&symbol_search=Search&number=100&format=html&sortby=symbol&match=E430027O22RIK) | structural constituent of ribosome |  | **-1.4** |
| 1435251_AT | sorting nexin 13 | [SNX13](http://www.gene.ucl.ac.uk/cgi-bin/nomenclature/searchgenes.pl?field=symbol&anchor=equals&symbol_search=Search&number=100&format=html&sortby=symbol&match=SNX13) | structural constituent of ribosome | [606589](http://www.ncbi.nlm.nih.gov/entrez/dispomim.cgi?id=606589) | **1.25** |
| 1435261_AT | gb:BQ174442 /DB_XREF=gi:20349933 /DB_XREF=UI-M-DJ2 |  | structural constituent of ribosome |  | **1.57** |
| 1435262_AT | gb:BB794719 /DB_XREF=gi:16964338 /DB_XREF=BB794719 |  | structural constituent of ribosome |  | **-1.44** |
| 1435272_AT | inositol 1,4,5-trisphosphate 3-kinase B | [ITPKB](http://www.gene.ucl.ac.uk/cgi-bin/nomenclature/searchgenes.pl?field=symbol&anchor=equals&symbol_search=Search&number=100&format=html&sortby=symbol&match=ITPKB) | structural constituent of ribosome | [147522](http://www.ncbi.nlm.nih.gov/entrez/dispomim.cgi?id=147522) | **1.79** |
| 1435280_AT | gb:BB303582 /DB_XREF=gi:16402066 /DB_XREF=BB303582 |  | structural constituent of ribosome |  | **1.47** |
| 1435290_X_AT | histocompatibility 2, class II antigen A, alpha | [H2-AA](http://www.gene.ucl.ac.uk/cgi-bin/nomenclature/searchgenes.pl?field=symbol&anchor=equals&symbol_search=Search&number=100&format=html&sortby=symbol&match=H2-AA) | structural constituent of ribosome |  | **-2.48** |
| 1435292_AT | TBC1 domain family, member 4 | [TBC1D4](http://www.gene.ucl.ac.uk/cgi-bin/nomenclature/searchgenes.pl?field=symbol&anchor=equals&symbol_search=Search&number=100&format=html&sortby=symbol&match=TBC1D4) | structural constituent of ribosome |  | **1.55** |
| 1435313_AT | Cd200 receptor 4 | [CD200R4](http://www.gene.ucl.ac.uk/cgi-bin/nomenclature/searchgenes.pl?field=symbol&anchor=equals&symbol_search=Search&number=100&format=html&sortby=symbol&match=CD200R4) | structural constituent of ribosome |  | **-5.48** |
| 1435322_AT | RIKEN cDNA 1110011C06 gene | [1110011C06RIK](http://www.gene.ucl.ac.uk/cgi-bin/nomenclature/searchgenes.pl?field=symbol&anchor=equals&symbol_search=Search&number=100&format=html&sortby=symbol&match=1110011C06RIK) | structural constituent of ribosome |  | **1.72** |
| 1435325_AT | ubiquitin specific protease 46 | [USP46](http://www.gene.ucl.ac.uk/cgi-bin/nomenclature/searchgenes.pl?field=symbol&anchor=equals&symbol_search=Search&number=100&format=html&sortby=symbol&match=USP46) | structural constituent of ribosome |  | **1.55** |
| 1435327_AT | expressed sequence AW112037 | [AW112037](http://www.gene.ucl.ac.uk/cgi-bin/nomenclature/searchgenes.pl?field=symbol&anchor=equals&symbol_search=Search&number=100&format=html&sortby=symbol&match=AW112037) | structural constituent of ribosome |  | **1.24** |
| 1435328_AT | cysteine and histidine rich 1 | [CYHR1](http://www.gene.ucl.ac.uk/cgi-bin/nomenclature/searchgenes.pl?field=symbol&anchor=equals&symbol_search=Search&number=100&format=html&sortby=symbol&match=CYHR1) | structural constituent of ribosome |  | **1.24** |
| 1435330_AT | expressed sequence AI447904 | [AI447904](http://www.gene.ucl.ac.uk/cgi-bin/nomenclature/searchgenes.pl?field=symbol&anchor=equals&symbol_search=Search&number=100&format=html&sortby=symbol&match=AI447904) | structural constituent of ribosome |  | **-1.57** |
| 1435331_AT | expressed sequence AI447904 | [AI447904](http://www.gene.ucl.ac.uk/cgi-bin/nomenclature/searchgenes.pl?field=symbol&anchor=equals&symbol_search=Search&number=100&format=html&sortby=symbol&match=AI447904) | structural constituent of ribosome;tRNA binding |  | **-4.99** |
| 1435333_AT | gb:AV156568 /DB_XREF=gi:16383280 /DB_XREF=AV156568 |  | structural molecule activity |  | **1.45** |
| 1435338_AT | hypothetical protein 5830411I20 | [5830411I20](http://www.gene.ucl.ac.uk/cgi-bin/nomenclature/searchgenes.pl?field=symbol&anchor=equals&symbol_search=Search&number=100&format=html&sortby=symbol&match=5830411I20) | structural molecule activity |  | **-2.13** |
| 1435342_AT | potassium inwardly-rectifying channel, subfamily K, member 6 | [KCNK6](http://www.gene.ucl.ac.uk/cgi-bin/nomenclature/searchgenes.pl?field=symbol&anchor=equals&symbol_search=Search&number=100&format=html&sortby=symbol&match=KCNK6) | structural molecule activity | [603939](http://www.ncbi.nlm.nih.gov/entrez/dispomim.cgi?id=603939) | **-1.43** |
| 1435343_AT | dedicator of cytokinesis 10 | [DOCK10](http://www.gene.ucl.ac.uk/cgi-bin/nomenclature/searchgenes.pl?field=symbol&anchor=equals&symbol_search=Search&number=100&format=html&sortby=symbol&match=DOCK10) | structural molecule activity |  | **-2.13** |
| 1435344_AT | RIKEN cDNA 1110029I05 gene | [1110029I05RIK](http://www.gene.ucl.ac.uk/cgi-bin/nomenclature/searchgenes.pl?field=symbol&anchor=equals&symbol_search=Search&number=100&format=html&sortby=symbol&match=1110029I05RIK) | structural molecule activity |  | **1.38** |
| 1435345_AT | cerebral endothelial cell adhesion molecule 1 | [CEECAM1](http://www.gene.ucl.ac.uk/cgi-bin/nomenclature/searchgenes.pl?field=symbol&anchor=equals&symbol_search=Search&number=100&format=html&sortby=symbol&match=CEECAM1) | structural molecule activity |  | **-1.62** |
| 1435351_AT | RIKEN cDNA 2310026E23 gene | [2310026E23RIK](http://www.gene.ucl.ac.uk/cgi-bin/nomenclature/searchgenes.pl?field=symbol&anchor=equals&symbol_search=Search&number=100&format=html&sortby=symbol&match=2310026E23RIK) | structural molecule activity |  | **2.16** |
| 1435353_A_AT | RIKEN cDNA 4933439C20 gene | [4933439C20RIK](http://www.gene.ucl.ac.uk/cgi-bin/nomenclature/searchgenes.pl?field=symbol&anchor=equals&symbol_search=Search&number=100&format=html&sortby=symbol&match=4933439C20RIK) | structural molecule activity |  | **-2.05** |
| 1435358_AT | gb:BB400076 /DB_XREF=gi:15411764 /DB_XREF=BB400076 |  | structural molecule activity |  | **1.48** |
| 1435361_AT | cDNA sequence AK129018 | [AK129018](http://www.gene.ucl.ac.uk/cgi-bin/nomenclature/searchgenes.pl?field=symbol&anchor=equals&symbol_search=Search&number=100&format=html&sortby=symbol&match=AK129018) | structural molecule activity |  | **2.14** |
| 1435370_A_AT | carboxylesterase 3 | [CES3](http://www.gene.ucl.ac.uk/cgi-bin/nomenclature/searchgenes.pl?field=symbol&anchor=equals&symbol_search=Search&number=100&format=html&sortby=symbol&match=CES3) | structural molecule activity |  | **2.71** |
| 1435372_A_AT | proliferation-associated 2G4 | [PA2G4](http://www.gene.ucl.ac.uk/cgi-bin/nomenclature/searchgenes.pl?field=symbol&anchor=equals&symbol_search=Search&number=100&format=html&sortby=symbol&match=PA2G4) | structural molecule activity | [602145](http://www.ncbi.nlm.nih.gov/entrez/dispomim.cgi?id=602145) | **-1.42** |
| 1435375_AT | cDNA sequence BC052328 | [BC052328](http://www.gene.ucl.ac.uk/cgi-bin/nomenclature/searchgenes.pl?field=symbol&anchor=equals&symbol_search=Search&number=100&format=html&sortby=symbol&match=BC052328) | structural molecule activity |  | **-2.19** |
| 1435379_AT | cDNA sequence AK122209 | [AK122209](http://www.gene.ucl.ac.uk/cgi-bin/nomenclature/searchgenes.pl?field=symbol&anchor=equals&symbol_search=Search&number=100&format=html&sortby=symbol&match=AK122209) | structural molecule activity |  | **-1.34** |
| 1435380_AT | gb:BG073490 /DB_XREF=gi:12556059 /DB_XREF=H3123B10 |  | structural molecule activity;actin binding;cytoskeletal protein binding;spectrin binding |  | **1.5** |
| 1435386_AT | Von Willebrand factor homolog | [VWF](http://www.gene.ucl.ac.uk/cgi-bin/nomenclature/searchgenes.pl?field=symbol&anchor=equals&symbol_search=Search&number=100&format=html&sortby=symbol&match=VWF) | structural molecule activity;calcium channel activity;ion channel activity;voltage-gated ion channel activity | [193400](http://www.ncbi.nlm.nih.gov/entrez/dispomim.cgi?id=193400) | **2.28** |
| 1435394_S_AT | ras homolog gene family, member C | [PPP2CZ](http://www.gene.ucl.ac.uk/cgi-bin/nomenclature/searchgenes.pl?field=symbol&anchor=equals&symbol_search=Search&number=100&format=html&sortby=symbol&match=PPP2CZ) | structural molecule activity;calmodulin binding | [165380](http://www.ncbi.nlm.nih.gov/entrez/dispomim.cgi?id=165380) | **-1.76** |
| 1435396_AT | gb:BM218509 /DB_XREF=gi:17778051 /DB_XREF=C0912A01 |  | structural molecule activity;calmodulin binding |  | **1.78** |
| 1435397_AT | cDNA sequence BC038156 | [BC038156](http://www.gene.ucl.ac.uk/cgi-bin/nomenclature/searchgenes.pl?field=symbol&anchor=equals&symbol_search=Search&number=100&format=html&sortby=symbol&match=BC038156) | structural molecule activity;cytoskeletal protein binding |  | **1.36** |
| 1435423_X_AT | RIKEN cDNA 4933433P14 gene | [4933433P14RIK](http://www.gene.ucl.ac.uk/cgi-bin/nomenclature/searchgenes.pl?field=symbol&anchor=equals&symbol_search=Search&number=100&format=html&sortby=symbol&match=4933433P14RIK) | structural molecule activity;cytoskeletal protein binding |  | **-1.27** |
| 1435429_X_AT | ribosomal protein S27-like | [RPS27L](http://www.gene.ucl.ac.uk/cgi-bin/nomenclature/searchgenes.pl?field=symbol&anchor=equals&symbol_search=Search&number=100&format=html&sortby=symbol&match=RPS27L) | structural molecule activity;extracellular matrix structural constituent |  | **-1.29** |
| 1435436_AT | gb:BI647951 /DB_XREF=gi:15562187 /DB_XREF=60327603 |  | structural molecule activity;extracellular matrix structural constituent conferring tensile strength |  | **2.09** |
| 1435446_A_AT | choline phosphotransferase 1 | [CHPT1](http://www.gene.ucl.ac.uk/cgi-bin/nomenclature/searchgenes.pl?field=symbol&anchor=equals&symbol_search=Search&number=100&format=html&sortby=symbol&match=CHPT1) | structural molecule activity;extracellular matrix structural constituent conferring tensile strength |  | **2.08** |
| 1435459_AT | flavin containing monooxygenase 2 | [FMO2](http://www.gene.ucl.ac.uk/cgi-bin/nomenclature/searchgenes.pl?field=symbol&anchor=equals&symbol_search=Search&number=100&format=html&sortby=symbol&match=FMO2) | structural molecule activity;extracellular matrix structural constituent conferring tensile strength | [603955](http://www.ncbi.nlm.nih.gov/entrez/dispomim.cgi?id=603955) | **2.54** |
| 1435472_AT | kringle containing transmembrane protein 1 | [KREMEN1](http://www.gene.ucl.ac.uk/cgi-bin/nomenclature/searchgenes.pl?field=symbol&anchor=equals&symbol_search=Search&number=100&format=html&sortby=symbol&match=KREMEN1) | structural molecule activity;extracellular matrix structural constituent;extracellular matrix structural constituent conferring tensile strength |  | **1.42** |
| 1435476_A_AT | Fc receptor, IgG, low affinity IIb | [FCGR2B](http://www.gene.ucl.ac.uk/cgi-bin/nomenclature/searchgenes.pl?field=symbol&anchor=equals&symbol_search=Search&number=100&format=html&sortby=symbol&match=FCGR2B) | structural molecule activity;extracellular matrix structural constituent;extracellular matrix structural constituent conferring tensile strength | [604590](http://www.ncbi.nlm.nih.gov/entrez/dispomim.cgi?id=604590) | **-2.11** |
| 1435477_S_AT | Fc receptor, IgG, low affinity IIb | [FCGR2B](http://www.gene.ucl.ac.uk/cgi-bin/nomenclature/searchgenes.pl?field=symbol&anchor=equals&symbol_search=Search&number=100&format=html&sortby=symbol&match=FCGR2B) | structural molecule activity;extracellular matrix structural constituent;extracellular matrix structural constituent conferring tensile strength | [604590](http://www.ncbi.nlm.nih.gov/entrez/dispomim.cgi?id=604590) | **-3.26** |
| 1435492_AT | RIKEN cDNA 1500012M23 gene | [1500012M23RIK](http://www.gene.ucl.ac.uk/cgi-bin/nomenclature/searchgenes.pl?field=symbol&anchor=equals&symbol_search=Search&number=100&format=html&sortby=symbol&match=1500012M23RIK) | structural molecule activity;extracellular matrix structural constituent;extracellular matrix structural constituent conferring tensile strength |  | **-1.38** |
| 1435504_AT | gb:BM217861 /DB_XREF=gi:17777216 /DB_XREF=C0903B02 |  | structural molecule activity;extracellular matrix structural constituent;extracellular matrix structural constituent conferring tensile strength |  | **1.47** |
| 1435518_AT | RAS related protein 1b | [RAP1B](http://www.gene.ucl.ac.uk/cgi-bin/nomenclature/searchgenes.pl?field=symbol&anchor=equals&symbol_search=Search&number=100&format=html&sortby=symbol&match=RAP1B) | structural molecule activity;extracellular matrix structural constituent;extracellular matrix structural constituent conferring tensile strength | [179530](http://www.ncbi.nlm.nih.gov/entrez/dispomim.cgi?id=179530) | **-1.27** |
| 1435526_AT | hypothetical protein A130072J07 | [A130072J07](http://www.gene.ucl.ac.uk/cgi-bin/nomenclature/searchgenes.pl?field=symbol&anchor=equals&symbol_search=Search&number=100&format=html&sortby=symbol&match=A130072J07) | structural molecule activity;extracellular matrix structural constituent;extracellular matrix structural constituent conferring tensile strength |  | **-1.31** |
| 1435530_AT | Calmodulin regulated spectrin-associated protein 1 | [CAMSAP1](http://www.gene.ucl.ac.uk/cgi-bin/nomenclature/searchgenes.pl?field=symbol&anchor=equals&symbol_search=Search&number=100&format=html&sortby=symbol&match=CAMSAP1) | structural molecule activity;extracellular matrix structural constituent;extracellular matrix structural constituent conferring tensile strength;heparin binding |  | **-1.39** |
| 1435542_S_AT | gb:BB231897 /DB_XREF=gi:16354491 /DB_XREF=BB231897 |  | structural molecule activity;extracellular matrix structural constituent;extracellular matrix structural constituent conferring tensile strength;heparin binding |  | **-1.37** |
| 1435554_AT | gb:BB771888 /DB_XREF=gi:16213579 /DB_XREF=BB771888 |  | structural molecule activity;GTP binding |  | **1.47** |
| 1435567_AT | gb:AI504378 /DB_XREF=gi:4402229 /DB_XREF=vl09b03.x |  | structural molecule activity;GTP binding |  | **1.61** |
| 1435585_AT | RIKEN cDNA 1110018P05 gene | [TCEAL7](http://www.gene.ucl.ac.uk/cgi-bin/nomenclature/searchgenes.pl?field=symbol&anchor=equals&symbol_search=Search&number=100&format=html&sortby=symbol&match=TCEAL7) | structural molecule activity;GTPase activity;GTP binding |  | **-15.87** |
| 1435593_X_AT | ribosomal protein S7 | [RPS7](http://www.gene.ucl.ac.uk/cgi-bin/nomenclature/searchgenes.pl?field=symbol&anchor=equals&symbol_search=Search&number=100&format=html&sortby=symbol&match=RPS7) | structural molecule activity;GTPase activity;GTP binding | [603658](http://www.ncbi.nlm.nih.gov/entrez/dispomim.cgi?id=603658) | **-1.49** |
| 1435594_AT | ADP-ribosylation factor-like 6 interacting protein 2 | [ARL6IP2](http://www.gene.ucl.ac.uk/cgi-bin/nomenclature/searchgenes.pl?field=symbol&anchor=equals&symbol_search=Search&number=100&format=html&sortby=symbol&match=ARL6IP2) | structural molecule activity;GTPase activity;GTP binding;structural constituent of cytoskeleton |  | **1.45** |
| 1435595_AT | RIKEN cDNA 1810011O10 gene | [1810011O10RIK](http://www.gene.ucl.ac.uk/cgi-bin/nomenclature/searchgenes.pl?field=symbol&anchor=equals&symbol_search=Search&number=100&format=html&sortby=symbol&match=1810011O10RIK) | structural molecule activity;GTPase activity;GTP binding;structural constituent of cytoskeleton |  | **2.39** |
| 1435600_S_AT | cDNA sequence BC020535 | [BC020535](http://www.gene.ucl.ac.uk/cgi-bin/nomenclature/searchgenes.pl?field=symbol&anchor=equals&symbol_search=Search&number=100&format=html&sortby=symbol&match=BC020535) | structural molecule activity;GTPase activity;GTP binding;structural constituent of cytoskeleton |  | **1.83** |
| 1435605_AT | actin-related protein 3-beta | [ARP3B](http://www.gene.ucl.ac.uk/cgi-bin/nomenclature/searchgenes.pl?field=symbol&anchor=equals&symbol_search=Search&number=100&format=html&sortby=symbol&match=ARP3B) | structural molecule activity;GTPase activity;GTP binding;structural constituent of cytoskeleton |  | **3.01** |
| 1435615_AT | zinc finger protein 365 | [ZFP365](http://www.gene.ucl.ac.uk/cgi-bin/nomenclature/searchgenes.pl?field=symbol&anchor=equals&symbol_search=Search&number=100&format=html&sortby=symbol&match=ZFP365) | structural molecule activity;GTPase activity;GTP binding;structural constituent of cytoskeleton |  | **-7.02** |
| 1435631_X_AT | SEC15-like 1 (S. cerevisiae) | [SEC15L1](http://www.gene.ucl.ac.uk/cgi-bin/nomenclature/searchgenes.pl?field=symbol&anchor=equals&symbol_search=Search&number=100&format=html&sortby=symbol&match=SEC15L1) | structural molecule activity;GTPase activity;GTP binding;structural constituent of cytoskeleton |  | **1.34** |
| 1435632_AT | RIKEN cDNA 1110001M19 gene | [1110001M19RIK](http://www.gene.ucl.ac.uk/cgi-bin/nomenclature/searchgenes.pl?field=symbol&anchor=equals&symbol_search=Search&number=100&format=html&sortby=symbol&match=1110001M19RIK) | structural molecule activity;GTPase activity;GTP binding;structural constituent of cytoskeleton |  | **-1.27** |
| 1435634_AT | RIKEN cDNA A030012M09 gene | [A030012M09RIK](http://www.gene.ucl.ac.uk/cgi-bin/nomenclature/searchgenes.pl?field=symbol&anchor=equals&symbol_search=Search&number=100&format=html&sortby=symbol&match=A030012M09RIK) | structural molecule activity;kinase activity;protein kinase activity;protein serine/threonine kinase activity;receptor activity;ATP binding;protein-tyrosine kinase activity;transferase activity |  | **1.38** |
| 1435635_AT | RIKEN cDNA A030012M09 gene | [A030012M09RIK](http://www.gene.ucl.ac.uk/cgi-bin/nomenclature/searchgenes.pl?field=symbol&anchor=equals&symbol_search=Search&number=100&format=html&sortby=symbol&match=A030012M09RIK) | structural molecule activity;motor activity;structural constituent of cytoskeleton |  | **1.53** |
| 1435644_AT | gb:BM234433 /DB_XREF=gi:17869703 /DB_XREF=K0349H11 |  | structural molecule activity;motor activity;structural constituent of muscle;structural constituent of cytoskeleton |  | **-2.29** |
| 1435648_AT | RIKEN cDNA B430119L13 gene | [B430119L13RIK](http://www.gene.ucl.ac.uk/cgi-bin/nomenclature/searchgenes.pl?field=symbol&anchor=equals&symbol_search=Search&number=100&format=html&sortby=symbol&match=B430119L13RIK) | structural molecule activity;protein binding |  | **1.94** |
| 1435652_A_AT | guanine nucleotide binding protein, alpha inhibiting 2 | [GNAI2](http://www.gene.ucl.ac.uk/cgi-bin/nomenclature/searchgenes.pl?field=symbol&anchor=equals&symbol_search=Search&number=100&format=html&sortby=symbol&match=GNAI2) | structural molecule activity;protein binding | [139360](http://www.ncbi.nlm.nih.gov/entrez/dispomim.cgi?id=139360) | **-1.26** |
| 1435653_AT | gb:BG070904 /DB_XREF=gi:12553396 /DB_XREF=H3091E12 |  | structural molecule activity;protein binding |  | **-1.6** |
| 1435656_AT | gb:AW122571 /DB_XREF=gi:6098096 /DB_XREF=UI-M-BH2. |  | structural molecule activity;protein binding;actin binding |  | **1.43** |
| 1435658_AT | solute carrier family 27 (fatty acid transporter), member 1 | [SLC27A1](http://www.gene.ucl.ac.uk/cgi-bin/nomenclature/searchgenes.pl?field=symbol&anchor=equals&symbol_search=Search&number=100&format=html&sortby=symbol&match=SLC27A1) | structural molecule activity;protein binding;actin binding;calcium ion binding;structural constituent of cytoskeleton | [600691](http://www.ncbi.nlm.nih.gov/entrez/dispomim.cgi?id=600691) | **1.66** |
| 1435666_AT | microtubule associated serine/threonine kinase 3 | [MAST3](http://www.gene.ucl.ac.uk/cgi-bin/nomenclature/searchgenes.pl?field=symbol&anchor=equals&symbol_search=Search&number=100&format=html&sortby=symbol&match=MAST3) | structural molecule activity;protein binding;actin binding;cytoskeletal protein binding |  | **-1.68** |
| 1435679_AT | optineurin | [OPTN](http://www.gene.ucl.ac.uk/cgi-bin/nomenclature/searchgenes.pl?field=symbol&anchor=equals&symbol_search=Search&number=100&format=html&sortby=symbol&match=OPTN) | structural molecule activity;protein binding;cadherin binding | [602432](http://www.ncbi.nlm.nih.gov/entrez/dispomim.cgi?id=602432) | **1.72** |
| 1435682_AT | leucyl-tRNA synthetase, mitochondrial | [LARS2](http://www.gene.ucl.ac.uk/cgi-bin/nomenclature/searchgenes.pl?field=symbol&anchor=equals&symbol_search=Search&number=100&format=html&sortby=symbol&match=LARS2) | structural molecule activity;protein binding;calcium ion binding | [604544](http://www.ncbi.nlm.nih.gov/entrez/dispomim.cgi?id=604544) | **1.23** |
| 1435693_AT | cDNA sequence BC012256 | [BC012256](http://www.gene.ucl.ac.uk/cgi-bin/nomenclature/searchgenes.pl?field=symbol&anchor=equals&symbol_search=Search&number=100&format=html&sortby=symbol&match=BC012256) | structural molecule activity;protein binding;calcium ion binding |  | **1.41** |
| 1435697_A_AT | pleckstrin homology, Sec7 and coiled-coil domains, binding protein | [PSCDBP](http://www.gene.ucl.ac.uk/cgi-bin/nomenclature/searchgenes.pl?field=symbol&anchor=equals&symbol_search=Search&number=100&format=html&sortby=symbol&match=PSCDBP) | structural molecule activity;protein binding;calcium ion binding | [604448](http://www.ncbi.nlm.nih.gov/entrez/dispomim.cgi?id=604448) | **-2.51** |
| 1435701_AT | 10 days neonate medulla oblongata cDNA, RIKEN full-length enriched library, clone:B830010I11 product:unclassifiable, full insert sequence |  | structural molecule activity;protein binding;DNA binding;transcription factor activity |  | **1.78** |
| 1435712_A_AT | ribosomal protein S18 | [RPS18](http://www.gene.ucl.ac.uk/cgi-bin/nomenclature/searchgenes.pl?field=symbol&anchor=equals&symbol_search=Search&number=100&format=html&sortby=symbol&match=RPS18) | structural molecule activity;protein binding;extracellular matrix structural constituent;extracellular matrix structural constituent conferring tensile strength | [180473](http://www.ncbi.nlm.nih.gov/entrez/dispomim.cgi?id=180473) | **-1.33** |
| 1435715_X_AT | thymine DNA glycosylase | [TDG](http://www.gene.ucl.ac.uk/cgi-bin/nomenclature/searchgenes.pl?field=symbol&anchor=equals&symbol_search=Search&number=100&format=html&sortby=symbol&match=TDG) | structural molecule activity;protein binding;extracellular matrix structural constituent;extracellular matrix structural constituent conferring tensile strength | [601423](http://www.ncbi.nlm.nih.gov/entrez/dispomim.cgi?id=601423) | **-1.32** |
| 1435716_X_AT | small nuclear ribonucleoprotein N | [SNRPN](http://www.gene.ucl.ac.uk/cgi-bin/nomenclature/searchgenes.pl?field=symbol&anchor=equals&symbol_search=Search&number=100&format=html&sortby=symbol&match=SNRPN) | structural molecule activity;protein binding;extracellular matrix structural constituent;extracellular matrix structural constituent conferring tensile strength | [182279](http://www.ncbi.nlm.nih.gov/entrez/dispomim.cgi?id=182279) | **1.71** |
| 1435725_X_AT | ribosomal protein L12 | [RPL12](http://www.gene.ucl.ac.uk/cgi-bin/nomenclature/searchgenes.pl?field=symbol&anchor=equals&symbol_search=Search&number=100&format=html&sortby=symbol&match=RPL12) | structural molecule activity;protein binding;extracellular matrix structural constituent;extracellular matrix structural constituent conferring tensile strength | [180475](http://www.ncbi.nlm.nih.gov/entrez/dispomim.cgi?id=180475) | **-1.37** |
| 1435732_X_AT | ATPase, H+ transporting, V0 subunit C | [ATP6V0C](http://www.gene.ucl.ac.uk/cgi-bin/nomenclature/searchgenes.pl?field=symbol&anchor=equals&symbol_search=Search&number=100&format=html&sortby=symbol&match=ATP6V0C) | structural molecule activity;protein binding;extracellular matrix structural constituent;extracellular matrix structural constituent conferring tensile strength | [108745](http://www.ncbi.nlm.nih.gov/entrez/dispomim.cgi?id=108745) | **-1.24** |
| 1435743_AT | RIKEN cDNA C130068N17 gene | [C130068N17RIK](http://www.gene.ucl.ac.uk/cgi-bin/nomenclature/searchgenes.pl?field=symbol&anchor=equals&symbol_search=Search&number=100&format=html&sortby=symbol&match=C130068N17RIK) | structural molecule activity;protein binding;protein transporter activity |  | **1.77** |
| 1435752_S_AT | ATP-binding cassette, sub-family C (CFTR/MRP), member 9 | [ABCC9](http://www.gene.ucl.ac.uk/cgi-bin/nomenclature/searchgenes.pl?field=symbol&anchor=equals&symbol_search=Search&number=100&format=html&sortby=symbol&match=ABCC9) | structural molecule activity;protein binding;scavenger receptor activity;receptor activity | [601439](http://www.ncbi.nlm.nih.gov/entrez/dispomim.cgi?id=601439) | **1.42** |
| 1435754_AT | DNA segment, Chr 6, Brigham & Women's Genetics 1452 expressed | [D6BWG1452E](http://www.gene.ucl.ac.uk/cgi-bin/nomenclature/searchgenes.pl?field=symbol&anchor=equals&symbol_search=Search&number=100&format=html&sortby=symbol&match=D6BWG1452E) | structural molecule activity;protein binding;serine-type endopeptidase inhibitor activity;endopeptidase inhibitor activity;extracellular matrix structural constituent conferring tensile strength |  | **1.22** |
| 1435763_AT | TBC1 domain family, member 16 | [TBC1D16](http://www.gene.ucl.ac.uk/cgi-bin/nomenclature/searchgenes.pl?field=symbol&anchor=equals&symbol_search=Search&number=100&format=html&sortby=symbol&match=TBC1D16) | structural molecule activity;protein binding;structural constituent of cytoskeleton |  | **1.54** |
| 1435777_AT | RIKEN cDNA E030018N11 gene | [E030018N11RIK](http://www.gene.ucl.ac.uk/cgi-bin/nomenclature/searchgenes.pl?field=symbol&anchor=equals&symbol_search=Search&number=100&format=html&sortby=symbol&match=E030018N11RIK) | structural molecule activity;protein binding;structural constituent of cytoskeleton |  | **-1.47** |
| 1435784_AT | expressed sequence AU019532 | [APG9L1](http://www.gene.ucl.ac.uk/cgi-bin/nomenclature/searchgenes.pl?field=symbol&anchor=equals&symbol_search=Search&number=100&format=html&sortby=symbol&match=APG9L1) | structural molecule activity;protein binding;structural constituent of cytoskeleton |  | **1.28** |
| 1435785_AT | EH-domain containing 2 | [EHD2](http://www.gene.ucl.ac.uk/cgi-bin/nomenclature/searchgenes.pl?field=symbol&anchor=equals&symbol_search=Search&number=100&format=html&sortby=symbol&match=EHD2) | structural molecule activity;protein binding;subtilase activity;extracellular matrix structural constituent;extracellular matrix structural constituent conferring tensile strength | [605890](http://www.ncbi.nlm.nih.gov/entrez/dispomim.cgi?id=605890) | **1.76** |
| 1435787_AT | protein phosphatase 1 (formerly 2C)-like | [PPM1L](http://www.gene.ucl.ac.uk/cgi-bin/nomenclature/searchgenes.pl?field=symbol&anchor=equals&symbol_search=Search&number=100&format=html&sortby=symbol&match=PPM1L) | structural molecule activity;structural constituent of cytoskeleton |  | **1.33** |
| 1435791_X_AT | ribosomal protein L17 | [RPL17](http://www.gene.ucl.ac.uk/cgi-bin/nomenclature/searchgenes.pl?field=symbol&anchor=equals&symbol_search=Search&number=100&format=html&sortby=symbol&match=RPL17) | structural molecule activity;structural constituent of cytoskeleton | [603661](http://www.ncbi.nlm.nih.gov/entrez/dispomim.cgi?id=603661) | **-1.57** |
| 1435803_A_AT | eukaryotic translation initiation factor 4E member 2 | [EIF4E2](http://www.gene.ucl.ac.uk/cgi-bin/nomenclature/searchgenes.pl?field=symbol&anchor=equals&symbol_search=Search&number=100&format=html&sortby=symbol&match=EIF4E2) | structural molecule activity;structural constituent of cytoskeleton | [605895](http://www.ncbi.nlm.nih.gov/entrez/dispomim.cgi?id=605895) | **-2.24** |
| 1435807_AT | cell division cycle 42 homolog (S. cerevisiae) | [CDC42](http://www.gene.ucl.ac.uk/cgi-bin/nomenclature/searchgenes.pl?field=symbol&anchor=equals&symbol_search=Search&number=100&format=html&sortby=symbol&match=CDC42) | structural molecule activity;structural constituent of cytoskeleton | [116952](http://www.ncbi.nlm.nih.gov/entrez/dispomim.cgi?id=116952) | **-1.62** |
| 1435808_AT | RIKEN cDNA A230051G13 gene | [A230051G13RIK](http://www.gene.ucl.ac.uk/cgi-bin/nomenclature/searchgenes.pl?field=symbol&anchor=equals&symbol_search=Search&number=100&format=html&sortby=symbol&match=A230051G13RIK) | structural molecule activity;structural constituent of cytoskeleton |  | **1.24** |
| 1435822_AT | RIKEN cDNA D830012I24 gene | [D830012I24RIK](http://www.gene.ucl.ac.uk/cgi-bin/nomenclature/searchgenes.pl?field=symbol&anchor=equals&symbol_search=Search&number=100&format=html&sortby=symbol&match=D830012I24RIK) | structural molecule activity;structural constituent of cytoskeleton |  | **-1.53** |
| 1435823_X_AT | EGF-like domain 7 | [EGFL7](http://www.gene.ucl.ac.uk/cgi-bin/nomenclature/searchgenes.pl?field=symbol&anchor=equals&symbol_search=Search&number=100&format=html&sortby=symbol&match=EGFL7) | structural molecule activity;structural constituent of myelin sheath | [608582](http://www.ncbi.nlm.nih.gov/entrez/dispomim.cgi?id=608582) | **1.43** |
| 1435831_AT | gb:BB427704 /DB_XREF=gi:16423841 /DB_XREF=BB427704 |  | structural molecule activity;structural constituent of myelin sheath |  | **1.99** |
| 1435836_AT | gb:BB553369 /DB_XREF=gi:16448272 /DB_XREF=BB553369 |  | structural molecule activity;structural constituent of myelin sheath |  | **1.6** |
| 1435841_S_AT | succinate-Coenzyme A ligase, GDP-forming, beta subunit | [SUCLG2](http://www.gene.ucl.ac.uk/cgi-bin/nomenclature/searchgenes.pl?field=symbol&anchor=equals&symbol_search=Search&number=100&format=html&sortby=symbol&match=SUCLG2) | structural molecule activity;structural constituent of myelin sheath | [603922](http://www.ncbi.nlm.nih.gov/entrez/dispomim.cgi?id=603922) | **1.82** |
| 1435855_X_AT | pyrroline-5-carboxylate synthetase (glutamate gamma-semialdehyde synthetase) | [PYCS](http://www.gene.ucl.ac.uk/cgi-bin/nomenclature/searchgenes.pl?field=symbol&anchor=equals&symbol_search=Search&number=100&format=html&sortby=symbol&match=PYCS) | structural molecule activity;transmembrane receptor activity;calcium ion binding | [138250](http://www.ncbi.nlm.nih.gov/entrez/dispomim.cgi?id=138250) | **-1.63** |
| 1435870_AT | Synaptonemal complex protein 3 | [SYCP3](http://www.gene.ucl.ac.uk/cgi-bin/nomenclature/searchgenes.pl?field=symbol&anchor=equals&symbol_search=Search&number=100&format=html&sortby=symbol&match=SYCP3) | structural molecule activity;zinc ion binding;protein binding;actin binding;calcium ion binding | [604759](http://www.ncbi.nlm.nih.gov/entrez/dispomim.cgi?id=604759) | **1.6** |
| 1435873_A_AT | ribosomal protein L13a | [RPL13A](http://www.gene.ucl.ac.uk/cgi-bin/nomenclature/searchgenes.pl?field=symbol&anchor=equals&symbol_search=Search&number=100&format=html&sortby=symbol&match=RPL13A) | structural molecule activity;zinc ion binding;protein binding;actin binding;calcium ion binding |  | **-1.36** |
| 1435878_AT | serine/threonine kinase 38 like | [STK38L](http://www.gene.ucl.ac.uk/cgi-bin/nomenclature/searchgenes.pl?field=symbol&anchor=equals&symbol_search=Search&number=100&format=html&sortby=symbol&match=STK38L) | succinate dehydrogenase activity |  | **1.34** |
| 1435888_AT | RIKEN cDNA 9030024J15 gene | [9030024J15RIK](http://www.gene.ucl.ac.uk/cgi-bin/nomenclature/searchgenes.pl?field=symbol&anchor=equals&symbol_search=Search&number=100&format=html&sortby=symbol&match=9030024J15RIK) | succinate dehydrogenase activity |  | **-2.43** |
| 1435893_AT | very low density lipoprotein receptor | [VLDLR](http://www.gene.ucl.ac.uk/cgi-bin/nomenclature/searchgenes.pl?field=symbol&anchor=equals&symbol_search=Search&number=100&format=html&sortby=symbol&match=VLDLR) | succinate-CoA ligase (ADP-forming) activity;succinate-CoA ligase activity;ligase activity;catalytic activity | [192977](http://www.ncbi.nlm.nih.gov/entrez/dispomim.cgi?id=192977) | **1.35** |
| 1435895_AT | RIKEN cDNA D930023J12 gene | [D930023J12RIK](http://www.gene.ucl.ac.uk/cgi-bin/nomenclature/searchgenes.pl?field=symbol&anchor=equals&symbol_search=Search&number=100&format=html&sortby=symbol&match=D930023J12RIK) | succinate-semialdehyde dehydrogenase activity;ligase activity;catalytic activity;succinate-CoA ligase (GDP-forming) activity |  | **-1.74** |
| 1435898_X_AT | guanosine diphosphate (GDP) dissociation inhibitor 3 | [GDI3](http://www.gene.ucl.ac.uk/cgi-bin/nomenclature/searchgenes.pl?field=symbol&anchor=equals&symbol_search=Search&number=100&format=html&sortby=symbol&match=GDI3) | succinate-semialdehyde dehydrogenase activity;ligase activity;catalytic activity;succinate-CoA ligase (GDP-forming) activity | [600767](http://www.ncbi.nlm.nih.gov/entrez/dispomim.cgi?id=600767) | **-1.28** |
| 1435903_AT | RIKEN cDNA B230315M08 gene | [B230315M08RIK](http://www.gene.ucl.ac.uk/cgi-bin/nomenclature/searchgenes.pl?field=symbol&anchor=equals&symbol_search=Search&number=100&format=html&sortby=symbol&match=B230315M08RIK) | sugar binding |  | **-2.31** |
| 1435911_S_AT | solute carrier family 2 (facilitated glucose transporter), member 12 | [SLC2A12](http://www.gene.ucl.ac.uk/cgi-bin/nomenclature/searchgenes.pl?field=symbol&anchor=equals&symbol_search=Search&number=100&format=html&sortby=symbol&match=SLC2A12) | sugar binding |  | **1.38** |
| 1435929_AT | RIKEN cDNA 9630033F20 gene | [9630033F20RIK](http://www.gene.ucl.ac.uk/cgi-bin/nomenclature/searchgenes.pl?field=symbol&anchor=equals&symbol_search=Search&number=100&format=html&sortby=symbol&match=9630033F20RIK) | sugar binding |  | **1.94** |
| 1435933_AT | RIKEN cDNA A230052E19 gene | [A230052E19RIK](http://www.gene.ucl.ac.uk/cgi-bin/nomenclature/searchgenes.pl?field=symbol&anchor=equals&symbol_search=Search&number=100&format=html&sortby=symbol&match=A230052E19RIK) | sugar binding |  | **-4.12** |
| 1435960_AT | gb:BI733057 /DB_XREF=gi:15710070 /DB_XREF=60335463 |  | sugar binding |  | **-2.76** |
| 1435967_S_AT | 3-hydroxyisobutyrate dehydrogenase | [HIBADH](http://www.gene.ucl.ac.uk/cgi-bin/nomenclature/searchgenes.pl?field=symbol&anchor=equals&symbol_search=Search&number=100&format=html&sortby=symbol&match=HIBADH) | sugar binding | [608475](http://www.ncbi.nlm.nih.gov/entrez/dispomim.cgi?id=608475) | **1.46** |
| 1435972_AT | calpastatin | [CAST](http://www.gene.ucl.ac.uk/cgi-bin/nomenclature/searchgenes.pl?field=symbol&anchor=equals&symbol_search=Search&number=100&format=html&sortby=symbol&match=CAST) | sugar binding | [114090](http://www.ncbi.nlm.nih.gov/entrez/dispomim.cgi?id=114090) | **1.24** |
| 1435986_X_AT | succinate dehydrogenase complex, subunit C, integral membrane protein | [SDHC](http://www.gene.ucl.ac.uk/cgi-bin/nomenclature/searchgenes.pl?field=symbol&anchor=equals&symbol_search=Search&number=100&format=html&sortby=symbol&match=SDHC) | sugar binding | [602413](http://www.ncbi.nlm.nih.gov/entrez/dispomim.cgi?id=602413) | **1.62** |
| 1435987_X_AT | RIKEN cDNA 1110059G02 gene | [1110059G02RIK](http://www.gene.ucl.ac.uk/cgi-bin/nomenclature/searchgenes.pl?field=symbol&anchor=equals&symbol_search=Search&number=100&format=html&sortby=symbol&match=1110059G02RIK) | sugar binding;calcium ion binding;hyaluronic acid binding |  | **-3.69** |
| 1435988_X_AT | IK cytokine | [IK](http://www.gene.ucl.ac.uk/cgi-bin/nomenclature/searchgenes.pl?field=symbol&anchor=equals&symbol_search=Search&number=100&format=html&sortby=symbol&match=IK) | sugar binding;galactose binding | [600549](http://www.ncbi.nlm.nih.gov/entrez/dispomim.cgi?id=600549) | **-1.38** |
| 1435990_AT | a disintegrin-like and metalloprotease (reprolysin type) with thrombospondin type 1 motif, 2 | [ADAMTS2](http://www.gene.ucl.ac.uk/cgi-bin/nomenclature/searchgenes.pl?field=symbol&anchor=equals&symbol_search=Search&number=100&format=html&sortby=symbol&match=ADAMTS2) | sugar binding;galactose binding | [604539](http://www.ncbi.nlm.nih.gov/entrez/dispomim.cgi?id=604539) | **-4.24** |
| 1436015_S_AT | serine/threonine kinase 4 | [STK4](http://www.gene.ucl.ac.uk/cgi-bin/nomenclature/searchgenes.pl?field=symbol&anchor=equals&symbol_search=Search&number=100&format=html&sortby=symbol&match=STK4) | sugar binding;receptor activity | [604965](http://www.ncbi.nlm.nih.gov/entrez/dispomim.cgi?id=604965) | **-1.4** |
| 1436016_X_AT | guanosine diphosphate (GDP) dissociation inhibitor 3 | [GDI3](http://www.gene.ucl.ac.uk/cgi-bin/nomenclature/searchgenes.pl?field=symbol&anchor=equals&symbol_search=Search&number=100&format=html&sortby=symbol&match=GDI3) | sugar binding;receptor activity | [600767](http://www.ncbi.nlm.nih.gov/entrez/dispomim.cgi?id=600767) | **-1.36** |
| 1436021_AT | RIKEN cDNA A930031D07 gene | [A930031D07RIK](http://www.gene.ucl.ac.uk/cgi-bin/nomenclature/searchgenes.pl?field=symbol&anchor=equals&symbol_search=Search&number=100&format=html&sortby=symbol&match=A930031D07RIK) | sugar binding;sulfotransferase activity;transferase activity |  | **2.33** |
| 1436025_AT | RIKEN cDNA A430106J12 gene | [A430106J12RIK](http://www.gene.ucl.ac.uk/cgi-bin/nomenclature/searchgenes.pl?field=symbol&anchor=equals&symbol_search=Search&number=100&format=html&sortby=symbol&match=A430106J12RIK) | sugar porter activity;receptor activity |  | **-1.81** |
| 1436026_AT | ES neuronal differentiation 2 | [END2](http://www.gene.ucl.ac.uk/cgi-bin/nomenclature/searchgenes.pl?field=symbol&anchor=equals&symbol_search=Search&number=100&format=html&sortby=symbol&match=END2) | sugar porter activity;receptor activity |  | **-1.59** |
| 1436029_AT | RIKEN cDNA 4930533K18 gene | [4930533K18RIK](http://www.gene.ucl.ac.uk/cgi-bin/nomenclature/searchgenes.pl?field=symbol&anchor=equals&symbol_search=Search&number=100&format=html&sortby=symbol&match=4930533K18RIK) | sugar porter activity;transporter activity;glucose transporter activity;insulin-responsive hydrogen:glucose transporter activity |  | **-1.64** |
| 1436042_AT | Talin 1 | [TLN1](http://www.gene.ucl.ac.uk/cgi-bin/nomenclature/searchgenes.pl?field=symbol&anchor=equals&symbol_search=Search&number=100&format=html&sortby=symbol&match=TLN1) | superoxide-generating NADPH oxidase activity | [186745](http://www.ncbi.nlm.nih.gov/entrez/dispomim.cgi?id=186745) | **-1.9** |
| 1436046_X_AT | ribosomal protein L29 | [RPL29](http://www.gene.ucl.ac.uk/cgi-bin/nomenclature/searchgenes.pl?field=symbol&anchor=equals&symbol_search=Search&number=100&format=html&sortby=symbol&match=RPL29) | superoxide-generating NADPH oxidase activity | [601832](http://www.ncbi.nlm.nih.gov/entrez/dispomim.cgi?id=601832) | **-1.28** |
| 1436050_X_AT | hairy and enhancer of split 6 (Drosophila) | [HES6](http://www.gene.ucl.ac.uk/cgi-bin/nomenclature/searchgenes.pl?field=symbol&anchor=equals&symbol_search=Search&number=100&format=html&sortby=symbol&match=HES6) | superoxide-generating NADPH oxidase activity;oxidoreductase activity;electron carrier activity |  | **-2.18** |
| 1436051_AT | RIKEN cDNA 9630007J19 gene | [9630007J19RIK](http://www.gene.ucl.ac.uk/cgi-bin/nomenclature/searchgenes.pl?field=symbol&anchor=equals&symbol_search=Search&number=100&format=html&sortby=symbol&match=9630007J19RIK) | symporter activity;binding |  | **-1.86** |
| 1436056_AT | kinesin family member 13B | [KIF13B](http://www.gene.ucl.ac.uk/cgi-bin/nomenclature/searchgenes.pl?field=symbol&anchor=equals&symbol_search=Search&number=100&format=html&sortby=symbol&match=KIF13B) | symporter activity;monocarboxylate porter activity;transporter activity | [607350](http://www.ncbi.nlm.nih.gov/entrez/dispomim.cgi?id=607350) | **-1.4** |
| 1436070_AT | gb:BM933153 /DB_XREF=gi:19392305 /DB_XREF=UI-M-BH3 |  | symporter activity;monocarboxylate porter activity;transporter activity;carrier activity |  | **1.54** |
| 1436092_AT | gb:BB336256 /DB_XREF=gi:15411598 /DB_XREF=BB336256 |  | symporter activity;monocarboxylate porter activity;transporter activity;carrier activity |  | **-1.87** |
| 1436098_AT | gb:BB667452 /DB_XREF=gi:16398901 /DB_XREF=BB667452 |  | symporter activity;sialic acid transporter activity;transporter activity |  | **1.36** |
| 1436103_AT | RAB3A interacting protein | [RAB3IP](http://www.gene.ucl.ac.uk/cgi-bin/nomenclature/searchgenes.pl?field=symbol&anchor=equals&symbol_search=Search&number=100&format=html&sortby=symbol&match=RAB3IP) | symporter activity;sodium:dicarboxylate symporter activity;glutamate:sodium symporter activity;sodium/excitatory glutamate symporter activity;carrier activity | [608686](http://www.ncbi.nlm.nih.gov/entrez/dispomim.cgi?id=608686) | **1.4** |
| 1436110_AT | 10 days neonate medulla oblongata cDNA, RIKEN full-length enriched library, clone:B830012K24 product:unknown EST, full insert sequence |  | symporter activity;transporter activity;ATP binding;carrier activity;ion transporter activity |  | **1.68** |
| 1436113_A_AT | gb:BB770696 /DB_XREF=gi:16197145 /DB_XREF=BB770696 |  | symporter activity;transporter activity;ATP binding;carrier activity;ion transporter activity |  | **1.22** |
| 1436115_AT | Gene model 266, (NCBI) | [LOC212539](http://www.gene.ucl.ac.uk/cgi-bin/nomenclature/searchgenes.pl?field=symbol&anchor=equals&symbol_search=Search&number=100&format=html&sortby=symbol&match=LOC212539) | symporter activity;transporter activity;carrier activity |  | **-12.63** |
| 1436119_AT | RIKEN cDNA D330038I09 gene | [D330038I09RIK](http://www.gene.ucl.ac.uk/cgi-bin/nomenclature/searchgenes.pl?field=symbol&anchor=equals&symbol_search=Search&number=100&format=html&sortby=symbol&match=D330038I09RIK) | symporter activity;transporter activity;cation:chloride symporter activity;amino acid-polyamine transporter activity;carrier activity |  | **-1.33** |
| 1436156_AT | cell division cycle and apoptosis regulator 1 | [CCAR1](http://www.gene.ucl.ac.uk/cgi-bin/nomenclature/searchgenes.pl?field=symbol&anchor=equals&symbol_search=Search&number=100&format=html&sortby=symbol&match=CCAR1) | TATA-binding protein binding |  | **-1.3** |
| 1436157_AT | cell division cycle and apoptosis regulator 1 | [CCAR1](http://www.gene.ucl.ac.uk/cgi-bin/nomenclature/searchgenes.pl?field=symbol&anchor=equals&symbol_search=Search&number=100&format=html&sortby=symbol&match=CCAR1) | TATA-binding protein binding |  | **-1.38** |
| 1436168_AT | RIKEN cDNA C730029A08 gene | [C730029A08RIK](http://www.gene.ucl.ac.uk/cgi-bin/nomenclature/searchgenes.pl?field=symbol&anchor=equals&symbol_search=Search&number=100&format=html&sortby=symbol&match=C730029A08RIK) | tau protein binding;lipid transporter activity;lipid binding;low-density lipoprotein receptor binding;antioxidant activity;heparin binding;beta-amyloid binding |  | **2.05** |
| 1436169_AT | RIKEN cDNA C730029A08 gene | [PAQR9](http://www.gene.ucl.ac.uk/cgi-bin/nomenclature/searchgenes.pl?field=symbol&anchor=equals&symbol_search=Search&number=100&format=html&sortby=symbol&match=PAQR9) | thiosulfate sulfurtransferase activity;3-mercaptopyruvate sulfurtransferase activity;transferase activity |  | **2.14** |
| 1436171_AT | RIKEN cDNA 6030405P05 gene | [6030405P05RIK](http://www.gene.ucl.ac.uk/cgi-bin/nomenclature/searchgenes.pl?field=symbol&anchor=equals&symbol_search=Search&number=100&format=html&sortby=symbol&match=6030405P05RIK) | thiosulfate sulfurtransferase activity;transferase activity |  | **-1.85** |
| 1436173_AT | Similar to Rho-GTPase-activating protein 7 (Rho-type GTPase-activating protein 7) (Deleted in liver cancer 1 protein homolog) (Dlc-1) (StAR-related lipid transfer protein 12) (StARD12) (START domain-containing protein 12) | [LOC434301](http://www.gene.ucl.ac.uk/cgi-bin/nomenclature/searchgenes.pl?field=symbol&anchor=equals&symbol_search=Search&number=100&format=html&sortby=symbol&match=LOC434301) | threonine-tRNA ligase activity;ATP binding;tRNA ligase activity |  | **1.78** |
| 1436185_AT | expressed sequence AI314180 | [AI314180](http://www.gene.ucl.ac.uk/cgi-bin/nomenclature/searchgenes.pl?field=symbol&anchor=equals&symbol_search=Search&number=100&format=html&sortby=symbol&match=AI314180) | thyroxine 5'-deiodinase activity;oxidoreductase activity;hydrolase activity |  | **1.28** |
| 1436186_AT | RIKEN cDNA 4432406C08 gene | [4432406C08RIK](http://www.gene.ucl.ac.uk/cgi-bin/nomenclature/searchgenes.pl?field=symbol&anchor=equals&symbol_search=Search&number=100&format=html&sortby=symbol&match=4432406C08RIK) | thyroxine 5'-deiodinase activity;oxidoreductase activity;hydrolase activity |  | **-5.43** |
| 1436188_A_AT | gb:AI837704 /DB_XREF=gi:5471917 /DB_XREF=UI-M-AK0- |  | transaminase activity;1-aminocyclopropane-1-carboxylate synthase activity;glutamine-phenylpyruvate transaminase activity;transferase activity |  | **-3.45** |
| 1436192_AT | ADP-ribosylation factor guanine nucleotide-exchange factor 2 (brefeldin A-inhibited) | [ARFGEF2](http://www.gene.ucl.ac.uk/cgi-bin/nomenclature/searchgenes.pl?field=symbol&anchor=equals&symbol_search=Search&number=100&format=html&sortby=symbol&match=ARFGEF2) | transaminase activity;alanine transaminase activity;transferase activity | [605371](http://www.ncbi.nlm.nih.gov/entrez/dispomim.cgi?id=605371) | **1.29** |
| 1436197_AT | CDNA sequence BC046418 | [BC046418](http://www.gene.ucl.ac.uk/cgi-bin/nomenclature/searchgenes.pl?field=symbol&anchor=equals&symbol_search=Search&number=100&format=html&sortby=symbol&match=BC046418) | transaminase activity;alanine transaminase activity;transferase activity |  | **-1.46** |
| 1436203_A_AT | RIKEN cDNA 1110059G02 gene | [1110059G02RIK](http://www.gene.ucl.ac.uk/cgi-bin/nomenclature/searchgenes.pl?field=symbol&anchor=equals&symbol_search=Search&number=100&format=html&sortby=symbol&match=1110059G02RIK) | transaminase activity;catalytic activity;alanine transaminase activity;transferase activity |  | **-5.26** |
| 1436204_AT | RIKEN cDNA 1110059G02 gene | [1110059G02RIK](http://www.gene.ucl.ac.uk/cgi-bin/nomenclature/searchgenes.pl?field=symbol&anchor=equals&symbol_search=Search&number=100&format=html&sortby=symbol&match=1110059G02RIK) | transaminase activity;glycine C-acetyltransferase activity;ligase activity;catalytic activity;acyltransferase activity;transferase activity |  | **-2.93** |
| 1436215_AT | inositol polyphosphate multikinase | [IPMK](http://www.gene.ucl.ac.uk/cgi-bin/nomenclature/searchgenes.pl?field=symbol&anchor=equals&symbol_search=Search&number=100&format=html&sortby=symbol&match=IPMK) | transaminase activity;transferase activity |  | **1.45** |
| 1436219_AT | RIKEN cDNA 4933403F05 gene | [4933403F05RIK](http://www.gene.ucl.ac.uk/cgi-bin/nomenclature/searchgenes.pl?field=symbol&anchor=equals&symbol_search=Search&number=100&format=html&sortby=symbol&match=4933403F05RIK) | transaminase activity;transferase activity |  | **1.6** |
| 1436233_AT | gb:BM123923 /DB_XREF=gi:17107691 /DB_XREF=L0532G03 |  | transaminase activity;transferase activity |  | **1.77** |
| 1436236_X_AT | gb:AI327078 /DB_XREF=gi:4061507 /DB_XREF=mf95b07.x |  | transaminase activity;transferase activity;alanine transaminase activity |  | **-1.74** |
| 1436241_S_AT | histone cell cycle regulation defective homolog A (S. cerevisiae) | [HIRA](http://www.gene.ucl.ac.uk/cgi-bin/nomenclature/searchgenes.pl?field=symbol&anchor=equals&symbol_search=Search&number=100&format=html&sortby=symbol&match=HIRA) | transcription coactivator activity |  | **1.46** |
| 1436255_AT | RIKEN cDNA D830044I16 gene | [D830044I16RIK](http://www.gene.ucl.ac.uk/cgi-bin/nomenclature/searchgenes.pl?field=symbol&anchor=equals&symbol_search=Search&number=100&format=html&sortby=symbol&match=D830044I16RIK) | transcription coactivator activity;DNA binding |  | **1.34** |
| 1436269_S_AT | protease, serine, 25 | [PRSS25](http://www.gene.ucl.ac.uk/cgi-bin/nomenclature/searchgenes.pl?field=symbol&anchor=equals&symbol_search=Search&number=100&format=html&sortby=symbol&match=PRSS25) | transcription coactivator activity;DNA binding;general RNA polymerase II transcription factor activity;protein C-terminus binding | [606441](http://www.ncbi.nlm.nih.gov/entrez/dispomim.cgi?id=606441) | **-1.42** |
| 1436278_AT | gb:BM934468 /DB_XREF=gi:19393620 /DB_XREF=UI-M-BH2 |  | transcription coactivator activity;DNA binding;general RNA polymerase II transcription factor activity;protein C-terminus binding |  | **1.57** |
| 1436303_AT | RIKEN cDNA 5033403D15 gene | [5033403D15RIK](http://www.gene.ucl.ac.uk/cgi-bin/nomenclature/searchgenes.pl?field=symbol&anchor=equals&symbol_search=Search&number=100&format=html&sortby=symbol&match=5033403D15RIK) | transcription coactivator activity;endonuclease activity;DNA-(apurinic or apyrimidinic site) lyase activity;protein binding;metal ion binding;3'-5' exonuclease activity;oxidoreductase activity;DNA binding;nuclease activity;phosphodiesterase I activity;lyase activity;ribonuclease H activity |  | **1.88** |
| 1436331_AT | RIKEN cDNA A630031M04 gene | [VPS13D](http://www.gene.ucl.ac.uk/cgi-bin/nomenclature/searchgenes.pl?field=symbol&anchor=equals&symbol_search=Search&number=100&format=html&sortby=symbol&match=VPS13D) | transcription coactivator activity;endonuclease activity;DNA-(apurinic or apyrimidinic site) lyase activity;protein binding;metal ion binding;oxidoreductase activity;3'-5' exonuclease activity;DNA binding;nuclease activity;lyase activity;phosphodiesterase I activity;ribonuclease H activity | [608877](http://www.ncbi.nlm.nih.gov/entrez/dispomim.cgi?id=608877) | **1.53** |
| 1436332_AT | heat shock protein, alpha-crystallin-related, B6 | [HSPB6](http://www.gene.ucl.ac.uk/cgi-bin/nomenclature/searchgenes.pl?field=symbol&anchor=equals&symbol_search=Search&number=100&format=html&sortby=symbol&match=HSPB6) | transcription coactivator activity;molecular_function unknown;protein binding;transcription corepressor activity |  | **1.39** |
| 1436339_AT | RIKEN cDNA 1810058I24 gene | [1810058I24RIK](http://www.gene.ucl.ac.uk/cgi-bin/nomenclature/searchgenes.pl?field=symbol&anchor=equals&symbol_search=Search&number=100&format=html&sortby=symbol&match=1810058I24RIK) | transcription coactivator activity;phosphotransferase activity, alcohol group as acceptor |  | **-1.33** |
| 1436344_AT | gb:BB005022 /DB_XREF=gi:16214884 /DB_XREF=BB005022 |  | transcription coactivator activity;protein binding |  | **1.42** |
| 1436350_AT | RIKEN cDNA D430039N05 gene | [D430039N05RIK](http://www.gene.ucl.ac.uk/cgi-bin/nomenclature/searchgenes.pl?field=symbol&anchor=equals&symbol_search=Search&number=100&format=html&sortby=symbol&match=D430039N05RIK) | transcription coactivator activity;protein binding;DNA binding;transcription factor activity |  | **-2.46** |
| 1436353_AT | RIKEN cDNA A230046K03 gene | [A230046K03RIK](http://www.gene.ucl.ac.uk/cgi-bin/nomenclature/searchgenes.pl?field=symbol&anchor=equals&symbol_search=Search&number=100&format=html&sortby=symbol&match=A230046K03RIK) | transcription coactivator activity;protein binding;DNA binding;transcription factor activity;RNA polymerase II transcription factor activity |  | **-1.6** |
| 1436355_AT | cDNA sequence BC044804 | [BC044804](http://www.gene.ucl.ac.uk/cgi-bin/nomenclature/searchgenes.pl?field=symbol&anchor=equals&symbol_search=Search&number=100&format=html&sortby=symbol&match=BC044804) | transcription coactivator activity;protein binding;DNA binding;transcription factor activity;RNA polymerase II transcription factor activity |  | **2.01** |
| 1436358_AT | ATP synthase mitochondrial F1 complex assembly factor 1 | [ATPAF1](http://www.gene.ucl.ac.uk/cgi-bin/nomenclature/searchgenes.pl?field=symbol&anchor=equals&symbol_search=Search&number=100&format=html&sortby=symbol&match=ATPAF1) | transcription coactivator activity;protein binding;gamma-tubulin binding | [608917](http://www.ncbi.nlm.nih.gov/entrez/dispomim.cgi?id=608917) | **1.62** |
| 1436367_AT | gb:BB119527 /DB_XREF=gi:16261957 /DB_XREF=BB119527 |  | transcription coactivator activity;protein binding;protein methyltransferase activity;methyltransferase activity;S-adenosylmethionine-dependent methyltransferase activity;transferase activity |  | **2.29** |
| 1436395_AT | Caspase recruitment domain family, member 6 | [CARD6](http://www.gene.ucl.ac.uk/cgi-bin/nomenclature/searchgenes.pl?field=symbol&anchor=equals&symbol_search=Search&number=100&format=html&sortby=symbol&match=CARD6) | transcription coactivator activity;thyroid hormone receptor binding;estrogen receptor binding;chromatin binding;retinoid X receptor binding;receptor activity;transcriptional activator activity |  | **-1.54** |
| 1436403_AT | cDNA sequence BC025575 | [BC025575](http://www.gene.ucl.ac.uk/cgi-bin/nomenclature/searchgenes.pl?field=symbol&anchor=equals&symbol_search=Search&number=100&format=html&sortby=symbol&match=BC025575) | transcription factor activity |  | **-2.35** |
| 1436440_AT | RIKEN cDNA B230107K20 gene | [B230107K20RIK](http://www.gene.ucl.ac.uk/cgi-bin/nomenclature/searchgenes.pl?field=symbol&anchor=equals&symbol_search=Search&number=100&format=html&sortby=symbol&match=B230107K20RIK) | transcription factor activity |  | **1.9** |
| 1436443_A_AT | KDEL (Lys-Asp-Glu-Leu) containing 1 | [KDELC1](http://www.gene.ucl.ac.uk/cgi-bin/nomenclature/searchgenes.pl?field=symbol&anchor=equals&symbol_search=Search&number=100&format=html&sortby=symbol&match=KDELC1) | transcription factor activity |  | **-1.43** |
| 1436452_X_AT | gb:AW824211 /DB_XREF=gi:7917288 /DB_XREF=us07a04.x |  | transcription factor activity |  | **-1.23** |
| 1436495_S_AT | zinc finger protein 260 | [ZFP260](http://www.gene.ucl.ac.uk/cgi-bin/nomenclature/searchgenes.pl?field=symbol&anchor=equals&symbol_search=Search&number=100&format=html&sortby=symbol&match=ZFP260) | transcription factor activity | [601505](http://www.ncbi.nlm.nih.gov/entrez/dispomim.cgi?id=601505) | **-1.63** |
| 1436501_AT | mitochondrial tumor suppressor 1 | [MTUS1](http://www.gene.ucl.ac.uk/cgi-bin/nomenclature/searchgenes.pl?field=symbol&anchor=equals&symbol_search=Search&number=100&format=html&sortby=symbol&match=MTUS1) | transcription factor activity;lipid binding |  | **1.35** |
| 1436502_AT | mitochondrial tumor suppressor 1 | [MTUS1](http://www.gene.ucl.ac.uk/cgi-bin/nomenclature/searchgenes.pl?field=symbol&anchor=equals&symbol_search=Search&number=100&format=html&sortby=symbol&match=MTUS1) | transcription initiation factor activity;DNA binding |  | **1.56** |
| 1436508_AT | RIKEN cDNA 2410014A08 gene | [2410014A08RIK](http://www.gene.ucl.ac.uk/cgi-bin/nomenclature/searchgenes.pl?field=symbol&anchor=equals&symbol_search=Search&number=100&format=html&sortby=symbol&match=2410014A08RIK) | transcription regulator activity |  | **-1.4** |
| 1436509_AT | RIKEN cDNA 2410014A08 gene | [2410014A08RIK](http://www.gene.ucl.ac.uk/cgi-bin/nomenclature/searchgenes.pl?field=symbol&anchor=equals&symbol_search=Search&number=100&format=html&sortby=symbol&match=2410014A08RIK) | transcription regulator activity |  | **-1.56** |
| 1436513_AT | RIKEN cDNA 3526402J09 gene | [3526402J09RIK](http://www.gene.ucl.ac.uk/cgi-bin/nomenclature/searchgenes.pl?field=symbol&anchor=equals&symbol_search=Search&number=100&format=html&sortby=symbol&match=3526402J09RIK) | transcription regulator activity |  | **-1.44** |
| 1436514_AT | glypican 4 | [GPC4](http://www.gene.ucl.ac.uk/cgi-bin/nomenclature/searchgenes.pl?field=symbol&anchor=equals&symbol_search=Search&number=100&format=html&sortby=symbol&match=GPC4) | transcription regulator activity | [300168](http://www.ncbi.nlm.nih.gov/entrez/dispomim.cgi?id=300168) | **1.24** |
| 1436520_AT | Expressed sequence AI450948 | [AI450948](http://www.gene.ucl.ac.uk/cgi-bin/nomenclature/searchgenes.pl?field=symbol&anchor=equals&symbol_search=Search&number=100&format=html&sortby=symbol&match=AI450948) | transcription regulator activity;structural constituent of ribosome |  | **-2.32** |
| 1436523_S_AT | RIKEN cDNA 1810022K09 gene | [1810022K09RIK](http://www.gene.ucl.ac.uk/cgi-bin/nomenclature/searchgenes.pl?field=symbol&anchor=equals&symbol_search=Search&number=100&format=html&sortby=symbol&match=1810022K09RIK) | transcriptional activator activity |  | **-1.36** |
| 1436531_AT | RIKEN cDNA A930035J23 gene | [A930035J23RIK](http://www.gene.ucl.ac.uk/cgi-bin/nomenclature/searchgenes.pl?field=symbol&anchor=equals&symbol_search=Search&number=100&format=html&sortby=symbol&match=A930035J23RIK) | transcriptional activator activity;transcription factor binding |  | **1.3** |
| 1436534_AT | RIKEN cDNA 1810007I17 gene | [1810007I17RIK](http://www.gene.ucl.ac.uk/cgi-bin/nomenclature/searchgenes.pl?field=symbol&anchor=equals&symbol_search=Search&number=100&format=html&sortby=symbol&match=1810007I17RIK) | transferase activity |  | **-1.32** |
| 1436535_AT | RIKEN cDNA A530054J02 gene | [A530054J02RIK](http://www.gene.ucl.ac.uk/cgi-bin/nomenclature/searchgenes.pl?field=symbol&anchor=equals&symbol_search=Search&number=100&format=html&sortby=symbol&match=A530054J02RIK) | transferase activity |  | **-1.65** |
| 1436542_AT | Prostaglandin E receptor 1 (subtype EP1) | [PTGER1](http://www.gene.ucl.ac.uk/cgi-bin/nomenclature/searchgenes.pl?field=symbol&anchor=equals&symbol_search=Search&number=100&format=html&sortby=symbol&match=PTGER1) | transferase activity | [176802](http://www.ncbi.nlm.nih.gov/entrez/dispomim.cgi?id=176802) | **-1.26** |
| 1436545_AT | deltex 4 homolog (Drosophila) | [DTX4](http://www.gene.ucl.ac.uk/cgi-bin/nomenclature/searchgenes.pl?field=symbol&anchor=equals&symbol_search=Search&number=100&format=html&sortby=symbol&match=DTX4) | transferase activity |  | **-1.9** |
| 1436546_AT | gb:BM502719 /DB_XREF=gi:18663951 /DB_XREF=ii41g05. |  | transferase activity |  | **-1.44** |
| 1436548_AT | gb:AW108427 /DB_XREF=gi:6079227 /DB_XREF=um30e07.x |  | transferase activity |  | **1.93** |
| 1436566_AT | Rab40b, member RAS oncogene family | [RAB40B](http://www.gene.ucl.ac.uk/cgi-bin/nomenclature/searchgenes.pl?field=symbol&anchor=equals&symbol_search=Search&number=100&format=html&sortby=symbol&match=RAB40B) | transferase activity, transferring acyl groups, acyl groups converted into alkyl on transfer;citrate (Si)-synthase activity;lyase activity;transferase activity |  | **-5.2** |
| 1436567_A_AT | NADH dehydrogenase (ubiquinone) 1 alpha subcomplex, 7 (B14.5a) | [NDUFA7](http://www.gene.ucl.ac.uk/cgi-bin/nomenclature/searchgenes.pl?field=symbol&anchor=equals&symbol_search=Search&number=100&format=html&sortby=symbol&match=NDUFA7) | transferase activity, transferring acyl groups, acyl groups converted into alkyl on transfer;citrate (Si)-synthase activity;lyase activity;transferase activity | [602139](http://www.ncbi.nlm.nih.gov/entrez/dispomim.cgi?id=602139) | **1.24** |
| 1436568_AT | junction adhesion molecule 2 | [JAM2](http://www.gene.ucl.ac.uk/cgi-bin/nomenclature/searchgenes.pl?field=symbol&anchor=equals&symbol_search=Search&number=100&format=html&sortby=symbol&match=JAM2) | transferase activity, transferring acyl groups, acyl groups converted into alkyl on transfer;citrate (Si)-synthase activity;lyase activity;transferase activity | [606870](http://www.ncbi.nlm.nih.gov/entrez/dispomim.cgi?id=606870) | **1.73** |
| 1436569_AT | RIKEN cDNA C030045D06 gene | [C030045D06RIK](http://www.gene.ucl.ac.uk/cgi-bin/nomenclature/searchgenes.pl?field=symbol&anchor=equals&symbol_search=Search&number=100&format=html&sortby=symbol&match=C030045D06RIK) | transferase activity, transferring glycosyl groups;acetylglucosaminyltransferase activity;N-acetyllactosaminide beta-1,6-N-acetylglucosaminyltransferase activity;transferase activity |  | **1.99** |
| 1436577_AT | Cdc42 guanine nucleotide exchange factor (GEF) 9 | [ARHGEF9](http://www.gene.ucl.ac.uk/cgi-bin/nomenclature/searchgenes.pl?field=symbol&anchor=equals&symbol_search=Search&number=100&format=html&sortby=symbol&match=ARHGEF9) | transferase activity, transferring glycosyl groups;alpha-N-acetylneuraminate alpha-2,8-sialyltransferase activity;transferase activity;sialyltransferase activity | [300429](http://www.ncbi.nlm.nih.gov/entrez/dispomim.cgi?id=300429) | **1.5** |
| 1436586_X_AT | ribosomal protein S14 | [RPS14](http://www.gene.ucl.ac.uk/cgi-bin/nomenclature/searchgenes.pl?field=symbol&anchor=equals&symbol_search=Search&number=100&format=html&sortby=symbol&match=RPS14) | transferase activity, transferring glycosyl groups;alpha-N-acetylneuraminate alpha-2,8-sialyltransferase activity;transferase activity;sialyltransferase activity | [130620](http://www.ncbi.nlm.nih.gov/entrez/dispomim.cgi?id=130620) | **-1.2** |
| 1436596_AT | H2A histone family, member V | [H2AFV](http://www.gene.ucl.ac.uk/cgi-bin/nomenclature/searchgenes.pl?field=symbol&anchor=equals&symbol_search=Search&number=100&format=html&sortby=symbol&match=H2AFV) | transferase activity, transferring glycosyl groups;ceramide glucosyltransferase activity;transferase activity |  | **1.49** |
| 1436607_A_AT | RIKEN cDNA C330002I19 gene | [C330002I19RIK](http://www.gene.ucl.ac.uk/cgi-bin/nomenclature/searchgenes.pl?field=symbol&anchor=equals&symbol_search=Search&number=100&format=html&sortby=symbol&match=C330002I19RIK) | transferase activity, transferring glycosyl groups;ceramide glucosyltransferase activity;transferase activity |  | **-1.55** |
| 1436613_AT | gb:AI507196 /DB_XREF=gi:4405047 /DB_XREF=vj38e02.x |  | transferase activity, transferring glycosyl groups;galactosyltransferase activity;UDP-galactose beta-N-acetylglucosamine beta-1,3-galactosyltransferase activity;transferase activity |  | **1.5** |
| 1436618_AT | sideroflexin 5 | [SFXN5](http://www.gene.ucl.ac.uk/cgi-bin/nomenclature/searchgenes.pl?field=symbol&anchor=equals&symbol_search=Search&number=100&format=html&sortby=symbol&match=SFXN5) | transferase activity, transferring glycosyl groups;galactosyltransferase activity;UDP-galactose beta-N-acetylglucosamine beta-1,3-galactosyltransferase activity;transferase activity |  | **2.17** |
| 1436622_AT | IQ motif and Sec7 domain 2 | [IQSEC2](http://www.gene.ucl.ac.uk/cgi-bin/nomenclature/searchgenes.pl?field=symbol&anchor=equals&symbol_search=Search&number=100&format=html&sortby=symbol&match=IQSEC2) | transferase activity, transferring glycosyl groups;glycogen (starch) synthase activity;catalytic activity;transferase activity |  | **1.64** |
| 1436659_AT | gb:BB757120 /DB_XREF=gi:16203363 /DB_XREF=BB757120 |  | transferase activity, transferring glycosyl groups;glycogen (starch) synthase activity;catalytic activity;transferase activity |  | **-11.35** |
| 1436665_A_AT | latent transforming growth factor beta binding protein 4 | [LTBP4](http://www.gene.ucl.ac.uk/cgi-bin/nomenclature/searchgenes.pl?field=symbol&anchor=equals&symbol_search=Search&number=100&format=html&sortby=symbol&match=LTBP4) | transferase activity, transferring glycosyl groups;mannosyltransferase activity;dolichyl-phosphate beta-D-mannosyltransferase activity;transferase activity | [604710](http://www.ncbi.nlm.nih.gov/entrez/dispomim.cgi?id=604710) | **1.57** |
| 1436698_X_AT | cDNA sequence BC054438 | [BC054438](http://www.gene.ucl.ac.uk/cgi-bin/nomenclature/searchgenes.pl?field=symbol&anchor=equals&symbol_search=Search&number=100&format=html&sortby=symbol&match=BC054438) | transferase activity, transferring glycosyl groups;N-acetylglucosaminyl-proteoglycan 4-beta-glucuronosyltransferase activity;transferase activity |  | **1.26** |
| 1436699_X_AT | gb:BB422726 /DB_XREF=gi:9244081 /DB_XREF=BB422726 |  | transferase activity, transferring glycosyl groups;nicotinate phosphoribosyltransferase activity;nicotinamide phosphoribosyltransferase activity;transferase activity |  | **-1.53** |
| 1436706_AT | transmembrane protein 32 | [TMEM32](http://www.gene.ucl.ac.uk/cgi-bin/nomenclature/searchgenes.pl?field=symbol&anchor=equals&symbol_search=Search&number=100&format=html&sortby=symbol&match=TMEM32) | transferase activity, transferring glycosyl groups;nicotinate phosphoribosyltransferase activity;nicotinamide phosphoribosyltransferase activity;transferase activity |  | **1.29** |
| 1436708_X_AT | minichromosome maintenance deficient 4 homolog (S. cerevisiae) | [MCM4](http://www.gene.ucl.ac.uk/cgi-bin/nomenclature/searchgenes.pl?field=symbol&anchor=equals&symbol_search=Search&number=100&format=html&sortby=symbol&match=MCM4) | transferase activity, transferring glycosyl groups;sialyltransferase activity;transferase activity | [602638](http://www.ncbi.nlm.nih.gov/entrez/dispomim.cgi?id=602638) | **-1.67** |
| 1436714_AT | LIM domain containing preferred translocation partner in lipoma | [LPP](http://www.gene.ucl.ac.uk/cgi-bin/nomenclature/searchgenes.pl?field=symbol&anchor=equals&symbol_search=Search&number=100&format=html&sortby=symbol&match=LPP) | transferase activity, transferring glycosyl groups;transferase activity | [600700](http://www.ncbi.nlm.nih.gov/entrez/dispomim.cgi?id=600700) | **-1.3** |
| 1436715_S_AT | CDP-diacylglycerol--inositol 3-phosphatidyltransferase (phosphatidylinositol synthase) | [CDIPT](http://www.gene.ucl.ac.uk/cgi-bin/nomenclature/searchgenes.pl?field=symbol&anchor=equals&symbol_search=Search&number=100&format=html&sortby=symbol&match=CDIPT) | transferase activity, transferring glycosyl groups;transferase activity | [605893](http://www.ncbi.nlm.nih.gov/entrez/dispomim.cgi?id=605893) | **-1.47** |
| 1436731_AT | zinc finger protein 533 | [ZFP533](http://www.gene.ucl.ac.uk/cgi-bin/nomenclature/searchgenes.pl?field=symbol&anchor=equals&symbol_search=Search&number=100&format=html&sortby=symbol&match=ZFP533) | transferase activity, transferring glycosyl groups;transferase activity;sialyltransferase activity |  | **-5.56** |
| 1436739_AT | angiotensin receptor 1 | [AGTR1](http://www.gene.ucl.ac.uk/cgi-bin/nomenclature/searchgenes.pl?field=symbol&anchor=equals&symbol_search=Search&number=100&format=html&sortby=symbol&match=AGTR1) | transferase activity, transferring glycosyl groups;transferase activity;sialyltransferase activity |  | **1.53** |
| 1436745_AT | expressed sequence AI467484 | [AI467484](http://www.gene.ucl.ac.uk/cgi-bin/nomenclature/searchgenes.pl?field=symbol&anchor=equals&symbol_search=Search&number=100&format=html&sortby=symbol&match=AI467484) | transferase activity, transferring groups other than amino-acyl groups |  | **-1.42** |
| 1436750_A_AT | 3-oxoacid CoA transferase 1 | [OXCT1](http://www.gene.ucl.ac.uk/cgi-bin/nomenclature/searchgenes.pl?field=symbol&anchor=equals&symbol_search=Search&number=100&format=html&sortby=symbol&match=OXCT1) | transferase activity, transferring groups other than amino-acyl groups | [245050](http://www.ncbi.nlm.nih.gov/entrez/dispomim.cgi?id=245050) | **1.33** |
| 1436759_X_AT | calponin 3, acidic | [CNN3](http://www.gene.ucl.ac.uk/cgi-bin/nomenclature/searchgenes.pl?field=symbol&anchor=equals&symbol_search=Search&number=100&format=html&sortby=symbol&match=CNN3) | transferase activity, transferring hexosyl groups | [602374](http://www.ncbi.nlm.nih.gov/entrez/dispomim.cgi?id=602374) | **-1.7** |
| 1436778_AT | cytochrome b-245, beta polypeptide | [CYBB](http://www.gene.ucl.ac.uk/cgi-bin/nomenclature/searchgenes.pl?field=symbol&anchor=equals&symbol_search=Search&number=100&format=html&sortby=symbol&match=CYBB) | transferase activity, transferring hexosyl groups;transferase activity | [300481](http://www.ncbi.nlm.nih.gov/entrez/dispomim.cgi?id=300481) | **-3.92** |
| 1436779_AT | cytochrome b-245, beta polypeptide | [CYBB](http://www.gene.ucl.ac.uk/cgi-bin/nomenclature/searchgenes.pl?field=symbol&anchor=equals&symbol_search=Search&number=100&format=html&sortby=symbol&match=CYBB) | transferase activity, transferring hexosyl groups;transferase activity | [300481](http://www.ncbi.nlm.nih.gov/entrez/dispomim.cgi?id=300481) | **-2.8** |
| 1436790_A_AT | SRY-box containing gene 11 | [SOX11](http://www.gene.ucl.ac.uk/cgi-bin/nomenclature/searchgenes.pl?field=symbol&anchor=equals&symbol_search=Search&number=100&format=html&sortby=symbol&match=SOX11) | transferase activity, transferring phosphorus-containing groups;2'-phosphotransferase activity | [600898](http://www.ncbi.nlm.nih.gov/entrez/dispomim.cgi?id=600898) | **-20.67** |
| 1436791_AT | wingless-related MMTV integration site 5A | [WNT5A](http://www.gene.ucl.ac.uk/cgi-bin/nomenclature/searchgenes.pl?field=symbol&anchor=equals&symbol_search=Search&number=100&format=html&sortby=symbol&match=WNT5A) | transferase activity;protein binding;ubiquitin-protein ligase activity;enzyme binding;chaperone activity;ubiquitin conjugating enzyme activity;magnesium ion binding;nucleotidyltransferase activity | [164975](http://www.ncbi.nlm.nih.gov/entrez/dispomim.cgi?id=164975) | **1.76** |
| 1436795_AT | RIKEN cDNA 9630058J23 gene | [9630058J23RIK](http://www.gene.ucl.ac.uk/cgi-bin/nomenclature/searchgenes.pl?field=symbol&anchor=equals&symbol_search=Search&number=100&format=html&sortby=symbol&match=9630058J23RIK) | transferrin receptor activity;receptor activity;peptidase activity;iron ion transporter activity |  | **1.41** |
| 1436796_AT | RIKEN cDNA 1110061A14 gene | [1110061A14RIK](http://www.gene.ucl.ac.uk/cgi-bin/nomenclature/searchgenes.pl?field=symbol&anchor=equals&symbol_search=Search&number=100&format=html&sortby=symbol&match=1110061A14RIK) | transferrin receptor activity;receptor activity;peptidase activity;iron ion transporter activity |  | **-1.61** |
| 1436803_A_AT | NADH dehydrogenase (ubiquinone) 1 beta subcomplex, 9 | [NDUFB9](http://www.gene.ucl.ac.uk/cgi-bin/nomenclature/searchgenes.pl?field=symbol&anchor=equals&symbol_search=Search&number=100&format=html&sortby=symbol&match=NDUFB9) | translation elongation factor activity | [601445](http://www.ncbi.nlm.nih.gov/entrez/dispomim.cgi?id=601445) | **1.24** |
| 1436808_X_AT | minichromosome maintenance deficient 5, cell division cycle 46 (S. cerevisiae) | [MCM5](http://www.gene.ucl.ac.uk/cgi-bin/nomenclature/searchgenes.pl?field=symbol&anchor=equals&symbol_search=Search&number=100&format=html&sortby=symbol&match=MCM5) | translation elongation factor activity | [602696](http://www.ncbi.nlm.nih.gov/entrez/dispomim.cgi?id=602696) | **-3.33** |
| 1436811_AT | potassium channel tetramerisation domain containing 3 | [KCTD3](http://www.gene.ucl.ac.uk/cgi-bin/nomenclature/searchgenes.pl?field=symbol&anchor=equals&symbol_search=Search&number=100&format=html&sortby=symbol&match=KCTD3) | translation elongation factor activity;aminopeptidase activity;metalloexopeptidase activity;hydrolase activity;peptidase activity |  | **-1.36** |
| 1436812_AT | fukutin related protein | [FKRP](http://www.gene.ucl.ac.uk/cgi-bin/nomenclature/searchgenes.pl?field=symbol&anchor=equals&symbol_search=Search&number=100&format=html&sortby=symbol&match=FKRP) | translation elongation factor activity;GTP binding | [606596](http://www.ncbi.nlm.nih.gov/entrez/dispomim.cgi?id=606596) | **1.27** |
| 1436822_X_AT | hexokinase 1 | [HK1](http://www.gene.ucl.ac.uk/cgi-bin/nomenclature/searchgenes.pl?field=symbol&anchor=equals&symbol_search=Search&number=100&format=html&sortby=symbol&match=HK1) | translation elongation factor activity;GTP binding | [142600](http://www.ncbi.nlm.nih.gov/entrez/dispomim.cgi?id=142600) | **-1.49** |
| 1436824_X_AT | ring finger protein 26 | [RNF26](http://www.gene.ucl.ac.uk/cgi-bin/nomenclature/searchgenes.pl?field=symbol&anchor=equals&symbol_search=Search&number=100&format=html&sortby=symbol&match=RNF26) | translation elongation factor activity;GTP binding | [606130](http://www.ncbi.nlm.nih.gov/entrez/dispomim.cgi?id=606130) | **-1.4** |
| 1436833_X_AT | tubulin tyrosine ligase-like 1 | [TTLL1](http://www.gene.ucl.ac.uk/cgi-bin/nomenclature/searchgenes.pl?field=symbol&anchor=equals&symbol_search=Search&number=100&format=html&sortby=symbol&match=TTLL1) | translation elongation factor activity;GTP binding | [608955](http://www.ncbi.nlm.nih.gov/entrez/dispomim.cgi?id=608955) | **-2.4** |
| 1436834_X_AT | malate dehydrogenase 1, NAD (soluble) | [MDH1](http://www.gene.ucl.ac.uk/cgi-bin/nomenclature/searchgenes.pl?field=symbol&anchor=equals&symbol_search=Search&number=100&format=html&sortby=symbol&match=MDH1) | translation elongation factor activity;receptor activity | [154200](http://www.ncbi.nlm.nih.gov/entrez/dispomim.cgi?id=154200) | **1.2** |
| 1436836_X_AT | calponin 3, acidic | [CNN3](http://www.gene.ucl.ac.uk/cgi-bin/nomenclature/searchgenes.pl?field=symbol&anchor=equals&symbol_search=Search&number=100&format=html&sortby=symbol&match=CNN3) | translation factor activity, nucleic acid binding;carbon-nitrogen ligase activity, with glutamine as amido-N-donor;ligase activity;glutamyl-tRNA(Gln) amidotransferase activity;transferase activity | [602374](http://www.ncbi.nlm.nih.gov/entrez/dispomim.cgi?id=602374) | **-2.09** |
| 1436838_X_AT | coactosin-like 1 (Dictyostelium) | [COTL1](http://www.gene.ucl.ac.uk/cgi-bin/nomenclature/searchgenes.pl?field=symbol&anchor=equals&symbol_search=Search&number=100&format=html&sortby=symbol&match=COTL1) | translation initiation factor activity | [606748](http://www.ncbi.nlm.nih.gov/entrez/dispomim.cgi?id=606748) | **-3.65** |
| 1436840_X_AT | ribosomal protein L35 | [RPL35](http://www.gene.ucl.ac.uk/cgi-bin/nomenclature/searchgenes.pl?field=symbol&anchor=equals&symbol_search=Search&number=100&format=html&sortby=symbol&match=RPL35) | translation initiation factor activity |  | **-1.53** |
| 1436841_AT | RIKEN cDNA B230380D07 gene | [B230380D07RIK](http://www.gene.ucl.ac.uk/cgi-bin/nomenclature/searchgenes.pl?field=symbol&anchor=equals&symbol_search=Search&number=100&format=html&sortby=symbol&match=B230380D07RIK) | translation initiation factor activity |  | **1.23** |
| 1436847_S_AT | cell division cycle associated 8 | [CDCA8](http://www.gene.ucl.ac.uk/cgi-bin/nomenclature/searchgenes.pl?field=symbol&anchor=equals&symbol_search=Search&number=100&format=html&sortby=symbol&match=CDCA8) | translation initiation factor activity |  | **-3.16** |
| 1436858_AT | muscleblind-like 2 | [MBNL2](http://www.gene.ucl.ac.uk/cgi-bin/nomenclature/searchgenes.pl?field=symbol&anchor=equals&symbol_search=Search&number=100&format=html&sortby=symbol&match=MBNL2) | translation initiation factor activity | [607327](http://www.ncbi.nlm.nih.gov/entrez/dispomim.cgi?id=607327) | **1.41** |
| 1436874_X_AT | solute carrier family 25 (mitochondrial carrier, adenine nucleotide translocator), member 5 | [SLC25A5](http://www.gene.ucl.ac.uk/cgi-bin/nomenclature/searchgenes.pl?field=symbol&anchor=equals&symbol_search=Search&number=100&format=html&sortby=symbol&match=SLC25A5) | translation initiation factor activity | [300150](http://www.ncbi.nlm.nih.gov/entrez/dispomim.cgi?id=300150) | **-1.57** |
| 1436884_X_AT | gb:BB699868 /DB_XREF=gi:16048692 /DB_XREF=BB699868 |  | translation initiation factor activity |  | **-1.36** |
| 1436891_AT | gb:AW536567 /DB_XREF=gi:7178984 /DB_XREF=G0106A11- |  | translation initiation factor activity |  | **1.76** |
| 1436900_X_AT | leptin receptor gene-related protein | [LEPROT](http://www.gene.ucl.ac.uk/cgi-bin/nomenclature/searchgenes.pl?field=symbol&anchor=equals&symbol_search=Search&number=100&format=html&sortby=symbol&match=LEPROT) | translation initiation factor activity |  | **-1.37** |
| 1436902_X_AT | thymosin, beta 10 | [TMSB10](http://www.gene.ucl.ac.uk/cgi-bin/nomenclature/searchgenes.pl?field=symbol&anchor=equals&symbol_search=Search&number=100&format=html&sortby=symbol&match=TMSB10) | translation initiation factor activity | [188399](http://www.ncbi.nlm.nih.gov/entrez/dispomim.cgi?id=188399) | **-5.67** |
| 1436905_X_AT | lysosomal-associated protein transmembrane 5 | [LAPTM5](http://www.gene.ucl.ac.uk/cgi-bin/nomenclature/searchgenes.pl?field=symbol&anchor=equals&symbol_search=Search&number=100&format=html&sortby=symbol&match=LAPTM5) | translation initiation factor activity | [601476](http://www.ncbi.nlm.nih.gov/entrez/dispomim.cgi?id=601476) | **-4.25** |
| 1436907_AT | neuron navigator 1 | [NAV1](http://www.gene.ucl.ac.uk/cgi-bin/nomenclature/searchgenes.pl?field=symbol&anchor=equals&symbol_search=Search&number=100&format=html&sortby=symbol&match=NAV1) | translation initiation factor activity |  | **-1.34** |
| 1436908_AT | pericentriolar material 1 | [PCM1](http://www.gene.ucl.ac.uk/cgi-bin/nomenclature/searchgenes.pl?field=symbol&anchor=equals&symbol_search=Search&number=100&format=html&sortby=symbol&match=PCM1) | translation initiation factor activity | [600299](http://www.ncbi.nlm.nih.gov/entrez/dispomim.cgi?id=600299) | **1.21** |
| 1436915_X_AT | lysosomal-associated protein transmembrane 4B | [LAPTM4B](http://www.gene.ucl.ac.uk/cgi-bin/nomenclature/searchgenes.pl?field=symbol&anchor=equals&symbol_search=Search&number=100&format=html&sortby=symbol&match=LAPTM4B) | translation initiation factor activity;DNA binding;transcription factor activity;GTP binding |  | **1.28** |
| 1436919_AT | tumor protein p53 inducible protein 11 | [TP53I11](http://www.gene.ucl.ac.uk/cgi-bin/nomenclature/searchgenes.pl?field=symbol&anchor=equals&symbol_search=Search&number=100&format=html&sortby=symbol&match=TP53I11) | translation initiation factor activity;general RNA polymerase II transcription factor activity;RNA polymerase II transcription factor activity |  | **1.45** |
| 1436921_AT | ATPase, Cu++ transporting, alpha polypeptide | [ATP7A](http://www.gene.ucl.ac.uk/cgi-bin/nomenclature/searchgenes.pl?field=symbol&anchor=equals&symbol_search=Search&number=100&format=html&sortby=symbol&match=ATP7A) | translation initiation factor activity;helicase activity;DNA binding;ATP-dependent helicase activity;nucleic acid binding;RNA binding;ATP binding;hydrolase activity | [300011](http://www.ncbi.nlm.nih.gov/entrez/dispomim.cgi?id=300011) | **-1.65** |
| 1436926_AT | estrogen related receptor, beta | [ESRRB](http://www.gene.ucl.ac.uk/cgi-bin/nomenclature/searchgenes.pl?field=symbol&anchor=equals&symbol_search=Search&number=100&format=html&sortby=symbol&match=ESRRB) | translation initiation factor activity;kinase activity;protein kinase activity;protein serine/threonine kinase activity;ATP binding;protein-tyrosine kinase activity;transferase activity | [602167](http://www.ncbi.nlm.nih.gov/entrez/dispomim.cgi?id=602167) | **2.04** |
| 1436934_S_AT | aconitase 2, mitochondrial | [ACO2](http://www.gene.ucl.ac.uk/cgi-bin/nomenclature/searchgenes.pl?field=symbol&anchor=equals&symbol_search=Search&number=100&format=html&sortby=symbol&match=ACO2) | translation initiation factor activity;methyltransferase activity;transferase activity | [100850](http://www.ncbi.nlm.nih.gov/entrez/dispomim.cgi?id=100850) | **1.3** |
| 1436935_X_AT | chloride channel, nucleotide-sensitive, 1A | [CLNS1A](http://www.gene.ucl.ac.uk/cgi-bin/nomenclature/searchgenes.pl?field=symbol&anchor=equals&symbol_search=Search&number=100&format=html&sortby=symbol&match=CLNS1A) | translation initiation factor activity;protein binding;heat shock protein activity | [602158](http://www.ncbi.nlm.nih.gov/entrez/dispomim.cgi?id=602158) | **-1.29** |
| 1436938_AT | RNA binding motif, single stranded interacting protein | [RBMS3](http://www.gene.ucl.ac.uk/cgi-bin/nomenclature/searchgenes.pl?field=symbol&anchor=equals&symbol_search=Search&number=100&format=html&sortby=symbol&match=RBMS3) | translation initiation factor activity;RNA binding | [605786](http://www.ncbi.nlm.nih.gov/entrez/dispomim.cgi?id=605786) | **-1.4** |
| 1436939_AT | cardiomyopathy associated 4 | [CMYA4](http://www.gene.ucl.ac.uk/cgi-bin/nomenclature/searchgenes.pl?field=symbol&anchor=equals&symbol_search=Search&number=100&format=html&sortby=symbol&match=CMYA4) | translation initiation factor activity;RNA binding |  | **1.29** |
| 1436944_X_AT | RIKEN cDNA 9030221M09 gene | [MGC65558](http://www.gene.ucl.ac.uk/cgi-bin/nomenclature/searchgenes.pl?field=symbol&anchor=equals&symbol_search=Search&number=100&format=html&sortby=symbol&match=MGC65558) | translation initiation factor activity;RNA binding |  | **-3.89** |
| 1436946_S_AT | guanine nucleotide binding protein (G protein), gamma 5 subunit | [GNG5](http://www.gene.ucl.ac.uk/cgi-bin/nomenclature/searchgenes.pl?field=symbol&anchor=equals&symbol_search=Search&number=100&format=html&sortby=symbol&match=GNG5) | translation initiation factor activity;RNA binding |  | **1.24** |
| 1436959_X_AT | nasal embryonic LHRH factor | [NELF](http://www.gene.ucl.ac.uk/cgi-bin/nomenclature/searchgenes.pl?field=symbol&anchor=equals&symbol_search=Search&number=100&format=html&sortby=symbol&match=NELF) | translation initiation factor activity;RNA binding | [608137](http://www.ncbi.nlm.nih.gov/entrez/dispomim.cgi?id=608137) | **-1.93** |
| 1436961_AT | heat shock 70kDa protein 12A | [HSPA12A](http://www.gene.ucl.ac.uk/cgi-bin/nomenclature/searchgenes.pl?field=symbol&anchor=equals&symbol_search=Search&number=100&format=html&sortby=symbol&match=HSPA12A) | translation initiation factor activity;RNA binding;calcium ion binding |  | **1.85** |
| 1436971_X_AT | tyrosine 3-monooxygenase/tryptophan 5-monooxygenase activation protein, zeta polypeptide | [YWHAZ](http://www.gene.ucl.ac.uk/cgi-bin/nomenclature/searchgenes.pl?field=symbol&anchor=equals&symbol_search=Search&number=100&format=html&sortby=symbol&match=YWHAZ) | translation initiation factor activity;translation factor activity, nucleic acid binding;nucleic acid binding;RNA binding | [601288](http://www.ncbi.nlm.nih.gov/entrez/dispomim.cgi?id=601288) | **-1.4** |
| 1436981_A_AT | gb:BB706206 /DB_XREF=gi:16055041 /DB_XREF=BB706206 |  | translation initiation factor activity;translation factor activity, nucleic acid binding;nucleic acid binding;RNA binding |  | **-1.58** |
| 1436990_S_AT | Nur77 downstream gene 2 | [NDG2](http://www.gene.ucl.ac.uk/cgi-bin/nomenclature/searchgenes.pl?field=symbol&anchor=equals&symbol_search=Search&number=100&format=html&sortby=symbol&match=NDG2) | translation regulator activity;nucleic acid binding;DNA binding;mRNA binding |  | **1.4** |
| 1436996_X_AT | P lysozyme structural | [LZP-S](http://www.gene.ucl.ac.uk/cgi-bin/nomenclature/searchgenes.pl?field=symbol&anchor=equals&symbol_search=Search&number=100&format=html&sortby=symbol&match=LZP-S) | translation regulator activity;protein serine/threonine phosphatase activity |  | **-3.69** |
| 1436997_X_AT | SH3-binding domain glutamic acid-rich protein like | [SH3BGRL](http://www.gene.ucl.ac.uk/cgi-bin/nomenclature/searchgenes.pl?field=symbol&anchor=equals&symbol_search=Search&number=100&format=html&sortby=symbol&match=SH3BGRL) | translation repressor activity, nucleic acid binding | [300190](http://www.ncbi.nlm.nih.gov/entrez/dispomim.cgi?id=300190) | **-1.64** |
| 1436999_AT | expressed sequence AL024069 | [AL024069](http://www.gene.ucl.ac.uk/cgi-bin/nomenclature/searchgenes.pl?field=symbol&anchor=equals&symbol_search=Search&number=100&format=html&sortby=symbol&match=AL024069) | transmembrane receptor activity |  | **-1.47** |
| 1437005_A_AT | ribosomal protein L18 | [RPL18](http://www.gene.ucl.ac.uk/cgi-bin/nomenclature/searchgenes.pl?field=symbol&anchor=equals&symbol_search=Search&number=100&format=html&sortby=symbol&match=RPL18) | transmembrane receptor activity | [604179](http://www.ncbi.nlm.nih.gov/entrez/dispomim.cgi?id=604179) | **-1.36** |
| 1437009_A_AT | zinc finger protein 364 | [ZFP364](http://www.gene.ucl.ac.uk/cgi-bin/nomenclature/searchgenes.pl?field=symbol&anchor=equals&symbol_search=Search&number=100&format=html&sortby=symbol&match=ZFP364) | transmembrane receptor activity |  | **-1.38** |
| 1437073_X_AT | gb:BB115446 /DB_XREF=gi:8768014 /DB_XREF=BB115446 |  | transmembrane receptor activity |  | **-1.41** |
| 1437077_AT | Expressed sequence AW495713 | [AW495713](http://www.gene.ucl.ac.uk/cgi-bin/nomenclature/searchgenes.pl?field=symbol&anchor=equals&symbol_search=Search&number=100&format=html&sortby=symbol&match=AW495713) | transmembrane receptor activity |  | **1.54** |
| 1437100_X_AT | proviral integration site 3 | [PIM3](http://www.gene.ucl.ac.uk/cgi-bin/nomenclature/searchgenes.pl?field=symbol&anchor=equals&symbol_search=Search&number=100&format=html&sortby=symbol&match=PIM3) | transmembrane receptor activity;IgG binding;IgE binding;receptor activity |  | **1.62** |
| 1437104_AT | RIKEN cDNA D130059B05 gene | [ARFGEF1](http://www.gene.ucl.ac.uk/cgi-bin/nomenclature/searchgenes.pl?field=symbol&anchor=equals&symbol_search=Search&number=100&format=html&sortby=symbol&match=ARFGEF1) | transmembrane receptor activity;protein binding | [604141](http://www.ncbi.nlm.nih.gov/entrez/dispomim.cgi?id=604141) | **1.58** |
| 1437110_AT | RIKEN cDNA 2810474O19 gene | [2810474O19RIK](http://www.gene.ucl.ac.uk/cgi-bin/nomenclature/searchgenes.pl?field=symbol&anchor=equals&symbol_search=Search&number=100&format=html&sortby=symbol&match=2810474O19RIK) | transmembrane receptor activity;protein binding;antigen binding;receptor activity |  | **-1.93** |
| 1437123_AT | multimerin 2 | [MMRN2](http://www.gene.ucl.ac.uk/cgi-bin/nomenclature/searchgenes.pl?field=symbol&anchor=equals&symbol_search=Search&number=100&format=html&sortby=symbol&match=MMRN2) | transmembrane receptor activity;receptor activity | [608925](http://www.ncbi.nlm.nih.gov/entrez/dispomim.cgi?id=608925) | **1.91** |
| 1437141_X_AT | RIKEN cDNA 4933427L07 gene | [4933427L07RIK](http://www.gene.ucl.ac.uk/cgi-bin/nomenclature/searchgenes.pl?field=symbol&anchor=equals&symbol_search=Search&number=100&format=html&sortby=symbol&match=4933427L07RIK) | transmembrane receptor activity;receptor activity;G-protein coupled receptor activity;non-G-protein coupled 7TM receptor activity |  | **1.92** |
| 1437148_AT | actin related protein 2/3 complex, subunit 2 | [ARPC2](http://www.gene.ucl.ac.uk/cgi-bin/nomenclature/searchgenes.pl?field=symbol&anchor=equals&symbol_search=Search&number=100&format=html&sortby=symbol&match=ARPC2) | transmembrane receptor activity;receptor activity;G-protein coupled receptor activity;oxidoreductase activity;non-G-protein coupled 7TM receptor activity | [604224](http://www.ncbi.nlm.nih.gov/entrez/dispomim.cgi?id=604224) | **-1.34** |
| 1437161_X_AT | RNA binding protein gene with multiple splicing | [RBPMS](http://www.gene.ucl.ac.uk/cgi-bin/nomenclature/searchgenes.pl?field=symbol&anchor=equals&symbol_search=Search&number=100&format=html&sortby=symbol&match=RBPMS) | transmembrane receptor activity;receptor activity;lipopolysaccharide binding;catalytic activity | [601558](http://www.ncbi.nlm.nih.gov/entrez/dispomim.cgi?id=601558) | **1.47** |
| 1437165_A_AT | procollagen C-proteinase enhancer protein | [PCOLCE](http://www.gene.ucl.ac.uk/cgi-bin/nomenclature/searchgenes.pl?field=symbol&anchor=equals&symbol_search=Search&number=100&format=html&sortby=symbol&match=PCOLCE) | transmembrane receptor activity;receptor activity;lipopolysaccharide binding;catalytic activity | [600270](http://www.ncbi.nlm.nih.gov/entrez/dispomim.cgi?id=600270) | **-1.39** |
| 1437172_X_AT | hydroxyacyl-Coenzyme A dehydrogenase/3-ketoacyl-Coenzyme A thiolase/enoyl-Coenzyme A hydratase (trifunctional protein), beta subunit | [HADHB](http://www.gene.ucl.ac.uk/cgi-bin/nomenclature/searchgenes.pl?field=symbol&anchor=equals&symbol_search=Search&number=100&format=html&sortby=symbol&match=HADHB) | transmembrane receptor activity;receptor activity;triacylated lipoprotein binding | [143450](http://www.ncbi.nlm.nih.gov/entrez/dispomim.cgi?id=143450) | **1.56** |
| 1437180_AT | RIKEN cDNA 6530403A03 gene | [6530403A03RIK](http://www.gene.ucl.ac.uk/cgi-bin/nomenclature/searchgenes.pl?field=symbol&anchor=equals&symbol_search=Search&number=100&format=html&sortby=symbol&match=6530403A03RIK) | transmembrane receptor activity;structural constituent of ribosome |  | **-1.27** |
| 1437185_S_AT | thymosin, beta 10 | [TMSB10](http://www.gene.ucl.ac.uk/cgi-bin/nomenclature/searchgenes.pl?field=symbol&anchor=equals&symbol_search=Search&number=100&format=html&sortby=symbol&match=TMSB10) | transmembrane receptor activity;sugar binding;mannose binding;calcium ion binding;receptor activity | [188399](http://www.ncbi.nlm.nih.gov/entrez/dispomim.cgi?id=188399) | **-4.02** |
| 1437192_X_AT | voltage-dependent anion channel 1 | [VDAC1](http://www.gene.ucl.ac.uk/cgi-bin/nomenclature/searchgenes.pl?field=symbol&anchor=equals&symbol_search=Search&number=100&format=html&sortby=symbol&match=VDAC1) | transmembrane receptor activity;Wnt-protein binding | [604492](http://www.ncbi.nlm.nih.gov/entrez/dispomim.cgi?id=604492) | **1.27** |
| 1437194_X_AT | RIKEN cDNA 1200011O22 gene | [1200011O22RIK](http://www.gene.ucl.ac.uk/cgi-bin/nomenclature/searchgenes.pl?field=symbol&anchor=equals&symbol_search=Search&number=100&format=html&sortby=symbol&match=1200011O22RIK) | transmembrane receptor activity;Wnt-protein binding |  | **-1.29** |
| 1437197_AT | RIKEN cDNA 9430041O17 gene | [9430041O17RIK](http://www.gene.ucl.ac.uk/cgi-bin/nomenclature/searchgenes.pl?field=symbol&anchor=equals&symbol_search=Search&number=100&format=html&sortby=symbol&match=9430041O17RIK) | transporter activity |  | **-2.92** |
| 1437209_AT | RIKEN cDNA 6230425F05 gene | [6230425F05RIK](http://www.gene.ucl.ac.uk/cgi-bin/nomenclature/searchgenes.pl?field=symbol&anchor=equals&symbol_search=Search&number=100&format=html&sortby=symbol&match=6230425F05RIK) | transporter activity |  | **-1.61** |
| 1437216_AT | RIKEN cDNA A430106J12 gene | [A430106J12RIK](http://www.gene.ucl.ac.uk/cgi-bin/nomenclature/searchgenes.pl?field=symbol&anchor=equals&symbol_search=Search&number=100&format=html&sortby=symbol&match=A430106J12RIK) | transporter activity |  | **-1.78** |
| 1437224_AT | reticulon 4 | [RTN4](http://www.gene.ucl.ac.uk/cgi-bin/nomenclature/searchgenes.pl?field=symbol&anchor=equals&symbol_search=Search&number=100&format=html&sortby=symbol&match=RTN4) | transporter activity | [604475](http://www.ncbi.nlm.nih.gov/entrez/dispomim.cgi?id=604475) | **-5.4** |
| 1437226_X_AT | MARCKS-like protein | [MLP](http://www.gene.ucl.ac.uk/cgi-bin/nomenclature/searchgenes.pl?field=symbol&anchor=equals&symbol_search=Search&number=100&format=html&sortby=symbol&match=MLP) | transporter activity | [602940](http://www.ncbi.nlm.nih.gov/entrez/dispomim.cgi?id=602940) | **-2.26** |
| 1437234_X_AT | gb:BB357347 /DB_XREF=gi:9069175 /DB_XREF=BB357347 |  | transporter activity |  | **-2.06** |
| 1437239_X_AT | polyhomeotic-like 2 (Drosophila) | [PHC2](http://www.gene.ucl.ac.uk/cgi-bin/nomenclature/searchgenes.pl?field=symbol&anchor=equals&symbol_search=Search&number=100&format=html&sortby=symbol&match=PHC2) | transporter activity | [602979](http://www.ncbi.nlm.nih.gov/entrez/dispomim.cgi?id=602979) | **-1.32** |
| 1437242_AT | cDNA sequence BC055368 | [BC055368](http://www.gene.ucl.ac.uk/cgi-bin/nomenclature/searchgenes.pl?field=symbol&anchor=equals&symbol_search=Search&number=100&format=html&sortby=symbol&match=BC055368) | transporter activity |  | **-1.42** |
| 1437250_AT | whn-dependent transcript 2 | [WDT2](http://www.gene.ucl.ac.uk/cgi-bin/nomenclature/searchgenes.pl?field=symbol&anchor=equals&symbol_search=Search&number=100&format=html&sortby=symbol&match=WDT2) | transporter activity |  | **2.91** |
| 1437252_AT | opposite strand transcription unit to Stag3 | [GATS](http://www.gene.ucl.ac.uk/cgi-bin/nomenclature/searchgenes.pl?field=symbol&anchor=equals&symbol_search=Search&number=100&format=html&sortby=symbol&match=GATS) | transporter activity |  | **1.35** |
| 1437259_AT | solute carrier family 9 (sodium/hydrogen exchanger), member 2 | [SLC9A2](http://www.gene.ucl.ac.uk/cgi-bin/nomenclature/searchgenes.pl?field=symbol&anchor=equals&symbol_search=Search&number=100&format=html&sortby=symbol&match=SLC9A2) | transporter activity | [600530](http://www.ncbi.nlm.nih.gov/entrez/dispomim.cgi?id=600530) | **1.54** |
| 1437279_X_AT | syndecan 1 | [SDC1](http://www.gene.ucl.ac.uk/cgi-bin/nomenclature/searchgenes.pl?field=symbol&anchor=equals&symbol_search=Search&number=100&format=html&sortby=symbol&match=SDC1) | transporter activity;ATPase activity;ATPase activity, coupled to transmembrane movement of substances;ATP-binding cassette (ABC) transporter activity;ATP binding | [186355](http://www.ncbi.nlm.nih.gov/entrez/dispomim.cgi?id=186355) | **-2.2** |
| 1437287_AT | RIKEN cDNA 1110020G09 gene | [1110020G09RIK](http://www.gene.ucl.ac.uk/cgi-bin/nomenclature/searchgenes.pl?field=symbol&anchor=equals&symbol_search=Search&number=100&format=html&sortby=symbol&match=1110020G09RIK) | transporter activity;ATPase activity;ATP-binding cassette (ABC) transporter activity;ATPase activity, coupled to transmembrane movement of substances;ATP binding |  | **2.27** |
| 1437308_S_AT | coagulation factor II (thrombin) receptor | [F2R](http://www.gene.ucl.ac.uk/cgi-bin/nomenclature/searchgenes.pl?field=symbol&anchor=equals&symbol_search=Search&number=100&format=html&sortby=symbol&match=F2R) | transporter activity;binding | [187930](http://www.ncbi.nlm.nih.gov/entrez/dispomim.cgi?id=187930) | **-1.26** |
| 1437313_X_AT | High mobility group box 2 | [HMGB2](http://www.gene.ucl.ac.uk/cgi-bin/nomenclature/searchgenes.pl?field=symbol&anchor=equals&symbol_search=Search&number=100&format=html&sortby=symbol&match=HMGB2) | transporter activity;binding | [163906](http://www.ncbi.nlm.nih.gov/entrez/dispomim.cgi?id=163906) | **-1.72** |
| 1437325_X_AT | pyrroline-5-carboxylate synthetase (glutamate gamma-semialdehyde synthetase) | [PYCS](http://www.gene.ucl.ac.uk/cgi-bin/nomenclature/searchgenes.pl?field=symbol&anchor=equals&symbol_search=Search&number=100&format=html&sortby=symbol&match=PYCS) | transporter activity;binding | [138250](http://www.ncbi.nlm.nih.gov/entrez/dispomim.cgi?id=138250) | **-2.38** |
| 1437331_A_AT | ADP-ribosylation factor 3 | [ARF3](http://www.gene.ucl.ac.uk/cgi-bin/nomenclature/searchgenes.pl?field=symbol&anchor=equals&symbol_search=Search&number=100&format=html&sortby=symbol&match=ARF3) | transporter activity;binding | [103190](http://www.ncbi.nlm.nih.gov/entrez/dispomim.cgi?id=103190) | **-1.45** |
| 1437355_AT | zinc finger, CCHC domain containing 5 | [ZCCHC5](http://www.gene.ucl.ac.uk/cgi-bin/nomenclature/searchgenes.pl?field=symbol&anchor=equals&symbol_search=Search&number=100&format=html&sortby=symbol&match=ZCCHC5) | transporter activity;binding |  | **-3.83** |
| 1437363_AT | homer homolog 1 (Drosophila) | [HOMER1](http://www.gene.ucl.ac.uk/cgi-bin/nomenclature/searchgenes.pl?field=symbol&anchor=equals&symbol_search=Search&number=100&format=html&sortby=symbol&match=HOMER1) | transporter activity;binding | [604798](http://www.ncbi.nlm.nih.gov/entrez/dispomim.cgi?id=604798) | **1.34** |
| 1437385_AT | gb:AV264768 /DB_XREF=gi:16389245 /DB_XREF=AV264768 |  | transporter activity;binding |  | **-1.75** |
| 1437401_AT | gb:BG075165 /DB_XREF=gi:12557734 /DB_XREF=H3144A06 |  | transporter activity;binding |  | **-1.68** |
| 1437433_AT | UDP-Gal:betaGlcNAc beta 1,3-galactosyltransferase, polypeptide 2 | [B3GALT2](http://www.gene.ucl.ac.uk/cgi-bin/nomenclature/searchgenes.pl?field=symbol&anchor=equals&symbol_search=Search&number=100&format=html&sortby=symbol&match=B3GALT2) | transporter activity;binding | [603018](http://www.ncbi.nlm.nih.gov/entrez/dispomim.cgi?id=603018) | **-2.59** |
| 1437442_AT | gb:BG067986 /DB_XREF=gi:12550555 /DB_XREF=H3060E02 |  | transporter activity;binding |  | **-1.68** |
| 1437455_A_AT | B-cell translocation gene 1, anti-proliferative | [BTG1](http://www.gene.ucl.ac.uk/cgi-bin/nomenclature/searchgenes.pl?field=symbol&anchor=equals&symbol_search=Search&number=100&format=html&sortby=symbol&match=BTG1) | transporter activity;binding | [109580](http://www.ncbi.nlm.nih.gov/entrez/dispomim.cgi?id=109580) | **-1.74** |
| 1437457_A_AT | myotrophin | [MTPN](http://www.gene.ucl.ac.uk/cgi-bin/nomenclature/searchgenes.pl?field=symbol&anchor=equals&symbol_search=Search&number=100&format=html&sortby=symbol&match=MTPN) | transporter activity;binding | [606484](http://www.ncbi.nlm.nih.gov/entrez/dispomim.cgi?id=606484) | **-1.52** |
| 1437465_A_AT | prolyl 4-hydroxylase, beta polypeptide | [P4HB](http://www.gene.ucl.ac.uk/cgi-bin/nomenclature/searchgenes.pl?field=symbol&anchor=equals&symbol_search=Search&number=100&format=html&sortby=symbol&match=P4HB) | transporter activity;binding | [176790](http://www.ncbi.nlm.nih.gov/entrez/dispomim.cgi?id=176790) | **-1.3** |
| 1437467_AT | activated leukocyte cell adhesion molecule | [ALCAM](http://www.gene.ucl.ac.uk/cgi-bin/nomenclature/searchgenes.pl?field=symbol&anchor=equals&symbol_search=Search&number=100&format=html&sortby=symbol&match=ALCAM) | transporter activity;calcium ion binding;solute:cation symporter activity;binding | [601662](http://www.ncbi.nlm.nih.gov/entrez/dispomim.cgi?id=601662) | **-2.24** |
| 1437476_AT | ribonucleotide reductase M2 B (TP53 inducible) | [RRM2B](http://www.gene.ucl.ac.uk/cgi-bin/nomenclature/searchgenes.pl?field=symbol&anchor=equals&symbol_search=Search&number=100&format=html&sortby=symbol&match=RRM2B) | transporter activity;catalytic activity | [604712](http://www.ncbi.nlm.nih.gov/entrez/dispomim.cgi?id=604712) | **1.52** |
| 1437478_S_AT | EF hand domain containing 2 | [EFHD2](http://www.gene.ucl.ac.uk/cgi-bin/nomenclature/searchgenes.pl?field=symbol&anchor=equals&symbol_search=Search&number=100&format=html&sortby=symbol&match=EFHD2) | transporter activity;catalytic activity |  | **-2.05** |
| 1437479_X_AT | T-box 3 | [TBX3](http://www.gene.ucl.ac.uk/cgi-bin/nomenclature/searchgenes.pl?field=symbol&anchor=equals&symbol_search=Search&number=100&format=html&sortby=symbol&match=TBX3) | transporter activity;cysteine-type endopeptidase activity;cysteine-type peptidase activity | [601621](http://www.ncbi.nlm.nih.gov/entrez/dispomim.cgi?id=601621) | **1.71** |
| 1437482_AT | steroid 5 alpha-reductase 2-like 2 | [SRD5A2L2](http://www.gene.ucl.ac.uk/cgi-bin/nomenclature/searchgenes.pl?field=symbol&anchor=equals&symbol_search=Search&number=100&format=html&sortby=symbol&match=SRD5A2L2) | transporter activity;DNA binding |  | **1.75** |
| 1437486_AT | retinoic acid induced 3 | [RAI3](http://www.gene.ucl.ac.uk/cgi-bin/nomenclature/searchgenes.pl?field=symbol&anchor=equals&symbol_search=Search&number=100&format=html&sortby=symbol&match=RAI3) | transporter activity;enzyme activator activity;GTPase activity;GTP binding;protein transporter activity | [604138](http://www.ncbi.nlm.nih.gov/entrez/dispomim.cgi?id=604138) | **1.65** |
| 1437489_X_AT | succinate dehydrogenase complex, subunit D, integral membrane protein | [SDHD](http://www.gene.ucl.ac.uk/cgi-bin/nomenclature/searchgenes.pl?field=symbol&anchor=equals&symbol_search=Search&number=100&format=html&sortby=symbol&match=SDHD) | transporter activity;glycine amidinotransferase activity;receptor activity;transferase activity | [602690](http://www.ncbi.nlm.nih.gov/entrez/dispomim.cgi?id=602690) | **1.43** |
| 1437496_AT | RIKEN cDNA 9330129D05 gene | [9330129D05RIK](http://www.gene.ucl.ac.uk/cgi-bin/nomenclature/searchgenes.pl?field=symbol&anchor=equals&symbol_search=Search&number=100&format=html&sortby=symbol&match=9330129D05RIK) | transporter activity;GTP binding;protein transporter activity |  | **1.54** |
| 1437497_A_AT | heat shock protein 1, alpha | [HSPCA](http://www.gene.ucl.ac.uk/cgi-bin/nomenclature/searchgenes.pl?field=symbol&anchor=equals&symbol_search=Search&number=100&format=html&sortby=symbol&match=HSPCA) | transporter activity;GTP binding;protein transporter activity | [140571](http://www.ncbi.nlm.nih.gov/entrez/dispomim.cgi?id=140571) | **-1.6** |
| 1437498_AT | gb:BB327336 /DB_XREF=gi:16403742 /DB_XREF=BB327336 |  | transporter activity;GTPase activity;enzyme activator activity;GTP binding;protein transporter activity |  | **1.55** |
| 1437503_A_AT | scotin gene | [SCOTIN](http://www.gene.ucl.ac.uk/cgi-bin/nomenclature/searchgenes.pl?field=symbol&anchor=equals&symbol_search=Search&number=100&format=html&sortby=symbol&match=SCOTIN) | transporter activity;GTPase activity;GTP binding | [607290](http://www.ncbi.nlm.nih.gov/entrez/dispomim.cgi?id=607290) | **-1.56** |
| 1437521_S_AT | RIKEN cDNA E230022H04 gene | [E230022H04RIK](http://www.gene.ucl.ac.uk/cgi-bin/nomenclature/searchgenes.pl?field=symbol&anchor=equals&symbol_search=Search&number=100&format=html&sortby=symbol&match=E230022H04RIK) | transporter activity;GTPase activity;GTP binding |  | **-1.36** |
| 1437552_AT | RIKEN cDNA 2410127L17 gene | [2410127L17RIK](http://www.gene.ucl.ac.uk/cgi-bin/nomenclature/searchgenes.pl?field=symbol&anchor=equals&symbol_search=Search&number=100&format=html&sortby=symbol&match=2410127L17RIK) | transporter activity;hydrogen-exporting ATPase activity, phosphorylative mechanism;hydrogen-transporting ATP synthase activity, rotational mechanism;ATPase activity;hydrogen ion transporter activity;hydrogen-transporting ATPase activity, rotational mechanism;hydrolase activity |  | **1.62** |
| 1437584_AT | gb:BE685667 /DB_XREF=gi:10073343 /DB_XREF=uu57b06. |  | transporter activity;hydrogen-transporting ATP synthase activity, rotational mechanism;hydrogen ion transporter activity;hydrogen-transporting ATPase activity, rotational mechanism;hydrolase activity |  | **-3.21** |
| 1437608_X_AT | gb:BB414446 /DB_XREF=gi:9235801 /DB_XREF=BB414446 |  | transporter activity;lipid binding;binding |  | **-1.35** |

| 1437610_X_AT | ribosomal protein S8 | [RPS8](http://www.gene.ucl.ac.uk/cgi-bin/nomenclature/searchgenes.pl?field=symbol&anchor=equals&symbol_search=Search&number=100&format=html&sortby=symbol&match=RPS8) | transporter activity;lipid binding;binding | [600357](http://www.ncbi.nlm.nih.gov/entrez/dispomim.cgi?id=600357) | **-1.51** |
| --- | --- | --- | --- | --- | --- |
| 1437615_S_AT | RIKEN cDNA 5730409F24 gene | [5730409F24RIK](http://www.gene.ucl.ac.uk/cgi-bin/nomenclature/searchgenes.pl?field=symbol&anchor=equals&symbol_search=Search&number=100&format=html&sortby=symbol&match=5730409F24RIK) | transporter activity;lipid binding;binding |  | **-1.45** |
| 1437621_X_AT | 3-phosphoglycerate dehydrogenase | [PHGDH](http://www.gene.ucl.ac.uk/cgi-bin/nomenclature/searchgenes.pl?field=symbol&anchor=equals&symbol_search=Search&number=100&format=html&sortby=symbol&match=PHGDH) | transporter activity;lipid binding;binding | [606879](http://www.ncbi.nlm.nih.gov/entrez/dispomim.cgi?id=606879) | **-2.18** |
| 1437624_X_AT | RIKEN cDNA 1110001K21 gene | [1110001K21RIK](http://www.gene.ucl.ac.uk/cgi-bin/nomenclature/searchgenes.pl?field=symbol&anchor=equals&symbol_search=Search&number=100&format=html&sortby=symbol&match=1110001K21RIK) | transporter activity;lipid binding;binding |  | **-1.36** |
| 1437637_AT | putative homeodomain transcription factor 2 | [PHTF2](http://www.gene.ucl.ac.uk/cgi-bin/nomenclature/searchgenes.pl?field=symbol&anchor=equals&symbol_search=Search&number=100&format=html&sortby=symbol&match=PHTF2) | transporter activity;lipid binding;binding |  | **1.38** |
| 1437643_AT | centromere protein J | [CENPJ](http://www.gene.ucl.ac.uk/cgi-bin/nomenclature/searchgenes.pl?field=symbol&anchor=equals&symbol_search=Search&number=100&format=html&sortby=symbol&match=CENPJ) | transporter activity;protein transporter activity |  | **-1.46** |
| 1437649_X_AT | peptidylprolyl isomerase B | [PPIB](http://www.gene.ucl.ac.uk/cgi-bin/nomenclature/searchgenes.pl?field=symbol&anchor=equals&symbol_search=Search&number=100&format=html&sortby=symbol&match=PPIB) | transporter activity;receptor activity | [123841](http://www.ncbi.nlm.nih.gov/entrez/dispomim.cgi?id=123841) | **-1.93** |
| 1437670_X_AT | CD151 antigen | [CD151](http://www.gene.ucl.ac.uk/cgi-bin/nomenclature/searchgenes.pl?field=symbol&anchor=equals&symbol_search=Search&number=100&format=html&sortby=symbol&match=CD151) | transporter activity;receptor activity | [602243](http://www.ncbi.nlm.nih.gov/entrez/dispomim.cgi?id=602243) | **-1.27** |
| 1437688_X_AT | ATPase, H+ transporting, lysosomal accessory protein 2 | [ATP6AP2](http://www.gene.ucl.ac.uk/cgi-bin/nomenclature/searchgenes.pl?field=symbol&anchor=equals&symbol_search=Search&number=100&format=html&sortby=symbol&match=ATP6AP2) | transporter activity;receptor activity;signal peptidase activity;hydrolase activity;peptidase activity |  | **-1.5** |
| 1437706_X_AT | gb:AW548239 /DB_XREF=gi:7193660 /DB_XREF=L0033D09- |  | transporter activity;sugar binding;calcium ion binding;receptor activity;collagen binding |  | **-1.26** |
| 1437709_X_AT | tetratricopeptide repeat domain 13 | [TTC13](http://www.gene.ucl.ac.uk/cgi-bin/nomenclature/searchgenes.pl?field=symbol&anchor=equals&symbol_search=Search&number=100&format=html&sortby=symbol&match=TTC13) | transporter activity;sugar porter activity;glucose transporter activity;insulin-responsive hydrogen:glucose transporter activity |  | **-1.43** |
| 1437711_X_AT | gb:BB519474 /DB_XREF=gi:9570932 /DB_XREF=BB519474 |  | transporter activity;water channel activity |  | **-1.56** |
| 1437716_X_AT | kinesin family member 22 | [KIF22](http://www.gene.ucl.ac.uk/cgi-bin/nomenclature/searchgenes.pl?field=symbol&anchor=equals&symbol_search=Search&number=100&format=html&sortby=symbol&match=KIF22) | tRNA binding | [603213](http://www.ncbi.nlm.nih.gov/entrez/dispomim.cgi?id=603213) | **-1.87** |
| 1437719_X_AT | RIKEN cDNA A230046K03 gene | [A230046K03RIK](http://www.gene.ucl.ac.uk/cgi-bin/nomenclature/searchgenes.pl?field=symbol&anchor=equals&symbol_search=Search&number=100&format=html&sortby=symbol&match=A230046K03RIK) | trypsin activity;calcium ion binding;serine-type endopeptidase activity;hydrolase activity;peptidase activity;chymotrypsin activity |  | **-1.68** |
| 1437724_X_AT | phosphatidylinositol membrane-associated 1 | [PITPNM1](http://www.gene.ucl.ac.uk/cgi-bin/nomenclature/searchgenes.pl?field=symbol&anchor=equals&symbol_search=Search&number=100&format=html&sortby=symbol&match=PITPNM1) | trypsin activity;classical-complement-pathway C3/C5 convertase activity;serine-type endopeptidase activity;hydrolase activity;chymotrypsin activity;peptidase activity | [608794](http://www.ncbi.nlm.nih.gov/entrez/dispomim.cgi?id=608794) | **-1.51** |
| 1437726_X_AT | complement component 1, q subcomponent, beta polypeptide | [C1QB](http://www.gene.ucl.ac.uk/cgi-bin/nomenclature/searchgenes.pl?field=symbol&anchor=equals&symbol_search=Search&number=100&format=html&sortby=symbol&match=C1QB) | trypsin activity;protein binding;insulin-like growth factor binding;serine-type endopeptidase activity;hydrolase activity;peptidase activity | [120570](http://www.ncbi.nlm.nih.gov/entrez/dispomim.cgi?id=120570) | **-4.07** |
| 1437730_AT | protein phosphatase 2 (formerly 2A), regulatory subunit B (PR 52), alpha isoform | [PPP2R2A](http://www.gene.ucl.ac.uk/cgi-bin/nomenclature/searchgenes.pl?field=symbol&anchor=equals&symbol_search=Search&number=100&format=html&sortby=symbol&match=PPP2R2A) | trypsin activity;protein binding;tryptase activity;serine-type endopeptidase activity;heparin binding;hydrolase activity;chymotrypsin activity | [604941](http://www.ncbi.nlm.nih.gov/entrez/dispomim.cgi?id=604941) | **1.28** |
| 1437742_AT | RAB21, member RAS oncogene family | [RAB21](http://www.gene.ucl.ac.uk/cgi-bin/nomenclature/searchgenes.pl?field=symbol&anchor=equals&symbol_search=Search&number=100&format=html&sortby=symbol&match=RAB21) | trypsin activity;serine-type endopeptidase activity;hydrolase activity;chymotrypsin activity;peptidase activity |  | **1.31** |
| 1437765_AT | gb:BB249892 /DB_XREF=gi:8942638 /DB_XREF=BB249892 |  | tubulin-tyrosine ligase activity;nucleic acid binding;ligase activity |  | **1.66** |
| 1437773_X_AT | DEAD (Asp-Glu-Ala-Asp) box polypeptide 17 | [DDX17](http://www.gene.ucl.ac.uk/cgi-bin/nomenclature/searchgenes.pl?field=symbol&anchor=equals&symbol_search=Search&number=100&format=html&sortby=symbol&match=DDX17) | tumor necrosis factor receptor activity;receptor activity | [608469](http://www.ncbi.nlm.nih.gov/entrez/dispomim.cgi?id=608469) | **-1.3** |
| 1437774_AT | RIKEN cDNA 1700020I14 gene | [1700020I14RIK](http://www.gene.ucl.ac.uk/cgi-bin/nomenclature/searchgenes.pl?field=symbol&anchor=equals&symbol_search=Search&number=100&format=html&sortby=symbol&match=1700020I14RIK) | two-component sensor molecule activity |  | **1.39** |
| 1437783_X_AT | transmembrane protein 4 | [TMEM4](http://www.gene.ucl.ac.uk/cgi-bin/nomenclature/searchgenes.pl?field=symbol&anchor=equals&symbol_search=Search&number=100&format=html&sortby=symbol&match=TMEM4) | ubiquitin activating enzyme activity;ligase activity;catalytic activity;ATP binding;NEDD8 activating enzyme activity | [605861](http://www.ncbi.nlm.nih.gov/entrez/dispomim.cgi?id=605861) | **-1.43** |
| 1437811_X_AT | gb:AV312368 /DB_XREF=gi:6277620 /DB_XREF=AV312368 |  | ubiquitin activating enzyme activity;ligase activity;catalytic activity;ATP binding;NEDD8 activating enzyme activity |  | **-3** |
| 1437838_X_AT | G-rich RNA sequence binding factor 1 | [GRSF1](http://www.gene.ucl.ac.uk/cgi-bin/nomenclature/searchgenes.pl?field=symbol&anchor=equals&symbol_search=Search&number=100&format=html&sortby=symbol&match=GRSF1) | ubiquitin thiolesterase activity;cysteine-type endopeptidase activity;hydrolase activity;cysteine-type peptidase activity | [604851](http://www.ncbi.nlm.nih.gov/entrez/dispomim.cgi?id=604851) | **1.26** |
| 1437843_S_AT | gb:AV303002 /DB_XREF=gi:6335516 /DB_XREF=AV303002 |  | ubiquitin thiolesterase activity;cysteine-type endopeptidase activity;hydrolase activity;cysteine-type peptidase activity |  | **-1.27** |
| 1437849_X_AT | armadillo repeat containing, X-linked 2 | [ARMCX2](http://www.gene.ucl.ac.uk/cgi-bin/nomenclature/searchgenes.pl?field=symbol&anchor=equals&symbol_search=Search&number=100&format=html&sortby=symbol&match=ARMCX2) | ubiquitin thiolesterase activity;cysteine-type endopeptidase activity;hydrolase activity;cysteine-type peptidase activity;peptidase activity | [300363](http://www.ncbi.nlm.nih.gov/entrez/dispomim.cgi?id=300363) | **-1.33** |
| 1437861_S_AT | gb:BB335101 /DB_XREF=gi:16404093 /DB_XREF=BB335101 |  | ubiquitin thiolesterase activity;cysteine-type endopeptidase activity;hydrolase activity;peptidase activity;cysteine-type peptidase activity |  | **1.4** |
| 1437863_AT | gb:BB667762 /DB_XREF=gi:16399211 /DB_XREF=BB667762 |  | ubiquitin thiolesterase activity;cysteine-type endopeptidase activity;ubiquitin-specific protease activity;hydrolase activity;peptidase activity;cysteine-type peptidase activity |  | **1.66** |
| 1437868_AT | gb:BE687858 /DB_XREF=gi:10075482 /DB_XREF=uw52c03. |  | ubiquitin-like activating enzyme activity;ubiquitin activating enzyme activity;ligase activity;catalytic activity;ATP binding |  | **-1.75** |
| 1437874_S_AT | hexosaminidase B | [HEXB](http://www.gene.ucl.ac.uk/cgi-bin/nomenclature/searchgenes.pl?field=symbol&anchor=equals&symbol_search=Search&number=100&format=html&sortby=symbol&match=HEXB) | ubiquitin-protein ligase activity | [606873](http://www.ncbi.nlm.nih.gov/entrez/dispomim.cgi?id=606873) | **-2.51** |
| 1437875_AT | gb:AV328403 /DB_XREF=gi:16395867 /DB_XREF=AV328403 |  | ubiquitin-protein ligase activity |  | **-1.26** |
| 1437878_S_AT | gb:BB711506 /DB_XREF=gi:16064675 /DB_XREF=BB711506 |  | UDP-activated nucleotide receptor activity;purinergic nucleotide receptor activity, G-protein coupled;receptor activity;G-protein coupled receptor activity;rhodopsin-like receptor activity;pyrimidine nucleotide binding |  | **-1.24** |
| 1437889_X_AT | biglycan | [BGN](http://www.gene.ucl.ac.uk/cgi-bin/nomenclature/searchgenes.pl?field=symbol&anchor=equals&symbol_search=Search&number=100&format=html&sortby=symbol&match=BGN) | UDP-sugar diphosphatase activity;metal ion binding;magnesium ion binding;hydrolase activity, acting on acid anhydrides, in phosphorus-containing anhydrides;hydrolase activity | [301870](http://www.ncbi.nlm.nih.gov/entrez/dispomim.cgi?id=301870) | **-3.16** |
| 1437902_S_AT | retinoic acid receptor responder (tazarotene induced) 2 | [RARRES2](http://www.gene.ucl.ac.uk/cgi-bin/nomenclature/searchgenes.pl?field=symbol&anchor=equals&symbol_search=Search&number=100&format=html&sortby=symbol&match=RARRES2) | uracil DNA N-glycosylase activity;hydrolase activity, acting on glycosyl bonds;hydrolase activity | [601973](http://www.ncbi.nlm.nih.gov/entrez/dispomim.cgi?id=601973) | **1.47** |
| 1437912_AT | IQ motif and Sec7 domain 3 | [IQSEC3](http://www.gene.ucl.ac.uk/cgi-bin/nomenclature/searchgenes.pl?field=symbol&anchor=equals&symbol_search=Search&number=100&format=html&sortby=symbol&match=IQSEC3) | uroporphyrinogen decarboxylase activity;carboxy-lyase activity;lyase activity |  | **-2.33** |
| 1437917_AT | RIKEN cDNA D530037H12 gene | [D530037H12RIK](http://www.gene.ucl.ac.uk/cgi-bin/nomenclature/searchgenes.pl?field=symbol&anchor=equals&symbol_search=Search&number=100&format=html&sortby=symbol&match=D530037H12RIK) | vascular endothelial growth factor receptor binding |  | **1.42** |
| 1437939_S_AT | gb:BM237633 /DB_XREF=gi:17872903 /DB_XREF=K0504E12 |  | vitamin-K-epoxide reductase (warfarin-sensitive) activity |  | **-1.71** |
| 1437945_X_AT | nucleosome assembly protein 1-like 1 | [NAP1L1](http://www.gene.ucl.ac.uk/cgi-bin/nomenclature/searchgenes.pl?field=symbol&anchor=equals&symbol_search=Search&number=100&format=html&sortby=symbol&match=NAP1L1) | voltage-gated chloride channel activity;voltage-gated ion channel activity;ion channel activity | [164060](http://www.ncbi.nlm.nih.gov/entrez/dispomim.cgi?id=164060) | **-1.41** |
| 1437947_X_AT | gb:AV036172 /DB_XREF=gi:4855837 /DB_XREF=AV036172 |  | voltage-gated ion channel activity;ion channel activity;oxidoreductase activity |  | **1.42** |
| 1437967_AT | gb:AV365582 /DB_XREF=gi:15406733 /DB_XREF=AV365582 |  | water channel activity;transporter activity |  | **-3.26** |
| 1437974_A_AT | hexokinase 1 | [HK1](http://www.gene.ucl.ac.uk/cgi-bin/nomenclature/searchgenes.pl?field=symbol&anchor=equals&symbol_search=Search&number=100&format=html&sortby=symbol&match=HK1) | water channel activity;water transporter activity | [142600](http://www.ncbi.nlm.nih.gov/entrez/dispomim.cgi?id=142600) | **-2.04** |
| 1438000_X_AT | zinc finger protein 622 | [ZFP622](http://www.gene.ucl.ac.uk/cgi-bin/nomenclature/searchgenes.pl?field=symbol&anchor=equals&symbol_search=Search&number=100&format=html&sortby=symbol&match=ZFP622) | zinc ion binding |  | **-1.4** |
| 1438001_X_AT | deleted in polyposis 1 | [DP1](http://www.gene.ucl.ac.uk/cgi-bin/nomenclature/searchgenes.pl?field=symbol&anchor=equals&symbol_search=Search&number=100&format=html&sortby=symbol&match=DP1) | zinc ion binding | [125265](http://www.ncbi.nlm.nih.gov/entrez/dispomim.cgi?id=125265) | **-1.61** |
| 1438006_AT | RIKEN cDNA 4933439F18 gene | [4933439F18RIK](http://www.gene.ucl.ac.uk/cgi-bin/nomenclature/searchgenes.pl?field=symbol&anchor=equals&symbol_search=Search&number=100&format=html&sortby=symbol&match=4933439F18RIK) | zinc ion binding |  | **1.51** |
| 1438009_AT | histone 1, H2ad | [HIST1H2AD](http://www.gene.ucl.ac.uk/cgi-bin/nomenclature/searchgenes.pl?field=symbol&anchor=equals&symbol_search=Search&number=100&format=html&sortby=symbol&match=HIST1H2AD) | zinc ion binding | [602792](http://www.ncbi.nlm.nih.gov/entrez/dispomim.cgi?id=602792) | **-4.92** |
| 1438011_AT | phosphate cytidylyltransferase 1, choline, alpha isoform | [PCYT1A](http://www.gene.ucl.ac.uk/cgi-bin/nomenclature/searchgenes.pl?field=symbol&anchor=equals&symbol_search=Search&number=100&format=html&sortby=symbol&match=PCYT1A) | zinc ion binding | [123695](http://www.ncbi.nlm.nih.gov/entrez/dispomim.cgi?id=123695) | **1.31** |
| 1438012_AT | protein phosphatase 1 (formerly 2C)-like | [PPM1L](http://www.gene.ucl.ac.uk/cgi-bin/nomenclature/searchgenes.pl?field=symbol&anchor=equals&symbol_search=Search&number=100&format=html&sortby=symbol&match=PPM1L) | zinc ion binding |  | **1.55** |
| 1438018_AT | hook homolog 1 (Drosophila) | [HOOK1](http://www.gene.ucl.ac.uk/cgi-bin/nomenclature/searchgenes.pl?field=symbol&anchor=equals&symbol_search=Search&number=100&format=html&sortby=symbol&match=HOOK1) | zinc ion binding | [607820](http://www.ncbi.nlm.nih.gov/entrez/dispomim.cgi?id=607820) | **1.45** |
| 1438022_AT | RIKEN cDNA D030060O14 gene | [RAB11FIP3](http://www.gene.ucl.ac.uk/cgi-bin/nomenclature/searchgenes.pl?field=symbol&anchor=equals&symbol_search=Search&number=100&format=html&sortby=symbol&match=RAB11FIP3) | zinc ion binding;actin binding |  | **1.78** |
| 1438024_AT | gb:AW554392 /DB_XREF=gi:7199815 /DB_XREF=L0240A10- |  | zinc ion binding;actin binding |  | **1.25** |
| 1438030_AT | RAS, guanyl releasing protein 3 | [RASGRP3](http://www.gene.ucl.ac.uk/cgi-bin/nomenclature/searchgenes.pl?field=symbol&anchor=equals&symbol_search=Search&number=100&format=html&sortby=symbol&match=RASGRP3) | zinc ion binding;alcohol dehydrogenase activity, zinc-dependent |  | **1.87** |
| 1438042_AT | short stature homeobox 2 | [SHOX2](http://www.gene.ucl.ac.uk/cgi-bin/nomenclature/searchgenes.pl?field=symbol&anchor=equals&symbol_search=Search&number=100&format=html&sortby=symbol&match=SHOX2) | zinc ion binding;alcohol dehydrogenase activity, zinc-dependent | [602504](http://www.ncbi.nlm.nih.gov/entrez/dispomim.cgi?id=602504) | **1.62** |
| 1438048_AT | gb:BB531986 /DB_XREF=gi:16446014 /DB_XREF=BB531986 |  | zinc ion binding;alcohol dehydrogenase activity, zinc-dependent |  | **1.69** |
| 1438058_S_AT | prostate tumor over expressed gene 1 | [PTOV1](http://www.gene.ucl.ac.uk/cgi-bin/nomenclature/searchgenes.pl?field=symbol&anchor=equals&symbol_search=Search&number=100&format=html&sortby=symbol&match=PTOV1) | zinc ion binding;calcium ion binding;metallopeptidase activity;metalloendopeptidase activity;gelatinase A activity;hydrolase activity |  | **1.25** |
| 1438059_AT | Transcribed locus, weakly similar to NP_899138.1 cortexin [Mus musculus] |  | zinc ion binding;calcium ion binding;metallopeptidase activity;metalloendopeptidase activity;macrophage elastase activity;hydrolase activity;peptidase activity |  | **-3.95** |
| 1438081_AT | gb:BB794635 /DB_XREF=gi:16964254 /DB_XREF=BB794635 |  | zinc ion binding;carbonate dehydratase activity;lyase activity |  | **1.93** |
| 1438091_A_AT | H2A histone family, member Z | [H2AFZ](http://www.gene.ucl.ac.uk/cgi-bin/nomenclature/searchgenes.pl?field=symbol&anchor=equals&symbol_search=Search&number=100&format=html&sortby=symbol&match=H2AFZ) | zinc ion binding;carbonate dehydratase activity;lyase activity | [142763](http://www.ncbi.nlm.nih.gov/entrez/dispomim.cgi?id=142763) | **-1.52** |
| 1438092_X_AT | gb:AV003424 /DB_XREF=gi:4780274 /DB_XREF=AV003424 |  | zinc ion binding;carbonate dehydratase activity;lyase activity |  | **-1.46** |
| 1438094_X_AT | RIKEN cDNA 2810409H07 gene | [2810409H07RIK](http://www.gene.ucl.ac.uk/cgi-bin/nomenclature/searchgenes.pl?field=symbol&anchor=equals&symbol_search=Search&number=100&format=html&sortby=symbol&match=2810409H07RIK) | zinc ion binding;carboxypeptidase activity;carboxypeptidase A activity;metallocarboxypeptidase activity;hydrolase activity |  | **-1.41** |
| 1438096_A_AT | deoxythymidylate kinase | [DTYMK](http://www.gene.ucl.ac.uk/cgi-bin/nomenclature/searchgenes.pl?field=symbol&anchor=equals&symbol_search=Search&number=100&format=html&sortby=symbol&match=DTYMK) | zinc ion binding;carboxypeptidase activity;carboxypeptidase A activity;metallocarboxypeptidase activity;hydrolase activity | [188345](http://www.ncbi.nlm.nih.gov/entrez/dispomim.cgi?id=188345) | **-1.43** |
| 1438118_X_AT | vimentin | [VIM](http://www.gene.ucl.ac.uk/cgi-bin/nomenclature/searchgenes.pl?field=symbol&anchor=equals&symbol_search=Search&number=100&format=html&sortby=symbol&match=VIM) | zinc ion binding;cytidine deaminase activity;hydrolase activity | [193060](http://www.ncbi.nlm.nih.gov/entrez/dispomim.cgi?id=193060) | **-1.85** |
| 1438168_X_AT | DEAD (Asp-Glu-Ala-Asp) box polypeptide 39 | [DDX39](http://www.gene.ucl.ac.uk/cgi-bin/nomenclature/searchgenes.pl?field=symbol&anchor=equals&symbol_search=Search&number=100&format=html&sortby=symbol&match=DDX39) | zinc ion binding;DNA binding;nucleic acid binding |  | **-1.88** |
| 1438175_X_AT | myomesin 2 | [MYOM2](http://www.gene.ucl.ac.uk/cgi-bin/nomenclature/searchgenes.pl?field=symbol&anchor=equals&symbol_search=Search&number=100&format=html&sortby=symbol&match=MYOM2) | zinc ion binding;DNA binding;nucleic acid binding | [603509](http://www.ncbi.nlm.nih.gov/entrez/dispomim.cgi?id=603509) | **-1.92** |
| 1438177_X_AT | ectonucleoside triphosphate diphosphohydrolase 4 | [ENTPD4](http://www.gene.ucl.ac.uk/cgi-bin/nomenclature/searchgenes.pl?field=symbol&anchor=equals&symbol_search=Search&number=100&format=html&sortby=symbol&match=ENTPD4) | zinc ion binding;DNA binding;nucleic acid binding | [607577](http://www.ncbi.nlm.nih.gov/entrez/dispomim.cgi?id=607577) | **1.46** |
| 1438183_X_AT | sorbitol dehydrogenase 1 | [SDH1](http://www.gene.ucl.ac.uk/cgi-bin/nomenclature/searchgenes.pl?field=symbol&anchor=equals&symbol_search=Search&number=100&format=html&sortby=symbol&match=SDH1) | zinc ion binding;DNA binding;nucleic acid binding |  | **1.85** |
| 1438206_A_AT | RIKEN cDNA 2610042O14 gene | [2610042O14RIK](http://www.gene.ucl.ac.uk/cgi-bin/nomenclature/searchgenes.pl?field=symbol&anchor=equals&symbol_search=Search&number=100&format=html&sortby=symbol&match=2610042O14RIK) | zinc ion binding;DNA binding;nucleic acid binding |  | **1.36** |
| 1438214_AT | trichorhinophalangeal syndrome I (human) | [TRPS1](http://www.gene.ucl.ac.uk/cgi-bin/nomenclature/searchgenes.pl?field=symbol&anchor=equals&symbol_search=Search&number=100&format=html&sortby=symbol&match=TRPS1) | zinc ion binding;DNA binding;nucleic acid binding | [604386](http://www.ncbi.nlm.nih.gov/entrez/dispomim.cgi?id=604386) | **-1.44** |
| 1438233_AT | RIKEN cDNA 6030443O07 gene | [6030443O07RIK](http://www.gene.ucl.ac.uk/cgi-bin/nomenclature/searchgenes.pl?field=symbol&anchor=equals&symbol_search=Search&number=100&format=html&sortby=symbol&match=6030443O07RIK) | zinc ion binding;DNA binding;nucleic acid binding |  | **-1.47** |
| 1438250_S_AT | TAF9 RNA polymerase II, TATA box binding protein (TBP)-associated factor | [TAF9](http://www.gene.ucl.ac.uk/cgi-bin/nomenclature/searchgenes.pl?field=symbol&anchor=equals&symbol_search=Search&number=100&format=html&sortby=symbol&match=TAF9) | zinc ion binding;DNA binding;nucleic acid binding;transcription factor activity | [600822](http://www.ncbi.nlm.nih.gov/entrez/dispomim.cgi?id=600822) | **-1.37** |
| 1438283_AT | RIKEN cDNA 3110057O12 gene | [3110057O12RIK](http://www.gene.ucl.ac.uk/cgi-bin/nomenclature/searchgenes.pl?field=symbol&anchor=equals&symbol_search=Search&number=100&format=html&sortby=symbol&match=3110057O12RIK) | zinc ion binding;DNA binding;nucleic acid binding;transcription factor activity;transcriptional activator activity;transcriptional repressor activity |  | **1.6** |
| 1438292_X_AT | adenosine kinase | [ADK](http://www.gene.ucl.ac.uk/cgi-bin/nomenclature/searchgenes.pl?field=symbol&anchor=equals&symbol_search=Search&number=100&format=html&sortby=symbol&match=ADK) | zinc ion binding;DNA binding;structural constituent of ribosome | [102750](http://www.ncbi.nlm.nih.gov/entrez/dispomim.cgi?id=102750) | **1.7** |
| 1438294_AT | spinocerebellar ataxia 1 homolog (human) | [SCA1](http://www.gene.ucl.ac.uk/cgi-bin/nomenclature/searchgenes.pl?field=symbol&anchor=equals&symbol_search=Search&number=100&format=html&sortby=symbol&match=SCA1) | zinc ion binding;DNA binding;structural constituent of ribosome | [601556](http://www.ncbi.nlm.nih.gov/entrez/dispomim.cgi?id=601556) | **1.31** |
| 1438312_S_AT | latent transforming growth factor beta binding protein 3 | [LTBP3](http://www.gene.ucl.ac.uk/cgi-bin/nomenclature/searchgenes.pl?field=symbol&anchor=equals&symbol_search=Search&number=100&format=html&sortby=symbol&match=LTBP3) | zinc ion binding;DNA binding;transcription factor activity;ubiquitin-protein ligase activity | [602090](http://www.ncbi.nlm.nih.gov/entrez/dispomim.cgi?id=602090) | **-1.22** |
| 1438317_A_AT | endonuclease G | [ENDOG](http://www.gene.ucl.ac.uk/cgi-bin/nomenclature/searchgenes.pl?field=symbol&anchor=equals&symbol_search=Search&number=100&format=html&sortby=symbol&match=ENDOG) | zinc ion binding;DNA binding;transcriptional activator activity | [600440](http://www.ncbi.nlm.nih.gov/entrez/dispomim.cgi?id=600440) | **1.54** |
| 1438318_X_AT | RIKEN cDNA 1500001L15 gene | [1500001L15RIK](http://www.gene.ucl.ac.uk/cgi-bin/nomenclature/searchgenes.pl?field=symbol&anchor=equals&symbol_search=Search&number=100&format=html&sortby=symbol&match=1500001L15RIK) | zinc ion binding;DNA binding;ubiquitin-protein ligase activity |  | **-1.31** |
| 1438320_S_AT | minichromosome maintenance deficient 7 (S. cerevisiae) | [MCM7](http://www.gene.ucl.ac.uk/cgi-bin/nomenclature/searchgenes.pl?field=symbol&anchor=equals&symbol_search=Search&number=100&format=html&sortby=symbol&match=MCM7) | zinc ion binding;DNA binding;ubiquitin-protein ligase activity | [600592](http://www.ncbi.nlm.nih.gov/entrez/dispomim.cgi?id=600592) | **-1.78** |
| 1438322_X_AT | farnesyl diphosphate farnesyl transferase 1 | [FDFT1](http://www.gene.ucl.ac.uk/cgi-bin/nomenclature/searchgenes.pl?field=symbol&anchor=equals&symbol_search=Search&number=100&format=html&sortby=symbol&match=FDFT1) | zinc ion binding;DNA binding;ubiquitin-protein ligase activity | [184420](http://www.ncbi.nlm.nih.gov/entrez/dispomim.cgi?id=184420) | **2.1** |
| 1438325_AT | ecotropic viral integration site 1 | [EVI1](http://www.gene.ucl.ac.uk/cgi-bin/nomenclature/searchgenes.pl?field=symbol&anchor=equals&symbol_search=Search&number=100&format=html&sortby=symbol&match=EVI1) | zinc ion binding;hydrolase activity, acting on carbon-nitrogen (but not peptide) bonds, in cyclic amidines;hydrolase activity | [165215](http://www.ncbi.nlm.nih.gov/entrez/dispomim.cgi?id=165215) | **1.81** |
| 1438360_X_AT | solute carrier family 25 (mitochondrial carrier, adenine nucleotide translocator), member 5 | [SLC25A5](http://www.gene.ucl.ac.uk/cgi-bin/nomenclature/searchgenes.pl?field=symbol&anchor=equals&symbol_search=Search&number=100&format=html&sortby=symbol&match=SLC25A5) | zinc ion binding;hydrolase activity, acting on carbon-nitrogen (but not peptide) bonds, in cyclic amidines;hydrolase activity | [300150](http://www.ncbi.nlm.nih.gov/entrez/dispomim.cgi?id=300150) | **-1.92** |
| 1438366_X_AT | chloride channel 3 | [CLCN3](http://www.gene.ucl.ac.uk/cgi-bin/nomenclature/searchgenes.pl?field=symbol&anchor=equals&symbol_search=Search&number=100&format=html&sortby=symbol&match=CLCN3) | zinc ion binding;L-iditol 2-dehydrogenase activity;oxidoreductase activity;alcohol dehydrogenase activity, zinc-dependent | [600580](http://www.ncbi.nlm.nih.gov/entrez/dispomim.cgi?id=600580) | **1.72** |
| 1438368_A_AT | matrin 3 | [MATR3](http://www.gene.ucl.ac.uk/cgi-bin/nomenclature/searchgenes.pl?field=symbol&anchor=equals&symbol_search=Search&number=100&format=html&sortby=symbol&match=MATR3) | zinc ion binding;L-iditol 2-dehydrogenase activity;oxidoreductase activity;alcohol dehydrogenase activity, zinc-dependent | [604706](http://www.ncbi.nlm.nih.gov/entrez/dispomim.cgi?id=604706) | **-1.3** |
| 1438385_S_AT | glutamic pyruvate transaminase (alanine aminotransferase) 2 | [GPT2](http://www.gene.ucl.ac.uk/cgi-bin/nomenclature/searchgenes.pl?field=symbol&anchor=equals&symbol_search=Search&number=100&format=html&sortby=symbol&match=GPT2) | zinc ion binding;ligase activity;ubiquitin-protein ligase activity | [607438](http://www.ncbi.nlm.nih.gov/entrez/dispomim.cgi?id=607438) | **1.41** |
| 1438386_X_AT | methionine adenosyltransferase II, alpha | [MAT2A](http://www.gene.ucl.ac.uk/cgi-bin/nomenclature/searchgenes.pl?field=symbol&anchor=equals&symbol_search=Search&number=100&format=html&sortby=symbol&match=MAT2A) | zinc ion binding;ligase activity;ubiquitin-protein ligase activity | [601468](http://www.ncbi.nlm.nih.gov/entrez/dispomim.cgi?id=601468) | **-1.28** |
| 1438391_X_AT | hydroxyacyl-Coenzyme A dehydrogenase type II | [HADH2](http://www.gene.ucl.ac.uk/cgi-bin/nomenclature/searchgenes.pl?field=symbol&anchor=equals&symbol_search=Search&number=100&format=html&sortby=symbol&match=HADH2) | zinc ion binding;ligase activity;ubiquitin-protein ligase activity | [300256](http://www.ncbi.nlm.nih.gov/entrez/dispomim.cgi?id=300256) | **1.34** |
| 1438422_AT | leucine rich repeat containing 20 | [LRRC20](http://www.gene.ucl.ac.uk/cgi-bin/nomenclature/searchgenes.pl?field=symbol&anchor=equals&symbol_search=Search&number=100&format=html&sortby=symbol&match=LRRC20) | zinc ion binding;ligase activity;ubiquitin-protein ligase activity;cytoskeletal protein binding |  | **1.58** |
| 1438426_AT | RIKEN cDNA 9330132O05 gene | [9330132O05RIK](http://www.gene.ucl.ac.uk/cgi-bin/nomenclature/searchgenes.pl?field=symbol&anchor=equals&symbol_search=Search&number=100&format=html&sortby=symbol&match=9330132O05RIK) | zinc ion binding;ligase activity;ubiquitin-protein ligase activity;peptidase activity |  | **-1.31** |
| 1438429_AT | RIKEN cDNA 2610319H10 gene | [2610319H10RIK](http://www.gene.ucl.ac.uk/cgi-bin/nomenclature/searchgenes.pl?field=symbol&anchor=equals&symbol_search=Search&number=100&format=html&sortby=symbol&match=2610319H10RIK) | zinc ion binding;membrane alanyl aminopeptidase activity;metallopeptidase activity;aminopeptidase activity;hydrolase activity |  | **-2.45** |
| 1438431_AT | gb:BB197269 /DB_XREF=gi:16271050 /DB_XREF=BB197269 |  | zinc ion binding;metal ion binding |  | **1.87** |
| 1438435_AT | gb:BB329313 /DB_XREF=gi:15411508 /DB_XREF=BB329313 |  | zinc ion binding;metal ion binding;copper ion binding |  | **-1.86** |
| 1438477_A_AT | Methylmalonyl CoA epimerase | [MCEE](http://www.gene.ucl.ac.uk/cgi-bin/nomenclature/searchgenes.pl?field=symbol&anchor=equals&symbol_search=Search&number=100&format=html&sortby=symbol&match=MCEE) | zinc ion binding;metallopeptidase activity;calcium ion binding;gelatinase A activity;metalloendopeptidase activity;hydrolase activity | [608419](http://www.ncbi.nlm.nih.gov/entrez/dispomim.cgi?id=608419) | **1.61** |
| 1438480_A_AT | thymocyte protein thy28 | [THY28](http://www.gene.ucl.ac.uk/cgi-bin/nomenclature/searchgenes.pl?field=symbol&anchor=equals&symbol_search=Search&number=100&format=html&sortby=symbol&match=THY28) | zinc ion binding;metallopeptidase activity;carboxypeptidase activity;carboxypeptidase A activity;metallocarboxypeptidase activity;binding;hydrolase activity |  | **-1.52** |
| 1438483_AT | gb:BB794641 /DB_XREF=gi:16964260 /DB_XREF=BB794641 |  | zinc ion binding;metallopeptidase activity;metalloendopeptidase activity;hydrolase activity |  | **4.2** |
| 1438512_AT | epididymal protein Av381126 | [BC048679](http://www.gene.ucl.ac.uk/cgi-bin/nomenclature/searchgenes.pl?field=symbol&anchor=equals&symbol_search=Search&number=100&format=html&sortby=symbol&match=BC048679) | zinc ion binding;metallopeptidase activity;metalloendopeptidase activity;hydrolase activity |  | **9.77** |
| 1438546_X_AT | solute carrier family 25 (mitochondrial carrier, adenine nucleotide translocator), member 5 | [SLC25A5](http://www.gene.ucl.ac.uk/cgi-bin/nomenclature/searchgenes.pl?field=symbol&anchor=equals&symbol_search=Search&number=100&format=html&sortby=symbol&match=SLC25A5) | zinc ion binding;metallopeptidase activity;metalloendopeptidase activity;hydrolase activity;peptidase activity | [300150](http://www.ncbi.nlm.nih.gov/entrez/dispomim.cgi?id=300150) | **-1.35** |
| 1438548_X_AT | RIKEN cDNA 5730411O18 gene | [5730411O18RIK](http://www.gene.ucl.ac.uk/cgi-bin/nomenclature/searchgenes.pl?field=symbol&anchor=equals&symbol_search=Search&number=100&format=html&sortby=symbol&match=5730411O18RIK) | zinc ion binding;metallopeptidase activity;metalloendopeptidase activity;hydrolase activity;peptidase activity |  | **1.21** |
| 1438559_X_AT | RIKEN cDNA 1110028E10 gene | [1110028E10RIK](http://www.gene.ucl.ac.uk/cgi-bin/nomenclature/searchgenes.pl?field=symbol&anchor=equals&symbol_search=Search&number=100&format=html&sortby=symbol&match=1110028E10RIK) | zinc ion binding;metallopeptidase activity;metalloendopeptidase activity;hydrolase activity;peptidase activity |  | **-1.36** |
| 1438560_X_AT | chaperonin subunit 4 (delta) | [CCT4](http://www.gene.ucl.ac.uk/cgi-bin/nomenclature/searchgenes.pl?field=symbol&anchor=equals&symbol_search=Search&number=100&format=html&sortby=symbol&match=CCT4) | zinc ion binding;metallopeptidase activity;metalloendopeptidase activity;procollagen N-endopeptidase activity;hydrolase activity | [605142](http://www.ncbi.nlm.nih.gov/entrez/dispomim.cgi?id=605142) | **-1.3** |
| 1438577_AT | gb:BB376947 /DB_XREF=gi:16407560 /DB_XREF=BB376947 |  | zinc ion binding;microtubule binding |  | **1.79** |
| 1438626_X_AT | ribosomal protein L14 | [RPL14](http://www.gene.ucl.ac.uk/cgi-bin/nomenclature/searchgenes.pl?field=symbol&anchor=equals&symbol_search=Search&number=100&format=html&sortby=symbol&match=RPL14) | zinc ion binding;molecular_function unknown;DNA binding;nucleic acid binding |  | **-1.41** |
| 1438629_X_AT | granulin | [GRN](http://www.gene.ucl.ac.uk/cgi-bin/nomenclature/searchgenes.pl?field=symbol&anchor=equals&symbol_search=Search&number=100&format=html&sortby=symbol&match=GRN) | zinc ion binding;molecular_function unknown;nucleic acid binding | [138945](http://www.ncbi.nlm.nih.gov/entrez/dispomim.cgi?id=138945) | **-2.06** |
| 1438630_X_AT | methionine adenosyltransferase II, alpha | [MAT2A](http://www.gene.ucl.ac.uk/cgi-bin/nomenclature/searchgenes.pl?field=symbol&anchor=equals&symbol_search=Search&number=100&format=html&sortby=symbol&match=MAT2A) | zinc ion binding;NADPH:quinone reductase activity;oxidoreductase activity;alcohol dehydrogenase activity, zinc-dependent | [601468](http://www.ncbi.nlm.nih.gov/entrez/dispomim.cgi?id=601468) | **-1.33** |
| 1438631_X_AT | tetratricopeptide repeat domain 13 | [TTC13](http://www.gene.ucl.ac.uk/cgi-bin/nomenclature/searchgenes.pl?field=symbol&anchor=equals&symbol_search=Search&number=100&format=html&sortby=symbol&match=TTC13) | zinc ion binding;nucleic acid binding |  | **-1.47** |
| 1438639_X_AT | RIKEN cDNA 4933417E01 gene | [4933417E01RIK](http://www.gene.ucl.ac.uk/cgi-bin/nomenclature/searchgenes.pl?field=symbol&anchor=equals&symbol_search=Search&number=100&format=html&sortby=symbol&match=4933417E01RIK) | zinc ion binding;nucleic acid binding |  | **1.46** |
| 1438641_X_AT | RIKEN cDNA 1500016O10 gene | [1500016O10RIK](http://www.gene.ucl.ac.uk/cgi-bin/nomenclature/searchgenes.pl?field=symbol&anchor=equals&symbol_search=Search&number=100&format=html&sortby=symbol&match=1500016O10RIK) | zinc ion binding;nucleic acid binding |  | **1.7** |
| 1438653_X_AT | gb:AV309164 /DB_XREF=gi:6362171 /DB_XREF=AV309164 |  | zinc ion binding;nucleic acid binding |  | **-1.51** |
| 1438655_A_AT | RIKEN cDNA 1100001I22 gene | [1100001I22RIK](http://www.gene.ucl.ac.uk/cgi-bin/nomenclature/searchgenes.pl?field=symbol&anchor=equals&symbol_search=Search&number=100&format=html&sortby=symbol&match=1100001I22RIK) | zinc ion binding;nucleic acid binding |  | **-1.44** |
| 1438660_AT | glucosaminyl (N-acetyl) transferase 2, I-branching enzyme | [GCNT2](http://www.gene.ucl.ac.uk/cgi-bin/nomenclature/searchgenes.pl?field=symbol&anchor=equals&symbol_search=Search&number=100&format=html&sortby=symbol&match=GCNT2) | zinc ion binding;nucleic acid binding | [600429](http://www.ncbi.nlm.nih.gov/entrez/dispomim.cgi?id=600429) | **1.42** |
| 1438666_AT | expressed sequence AI194318 | [AI194318](http://www.gene.ucl.ac.uk/cgi-bin/nomenclature/searchgenes.pl?field=symbol&anchor=equals&symbol_search=Search&number=100&format=html&sortby=symbol&match=AI194318) | zinc ion binding;nucleic acid binding |  | **-1.65** |
| 1438667_AT | RIKEN cDNA 5730410E15 gene | [5730410E15RIK](http://www.gene.ucl.ac.uk/cgi-bin/nomenclature/searchgenes.pl?field=symbol&anchor=equals&symbol_search=Search&number=100&format=html&sortby=symbol&match=5730410E15RIK) | zinc ion binding;nucleic acid binding |  | **1.53** |
| 1438673_AT | Solute carrier family 4, sodium bicarbonate cotransporter, member 7 | [SLC4A7](http://www.gene.ucl.ac.uk/cgi-bin/nomenclature/searchgenes.pl?field=symbol&anchor=equals&symbol_search=Search&number=100&format=html&sortby=symbol&match=SLC4A7) | zinc ion binding;nucleic acid binding | [603353](http://www.ncbi.nlm.nih.gov/entrez/dispomim.cgi?id=603353) | **-1.26** |
| 1438678_AT | RIKEN cDNA 1500011K16 gene | [1500011K16RIK](http://www.gene.ucl.ac.uk/cgi-bin/nomenclature/searchgenes.pl?field=symbol&anchor=equals&symbol_search=Search&number=100&format=html&sortby=symbol&match=1500011K16RIK) | zinc ion binding;nucleic acid binding |  | **1.52** |
| 1438714_AT | gb:BB622498 /DB_XREF=gi:16461523 /DB_XREF=BB622498 |  | zinc ion binding;nucleic acid binding |  | **-1.36** |
| 1438754_AT | gb:AV372127 /DB_XREF=gi:16397873 /DB_XREF=AV372127 |  | zinc ion binding;nucleic acid binding |  | **-1.59** |
| 1438758_AT | gb:AU046270 /DB_XREF=gi:3982453 /DB_XREF=AU046270 |  | zinc ion binding;nucleic acid binding |  | **1.81** |
| 1438759_X_AT | gb:AU046270 /DB_XREF=gi:3982453 /DB_XREF=AU046270 |  | zinc ion binding;nucleic acid binding |  | **1.78** |
| 1438761_A_AT | ornithine decarboxylase, structural 1 | [ODC1](http://www.gene.ucl.ac.uk/cgi-bin/nomenclature/searchgenes.pl?field=symbol&anchor=equals&symbol_search=Search&number=100&format=html&sortby=symbol&match=ODC1) | zinc ion binding;nucleic acid binding | [165640](http://www.ncbi.nlm.nih.gov/entrez/dispomim.cgi?id=165640) | **-1.61** |
| 1438852_X_AT | minichromosome maintenance deficient 6 (MIS5 homolog, S. pombe) (S. cerevisiae) | [MCM6](http://www.gene.ucl.ac.uk/cgi-bin/nomenclature/searchgenes.pl?field=symbol&anchor=equals&symbol_search=Search&number=100&format=html&sortby=symbol&match=MCM6) | zinc ion binding;nucleic acid binding | [601806](http://www.ncbi.nlm.nih.gov/entrez/dispomim.cgi?id=601806) | **-1.83** |
| 1438853_X_AT | DEAD (Asp-Glu-Ala-Asp) box polypeptide 54 | [DDX54](http://www.gene.ucl.ac.uk/cgi-bin/nomenclature/searchgenes.pl?field=symbol&anchor=equals&symbol_search=Search&number=100&format=html&sortby=symbol&match=DDX54) | zinc ion binding;nucleic acid binding |  | **-1.28** |
| 1438855_X_AT | gb:BB233088 /DB_XREF=gi:8912943 /DB_XREF=BB233088 |  | zinc ion binding;nucleic acid binding |  | **-1.5** |
| 1438858_X_AT | gb:AV018723 /DB_XREF=gi:4795715 /DB_XREF=AV018723 |  | zinc ion binding;nucleic acid binding |  | **-3.29** |
| 1438861_AT | basonuclin 2 | [BNC2](http://www.gene.ucl.ac.uk/cgi-bin/nomenclature/searchgenes.pl?field=symbol&anchor=equals&symbol_search=Search&number=100&format=html&sortby=symbol&match=BNC2) | zinc ion binding;nucleic acid binding;DNA binding | [608669](http://www.ncbi.nlm.nih.gov/entrez/dispomim.cgi?id=608669) | **-1.73** |
| 1438902_A_AT | heat shock protein 1, alpha | [HSPCA](http://www.gene.ucl.ac.uk/cgi-bin/nomenclature/searchgenes.pl?field=symbol&anchor=equals&symbol_search=Search&number=100&format=html&sortby=symbol&match=HSPCA) | zinc ion binding;nucleic acid binding;DNA binding | [140571](http://www.ncbi.nlm.nih.gov/entrez/dispomim.cgi?id=140571) | **-1.59** |
| 1438910_A_AT | erythrocyte protein band 7.2 | [STOM](http://www.gene.ucl.ac.uk/cgi-bin/nomenclature/searchgenes.pl?field=symbol&anchor=equals&symbol_search=Search&number=100&format=html&sortby=symbol&match=STOM) | zinc ion binding;nucleic acid binding;DNA binding | [133090](http://www.ncbi.nlm.nih.gov/entrez/dispomim.cgi?id=133090) | **1.38** |
| 1438922_X_AT | solute carrier family 25 (mitochondrial carrier, adenine nucleotide translocator), member 5 | [SLC25A5](http://www.gene.ucl.ac.uk/cgi-bin/nomenclature/searchgenes.pl?field=symbol&anchor=equals&symbol_search=Search&number=100&format=html&sortby=symbol&match=SLC25A5) | zinc ion binding;nucleic acid binding;DNA binding | [300150](http://www.ncbi.nlm.nih.gov/entrez/dispomim.cgi?id=300150) | **-1.67** |
| 1438925_X_AT | ATPase, H+ transporting, V0 subunit C | [ATP6V0C](http://www.gene.ucl.ac.uk/cgi-bin/nomenclature/searchgenes.pl?field=symbol&anchor=equals&symbol_search=Search&number=100&format=html&sortby=symbol&match=ATP6V0C) | zinc ion binding;nucleic acid binding;DNA binding | [108745](http://www.ncbi.nlm.nih.gov/entrez/dispomim.cgi?id=108745) | **-1.33** |
| 1438933_X_AT | RAS, guanyl releasing protein 2 | [RASGRP2](http://www.gene.ucl.ac.uk/cgi-bin/nomenclature/searchgenes.pl?field=symbol&anchor=equals&symbol_search=Search&number=100&format=html&sortby=symbol&match=RASGRP2) | zinc ion binding;nucleic acid binding;DNA binding;transcription factor activity | [605577](http://www.ncbi.nlm.nih.gov/entrez/dispomim.cgi?id=605577) | **1.58** |
| 1438940_X_AT | high mobility group nucleosomal binding domain 1 | [HMGN1](http://www.gene.ucl.ac.uk/cgi-bin/nomenclature/searchgenes.pl?field=symbol&anchor=equals&symbol_search=Search&number=100&format=html&sortby=symbol&match=HMGN1) | zinc ion binding;nucleic acid binding;DNA binding;transcription factor activity;transcription regulator activity | [163920](http://www.ncbi.nlm.nih.gov/entrez/dispomim.cgi?id=163920) | **-1.5** |
| 1438941_X_AT | adenosine monophosphate deaminase 2 (isoform L) | [AMPD2](http://www.gene.ucl.ac.uk/cgi-bin/nomenclature/searchgenes.pl?field=symbol&anchor=equals&symbol_search=Search&number=100&format=html&sortby=symbol&match=AMPD2) | zinc ion binding;nucleic acid binding;DNA binding;transcription factor activity;transcriptional activator activity;transcriptional repressor activity | [102771](http://www.ncbi.nlm.nih.gov/entrez/dispomim.cgi?id=102771) | **-1.98** |
| 1438943_X_AT | ribophorin I | [RPN1](http://www.gene.ucl.ac.uk/cgi-bin/nomenclature/searchgenes.pl?field=symbol&anchor=equals&symbol_search=Search&number=100&format=html&sortby=symbol&match=RPN1) | zinc ion binding;nucleic acid binding;RNA binding | [180470](http://www.ncbi.nlm.nih.gov/entrez/dispomim.cgi?id=180470) | **-1.42** |
| 1438948_X_AT | benzodiazepine receptor, peripheral | [BZRP](http://www.gene.ucl.ac.uk/cgi-bin/nomenclature/searchgenes.pl?field=symbol&anchor=equals&symbol_search=Search&number=100&format=html&sortby=symbol&match=BZRP) | zinc ion binding;nucleic acid binding;RNA binding | [109610](http://www.ncbi.nlm.nih.gov/entrez/dispomim.cgi?id=109610) | **-2.33** |
| 1438957_X_AT | CDP-diacylglycerol synthase (phosphatidate cytidylyltransferase) 2 | [CDS2](http://www.gene.ucl.ac.uk/cgi-bin/nomenclature/searchgenes.pl?field=symbol&anchor=equals&symbol_search=Search&number=100&format=html&sortby=symbol&match=CDS2) | zinc ion binding;nucleic acid binding;RNA binding | [603549](http://www.ncbi.nlm.nih.gov/entrez/dispomim.cgi?id=603549) | **1.48** |
| 1438961_S_AT | bleomycin hydrolase | [BLMH](http://www.gene.ucl.ac.uk/cgi-bin/nomenclature/searchgenes.pl?field=symbol&anchor=equals&symbol_search=Search&number=100&format=html&sortby=symbol&match=BLMH) | zinc ion binding;nucleic acid binding;RNA binding | [602403](http://www.ncbi.nlm.nih.gov/entrez/dispomim.cgi?id=602403) | **-1.81** |
| 1438967_X_AT | anti-Mullerian hormone type 2 receptor | [AMHR2](http://www.gene.ucl.ac.uk/cgi-bin/nomenclature/searchgenes.pl?field=symbol&anchor=equals&symbol_search=Search&number=100&format=html&sortby=symbol&match=AMHR2) | zinc ion binding;nucleic acid binding;transcription factor activity | [600956](http://www.ncbi.nlm.nih.gov/entrez/dispomim.cgi?id=600956) | **1.58** |
| 1438976_X_AT | methionine adenosyltransferase II, alpha | [MAT2A](http://www.gene.ucl.ac.uk/cgi-bin/nomenclature/searchgenes.pl?field=symbol&anchor=equals&symbol_search=Search&number=100&format=html&sortby=symbol&match=MAT2A) | zinc ion binding;nucleic acid binding;ubiquitin-protein ligase activity | [601468](http://www.ncbi.nlm.nih.gov/entrez/dispomim.cgi?id=601468) | **-1.35** |
| 1438988_X_AT | gb:AV067695 /DB_XREF=gi:5187523 /DB_XREF=AV067695 |  | zinc ion binding;nucleic acid binding;ubiquitin-protein ligase activity |  | **-2.99** |
| 1439012_A_AT | deoxycytidine kinase | [DCK](http://www.gene.ucl.ac.uk/cgi-bin/nomenclature/searchgenes.pl?field=symbol&anchor=equals&symbol_search=Search&number=100&format=html&sortby=symbol&match=DCK) | zinc ion binding;nucleic acid binding;ubiquitin-protein ligase activity | [125450](http://www.ncbi.nlm.nih.gov/entrez/dispomim.cgi?id=125450) | **-2.77** |
| 1439017_X_AT | adiponectin receptor 1 | [ADIPOR1](http://www.gene.ucl.ac.uk/cgi-bin/nomenclature/searchgenes.pl?field=symbol&anchor=equals&symbol_search=Search&number=100&format=html&sortby=symbol&match=ADIPOR1) | zinc ion binding;nucleic acid binding;ubiquitin-protein ligase activity | [607945](http://www.ncbi.nlm.nih.gov/entrez/dispomim.cgi?id=607945) | **1.28** |
| 1439019_AT | Fraser syndrome 1 homolog (human) | [FRAS1](http://www.gene.ucl.ac.uk/cgi-bin/nomenclature/searchgenes.pl?field=symbol&anchor=equals&symbol_search=Search&number=100&format=html&sortby=symbol&match=FRAS1) | zinc ion binding;O-sialoglycoprotein endopeptidase activity;peptidase activity | [607830](http://www.ncbi.nlm.nih.gov/entrez/dispomim.cgi?id=607830) | **-1.84** |
| 1439029_AT | glutamic pyruvate transaminase (alanine aminotransferase) 2 | [GPT2](http://www.gene.ucl.ac.uk/cgi-bin/nomenclature/searchgenes.pl?field=symbol&anchor=equals&symbol_search=Search&number=100&format=html&sortby=symbol&match=GPT2) | zinc ion binding;O-sialoglycoprotein endopeptidase activity;peptidase activity | [607438](http://www.ncbi.nlm.nih.gov/entrez/dispomim.cgi?id=607438) | **1.76** |
| 1439035_AT | Zinc finger protein 322a | [ZFP322A](http://www.gene.ucl.ac.uk/cgi-bin/nomenclature/searchgenes.pl?field=symbol&anchor=equals&symbol_search=Search&number=100&format=html&sortby=symbol&match=ZFP322A) | zinc ion binding;oxidoreductase activity;protein-methionine-R-oxide reductase activity |  | **-1.42** |
| 1439036_A_AT | ATPase, Na+/K+ transporting, beta 1 polypeptide | [ATP1B1](http://www.gene.ucl.ac.uk/cgi-bin/nomenclature/searchgenes.pl?field=symbol&anchor=equals&symbol_search=Search&number=100&format=html&sortby=symbol&match=ATP1B1) | zinc ion binding;oxidoreductase activity;protein-methionine-R-oxide reductase activity | [182330](http://www.ncbi.nlm.nih.gov/entrez/dispomim.cgi?id=182330) | **1.36** |
| 1439044_AT | zinc finger protein 354C | [ZFP354C](http://www.gene.ucl.ac.uk/cgi-bin/nomenclature/searchgenes.pl?field=symbol&anchor=equals&symbol_search=Search&number=100&format=html&sortby=symbol&match=ZFP354C) | zinc ion binding;phosphoprotein phosphatase activity;carbonate dehydratase activity;protein tyrosine phosphatase activity;hydrolase activity |  | **-1.75** |
| 1439066_AT | angiopoietin 1 | [ANGPT1](http://www.gene.ucl.ac.uk/cgi-bin/nomenclature/searchgenes.pl?field=symbol&anchor=equals&symbol_search=Search&number=100&format=html&sortby=symbol&match=ANGPT1) | zinc ion binding;protein binding | [601667](http://www.ncbi.nlm.nih.gov/entrez/dispomim.cgi?id=601667) | **1.68** |
| 1439069_A_AT | RIKEN cDNA 4933439C20 gene | [MGC65558](http://www.gene.ucl.ac.uk/cgi-bin/nomenclature/searchgenes.pl?field=symbol&anchor=equals&symbol_search=Search&number=100&format=html&sortby=symbol&match=MGC65558) | zinc ion binding;protein binding;DNA binding;ubiquitin-protein ligase activity |  | **-1.5** |
| 1439070_X_AT | RIKEN cDNA 9030221M09 gene | [MGC65558](http://www.gene.ucl.ac.uk/cgi-bin/nomenclature/searchgenes.pl?field=symbol&anchor=equals&symbol_search=Search&number=100&format=html&sortby=symbol&match=MGC65558) | zinc ion binding;protein binding;DNA binding;ubiquitin-protein ligase activity;transcriptional repressor activity |  | **-2.46** |
| 1439079_A_AT | Erbb2 interacting protein | [ERBB2IP](http://www.gene.ucl.ac.uk/cgi-bin/nomenclature/searchgenes.pl?field=symbol&anchor=equals&symbol_search=Search&number=100&format=html&sortby=symbol&match=ERBB2IP) | zinc ion binding;protein binding;ligase activity;ubiquitin-protein ligase activity | [606944](http://www.ncbi.nlm.nih.gov/entrez/dispomim.cgi?id=606944) | **-1.59** |
| 1439096_AT | RIKEN cDNA 5330420D20 gene | [5330420D20RIK](http://www.gene.ucl.ac.uk/cgi-bin/nomenclature/searchgenes.pl?field=symbol&anchor=equals&symbol_search=Search&number=100&format=html&sortby=symbol&match=5330420D20RIK) | zinc ion binding;protein binding;NADPH:quinone reductase activity;alcohol dehydrogenase activity, zinc-dependent |  | **1.55** |
| 1439127_AT | expressed sequence AI314180 | [AI314180](http://www.gene.ucl.ac.uk/cgi-bin/nomenclature/searchgenes.pl?field=symbol&anchor=equals&symbol_search=Search&number=100&format=html&sortby=symbol&match=AI314180) | zinc ion binding;protein binding;PDZ domain binding;protein homooligomerization activity;ubiquitin-protein ligase activity |  | **1.54** |
| 1439150_X_AT | gb:BB480256 /DB_XREF=gi:9397865 /DB_XREF=BB480256 |  | zinc ion binding;protein binding;phosphoprotein phosphatase activity;receptor activity;carbonate dehydratase activity;protein tyrosine phosphatase activity;prenylated protein tyrosine phosphatase activity;hydrolase activity |  | **3.31** |
| 1439151_AT | methionine sulfoxide reductase B3 | [MSRB3](http://www.gene.ucl.ac.uk/cgi-bin/nomenclature/searchgenes.pl?field=symbol&anchor=equals&symbol_search=Search&number=100&format=html&sortby=symbol&match=MSRB3) | zinc ion binding;protein binding;ubiquitin-protein ligase activity |  | **1.45** |
| 1439153_AT | IBR domain containing 2 | [IBRDC2](http://www.gene.ucl.ac.uk/cgi-bin/nomenclature/searchgenes.pl?field=symbol&anchor=equals&symbol_search=Search&number=100&format=html&sortby=symbol&match=IBRDC2) | zinc ion binding;protein binding;ubiquitin-protein ligase activity |  | **1.8** |
| 1439184_S_AT | thioredoxin-like 5 | [TXNL5](http://www.gene.ucl.ac.uk/cgi-bin/nomenclature/searchgenes.pl?field=symbol&anchor=equals&symbol_search=Search&number=100&format=html&sortby=symbol&match=TXNL5) | zinc ion binding;protein carrier activity |  | **-1.34** |
| 1439185_X_AT | RIKEN cDNA D430028G21 gene | [D430028G21RIK](http://www.gene.ucl.ac.uk/cgi-bin/nomenclature/searchgenes.pl?field=symbol&anchor=equals&symbol_search=Search&number=100&format=html&sortby=symbol&match=D430028G21RIK) | zinc ion binding;Rac guanyl-nucleotide exchange factor activity;guanyl-nucleotide exchange factor activity;microtubule binding;Rac GTPase binding;Rho guanyl-nucleotide exchange factor activity;diacylglycerol binding;Rho GTPase binding |  | **1.45** |
| 1439200_X_AT | gb:BE686792 /DB_XREF=gi:10074416 /DB_XREF=uu66g08. |  | zinc ion binding;Rac guanyl-nucleotide exchange factor activity;microtubule binding;guanyl-nucleotide exchange factor activity;Rac GTPase binding;diacylglycerol binding;Rho guanyl-nucleotide exchange factor activity;Rho GTPase binding |  | **9.55** |
| 1439204_AT | sodium channel, voltage-gated, type III, alpha polypeptide | [SCN3A](http://www.gene.ucl.ac.uk/cgi-bin/nomenclature/searchgenes.pl?field=symbol&anchor=equals&symbol_search=Search&number=100&format=html&sortby=symbol&match=SCN3A) | zinc ion binding;receptor activity;ubiquitin-protein ligase activity | [182391](http://www.ncbi.nlm.nih.gov/entrez/dispomim.cgi?id=182391) | **-3.32** |
| 1439251_AT | gb:BI133445 /DB_XREF=gi:14583693 /DB_XREF=UI-M-BH3 |  | zinc ion binding;receptor activity;ubiquitin-protein ligase activity |  | **-2.65** |
| 1439253_X_AT | RIKEN cDNA 2610524G07 gene | [2610524G07RIK](http://www.gene.ucl.ac.uk/cgi-bin/nomenclature/searchgenes.pl?field=symbol&anchor=equals&symbol_search=Search&number=100&format=html&sortby=symbol&match=2610524G07RIK) | zinc ion binding;stromelysin 1 activity;metallopeptidase activity;calcium ion binding;metalloendopeptidase activity;hydrolase activity;peptidase activity |  | **-1.59** |
| 1439255_S_AT | transmembrane 7 superfamily member 1 | [TM7SF1](http://www.gene.ucl.ac.uk/cgi-bin/nomenclature/searchgenes.pl?field=symbol&anchor=equals&symbol_search=Search&number=100&format=html&sortby=symbol&match=TM7SF1) | zinc ion binding;ubiquitin-protein ligase activity | [604658](http://www.ncbi.nlm.nih.gov/entrez/dispomim.cgi?id=604658) | **-1.99** |
| 1439257_X_AT | ribophorin I | [RPN1](http://www.gene.ucl.ac.uk/cgi-bin/nomenclature/searchgenes.pl?field=symbol&anchor=equals&symbol_search=Search&number=100&format=html&sortby=symbol&match=RPN1) | zinc ion binding;ubiquitin-protein ligase activity | [180470](http://www.ncbi.nlm.nih.gov/entrez/dispomim.cgi?id=180470) | **-1.44** |
| 1439267_X_AT | cytochrome c oxidase, subunit Va | [COX5A](http://www.gene.ucl.ac.uk/cgi-bin/nomenclature/searchgenes.pl?field=symbol&anchor=equals&symbol_search=Search&number=100&format=html&sortby=symbol&match=COX5A) | zinc ion binding;ubiquitin-protein ligase activity | [603773](http://www.ncbi.nlm.nih.gov/entrez/dispomim.cgi?id=603773) | **1.26** |
| 1439270_X_AT | RAN, member RAS oncogene family | [RAN](http://www.gene.ucl.ac.uk/cgi-bin/nomenclature/searchgenes.pl?field=symbol&anchor=equals&symbol_search=Search&number=100&format=html&sortby=symbol&match=RAN) | zinc ion binding;ubiquitin-protein ligase activity | [601179](http://www.ncbi.nlm.nih.gov/entrez/dispomim.cgi?id=601179) | **-1.24** |
| 1439271_X_AT | IK cytokine | [IK](http://www.gene.ucl.ac.uk/cgi-bin/nomenclature/searchgenes.pl?field=symbol&anchor=equals&symbol_search=Search&number=100&format=html&sortby=symbol&match=IK) | zinc ion binding;ubiquitin-protein ligase activity | [600549](http://www.ncbi.nlm.nih.gov/entrez/dispomim.cgi?id=600549) | **-1.41** |
| 1439274_AT | RIKEN cDNA A230083H22 gene | [A230083H22RIK](http://www.gene.ucl.ac.uk/cgi-bin/nomenclature/searchgenes.pl?field=symbol&anchor=equals&symbol_search=Search&number=100&format=html&sortby=symbol&match=A230083H22RIK) | zinc ion binding;ubiquitin-protein ligase activity |  | **-5.48** |
| 1439343_AT | gb:BB202287 /DB_XREF=gi:16271691 /DB_XREF=BB202287 |  | zinc ion binding;ubiquitin-protein ligase activity |  | **1.76** |
| 1439364_A_AT | matrix metalloproteinase 2 | [MMP2](http://www.gene.ucl.ac.uk/cgi-bin/nomenclature/searchgenes.pl?field=symbol&anchor=equals&symbol_search=Search&number=100&format=html&sortby=symbol&match=MMP2) | zinc ion binding;ubiquitin-protein ligase activity | [120360](http://www.ncbi.nlm.nih.gov/entrez/dispomim.cgi?id=120360) | **-2.87** |
| 1439368_A_AT | solute carrier family 9 (sodium/hydrogen exchanger), isoform 3 regulator 2 | [SLC9A3R2](http://www.gene.ucl.ac.uk/cgi-bin/nomenclature/searchgenes.pl?field=symbol&anchor=equals&symbol_search=Search&number=100&format=html&sortby=symbol&match=SLC9A3R2) | zinc ion binding;ubiquitin-protein ligase activity | [606553](http://www.ncbi.nlm.nih.gov/entrez/dispomim.cgi?id=606553) | **2.26** |
| 1439369_X_AT | solute carrier family 9 (sodium/hydrogen exchanger), isoform 3 regulator 2 | [SLC9A3R2](http://www.gene.ucl.ac.uk/cgi-bin/nomenclature/searchgenes.pl?field=symbol&anchor=equals&symbol_search=Search&number=100&format=html&sortby=symbol&match=SLC9A3R2) | zinc ion binding;ubiquitin-protein ligase activity | [606553](http://www.ncbi.nlm.nih.gov/entrez/dispomim.cgi?id=606553) | **2.05** |
| 1439374_X_AT | ribosomal protein S10 | [RPS10](http://www.gene.ucl.ac.uk/cgi-bin/nomenclature/searchgenes.pl?field=symbol&anchor=equals&symbol_search=Search&number=100&format=html&sortby=symbol&match=RPS10) | zinc ion binding;ubiquitin-protein ligase activity | [603632](http://www.ncbi.nlm.nih.gov/entrez/dispomim.cgi?id=603632) | **-1.86** |
| 1439377_X_AT | cell division cycle 20 homolog (S. cerevisiae) | [CDC20](http://www.gene.ucl.ac.uk/cgi-bin/nomenclature/searchgenes.pl?field=symbol&anchor=equals&symbol_search=Search&number=100&format=html&sortby=symbol&match=CDC20) | zinc ion binding;ubiquitin-protein ligase activity;peptidase activity | [603618](http://www.ncbi.nlm.nih.gov/entrez/dispomim.cgi?id=603618) | **-2.64** |
| 1439380_X_AT | GTL2, imprinted maternally expressed untranslated mRNA | [GTL2](http://www.gene.ucl.ac.uk/cgi-bin/nomenclature/searchgenes.pl?field=symbol&anchor=equals&symbol_search=Search&number=100&format=html&sortby=symbol&match=GTL2) | zinc ion transporter activity;cation transporter activity |  | **-1.77** |
| 1439399_A_AT | RNA, U22 small nucleolar | [RNU22](http://www.gene.ucl.ac.uk/cgi-bin/nomenclature/searchgenes.pl?field=symbol&anchor=equals&symbol_search=Search&number=100&format=html&sortby=symbol&match=RNU22) | zinc ion transporter activity;metal ion transporter activity |  | **-2.22** |
| 1439405_X_AT | gb:AV296285 /DB_XREF=gi:6328302 /DB_XREF=AV296285 |  | zinc ion transporter activity;metal ion transporter activity |  | **-1.44** |
| 1439406_X_AT | phenylalanine-tRNA synthetase 1 (mitochondrial) | [FARS1](http://www.gene.ucl.ac.uk/cgi-bin/nomenclature/searchgenes.pl?field=symbol&anchor=equals&symbol_search=Search&number=100&format=html&sortby=symbol&match=FARS1) | zymosan binding |  | **1.55** |
| 1439410_X_AT | DNA segment, Chr 11, ERATO Doi 333, expressed | [D11ERTD333E](http://www.gene.ucl.ac.uk/cgi-bin/nomenclature/searchgenes.pl?field=symbol&anchor=equals&symbol_search=Search&number=100&format=html&sortby=symbol&match=D11ERTD333E) |  |  | **1.36** |
| 1439412_AT | gb:BB167922 /DB_XREF=gi:8823863 /DB_XREF=BB167922 |  |  |  | **-2.04** |
| 1439413_X_AT | mortality factor 4 like 2 | [MORF4L2](http://www.gene.ucl.ac.uk/cgi-bin/nomenclature/searchgenes.pl?field=symbol&anchor=equals&symbol_search=Search&number=100&format=html&sortby=symbol&match=MORF4L2) |  | [300409](http://www.ncbi.nlm.nih.gov/entrez/dispomim.cgi?id=300409) | **-1.47** |
| 1439426_X_AT | P lysozyme structural | [LZP-S](http://www.gene.ucl.ac.uk/cgi-bin/nomenclature/searchgenes.pl?field=symbol&anchor=equals&symbol_search=Search&number=100&format=html&sortby=symbol&match=LZP-S) |  |  | **-5.94** |
| 1439432_X_AT | mortality factor 4 like 2 | [MORF4L2](http://www.gene.ucl.ac.uk/cgi-bin/nomenclature/searchgenes.pl?field=symbol&anchor=equals&symbol_search=Search&number=100&format=html&sortby=symbol&match=MORF4L2) |  | [300409](http://www.ncbi.nlm.nih.gov/entrez/dispomim.cgi?id=300409) | **-1.63** |
| 1439450_X_AT | RIKEN cDNA A230046K03 gene | [A230046K03RIK](http://www.gene.ucl.ac.uk/cgi-bin/nomenclature/searchgenes.pl?field=symbol&anchor=equals&symbol_search=Search&number=100&format=html&sortby=symbol&match=A230046K03RIK) |  |  | **-1.44** |
| 1439455_X_AT | capping protein (actin filament) muscle Z-line, alpha 1 | [CAPZA1](http://www.gene.ucl.ac.uk/cgi-bin/nomenclature/searchgenes.pl?field=symbol&anchor=equals&symbol_search=Search&number=100&format=html&sortby=symbol&match=CAPZA1) |  | [601580](http://www.ncbi.nlm.nih.gov/entrez/dispomim.cgi?id=601580) | **-1.79** |
| 1439456_X_AT | ATPase, H+ transporting, lysosomal accessory protein 2 | [ATP6AP2](http://www.gene.ucl.ac.uk/cgi-bin/nomenclature/searchgenes.pl?field=symbol&anchor=equals&symbol_search=Search&number=100&format=html&sortby=symbol&match=ATP6AP2) |  |  | **-1.55** |
| 1439464_S_AT | testis expressed gene 10 | [TEX10](http://www.gene.ucl.ac.uk/cgi-bin/nomenclature/searchgenes.pl?field=symbol&anchor=equals&symbol_search=Search&number=100&format=html&sortby=symbol&match=TEX10) |  |  | **-1.38** |
| 1439466_S_AT | Expressed sequence C77604 | [C77604](http://www.gene.ucl.ac.uk/cgi-bin/nomenclature/searchgenes.pl?field=symbol&anchor=equals&symbol_search=Search&number=100&format=html&sortby=symbol&match=C77604) |  |  | **-1.54** |
| 1439478_AT | mitochondrial acyl-CoA thioesterase 1 | [MTE1](http://www.gene.ucl.ac.uk/cgi-bin/nomenclature/searchgenes.pl?field=symbol&anchor=equals&symbol_search=Search&number=100&format=html&sortby=symbol&match=MTE1) |  |  | **1.4** |
| 1439505_AT | gb:AA210377 /DB_XREF=gi:1807624 /DB_XREF=mu72d07.r |  |  |  | **1.4** |
| 1439509_AT | RIKEN cDNA 2900008C10 gene | [2900008C10RIK](http://www.gene.ucl.ac.uk/cgi-bin/nomenclature/searchgenes.pl?field=symbol&anchor=equals&symbol_search=Search&number=100&format=html&sortby=symbol&match=2900008C10RIK) |  |  | **1.85** |
| 1439518_AT | multimerin 2 | [MMRN2](http://www.gene.ucl.ac.uk/cgi-bin/nomenclature/searchgenes.pl?field=symbol&anchor=equals&symbol_search=Search&number=100&format=html&sortby=symbol&match=MMRN2) |  | [608925](http://www.ncbi.nlm.nih.gov/entrez/dispomim.cgi?id=608925) | **1.84** |
| 1439521_AT | Adult male aorta and vein cDNA, RIKEN full-length enriched library, clone:A530001O10 product:unknown EST, full insert sequence |  |  |  | **2.13** |
| 1439526_AT | Adult male cecum cDNA, RIKEN full-length enriched library, clone:9130022D06 product:unknown EST, full insert sequence |  |  |  | **-1.75** |
| 1439539_AT | translocating chain-associating membrane protein 2 | [TRAM2](http://www.gene.ucl.ac.uk/cgi-bin/nomenclature/searchgenes.pl?field=symbol&anchor=equals&symbol_search=Search&number=100&format=html&sortby=symbol&match=TRAM2) |  | [608485](http://www.ncbi.nlm.nih.gov/entrez/dispomim.cgi?id=608485) | **-1.42** |
| 1439556_AT | Neural cell adhesion molecule 1 | [NCAM1](http://www.gene.ucl.ac.uk/cgi-bin/nomenclature/searchgenes.pl?field=symbol&anchor=equals&symbol_search=Search&number=100&format=html&sortby=symbol&match=NCAM1) |  | [116930](http://www.ncbi.nlm.nih.gov/entrez/dispomim.cgi?id=116930) | **-4.23** |
| 1439582_AT | gb:BG074706 /DB_XREF=gi:12557275 /DB_XREF=H3138C11 |  |  |  | **-1.53** |
| 1439622_AT | Ras association (RalGDS/AF-6) domain family 4 | [RASSF4](http://www.gene.ucl.ac.uk/cgi-bin/nomenclature/searchgenes.pl?field=symbol&anchor=equals&symbol_search=Search&number=100&format=html&sortby=symbol&match=RASSF4) |  |  | **-1.59** |
| 1439658_AT | leiomodin 3 (fetal) | [LMOD3](http://www.gene.ucl.ac.uk/cgi-bin/nomenclature/searchgenes.pl?field=symbol&anchor=equals&symbol_search=Search&number=100&format=html&sortby=symbol&match=LMOD3) |  |  | **1.3** |
| 1439665_AT | G protein-coupled receptor 23 | [GPR23](http://www.gene.ucl.ac.uk/cgi-bin/nomenclature/searchgenes.pl?field=symbol&anchor=equals&symbol_search=Search&number=100&format=html&sortby=symbol&match=GPR23) |  | [300086](http://www.ncbi.nlm.nih.gov/entrez/dispomim.cgi?id=300086) | **-2.61** |
| 1439669_AT | RIKEN cDNA 6430571L13 gene | [6430571L13RIK](http://www.gene.ucl.ac.uk/cgi-bin/nomenclature/searchgenes.pl?field=symbol&anchor=equals&symbol_search=Search&number=100&format=html&sortby=symbol&match=6430571L13RIK) |  |  | **1.86** |
| 1439675_AT | RIKEN cDNA 4933429D07 gene | [4933429D07RIK](http://www.gene.ucl.ac.uk/cgi-bin/nomenclature/searchgenes.pl?field=symbol&anchor=equals&symbol_search=Search&number=100&format=html&sortby=symbol&match=4933429D07RIK) |  |  | **1.68** |
| 1439740_S_AT | expressed sequence AI481316 | [AI481316](http://www.gene.ucl.ac.uk/cgi-bin/nomenclature/searchgenes.pl?field=symbol&anchor=equals&symbol_search=Search&number=100&format=html&sortby=symbol&match=AI481316) |  |  | **-1.76** |
| 1439741_X_AT | expressed sequence AI481316 | [AI481316](http://www.gene.ucl.ac.uk/cgi-bin/nomenclature/searchgenes.pl?field=symbol&anchor=equals&symbol_search=Search&number=100&format=html&sortby=symbol&match=AI481316) |  |  | **-1.6** |
| 1439764_S_AT | RIKEN cDNA C330012H03 gene | [C330012H03RIK](http://www.gene.ucl.ac.uk/cgi-bin/nomenclature/searchgenes.pl?field=symbol&anchor=equals&symbol_search=Search&number=100&format=html&sortby=symbol&match=C330012H03RIK) |  |  | **-3.14** |
| 1439774_AT | paired related homeobox 1 | [PRRX1](http://www.gene.ucl.ac.uk/cgi-bin/nomenclature/searchgenes.pl?field=symbol&anchor=equals&symbol_search=Search&number=100&format=html&sortby=symbol&match=PRRX1) |  | [167420](http://www.ncbi.nlm.nih.gov/entrez/dispomim.cgi?id=167420) | **-1.56** |
| 1439804_AT | gb:AW489408 /DB_XREF=gi:7059678 /DB_XREF=UI-M-BH3- |  |  |  | **1.44** |
| 1439827_AT | a disintegrin-like and metalloprotease (reprolysin type) with thrombospondin type 1 motif, 12 | [ADAMTS12](http://www.gene.ucl.ac.uk/cgi-bin/nomenclature/searchgenes.pl?field=symbol&anchor=equals&symbol_search=Search&number=100&format=html&sortby=symbol&match=ADAMTS12) |  | [606184](http://www.ncbi.nlm.nih.gov/entrez/dispomim.cgi?id=606184) | **-1.85** |
| 1439836_AT | gb:BB138331 /DB_XREF=gi:8793268 /DB_XREF=BB138331 |  |  |  | **2.43** |
| 1439855_AT | cDNA sequence BC023818 | [BC023818](http://www.gene.ucl.ac.uk/cgi-bin/nomenclature/searchgenes.pl?field=symbol&anchor=equals&symbol_search=Search&number=100&format=html&sortby=symbol&match=BC023818) |  |  | **2.11** |
| 1439859_AT | RIKEN cDNA 9630033F20 gene | [9630033F20RIK](http://www.gene.ucl.ac.uk/cgi-bin/nomenclature/searchgenes.pl?field=symbol&anchor=equals&symbol_search=Search&number=100&format=html&sortby=symbol&match=9630033F20RIK) |  |  | **1.62** |
| 1439902_AT | gb:BB552085 /DB_XREF=gi:16448122 /DB_XREF=BB552085 |  |  |  | **-2.24** |
| 1439925_AT | gb:BQ031166 /DB_XREF=gi:19766445 /DB_XREF=UI-1-CF0 |  |  |  | **1.67** |
| 1439926_AT | hypothetical protein 4632417D23 | [4632417D23](http://www.gene.ucl.ac.uk/cgi-bin/nomenclature/searchgenes.pl?field=symbol&anchor=equals&symbol_search=Search&number=100&format=html&sortby=symbol&match=4632417D23) |  |  | **1.57** |
| 1439956_AT | Adult male aorta and vein cDNA, RIKEN full-length enriched library, clone:A530049N04 product:unknown EST, full insert sequence |  |  |  | **-2.15** |
| 1439959_AT | Fibroblast growth factor 11 | [FGF11](http://www.gene.ucl.ac.uk/cgi-bin/nomenclature/searchgenes.pl?field=symbol&anchor=equals&symbol_search=Search&number=100&format=html&sortby=symbol&match=FGF11) |  | [601514](http://www.ncbi.nlm.nih.gov/entrez/dispomim.cgi?id=601514) | **-2.77** |
| 1439965_AT | gb:BM933678 /DB_XREF=gi:19392830 /DB_XREF=UI-M-BZ1 |  |  |  | **-1.54** |
| 1440008_AT | RIKEN cDNA 2310043L19 gene | [2310043L19RIK](http://www.gene.ucl.ac.uk/cgi-bin/nomenclature/searchgenes.pl?field=symbol&anchor=equals&symbol_search=Search&number=100&format=html&sortby=symbol&match=2310043L19RIK) |  |  | **-1.99** |
| 1440085_AT | ectodysplasin A2 isoform receptor | [EDA2R](http://www.gene.ucl.ac.uk/cgi-bin/nomenclature/searchgenes.pl?field=symbol&anchor=equals&symbol_search=Search&number=100&format=html&sortby=symbol&match=EDA2R) |  | [300276](http://www.ncbi.nlm.nih.gov/entrez/dispomim.cgi?id=300276) | **-5.38** |
| 1440092_AT | gb:BB497623 /DB_XREF=gi:16441975 /DB_XREF=BB497623 |  |  |  | **-1.46** |
| 1440167_S_AT | LIM domain containing preferred translocation partner in lipoma | [LPP](http://www.gene.ucl.ac.uk/cgi-bin/nomenclature/searchgenes.pl?field=symbol&anchor=equals&symbol_search=Search&number=100&format=html&sortby=symbol&match=LPP) |  | [600700](http://www.ncbi.nlm.nih.gov/entrez/dispomim.cgi?id=600700) | **-1.38** |
| 1440185_X_AT | ubiquitin-like 1 (sentrin) activating enzyme E1A | [UBLE1A](http://www.gene.ucl.ac.uk/cgi-bin/nomenclature/searchgenes.pl?field=symbol&anchor=equals&symbol_search=Search&number=100&format=html&sortby=symbol&match=UBLE1A) |  |  | **1.47** |
| 1440225_AT | RIKEN cDNA 8430401C09 gene | [GPR116](http://www.gene.ucl.ac.uk/cgi-bin/nomenclature/searchgenes.pl?field=symbol&anchor=equals&symbol_search=Search&number=100&format=html&sortby=symbol&match=GPR116) |  |  | **2.42** |
| 1440226_AT | cDNA sequence BC029103 | [BC029103](http://www.gene.ucl.ac.uk/cgi-bin/nomenclature/searchgenes.pl?field=symbol&anchor=equals&symbol_search=Search&number=100&format=html&sortby=symbol&match=BC029103) |  |  | **-1.49** |
| 1440234_AT | RIKEN cDNA 1810012P15 gene | [1810012P15RIK](http://www.gene.ucl.ac.uk/cgi-bin/nomenclature/searchgenes.pl?field=symbol&anchor=equals&symbol_search=Search&number=100&format=html&sortby=symbol&match=1810012P15RIK) |  |  | **1.74** |
| 1440254_AT | gb:W65070 /DB_XREF=gi:1372720 /DB_XREF=me01c06.r1 |  |  |  | **-1.34** |
| 1440335_AT | gb:AV020525 /DB_XREF=gi:4797517 /DB_XREF=AV020525 |  |  |  | **2.02** |
| 1440348_AT | Zinc finger, FYVE domain containing 9 | [ZFYVE9](http://www.gene.ucl.ac.uk/cgi-bin/nomenclature/searchgenes.pl?field=symbol&anchor=equals&symbol_search=Search&number=100&format=html&sortby=symbol&match=ZFYVE9) |  |  | **1.33** |
| 1440358_AT | RIKEN cDNA D530030K12 gene | [ARHGEF15](http://www.gene.ucl.ac.uk/cgi-bin/nomenclature/searchgenes.pl?field=symbol&anchor=equals&symbol_search=Search&number=100&format=html&sortby=symbol&match=ARHGEF15) |  |  | **2.15** |
| 1440384_AT | RIKEN cDNA 3632431M01 gene | [3632431M01RIK](http://www.gene.ucl.ac.uk/cgi-bin/nomenclature/searchgenes.pl?field=symbol&anchor=equals&symbol_search=Search&number=100&format=html&sortby=symbol&match=3632431M01RIK) |  |  | **1.37** |
| 1440397_AT | gb:BB464523 /DB_XREF=gi:16426998 /DB_XREF=BB464523 |  |  |  | **-1.61** |
| 1440452_AT | dystrophin related protein 2 | [DRP2](http://www.gene.ucl.ac.uk/cgi-bin/nomenclature/searchgenes.pl?field=symbol&anchor=equals&symbol_search=Search&number=100&format=html&sortby=symbol&match=DRP2) |  | [300052](http://www.ncbi.nlm.nih.gov/entrez/dispomim.cgi?id=300052) | **-1.41** |
| 1440461_AT | RIKEN cDNA 0910001A06 gene | [0910001A06RIK](http://www.gene.ucl.ac.uk/cgi-bin/nomenclature/searchgenes.pl?field=symbol&anchor=equals&symbol_search=Search&number=100&format=html&sortby=symbol&match=0910001A06RIK) |  |  | **-1.91** |
| 1440545_AT | gb:BQ176278 /DB_XREF=gi:20351770 /DB_XREF=UI-M-DJ2 |  |  |  | **5.79** |
| 1440569_AT | solute carrier family 16 (monocarboxylic acid transporters), member 10 | [SLC16A10](http://www.gene.ucl.ac.uk/cgi-bin/nomenclature/searchgenes.pl?field=symbol&anchor=equals&symbol_search=Search&number=100&format=html&sortby=symbol&match=SLC16A10) |  | [607550](http://www.ncbi.nlm.nih.gov/entrez/dispomim.cgi?id=607550) | **2.73** |
| 1440603_AT | Adult male corpus striatum cDNA, RIKEN full-length enriched library, clone:C030004I15 product:unclassifiable, full insert sequence |  |  |  | **1.57** |
| 1440621_AT | gb:AW551461 /DB_XREF=gi:7196889 /DB_XREF=L0079C03- |  |  |  | **1.78** |
| 1440715_S_AT | DNA segment, Chr 11, ERATO Doi 497, expressed | [D11ERTD497E](http://www.gene.ucl.ac.uk/cgi-bin/nomenclature/searchgenes.pl?field=symbol&anchor=equals&symbol_search=Search&number=100&format=html&sortby=symbol&match=D11ERTD497E) |  |  | **1.37** |
| 1440830_AT | RIKEN cDNA 8430401C09 gene | [GPR116](http://www.gene.ucl.ac.uk/cgi-bin/nomenclature/searchgenes.pl?field=symbol&anchor=equals&symbol_search=Search&number=100&format=html&sortby=symbol&match=GPR116) |  |  | **2.49** |
| 1440831_AT | RIKEN cDNA 6230421P05 gene | [6230421P05RIK](http://www.gene.ucl.ac.uk/cgi-bin/nomenclature/searchgenes.pl?field=symbol&anchor=equals&symbol_search=Search&number=100&format=html&sortby=symbol&match=6230421P05RIK) |  |  | **-1.32** |
| 1440838_AT | Expressed sequence AI852064 | [AI852064](http://www.gene.ucl.ac.uk/cgi-bin/nomenclature/searchgenes.pl?field=symbol&anchor=equals&symbol_search=Search&number=100&format=html&sortby=symbol&match=AI852064) |  |  | **1.62** |
| 1440840_AT | RIKEN cDNA D630004K10 gene | [D630004K10RIK](http://www.gene.ucl.ac.uk/cgi-bin/nomenclature/searchgenes.pl?field=symbol&anchor=equals&symbol_search=Search&number=100&format=html&sortby=symbol&match=D630004K10RIK) |  |  | **2.77** |
| 1440849_AT | gb:AV327187 /DB_XREF=gi:16395610 /DB_XREF=AV327187 |  |  |  | **1.68** |
| 1440870_AT | gb:BB356786 /DB_XREF=gi:16405764 /DB_XREF=BB356786 |  |  |  | **2.14** |
| 1440878_AT | gb:BB795285 /DB_XREF=gi:16964904 /DB_XREF=BB795285 |  |  |  | **-18.25** |
| 1440911_AT | procollagen, type XXIII, alpha 1 | [COL23A1](http://www.gene.ucl.ac.uk/cgi-bin/nomenclature/searchgenes.pl?field=symbol&anchor=equals&symbol_search=Search&number=100&format=html&sortby=symbol&match=COL23A1) |  |  | **-1.71** |
| 1440920_AT | matrix metalloproteinase 14 (membrane-inserted) | [MMP14](http://www.gene.ucl.ac.uk/cgi-bin/nomenclature/searchgenes.pl?field=symbol&anchor=equals&symbol_search=Search&number=100&format=html&sortby=symbol&match=MMP14) |  | [600754](http://www.ncbi.nlm.nih.gov/entrez/dispomim.cgi?id=600754) | **-1.86** |
| 1440926_AT | gb:BB555042 /DB_XREF=gi:16448504 /DB_XREF=BB555042 |  |  |  | **2.12** |
| 1440953_AT | RIKEN cDNA 1700096K18 gene | [1700096K18RIK](http://www.gene.ucl.ac.uk/cgi-bin/nomenclature/searchgenes.pl?field=symbol&anchor=equals&symbol_search=Search&number=100&format=html&sortby=symbol&match=1700096K18RIK) |  |  | **1.6** |
| 1440965_AT | Phosphatidylinositol glycan, class L | [PIGL](http://www.gene.ucl.ac.uk/cgi-bin/nomenclature/searchgenes.pl?field=symbol&anchor=equals&symbol_search=Search&number=100&format=html&sortby=symbol&match=PIGL) |  | [605947](http://www.ncbi.nlm.nih.gov/entrez/dispomim.cgi?id=605947) | **1.45** |
| 1440975_AT | RIKEN cDNA 1810057P16 gene | [1810057P16RIK](http://www.gene.ucl.ac.uk/cgi-bin/nomenclature/searchgenes.pl?field=symbol&anchor=equals&symbol_search=Search&number=100&format=html&sortby=symbol&match=1810057P16RIK) |  |  | **-1.86** |
| 1440989_AT | gb:BB183373 /DB_XREF=gi:16270232 /DB_XREF=BB183373 |  |  |  | **1.45** |
| 1441058_AT | inositol 1,4,5-trisphosphate 3-kinase B | [ITPKB](http://www.gene.ucl.ac.uk/cgi-bin/nomenclature/searchgenes.pl?field=symbol&anchor=equals&symbol_search=Search&number=100&format=html&sortby=symbol&match=ITPKB) |  | [147522](http://www.ncbi.nlm.nih.gov/entrez/dispomim.cgi?id=147522) | **1.58** |
| 1441065_AT | Adult male corpora quadrigemina cDNA, RIKEN full-length enriched library, clone:B230204J04 product:unknown EST, full insert sequence |  |  |  | **-1.57** |
| 1441069_AT | gb:BG069709 /DB_XREF=gi:12552278 /DB_XREF=H3079C06 |  |  |  | **1.72** |
| 1441072_AT | gb:BB353142 /DB_XREF=gi:9064970 /DB_XREF=BB353142 |  |  |  | **-1.25** |
| 1441081_A_AT | RIKEN cDNA 1110038B12 gene | [1110038B12RIK](http://www.gene.ucl.ac.uk/cgi-bin/nomenclature/searchgenes.pl?field=symbol&anchor=equals&symbol_search=Search&number=100&format=html&sortby=symbol&match=1110038B12RIK) |  |  | **-2.08** |
| 1441108_AT | gb:AU040876 /DB_XREF=gi:3954700 /DB_XREF=AU040876 |  |  |  | **-1.85** |
| 1441111_AT | Similar to myosin light chain kinase | [LOC238564](http://www.gene.ucl.ac.uk/cgi-bin/nomenclature/searchgenes.pl?field=symbol&anchor=equals&symbol_search=Search&number=100&format=html&sortby=symbol&match=LOC238564) |  |  | **3.47** |
| 1441137_AT | Bicaudal C homolog 1 (Drosophila) | [BICC1](http://www.gene.ucl.ac.uk/cgi-bin/nomenclature/searchgenes.pl?field=symbol&anchor=equals&symbol_search=Search&number=100&format=html&sortby=symbol&match=BICC1) |  |  | **-1.55** |
| 1441150_X_AT | gb:BB829165 /DB_XREF=gi:17007408 /DB_XREF=BB829165 |  |  |  | **1.9** |
| 1441208_AT | gb:AW546142 /DB_XREF=gi:7191549 /DB_XREF=L0004C01- |  |  |  | **1.85** |
| 1441228_AT | Gene model 1075, (NCBI) | [LOC381823](http://www.gene.ucl.ac.uk/cgi-bin/nomenclature/searchgenes.pl?field=symbol&anchor=equals&symbol_search=Search&number=100&format=html&sortby=symbol&match=LOC381823) |  |  | **2.37** |
| 1441259_S_AT | WD repeat domain 10 | [WDR10](http://www.gene.ucl.ac.uk/cgi-bin/nomenclature/searchgenes.pl?field=symbol&anchor=equals&symbol_search=Search&number=100&format=html&sortby=symbol&match=WDR10) |  | [606045](http://www.ncbi.nlm.nih.gov/entrez/dispomim.cgi?id=606045) | **-3.21** |
| 1441285_AT | gb:AW124536 /DB_XREF=gi:6100066 /DB_XREF=UI-M-BH2. |  |  |  | **1.35** |
| 1441396_AT | gb:AV328619 /DB_XREF=gi:16395920 /DB_XREF=AV328619 |  |  |  | **1.86** |
| 1441501_AT | RIKEN cDNA 9330147J08 gene | [9330147J08RIK](http://www.gene.ucl.ac.uk/cgi-bin/nomenclature/searchgenes.pl?field=symbol&anchor=equals&symbol_search=Search&number=100&format=html&sortby=symbol&match=9330147J08RIK) |  |  | **1.51** |
| 1441531_AT | gb:BB143801 /DB_XREF=gi:15408558 /DB_XREF=BB143801 |  |  |  | **2.03** |
| 1441581_AT | ankyrin repeat and SOCS box-containing protein 10 | [ASB10](http://www.gene.ucl.ac.uk/cgi-bin/nomenclature/searchgenes.pl?field=symbol&anchor=equals&symbol_search=Search&number=100&format=html&sortby=symbol&match=ASB10) |  |  | **1.82** |
| 1441584_AT | Friend leukemia integration 1 | [FLI1](http://www.gene.ucl.ac.uk/cgi-bin/nomenclature/searchgenes.pl?field=symbol&anchor=equals&symbol_search=Search&number=100&format=html&sortby=symbol&match=FLI1) |  | [193067](http://www.ncbi.nlm.nih.gov/entrez/dispomim.cgi?id=193067) | **1.38** |
| 1441682_S_AT | exportin, tRNA (nuclear export receptor for tRNAs) | [XPOT](http://www.gene.ucl.ac.uk/cgi-bin/nomenclature/searchgenes.pl?field=symbol&anchor=equals&symbol_search=Search&number=100&format=html&sortby=symbol&match=XPOT) |  | [603180](http://www.ncbi.nlm.nih.gov/entrez/dispomim.cgi?id=603180) | **-1.31** |
| 1441751_AT | gb:BE947079 /DB_XREF=gi:10524838 /DB_XREF=UI-M-BH3 |  |  |  | **1.51** |
| 1441878_S_AT | RIKEN cDNA 1810049H13 gene | [1810049H13RIK](http://www.gene.ucl.ac.uk/cgi-bin/nomenclature/searchgenes.pl?field=symbol&anchor=equals&symbol_search=Search&number=100&format=html&sortby=symbol&match=1810049H13RIK) |  |  | **1.41** |
| 1441894_S_AT | GRP1 (general receptor for phosphoinositides 1)-associated scaffold protein | [GRASP](http://www.gene.ucl.ac.uk/cgi-bin/nomenclature/searchgenes.pl?field=symbol&anchor=equals&symbol_search=Search&number=100&format=html&sortby=symbol&match=GRASP) |  |  | **1.24** |
| 1441912_X_AT | complement component 2 (within H-2S) | [C2](http://www.gene.ucl.ac.uk/cgi-bin/nomenclature/searchgenes.pl?field=symbol&anchor=equals&symbol_search=Search&number=100&format=html&sortby=symbol&match=C2) |  | [217000](http://www.ncbi.nlm.nih.gov/entrez/dispomim.cgi?id=217000) | **1.43** |
| 1441915_S_AT | RIKEN cDNA 2310076L09 gene | [2310076L09RIK](http://www.gene.ucl.ac.uk/cgi-bin/nomenclature/searchgenes.pl?field=symbol&anchor=equals&symbol_search=Search&number=100&format=html&sortby=symbol&match=2310076L09RIK) |  |  | **1.87** |
| 1441937_S_AT | gb:AV371921 /DB_XREF=gi:6419568 /DB_XREF=AV371921 |  |  |  | **1.56** |
| 1441959_S_AT | RIKEN cDNA 1200003C05 gene | [1200003C05RIK](http://www.gene.ucl.ac.uk/cgi-bin/nomenclature/searchgenes.pl?field=symbol&anchor=equals&symbol_search=Search&number=100&format=html&sortby=symbol&match=1200003C05RIK) |  |  | **-1.51** |
| 1441971_AT | gb:AW543723 /DB_XREF=gi:7186140 /DB_XREF=C0173D05- |  |  |  | **-1.96** |
| 1441972_AT | RIKEN cDNA 6230424C14 gene | [6230424C14RIK](http://www.gene.ucl.ac.uk/cgi-bin/nomenclature/searchgenes.pl?field=symbol&anchor=equals&symbol_search=Search&number=100&format=html&sortby=symbol&match=6230424C14RIK) |  |  | **1.84** |
| 1441995_AT | gb:AW493638 /DB_XREF=gi:7063919 /DB_XREF=UI-M-BH3- |  |  |  | **-2.5** |
| 1442033_AT | RIKEN cDNA 9130217G22 gene | [9130217G22RIK](http://www.gene.ucl.ac.uk/cgi-bin/nomenclature/searchgenes.pl?field=symbol&anchor=equals&symbol_search=Search&number=100&format=html&sortby=symbol&match=9130217G22RIK) |  |  | **2.11** |
| 1442076_AT | cordon-bleu | [COBL](http://www.gene.ucl.ac.uk/cgi-bin/nomenclature/searchgenes.pl?field=symbol&anchor=equals&symbol_search=Search&number=100&format=html&sortby=symbol&match=COBL) |  |  | **-1.23** |
| 1442082_AT | gb:BB333624 /DB_XREF=gi:16404014 /DB_XREF=BB333624 |  |  |  | **-3.35** |
| 1442118_AT | gb:AW215795 /DB_XREF=gi:6526490 /DB_XREF=uo99b12.x |  |  |  | **-1.78** |
| 1442121_AT | cyclic AMP-regulated phosphoprotein, 21 | [ARPP21](http://www.gene.ucl.ac.uk/cgi-bin/nomenclature/searchgenes.pl?field=symbol&anchor=equals&symbol_search=Search&number=100&format=html&sortby=symbol&match=ARPP21) |  |  | **-2.91** |
| 1442145_AT | RIKEN cDNA A130034M23 gene | [A130034M23RIK](http://www.gene.ucl.ac.uk/cgi-bin/nomenclature/searchgenes.pl?field=symbol&anchor=equals&symbol_search=Search&number=100&format=html&sortby=symbol&match=A130034M23RIK) |  |  | **-1.86** |
| 1442169_AT | very low density lipoprotein receptor | [VLDLR](http://www.gene.ucl.ac.uk/cgi-bin/nomenclature/searchgenes.pl?field=symbol&anchor=equals&symbol_search=Search&number=100&format=html&sortby=symbol&match=VLDLR) |  | [192977](http://www.ncbi.nlm.nih.gov/entrez/dispomim.cgi?id=192977) | **1.41** |
| 1442325_AT | RIKEN cDNA C530046L02 gene | [C530046L02RIK](http://www.gene.ucl.ac.uk/cgi-bin/nomenclature/searchgenes.pl?field=symbol&anchor=equals&symbol_search=Search&number=100&format=html&sortby=symbol&match=C530046L02RIK) |  |  | **1.72** |
| 1442340_X_AT | cysteine rich protein 61 | [CYR61](http://www.gene.ucl.ac.uk/cgi-bin/nomenclature/searchgenes.pl?field=symbol&anchor=equals&symbol_search=Search&number=100&format=html&sortby=symbol&match=CYR61) |  | [602369](http://www.ncbi.nlm.nih.gov/entrez/dispomim.cgi?id=602369) | **1.65** |
| 1442434_AT | DNA segment, Chr 8, ERATO Doi 82, expressed | [D8ERTD82E](http://www.gene.ucl.ac.uk/cgi-bin/nomenclature/searchgenes.pl?field=symbol&anchor=equals&symbol_search=Search&number=100&format=html&sortby=symbol&match=D8ERTD82E) |  |  | **2.38** |
| 1442445_AT | RIKEN cDNA 2610027H17 gene | [2610027H17RIK](http://www.gene.ucl.ac.uk/cgi-bin/nomenclature/searchgenes.pl?field=symbol&anchor=equals&symbol_search=Search&number=100&format=html&sortby=symbol&match=2610027H17RIK) |  |  | **1.72** |
| 1442466_A_AT | RIKEN cDNA B430315C20 gene | [B430315C20RIK](http://www.gene.ucl.ac.uk/cgi-bin/nomenclature/searchgenes.pl?field=symbol&anchor=equals&symbol_search=Search&number=100&format=html&sortby=symbol&match=B430315C20RIK) |  |  | **1.65** |
| 1442537_AT | Adult male aorta and vein cDNA, RIKEN full-length enriched library, clone:A530016B22 product:unknown EST, full insert sequence |  |  |  | **1.45** |
| 1442542_AT | RIKEN cDNA B130023L16 gene | [B130023L16RIK](http://www.gene.ucl.ac.uk/cgi-bin/nomenclature/searchgenes.pl?field=symbol&anchor=equals&symbol_search=Search&number=100&format=html&sortby=symbol&match=B130023L16RIK) |  |  | **-1.6** |
| 1442548_AT | gb:BM234411 /DB_XREF=gi:17869681 /DB_XREF=K0349F10 |  |  |  | **1.64** |
| 1442590_AT | gb:BB366863 /DB_XREF=gi:16406479 /DB_XREF=BB366863 |  |  |  | **-2.21** |
| 1442625_AT | gb:BG070581 /DB_XREF=gi:12553150 /DB_XREF=H3088H06 |  |  |  | **1.54** |
| 1442680_AT | Neural cell adhesion molecule 1 | [NCAM1](http://www.gene.ucl.ac.uk/cgi-bin/nomenclature/searchgenes.pl?field=symbol&anchor=equals&symbol_search=Search&number=100&format=html&sortby=symbol&match=NCAM1) |  | [116930](http://www.ncbi.nlm.nih.gov/entrez/dispomim.cgi?id=116930) | **-5.39** |
| 1442760_X_AT | gb:BB206454 /DB_XREF=gi:16352122 /DB_XREF=BB206454 |  |  |  | **-1.56** |
| 1442791_X_AT | RIKEN cDNA 6720407P12 gene | [6720407P12RIK](http://www.gene.ucl.ac.uk/cgi-bin/nomenclature/searchgenes.pl?field=symbol&anchor=equals&symbol_search=Search&number=100&format=html&sortby=symbol&match=6720407P12RIK) |  |  | **-2.66** |
| 1442827_AT | toll-like receptor 4 | [TLR4](http://www.gene.ucl.ac.uk/cgi-bin/nomenclature/searchgenes.pl?field=symbol&anchor=equals&symbol_search=Search&number=100&format=html&sortby=symbol&match=TLR4) |  | [603030](http://www.ncbi.nlm.nih.gov/entrez/dispomim.cgi?id=603030) | **-2.15** |
| 1442977_AT | gb:BG075843 /DB_XREF=gi:12558412 /DB_XREF=H3152D12 |  |  |  | **-2.02** |
| 1443017_AT | cytoplasmic polyadenylation element binding protein 2 | [CPEB2](http://www.gene.ucl.ac.uk/cgi-bin/nomenclature/searchgenes.pl?field=symbol&anchor=equals&symbol_search=Search&number=100&format=html&sortby=symbol&match=CPEB2) |  |  | **-1.91** |
| 1443018_AT | Neural cell adhesion molecule 1 | [NCAM1](http://www.gene.ucl.ac.uk/cgi-bin/nomenclature/searchgenes.pl?field=symbol&anchor=equals&symbol_search=Search&number=100&format=html&sortby=symbol&match=NCAM1) |  | [116930](http://www.ncbi.nlm.nih.gov/entrez/dispomim.cgi?id=116930) | **-2.69** |
| 1443161_AT | gb:BM240648 /DB_XREF=gi:17875918 /DB_XREF=K0605C04 |  |  |  | **-1.37** |
| 1443162_AT | gb:BB043897 /DB_XREF=gi:16259271 /DB_XREF=BB043897 |  |  |  | **1.6** |
| 1443299_AT | gb:BB480432 /DB_XREF=gi:16440686 /DB_XREF=BB480432 |  |  |  | **-2.11** |
| 1443341_AT | gb:BM231350 /DB_XREF=gi:17866620 /DB_XREF=K0308F06 |  |  |  | **-2.53** |
| 1443575_AT | RIKEN cDNA 2310040G24 gene | [2310040G24RIK](http://www.gene.ucl.ac.uk/cgi-bin/nomenclature/searchgenes.pl?field=symbol&anchor=equals&symbol_search=Search&number=100&format=html&sortby=symbol&match=2310040G24RIK) |  |  | **2.23** |
| 1443632_AT | obscurin, cytoskeletal calmodulin and titin-interacting RhoGEF | [BC046431](http://www.gene.ucl.ac.uk/cgi-bin/nomenclature/searchgenes.pl?field=symbol&anchor=equals&symbol_search=Search&number=100&format=html&sortby=symbol&match=BC046431) |  |  | **1.41** |
| 1443715_AT | Similar to hypothetical protein FLJ30829; RNA-binding region (RNP1, RRM) containing 6 | [LOC380843](http://www.gene.ucl.ac.uk/cgi-bin/nomenclature/searchgenes.pl?field=symbol&anchor=equals&symbol_search=Search&number=100&format=html&sortby=symbol&match=LOC380843) |  |  | **-1.48** |
| 1443721_X_AT | cDNA sequence BC019206 | [BC019206](http://www.gene.ucl.ac.uk/cgi-bin/nomenclature/searchgenes.pl?field=symbol&anchor=equals&symbol_search=Search&number=100&format=html&sortby=symbol&match=BC019206) |  |  | **-1.71** |
| 1443783_X_AT | gb:AV086906 /DB_XREF=gi:5218354 /DB_XREF=AV086906 |  |  |  | **-3.05** |
| 1443814_X_AT | cathepsin H | [CTSH](http://www.gene.ucl.ac.uk/cgi-bin/nomenclature/searchgenes.pl?field=symbol&anchor=equals&symbol_search=Search&number=100&format=html&sortby=symbol&match=CTSH) |  | [116820](http://www.ncbi.nlm.nih.gov/entrez/dispomim.cgi?id=116820) | **-2.1** |
| 1443822_S_AT | gb:AV347904 /DB_XREF=gi:6388963 /DB_XREF=AV347904 |  |  |  | **1.45** |
| 1443823_S_AT | gb:AV325919 /DB_XREF=gi:6365971 /DB_XREF=AV325919 |  |  |  | **1.24** |
| 1443827_X_AT | cDNA sequence BC004044 | [BC004044](http://www.gene.ucl.ac.uk/cgi-bin/nomenclature/searchgenes.pl?field=symbol&anchor=equals&symbol_search=Search&number=100&format=html&sortby=symbol&match=BC004044) |  |  | **-1.9** |
| 1443832_S_AT | serum deprivation response | [SDPR](http://www.gene.ucl.ac.uk/cgi-bin/nomenclature/searchgenes.pl?field=symbol&anchor=equals&symbol_search=Search&number=100&format=html&sortby=symbol&match=SDPR) |  | [606728](http://www.ncbi.nlm.nih.gov/entrez/dispomim.cgi?id=606728) | **1.92** |
| 1443847_X_AT | gb:BB342212 /DB_XREF=gi:9051921 /DB_XREF=BB342212 |  |  |  | **-1.62** |
| 1443866_AT | RIKEN cDNA A930016D02 gene | [A930016D02RIK](http://www.gene.ucl.ac.uk/cgi-bin/nomenclature/searchgenes.pl?field=symbol&anchor=equals&symbol_search=Search&number=100&format=html&sortby=symbol&match=A930016D02RIK) |  |  | **3.78** |
| 1443870_AT | ATP-binding cassette, sub-family C (CFTR/MRP), member 4 | [ABCC4](http://www.gene.ucl.ac.uk/cgi-bin/nomenclature/searchgenes.pl?field=symbol&anchor=equals&symbol_search=Search&number=100&format=html&sortby=symbol&match=ABCC4) |  | [605250](http://www.ncbi.nlm.nih.gov/entrez/dispomim.cgi?id=605250) | **1.8** |
| 1443873_AT | DNA segment, Chr 18, ERATO Doi 653, expressed | [D18ERTD653E](http://www.gene.ucl.ac.uk/cgi-bin/nomenclature/searchgenes.pl?field=symbol&anchor=equals&symbol_search=Search&number=100&format=html&sortby=symbol&match=D18ERTD653E) |  |  | **1.64** |
| 1443901_AT | gb:BB481579 /DB_XREF=gi:16440825 /DB_XREF=BB481579 |  |  |  | **1.35** |
| 1443904_AT | cDNA sequence BC044804 | [BC044804](http://www.gene.ucl.ac.uk/cgi-bin/nomenclature/searchgenes.pl?field=symbol&anchor=equals&symbol_search=Search&number=100&format=html&sortby=symbol&match=BC044804) |  |  | **1.78** |
| 1443932_AT | kelch domain containing 1 | [KLHDC1](http://www.gene.ucl.ac.uk/cgi-bin/nomenclature/searchgenes.pl?field=symbol&anchor=equals&symbol_search=Search&number=100&format=html&sortby=symbol&match=KLHDC1) |  |  | **1.55** |
| 1443960_AT | gb:BQ175377 /DB_XREF=gi:20350869 /DB_XREF=UI-M-DJ2 |  |  |  | **1.52** |
| 1443962_AT | RIKEN cDNA A330080J22 gene | [TFDP2](http://www.gene.ucl.ac.uk/cgi-bin/nomenclature/searchgenes.pl?field=symbol&anchor=equals&symbol_search=Search&number=100&format=html&sortby=symbol&match=TFDP2) |  | [602160](http://www.ncbi.nlm.nih.gov/entrez/dispomim.cgi?id=602160) | **1.48** |
| 1444028_S_AT | dedicator of cytokinesis 9 | [DOCK9](http://www.gene.ucl.ac.uk/cgi-bin/nomenclature/searchgenes.pl?field=symbol&anchor=equals&symbol_search=Search&number=100&format=html&sortby=symbol&match=DOCK9) |  | [607325](http://www.ncbi.nlm.nih.gov/entrez/dispomim.cgi?id=607325) | **1.43** |
| 1444085_AT | RIKEN cDNA 5430420P03 gene | [5430420P03RIK](http://www.gene.ucl.ac.uk/cgi-bin/nomenclature/searchgenes.pl?field=symbol&anchor=equals&symbol_search=Search&number=100&format=html&sortby=symbol&match=5430420P03RIK) |  |  | **1.45** |
| 1444089_AT | spectrin beta 2 | [SPNB2](http://www.gene.ucl.ac.uk/cgi-bin/nomenclature/searchgenes.pl?field=symbol&anchor=equals&symbol_search=Search&number=100&format=html&sortby=symbol&match=SPNB2) |  |  | **-1.47** |
| 1444091_A_AT | RIKEN cDNA 6430571L13 gene | [6430571L13RIK](http://www.gene.ucl.ac.uk/cgi-bin/nomenclature/searchgenes.pl?field=symbol&anchor=equals&symbol_search=Search&number=100&format=html&sortby=symbol&match=6430571L13RIK) |  |  | **2.15** |
| 1444109_AT | RIKEN cDNA C130009A20 gene | [C130009A20RIK](http://www.gene.ucl.ac.uk/cgi-bin/nomenclature/searchgenes.pl?field=symbol&anchor=equals&symbol_search=Search&number=100&format=html&sortby=symbol&match=C130009A20RIK) |  |  | **1.53** |
| 1444112_AT | gb:BQ174086 /DB_XREF=gi:20349577 /DB_XREF=UI-M-DJ2 |  |  |  | **1.42** |
| 1444141_AT | sorting nexin 13 | [SNX13](http://www.gene.ucl.ac.uk/cgi-bin/nomenclature/searchgenes.pl?field=symbol&anchor=equals&symbol_search=Search&number=100&format=html&sortby=symbol&match=SNX13) |  | [606589](http://www.ncbi.nlm.nih.gov/entrez/dispomim.cgi?id=606589) | **1.42** |
| 1444144_AT | gb:AI661895 /DB_XREF=gi:4765478 /DB_XREF=mz58c03.x |  |  |  | **-1.52** |
| 1444157_A_AT | jumonji, AT rich interactive domain 1C (Rbp2 like) | [JARID1C](http://www.gene.ucl.ac.uk/cgi-bin/nomenclature/searchgenes.pl?field=symbol&anchor=equals&symbol_search=Search&number=100&format=html&sortby=symbol&match=JARID1C) |  | [314690](http://www.ncbi.nlm.nih.gov/entrez/dispomim.cgi?id=314690) | **-1.46** |
| 1444176_AT | ATPase, H+ transporting, V0 subunit D, isoform 2 | [ATP6V0D2](http://www.gene.ucl.ac.uk/cgi-bin/nomenclature/searchgenes.pl?field=symbol&anchor=equals&symbol_search=Search&number=100&format=html&sortby=symbol&match=ATP6V0D2) |  |  | **-3.55** |
| 1444232_AT | protein kinase, cGMP-dependent, type I | [PRKG1](http://www.gene.ucl.ac.uk/cgi-bin/nomenclature/searchgenes.pl?field=symbol&anchor=equals&symbol_search=Search&number=100&format=html&sortby=symbol&match=PRKG1) |  | [176894](http://www.ncbi.nlm.nih.gov/entrez/dispomim.cgi?id=176894) | **2.16** |
| 1444302_AT | cDNA sequence BC049816 | [BC049816](http://www.gene.ucl.ac.uk/cgi-bin/nomenclature/searchgenes.pl?field=symbol&anchor=equals&symbol_search=Search&number=100&format=html&sortby=symbol&match=BC049816) |  |  | **-2.1** |
| 1444307_AT | gb:AW491448 /DB_XREF=gi:7061729 /DB_XREF=UI-M-BH3- |  |  |  | **2.85** |
| 1444341_AT | RIKEN cDNA 8030451F13 gene | [8030451F13RIK](http://www.gene.ucl.ac.uk/cgi-bin/nomenclature/searchgenes.pl?field=symbol&anchor=equals&symbol_search=Search&number=100&format=html&sortby=symbol&match=8030451F13RIK) |  |  | **1.24** |
| 1444343_AT | RIKEN cDNA E030024I16 gene | [E030024I16RIK](http://www.gene.ucl.ac.uk/cgi-bin/nomenclature/searchgenes.pl?field=symbol&anchor=equals&symbol_search=Search&number=100&format=html&sortby=symbol&match=E030024I16RIK) |  |  | **-1.59** |
| 1444409_AT | gb:BQ176035 /DB_XREF=gi:20351527 /DB_XREF=UI-M-DJ2 |  |  |  | **1.78** |
| 1444422_AT | Protocadherin 19 | [PCDH19](http://www.gene.ucl.ac.uk/cgi-bin/nomenclature/searchgenes.pl?field=symbol&anchor=equals&symbol_search=Search&number=100&format=html&sortby=symbol&match=PCDH19) |  | [300460](http://www.ncbi.nlm.nih.gov/entrez/dispomim.cgi?id=300460) | **1.47** |
| 1444429_AT | Calcium channel, voltage dependent, alpha2/delta subunit 3 | [CACNA2D3](http://www.gene.ucl.ac.uk/cgi-bin/nomenclature/searchgenes.pl?field=symbol&anchor=equals&symbol_search=Search&number=100&format=html&sortby=symbol&match=CACNA2D3) |  | [606399](http://www.ncbi.nlm.nih.gov/entrez/dispomim.cgi?id=606399) | **2.98** |
| 1444460_AT | ubiquitin-conjugating enzyme E2R 2 | [UBE2R2](http://www.gene.ucl.ac.uk/cgi-bin/nomenclature/searchgenes.pl?field=symbol&anchor=equals&symbol_search=Search&number=100&format=html&sortby=symbol&match=UBE2R2) |  |  | **1.22** |
| 1444480_AT | gb:BB151516 /DB_XREF=gi:16267942 /DB_XREF=BB151516 |  |  |  | **-4.29** |
| 1444494_AT | Kelch repeat and BTB (POZ) domain containing 10 | [KBTBD10](http://www.gene.ucl.ac.uk/cgi-bin/nomenclature/searchgenes.pl?field=symbol&anchor=equals&symbol_search=Search&number=100&format=html&sortby=symbol&match=KBTBD10) |  | [607701](http://www.ncbi.nlm.nih.gov/entrez/dispomim.cgi?id=607701) | **-1.3** |
| 1444504_AT | RIKEN cDNA 1110001P11 gene | [1110001P11RIK](http://www.gene.ucl.ac.uk/cgi-bin/nomenclature/searchgenes.pl?field=symbol&anchor=equals&symbol_search=Search&number=100&format=html&sortby=symbol&match=1110001P11RIK) |  |  | **1.62** |
| 1444511_AT | solute carrier family 41, member 1 | [SLC41A1](http://www.gene.ucl.ac.uk/cgi-bin/nomenclature/searchgenes.pl?field=symbol&anchor=equals&symbol_search=Search&number=100&format=html&sortby=symbol&match=SLC41A1) |  |  | **2.03** |
| 1444525_AT | gb:BM238996 /DB_XREF=gi:17874266 /DB_XREF=K0527B01 |  |  |  | **1.85** |
| 1444531_AT | Adult male urinary bladder cDNA, RIKEN full-length enriched library, clone:9530055M18 product:unknown EST, full insert sequence |  |  |  | **1.71** |
| 1444612_AT | RIKEN cDNA 3222402P14 gene | [3222402P14RIK](http://www.gene.ucl.ac.uk/cgi-bin/nomenclature/searchgenes.pl?field=symbol&anchor=equals&symbol_search=Search&number=100&format=html&sortby=symbol&match=3222402P14RIK) |  |  | **1.25** |
| 1444643_AT | gb:BB133024 /DB_XREF=gi:16266511 /DB_XREF=BB133024 |  |  |  | **1.43** |
| 1444665_AT | RIKEN cDNA 4930402E16 gene | [4930402E16RIK](http://www.gene.ucl.ac.uk/cgi-bin/nomenclature/searchgenes.pl?field=symbol&anchor=equals&symbol_search=Search&number=100&format=html&sortby=symbol&match=4930402E16RIK) |  |  | **2.15** |
| 1444789_AT | gb:AI427762 /DB_XREF=gi:4273688 /DB_XREF=mk18b02.x |  |  |  | **-1.21** |
| 1445203_AT | PDZ domain containing 3 | [PDZK3](http://www.gene.ucl.ac.uk/cgi-bin/nomenclature/searchgenes.pl?field=symbol&anchor=equals&symbol_search=Search&number=100&format=html&sortby=symbol&match=PDZK3) |  |  | **1.51** |
| 1445315_AT | gb:BB266062 /DB_XREF=gi:16400327 /DB_XREF=BB266062 |  |  |  | **1.53** |
| 1445443_AT | gb:BM207275 /DB_XREF=gi:17763037 /DB_XREF=C0610E06 |  |  |  | **-1.47** |
| 1445518_AT | gb:BG061923 /DB_XREF=gi:12531906 /DB_XREF=L0953F01 |  |  |  | **-1.54** |
| 1445526_AT | gb:AU045440 /DB_XREF=gi:3981623 /DB_XREF=AU045440 |  |  |  | **1.95** |
| 1445534_AT | gb:BM206272 /DB_XREF=gi:17761690 /DB_XREF=C0295D07 |  |  |  | **1.48** |
| 1445597_S_AT | HRAS like suppressor 3 | [HRASLS3](http://www.gene.ucl.ac.uk/cgi-bin/nomenclature/searchgenes.pl?field=symbol&anchor=equals&symbol_search=Search&number=100&format=html&sortby=symbol&match=HRASLS3) |  |  | **1.73** |
| 1445626_AT | gb:AI426376 /DB_XREF=gi:4272302 /DB_XREF=mm48c01.x |  |  |  | **-4.82** |
| 1445632_AT | gb:BB735612 /DB_XREF=gi:16134762 /DB_XREF=BB735612 |  |  |  | **1.34** |
| 1445684_S_AT | histone deacetylase 2 | [HDAC2](http://www.gene.ucl.ac.uk/cgi-bin/nomenclature/searchgenes.pl?field=symbol&anchor=equals&symbol_search=Search&number=100&format=html&sortby=symbol&match=HDAC2) |  | [605164](http://www.ncbi.nlm.nih.gov/entrez/dispomim.cgi?id=605164) | **-1.29** |
| 1445758_AT | gb:BG076276 /DB_XREF=gi:12558845 /DB_XREF=H3157G06 |  |  |  | **-1.86** |
| 1445841_AT | RIKEN cDNA 9430028I06 gene | [9430028I06RIK](http://www.gene.ucl.ac.uk/cgi-bin/nomenclature/searchgenes.pl?field=symbol&anchor=equals&symbol_search=Search&number=100&format=html&sortby=symbol&match=9430028I06RIK) |  |  | **1.61** |
| 1445868_AT | cytoplasmic polyadenylation element binding protein 3 | [CPEB3](http://www.gene.ucl.ac.uk/cgi-bin/nomenclature/searchgenes.pl?field=symbol&anchor=equals&symbol_search=Search&number=100&format=html&sortby=symbol&match=CPEB3) |  |  | **1.56** |
| 1445882_AT | gb:BB230267 /DB_XREF=gi:16354366 /DB_XREF=BB230267 |  |  |  | **-1.89** |
| 1445897_S_AT | interferon-induced protein 35 | [IFI35](http://www.gene.ucl.ac.uk/cgi-bin/nomenclature/searchgenes.pl?field=symbol&anchor=equals&symbol_search=Search&number=100&format=html&sortby=symbol&match=IFI35) |  | [600735](http://www.ncbi.nlm.nih.gov/entrez/dispomim.cgi?id=600735) | **-1.24** |
| 1445966_AT | gb:BG075586 /DB_XREF=gi:12558155 /DB_XREF=H3149A09 |  |  |  | **-1.45** |
| 1446069_AT | RIKEN cDNA D930036B08 gene | [D930036B08RIK](http://www.gene.ucl.ac.uk/cgi-bin/nomenclature/searchgenes.pl?field=symbol&anchor=equals&symbol_search=Search&number=100&format=html&sortby=symbol&match=D930036B08RIK) |  |  | **1.63** |
| 1446190_AT | gb:BB269897 /DB_XREF=gi:16400580 /DB_XREF=BB269897 |  |  |  | **-2.31** |
| 1446326_AT | procollagen, type I, alpha 2 | [COL1A2](http://www.gene.ucl.ac.uk/cgi-bin/nomenclature/searchgenes.pl?field=symbol&anchor=equals&symbol_search=Search&number=100&format=html&sortby=symbol&match=COL1A2) |  | [120160](http://www.ncbi.nlm.nih.gov/entrez/dispomim.cgi?id=120160) | **-2.16** |
| 1446353_AT | RIKEN cDNA 2310057H16 gene | [TUBB6](http://www.gene.ucl.ac.uk/cgi-bin/nomenclature/searchgenes.pl?field=symbol&anchor=equals&symbol_search=Search&number=100&format=html&sortby=symbol&match=TUBB6) |  |  | **2.11** |
| 1446417_AT | gb:AW553625 /DB_XREF=gi:7199048 /DB_XREF=L0229F06- |  |  |  | **-1.89** |
| 1446542_AT | gb:AW553564 /DB_XREF=gi:7198987 /DB_XREF=L0228G09- |  |  |  | **1.63** |
| 1446563_AT | 13 days embryo forelimb cDNA, RIKEN full-length enriched library, clone:5930439P04 product:unknown EST, full insert sequence |  |  |  | **-5.35** |
| 1446619_AT | RIKEN cDNA A130038J17 gene | [A130038J17RIK](http://www.gene.ucl.ac.uk/cgi-bin/nomenclature/searchgenes.pl?field=symbol&anchor=equals&symbol_search=Search&number=100&format=html&sortby=symbol&match=A130038J17RIK) |  |  | **2.47** |
| 1446771_AT | gb:BB047533 /DB_XREF=gi:15403989 /DB_XREF=BB047533 |  |  |  | **1.5** |
| 1446921_AT | gb:BM204670 /DB_XREF=gi:17759575 /DB_XREF=C0272G10 |  |  |  | **-1.94** |
| 1446928_AT | RIKEN cDNA 1700025B16 gene | [1700025B16RIK](http://www.gene.ucl.ac.uk/cgi-bin/nomenclature/searchgenes.pl?field=symbol&anchor=equals&symbol_search=Search&number=100&format=html&sortby=symbol&match=1700025B16RIK) |  |  | **-2.1** |
| 1446930_AT | gb:BI180598 /DB_XREF=gi:14646409 /DB_XREF=LRH16C4 |  |  |  | **-5.38** |
| 1446967_AT | 0 day neonate eyeball cDNA, RIKEN full-length enriched library, clone:E130106L10 product:unclassifiable, full insert sequence |  |  |  | **1.33** |
| 1447057_AT | gb:BB343027 /DB_XREF=gi:16404608 /DB_XREF=BB343027 |  |  |  | **1.45** |
| 1447184_AT | gb:AU021760 /DB_XREF=gi:3386719 /DB_XREF=AU021760 |  |  |  | **2.32** |
| 1447223_AT | gb:AW120584 /DB_XREF=gi:6095917 /DB_XREF=UI-M-BH1- |  |  |  | **1.82** |
| 1447234_S_AT | sorting nexin 6 | [SNX6](http://www.gene.ucl.ac.uk/cgi-bin/nomenclature/searchgenes.pl?field=symbol&anchor=equals&symbol_search=Search&number=100&format=html&sortby=symbol&match=SNX6) |  | [606098](http://www.ncbi.nlm.nih.gov/entrez/dispomim.cgi?id=606098) | **-1.62** |
| 1447250_A_AT | RIKEN cDNA 2610301F02 gene | [2610301F02RIK](http://www.gene.ucl.ac.uk/cgi-bin/nomenclature/searchgenes.pl?field=symbol&anchor=equals&symbol_search=Search&number=100&format=html&sortby=symbol&match=2610301F02RIK) |  |  | **-8.66** |
| 1447255_AT | RIKEN cDNA 2310015A10 gene | [2310015A10RIK](http://www.gene.ucl.ac.uk/cgi-bin/nomenclature/searchgenes.pl?field=symbol&anchor=equals&symbol_search=Search&number=100&format=html&sortby=symbol&match=2310015A10RIK) |  |  | **1.6** |
| 1447320_X_AT | RNA polymerase 1-3 | [RPO1-3](http://www.gene.ucl.ac.uk/cgi-bin/nomenclature/searchgenes.pl?field=symbol&anchor=equals&symbol_search=Search&number=100&format=html&sortby=symbol&match=RPO1-3) |  |  | **-1.53** |
| 1447322_AT | gb:AI845029 /DB_XREF=gi:5488935 /DB_XREF=UI-M-BG0- |  |  |  | **1.86** |
| 1447396_AT | gb:BG072185 /DB_XREF=gi:12554754 /DB_XREF=H3107G03 |  |  |  | **2.05** |
| 1447475_AT | Gene model 967, (NCBI) | [AI414343](http://www.gene.ucl.ac.uk/cgi-bin/nomenclature/searchgenes.pl?field=symbol&anchor=equals&symbol_search=Search&number=100&format=html&sortby=symbol&match=AI414343) |  |  | **1.47** |
| 1447522_S_AT | gb:AI662480 /DB_XREF=gi:4766063 /DB_XREF=mt28h09.x |  |  |  | **1.3** |
| 1447541_S_AT | integrin, alpha E, epithelial-associated | [ITGAE](http://www.gene.ucl.ac.uk/cgi-bin/nomenclature/searchgenes.pl?field=symbol&anchor=equals&symbol_search=Search&number=100&format=html&sortby=symbol&match=ITGAE) |  | [604682](http://www.ncbi.nlm.nih.gov/entrez/dispomim.cgi?id=604682) | **1.62** |
| 1447584_S_AT | gb:AI642973 /DB_XREF=gi:4721448 /DB_XREF=vd98a02.x |  |  |  | **2.02** |
| 1447585_S_AT | mitochondrial ribosomal protein S6 | [MRPS6](http://www.gene.ucl.ac.uk/cgi-bin/nomenclature/searchgenes.pl?field=symbol&anchor=equals&symbol_search=Search&number=100&format=html&sortby=symbol&match=MRPS6) |  |  | **1.39** |
| 1447621_S_AT | RIKEN cDNA 2610307O08 gene | [2610307O08RIK](http://www.gene.ucl.ac.uk/cgi-bin/nomenclature/searchgenes.pl?field=symbol&anchor=equals&symbol_search=Search&number=100&format=html&sortby=symbol&match=2610307O08RIK) |  |  | **-3.45** |
| 1447640_S_AT | gb:BB554874 /DB_XREF=gi:9641240 /DB_XREF=BB554874 |  |  |  | **-1.43** |
| 1447657_S_AT | synaptopodin 2-like | [SYNPO2L](http://www.gene.ucl.ac.uk/cgi-bin/nomenclature/searchgenes.pl?field=symbol&anchor=equals&symbol_search=Search&number=100&format=html&sortby=symbol&match=SYNPO2L) |  |  | **-1.71** |
| 1447679_S_AT | BMS1-like, ribosome assembly protein (yeast) | [BMS1L](http://www.gene.ucl.ac.uk/cgi-bin/nomenclature/searchgenes.pl?field=symbol&anchor=equals&symbol_search=Search&number=100&format=html&sortby=symbol&match=BMS1L) |  |  | **-1.23** |
| 1447693_S_AT | gb:BB350308 /DB_XREF=gi:9062136 /DB_XREF=BB350308 |  |  |  | **1.3** |
| 1447754_X_AT | THAP domain containing 4 | [THAP4](http://www.gene.ucl.ac.uk/cgi-bin/nomenclature/searchgenes.pl?field=symbol&anchor=equals&symbol_search=Search&number=100&format=html&sortby=symbol&match=THAP4) |  |  | **1.28** |
| 1447774_X_AT | RIKEN cDNA 5730469M10 gene | [5730469M10RIK](http://www.gene.ucl.ac.uk/cgi-bin/nomenclature/searchgenes.pl?field=symbol&anchor=equals&symbol_search=Search&number=100&format=html&sortby=symbol&match=5730469M10RIK) |  |  | **2.47** |
| 1447803_X_AT | gb:BB136012 /DB_XREF=gi:8790949 /DB_XREF=BB136012 |  |  |  | **-1.7** |
| 1447806_S_AT | serine/threonine kinase 23 | [STK23](http://www.gene.ucl.ac.uk/cgi-bin/nomenclature/searchgenes.pl?field=symbol&anchor=equals&symbol_search=Search&number=100&format=html&sortby=symbol&match=STK23) |  |  | **-1.7** |
| 1447807_S_AT | Pleckstrin homology domain containing, family H (with MyTH4 domain) member 1 | [PLEKHH1](http://www.gene.ucl.ac.uk/cgi-bin/nomenclature/searchgenes.pl?field=symbol&anchor=equals&symbol_search=Search&number=100&format=html&sortby=symbol&match=PLEKHH1) |  |  | **-1.27** |
| 1447813_X_AT | gb:AV247013 /DB_XREF=gi:6234472 /DB_XREF=AV247013 |  |  |  | **-3.86** |
| 1447816_X_AT | cDNA sequence BC019806 | [BC019806](http://www.gene.ucl.ac.uk/cgi-bin/nomenclature/searchgenes.pl?field=symbol&anchor=equals&symbol_search=Search&number=100&format=html&sortby=symbol&match=BC019806) |  |  | **1.33** |
| 1447819_X_AT | gb:BB546487 /DB_XREF=gi:9617915 /DB_XREF=BB546487 |  |  |  | **-1.64** |
| 1447822_X_AT | RIKEN cDNA 2700038N03 gene | [2700038N03RIK](http://www.gene.ucl.ac.uk/cgi-bin/nomenclature/searchgenes.pl?field=symbol&anchor=equals&symbol_search=Search&number=100&format=html&sortby=symbol&match=2700038N03RIK) |  |  | **1.58** |
| 1447852_X_AT | RIKEN cDNA 2900002H16 gene | [2900002H16RIK](http://www.gene.ucl.ac.uk/cgi-bin/nomenclature/searchgenes.pl?field=symbol&anchor=equals&symbol_search=Search&number=100&format=html&sortby=symbol&match=2900002H16RIK) |  |  | **1.4** |
| 1447854_S_AT | histone 2, H2bb | [HIST2H2BB](http://www.gene.ucl.ac.uk/cgi-bin/nomenclature/searchgenes.pl?field=symbol&anchor=equals&symbol_search=Search&number=100&format=html&sortby=symbol&match=HIST2H2BB) |  |  | **1.32** |
| 1447878_S_AT | fibroblast growth factor receptor-like 1 | [FGFRL1](http://www.gene.ucl.ac.uk/cgi-bin/nomenclature/searchgenes.pl?field=symbol&anchor=equals&symbol_search=Search&number=100&format=html&sortby=symbol&match=FGFRL1) |  | [605830](http://www.ncbi.nlm.nih.gov/entrez/dispomim.cgi?id=605830) | **-1.7** |
| 1447881_X_AT | gb:BB152563 /DB_XREF=gi:8807500 /DB_XREF=BB152563 |  |  |  | **1.43** |
| 1447883_X_AT | microtubule-associated protein 1 light chain 3 alpha | [MAP1LC3A](http://www.gene.ucl.ac.uk/cgi-bin/nomenclature/searchgenes.pl?field=symbol&anchor=equals&symbol_search=Search&number=100&format=html&sortby=symbol&match=MAP1LC3A) |  | [601242](http://www.ncbi.nlm.nih.gov/entrez/dispomim.cgi?id=601242) | **-1.36** |
| 1447900_X_AT | ectonucleoside triphosphate diphosphohydrolase 4 | [ENTPD4](http://www.gene.ucl.ac.uk/cgi-bin/nomenclature/searchgenes.pl?field=symbol&anchor=equals&symbol_search=Search&number=100&format=html&sortby=symbol&match=ENTPD4) |  | [607577](http://www.ncbi.nlm.nih.gov/entrez/dispomim.cgi?id=607577) | **1.51** |
| 1447919_X_AT | NADH dehydrogenase (ubiquinone) 1, alpha/beta subcomplex, 1 | [NDUFAB1](http://www.gene.ucl.ac.uk/cgi-bin/nomenclature/searchgenes.pl?field=symbol&anchor=equals&symbol_search=Search&number=100&format=html&sortby=symbol&match=NDUFAB1) |  | [603836](http://www.ncbi.nlm.nih.gov/entrez/dispomim.cgi?id=603836) | **1.37** |
| 1447930_AT | CDNA sequence BC065123 | [BC065123](http://www.gene.ucl.ac.uk/cgi-bin/nomenclature/searchgenes.pl?field=symbol&anchor=equals&symbol_search=Search&number=100&format=html&sortby=symbol&match=BC065123) |  |  | **-1.66** |
| 1447934_AT | RIKEN cDNA 9630033F20 gene | [9630033F20RIK](http://www.gene.ucl.ac.uk/cgi-bin/nomenclature/searchgenes.pl?field=symbol&anchor=equals&symbol_search=Search&number=100&format=html&sortby=symbol&match=9630033F20RIK) |  |  | **2.08** |
| 1447936_AT | RIKEN cDNA 2410006H16 gene | [2410006H16RIK](http://www.gene.ucl.ac.uk/cgi-bin/nomenclature/searchgenes.pl?field=symbol&anchor=equals&symbol_search=Search&number=100&format=html&sortby=symbol&match=2410006H16RIK) |  |  | **-1.66** |
| 1448013_AT | ubiquitin specific protease 24 | [USP24](http://www.gene.ucl.ac.uk/cgi-bin/nomenclature/searchgenes.pl?field=symbol&anchor=equals&symbol_search=Search&number=100&format=html&sortby=symbol&match=USP24) |  |  | **1.4** |
| 1448014_S_AT | ubiquitin specific protease 24 | [USP24](http://www.gene.ucl.ac.uk/cgi-bin/nomenclature/searchgenes.pl?field=symbol&anchor=equals&symbol_search=Search&number=100&format=html&sortby=symbol&match=USP24) |  |  | **1.44** |
| 1448024_AT | RIKEN cDNA B430320C24 gene | [B430320C24RIK](http://www.gene.ucl.ac.uk/cgi-bin/nomenclature/searchgenes.pl?field=symbol&anchor=equals&symbol_search=Search&number=100&format=html&sortby=symbol&match=B430320C24RIK) |  |  | **2.34** |
| 1448025_AT | gb:AI662854 /DB_XREF=gi:4766437 /DB_XREF=vb29c05.x |  |  |  | **-9.67** |
| 1448028_AT | RIKEN cDNA C530046L02 gene | [C530046L02RIK](http://www.gene.ucl.ac.uk/cgi-bin/nomenclature/searchgenes.pl?field=symbol&anchor=equals&symbol_search=Search&number=100&format=html&sortby=symbol&match=C530046L02RIK) |  |  | **1.64** |
| 1448029_AT | T-box 3 | [TBX3](http://www.gene.ucl.ac.uk/cgi-bin/nomenclature/searchgenes.pl?field=symbol&anchor=equals&symbol_search=Search&number=100&format=html&sortby=symbol&match=TBX3) |  | [601621](http://www.ncbi.nlm.nih.gov/entrez/dispomim.cgi?id=601621) | **1.41** |
| 1448061_AT | gb:AA183642 /DB_XREF=gi:1767802 /DB_XREF=mt31d02.r |  |  |  | **-1.9** |
| 1448100_AT | RIKEN cDNA 4833439L19 gene | [4833439L19RIK](http://www.gene.ucl.ac.uk/cgi-bin/nomenclature/searchgenes.pl?field=symbol&anchor=equals&symbol_search=Search&number=100&format=html&sortby=symbol&match=4833439L19RIK) |  |  | **1.38** |
| 1448103_S_AT | non-POU-domain-containing, octamer binding protein | [NONO](http://www.gene.ucl.ac.uk/cgi-bin/nomenclature/searchgenes.pl?field=symbol&anchor=equals&symbol_search=Search&number=100&format=html&sortby=symbol&match=NONO) |  | [300084](http://www.ncbi.nlm.nih.gov/entrez/dispomim.cgi?id=300084) | **-1.28** |
| 1448104_AT | aldehyde dehydrogenase family 6, subfamily A1 | [ALDH6A1](http://www.gene.ucl.ac.uk/cgi-bin/nomenclature/searchgenes.pl?field=symbol&anchor=equals&symbol_search=Search&number=100&format=html&sortby=symbol&match=ALDH6A1) |  | [603178](http://www.ncbi.nlm.nih.gov/entrez/dispomim.cgi?id=603178) | **1.58** |
| 1448106_AT | RIKEN cDNA 1200016B17 gene | [1200016B17RIK](http://www.gene.ucl.ac.uk/cgi-bin/nomenclature/searchgenes.pl?field=symbol&anchor=equals&symbol_search=Search&number=100&format=html&sortby=symbol&match=1200016B17RIK) |  |  | **-1.3** |
| 1448117_AT | kit ligand | [KITL](http://www.gene.ucl.ac.uk/cgi-bin/nomenclature/searchgenes.pl?field=symbol&anchor=equals&symbol_search=Search&number=100&format=html&sortby=symbol&match=KITL) |  |  | **1.89** |
| 1448119_AT | 2,3-bisphosphoglycerate mutase | [BPGM](http://www.gene.ucl.ac.uk/cgi-bin/nomenclature/searchgenes.pl?field=symbol&anchor=equals&symbol_search=Search&number=100&format=html&sortby=symbol&match=BPGM) |  | [222800](http://www.ncbi.nlm.nih.gov/entrez/dispomim.cgi?id=222800) | **1.74** |
| 1448121_AT | WW domain binding protein 2 | [WBP2](http://www.gene.ucl.ac.uk/cgi-bin/nomenclature/searchgenes.pl?field=symbol&anchor=equals&symbol_search=Search&number=100&format=html&sortby=symbol&match=WBP2) |  | [606962](http://www.ncbi.nlm.nih.gov/entrez/dispomim.cgi?id=606962) | **1.48** |
| 1448123_S_AT | gb:NM_009369.1 /DB_XREF=gi:6678320 /GEN=Tgfbi /FEA |  |  |  | **-2.16** |
| 1448124_AT | glucuronidase, beta | [GUSB](http://www.gene.ucl.ac.uk/cgi-bin/nomenclature/searchgenes.pl?field=symbol&anchor=equals&symbol_search=Search&number=100&format=html&sortby=symbol&match=GUSB) |  | [253220](http://www.ncbi.nlm.nih.gov/entrez/dispomim.cgi?id=253220) | **-1.95** |
| 1448128_AT | protective protein for beta-galactosidase | [PPGB](http://www.gene.ucl.ac.uk/cgi-bin/nomenclature/searchgenes.pl?field=symbol&anchor=equals&symbol_search=Search&number=100&format=html&sortby=symbol&match=PPGB) |  | [256540](http://www.ncbi.nlm.nih.gov/entrez/dispomim.cgi?id=256540) | **-1.47** |
| 1448129_AT | actin related protein 2/3 complex, subunit 5 | [ARPC5](http://www.gene.ucl.ac.uk/cgi-bin/nomenclature/searchgenes.pl?field=symbol&anchor=equals&symbol_search=Search&number=100&format=html&sortby=symbol&match=ARPC5) |  | [604227](http://www.ncbi.nlm.nih.gov/entrez/dispomim.cgi?id=604227) | **-1.98** |
| 1448130_AT | farnesyl diphosphate farnesyl transferase 1 | [FDFT1](http://www.gene.ucl.ac.uk/cgi-bin/nomenclature/searchgenes.pl?field=symbol&anchor=equals&symbol_search=Search&number=100&format=html&sortby=symbol&match=FDFT1) |  | [184420](http://www.ncbi.nlm.nih.gov/entrez/dispomim.cgi?id=184420) | **1.85** |
| 1448131_AT | mitofusin 2 | [MFN2](http://www.gene.ucl.ac.uk/cgi-bin/nomenclature/searchgenes.pl?field=symbol&anchor=equals&symbol_search=Search&number=100&format=html&sortby=symbol&match=MFN2) |  | [608507](http://www.ncbi.nlm.nih.gov/entrez/dispomim.cgi?id=608507) | **1.51** |
| 1448136_AT | ectonucleotide pyrophosphatase/phosphodiesterase 2 | [ENPP2](http://www.gene.ucl.ac.uk/cgi-bin/nomenclature/searchgenes.pl?field=symbol&anchor=equals&symbol_search=Search&number=100&format=html&sortby=symbol&match=ENPP2) |  | [601060](http://www.ncbi.nlm.nih.gov/entrez/dispomim.cgi?id=601060) | **1.55** |
| 1448143_AT | aldehyde dehydrogenase 2, mitochondrial | [ALDH2](http://www.gene.ucl.ac.uk/cgi-bin/nomenclature/searchgenes.pl?field=symbol&anchor=equals&symbol_search=Search&number=100&format=html&sortby=symbol&match=ALDH2) |  | [100650](http://www.ncbi.nlm.nih.gov/entrez/dispomim.cgi?id=100650) | **1.48** |
| 1448145_AT | WW domain containing E3 ubiquitin protein ligase 2 | [WWP2](http://www.gene.ucl.ac.uk/cgi-bin/nomenclature/searchgenes.pl?field=symbol&anchor=equals&symbol_search=Search&number=100&format=html&sortby=symbol&match=WWP2) |  | [602308](http://www.ncbi.nlm.nih.gov/entrez/dispomim.cgi?id=602308) | **-1.52** |
| 1448148_AT | granulin | [GRN](http://www.gene.ucl.ac.uk/cgi-bin/nomenclature/searchgenes.pl?field=symbol&anchor=equals&symbol_search=Search&number=100&format=html&sortby=symbol&match=GRN) |  | [138945](http://www.ncbi.nlm.nih.gov/entrez/dispomim.cgi?id=138945) | **-1.98** |
| 1448149_AT | catenin alpha 1 | [CATNA1](http://www.gene.ucl.ac.uk/cgi-bin/nomenclature/searchgenes.pl?field=symbol&anchor=equals&symbol_search=Search&number=100&format=html&sortby=symbol&match=CATNA1) |  |  | **-1.21** |
| 1448152_AT | insulin-like growth factor 2 | [IGF2](http://www.gene.ucl.ac.uk/cgi-bin/nomenclature/searchgenes.pl?field=symbol&anchor=equals&symbol_search=Search&number=100&format=html&sortby=symbol&match=IGF2) |  | [147470](http://www.ncbi.nlm.nih.gov/entrez/dispomim.cgi?id=147470) | **-12.56** |
| 1448153_AT | cytochrome c oxidase, subunit Va | [COX5A](http://www.gene.ucl.ac.uk/cgi-bin/nomenclature/searchgenes.pl?field=symbol&anchor=equals&symbol_search=Search&number=100&format=html&sortby=symbol&match=COX5A) |  | [603773](http://www.ncbi.nlm.nih.gov/entrez/dispomim.cgi?id=603773) | **1.21** |
| 1448154_AT | N-myc downstream regulated gene 2 | [NDRG2](http://www.gene.ucl.ac.uk/cgi-bin/nomenclature/searchgenes.pl?field=symbol&anchor=equals&symbol_search=Search&number=100&format=html&sortby=symbol&match=NDRG2) |  | [605272](http://www.ncbi.nlm.nih.gov/entrez/dispomim.cgi?id=605272) | **1.39** |
| 1448157_S_AT | ribosomal protein 10 | [RPL10](http://www.gene.ucl.ac.uk/cgi-bin/nomenclature/searchgenes.pl?field=symbol&anchor=equals&symbol_search=Search&number=100&format=html&sortby=symbol&match=RPL10) |  | [312173](http://www.ncbi.nlm.nih.gov/entrez/dispomim.cgi?id=312173) | **-1.2** |
| 1448160_AT | lymphocyte cytosolic protein 1 | [LCP1](http://www.gene.ucl.ac.uk/cgi-bin/nomenclature/searchgenes.pl?field=symbol&anchor=equals&symbol_search=Search&number=100&format=html&sortby=symbol&match=LCP1) |  | [153430](http://www.ncbi.nlm.nih.gov/entrez/dispomim.cgi?id=153430) | **-3.52** |
| 1448162_AT | vascular cell adhesion molecule 1 | [VCAM1](http://www.gene.ucl.ac.uk/cgi-bin/nomenclature/searchgenes.pl?field=symbol&anchor=equals&symbol_search=Search&number=100&format=html&sortby=symbol&match=VCAM1) |  | [192225](http://www.ncbi.nlm.nih.gov/entrez/dispomim.cgi?id=192225) | **-3.29** |
| 1448167_AT | interferon gamma receptor 1 | [IFNGR1](http://www.gene.ucl.ac.uk/cgi-bin/nomenclature/searchgenes.pl?field=symbol&anchor=equals&symbol_search=Search&number=100&format=html&sortby=symbol&match=IFNGR1) |  | [107470](http://www.ncbi.nlm.nih.gov/entrez/dispomim.cgi?id=107470) | **-1.33** |
| 1448169_AT | keratin complex 1, acidic, gene 18 | [KRT1-18](http://www.gene.ucl.ac.uk/cgi-bin/nomenclature/searchgenes.pl?field=symbol&anchor=equals&symbol_search=Search&number=100&format=html&sortby=symbol&match=KRT1-18) |  |  | **-1.9** |
| 1448172_AT | malate dehydrogenase 1, NAD (soluble) | [MDH1](http://www.gene.ucl.ac.uk/cgi-bin/nomenclature/searchgenes.pl?field=symbol&anchor=equals&symbol_search=Search&number=100&format=html&sortby=symbol&match=MDH1) |  | [154200](http://www.ncbi.nlm.nih.gov/entrez/dispomim.cgi?id=154200) | **1.31** |
| 1448174_AT | cullin 1 | [CUL1](http://www.gene.ucl.ac.uk/cgi-bin/nomenclature/searchgenes.pl?field=symbol&anchor=equals&symbol_search=Search&number=100&format=html&sortby=symbol&match=CUL1) |  | [603134](http://www.ncbi.nlm.nih.gov/entrez/dispomim.cgi?id=603134) | **-1.23** |
| 1448178_A_AT | chaperonin subunit 3 (gamma) | [CCT3](http://www.gene.ucl.ac.uk/cgi-bin/nomenclature/searchgenes.pl?field=symbol&anchor=equals&symbol_search=Search&number=100&format=html&sortby=symbol&match=CCT3) |  | [600114](http://www.ncbi.nlm.nih.gov/entrez/dispomim.cgi?id=600114) | **-1.33** |
| 1448180_A_AT | hematological and neurological expressed sequence 1 | [HN1](http://www.gene.ucl.ac.uk/cgi-bin/nomenclature/searchgenes.pl?field=symbol&anchor=equals&symbol_search=Search&number=100&format=html&sortby=symbol&match=HN1) |  |  | **-2.6** |
| 1448188_AT | uncoupling protein 2, mitochondrial | [UCP2](http://www.gene.ucl.ac.uk/cgi-bin/nomenclature/searchgenes.pl?field=symbol&anchor=equals&symbol_search=Search&number=100&format=html&sortby=symbol&match=UCP2) |  | [601693](http://www.ncbi.nlm.nih.gov/entrez/dispomim.cgi?id=601693) | **-1.57** |
| 1448193_AT | RIKEN cDNA 5730403B10 gene | [5730403B10RIK](http://www.gene.ucl.ac.uk/cgi-bin/nomenclature/searchgenes.pl?field=symbol&anchor=equals&symbol_search=Search&number=100&format=html&sortby=symbol&match=5730403B10RIK) |  |  | **1.27** |
| 1448198_A_AT | NADH dehydrogenase (ubiquinone) 1 beta subcomplex 8 | [NDUFB8](http://www.gene.ucl.ac.uk/cgi-bin/nomenclature/searchgenes.pl?field=symbol&anchor=equals&symbol_search=Search&number=100&format=html&sortby=symbol&match=NDUFB8) |  | [602140](http://www.ncbi.nlm.nih.gov/entrez/dispomim.cgi?id=602140) | **1.2** |
| 1448199_AT | ankyrin repeat domain 10 | [ANKRD10](http://www.gene.ucl.ac.uk/cgi-bin/nomenclature/searchgenes.pl?field=symbol&anchor=equals&symbol_search=Search&number=100&format=html&sortby=symbol&match=ANKRD10) |  |  | **-2.35** |
| 1448202_X_AT | RIKEN cDNA 2610524G07 gene | [2610524G07RIK](http://www.gene.ucl.ac.uk/cgi-bin/nomenclature/searchgenes.pl?field=symbol&anchor=equals&symbol_search=Search&number=100&format=html&sortby=symbol&match=2610524G07RIK) |  |  | **-1.95** |
| 1448208_AT | MAD homolog 1 (Drosophila) | [SMAD1](http://www.gene.ucl.ac.uk/cgi-bin/nomenclature/searchgenes.pl?field=symbol&anchor=equals&symbol_search=Search&number=100&format=html&sortby=symbol&match=SMAD1) |  | [601595](http://www.ncbi.nlm.nih.gov/entrez/dispomim.cgi?id=601595) | **-1.36** |
| 1448211_AT | RIKEN cDNA 0610006O14 gene | [0610006O14RIK](http://www.gene.ucl.ac.uk/cgi-bin/nomenclature/searchgenes.pl?field=symbol&anchor=equals&symbol_search=Search&number=100&format=html&sortby=symbol&match=0610006O14RIK) |  |  | **1.25** |
| 1448213_AT | annexin A1 | [ANXA1](http://www.gene.ucl.ac.uk/cgi-bin/nomenclature/searchgenes.pl?field=symbol&anchor=equals&symbol_search=Search&number=100&format=html&sortby=symbol&match=ANXA1) |  | [151690](http://www.ncbi.nlm.nih.gov/entrez/dispomim.cgi?id=151690) | **-2.48** |
| 1448214_AT | pyruvate dehydrogenase (lipoamide) beta | [PDHB](http://www.gene.ucl.ac.uk/cgi-bin/nomenclature/searchgenes.pl?field=symbol&anchor=equals&symbol_search=Search&number=100&format=html&sortby=symbol&match=PDHB) |  | [179060](http://www.ncbi.nlm.nih.gov/entrez/dispomim.cgi?id=179060) | **1.39** |
| 1448215_A_AT | dipeptidylpeptidase 3 | [DPP3](http://www.gene.ucl.ac.uk/cgi-bin/nomenclature/searchgenes.pl?field=symbol&anchor=equals&symbol_search=Search&number=100&format=html&sortby=symbol&match=DPP3) |  | [606818](http://www.ncbi.nlm.nih.gov/entrez/dispomim.cgi?id=606818) | **-1.49** |
| 1448217_A_AT | ribosomal protein L27 | [RPL27](http://www.gene.ucl.ac.uk/cgi-bin/nomenclature/searchgenes.pl?field=symbol&anchor=equals&symbol_search=Search&number=100&format=html&sortby=symbol&match=RPL27) |  | [607526](http://www.ncbi.nlm.nih.gov/entrez/dispomim.cgi?id=607526) | **-1.4** |
| 1448218_S_AT | tyrosine 3-monooxygenase/tryptophan 5-monooxygenase activation protein, zeta polypeptide | [YWHAZ](http://www.gene.ucl.ac.uk/cgi-bin/nomenclature/searchgenes.pl?field=symbol&anchor=equals&symbol_search=Search&number=100&format=html&sortby=symbol&match=YWHAZ) |  | [601288](http://www.ncbi.nlm.nih.gov/entrez/dispomim.cgi?id=601288) | **-1.28** |
| 1448219_A_AT | tyrosine 3-monooxygenase/tryptophan 5-monooxygenase activation protein, zeta polypeptide | [YWHAZ](http://www.gene.ucl.ac.uk/cgi-bin/nomenclature/searchgenes.pl?field=symbol&anchor=equals&symbol_search=Search&number=100&format=html&sortby=symbol&match=YWHAZ) |  | [601288](http://www.ncbi.nlm.nih.gov/entrez/dispomim.cgi?id=601288) | **-1.37** |
| 1448226_AT | ribonucleotide reductase M2 | [RRM2](http://www.gene.ucl.ac.uk/cgi-bin/nomenclature/searchgenes.pl?field=symbol&anchor=equals&symbol_search=Search&number=100&format=html&sortby=symbol&match=RRM2) |  | [180390](http://www.ncbi.nlm.nih.gov/entrez/dispomim.cgi?id=180390) | **-3.32** |
| 1448228_AT | lysyl oxidase | [LOX](http://www.gene.ucl.ac.uk/cgi-bin/nomenclature/searchgenes.pl?field=symbol&anchor=equals&symbol_search=Search&number=100&format=html&sortby=symbol&match=LOX) |  | [153455](http://www.ncbi.nlm.nih.gov/entrez/dispomim.cgi?id=153455) | **-2.5** |
| 1448232_X_AT | tubulin, alpha 6 | [TUBA6](http://www.gene.ucl.ac.uk/cgi-bin/nomenclature/searchgenes.pl?field=symbol&anchor=equals&symbol_search=Search&number=100&format=html&sortby=symbol&match=TUBA6) |  |  | **-1.61** |
| 1448237_X_AT | lactate dehydrogenase 2, B chain | [LDH2](http://www.gene.ucl.ac.uk/cgi-bin/nomenclature/searchgenes.pl?field=symbol&anchor=equals&symbol_search=Search&number=100&format=html&sortby=symbol&match=LDH2) |  |  | **1.55** |
| 1448241_AT | GM2 ganglioside activator protein | [GM2A](http://www.gene.ucl.ac.uk/cgi-bin/nomenclature/searchgenes.pl?field=symbol&anchor=equals&symbol_search=Search&number=100&format=html&sortby=symbol&match=GM2A) |  | [272750](http://www.ncbi.nlm.nih.gov/entrez/dispomim.cgi?id=272750) | **-1.85** |
| 1448242_AT | Sec61 alpha 1 subunit (S. cerevisiae) | [SEC61A1](http://www.gene.ucl.ac.uk/cgi-bin/nomenclature/searchgenes.pl?field=symbol&anchor=equals&symbol_search=Search&number=100&format=html&sortby=symbol&match=SEC61A1) |  |  | **-1.34** |
| 1448244_AT | lysophospholipase 1 | [LYPLA1](http://www.gene.ucl.ac.uk/cgi-bin/nomenclature/searchgenes.pl?field=symbol&anchor=equals&symbol_search=Search&number=100&format=html&sortby=symbol&match=LYPLA1) |  | [605599](http://www.ncbi.nlm.nih.gov/entrez/dispomim.cgi?id=605599) | **1.26** |
| 1448245_AT | laminin receptor 1 (ribosomal protein SA) | [LAMR1](http://www.gene.ucl.ac.uk/cgi-bin/nomenclature/searchgenes.pl?field=symbol&anchor=equals&symbol_search=Search&number=100&format=html&sortby=symbol&match=LAMR1) |  | [150370](http://www.ncbi.nlm.nih.gov/entrez/dispomim.cgi?id=150370) | **-1.37** |
| 1448252_A_AT | eukaryotic translation elongation factor 1 beta 2 | [EEF1B2](http://www.gene.ucl.ac.uk/cgi-bin/nomenclature/searchgenes.pl?field=symbol&anchor=equals&symbol_search=Search&number=100&format=html&sortby=symbol&match=EEF1B2) |  | [600655](http://www.ncbi.nlm.nih.gov/entrez/dispomim.cgi?id=600655) | **-1.32** |
| 1448258_A_AT | RIKEN cDNA 1810004F21 gene | [1810004F21RIK](http://www.gene.ucl.ac.uk/cgi-bin/nomenclature/searchgenes.pl?field=symbol&anchor=equals&symbol_search=Search&number=100&format=html&sortby=symbol&match=1810004F21RIK) |  |  | **-1.37** |
| 1448259_AT | follistatin-like 1 | [FSTL1](http://www.gene.ucl.ac.uk/cgi-bin/nomenclature/searchgenes.pl?field=symbol&anchor=equals&symbol_search=Search&number=100&format=html&sortby=symbol&match=FSTL1) |  | [605547](http://www.ncbi.nlm.nih.gov/entrez/dispomim.cgi?id=605547) | **-1.81** |
| 1448260_AT | ubiquitin carboxy-terminal hydrolase L1 | [UCHL1](http://www.gene.ucl.ac.uk/cgi-bin/nomenclature/searchgenes.pl?field=symbol&anchor=equals&symbol_search=Search&number=100&format=html&sortby=symbol&match=UCHL1) |  | [191342](http://www.ncbi.nlm.nih.gov/entrez/dispomim.cgi?id=191342) | **-2.25** |
| 1448263_A_AT | CNDP dipeptidase 2 (metallopeptidase M20 family) | [CNDP2](http://www.gene.ucl.ac.uk/cgi-bin/nomenclature/searchgenes.pl?field=symbol&anchor=equals&symbol_search=Search&number=100&format=html&sortby=symbol&match=CNDP2) |  | [169800](http://www.ncbi.nlm.nih.gov/entrez/dispomim.cgi?id=169800) | **-1.88** |
| 1448264_A_AT | eukaryotic translation initiation factor 3, subunit 2 (beta) | [EIF3S2](http://www.gene.ucl.ac.uk/cgi-bin/nomenclature/searchgenes.pl?field=symbol&anchor=equals&symbol_search=Search&number=100&format=html&sortby=symbol&match=EIF3S2) |  | [603911](http://www.ncbi.nlm.nih.gov/entrez/dispomim.cgi?id=603911) | **-1.42** |
| 1448269_A_AT | kelch-like 13 (Drosophila) | [KLHL13](http://www.gene.ucl.ac.uk/cgi-bin/nomenclature/searchgenes.pl?field=symbol&anchor=equals&symbol_search=Search&number=100&format=html&sortby=symbol&match=KLHL13) |  |  | **-1.63** |
| 1448283_A_AT | ubiquitin-like 1 (sentrin) activating enzyme E1B | [UBLE1B](http://www.gene.ucl.ac.uk/cgi-bin/nomenclature/searchgenes.pl?field=symbol&anchor=equals&symbol_search=Search&number=100&format=html&sortby=symbol&match=UBLE1B) |  |  | **-1.21** |
| 1448284_A_AT | NADH dehydrogenase (ubiquinone) 1, subcomplex unknown, 1 | [NDUFC1](http://www.gene.ucl.ac.uk/cgi-bin/nomenclature/searchgenes.pl?field=symbol&anchor=equals&symbol_search=Search&number=100&format=html&sortby=symbol&match=NDUFC1) |  | [603844](http://www.ncbi.nlm.nih.gov/entrez/dispomim.cgi?id=603844) | **1.4** |
| 1448286_AT | hydroxyacyl-Coenzyme A dehydrogenase type II | [HADH2](http://www.gene.ucl.ac.uk/cgi-bin/nomenclature/searchgenes.pl?field=symbol&anchor=equals&symbol_search=Search&number=100&format=html&sortby=symbol&match=HADH2) |  | [300256](http://www.ncbi.nlm.nih.gov/entrez/dispomim.cgi?id=300256) | **1.29** |
| 1448300_AT | microsomal glutathione S-transferase 3 | [MGST3](http://www.gene.ucl.ac.uk/cgi-bin/nomenclature/searchgenes.pl?field=symbol&anchor=equals&symbol_search=Search&number=100&format=html&sortby=symbol&match=MGST3) |  | [604564](http://www.ncbi.nlm.nih.gov/entrez/dispomim.cgi?id=604564) | **1.38** |
| 1448301_S_AT | serine (or cysteine) proteinase inhibitor, clade B, member 1a | [SERPINB1A](http://www.gene.ucl.ac.uk/cgi-bin/nomenclature/searchgenes.pl?field=symbol&anchor=equals&symbol_search=Search&number=100&format=html&sortby=symbol&match=SERPINB1A) |  |  | **-2.25** |
| 1448303_AT | glycoprotein (transmembrane) nmb | [GPNMB](http://www.gene.ucl.ac.uk/cgi-bin/nomenclature/searchgenes.pl?field=symbol&anchor=equals&symbol_search=Search&number=100&format=html&sortby=symbol&match=GPNMB) |  | [604368](http://www.ncbi.nlm.nih.gov/entrez/dispomim.cgi?id=604368) | **-10.29** |
| 1448316_AT | chemokine-like factor super family 3 | [CKLFSF3](http://www.gene.ucl.ac.uk/cgi-bin/nomenclature/searchgenes.pl?field=symbol&anchor=equals&symbol_search=Search&number=100&format=html&sortby=symbol&match=CKLFSF3) |  | [607886](http://www.ncbi.nlm.nih.gov/entrez/dispomim.cgi?id=607886) | **-1.56** |
| 1448322_A_AT | cytochrome c oxidase subunit IV isoform 1 | [COX4I1](http://www.gene.ucl.ac.uk/cgi-bin/nomenclature/searchgenes.pl?field=symbol&anchor=equals&symbol_search=Search&number=100&format=html&sortby=symbol&match=COX4I1) |  | [123864](http://www.ncbi.nlm.nih.gov/entrez/dispomim.cgi?id=123864) | **1.23** |
| 1448323_A_AT | biglycan | [BGN](http://www.gene.ucl.ac.uk/cgi-bin/nomenclature/searchgenes.pl?field=symbol&anchor=equals&symbol_search=Search&number=100&format=html&sortby=symbol&match=BGN) |  | [301870](http://www.ncbi.nlm.nih.gov/entrez/dispomim.cgi?id=301870) | **-2.74** |
| 1448328_AT | SH3-domain binding protein 2 | [SH3BP2](http://www.gene.ucl.ac.uk/cgi-bin/nomenclature/searchgenes.pl?field=symbol&anchor=equals&symbol_search=Search&number=100&format=html&sortby=symbol&match=SH3BP2) |  | [602104](http://www.ncbi.nlm.nih.gov/entrez/dispomim.cgi?id=602104) | **-2.48** |
| 1448330_AT | glutathione S-transferase, mu 1 | [GSTM1](http://www.gene.ucl.ac.uk/cgi-bin/nomenclature/searchgenes.pl?field=symbol&anchor=equals&symbol_search=Search&number=100&format=html&sortby=symbol&match=GSTM1) |  |  | **1.83** |
| 1448332_AT | peroxisome biogenesis factor 19 | [PEX19](http://www.gene.ucl.ac.uk/cgi-bin/nomenclature/searchgenes.pl?field=symbol&anchor=equals&symbol_search=Search&number=100&format=html&sortby=symbol&match=PEX19) |  | [600279](http://www.ncbi.nlm.nih.gov/entrez/dispomim.cgi?id=600279) | **1.38** |
| 1448337_AT | RIKEN cDNA 2410003P15 gene | [2410003P15RIK](http://www.gene.ucl.ac.uk/cgi-bin/nomenclature/searchgenes.pl?field=symbol&anchor=equals&symbol_search=Search&number=100&format=html&sortby=symbol&match=2410003P15RIK) |  |  | **1.55** |
| 1448340_AT | DNA segment, Chr 9, Wayne State University 20, expressed | [D9WSU20E](http://www.gene.ucl.ac.uk/cgi-bin/nomenclature/searchgenes.pl?field=symbol&anchor=equals&symbol_search=Search&number=100&format=html&sortby=symbol&match=D9WSU20E) |  |  | **1.49** |
| 1448344_AT | ribosomal protein S12 | [RPS12](http://www.gene.ucl.ac.uk/cgi-bin/nomenclature/searchgenes.pl?field=symbol&anchor=equals&symbol_search=Search&number=100&format=html&sortby=symbol&match=RPS12) |  | [603660](http://www.ncbi.nlm.nih.gov/entrez/dispomim.cgi?id=603660) | **-1.45** |
| 1448346_AT | cofilin 1, non-muscle | [CFL1](http://www.gene.ucl.ac.uk/cgi-bin/nomenclature/searchgenes.pl?field=symbol&anchor=equals&symbol_search=Search&number=100&format=html&sortby=symbol&match=CFL1) |  | [601442](http://www.ncbi.nlm.nih.gov/entrez/dispomim.cgi?id=601442) | **-1.85** |
| 1448347_A_AT | GPI-anchored membrane protein 1 | [GPIAP1](http://www.gene.ucl.ac.uk/cgi-bin/nomenclature/searchgenes.pl?field=symbol&anchor=equals&symbol_search=Search&number=100&format=html&sortby=symbol&match=GPIAP1) |  |  | **-1.63** |
| 1448353_X_AT | ribophorin I | [RPN1](http://www.gene.ucl.ac.uk/cgi-bin/nomenclature/searchgenes.pl?field=symbol&anchor=equals&symbol_search=Search&number=100&format=html&sortby=symbol&match=RPN1) |  | [180470](http://www.ncbi.nlm.nih.gov/entrez/dispomim.cgi?id=180470) | **-1.27** |
| 1448358_S_AT | small nuclear ribonucleoprotein polypeptide G | [SNRPG](http://www.gene.ucl.ac.uk/cgi-bin/nomenclature/searchgenes.pl?field=symbol&anchor=equals&symbol_search=Search&number=100&format=html&sortby=symbol&match=SNRPG) |  | [603542](http://www.ncbi.nlm.nih.gov/entrez/dispomim.cgi?id=603542) | **-1.23** |
| 1448361_AT | tetratricopeptide repeat domain 3 | [TTC3](http://www.gene.ucl.ac.uk/cgi-bin/nomenclature/searchgenes.pl?field=symbol&anchor=equals&symbol_search=Search&number=100&format=html&sortby=symbol&match=TTC3) |  | [602259](http://www.ncbi.nlm.nih.gov/entrez/dispomim.cgi?id=602259) | **-1.28** |
| 1448372_A_AT | transmembrane protein 4 | [TMEM4](http://www.gene.ucl.ac.uk/cgi-bin/nomenclature/searchgenes.pl?field=symbol&anchor=equals&symbol_search=Search&number=100&format=html&sortby=symbol&match=TMEM4) |  | [605861](http://www.ncbi.nlm.nih.gov/entrez/dispomim.cgi?id=605861) | **-1.22** |
| 1448373_AT | mitochondrial ribosomal protein L18 | [MRPL18](http://www.gene.ucl.ac.uk/cgi-bin/nomenclature/searchgenes.pl?field=symbol&anchor=equals&symbol_search=Search&number=100&format=html&sortby=symbol&match=MRPL18) |  |  | **1.29** |
| 1448380_AT | lectin, galactoside-binding, soluble, 3 binding protein | [LGALS3BP](http://www.gene.ucl.ac.uk/cgi-bin/nomenclature/searchgenes.pl?field=symbol&anchor=equals&symbol_search=Search&number=100&format=html&sortby=symbol&match=LGALS3BP) |  | [600626](http://www.ncbi.nlm.nih.gov/entrez/dispomim.cgi?id=600626) | **-1.39** |
| 1448382_AT | enoyl-Coenzyme A, hydratase/3-hydroxyacyl Coenzyme A dehydrogenase | [EHHADH](http://www.gene.ucl.ac.uk/cgi-bin/nomenclature/searchgenes.pl?field=symbol&anchor=equals&symbol_search=Search&number=100&format=html&sortby=symbol&match=EHHADH) |  | [607037](http://www.ncbi.nlm.nih.gov/entrez/dispomim.cgi?id=607037) | **1.93** |
| 1448383_AT | gb:NM_008608.1 /DB_XREF=gi:6678897 /GEN=Mmp14 /FEA |  |  |  | **-2.66** |
| 1448392_AT | secreted acidic cysteine rich glycoprotein | [SPARC](http://www.gene.ucl.ac.uk/cgi-bin/nomenclature/searchgenes.pl?field=symbol&anchor=equals&symbol_search=Search&number=100&format=html&sortby=symbol&match=SPARC) |  | [182120](http://www.ncbi.nlm.nih.gov/entrez/dispomim.cgi?id=182120) | **-1.86** |
| 1448395_AT | secreted frizzled-related sequence protein 1 | [SFRP1](http://www.gene.ucl.ac.uk/cgi-bin/nomenclature/searchgenes.pl?field=symbol&anchor=equals&symbol_search=Search&number=100&format=html&sortby=symbol&match=SFRP1) |  | [604156](http://www.ncbi.nlm.nih.gov/entrez/dispomim.cgi?id=604156) | **-1.81** |
| 1448400_A_AT | SWI/SNF related, matrix associated, actin dependent regulator of chromatin, subfamily d, member 2 | [SMARCD2](http://www.gene.ucl.ac.uk/cgi-bin/nomenclature/searchgenes.pl?field=symbol&anchor=equals&symbol_search=Search&number=100&format=html&sortby=symbol&match=SMARCD2) |  | [601736](http://www.ncbi.nlm.nih.gov/entrez/dispomim.cgi?id=601736) | **-1.45** |
| 1448405_A_AT | CREBBP/EP300 inhibitory protein 1 | [CRI1](http://www.gene.ucl.ac.uk/cgi-bin/nomenclature/searchgenes.pl?field=symbol&anchor=equals&symbol_search=Search&number=100&format=html&sortby=symbol&match=CRI1) |  | [605894](http://www.ncbi.nlm.nih.gov/entrez/dispomim.cgi?id=605894) | **-1.25** |
| 1448411_AT | Wolfram syndrome 1 homolog (human) | [WFS1](http://www.gene.ucl.ac.uk/cgi-bin/nomenclature/searchgenes.pl?field=symbol&anchor=equals&symbol_search=Search&number=100&format=html&sortby=symbol&match=WFS1) |  | [606201](http://www.ncbi.nlm.nih.gov/entrez/dispomim.cgi?id=606201) | **1.29** |
| 1448412_A_AT | RIKEN cDNA 0610009M14 gene | [0610009M14RIK](http://www.gene.ucl.ac.uk/cgi-bin/nomenclature/searchgenes.pl?field=symbol&anchor=equals&symbol_search=Search&number=100&format=html&sortby=symbol&match=0610009M14RIK) |  |  | **-1.44** |
| 1448416_AT | matrix gamma-carboxyglutamate (gla) protein | [MGLAP](http://www.gene.ucl.ac.uk/cgi-bin/nomenclature/searchgenes.pl?field=symbol&anchor=equals&symbol_search=Search&number=100&format=html&sortby=symbol&match=MGLAP) |  |  | **-2.49** |
| 1448421_S_AT | asporin | [ASPN](http://www.gene.ucl.ac.uk/cgi-bin/nomenclature/searchgenes.pl?field=symbol&anchor=equals&symbol_search=Search&number=100&format=html&sortby=symbol&match=ASPN) |  | [608135](http://www.ncbi.nlm.nih.gov/entrez/dispomim.cgi?id=608135) | **-2.3** |
| 1448424_AT | frizzled-related protein | [FRZB](http://www.gene.ucl.ac.uk/cgi-bin/nomenclature/searchgenes.pl?field=symbol&anchor=equals&symbol_search=Search&number=100&format=html&sortby=symbol&match=FRZB) |  | [605083](http://www.ncbi.nlm.nih.gov/entrez/dispomim.cgi?id=605083) | **2.13** |
| 1448425_AT | eukaryotic translation initiation factor 3, subunit 10 (theta) | [EIF3S10](http://www.gene.ucl.ac.uk/cgi-bin/nomenclature/searchgenes.pl?field=symbol&anchor=equals&symbol_search=Search&number=100&format=html&sortby=symbol&match=EIF3S10) |  | [602039](http://www.ncbi.nlm.nih.gov/entrez/dispomim.cgi?id=602039) | **-1.21** |
| 1448427_AT | NADH dehydrogenase (ubiquinone) 1 alpha subcomplex, 6 (B14) | [NDUFA6](http://www.gene.ucl.ac.uk/cgi-bin/nomenclature/searchgenes.pl?field=symbol&anchor=equals&symbol_search=Search&number=100&format=html&sortby=symbol&match=NDUFA6) |  | [602138](http://www.ncbi.nlm.nih.gov/entrez/dispomim.cgi?id=602138) | **1.27** |
| 1448433_A_AT | procollagen C-proteinase enhancer protein | [PCOLCE](http://www.gene.ucl.ac.uk/cgi-bin/nomenclature/searchgenes.pl?field=symbol&anchor=equals&symbol_search=Search&number=100&format=html&sortby=symbol&match=PCOLCE) |  | [600270](http://www.ncbi.nlm.nih.gov/entrez/dispomim.cgi?id=600270) | **-1.58** |
| 1448438_AT | Der1-like domain family, member 2 | [DERL2](http://www.gene.ucl.ac.uk/cgi-bin/nomenclature/searchgenes.pl?field=symbol&anchor=equals&symbol_search=Search&number=100&format=html&sortby=symbol&match=DERL2) |  |  | **-1.27** |
| 1448441_AT | CDC28 protein kinase 1b | [CKS1B](http://www.gene.ucl.ac.uk/cgi-bin/nomenclature/searchgenes.pl?field=symbol&anchor=equals&symbol_search=Search&number=100&format=html&sortby=symbol&match=CKS1B) |  | [116900](http://www.ncbi.nlm.nih.gov/entrez/dispomim.cgi?id=116900) | **-1.43** |
| 1448451_AT | adenylate kinase 2 | [AK2](http://www.gene.ucl.ac.uk/cgi-bin/nomenclature/searchgenes.pl?field=symbol&anchor=equals&symbol_search=Search&number=100&format=html&sortby=symbol&match=AK2) |  | [103020](http://www.ncbi.nlm.nih.gov/entrez/dispomim.cgi?id=103020) | **-1.24** |
| 1448454_AT | splicing factor, arginine/serine-rich 6 | [SFRS6](http://www.gene.ucl.ac.uk/cgi-bin/nomenclature/searchgenes.pl?field=symbol&anchor=equals&symbol_search=Search&number=100&format=html&sortby=symbol&match=SFRS6) |  | [601944](http://www.ncbi.nlm.nih.gov/entrez/dispomim.cgi?id=601944) | **-1.34** |
| 1448462_AT | thymine DNA glycosylase | [TDG](http://www.gene.ucl.ac.uk/cgi-bin/nomenclature/searchgenes.pl?field=symbol&anchor=equals&symbol_search=Search&number=100&format=html&sortby=symbol&match=TDG) |  | [601423](http://www.ncbi.nlm.nih.gov/entrez/dispomim.cgi?id=601423) | **-1.44** |
| 1448475_AT | olfactomedin-like 3 | [OLFML3](http://www.gene.ucl.ac.uk/cgi-bin/nomenclature/searchgenes.pl?field=symbol&anchor=equals&symbol_search=Search&number=100&format=html&sortby=symbol&match=OLFML3) |  |  | **-1.85** |
| 1448476_AT | nucleosome assembly protein 1-like 4 | [NAP1L4](http://www.gene.ucl.ac.uk/cgi-bin/nomenclature/searchgenes.pl?field=symbol&anchor=equals&symbol_search=Search&number=100&format=html&sortby=symbol&match=NAP1L4) |  | [601651](http://www.ncbi.nlm.nih.gov/entrez/dispomim.cgi?id=601651) | **1.23** |
| 1448480_AT | RIKEN cDNA 1110017C15 gene | [1110017C15RIK](http://www.gene.ucl.ac.uk/cgi-bin/nomenclature/searchgenes.pl?field=symbol&anchor=equals&symbol_search=Search&number=100&format=html&sortby=symbol&match=1110017C15RIK) |  |  | **-1.55** |
| 1448483_A_AT | NADH dehydrogenase (ubiquinone) 1 beta subcomplex, 2 | [NDUFB2](http://www.gene.ucl.ac.uk/cgi-bin/nomenclature/searchgenes.pl?field=symbol&anchor=equals&symbol_search=Search&number=100&format=html&sortby=symbol&match=NDUFB2) |  | [603838](http://www.ncbi.nlm.nih.gov/entrez/dispomim.cgi?id=603838) | **1.33** |
| 1448484_AT | S-adenosylmethionine decarboxylase 1 | [AMD1](http://www.gene.ucl.ac.uk/cgi-bin/nomenclature/searchgenes.pl?field=symbol&anchor=equals&symbol_search=Search&number=100&format=html&sortby=symbol&match=AMD1) |  | [180980](http://www.ncbi.nlm.nih.gov/entrez/dispomim.cgi?id=180980) | **1.5** |
| 1448486_AT | methylmalonyl-Coenzyme A mutase | [MUT](http://www.gene.ucl.ac.uk/cgi-bin/nomenclature/searchgenes.pl?field=symbol&anchor=equals&symbol_search=Search&number=100&format=html&sortby=symbol&match=MUT) |  | 609058 251000 | **1.5** |
| 1448488_AT | mitochondrial ribosomal protein S5 | [MRPS5](http://www.gene.ucl.ac.uk/cgi-bin/nomenclature/searchgenes.pl?field=symbol&anchor=equals&symbol_search=Search&number=100&format=html&sortby=symbol&match=MRPS5) |  |  | **1.27** |
| 1448491_AT | enoyl coenzyme A hydratase 1, peroxisomal | [ECH1](http://www.gene.ucl.ac.uk/cgi-bin/nomenclature/searchgenes.pl?field=symbol&anchor=equals&symbol_search=Search&number=100&format=html&sortby=symbol&match=ECH1) |  | [600696](http://www.ncbi.nlm.nih.gov/entrez/dispomim.cgi?id=600696) | **1.95** |
| 1448499_A_AT | epoxide hydrolase 2, cytoplasmic | [EPHX2](http://www.gene.ucl.ac.uk/cgi-bin/nomenclature/searchgenes.pl?field=symbol&anchor=equals&symbol_search=Search&number=100&format=html&sortby=symbol&match=EPHX2) |  | [132811](http://www.ncbi.nlm.nih.gov/entrez/dispomim.cgi?id=132811) | **3.18** |
| 1448502_AT | solute carrier family 16 (monocarboxylic acid transporters), member 7 | [SLC16A7](http://www.gene.ucl.ac.uk/cgi-bin/nomenclature/searchgenes.pl?field=symbol&anchor=equals&symbol_search=Search&number=100&format=html&sortby=symbol&match=SLC16A7) |  | [603654](http://www.ncbi.nlm.nih.gov/entrez/dispomim.cgi?id=603654) | **12.43** |
| 1448504_A_AT | gb:BC018354.1 /DB_XREF=gi:17390827 /FEA=FLmRNA /CN |  |  |  | **-1.23** |
| 1448505_AT | nuclear DNA binding protein | [C1D](http://www.gene.ucl.ac.uk/cgi-bin/nomenclature/searchgenes.pl?field=symbol&anchor=equals&symbol_search=Search&number=100&format=html&sortby=symbol&match=C1D) |  | [606997](http://www.ncbi.nlm.nih.gov/entrez/dispomim.cgi?id=606997) | **-1.27** |
| 1448510_AT | ephrin A1 | [EFNA1](http://www.gene.ucl.ac.uk/cgi-bin/nomenclature/searchgenes.pl?field=symbol&anchor=equals&symbol_search=Search&number=100&format=html&sortby=symbol&match=EFNA1) |  | [191164](http://www.ncbi.nlm.nih.gov/entrez/dispomim.cgi?id=191164) | **1.75** |
| 1448513_A_AT | Niemann Pick type C2 | [NPC2](http://www.gene.ucl.ac.uk/cgi-bin/nomenclature/searchgenes.pl?field=symbol&anchor=equals&symbol_search=Search&number=100&format=html&sortby=symbol&match=NPC2) |  | [601015](http://www.ncbi.nlm.nih.gov/entrez/dispomim.cgi?id=601015) | **-1.52** |
| 1448530_AT | guanosine monophosphate reductase | [GMPR](http://www.gene.ucl.ac.uk/cgi-bin/nomenclature/searchgenes.pl?field=symbol&anchor=equals&symbol_search=Search&number=100&format=html&sortby=symbol&match=GMPR) |  | [139265](http://www.ncbi.nlm.nih.gov/entrez/dispomim.cgi?id=139265) | **1.5** |
| 1448533_AT | cytoskeleton-associated protein 1 | [CKAP1](http://www.gene.ucl.ac.uk/cgi-bin/nomenclature/searchgenes.pl?field=symbol&anchor=equals&symbol_search=Search&number=100&format=html&sortby=symbol&match=CKAP1) |  | [601303](http://www.ncbi.nlm.nih.gov/entrez/dispomim.cgi?id=601303) | **-1.37** |
| 1448540_A_AT | RIKEN cDNA 0610012G03 gene | [0610012G03RIK](http://www.gene.ucl.ac.uk/cgi-bin/nomenclature/searchgenes.pl?field=symbol&anchor=equals&symbol_search=Search&number=100&format=html&sortby=symbol&match=0610012G03RIK) |  |  | **1.35** |
| 1448544_AT | carnitine acetyltransferase | [CRAT](http://www.gene.ucl.ac.uk/cgi-bin/nomenclature/searchgenes.pl?field=symbol&anchor=equals&symbol_search=Search&number=100&format=html&sortby=symbol&match=CRAT) |  | [600184](http://www.ncbi.nlm.nih.gov/entrez/dispomim.cgi?id=600184) | **1.46** |
| 1448555_AT | DNA segment, Chr 15, ERATO Doi 682, expressed | [D15ERTD682E](http://www.gene.ucl.ac.uk/cgi-bin/nomenclature/searchgenes.pl?field=symbol&anchor=equals&symbol_search=Search&number=100&format=html&sortby=symbol&match=D15ERTD682E) |  |  | **1.52** |
| 1448558_A_AT | phospholipase A2, group IVA (cytosolic, calcium-dependent) | [PLA2G4A](http://www.gene.ucl.ac.uk/cgi-bin/nomenclature/searchgenes.pl?field=symbol&anchor=equals&symbol_search=Search&number=100&format=html&sortby=symbol&match=PLA2G4A) |  | [600522](http://www.ncbi.nlm.nih.gov/entrez/dispomim.cgi?id=600522) | **-1.72** |
| 1448561_AT | neutrophil cytosolic factor 2 | [NCF2](http://www.gene.ucl.ac.uk/cgi-bin/nomenclature/searchgenes.pl?field=symbol&anchor=equals&symbol_search=Search&number=100&format=html&sortby=symbol&match=NCF2) |  | [233710](http://www.ncbi.nlm.nih.gov/entrez/dispomim.cgi?id=233710) | **-1.81** |
| 1448563_AT | prohibitin | [PHB](http://www.gene.ucl.ac.uk/cgi-bin/nomenclature/searchgenes.pl?field=symbol&anchor=equals&symbol_search=Search&number=100&format=html&sortby=symbol&match=PHB) |  | [176705](http://www.ncbi.nlm.nih.gov/entrez/dispomim.cgi?id=176705) | **1.29** |
| 1448566_AT | solute carrier family 40 (iron-regulated transporter), member 1 | [SLC40A1](http://www.gene.ucl.ac.uk/cgi-bin/nomenclature/searchgenes.pl?field=symbol&anchor=equals&symbol_search=Search&number=100&format=html&sortby=symbol&match=SLC40A1) |  | [604653](http://www.ncbi.nlm.nih.gov/entrez/dispomim.cgi?id=604653) | **1.38** |
| 1448569_AT | CD8 antigen, beta chain 1 | [CD8B1](http://www.gene.ucl.ac.uk/cgi-bin/nomenclature/searchgenes.pl?field=symbol&anchor=equals&symbol_search=Search&number=100&format=html&sortby=symbol&match=CD8B1) |  | [186730](http://www.ncbi.nlm.nih.gov/entrez/dispomim.cgi?id=186730) | **-1.59** |
| 1448575_AT | interleukin 7 receptor | [IL7R](http://www.gene.ucl.ac.uk/cgi-bin/nomenclature/searchgenes.pl?field=symbol&anchor=equals&symbol_search=Search&number=100&format=html&sortby=symbol&match=IL7R) |  | [146661](http://www.ncbi.nlm.nih.gov/entrez/dispomim.cgi?id=146661) | **-2.2** |
| 1448576_AT | interleukin 7 receptor | [IL7R](http://www.gene.ucl.ac.uk/cgi-bin/nomenclature/searchgenes.pl?field=symbol&anchor=equals&symbol_search=Search&number=100&format=html&sortby=symbol&match=IL7R) |  | [146661](http://www.ncbi.nlm.nih.gov/entrez/dispomim.cgi?id=146661) | **-6.88** |
| 1448579_AT | golgi apparatus protein 1 | [GLG1](http://www.gene.ucl.ac.uk/cgi-bin/nomenclature/searchgenes.pl?field=symbol&anchor=equals&symbol_search=Search&number=100&format=html&sortby=symbol&match=GLG1) |  | [600753](http://www.ncbi.nlm.nih.gov/entrez/dispomim.cgi?id=600753) | **-1.25** |
| 1448580_AT | Golgi apparatus protein 1 | [GLG1](http://www.gene.ucl.ac.uk/cgi-bin/nomenclature/searchgenes.pl?field=symbol&anchor=equals&symbol_search=Search&number=100&format=html&sortby=symbol&match=GLG1) |  | [600753](http://www.ncbi.nlm.nih.gov/entrez/dispomim.cgi?id=600753) | **-1.24** |
| 1448589_AT | NADH dehydrogenase (ubiquinone) 1 beta subcomplex, 5 | [NDUFB5](http://www.gene.ucl.ac.uk/cgi-bin/nomenclature/searchgenes.pl?field=symbol&anchor=equals&symbol_search=Search&number=100&format=html&sortby=symbol&match=NDUFB5) |  | [603841](http://www.ncbi.nlm.nih.gov/entrez/dispomim.cgi?id=603841) | **1.32** |
| 1448590_AT | procollagen, type VI, alpha 1 | [COL6A1](http://www.gene.ucl.ac.uk/cgi-bin/nomenclature/searchgenes.pl?field=symbol&anchor=equals&symbol_search=Search&number=100&format=html&sortby=symbol&match=COL6A1) |  | [120220](http://www.ncbi.nlm.nih.gov/entrez/dispomim.cgi?id=120220) | **-1.7** |
| 1448591_AT | cathepsin S | [CTSS](http://www.gene.ucl.ac.uk/cgi-bin/nomenclature/searchgenes.pl?field=symbol&anchor=equals&symbol_search=Search&number=100&format=html&sortby=symbol&match=CTSS) |  | [116845](http://www.ncbi.nlm.nih.gov/entrez/dispomim.cgi?id=116845) | **-6.59** |
| 1448592_AT | cartilage associated protein | [CRTAP](http://www.gene.ucl.ac.uk/cgi-bin/nomenclature/searchgenes.pl?field=symbol&anchor=equals&symbol_search=Search&number=100&format=html&sortby=symbol&match=CRTAP) |  | [605497](http://www.ncbi.nlm.nih.gov/entrez/dispomim.cgi?id=605497) | **-1.4** |
| 1448593_AT | WNT1 inducible signaling pathway protein 1 | [WISP1](http://www.gene.ucl.ac.uk/cgi-bin/nomenclature/searchgenes.pl?field=symbol&anchor=equals&symbol_search=Search&number=100&format=html&sortby=symbol&match=WISP1) |  | [603398](http://www.ncbi.nlm.nih.gov/entrez/dispomim.cgi?id=603398) | **-4.83** |
| 1448594_AT | WNT1 inducible signaling pathway protein 1 | [WISP1](http://www.gene.ucl.ac.uk/cgi-bin/nomenclature/searchgenes.pl?field=symbol&anchor=equals&symbol_search=Search&number=100&format=html&sortby=symbol&match=WISP1) |  | [603398](http://www.ncbi.nlm.nih.gov/entrez/dispomim.cgi?id=603398) | **-27.42** |
| 1448595_A_AT | reduced expression 3 | [REX3](http://www.gene.ucl.ac.uk/cgi-bin/nomenclature/searchgenes.pl?field=symbol&anchor=equals&symbol_search=Search&number=100&format=html&sortby=symbol&match=REX3) |  |  | **-9.12** |
| 1448602_AT | muscle glycogen phosphorylase | [PYGM](http://www.gene.ucl.ac.uk/cgi-bin/nomenclature/searchgenes.pl?field=symbol&anchor=equals&symbol_search=Search&number=100&format=html&sortby=symbol&match=PYGM) |  | [608455](http://www.ncbi.nlm.nih.gov/entrez/dispomim.cgi?id=608455) | **1.4** |
| 1448604_AT | uridine monophosphate kinase | [UMPK](http://www.gene.ucl.ac.uk/cgi-bin/nomenclature/searchgenes.pl?field=symbol&anchor=equals&symbol_search=Search&number=100&format=html&sortby=symbol&match=UMPK) |  |  | **-1.66** |
| 1448605_AT | ras homolog gene family, member C | [PPP2CZ](http://www.gene.ucl.ac.uk/cgi-bin/nomenclature/searchgenes.pl?field=symbol&anchor=equals&symbol_search=Search&number=100&format=html&sortby=symbol&match=PPP2CZ) |  | [165380](http://www.ncbi.nlm.nih.gov/entrez/dispomim.cgi?id=165380) | **-1.68** |
| 1448606_AT | endothelial differentiation, lysophosphatidic acid G-protein-coupled receptor, 2 | [EDG2](http://www.gene.ucl.ac.uk/cgi-bin/nomenclature/searchgenes.pl?field=symbol&anchor=equals&symbol_search=Search&number=100&format=html&sortby=symbol&match=EDG2) |  | [602282](http://www.ncbi.nlm.nih.gov/entrez/dispomim.cgi?id=602282) | **-1.36** |
| 1448607_AT | pre-B-cell colony-enhancing factor 1 | [PBEF1](http://www.gene.ucl.ac.uk/cgi-bin/nomenclature/searchgenes.pl?field=symbol&anchor=equals&symbol_search=Search&number=100&format=html&sortby=symbol&match=PBEF1) |  | [608764](http://www.ncbi.nlm.nih.gov/entrez/dispomim.cgi?id=608764) | **1.84** |
| 1448609_AT | thiosulfate sulfurtransferase, mitochondrial | [TST](http://www.gene.ucl.ac.uk/cgi-bin/nomenclature/searchgenes.pl?field=symbol&anchor=equals&symbol_search=Search&number=100&format=html&sortby=symbol&match=TST) |  | [180370](http://www.ncbi.nlm.nih.gov/entrez/dispomim.cgi?id=180370) | **2.23** |
| 1448610_A_AT | superoxide dismutase 2, mitochondrial | [SOD2](http://www.gene.ucl.ac.uk/cgi-bin/nomenclature/searchgenes.pl?field=symbol&anchor=equals&symbol_search=Search&number=100&format=html&sortby=symbol&match=SOD2) |  | [147460](http://www.ncbi.nlm.nih.gov/entrez/dispomim.cgi?id=147460) | **1.34** |
| 1448617_AT | CD53 antigen | [CD53](http://www.gene.ucl.ac.uk/cgi-bin/nomenclature/searchgenes.pl?field=symbol&anchor=equals&symbol_search=Search&number=100&format=html&sortby=symbol&match=CD53) |  | [151525](http://www.ncbi.nlm.nih.gov/entrez/dispomim.cgi?id=151525) | **-3.71** |
| 1448620_AT | Fc receptor, IgG, low affinity III | [FCGR3](http://www.gene.ucl.ac.uk/cgi-bin/nomenclature/searchgenes.pl?field=symbol&anchor=equals&symbol_search=Search&number=100&format=html&sortby=symbol&match=FCGR3) |  |  | **-3.04** |
| 1448630_A_AT | succinate dehydrogenase complex, subunit C, integral membrane protein | [SDHC](http://www.gene.ucl.ac.uk/cgi-bin/nomenclature/searchgenes.pl?field=symbol&anchor=equals&symbol_search=Search&number=100&format=html&sortby=symbol&match=SDHC) |  | [602413](http://www.ncbi.nlm.nih.gov/entrez/dispomim.cgi?id=602413) | **1.52** |
| 1448632_AT | proteasome (prosome, macropain) subunit, beta type 10 | [PSMB10](http://www.gene.ucl.ac.uk/cgi-bin/nomenclature/searchgenes.pl?field=symbol&anchor=equals&symbol_search=Search&number=100&format=html&sortby=symbol&match=PSMB10) |  | [176847](http://www.ncbi.nlm.nih.gov/entrez/dispomim.cgi?id=176847) | **-1.35** |
| 1448635_AT | SMC2 structural maintenance of chromosomes 2-like 1 (yeast) | [SMC2L1](http://www.gene.ucl.ac.uk/cgi-bin/nomenclature/searchgenes.pl?field=symbol&anchor=equals&symbol_search=Search&number=100&format=html&sortby=symbol&match=SMC2L1) |  | [605576](http://www.ncbi.nlm.nih.gov/entrez/dispomim.cgi?id=605576) | **-1.88** |
| 1448637_AT | RIKEN cDNA 2610034E13 gene | [MED25](http://www.gene.ucl.ac.uk/cgi-bin/nomenclature/searchgenes.pl?field=symbol&anchor=equals&symbol_search=Search&number=100&format=html&sortby=symbol&match=MED25) |  |  | **1.28** |
| 1448642_AT | poly(rC) binding protein 1 | [PCBP1](http://www.gene.ucl.ac.uk/cgi-bin/nomenclature/searchgenes.pl?field=symbol&anchor=equals&symbol_search=Search&number=100&format=html&sortby=symbol&match=PCBP1) |  | [601209](http://www.ncbi.nlm.nih.gov/entrez/dispomim.cgi?id=601209) | **-1.21** |
| 1448647_AT | mannosidase 2, alpha 1 | [MAN2A1](http://www.gene.ucl.ac.uk/cgi-bin/nomenclature/searchgenes.pl?field=symbol&anchor=equals&symbol_search=Search&number=100&format=html&sortby=symbol&match=MAN2A1) |  | [154582](http://www.ncbi.nlm.nih.gov/entrez/dispomim.cgi?id=154582) | **-1.35** |
| 1448648_AT | RIKEN cDNA 9130005N14 gene | [9130005N14RIK](http://www.gene.ucl.ac.uk/cgi-bin/nomenclature/searchgenes.pl?field=symbol&anchor=equals&symbol_search=Search&number=100&format=html&sortby=symbol&match=9130005N14RIK) |  |  | **-1.44** |
| 1448651_AT | nudix (nucleoside diphosphate linked moiety X)-type motif 5 | [NUDT5](http://www.gene.ucl.ac.uk/cgi-bin/nomenclature/searchgenes.pl?field=symbol&anchor=equals&symbol_search=Search&number=100&format=html&sortby=symbol&match=NUDT5) |  |  | **-1.69** |
| 1448655_AT | low density lipoprotein receptor-related protein 1 | [LRP1](http://www.gene.ucl.ac.uk/cgi-bin/nomenclature/searchgenes.pl?field=symbol&anchor=equals&symbol_search=Search&number=100&format=html&sortby=symbol&match=LRP1) |  | [107770](http://www.ncbi.nlm.nih.gov/entrez/dispomim.cgi?id=107770) | **-1.48** |
| 1448657_A_AT | DnaJ (Hsp40) homolog, subfamily B, member 10 | [DNAJB10](http://www.gene.ucl.ac.uk/cgi-bin/nomenclature/searchgenes.pl?field=symbol&anchor=equals&symbol_search=Search&number=100&format=html&sortby=symbol&match=DNAJB10) |  |  | **1.23** |
| 1448665_AT | dystrophin, muscular dystrophy | [DMD](http://www.gene.ucl.ac.uk/cgi-bin/nomenclature/searchgenes.pl?field=symbol&anchor=equals&symbol_search=Search&number=100&format=html&sortby=symbol&match=DMD) |  | [300377](http://www.ncbi.nlm.nih.gov/entrez/dispomim.cgi?id=300377) | **3.15** |
| 1448672_A_AT | zinc finger protein 289 | [ZFP289](http://www.gene.ucl.ac.uk/cgi-bin/nomenclature/searchgenes.pl?field=symbol&anchor=equals&symbol_search=Search&number=100&format=html&sortby=symbol&match=ZFP289) |  |  | **1.4** |
| 1448673_AT | poliovirus receptor-related 3 | [PVRL3](http://www.gene.ucl.ac.uk/cgi-bin/nomenclature/searchgenes.pl?field=symbol&anchor=equals&symbol_search=Search&number=100&format=html&sortby=symbol&match=PVRL3) |  | [607147](http://www.ncbi.nlm.nih.gov/entrez/dispomim.cgi?id=607147) | **-1.86** |
| 1448676_AT | calcium/calmodulin-dependent protein kinase II, beta | [CAMK2B](http://www.gene.ucl.ac.uk/cgi-bin/nomenclature/searchgenes.pl?field=symbol&anchor=equals&symbol_search=Search&number=100&format=html&sortby=symbol&match=CAMK2B) |  | [607707](http://www.ncbi.nlm.nih.gov/entrez/dispomim.cgi?id=607707) | **-2.1** |
| 1448682_AT | dynein, cytoplasmic, light chain 1 | [DNCLC1](http://www.gene.ucl.ac.uk/cgi-bin/nomenclature/searchgenes.pl?field=symbol&anchor=equals&symbol_search=Search&number=100&format=html&sortby=symbol&match=DNCLC1) |  |  | **-2.17** |
| 1448688_AT | podocalyxin-like | [PODXL](http://www.gene.ucl.ac.uk/cgi-bin/nomenclature/searchgenes.pl?field=symbol&anchor=equals&symbol_search=Search&number=100&format=html&sortby=symbol&match=PODXL) |  | [602632](http://www.ncbi.nlm.nih.gov/entrez/dispomim.cgi?id=602632) | **1.82** |
| 1448691_AT | expressed sequence AI663987 | [UBQLN4](http://www.gene.ucl.ac.uk/cgi-bin/nomenclature/searchgenes.pl?field=symbol&anchor=equals&symbol_search=Search&number=100&format=html&sortby=symbol&match=UBQLN4) |  | [605440](http://www.ncbi.nlm.nih.gov/entrez/dispomim.cgi?id=605440) | **1.22** |
| 1448692_AT | expressed sequence AI663987 | [UBQLN4](http://www.gene.ucl.ac.uk/cgi-bin/nomenclature/searchgenes.pl?field=symbol&anchor=equals&symbol_search=Search&number=100&format=html&sortby=symbol&match=UBQLN4) |  | [605440](http://www.ncbi.nlm.nih.gov/entrez/dispomim.cgi?id=605440) | **1.36** |
| 1448694_AT | Jun oncogene | [JUN](http://www.gene.ucl.ac.uk/cgi-bin/nomenclature/searchgenes.pl?field=symbol&anchor=equals&symbol_search=Search&number=100&format=html&sortby=symbol&match=JUN) |  | [165160](http://www.ncbi.nlm.nih.gov/entrez/dispomim.cgi?id=165160) | **1.34** |
| 1448697_S_AT | ribosomal protein L36a-like | [RPL36AL](http://www.gene.ucl.ac.uk/cgi-bin/nomenclature/searchgenes.pl?field=symbol&anchor=equals&symbol_search=Search&number=100&format=html&sortby=symbol&match=RPL36AL) |  | [180469](http://www.ncbi.nlm.nih.gov/entrez/dispomim.cgi?id=180469) | **-1.3** |
| 1448698_AT | cyclin D1 | [CCND1](http://www.gene.ucl.ac.uk/cgi-bin/nomenclature/searchgenes.pl?field=symbol&anchor=equals&symbol_search=Search&number=100&format=html&sortby=symbol&match=CCND1) |  | [168461](http://www.ncbi.nlm.nih.gov/entrez/dispomim.cgi?id=168461) | **-1.45** |
| 1448704_S_AT | histocompatibility 47 | [H47](http://www.gene.ucl.ac.uk/cgi-bin/nomenclature/searchgenes.pl?field=symbol&anchor=equals&symbol_search=Search&number=100&format=html&sortby=symbol&match=H47) |  |  | **-1.28** |
| 1448710_AT | chemokine (C-X-C motif) receptor 4 | [CXCR4](http://www.gene.ucl.ac.uk/cgi-bin/nomenclature/searchgenes.pl?field=symbol&anchor=equals&symbol_search=Search&number=100&format=html&sortby=symbol&match=CXCR4) |  | [162643](http://www.ncbi.nlm.nih.gov/entrez/dispomim.cgi?id=162643) | **-4.15** |
| 1448717_AT | glutaryl-Coenzyme A dehydrogenase | [GCDH](http://www.gene.ucl.ac.uk/cgi-bin/nomenclature/searchgenes.pl?field=symbol&anchor=equals&symbol_search=Search&number=100&format=html&sortby=symbol&match=GCDH) |  | [608801](http://www.ncbi.nlm.nih.gov/entrez/dispomim.cgi?id=608801) | **1.62** |
| 1448725_AT | poly (ADP-ribose) glycohydrolase | [PARG](http://www.gene.ucl.ac.uk/cgi-bin/nomenclature/searchgenes.pl?field=symbol&anchor=equals&symbol_search=Search&number=100&format=html&sortby=symbol&match=PARG) |  | [603501](http://www.ncbi.nlm.nih.gov/entrez/dispomim.cgi?id=603501) | **-1.22** |
| 1448729_A_AT | septin 4 | [SEPT4](http://www.gene.ucl.ac.uk/cgi-bin/nomenclature/searchgenes.pl?field=symbol&anchor=equals&symbol_search=Search&number=100&format=html&sortby=symbol&match=SEPT4) |  |  | **1.33** |
| 1448732_AT | cathepsin B | [CTSB](http://www.gene.ucl.ac.uk/cgi-bin/nomenclature/searchgenes.pl?field=symbol&anchor=equals&symbol_search=Search&number=100&format=html&sortby=symbol&match=CTSB) |  | [116810](http://www.ncbi.nlm.nih.gov/entrez/dispomim.cgi?id=116810) | **-1.46** |
| 1448736_A_AT | hypoxanthine guanine phosphoribosyl transferase 1 | [HPRT1](http://www.gene.ucl.ac.uk/cgi-bin/nomenclature/searchgenes.pl?field=symbol&anchor=equals&symbol_search=Search&number=100&format=html&sortby=symbol&match=HPRT1) |  | [308000](http://www.ncbi.nlm.nih.gov/entrez/dispomim.cgi?id=308000) | **-1.24** |
| 1448737_AT | transmembrane 4 superfamily member 2 | [TM4SF2](http://www.gene.ucl.ac.uk/cgi-bin/nomenclature/searchgenes.pl?field=symbol&anchor=equals&symbol_search=Search&number=100&format=html&sortby=symbol&match=TM4SF2) |  | [300096](http://www.ncbi.nlm.nih.gov/entrez/dispomim.cgi?id=300096) | **1.36** |
| 1448740_AT | RAN guanine nucleotide release factor | [RANGNRF](http://www.gene.ucl.ac.uk/cgi-bin/nomenclature/searchgenes.pl?field=symbol&anchor=equals&symbol_search=Search&number=100&format=html&sortby=symbol&match=RANGNRF) |  | [607954](http://www.ncbi.nlm.nih.gov/entrez/dispomim.cgi?id=607954) | **1.21** |
| 1448743_AT | synovial sarcoma, X breakpoint 2 interacting protein | [SSX2IP](http://www.gene.ucl.ac.uk/cgi-bin/nomenclature/searchgenes.pl?field=symbol&anchor=equals&symbol_search=Search&number=100&format=html&sortby=symbol&match=SSX2IP) |  | [608690](http://www.ncbi.nlm.nih.gov/entrez/dispomim.cgi?id=608690) | **1.51** |
| 1448744_AT | gb:BC004002.1 /DB_XREF=gi:13278372 /FEA=FLmRNA /CN |  |  |  | **-1.61** |
| 1448748_AT | pleckstrin | [PLEK](http://www.gene.ucl.ac.uk/cgi-bin/nomenclature/searchgenes.pl?field=symbol&anchor=equals&symbol_search=Search&number=100&format=html&sortby=symbol&match=PLEK) |  | [173570](http://www.ncbi.nlm.nih.gov/entrez/dispomim.cgi?id=173570) | **-3.08** |
| 1448749_AT | pleckstrin | [PLEK](http://www.gene.ucl.ac.uk/cgi-bin/nomenclature/searchgenes.pl?field=symbol&anchor=equals&symbol_search=Search&number=100&format=html&sortby=symbol&match=PLEK) |  | [173570](http://www.ncbi.nlm.nih.gov/entrez/dispomim.cgi?id=173570) | **-2.37** |
| 1448752_AT | carbonic anhydrase 2 | [CAR2](http://www.gene.ucl.ac.uk/cgi-bin/nomenclature/searchgenes.pl?field=symbol&anchor=equals&symbol_search=Search&number=100&format=html&sortby=symbol&match=CAR2) |  |  | **2.09** |
| 1448754_AT | retinol binding protein 1, cellular | [RBP1](http://www.gene.ucl.ac.uk/cgi-bin/nomenclature/searchgenes.pl?field=symbol&anchor=equals&symbol_search=Search&number=100&format=html&sortby=symbol&match=RBP1) |  | [180260](http://www.ncbi.nlm.nih.gov/entrez/dispomim.cgi?id=180260) | **-1.57** |
| 1448755_AT | procollagen, type XV | [COL15A1](http://www.gene.ucl.ac.uk/cgi-bin/nomenclature/searchgenes.pl?field=symbol&anchor=equals&symbol_search=Search&number=100&format=html&sortby=symbol&match=COL15A1) |  | [120325](http://www.ncbi.nlm.nih.gov/entrez/dispomim.cgi?id=120325) | **-1.33** |
| 1448770_A_AT | ATPase inhibitory factor 1 | [ATPIF1](http://www.gene.ucl.ac.uk/cgi-bin/nomenclature/searchgenes.pl?field=symbol&anchor=equals&symbol_search=Search&number=100&format=html&sortby=symbol&match=ATPIF1) |  |  | **-1.85** |
| 1448774_AT | stomatin (Epb7.2)-like 2 | [STOML2](http://www.gene.ucl.ac.uk/cgi-bin/nomenclature/searchgenes.pl?field=symbol&anchor=equals&symbol_search=Search&number=100&format=html&sortby=symbol&match=STOML2) |  | [608292](http://www.ncbi.nlm.nih.gov/entrez/dispomim.cgi?id=608292) | **1.2** |
| 1448775_AT | interferon activated gene 203 | [IFI203](http://www.gene.ucl.ac.uk/cgi-bin/nomenclature/searchgenes.pl?field=symbol&anchor=equals&symbol_search=Search&number=100&format=html&sortby=symbol&match=IFI203) |  |  | **-1.39** |
| 1448780_AT | solute carrier family 12, member 2 | [SLC12A2](http://www.gene.ucl.ac.uk/cgi-bin/nomenclature/searchgenes.pl?field=symbol&anchor=equals&symbol_search=Search&number=100&format=html&sortby=symbol&match=SLC12A2) |  | [600840](http://www.ncbi.nlm.nih.gov/entrez/dispomim.cgi?id=600840) | **1.37** |
| 1448786_AT | RIKEN cDNA 1100001H23 gene | [1100001H23RIK](http://www.gene.ucl.ac.uk/cgi-bin/nomenclature/searchgenes.pl?field=symbol&anchor=equals&symbol_search=Search&number=100&format=html&sortby=symbol&match=1100001H23RIK) |  |  | **1.86** |
| 1448791_AT | sorting nexin 5 | [SNX5](http://www.gene.ucl.ac.uk/cgi-bin/nomenclature/searchgenes.pl?field=symbol&anchor=equals&symbol_search=Search&number=100&format=html&sortby=symbol&match=SNX5) |  | [605937](http://www.ncbi.nlm.nih.gov/entrez/dispomim.cgi?id=605937) | **-1.3** |
| 1448796_S_AT | transforming growth factor beta regulated gene 4 | [TBRG4](http://www.gene.ucl.ac.uk/cgi-bin/nomenclature/searchgenes.pl?field=symbol&anchor=equals&symbol_search=Search&number=100&format=html&sortby=symbol&match=TBRG4) |  |  | **1.34** |
| 1448800_AT | reticulon 4 interacting protein 1 | [RTN4IP1](http://www.gene.ucl.ac.uk/cgi-bin/nomenclature/searchgenes.pl?field=symbol&anchor=equals&symbol_search=Search&number=100&format=html&sortby=symbol&match=RTN4IP1) |  |  | **1.58** |
| 1448803_AT | golgi autoantigen, golgin subfamily a, 4 | [GOLGA4](http://www.gene.ucl.ac.uk/cgi-bin/nomenclature/searchgenes.pl?field=symbol&anchor=equals&symbol_search=Search&number=100&format=html&sortby=symbol&match=GOLGA4) |  | [602509](http://www.ncbi.nlm.nih.gov/entrez/dispomim.cgi?id=602509) | **1.34** |
| 1448811_AT | mitochondrial ribosomal protein L2 | [MRPL2](http://www.gene.ucl.ac.uk/cgi-bin/nomenclature/searchgenes.pl?field=symbol&anchor=equals&symbol_search=Search&number=100&format=html&sortby=symbol&match=MRPL2) |  |  | **1.3** |
| 1448814_AT | growth factor receptor bound protein 2-associated protein 1 | [GAB1](http://www.gene.ucl.ac.uk/cgi-bin/nomenclature/searchgenes.pl?field=symbol&anchor=equals&symbol_search=Search&number=100&format=html&sortby=symbol&match=GAB1) |  | [604439](http://www.ncbi.nlm.nih.gov/entrez/dispomim.cgi?id=604439) | **1.42** |
| 1448825_AT | pyruvate dehydrogenase kinase, isoenzyme 2 | [PDK2](http://www.gene.ucl.ac.uk/cgi-bin/nomenclature/searchgenes.pl?field=symbol&anchor=equals&symbol_search=Search&number=100&format=html&sortby=symbol&match=PDK2) |  | [602525](http://www.ncbi.nlm.nih.gov/entrez/dispomim.cgi?id=602525) | **1.78** |
| 1448830_AT | dual specificity phosphatase 1 | [DUSP1](http://www.gene.ucl.ac.uk/cgi-bin/nomenclature/searchgenes.pl?field=symbol&anchor=equals&symbol_search=Search&number=100&format=html&sortby=symbol&match=DUSP1) |  | [600714](http://www.ncbi.nlm.nih.gov/entrez/dispomim.cgi?id=600714) | **1.58** |
| 1448835_AT | E2F transcription factor 6 | [E2F6](http://www.gene.ucl.ac.uk/cgi-bin/nomenclature/searchgenes.pl?field=symbol&anchor=equals&symbol_search=Search&number=100&format=html&sortby=symbol&match=E2F6) |  | [602944](http://www.ncbi.nlm.nih.gov/entrez/dispomim.cgi?id=602944) | **1.54** |
| 1448839_AT | DNA segment, Chr 17, ERATO Doi 288, expressed | [D17ERTD288E](http://www.gene.ucl.ac.uk/cgi-bin/nomenclature/searchgenes.pl?field=symbol&anchor=equals&symbol_search=Search&number=100&format=html&sortby=symbol&match=D17ERTD288E) |  |  | **1.77** |
| 1448844_AT | RIKEN cDNA 1810044O22 gene | [1810044O22RIK](http://www.gene.ucl.ac.uk/cgi-bin/nomenclature/searchgenes.pl?field=symbol&anchor=equals&symbol_search=Search&number=100&format=html&sortby=symbol&match=1810044O22RIK) |  |  | **1.3** |
| 1448856_A_AT | methionine sulfoxide reductase A | [MSRA](http://www.gene.ucl.ac.uk/cgi-bin/nomenclature/searchgenes.pl?field=symbol&anchor=equals&symbol_search=Search&number=100&format=html&sortby=symbol&match=MSRA) |  | [601250](http://www.ncbi.nlm.nih.gov/entrez/dispomim.cgi?id=601250) | **1.33** |
| 1448858_AT | Unc-51 like kinase 2 (C. elegans) | [ULK2](http://www.gene.ucl.ac.uk/cgi-bin/nomenclature/searchgenes.pl?field=symbol&anchor=equals&symbol_search=Search&number=100&format=html&sortby=symbol&match=ULK2) |  | [608650](http://www.ncbi.nlm.nih.gov/entrez/dispomim.cgi?id=608650) | **1.24** |
| 1448862_AT | intercellular adhesion molecule 2 | [ICAM2](http://www.gene.ucl.ac.uk/cgi-bin/nomenclature/searchgenes.pl?field=symbol&anchor=equals&symbol_search=Search&number=100&format=html&sortby=symbol&match=ICAM2) |  | [146630](http://www.ncbi.nlm.nih.gov/entrez/dispomim.cgi?id=146630) | **1.69** |
| 1448864_AT | SNF related kinase | [SNRK](http://www.gene.ucl.ac.uk/cgi-bin/nomenclature/searchgenes.pl?field=symbol&anchor=equals&symbol_search=Search&number=100&format=html&sortby=symbol&match=SNRK) |  |  | **1.69** |
| 1448870_AT | latent transforming growth factor beta binding protein 1 | [LTBP1](http://www.gene.ucl.ac.uk/cgi-bin/nomenclature/searchgenes.pl?field=symbol&anchor=equals&symbol_search=Search&number=100&format=html&sortby=symbol&match=LTBP1) |  | [150390](http://www.ncbi.nlm.nih.gov/entrez/dispomim.cgi?id=150390) | **-1.48** |
| 1448876_AT | Ellis van Creveld gene homolog (human) | [EVC](http://www.gene.ucl.ac.uk/cgi-bin/nomenclature/searchgenes.pl?field=symbol&anchor=equals&symbol_search=Search&number=100&format=html&sortby=symbol&match=EVC) |  | [604831](http://www.ncbi.nlm.nih.gov/entrez/dispomim.cgi?id=604831) | **1.46** |
| 1448883_AT | legumain | [LGMN](http://www.gene.ucl.ac.uk/cgi-bin/nomenclature/searchgenes.pl?field=symbol&anchor=equals&symbol_search=Search&number=100&format=html&sortby=symbol&match=LGMN) |  | [602620](http://www.ncbi.nlm.nih.gov/entrez/dispomim.cgi?id=602620) | **-2.55** |
| 1448884_AT | general transcription factor II E, polypeptide 2 (beta subunit) | [GTF2E2](http://www.gene.ucl.ac.uk/cgi-bin/nomenclature/searchgenes.pl?field=symbol&anchor=equals&symbol_search=Search&number=100&format=html&sortby=symbol&match=GTF2E2) |  | [189964](http://www.ncbi.nlm.nih.gov/entrez/dispomim.cgi?id=189964) | **-1.31** |
| 1448885_AT | RAP2B, member of RAS oncogene family | [RAP2B](http://www.gene.ucl.ac.uk/cgi-bin/nomenclature/searchgenes.pl?field=symbol&anchor=equals&symbol_search=Search&number=100&format=html&sortby=symbol&match=RAP2B) |  | [179541](http://www.ncbi.nlm.nih.gov/entrez/dispomim.cgi?id=179541) | **-1.73** |
| 1448889_AT | solute carrier family 38, member 4 | [SLC38A4](http://www.gene.ucl.ac.uk/cgi-bin/nomenclature/searchgenes.pl?field=symbol&anchor=equals&symbol_search=Search&number=100&format=html&sortby=symbol&match=SLC38A4) |  | [608065](http://www.ncbi.nlm.nih.gov/entrez/dispomim.cgi?id=608065) | **-2.56** |
| 1448890_AT | Kruppel-like factor 2 (lung) | [KLF2](http://www.gene.ucl.ac.uk/cgi-bin/nomenclature/searchgenes.pl?field=symbol&anchor=equals&symbol_search=Search&number=100&format=html&sortby=symbol&match=KLF2) |  | [602016](http://www.ncbi.nlm.nih.gov/entrez/dispomim.cgi?id=602016) | **1.74** |
| 1448891_AT | macrophage scavenger receptor 2 | [MSR2](http://www.gene.ucl.ac.uk/cgi-bin/nomenclature/searchgenes.pl?field=symbol&anchor=equals&symbol_search=Search&number=100&format=html&sortby=symbol&match=MSR2) |  |  | **-1.7** |
| 1448892_AT | dedicator of cytokinesis 7 | [DOCK7](http://www.gene.ucl.ac.uk/cgi-bin/nomenclature/searchgenes.pl?field=symbol&anchor=equals&symbol_search=Search&number=100&format=html&sortby=symbol&match=DOCK7) |  |  | **-1.5** |
| 1448894_AT | aldo-keto reductase family 1, member B8 | [AKR1B8](http://www.gene.ucl.ac.uk/cgi-bin/nomenclature/searchgenes.pl?field=symbol&anchor=equals&symbol_search=Search&number=100&format=html&sortby=symbol&match=AKR1B8) |  |  | **-1.8** |
| 1448897_AT | makorin, ring finger protein, 2 | [LOC212815](http://www.gene.ucl.ac.uk/cgi-bin/nomenclature/searchgenes.pl?field=symbol&anchor=equals&symbol_search=Search&number=100&format=html&sortby=symbol&match=LOC212815) |  | [608426](http://www.ncbi.nlm.nih.gov/entrez/dispomim.cgi?id=608426) | **1.43** |
| 1448900_AT | DNA segment, Chr 16, human D22S680E, expressed | [D16H22S680E](http://www.gene.ucl.ac.uk/cgi-bin/nomenclature/searchgenes.pl?field=symbol&anchor=equals&symbol_search=Search&number=100&format=html&sortby=symbol&match=D16H22S680E) |  |  | **1.58** |
| 1448901_AT | carboxypeptidase X 1 (M14 family) | [CPXM1](http://www.gene.ucl.ac.uk/cgi-bin/nomenclature/searchgenes.pl?field=symbol&anchor=equals&symbol_search=Search&number=100&format=html&sortby=symbol&match=CPXM1) |  |  | **-2.25** |
| 1448903_AT | selenoprotein | [SEP15](http://www.gene.ucl.ac.uk/cgi-bin/nomenclature/searchgenes.pl?field=symbol&anchor=equals&symbol_search=Search&number=100&format=html&sortby=symbol&match=SEP15) |  | [606254](http://www.ncbi.nlm.nih.gov/entrez/dispomim.cgi?id=606254) | **-1.32** |
| 1448908_AT | phosphatidic acid phosphatase type 2B | [PPAP2B](http://www.gene.ucl.ac.uk/cgi-bin/nomenclature/searchgenes.pl?field=symbol&anchor=equals&symbol_search=Search&number=100&format=html&sortby=symbol&match=PPAP2B) |  | [607125](http://www.ncbi.nlm.nih.gov/entrez/dispomim.cgi?id=607125) | **1.3** |
| 1448918_AT | solute carrier organic anion transporter family, member 3a1 | [SLCO3A1](http://www.gene.ucl.ac.uk/cgi-bin/nomenclature/searchgenes.pl?field=symbol&anchor=equals&symbol_search=Search&number=100&format=html&sortby=symbol&match=SLCO3A1) |  |  | **2.34** |
| 1448919_AT | RIKEN cDNA 1110055L24 gene | [1110055L24RIK](http://www.gene.ucl.ac.uk/cgi-bin/nomenclature/searchgenes.pl?field=symbol&anchor=equals&symbol_search=Search&number=100&format=html&sortby=symbol&match=1110055L24RIK) |  |  | **-1.79** |
| 1448921_A_AT | mitochondrial ribosomal protein S9 | [MRPS9](http://www.gene.ucl.ac.uk/cgi-bin/nomenclature/searchgenes.pl?field=symbol&anchor=equals&symbol_search=Search&number=100&format=html&sortby=symbol&match=MRPS9) |  |  | **1.39** |
| 1448926_AT | homeo box A5 | [HOXA5](http://www.gene.ucl.ac.uk/cgi-bin/nomenclature/searchgenes.pl?field=symbol&anchor=equals&symbol_search=Search&number=100&format=html&sortby=symbol&match=HOXA5) |  | [142952](http://www.ncbi.nlm.nih.gov/entrez/dispomim.cgi?id=142952) | **1.4** |
| 1448934_AT | NADH dehydrogenase (ubiquinone) 1 alpha subcomplex 10 | [NDUFA10](http://www.gene.ucl.ac.uk/cgi-bin/nomenclature/searchgenes.pl?field=symbol&anchor=equals&symbol_search=Search&number=100&format=html&sortby=symbol&match=NDUFA10) |  | [603835](http://www.ncbi.nlm.nih.gov/entrez/dispomim.cgi?id=603835) | **1.37** |
| 1448943_AT | neuropilin 1 | [NRP1](http://www.gene.ucl.ac.uk/cgi-bin/nomenclature/searchgenes.pl?field=symbol&anchor=equals&symbol_search=Search&number=100&format=html&sortby=symbol&match=NRP1) |  | [602069](http://www.ncbi.nlm.nih.gov/entrez/dispomim.cgi?id=602069) | **1.34** |
| 1448944_AT | neuropilin 1 | [NRP1](http://www.gene.ucl.ac.uk/cgi-bin/nomenclature/searchgenes.pl?field=symbol&anchor=equals&symbol_search=Search&number=100&format=html&sortby=symbol&match=NRP1) |  | [602069](http://www.ncbi.nlm.nih.gov/entrez/dispomim.cgi?id=602069) | **1.46** |
| 1448949_AT | carbonic anhydrase 4 | [CAR4](http://www.gene.ucl.ac.uk/cgi-bin/nomenclature/searchgenes.pl?field=symbol&anchor=equals&symbol_search=Search&number=100&format=html&sortby=symbol&match=CAR4) |  |  | **1.74** |
| 1448956_AT | START domain containing 10 | [STARD10](http://www.gene.ucl.ac.uk/cgi-bin/nomenclature/searchgenes.pl?field=symbol&anchor=equals&symbol_search=Search&number=100&format=html&sortby=symbol&match=STARD10) |  |  | **1.48** |
| 1448959_AT | NADH dehydrogenase (ubiquinone) Fe-S protein 4 | [NDUFS4](http://www.gene.ucl.ac.uk/cgi-bin/nomenclature/searchgenes.pl?field=symbol&anchor=equals&symbol_search=Search&number=100&format=html&sortby=symbol&match=NDUFS4) |  | [602694](http://www.ncbi.nlm.nih.gov/entrez/dispomim.cgi?id=602694) | **1.22** |
| 1448962_AT | myosin heavy chain 11, smooth muscle | [MYH11](http://www.gene.ucl.ac.uk/cgi-bin/nomenclature/searchgenes.pl?field=symbol&anchor=equals&symbol_search=Search&number=100&format=html&sortby=symbol&match=MYH11) |  | [160745](http://www.ncbi.nlm.nih.gov/entrez/dispomim.cgi?id=160745) | **3.17** |
| 1448967_AT | nipsnap homolog 3A (C. elegans) | [NIPSNAP3A](http://www.gene.ucl.ac.uk/cgi-bin/nomenclature/searchgenes.pl?field=symbol&anchor=equals&symbol_search=Search&number=100&format=html&sortby=symbol&match=NIPSNAP3A) |  | 608871 608872 | **-1.37** |
| 1448968_AT | DNA segment, Chr 7, Wayne State University 128, expressed | [D7WSU128E](http://www.gene.ucl.ac.uk/cgi-bin/nomenclature/searchgenes.pl?field=symbol&anchor=equals&symbol_search=Search&number=100&format=html&sortby=symbol&match=D7WSU128E) |  |  | **-1.37** |
| 1448987_AT | acetyl-Coenzyme A dehydrogenase, long-chain | [ACADL](http://www.gene.ucl.ac.uk/cgi-bin/nomenclature/searchgenes.pl?field=symbol&anchor=equals&symbol_search=Search&number=100&format=html&sortby=symbol&match=ACADL) |  | [201460](http://www.ncbi.nlm.nih.gov/entrez/dispomim.cgi?id=201460) | **1.46** |
| 1448990_A_AT | myosin IB | [MYO1B](http://www.gene.ucl.ac.uk/cgi-bin/nomenclature/searchgenes.pl?field=symbol&anchor=equals&symbol_search=Search&number=100&format=html&sortby=symbol&match=MYO1B) |  | [606537](http://www.ncbi.nlm.nih.gov/entrez/dispomim.cgi?id=606537) | **1.41** |
| 1448997_AT | pleckstrin homology, Sec7 and coiled-coil domains 1 | [PSCD1](http://www.gene.ucl.ac.uk/cgi-bin/nomenclature/searchgenes.pl?field=symbol&anchor=equals&symbol_search=Search&number=100&format=html&sortby=symbol&match=PSCD1) |  | [182115](http://www.ncbi.nlm.nih.gov/entrez/dispomim.cgi?id=182115) | **1.84** |
| 1449000_AT | DNA segment, Chr 10, Johns Hopkins University 81 expressed | [D10JHU81E](http://www.gene.ucl.ac.uk/cgi-bin/nomenclature/searchgenes.pl?field=symbol&anchor=equals&symbol_search=Search&number=100&format=html&sortby=symbol&match=D10JHU81E) |  |  | **1.47** |
| 1449001_AT | isovaleryl coenzyme A dehydrogenase | [IVD](http://www.gene.ucl.ac.uk/cgi-bin/nomenclature/searchgenes.pl?field=symbol&anchor=equals&symbol_search=Search&number=100&format=html&sortby=symbol&match=IVD) |  | [607036](http://www.ncbi.nlm.nih.gov/entrez/dispomim.cgi?id=607036) | **1.4** |
| 1449005_AT | solute carrier family 16 (monocarboxylic acid transporters), member 3 | [SLC16A3](http://www.gene.ucl.ac.uk/cgi-bin/nomenclature/searchgenes.pl?field=symbol&anchor=equals&symbol_search=Search&number=100&format=html&sortby=symbol&match=SLC16A3) |  | [603877](http://www.ncbi.nlm.nih.gov/entrez/dispomim.cgi?id=603877) | **-1.63** |
| 1449015_AT | resistin like alpha | [RETNLA](http://www.gene.ucl.ac.uk/cgi-bin/nomenclature/searchgenes.pl?field=symbol&anchor=equals&symbol_search=Search&number=100&format=html&sortby=symbol&match=RETNLA) |  |  | **2.15** |
| 1449017_AT | nuclear transport factor 2 | [NUTF2](http://www.gene.ucl.ac.uk/cgi-bin/nomenclature/searchgenes.pl?field=symbol&anchor=equals&symbol_search=Search&number=100&format=html&sortby=symbol&match=NUTF2) |  | [605813](http://www.ncbi.nlm.nih.gov/entrez/dispomim.cgi?id=605813) | **1.24** |
| 1449018_AT | profilin 1 | [PFN1](http://www.gene.ucl.ac.uk/cgi-bin/nomenclature/searchgenes.pl?field=symbol&anchor=equals&symbol_search=Search&number=100&format=html&sortby=symbol&match=PFN1) |  | [176610](http://www.ncbi.nlm.nih.gov/entrez/dispomim.cgi?id=176610) | **-1.64** |
| 1449022_AT | nestin | [NES](http://www.gene.ucl.ac.uk/cgi-bin/nomenclature/searchgenes.pl?field=symbol&anchor=equals&symbol_search=Search&number=100&format=html&sortby=symbol&match=NES) |  | [600915](http://www.ncbi.nlm.nih.gov/entrez/dispomim.cgi?id=600915) | **-2.19** |
| 1449024_A_AT | hexosaminidase A | [HEXA](http://www.gene.ucl.ac.uk/cgi-bin/nomenclature/searchgenes.pl?field=symbol&anchor=equals&symbol_search=Search&number=100&format=html&sortby=symbol&match=HEXA) |  | [606869](http://www.ncbi.nlm.nih.gov/entrez/dispomim.cgi?id=606869) | **-1.85** |
| 1449027_AT | ras homolog gene family, member U | [RHOU](http://www.gene.ucl.ac.uk/cgi-bin/nomenclature/searchgenes.pl?field=symbol&anchor=equals&symbol_search=Search&number=100&format=html&sortby=symbol&match=RHOU) |  | [606366](http://www.ncbi.nlm.nih.gov/entrez/dispomim.cgi?id=606366) | **2.77** |
| 1449028_AT | ras homolog gene family, member U | [RHOU](http://www.gene.ucl.ac.uk/cgi-bin/nomenclature/searchgenes.pl?field=symbol&anchor=equals&symbol_search=Search&number=100&format=html&sortby=symbol&match=RHOU) |  | [606366](http://www.ncbi.nlm.nih.gov/entrez/dispomim.cgi?id=606366) | **2.36** |
| 1449037_AT | cAMP responsive element modulator | [CREM](http://www.gene.ucl.ac.uk/cgi-bin/nomenclature/searchgenes.pl?field=symbol&anchor=equals&symbol_search=Search&number=100&format=html&sortby=symbol&match=CREM) |  | [123812](http://www.ncbi.nlm.nih.gov/entrez/dispomim.cgi?id=123812) | **-1.47** |
| 1449044_AT | eukaryotic translation elongation factor 1 epsilon 1 | [EEF1E1](http://www.gene.ucl.ac.uk/cgi-bin/nomenclature/searchgenes.pl?field=symbol&anchor=equals&symbol_search=Search&number=100&format=html&sortby=symbol&match=EEF1E1) |  |  | **-1.4** |
| 1449049_AT | toll-like receptor 1 | [TLR1](http://www.gene.ucl.ac.uk/cgi-bin/nomenclature/searchgenes.pl?field=symbol&anchor=equals&symbol_search=Search&number=100&format=html&sortby=symbol&match=TLR1) |  | [601194](http://www.ncbi.nlm.nih.gov/entrez/dispomim.cgi?id=601194) | **-3.64** |
| 1449051_AT | peroxisome proliferator activated receptor alpha | [PPARA](http://www.gene.ucl.ac.uk/cgi-bin/nomenclature/searchgenes.pl?field=symbol&anchor=equals&symbol_search=Search&number=100&format=html&sortby=symbol&match=PPARA) |  | [170998](http://www.ncbi.nlm.nih.gov/entrez/dispomim.cgi?id=170998) | **1.64** |
| 1449059_A_AT | 3-oxoacid CoA transferase 1 | [OXCT1](http://www.gene.ucl.ac.uk/cgi-bin/nomenclature/searchgenes.pl?field=symbol&anchor=equals&symbol_search=Search&number=100&format=html&sortby=symbol&match=OXCT1) |  | [245050](http://www.ncbi.nlm.nih.gov/entrez/dispomim.cgi?id=245050) | **1.32** |
| 1449066_A_AT | Rho guanine nucleotide exchange factor (GEF7) | [ARHGEF7](http://www.gene.ucl.ac.uk/cgi-bin/nomenclature/searchgenes.pl?field=symbol&anchor=equals&symbol_search=Search&number=100&format=html&sortby=symbol&match=ARHGEF7) |  | [605477](http://www.ncbi.nlm.nih.gov/entrez/dispomim.cgi?id=605477) | **1.31** |
| 1449078_AT | sialyltransferase 10 (alpha-2,3-sialyltransferase VI) | [SIAT10](http://www.gene.ucl.ac.uk/cgi-bin/nomenclature/searchgenes.pl?field=symbol&anchor=equals&symbol_search=Search&number=100&format=html&sortby=symbol&match=SIAT10) |  | [607156](http://www.ncbi.nlm.nih.gov/entrez/dispomim.cgi?id=607156) | **1.35** |
| 1449079_S_AT | gb:AV373417 /DB_XREF=gi:15406845 /DB_XREF=AV373417 |  |  |  | **1.31** |
| 1449081_AT | carboxylesterase 3 | [CES3](http://www.gene.ucl.ac.uk/cgi-bin/nomenclature/searchgenes.pl?field=symbol&anchor=equals&symbol_search=Search&number=100&format=html&sortby=symbol&match=CES3) |  |  | **2.41** |
| 1449082_AT | microfibrillar associated protein 5 | [MFAP5](http://www.gene.ucl.ac.uk/cgi-bin/nomenclature/searchgenes.pl?field=symbol&anchor=equals&symbol_search=Search&number=100&format=html&sortby=symbol&match=MFAP5) |  | [601103](http://www.ncbi.nlm.nih.gov/entrez/dispomim.cgi?id=601103) | **-2.86** |
| 1449084_S_AT | SH3 domain protein D19 | [SH3D19](http://www.gene.ucl.ac.uk/cgi-bin/nomenclature/searchgenes.pl?field=symbol&anchor=equals&symbol_search=Search&number=100&format=html&sortby=symbol&match=SH3D19) |  |  | **-2.18** |
| 1449086_AT | ring finger protein 141 | [RNF141](http://www.gene.ucl.ac.uk/cgi-bin/nomenclature/searchgenes.pl?field=symbol&anchor=equals&symbol_search=Search&number=100&format=html&sortby=symbol&match=RNF141) |  |  | **1.21** |
| 1449088_AT | fructose bisphosphatase 2 | [FBP2](http://www.gene.ucl.ac.uk/cgi-bin/nomenclature/searchgenes.pl?field=symbol&anchor=equals&symbol_search=Search&number=100&format=html&sortby=symbol&match=FBP2) |  | [603027](http://www.ncbi.nlm.nih.gov/entrez/dispomim.cgi?id=603027) | **1.84** |
| 1449093_AT | cardiotrophin 1 | [CTF1](http://www.gene.ucl.ac.uk/cgi-bin/nomenclature/searchgenes.pl?field=symbol&anchor=equals&symbol_search=Search&number=100&format=html&sortby=symbol&match=CTF1) |  | [600435](http://www.ncbi.nlm.nih.gov/entrez/dispomim.cgi?id=600435) | **1.49** |
| 1449094_AT | gap junction membrane channel protein alpha 7 | [GJA7](http://www.gene.ucl.ac.uk/cgi-bin/nomenclature/searchgenes.pl?field=symbol&anchor=equals&symbol_search=Search&number=100&format=html&sortby=symbol&match=GJA7) |  | [608655](http://www.ncbi.nlm.nih.gov/entrez/dispomim.cgi?id=608655) | **1.41** |
| 1449108_AT | ferredoxin 1 | [FDX1](http://www.gene.ucl.ac.uk/cgi-bin/nomenclature/searchgenes.pl?field=symbol&anchor=equals&symbol_search=Search&number=100&format=html&sortby=symbol&match=FDX1) |  | [103260](http://www.ncbi.nlm.nih.gov/entrez/dispomim.cgi?id=103260) | **1.64** |
| 1449110_AT | ras homolog gene family, member B | [RHOB](http://www.gene.ucl.ac.uk/cgi-bin/nomenclature/searchgenes.pl?field=symbol&anchor=equals&symbol_search=Search&number=100&format=html&sortby=symbol&match=RHOB) |  | [165370](http://www.ncbi.nlm.nih.gov/entrez/dispomim.cgi?id=165370) | **1.36** |
| 1449111_A_AT | growth factor receptor bound protein 2 | [GRB2](http://www.gene.ucl.ac.uk/cgi-bin/nomenclature/searchgenes.pl?field=symbol&anchor=equals&symbol_search=Search&number=100&format=html&sortby=symbol&match=GRB2) |  | [108355](http://www.ncbi.nlm.nih.gov/entrez/dispomim.cgi?id=108355) | **-1.4** |
| 1449118_AT | dihydrolipoamide branched chain transacylase E2 | [DBT](http://www.gene.ucl.ac.uk/cgi-bin/nomenclature/searchgenes.pl?field=symbol&anchor=equals&symbol_search=Search&number=100&format=html&sortby=symbol&match=DBT) |  | [248610](http://www.ncbi.nlm.nih.gov/entrez/dispomim.cgi?id=248610) | **1.58** |
| 1449127_AT | selectin, platelet (p-selectin) ligand | [SELPL](http://www.gene.ucl.ac.uk/cgi-bin/nomenclature/searchgenes.pl?field=symbol&anchor=equals&symbol_search=Search&number=100&format=html&sortby=symbol&match=SELPL) |  |  | **-1.85** |
| 1449135_AT | SRY-box containing gene 18 | [SOX18](http://www.gene.ucl.ac.uk/cgi-bin/nomenclature/searchgenes.pl?field=symbol&anchor=equals&symbol_search=Search&number=100&format=html&sortby=symbol&match=SOX18) |  | [601618](http://www.ncbi.nlm.nih.gov/entrez/dispomim.cgi?id=601618) | **1.82** |
| 1449137_AT | pyruvate dehydrogenase E1 alpha 1 | [PDHA1](http://www.gene.ucl.ac.uk/cgi-bin/nomenclature/searchgenes.pl?field=symbol&anchor=equals&symbol_search=Search&number=100&format=html&sortby=symbol&match=PDHA1) |  | [300502](http://www.ncbi.nlm.nih.gov/entrez/dispomim.cgi?id=300502) | **1.51** |
| 1449139_AT | RIKEN cDNA 2310008M10 gene | [2310008M10RIK](http://www.gene.ucl.ac.uk/cgi-bin/nomenclature/searchgenes.pl?field=symbol&anchor=equals&symbol_search=Search&number=100&format=html&sortby=symbol&match=2310008M10RIK) |  |  | **-1.36** |
| 1449142_A_AT | RIKEN cDNA 2610311I19 gene | [2610311I19RIK](http://www.gene.ucl.ac.uk/cgi-bin/nomenclature/searchgenes.pl?field=symbol&anchor=equals&symbol_search=Search&number=100&format=html&sortby=symbol&match=2610311I19RIK) |  |  | **-1.48** |
| 1449145_A_AT | caveolin, caveolae protein 1 | [CAV1](http://www.gene.ucl.ac.uk/cgi-bin/nomenclature/searchgenes.pl?field=symbol&anchor=equals&symbol_search=Search&number=100&format=html&sortby=symbol&match=CAV1) |  | [601047](http://www.ncbi.nlm.nih.gov/entrez/dispomim.cgi?id=601047) | **1.56** |
| 1449146_AT | Notch gene homolog 4 (Drosophila) | [NOTCH4](http://www.gene.ucl.ac.uk/cgi-bin/nomenclature/searchgenes.pl?field=symbol&anchor=equals&symbol_search=Search&number=100&format=html&sortby=symbol&match=NOTCH4) |  | [164951](http://www.ncbi.nlm.nih.gov/entrez/dispomim.cgi?id=164951) | **2.18** |
| 1449153_AT | matrix metalloproteinase 12 | [MMP12](http://www.gene.ucl.ac.uk/cgi-bin/nomenclature/searchgenes.pl?field=symbol&anchor=equals&symbol_search=Search&number=100&format=html&sortby=symbol&match=MMP12) |  | [601046](http://www.ncbi.nlm.nih.gov/entrez/dispomim.cgi?id=601046) | **-25.18** |
| 1449164_AT | CD68 antigen | [CD68](http://www.gene.ucl.ac.uk/cgi-bin/nomenclature/searchgenes.pl?field=symbol&anchor=equals&symbol_search=Search&number=100&format=html&sortby=symbol&match=CD68) |  | [153634](http://www.ncbi.nlm.nih.gov/entrez/dispomim.cgi?id=153634) | **-3.25** |
| 1449168_A_AT | A kinase (PRKA) anchor protein 2 | [AKAP2](http://www.gene.ucl.ac.uk/cgi-bin/nomenclature/searchgenes.pl?field=symbol&anchor=equals&symbol_search=Search&number=100&format=html&sortby=symbol&match=AKAP2) |  | [604582](http://www.ncbi.nlm.nih.gov/entrez/dispomim.cgi?id=604582) | **1.44** |
| 1449175_AT | G-protein coupled receptor 65 | [GPR65](http://www.gene.ucl.ac.uk/cgi-bin/nomenclature/searchgenes.pl?field=symbol&anchor=equals&symbol_search=Search&number=100&format=html&sortby=symbol&match=GPR65) |  | [604620](http://www.ncbi.nlm.nih.gov/entrez/dispomim.cgi?id=604620) | **-2.09** |
| 1449178_AT | PDZ and LIM domain 3 | [PDLIM3](http://www.gene.ucl.ac.uk/cgi-bin/nomenclature/searchgenes.pl?field=symbol&anchor=equals&symbol_search=Search&number=100&format=html&sortby=symbol&match=PDLIM3) |  | [605889](http://www.ncbi.nlm.nih.gov/entrez/dispomim.cgi?id=605889) | **-2.06** |
| 1449180_AT | potassium channel modulatory factor 1 | [KCMF1](http://www.gene.ucl.ac.uk/cgi-bin/nomenclature/searchgenes.pl?field=symbol&anchor=equals&symbol_search=Search&number=100&format=html&sortby=symbol&match=KCMF1) |  |  | **1.71** |
| 1449181_AT | ferrochelatase | [FECH](http://www.gene.ucl.ac.uk/cgi-bin/nomenclature/searchgenes.pl?field=symbol&anchor=equals&symbol_search=Search&number=100&format=html&sortby=symbol&match=FECH) |  | [177000](http://www.ncbi.nlm.nih.gov/entrez/dispomim.cgi?id=177000) | **1.42** |
| 1449190_A_AT | ectonucleoside triphosphate diphosphohydrolase 4 | [ENTPD4](http://www.gene.ucl.ac.uk/cgi-bin/nomenclature/searchgenes.pl?field=symbol&anchor=equals&symbol_search=Search&number=100&format=html&sortby=symbol&match=ENTPD4) |  | [607577](http://www.ncbi.nlm.nih.gov/entrez/dispomim.cgi?id=607577) | **1.55** |
| 1449194_AT | mitochondrial ribosomal protein S25 | [MRPS25](http://www.gene.ucl.ac.uk/cgi-bin/nomenclature/searchgenes.pl?field=symbol&anchor=equals&symbol_search=Search&number=100&format=html&sortby=symbol&match=MRPS25) |  |  | **1.22** |
| 1449195_S_AT | chemokine (C-X-C motif) ligand 16 | [CXCL16](http://www.gene.ucl.ac.uk/cgi-bin/nomenclature/searchgenes.pl?field=symbol&anchor=equals&symbol_search=Search&number=100&format=html&sortby=symbol&match=CXCL16) |  | [605398](http://www.ncbi.nlm.nih.gov/entrez/dispomim.cgi?id=605398) | **-3.88** |
| 1449196_A_AT | ribosomal protein S27a | [RPS27A](http://www.gene.ucl.ac.uk/cgi-bin/nomenclature/searchgenes.pl?field=symbol&anchor=equals&symbol_search=Search&number=100&format=html&sortby=symbol&match=RPS27A) |  | [191343](http://www.ncbi.nlm.nih.gov/entrez/dispomim.cgi?id=191343) | **-1.42** |
| 1449206_AT | mitsugumin 29 | [MG29](http://www.gene.ucl.ac.uk/cgi-bin/nomenclature/searchgenes.pl?field=symbol&anchor=equals&symbol_search=Search&number=100&format=html&sortby=symbol&match=MG29) |  |  | **-1.59** |
| 1449214_A_AT | optic atrophy 1 homolog (human) | [OPA1](http://www.gene.ucl.ac.uk/cgi-bin/nomenclature/searchgenes.pl?field=symbol&anchor=equals&symbol_search=Search&number=100&format=html&sortby=symbol&match=OPA1) |  | [605290](http://www.ncbi.nlm.nih.gov/entrez/dispomim.cgi?id=605290) | **1.47** |
| 1449218_AT | gb:NM_007751.1 /DB_XREF=gi:6680994 /GEN=Cox8b /FEA |  |  |  | **1.3** |
| 1449229_A_AT | cyclin-dependent kinase-like 2 (CDC2-related kinase) | [CDKL2](http://www.gene.ucl.ac.uk/cgi-bin/nomenclature/searchgenes.pl?field=symbol&anchor=equals&symbol_search=Search&number=100&format=html&sortby=symbol&match=CDKL2) |  | [603442](http://www.ncbi.nlm.nih.gov/entrez/dispomim.cgi?id=603442) | **-1.5** |
| 1449243_A_AT | ribosomal protein S19 | [RPS19](http://www.gene.ucl.ac.uk/cgi-bin/nomenclature/searchgenes.pl?field=symbol&anchor=equals&symbol_search=Search&number=100&format=html&sortby=symbol&match=RPS19) |  | [603474](http://www.ncbi.nlm.nih.gov/entrez/dispomim.cgi?id=603474) | **-1.3** |
| 1449254_AT | secreted phosphoprotein 1 | [SPP1](http://www.gene.ucl.ac.uk/cgi-bin/nomenclature/searchgenes.pl?field=symbol&anchor=equals&symbol_search=Search&number=100&format=html&sortby=symbol&match=SPP1) |  | [166490](http://www.ncbi.nlm.nih.gov/entrez/dispomim.cgi?id=166490) | **-22.24** |
| 1449258_AT | DNA segment, Chr 11, Wayne State University 99, expressed | [D11WSU99E](http://www.gene.ucl.ac.uk/cgi-bin/nomenclature/searchgenes.pl?field=symbol&anchor=equals&symbol_search=Search&number=100&format=html&sortby=symbol&match=D11WSU99E) |  |  | **-1.68** |
| 1449273_AT | cytoplasmic FMR1 interacting protein 2 | [CYFIP2](http://www.gene.ucl.ac.uk/cgi-bin/nomenclature/searchgenes.pl?field=symbol&anchor=equals&symbol_search=Search&number=100&format=html&sortby=symbol&match=CYFIP2) |  | [606323](http://www.ncbi.nlm.nih.gov/entrez/dispomim.cgi?id=606323) | **-6.77** |
| 1449275_AT | RIKEN cDNA 2310038H17 gene | [2310038H17RIK](http://www.gene.ucl.ac.uk/cgi-bin/nomenclature/searchgenes.pl?field=symbol&anchor=equals&symbol_search=Search&number=100&format=html&sortby=symbol&match=2310038H17RIK) |  |  | **-1.31** |
| 1449278_AT | eukaryotic translation initiation factor 2 alpha kinase 3 | [EIF2AK3](http://www.gene.ucl.ac.uk/cgi-bin/nomenclature/searchgenes.pl?field=symbol&anchor=equals&symbol_search=Search&number=100&format=html&sortby=symbol&match=EIF2AK3) |  | [604032](http://www.ncbi.nlm.nih.gov/entrez/dispomim.cgi?id=604032) | **-1.38** |
| 1449289_A_AT | beta-2 microglobulin | [B2M](http://www.gene.ucl.ac.uk/cgi-bin/nomenclature/searchgenes.pl?field=symbol&anchor=equals&symbol_search=Search&number=100&format=html&sortby=symbol&match=B2M) |  | [109700](http://www.ncbi.nlm.nih.gov/entrez/dispomim.cgi?id=109700) | **-1.33** |
| 1449303_AT | sestrin 3 | [SESN3](http://www.gene.ucl.ac.uk/cgi-bin/nomenclature/searchgenes.pl?field=symbol&anchor=equals&symbol_search=Search&number=100&format=html&sortby=symbol&match=SESN3) |  | [607768](http://www.ncbi.nlm.nih.gov/entrez/dispomim.cgi?id=607768) | **-1.37** |
| 1449311_AT | BTB and CNC homology 1 | [BACH1](http://www.gene.ucl.ac.uk/cgi-bin/nomenclature/searchgenes.pl?field=symbol&anchor=equals&symbol_search=Search&number=100&format=html&sortby=symbol&match=BACH1) |  | [602751](http://www.ncbi.nlm.nih.gov/entrez/dispomim.cgi?id=602751) | **-1.56** |
| 1449319_AT | thrombospondin type 1 domain containing gene | [RSPONDIN](http://www.gene.ucl.ac.uk/cgi-bin/nomenclature/searchgenes.pl?field=symbol&anchor=equals&symbol_search=Search&number=100&format=html&sortby=symbol&match=RSPONDIN) |  |  | **1.66** |
| 1449323_A_AT | ribosomal protein L3 | [RPL3](http://www.gene.ucl.ac.uk/cgi-bin/nomenclature/searchgenes.pl?field=symbol&anchor=equals&symbol_search=Search&number=100&format=html&sortby=symbol&match=RPL3) |  | [604163](http://www.ncbi.nlm.nih.gov/entrez/dispomim.cgi?id=604163) | **-2.19** |
| 1449334_AT | tissue inhibitor of metalloproteinase 3 | [TIMP3](http://www.gene.ucl.ac.uk/cgi-bin/nomenclature/searchgenes.pl?field=symbol&anchor=equals&symbol_search=Search&number=100&format=html&sortby=symbol&match=TIMP3) |  | [188826](http://www.ncbi.nlm.nih.gov/entrez/dispomim.cgi?id=188826) | **2.46** |
| 1449335_AT | tissue inhibitor of metalloproteinase 3 | [TIMP3](http://www.gene.ucl.ac.uk/cgi-bin/nomenclature/searchgenes.pl?field=symbol&anchor=equals&symbol_search=Search&number=100&format=html&sortby=symbol&match=TIMP3) |  | [188826](http://www.ncbi.nlm.nih.gov/entrez/dispomim.cgi?id=188826) | **2.15** |
| 1449336_A_AT | STE20-like kinase (yeast) | [SLK](http://www.gene.ucl.ac.uk/cgi-bin/nomenclature/searchgenes.pl?field=symbol&anchor=equals&symbol_search=Search&number=100&format=html&sortby=symbol&match=SLK) |  |  | **-1.49** |
| 1449352_AT | polymerase (DNA directed), lambda | [POLL](http://www.gene.ucl.ac.uk/cgi-bin/nomenclature/searchgenes.pl?field=symbol&anchor=equals&symbol_search=Search&number=100&format=html&sortby=symbol&match=POLL) |  | [606343](http://www.ncbi.nlm.nih.gov/entrez/dispomim.cgi?id=606343) | **1.47** |
| 1449360_AT | colony stimulating factor 2 receptor, beta 2, low-affinity (granulocyte-macrophage) | [CSF2RB2](http://www.gene.ucl.ac.uk/cgi-bin/nomenclature/searchgenes.pl?field=symbol&anchor=equals&symbol_search=Search&number=100&format=html&sortby=symbol&match=CSF2RB2) |  |  | **-3.17** |
| 1449370_AT | SRY-box containing gene 4 | [SOX4](http://www.gene.ucl.ac.uk/cgi-bin/nomenclature/searchgenes.pl?field=symbol&anchor=equals&symbol_search=Search&number=100&format=html&sortby=symbol&match=SOX4) |  | [184430](http://www.ncbi.nlm.nih.gov/entrez/dispomim.cgi?id=184430) | **-2.53** |
| 1449372_AT | DnaJ (Hsp40) homolog, subfamily C, member 3 | [DNAJC3](http://www.gene.ucl.ac.uk/cgi-bin/nomenclature/searchgenes.pl?field=symbol&anchor=equals&symbol_search=Search&number=100&format=html&sortby=symbol&match=DNAJC3) |  | [601184](http://www.ncbi.nlm.nih.gov/entrez/dispomim.cgi?id=601184) | **-1.32** |
| 1449379_AT | kinase insert domain protein receptor | [KDR](http://www.gene.ucl.ac.uk/cgi-bin/nomenclature/searchgenes.pl?field=symbol&anchor=equals&symbol_search=Search&number=100&format=html&sortby=symbol&match=KDR) |  | [191306](http://www.ncbi.nlm.nih.gov/entrez/dispomim.cgi?id=191306) | **1.86** |
| 1449383_AT | adenylosuccinate synthetase like 1 | [ADSSL1](http://www.gene.ucl.ac.uk/cgi-bin/nomenclature/searchgenes.pl?field=symbol&anchor=equals&symbol_search=Search&number=100&format=html&sortby=symbol&match=ADSSL1) |  | [103060](http://www.ncbi.nlm.nih.gov/entrez/dispomim.cgi?id=103060) | **1.81** |
| 1449388_AT | thrombospondin 4 | [THBS4](http://www.gene.ucl.ac.uk/cgi-bin/nomenclature/searchgenes.pl?field=symbol&anchor=equals&symbol_search=Search&number=100&format=html&sortby=symbol&match=THBS4) |  | [600715](http://www.ncbi.nlm.nih.gov/entrez/dispomim.cgi?id=600715) | **-5.11** |
| 1449396_AT | amine oxidase, copper containing 3 | [AOC3](http://www.gene.ucl.ac.uk/cgi-bin/nomenclature/searchgenes.pl?field=symbol&anchor=equals&symbol_search=Search&number=100&format=html&sortby=symbol&match=AOC3) |  | [603735](http://www.ncbi.nlm.nih.gov/entrez/dispomim.cgi?id=603735) | **1.62** |
| 1449398_AT | ribosomal protein L3-like | [RPL3L](http://www.gene.ucl.ac.uk/cgi-bin/nomenclature/searchgenes.pl?field=symbol&anchor=equals&symbol_search=Search&number=100&format=html&sortby=symbol&match=RPL3L) |  |  | **1.62** |
| 1449401_AT | complement component 1, q subcomponent, gamma polypeptide | [C1QG](http://www.gene.ucl.ac.uk/cgi-bin/nomenclature/searchgenes.pl?field=symbol&anchor=equals&symbol_search=Search&number=100&format=html&sortby=symbol&match=C1QG) |  | [120575](http://www.ncbi.nlm.nih.gov/entrez/dispomim.cgi?id=120575) | **-3.52** |
| 1449404_AT | phosphatidylinositol-4-phosphate 5-kinase, type II, alpha | [PIP5K2A](http://www.gene.ucl.ac.uk/cgi-bin/nomenclature/searchgenes.pl?field=symbol&anchor=equals&symbol_search=Search&number=100&format=html&sortby=symbol&match=PIP5K2A) |  | [603140](http://www.ncbi.nlm.nih.gov/entrez/dispomim.cgi?id=603140) | **-1.59** |
| 1449405_AT | tensin | [TNS](http://www.gene.ucl.ac.uk/cgi-bin/nomenclature/searchgenes.pl?field=symbol&anchor=equals&symbol_search=Search&number=100&format=html&sortby=symbol&match=TNS) |  | [600076](http://www.ncbi.nlm.nih.gov/entrez/dispomim.cgi?id=600076) | **1.39** |
| 1449406_AT | cysteine and histidine rich 1 | [CYHR1](http://www.gene.ucl.ac.uk/cgi-bin/nomenclature/searchgenes.pl?field=symbol&anchor=equals&symbol_search=Search&number=100&format=html&sortby=symbol&match=CYHR1) |  |  | **1.34** |
| 1449408_AT | junction adhesion molecule 2 | [JAM2](http://www.gene.ucl.ac.uk/cgi-bin/nomenclature/searchgenes.pl?field=symbol&anchor=equals&symbol_search=Search&number=100&format=html&sortby=symbol&match=JAM2) |  | [606870](http://www.ncbi.nlm.nih.gov/entrez/dispomim.cgi?id=606870) | **1.43** |
| 1449410_A_AT | growth arrest specific 5 | [GAS5](http://www.gene.ucl.ac.uk/cgi-bin/nomenclature/searchgenes.pl?field=symbol&anchor=equals&symbol_search=Search&number=100&format=html&sortby=symbol&match=GAS5) |  |  | **-1.56** |
| 1449438_AT | dolichol-phosphate (beta-D) mannosyltransferase 1 | [DPM1](http://www.gene.ucl.ac.uk/cgi-bin/nomenclature/searchgenes.pl?field=symbol&anchor=equals&symbol_search=Search&number=100&format=html&sortby=symbol&match=DPM1) |  | [603503](http://www.ncbi.nlm.nih.gov/entrez/dispomim.cgi?id=603503) | **1.24** |
| 1449442_AT | peroxisomal biogenesis factor 11a | [PEX11A](http://www.gene.ucl.ac.uk/cgi-bin/nomenclature/searchgenes.pl?field=symbol&anchor=equals&symbol_search=Search&number=100&format=html&sortby=symbol&match=PEX11A) |  | [603866](http://www.ncbi.nlm.nih.gov/entrez/dispomim.cgi?id=603866) | **1.66** |
| 1449443_AT | 2,4-dienoyl CoA reductase 1, mitochondrial | [DECR1](http://www.gene.ucl.ac.uk/cgi-bin/nomenclature/searchgenes.pl?field=symbol&anchor=equals&symbol_search=Search&number=100&format=html&sortby=symbol&match=DECR1) |  | [222745](http://www.ncbi.nlm.nih.gov/entrez/dispomim.cgi?id=222745) | **2.29** |
| 1449455_AT | hemopoietic cell kinase | [HCK](http://www.gene.ucl.ac.uk/cgi-bin/nomenclature/searchgenes.pl?field=symbol&anchor=equals&symbol_search=Search&number=100&format=html&sortby=symbol&match=HCK) |  | [142370](http://www.ncbi.nlm.nih.gov/entrez/dispomim.cgi?id=142370) | **-1.73** |
| 1449491_AT | caspase recruitment domain family, member 10 | [CARD10](http://www.gene.ucl.ac.uk/cgi-bin/nomenclature/searchgenes.pl?field=symbol&anchor=equals&symbol_search=Search&number=100&format=html&sortby=symbol&match=CARD10) |  | [607209](http://www.ncbi.nlm.nih.gov/entrez/dispomim.cgi?id=607209) | **1.72** |
| 1449507_A_AT | CD47 antigen (Rh-related antigen, integrin-associated signal transducer) | [CD47](http://www.gene.ucl.ac.uk/cgi-bin/nomenclature/searchgenes.pl?field=symbol&anchor=equals&symbol_search=Search&number=100&format=html&sortby=symbol&match=CD47) |  | [601028](http://www.ncbi.nlm.nih.gov/entrez/dispomim.cgi?id=601028) | **-1.27** |
| 1449516_A_AT | regulator of G-protein signaling 3 | [RGS3](http://www.gene.ucl.ac.uk/cgi-bin/nomenclature/searchgenes.pl?field=symbol&anchor=equals&symbol_search=Search&number=100&format=html&sortby=symbol&match=RGS3) |  | [602189](http://www.ncbi.nlm.nih.gov/entrez/dispomim.cgi?id=602189) | **1.47** |
| 1449519_AT | growth arrest and DNA-damage-inducible 45 alpha | [GADD45A](http://www.gene.ucl.ac.uk/cgi-bin/nomenclature/searchgenes.pl?field=symbol&anchor=equals&symbol_search=Search&number=100&format=html&sortby=symbol&match=GADD45A) |  | [126335](http://www.ncbi.nlm.nih.gov/entrez/dispomim.cgi?id=126335) | **-1.79** |
| 1449528_AT | c-fos induced growth factor | [FIGF](http://www.gene.ucl.ac.uk/cgi-bin/nomenclature/searchgenes.pl?field=symbol&anchor=equals&symbol_search=Search&number=100&format=html&sortby=symbol&match=FIGF) |  | [300091](http://www.ncbi.nlm.nih.gov/entrez/dispomim.cgi?id=300091) | **1.35** |
| 1449530_AT | trichorhinophalangeal syndrome I (human) | [TRPS1](http://www.gene.ucl.ac.uk/cgi-bin/nomenclature/searchgenes.pl?field=symbol&anchor=equals&symbol_search=Search&number=100&format=html&sortby=symbol&match=TRPS1) |  | [604386](http://www.ncbi.nlm.nih.gov/entrez/dispomim.cgi?id=604386) | **-1.39** |
| 1449532_AT | cholinergic receptor, nicotinic, gamma polypeptide | [CHRNG](http://www.gene.ucl.ac.uk/cgi-bin/nomenclature/searchgenes.pl?field=symbol&anchor=equals&symbol_search=Search&number=100&format=html&sortby=symbol&match=CHRNG) |  | [100730](http://www.ncbi.nlm.nih.gov/entrez/dispomim.cgi?id=100730) | **-32.98** |
| 1449536_AT | potassium intermediate/small conductance calcium-activated channel, subfamily N, member 1 | [KCNN1](http://www.gene.ucl.ac.uk/cgi-bin/nomenclature/searchgenes.pl?field=symbol&anchor=equals&symbol_search=Search&number=100&format=html&sortby=symbol&match=KCNN1) |  | [602982](http://www.ncbi.nlm.nih.gov/entrez/dispomim.cgi?id=602982) | **1.56** |
| 1449547_AT | ankyrin repeat and SOCS box-containing protein 14 | [ASB14](http://www.gene.ucl.ac.uk/cgi-bin/nomenclature/searchgenes.pl?field=symbol&anchor=equals&symbol_search=Search&number=100&format=html&sortby=symbol&match=ASB14) |  |  | **1.77** |
| 1449556_AT | histocompatibility 2, T region locus 23 | [H2-T23](http://www.gene.ucl.ac.uk/cgi-bin/nomenclature/searchgenes.pl?field=symbol&anchor=equals&symbol_search=Search&number=100&format=html&sortby=symbol&match=H2-T23) |  |  | **-1.48** |
| 1449591_AT | caspase 4, apoptosis-related cysteine protease | [CASP4](http://www.gene.ucl.ac.uk/cgi-bin/nomenclature/searchgenes.pl?field=symbol&anchor=equals&symbol_search=Search&number=100&format=html&sortby=symbol&match=CASP4) |  | [602664](http://www.ncbi.nlm.nih.gov/entrez/dispomim.cgi?id=602664) | **-1.9** |
| 1449592_AT | transcription factor 15 | [TCF15](http://www.gene.ucl.ac.uk/cgi-bin/nomenclature/searchgenes.pl?field=symbol&anchor=equals&symbol_search=Search&number=100&format=html&sortby=symbol&match=TCF15) |  | [601010](http://www.ncbi.nlm.nih.gov/entrez/dispomim.cgi?id=601010) | **2.19** |
| 1449615_S_AT | high density lipoprotein (HDL) binding protein | [HDLBP](http://www.gene.ucl.ac.uk/cgi-bin/nomenclature/searchgenes.pl?field=symbol&anchor=equals&symbol_search=Search&number=100&format=html&sortby=symbol&match=HDLBP) |  | [142695](http://www.ncbi.nlm.nih.gov/entrez/dispomim.cgi?id=142695) | **1.54** |
| 1449618_S_AT | RIKEN cDNA 2900092E17 gene | [2900092E17RIK](http://www.gene.ucl.ac.uk/cgi-bin/nomenclature/searchgenes.pl?field=symbol&anchor=equals&symbol_search=Search&number=100&format=html&sortby=symbol&match=2900092E17RIK) |  |  | **-1.25** |
| 1449620_S_AT | gb:AW125421 /DB_XREF=gi:6100951 /DB_XREF=UI-M-BH2. |  |  |  | **1.32** |
| 1449626_S_AT | acyl-Coenzyme A binding domain containing 4 | [ACBD4](http://www.gene.ucl.ac.uk/cgi-bin/nomenclature/searchgenes.pl?field=symbol&anchor=equals&symbol_search=Search&number=100&format=html&sortby=symbol&match=ACBD4) |  |  | **-1.48** |
| 1449628_S_AT | START domain containing 7 | [STARD7](http://www.gene.ucl.ac.uk/cgi-bin/nomenclature/searchgenes.pl?field=symbol&anchor=equals&symbol_search=Search&number=100&format=html&sortby=symbol&match=STARD7) |  |  | **1.26** |
| 1449629_S_AT | gb:AW046420 /DB_XREF=gi:5906949 /DB_XREF=UI-M-BH1- |  |  |  | **1.37** |
| 1449630_S_AT | MAP/microtubule affinity-regulating kinase 1 | [MARK1](http://www.gene.ucl.ac.uk/cgi-bin/nomenclature/searchgenes.pl?field=symbol&anchor=equals&symbol_search=Search&number=100&format=html&sortby=symbol&match=MARK1) |  | [606511](http://www.ncbi.nlm.nih.gov/entrez/dispomim.cgi?id=606511) | **1.86** |
| 1449632_S_AT | FK506 binding protein 10 | [FKBP10](http://www.gene.ucl.ac.uk/cgi-bin/nomenclature/searchgenes.pl?field=symbol&anchor=equals&symbol_search=Search&number=100&format=html&sortby=symbol&match=FKBP10) |  | [607063](http://www.ncbi.nlm.nih.gov/entrez/dispomim.cgi?id=607063) | **-2.12** |
| 1449648_S_AT | RNA polymerase 1-1 | [RPO1-1](http://www.gene.ucl.ac.uk/cgi-bin/nomenclature/searchgenes.pl?field=symbol&anchor=equals&symbol_search=Search&number=100&format=html&sortby=symbol&match=RPO1-1) |  |  | **-1.57** |
| 1449664_S_AT | ring finger protein 20 | [RNF20](http://www.gene.ucl.ac.uk/cgi-bin/nomenclature/searchgenes.pl?field=symbol&anchor=equals&symbol_search=Search&number=100&format=html&sortby=symbol&match=RNF20) |  | [607699](http://www.ncbi.nlm.nih.gov/entrez/dispomim.cgi?id=607699) | **-1.35** |
| 1449677_S_AT | DNA segment, Chr 4, ERATO Doi 89, expressed | [TMEM38B](http://www.gene.ucl.ac.uk/cgi-bin/nomenclature/searchgenes.pl?field=symbol&anchor=equals&symbol_search=Search&number=100&format=html&sortby=symbol&match=TMEM38B) |  |  | **-1.32** |
| 1449679_S_AT | syntaxin 5A | [STX5A](http://www.gene.ucl.ac.uk/cgi-bin/nomenclature/searchgenes.pl?field=symbol&anchor=equals&symbol_search=Search&number=100&format=html&sortby=symbol&match=STX5A) |  | [603189](http://www.ncbi.nlm.nih.gov/entrez/dispomim.cgi?id=603189) | **-1.29** |
| 1449682_S_AT | RIKEN cDNA 2410129E14 gene | [2410129E14RIK](http://www.gene.ucl.ac.uk/cgi-bin/nomenclature/searchgenes.pl?field=symbol&anchor=equals&symbol_search=Search&number=100&format=html&sortby=symbol&match=2410129E14RIK) |  |  | **-10.37** |
| 1449686_S_AT | sterol carrier protein 2, liver | [SCP2](http://www.gene.ucl.ac.uk/cgi-bin/nomenclature/searchgenes.pl?field=symbol&anchor=equals&symbol_search=Search&number=100&format=html&sortby=symbol&match=SCP2) |  | [184755](http://www.ncbi.nlm.nih.gov/entrez/dispomim.cgi?id=184755) | **1.24** |
| 1449697_S_AT | mitofusin 1 | [MFN1](http://www.gene.ucl.ac.uk/cgi-bin/nomenclature/searchgenes.pl?field=symbol&anchor=equals&symbol_search=Search&number=100&format=html&sortby=symbol&match=MFN1) |  | [608506](http://www.ncbi.nlm.nih.gov/entrez/dispomim.cgi?id=608506) | **1.72** |
| 1449710_S_AT | ATP synthase, H+ transporting, mitochondrial F1 complex, alpha subunit, isoform 1 | [ATP5A1](http://www.gene.ucl.ac.uk/cgi-bin/nomenclature/searchgenes.pl?field=symbol&anchor=equals&symbol_search=Search&number=100&format=html&sortby=symbol&match=ATP5A1) |  | [164360](http://www.ncbi.nlm.nih.gov/entrez/dispomim.cgi?id=164360) | **1.25** |
| 1449799_S_AT | plakophilin 2 | [PKP2](http://www.gene.ucl.ac.uk/cgi-bin/nomenclature/searchgenes.pl?field=symbol&anchor=equals&symbol_search=Search&number=100&format=html&sortby=symbol&match=PKP2) |  | [602861](http://www.ncbi.nlm.nih.gov/entrez/dispomim.cgi?id=602861) | **-2.06** |
| 1449818_AT | ATP-binding cassette, sub-family B (MDR/TAP), member 4 | [ABCB4](http://www.gene.ucl.ac.uk/cgi-bin/nomenclature/searchgenes.pl?field=symbol&anchor=equals&symbol_search=Search&number=100&format=html&sortby=symbol&match=ABCB4) |  | [171060](http://www.ncbi.nlm.nih.gov/entrez/dispomim.cgi?id=171060) | **1.5** |
| 1449823_AT | dachshund 2 (Drosophila) | [DACH2](http://www.gene.ucl.ac.uk/cgi-bin/nomenclature/searchgenes.pl?field=symbol&anchor=equals&symbol_search=Search&number=100&format=html&sortby=symbol&match=DACH2) |  |  | **10.45** |
| 1449824_AT | Proteoglycan 4 (megakaryocyte stimulating factor, articular superficial zone protein) | [TPR](http://www.gene.ucl.ac.uk/cgi-bin/nomenclature/searchgenes.pl?field=symbol&anchor=equals&symbol_search=Search&number=100&format=html&sortby=symbol&match=TPR) |  | 604283 189940 | **-4.71** |
| 1449834_AT | PDZ domain containing, X chromosome | [PDZX](http://www.gene.ucl.ac.uk/cgi-bin/nomenclature/searchgenes.pl?field=symbol&anchor=equals&symbol_search=Search&number=100&format=html&sortby=symbol&match=PDZX) |  |  | **2.83** |
| 1449839_AT | caspase 3, apoptosis related cysteine protease | [CASP3](http://www.gene.ucl.ac.uk/cgi-bin/nomenclature/searchgenes.pl?field=symbol&anchor=equals&symbol_search=Search&number=100&format=html&sortby=symbol&match=CASP3) |  | [600636](http://www.ncbi.nlm.nih.gov/entrez/dispomim.cgi?id=600636) | **-2.47** |
| 1449846_AT | eosinophil-associated, ribonuclease A family, member 2 | [EAR2](http://www.gene.ucl.ac.uk/cgi-bin/nomenclature/searchgenes.pl?field=symbol&anchor=equals&symbol_search=Search&number=100&format=html&sortby=symbol&match=EAR2) |  |  | **-1.54** |
| 1449852_A_AT | EH-domain containing 4 | [EHD4](http://www.gene.ucl.ac.uk/cgi-bin/nomenclature/searchgenes.pl?field=symbol&anchor=equals&symbol_search=Search&number=100&format=html&sortby=symbol&match=EHD4) |  | [605892](http://www.ncbi.nlm.nih.gov/entrez/dispomim.cgi?id=605892) | **-1.77** |
| 1449860_AT | RIKEN cDNA 2310056K19 gene | [2310056K19RIK](http://www.gene.ucl.ac.uk/cgi-bin/nomenclature/searchgenes.pl?field=symbol&anchor=equals&symbol_search=Search&number=100&format=html&sortby=symbol&match=2310056K19RIK) |  |  | **1.47** |
| 1449875_S_AT | histocompatibility 2, T region locus 10 | [H2-T10](http://www.gene.ucl.ac.uk/cgi-bin/nomenclature/searchgenes.pl?field=symbol&anchor=equals&symbol_search=Search&number=100&format=html&sortby=symbol&match=H2-T10) |  |  | **-1.53** |
| 1449876_AT | protein kinase, cGMP-dependent, type I | [PRKG1](http://www.gene.ucl.ac.uk/cgi-bin/nomenclature/searchgenes.pl?field=symbol&anchor=equals&symbol_search=Search&number=100&format=html&sortby=symbol&match=PRKG1) |  | [176894](http://www.ncbi.nlm.nih.gov/entrez/dispomim.cgi?id=176894) | **1.93** |
| 1449888_AT | endothelial PAS domain protein 1 | [EPAS1](http://www.gene.ucl.ac.uk/cgi-bin/nomenclature/searchgenes.pl?field=symbol&anchor=equals&symbol_search=Search&number=100&format=html&sortby=symbol&match=EPAS1) |  | [603349](http://www.ncbi.nlm.nih.gov/entrez/dispomim.cgi?id=603349) | **2.62** |
| 1449893_A_AT | leucine-rich repeats and immunoglobulin-like domains 1 | [LRIG1](http://www.gene.ucl.ac.uk/cgi-bin/nomenclature/searchgenes.pl?field=symbol&anchor=equals&symbol_search=Search&number=100&format=html&sortby=symbol&match=LRIG1) |  | [608868](http://www.ncbi.nlm.nih.gov/entrez/dispomim.cgi?id=608868) | **1.58** |
| 1449930_A_AT | signal sequence receptor, beta | [SSR2](http://www.gene.ucl.ac.uk/cgi-bin/nomenclature/searchgenes.pl?field=symbol&anchor=equals&symbol_search=Search&number=100&format=html&sortby=symbol&match=SSR2) |  | [600867](http://www.ncbi.nlm.nih.gov/entrez/dispomim.cgi?id=600867) | **-1.38** |
| 1449942_A_AT | integrin linked kinase | [ILK](http://www.gene.ucl.ac.uk/cgi-bin/nomenclature/searchgenes.pl?field=symbol&anchor=equals&symbol_search=Search&number=100&format=html&sortby=symbol&match=ILK) |  | [602366](http://www.ncbi.nlm.nih.gov/entrez/dispomim.cgi?id=602366) | **-1.47** |
| 1449964_A_AT | malonyl-CoA decarboxylase | [MLYCD](http://www.gene.ucl.ac.uk/cgi-bin/nomenclature/searchgenes.pl?field=symbol&anchor=equals&symbol_search=Search&number=100&format=html&sortby=symbol&match=MLYCD) |  | [606761](http://www.ncbi.nlm.nih.gov/entrez/dispomim.cgi?id=606761) | **1.32** |
| 1449968_S_AT | acyl-Coenzyme A thioesterase 3, mitochondrial | [ACATE2](http://www.gene.ucl.ac.uk/cgi-bin/nomenclature/searchgenes.pl?field=symbol&anchor=equals&symbol_search=Search&number=100&format=html&sortby=symbol&match=ACATE2) |  |  | **-1.88** |
| 1450006_AT | nuclear receptor coactivator 4 | [NCOA4](http://www.gene.ucl.ac.uk/cgi-bin/nomenclature/searchgenes.pl?field=symbol&anchor=equals&symbol_search=Search&number=100&format=html&sortby=symbol&match=NCOA4) |  | [601984](http://www.ncbi.nlm.nih.gov/entrez/dispomim.cgi?id=601984) | **1.35** |
| 1450016_AT | cyclin G1 | [CCNG1](http://www.gene.ucl.ac.uk/cgi-bin/nomenclature/searchgenes.pl?field=symbol&anchor=equals&symbol_search=Search&number=100&format=html&sortby=symbol&match=CCNG1) |  | [601578](http://www.ncbi.nlm.nih.gov/entrez/dispomim.cgi?id=601578) | **1.55** |
| 1450017_AT | gb:BG065754 /DB_XREF=gi:12548317 /DB_XREF=H3034H06 |  |  |  | **1.56** |
| 1450020_AT | chemokine (C-X3-C) receptor 1 | [CX3CR1](http://www.gene.ucl.ac.uk/cgi-bin/nomenclature/searchgenes.pl?field=symbol&anchor=equals&symbol_search=Search&number=100&format=html&sortby=symbol&match=CX3CR1) |  | [601470](http://www.ncbi.nlm.nih.gov/entrez/dispomim.cgi?id=601470) | **-3.42** |
| 1450025_AT | par-6 partitioning defective 6 homolog gamma (C. elegans) | [PARD6G](http://www.gene.ucl.ac.uk/cgi-bin/nomenclature/searchgenes.pl?field=symbol&anchor=equals&symbol_search=Search&number=100&format=html&sortby=symbol&match=PARD6G) |  | [608976](http://www.ncbi.nlm.nih.gov/entrez/dispomim.cgi?id=608976) | **1.58** |
| 1450044_AT | frizzled homolog 7 (Drosophila) | [FZD7](http://www.gene.ucl.ac.uk/cgi-bin/nomenclature/searchgenes.pl?field=symbol&anchor=equals&symbol_search=Search&number=100&format=html&sortby=symbol&match=FZD7) |  | [603410](http://www.ncbi.nlm.nih.gov/entrez/dispomim.cgi?id=603410) | **1.45** |
| 1450047_AT | heparan sulfate 6-O-sulfotransferase 2 | [HS6ST2](http://www.gene.ucl.ac.uk/cgi-bin/nomenclature/searchgenes.pl?field=symbol&anchor=equals&symbol_search=Search&number=100&format=html&sortby=symbol&match=HS6ST2) |  |  | **-2.17** |
| 1450048_A_AT | isocitrate dehydrogenase 2 (NADP+), mitochondrial | [IDH2](http://www.gene.ucl.ac.uk/cgi-bin/nomenclature/searchgenes.pl?field=symbol&anchor=equals&symbol_search=Search&number=100&format=html&sortby=symbol&match=IDH2) |  | [147650](http://www.ncbi.nlm.nih.gov/entrez/dispomim.cgi?id=147650) | **1.58** |
| 1450062_A_AT | melanoma antigen, family D, 1 | [MAGED1](http://www.gene.ucl.ac.uk/cgi-bin/nomenclature/searchgenes.pl?field=symbol&anchor=equals&symbol_search=Search&number=100&format=html&sortby=symbol&match=MAGED1) |  | [300224](http://www.ncbi.nlm.nih.gov/entrez/dispomim.cgi?id=300224) | **-1.81** |
| 1450065_AT | adenylate cyclase 7 | [ADCY7](http://www.gene.ucl.ac.uk/cgi-bin/nomenclature/searchgenes.pl?field=symbol&anchor=equals&symbol_search=Search&number=100&format=html&sortby=symbol&match=ADCY7) |  | [600385](http://www.ncbi.nlm.nih.gov/entrez/dispomim.cgi?id=600385) | **-2.52** |
| 1450069_A_AT | CUG triplet repeat,RNA binding protein 2 | [CUGBP2](http://www.gene.ucl.ac.uk/cgi-bin/nomenclature/searchgenes.pl?field=symbol&anchor=equals&symbol_search=Search&number=100&format=html&sortby=symbol&match=CUGBP2) |  | [602538](http://www.ncbi.nlm.nih.gov/entrez/dispomim.cgi?id=602538) | **-1.39** |
| 1450084_S_AT | influenza virus NS1A binding protein | [IVNS1ABP](http://www.gene.ucl.ac.uk/cgi-bin/nomenclature/searchgenes.pl?field=symbol&anchor=equals&symbol_search=Search&number=100&format=html&sortby=symbol&match=IVNS1ABP) |  |  | **1.29** |
| 1450085_AT | angiopoietin-like 2 | [ANGPTL2](http://www.gene.ucl.ac.uk/cgi-bin/nomenclature/searchgenes.pl?field=symbol&anchor=equals&symbol_search=Search&number=100&format=html&sortby=symbol&match=ANGPTL2) |  | [605001](http://www.ncbi.nlm.nih.gov/entrez/dispomim.cgi?id=605001) | **-1.64** |
| 1450095_A_AT | acylphosphatase 1, erythrocyte (common) type | [ACYP1](http://www.gene.ucl.ac.uk/cgi-bin/nomenclature/searchgenes.pl?field=symbol&anchor=equals&symbol_search=Search&number=100&format=html&sortby=symbol&match=ACYP1) |  | [600875](http://www.ncbi.nlm.nih.gov/entrez/dispomim.cgi?id=600875) | **1.45** |
| 1450097_S_AT | guanine nucleotide binding protein, alpha 12 | [GNA12](http://www.gene.ucl.ac.uk/cgi-bin/nomenclature/searchgenes.pl?field=symbol&anchor=equals&symbol_search=Search&number=100&format=html&sortby=symbol&match=GNA12) |  | [604394](http://www.ncbi.nlm.nih.gov/entrez/dispomim.cgi?id=604394) | **1.4** |
| 1450099_A_AT | glucosidase, beta, acid | [GBA](http://www.gene.ucl.ac.uk/cgi-bin/nomenclature/searchgenes.pl?field=symbol&anchor=equals&symbol_search=Search&number=100&format=html&sortby=symbol&match=GBA) |  | [606463](http://www.ncbi.nlm.nih.gov/entrez/dispomim.cgi?id=606463) | **-1.47** |
| 1450102_A_AT | autocrine motility factor receptor | [AMFR](http://www.gene.ucl.ac.uk/cgi-bin/nomenclature/searchgenes.pl?field=symbol&anchor=equals&symbol_search=Search&number=100&format=html&sortby=symbol&match=AMFR) |  | [603243](http://www.ncbi.nlm.nih.gov/entrez/dispomim.cgi?id=603243) | **1.22** |
| 1450111_A_AT | nudix (nucleoside diphosphate linked moiety X)-type motif 8 | [NUDT8](http://www.gene.ucl.ac.uk/cgi-bin/nomenclature/searchgenes.pl?field=symbol&anchor=equals&symbol_search=Search&number=100&format=html&sortby=symbol&match=NUDT8) |  |  | **1.73** |
| 1450122_AT | protein tyrosine phosphatase, receptor type, G | [PTPRG](http://www.gene.ucl.ac.uk/cgi-bin/nomenclature/searchgenes.pl?field=symbol&anchor=equals&symbol_search=Search&number=100&format=html&sortby=symbol&match=PTPRG) |  | [176886](http://www.ncbi.nlm.nih.gov/entrez/dispomim.cgi?id=176886) | **1.54** |
| 1450138_A_AT | serine (or cysteine) proteinase inhibitor, clade B, member 6a | [SERPINB6A](http://www.gene.ucl.ac.uk/cgi-bin/nomenclature/searchgenes.pl?field=symbol&anchor=equals&symbol_search=Search&number=100&format=html&sortby=symbol&match=SERPINB6A) |  |  | **-3.47** |
| 1450150_A_AT | ribosomal protein L13 | [RPL13](http://www.gene.ucl.ac.uk/cgi-bin/nomenclature/searchgenes.pl?field=symbol&anchor=equals&symbol_search=Search&number=100&format=html&sortby=symbol&match=RPL13) |  | [113703](http://www.ncbi.nlm.nih.gov/entrez/dispomim.cgi?id=113703) | **-1.22** |
| 1450165_AT | schlafen 2 | [SLFN2](http://www.gene.ucl.ac.uk/cgi-bin/nomenclature/searchgenes.pl?field=symbol&anchor=equals&symbol_search=Search&number=100&format=html&sortby=symbol&match=SLFN2) |  |  | **-1.96** |
| 1450191_A_AT | SRY-box containing gene 13 | [SOX13](http://www.gene.ucl.ac.uk/cgi-bin/nomenclature/searchgenes.pl?field=symbol&anchor=equals&symbol_search=Search&number=100&format=html&sortby=symbol&match=SOX13) |  | [604748](http://www.ncbi.nlm.nih.gov/entrez/dispomim.cgi?id=604748) | **1.65** |
| 1450196_S_AT | glycogen synthase 1, muscle | [GYS1](http://www.gene.ucl.ac.uk/cgi-bin/nomenclature/searchgenes.pl?field=symbol&anchor=equals&symbol_search=Search&number=100&format=html&sortby=symbol&match=GYS1) |  | [138570](http://www.ncbi.nlm.nih.gov/entrez/dispomim.cgi?id=138570) | **1.35** |
| 1450198_AT | dual specificity phosphatase 13 | [DUSP13](http://www.gene.ucl.ac.uk/cgi-bin/nomenclature/searchgenes.pl?field=symbol&anchor=equals&symbol_search=Search&number=100&format=html&sortby=symbol&match=DUSP13) |  |  | **1.64** |
| 1450234_AT | membrane-spanning 4-domains, subfamily A, member 6C | [MS4A6C](http://www.gene.ucl.ac.uk/cgi-bin/nomenclature/searchgenes.pl?field=symbol&anchor=equals&symbol_search=Search&number=100&format=html&sortby=symbol&match=MS4A6C) |  |  | **-2.95** |
| 1450241_A_AT | ecotropic viral integration site 2a | [EVI2A](http://www.gene.ucl.ac.uk/cgi-bin/nomenclature/searchgenes.pl?field=symbol&anchor=equals&symbol_search=Search&number=100&format=html&sortby=symbol&match=EVI2A) |  | [158380](http://www.ncbi.nlm.nih.gov/entrez/dispomim.cgi?id=158380) | **-2.6** |
| 1450243_A_AT | Down syndrome critical region gene 1-like 1 | [DSCR1L1](http://www.gene.ucl.ac.uk/cgi-bin/nomenclature/searchgenes.pl?field=symbol&anchor=equals&symbol_search=Search&number=100&format=html&sortby=symbol&match=DSCR1L1) |  | [604876](http://www.ncbi.nlm.nih.gov/entrez/dispomim.cgi?id=604876) | **1.71** |
| 1450244_A_AT | mitogen activated protein kinase kinase kinase kinase 2 | [MAP4K2](http://www.gene.ucl.ac.uk/cgi-bin/nomenclature/searchgenes.pl?field=symbol&anchor=equals&symbol_search=Search&number=100&format=html&sortby=symbol&match=MAP4K2) |  | [603166](http://www.ncbi.nlm.nih.gov/entrez/dispomim.cgi?id=603166) | **1.39** |
| 1450251_A_AT | ligand of numb-protein X 1 | [LNX1](http://www.gene.ucl.ac.uk/cgi-bin/nomenclature/searchgenes.pl?field=symbol&anchor=equals&symbol_search=Search&number=100&format=html&sortby=symbol&match=LNX1) |  |  | **2.07** |
| 1450269_A_AT | phosphofructokinase, liver, B-type | [PFKL](http://www.gene.ucl.ac.uk/cgi-bin/nomenclature/searchgenes.pl?field=symbol&anchor=equals&symbol_search=Search&number=100&format=html&sortby=symbol&match=PFKL) |  | [171860](http://www.ncbi.nlm.nih.gov/entrez/dispomim.cgi?id=171860) | **1.37** |
| 1450291_S_AT | gb:NM_022429.1 /DB_XREF=gi:11967954 /GEN=Ms4a9 /FE |  |  |  | **-3.18** |
| 1450295_S_AT | poliovirus receptor | [PVR](http://www.gene.ucl.ac.uk/cgi-bin/nomenclature/searchgenes.pl?field=symbol&anchor=equals&symbol_search=Search&number=100&format=html&sortby=symbol&match=PVR) |  | [173850](http://www.ncbi.nlm.nih.gov/entrez/dispomim.cgi?id=173850) | **-2.07** |
| 1450318_A_AT | purinergic receptor P2Y, G-protein coupled 2 | [P2RY2](http://www.gene.ucl.ac.uk/cgi-bin/nomenclature/searchgenes.pl?field=symbol&anchor=equals&symbol_search=Search&number=100&format=html&sortby=symbol&match=P2RY2) |  | [600041](http://www.ncbi.nlm.nih.gov/entrez/dispomim.cgi?id=600041) | **1.83** |
| 1450333_A_AT | GATA binding protein 2 | [GATA2](http://www.gene.ucl.ac.uk/cgi-bin/nomenclature/searchgenes.pl?field=symbol&anchor=equals&symbol_search=Search&number=100&format=html&sortby=symbol&match=GATA2) |  | [137295](http://www.ncbi.nlm.nih.gov/entrez/dispomim.cgi?id=137295) | **1.5** |
| 1450344_A_AT | prostaglandin E receptor 3 (subtype EP3) | [PTGER3](http://www.gene.ucl.ac.uk/cgi-bin/nomenclature/searchgenes.pl?field=symbol&anchor=equals&symbol_search=Search&number=100&format=html&sortby=symbol&match=PTGER3) |  | [176806](http://www.ncbi.nlm.nih.gov/entrez/dispomim.cgi?id=176806) | **1.69** |
| 1450350_A_AT | Jun dimerization protein 2 | [JUNDM2](http://www.gene.ucl.ac.uk/cgi-bin/nomenclature/searchgenes.pl?field=symbol&anchor=equals&symbol_search=Search&number=100&format=html&sortby=symbol&match=JUNDM2) |  |  | **-1.48** |
| 1450355_A_AT | capping protein (actin filament), gelsolin-like | [CAPG](http://www.gene.ucl.ac.uk/cgi-bin/nomenclature/searchgenes.pl?field=symbol&anchor=equals&symbol_search=Search&number=100&format=html&sortby=symbol&match=CAPG) |  | [153615](http://www.ncbi.nlm.nih.gov/entrez/dispomim.cgi?id=153615) | **-3.14** |
| 1450372_A_AT | ribosomal protein L18 | [RPL18](http://www.gene.ucl.ac.uk/cgi-bin/nomenclature/searchgenes.pl?field=symbol&anchor=equals&symbol_search=Search&number=100&format=html&sortby=symbol&match=RPL18) |  | [604179](http://www.ncbi.nlm.nih.gov/entrez/dispomim.cgi?id=604179) | **-1.27** |
| 1450376_AT | Max interacting protein 1 | [MXI1](http://www.gene.ucl.ac.uk/cgi-bin/nomenclature/searchgenes.pl?field=symbol&anchor=equals&symbol_search=Search&number=100&format=html&sortby=symbol&match=MXI1) |  | [600020](http://www.ncbi.nlm.nih.gov/entrez/dispomim.cgi?id=600020) | **1.39** |
| 1450379_AT | moesin | [MSN](http://www.gene.ucl.ac.uk/cgi-bin/nomenclature/searchgenes.pl?field=symbol&anchor=equals&symbol_search=Search&number=100&format=html&sortby=symbol&match=MSN) |  | [309845](http://www.ncbi.nlm.nih.gov/entrez/dispomim.cgi?id=309845) | **-1.33** |
| 1450380_AT | expressed sequence AU040950 | [AU040950](http://www.gene.ucl.ac.uk/cgi-bin/nomenclature/searchgenes.pl?field=symbol&anchor=equals&symbol_search=Search&number=100&format=html&sortby=symbol&match=AU040950) |  |  | **1.35** |
| 1450382_AT | neurofibromatosis 2 | [NF2](http://www.gene.ucl.ac.uk/cgi-bin/nomenclature/searchgenes.pl?field=symbol&anchor=equals&symbol_search=Search&number=100&format=html&sortby=symbol&match=NF2) |  | [607379](http://www.ncbi.nlm.nih.gov/entrez/dispomim.cgi?id=607379) | **1.26** |
| 1450387_S_AT | gb:NM_009647.1 /DB_XREF=gi:6753021 /GEN=Ak4 /FEA=F |  |  |  | **2.72** |
| 1450391_A_AT | monoglyceride lipase | [MGLL](http://www.gene.ucl.ac.uk/cgi-bin/nomenclature/searchgenes.pl?field=symbol&anchor=equals&symbol_search=Search&number=100&format=html&sortby=symbol&match=MGLL) |  |  | **2** |
| 1450395_AT | solute carrier family 22 (organic cation transporter), member 5 | [SLC22A5](http://www.gene.ucl.ac.uk/cgi-bin/nomenclature/searchgenes.pl?field=symbol&anchor=equals&symbol_search=Search&number=100&format=html&sortby=symbol&match=SLC22A5) |  | [603377](http://www.ncbi.nlm.nih.gov/entrez/dispomim.cgi?id=603377) | **1.49** |
| 1450397_AT | microtubule-associated protein 1 B | [MTAP1B](http://www.gene.ucl.ac.uk/cgi-bin/nomenclature/searchgenes.pl?field=symbol&anchor=equals&symbol_search=Search&number=100&format=html&sortby=symbol&match=MTAP1B) |  |  | **-2.66** |
| 1450429_AT | calpain 6 | [CAPN6](http://www.gene.ucl.ac.uk/cgi-bin/nomenclature/searchgenes.pl?field=symbol&anchor=equals&symbol_search=Search&number=100&format=html&sortby=symbol&match=CAPN6) |  | [300146](http://www.ncbi.nlm.nih.gov/entrez/dispomim.cgi?id=300146) | **-2.68** |
| 1450430_AT | mannose receptor, C type 1 | [MRC1](http://www.gene.ucl.ac.uk/cgi-bin/nomenclature/searchgenes.pl?field=symbol&anchor=equals&symbol_search=Search&number=100&format=html&sortby=symbol&match=MRC1) |  | [153618](http://www.ncbi.nlm.nih.gov/entrez/dispomim.cgi?id=153618) | **-1.62** |
| 1450437_A_AT | neural cell adhesion molecule 1 | [NCAM1](http://www.gene.ucl.ac.uk/cgi-bin/nomenclature/searchgenes.pl?field=symbol&anchor=equals&symbol_search=Search&number=100&format=html&sortby=symbol&match=NCAM1) |  | [116930](http://www.ncbi.nlm.nih.gov/entrez/dispomim.cgi?id=116930) | **-5.82** |
| 1450449_A_AT | RIKEN cDNA 2900002H16 gene | [2900002H16RIK](http://www.gene.ucl.ac.uk/cgi-bin/nomenclature/searchgenes.pl?field=symbol&anchor=equals&symbol_search=Search&number=100&format=html&sortby=symbol&match=2900002H16RIK) |  |  | **1.62** |
| 1450490_AT | potassium voltage-gated channel, shaker-related subfamily, member 7 | [KCNA7](http://www.gene.ucl.ac.uk/cgi-bin/nomenclature/searchgenes.pl?field=symbol&anchor=equals&symbol_search=Search&number=100&format=html&sortby=symbol&match=KCNA7) |  | [176268](http://www.ncbi.nlm.nih.gov/entrez/dispomim.cgi?id=176268) | **1.93** |
| 1450494_X_AT | CEA-related cell adhesion molecule 1 | [CEACAM1](http://www.gene.ucl.ac.uk/cgi-bin/nomenclature/searchgenes.pl?field=symbol&anchor=equals&symbol_search=Search&number=100&format=html&sortby=symbol&match=CEACAM1) |  | [109770](http://www.ncbi.nlm.nih.gov/entrez/dispomim.cgi?id=109770) | **1.94** |
| 1450515_AT | potassium inwardly rectifying channel, subfamily J, member 11 | [KCNJ11](http://www.gene.ucl.ac.uk/cgi-bin/nomenclature/searchgenes.pl?field=symbol&anchor=equals&symbol_search=Search&number=100&format=html&sortby=symbol&match=KCNJ11) |  | [600937](http://www.ncbi.nlm.nih.gov/entrez/dispomim.cgi?id=600937) | **1.66** |
| 1450541_AT | plasmacytoma variant translocation 1 | [PVT1](http://www.gene.ucl.ac.uk/cgi-bin/nomenclature/searchgenes.pl?field=symbol&anchor=equals&symbol_search=Search&number=100&format=html&sortby=symbol&match=PVT1) |  |  | **-1.56** |
| 1450556_AT | spectrin beta 1 | [SPNB1](http://www.gene.ucl.ac.uk/cgi-bin/nomenclature/searchgenes.pl?field=symbol&anchor=equals&symbol_search=Search&number=100&format=html&sortby=symbol&match=SPNB1) |  |  | **1.83** |
| 1450557_AT | sodium channel, voltage-gated, type IV, alpha polypeptide | [SCN4A](http://www.gene.ucl.ac.uk/cgi-bin/nomenclature/searchgenes.pl?field=symbol&anchor=equals&symbol_search=Search&number=100&format=html&sortby=symbol&match=SCN4A) |  | [603967](http://www.ncbi.nlm.nih.gov/entrez/dispomim.cgi?id=603967) | **1.74** |
| 1450625_AT | procollagen, type V, alpha 2 | [COL5A2](http://www.gene.ucl.ac.uk/cgi-bin/nomenclature/searchgenes.pl?field=symbol&anchor=equals&symbol_search=Search&number=100&format=html&sortby=symbol&match=COL5A2) |  | [120190](http://www.ncbi.nlm.nih.gov/entrez/dispomim.cgi?id=120190) | **-5.41** |
| 1450626_AT | mannosidase, beta A, lysosomal | [MANBA](http://www.gene.ucl.ac.uk/cgi-bin/nomenclature/searchgenes.pl?field=symbol&anchor=equals&symbol_search=Search&number=100&format=html&sortby=symbol&match=MANBA) |  | [248510](http://www.ncbi.nlm.nih.gov/entrez/dispomim.cgi?id=248510) | **-1.27** |
| 1450627_AT | progressive ankylosis | [ANK](http://www.gene.ucl.ac.uk/cgi-bin/nomenclature/searchgenes.pl?field=symbol&anchor=equals&symbol_search=Search&number=100&format=html&sortby=symbol&match=ANK) |  |  | **1.46** |
| 1450634_AT | ATPase, H+ transporting, V1 subunit A, isoform 1 | [ATP6V1A1](http://www.gene.ucl.ac.uk/cgi-bin/nomenclature/searchgenes.pl?field=symbol&anchor=equals&symbol_search=Search&number=100&format=html&sortby=symbol&match=ATP6V1A1) |  |  | **-1.35** |
| 1450639_AT | solute carrier family 28 (sodium-coupled nucleoside transporter), member 2 | [SLC28A2](http://www.gene.ucl.ac.uk/cgi-bin/nomenclature/searchgenes.pl?field=symbol&anchor=equals&symbol_search=Search&number=100&format=html&sortby=symbol&match=SLC28A2) |  | [606208](http://www.ncbi.nlm.nih.gov/entrez/dispomim.cgi?id=606208) | **2.06** |
| 1450641_AT | vimentin | [VIM](http://www.gene.ucl.ac.uk/cgi-bin/nomenclature/searchgenes.pl?field=symbol&anchor=equals&symbol_search=Search&number=100&format=html&sortby=symbol&match=VIM) |  | [193060](http://www.ncbi.nlm.nih.gov/entrez/dispomim.cgi?id=193060) | **-1.82** |
| 1450643_S_AT | acyl-CoA synthetase long-chain family member 1 | [ACSL1](http://www.gene.ucl.ac.uk/cgi-bin/nomenclature/searchgenes.pl?field=symbol&anchor=equals&symbol_search=Search&number=100&format=html&sortby=symbol&match=ACSL1) |  | [152425](http://www.ncbi.nlm.nih.gov/entrez/dispomim.cgi?id=152425) | **1.93** |
| 1450648_S_AT | histocompatibility 2, class II antigen A, beta 1 | [RMCS1](http://www.gene.ucl.ac.uk/cgi-bin/nomenclature/searchgenes.pl?field=symbol&anchor=equals&symbol_search=Search&number=100&format=html&sortby=symbol&match=RMCS1) |  |  | **-1.74** |
| 1450649_AT | gb:NM_025277.1 /DB_XREF=gi:13384615 /GEN=Gng10 /FE |  |  |  | **-1.4** |
| 1450652_AT | cathepsin K | [CTSK](http://www.gene.ucl.ac.uk/cgi-bin/nomenclature/searchgenes.pl?field=symbol&anchor=equals&symbol_search=Search&number=100&format=html&sortby=symbol&match=CTSK) |  | [601105](http://www.ncbi.nlm.nih.gov/entrez/dispomim.cgi?id=601105) | **-2.64** |
| 1450656_AT | guanine nucleotide binding protein, alpha 13 | [GNA13](http://www.gene.ucl.ac.uk/cgi-bin/nomenclature/searchgenes.pl?field=symbol&anchor=equals&symbol_search=Search&number=100&format=html&sortby=symbol&match=GNA13) |  | [604406](http://www.ncbi.nlm.nih.gov/entrez/dispomim.cgi?id=604406) | **-1.32** |
| 1450660_AT | 6-pyruvoyl-tetrahydropterin synthase | [PTS](http://www.gene.ucl.ac.uk/cgi-bin/nomenclature/searchgenes.pl?field=symbol&anchor=equals&symbol_search=Search&number=100&format=html&sortby=symbol&match=PTS) |  | [261640](http://www.ncbi.nlm.nih.gov/entrez/dispomim.cgi?id=261640) | **1.36** |
| 1450667_A_AT | citrate synthase | [CS](http://www.gene.ucl.ac.uk/cgi-bin/nomenclature/searchgenes.pl?field=symbol&anchor=equals&symbol_search=Search&number=100&format=html&sortby=symbol&match=CS) |  | [118950](http://www.ncbi.nlm.nih.gov/entrez/dispomim.cgi?id=118950) | **1.32** |
| 1450676_AT | transcription elongation factor B (SIII), polypeptide 3 | [TCEB3](http://www.gene.ucl.ac.uk/cgi-bin/nomenclature/searchgenes.pl?field=symbol&anchor=equals&symbol_search=Search&number=100&format=html&sortby=symbol&match=TCEB3) |  | [600786](http://www.ncbi.nlm.nih.gov/entrez/dispomim.cgi?id=600786) | **1.53** |
| 1450678_AT | gb:NM_008404.1 /DB_XREF=gi:6680489 /GEN=Itgb2 /FEA |  |  |  | **-2.95** |
| 1450679_AT | RIKEN cDNA 5730470L24 gene | [5730470L24RIK](http://www.gene.ucl.ac.uk/cgi-bin/nomenclature/searchgenes.pl?field=symbol&anchor=equals&symbol_search=Search&number=100&format=html&sortby=symbol&match=5730470L24RIK) |  |  | **-1.68** |
| 1450690_AT | RAN binding protein 2 | [RANBP2](http://www.gene.ucl.ac.uk/cgi-bin/nomenclature/searchgenes.pl?field=symbol&anchor=equals&symbol_search=Search&number=100&format=html&sortby=symbol&match=RANBP2) |  |  | **-1.2** |
| 1450694_AT | FK506 binding protein 2 | [FKBP2](http://www.gene.ucl.ac.uk/cgi-bin/nomenclature/searchgenes.pl?field=symbol&anchor=equals&symbol_search=Search&number=100&format=html&sortby=symbol&match=FKBP2) |  | [186946](http://www.ncbi.nlm.nih.gov/entrez/dispomim.cgi?id=186946) | **-1.54** |
| 1450699_AT | selenium binding protein 1 | [SELENBP1](http://www.gene.ucl.ac.uk/cgi-bin/nomenclature/searchgenes.pl?field=symbol&anchor=equals&symbol_search=Search&number=100&format=html&sortby=symbol&match=SELENBP1) |  | [604188](http://www.ncbi.nlm.nih.gov/entrez/dispomim.cgi?id=604188) | **2.1** |
| 1450707_AT | double C2, gamma | [DOC2G](http://www.gene.ucl.ac.uk/cgi-bin/nomenclature/searchgenes.pl?field=symbol&anchor=equals&symbol_search=Search&number=100&format=html&sortby=symbol&match=DOC2G) |  |  | **1.61** |
| 1450714_AT | ornithine decarboxylase antizyme inhibitor | [OAZIN](http://www.gene.ucl.ac.uk/cgi-bin/nomenclature/searchgenes.pl?field=symbol&anchor=equals&symbol_search=Search&number=100&format=html&sortby=symbol&match=OAZIN) |  | [607909](http://www.ncbi.nlm.nih.gov/entrez/dispomim.cgi?id=607909) | **-1.77** |
| 1450722_AT | nucleoporin 50 | [NUP50](http://www.gene.ucl.ac.uk/cgi-bin/nomenclature/searchgenes.pl?field=symbol&anchor=equals&symbol_search=Search&number=100&format=html&sortby=symbol&match=NUP50) |  | [604646](http://www.ncbi.nlm.nih.gov/entrez/dispomim.cgi?id=604646) | **-1.46** |
| 1450727_A_AT | polymerase (DNA-directed), delta interacting protein 2 | [POLDIP2](http://www.gene.ucl.ac.uk/cgi-bin/nomenclature/searchgenes.pl?field=symbol&anchor=equals&symbol_search=Search&number=100&format=html&sortby=symbol&match=POLDIP2) |  |  | **1.28** |
| 1450729_AT | heparan sulfate 2-O-sulfotransferase 1 | [HS2ST1](http://www.gene.ucl.ac.uk/cgi-bin/nomenclature/searchgenes.pl?field=symbol&anchor=equals&symbol_search=Search&number=100&format=html&sortby=symbol&match=HS2ST1) |  | [604844](http://www.ncbi.nlm.nih.gov/entrez/dispomim.cgi?id=604844) | **-1.59** |
| 1450732_A_AT | bicaudal D homolog 2 (Drosophila) | [BICD2](http://www.gene.ucl.ac.uk/cgi-bin/nomenclature/searchgenes.pl?field=symbol&anchor=equals&symbol_search=Search&number=100&format=html&sortby=symbol&match=BICD2) |  |  | **-1.23** |
| 1450738_AT | gb:NM_016705.1 /DB_XREF=gi:7710045 /GEN=Kif21a /FE |  |  |  | **1.64** |
| 1450747_AT | kelch-like ECH-associated protein 1 | [KEAP1](http://www.gene.ucl.ac.uk/cgi-bin/nomenclature/searchgenes.pl?field=symbol&anchor=equals&symbol_search=Search&number=100&format=html&sortby=symbol&match=KEAP1) |  | [606016](http://www.ncbi.nlm.nih.gov/entrez/dispomim.cgi?id=606016) | **1.44** |
| 1450756_S_AT | cullin 3 | [CUL3](http://www.gene.ucl.ac.uk/cgi-bin/nomenclature/searchgenes.pl?field=symbol&anchor=equals&symbol_search=Search&number=100&format=html&sortby=symbol&match=CUL3) |  | [603136](http://www.ncbi.nlm.nih.gov/entrez/dispomim.cgi?id=603136) | **1.63** |
| 1450759_AT | gb:NM_007556.1 /DB_XREF=gi:6680797 /GEN=Bmp6 /FEA= |  |  |  | **4.13** |
| 1450767_AT | neural precursor cell expressed, developmentally down-regulated gene 9 | [NEDD9](http://www.gene.ucl.ac.uk/cgi-bin/nomenclature/searchgenes.pl?field=symbol&anchor=equals&symbol_search=Search&number=100&format=html&sortby=symbol&match=NEDD9) |  | [602265](http://www.ncbi.nlm.nih.gov/entrez/dispomim.cgi?id=602265) | **1.47** |
| 1450768_AT | discs, large homolog 1 (Drosophila) | [DLGH1](http://www.gene.ucl.ac.uk/cgi-bin/nomenclature/searchgenes.pl?field=symbol&anchor=equals&symbol_search=Search&number=100&format=html&sortby=symbol&match=DLGH1) |  |  | **1.6** |
| 1450770_AT | RIKEN cDNA 3632451O06 gene | [3632451O06RIK](http://www.gene.ucl.ac.uk/cgi-bin/nomenclature/searchgenes.pl?field=symbol&anchor=equals&symbol_search=Search&number=100&format=html&sortby=symbol&match=3632451O06RIK) |  |  | **-3.81** |
| 1450776_AT | expressed sequence AU041707 | [AU041707](http://www.gene.ucl.ac.uk/cgi-bin/nomenclature/searchgenes.pl?field=symbol&anchor=equals&symbol_search=Search&number=100&format=html&sortby=symbol&match=AU041707) |  |  | **1.41** |
| 1450780_S_AT | high mobility group AT-hook 2 | [HMGA2](http://www.gene.ucl.ac.uk/cgi-bin/nomenclature/searchgenes.pl?field=symbol&anchor=equals&symbol_search=Search&number=100&format=html&sortby=symbol&match=HMGA2) |  | [600698](http://www.ncbi.nlm.nih.gov/entrez/dispomim.cgi?id=600698) | **-1.57** |
| 1450783_AT | interferon-induced protein with tetratricopeptide repeats 1 | [IFIT1](http://www.gene.ucl.ac.uk/cgi-bin/nomenclature/searchgenes.pl?field=symbol&anchor=equals&symbol_search=Search&number=100&format=html&sortby=symbol&match=IFIT1) |  | [147690](http://www.ncbi.nlm.nih.gov/entrez/dispomim.cgi?id=147690) | **-1.75** |
| 1450784_AT | gb:NM_016678.1 /DB_XREF=gi:7710063 /GEN=Reck /FEA= |  |  |  | **1.38** |
| 1450792_AT | TYRO protein tyrosine kinase binding protein | [TYROBP](http://www.gene.ucl.ac.uk/cgi-bin/nomenclature/searchgenes.pl?field=symbol&anchor=equals&symbol_search=Search&number=100&format=html&sortby=symbol&match=TYROBP) |  | [604142](http://www.ncbi.nlm.nih.gov/entrez/dispomim.cgi?id=604142) | **-6.09** |
| 1450813_A_AT | troponin I, skeletal, slow 1 | [TNNI1](http://www.gene.ucl.ac.uk/cgi-bin/nomenclature/searchgenes.pl?field=symbol&anchor=equals&symbol_search=Search&number=100&format=html&sortby=symbol&match=TNNI1) |  | [191042](http://www.ncbi.nlm.nih.gov/entrez/dispomim.cgi?id=191042) | **-1.9** |
| 1450815_S_AT | RIKEN cDNA 2410018M08 gene | [CHCHD2](http://www.gene.ucl.ac.uk/cgi-bin/nomenclature/searchgenes.pl?field=symbol&anchor=equals&symbol_search=Search&number=100&format=html&sortby=symbol&match=CHCHD2) |  |  | **1.24** |
| 1450840_A_AT | ribosomal protein L39 | [RPL39](http://www.gene.ucl.ac.uk/cgi-bin/nomenclature/searchgenes.pl?field=symbol&anchor=equals&symbol_search=Search&number=100&format=html&sortby=symbol&match=RPL39) |  | [601904](http://www.ncbi.nlm.nih.gov/entrez/dispomim.cgi?id=601904) | **-1.26** |
| 1450841_AT | intergral membrane protein 1 | [ITM1](http://www.gene.ucl.ac.uk/cgi-bin/nomenclature/searchgenes.pl?field=symbol&anchor=equals&symbol_search=Search&number=100&format=html&sortby=symbol&match=ITM1) |  | [601134](http://www.ncbi.nlm.nih.gov/entrez/dispomim.cgi?id=601134) | **-1.53** |
| 1450843_A_AT | serine (or cysteine) proteinase inhibitor, clade H, member 1 | [SERPINH1](http://www.gene.ucl.ac.uk/cgi-bin/nomenclature/searchgenes.pl?field=symbol&anchor=equals&symbol_search=Search&number=100&format=html&sortby=symbol&match=SERPINH1) |  | [600943](http://www.ncbi.nlm.nih.gov/entrez/dispomim.cgi?id=600943) | **-2.05** |
| 1450845_A_AT | basic leucine zipper and W2 domains 1 | [BZW1](http://www.gene.ucl.ac.uk/cgi-bin/nomenclature/searchgenes.pl?field=symbol&anchor=equals&symbol_search=Search&number=100&format=html&sortby=symbol&match=BZW1) |  |  | **-1.44** |
| 1450846_AT | basic leucine zipper and W2 domains 1 | [BZW1](http://www.gene.ucl.ac.uk/cgi-bin/nomenclature/searchgenes.pl?field=symbol&anchor=equals&symbol_search=Search&number=100&format=html&sortby=symbol&match=BZW1) |  |  | **-1.38** |
| 1450848_AT | death associated protein 3 | [DAP3](http://www.gene.ucl.ac.uk/cgi-bin/nomenclature/searchgenes.pl?field=symbol&anchor=equals&symbol_search=Search&number=100&format=html&sortby=symbol&match=DAP3) |  | [602074](http://www.ncbi.nlm.nih.gov/entrez/dispomim.cgi?id=602074) | **1.23** |
| 1450851_AT | WD repeat domain 1 | [WDR1](http://www.gene.ucl.ac.uk/cgi-bin/nomenclature/searchgenes.pl?field=symbol&anchor=equals&symbol_search=Search&number=100&format=html&sortby=symbol&match=WDR1) |  | [604734](http://www.ncbi.nlm.nih.gov/entrez/dispomim.cgi?id=604734) | **-1.26** |
| 1450857_A_AT | procollagen, type I, alpha 2 | [COL1A2](http://www.gene.ucl.ac.uk/cgi-bin/nomenclature/searchgenes.pl?field=symbol&anchor=equals&symbol_search=Search&number=100&format=html&sortby=symbol&match=COL1A2) |  | [120160](http://www.ncbi.nlm.nih.gov/entrez/dispomim.cgi?id=120160) | **-2.12** |
| 1450863_A_AT | double cortin and calcium/calmodulin-dependent protein kinase-like 1 | [DCAMKL1](http://www.gene.ucl.ac.uk/cgi-bin/nomenclature/searchgenes.pl?field=symbol&anchor=equals&symbol_search=Search&number=100&format=html&sortby=symbol&match=DCAMKL1) |  | [604742](http://www.ncbi.nlm.nih.gov/entrez/dispomim.cgi?id=604742) | **-2.17** |
| 1450865_S_AT | mitochondrial ribosomal protein S24 | [MRPS24](http://www.gene.ucl.ac.uk/cgi-bin/nomenclature/searchgenes.pl?field=symbol&anchor=equals&symbol_search=Search&number=100&format=html&sortby=symbol&match=MRPS24) |  |  | **1.33** |
| 1450869_AT | fibroblast growth factor 1 | [FGF1](http://www.gene.ucl.ac.uk/cgi-bin/nomenclature/searchgenes.pl?field=symbol&anchor=equals&symbol_search=Search&number=100&format=html&sortby=symbol&match=FGF1) |  | [131220](http://www.ncbi.nlm.nih.gov/entrez/dispomim.cgi?id=131220) | **1.79** |
| 1450872_S_AT | lysosomal acid lipase 1 | [LIP1](http://www.gene.ucl.ac.uk/cgi-bin/nomenclature/searchgenes.pl?field=symbol&anchor=equals&symbol_search=Search&number=100&format=html&sortby=symbol&match=LIP1) |  |  | **-1.71** |
| 1450878_AT | sorcin | [SRI](http://www.gene.ucl.ac.uk/cgi-bin/nomenclature/searchgenes.pl?field=symbol&anchor=equals&symbol_search=Search&number=100&format=html&sortby=symbol&match=SRI) |  | [182520](http://www.ncbi.nlm.nih.gov/entrez/dispomim.cgi?id=182520) | **-1.28** |
| 1450880_AT | mitochondrial ribosomal protein L16 | [MRPL16](http://www.gene.ucl.ac.uk/cgi-bin/nomenclature/searchgenes.pl?field=symbol&anchor=equals&symbol_search=Search&number=100&format=html&sortby=symbol&match=MRPL16) |  |  | **1.37** |
| 1450881_S_AT | transmembrane 7 superfamily member 1 | [TM7SF1](http://www.gene.ucl.ac.uk/cgi-bin/nomenclature/searchgenes.pl?field=symbol&anchor=equals&symbol_search=Search&number=100&format=html&sortby=symbol&match=TM7SF1) |  | [604658](http://www.ncbi.nlm.nih.gov/entrez/dispomim.cgi?id=604658) | **-1.45** |
| 1450882_S_AT | transmembrane 7 superfamily member 1 | [TM7SF1](http://www.gene.ucl.ac.uk/cgi-bin/nomenclature/searchgenes.pl?field=symbol&anchor=equals&symbol_search=Search&number=100&format=html&sortby=symbol&match=TM7SF1) |  | [604658](http://www.ncbi.nlm.nih.gov/entrez/dispomim.cgi?id=604658) | **-1.55** |
| 1450883_A_AT | CD36 antigen | [CD36](http://www.gene.ucl.ac.uk/cgi-bin/nomenclature/searchgenes.pl?field=symbol&anchor=equals&symbol_search=Search&number=100&format=html&sortby=symbol&match=CD36) |  | [173510](http://www.ncbi.nlm.nih.gov/entrez/dispomim.cgi?id=173510) | **1.84** |
| 1450884_AT | CD36 antigen | [CD36](http://www.gene.ucl.ac.uk/cgi-bin/nomenclature/searchgenes.pl?field=symbol&anchor=equals&symbol_search=Search&number=100&format=html&sortby=symbol&match=CD36) |  | [173510](http://www.ncbi.nlm.nih.gov/entrez/dispomim.cgi?id=173510) | **2.37** |
| 1450897_AT | expressed sequence AU014947 | [AU014947](http://www.gene.ucl.ac.uk/cgi-bin/nomenclature/searchgenes.pl?field=symbol&anchor=equals&symbol_search=Search&number=100&format=html&sortby=symbol&match=AU014947) |  |  | **1.27** |
| 1450899_AT | neural precursor cell expressed, developmentally down-regulated gene 1 | [NEDD1](http://www.gene.ucl.ac.uk/cgi-bin/nomenclature/searchgenes.pl?field=symbol&anchor=equals&symbol_search=Search&number=100&format=html&sortby=symbol&match=NEDD1) |  | [600372](http://www.ncbi.nlm.nih.gov/entrez/dispomim.cgi?id=600372) | **1.53** |
| 1450904_AT | RIKEN cDNA 0610041E09 gene | [0610041E09RIK](http://www.gene.ucl.ac.uk/cgi-bin/nomenclature/searchgenes.pl?field=symbol&anchor=equals&symbol_search=Search&number=100&format=html&sortby=symbol&match=0610041E09RIK) |  |  | **-1.56** |
| 1450905_AT | plexin C1 | [PLXNC1](http://www.gene.ucl.ac.uk/cgi-bin/nomenclature/searchgenes.pl?field=symbol&anchor=equals&symbol_search=Search&number=100&format=html&sortby=symbol&match=PLXNC1) |  | [604259](http://www.ncbi.nlm.nih.gov/entrez/dispomim.cgi?id=604259) | **-1.97** |
| 1450908_AT | eukaryotic translation initiation factor 4E | [EIF4E](http://www.gene.ucl.ac.uk/cgi-bin/nomenclature/searchgenes.pl?field=symbol&anchor=equals&symbol_search=Search&number=100&format=html&sortby=symbol&match=EIF4E) |  | [133440](http://www.ncbi.nlm.nih.gov/entrez/dispomim.cgi?id=133440) | **1.29** |
| 1450909_AT | eukaryotic translation initiation factor 4E | [EIF4E](http://www.gene.ucl.ac.uk/cgi-bin/nomenclature/searchgenes.pl?field=symbol&anchor=equals&symbol_search=Search&number=100&format=html&sortby=symbol&match=EIF4E) |  | [133440](http://www.ncbi.nlm.nih.gov/entrez/dispomim.cgi?id=133440) | **1.54** |
| 1450911_AT | peptidylprolyl isomerase B | [PPIB](http://www.gene.ucl.ac.uk/cgi-bin/nomenclature/searchgenes.pl?field=symbol&anchor=equals&symbol_search=Search&number=100&format=html&sortby=symbol&match=PPIB) |  | [123841](http://www.ncbi.nlm.nih.gov/entrez/dispomim.cgi?id=123841) | **-1.72** |
| 1450914_AT | protein phosphatase 1, regulatory (inhibitor) subunit 14B | [PPP1R14B](http://www.gene.ucl.ac.uk/cgi-bin/nomenclature/searchgenes.pl?field=symbol&anchor=equals&symbol_search=Search&number=100&format=html&sortby=symbol&match=PPP1R14B) |  | [601140](http://www.ncbi.nlm.nih.gov/entrez/dispomim.cgi?id=601140) | **-1.34** |
| 1450917_AT | myomesin 2 | [MYOM2](http://www.gene.ucl.ac.uk/cgi-bin/nomenclature/searchgenes.pl?field=symbol&anchor=equals&symbol_search=Search&number=100&format=html&sortby=symbol&match=MYOM2) |  | [603509](http://www.ncbi.nlm.nih.gov/entrez/dispomim.cgi?id=603509) | **-1.7** |
| 1450919_AT | membrane protein, palmitoylated | [MPP1](http://www.gene.ucl.ac.uk/cgi-bin/nomenclature/searchgenes.pl?field=symbol&anchor=equals&symbol_search=Search&number=100&format=html&sortby=symbol&match=MPP1) |  | [305360](http://www.ncbi.nlm.nih.gov/entrez/dispomim.cgi?id=305360) | **-1.37** |
| 1450923_AT | transforming growth factor, beta 2 | [TGFB2](http://www.gene.ucl.ac.uk/cgi-bin/nomenclature/searchgenes.pl?field=symbol&anchor=equals&symbol_search=Search&number=100&format=html&sortby=symbol&match=TGFB2) |  | [190220](http://www.ncbi.nlm.nih.gov/entrez/dispomim.cgi?id=190220) | **-1.47** |
| 1450925_A_AT | ribosomal protein S27-like | [RPS27L](http://www.gene.ucl.ac.uk/cgi-bin/nomenclature/searchgenes.pl?field=symbol&anchor=equals&symbol_search=Search&number=100&format=html&sortby=symbol&match=RPS27L) |  |  | **-1.3** |
| 1450932_S_AT | dedicator of cytokinesis 9 | [DOCK9](http://www.gene.ucl.ac.uk/cgi-bin/nomenclature/searchgenes.pl?field=symbol&anchor=equals&symbol_search=Search&number=100&format=html&sortby=symbol&match=DOCK9) |  | [607325](http://www.ncbi.nlm.nih.gov/entrez/dispomim.cgi?id=607325) | **1.79** |
| 1450941_AT | gb:AV227603 /DB_XREF=gi:16385465 /DB_XREF=AV227603 |  |  |  | **-1.36** |
| 1450958_AT | transmembrane 4 superfamily member 1 | [TM4SF1](http://www.gene.ucl.ac.uk/cgi-bin/nomenclature/searchgenes.pl?field=symbol&anchor=equals&symbol_search=Search&number=100&format=html&sortby=symbol&match=TM4SF1) |  | [191155](http://www.ncbi.nlm.nih.gov/entrez/dispomim.cgi?id=191155) | **1.29** |
| 1450961_A_AT | transcription elongation factor A (SII)-like 5 | [TCEAL5](http://www.gene.ucl.ac.uk/cgi-bin/nomenclature/searchgenes.pl?field=symbol&anchor=equals&symbol_search=Search&number=100&format=html&sortby=symbol&match=TCEAL5) |  |  | **-10.39** |
| 1450964_A_AT | oxysterol binding protein-like 9 | [OSBPL9](http://www.gene.ucl.ac.uk/cgi-bin/nomenclature/searchgenes.pl?field=symbol&anchor=equals&symbol_search=Search&number=100&format=html&sortby=symbol&match=OSBPL9) |  | [606737](http://www.ncbi.nlm.nih.gov/entrez/dispomim.cgi?id=606737) | **1.21** |
| 1450968_AT | ubiquinol-cytochrome c reductase, Rieske iron-sulfur polypeptide 1 | [UQCRFS1](http://www.gene.ucl.ac.uk/cgi-bin/nomenclature/searchgenes.pl?field=symbol&anchor=equals&symbol_search=Search&number=100&format=html&sortby=symbol&match=UQCRFS1) |  | [191327](http://www.ncbi.nlm.nih.gov/entrez/dispomim.cgi?id=191327) | **1.28** |
| 1450969_AT | propionyl Coenzyme A carboxylase, beta polypeptide | [PCCB](http://www.gene.ucl.ac.uk/cgi-bin/nomenclature/searchgenes.pl?field=symbol&anchor=equals&symbol_search=Search&number=100&format=html&sortby=symbol&match=PCCB) |  | [232050](http://www.ncbi.nlm.nih.gov/entrez/dispomim.cgi?id=232050) | **1.45** |
| 1450970_AT | glutamate oxaloacetate transaminase 1, soluble | [GOT1](http://www.gene.ucl.ac.uk/cgi-bin/nomenclature/searchgenes.pl?field=symbol&anchor=equals&symbol_search=Search&number=100&format=html&sortby=symbol&match=GOT1) |  | [138180](http://www.ncbi.nlm.nih.gov/entrez/dispomim.cgi?id=138180) | **1.48** |
| 1450976_AT | N-myc downstream regulated gene 1 | [NDRG1](http://www.gene.ucl.ac.uk/cgi-bin/nomenclature/searchgenes.pl?field=symbol&anchor=equals&symbol_search=Search&number=100&format=html&sortby=symbol&match=NDRG1) |  | [605262](http://www.ncbi.nlm.nih.gov/entrez/dispomim.cgi?id=605262) | **1.36** |
| 1450986_AT | nucleolar protein 5 | [NOL5](http://www.gene.ucl.ac.uk/cgi-bin/nomenclature/searchgenes.pl?field=symbol&anchor=equals&symbol_search=Search&number=100&format=html&sortby=symbol&match=NOL5) |  |  | **-1.76** |
| 1450990_AT | glypican 3 | [GPC3](http://www.gene.ucl.ac.uk/cgi-bin/nomenclature/searchgenes.pl?field=symbol&anchor=equals&symbol_search=Search&number=100&format=html&sortby=symbol&match=GPC3) |  | [300037](http://www.ncbi.nlm.nih.gov/entrez/dispomim.cgi?id=300037) | **1.66** |
| 1450994_AT | Rho-associated coiled-coil forming kinase 1 | [ROCK1](http://www.gene.ucl.ac.uk/cgi-bin/nomenclature/searchgenes.pl?field=symbol&anchor=equals&symbol_search=Search&number=100&format=html&sortby=symbol&match=ROCK1) |  | [601702](http://www.ncbi.nlm.nih.gov/entrez/dispomim.cgi?id=601702) | **-1.26** |
| 1450997_AT | serine/threonine kinase 17b (apoptosis-inducing) | [STK17B](http://www.gene.ucl.ac.uk/cgi-bin/nomenclature/searchgenes.pl?field=symbol&anchor=equals&symbol_search=Search&number=100&format=html&sortby=symbol&match=STK17B) |  | [604727](http://www.ncbi.nlm.nih.gov/entrez/dispomim.cgi?id=604727) | **-2.35** |
| 1451000_AT | RIKEN cDNA 1810020E01 gene | [1810020E01RIK](http://www.gene.ucl.ac.uk/cgi-bin/nomenclature/searchgenes.pl?field=symbol&anchor=equals&symbol_search=Search&number=100&format=html&sortby=symbol&match=1810020E01RIK) |  |  | **1.24** |
| 1451002_AT | aconitase 2, mitochondrial | [ACO2](http://www.gene.ucl.ac.uk/cgi-bin/nomenclature/searchgenes.pl?field=symbol&anchor=equals&symbol_search=Search&number=100&format=html&sortby=symbol&match=ACO2) |  | [100850](http://www.ncbi.nlm.nih.gov/entrez/dispomim.cgi?id=100850) | **1.3** |
| 1451003_AT | mitogen-activated protein kinase kinase kinase 7 interacting protein 2 | [MAP3K7IP2](http://www.gene.ucl.ac.uk/cgi-bin/nomenclature/searchgenes.pl?field=symbol&anchor=equals&symbol_search=Search&number=100&format=html&sortby=symbol&match=MAP3K7IP2) |  | [605101](http://www.ncbi.nlm.nih.gov/entrez/dispomim.cgi?id=605101) | **1.37** |
| 1451011_AT | zinc finger protein 358 | [ZFP358](http://www.gene.ucl.ac.uk/cgi-bin/nomenclature/searchgenes.pl?field=symbol&anchor=equals&symbol_search=Search&number=100&format=html&sortby=symbol&match=ZFP358) |  |  | **1.44** |
| 1451016_AT | interferon-related developmental regulator 2 | [IFRD2](http://www.gene.ucl.ac.uk/cgi-bin/nomenclature/searchgenes.pl?field=symbol&anchor=equals&symbol_search=Search&number=100&format=html&sortby=symbol&match=IFRD2) |  | [602725](http://www.ncbi.nlm.nih.gov/entrez/dispomim.cgi?id=602725) | **1.56** |
| 1451021_A_AT | Kruppel-like factor 5 | [KLF5](http://www.gene.ucl.ac.uk/cgi-bin/nomenclature/searchgenes.pl?field=symbol&anchor=equals&symbol_search=Search&number=100&format=html&sortby=symbol&match=KLF5) |  | [602903](http://www.ncbi.nlm.nih.gov/entrez/dispomim.cgi?id=602903) | **-3.65** |
| 1451035_A_AT | aldo-keto reductase family 1, member A4 (aldehyde reductase) | [AKR1A4](http://www.gene.ucl.ac.uk/cgi-bin/nomenclature/searchgenes.pl?field=symbol&anchor=equals&symbol_search=Search&number=100&format=html&sortby=symbol&match=AKR1A4) |  |  | **-1.37** |
| 1451036_AT | spastic paraplegia 21 homolog (human) | [SPG21](http://www.gene.ucl.ac.uk/cgi-bin/nomenclature/searchgenes.pl?field=symbol&anchor=equals&symbol_search=Search&number=100&format=html&sortby=symbol&match=SPG21) |  | [608181](http://www.ncbi.nlm.nih.gov/entrez/dispomim.cgi?id=608181) | **-1.84** |
| 1451038_AT | apelin | [APLN](http://www.gene.ucl.ac.uk/cgi-bin/nomenclature/searchgenes.pl?field=symbol&anchor=equals&symbol_search=Search&number=100&format=html&sortby=symbol&match=APLN) |  | [300297](http://www.ncbi.nlm.nih.gov/entrez/dispomim.cgi?id=300297) | **1.46** |
| 1451047_AT | integral membrane protein 2A | [ITM2A](http://www.gene.ucl.ac.uk/cgi-bin/nomenclature/searchgenes.pl?field=symbol&anchor=equals&symbol_search=Search&number=100&format=html&sortby=symbol&match=ITM2A) |  | [300222](http://www.ncbi.nlm.nih.gov/entrez/dispomim.cgi?id=300222) | **-1.34** |
| 1451064_A_AT | phosphoserine aminotransferase 1 | [PSAT1](http://www.gene.ucl.ac.uk/cgi-bin/nomenclature/searchgenes.pl?field=symbol&anchor=equals&symbol_search=Search&number=100&format=html&sortby=symbol&match=PSAT1) |  |  | **-3.22** |
| 1451065_A_AT | DEAD (Asp-Glu-Ala-Asp) box polypeptide 39 | [DDX39](http://www.gene.ucl.ac.uk/cgi-bin/nomenclature/searchgenes.pl?field=symbol&anchor=equals&symbol_search=Search&number=100&format=html&sortby=symbol&match=DDX39) |  |  | **-1.76** |
| 1451068_S_AT | ribosomal protein S25 | [RPS25](http://www.gene.ucl.ac.uk/cgi-bin/nomenclature/searchgenes.pl?field=symbol&anchor=equals&symbol_search=Search&number=100&format=html&sortby=symbol&match=RPS25) |  | [180465](http://www.ncbi.nlm.nih.gov/entrez/dispomim.cgi?id=180465) | **-1.2** |
| 1451069_AT | proviral integration site 3 | [PIM3](http://www.gene.ucl.ac.uk/cgi-bin/nomenclature/searchgenes.pl?field=symbol&anchor=equals&symbol_search=Search&number=100&format=html&sortby=symbol&match=PIM3) |  |  | **1.64** |
| 1451077_AT | ribosomal protein L5 | [RPL5](http://www.gene.ucl.ac.uk/cgi-bin/nomenclature/searchgenes.pl?field=symbol&anchor=equals&symbol_search=Search&number=100&format=html&sortby=symbol&match=RPL5) |  | [603634](http://www.ncbi.nlm.nih.gov/entrez/dispomim.cgi?id=603634) | **-1.35** |
| 1451083_S_AT | alanyl-tRNA synthetase | [AARS](http://www.gene.ucl.ac.uk/cgi-bin/nomenclature/searchgenes.pl?field=symbol&anchor=equals&symbol_search=Search&number=100&format=html&sortby=symbol&match=AARS) |  | [601065](http://www.ncbi.nlm.nih.gov/entrez/dispomim.cgi?id=601065) | **1.32** |
| 1451084_AT | electron transferring flavoprotein, dehydrogenase | [ETFDH](http://www.gene.ucl.ac.uk/cgi-bin/nomenclature/searchgenes.pl?field=symbol&anchor=equals&symbol_search=Search&number=100&format=html&sortby=symbol&match=ETFDH) |  | [231675](http://www.ncbi.nlm.nih.gov/entrez/dispomim.cgi?id=231675) | **1.96** |
| 1451085_AT | RIKEN cDNA C030006K11 gene | [C030006K11RIK](http://www.gene.ucl.ac.uk/cgi-bin/nomenclature/searchgenes.pl?field=symbol&anchor=equals&symbol_search=Search&number=100&format=html&sortby=symbol&match=C030006K11RIK) |  |  | **1.68** |
| 1451091_AT | thioredoxin domain containing 5 | [TXNDC5](http://www.gene.ucl.ac.uk/cgi-bin/nomenclature/searchgenes.pl?field=symbol&anchor=equals&symbol_search=Search&number=100&format=html&sortby=symbol&match=TXNDC5) |  | [607289](http://www.ncbi.nlm.nih.gov/entrez/dispomim.cgi?id=607289) | **1.26** |
| 1451095_AT | asparagine synthetase | [ASNS](http://www.gene.ucl.ac.uk/cgi-bin/nomenclature/searchgenes.pl?field=symbol&anchor=equals&symbol_search=Search&number=100&format=html&sortby=symbol&match=ASNS) |  | [108370](http://www.ncbi.nlm.nih.gov/entrez/dispomim.cgi?id=108370) | **-1.57** |
| 1451096_AT | NADH dehydrogenase (ubiquinone) Fe-S protein 2 | [NDUFS2](http://www.gene.ucl.ac.uk/cgi-bin/nomenclature/searchgenes.pl?field=symbol&anchor=equals&symbol_search=Search&number=100&format=html&sortby=symbol&match=NDUFS2) |  | [602985](http://www.ncbi.nlm.nih.gov/entrez/dispomim.cgi?id=602985) | **1.34** |
| 1451097_AT | gb:BC015289.1 /DB_XREF=gi:15929730 /FEA=FLmRNA /CN |  |  |  | **-1.33** |
| 1451099_AT | membrane bound C2 domain containing protein | [MBC2](http://www.gene.ucl.ac.uk/cgi-bin/nomenclature/searchgenes.pl?field=symbol&anchor=equals&symbol_search=Search&number=100&format=html&sortby=symbol&match=MBC2) |  |  | **-1.43** |
| 1451104_A_AT | U1 small nuclear ribonucleoprotein polypeptide A | [SNRP70](http://www.gene.ucl.ac.uk/cgi-bin/nomenclature/searchgenes.pl?field=symbol&anchor=equals&symbol_search=Search&number=100&format=html&sortby=symbol&match=SNRP70) |  | [180740](http://www.ncbi.nlm.nih.gov/entrez/dispomim.cgi?id=180740) | **-1.44** |
| 1451105_AT | RIKEN cDNA B130052G07 gene | [B130052G07RIK](http://www.gene.ucl.ac.uk/cgi-bin/nomenclature/searchgenes.pl?field=symbol&anchor=equals&symbol_search=Search&number=100&format=html&sortby=symbol&match=B130052G07RIK) |  |  | **-10.76** |
| 1451110_AT | EGL nine homolog 1 (C. elegans) | [EGLN1](http://www.gene.ucl.ac.uk/cgi-bin/nomenclature/searchgenes.pl?field=symbol&anchor=equals&symbol_search=Search&number=100&format=html&sortby=symbol&match=EGLN1) |  | [606425](http://www.ncbi.nlm.nih.gov/entrez/dispomim.cgi?id=606425) | **2.54** |
| 1451112_S_AT | death-associated protein | [DAP](http://www.gene.ucl.ac.uk/cgi-bin/nomenclature/searchgenes.pl?field=symbol&anchor=equals&symbol_search=Search&number=100&format=html&sortby=symbol&match=DAP) |  | [600954](http://www.ncbi.nlm.nih.gov/entrez/dispomim.cgi?id=600954) | **-1.64** |
| 1451113_A_AT | IK cytokine | [IK](http://www.gene.ucl.ac.uk/cgi-bin/nomenclature/searchgenes.pl?field=symbol&anchor=equals&symbol_search=Search&number=100&format=html&sortby=symbol&match=IK) |  | [600549](http://www.ncbi.nlm.nih.gov/entrez/dispomim.cgi?id=600549) | **-1.36** |
| 1451120_AT | RNA polymerase 1-3 | [RPO1-3](http://www.gene.ucl.ac.uk/cgi-bin/nomenclature/searchgenes.pl?field=symbol&anchor=equals&symbol_search=Search&number=100&format=html&sortby=symbol&match=RPO1-3) |  |  | **-1.42** |
| 1451124_AT | superoxide dismutase 1, soluble | [SOD1](http://www.gene.ucl.ac.uk/cgi-bin/nomenclature/searchgenes.pl?field=symbol&anchor=equals&symbol_search=Search&number=100&format=html&sortby=symbol&match=SOD1) |  | [147450](http://www.ncbi.nlm.nih.gov/entrez/dispomim.cgi?id=147450) | **1.22** |
| 1451125_AT | cDNA sequence BC017133 | [BC017133](http://www.gene.ucl.ac.uk/cgi-bin/nomenclature/searchgenes.pl?field=symbol&anchor=equals&symbol_search=Search&number=100&format=html&sortby=symbol&match=BC017133) |  |  | **1.65** |
| 1451127_AT | expressed sequence AW146242 | [AW146242](http://www.gene.ucl.ac.uk/cgi-bin/nomenclature/searchgenes.pl?field=symbol&anchor=equals&symbol_search=Search&number=100&format=html&sortby=symbol&match=AW146242) |  |  | **1.35** |
| 1451145_S_AT | RIKEN cDNA 0610039A15 gene | [0610039A15RIK](http://www.gene.ucl.ac.uk/cgi-bin/nomenclature/searchgenes.pl?field=symbol&anchor=equals&symbol_search=Search&number=100&format=html&sortby=symbol&match=0610039A15RIK) |  |  | **-1.24** |
| 1451148_AT | PTEN induced putative kinase 1 | [PINK1](http://www.gene.ucl.ac.uk/cgi-bin/nomenclature/searchgenes.pl?field=symbol&anchor=equals&symbol_search=Search&number=100&format=html&sortby=symbol&match=PINK1) |  | [608309](http://www.ncbi.nlm.nih.gov/entrez/dispomim.cgi?id=608309) | **1.5** |
| 1451149_AT | phosphoglucomutase 2 | [PGM2](http://www.gene.ucl.ac.uk/cgi-bin/nomenclature/searchgenes.pl?field=symbol&anchor=equals&symbol_search=Search&number=100&format=html&sortby=symbol&match=PGM2) |  |  | **1.39** |
| 1451152_A_AT | ATPase, Na+/K+ transporting, beta 1 polypeptide | [ATP1B1](http://www.gene.ucl.ac.uk/cgi-bin/nomenclature/searchgenes.pl?field=symbol&anchor=equals&symbol_search=Search&number=100&format=html&sortby=symbol&match=ATP1B1) |  | [182330](http://www.ncbi.nlm.nih.gov/entrez/dispomim.cgi?id=182330) | **1.38** |
| 1451154_A_AT | CUG triplet repeat,RNA binding protein 2 | [CUGBP2](http://www.gene.ucl.ac.uk/cgi-bin/nomenclature/searchgenes.pl?field=symbol&anchor=equals&symbol_search=Search&number=100&format=html&sortby=symbol&match=CUGBP2) |  | [602538](http://www.ncbi.nlm.nih.gov/entrez/dispomim.cgi?id=602538) | **-1.35** |
| 1451156_S_AT | very low density lipoprotein receptor | [VLDLR](http://www.gene.ucl.ac.uk/cgi-bin/nomenclature/searchgenes.pl?field=symbol&anchor=equals&symbol_search=Search&number=100&format=html&sortby=symbol&match=VLDLR) |  | [192977](http://www.ncbi.nlm.nih.gov/entrez/dispomim.cgi?id=192977) | **1.47** |
| 1451160_S_AT | DNA segment, Chr 7, ERATO Doi 458, expressed | [D7ERTD458E](http://www.gene.ucl.ac.uk/cgi-bin/nomenclature/searchgenes.pl?field=symbol&anchor=equals&symbol_search=Search&number=100&format=html&sortby=symbol&match=D7ERTD458E) |  |  | **-1.89** |
| 1451161_A_AT | EGF-like module containing, mucin-like, hormone receptor-like sequence 1 | [EMR1](http://www.gene.ucl.ac.uk/cgi-bin/nomenclature/searchgenes.pl?field=symbol&anchor=equals&symbol_search=Search&number=100&format=html&sortby=symbol&match=EMR1) |  | [600493](http://www.ncbi.nlm.nih.gov/entrez/dispomim.cgi?id=600493) | **-2.51** |
| 1451175_AT | RIKEN cDNA 1810011E08 gene | [1810011E08RIK](http://www.gene.ucl.ac.uk/cgi-bin/nomenclature/searchgenes.pl?field=symbol&anchor=equals&symbol_search=Search&number=100&format=html&sortby=symbol&match=1810011E08RIK) |  |  | **-1.48** |
| 1451184_AT | heterogeneous nuclear ribonucleoprotein A3 | [HNRPA3](http://www.gene.ucl.ac.uk/cgi-bin/nomenclature/searchgenes.pl?field=symbol&anchor=equals&symbol_search=Search&number=100&format=html&sortby=symbol&match=HNRPA3) |  | [605372](http://www.ncbi.nlm.nih.gov/entrez/dispomim.cgi?id=605372) | **-1.35** |
| 1451187_AT | RIKEN cDNA 0610037P05 gene | [0610037P05RIK](http://www.gene.ucl.ac.uk/cgi-bin/nomenclature/searchgenes.pl?field=symbol&anchor=equals&symbol_search=Search&number=100&format=html&sortby=symbol&match=0610037P05RIK) |  |  | **1.27** |
| 1451188_AT | WD repeat domain 26 | [WDR26](http://www.gene.ucl.ac.uk/cgi-bin/nomenclature/searchgenes.pl?field=symbol&anchor=equals&symbol_search=Search&number=100&format=html&sortby=symbol&match=WDR26) |  |  | **-1.3** |
| 1451201_S_AT | ribonuclease/angiogenin inhibitor 1 | [RNH1](http://www.gene.ucl.ac.uk/cgi-bin/nomenclature/searchgenes.pl?field=symbol&anchor=equals&symbol_search=Search&number=100&format=html&sortby=symbol&match=RNH1) |  |  | **-1.85** |
| 1451204_AT | RIKEN cDNA 4933425F03 gene | [4933425F03RIK](http://www.gene.ucl.ac.uk/cgi-bin/nomenclature/searchgenes.pl?field=symbol&anchor=equals&symbol_search=Search&number=100&format=html&sortby=symbol&match=4933425F03RIK) |  |  | **1.37** |
| 1451206_S_AT | pleckstrin homology, Sec7 and coiled-coil domains, binding protein | [PSCDBP](http://www.gene.ucl.ac.uk/cgi-bin/nomenclature/searchgenes.pl?field=symbol&anchor=equals&symbol_search=Search&number=100&format=html&sortby=symbol&match=PSCDBP) |  | [604448](http://www.ncbi.nlm.nih.gov/entrez/dispomim.cgi?id=604448) | **-2.57** |
| 1451209_AT | longevity assurance homolog 5 (S. cerevisiae) | [LASS5](http://www.gene.ucl.ac.uk/cgi-bin/nomenclature/searchgenes.pl?field=symbol&anchor=equals&symbol_search=Search&number=100&format=html&sortby=symbol&match=LASS5) |  |  | **-1.34** |
| 1451212_AT | RIKEN cDNA 2410030J07 gene | [2410030J07RIK](http://www.gene.ucl.ac.uk/cgi-bin/nomenclature/searchgenes.pl?field=symbol&anchor=equals&symbol_search=Search&number=100&format=html&sortby=symbol&match=2410030J07RIK) |  |  | **1.55** |
| 1451225_AT | protein tyrosine phosphatase, non-receptor type 11 | [PTPN11](http://www.gene.ucl.ac.uk/cgi-bin/nomenclature/searchgenes.pl?field=symbol&anchor=equals&symbol_search=Search&number=100&format=html&sortby=symbol&match=PTPN11) |  | [176876](http://www.ncbi.nlm.nih.gov/entrez/dispomim.cgi?id=176876) | **1.21** |
| 1451230_A_AT | WW domain binding protein 5 | [WBP5](http://www.gene.ucl.ac.uk/cgi-bin/nomenclature/searchgenes.pl?field=symbol&anchor=equals&symbol_search=Search&number=100&format=html&sortby=symbol&match=WBP5) |  |  | **-1.77** |
| 1451237_S_AT | RNA binding motif protein 7 | [RBM7](http://www.gene.ucl.ac.uk/cgi-bin/nomenclature/searchgenes.pl?field=symbol&anchor=equals&symbol_search=Search&number=100&format=html&sortby=symbol&match=RBM7) |  |  | **-1.35** |
| 1451240_A_AT | glyoxalase 1 | [GLO1](http://www.gene.ucl.ac.uk/cgi-bin/nomenclature/searchgenes.pl?field=symbol&anchor=equals&symbol_search=Search&number=100&format=html&sortby=symbol&match=GLO1) |  | [138750](http://www.ncbi.nlm.nih.gov/entrez/dispomim.cgi?id=138750) | **1.25** |
| 1451242_A_AT | protein phosphatase 5, catalytic subunit | [PPP5C](http://www.gene.ucl.ac.uk/cgi-bin/nomenclature/searchgenes.pl?field=symbol&anchor=equals&symbol_search=Search&number=100&format=html&sortby=symbol&match=PPP5C) |  | [600658](http://www.ncbi.nlm.nih.gov/entrez/dispomim.cgi?id=600658) | **1.31** |
| 1451247_AT | RIKEN cDNA 1200003O06 gene | [1200003O06RIK](http://www.gene.ucl.ac.uk/cgi-bin/nomenclature/searchgenes.pl?field=symbol&anchor=equals&symbol_search=Search&number=100&format=html&sortby=symbol&match=1200003O06RIK) |  |  | **-1.4** |
| 1451263_A_AT | fatty acid binding protein 4, adipocyte | [FABP4](http://www.gene.ucl.ac.uk/cgi-bin/nomenclature/searchgenes.pl?field=symbol&anchor=equals&symbol_search=Search&number=100&format=html&sortby=symbol&match=FABP4) |  | [600434](http://www.ncbi.nlm.nih.gov/entrez/dispomim.cgi?id=600434) | **1.37** |
| 1451271_A_AT | acetyl-Coenzyme A acetyltransferase 1 | [ACAT1](http://www.gene.ucl.ac.uk/cgi-bin/nomenclature/searchgenes.pl?field=symbol&anchor=equals&symbol_search=Search&number=100&format=html&sortby=symbol&match=ACAT1) |  | [607809](http://www.ncbi.nlm.nih.gov/entrez/dispomim.cgi?id=607809) | **1.96** |
| 1451274_AT | oxoglutarate dehydrogenase (lipoamide) | [OGDH](http://www.gene.ucl.ac.uk/cgi-bin/nomenclature/searchgenes.pl?field=symbol&anchor=equals&symbol_search=Search&number=100&format=html&sortby=symbol&match=OGDH) |  | [203740](http://www.ncbi.nlm.nih.gov/entrez/dispomim.cgi?id=203740) | **1.5** |
| 1451277_AT | zinc binding alcohol dehydrogenase, domain containing 2 | [ZADH2](http://www.gene.ucl.ac.uk/cgi-bin/nomenclature/searchgenes.pl?field=symbol&anchor=equals&symbol_search=Search&number=100&format=html&sortby=symbol&match=ZADH2) |  |  | **1.44** |
| 1451280_AT | RIKEN cDNA 0710001E13 gene | [0710001E13RIK](http://www.gene.ucl.ac.uk/cgi-bin/nomenclature/searchgenes.pl?field=symbol&anchor=equals&symbol_search=Search&number=100&format=html&sortby=symbol&match=0710001E13RIK) |  |  | **-2.29** |
| 1451283_AT | RIKEN cDNA 1810073G14 gene | [1810073G14RIK](http://www.gene.ucl.ac.uk/cgi-bin/nomenclature/searchgenes.pl?field=symbol&anchor=equals&symbol_search=Search&number=100&format=html&sortby=symbol&match=1810073G14RIK) |  |  | **1.37** |
| 1451287_S_AT | RIKEN cDNA 2810003C17 gene | [2810003C17RIK](http://www.gene.ucl.ac.uk/cgi-bin/nomenclature/searchgenes.pl?field=symbol&anchor=equals&symbol_search=Search&number=100&format=html&sortby=symbol&match=2810003C17RIK) |  |  | **-7.76** |
| 1451289_AT | Double cortin and calcium/calmodulin-dependent protein kinase-like 1 | [DCAMKL1](http://www.gene.ucl.ac.uk/cgi-bin/nomenclature/searchgenes.pl?field=symbol&anchor=equals&symbol_search=Search&number=100&format=html&sortby=symbol&match=DCAMKL1) |  | [604742](http://www.ncbi.nlm.nih.gov/entrez/dispomim.cgi?id=604742) | **-5.05** |
| 1451294_S_AT | small nuclear ribonucleoprotein E | [SNRPE](http://www.gene.ucl.ac.uk/cgi-bin/nomenclature/searchgenes.pl?field=symbol&anchor=equals&symbol_search=Search&number=100&format=html&sortby=symbol&match=SNRPE) |  | [128260](http://www.ncbi.nlm.nih.gov/entrez/dispomim.cgi?id=128260) | **-1.57** |
| 1451295_A_AT | chromodomain helicase DNA binding protein 4 | [CHD4](http://www.gene.ucl.ac.uk/cgi-bin/nomenclature/searchgenes.pl?field=symbol&anchor=equals&symbol_search=Search&number=100&format=html&sortby=symbol&match=CHD4) |  | [603277](http://www.ncbi.nlm.nih.gov/entrez/dispomim.cgi?id=603277) | **-1.33** |
| 1451304_AT | RIKEN cDNA 2310076O21 gene | [2310076O21RIK](http://www.gene.ucl.ac.uk/cgi-bin/nomenclature/searchgenes.pl?field=symbol&anchor=equals&symbol_search=Search&number=100&format=html&sortby=symbol&match=2310076O21RIK) |  |  | **1.98** |
| 1451307_AT | mitochondrial ribosomal protein L14 | [MRPL14](http://www.gene.ucl.ac.uk/cgi-bin/nomenclature/searchgenes.pl?field=symbol&anchor=equals&symbol_search=Search&number=100&format=html&sortby=symbol&match=MRPL14) |  |  | **1.38** |
| 1451310_A_AT | cathepsin L | [CTSL](http://www.gene.ucl.ac.uk/cgi-bin/nomenclature/searchgenes.pl?field=symbol&anchor=equals&symbol_search=Search&number=100&format=html&sortby=symbol&match=CTSL) |  | [116880](http://www.ncbi.nlm.nih.gov/entrez/dispomim.cgi?id=116880) | **-1.49** |
| 1451311_A_AT | adiponectin receptor 1 | [ADIPOR1](http://www.gene.ucl.ac.uk/cgi-bin/nomenclature/searchgenes.pl?field=symbol&anchor=equals&symbol_search=Search&number=100&format=html&sortby=symbol&match=ADIPOR1) |  | [607945](http://www.ncbi.nlm.nih.gov/entrez/dispomim.cgi?id=607945) | **1.26** |
| 1451312_AT | NADH dehydrogenase (ubiquinone) Fe-S protein 7 | [NDUFS7](http://www.gene.ucl.ac.uk/cgi-bin/nomenclature/searchgenes.pl?field=symbol&anchor=equals&symbol_search=Search&number=100&format=html&sortby=symbol&match=NDUFS7) |  | [601825](http://www.ncbi.nlm.nih.gov/entrez/dispomim.cgi?id=601825) | **1.45** |
| 1451313_A_AT | RIKEN cDNA 1110067D22 gene | [1110067D22RIK](http://www.gene.ucl.ac.uk/cgi-bin/nomenclature/searchgenes.pl?field=symbol&anchor=equals&symbol_search=Search&number=100&format=html&sortby=symbol&match=1110067D22RIK) |  |  | **2.1** |
| 1451322_AT | RIKEN cDNA 2310016A09 gene | [2310016A09RIK](http://www.gene.ucl.ac.uk/cgi-bin/nomenclature/searchgenes.pl?field=symbol&anchor=equals&symbol_search=Search&number=100&format=html&sortby=symbol&match=2310016A09RIK) |  |  | **2.2** |
| 1451327_A_AT | interleukin 1 receptor-like 1 ligand | [IL1RL1L](http://www.gene.ucl.ac.uk/cgi-bin/nomenclature/searchgenes.pl?field=symbol&anchor=equals&symbol_search=Search&number=100&format=html&sortby=symbol&match=IL1RL1L) |  |  | **1.29** |
| 1451335_AT | placenta-specific 8 | [PLAC8](http://www.gene.ucl.ac.uk/cgi-bin/nomenclature/searchgenes.pl?field=symbol&anchor=equals&symbol_search=Search&number=100&format=html&sortby=symbol&match=PLAC8) |  | [607515](http://www.ncbi.nlm.nih.gov/entrez/dispomim.cgi?id=607515) | **-5.06** |
| 1451336_AT | lectin, galactose binding, soluble 4 | [LGALS4](http://www.gene.ucl.ac.uk/cgi-bin/nomenclature/searchgenes.pl?field=symbol&anchor=equals&symbol_search=Search&number=100&format=html&sortby=symbol&match=LGALS4) |  | [602518](http://www.ncbi.nlm.nih.gov/entrez/dispomim.cgi?id=602518) | **1.47** |
| 1451341_S_AT | expressed sequence AI840826 | [AI840826](http://www.gene.ucl.ac.uk/cgi-bin/nomenclature/searchgenes.pl?field=symbol&anchor=equals&symbol_search=Search&number=100&format=html&sortby=symbol&match=AI840826) |  |  | **-1.48** |
| 1451350_A_AT | leptin receptor gene-related protein | [LEPROT](http://www.gene.ucl.ac.uk/cgi-bin/nomenclature/searchgenes.pl?field=symbol&anchor=equals&symbol_search=Search&number=100&format=html&sortby=symbol&match=LEPROT) |  |  | **-1.38** |
| 1451353_AT | transmembrane 6 superfamily member 1 | [TM6SF1](http://www.gene.ucl.ac.uk/cgi-bin/nomenclature/searchgenes.pl?field=symbol&anchor=equals&symbol_search=Search&number=100&format=html&sortby=symbol&match=TM6SF1) |  | [606562](http://www.ncbi.nlm.nih.gov/entrez/dispomim.cgi?id=606562) | **-1.9** |
| 1451355_AT | cancer related gene-liver 1 | [CRG-L1](http://www.gene.ucl.ac.uk/cgi-bin/nomenclature/searchgenes.pl?field=symbol&anchor=equals&symbol_search=Search&number=100&format=html&sortby=symbol&match=CRG-L1) |  |  | **2.06** |
| 1451357_AT | RIKEN cDNA E130307M08 gene | [E130307M08RIK](http://www.gene.ucl.ac.uk/cgi-bin/nomenclature/searchgenes.pl?field=symbol&anchor=equals&symbol_search=Search&number=100&format=html&sortby=symbol&match=E130307M08RIK) |  |  | **1.31** |
| 1451362_AT | RAB7, member RAS oncogene family-like 1 | [RAB7L1](http://www.gene.ucl.ac.uk/cgi-bin/nomenclature/searchgenes.pl?field=symbol&anchor=equals&symbol_search=Search&number=100&format=html&sortby=symbol&match=RAB7L1) |  | [603949](http://www.ncbi.nlm.nih.gov/entrez/dispomim.cgi?id=603949) | **-1.4** |
| 1451398_AT | cDNA sequence BC009118 | [BC009118](http://www.gene.ucl.ac.uk/cgi-bin/nomenclature/searchgenes.pl?field=symbol&anchor=equals&symbol_search=Search&number=100&format=html&sortby=symbol&match=BC009118) |  |  | **1.47** |
| 1451405_AT | propionyl-Coenzyme A carboxylase, alpha polypeptide | [PCCA](http://www.gene.ucl.ac.uk/cgi-bin/nomenclature/searchgenes.pl?field=symbol&anchor=equals&symbol_search=Search&number=100&format=html&sortby=symbol&match=PCCA) |  | [232000](http://www.ncbi.nlm.nih.gov/entrez/dispomim.cgi?id=232000) | **1.7** |
| 1451413_AT | calpastatin | [CAST](http://www.gene.ucl.ac.uk/cgi-bin/nomenclature/searchgenes.pl?field=symbol&anchor=equals&symbol_search=Search&number=100&format=html&sortby=symbol&match=CAST) |  | [114090](http://www.ncbi.nlm.nih.gov/entrez/dispomim.cgi?id=114090) | **1.31** |
| 1451415_AT | RIKEN cDNA 1810011O10 gene | [1810011O10RIK](http://www.gene.ucl.ac.uk/cgi-bin/nomenclature/searchgenes.pl?field=symbol&anchor=equals&symbol_search=Search&number=100&format=html&sortby=symbol&match=1810011O10RIK) |  |  | **2.26** |
| 1451421_A_AT | leucine zipper domain protein | [LZF](http://www.gene.ucl.ac.uk/cgi-bin/nomenclature/searchgenes.pl?field=symbol&anchor=equals&symbol_search=Search&number=100&format=html&sortby=symbol&match=LZF) |  |  | **-1.52** |
| 1451427_A_AT | EGF-like domain 7 | [EGFL7](http://www.gene.ucl.ac.uk/cgi-bin/nomenclature/searchgenes.pl?field=symbol&anchor=equals&symbol_search=Search&number=100&format=html&sortby=symbol&match=EGFL7) |  | [608582](http://www.ncbi.nlm.nih.gov/entrez/dispomim.cgi?id=608582) | **1.56** |
| 1451428_X_AT | EGF-like domain 7 | [EGFL7](http://www.gene.ucl.ac.uk/cgi-bin/nomenclature/searchgenes.pl?field=symbol&anchor=equals&symbol_search=Search&number=100&format=html&sortby=symbol&match=EGFL7) |  | [608582](http://www.ncbi.nlm.nih.gov/entrez/dispomim.cgi?id=608582) | **1.46** |
| 1451440_AT | chondrolectin | [CHODL](http://www.gene.ucl.ac.uk/cgi-bin/nomenclature/searchgenes.pl?field=symbol&anchor=equals&symbol_search=Search&number=100&format=html&sortby=symbol&match=CHODL) |  | [607247](http://www.ncbi.nlm.nih.gov/entrez/dispomim.cgi?id=607247) | **-1.89** |
| 1451442_AT | RIKEN cDNA 4931428D14 gene | [4931428D14RIK](http://www.gene.ucl.ac.uk/cgi-bin/nomenclature/searchgenes.pl?field=symbol&anchor=equals&symbol_search=Search&number=100&format=html&sortby=symbol&match=4931428D14RIK) |  |  | **-1.62** |
| 1451446_AT | anthrax toxin receptor 1 | [ANTXR1](http://www.gene.ucl.ac.uk/cgi-bin/nomenclature/searchgenes.pl?field=symbol&anchor=equals&symbol_search=Search&number=100&format=html&sortby=symbol&match=ANTXR1) |  | [606410](http://www.ncbi.nlm.nih.gov/entrez/dispomim.cgi?id=606410) | **-1.96** |
| 1451447_AT | CUE domain containing 1 | [CUEDC1](http://www.gene.ucl.ac.uk/cgi-bin/nomenclature/searchgenes.pl?field=symbol&anchor=equals&symbol_search=Search&number=100&format=html&sortby=symbol&match=CUEDC1) |  |  | **1.49** |
| 1451453_AT | death-associated kinase 2 | [DAPK2](http://www.gene.ucl.ac.uk/cgi-bin/nomenclature/searchgenes.pl?field=symbol&anchor=equals&symbol_search=Search&number=100&format=html&sortby=symbol&match=DAPK2) |  |  | **-1.48** |
| 1451458_AT | transmembrane protein 2 | [TMEM2](http://www.gene.ucl.ac.uk/cgi-bin/nomenclature/searchgenes.pl?field=symbol&anchor=equals&symbol_search=Search&number=100&format=html&sortby=symbol&match=TMEM2) |  | [605835](http://www.ncbi.nlm.nih.gov/entrez/dispomim.cgi?id=605835) | **-1.5** |
| 1451465_AT | RIKEN cDNA 2300004C15 gene | [2300004C15RIK](http://www.gene.ucl.ac.uk/cgi-bin/nomenclature/searchgenes.pl?field=symbol&anchor=equals&symbol_search=Search&number=100&format=html&sortby=symbol&match=2300004C15RIK) |  |  | **1.23** |
| 1451485_AT | RIKEN cDNA 3300001P08 gene | [3300001P08RIK](http://www.gene.ucl.ac.uk/cgi-bin/nomenclature/searchgenes.pl?field=symbol&anchor=equals&symbol_search=Search&number=100&format=html&sortby=symbol&match=3300001P08RIK) |  |  | **-1.54** |
| 1451489_AT | RIKEN cDNA 1810012H11 gene | [1810012H11RIK](http://www.gene.ucl.ac.uk/cgi-bin/nomenclature/searchgenes.pl?field=symbol&anchor=equals&symbol_search=Search&number=100&format=html&sortby=symbol&match=1810012H11RIK) |  |  | **1.3** |
| 1451500_AT | Usher syndrome 1C binding protein 1 | [USHBP1](http://www.gene.ucl.ac.uk/cgi-bin/nomenclature/searchgenes.pl?field=symbol&anchor=equals&symbol_search=Search&number=100&format=html&sortby=symbol&match=USHBP1) |  |  | **2.05** |
| 1451501_A_AT | growth hormone receptor | [GHR](http://www.gene.ucl.ac.uk/cgi-bin/nomenclature/searchgenes.pl?field=symbol&anchor=equals&symbol_search=Search&number=100&format=html&sortby=symbol&match=GHR) |  | [600946](http://www.ncbi.nlm.nih.gov/entrez/dispomim.cgi?id=600946) | **1.37** |
| 1451504_AT | coiled-coil-helix-coiled-coil-helix domain containing 3 | [CHCHD3](http://www.gene.ucl.ac.uk/cgi-bin/nomenclature/searchgenes.pl?field=symbol&anchor=equals&symbol_search=Search&number=100&format=html&sortby=symbol&match=CHCHD3) |  |  | **1.57** |
| 1451512_S_AT | 3-hydroxyisobutyryl-Coenzyme A hydrolase | [HIBCH](http://www.gene.ucl.ac.uk/cgi-bin/nomenclature/searchgenes.pl?field=symbol&anchor=equals&symbol_search=Search&number=100&format=html&sortby=symbol&match=HIBCH) |  |  | **1.53** |
| 1451536_AT | RIKEN cDNA 1300002C08 gene | [1300002C08RIK](http://www.gene.ucl.ac.uk/cgi-bin/nomenclature/searchgenes.pl?field=symbol&anchor=equals&symbol_search=Search&number=100&format=html&sortby=symbol&match=1300002C08RIK) |  |  | **1.48** |
| 1451559_A_AT | Dehydrogenase/reductase (SDR family) member 4 | [DHRS4](http://www.gene.ucl.ac.uk/cgi-bin/nomenclature/searchgenes.pl?field=symbol&anchor=equals&symbol_search=Search&number=100&format=html&sortby=symbol&match=DHRS4) |  |  | **2.24** |
| 1451570_A_AT | RIKEN cDNA 6720467C03 gene | [6720467C03RIK](http://www.gene.ucl.ac.uk/cgi-bin/nomenclature/searchgenes.pl?field=symbol&anchor=equals&symbol_search=Search&number=100&format=html&sortby=symbol&match=6720467C03RIK) |  |  | **-1.31** |
| 1451575_A_AT | nudix (nucleotide diphosphate linked moiety X)-type motif 3 | [NUDT3](http://www.gene.ucl.ac.uk/cgi-bin/nomenclature/searchgenes.pl?field=symbol&anchor=equals&symbol_search=Search&number=100&format=html&sortby=symbol&match=NUDT3) |  |  | **1.31** |
| 1451591_A_AT | ephrin B1 | [EFNB1](http://www.gene.ucl.ac.uk/cgi-bin/nomenclature/searchgenes.pl?field=symbol&anchor=equals&symbol_search=Search&number=100&format=html&sortby=symbol&match=EFNB1) |  | [300035](http://www.ncbi.nlm.nih.gov/entrez/dispomim.cgi?id=300035) | **1.58** |
| 1451601_A_AT | cDNA sequence BC011467 | [BC011467](http://www.gene.ucl.ac.uk/cgi-bin/nomenclature/searchgenes.pl?field=symbol&anchor=equals&symbol_search=Search&number=100&format=html&sortby=symbol&match=BC011467) |  |  | **1.5** |
| 1451611_AT | HRAS like suppressor 3 | [HRASLS3](http://www.gene.ucl.ac.uk/cgi-bin/nomenclature/searchgenes.pl?field=symbol&anchor=equals&symbol_search=Search&number=100&format=html&sortby=symbol&match=HRASLS3) |  |  | **1.92** |
| 1451634_AT | RIKEN cDNA 2810051F02 gene | [2810051F02RIK](http://www.gene.ucl.ac.uk/cgi-bin/nomenclature/searchgenes.pl?field=symbol&anchor=equals&symbol_search=Search&number=100&format=html&sortby=symbol&match=2810051F02RIK) |  |  | **-1.5** |
| 1451643_A_AT | RAB4B, member RAS oncogene family | [RAB4B](http://www.gene.ucl.ac.uk/cgi-bin/nomenclature/searchgenes.pl?field=symbol&anchor=equals&symbol_search=Search&number=100&format=html&sortby=symbol&match=RAB4B) |  |  | **-1.69** |
| 1451649_A_AT | RIKEN cDNA 2410118I19 gene | [2410118I19RIK](http://www.gene.ucl.ac.uk/cgi-bin/nomenclature/searchgenes.pl?field=symbol&anchor=equals&symbol_search=Search&number=100&format=html&sortby=symbol&match=2410118I19RIK) |  |  | **-1.35** |
| 1451666_AT | ATP citrate lyase | [ACLY](http://www.gene.ucl.ac.uk/cgi-bin/nomenclature/searchgenes.pl?field=symbol&anchor=equals&symbol_search=Search&number=100&format=html&sortby=symbol&match=ACLY) |  | [108728](http://www.ncbi.nlm.nih.gov/entrez/dispomim.cgi?id=108728) | **2.05** |
| 1451667_AT | RIKEN cDNA C530043G21 gene | [C530043G21RIK](http://www.gene.ucl.ac.uk/cgi-bin/nomenclature/searchgenes.pl?field=symbol&anchor=equals&symbol_search=Search&number=100&format=html&sortby=symbol&match=C530043G21RIK) |  |  | **1.77** |
| 1451669_AT | protein phosphatase 1B, magnesium dependent, beta isoform | [PPM1B](http://www.gene.ucl.ac.uk/cgi-bin/nomenclature/searchgenes.pl?field=symbol&anchor=equals&symbol_search=Search&number=100&format=html&sortby=symbol&match=PPM1B) |  | [603770](http://www.ncbi.nlm.nih.gov/entrez/dispomim.cgi?id=603770) | **1.35** |
| 1451703_S_AT | gb:M11310.1 /DB_XREF=gi:192009 /FEA=FLmRNA /CNT=3 |  |  |  | **-1.33** |
| 1451707_S_AT | solute carrier family 41, member 3 | [SLC41A3](http://www.gene.ucl.ac.uk/cgi-bin/nomenclature/searchgenes.pl?field=symbol&anchor=equals&symbol_search=Search&number=100&format=html&sortby=symbol&match=SLC41A3) |  |  | **1.79** |
| 1451721_A_AT | histocompatibility 2, class II antigen A, beta 1 | [H2-AB1](http://www.gene.ucl.ac.uk/cgi-bin/nomenclature/searchgenes.pl?field=symbol&anchor=equals&symbol_search=Search&number=100&format=html&sortby=symbol&match=H2-AB1) |  |  | **-3.1** |
| 1451742_A_AT | UDP-glucose pyrophosphorylase 2 | [UGP2](http://www.gene.ucl.ac.uk/cgi-bin/nomenclature/searchgenes.pl?field=symbol&anchor=equals&symbol_search=Search&number=100&format=html&sortby=symbol&match=UGP2) |  | [191760](http://www.ncbi.nlm.nih.gov/entrez/dispomim.cgi?id=191760) | **1.41** |
| 1451744_A_AT | zinc binding alcohol dehydrogenase, domain containing 1 | [ZADH1](http://www.gene.ucl.ac.uk/cgi-bin/nomenclature/searchgenes.pl?field=symbol&anchor=equals&symbol_search=Search&number=100&format=html&sortby=symbol&match=ZADH1) |  | [608642](http://www.ncbi.nlm.nih.gov/entrez/dispomim.cgi?id=608642) | **1.45** |
| 1451755_A_AT | apolipoprotein B editing complex 1 | [APOBEC1](http://www.gene.ucl.ac.uk/cgi-bin/nomenclature/searchgenes.pl?field=symbol&anchor=equals&symbol_search=Search&number=100&format=html&sortby=symbol&match=APOBEC1) |  | [600130](http://www.ncbi.nlm.nih.gov/entrez/dispomim.cgi?id=600130) | **-1.83** |
| 1451756_AT | FMS-like tyrosine kinase 1 | [FLT1](http://www.gene.ucl.ac.uk/cgi-bin/nomenclature/searchgenes.pl?field=symbol&anchor=equals&symbol_search=Search&number=100&format=html&sortby=symbol&match=FLT1) |  | [165070](http://www.ncbi.nlm.nih.gov/entrez/dispomim.cgi?id=165070) | **2.43** |
| 1451760_S_AT | expressed sequence AI317395 | [AI317395](http://www.gene.ucl.ac.uk/cgi-bin/nomenclature/searchgenes.pl?field=symbol&anchor=equals&symbol_search=Search&number=100&format=html&sortby=symbol&match=AI317395) |  |  | **1.39** |
| 1451780_AT | B-cell linker | [BLNK](http://www.gene.ucl.ac.uk/cgi-bin/nomenclature/searchgenes.pl?field=symbol&anchor=equals&symbol_search=Search&number=100&format=html&sortby=symbol&match=BLNK) |  | [604515](http://www.ncbi.nlm.nih.gov/entrez/dispomim.cgi?id=604515) | **-1.86** |
| 1451791_AT | tissue factor pathway inhibitor | [TFPI](http://www.gene.ucl.ac.uk/cgi-bin/nomenclature/searchgenes.pl?field=symbol&anchor=equals&symbol_search=Search&number=100&format=html&sortby=symbol&match=TFPI) |  | [152310](http://www.ncbi.nlm.nih.gov/entrez/dispomim.cgi?id=152310) | **-1.81** |
| 1451794_AT | RIKEN cDNA C630016B22 gene | [C630016B22RIK](http://www.gene.ucl.ac.uk/cgi-bin/nomenclature/searchgenes.pl?field=symbol&anchor=equals&symbol_search=Search&number=100&format=html&sortby=symbol&match=C630016B22RIK) |  |  | **1.47** |
| 1451798_AT | interleukin 1 receptor antagonist | [IL1RN](http://www.gene.ucl.ac.uk/cgi-bin/nomenclature/searchgenes.pl?field=symbol&anchor=equals&symbol_search=Search&number=100&format=html&sortby=symbol&match=IL1RN) |  | [147679](http://www.ncbi.nlm.nih.gov/entrez/dispomim.cgi?id=147679) | **-2.08** |
| 1451801_AT | triadin | [TRDN](http://www.gene.ucl.ac.uk/cgi-bin/nomenclature/searchgenes.pl?field=symbol&anchor=equals&symbol_search=Search&number=100&format=html&sortby=symbol&match=TRDN) |  | [603283](http://www.ncbi.nlm.nih.gov/entrez/dispomim.cgi?id=603283) | **-2.18** |
| 1451803_A_AT | vascular endothelial growth factor B | [VEGFB](http://www.gene.ucl.ac.uk/cgi-bin/nomenclature/searchgenes.pl?field=symbol&anchor=equals&symbol_search=Search&number=100&format=html&sortby=symbol&match=VEGFB) |  | [601398](http://www.ncbi.nlm.nih.gov/entrez/dispomim.cgi?id=601398) | **1.69** |
| 1451810_AT | open reading frame 63 | [ORF63](http://www.gene.ucl.ac.uk/cgi-bin/nomenclature/searchgenes.pl?field=symbol&anchor=equals&symbol_search=Search&number=100&format=html&sortby=symbol&match=ORF63) |  |  | **-2** |
| 1451825_A_AT | coatomer protein complex, subunit zeta 1 | [COPZ1](http://www.gene.ucl.ac.uk/cgi-bin/nomenclature/searchgenes.pl?field=symbol&anchor=equals&symbol_search=Search&number=100&format=html&sortby=symbol&match=COPZ1) |  |  | **-1.24** |
| 1451827_A_AT | NADPH oxidase 4 | [NOX4](http://www.gene.ucl.ac.uk/cgi-bin/nomenclature/searchgenes.pl?field=symbol&anchor=equals&symbol_search=Search&number=100&format=html&sortby=symbol&match=NOX4) |  | [605261](http://www.ncbi.nlm.nih.gov/entrez/dispomim.cgi?id=605261) | **-2.18** |
| 1451828_A_AT | acyl-CoA synthetase long-chain family member 4 | [ACSL4](http://www.gene.ucl.ac.uk/cgi-bin/nomenclature/searchgenes.pl?field=symbol&anchor=equals&symbol_search=Search&number=100&format=html&sortby=symbol&match=ACSL4) |  | [300157](http://www.ncbi.nlm.nih.gov/entrez/dispomim.cgi?id=300157) | **-1.75** |
| 1451841_A_AT | nuclear receptor co-repressor 2 | [NCOR2](http://www.gene.ucl.ac.uk/cgi-bin/nomenclature/searchgenes.pl?field=symbol&anchor=equals&symbol_search=Search&number=100&format=html&sortby=symbol&match=NCOR2) |  | [600848](http://www.ncbi.nlm.nih.gov/entrez/dispomim.cgi?id=600848) | **1.37** |
| 1451860_A_AT | tripartite motif protein 30 | [TRIM30](http://www.gene.ucl.ac.uk/cgi-bin/nomenclature/searchgenes.pl?field=symbol&anchor=equals&symbol_search=Search&number=100&format=html&sortby=symbol&match=TRIM30) |  |  | **-1.54** |
| 1451872_A_AT | neuralized-like homolog (Drosophila) | [NEURL](http://www.gene.ucl.ac.uk/cgi-bin/nomenclature/searchgenes.pl?field=symbol&anchor=equals&symbol_search=Search&number=100&format=html&sortby=symbol&match=NEURL) |  | [603804](http://www.ncbi.nlm.nih.gov/entrez/dispomim.cgi?id=603804) | **1.47** |
| 1451912_A_AT | fibroblast growth factor receptor-like 1 | [FGFRL1](http://www.gene.ucl.ac.uk/cgi-bin/nomenclature/searchgenes.pl?field=symbol&anchor=equals&symbol_search=Search&number=100&format=html&sortby=symbol&match=FGFRL1) |  | [605830](http://www.ncbi.nlm.nih.gov/entrez/dispomim.cgi?id=605830) | **-1.79** |
| 1451918_A_AT | loss of heterozygosity, 12, chromosomal region 1 homolog (human) | [LOH12CR1](http://www.gene.ucl.ac.uk/cgi-bin/nomenclature/searchgenes.pl?field=symbol&anchor=equals&symbol_search=Search&number=100&format=html&sortby=symbol&match=LOH12CR1) |  |  | **1.4** |
| 1451939_A_AT | sushi-repeat-containing protein | [SRPX](http://www.gene.ucl.ac.uk/cgi-bin/nomenclature/searchgenes.pl?field=symbol&anchor=equals&symbol_search=Search&number=100&format=html&sortby=symbol&match=SRPX) |  | [300187](http://www.ncbi.nlm.nih.gov/entrez/dispomim.cgi?id=300187) | **-1.57** |
| 1451941_A_AT | Fc receptor, IgG, low affinity IIb | [FCGR2B](http://www.gene.ucl.ac.uk/cgi-bin/nomenclature/searchgenes.pl?field=symbol&anchor=equals&symbol_search=Search&number=100&format=html&sortby=symbol&match=FCGR2B) |  | [604590](http://www.ncbi.nlm.nih.gov/entrez/dispomim.cgi?id=604590) | **-2.7** |
| 1451943_A_AT | protein phosphatase 1A, magnesium dependent, alpha isoform | [PPM1A](http://www.gene.ucl.ac.uk/cgi-bin/nomenclature/searchgenes.pl?field=symbol&anchor=equals&symbol_search=Search&number=100&format=html&sortby=symbol&match=PPM1A) |  | [606108](http://www.ncbi.nlm.nih.gov/entrez/dispomim.cgi?id=606108) | **1.66** |
| 1451959_A_AT | vascular endothelial growth factor A | [VEGFA](http://www.gene.ucl.ac.uk/cgi-bin/nomenclature/searchgenes.pl?field=symbol&anchor=equals&symbol_search=Search&number=100&format=html&sortby=symbol&match=VEGFA) |  |  | **1.69** |
| 1451961_A_AT | myelin basic protein | [MBP](http://www.gene.ucl.ac.uk/cgi-bin/nomenclature/searchgenes.pl?field=symbol&anchor=equals&symbol_search=Search&number=100&format=html&sortby=symbol&match=MBP) |  | [159430](http://www.ncbi.nlm.nih.gov/entrez/dispomim.cgi?id=159430) | **1.86** |
| 1451973_AT | ubiquitin-activating enzyme E1-domain containing 1 | [UBE1DC1](http://www.gene.ucl.ac.uk/cgi-bin/nomenclature/searchgenes.pl?field=symbol&anchor=equals&symbol_search=Search&number=100&format=html&sortby=symbol&match=UBE1DC1) |  |  | **-1.55** |
| 1451975_AT | RIKEN cDNA 2810453I06 gene | [2810453I06RIK](http://www.gene.ucl.ac.uk/cgi-bin/nomenclature/searchgenes.pl?field=symbol&anchor=equals&symbol_search=Search&number=100&format=html&sortby=symbol&match=2810453I06RIK) |  |  | **1.36** |
| 1451978_AT | lysyl oxidase-like 1 | [LOXL1](http://www.gene.ucl.ac.uk/cgi-bin/nomenclature/searchgenes.pl?field=symbol&anchor=equals&symbol_search=Search&number=100&format=html&sortby=symbol&match=LOXL1) |  | [153456](http://www.ncbi.nlm.nih.gov/entrez/dispomim.cgi?id=153456) | **-1.58** |
| 1451985_AT | Leucine-rich repeat kinase 1 | [LRRK1](http://www.gene.ucl.ac.uk/cgi-bin/nomenclature/searchgenes.pl?field=symbol&anchor=equals&symbol_search=Search&number=100&format=html&sortby=symbol&match=LRRK1) |  |  | **-1.45** |
| 1451987_AT | arrestin, beta 2 | [ARRB2](http://www.gene.ucl.ac.uk/cgi-bin/nomenclature/searchgenes.pl?field=symbol&anchor=equals&symbol_search=Search&number=100&format=html&sortby=symbol&match=ARRB2) |  | [107941](http://www.ncbi.nlm.nih.gov/entrez/dispomim.cgi?id=107941) | **-3.72** |
| 1451992_AT | adrenergic receptor kinase, beta 1 | [ADRBK1](http://www.gene.ucl.ac.uk/cgi-bin/nomenclature/searchgenes.pl?field=symbol&anchor=equals&symbol_search=Search&number=100&format=html&sortby=symbol&match=ADRBK1) |  | [109635](http://www.ncbi.nlm.nih.gov/entrez/dispomim.cgi?id=109635) | **1.21** |
| 1452005_AT | dihydrolipoamide S-acetyltransferase (E2 component of pyruvate dehydrogenase complex) | [DLAT](http://www.gene.ucl.ac.uk/cgi-bin/nomenclature/searchgenes.pl?field=symbol&anchor=equals&symbol_search=Search&number=100&format=html&sortby=symbol&match=DLAT) |  | [608770](http://www.ncbi.nlm.nih.gov/entrez/dispomim.cgi?id=608770) | **1.43** |
| 1452008_AT | RIKEN cDNA 9130422G05 gene | [9130422G05RIK](http://www.gene.ucl.ac.uk/cgi-bin/nomenclature/searchgenes.pl?field=symbol&anchor=equals&symbol_search=Search&number=100&format=html&sortby=symbol&match=9130422G05RIK) |  |  | **-1.8** |
| 1452014_A_AT | insulin-like growth factor 1 | [IGF1](http://www.gene.ucl.ac.uk/cgi-bin/nomenclature/searchgenes.pl?field=symbol&anchor=equals&symbol_search=Search&number=100&format=html&sortby=symbol&match=IGF1) |  | [147440](http://www.ncbi.nlm.nih.gov/entrez/dispomim.cgi?id=147440) | **-1.88** |
| 1452016_AT | arachidonate 5-lipoxygenase activating protein | [ALOX5AP](http://www.gene.ucl.ac.uk/cgi-bin/nomenclature/searchgenes.pl?field=symbol&anchor=equals&symbol_search=Search&number=100&format=html&sortby=symbol&match=ALOX5AP) |  | [603700](http://www.ncbi.nlm.nih.gov/entrez/dispomim.cgi?id=603700) | **-2.06** |
| 1452026_A_AT | phospholipase A2, group XIIA | [PLA2G12A](http://www.gene.ucl.ac.uk/cgi-bin/nomenclature/searchgenes.pl?field=symbol&anchor=equals&symbol_search=Search&number=100&format=html&sortby=symbol&match=PLA2G12A) |  |  | **1.97** |
| 1452027_A_AT | transformation related protein 63 | [TRP63](http://www.gene.ucl.ac.uk/cgi-bin/nomenclature/searchgenes.pl?field=symbol&anchor=equals&symbol_search=Search&number=100&format=html&sortby=symbol&match=TRP63) |  |  | **-2.2** |
| 1452037_AT | mannoside acetylglucosaminyltransferase 2 | [MGAT2](http://www.gene.ucl.ac.uk/cgi-bin/nomenclature/searchgenes.pl?field=symbol&anchor=equals&symbol_search=Search&number=100&format=html&sortby=symbol&match=MGAT2) |  | [602616](http://www.ncbi.nlm.nih.gov/entrez/dispomim.cgi?id=602616) | **-1.54** |
| 1452038_AT | capping protein (actin filament) muscle Z-line, alpha 1 | [CAPZA1](http://www.gene.ucl.ac.uk/cgi-bin/nomenclature/searchgenes.pl?field=symbol&anchor=equals&symbol_search=Search&number=100&format=html&sortby=symbol&match=CAPZA1) |  | [601580](http://www.ncbi.nlm.nih.gov/entrez/dispomim.cgi?id=601580) | **-1.57** |
| 1452044_AT | actin related protein 2/3 complex, subunit 5-like | [ARPC5L](http://www.gene.ucl.ac.uk/cgi-bin/nomenclature/searchgenes.pl?field=symbol&anchor=equals&symbol_search=Search&number=100&format=html&sortby=symbol&match=ARPC5L) |  |  | **1.32** |
| 1452049_AT | ribosomal protein L7-like 1 | [RPL7L1](http://www.gene.ucl.ac.uk/cgi-bin/nomenclature/searchgenes.pl?field=symbol&anchor=equals&symbol_search=Search&number=100&format=html&sortby=symbol&match=RPL7L1) |  |  | **-1.27** |
| 1452050_AT | calcium/calmodulin-dependent protein kinase ID | [CAMK1D](http://www.gene.ucl.ac.uk/cgi-bin/nomenclature/searchgenes.pl?field=symbol&anchor=equals&symbol_search=Search&number=100&format=html&sortby=symbol&match=CAMK1D) |  | [607957](http://www.ncbi.nlm.nih.gov/entrez/dispomim.cgi?id=607957) | **-2.09** |
| 1452051_AT | ARP3 actin-related protein 3 homolog (yeast) | [ACTR3](http://www.gene.ucl.ac.uk/cgi-bin/nomenclature/searchgenes.pl?field=symbol&anchor=equals&symbol_search=Search&number=100&format=html&sortby=symbol&match=ACTR3) |  | [604222](http://www.ncbi.nlm.nih.gov/entrez/dispomim.cgi?id=604222) | **-1.76** |
| 1452059_AT | solute carrier family 35, member F5 | [SLC35F5](http://www.gene.ucl.ac.uk/cgi-bin/nomenclature/searchgenes.pl?field=symbol&anchor=equals&symbol_search=Search&number=100&format=html&sortby=symbol&match=SLC35F5) |  |  | **-1.78** |
| 1452072_AT | myc target 1 | [MYCT1](http://www.gene.ucl.ac.uk/cgi-bin/nomenclature/searchgenes.pl?field=symbol&anchor=equals&symbol_search=Search&number=100&format=html&sortby=symbol&match=MYCT1) |  |  | **2.22** |
| 1452083_A_AT | praja1, RING-H2 motif containing | [PJA1](http://www.gene.ucl.ac.uk/cgi-bin/nomenclature/searchgenes.pl?field=symbol&anchor=equals&symbol_search=Search&number=100&format=html&sortby=symbol&match=PJA1) |  | [300420](http://www.ncbi.nlm.nih.gov/entrez/dispomim.cgi?id=300420) | **-1.51** |
| 1452087_AT | epithelial stromal interaction 1 (breast) | [EPSTI1](http://www.gene.ucl.ac.uk/cgi-bin/nomenclature/searchgenes.pl?field=symbol&anchor=equals&symbol_search=Search&number=100&format=html&sortby=symbol&match=EPSTI1) |  | [607441](http://www.ncbi.nlm.nih.gov/entrez/dispomim.cgi?id=607441) | **-2.11** |
| 1452101_AT | bleomycin hydrolase | [BLMH](http://www.gene.ucl.ac.uk/cgi-bin/nomenclature/searchgenes.pl?field=symbol&anchor=equals&symbol_search=Search&number=100&format=html&sortby=symbol&match=BLMH) |  | [602403](http://www.ncbi.nlm.nih.gov/entrez/dispomim.cgi?id=602403) | **-1.49** |
| 1452105_A_AT | tuberous sclerosis 2 | [TSC2](http://www.gene.ucl.ac.uk/cgi-bin/nomenclature/searchgenes.pl?field=symbol&anchor=equals&symbol_search=Search&number=100&format=html&sortby=symbol&match=TSC2) |  | [191092](http://www.ncbi.nlm.nih.gov/entrez/dispomim.cgi?id=191092) | **1.38** |
| 1452106_AT | gb:AA223007 /DB_XREF=gi:1843285 /DB_XREF=mw01g12.r |  |  |  | **-13.23** |
| 1452107_S_AT | nephronectin | [NPNT](http://www.gene.ucl.ac.uk/cgi-bin/nomenclature/searchgenes.pl?field=symbol&anchor=equals&symbol_search=Search&number=100&format=html&sortby=symbol&match=NPNT) |  |  | **-13.92** |
| 1452122_AT | expressed sequence AI314180 | [AI314180](http://www.gene.ucl.ac.uk/cgi-bin/nomenclature/searchgenes.pl?field=symbol&anchor=equals&symbol_search=Search&number=100&format=html&sortby=symbol&match=AI314180) |  |  | **1.35** |
| 1452124_AT | RIKEN cDNA 2900054D09 gene | [2900054D09RIK](http://www.gene.ucl.ac.uk/cgi-bin/nomenclature/searchgenes.pl?field=symbol&anchor=equals&symbol_search=Search&number=100&format=html&sortby=symbol&match=2900054D09RIK) |  |  | **-1.44** |
| 1452143_AT | Spectrin beta 2 | [SPNB2](http://www.gene.ucl.ac.uk/cgi-bin/nomenclature/searchgenes.pl?field=symbol&anchor=equals&symbol_search=Search&number=100&format=html&sortby=symbol&match=SPNB2) |  |  | **1.4** |
| 1452157_AT | Glutamyl-prolyl-tRNA synthetase | [EPRS](http://www.gene.ucl.ac.uk/cgi-bin/nomenclature/searchgenes.pl?field=symbol&anchor=equals&symbol_search=Search&number=100&format=html&sortby=symbol&match=EPRS) |  | [138295](http://www.ncbi.nlm.nih.gov/entrez/dispomim.cgi?id=138295) | **1.26** |
| 1452158_AT | glutamyl-prolyl-tRNA synthetase | [EPRS](http://www.gene.ucl.ac.uk/cgi-bin/nomenclature/searchgenes.pl?field=symbol&anchor=equals&symbol_search=Search&number=100&format=html&sortby=symbol&match=EPRS) |  | [138295](http://www.ncbi.nlm.nih.gov/entrez/dispomim.cgi?id=138295) | **1.23** |
| 1452159_AT | RIKEN cDNA 2310001A20 gene | [2310001A20RIK](http://www.gene.ucl.ac.uk/cgi-bin/nomenclature/searchgenes.pl?field=symbol&anchor=equals&symbol_search=Search&number=100&format=html&sortby=symbol&match=2310001A20RIK) |  |  | **1.32** |
| 1452161_AT | TCDD-inducible poly(ADP-ribose) polymerase | [TIPARP](http://www.gene.ucl.ac.uk/cgi-bin/nomenclature/searchgenes.pl?field=symbol&anchor=equals&symbol_search=Search&number=100&format=html&sortby=symbol&match=TIPARP) |  |  | **-1.39** |
| 1452163_AT | E26 avian leukemia oncogene 1, 5' domain | [ETS1](http://www.gene.ucl.ac.uk/cgi-bin/nomenclature/searchgenes.pl?field=symbol&anchor=equals&symbol_search=Search&number=100&format=html&sortby=symbol&match=ETS1) |  | [164720](http://www.ncbi.nlm.nih.gov/entrez/dispomim.cgi?id=164720) | **1.52** |
| 1452169_A_AT | diacylglycerol kinase zeta | [DGKZ](http://www.gene.ucl.ac.uk/cgi-bin/nomenclature/searchgenes.pl?field=symbol&anchor=equals&symbol_search=Search&number=100&format=html&sortby=symbol&match=DGKZ) |  | [601441](http://www.ncbi.nlm.nih.gov/entrez/dispomim.cgi?id=601441) | **-1.3** |
| 1452172_AT | RIKEN cDNA 2810421I24 gene | [2810421I24RIK](http://www.gene.ucl.ac.uk/cgi-bin/nomenclature/searchgenes.pl?field=symbol&anchor=equals&symbol_search=Search&number=100&format=html&sortby=symbol&match=2810421I24RIK) |  |  | **1.49** |
| 1452173_AT | hydroxyacyl-Coenzyme A dehydrogenase/3-ketoacyl-Coenzyme A thiolase/enoyl-Coenzyme A hydratase (trifunctional protein), alpha subunit | [HADHA](http://www.gene.ucl.ac.uk/cgi-bin/nomenclature/searchgenes.pl?field=symbol&anchor=equals&symbol_search=Search&number=100&format=html&sortby=symbol&match=HADHA) |  | [600890](http://www.ncbi.nlm.nih.gov/entrez/dispomim.cgi?id=600890) | **1.78** |
| 1452175_AT | RIKEN cDNA 1810026J23 gene | [1810026J23RIK](http://www.gene.ucl.ac.uk/cgi-bin/nomenclature/searchgenes.pl?field=symbol&anchor=equals&symbol_search=Search&number=100&format=html&sortby=symbol&match=1810026J23RIK) |  |  | **1.22** |
| 1452181_AT | Cytoskeleton-associated protein 4 | [CKAP4](http://www.gene.ucl.ac.uk/cgi-bin/nomenclature/searchgenes.pl?field=symbol&anchor=equals&symbol_search=Search&number=100&format=html&sortby=symbol&match=CKAP4) |  |  | **-1.6** |
| 1452183_A_AT | GTL2, imprinted maternally expressed untranslated mRNA | [GTL2](http://www.gene.ucl.ac.uk/cgi-bin/nomenclature/searchgenes.pl?field=symbol&anchor=equals&symbol_search=Search&number=100&format=html&sortby=symbol&match=GTL2) |  |  | **-2.45** |
| 1452184_AT | NADH dehydrogenase (ubiquinone) 1 beta subcomplex, 9 | [NDUFB9](http://www.gene.ucl.ac.uk/cgi-bin/nomenclature/searchgenes.pl?field=symbol&anchor=equals&symbol_search=Search&number=100&format=html&sortby=symbol&match=NDUFB9) |  | [601445](http://www.ncbi.nlm.nih.gov/entrez/dispomim.cgi?id=601445) | **1.25** |
| 1452190_AT | RIKEN cDNA 2510048K03 gene | [PRCP](http://www.gene.ucl.ac.uk/cgi-bin/nomenclature/searchgenes.pl?field=symbol&anchor=equals&symbol_search=Search&number=100&format=html&sortby=symbol&match=PRCP) |  | [176785](http://www.ncbi.nlm.nih.gov/entrez/dispomim.cgi?id=176785) | **-1.45** |
| 1452197_AT | SMC4 structural maintenance of chromosomes 4-like 1 (yeast) | [SMC4L1](http://www.gene.ucl.ac.uk/cgi-bin/nomenclature/searchgenes.pl?field=symbol&anchor=equals&symbol_search=Search&number=100&format=html&sortby=symbol&match=SMC4L1) |  | [605575](http://www.ncbi.nlm.nih.gov/entrez/dispomim.cgi?id=605575) | **-1.82** |
| 1452204_AT | ankyrin repeat and SAM domain containing 1 | [ANKS1](http://www.gene.ucl.ac.uk/cgi-bin/nomenclature/searchgenes.pl?field=symbol&anchor=equals&symbol_search=Search&number=100&format=html&sortby=symbol&match=ANKS1) |  | [608994](http://www.ncbi.nlm.nih.gov/entrez/dispomim.cgi?id=608994) | **1.5** |
| 1452206_AT | succinate-Coenzyme A ligase, ADP-forming, beta subunit | [SUCLA2](http://www.gene.ucl.ac.uk/cgi-bin/nomenclature/searchgenes.pl?field=symbol&anchor=equals&symbol_search=Search&number=100&format=html&sortby=symbol&match=SUCLA2) |  | [603921](http://www.ncbi.nlm.nih.gov/entrez/dispomim.cgi?id=603921) | **1.2** |
| 1452210_AT | DNA2 DNA replication helicase 2-like (yeast) | [DNA2L](http://www.gene.ucl.ac.uk/cgi-bin/nomenclature/searchgenes.pl?field=symbol&anchor=equals&symbol_search=Search&number=100&format=html&sortby=symbol&match=DNA2L) |  | [601810](http://www.ncbi.nlm.nih.gov/entrez/dispomim.cgi?id=601810) | **-1.59** |
| 1452213_AT | testis expressed gene 2 | [TEX2](http://www.gene.ucl.ac.uk/cgi-bin/nomenclature/searchgenes.pl?field=symbol&anchor=equals&symbol_search=Search&number=100&format=html&sortby=symbol&match=TEX2) |  |  | **1.83** |
| 1452224_AT | zinc finger, CW-type with coiled-coil domain 3 | [ZCWCC3](http://www.gene.ucl.ac.uk/cgi-bin/nomenclature/searchgenes.pl?field=symbol&anchor=equals&symbol_search=Search&number=100&format=html&sortby=symbol&match=ZCWCC3) |  |  | **-1.28** |
| 1452231_X_AT | interferon activated gene 203 | [IFI203](http://www.gene.ucl.ac.uk/cgi-bin/nomenclature/searchgenes.pl?field=symbol&anchor=equals&symbol_search=Search&number=100&format=html&sortby=symbol&match=IFI203) |  |  | **-1.49** |
| 1452241_AT | topoisomerase (DNA) II beta binding protein | [TOPBP1](http://www.gene.ucl.ac.uk/cgi-bin/nomenclature/searchgenes.pl?field=symbol&anchor=equals&symbol_search=Search&number=100&format=html&sortby=symbol&match=TOPBP1) |  | [607760](http://www.ncbi.nlm.nih.gov/entrez/dispomim.cgi?id=607760) | **-1.35** |
| 1452242_AT | RIKEN cDNA 1200008O12 gene | [1200008O12RIK](http://www.gene.ucl.ac.uk/cgi-bin/nomenclature/searchgenes.pl?field=symbol&anchor=equals&symbol_search=Search&number=100&format=html&sortby=symbol&match=1200008O12RIK) |  |  | **-2.01** |
| 1452250_A_AT | procollagen, type VI, alpha 2 | [COL6A2](http://www.gene.ucl.ac.uk/cgi-bin/nomenclature/searchgenes.pl?field=symbol&anchor=equals&symbol_search=Search&number=100&format=html&sortby=symbol&match=COL6A2) |  | [120240](http://www.ncbi.nlm.nih.gov/entrez/dispomim.cgi?id=120240) | **-2.03** |
| 1452253_AT | cysteine-rich motor neuron 1 | [CRIM1](http://www.gene.ucl.ac.uk/cgi-bin/nomenclature/searchgenes.pl?field=symbol&anchor=equals&symbol_search=Search&number=100&format=html&sortby=symbol&match=CRIM1) |  | [606189](http://www.ncbi.nlm.nih.gov/entrez/dispomim.cgi?id=606189) | **1.93** |
| 1452257_AT | 3-hydroxybutyrate dehydrogenase (heart, mitochondrial) | [BDH](http://www.gene.ucl.ac.uk/cgi-bin/nomenclature/searchgenes.pl?field=symbol&anchor=equals&symbol_search=Search&number=100&format=html&sortby=symbol&match=BDH) |  | [603063](http://www.ncbi.nlm.nih.gov/entrez/dispomim.cgi?id=603063) | **2.96** |
| 1452262_AT | GrpE-like 2, mitochondrial | [GRPEL2](http://www.gene.ucl.ac.uk/cgi-bin/nomenclature/searchgenes.pl?field=symbol&anchor=equals&symbol_search=Search&number=100&format=html&sortby=symbol&match=GRPEL2) |  |  | **-1.25** |
| 1452264_AT | tensin like C1 domain-containing phosphatase | [TENC1](http://www.gene.ucl.ac.uk/cgi-bin/nomenclature/searchgenes.pl?field=symbol&anchor=equals&symbol_search=Search&number=100&format=html&sortby=symbol&match=TENC1) |  | [607717](http://www.ncbi.nlm.nih.gov/entrez/dispomim.cgi?id=607717) | **1.4** |
| 1452265_AT | CLIP associating protein 1 | [CLASP1](http://www.gene.ucl.ac.uk/cgi-bin/nomenclature/searchgenes.pl?field=symbol&anchor=equals&symbol_search=Search&number=100&format=html&sortby=symbol&match=CLASP1) |  | [605852](http://www.ncbi.nlm.nih.gov/entrez/dispomim.cgi?id=605852) | **-1.28** |
| 1452281_AT | Son of sevenless homolog 2 (Drosophila) | [SOS2](http://www.gene.ucl.ac.uk/cgi-bin/nomenclature/searchgenes.pl?field=symbol&anchor=equals&symbol_search=Search&number=100&format=html&sortby=symbol&match=SOS2) |  | [601247](http://www.ncbi.nlm.nih.gov/entrez/dispomim.cgi?id=601247) | **1.26** |
| 1452284_AT | Protein tyrosine phosphatase, receptor type Z, polypeptide 1 | [PTPRZ1](http://www.gene.ucl.ac.uk/cgi-bin/nomenclature/searchgenes.pl?field=symbol&anchor=equals&symbol_search=Search&number=100&format=html&sortby=symbol&match=PTPRZ1) |  | [176891](http://www.ncbi.nlm.nih.gov/entrez/dispomim.cgi?id=176891) | **1.8** |
| 1452285_A_AT | eukaryotic translation initiation factor 3, subunit 5 (epsilon) | [EIF3S5](http://www.gene.ucl.ac.uk/cgi-bin/nomenclature/searchgenes.pl?field=symbol&anchor=equals&symbol_search=Search&number=100&format=html&sortby=symbol&match=EIF3S5) |  | [603914](http://www.ncbi.nlm.nih.gov/entrez/dispomim.cgi?id=603914) | **1.21** |
| 1452291_AT | centaurin, delta 1 | [CENTD1](http://www.gene.ucl.ac.uk/cgi-bin/nomenclature/searchgenes.pl?field=symbol&anchor=equals&symbol_search=Search&number=100&format=html&sortby=symbol&match=CENTD1) |  | [606645](http://www.ncbi.nlm.nih.gov/entrez/dispomim.cgi?id=606645) | **1.85** |
| 1452298_A_AT | myosin Vb | [MYO5B](http://www.gene.ucl.ac.uk/cgi-bin/nomenclature/searchgenes.pl?field=symbol&anchor=equals&symbol_search=Search&number=100&format=html&sortby=symbol&match=MYO5B) |  | [606540](http://www.ncbi.nlm.nih.gov/entrez/dispomim.cgi?id=606540) | **1.6** |
| 1452299_AT | WW domain containing E3 ubiquitin protein ligase 1 | [WWP1](http://www.gene.ucl.ac.uk/cgi-bin/nomenclature/searchgenes.pl?field=symbol&anchor=equals&symbol_search=Search&number=100&format=html&sortby=symbol&match=WWP1) |  | [602307](http://www.ncbi.nlm.nih.gov/entrez/dispomim.cgi?id=602307) | **1.46** |
| 1452308_A_AT | ATPase, Na+/K+ transporting, alpha 2 polypeptide | [ATP1A2](http://www.gene.ucl.ac.uk/cgi-bin/nomenclature/searchgenes.pl?field=symbol&anchor=equals&symbol_search=Search&number=100&format=html&sortby=symbol&match=ATP1A2) |  | [182340](http://www.ncbi.nlm.nih.gov/entrez/dispomim.cgi?id=182340) | **1.37** |
| 1452309_AT | RIKEN cDNA 4933421H10 gene | [4933421H10RIK](http://www.gene.ucl.ac.uk/cgi-bin/nomenclature/searchgenes.pl?field=symbol&anchor=equals&symbol_search=Search&number=100&format=html&sortby=symbol&match=4933421H10RIK) |  |  | **1.39** |
| 1452321_AT | WD repeat domain 9 | [WDR9](http://www.gene.ucl.ac.uk/cgi-bin/nomenclature/searchgenes.pl?field=symbol&anchor=equals&symbol_search=Search&number=100&format=html&sortby=symbol&match=WDR9) |  |  | **-1.36** |
| 1452327_AT | DNA segment, Chr 6, ERATO Doi 349, expressed | [IQSEC1](http://www.gene.ucl.ac.uk/cgi-bin/nomenclature/searchgenes.pl?field=symbol&anchor=equals&symbol_search=Search&number=100&format=html&sortby=symbol&match=IQSEC1) |  |  | **1.39** |
| 1452330_A_AT | RIKEN cDNA 1200013A08 gene | [1200013A08RIK](http://www.gene.ucl.ac.uk/cgi-bin/nomenclature/searchgenes.pl?field=symbol&anchor=equals&symbol_search=Search&number=100&format=html&sortby=symbol&match=1200013A08RIK) |  |  | **-1.7** |
| 1452331_S_AT | RIKEN cDNA 4732486I23 gene | [4732486I23RIK](http://www.gene.ucl.ac.uk/cgi-bin/nomenclature/searchgenes.pl?field=symbol&anchor=equals&symbol_search=Search&number=100&format=html&sortby=symbol&match=4732486I23RIK) |  |  | **1.52** |
| 1452335_AT | RIKEN cDNA 2810423E13 gene | [2810423E13RIK](http://www.gene.ucl.ac.uk/cgi-bin/nomenclature/searchgenes.pl?field=symbol&anchor=equals&symbol_search=Search&number=100&format=html&sortby=symbol&match=2810423E13RIK) |  |  | **1.43** |
| 1452338_S_AT | intersectin 1 (SH3 domain protein 1A) | [ITSN1](http://www.gene.ucl.ac.uk/cgi-bin/nomenclature/searchgenes.pl?field=symbol&anchor=equals&symbol_search=Search&number=100&format=html&sortby=symbol&match=ITSN1) |  | [602442](http://www.ncbi.nlm.nih.gov/entrez/dispomim.cgi?id=602442) | **1.26** |
| 1452341_AT | enoyl Coenzyme A hydratase, short chain, 1, mitochondrial | [ECHS1](http://www.gene.ucl.ac.uk/cgi-bin/nomenclature/searchgenes.pl?field=symbol&anchor=equals&symbol_search=Search&number=100&format=html&sortby=symbol&match=ECHS1) |  | [602292](http://www.ncbi.nlm.nih.gov/entrez/dispomim.cgi?id=602292) | **1.71** |
| 1452348_S_AT | gb:AI481797 /DB_XREF=gi:4375023 /DB_XREF=vf99b08.x |  |  |  | **-1.82** |
| 1452349_X_AT | interferon activated gene 205 | [IFI205](http://www.gene.ucl.ac.uk/cgi-bin/nomenclature/searchgenes.pl?field=symbol&anchor=equals&symbol_search=Search&number=100&format=html&sortby=symbol&match=IFI205) |  |  | **-1.96** |
| 1452357_AT | glycoprotein Ib, beta polypeptide | [GP1BB](http://www.gene.ucl.ac.uk/cgi-bin/nomenclature/searchgenes.pl?field=symbol&anchor=equals&symbol_search=Search&number=100&format=html&sortby=symbol&match=GP1BB) |  | [138720](http://www.ncbi.nlm.nih.gov/entrez/dispomim.cgi?id=138720) | **-1.58** |
| 1452366_AT | RIKEN cDNA 4732435N03 gene | [4732435N03RIK](http://www.gene.ucl.ac.uk/cgi-bin/nomenclature/searchgenes.pl?field=symbol&anchor=equals&symbol_search=Search&number=100&format=html&sortby=symbol&match=4732435N03RIK) |  |  | **-1.49** |
| 1452374_AT | zinc finger protein 322a | [ZFP322A](http://www.gene.ucl.ac.uk/cgi-bin/nomenclature/searchgenes.pl?field=symbol&anchor=equals&symbol_search=Search&number=100&format=html&sortby=symbol&match=ZFP322A) |  |  | **-1.47** |
| 1452375_AT | aldehyde dehydrogenase 4 family, member A1 | [ALDH4A1](http://www.gene.ucl.ac.uk/cgi-bin/nomenclature/searchgenes.pl?field=symbol&anchor=equals&symbol_search=Search&number=100&format=html&sortby=symbol&match=ALDH4A1) |  | [606811](http://www.ncbi.nlm.nih.gov/entrez/dispomim.cgi?id=606811) | **1.77** |
| 1452382_AT | neoplastic progression 1 | [NPN1](http://www.gene.ucl.ac.uk/cgi-bin/nomenclature/searchgenes.pl?field=symbol&anchor=equals&symbol_search=Search&number=100&format=html&sortby=symbol&match=NPN1) |  |  | **-4.84** |
| 1452404_AT | Phosphatase and actin regulator 2 | [PHACTR2](http://www.gene.ucl.ac.uk/cgi-bin/nomenclature/searchgenes.pl?field=symbol&anchor=equals&symbol_search=Search&number=100&format=html&sortby=symbol&match=PHACTR2) |  | [608724](http://www.ncbi.nlm.nih.gov/entrez/dispomim.cgi?id=608724) | **1.35** |
| 1452406_X_AT | erythroid differentiation regulator 1 | [ERDR1](http://www.gene.ucl.ac.uk/cgi-bin/nomenclature/searchgenes.pl?field=symbol&anchor=equals&symbol_search=Search&number=100&format=html&sortby=symbol&match=ERDR1) |  |  | **5.02** |
| 1452415_AT | Actinin, alpha 1 | [ACTN1](http://www.gene.ucl.ac.uk/cgi-bin/nomenclature/searchgenes.pl?field=symbol&anchor=equals&symbol_search=Search&number=100&format=html&sortby=symbol&match=ACTN1) |  |  | **-2.13** |
| 1452418_AT | RIKEN cDNA 1200016E24 gene | [1200016E24RIK](http://www.gene.ucl.ac.uk/cgi-bin/nomenclature/searchgenes.pl?field=symbol&anchor=equals&symbol_search=Search&number=100&format=html&sortby=symbol&match=1200016E24RIK) |  |  | **1.47** |
| 1452428_A_AT | beta-2 microglobulin | [B2M](http://www.gene.ucl.ac.uk/cgi-bin/nomenclature/searchgenes.pl?field=symbol&anchor=equals&symbol_search=Search&number=100&format=html&sortby=symbol&match=B2M) |  | [109700](http://www.ncbi.nlm.nih.gov/entrez/dispomim.cgi?id=109700) | **-1.22** |
| 1452431_S_AT | histocompatibility 2, class II antigen A, alpha | [H2-AA](http://www.gene.ucl.ac.uk/cgi-bin/nomenclature/searchgenes.pl?field=symbol&anchor=equals&symbol_search=Search&number=100&format=html&sortby=symbol&match=H2-AA) |  |  | **-2.96** |
| 1452442_AT | Ubiquitin specific protease 13 (isopeptidase T-3) | [USP13](http://www.gene.ucl.ac.uk/cgi-bin/nomenclature/searchgenes.pl?field=symbol&anchor=equals&symbol_search=Search&number=100&format=html&sortby=symbol&match=USP13) |  | [603591](http://www.ncbi.nlm.nih.gov/entrez/dispomim.cgi?id=603591) | **1.63** |
| 1452445_AT | solute carrier family 41, member 2 | [SLC41A2](http://www.gene.ucl.ac.uk/cgi-bin/nomenclature/searchgenes.pl?field=symbol&anchor=equals&symbol_search=Search&number=100&format=html&sortby=symbol&match=SLC41A2) |  |  | **-9.13** |
| 1452464_A_AT | methionine aminopeptidase-like 1 | [METAPL1](http://www.gene.ucl.ac.uk/cgi-bin/nomenclature/searchgenes.pl?field=symbol&anchor=equals&symbol_search=Search&number=100&format=html&sortby=symbol&match=METAPL1) |  |  | **1.32** |
| 1452467_AT | methylmalonic aciduria (cobalamin deficiency) type B homolog (human) | [MMAB](http://www.gene.ucl.ac.uk/cgi-bin/nomenclature/searchgenes.pl?field=symbol&anchor=equals&symbol_search=Search&number=100&format=html&sortby=symbol&match=MMAB) |  | [607568](http://www.ncbi.nlm.nih.gov/entrez/dispomim.cgi?id=607568) | **1.49** |
| 1452478_AT | Heart alpha-kinase | [ALPK2](http://www.gene.ucl.ac.uk/cgi-bin/nomenclature/searchgenes.pl?field=symbol&anchor=equals&symbol_search=Search&number=100&format=html&sortby=symbol&match=ALPK2) |  |  | **1.77** |
| 1452483_A_AT | CD44 antigen | [CD44](http://www.gene.ucl.ac.uk/cgi-bin/nomenclature/searchgenes.pl?field=symbol&anchor=equals&symbol_search=Search&number=100&format=html&sortby=symbol&match=CD44) |  | [107269](http://www.ncbi.nlm.nih.gov/entrez/dispomim.cgi?id=107269) | **-4.41** |
| 1452485_AT | phosphatase, orphan 1 | [PHOSPHO1](http://www.gene.ucl.ac.uk/cgi-bin/nomenclature/searchgenes.pl?field=symbol&anchor=equals&symbol_search=Search&number=100&format=html&sortby=symbol&match=PHOSPHO1) |  |  | **1.88** |
| 1452520_A_AT | cholinergic receptor, nicotinic, gamma polypeptide | [CHRNG](http://www.gene.ucl.ac.uk/cgi-bin/nomenclature/searchgenes.pl?field=symbol&anchor=equals&symbol_search=Search&number=100&format=html&sortby=symbol&match=CHRNG) |  | [100730](http://www.ncbi.nlm.nih.gov/entrez/dispomim.cgi?id=100730) | **-41.44** |
| 1452527_A_AT | purinergic receptor P2X, ligand-gated ion channel 4 | [P2RX4](http://www.gene.ucl.ac.uk/cgi-bin/nomenclature/searchgenes.pl?field=symbol&anchor=equals&symbol_search=Search&number=100&format=html&sortby=symbol&match=P2RX4) |  | [600846](http://www.ncbi.nlm.nih.gov/entrez/dispomim.cgi?id=600846) | **-1.76** |
| 1452534_A_AT | High mobility group box 2 | [HMGB2](http://www.gene.ucl.ac.uk/cgi-bin/nomenclature/searchgenes.pl?field=symbol&anchor=equals&symbol_search=Search&number=100&format=html&sortby=symbol&match=HMGB2) |  | [163906](http://www.ncbi.nlm.nih.gov/entrez/dispomim.cgi?id=163906) | **-1.71** |
| 1452545_A_AT | integrin beta 1 (fibronectin receptor beta) | [ITGB1](http://www.gene.ucl.ac.uk/cgi-bin/nomenclature/searchgenes.pl?field=symbol&anchor=equals&symbol_search=Search&number=100&format=html&sortby=symbol&match=ITGB1) |  | [135630](http://www.ncbi.nlm.nih.gov/entrez/dispomim.cgi?id=135630) | **-1.49** |
| 1452579_AT | RIKEN cDNA 2310020H20 gene | [2310020H20RIK](http://www.gene.ucl.ac.uk/cgi-bin/nomenclature/searchgenes.pl?field=symbol&anchor=equals&symbol_search=Search&number=100&format=html&sortby=symbol&match=2310020H20RIK) |  |  | **1.54** |
| 1452581_AT | RIKEN cDNA 2610304G08 gene | [2610304G08RIK](http://www.gene.ucl.ac.uk/cgi-bin/nomenclature/searchgenes.pl?field=symbol&anchor=equals&symbol_search=Search&number=100&format=html&sortby=symbol&match=2610304G08RIK) |  |  | **1.37** |
| 1452585_AT | mitochondrial ribosomal protein S28 | [MRPS28](http://www.gene.ucl.ac.uk/cgi-bin/nomenclature/searchgenes.pl?field=symbol&anchor=equals&symbol_search=Search&number=100&format=html&sortby=symbol&match=MRPS28) |  |  | **1.2** |
| 1452587_AT | ARP2 actin-related protein 2 homolog (yeast) | [ACTR2](http://www.gene.ucl.ac.uk/cgi-bin/nomenclature/searchgenes.pl?field=symbol&anchor=equals&symbol_search=Search&number=100&format=html&sortby=symbol&match=ACTR2) |  | [604221](http://www.ncbi.nlm.nih.gov/entrez/dispomim.cgi?id=604221) | **-1.22** |
| 1452590_A_AT | hypothetical LOC211623 | [MGC41689](http://www.gene.ucl.ac.uk/cgi-bin/nomenclature/searchgenes.pl?field=symbol&anchor=equals&symbol_search=Search&number=100&format=html&sortby=symbol&match=MGC41689) |  |  | **3.08** |
| 1452607_AT | RIKEN cDNA 2610030H06 gene | [2610030H06RIK](http://www.gene.ucl.ac.uk/cgi-bin/nomenclature/searchgenes.pl?field=symbol&anchor=equals&symbol_search=Search&number=100&format=html&sortby=symbol&match=2610030H06RIK) |  |  | **-1.41** |
| 1452615_S_AT | tRNA splicing 2' phosphotransferase 1 homolog (S. cerevisiae) | [TPT1H](http://www.gene.ucl.ac.uk/cgi-bin/nomenclature/searchgenes.pl?field=symbol&anchor=equals&symbol_search=Search&number=100&format=html&sortby=symbol&match=TPT1H) |  |  | **1.48** |
| 1452628_AT | RIKEN cDNA 2810002N01 gene | [BAG5](http://www.gene.ucl.ac.uk/cgi-bin/nomenclature/searchgenes.pl?field=symbol&anchor=equals&symbol_search=Search&number=100&format=html&sortby=symbol&match=BAG5) |  | [603885](http://www.ncbi.nlm.nih.gov/entrez/dispomim.cgi?id=603885) | **1.4** |
| 1452661_AT | transferrin receptor | [TFRC](http://www.gene.ucl.ac.uk/cgi-bin/nomenclature/searchgenes.pl?field=symbol&anchor=equals&symbol_search=Search&number=100&format=html&sortby=symbol&match=TFRC) |  | [190010](http://www.ncbi.nlm.nih.gov/entrez/dispomim.cgi?id=190010) | **-2.54** |
| 1452664_A_AT | Transmembrane 7 superfamily member 3 | [TM7SF3](http://www.gene.ucl.ac.uk/cgi-bin/nomenclature/searchgenes.pl?field=symbol&anchor=equals&symbol_search=Search&number=100&format=html&sortby=symbol&match=TM7SF3) |  | [605181](http://www.ncbi.nlm.nih.gov/entrez/dispomim.cgi?id=605181) | **-1.28** |
| 1452666_A_AT | RIKEN cDNA 1110063G11 gene | [1110063G11RIK](http://www.gene.ucl.ac.uk/cgi-bin/nomenclature/searchgenes.pl?field=symbol&anchor=equals&symbol_search=Search&number=100&format=html&sortby=symbol&match=1110063G11RIK) |  |  | **1.53** |
| 1452669_AT | RIKEN cDNA 2810012G03 gene | [2810012G03RIK](http://www.gene.ucl.ac.uk/cgi-bin/nomenclature/searchgenes.pl?field=symbol&anchor=equals&symbol_search=Search&number=100&format=html&sortby=symbol&match=2810012G03RIK) |  |  | **-1.3** |
| 1452670_AT | Myosin, light polypeptide 9, regulatory | [MYL9](http://www.gene.ucl.ac.uk/cgi-bin/nomenclature/searchgenes.pl?field=symbol&anchor=equals&symbol_search=Search&number=100&format=html&sortby=symbol&match=MYL9) |  |  | **1.9** |
| 1452679_AT | RIKEN cDNA 2410129E14 gene | [2410129E14RIK](http://www.gene.ucl.ac.uk/cgi-bin/nomenclature/searchgenes.pl?field=symbol&anchor=equals&symbol_search=Search&number=100&format=html&sortby=symbol&match=2410129E14RIK) |  |  | **-7.65** |
| 1452684_AT | AKT1 substrate 1 (proline-rich) | [AKT1S1](http://www.gene.ucl.ac.uk/cgi-bin/nomenclature/searchgenes.pl?field=symbol&anchor=equals&symbol_search=Search&number=100&format=html&sortby=symbol&match=AKT1S1) |  |  | **1.59** |
| 1452685_AT | RIKEN cDNA 0610009O20 gene | [0610009O20RIK](http://www.gene.ucl.ac.uk/cgi-bin/nomenclature/searchgenes.pl?field=symbol&anchor=equals&symbol_search=Search&number=100&format=html&sortby=symbol&match=0610009O20RIK) |  |  | **1.49** |
| 1452691_AT | RNA binding motif protein 17 | [RBM17](http://www.gene.ucl.ac.uk/cgi-bin/nomenclature/searchgenes.pl?field=symbol&anchor=equals&symbol_search=Search&number=100&format=html&sortby=symbol&match=RBM17) |  | [606935](http://www.ncbi.nlm.nih.gov/entrez/dispomim.cgi?id=606935) | **-1.32** |
| 1452692_A_AT | NADH dehydrogenase (ubiquinone) flavoprotein 2 | [NDUFV2](http://www.gene.ucl.ac.uk/cgi-bin/nomenclature/searchgenes.pl?field=symbol&anchor=equals&symbol_search=Search&number=100&format=html&sortby=symbol&match=NDUFV2) |  | [600532](http://www.ncbi.nlm.nih.gov/entrez/dispomim.cgi?id=600532) | **1.23** |
| 1452709_AT | polymerase (DNA-directed), delta interacting protein 3 | [POLDIP3](http://www.gene.ucl.ac.uk/cgi-bin/nomenclature/searchgenes.pl?field=symbol&anchor=equals&symbol_search=Search&number=100&format=html&sortby=symbol&match=POLDIP3) |  |  | **-1.27** |
| 1452714_AT | RIKEN cDNA 1200003E16 gene | [1200003E16RIK](http://www.gene.ucl.ac.uk/cgi-bin/nomenclature/searchgenes.pl?field=symbol&anchor=equals&symbol_search=Search&number=100&format=html&sortby=symbol&match=1200003E16RIK) |  |  | **1.35** |
| 1452716_AT | RIKEN cDNA 5730469M10 gene | [5730469M10RIK](http://www.gene.ucl.ac.uk/cgi-bin/nomenclature/searchgenes.pl?field=symbol&anchor=equals&symbol_search=Search&number=100&format=html&sortby=symbol&match=5730469M10RIK) |  |  | **2.35** |
| 1452717_AT | solute carrier family 25 (mitochondrial carrier, phosphate carrier), member 24 | [SLC25A24](http://www.gene.ucl.ac.uk/cgi-bin/nomenclature/searchgenes.pl?field=symbol&anchor=equals&symbol_search=Search&number=100&format=html&sortby=symbol&match=SLC25A24) |  | [608744](http://www.ncbi.nlm.nih.gov/entrez/dispomim.cgi?id=608744) | **-3.75** |
| 1452727_AT | RIKEN cDNA 1300003K24 gene | [1300003K24RIK](http://www.gene.ucl.ac.uk/cgi-bin/nomenclature/searchgenes.pl?field=symbol&anchor=equals&symbol_search=Search&number=100&format=html&sortby=symbol&match=1300003K24RIK) |  |  | **1.26** |
| 1452739_AT | F-box only protein 7 | [FBXO7](http://www.gene.ucl.ac.uk/cgi-bin/nomenclature/searchgenes.pl?field=symbol&anchor=equals&symbol_search=Search&number=100&format=html&sortby=symbol&match=FBXO7) |  | [605648](http://www.ncbi.nlm.nih.gov/entrez/dispomim.cgi?id=605648) | **1.4** |
| 1452740_AT | myosin heavy chain 10, non-muscle | [MYH10](http://www.gene.ucl.ac.uk/cgi-bin/nomenclature/searchgenes.pl?field=symbol&anchor=equals&symbol_search=Search&number=100&format=html&sortby=symbol&match=MYH10) |  | [160776](http://www.ncbi.nlm.nih.gov/entrez/dispomim.cgi?id=160776) | **-1.51** |
| 1452751_AT | RIKEN cDNA 3110018A08 gene | [3110018A08RIK](http://www.gene.ucl.ac.uk/cgi-bin/nomenclature/searchgenes.pl?field=symbol&anchor=equals&symbol_search=Search&number=100&format=html&sortby=symbol&match=3110018A08RIK) |  |  | **1.43** |
| 1452757_S_AT | gb:AK011116.1 /DB_XREF=gi:12847032 /GEN=Hba-a1 /FE |  |  |  | **2.32** |
| 1452759_S_AT | PTPRF interacting protein, binding protein 1 (liprin beta 1) | [PPFIBP1](http://www.gene.ucl.ac.uk/cgi-bin/nomenclature/searchgenes.pl?field=symbol&anchor=equals&symbol_search=Search&number=100&format=html&sortby=symbol&match=PPFIBP1) |  | [603141](http://www.ncbi.nlm.nih.gov/entrez/dispomim.cgi?id=603141) | **-1.75** |
| 1452766_AT | RIKEN cDNA 2900041A09 gene | [2900041A09RIK](http://www.gene.ucl.ac.uk/cgi-bin/nomenclature/searchgenes.pl?field=symbol&anchor=equals&symbol_search=Search&number=100&format=html&sortby=symbol&match=2900041A09RIK) |  |  | **2.23** |
| 1452768_AT | testis expressed gene 261 | [TEX261](http://www.gene.ucl.ac.uk/cgi-bin/nomenclature/searchgenes.pl?field=symbol&anchor=equals&symbol_search=Search&number=100&format=html&sortby=symbol&match=TEX261) |  |  | **1.39** |
| 1452770_AT | vitamin K epoxide reductase complex, subunit 1 | [VKORC1](http://www.gene.ucl.ac.uk/cgi-bin/nomenclature/searchgenes.pl?field=symbol&anchor=equals&symbol_search=Search&number=100&format=html&sortby=symbol&match=VKORC1) |  | [608547](http://www.ncbi.nlm.nih.gov/entrez/dispomim.cgi?id=608547) | **-1.56** |
| 1452771_S_AT | acyl-CoA synthetase long-chain family member 3 | [ACSL3](http://www.gene.ucl.ac.uk/cgi-bin/nomenclature/searchgenes.pl?field=symbol&anchor=equals&symbol_search=Search&number=100&format=html&sortby=symbol&match=ACSL3) |  | [602371](http://www.ncbi.nlm.nih.gov/entrez/dispomim.cgi?id=602371) | **-1.83** |
| 1452778_X_AT | nucleosome assembly protein 1-like 1 | [NAP1L1](http://www.gene.ucl.ac.uk/cgi-bin/nomenclature/searchgenes.pl?field=symbol&anchor=equals&symbol_search=Search&number=100&format=html&sortby=symbol&match=NAP1L1) |  | [164060](http://www.ncbi.nlm.nih.gov/entrez/dispomim.cgi?id=164060) | **-1.45** |
| 1452782_A_AT | thioredoxin 2 | [TXN2](http://www.gene.ucl.ac.uk/cgi-bin/nomenclature/searchgenes.pl?field=symbol&anchor=equals&symbol_search=Search&number=100&format=html&sortby=symbol&match=TXN2) |  | [609063](http://www.ncbi.nlm.nih.gov/entrez/dispomim.cgi?id=609063) | **1.22** |
| 1452783_AT | RIKEN cDNA 1600019O04 gene | [1600019O04RIK](http://www.gene.ucl.ac.uk/cgi-bin/nomenclature/searchgenes.pl?field=symbol&anchor=equals&symbol_search=Search&number=100&format=html&sortby=symbol&match=1600019O04RIK) |  |  | **-1.5** |
| 1452784_AT | integrin alpha V | [ITGAV](http://www.gene.ucl.ac.uk/cgi-bin/nomenclature/searchgenes.pl?field=symbol&anchor=equals&symbol_search=Search&number=100&format=html&sortby=symbol&match=ITGAV) |  | [193210](http://www.ncbi.nlm.nih.gov/entrez/dispomim.cgi?id=193210) | **-1.42** |
| 1452785_AT | RIKEN cDNA 1700034H14 gene | [1700034H14RIK](http://www.gene.ucl.ac.uk/cgi-bin/nomenclature/searchgenes.pl?field=symbol&anchor=equals&symbol_search=Search&number=100&format=html&sortby=symbol&match=1700034H14RIK) |  |  | **1.31** |
| 1452787_A_AT | heterogeneous nuclear ribonucleoproteins methyltransferase-like 2 (S. cerevisiae) | [HRMT1L2](http://www.gene.ucl.ac.uk/cgi-bin/nomenclature/searchgenes.pl?field=symbol&anchor=equals&symbol_search=Search&number=100&format=html&sortby=symbol&match=HRMT1L2) |  | [602950](http://www.ncbi.nlm.nih.gov/entrez/dispomim.cgi?id=602950) | **-1.51** |
| 1452790_X_AT | NADH dehydrogenase (ubiquinone) 1 alpha subcomplex, 3 | [NDUFA3](http://www.gene.ucl.ac.uk/cgi-bin/nomenclature/searchgenes.pl?field=symbol&anchor=equals&symbol_search=Search&number=100&format=html&sortby=symbol&match=NDUFA3) |  | [603832](http://www.ncbi.nlm.nih.gov/entrez/dispomim.cgi?id=603832) | **1.34** |
| 1452791_AT | RIKEN cDNA 2310002F18 gene | [2310002F18RIK](http://www.gene.ucl.ac.uk/cgi-bin/nomenclature/searchgenes.pl?field=symbol&anchor=equals&symbol_search=Search&number=100&format=html&sortby=symbol&match=2310002F18RIK) |  |  | **1.34** |
| 1452798_S_AT | RIKEN cDNA 2310009E07 gene | [MAPK1IP1](http://www.gene.ucl.ac.uk/cgi-bin/nomenclature/searchgenes.pl?field=symbol&anchor=equals&symbol_search=Search&number=100&format=html&sortby=symbol&match=MAPK1IP1) |  |  | **1.34** |
| 1452800_A_AT | RIKEN cDNA 0610008C08 gene | [0610008C08RIK](http://www.gene.ucl.ac.uk/cgi-bin/nomenclature/searchgenes.pl?field=symbol&anchor=equals&symbol_search=Search&number=100&format=html&sortby=symbol&match=0610008C08RIK) |  |  | **1.39** |
| 1452806_AT | RIKEN cDNA 1500016O10 gene | [1500016O10RIK](http://www.gene.ucl.ac.uk/cgi-bin/nomenclature/searchgenes.pl?field=symbol&anchor=equals&symbol_search=Search&number=100&format=html&sortby=symbol&match=1500016O10RIK) |  |  | **1.73** |
| 1452823_AT | glutathione S-transferase kappa 1 | [GSTK1](http://www.gene.ucl.ac.uk/cgi-bin/nomenclature/searchgenes.pl?field=symbol&anchor=equals&symbol_search=Search&number=100&format=html&sortby=symbol&match=GSTK1) |  |  | **1.73** |
| 1452833_AT | Rap guanine nucleotide exchange factor (GEF) 2 | [RAPGEF2](http://www.gene.ucl.ac.uk/cgi-bin/nomenclature/searchgenes.pl?field=symbol&anchor=equals&symbol_search=Search&number=100&format=html&sortby=symbol&match=RAPGEF2) |  |  | **1.46** |
| 1452865_AT | Leucine rich repeat containing 27 | [LRRC27](http://www.gene.ucl.ac.uk/cgi-bin/nomenclature/searchgenes.pl?field=symbol&anchor=equals&symbol_search=Search&number=100&format=html&sortby=symbol&match=LRRC27) |  |  | **2.35** |
| 1452868_AT | ubiquitin specific protease 24 | [USP24](http://www.gene.ucl.ac.uk/cgi-bin/nomenclature/searchgenes.pl?field=symbol&anchor=equals&symbol_search=Search&number=100&format=html&sortby=symbol&match=USP24) |  |  | **1.35** |
| 1452869_AT | RIKEN cDNA 1110021E09 gene | [1110021E09RIK](http://www.gene.ucl.ac.uk/cgi-bin/nomenclature/searchgenes.pl?field=symbol&anchor=equals&symbol_search=Search&number=100&format=html&sortby=symbol&match=1110021E09RIK) |  |  | **-1.38** |
| 1452870_AT | apoptotic protease activating factor 1 | [APAF1](http://www.gene.ucl.ac.uk/cgi-bin/nomenclature/searchgenes.pl?field=symbol&anchor=equals&symbol_search=Search&number=100&format=html&sortby=symbol&match=APAF1) |  | [602233](http://www.ncbi.nlm.nih.gov/entrez/dispomim.cgi?id=602233) | **-1.36** |
| 1452872_AT | RIKEN cDNA 2900054D09 gene | [2900054D09RIK](http://www.gene.ucl.ac.uk/cgi-bin/nomenclature/searchgenes.pl?field=symbol&anchor=equals&symbol_search=Search&number=100&format=html&sortby=symbol&match=2900054D09RIK) |  |  | **-1.31** |
| 1452876_X_AT | RIKEN cDNA 2610044O15 gene | [2610044O15RIK](http://www.gene.ucl.ac.uk/cgi-bin/nomenclature/searchgenes.pl?field=symbol&anchor=equals&symbol_search=Search&number=100&format=html&sortby=symbol&match=2610044O15RIK) |  |  | **1.56** |
| 1452895_AT | RIKEN cDNA 2610017J04 gene | [2610017J04RIK](http://www.gene.ucl.ac.uk/cgi-bin/nomenclature/searchgenes.pl?field=symbol&anchor=equals&symbol_search=Search&number=100&format=html&sortby=symbol&match=2610017J04RIK) |  |  | **1.36** |
| 1452905_AT | GTL2, imprinted maternally expressed untranslated mRNA | [GTL2](http://www.gene.ucl.ac.uk/cgi-bin/nomenclature/searchgenes.pl?field=symbol&anchor=equals&symbol_search=Search&number=100&format=html&sortby=symbol&match=GTL2) |  |  | **-1.58** |
| 1452913_AT | Purkinje cell protein 4-like 1 | [PCP4L1](http://www.gene.ucl.ac.uk/cgi-bin/nomenclature/searchgenes.pl?field=symbol&anchor=equals&symbol_search=Search&number=100&format=html&sortby=symbol&match=PCP4L1) |  |  | **2.3** |
| 1452914_AT | RIKEN cDNA 2410024N18 gene | [2410024N18RIK](http://www.gene.ucl.ac.uk/cgi-bin/nomenclature/searchgenes.pl?field=symbol&anchor=equals&symbol_search=Search&number=100&format=html&sortby=symbol&match=2410024N18RIK) |  |  | **2.6** |
| 1452915_AT | protein kinase, cAMP dependent regulatory, type II alpha | [PRKAR2A](http://www.gene.ucl.ac.uk/cgi-bin/nomenclature/searchgenes.pl?field=symbol&anchor=equals&symbol_search=Search&number=100&format=html&sortby=symbol&match=PRKAR2A) |  | [176910](http://www.ncbi.nlm.nih.gov/entrez/dispomim.cgi?id=176910) | **1.44** |
| 1452929_AT | restin (Reed-Steinberg cell-expressed intermediate filament-associated protein) | [RSN](http://www.gene.ucl.ac.uk/cgi-bin/nomenclature/searchgenes.pl?field=symbol&anchor=equals&symbol_search=Search&number=100&format=html&sortby=symbol&match=RSN) |  | [179838](http://www.ncbi.nlm.nih.gov/entrez/dispomim.cgi?id=179838) | **1.31** |
| 1452942_AT | RIKEN cDNA 4930438D12 gene | [4930438D12RIK](http://www.gene.ucl.ac.uk/cgi-bin/nomenclature/searchgenes.pl?field=symbol&anchor=equals&symbol_search=Search&number=100&format=html&sortby=symbol&match=4930438D12RIK) |  |  | **1.87** |
| 1452947_AT | G protein-coupled receptor, family C, group 5, member C | [GPRC5C](http://www.gene.ucl.ac.uk/cgi-bin/nomenclature/searchgenes.pl?field=symbol&anchor=equals&symbol_search=Search&number=100&format=html&sortby=symbol&match=GPRC5C) |  | [605949](http://www.ncbi.nlm.nih.gov/entrez/dispomim.cgi?id=605949) | **-1.65** |
| 1452968_AT | collagen triple helix repeat containing 1 | [CTHRC1](http://www.gene.ucl.ac.uk/cgi-bin/nomenclature/searchgenes.pl?field=symbol&anchor=equals&symbol_search=Search&number=100&format=html&sortby=symbol&match=CTHRC1) |  |  | **-3.6** |
| 1452972_AT | RIKEN cDNA 1700013G20 gene | [1700013G20RIK](http://www.gene.ucl.ac.uk/cgi-bin/nomenclature/searchgenes.pl?field=symbol&anchor=equals&symbol_search=Search&number=100&format=html&sortby=symbol&match=1700013G20RIK) |  |  | **1.39** |
| 1452973_AT | RIKEN cDNA A930026L03 gene | [A930026L03RIK](http://www.gene.ucl.ac.uk/cgi-bin/nomenclature/searchgenes.pl?field=symbol&anchor=equals&symbol_search=Search&number=100&format=html&sortby=symbol&match=A930026L03RIK) |  |  | **1.64** |
| 1452976_A_AT | solute carrier family 9 (sodium/hydrogen exchanger), isoform 3 regulator 2 | [SLC9A3R2](http://www.gene.ucl.ac.uk/cgi-bin/nomenclature/searchgenes.pl?field=symbol&anchor=equals&symbol_search=Search&number=100&format=html&sortby=symbol&match=SLC9A3R2) |  | [606553](http://www.ncbi.nlm.nih.gov/entrez/dispomim.cgi?id=606553) | **2.05** |
| 1452984_AT | RIKEN cDNA 5730405I09 gene | [5730405I09RIK](http://www.gene.ucl.ac.uk/cgi-bin/nomenclature/searchgenes.pl?field=symbol&anchor=equals&symbol_search=Search&number=100&format=html&sortby=symbol&match=5730405I09RIK) |  |  | **1.53** |
| 1452985_AT | uveal autoantigen with coiled-coil domains and ankyrin repeats | [UACA](http://www.gene.ucl.ac.uk/cgi-bin/nomenclature/searchgenes.pl?field=symbol&anchor=equals&symbol_search=Search&number=100&format=html&sortby=symbol&match=UACA) |  |  | **1.63** |
| 1452996_A_AT | apoptosis, caspase activation inhibitor | [AVEN](http://www.gene.ucl.ac.uk/cgi-bin/nomenclature/searchgenes.pl?field=symbol&anchor=equals&symbol_search=Search&number=100&format=html&sortby=symbol&match=AVEN) |  | [605265](http://www.ncbi.nlm.nih.gov/entrez/dispomim.cgi?id=605265) | **-1.34** |
| 1453003_AT | sortilin-related receptor, LDLR class A repeats-containing | [SORL1](http://www.gene.ucl.ac.uk/cgi-bin/nomenclature/searchgenes.pl?field=symbol&anchor=equals&symbol_search=Search&number=100&format=html&sortby=symbol&match=SORL1) |  | [602005](http://www.ncbi.nlm.nih.gov/entrez/dispomim.cgi?id=602005) | **1.89** |
| 1453022_AT | GPI-anchored HDL-binding protein 1 | [GPIHBP1](http://www.gene.ucl.ac.uk/cgi-bin/nomenclature/searchgenes.pl?field=symbol&anchor=equals&symbol_search=Search&number=100&format=html&sortby=symbol&match=GPIHBP1) |  |  | **1.86** |
| 1453055_AT | sema domain, transmembrane domain (TM), and cytoplasmic domain, (semaphorin) 6D | [SEMA6D](http://www.gene.ucl.ac.uk/cgi-bin/nomenclature/searchgenes.pl?field=symbol&anchor=equals&symbol_search=Search&number=100&format=html&sortby=symbol&match=SEMA6D) |  |  | **1.28** |
| 1453061_AT | RIKEN cDNA 2610018O07 gene | [2610018O07RIK](http://www.gene.ucl.ac.uk/cgi-bin/nomenclature/searchgenes.pl?field=symbol&anchor=equals&symbol_search=Search&number=100&format=html&sortby=symbol&match=2610018O07RIK) |  |  | **1.24** |
| 1453074_AT | Dual specificity phosphatase 23 | [DUSP23](http://www.gene.ucl.ac.uk/cgi-bin/nomenclature/searchgenes.pl?field=symbol&anchor=equals&symbol_search=Search&number=100&format=html&sortby=symbol&match=DUSP23) |  |  | **1.34** |
| 1453099_AT | casein kinase II, alpha 2, polypeptide | [CSNK2A2](http://www.gene.ucl.ac.uk/cgi-bin/nomenclature/searchgenes.pl?field=symbol&anchor=equals&symbol_search=Search&number=100&format=html&sortby=symbol&match=CSNK2A2) |  | [115442](http://www.ncbi.nlm.nih.gov/entrez/dispomim.cgi?id=115442) | **-1.67** |
| 1453106_A_AT | RNA (guanine-7-) methyltransferase | [RNMT](http://www.gene.ucl.ac.uk/cgi-bin/nomenclature/searchgenes.pl?field=symbol&anchor=equals&symbol_search=Search&number=100&format=html&sortby=symbol&match=RNMT) |  | [603514](http://www.ncbi.nlm.nih.gov/entrez/dispomim.cgi?id=603514) | **-1.59** |
| 1453111_A_AT | DNA segment, Chr 11, ERATO Doi 333, expressed | [D11ERTD333E](http://www.gene.ucl.ac.uk/cgi-bin/nomenclature/searchgenes.pl?field=symbol&anchor=equals&symbol_search=Search&number=100&format=html&sortby=symbol&match=D11ERTD333E) |  |  | **1.81** |
| 1453118_S_AT | ribosomal protein L22 | [RPL22](http://www.gene.ucl.ac.uk/cgi-bin/nomenclature/searchgenes.pl?field=symbol&anchor=equals&symbol_search=Search&number=100&format=html&sortby=symbol&match=RPL22) |  | [180474](http://www.ncbi.nlm.nih.gov/entrez/dispomim.cgi?id=180474) | **-1.23** |
| 1453125_AT | RIKEN cDNA 6230403H02 gene | [6230403H02RIK](http://www.gene.ucl.ac.uk/cgi-bin/nomenclature/searchgenes.pl?field=symbol&anchor=equals&symbol_search=Search&number=100&format=html&sortby=symbol&match=6230403H02RIK) |  |  | **-10.18** |
| 1453131_AT | DNA segment, Chr 11, ERATO Doi 736, expressed | [D11ERTD736E](http://www.gene.ucl.ac.uk/cgi-bin/nomenclature/searchgenes.pl?field=symbol&anchor=equals&symbol_search=Search&number=100&format=html&sortby=symbol&match=D11ERTD736E) |  |  | **2.17** |
| 1453135_AT | fibronectin type III domain containing 5 | [FNDC5](http://www.gene.ucl.ac.uk/cgi-bin/nomenclature/searchgenes.pl?field=symbol&anchor=equals&symbol_search=Search&number=100&format=html&sortby=symbol&match=FNDC5) |  |  | **1.4** |
| 1453141_AT | RIKEN cDNA 0610009L18 gene | [0610009L18RIK](http://www.gene.ucl.ac.uk/cgi-bin/nomenclature/searchgenes.pl?field=symbol&anchor=equals&symbol_search=Search&number=100&format=html&sortby=symbol&match=0610009L18RIK) |  |  | **1.33** |
| 1453145_AT | RIKEN cDNA 4933439C20 gene | [4933439C20RIK](http://www.gene.ucl.ac.uk/cgi-bin/nomenclature/searchgenes.pl?field=symbol&anchor=equals&symbol_search=Search&number=100&format=html&sortby=symbol&match=4933439C20RIK) |  |  | **-5.27** |
| 1453152_AT | MAM domain containing 2 | [MAMDC2](http://www.gene.ucl.ac.uk/cgi-bin/nomenclature/searchgenes.pl?field=symbol&anchor=equals&symbol_search=Search&number=100&format=html&sortby=symbol&match=MAMDC2) |  |  | **-1.91** |
| 1453156_S_AT | RIKEN cDNA 9130222H03 gene | [9130222H03RIK](http://www.gene.ucl.ac.uk/cgi-bin/nomenclature/searchgenes.pl?field=symbol&anchor=equals&symbol_search=Search&number=100&format=html&sortby=symbol&match=9130222H03RIK) |  |  | **1.5** |
| 1453166_AT | RIKEN cDNA D130073L02 gene | [D130073L02RIK](http://www.gene.ucl.ac.uk/cgi-bin/nomenclature/searchgenes.pl?field=symbol&anchor=equals&symbol_search=Search&number=100&format=html&sortby=symbol&match=D130073L02RIK) |  |  | **-1.31** |
| 1453169_A_AT | general transcription factor II H, polypeptide 1 | [GTF2H1](http://www.gene.ucl.ac.uk/cgi-bin/nomenclature/searchgenes.pl?field=symbol&anchor=equals&symbol_search=Search&number=100&format=html&sortby=symbol&match=GTF2H1) |  | [189972](http://www.ncbi.nlm.nih.gov/entrez/dispomim.cgi?id=189972) | **-1.22** |
| 1453187_AT | RIKEN cDNA 1810027I20 gene | [OCIAD2](http://www.gene.ucl.ac.uk/cgi-bin/nomenclature/searchgenes.pl?field=symbol&anchor=equals&symbol_search=Search&number=100&format=html&sortby=symbol&match=OCIAD2) |  |  | **-1.72** |
| 1453188_AT | RIKEN cDNA 6230424C14 gene | [6230424C14RIK](http://www.gene.ucl.ac.uk/cgi-bin/nomenclature/searchgenes.pl?field=symbol&anchor=equals&symbol_search=Search&number=100&format=html&sortby=symbol&match=6230424C14RIK) |  |  | **1.68** |
| 1453208_AT | RIKEN cDNA 2700089E24 gene | [2700089E24RIK](http://www.gene.ucl.ac.uk/cgi-bin/nomenclature/searchgenes.pl?field=symbol&anchor=equals&symbol_search=Search&number=100&format=html&sortby=symbol&match=2700089E24RIK) |  |  | **1.4** |
| 1453228_AT | Syntaxin 11 | [STX11](http://www.gene.ucl.ac.uk/cgi-bin/nomenclature/searchgenes.pl?field=symbol&anchor=equals&symbol_search=Search&number=100&format=html&sortby=symbol&match=STX11) |  | [605014](http://www.ncbi.nlm.nih.gov/entrez/dispomim.cgi?id=605014) | **-1.87** |
| 1453257_AT | DNA segment, Chr 8, ERATO Doi 319, expressed | [AGPAT5](http://www.gene.ucl.ac.uk/cgi-bin/nomenclature/searchgenes.pl?field=symbol&anchor=equals&symbol_search=Search&number=100&format=html&sortby=symbol&match=AGPAT5) |  |  | **-1.35** |
| 1453278_A_AT | RIKEN cDNA 1700024K14 gene | [RSNL2](http://www.gene.ucl.ac.uk/cgi-bin/nomenclature/searchgenes.pl?field=symbol&anchor=equals&symbol_search=Search&number=100&format=html&sortby=symbol&match=RSNL2) |  |  | **1.5** |
| 1453285_AT | RIKEN cDNA 2600017H02 gene | [2600017H02RIK](http://www.gene.ucl.ac.uk/cgi-bin/nomenclature/searchgenes.pl?field=symbol&anchor=equals&symbol_search=Search&number=100&format=html&sortby=symbol&match=2600017H02RIK) |  |  | **1.82** |
| 1453286_AT | plexin A2 | [PLXNA2](http://www.gene.ucl.ac.uk/cgi-bin/nomenclature/searchgenes.pl?field=symbol&anchor=equals&symbol_search=Search&number=100&format=html&sortby=symbol&match=PLXNA2) |  | [601054](http://www.ncbi.nlm.nih.gov/entrez/dispomim.cgi?id=601054) | **1.31** |
| 1453313_AT | sestrin 3 | [SESN3](http://www.gene.ucl.ac.uk/cgi-bin/nomenclature/searchgenes.pl?field=symbol&anchor=equals&symbol_search=Search&number=100&format=html&sortby=symbol&match=SESN3) |  | [607768](http://www.ncbi.nlm.nih.gov/entrez/dispomim.cgi?id=607768) | **-1.78** |
| 1453317_A_AT | KH domain containing, RNA binding, signal transduction associated 3 | [KHDRBS3](http://www.gene.ucl.ac.uk/cgi-bin/nomenclature/searchgenes.pl?field=symbol&anchor=equals&symbol_search=Search&number=100&format=html&sortby=symbol&match=KHDRBS3) |  |  | **1.55** |
| 1453323_AT | RIKEN cDNA 2900079G21 gene | [2900079G21RIK](http://www.gene.ucl.ac.uk/cgi-bin/nomenclature/searchgenes.pl?field=symbol&anchor=equals&symbol_search=Search&number=100&format=html&sortby=symbol&match=2900079G21RIK) |  |  | **-2.11** |
| 1453355_AT | RIKEN cDNA 1810073P09 gene | [1810073P09RIK](http://www.gene.ucl.ac.uk/cgi-bin/nomenclature/searchgenes.pl?field=symbol&anchor=equals&symbol_search=Search&number=100&format=html&sortby=symbol&match=1810073P09RIK) |  |  | **2.06** |
| 1453369_A_AT | FUN14 domain containing 1 | [FUNDC1](http://www.gene.ucl.ac.uk/cgi-bin/nomenclature/searchgenes.pl?field=symbol&anchor=equals&symbol_search=Search&number=100&format=html&sortby=symbol&match=FUNDC1) |  |  | **1.31** |
| 1453393_A_AT | carbohydrate (chondroitin 6/keratan) sulfotransferase 4 | [CHST4](http://www.gene.ucl.ac.uk/cgi-bin/nomenclature/searchgenes.pl?field=symbol&anchor=equals&symbol_search=Search&number=100&format=html&sortby=symbol&match=CHST4) |  |  | **2.22** |
| 1453406_A_AT | RAB28, member RAS oncogene family | [RAB28](http://www.gene.ucl.ac.uk/cgi-bin/nomenclature/searchgenes.pl?field=symbol&anchor=equals&symbol_search=Search&number=100&format=html&sortby=symbol&match=RAB28) |  |  | **1.44** |
| 1453416_AT | growth arrest-specific 2 like 3 | [GAS2L3](http://www.gene.ucl.ac.uk/cgi-bin/nomenclature/searchgenes.pl?field=symbol&anchor=equals&symbol_search=Search&number=100&format=html&sortby=symbol&match=GAS2L3) |  |  | **-1.65** |
| 1453422_A_AT | RIKEN cDNA 1110020G09 gene | [1110020G09RIK](http://www.gene.ucl.ac.uk/cgi-bin/nomenclature/searchgenes.pl?field=symbol&anchor=equals&symbol_search=Search&number=100&format=html&sortby=symbol&match=1110020G09RIK) |  |  | **1.88** |
| 1453435_A_AT | flavin containing monooxygenase 2 | [FMO2](http://www.gene.ucl.ac.uk/cgi-bin/nomenclature/searchgenes.pl?field=symbol&anchor=equals&symbol_search=Search&number=100&format=html&sortby=symbol&match=FMO2) |  | [603955](http://www.ncbi.nlm.nih.gov/entrez/dispomim.cgi?id=603955) | **2.25** |
| 1453467_S_AT | ribosomal protein S15a | [SYN1](http://www.gene.ucl.ac.uk/cgi-bin/nomenclature/searchgenes.pl?field=symbol&anchor=equals&symbol_search=Search&number=100&format=html&sortby=symbol&match=SYN1) |  | [313440](http://www.ncbi.nlm.nih.gov/entrez/dispomim.cgi?id=313440) | **-1.35** |
| 1453472_A_AT | SLAM family member 7 | [SLAMF7](http://www.gene.ucl.ac.uk/cgi-bin/nomenclature/searchgenes.pl?field=symbol&anchor=equals&symbol_search=Search&number=100&format=html&sortby=symbol&match=SLAMF7) |  | [606625](http://www.ncbi.nlm.nih.gov/entrez/dispomim.cgi?id=606625) | **-2.39** |
| 1453473_A_AT | t-complex testis expressed 1 | [TCTEX1](http://www.gene.ucl.ac.uk/cgi-bin/nomenclature/searchgenes.pl?field=symbol&anchor=equals&symbol_search=Search&number=100&format=html&sortby=symbol&match=TCTEX1) |  |  | **1.78** |
| 1453485_S_AT | RIKEN cDNA 1110005A03 gene | [1110005A03RIK](http://www.gene.ucl.ac.uk/cgi-bin/nomenclature/searchgenes.pl?field=symbol&anchor=equals&symbol_search=Search&number=100&format=html&sortby=symbol&match=1110005A03RIK) |  |  | **1.35** |
| 1453552_AT | RIKEN cDNA 2310014F07 gene | [2310014F07RIK](http://www.gene.ucl.ac.uk/cgi-bin/nomenclature/searchgenes.pl?field=symbol&anchor=equals&symbol_search=Search&number=100&format=html&sortby=symbol&match=2310014F07RIK) |  |  | **3.36** |
| 1453572_A_AT | proteolipid protein 2 | [PLP2](http://www.gene.ucl.ac.uk/cgi-bin/nomenclature/searchgenes.pl?field=symbol&anchor=equals&symbol_search=Search&number=100&format=html&sortby=symbol&match=PLP2) |  | [300112](http://www.ncbi.nlm.nih.gov/entrez/dispomim.cgi?id=300112) | **-1.86** |
| 1453592_AT | RIKEN cDNA 9430028I06 gene | [9430028I06RIK](http://www.gene.ucl.ac.uk/cgi-bin/nomenclature/searchgenes.pl?field=symbol&anchor=equals&symbol_search=Search&number=100&format=html&sortby=symbol&match=9430028I06RIK) |  |  | **1.73** |
| 1453623_A_AT | RAD23a homolog (S. cerevisiae) | [RAD23A](http://www.gene.ucl.ac.uk/cgi-bin/nomenclature/searchgenes.pl?field=symbol&anchor=equals&symbol_search=Search&number=100&format=html&sortby=symbol&match=RAD23A) |  | [600061](http://www.ncbi.nlm.nih.gov/entrez/dispomim.cgi?id=600061) | **1.45** |
| 1453628_S_AT | Leucine-rich repeat-containing 2 | [LRRC2](http://www.gene.ucl.ac.uk/cgi-bin/nomenclature/searchgenes.pl?field=symbol&anchor=equals&symbol_search=Search&number=100&format=html&sortby=symbol&match=LRRC2) |  | [607180](http://www.ncbi.nlm.nih.gov/entrez/dispomim.cgi?id=607180) | **1.82** |
| 1453634_A_AT | endoplasmic reticulum protein 29 | [ERP29](http://www.gene.ucl.ac.uk/cgi-bin/nomenclature/searchgenes.pl?field=symbol&anchor=equals&symbol_search=Search&number=100&format=html&sortby=symbol&match=ERP29) |  |  | **-1.61** |
| 1453651_A_AT | brain protein 44-like | [BRP44L](http://www.gene.ucl.ac.uk/cgi-bin/nomenclature/searchgenes.pl?field=symbol&anchor=equals&symbol_search=Search&number=100&format=html&sortby=symbol&match=BRP44L) |  |  | **1.43** |
| 1453657_AT | RIKEN cDNA 2310065F04 gene | [2310065F04RIK](http://www.gene.ucl.ac.uk/cgi-bin/nomenclature/searchgenes.pl?field=symbol&anchor=equals&symbol_search=Search&number=100&format=html&sortby=symbol&match=2310065F04RIK) |  |  | **2.85** |
| 1453725_A_AT | mitchondrial ribosomal protein S7 | [MRPS7](http://www.gene.ucl.ac.uk/cgi-bin/nomenclature/searchgenes.pl?field=symbol&anchor=equals&symbol_search=Search&number=100&format=html&sortby=symbol&match=MRPS7) |  |  | **1.25** |
| 1453732_AT | RIKEN cDNA 6230416A05 gene | [6230416A05RIK](http://www.gene.ucl.ac.uk/cgi-bin/nomenclature/searchgenes.pl?field=symbol&anchor=equals&symbol_search=Search&number=100&format=html&sortby=symbol&match=6230416A05RIK) |  |  | **1.54** |
| 1453738_AT | RIKEN cDNA C330018D20 gene | [C330018D20RIK](http://www.gene.ucl.ac.uk/cgi-bin/nomenclature/searchgenes.pl?field=symbol&anchor=equals&symbol_search=Search&number=100&format=html&sortby=symbol&match=C330018D20RIK) |  |  | **1.38** |
| 1453744_A_AT | RIKEN cDNA 5530600A18 gene | [5530600A18RIK](http://www.gene.ucl.ac.uk/cgi-bin/nomenclature/searchgenes.pl?field=symbol&anchor=equals&symbol_search=Search&number=100&format=html&sortby=symbol&match=5530600A18RIK) |  |  | **1.57** |
| 1453746_AT | formin binding protein 1 | [FNBP1](http://www.gene.ucl.ac.uk/cgi-bin/nomenclature/searchgenes.pl?field=symbol&anchor=equals&symbol_search=Search&number=100&format=html&sortby=symbol&match=FNBP1) |  | [606191](http://www.ncbi.nlm.nih.gov/entrez/dispomim.cgi?id=606191) | **-1.39** |
| 1453752_AT | ribosomal protein L17 | [RPL17](http://www.gene.ucl.ac.uk/cgi-bin/nomenclature/searchgenes.pl?field=symbol&anchor=equals&symbol_search=Search&number=100&format=html&sortby=symbol&match=RPL17) |  | [603661](http://www.ncbi.nlm.nih.gov/entrez/dispomim.cgi?id=603661) | **-2.4** |
| 1453782_AT | RIKEN cDNA 3021401C12 gene | [3021401C12RIK](http://www.gene.ucl.ac.uk/cgi-bin/nomenclature/searchgenes.pl?field=symbol&anchor=equals&symbol_search=Search&number=100&format=html&sortby=symbol&match=3021401C12RIK) |  |  | **1.57** |
| 1453795_AT | RIKEN cDNA B430104H02 gene | [B430104H02RIK](http://www.gene.ucl.ac.uk/cgi-bin/nomenclature/searchgenes.pl?field=symbol&anchor=equals&symbol_search=Search&number=100&format=html&sortby=symbol&match=B430104H02RIK) |  |  | **1.45** |
| 1453836_A_AT | monoglyceride lipase | [MGLL](http://www.gene.ucl.ac.uk/cgi-bin/nomenclature/searchgenes.pl?field=symbol&anchor=equals&symbol_search=Search&number=100&format=html&sortby=symbol&match=MGLL) |  |  | **2.15** |
| 1453855_AT | RIKEN cDNA 1810057P16 gene | [1810057P16RIK](http://www.gene.ucl.ac.uk/cgi-bin/nomenclature/searchgenes.pl?field=symbol&anchor=equals&symbol_search=Search&number=100&format=html&sortby=symbol&match=1810057P16RIK) |  |  | **-1.67** |
| 1453886_A_AT | solute carrier family 25 (mitochondrial carrier, phosphate carrier), member 26 | [SLC25A26](http://www.gene.ucl.ac.uk/cgi-bin/nomenclature/searchgenes.pl?field=symbol&anchor=equals&symbol_search=Search&number=100&format=html&sortby=symbol&match=SLC25A26) |  |  | **1.51** |
| 1453908_AT | protein tyrosine phosphatase, receptor type, B | [PTPRB](http://www.gene.ucl.ac.uk/cgi-bin/nomenclature/searchgenes.pl?field=symbol&anchor=equals&symbol_search=Search&number=100&format=html&sortby=symbol&match=PTPRB) |  | [176882](http://www.ncbi.nlm.nih.gov/entrez/dispomim.cgi?id=176882) | **2.68** |
| 1453973_S_AT | RIKEN cDNA 2900057H02 gene | [2900057H02RIK](http://www.gene.ucl.ac.uk/cgi-bin/nomenclature/searchgenes.pl?field=symbol&anchor=equals&symbol_search=Search&number=100&format=html&sortby=symbol&match=2900057H02RIK) |  |  | **2.13** |
| 1453987_AT | BTB (POZ) domain containing 5 | [BTBD5](http://www.gene.ucl.ac.uk/cgi-bin/nomenclature/searchgenes.pl?field=symbol&anchor=equals&symbol_search=Search&number=100&format=html&sortby=symbol&match=BTBD5) |  |  | **-1.88** |
| 1453988_A_AT | insulin degrading enzyme | [IDE](http://www.gene.ucl.ac.uk/cgi-bin/nomenclature/searchgenes.pl?field=symbol&anchor=equals&symbol_search=Search&number=100&format=html&sortby=symbol&match=IDE) |  | [146680](http://www.ncbi.nlm.nih.gov/entrez/dispomim.cgi?id=146680) | **1.37** |
| 1454015_A_AT | cadherin 13 | [CDH13](http://www.gene.ucl.ac.uk/cgi-bin/nomenclature/searchgenes.pl?field=symbol&anchor=equals&symbol_search=Search&number=100&format=html&sortby=symbol&match=CDH13) |  | [601364](http://www.ncbi.nlm.nih.gov/entrez/dispomim.cgi?id=601364) | **-1.47** |
| 1454036_A_AT | ubiquitin specific protease 15 | [USP15](http://www.gene.ucl.ac.uk/cgi-bin/nomenclature/searchgenes.pl?field=symbol&anchor=equals&symbol_search=Search&number=100&format=html&sortby=symbol&match=USP15) |  | [604731](http://www.ncbi.nlm.nih.gov/entrez/dispomim.cgi?id=604731) | **1.45** |
| 1454037_A_AT | FMS-like tyrosine kinase 1 | [FLT1](http://www.gene.ucl.ac.uk/cgi-bin/nomenclature/searchgenes.pl?field=symbol&anchor=equals&symbol_search=Search&number=100&format=html&sortby=symbol&match=FLT1) |  | [165070](http://www.ncbi.nlm.nih.gov/entrez/dispomim.cgi?id=165070) | **2.57** |
| 1454060_A_AT | neuroblastoma ras oncogene | [NRAS](http://www.gene.ucl.ac.uk/cgi-bin/nomenclature/searchgenes.pl?field=symbol&anchor=equals&symbol_search=Search&number=100&format=html&sortby=symbol&match=NRAS) |  | [164790](http://www.ncbi.nlm.nih.gov/entrez/dispomim.cgi?id=164790) | **-1.59** |
| 1454075_S_AT | nudix (nucleoside diphosphate linked moiety X)-type motif 13 | [NUDT13](http://www.gene.ucl.ac.uk/cgi-bin/nomenclature/searchgenes.pl?field=symbol&anchor=equals&symbol_search=Search&number=100&format=html&sortby=symbol&match=NUDT13) |  |  | **1.35** |
| 1454137_S_AT | RGM domain family, member C | [HFE2](http://www.gene.ucl.ac.uk/cgi-bin/nomenclature/searchgenes.pl?field=symbol&anchor=equals&symbol_search=Search&number=100&format=html&sortby=symbol&match=HFE2) |  | [608374](http://www.ncbi.nlm.nih.gov/entrez/dispomim.cgi?id=608374) | **1.3** |
| 1454149_A_AT | cyclin L2 | [CCNL2](http://www.gene.ucl.ac.uk/cgi-bin/nomenclature/searchgenes.pl?field=symbol&anchor=equals&symbol_search=Search&number=100&format=html&sortby=symbol&match=CCNL2) |  |  | **-1.45** |
| 1454168_A_AT | suppressor of K+ transport defect 3 | [SKD3](http://www.gene.ucl.ac.uk/cgi-bin/nomenclature/searchgenes.pl?field=symbol&anchor=equals&symbol_search=Search&number=100&format=html&sortby=symbol&match=SKD3) |  |  | **1.83** |
| 1454197_A_AT | DNA segment, Chr 19, ERATO Doi 678, expressed | [D19ERTD678E](http://www.gene.ucl.ac.uk/cgi-bin/nomenclature/searchgenes.pl?field=symbol&anchor=equals&symbol_search=Search&number=100&format=html&sortby=symbol&match=D19ERTD678E) |  |  | **-1.55** |
| 1454268_A_AT | cytochrome b-245, alpha polypeptide | [CYBA](http://www.gene.ucl.ac.uk/cgi-bin/nomenclature/searchgenes.pl?field=symbol&anchor=equals&symbol_search=Search&number=100&format=html&sortby=symbol&match=CYBA) |  | [608508](http://www.ncbi.nlm.nih.gov/entrez/dispomim.cgi?id=608508) | **-2.54** |
| 1454397_AT | RIKEN cDNA 4632418H02 gene | [4632418H02RIK](http://www.gene.ucl.ac.uk/cgi-bin/nomenclature/searchgenes.pl?field=symbol&anchor=equals&symbol_search=Search&number=100&format=html&sortby=symbol&match=4632418H02RIK) |  |  | **2.06** |
| 1454607_S_AT | phosphoserine aminotransferase 1 | [PSAT1](http://www.gene.ucl.ac.uk/cgi-bin/nomenclature/searchgenes.pl?field=symbol&anchor=equals&symbol_search=Search&number=100&format=html&sortby=symbol&match=PSAT1) |  |  | **-3.38** |
| 1454613_AT | gb:AV162270 /DB_XREF=gi:16383390 /DB_XREF=AV162270 |  |  |  | **-2.07** |
| 1454614_AT | RIKEN cDNA 1810013D10 gene | [1810013D10RIK](http://www.gene.ucl.ac.uk/cgi-bin/nomenclature/searchgenes.pl?field=symbol&anchor=equals&symbol_search=Search&number=100&format=html&sortby=symbol&match=1810013D10RIK) |  |  | **1.35** |
| 1454620_X_AT | ribosomal protein S6 | [RPS6](http://www.gene.ucl.ac.uk/cgi-bin/nomenclature/searchgenes.pl?field=symbol&anchor=equals&symbol_search=Search&number=100&format=html&sortby=symbol&match=RPS6) |  | [180460](http://www.ncbi.nlm.nih.gov/entrez/dispomim.cgi?id=180460) | **-1.27** |
| 1454626_AT | clathrin, heavy polypeptide (Hc) | [CLTC](http://www.gene.ucl.ac.uk/cgi-bin/nomenclature/searchgenes.pl?field=symbol&anchor=equals&symbol_search=Search&number=100&format=html&sortby=symbol&match=CLTC) |  | [118955](http://www.ncbi.nlm.nih.gov/entrez/dispomim.cgi?id=118955) | **-1.26** |
| 1454628_AT | RIKEN cDNA A930037G23 gene | [A930037G23RIK](http://www.gene.ucl.ac.uk/cgi-bin/nomenclature/searchgenes.pl?field=symbol&anchor=equals&symbol_search=Search&number=100&format=html&sortby=symbol&match=A930037G23RIK) |  |  | **-2.04** |
| 1454629_AT | RIKEN cDNA A930037G23 gene | [A930037G23RIK](http://www.gene.ucl.ac.uk/cgi-bin/nomenclature/searchgenes.pl?field=symbol&anchor=equals&symbol_search=Search&number=100&format=html&sortby=symbol&match=A930037G23RIK) |  |  | **-1.78** |
| 1454636_AT | chromobox homolog 5 (Drosophila HP1a) | [CBX5](http://www.gene.ucl.ac.uk/cgi-bin/nomenclature/searchgenes.pl?field=symbol&anchor=equals&symbol_search=Search&number=100&format=html&sortby=symbol&match=CBX5) |  | [604478](http://www.ncbi.nlm.nih.gov/entrez/dispomim.cgi?id=604478) | **-1.31** |
| 1454640_AT | coiled-coil-helix-coiled-coil-helix domain containing 7 | [CHCHD7](http://www.gene.ucl.ac.uk/cgi-bin/nomenclature/searchgenes.pl?field=symbol&anchor=equals&symbol_search=Search&number=100&format=html&sortby=symbol&match=CHCHD7) |  |  | **1.47** |
| 1454641_AT | CGG triplet repeat binding protein 1 | [CGGBP1](http://www.gene.ucl.ac.uk/cgi-bin/nomenclature/searchgenes.pl?field=symbol&anchor=equals&symbol_search=Search&number=100&format=html&sortby=symbol&match=CGGBP1) |  | [603363](http://www.ncbi.nlm.nih.gov/entrez/dispomim.cgi?id=603363) | **-1.28** |
| 1454645_AT | mahogunin, ring finger 1 | [MGRN1](http://www.gene.ucl.ac.uk/cgi-bin/nomenclature/searchgenes.pl?field=symbol&anchor=equals&symbol_search=Search&number=100&format=html&sortby=symbol&match=MGRN1) |  | [607559](http://www.ncbi.nlm.nih.gov/entrez/dispomim.cgi?id=607559) | **1.39** |
| 1454647_AT | RIKEN cDNA 5730439E10 gene | [5730439E10RIK](http://www.gene.ucl.ac.uk/cgi-bin/nomenclature/searchgenes.pl?field=symbol&anchor=equals&symbol_search=Search&number=100&format=html&sortby=symbol&match=5730439E10RIK) |  |  | **2.2** |
| 1454650_AT | gb:BQ175280 /DB_XREF=gi:20350772 /DB_XREF=UI-M-DJ2 |  |  |  | **-1.44** |
| 1454677_AT | tissue inhibitor of metalloproteinase 2 | [TIMP2](http://www.gene.ucl.ac.uk/cgi-bin/nomenclature/searchgenes.pl?field=symbol&anchor=equals&symbol_search=Search&number=100&format=html&sortby=symbol&match=TIMP2) |  | [188825](http://www.ncbi.nlm.nih.gov/entrez/dispomim.cgi?id=188825) | **-1.27** |
| 1454680_AT | DNA segment, Chr 5, ERATO Doi 579, expressed | [D5ERTD579E](http://www.gene.ucl.ac.uk/cgi-bin/nomenclature/searchgenes.pl?field=symbol&anchor=equals&symbol_search=Search&number=100&format=html&sortby=symbol&match=D5ERTD579E) |  |  | **1.21** |
| 1454694_A_AT | topoisomerase (DNA) II alpha | [TOP2A](http://www.gene.ucl.ac.uk/cgi-bin/nomenclature/searchgenes.pl?field=symbol&anchor=equals&symbol_search=Search&number=100&format=html&sortby=symbol&match=TOP2A) |  | [126430](http://www.ncbi.nlm.nih.gov/entrez/dispomim.cgi?id=126430) | **-2.15** |
| 1454696_AT | LOC433812 | [LOC433812](http://www.gene.ucl.ac.uk/cgi-bin/nomenclature/searchgenes.pl?field=symbol&anchor=equals&symbol_search=Search&number=100&format=html&sortby=symbol&match=LOC433812) |  |  | **-1.23** |
| 1454704_AT | RIKEN cDNA 9330185J12 gene | [9330185J12RIK](http://www.gene.ucl.ac.uk/cgi-bin/nomenclature/searchgenes.pl?field=symbol&anchor=equals&symbol_search=Search&number=100&format=html&sortby=symbol&match=9330185J12RIK) |  |  | **-1.21** |
| 1454705_AT | DNA segment, Chr 15, ERATO Doi 621, expressed | [D15ERTD621E](http://www.gene.ucl.ac.uk/cgi-bin/nomenclature/searchgenes.pl?field=symbol&anchor=equals&symbol_search=Search&number=100&format=html&sortby=symbol&match=D15ERTD621E) |  |  | **-1.32** |
| 1454708_AT | actin-binding LIM protein 1 | [ABLIM1](http://www.gene.ucl.ac.uk/cgi-bin/nomenclature/searchgenes.pl?field=symbol&anchor=equals&symbol_search=Search&number=100&format=html&sortby=symbol&match=ABLIM1) |  | [602330](http://www.ncbi.nlm.nih.gov/entrez/dispomim.cgi?id=602330) | **-1.67** |
| 1454712_AT | mitochondrial carrier triple repeat 1 | [MCART1](http://www.gene.ucl.ac.uk/cgi-bin/nomenclature/searchgenes.pl?field=symbol&anchor=equals&symbol_search=Search&number=100&format=html&sortby=symbol&match=MCART1) |  |  | **1.26** |
| 1454714_X_AT | 3-phosphoglycerate dehydrogenase | [PHGDH](http://www.gene.ucl.ac.uk/cgi-bin/nomenclature/searchgenes.pl?field=symbol&anchor=equals&symbol_search=Search&number=100&format=html&sortby=symbol&match=PHGDH) |  | [606879](http://www.ncbi.nlm.nih.gov/entrez/dispomim.cgi?id=606879) | **-2.06** |
| 1454716_X_AT | cytochrome c oxidase, subunit Vb | [COX5B](http://www.gene.ucl.ac.uk/cgi-bin/nomenclature/searchgenes.pl?field=symbol&anchor=equals&symbol_search=Search&number=100&format=html&sortby=symbol&match=COX5B) |  | [123866](http://www.ncbi.nlm.nih.gov/entrez/dispomim.cgi?id=123866) | **1.2** |
| 1454720_AT | amyloid beta (A4) precursor protein-binding, family A, member 3 | [APBA3](http://www.gene.ucl.ac.uk/cgi-bin/nomenclature/searchgenes.pl?field=symbol&anchor=equals&symbol_search=Search&number=100&format=html&sortby=symbol&match=APBA3) |  | [604262](http://www.ncbi.nlm.nih.gov/entrez/dispomim.cgi?id=604262) | **3.52** |
| 1454725_AT | RIKEN cDNA G430041M01 gene | [G430041M01RIK](http://www.gene.ucl.ac.uk/cgi-bin/nomenclature/searchgenes.pl?field=symbol&anchor=equals&symbol_search=Search&number=100&format=html&sortby=symbol&match=G430041M01RIK) |  |  | **-1.41** |
| 1454728_S_AT | gb:AW610650 /DB_XREF=gi:7315466 /DB_XREF=up35d10.x |  |  |  | **-1.49** |
| 1454745_AT | RIKEN cDNA B130017I01 gene | [B130017I01RIK](http://www.gene.ucl.ac.uk/cgi-bin/nomenclature/searchgenes.pl?field=symbol&anchor=equals&symbol_search=Search&number=100&format=html&sortby=symbol&match=B130017I01RIK) |  |  | **1.48** |
| 1454749_AT | pericentrin 2 | [PCNT2](http://www.gene.ucl.ac.uk/cgi-bin/nomenclature/searchgenes.pl?field=symbol&anchor=equals&symbol_search=Search&number=100&format=html&sortby=symbol&match=PCNT2) |  | [605925](http://www.ncbi.nlm.nih.gov/entrez/dispomim.cgi?id=605925) | **1.53** |
| 1454755_AT | inositol 1,4,5-trisphosphate 3-kinase C | [ITPKC](http://www.gene.ucl.ac.uk/cgi-bin/nomenclature/searchgenes.pl?field=symbol&anchor=equals&symbol_search=Search&number=100&format=html&sortby=symbol&match=ITPKC) |  | [606476](http://www.ncbi.nlm.nih.gov/entrez/dispomim.cgi?id=606476) | **-1.41** |
| 1454758_A_AT | transforming growth factor beta 1 induced transcript 4 | [TGFB1I4](http://www.gene.ucl.ac.uk/cgi-bin/nomenclature/searchgenes.pl?field=symbol&anchor=equals&symbol_search=Search&number=100&format=html&sortby=symbol&match=TGFB1I4) |  | [607715](http://www.ncbi.nlm.nih.gov/entrez/dispomim.cgi?id=607715) | **-1.27** |
| 1454764_S_AT | gb:BF165681 /DB_XREF=gi:11046033 /DB_XREF=60177798 |  |  |  | **-2.56** |
| 1454778_X_AT | B6-derived CD11 +ve dendritic cells cDNA, RIKEN full-length enriched library, clone:F730014O09 product:40S RIBOSOMAL PROTEIN S28 homolog [Ictalurus punctatus], full insert sequence |  |  |  | **-1.21** |
| 1454783_AT | interleukin 13 receptor, alpha 1 | [IL13RA1](http://www.gene.ucl.ac.uk/cgi-bin/nomenclature/searchgenes.pl?field=symbol&anchor=equals&symbol_search=Search&number=100&format=html&sortby=symbol&match=IL13RA1) |  | [300119](http://www.ncbi.nlm.nih.gov/entrez/dispomim.cgi?id=300119) | **-2.34** |
| 1454788_AT | ADP-ribosylation factor-like 7 | [ARL7](http://www.gene.ucl.ac.uk/cgi-bin/nomenclature/searchgenes.pl?field=symbol&anchor=equals&symbol_search=Search&number=100&format=html&sortby=symbol&match=ARL7) |  | [604787](http://www.ncbi.nlm.nih.gov/entrez/dispomim.cgi?id=604787) | **-2.35** |
| 1454795_AT | Cobl-like 1 | [COBLL1](http://www.gene.ucl.ac.uk/cgi-bin/nomenclature/searchgenes.pl?field=symbol&anchor=equals&symbol_search=Search&number=100&format=html&sortby=symbol&match=COBLL1) |  |  | **1.72** |
| 1454806_AT | DNA segment, Chr 12, ERATO Doi 553, expressed | [D12ERTD553E](http://www.gene.ucl.ac.uk/cgi-bin/nomenclature/searchgenes.pl?field=symbol&anchor=equals&symbol_search=Search&number=100&format=html&sortby=symbol&match=D12ERTD553E) |  |  | **-1.87** |
| 1454817_AT | RIKEN cDNA 6230425C22 gene | [6230425C22RIK](http://www.gene.ucl.ac.uk/cgi-bin/nomenclature/searchgenes.pl?field=symbol&anchor=equals&symbol_search=Search&number=100&format=html&sortby=symbol&match=6230425C22RIK) |  |  | **-1.65** |
| 1454824_S_AT | mitochondrial tumor suppressor 1 | [MTUS1](http://www.gene.ucl.ac.uk/cgi-bin/nomenclature/searchgenes.pl?field=symbol&anchor=equals&symbol_search=Search&number=100&format=html&sortby=symbol&match=MTUS1) |  |  | **1.45** |
| 1454838_S_AT | expressed sequence AW548124 | [AW548124](http://www.gene.ucl.ac.uk/cgi-bin/nomenclature/searchgenes.pl?field=symbol&anchor=equals&symbol_search=Search&number=100&format=html&sortby=symbol&match=AW548124) |  |  | **1.77** |
| 1454840_AT | Methylcrotonoyl-Coenzyme A carboxylase 2 (beta) | [MCCC2](http://www.gene.ucl.ac.uk/cgi-bin/nomenclature/searchgenes.pl?field=symbol&anchor=equals&symbol_search=Search&number=100&format=html&sortby=symbol&match=MCCC2) |  | 609014 210210 | **1.74** |
| 1454844_AT | gb:AW049955 /DB_XREF=gi:5910484 /DB_XREF=UI-M-BH1- |  |  |  | **1.59** |
| 1454845_X_AT | gb:AW049955 /DB_XREF=gi:5910484 /DB_XREF=UI-M-BH1- |  |  |  | **1.55** |
| 1454856_X_AT | ribosomal protein L35 | [RPL35](http://www.gene.ucl.ac.uk/cgi-bin/nomenclature/searchgenes.pl?field=symbol&anchor=equals&symbol_search=Search&number=100&format=html&sortby=symbol&match=RPL35) |  |  | **-1.33** |
| 1454868_AT | expressed sequence AV028368 | [AV028368](http://www.gene.ucl.ac.uk/cgi-bin/nomenclature/searchgenes.pl?field=symbol&anchor=equals&symbol_search=Search&number=100&format=html&sortby=symbol&match=AV028368) |  |  | **1.47** |
| 1454877_AT | SERTA domain containing 4 | [SERTAD4](http://www.gene.ucl.ac.uk/cgi-bin/nomenclature/searchgenes.pl?field=symbol&anchor=equals&symbol_search=Search&number=100&format=html&sortby=symbol&match=SERTAD4) |  |  | **-1.97** |
| 1454878_AT | RIKEN cDNA 2310047C04 gene | [2310047C04RIK](http://www.gene.ucl.ac.uk/cgi-bin/nomenclature/searchgenes.pl?field=symbol&anchor=equals&symbol_search=Search&number=100&format=html&sortby=symbol&match=2310047C04RIK) |  |  | **-1.97** |
| 1454881_S_AT | uroplakin 3B | [UPK3B](http://www.gene.ucl.ac.uk/cgi-bin/nomenclature/searchgenes.pl?field=symbol&anchor=equals&symbol_search=Search&number=100&format=html&sortby=symbol&match=UPK3B) |  |  | **2.49** |
| 1454882_AT | l(3)mbt-like 3 (Drosophila) | [L3MBTL3](http://www.gene.ucl.ac.uk/cgi-bin/nomenclature/searchgenes.pl?field=symbol&anchor=equals&symbol_search=Search&number=100&format=html&sortby=symbol&match=L3MBTL3) |  |  | **-1.37** |
| 1454888_AT | Prefoldin 4 | [PFDN4](http://www.gene.ucl.ac.uk/cgi-bin/nomenclature/searchgenes.pl?field=symbol&anchor=equals&symbol_search=Search&number=100&format=html&sortby=symbol&match=PFDN4) |  | [604898](http://www.ncbi.nlm.nih.gov/entrez/dispomim.cgi?id=604898) | **-1.34** |
| 1454898_S_AT | RIKEN cDNA 4833421E05 gene | [4833421E05RIK](http://www.gene.ucl.ac.uk/cgi-bin/nomenclature/searchgenes.pl?field=symbol&anchor=equals&symbol_search=Search&number=100&format=html&sortby=symbol&match=4833421E05RIK) |  |  | **-1.69** |
| 1454916_S_AT | ADP-ribosylation factor interacting protein 1 | [ARFIP1](http://www.gene.ucl.ac.uk/cgi-bin/nomenclature/searchgenes.pl?field=symbol&anchor=equals&symbol_search=Search&number=100&format=html&sortby=symbol&match=ARFIP1) |  | [605928](http://www.ncbi.nlm.nih.gov/entrez/dispomim.cgi?id=605928) | **-1.54** |
| 1454921_AT | Gene model 561, (NCBI) | [LOC228715](http://www.gene.ucl.ac.uk/cgi-bin/nomenclature/searchgenes.pl?field=symbol&anchor=equals&symbol_search=Search&number=100&format=html&sortby=symbol&match=LOC228715) |  |  | **1.23** |
| 1454935_AT | gb:BB521491 /DB_XREF=gi:16443514 /DB_XREF=BB521491 |  |  |  | **1.39** |
| 1454961_AT | synaptojanin 1 | [SYNJ1](http://www.gene.ucl.ac.uk/cgi-bin/nomenclature/searchgenes.pl?field=symbol&anchor=equals&symbol_search=Search&number=100&format=html&sortby=symbol&match=SYNJ1) |  | [604297](http://www.ncbi.nlm.nih.gov/entrez/dispomim.cgi?id=604297) | **-1.33** |
| 1454965_AT | RIKEN cDNA D430039N05 gene | [D430039N05RIK](http://www.gene.ucl.ac.uk/cgi-bin/nomenclature/searchgenes.pl?field=symbol&anchor=equals&symbol_search=Search&number=100&format=html&sortby=symbol&match=D430039N05RIK) |  |  | **-2.61** |
| 1454971_X_AT | transforming growth factor beta 1 induced transcript 4 | [TGFB1I4](http://www.gene.ucl.ac.uk/cgi-bin/nomenclature/searchgenes.pl?field=symbol&anchor=equals&symbol_search=Search&number=100&format=html&sortby=symbol&match=TGFB1I4) |  | [607715](http://www.ncbi.nlm.nih.gov/entrez/dispomim.cgi?id=607715) | **-1.3** |
| 1454976_AT | Adult male urinary bladder cDNA, RIKEN full-length enriched library, clone:9530055M18 product:unknown EST, full insert sequence |  |  |  | **1.6** |
| 1454980_AT | RIKEN cDNA 4930402E16 gene | [4930402E16RIK](http://www.gene.ucl.ac.uk/cgi-bin/nomenclature/searchgenes.pl?field=symbol&anchor=equals&symbol_search=Search&number=100&format=html&sortby=symbol&match=4930402E16RIK) |  |  | **1.63** |
| 1454984_AT | gb:AV246615 /DB_XREF=gi:16387119 /DB_XREF=AV246615 |  |  |  | **1.48** |
| 1454997_AT | RIKEN cDNA D430026P16 gene | [MSRB3](http://www.gene.ucl.ac.uk/cgi-bin/nomenclature/searchgenes.pl?field=symbol&anchor=equals&symbol_search=Search&number=100&format=html&sortby=symbol&match=MSRB3) |  |  | **1.31** |
| 1455001_X_AT | gb:BF659630 /DB_XREF=gi:11924764 /DB_XREF=uz88h02. |  |  |  | **-1.66** |
| 1455014_AT | Expressed sequence AV009015 | [AV009015](http://www.gene.ucl.ac.uk/cgi-bin/nomenclature/searchgenes.pl?field=symbol&anchor=equals&symbol_search=Search&number=100&format=html&sortby=symbol&match=AV009015) |  |  | **1.33** |
| 1455018_AT | Lemur tyrosine kinase 2 | [LMTK2](http://www.gene.ucl.ac.uk/cgi-bin/nomenclature/searchgenes.pl?field=symbol&anchor=equals&symbol_search=Search&number=100&format=html&sortby=symbol&match=LMTK2) |  |  | **1.42** |
| 1455019_X_AT | Cytoskeleton-associated protein 4 | [CKAP4](http://www.gene.ucl.ac.uk/cgi-bin/nomenclature/searchgenes.pl?field=symbol&anchor=equals&symbol_search=Search&number=100&format=html&sortby=symbol&match=CKAP4) |  |  | **-2.98** |
| 1455029_AT | gb:BB342219 /DB_XREF=gi:16404563 /DB_XREF=BB342219 |  |  |  | **1.6** |
| 1455030_AT | 16 days neonate cerebellum cDNA, RIKEN full-length enriched library, clone:9630021C19 product:unknown EST, full insert sequence |  |  |  | **-1.42** |
| 1455037_AT | plexin A2 | [PLXNA2](http://www.gene.ucl.ac.uk/cgi-bin/nomenclature/searchgenes.pl?field=symbol&anchor=equals&symbol_search=Search&number=100&format=html&sortby=symbol&match=PLXNA2) |  | [601054](http://www.ncbi.nlm.nih.gov/entrez/dispomim.cgi?id=601054) | **1.37** |
| 1455039_A_AT | Transcriptional regulator, SIN3B (yeast) | [SIN3B](http://www.gene.ucl.ac.uk/cgi-bin/nomenclature/searchgenes.pl?field=symbol&anchor=equals&symbol_search=Search&number=100&format=html&sortby=symbol&match=SIN3B) |  | [607777](http://www.ncbi.nlm.nih.gov/entrez/dispomim.cgi?id=607777) | **-1.47** |
| 1455043_AT | transportin 1 | [TNPO1](http://www.gene.ucl.ac.uk/cgi-bin/nomenclature/searchgenes.pl?field=symbol&anchor=equals&symbol_search=Search&number=100&format=html&sortby=symbol&match=TNPO1) |  | [602901](http://www.ncbi.nlm.nih.gov/entrez/dispomim.cgi?id=602901) | **-1.59** |
| 1455048_AT | immunoglobulin superfamily, member 3 | [IGSF3](http://www.gene.ucl.ac.uk/cgi-bin/nomenclature/searchgenes.pl?field=symbol&anchor=equals&symbol_search=Search&number=100&format=html&sortby=symbol&match=IGSF3) |  | [603491](http://www.ncbi.nlm.nih.gov/entrez/dispomim.cgi?id=603491) | **-1.47** |
| 1455054_A_AT | testis derived transcript 3, pseudogene | [TES3-PS](http://www.gene.ucl.ac.uk/cgi-bin/nomenclature/searchgenes.pl?field=symbol&anchor=equals&symbol_search=Search&number=100&format=html&sortby=symbol&match=TES3-PS) |  |  | **-1.27** |
| 1455061_A_AT | acetyl-Coenzyme A acyltransferase 2 (mitochondrial 3-oxoacyl-Coenzyme A thiolase) | [ACAA2](http://www.gene.ucl.ac.uk/cgi-bin/nomenclature/searchgenes.pl?field=symbol&anchor=equals&symbol_search=Search&number=100&format=html&sortby=symbol&match=ACAA2) |  | [604770](http://www.ncbi.nlm.nih.gov/entrez/dispomim.cgi?id=604770) | **1.93** |
| 1455065_X_AT | glucosamine-6-phosphate deaminase 1 | [GNPDA1](http://www.gene.ucl.ac.uk/cgi-bin/nomenclature/searchgenes.pl?field=symbol&anchor=equals&symbol_search=Search&number=100&format=html&sortby=symbol&match=GNPDA1) |  | [601798](http://www.ncbi.nlm.nih.gov/entrez/dispomim.cgi?id=601798) | **-1.44** |
| 1455090_AT | gb:BF681826 /DB_XREF=gi:11955721 /DB_XREF=60211765 |  |  |  | **-1.62** |
| 1455098_A_AT | vitronectin | [VTN](http://www.gene.ucl.ac.uk/cgi-bin/nomenclature/searchgenes.pl?field=symbol&anchor=equals&symbol_search=Search&number=100&format=html&sortby=symbol&match=VTN) |  | [193190](http://www.ncbi.nlm.nih.gov/entrez/dispomim.cgi?id=193190) | **2.63** |
| 1455105_AT | protein tyrosine phosphatase, non-receptor type 12 | [PTPN12](http://www.gene.ucl.ac.uk/cgi-bin/nomenclature/searchgenes.pl?field=symbol&anchor=equals&symbol_search=Search&number=100&format=html&sortby=symbol&match=PTPN12) |  | [600079](http://www.ncbi.nlm.nih.gov/entrez/dispomim.cgi?id=600079) | **-1.46** |
| 1455106_A_AT | creatine kinase, brain | [CKB](http://www.gene.ucl.ac.uk/cgi-bin/nomenclature/searchgenes.pl?field=symbol&anchor=equals&symbol_search=Search&number=100&format=html&sortby=symbol&match=CKB) |  | [123280](http://www.ncbi.nlm.nih.gov/entrez/dispomim.cgi?id=123280) | **-1.91** |
| 1455112_AT | apoptosis-inducing factor (AIF)-like mitochondrion-associated inducer of death | [AMID](http://www.gene.ucl.ac.uk/cgi-bin/nomenclature/searchgenes.pl?field=symbol&anchor=equals&symbol_search=Search&number=100&format=html&sortby=symbol&match=AMID) |  |  | **2.08** |
| 1455137_AT | gb:AW536472 /DB_XREF=gi:7178889 /DB_XREF=G0104H06- |  |  |  | **1.27** |
| 1455138_X_AT | cofilin 1, non-muscle | [CFL1](http://www.gene.ucl.ac.uk/cgi-bin/nomenclature/searchgenes.pl?field=symbol&anchor=equals&symbol_search=Search&number=100&format=html&sortby=symbol&match=CFL1) |  | [601442](http://www.ncbi.nlm.nih.gov/entrez/dispomim.cgi?id=601442) | **-1.75** |
| 1455140_AT | gb:AV376568 /DB_XREF=gi:15412300 /DB_XREF=AV376568 |  |  |  | **1.96** |
| 1455144_S_AT | expressed sequence AU040829 | [AU040829](http://www.gene.ucl.ac.uk/cgi-bin/nomenclature/searchgenes.pl?field=symbol&anchor=equals&symbol_search=Search&number=100&format=html&sortby=symbol&match=AU040829) |  |  | **1.59** |
| 1455160_AT | RIKEN cDNA 2610203C20 gene | [2610203C20RIK](http://www.gene.ucl.ac.uk/cgi-bin/nomenclature/searchgenes.pl?field=symbol&anchor=equals&symbol_search=Search&number=100&format=html&sortby=symbol&match=2610203C20RIK) |  |  | **-1.51** |
| 1455164_AT | Cdc42 GTPase-activating protein | [CDGAP](http://www.gene.ucl.ac.uk/cgi-bin/nomenclature/searchgenes.pl?field=symbol&anchor=equals&symbol_search=Search&number=100&format=html&sortby=symbol&match=CDGAP) |  |  | **1.86** |
| 1455184_AT | hypothetical protein B230364F10 | [B230364F10](http://www.gene.ucl.ac.uk/cgi-bin/nomenclature/searchgenes.pl?field=symbol&anchor=equals&symbol_search=Search&number=100&format=html&sortby=symbol&match=B230364F10) |  |  | **-1.5** |
| 1455203_AT | RIKEN cDNA A930003A15 gene | [A930003A15RIK](http://www.gene.ucl.ac.uk/cgi-bin/nomenclature/searchgenes.pl?field=symbol&anchor=equals&symbol_search=Search&number=100&format=html&sortby=symbol&match=A930003A15RIK) |  |  | **-12.01** |
| 1455204_AT | phosphatidylinositol transfer protein, cytoplasmic 1 | [PITPNC1](http://www.gene.ucl.ac.uk/cgi-bin/nomenclature/searchgenes.pl?field=symbol&anchor=equals&symbol_search=Search&number=100&format=html&sortby=symbol&match=PITPNC1) |  | [605134](http://www.ncbi.nlm.nih.gov/entrez/dispomim.cgi?id=605134) | **1.55** |
| 1455206_AT | hypothetical protein C130006E23 | [C130006E23](http://www.gene.ucl.ac.uk/cgi-bin/nomenclature/searchgenes.pl?field=symbol&anchor=equals&symbol_search=Search&number=100&format=html&sortby=symbol&match=C130006E23) |  |  | **-1.45** |
| 1455214_AT | microphthalmia-associated transcription factor | [MITF](http://www.gene.ucl.ac.uk/cgi-bin/nomenclature/searchgenes.pl?field=symbol&anchor=equals&symbol_search=Search&number=100&format=html&sortby=symbol&match=MITF) |  | [156845](http://www.ncbi.nlm.nih.gov/entrez/dispomim.cgi?id=156845) | **1.22** |
| 1455224_AT | Angiopoietin-like 1 | [ANGPTL1](http://www.gene.ucl.ac.uk/cgi-bin/nomenclature/searchgenes.pl?field=symbol&anchor=equals&symbol_search=Search&number=100&format=html&sortby=symbol&match=ANGPTL1) |  | [603874](http://www.ncbi.nlm.nih.gov/entrez/dispomim.cgi?id=603874) | **-1.78** |
| 1455226_AT | similar to spectrin beta 1 | [LOC383567](http://www.gene.ucl.ac.uk/cgi-bin/nomenclature/searchgenes.pl?field=symbol&anchor=equals&symbol_search=Search&number=100&format=html&sortby=symbol&match=LOC383567) |  |  | **1.4** |
| 1455227_AT | RIKEN cDNA B230106I24 gene | [B230106I24RIK](http://www.gene.ucl.ac.uk/cgi-bin/nomenclature/searchgenes.pl?field=symbol&anchor=equals&symbol_search=Search&number=100&format=html&sortby=symbol&match=B230106I24RIK) |  |  | **1.65** |
| 1455235_X_AT | lactate dehydrogenase 2, B chain | [LDH2](http://www.gene.ucl.ac.uk/cgi-bin/nomenclature/searchgenes.pl?field=symbol&anchor=equals&symbol_search=Search&number=100&format=html&sortby=symbol&match=LDH2) |  |  | **1.53** |
| 1455245_X_AT | ribosomal protein L13 | [RPL13](http://www.gene.ucl.ac.uk/cgi-bin/nomenclature/searchgenes.pl?field=symbol&anchor=equals&symbol_search=Search&number=100&format=html&sortby=symbol&match=RPL13) |  | [113703](http://www.ncbi.nlm.nih.gov/entrez/dispomim.cgi?id=113703) | **-1.22** |
| 1455246_AT | gb:BF137264 /DB_XREF=gi:10976304 /DB_XREF=60178162 |  |  |  | **-1.37** |
| 1455267_AT | estrogen-related receptor gamma | [ESRRG](http://www.gene.ucl.ac.uk/cgi-bin/nomenclature/searchgenes.pl?field=symbol&anchor=equals&symbol_search=Search&number=100&format=html&sortby=symbol&match=ESRRG) |  | [602969](http://www.ncbi.nlm.nih.gov/entrez/dispomim.cgi?id=602969) | **1.35** |
| 1455269_A_AT | coronin, actin binding protein 1A | [CORO1A](http://www.gene.ucl.ac.uk/cgi-bin/nomenclature/searchgenes.pl?field=symbol&anchor=equals&symbol_search=Search&number=100&format=html&sortby=symbol&match=CORO1A) |  | [605000](http://www.ncbi.nlm.nih.gov/entrez/dispomim.cgi?id=605000) | **-5.53** |
| 1455271_AT | RIKEN cDNA 1810020C19 gene | [1810020C19RIK](http://www.gene.ucl.ac.uk/cgi-bin/nomenclature/searchgenes.pl?field=symbol&anchor=equals&symbol_search=Search&number=100&format=html&sortby=symbol&match=1810020C19RIK) |  |  | **-3.32** |
| 1455287_AT | hypothetical protein 5830411I20 | [5830411I20](http://www.gene.ucl.ac.uk/cgi-bin/nomenclature/searchgenes.pl?field=symbol&anchor=equals&symbol_search=Search&number=100&format=html&sortby=symbol&match=5830411I20) |  |  | **-1.54** |
| 1455291_S_AT | DNA segment, Chr 6, ERATO Doi 365, expressed | [D6ERTD365E](http://www.gene.ucl.ac.uk/cgi-bin/nomenclature/searchgenes.pl?field=symbol&anchor=equals&symbol_search=Search&number=100&format=html&sortby=symbol&match=D6ERTD365E) |  |  | **1.35** |
| 1455319_X_AT | ribosomal protein S8 | [RPS8](http://www.gene.ucl.ac.uk/cgi-bin/nomenclature/searchgenes.pl?field=symbol&anchor=equals&symbol_search=Search&number=100&format=html&sortby=symbol&match=RPS8) |  | [600357](http://www.ncbi.nlm.nih.gov/entrez/dispomim.cgi?id=600357) | **-1.53** |
| 1455320_AT | gb:BQ176847 /DB_XREF=gi:20352339 /DB_XREF=UI-M-DJ2 |  |  |  | **1.65** |
| 1455332_X_AT | Fc receptor, IgG, low affinity IIb | [FCGR2B](http://www.gene.ucl.ac.uk/cgi-bin/nomenclature/searchgenes.pl?field=symbol&anchor=equals&symbol_search=Search&number=100&format=html&sortby=symbol&match=FCGR2B) |  | [604590](http://www.ncbi.nlm.nih.gov/entrez/dispomim.cgi?id=604590) | **-2.46** |
| 1455342_AT | gb:BM232966 /DB_XREF=gi:17868236 /DB_XREF=K0330E07 |  |  |  | **-7.14** |
| 1455346_AT | gb:BB477214 /DB_XREF=gi:16440274 /DB_XREF=BB477214 |  |  |  | **1.96** |
| 1455349_AT | RAS related protein 1b | [RAP1B](http://www.gene.ucl.ac.uk/cgi-bin/nomenclature/searchgenes.pl?field=symbol&anchor=equals&symbol_search=Search&number=100&format=html&sortby=symbol&match=RAP1B) |  | [179530](http://www.ncbi.nlm.nih.gov/entrez/dispomim.cgi?id=179530) | **-1.28** |
| 1455350_AT | RIKEN cDNA B830009D23 gene | [B830009D23RIK](http://www.gene.ucl.ac.uk/cgi-bin/nomenclature/searchgenes.pl?field=symbol&anchor=equals&symbol_search=Search&number=100&format=html&sortby=symbol&match=B830009D23RIK) |  |  | **-2.12** |
| 1455352_AT | RIKEN cDNA 2610101N10 gene | [2610101N10RIK](http://www.gene.ucl.ac.uk/cgi-bin/nomenclature/searchgenes.pl?field=symbol&anchor=equals&symbol_search=Search&number=100&format=html&sortby=symbol&match=2610101N10RIK) |  |  | **1.35** |
| 1455364_A_AT | ribosomal protein S7 | [RPS7](http://www.gene.ucl.ac.uk/cgi-bin/nomenclature/searchgenes.pl?field=symbol&anchor=equals&symbol_search=Search&number=100&format=html&sortby=symbol&match=RPS7) |  | [603658](http://www.ncbi.nlm.nih.gov/entrez/dispomim.cgi?id=603658) | **-1.35** |
| 1455372_AT | gb:BB770826 /DB_XREF=gi:16197205 /DB_XREF=BB770826 |  |  |  | **1.76** |
| 1455376_AT | RIKEN cDNA 1300010F03 gene | [1300010F03RIK](http://www.gene.ucl.ac.uk/cgi-bin/nomenclature/searchgenes.pl?field=symbol&anchor=equals&symbol_search=Search&number=100&format=html&sortby=symbol&match=1300010F03RIK) |  |  | **1.32** |
| 1455385_AT | SEC15-like 1 (S. cerevisiae) | [SEC15L1](http://www.gene.ucl.ac.uk/cgi-bin/nomenclature/searchgenes.pl?field=symbol&anchor=equals&symbol_search=Search&number=100&format=html&sortby=symbol&match=SEC15L1) |  |  | **1.32** |
| 1455388_AT | RIKEN cDNA A030012M09 gene | [A030012M09RIK](http://www.gene.ucl.ac.uk/cgi-bin/nomenclature/searchgenes.pl?field=symbol&anchor=equals&symbol_search=Search&number=100&format=html&sortby=symbol&match=A030012M09RIK) |  |  | **1.29** |
| 1455391_AT | RAD23a homolog (S. cerevisiae) | [RAD23A](http://www.gene.ucl.ac.uk/cgi-bin/nomenclature/searchgenes.pl?field=symbol&anchor=equals&symbol_search=Search&number=100&format=html&sortby=symbol&match=RAD23A) |  | [600061](http://www.ncbi.nlm.nih.gov/entrez/dispomim.cgi?id=600061) | **1.21** |
| 1455398_AT | RIKEN cDNA E430036I04 gene | [E430036I04RIK](http://www.gene.ucl.ac.uk/cgi-bin/nomenclature/searchgenes.pl?field=symbol&anchor=equals&symbol_search=Search&number=100&format=html&sortby=symbol&match=E430036I04RIK) |  |  | **1.43** |
| 1455399_AT | Connector enhancer of kinase suppressor of Ras 1 | [CNKSR1](http://www.gene.ucl.ac.uk/cgi-bin/nomenclature/searchgenes.pl?field=symbol&anchor=equals&symbol_search=Search&number=100&format=html&sortby=symbol&match=CNKSR1) |  | [603272](http://www.ncbi.nlm.nih.gov/entrez/dispomim.cgi?id=603272) | **-1.69** |
| 1455405_AT | gb:AV229693 /DB_XREF=gi:16385660 /DB_XREF=AV229693 |  |  |  | **-1.35** |
| 1455426_AT | gb:AV226618 /DB_XREF=gi:16385351 /DB_XREF=AV226618 |  |  |  | **-2** |
| 1455439_A_AT | lectin, galactose binding, soluble 1 | [LGALS1](http://www.gene.ucl.ac.uk/cgi-bin/nomenclature/searchgenes.pl?field=symbol&anchor=equals&symbol_search=Search&number=100&format=html&sortby=symbol&match=LGALS1) |  | [150570](http://www.ncbi.nlm.nih.gov/entrez/dispomim.cgi?id=150570) | **-1.83** |
| 1455446_X_AT | acyl-Coenzyme A dehydrogenase, short/branched chain | [ACADSB](http://www.gene.ucl.ac.uk/cgi-bin/nomenclature/searchgenes.pl?field=symbol&anchor=equals&symbol_search=Search&number=100&format=html&sortby=symbol&match=ACADSB) |  | [600301](http://www.ncbi.nlm.nih.gov/entrez/dispomim.cgi?id=600301) | **1.52** |
| 1455448_AT | neural stem cell-derived dendrite regulator | [NSDDR](http://www.gene.ucl.ac.uk/cgi-bin/nomenclature/searchgenes.pl?field=symbol&anchor=equals&symbol_search=Search&number=100&format=html&sortby=symbol&match=NSDDR) |  |  | **1.58** |
| 1455450_AT | gb:AV231357 /DB_XREF=gi:16385846 /DB_XREF=AV231357 |  |  |  | **2.06** |
| 1455456_A_AT | translocase of inner mitochondrial membrane 50 homolog (yeast) | [TIMM50](http://www.gene.ucl.ac.uk/cgi-bin/nomenclature/searchgenes.pl?field=symbol&anchor=equals&symbol_search=Search&number=100&format=html&sortby=symbol&match=TIMM50) |  | [607381](http://www.ncbi.nlm.nih.gov/entrez/dispomim.cgi?id=607381) | **1.3** |
| 1455462_AT | adenylate cyclase 2 | [ADCY2](http://www.gene.ucl.ac.uk/cgi-bin/nomenclature/searchgenes.pl?field=symbol&anchor=equals&symbol_search=Search&number=100&format=html&sortby=symbol&match=ADCY2) |  | [103071](http://www.ncbi.nlm.nih.gov/entrez/dispomim.cgi?id=103071) | **1.46** |
| 1455464_X_AT | gb:BB427704 /DB_XREF=gi:16423841 /DB_XREF=BB427704 |  |  |  | **2.02** |
| 1455466_AT | G protein-coupled receptor 133 | [GPR133](http://www.gene.ucl.ac.uk/cgi-bin/nomenclature/searchgenes.pl?field=symbol&anchor=equals&symbol_search=Search&number=100&format=html&sortby=symbol&match=GPR133) |  |  | **1.77** |
| 1455468_AT | RIKEN cDNA 9330164H19 gene | [9330164H19RIK](http://www.gene.ucl.ac.uk/cgi-bin/nomenclature/searchgenes.pl?field=symbol&anchor=equals&symbol_search=Search&number=100&format=html&sortby=symbol&match=9330164H19RIK) |  |  | **-1.65** |
| 1455473_AT | gb:AV347341 /DB_XREF=gi:16397092 /DB_XREF=AV347341 |  |  |  | **1.54** |
| 1455475_AT | RIKEN cDNA 3110057O12 gene | [3110057O12RIK](http://www.gene.ucl.ac.uk/cgi-bin/nomenclature/searchgenes.pl?field=symbol&anchor=equals&symbol_search=Search&number=100&format=html&sortby=symbol&match=3110057O12RIK) |  |  | **1.58** |
| 1455485_X_AT | ribosomal protein L13a | [RPL13A](http://www.gene.ucl.ac.uk/cgi-bin/nomenclature/searchgenes.pl?field=symbol&anchor=equals&symbol_search=Search&number=100&format=html&sortby=symbol&match=RPL13A) |  |  | **-1.34** |
| 1455494_AT | gb:BI794771 /DB_XREF=gi:15822496 /DB_XREF=ic64f12. |  |  |  | **-3.04** |
| 1455506_AT | gb:BE650825 /DB_XREF=gi:9976649 /DB_XREF=UI-M-BH3- |  |  |  | **2.06** |
| 1455515_AT | gb:BQ177117 /DB_XREF=gi:20352609 /DB_XREF=UI-M-DJ2 |  |  |  | **-2.53** |
| 1455522_AT | RIKEN cDNA D530030K12 gene | [ARHGEF15](http://www.gene.ucl.ac.uk/cgi-bin/nomenclature/searchgenes.pl?field=symbol&anchor=equals&symbol_search=Search&number=100&format=html&sortby=symbol&match=ARHGEF15) |  |  | **2.18** |
| 1455531_AT | RIKEN cDNA A930031D07 gene | [A930031D07RIK](http://www.gene.ucl.ac.uk/cgi-bin/nomenclature/searchgenes.pl?field=symbol&anchor=equals&symbol_search=Search&number=100&format=html&sortby=symbol&match=A930031D07RIK) |  |  | **2.79** |
| 1455548_AT | discs, large homolog-associated protein 4 (Drosophila) | [DLGAP4](http://www.gene.ucl.ac.uk/cgi-bin/nomenclature/searchgenes.pl?field=symbol&anchor=equals&symbol_search=Search&number=100&format=html&sortby=symbol&match=DLGAP4) |  |  | **-2.38** |
| 1455549_AT | SEC14 and spectrin domains 1 | [SESTD1](http://www.gene.ucl.ac.uk/cgi-bin/nomenclature/searchgenes.pl?field=symbol&anchor=equals&symbol_search=Search&number=100&format=html&sortby=symbol&match=SESTD1) |  |  | **-1.47** |
| 1455556_AT | Notch gene homolog 2 (Drosophila) | [NOTCH2](http://www.gene.ucl.ac.uk/cgi-bin/nomenclature/searchgenes.pl?field=symbol&anchor=equals&symbol_search=Search&number=100&format=html&sortby=symbol&match=NOTCH2) |  | [600275](http://www.ncbi.nlm.nih.gov/entrez/dispomim.cgi?id=600275) | **-1.66** |
| 1455558_AT | Gene model 114, (NCBI) | [LOC228730](http://www.gene.ucl.ac.uk/cgi-bin/nomenclature/searchgenes.pl?field=symbol&anchor=equals&symbol_search=Search&number=100&format=html&sortby=symbol&match=LOC228730) |  |  | **1.78** |
| 1455570_X_AT | calponin 3, acidic | [CNN3](http://www.gene.ucl.ac.uk/cgi-bin/nomenclature/searchgenes.pl?field=symbol&anchor=equals&symbol_search=Search&number=100&format=html&sortby=symbol&match=CNN3) |  | [602374](http://www.ncbi.nlm.nih.gov/entrez/dispomim.cgi?id=602374) | **-1.86** |
| 1455572_X_AT | ribosomal protein S18 | [RPS18](http://www.gene.ucl.ac.uk/cgi-bin/nomenclature/searchgenes.pl?field=symbol&anchor=equals&symbol_search=Search&number=100&format=html&sortby=symbol&match=RPS18) |  | [180473](http://www.ncbi.nlm.nih.gov/entrez/dispomim.cgi?id=180473) | **-1.3** |
| 1455578_X_AT | ribosomal protein L41 | [RPL41](http://www.gene.ucl.ac.uk/cgi-bin/nomenclature/searchgenes.pl?field=symbol&anchor=equals&symbol_search=Search&number=100&format=html&sortby=symbol&match=RPL41) |  |  | **-1.27** |
| 1455607_AT | thrombospondin, type I, domain 2 | [THSD2](http://www.gene.ucl.ac.uk/cgi-bin/nomenclature/searchgenes.pl?field=symbol&anchor=equals&symbol_search=Search&number=100&format=html&sortby=symbol&match=THSD2) |  |  | **-2.24** |
| 1455617_AT | RIKEN cDNA 0910001K20 gene | [0910001K20RIK](http://www.gene.ucl.ac.uk/cgi-bin/nomenclature/searchgenes.pl?field=symbol&anchor=equals&symbol_search=Search&number=100&format=html&sortby=symbol&match=0910001K20RIK) |  |  | **1.35** |
| 1455622_AT | podocalyxin-like 2 | [PODXL2](http://www.gene.ucl.ac.uk/cgi-bin/nomenclature/searchgenes.pl?field=symbol&anchor=equals&symbol_search=Search&number=100&format=html&sortby=symbol&match=PODXL2) |  |  | **2.31** |
| 1455627_AT | procollagen, type VIII, alpha 1 | [COL8A1](http://www.gene.ucl.ac.uk/cgi-bin/nomenclature/searchgenes.pl?field=symbol&anchor=equals&symbol_search=Search&number=100&format=html&sortby=symbol&match=COL8A1) |  | [120251](http://www.ncbi.nlm.nih.gov/entrez/dispomim.cgi?id=120251) | **-3.9** |
| 1455642_A_AT | F-box only protein 23 | [TM4SF17](http://www.gene.ucl.ac.uk/cgi-bin/nomenclature/searchgenes.pl?field=symbol&anchor=equals&symbol_search=Search&number=100&format=html&sortby=symbol&match=TM4SF17) |  |  | **-1.82** |
| 1455649_AT | gb:BB106428 /DB_XREF=gi:8758996 /DB_XREF=BB106428 |  |  |  | **-2.39** |
| 1455655_A_AT | TAR DNA binding protein | [TARDBP](http://www.gene.ucl.ac.uk/cgi-bin/nomenclature/searchgenes.pl?field=symbol&anchor=equals&symbol_search=Search&number=100&format=html&sortby=symbol&match=TARDBP) |  | [605078](http://www.ncbi.nlm.nih.gov/entrez/dispomim.cgi?id=605078) | **-1.21** |
| 1455660_AT | gb:BB769628 /DB_XREF=gi:16212170 /DB_XREF=BB769628 |  |  |  | **-2.67** |
| 1455679_AT | RIKEN cDNA 5830411E10 gene | [5830411E10RIK](http://www.gene.ucl.ac.uk/cgi-bin/nomenclature/searchgenes.pl?field=symbol&anchor=equals&symbol_search=Search&number=100&format=html&sortby=symbol&match=5830411E10RIK) |  |  | **-2.07** |
| 1455680_AT | RIKEN cDNA 9630025H16 gene | [9630025H16RIK](http://www.gene.ucl.ac.uk/cgi-bin/nomenclature/searchgenes.pl?field=symbol&anchor=equals&symbol_search=Search&number=100&format=html&sortby=symbol&match=9630025H16RIK) |  |  | **1.74** |
| 1455688_AT | hypothetical LOC98434 | [LOC98434](http://www.gene.ucl.ac.uk/cgi-bin/nomenclature/searchgenes.pl?field=symbol&anchor=equals&symbol_search=Search&number=100&format=html&sortby=symbol&match=LOC98434) |  |  | **-1.39** |
| 1455700_AT | RIKEN cDNA 1700007D05 gene | [1700007D05RIK](http://www.gene.ucl.ac.uk/cgi-bin/nomenclature/searchgenes.pl?field=symbol&anchor=equals&symbol_search=Search&number=100&format=html&sortby=symbol&match=1700007D05RIK) |  |  | **1.36** |
| 1455719_AT | tubulin, beta 5 | [TUBB5](http://www.gene.ucl.ac.uk/cgi-bin/nomenclature/searchgenes.pl?field=symbol&anchor=equals&symbol_search=Search&number=100&format=html&sortby=symbol&match=TUBB5) |  | [602662](http://www.ncbi.nlm.nih.gov/entrez/dispomim.cgi?id=602662) | **-1.37** |
| 1455725_A_AT | H3 histone, family 3B | [H3F3B](http://www.gene.ucl.ac.uk/cgi-bin/nomenclature/searchgenes.pl?field=symbol&anchor=equals&symbol_search=Search&number=100&format=html&sortby=symbol&match=H3F3B) |  | [601058](http://www.ncbi.nlm.nih.gov/entrez/dispomim.cgi?id=601058) | **-1.31** |
| 1455731_AT | solute carrier family 29 (nucleoside transporters), member 3 | [SLC29A3](http://www.gene.ucl.ac.uk/cgi-bin/nomenclature/searchgenes.pl?field=symbol&anchor=equals&symbol_search=Search&number=100&format=html&sortby=symbol&match=SLC29A3) |  |  | **-1.51** |
| 1455750_AT | RIKEN cDNA A230067G21 gene | [9630019E01RIK](http://www.gene.ucl.ac.uk/cgi-bin/nomenclature/searchgenes.pl?field=symbol&anchor=equals&symbol_search=Search&number=100&format=html&sortby=symbol&match=9630019E01RIK) |  |  | **1.42** |
| 1455762_AT | RIKEN cDNA C330002I19 gene | [C330002I19RIK](http://www.gene.ucl.ac.uk/cgi-bin/nomenclature/searchgenes.pl?field=symbol&anchor=equals&symbol_search=Search&number=100&format=html&sortby=symbol&match=C330002I19RIK) |  |  | **-1.93** |
| 1455767_X_AT | ribosomal protein L21 | [RPL21](http://www.gene.ucl.ac.uk/cgi-bin/nomenclature/searchgenes.pl?field=symbol&anchor=equals&symbol_search=Search&number=100&format=html&sortby=symbol&match=RPL21) |  | [603636](http://www.ncbi.nlm.nih.gov/entrez/dispomim.cgi?id=603636) | **-1.28** |
| 1455768_AT | gb:BG071996 /DB_XREF=gi:12554565 /DB_XREF=H3105E07 |  |  |  | **-1.49** |
| 1455773_AT | gb:BG797225 /DB_XREF=gi:14143794 /DB_XREF=ic12d08. |  |  |  | **1.5** |
| 1455777_X_AT | hydroxysteroid (17-beta) dehydrogenase 4 | [HSD17B4](http://www.gene.ucl.ac.uk/cgi-bin/nomenclature/searchgenes.pl?field=symbol&anchor=equals&symbol_search=Search&number=100&format=html&sortby=symbol&match=HSD17B4) |  | [601860](http://www.ncbi.nlm.nih.gov/entrez/dispomim.cgi?id=601860) | **1.59** |
| 1455785_AT | gb:BQ175978 /DB_XREF=gi:20351470 /DB_XREF=UI-M-DJ2 |  |  |  | **1.97** |
| 1455794_AT | RIKEN cDNA D130058I21 gene | [D130058I21RIK](http://www.gene.ucl.ac.uk/cgi-bin/nomenclature/searchgenes.pl?field=symbol&anchor=equals&symbol_search=Search&number=100&format=html&sortby=symbol&match=D130058I21RIK) |  |  | **2.13** |
| 1455795_AT | squamous cell carcinoma antigen recognized by T cells 2 | [SART2](http://www.gene.ucl.ac.uk/cgi-bin/nomenclature/searchgenes.pl?field=symbol&anchor=equals&symbol_search=Search&number=100&format=html&sortby=symbol&match=SART2) |  | [605942](http://www.ncbi.nlm.nih.gov/entrez/dispomim.cgi?id=605942) | **-2.1** |
| 1455798_AT | galactokinase 2 | [GALK2](http://www.gene.ucl.ac.uk/cgi-bin/nomenclature/searchgenes.pl?field=symbol&anchor=equals&symbol_search=Search&number=100&format=html&sortby=symbol&match=GALK2) |  | [137028](http://www.ncbi.nlm.nih.gov/entrez/dispomim.cgi?id=137028) | **-1.84** |
| 1455806_X_AT | RIKEN cDNA 2410011G03 gene | [2410011G03RIK](http://www.gene.ucl.ac.uk/cgi-bin/nomenclature/searchgenes.pl?field=symbol&anchor=equals&symbol_search=Search&number=100&format=html&sortby=symbol&match=2410011G03RIK) |  |  | **1.33** |
| 1455814_X_AT | DEAD (Asp-Glu-Ala-Asp) box polypeptide 39 | [DDX39](http://www.gene.ucl.ac.uk/cgi-bin/nomenclature/searchgenes.pl?field=symbol&anchor=equals&symbol_search=Search&number=100&format=html&sortby=symbol&match=DDX39) |  |  | **-1.91** |
| 1455816_A_AT | potassium channel tetramerisation domain containing 3 | [KCTD3](http://www.gene.ucl.ac.uk/cgi-bin/nomenclature/searchgenes.pl?field=symbol&anchor=equals&symbol_search=Search&number=100&format=html&sortby=symbol&match=KCTD3) |  |  | **-1.83** |
| 1455824_X_AT | intergral membrane protein 1 | [ITM1](http://www.gene.ucl.ac.uk/cgi-bin/nomenclature/searchgenes.pl?field=symbol&anchor=equals&symbol_search=Search&number=100&format=html&sortby=symbol&match=ITM1) |  | [601134](http://www.ncbi.nlm.nih.gov/entrez/dispomim.cgi?id=601134) | **-1.68** |
| 1455825_S_AT | ligand of numb-protein X 1 | [LNX1](http://www.gene.ucl.ac.uk/cgi-bin/nomenclature/searchgenes.pl?field=symbol&anchor=equals&symbol_search=Search&number=100&format=html&sortby=symbol&match=LNX1) |  |  | **1.6** |
| 1455826_A_AT | beta-site APP cleaving enzyme 1 | [BACE1](http://www.gene.ucl.ac.uk/cgi-bin/nomenclature/searchgenes.pl?field=symbol&anchor=equals&symbol_search=Search&number=100&format=html&sortby=symbol&match=BACE1) |  | [604252](http://www.ncbi.nlm.nih.gov/entrez/dispomim.cgi?id=604252) | **-1.51** |
| 1455827_AT | muscleblind-like 2 | [MBNL2](http://www.gene.ucl.ac.uk/cgi-bin/nomenclature/searchgenes.pl?field=symbol&anchor=equals&symbol_search=Search&number=100&format=html&sortby=symbol&match=MBNL2) |  | [607327](http://www.ncbi.nlm.nih.gov/entrez/dispomim.cgi?id=607327) | **1.21** |
| 1455842_X_AT | gb:AW536567 /DB_XREF=gi:7178984 /DB_XREF=G0106A11- |  |  |  | **1.69** |
| 1455855_X_AT | heterogeneous nuclear ribonucleoprotein A/B | [HNRPAB](http://www.gene.ucl.ac.uk/cgi-bin/nomenclature/searchgenes.pl?field=symbol&anchor=equals&symbol_search=Search&number=100&format=html&sortby=symbol&match=HNRPAB) |  | [602688](http://www.ncbi.nlm.nih.gov/entrez/dispomim.cgi?id=602688) | **-1.29** |
| 1455862_AT | RIKEN cDNA 9630054F20 gene | [9630054F20RIK](http://www.gene.ucl.ac.uk/cgi-bin/nomenclature/searchgenes.pl?field=symbol&anchor=equals&symbol_search=Search&number=100&format=html&sortby=symbol&match=9630054F20RIK) |  |  | **-2.03** |
| 1455871_S_AT | Tax1 (human T-cell leukemia virus type I) binding protein 3 | [RPL13](http://www.gene.ucl.ac.uk/cgi-bin/nomenclature/searchgenes.pl?field=symbol&anchor=equals&symbol_search=Search&number=100&format=html&sortby=symbol&match=RPL13) |  | [113703](http://www.ncbi.nlm.nih.gov/entrez/dispomim.cgi?id=113703) | **-1.28** |
| 1455884_AT | dipeptidylpeptidase 9 | [DPP9](http://www.gene.ucl.ac.uk/cgi-bin/nomenclature/searchgenes.pl?field=symbol&anchor=equals&symbol_search=Search&number=100&format=html&sortby=symbol&match=DPP9) |  | [608258](http://www.ncbi.nlm.nih.gov/entrez/dispomim.cgi?id=608258) | **1.43** |
| 1455897_X_AT | high mobility group nucleosomal binding domain 1 | [HMGN1](http://www.gene.ucl.ac.uk/cgi-bin/nomenclature/searchgenes.pl?field=symbol&anchor=equals&symbol_search=Search&number=100&format=html&sortby=symbol&match=HMGN1) |  | [163920](http://www.ncbi.nlm.nih.gov/entrez/dispomim.cgi?id=163920) | **-1.4** |
| 1455899_X_AT | suppressor of cytokine signaling 3 | [SOCS3](http://www.gene.ucl.ac.uk/cgi-bin/nomenclature/searchgenes.pl?field=symbol&anchor=equals&symbol_search=Search&number=100&format=html&sortby=symbol&match=SOCS3) |  | [604176](http://www.ncbi.nlm.nih.gov/entrez/dispomim.cgi?id=604176) | **-3.77** |
| 1455901_AT | choline phosphotransferase 1 | [CHPT1](http://www.gene.ucl.ac.uk/cgi-bin/nomenclature/searchgenes.pl?field=symbol&anchor=equals&symbol_search=Search&number=100&format=html&sortby=symbol&match=CHPT1) |  |  | **1.73** |
| 1455908_A_AT | serine caroboxypeptidase 1 | [SCPEP1](http://www.gene.ucl.ac.uk/cgi-bin/nomenclature/searchgenes.pl?field=symbol&anchor=equals&symbol_search=Search&number=100&format=html&sortby=symbol&match=SCPEP1) |  |  | **-1.56** |
| 1455950_X_AT | ribosomal protein L35 | [RPL35](http://www.gene.ucl.ac.uk/cgi-bin/nomenclature/searchgenes.pl?field=symbol&anchor=equals&symbol_search=Search&number=100&format=html&sortby=symbol&match=RPL35) |  |  | **-1.5** |
| 1455958_S_AT | RIKEN cDNA 9130017A15 gene | [9130017A15RIK](http://www.gene.ucl.ac.uk/cgi-bin/nomenclature/searchgenes.pl?field=symbol&anchor=equals&symbol_search=Search&number=100&format=html&sortby=symbol&match=9130017A15RIK) |  |  | **1.57** |
| 1455968_X_AT | gb:AA409396 /DB_XREF=gi:2066790 /DB_XREF=EST01875 |  |  |  | **-1.22** |
| 1455972_X_AT | L-3-hydroxyacyl-Coenzyme A dehydrogenase, short chain | [HADHSC](http://www.gene.ucl.ac.uk/cgi-bin/nomenclature/searchgenes.pl?field=symbol&anchor=equals&symbol_search=Search&number=100&format=html&sortby=symbol&match=HADHSC) |  | [601609](http://www.ncbi.nlm.nih.gov/entrez/dispomim.cgi?id=601609) | **1.48** |
| 1455978_A_AT | matrilin 2 | [MATN2](http://www.gene.ucl.ac.uk/cgi-bin/nomenclature/searchgenes.pl?field=symbol&anchor=equals&symbol_search=Search&number=100&format=html&sortby=symbol&match=MATN2) |  | [602108](http://www.ncbi.nlm.nih.gov/entrez/dispomim.cgi?id=602108) | **-2.32** |
| 1455980_A_AT | gb:BB770972 /DB_XREF=gi:16197271 /DB_XREF=BB770972 |  |  |  | **-4.75** |
| 1455988_A_AT | chaperonin subunit 6a (zeta) | [CCT6A](http://www.gene.ucl.ac.uk/cgi-bin/nomenclature/searchgenes.pl?field=symbol&anchor=equals&symbol_search=Search&number=100&format=html&sortby=symbol&match=CCT6A) |  | [104613](http://www.ncbi.nlm.nih.gov/entrez/dispomim.cgi?id=104613) | **-1.29** |
| 1455991_AT | cDNA sequence BC037135 | [CCBL2](http://www.gene.ucl.ac.uk/cgi-bin/nomenclature/searchgenes.pl?field=symbol&anchor=equals&symbol_search=Search&number=100&format=html&sortby=symbol&match=CCBL2) |  |  | **1.4** |
| 1456014_S_AT | cDNA sequence BC032204 | [BC032204](http://www.gene.ucl.ac.uk/cgi-bin/nomenclature/searchgenes.pl?field=symbol&anchor=equals&symbol_search=Search&number=100&format=html&sortby=symbol&match=BC032204) |  |  | **-2.9** |
| 1456015_X_AT | gb:AW228218 /DB_XREF=gi:6557514 /DB_XREF=up14h04.x |  |  |  | **1.28** |
| 1456028_X_AT | Myristoylated alanine rich protein kinase C substrate | [MARCKS](http://www.gene.ucl.ac.uk/cgi-bin/nomenclature/searchgenes.pl?field=symbol&anchor=equals&symbol_search=Search&number=100&format=html&sortby=symbol&match=MARCKS) |  | [177061](http://www.ncbi.nlm.nih.gov/entrez/dispomim.cgi?id=177061) | **-1.43** |
| 1456029_A_AT | Tetratricopeptide repeat domain 7B | [TTC7B](http://www.gene.ucl.ac.uk/cgi-bin/nomenclature/searchgenes.pl?field=symbol&anchor=equals&symbol_search=Search&number=100&format=html&sortby=symbol&match=TTC7B) |  |  | **1.53** |
| 1456032_X_AT | H2A histone family, member Z | [H2AFZ](http://www.gene.ucl.ac.uk/cgi-bin/nomenclature/searchgenes.pl?field=symbol&anchor=equals&symbol_search=Search&number=100&format=html&sortby=symbol&match=H2AFZ) |  | [142763](http://www.ncbi.nlm.nih.gov/entrez/dispomim.cgi?id=142763) | **-1.6** |
| 1456033_AT | T-box 4 | [TBX4](http://www.gene.ucl.ac.uk/cgi-bin/nomenclature/searchgenes.pl?field=symbol&anchor=equals&symbol_search=Search&number=100&format=html&sortby=symbol&match=TBX4) |  | [601719](http://www.ncbi.nlm.nih.gov/entrez/dispomim.cgi?id=601719) | **-2.24** |
| 1456048_AT | gb:BB281000 /DB_XREF=gi:15411106 /DB_XREF=BB281000 |  |  |  | **1.61** |
| 1456056_A_AT | DNA segment, Chr 6, Wayne State University 116, expressed | [D6WSU116E](http://www.gene.ucl.ac.uk/cgi-bin/nomenclature/searchgenes.pl?field=symbol&anchor=equals&symbol_search=Search&number=100&format=html&sortby=symbol&match=D6WSU116E) |  |  | **-1.39** |
| 1456060_AT | avian musculoaponeurotic fibrosarcoma (v-maf) AS42 oncogene homolog | [MAF](http://www.gene.ucl.ac.uk/cgi-bin/nomenclature/searchgenes.pl?field=symbol&anchor=equals&symbol_search=Search&number=100&format=html&sortby=symbol&match=MAF) |  | [177075](http://www.ncbi.nlm.nih.gov/entrez/dispomim.cgi?id=177075) | **-1.37** |
| 1456064_AT | expressed sequence AI504432 | [AI504432](http://www.gene.ucl.ac.uk/cgi-bin/nomenclature/searchgenes.pl?field=symbol&anchor=equals&symbol_search=Search&number=100&format=html&sortby=symbol&match=AI504432) |  |  | **-5.3** |
| 1456067_AT | GLI-Kruppel family member GLI3 | [GLI3](http://www.gene.ucl.ac.uk/cgi-bin/nomenclature/searchgenes.pl?field=symbol&anchor=equals&symbol_search=Search&number=100&format=html&sortby=symbol&match=GLI3) |  | [165240](http://www.ncbi.nlm.nih.gov/entrez/dispomim.cgi?id=165240) | **-1.59** |
| 1456070_AT | gb:AI507538 /DB_XREF=gi:4405389 /DB_XREF=vj77d05.x |  |  |  | **1.45** |
| 1456071_A_AT | cytochrome c, somatic | [CYCS](http://www.gene.ucl.ac.uk/cgi-bin/nomenclature/searchgenes.pl?field=symbol&anchor=equals&symbol_search=Search&number=100&format=html&sortby=symbol&match=CYCS) |  | [123970](http://www.ncbi.nlm.nih.gov/entrez/dispomim.cgi?id=123970) | **1.22** |
| 1456079_X_AT | apurinic/apyrimidinic endonuclease 1 | [APEX1](http://www.gene.ucl.ac.uk/cgi-bin/nomenclature/searchgenes.pl?field=symbol&anchor=equals&symbol_search=Search&number=100&format=html&sortby=symbol&match=APEX1) |  | [107748](http://www.ncbi.nlm.nih.gov/entrez/dispomim.cgi?id=107748) | **-1.81** |
| 1456081_A_AT | acetoacetyl-CoA synthetase | [AACS](http://www.gene.ucl.ac.uk/cgi-bin/nomenclature/searchgenes.pl?field=symbol&anchor=equals&symbol_search=Search&number=100&format=html&sortby=symbol&match=AACS) |  |  | **1.61** |
| 1456085_X_AT | CD151 antigen | [CD151](http://www.gene.ucl.ac.uk/cgi-bin/nomenclature/searchgenes.pl?field=symbol&anchor=equals&symbol_search=Search&number=100&format=html&sortby=symbol&match=CD151) |  | [602243](http://www.ncbi.nlm.nih.gov/entrez/dispomim.cgi?id=602243) | **-1.3** |
| 1456090_AT | pyruvate dehydrogenase complex, component X | [PDHX](http://www.gene.ucl.ac.uk/cgi-bin/nomenclature/searchgenes.pl?field=symbol&anchor=equals&symbol_search=Search&number=100&format=html&sortby=symbol&match=PDHX) |  | [608769](http://www.ncbi.nlm.nih.gov/entrez/dispomim.cgi?id=608769) | **1.42** |
| 1456111_AT | gb:BB072624 /DB_XREF=gi:16260157 /DB_XREF=BB072624 |  |  |  | **1.53** |
| 1456125_A_AT | dynein, cytoplasmic, light chain 1 | [DNCLC1](http://www.gene.ucl.ac.uk/cgi-bin/nomenclature/searchgenes.pl?field=symbol&anchor=equals&symbol_search=Search&number=100&format=html&sortby=symbol&match=DNCLC1) |  |  | **-2.2** |
| 1456131_X_AT | dystroglycan 1 | [DAG1](http://www.gene.ucl.ac.uk/cgi-bin/nomenclature/searchgenes.pl?field=symbol&anchor=equals&symbol_search=Search&number=100&format=html&sortby=symbol&match=DAG1) |  | [128239](http://www.ncbi.nlm.nih.gov/entrez/dispomim.cgi?id=128239) | **-1.39** |
| 1456147_AT | sialyltransferase 8 (alpha-2, 8-sialyltransferase) F | [SIAT8F](http://www.gene.ucl.ac.uk/cgi-bin/nomenclature/searchgenes.pl?field=symbol&anchor=equals&symbol_search=Search&number=100&format=html&sortby=symbol&match=SIAT8F) |  |  | **1.82** |
| 1456174_X_AT | N-myc downstream regulated gene 1 | [NDRG1](http://www.gene.ucl.ac.uk/cgi-bin/nomenclature/searchgenes.pl?field=symbol&anchor=equals&symbol_search=Search&number=100&format=html&sortby=symbol&match=NDRG1) |  | [605262](http://www.ncbi.nlm.nih.gov/entrez/dispomim.cgi?id=605262) | **1.4** |
| 1456177_X_AT | RIKEN cDNA 3110006P09 gene | [3110006P09RIK](http://www.gene.ucl.ac.uk/cgi-bin/nomenclature/searchgenes.pl?field=symbol&anchor=equals&symbol_search=Search&number=100&format=html&sortby=symbol&match=3110006P09RIK) |  |  | **-1.27** |
| 1456205_X_AT | tubulin cofactor a | [TBCA](http://www.gene.ucl.ac.uk/cgi-bin/nomenclature/searchgenes.pl?field=symbol&anchor=equals&symbol_search=Search&number=100&format=html&sortby=symbol&match=TBCA) |  |  | **-1.29** |
| 1456212_X_AT | suppressor of cytokine signaling 3 | [SOCS3](http://www.gene.ucl.ac.uk/cgi-bin/nomenclature/searchgenes.pl?field=symbol&anchor=equals&symbol_search=Search&number=100&format=html&sortby=symbol&match=SOCS3) |  | [604176](http://www.ncbi.nlm.nih.gov/entrez/dispomim.cgi?id=604176) | **-3.63** |
| 1456214_AT | protocadherin 7 | [PCDH7](http://www.gene.ucl.ac.uk/cgi-bin/nomenclature/searchgenes.pl?field=symbol&anchor=equals&symbol_search=Search&number=100&format=html&sortby=symbol&match=PCDH7) |  | [602988](http://www.ncbi.nlm.nih.gov/entrez/dispomim.cgi?id=602988) | **-2.28** |
| 1456215_AT | transcription factor A, mitochondrial | [TFAM](http://www.gene.ucl.ac.uk/cgi-bin/nomenclature/searchgenes.pl?field=symbol&anchor=equals&symbol_search=Search&number=100&format=html&sortby=symbol&match=TFAM) |  | 157670 600438 | **1.3** |
| 1456228_X_AT | myelin basic protein | [MBP](http://www.gene.ucl.ac.uk/cgi-bin/nomenclature/searchgenes.pl?field=symbol&anchor=equals&symbol_search=Search&number=100&format=html&sortby=symbol&match=MBP) |  | [159430](http://www.ncbi.nlm.nih.gov/entrez/dispomim.cgi?id=159430) | **2.06** |
| 1456242_AT | LOC433110 | [LOC433110](http://www.gene.ucl.ac.uk/cgi-bin/nomenclature/searchgenes.pl?field=symbol&anchor=equals&symbol_search=Search&number=100&format=html&sortby=symbol&match=LOC433110) |  |  | **-13.24** |
| 1456250_X_AT | transforming growth factor, beta induced | [TGFBI](http://www.gene.ucl.ac.uk/cgi-bin/nomenclature/searchgenes.pl?field=symbol&anchor=equals&symbol_search=Search&number=100&format=html&sortby=symbol&match=TGFBI) |  | [601692](http://www.ncbi.nlm.nih.gov/entrez/dispomim.cgi?id=601692) | **-2.19** |
| 1456251_X_AT | benzodiazepine receptor, peripheral | [BZRP](http://www.gene.ucl.ac.uk/cgi-bin/nomenclature/searchgenes.pl?field=symbol&anchor=equals&symbol_search=Search&number=100&format=html&sortby=symbol&match=BZRP) |  | [109610](http://www.ncbi.nlm.nih.gov/entrez/dispomim.cgi?id=109610) | **-2.64** |
| 1456255_AT | expressed sequence AI314180 | [AI314180](http://www.gene.ucl.ac.uk/cgi-bin/nomenclature/searchgenes.pl?field=symbol&anchor=equals&symbol_search=Search&number=100&format=html&sortby=symbol&match=AI314180) |  |  | **1.76** |
| 1456279_A_AT | B-cell receptor-associated protein 31 | [BCAP31](http://www.gene.ucl.ac.uk/cgi-bin/nomenclature/searchgenes.pl?field=symbol&anchor=equals&symbol_search=Search&number=100&format=html&sortby=symbol&match=BCAP31) |  | [300398](http://www.ncbi.nlm.nih.gov/entrez/dispomim.cgi?id=300398) | **-1.23** |
| 1456292_A_AT | vimentin | [VIM](http://www.gene.ucl.ac.uk/cgi-bin/nomenclature/searchgenes.pl?field=symbol&anchor=equals&symbol_search=Search&number=100&format=html&sortby=symbol&match=VIM) |  | [193060](http://www.ncbi.nlm.nih.gov/entrez/dispomim.cgi?id=193060) | **-2.07** |
| 1456307_S_AT | Adenylate cyclase 7 | [ADCY7](http://www.gene.ucl.ac.uk/cgi-bin/nomenclature/searchgenes.pl?field=symbol&anchor=equals&symbol_search=Search&number=100&format=html&sortby=symbol&match=ADCY7) |  | [600385](http://www.ncbi.nlm.nih.gov/entrez/dispomim.cgi?id=600385) | **-2.05** |
| 1456310_A_AT | RIKEN cDNA 2610002J02 gene | [2610002J02RIK](http://www.gene.ucl.ac.uk/cgi-bin/nomenclature/searchgenes.pl?field=symbol&anchor=equals&symbol_search=Search&number=100&format=html&sortby=symbol&match=2610002J02RIK) |  |  | **-3.49** |
| 1456315_A_AT | protein tyrosine phosphatase-like (proline instead of catalytic arginine), member a | [PTPLA](http://www.gene.ucl.ac.uk/cgi-bin/nomenclature/searchgenes.pl?field=symbol&anchor=equals&symbol_search=Search&number=100&format=html&sortby=symbol&match=PTPLA) |  |  | **-1.79** |
| 1456319_AT | gb:BG065719 /DB_XREF=gi:12548282 /DB_XREF=H3034D11 |  |  |  | **-2.09** |
| 1456325_AT | cDNA sequence BC027174 | [BC027174](http://www.gene.ucl.ac.uk/cgi-bin/nomenclature/searchgenes.pl?field=symbol&anchor=equals&symbol_search=Search&number=100&format=html&sortby=symbol&match=BC027174) |  |  | **1.4** |
| 1456377_X_AT | RIKEN cDNA 0610025L06 gene | [0610025L06RIK](http://www.gene.ucl.ac.uk/cgi-bin/nomenclature/searchgenes.pl?field=symbol&anchor=equals&symbol_search=Search&number=100&format=html&sortby=symbol&match=0610025L06RIK) |  |  | **-2.78** |
| 1456380_X_AT | calponin 3, acidic | [CNN3](http://www.gene.ucl.ac.uk/cgi-bin/nomenclature/searchgenes.pl?field=symbol&anchor=equals&symbol_search=Search&number=100&format=html&sortby=symbol&match=CNN3) |  | [602374](http://www.ncbi.nlm.nih.gov/entrez/dispomim.cgi?id=602374) | **-2.96** |
| 1456389_AT | RIKEN cDNA 9130203F04 gene | [9130203F04RIK](http://www.gene.ucl.ac.uk/cgi-bin/nomenclature/searchgenes.pl?field=symbol&anchor=equals&symbol_search=Search&number=100&format=html&sortby=symbol&match=9130203F04RIK) |  |  | **-1.51** |
| 1456407_A_AT | tousled-like kinase 1 | [TLK1](http://www.gene.ucl.ac.uk/cgi-bin/nomenclature/searchgenes.pl?field=symbol&anchor=equals&symbol_search=Search&number=100&format=html&sortby=symbol&match=TLK1) |  | [608438](http://www.ncbi.nlm.nih.gov/entrez/dispomim.cgi?id=608438) | **1.31** |
| 1456436_X_AT | ribosomal protein S20 | [RPS20](http://www.gene.ucl.ac.uk/cgi-bin/nomenclature/searchgenes.pl?field=symbol&anchor=equals&symbol_search=Search&number=100&format=html&sortby=symbol&match=RPS20) |  | [603682](http://www.ncbi.nlm.nih.gov/entrez/dispomim.cgi?id=603682) | **-1.24** |
| 1456438_X_AT | ribophorin I | [RPN1](http://www.gene.ucl.ac.uk/cgi-bin/nomenclature/searchgenes.pl?field=symbol&anchor=equals&symbol_search=Search&number=100&format=html&sortby=symbol&match=RPN1) |  | [180470](http://www.ncbi.nlm.nih.gov/entrez/dispomim.cgi?id=180470) | **-1.44** |
| 1456442_AT | expressed sequence C76746 | [C76746](http://www.gene.ucl.ac.uk/cgi-bin/nomenclature/searchgenes.pl?field=symbol&anchor=equals&symbol_search=Search&number=100&format=html&sortby=symbol&match=C76746) |  |  | **-2.06** |
| 1456480_AT | RIKEN cDNA 9330186A19 gene | [9330186A19RIK](http://www.gene.ucl.ac.uk/cgi-bin/nomenclature/searchgenes.pl?field=symbol&anchor=equals&symbol_search=Search&number=100&format=html&sortby=symbol&match=9330186A19RIK) |  |  | **1.51** |
| 1456487_AT | gb:AI848263 /DB_XREF=gi:5492169 /DB_XREF=UI-M-AH1- |  |  |  | **2.58** |
| 1456495_S_AT | oxysterol binding protein-like 6 | [OSBPL6](http://www.gene.ucl.ac.uk/cgi-bin/nomenclature/searchgenes.pl?field=symbol&anchor=equals&symbol_search=Search&number=100&format=html&sortby=symbol&match=OSBPL6) |  | [606734](http://www.ncbi.nlm.nih.gov/entrez/dispomim.cgi?id=606734) | **2.01** |
| 1456497_X_AT | ribosomal protein S10 | [RPS10](http://www.gene.ucl.ac.uk/cgi-bin/nomenclature/searchgenes.pl?field=symbol&anchor=equals&symbol_search=Search&number=100&format=html&sortby=symbol&match=RPS10) |  | [603632](http://www.ncbi.nlm.nih.gov/entrez/dispomim.cgi?id=603632) | **-1.37** |
| 1456530_X_AT | elongation of very long chain fatty acids (FEN1/Elo2, SUR4/Elo3, yeast)-like 1 | [ELOVL1](http://www.gene.ucl.ac.uk/cgi-bin/nomenclature/searchgenes.pl?field=symbol&anchor=equals&symbol_search=Search&number=100&format=html&sortby=symbol&match=ELOVL1) |  |  | **-1.43** |
| 1456532_AT | platelet-derived growth factor, D polypeptide | [PDGFD](http://www.gene.ucl.ac.uk/cgi-bin/nomenclature/searchgenes.pl?field=symbol&anchor=equals&symbol_search=Search&number=100&format=html&sortby=symbol&match=PDGFD) |  |  | **1.74** |
| 1456533_AT | RIKEN cDNA 1100001I19 gene | [1100001I19RIK](http://www.gene.ucl.ac.uk/cgi-bin/nomenclature/searchgenes.pl?field=symbol&anchor=equals&symbol_search=Search&number=100&format=html&sortby=symbol&match=1100001I19RIK) |  |  | **-1.46** |
| 1456542_S_AT | Glutaminyl-tRNA synthase (glutamine-hydrolyzing)-like 1 | [QRSL1](http://www.gene.ucl.ac.uk/cgi-bin/nomenclature/searchgenes.pl?field=symbol&anchor=equals&symbol_search=Search&number=100&format=html&sortby=symbol&match=QRSL1) |  |  | **1.39** |
| 1456551_AT | gb:BB313400 /DB_XREF=gi:16402889 /DB_XREF=BB313400 |  |  |  | **1.47** |
| 1456563_AT | gb:BB769119 /DB_XREF=gi:16211661 /DB_XREF=BB769119 |  |  |  | **-1.71** |
| 1456567_X_AT | granulin | [GRN](http://www.gene.ucl.ac.uk/cgi-bin/nomenclature/searchgenes.pl?field=symbol&anchor=equals&symbol_search=Search&number=100&format=html&sortby=symbol&match=GRN) |  | [138945](http://www.ncbi.nlm.nih.gov/entrez/dispomim.cgi?id=138945) | **-1.64** |
| 1456573_X_AT | nicotinamide nucleotide transhydrogenase | [NNT](http://www.gene.ucl.ac.uk/cgi-bin/nomenclature/searchgenes.pl?field=symbol&anchor=equals&symbol_search=Search&number=100&format=html&sortby=symbol&match=NNT) |  | [607878](http://www.ncbi.nlm.nih.gov/entrez/dispomim.cgi?id=607878) | **1.47** |
| 1456581_X_AT | guanosine diphosphate (GDP) dissociation inhibitor 3 | [GDI3](http://www.gene.ucl.ac.uk/cgi-bin/nomenclature/searchgenes.pl?field=symbol&anchor=equals&symbol_search=Search&number=100&format=html&sortby=symbol&match=GDI3) |  | [600767](http://www.ncbi.nlm.nih.gov/entrez/dispomim.cgi?id=600767) | **-1.36** |
| 1456609_AT | RIKEN cDNA 1810006K23 gene | [1810006K23RIK](http://www.gene.ucl.ac.uk/cgi-bin/nomenclature/searchgenes.pl?field=symbol&anchor=equals&symbol_search=Search&number=100&format=html&sortby=symbol&match=1810006K23RIK) |  |  | **1.4** |
| 1456614_AT | gb:BF122715 /DB_XREF=gi:10961755 /DB_XREF=60176124 |  |  |  | **1.44** |
| 1456616_A_AT | basigin | [BSG](http://www.gene.ucl.ac.uk/cgi-bin/nomenclature/searchgenes.pl?field=symbol&anchor=equals&symbol_search=Search&number=100&format=html&sortby=symbol&match=BSG) |  | [109480](http://www.ncbi.nlm.nih.gov/entrez/dispomim.cgi?id=109480) | **1.26** |
| 1456623_AT | tropomyosin 1, alpha | [TPM1](http://www.gene.ucl.ac.uk/cgi-bin/nomenclature/searchgenes.pl?field=symbol&anchor=equals&symbol_search=Search&number=100&format=html&sortby=symbol&match=TPM1) |  | [191010](http://www.ncbi.nlm.nih.gov/entrez/dispomim.cgi?id=191010) | **-1.73** |
| 1456629_AT | DNA segment, Chr 17, ERATO Doi 288, expressed | [D17ERTD288E](http://www.gene.ucl.ac.uk/cgi-bin/nomenclature/searchgenes.pl?field=symbol&anchor=equals&symbol_search=Search&number=100&format=html&sortby=symbol&match=D17ERTD288E) |  |  | **1.69** |
| 1456642_X_AT | S100 calcium binding protein A10 (calpactin) | [S100A10](http://www.gene.ucl.ac.uk/cgi-bin/nomenclature/searchgenes.pl?field=symbol&anchor=equals&symbol_search=Search&number=100&format=html&sortby=symbol&match=S100A10) |  | [114085](http://www.ncbi.nlm.nih.gov/entrez/dispomim.cgi?id=114085) | **-2.4** |
| 1456663_X_AT | RIKEN cDNA 2410018G23 gene | [2410018G23RIK](http://www.gene.ucl.ac.uk/cgi-bin/nomenclature/searchgenes.pl?field=symbol&anchor=equals&symbol_search=Search&number=100&format=html&sortby=symbol&match=2410018G23RIK) |  |  | **-1.21** |
| 1456664_X_AT | heterogeneous nuclear ribonucleoprotein F | [HNRPF](http://www.gene.ucl.ac.uk/cgi-bin/nomenclature/searchgenes.pl?field=symbol&anchor=equals&symbol_search=Search&number=100&format=html&sortby=symbol&match=HNRPF) |  | [601037](http://www.ncbi.nlm.nih.gov/entrez/dispomim.cgi?id=601037) | **-1.39** |
| 1456700_X_AT | Myristoylated alanine rich protein kinase C substrate | [MARCKS](http://www.gene.ucl.ac.uk/cgi-bin/nomenclature/searchgenes.pl?field=symbol&anchor=equals&symbol_search=Search&number=100&format=html&sortby=symbol&match=MARCKS) |  | [177061](http://www.ncbi.nlm.nih.gov/entrez/dispomim.cgi?id=177061) | **-1.87** |
| 1456702_X_AT | methionine adenosyltransferase II, alpha | [MAT2A](http://www.gene.ucl.ac.uk/cgi-bin/nomenclature/searchgenes.pl?field=symbol&anchor=equals&symbol_search=Search&number=100&format=html&sortby=symbol&match=MAT2A) |  | [601468](http://www.ncbi.nlm.nih.gov/entrez/dispomim.cgi?id=601468) | **-1.84** |
| 1456730_X_AT | gb:BB094081 /DB_XREF=gi:8677330 /DB_XREF=BB094081 |  |  |  | **-1.34** |
| 1456733_X_AT | serine (or cysteine) proteinase inhibitor, clade H, member 1 | [SERPINH1](http://www.gene.ucl.ac.uk/cgi-bin/nomenclature/searchgenes.pl?field=symbol&anchor=equals&symbol_search=Search&number=100&format=html&sortby=symbol&match=SERPINH1) |  | [600943](http://www.ncbi.nlm.nih.gov/entrez/dispomim.cgi?id=600943) | **-2.84** |
| 1456735_X_AT | acid phosphatase-like 2 | [ACPL2](http://www.gene.ucl.ac.uk/cgi-bin/nomenclature/searchgenes.pl?field=symbol&anchor=equals&symbol_search=Search&number=100&format=html&sortby=symbol&match=ACPL2) |  |  | **-2.37** |
| 1456739_X_AT | armadillo repeat containing, X-linked 2 | [ARMCX2](http://www.gene.ucl.ac.uk/cgi-bin/nomenclature/searchgenes.pl?field=symbol&anchor=equals&symbol_search=Search&number=100&format=html&sortby=symbol&match=ARMCX2) |  | [300363](http://www.ncbi.nlm.nih.gov/entrez/dispomim.cgi?id=300363) | **-2.66** |
| 1456741_S_AT | glycoprotein m6a | [GPM6A](http://www.gene.ucl.ac.uk/cgi-bin/nomenclature/searchgenes.pl?field=symbol&anchor=equals&symbol_search=Search&number=100&format=html&sortby=symbol&match=GPM6A) |  | [601275](http://www.ncbi.nlm.nih.gov/entrez/dispomim.cgi?id=601275) | **1.83** |
| 1456743_X_AT | mortality factor 4 like 2 | [MORF4L2](http://www.gene.ucl.ac.uk/cgi-bin/nomenclature/searchgenes.pl?field=symbol&anchor=equals&symbol_search=Search&number=100&format=html&sortby=symbol&match=MORF4L2) |  | [300409](http://www.ncbi.nlm.nih.gov/entrez/dispomim.cgi?id=300409) | **-1.54** |
| 1456753_AT | gb:AW209075 /DB_XREF=gi:6515015 /DB_XREF=un99e08.x |  |  |  | **1.44** |
| 1456755_AT | RIKEN cDNA 2310001H13 gene | [2310001H13RIK](http://www.gene.ucl.ac.uk/cgi-bin/nomenclature/searchgenes.pl?field=symbol&anchor=equals&symbol_search=Search&number=100&format=html&sortby=symbol&match=2310001H13RIK) |  |  | **1.55** |
| 1456756_AT | RIKEN cDNA E430016P22 gene | [E430016P22RIK](http://www.gene.ucl.ac.uk/cgi-bin/nomenclature/searchgenes.pl?field=symbol&anchor=equals&symbol_search=Search&number=100&format=html&sortby=symbol&match=E430016P22RIK) |  |  | **-1.81** |
| 1456762_AT | gb:BM237714 /DB_XREF=gi:17872984 /DB_XREF=K0505H05 |  |  |  | **1.75** |
| 1456768_A_AT | multimerin 2 | [MMRN2](http://www.gene.ucl.ac.uk/cgi-bin/nomenclature/searchgenes.pl?field=symbol&anchor=equals&symbol_search=Search&number=100&format=html&sortby=symbol&match=MMRN2) |  | [608925](http://www.ncbi.nlm.nih.gov/entrez/dispomim.cgi?id=608925) | **1.64** |
| 1456769_AT | dual specificity phosphatase 3 (vaccinia virus phosphatase VH1-related) | [MPP3](http://www.gene.ucl.ac.uk/cgi-bin/nomenclature/searchgenes.pl?field=symbol&anchor=equals&symbol_search=Search&number=100&format=html&sortby=symbol&match=MPP3) |  | 600183 601114 | **-1.73** |
| 1456772_AT | neutrophil cytosolic factor 1 | [NCF1](http://www.gene.ucl.ac.uk/cgi-bin/nomenclature/searchgenes.pl?field=symbol&anchor=equals&symbol_search=Search&number=100&format=html&sortby=symbol&match=NCF1) |  | [608512](http://www.ncbi.nlm.nih.gov/entrez/dispomim.cgi?id=608512) | **-1.74** |
| 1456783_AT | RIKEN cDNA 9330107J05 gene | [9330107J05RIK](http://www.gene.ucl.ac.uk/cgi-bin/nomenclature/searchgenes.pl?field=symbol&anchor=equals&symbol_search=Search&number=100&format=html&sortby=symbol&match=9330107J05RIK) |  |  | **-3.09** |
| 1456785_AT | cofactor required for Sp1 transcriptional activation, subunit 2 | [CRSP2](http://www.gene.ucl.ac.uk/cgi-bin/nomenclature/searchgenes.pl?field=symbol&anchor=equals&symbol_search=Search&number=100&format=html&sortby=symbol&match=CRSP2) |  | [300182](http://www.ncbi.nlm.nih.gov/entrez/dispomim.cgi?id=300182) | **1.35** |
| 1456830_AT | gb:BB542221 /DB_XREF=gi:15412374 /DB_XREF=BB542221 |  |  |  | **1.43** |
| 1456867_X_AT | serologically defined breast cancer antigen 84 | [SDBCAG84](http://www.gene.ucl.ac.uk/cgi-bin/nomenclature/searchgenes.pl?field=symbol&anchor=equals&symbol_search=Search&number=100&format=html&sortby=symbol&match=SDBCAG84) |  |  | **-1.23** |
| 1456873_AT | gb:BB236747 /DB_XREF=gi:8916283 /DB_XREF=BB236747 |  |  |  | **1.36** |
| 1456886_AT | gb:BF454116 /DB_XREF=gi:11520212 /DB_XREF=maa58d03 |  |  |  | **1.31** |
| 1456888_AT | hypothetical protein C230090D14 | [C230090D14](http://www.gene.ucl.ac.uk/cgi-bin/nomenclature/searchgenes.pl?field=symbol&anchor=equals&symbol_search=Search&number=100&format=html&sortby=symbol&match=C230090D14) |  |  | **-1.32** |
| 1456893_AT | gb:BF660952 /DB_XREF=gi:11926086 /DB_XREF=maa70g11 |  |  |  | **-1.45** |
| 1456912_AT | RIKEN cDNA 6430544H17 gene | [6430544H17RIK](http://www.gene.ucl.ac.uk/cgi-bin/nomenclature/searchgenes.pl?field=symbol&anchor=equals&symbol_search=Search&number=100&format=html&sortby=symbol&match=6430544H17RIK) |  |  | **1.4** |
| 1456913_AT | Tropomodulin 3 | [TMOD3](http://www.gene.ucl.ac.uk/cgi-bin/nomenclature/searchgenes.pl?field=symbol&anchor=equals&symbol_search=Search&number=100&format=html&sortby=symbol&match=TMOD3) |  | [605112](http://www.ncbi.nlm.nih.gov/entrez/dispomim.cgi?id=605112) | **1.49** |
| 1457031_AT | RIKEN cDNA 9830160G03 gene | [9830160G03RIK](http://www.gene.ucl.ac.uk/cgi-bin/nomenclature/searchgenes.pl?field=symbol&anchor=equals&symbol_search=Search&number=100&format=html&sortby=symbol&match=9830160G03RIK) |  |  | **1.38** |
